# Supplementary figures and images for: Millipede genomes reveal unique adaptations during myriapod evolution
Source: PLoS Biol. 2020 Sep 29;18(9):e3000636. doi: 10.1371/journal.pbio.3000636 (PMC7523956; doi:10.1371/journal.pbio.3000636)

a)

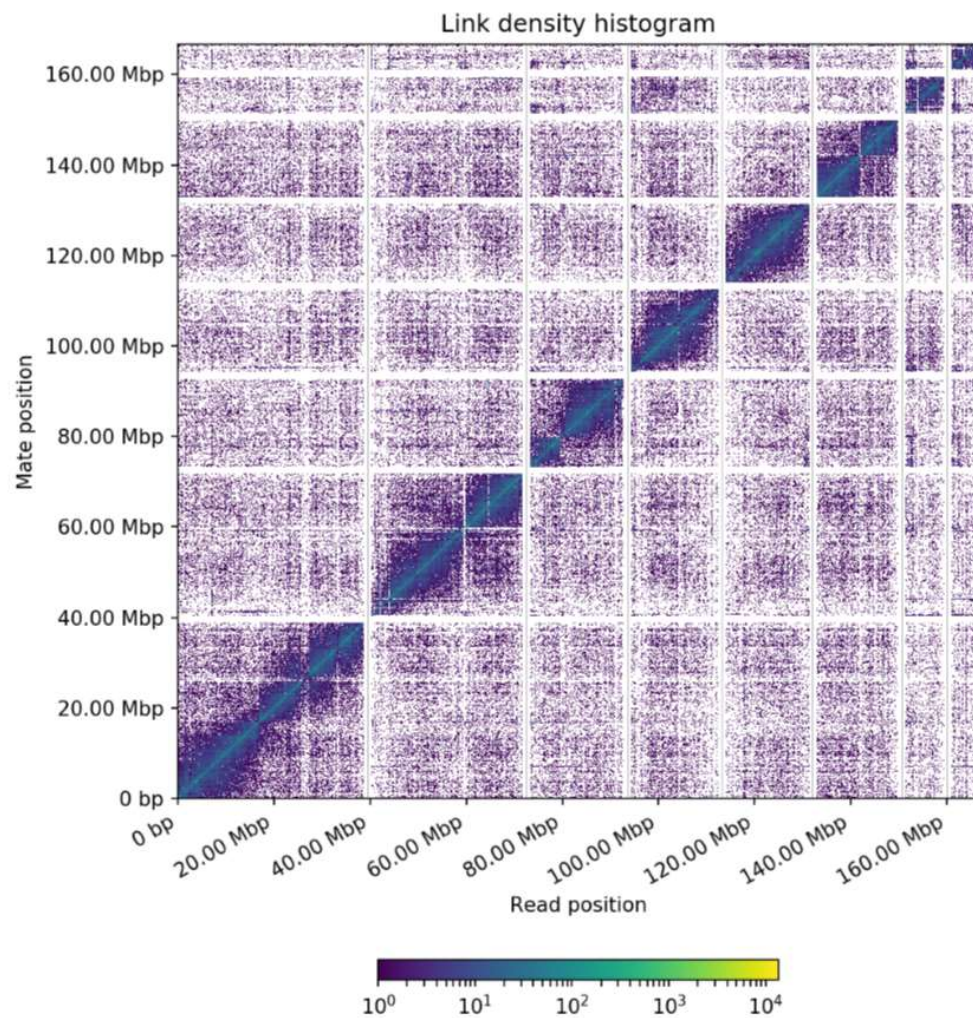

b)

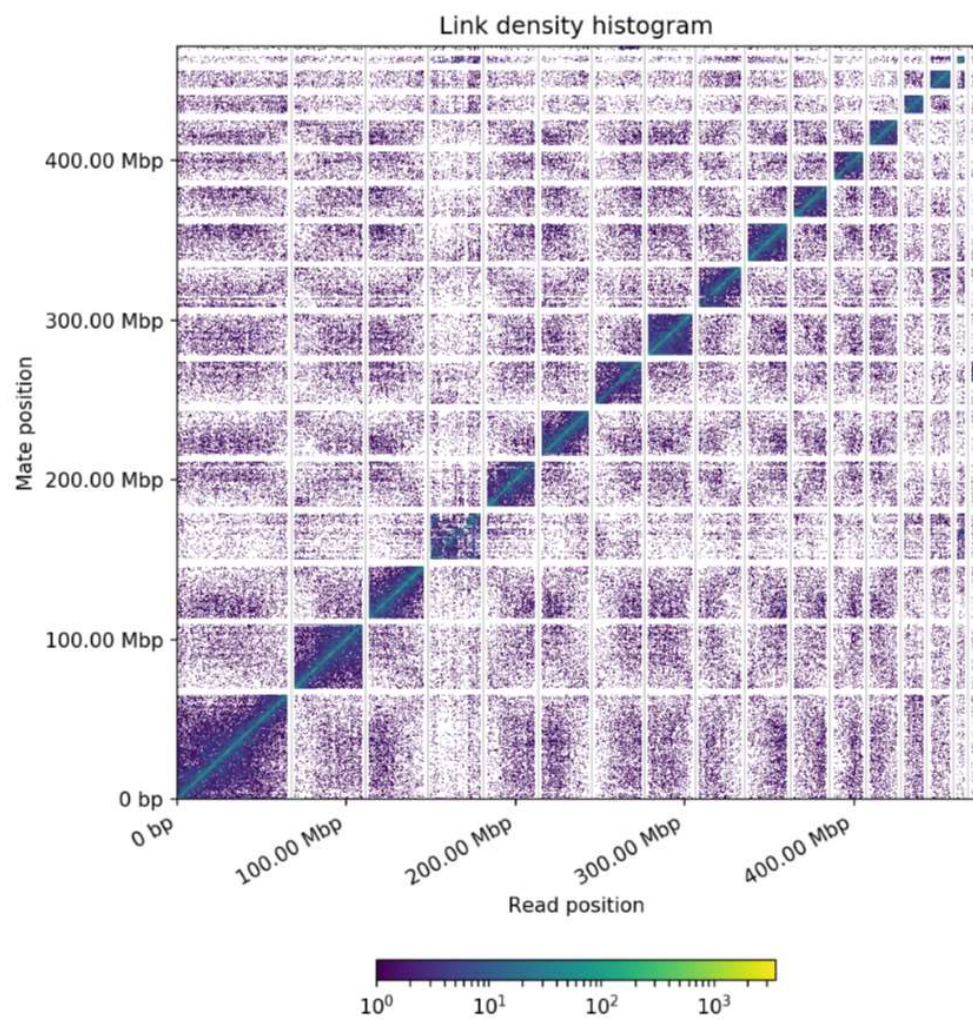

Supplement: S1 Fig — Hi-C information of H. holstii (a) and T. corallinus (b). The x- and y-axes give the mapping positions of the first and second read in the read pair, respectively, grouped into bins. The colour of each square gives the number of read pairs within that bin. White vertical and black horizontal lines have been added to show the borders between scaffolds. Scaffolds less than 1 Mb are excluded. (PDF) [file pbio.3000636.s001.pdf]

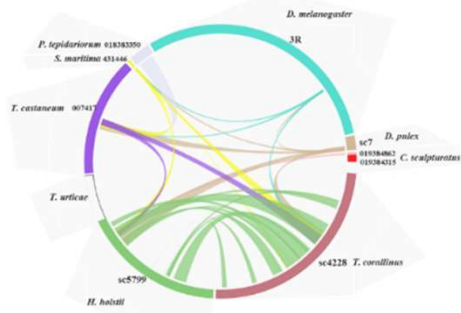

Min dots=7

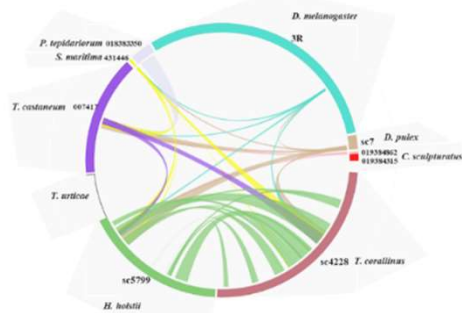

Min dots=6

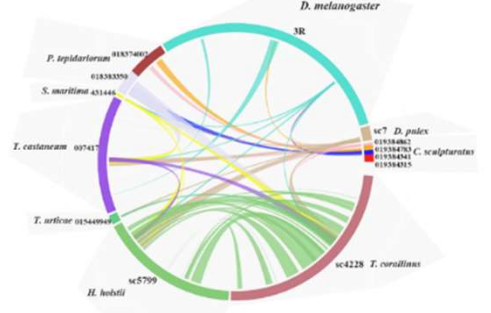

Min dots=5

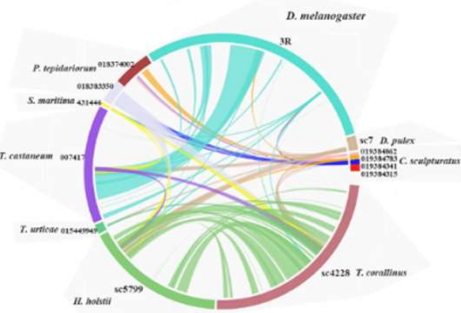

Min dots=4

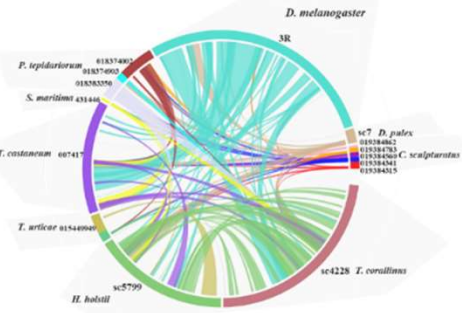

Min dots=3

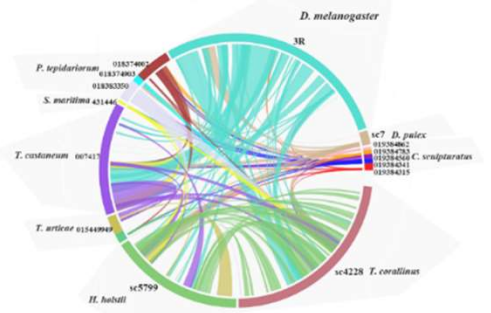

Min dots=2

Supplement: S3 Fig — (PDF) [file pbio.3000636.s003.pdf]

***H. holstii***

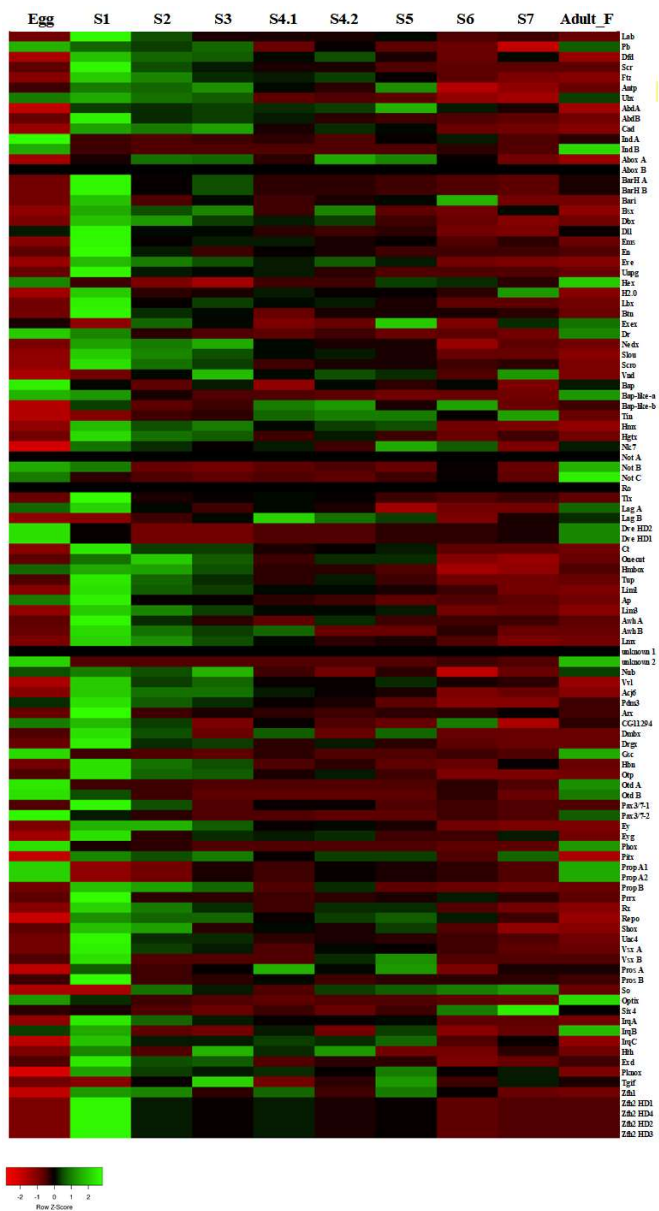

***T. corallinus***

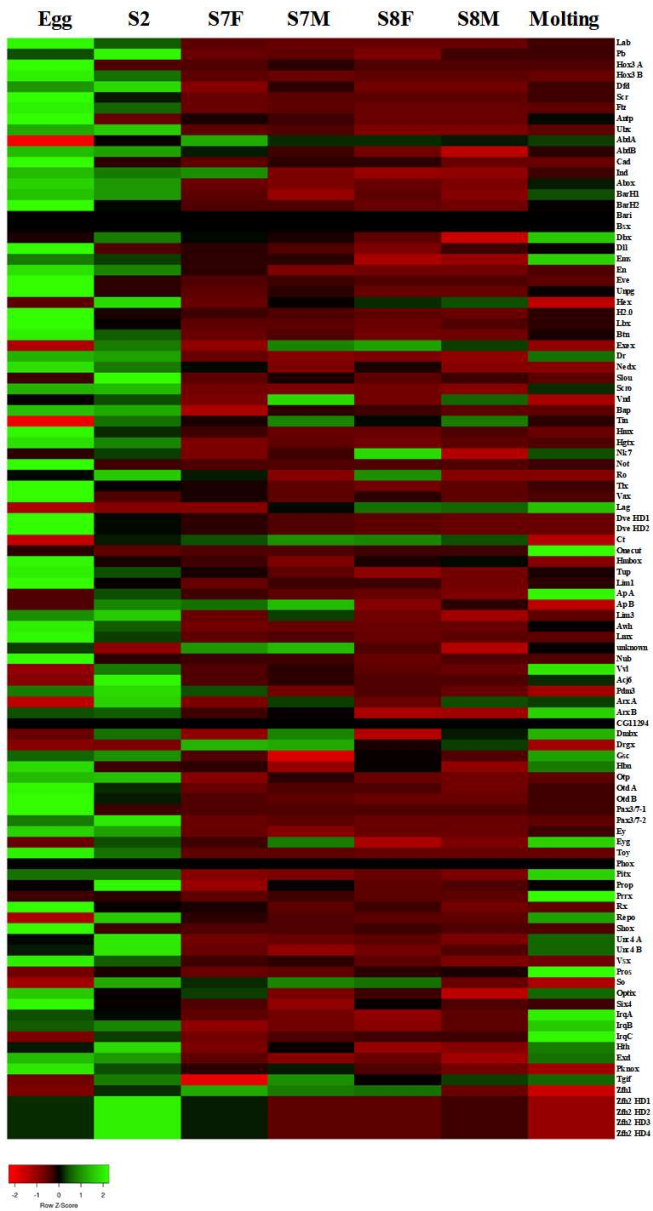

Supplement: S4 Fig — (PDF) [file pbio.3000636.s004.pdf]

a)

*Helicorhormorpha holstii*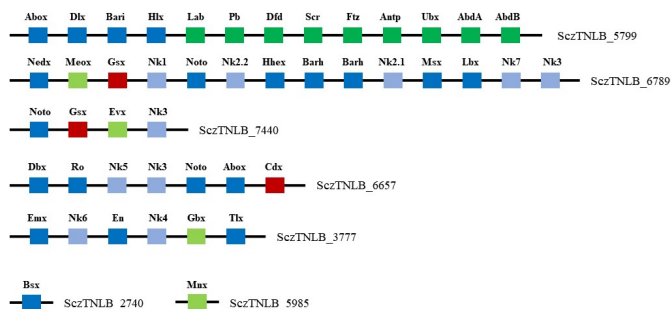*Trigoniulus corallinus*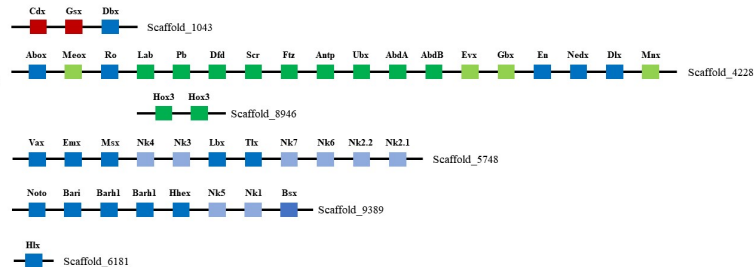

b)

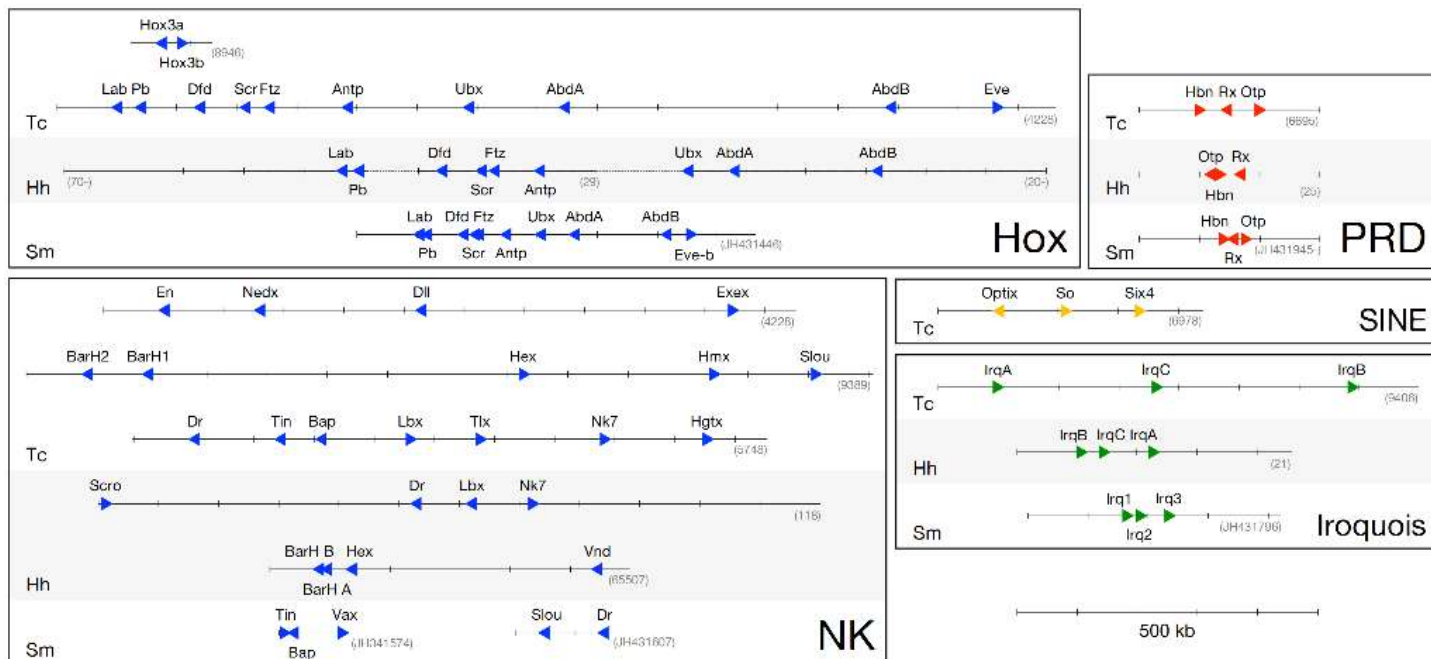

c)

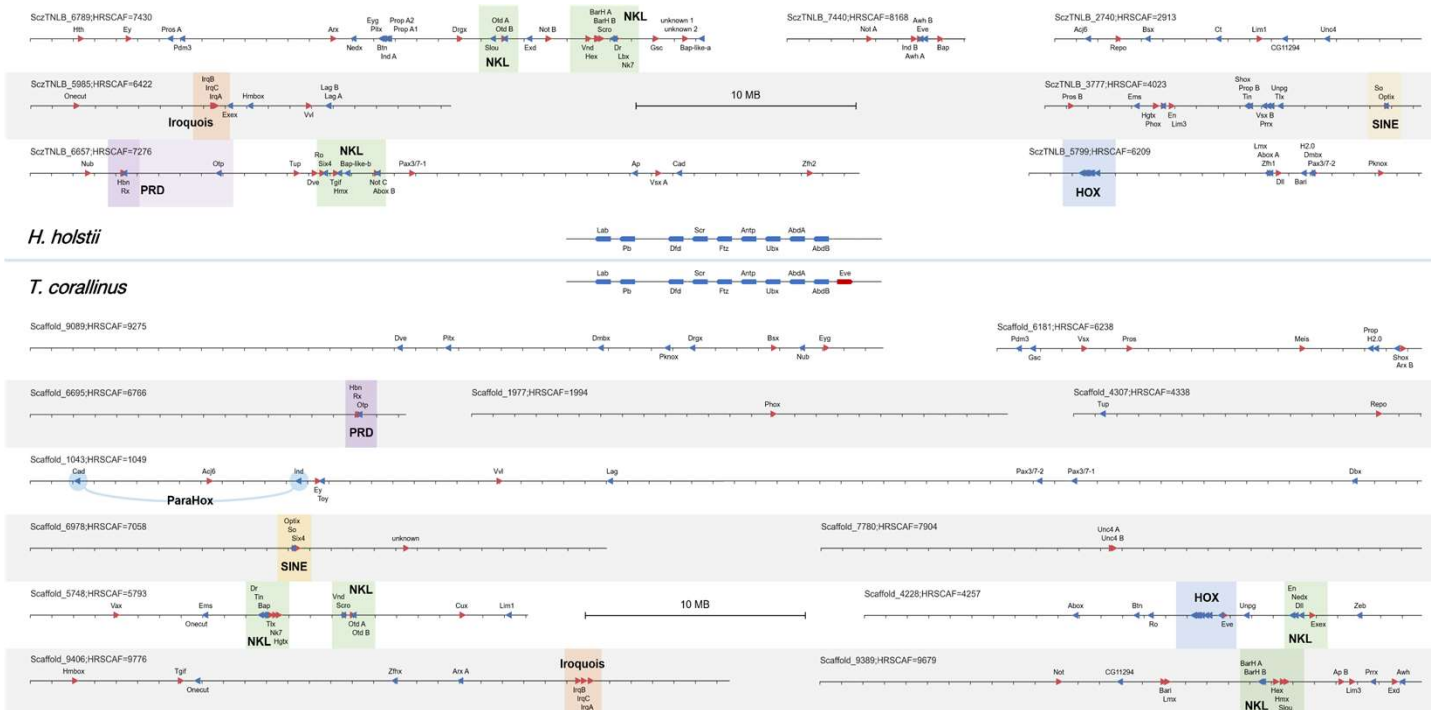

Supplement: S6 Fig — (a) Schematic diagram showing the ANTP-class homeobox gene arrangement in the 2 millipede genomes; (b) schematic diagram showing the homeobox gene clusters in the myriapod genomes. (c) Schematic diagram showing the homeobox genes in the 2 millipede genomes. Details of the genomic locations and sequences of all these homeobox genes can be found in S3 Data. Gene tree can be found in S7 Fig. Hh, H. holstii; Sm, S. maritima; Tc, T. corallinus. (PDF) [file pbio.3000636.s006.pdf]

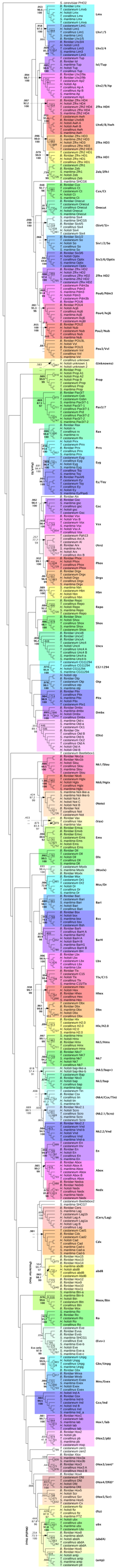

Supplement: S7 Fig — The sequence alignment can be found in S8 Data. (PDF) [file pbio.3000636.s007.pdf]

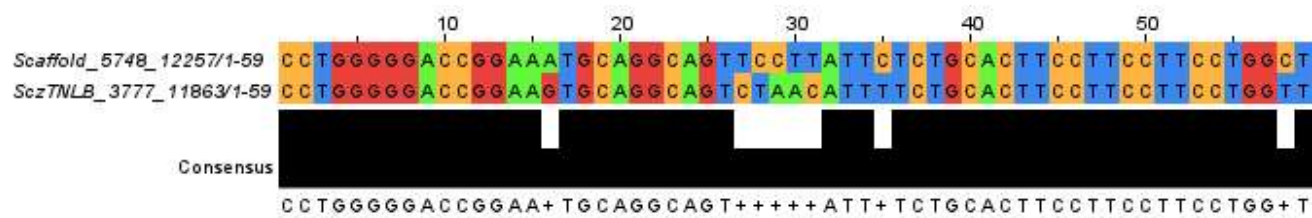

Supplement: S8 Fig — (PDF) [file pbio.3000636.s008.pdf]

a)

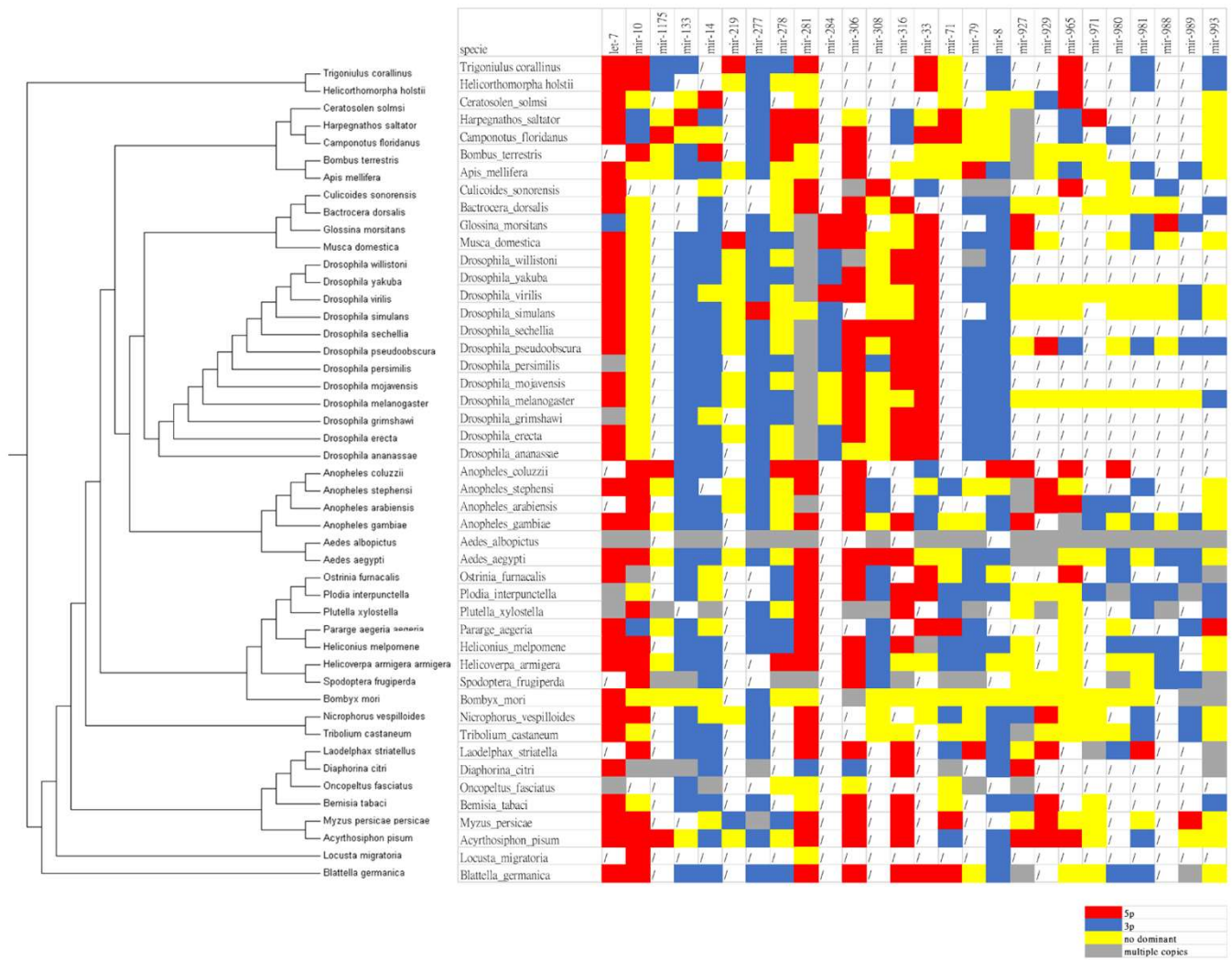

b)

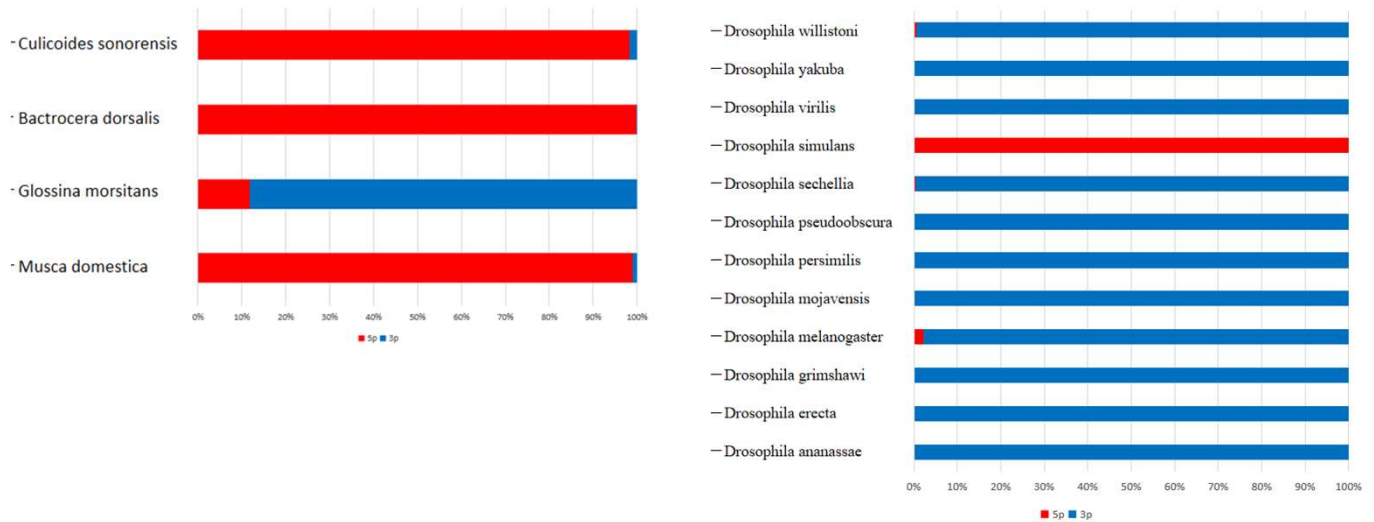

Supplement: S9 Fig — Cases of microRNAs undergone arm switching in insect and millipede genomes (a). Red boxes represent 5p arm dominance, blue boxes represent 3p arm dominance, yellow boxes represent cases for which microRNA dominant arm cannot be determined based on the cut-off set up in this study, and grey boxes represent multiple copies of microRNAs in respective genomes and so cases of arms usage are not determined; (b) microRNA arm switching cases of let-7 (left) and miR-277 (right) in insects. The red and blue colour represent the 5p arm and 3p arm, respectively. Data underlying this figure can be found in S8 Data. (PDF) [file pbio.3000636.s009.pdf]

a)

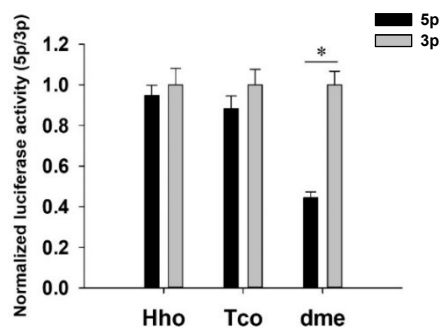

b) Dme-iab-8

Targets comparison

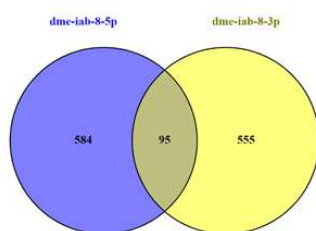

Molecular function

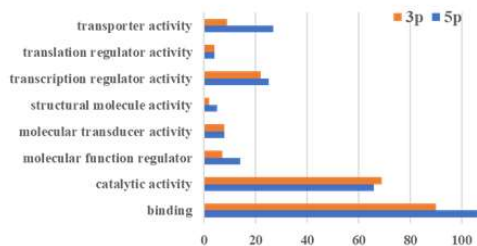

Biological process

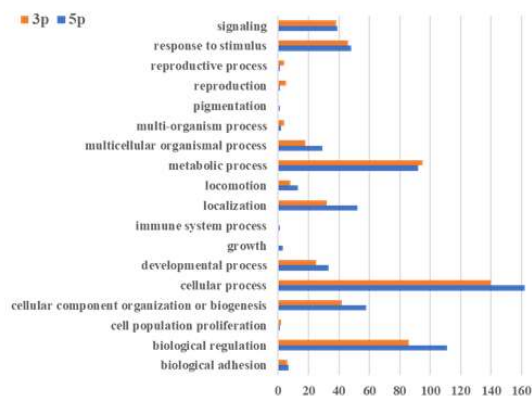

c) Hho-iab-8

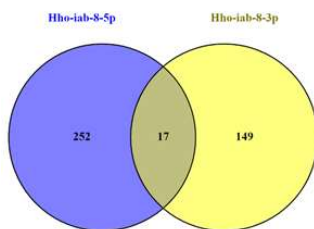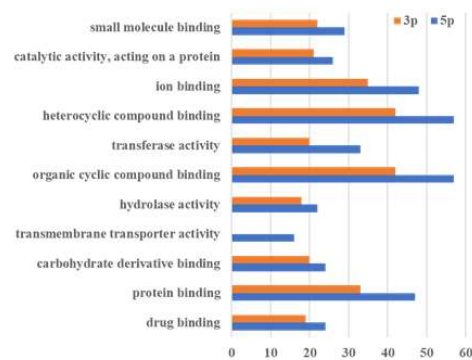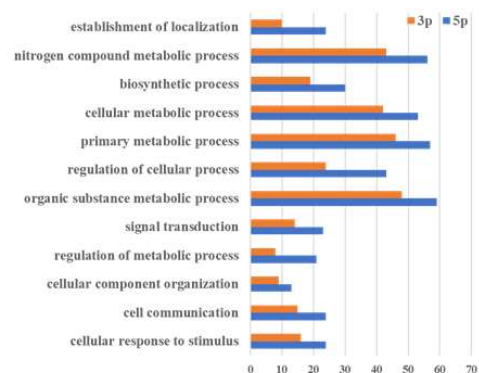

d) Tco-iab-8

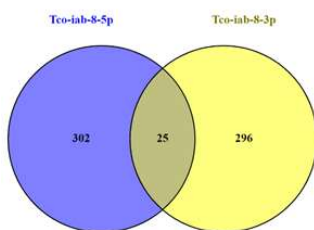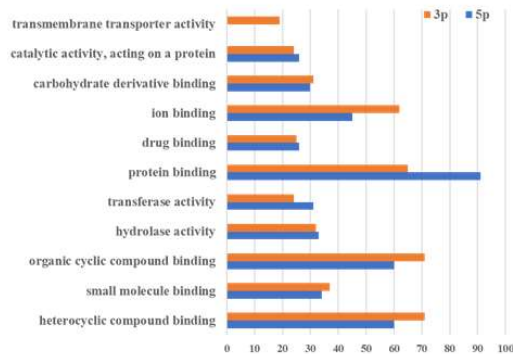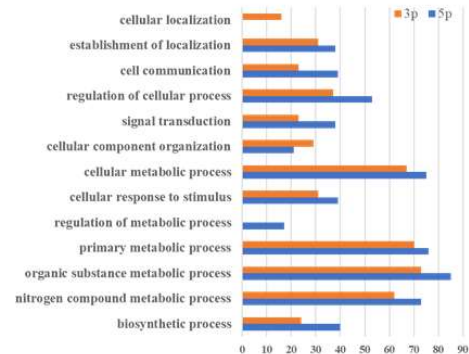

Supplement: S10 Fig — (a) Differential arm target repression ability by different arthropod species of miR-iab-8. Bars represent mean with SEM; t test was used to determine significant difference between 5p and 3p. *p < 0.05; (b–d) predicted number of targets and their GO of miR-iab-8-5p and miR-iab-8-3p in fly and millipedes. The underlying data of this figure can be found in S8 Data. Dme, D. melanogaster; GO, gene ontology; Hho, H. holistii; Tco, T. corallinus. (PDF) [file pbio.3000636.s010.pdf]

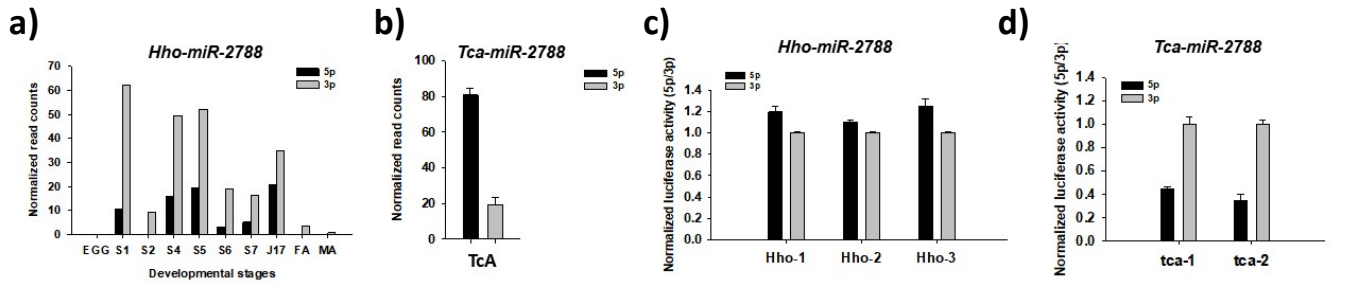

**e) *tca-mir-2788***  
Targets comparison

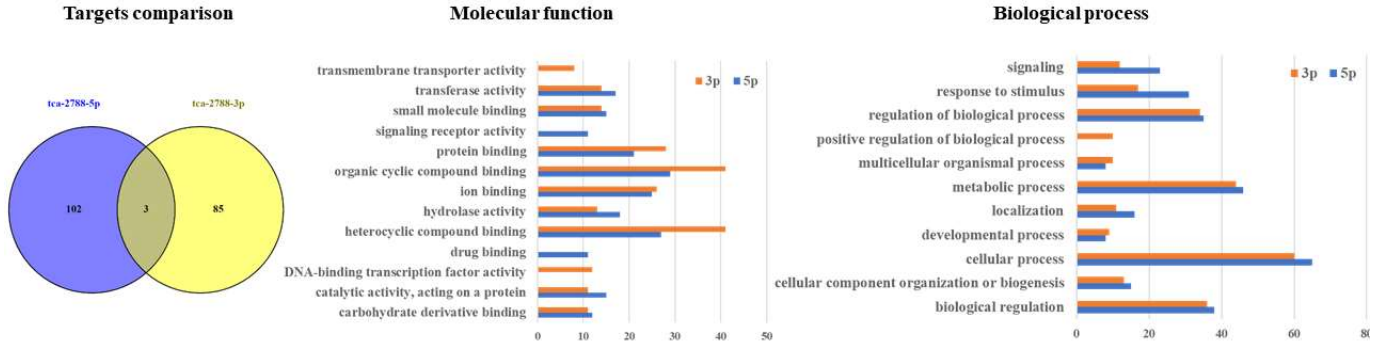

**f) *Hho-mir-2788***

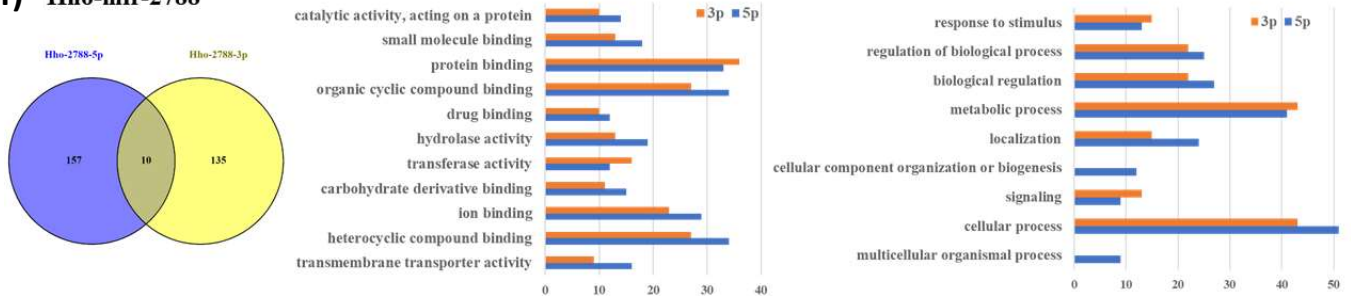

Supplement: S11 Fig — (a–b) Small RNA read counts of miR-2788 in different developmental stages in millipede H. holstii and in TcA cell line of beetle Tribolium castaneum; (c–d) luciferase activity showing the differential arm target (i.e., miR-2788-5p and -3p sensor) repression ability between miR-2788 carrying different flanking sequence of H. holstii and T. castaneum. Bars represent mean with SEM; (e–f) predicted number of targets and their GO of miR-2788-5p and miR-2788-3p in beetle T. castaneum and millipede H. holstii. The underlying data of this figure can be found in S8 Data. FA, adult female; GO, gene ontology; J17, juvenile; MA, adult male; S1–S7, stadia I–VII; TcA, TcA cell line. (PDF) [file pbio.3000636.s011.pdf]

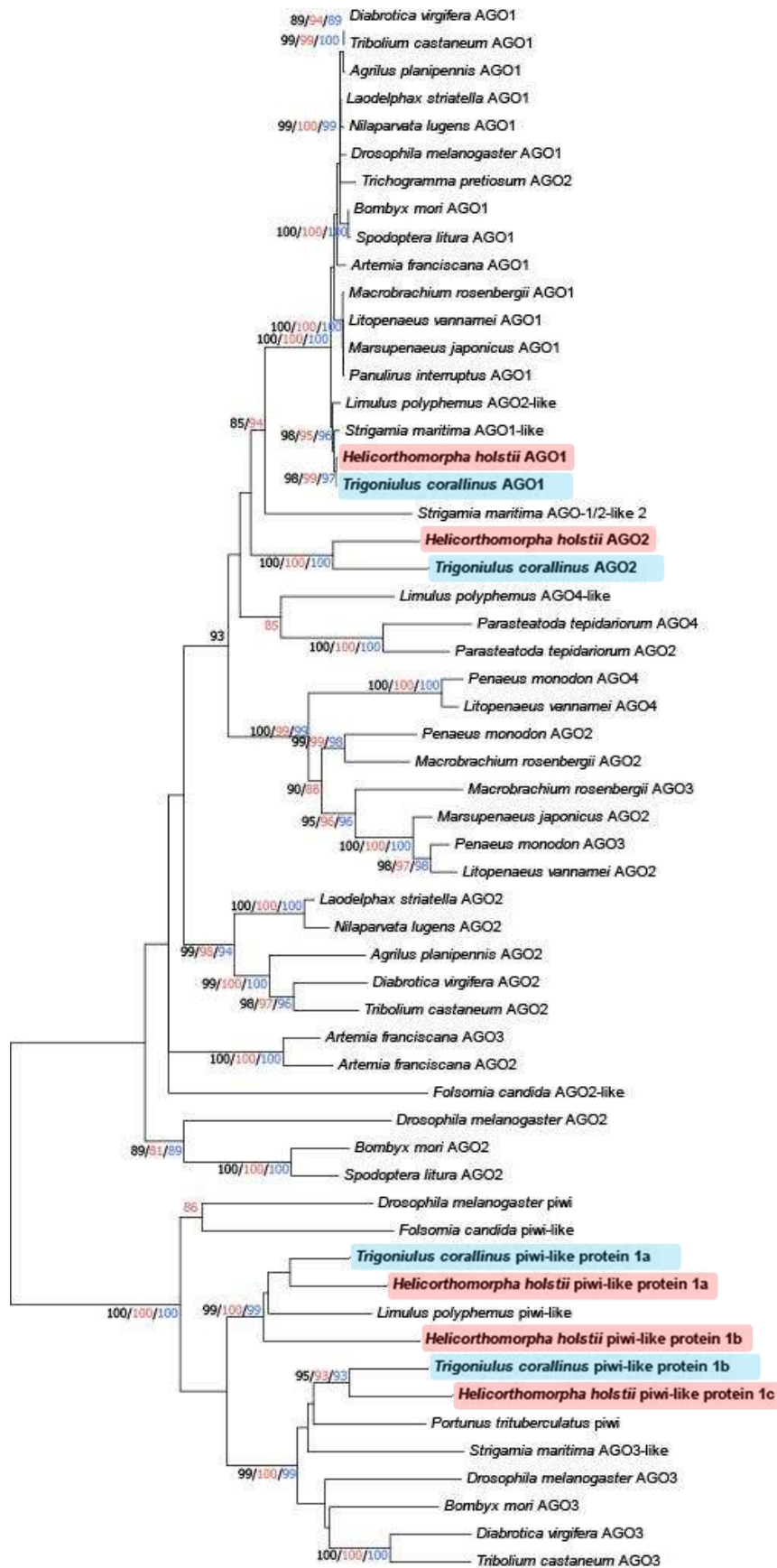

Supplement: S12 Fig — Alignments of protein sequences were made with MUSCLE and the tree built with MEGA 7.0, with 1,000 bootstrap replicates. Maximum likelihood (black), maximum parsimony (red), and neighbour joining (blue) algorithm were adopted, and corresponding high-confidence bootstrap values are shown. The sequence alignment can be found in S8 Data. AGO, Argonaute. (PDF) [file pbio.3000636.s012.pdf]

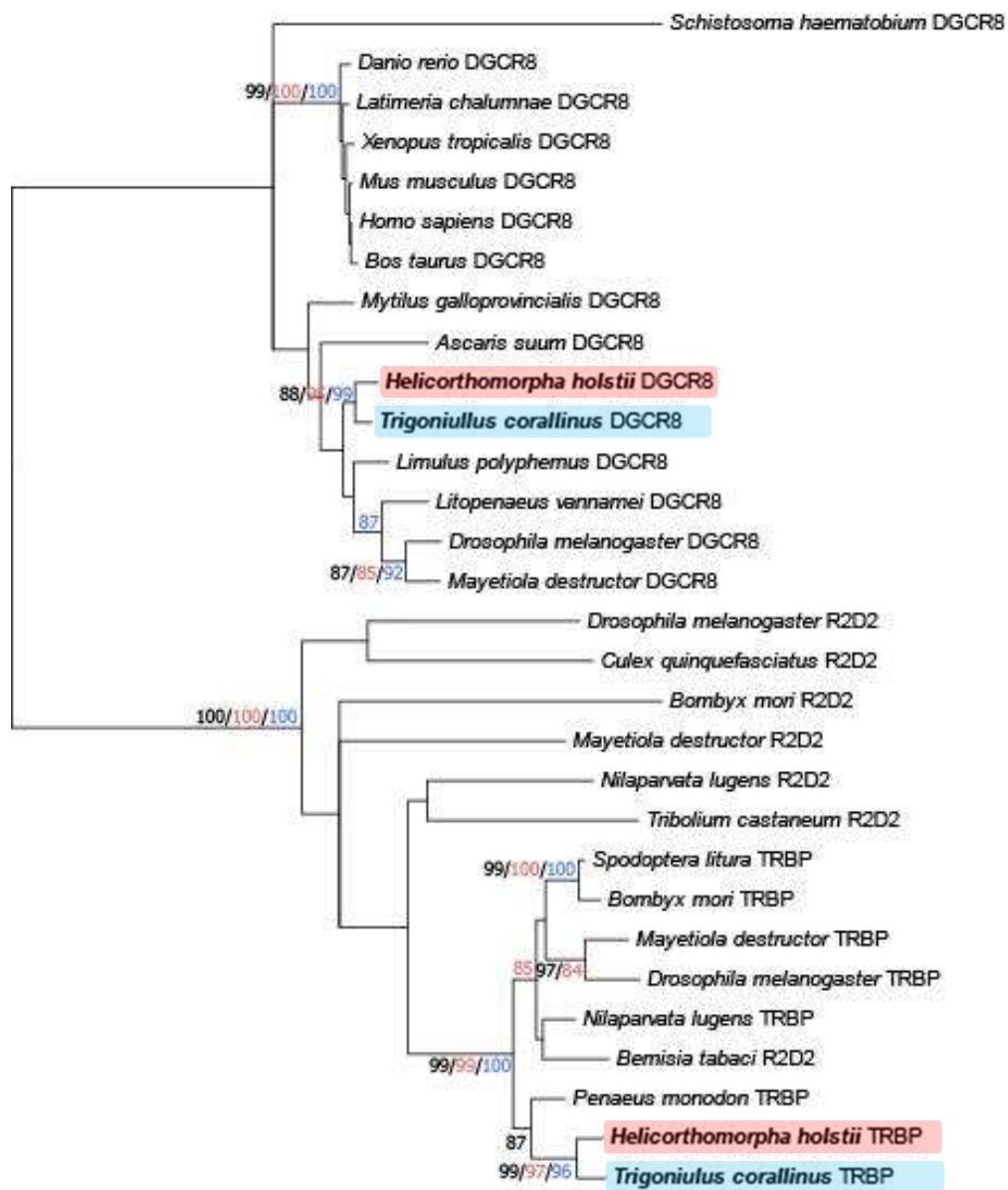

0.50

Supplement: S13 Fig — Alignments of protein sequences were made with MUSCLE and the tree built with MEGA 7.0, with 1,000 bootstrap replicates. Maximum likelihood (black), maximum parsimony (red) and neighbour joining (blue) algorithm were adopted, and corresponding high-confidence bootstrap values are shown. The sequence alignment can be found in S8 Data. (PDF) [file pbio.3000636.s013.pdf]

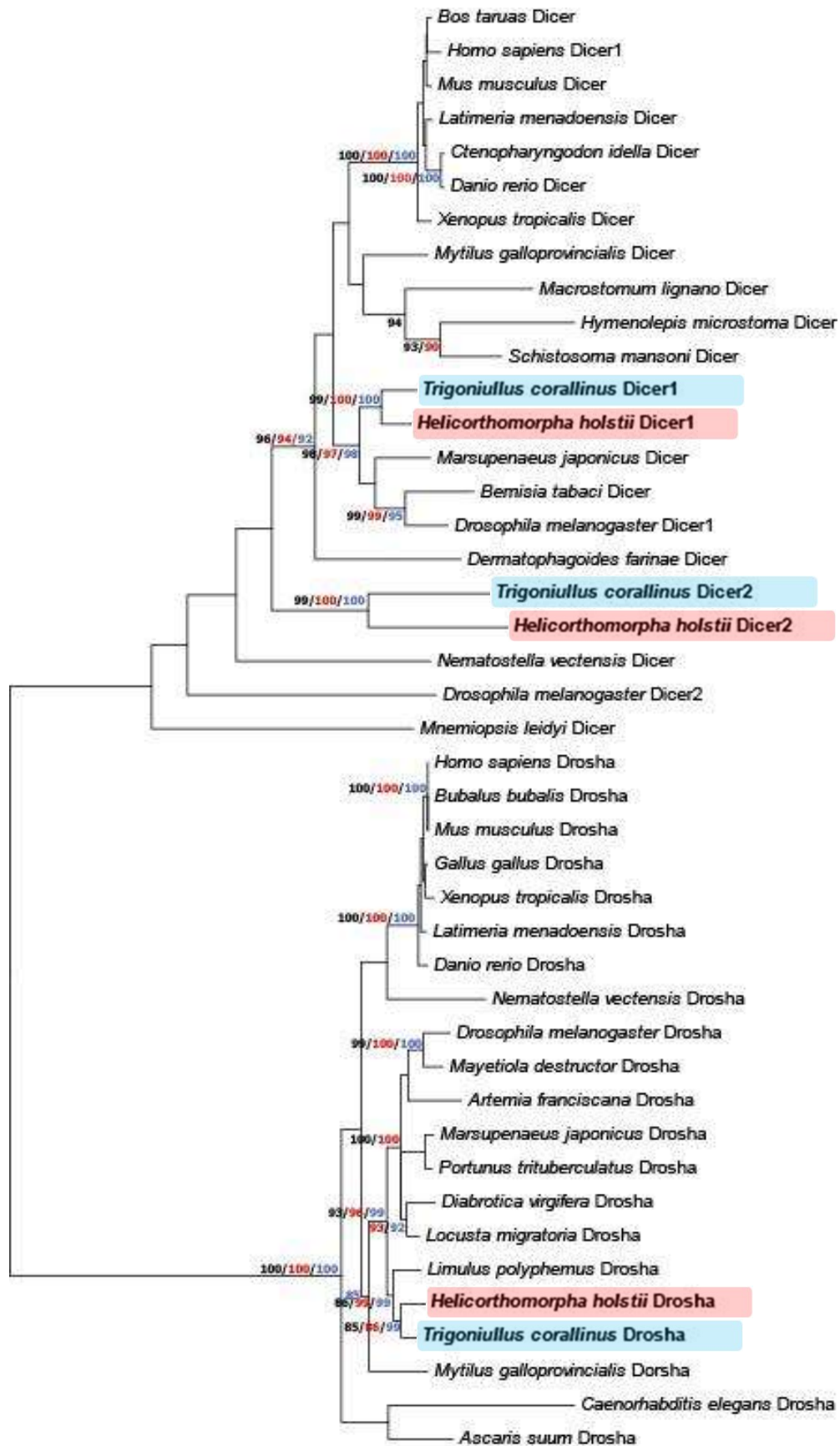

0.50

Supplement: S14 Fig — Alignments of protein sequences were made with MUSCLE and the tree built with MEGA 7.0, with 1,000 bootstrap replicates. Maximum likelihood (black), maximum parsimony (red) and neighbour joining (blue) algorithm were adopted, and corresponding high-confidence bootstrap values are shown. The sequence alignment can be found in S8 Data. (PDF) [file pbio.3000636.s014.pdf]

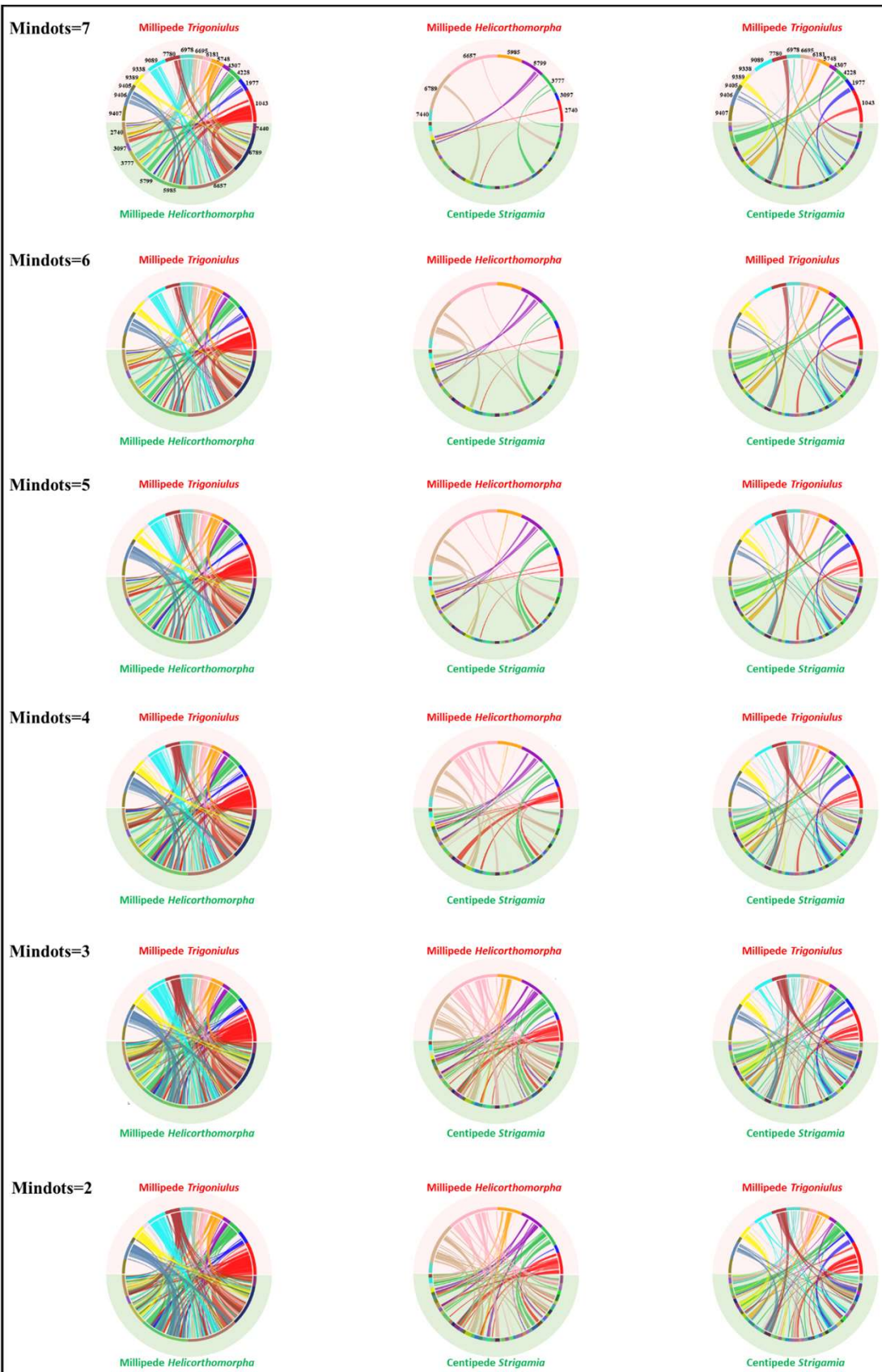

Supplement: S16 Fig — (PDF) [file pbio.3000636.s016.pdf]

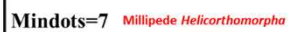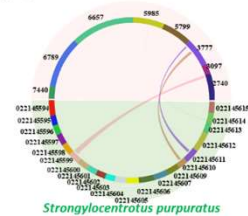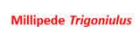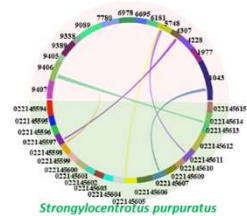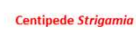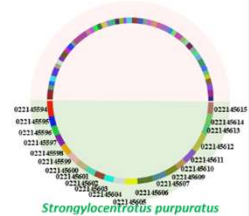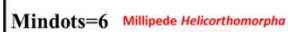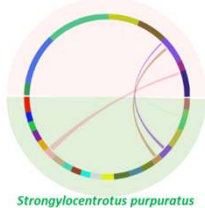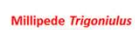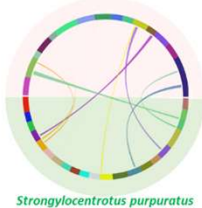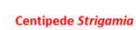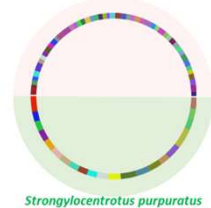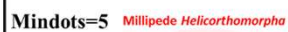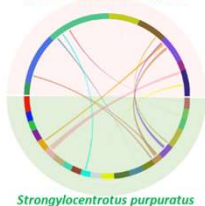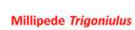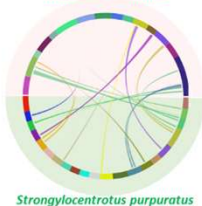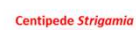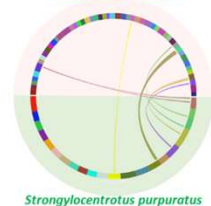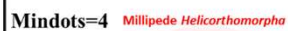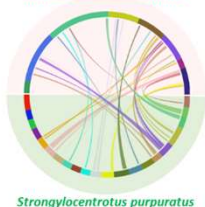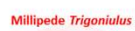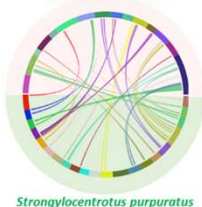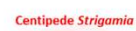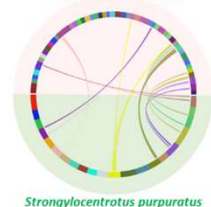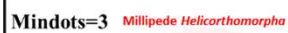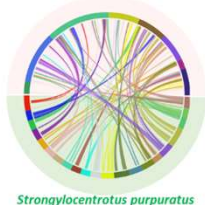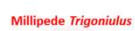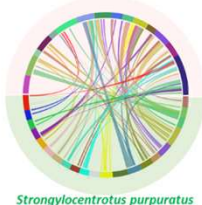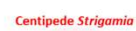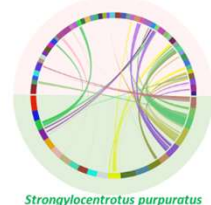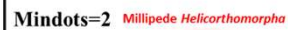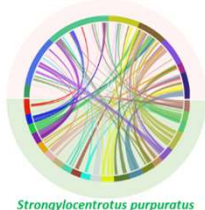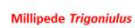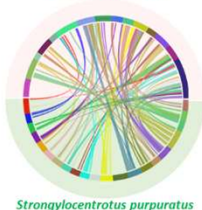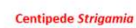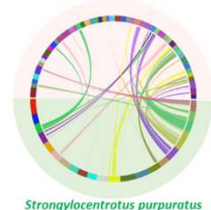

Supplement: S19 Fig — (PDF) [file pbio.3000636.s019.pdf]

**S1 Table. Genome sequencing data information.**

**
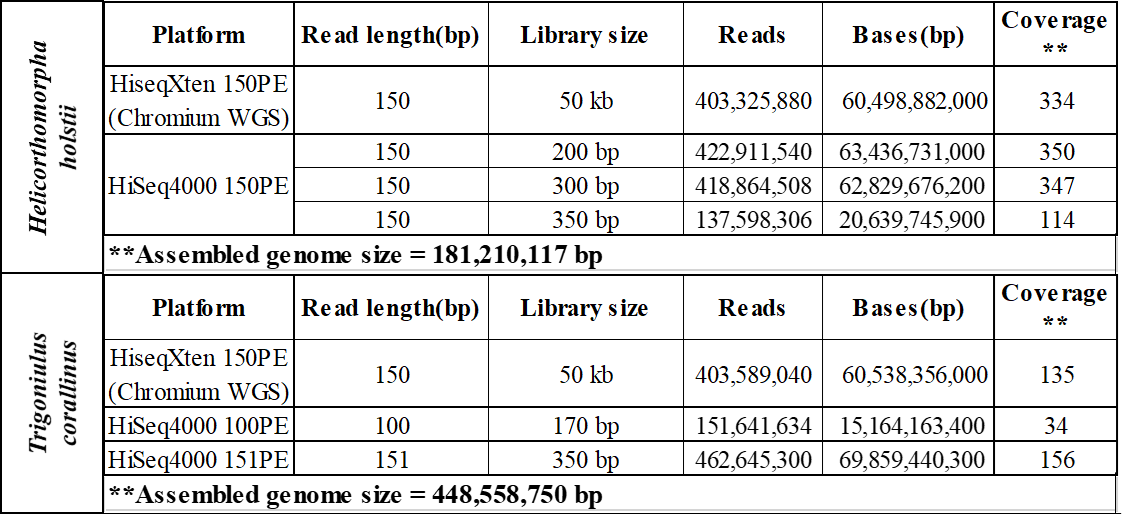
**

Supplement: S1 Table — (DOCX) [file pbio.3000636.s021.docx]
