# Supplementary material for: Millipede genomes reveal unique adaptations during myriapod evolution
Source: PLoS Biol. 2020 Sep 29;18(9):e3000636. doi: 10.1371/journal.pbio.3000636 (PMC7523956; doi:10.1371/journal.pbio.3000636)
Supplement: S2 Fig — Specific gains of homeobox genes between myriapods and insects (a) and between centipede and millipedes (b). A total of 108 and 105 homeobox genes could be identified in the genomes of H. holstii and T. corallinus, respectively, which is comparable to the 112 homeobox genes that could be identified in the centipede S. maritima [9]. (PDF) [file pbio.3000636.s002.pdf]

a)

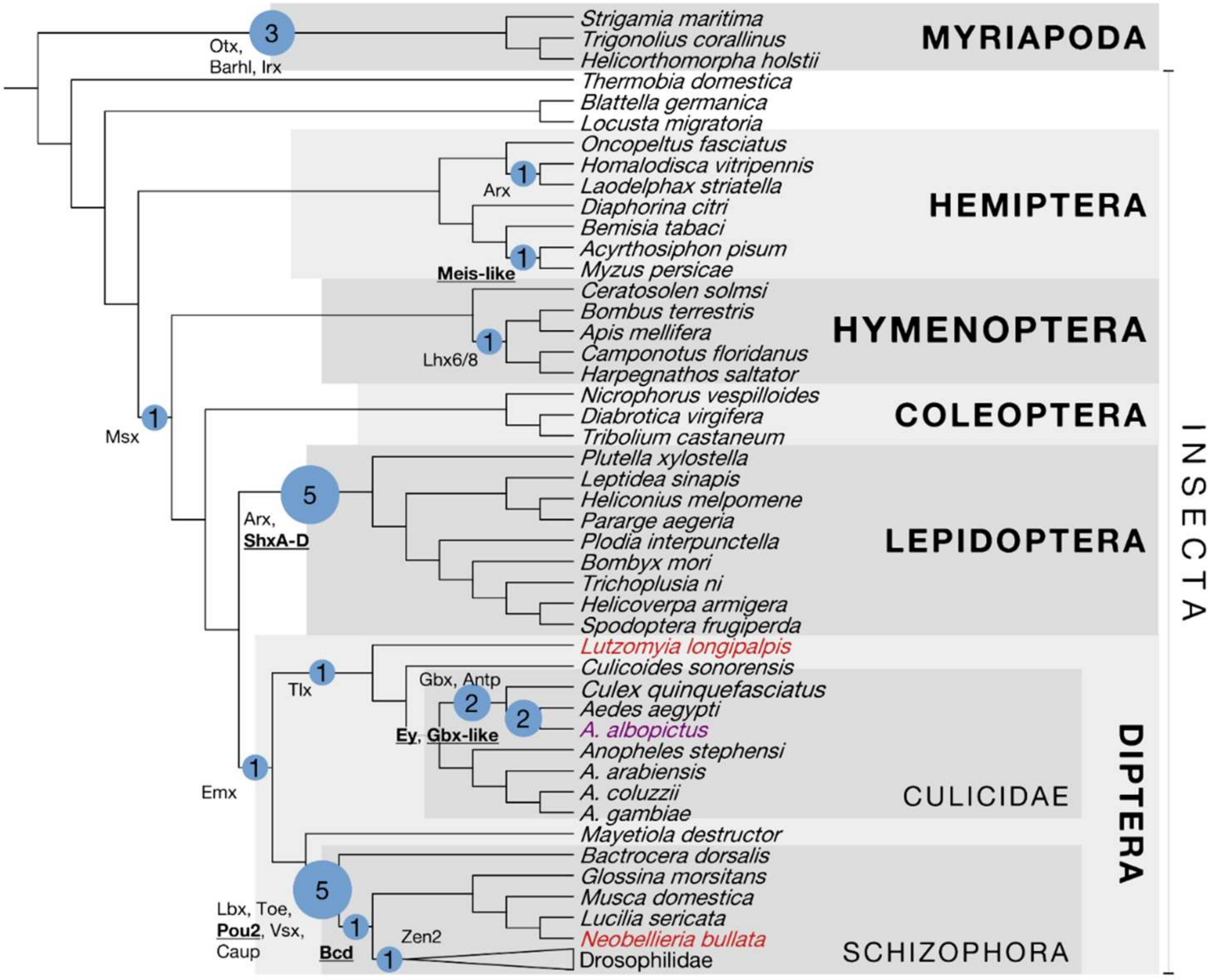

b)

	Hox3	*Bap*	*Abox*	*Vax*	*Lag*	*Awh*	*Toy*	*Prop*	*Unc4*	*Vsx*	*Pros*	*Msx/x*	*Mkx*	*Ind*	*Ap*	*Arx*	*Cad*	*Vnd*	*Eve*	*Btn*	*Otd*	*Zfh2/Zfhx*	*Not*	*Unknowns*
H. holstii	0	3	2	0	2	2	0	3	1	2	2	0	0	2	1	1	1	1	1	1	2	1	3	2
T. corallinus	2	1	1	1	1	1	1	1	2	1	1	0	0	1	2	2	1	1	1	1	2	1	1	1
S. maritima	2	1	1	1	1	1	1	1	2	1	1	1	1	2	1	1	3	2	3	2	3	1*	(2)	4
