# Supplementary material for: Millipede genomes reveal unique adaptations during myriapod evolution
Source: PLoS Biol. 2020 Sep 29;18(9):e3000636. doi: 10.1371/journal.pbio.3000636 (PMC7523956; doi:10.1371/journal.pbio.3000636)
Supplement: S15 Fig — (PDF) [file pbio.3000636.s015.pdf]

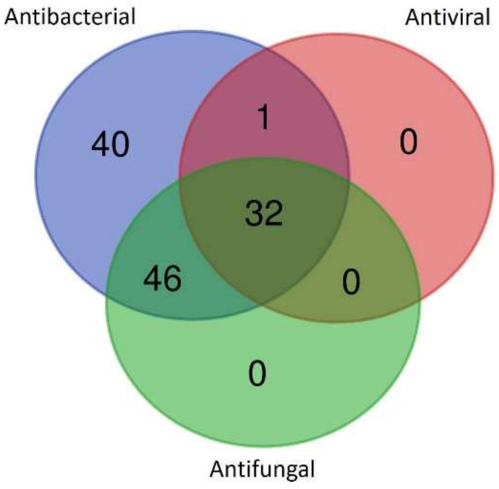

Names	total	elements
Antibacterial (AB)<br>Antifungal (AF)<br>Antiviral (AV)	32	Tco_000311-T1 Tco_001915-T1 Tco_002832-T1 Tco_001251-T1 Tco_016249-T1 Tco_001420-T1 Tco_001070-T1 Tco_002615-T1 Tco_000239-T1 Tco_005301-T1 Tco_015501-T1 Tco_015373-T1 Tco_001162-T1 Tco_001451-T1 Tco_001163-T1 Tco_002091-T1 Tco_002752-T1 Tco_003228-T1 Tco_000540-T1 Tco_001185-T1 Tco_000906-T1 Tco_000890-T1 Tco_001951-T1 Tco_002568-T1 Tco_002594-T1 Tco_001143-T1 Tco_002114-T1 Tco_000341-T1 Tco_002741-T1 Tco_017346-T1 Tco_002380-T1 Tco_000309-T1
Antibacterial (AB)<br>Antiviral (AV)	1	Tco_001406-T1
Antibacterial (AB)<br>Antifungal (AF)	46	Tco_003182-T1 Tco_001167-T1 Tco_001409-T1 Tco_002698-T1 Tco_000576-T1 Tco_002039-T1 Tco_002228-T1 Tco_003041-T1 Tco_000156-T1 Tco_001113-T1 Tco_015031-T1 Tco_002327-T1 Tco_002043-T1 Tco_001581-T1 Tco_000147-T1 Tco_000689-T1 Tco_000888-T1 Tco_001757-T1 Tco_000440-T1 Tco_000541-T1 Tco_000208-T1 Tco_000683-T1 Tco_001885-T1 Tco_000684-T1 Tco_003294-T1 Tco_000373-T1 Tco_000605-T2 Tco_003032-T1 Tco_001985-T1 Tco_001393-T1 Tco_015801-T1 Tco_001446-T1 Tco_018662-T1 Tco_001527-T1 Tco_001751-T1 Tco_001107-T1 Tco_000860-T1 Tco_001096-T2 Tco_002007-T1 Tco_002083-T1 Tco_002932-T1 Tco_001750-T2 Tco_002587-T1 Tco_000972-T1 Tco_002264-T1 Tco_001452-T1
Antibacterial (AB)	40	Tco_002735-T1 Tco_000913-T1 Tco_000969-T1 Tco_001522-T2 Tco_001613-T1 Tco_001905-T1 Tco_001157-T1 Tco_002758-T1 Tco_015819-T1 Tco_001470-T1 Tco_001156-T1 Tco_001457-T2 Tco_000441-T1 Tco_000682-T1 Tco_002715-T1 Tco_012839-T1 Tco_002770-T1 Tco_000607-T1 Tco_002292-T1 Tco_001582-T1 Tco_000189-T1 Tco_001455-T1 Tco_019078-T2 Tco_001036-T1 Tco_001241-T1 Tco_009231-T1 Tco_001242-T1 Tco_004944-T1 Tco_001249-T1 Tco_001467-T1 Tco_002216-T1 Tco_001198-T1 Tco_000246-T1 Tco_018508-T1 Tco_000393-T1 Tco_001518-T1 Tco_002756-T1 Tco_001074-T1 Tco_001590-T1 Tco_001464-T1
