# Supplementary material for: Millipede genomes reveal unique adaptations during myriapod evolution
Source: PLoS Biol. 2020 Sep 29;18(9):e3000636. doi: 10.1371/journal.pbio.3000636 (PMC7523956; doi:10.1371/journal.pbio.3000636)
Supplement: S18 Fig — (PDF) [file pbio.3000636.s018.pdf]

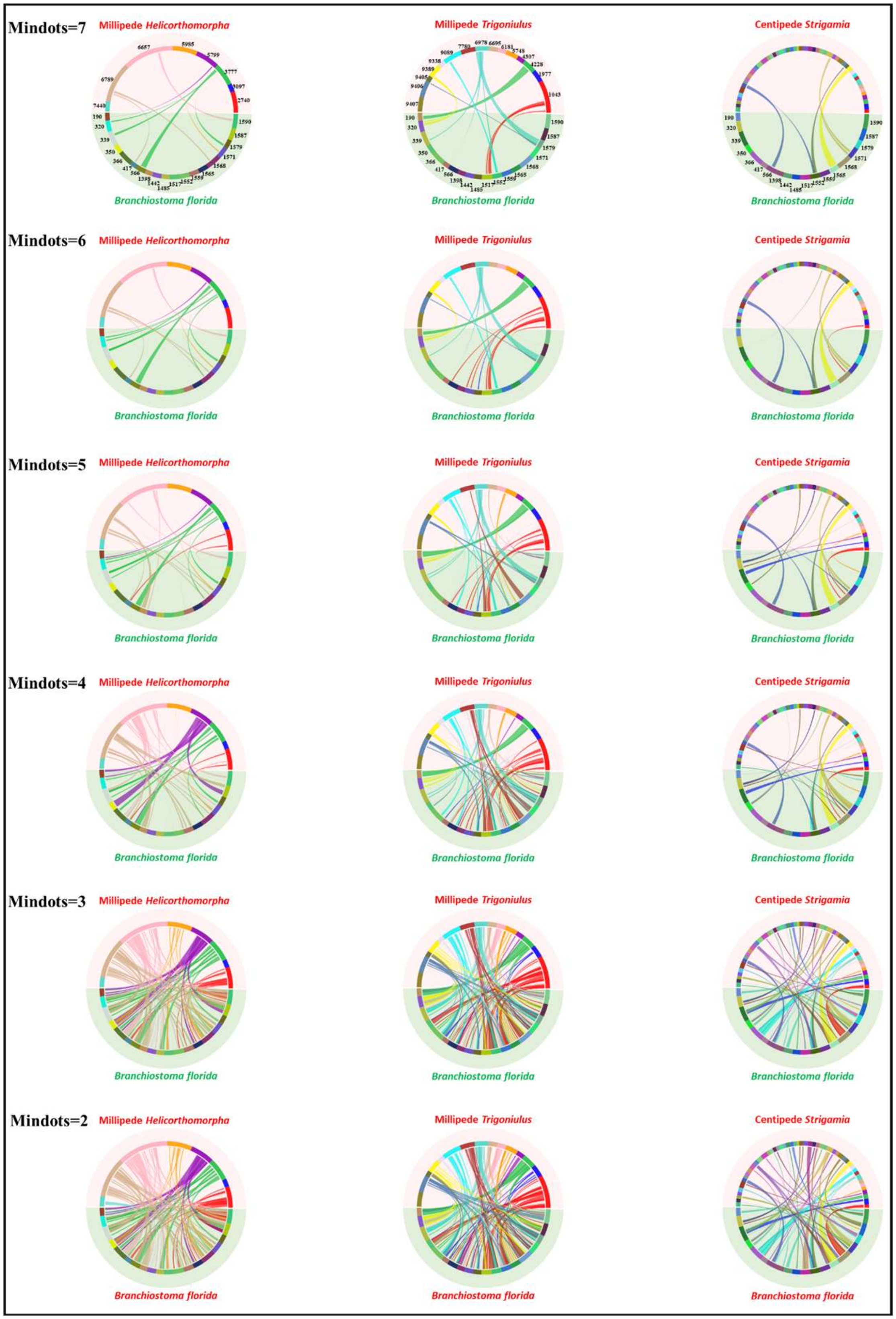
Mindots=7
Millipede Helicorthomorpha
Millipede Trigoniulus
Centipede Strigamia
Branchiostoma florida
Mindots=6
Mindots=5
Mindots=4
Mindots=3
Mindots=2
