# Supplementary material for: Millipede genomes reveal unique adaptations during myriapod evolution
Source: PLoS Biol. 2020 Sep 29;18(9):e3000636. doi: 10.1371/journal.pbio.3000636 (PMC7523956; doi:10.1371/journal.pbio.3000636)
Supplement: S2 Table — (DOCX) [file pbio.3000636.s022.docx]

**S2 Table. Genome size prediction by GenomeScope.**

| ***H. holstii* genome size:** 159.1 Mb predicted by GenomeScope v2.0 |
| --- |
| Ploidy = 2  K-mer length = 31 |
| property Min Max  Homozygous (aa) 99.0097% 99.0386%  Heterozygous (ab) 0.961443% 0.990263%  Genome Haploid Length 158,547,979 bp 159,123,826 bp  Genome Repeat Length 18,970,309 bp 19,039,209 bp  Genome Unique Length 139,577,670 bp 140,084,616 bp  Model Fit 91.7075% 95.1623%  Read Error Rate 1.29169% 1.29169% |
| ***T. corallinus* genome size:** 437.4 Mb predicted by GenomeScope v2.0 |
| Ploidy = 2  K-mer length = 31 |
| property Min Max  Homozygous (aa) 99.7103% 99.7606%  Heterozygous (ab) 0.239431% 0.289747%  Genome Haploid Length 435,824,399 bp 437,430,062 bp  Genome Repeat Length 105,678,821 bp 106,068,162 bp  Genome Unique Length 330,145,578 bp 331,361,900 bp  Model Fit 82.3667% 97.8995%  Read Error Rate 0.848443% 0.848443% |
