# Supplementary material for: Millipede genomes reveal unique adaptations during myriapod evolution
Source: PLoS Biol. 2020 Sep 29;18(9):e3000636. doi: 10.1371/journal.pbio.3000636 (PMC7523956; doi:10.1371/journal.pbio.3000636)
Supplement: S3 Table — (DOCX) [file pbio.3000636.s023.docx]

**S3 Table. Comparison of transposable elements in the three myriapod genomes.**
