# Supplementary material for: Millipede genomes reveal unique adaptations during myriapod evolution
Source: PLoS Biol. 2020 Sep 29;18(9):e3000636. doi: 10.1371/journal.pbio.3000636 (PMC7523956; doi:10.1371/journal.pbio.3000636)
Supplement: S5 Table — (DOCX) [file pbio.3000636.s025.docx]

**S5 Table. Transcriptome sequencing data information of *Helicorthomorpha holstii* and *Trigoniulus corallinus*.**

**
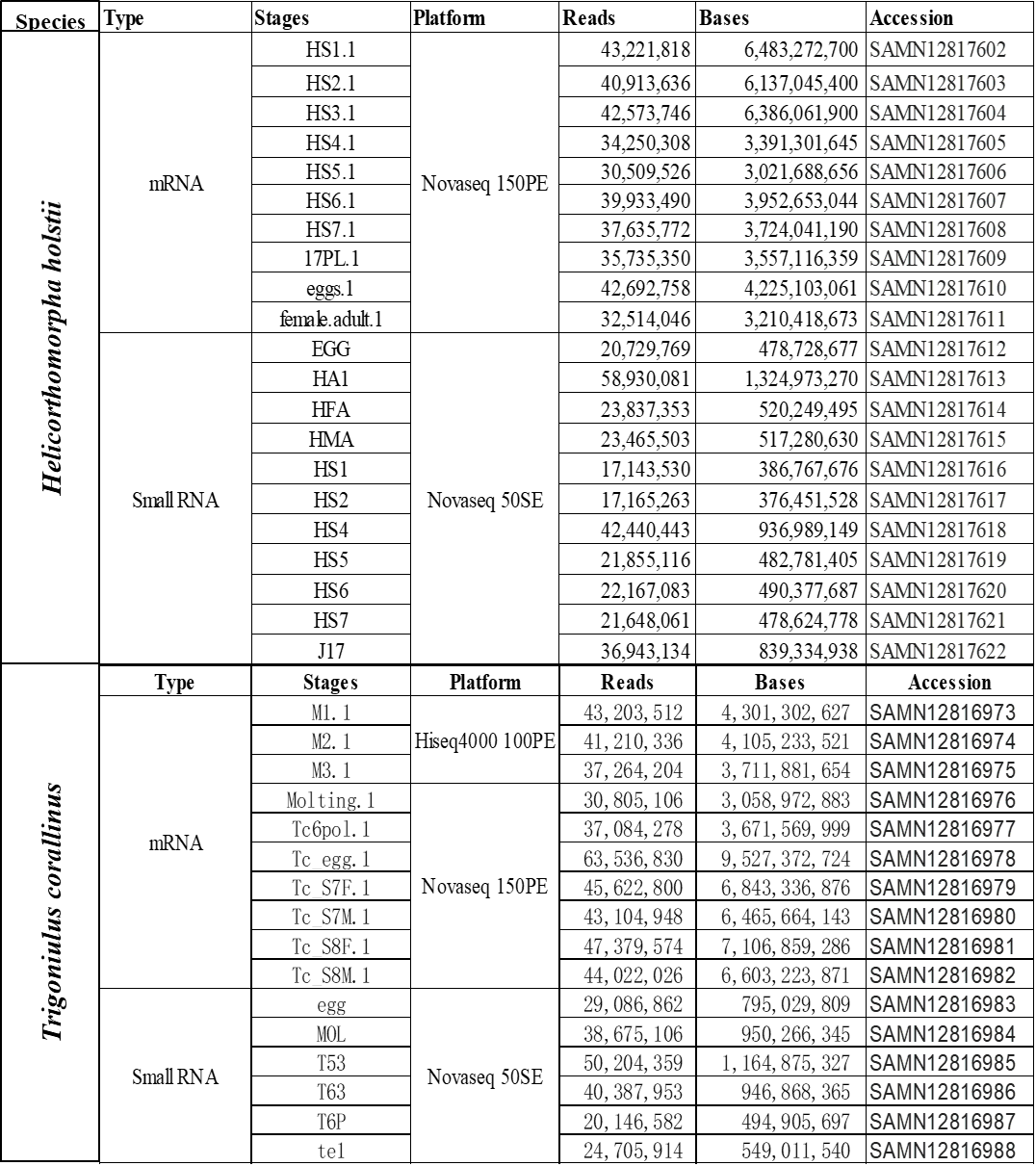
**
