# Supplementary material for: Millipede genomes reveal unique adaptations during myriapod evolution
Source: PLoS Biol. 2020 Sep 29;18(9):e3000636. doi: 10.1371/journal.pbio.3000636 (PMC7523956; doi:10.1371/journal.pbio.3000636)
Supplement: S6 Table — (DOCX) [file pbio.3000636.s026.docx]

**S6 Table. Sequence information of primers used in this study.**

| **Target** | **Forward primer (5’ to 3’)** | **Reverse primer (5’ to 3’)** |
| --- | --- | --- |
| Hho-iab-8 | GCGGAATTCGGTCAACGGTTTGGTCCAGTTTCTT | GCGCTCGAGATCGTTCGCAATCGAGATCGTAAAG |
| Tco-iab-8 | GCGGAATTCTGGTCATCATCTTCAAGTCGTCG | GCGCTCGAGAATTAAGTTCAAATCGGATGCTTTG |
| Hho-mir-2788-1 | GCGGAATTCTTTCTTTTTACGTTGATTTCATTTTGTT | GCGCTCGAGTTTGAAAGTCTGTGATCTGAGTGATGT |
| Hho-mir-2788-2 | GCGGAATTCTGCTAAGTCCCAAGTGACGCTAG | GCGCTCGAGTTTGAAAGTCTGTGATCTGAGTGATGT |
| Hho-mir-2788-3 | GCGGAATTCTGTAACTAAAATAAGACATAGCTGAGATTG | GCGCTCGAGTCAGTGTGCCTGAAGAATGTACTGAG |
| Tca-mir-2788-1 | GCGGAATTCGTCCGACCACATCATCGCATACTCC | GCGCTCGAGGCAACTTAATTGTGCCACTCCTTCG |
| Tca-mir-2788-2 | GCGGAATTCTCAGACTTCGAAACGACACGGAGG | GCGCTCGAGGCAACTTAATTGTGCCACTCCTTCG |
| Hho-abdA | GCCTCGAGCTCTCTATCTCTCGATCTATATCTC | CCGGCGGCCGCAGAATACCCAATACTCCTAAACC |
| Hho-abdB | GCCTCGAGGAAATTCCTATAACTCCCTCCA  GCCTCGAGGTCATCTGAATCACAAATTCATA | CCGGCGGCCGCGTTGCTAATGTCGCATATTCAT  CCGGCGGCCGCCCTTAGATATAATAGCAACAAAAGC |
| Hho-Ubx | GCCTCGAGATCCATCATGCAGGTAGCGTAA | CCGGCGGCCGCACAGTATTATTCTACCGTTGGAGTTG |
| Tco-abdA | GCCTCGAGCCATTCATTGATCGCGTCTAAAT  GCCTCGAGTAGGTCCTATGGTGTTTTTGTCT  GCCTCGAGCATCAAAGGGTGGTATAAACTGA  GCCTCGAGCGGAATTGATAATTATGGTGGAG | CCGGCGGCCGCGGTCAGTATGATAGTGGTCAATA  CCGGCGGCCGCTTCAAAGGATATTTCAACAGTGGG  CCGGCGGCCGCTCGCGTAAAATCAGACTAGGT  CCGGCGGCCGCACATAAGCAACTTACTATCCCC |
| Tco-abdB | GCCTCGAGTATAACATCCAACTGCAAAACAA | CCGGCGGCCGCAATAATAAAAATGGCAACAAAAATATCAG |
| Tco-Ubx | GCCTCGAGCAGCCCACAATGCAGGTAAG | CCGGCGGCCGCCTTTCCTCCAGGAACATTTCACTAA |
