# Supplementary material for: Millipede genomes reveal unique adaptations during myriapod evolution
Source: PLoS Biol. 2020 Sep 29;18(9):e3000636. doi: 10.1371/journal.pbio.3000636 (PMC7523956; doi:10.1371/journal.pbio.3000636)
Supplement: S1 Text — TE, transposable element. (DOCX) [file pbio.3000636.s027.docx]

**S1 Text. Transposable elements.**

Approximately 40% of the 176Mb assembled genome of the centipede *Strigamia maritima* is composed of repetitive DNA (70Mb), with TEs accounting for almost all of this (S3 Table, S2 Data). At 182Mb, the millipede *Helicorthomorpha holstii* has a similar assembled genome size to the centipede *S. maritima*, but a much-reduced repeat content, with repeats accounting for ~19% of its genome (35Mb), again with TEs composing almost all of this (S3 Table, S2 Data). In comparison, the millipede *Trigoniulus corallinus* has an assembled genome size of more than double that of the other two myriapods at 449 Mb, of which ~55% is repeat (245Mb), with TEs accounting for ~47% and other repeats accounting for ~8% of the genome. Thus, the genome of *T. corallinus* has undergone a significant expansion in size and in TE content relative to the other two myriapods.

Analyses of sequence divergence among annotated TEs suggest that all three myriapod genomes have experienced recent spikes in TE activity, however, the specific pattern of activity difference among species. Strikingly, there is evidence of a particularly large and recent expansion of LTR TEs in the centipede *S. maritima*, but very limited evidence for activity prior to this, suggesting a recent invasion of the genome by copia and gypsy LTR TEs)*.* In the millipede *H. holstii*, there is evidence of a much more modest recent expansion of both LTR TEs and DNA TEs, while there is evidence of a more prolonged expansion including SINEs, LINEs, and DNA TEs, but very little LTR TE activity, in the millipede *T. corallinus*.

For all three available myriapod genomes, a large proportion of the total assembled genome consists of unclassified TEs (11-26%,), highlighting the need for more research on TEs in these taxa. In the centipede *S. maritima,* almost all identified repeats are LTR elements, with *copia*-like elements (~6.39%) and *gypsy*-like elements (~4.29%) representing the main LTR TE superfamilies. In the millipede *H. holstii, copia*-like elements (~2.87%) represent the largest identified TE proportion, followed by Maverick DNA TEs (2.77%) and *gypsy*-like elements (~0.62%) (S2 Data). Meanwhile, in the millipede *T. corallinus*, LINE RTE-BovB elements (8.43%) make up the largest proportion of identified repeats, followed by SINE tRNA-Deu elements (4.2%), LINE I elements (3.79%), TcMar-Tc1 DNA TEs (3.17%), LINE CR1 elements (2.73%), hAT-Tip100 DNA TEs (1.99%), *gypsy*-like elements (1.4%) and hAT-Charlie DNA TEs (1.34%) (S2 Data). Unlike the other two genomes, there appear to be very few copia-like elements present in the *T. corallinus* genome, but there is a much more cosmopolitan set among the main identified TEs.

The main repeat types identified differ considerably among the available myriapod genomes. Complex retrotransposons, particularly *copia*-like and *gypsy*-like long-terminal repeat (LTR) TEs are the dominant TE class present in the genome of the centipede *S. maritima*, while Maverick DNA TEs are also dominant in the genome of the millipede *H. holstii* (S2 Data). Meanwhile, a more cosmopolitan set of TEs are identified in the genome of *T. corallinus,* including SINEs, LINEs, DNA TEs and *gypsy*-like LTR TEs.

Kimura distance-based divergence analyses suggest varying patterns of TE activity among sequenced myriapod genomes, but there is an overall indication of a recent increase in activity across all three genomes. In the centipede *S. maritima* there is evidence of a recent exponential increase in transposon activity, with the most frequent TE sequence divergence relative to consensus being 1-2% (Figure 2, Repeat Landscapes). However, there is a drop-in divergence at the 0% Kimura substitution level, suggesting that this large burst in activity is slowing down (Figure 2, Repeat Landscapes). Divergence peaks were most pronounced in LTR elements and unclassified elements, reflecting the composition of repeat classes in the *S. maritima* genome. There is evidence of a similar recent burst of activity for the millipede *H. holstii*, but at a reduced scale and with a greater contribution from DNA elements, although LTR and unclassified elements remain a major contributor, again representing the TE composition of the genome (Figure 2, Repeat Landscapes). The pattern is more complex for the millipede *T. corallinus*, in line with the more diverse repeat content of its genome. The repeat landscape for *T. corallinus* suggests a more sustained period of transposon activity, across a larger set of TE classes, especially TcMar and hAT DNA TEs, unclassified TEs, RTE and Jockey-I LINEs, and Deu SINEs, along with some lesser evidence of recent gypsy LTR activity. Again, TE activity appears to be tailing off in *T. corallinus*, with a reduced proportion of TEs represented at the 0% Kimura substitution level.

The detailed transposable elements annotations for each of *H. holstii* and *T. corallinus* genomes are shown in S2 Data. Comparison of the contents between the two millipede genomes to the centipede genome are shown in S3 Table.
