# Supplementary material for: Millipede genomes reveal unique adaptations during myriapod evolution
Source: PLoS Biol. 2020 Sep 29;18(9):e3000636. doi: 10.1371/journal.pbio.3000636 (PMC7523956; doi:10.1371/journal.pbio.3000636)
Supplement: S2 Text — (DOCX) [file pbio.3000636.s028.docx]

**S2 Text. MicroRNAs**

The sequences, location and arm usage of conserved and novel microRNAs of *H. holistii* and *T. corallinus* are shown in S4 Data. The detailed information of the predicted microRNA hairpin structure with mapped reads of *H. holistii* and *T. corallinus* are shown in S5-6 Data.

**MicroRNA arm switching**

Many miRNA loci produce significant quantities of mature miRNAs from both arms with different amounts (5p or 3p), and RISC-loaded miRNA will then bind complementary to the 3’UTR of mRNA. These result in the suppression of gene expression by either, promoting mRNA cleavage, translational repression, or decay due to deadenylation (Ghildiyal and Zamore, 2009). In animals, complementary base pairing of nucleotides 2 to 8 in the 5’ of the miRNA (also known as the seed region) is pivotal and efficient for targeting to mRNA (Krol et al 2010; Griffiths-Jones et al 2011). Since the sequences of alternative mature miRNAs derived from opposite complimentary arms are different, mature miRNAs derived from the same hairpin will also regulate distinct sets of genes (Marco et al 2010, 2012; Berezikov 2011; Griffiths-Jones et al 2011). Interestingly, the choice of dominant arm expression can be swapped at different situations, a termed known as microRNA arm switching, such as a-b) developmental stages, tissues (e.g. Ro et al 2007; Ruby et al 2007; Glazov et al 2008; Chiang et al 2010; Jagadeeswaran et al 2010; Cloonan et al 2011; Biryukova et al 2014; Gong et al 2014; Pundhir and Goodkin 2015); c) pathological statuses (e.g. gastric cancer, Li et al 2012); and d) species (e.g. de Wit et al 2009; Marco et al 2010; Griffiths-Jones et al 2011; Brawand et al 2014; Sadd et al 2015). Comparing conserved microRNAs between the two millipedes and also all available insect genomes with small RNA sequencing data, we found multiple cases of microRNAs undergoing microRNA arm switching, including let-7 and miR-277 (S9 Fig).

To decipher how microRNA arm switching could potentially contribute to evolution between myriapods and insects, we focused on the two microRNAs iab-8 and miR-2788, which were previously only known from insects but are now also known to be conserved in centipede and millipedes (Chipman et al 2014; this study). Using the sensor assays, we found no obvious arm target repression preference of iab-8 of the two millipedes, but the *Drosophila* iab-8 have higher 5p dominant arm and target repression ability (S10 Fig). These data suggest that different arm usage of iab-8 has evolved between insects and myriapods. In the small RNA sequencing of the beetle *Tribolium castaneum* cell line and different developmental stages of millipedes *H. holstii*, miR-2788 shows different arm preferences (5p dominance in *T. castaneum* and 3p dominance in *H. holstii*) (S11 Fig).

To test the targeting properties of *T. castaneum* and *H. holstii* miR-2788, a set of luciferase reporters containing perfect target sites for both 5p and 3p mature miR-2788 were constructed and co-transfected into S2 cells, with expression constructs driving production of either species’ miR-2788. Repression of target sites for the 5p and 3p arms of each hairpin was found to correlate with their relative production as determined by next-generation sequencing (S11 Fig). As sequence conservation outside microRNA hairpin sequences in the flanking sequences have been identified across species (Kenny et al 2015), and the dominant usage of arms of microRNA candidates such as miR-10 in insects are not governed by thermodynamics (Griffiths-Jones et al 2011), microRNA flanking sequence has been suggested as a potential candidate to govern arm switching (Griffiths-Jones et al 2011; Kenny et al 2015). The flanking sequences of both *T. castaneum* and *H. holstii* miR-2788 were deleted and transfected to S2 cells for luciferase reporter assays, and our results revealed that the same dominant arm is associated with various flanking sequences (S11 Fig). This suggests that the governance of microRNA arm switching could be under multiple mechanisms, and candidate-specific during evolution, presenting an additional means of adaptation.

**References:**

Berezikov E. Evolution of microRNA diversity and regulation in animals. Nat Rev Genet. 2011;12: 846-860.

Biryukova I, Ye T, Levashina E. Transcriptome-wide analysis of microRNA expression in the malaria mosquito *Anopheles gambiae*. BMC Genomics. 2014;15(1): 557.

Brawand D, Wagner CE, Li YI, Malinsky M, Keller I, Fan S, et al. The genomic substrate for adaptive radiation in African cichlid fish. Nature. 2014;513: 375.

Chiang HR, Schoenfeld LW, Ruby JG, Auyeung VC, Spies N, Baek D, et al. Mammalian microRNAs: experimental evaluation of novel and previously annotated genes. Genes Dev. 2010;24(10): 992-1009.

Chipman AD, Ferrier DEK, Brena C, Qu J, Hughes DST, Schröder R, et al. The First Myriapod Genome Sequence Reveals Conservative Arthropod Gene Content and Genome Organisation in the Centipede *Strigamia maritima*. PLoS Biol. 2014;12(11): e1002005.

Cloonan N, Wani S, Xu Q, Gu J, Lea K, Heater S, et al. MicroRNAs and their isomiRs function cooperatively to target common biological pathways. Genome biology. 2011;12(12): R126.

de Wit E, Linsen SEV, Cuppen E, Berezikov E. Repertoire and evolution of miRNA genes in four divergent nematode species. Genome Research. 2009;19(11): 2064-74.

Fontanetti CS, Campos KA, Prado RA, da Silva Souza T. Cytogenetic Studies in Diplopoda. Cytologia. 2002;67(3): 253-60.

Ghildiyal M, Zamore PD. Small silencing RNAs: an expanding universe. Nat Rev Genet. 2009;10(2): 94-108.

Glazov EA, Cottee PA, Barris WC, Moore RJ, Dalrymple BP, Tizard ML. A microRNA catalog of the developing chicken embryo identified by a deep sequencing approach. Genome Res. 2008;18(6): 957-64.

Gong J, Wu Y, Zhang X, Liao Y, Sibanda VL, Liu W, et al. Comprehensive analysis of human small RNA sequencing data provides insights into expression profiles and miRNA editing. RNA Biol. 2014;11(11): 1375-85.

Griffiths-Jones S, Hui JHL, Marco A, Ronshaugen M. MicroRNA evolution by arm switching. EMBO Rep. 2011;12(2): 172-7.

Jagadeeswaran G, Zheng Y, Sumathipala N, Jiang H, Arrese EL, Soulages JL, et al. Deep sequencing of small RNA libraries reveals dynamic regulation of conserved and novel microRNAs and microRNA-stars during silkworm development. BMC Genomics. 2010;11: 52-69.

Kenny NJ, Sin YW, Hayward A, Paps J, Chu KH, Hui JHL. The phylogenetic utility and functional constraint of microRNA flanking sequences. Proc R Soc B. 2015;282(1803): 20142983.

Krol J, Loedige I, Filipowicz W. The widespread regulation of microRNA biogenesis, function and decay. Nat Rev Genet. 2010;11(9): 597-610.

Li Z, Huang H, Chen P, He M, Li Y, Arnovitz S, et al. miR-196b directly targets both HOXA9/MEIS1 oncogenes and FAS tumour suppressor in MLL-rearranged leukaemia. Nature Commun. 2012;3: 688.

Marco A, Hui JH, Ronshaugen M, Griffiths-Jones S. Functional shifts in insect microRNA evolution. Genome Biol Evol. 2010;2: 686-96.

Marco A, Hooks K, Griffiths-Jones S. Evolution and function of the extended miR-2 microRNA family. RNA Biol. 2012;9(3): 242-8.

Pundhir S, Gorodkin J. Differential and coherent processing patterns from small RNAs. Sci Rep. 2015;5: 12062.

Ro S, Park C, Young D, Sanders KM, Yan W. Tissue-dependent paired expression of miRNAs. Nucleic Acids Res. 2007;35(17): 5944-53.

Ruby JG, Stark A, Johnston WK, Kellis M, Bartel DP, Lai EC. Evolution, biogenesis, expression, and target predictions of a substantially expanded set of *Drosophila* microRNAs. Genome Res. 2007;17(12):1850-64.

Sadd BM, Barribeau SM, Bloch G, de Graaf DC, Dearden P, Elsik CG, et al. The genomes of two key bumblebee species with primitive eusocial organization. Genome Biol. 2015;16(1): 76.
