# Supplementary material for: Millipede genomes reveal unique adaptations during myriapod evolution
Source: PLoS Biol. 2020 Sep 29;18(9):e3000636. doi: 10.1371/journal.pbio.3000636 (PMC7523956; doi:10.1371/journal.pbio.3000636)
Supplement: S6 Data — (PDF) [file pbio.3000636.s034.pdf]

Provisional ID	:	Scaffold_1043_1001
Score total	:	601296.3
Score for star read(s)	:	3.9
Score for read counts	:	601286.3
Score for mfe	:	1.5
Score for randfold	:	1.6
Score for cons. seed	:	3
Total read count	:	1179409
Mature read count	:	1135796
Loop read count	:	0
Star read count	:	43613

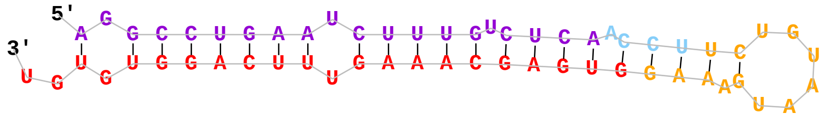

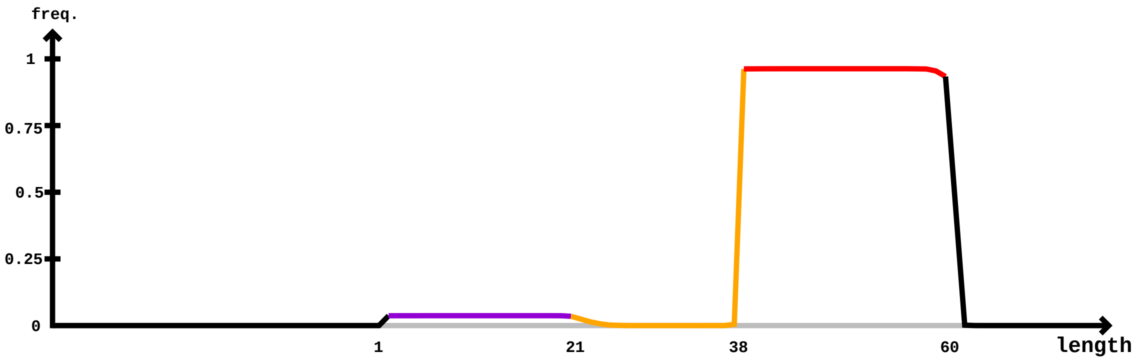

Star Mature

```
5'- uaucuccaucauuuucaugcgaaacuuucuggcaggccugaaucuuugucucaaccuucuguaaugaaaggugagcaaaguuucaggugugu uuggggacuucuuuu   -3'   obs
    uaucuccaucauuuucaugcgaaacuuucuggcaggccugaaucuuugucucaaccuucuguaaugaaaggugagcaaaguuucaggugugu uuggggacuucuuuu         exp
    ..(((((.....((((....))))......((((.(((((((.(((((.((((.(((((......).))))))))))))).))))))).))))..)))))........   reads   mm   sample
    ................................Uaggccugaaucuuugucuc.......................................................   1   1   T63
    ................................Uaggccugaaucuuugucuca......................................................   1   1   T63
    .................................aggccugaaucuuugucG........................................................   1   1   T63
    .................................aggccuAaaucuuugucu........................................................   1   1   T63
    .................................aggccugaaucuuAgucu........................................................   1   1   T63
    .................................aggccugaaucuuuguUu........................................................   1   1   T63
    .................................aggccugaaucuuugucA........................................................   16   1   T63
    .................................aggccugaaucuuugucu........................................................   54   0   T63
    .................................agCccugaaucuuugucuc.......................................................   1   1   T63
    .................................aggccugaaucuuugucuU.......................................................   5   1   T63
    .................................aggccugaauGuuugucuc.......................................................   1   1   T63
    .................................aggccugaaucuuugucuc.......................................................   857   0   T63
    .................................aggccCgaaucuuugucuc.......................................................   1   1   T63
    .................................aggccugaaucuuAgucuc.......................................................   1   1   T63
    .................................aggccuCaaucuuugucuc.......................................................   1   1   T63
    .................................aggccugaaucuuugucuA.......................................................   8   1   T63
    .................................aggccugaauUuuugucuc.......................................................   1   1   T63
    .................................aggccugaUucuuugucuc.......................................................   2   1   T63
    .................................aggccugaUucuuugucuca......................................................   3   1   T63
    .................................aggUcugaaucuuugucuca......................................................   4   1   T63
    .................................aggccugaaCcuuugucuca......................................................   2   1   T63
    .................................aggccugaaucuuugucuAa......................................................   2   1   T63
    .................................aggccAgaaucuuugucuca......................................................   4   1   T63
    .................................aggccGgaaucuuugucuca......................................................   1   1   T63
    .................................aggccuAaaucuuugucuca......................................................   2   1   T63
    .................................aCgccugaaucuuugucuca......................................................   1   1   T63
    .................................aggccugaaucuuugucucU......................................................   8   1   T63
    .................................aggccugaaucuuugucucC......................................................   1   1   T63
    .................................aggccCgaaucuuugucuca......................................................   1   1   T63
    .................................aggccugaaucuuugucuca......................................................   4649   0   T63
    .................................aggcUugaaucuuugucuca......................................................   1   1   T63
    .................................aggccugaaucuuAgucuca......................................................   5   1   T63
    .................................aggccugaaucuCugucuca......................................................   2   1   T63
```

Star Mature

```
uaucuccaucauuuucaugcgaaacuuucuggcaggccugaaucuuugucucaaccuucuguaaugaaaggugagcaaaguuucaggugugguuuggggacuucuuuu
.................................aggccugaaucuuugucuGa......................................................  1     1   T63
.................................Cggccugaaucuuugucuca......................................................  1     1   T63
.................................aggccugaaucAuugucuca......................................................  1     1   T63
.................................Gggccugaaucuuugucuca......................................................  1     1   T63
.................................aggccugaaucuuGgucuca......................................................  2     1   T63
.................................aggccugaaucuuuguUuca......................................................  1     1   T63
.................................aggccuCaaucuuugucuca......................................................  1     1   T63
.................................aggccugUaucuuugucuca......................................................  2     1   T63
.................................aggccugaaucuuugucuUa......................................................  2     1   T63
.................................agAccugaaucuuugucuca......................................................  4     1   T63
.................................aggccugaaucCuugucuca......................................................  1     1   T63
.................................aggccuUaaucuuugucuca......................................................  2     1   T63
.................................aggccugaaucuuugucucG......................................................  2     1   T63
.................................aggccugaaucuuCgucucaa.....................................................  1     1   T63
.................................aggccugaaucuCugucucaa.....................................................  1     1   T63
.................................aggccugaaucuuuguGucaa.....................................................  1     1   T63
.................................aggccugaaucuAugucucaa.....................................................  1     1   T63
.................................agCccugaaucuuugucucaa.....................................................  1     1   T63
.................................aggUcugaaucuuugucucaa.....................................................  3     1   T63
.................................aggccugaaucuuugucucaU.....................................................  45    1   T63
.................................aggccugaaucuuugAcucaa.....................................................  1     1   T63
.................................aggccugaaucCuugucucaa.....................................................  5     1   T63
.................................aggccugaaucuuugucuAaa.....................................................  1     1   T63
.................................aggccugaaucuuugucucaa.....................................................  2688  0   T63
.................................aggccuCaaucuuugucucaa.....................................................  1     1   T63
.................................Gggccugaaucuuugucucaa.....................................................  1     1   T63
.................................aggccCgaaucuuugucucaa.....................................................  2     1   T63
.................................aggccugaaucuuugucucaG.....................................................  1     1   T63
.................................aUgccugaaucuuugucucaa.....................................................  1     1   T63
.................................aggccugaaucuuAgucucaa.....................................................  1     1   T63
.................................aggccugUaucuuugucucaa.....................................................  2     1   T63
.................................aAgccugaaucuuugucucaa.....................................................  1     1   T63
.................................agAccugaaucuuugucucaa.....................................................  1     1   T63
.................................aggccugaaucuuugucuUaa.....................................................  1     1   T63
.................................aggccugaaucuuugucucaaA....................................................  32    1   T63
.................................aggccugaaCcuuugucucaac....................................................  2     1   T63
.................................aggccugaaucuuugucucaaG....................................................  3     1   T63
.................................aggUcugaaucuuugucucaac....................................................  2     1   T63
.................................aCgccugaaucuuugucucaac....................................................  1     1   T63
.................................aggccugaaucuuugucucaaU....................................................  98    1   T63
.................................aggccugUaucuuugucucaac....................................................  1     1   T63
.................................aggccugaaucuuAgucucaac....................................................  2     1   T63
.................................Gggccugaaucuuugucucaac....................................................  1     1   T63
.................................aggccugaauUuuugucucaac....................................................  1     1   T63
.................................agUccugaaucuuugucucaac....................................................  1     1   T63
.................................aggccugaaucuuugucucaac....................................................  1005  0   T63
.................................aggccuAaaucuuugucucaac....................................................  1     1   T63
.................................aggccugaaucuGugucucaac....................................................  1     1   T63
.................................aggccugaUucuuugucucaac....................................................  1     1   T63
.................................agAccugaaucuuugucucaac....................................................  2     1   T63
.................................aggAcugaaucuuugucucaac....................................................  1     1   T63
.................................aggccugaaucuuugucucaacA...................................................  29    1   T63
.................................aggccugaaucuuugucucaacc...................................................  719   0   T63
.................................aggccugaaucuuugucucaacG...................................................  5     1   T63
.................................agAccugaaucuuugucucaacc...................................................  1     1   T63
.................................aggUcugaaucuuugucucaacc...................................................  1     1   T63
.................................aggccugaaucuuugucucaacU...................................................  24    1   T63
.................................aUgccugaaucuuugucucaacc...................................................  1     1   T63
.................................aggccugaaucuuugucucaacAu..................................................  5     1   T63
.................................aUgccugaaucuuugucucaaccu..................................................  1     1   T63
.................................Gggccugaaucuuugucucaaccu..................................................  1     1   T63
.................................aggccugaaucuuugucucaaUcu..................................................  1     1   T63
.................................aggccugaaucuuugucucaaccC..................................................  1     1   T63
.................................aggccCgaaucuuugucucaaccu..................................................  1     1   T63
.................................aggccugaaucuuugucucaaccA..................................................  3     1   T63
.................................aggccugaaucuuugucucaaccu..................................................  281   0   T63
.................................aggccugaaucuuugAcucaaccu..................................................  1     1   T63
.................................aCgccugaaucuuugucucaaccu..................................................  1     1   T63
.................................aggccugGaucuuugucucaaccu..................................................  1     1   T63
.................................aggccugaaucuuugucucaaccuu.................................................  93    0   T63
```

Star Mature

```
uaucuccaucauuuucaugcgaaacuuucuggcaggccugaaucuuugucucaaccuucuguaaugaaaggugagcaaaguuucagguguguuugggggacuucuuuu
.................................aggccugaaucuuugucucaaccAu..................................................  1    1  T63
.................................aggccugaaucuuugucucaaccuA..................................................  2    1  T63
.................................aggccugaaucuuugucucaacAuu..................................................  1    1  T63
.................................aggccugaaucuuugucucaaccuuU.................................................  5    1  T63
..................................ggccugaaucuuugucuc........................................................  2    0  T63
..................................ggccugaaucuuugucuca.......................................................  2    0  T63
..................................ggccugaaucuuugucucaa......................................................  2    0  T63
..................................ggccugaaucuuugucucaacc....................................................  1    0  T63
..................................ggccugaaucuuugucucaacA....................................................  2    1  T63
..................................ggccugaaucuuugucucaaccu...................................................  2    0  T63
..................................ggccugaaucuuugucucaaccuu..................................................  4    0  T63
..................................ggccugaaucuuugucucaaccuuU.................................................  1    1  T63
...................................gccugaaucuuugucucaaccuu..................................................  1    0  T63
....................................ccugaaucuuugucucaac.....................................................  2    0  T63
....................................ccugaaucuuugucucaacc....................................................  2    0  T63
....................................ccugaaucuuugucucaaccu...................................................  9    0  T63
.....................................cugaaucuuugucucaac.....................................................  2    0  T63
.....................................cugaaucuuugucucaacc....................................................  1    0  T63
.....................................cugaaucuuugucucaaccu...................................................  3    0  T63
.....................................cugaaucuuugucucaaccuu..................................................  1    0  T63
......................................ugaaucuuugucucaaccu...................................................  2    0  T63
.................................................................gaaaAgugagcaaaguuucaggugugu................  2    1  T63
.................................................................gGaaggugagcaaaguuucaggugugu................  1    1  T63
.................................................................gaGaggugagcaaaguuucaggugugu................  1    1  T63
.................................................................gaaaggugagcaaaguuucaggugugu................  1    0  T63
..................................................................aaGggugagcaaaguuucaggugugu................  2    1  T63
..................................................................Gaaggugagcaaaguuucaggugugu................  1    1  T63
....................................................................Gggugagcaaaguuucaggugugu................  1    1  T63
.....................................................................Ugugagcaaaguuucaggu....................  1    1  T63
.....................................................................ggugagcaaaguuucaggugu..................  14   0  T63
.....................................................................ggugagcaaaguuucagguguU.................  10   1  T63
.....................................................................ggugagcaaaguuucaggugug.................  25   0  T63
.....................................................................ggCgagcaaaguuucaggugugu................  1    1  T63
.....................................................................ggugagcaaaguuucaggugugA................  8    1  T63
.....................................................................Agugagcaaaguuucaggugugu................  1    1  T63
.....................................................................ggugagcaaaguuucGggugugu................  1    1  T63
.....................................................................ggugagcaaaguuucagguguUu................  6    1  T63
.....................................................................Ugugagcaaaguuucaggugugu................  41   1  T63
.....................................................................Cgugagcaaaguuucaggugugu................  1    1  T63
.....................................................................ggugagcaaaguuAcaggugugu................  1    1  T63
.....................................................................ggugagcaaaguuucaggugugu................  186  0  T63
.....................................................................ggugagcaaaguuucagguguguu...............  5    0  T63
.....................................................................ggugagcaaaguuucaggugugu uu..............  1    0  T63
......................................................................gugagcaaaguuucaggA....................  1    1  T63
......................................................................gugagcaaaguuucaggu....................  57   0  T63
......................................................................gugagcaaaguuucagguA...................  1    1  T63
......................................................................gugagcaaaguuucaggug...................  66   0  T63
......................................................................gugagcaaaguuucagguU...................  1    1  T63
......................................................................gugagcaaaguuucaggGgu..................  1    1  T63
......................................................................gugagcaaaCuucaggugu..................  1    1  T63
......................................................................gugUgcaaaguuucaggugu..................  1    1  T63
......................................................................gugagcaaaguuucagUugu..................  1    1  T63
......................................................................gugaUcaaaguuucaggugu..................  2    1  T63
......................................................................gugagcaaaguuucaggugC..................  1    1  T63
......................................................................gugagcaaaguuucaggugA..................  3    1  T63
......................................................................gugagcaaaguuucaAgugu..................  1    1  T63
......................................................................gugagcaaaguuucaggugu..................  921  0  T63
......................................................................gugagcaaaguCucaggugu..................  1    1  T63
......................................................................gugaAcaaaguuucaggugu..................  1    1  T63
......................................................................gugagcaaagCuucaggugu..................  1    1  T63
......................................................................gugagcaaaguuucaggGgug.................  3    1  T63
......................................................................gugagcaaaguuucagguguU.................  286  1  T63
......................................................................gugagcaaaguuuUaggugug.................  2    1  T63
......................................................................gugaAcaaaguuucaggugug.................  2    1  T63
......................................................................gugagcaaaguuucaCgugug.................  1    1  T63
......................................................................guUagcaaaguuucaggugug.................  1    1  T63
......................................................................gugagcaaaguuucagAugug.................  1    1  T63
......................................................................gugagcaaaguuucaggAgug.................  2    1  T63
......................................................................gugagcaaaguuucaggugAg.................  1    1  T63
......................................................................gugUgcaaaguuucaggugug.................  1    1  T63
```

```
                                Star                                 Mature
uaucuccaucauuuucaugcgaaacuuucuggcaggccugaaucuuugucucaaccuucuguaaugaaaggugagcaaaguuucaggugugguuggggacuucuuuu
......................................................................gugagcaaaguuucaggugug................  2084    0  T63
......................................................................gugagcaaagAuucaggugug................  1       1  T63
......................................................................guCagcaaaguuucaggugug................  1       1  T63
......................................................................gugagcaaaguuucagCugug................  1       1  T63
......................................................................gugagcaaaguuucUggugug................  2       1  T63
......................................................................gugagGaaaguuucaggugug................  1       1  T63
......................................................................gugagcaaUguuucaggugug................  5       1  T63
......................................................................gugagcaaaguuucagUugug................  2       1  T63
......................................................................gugagcaaaguuucagguguA................  2       1  T63
......................................................................gugagcUaaguuucaggugug................  1       1  T63
......................................................................gugagcaaaguuucagguguC................  3       1  T63
......................................................................gugagcaaaguuucaggugGg................  1       1  T63
......................................................................gugagcaaaguuucaAgugugu...............  27      1  T63
......................................................................guUagcaaaguuucaggugugu...............  10      1  T63
......................................................................gugagcaaCguuucaggugugu...............  6       1  T63
......................................................................gugagUaaaguuucaggugugu...............  69      1  T63
......................................................................gGgagcaaaguuucaggugugu...............  16      1  T63
......................................................................gugagcaaaguuuAaggugugu...............  15      1  T63
......................................................................gugagcaaaguuCcaggugugu...............  9       1  T63
......................................................................gugagcaaaguuucaggugugC...............  77      1  T63
......................................................................gugUgcaaaguuucaggugugu...............  36      1  T63
......................................................................gugagcaaaguuuGaggugugu...............  34      1  T63
......................................................................gugagcaaGguuucaggugugu...............  13      1  T63
......................................................................gugagcaaaguuucaggugugA...............  1441    1  T63
......................................................................gugaCcaaaguuucaggugugu...............  11      1  T63
......................................................................gugagcGaaguuucaggugugu...............  5       1  T63
......................................................................gugagcUaaguuucaggugugu...............  5       1  T63
......................................................................gugagcaaaguuucagguCugu...............  15      1  T63
......................................................................gugagcaaaguuucaggugCgu...............  10      1  T63
......................................................................gugaUcaaaguuucaggugugu...............  18      1  T63
......................................................................gugagGaaaguuucaggugugu...............  26      1  T63
......................................................................gugagcaaaguGucaggugugu...............  2       1  T63
......................................................................gugagcaaaguuucagguguCu...............  8       1  T63
......................................................................gugagcaaaguuucagguguAu...............  12      1  T63
......................................................................gugagcaaaguuucaggugAgu...............  39      1  T63
......................................................................gugagcaaaguuuUaggugugu...............  40      1  T63
......................................................................gugagcaaaguuucaggugGgu...............  16      1  T63
......................................................................gugagcaaaguuucaggugugu...............  110426  0  T63
......................................................................gugagAaaaguuucaggugugu...............  12      1  T63
......................................................................gugagcaUaguuucaggugugu...............  8       1  T63
......................................................................gugagcaaaguAucaggugugu...............  14      1  T63
......................................................................gugCgcaaaguuucaggugugu...............  5       1  T63
......................................................................gugagcaaaCuuucaggugugu...............  41      1  T63
......................................................................gugagcaaaguuAcaggugugu...............  34      1  T63
......................................................................gugagcaaaguuucCggugugu...............  10      1  T63
......................................................................gugagcaaaAuuucaggugugu...............  10      1  T63
......................................................................gugagcaaaguCucaggugugu...............  7       1  T63
......................................................................gugagcaaaguuucGggugugu...............  68      1  T63
......................................................................Uugagcaaaguuucaggugugu...............  14      1  T63
......................................................................gugagcaaaguuucagUugugu...............  21      1  T63
......................................................................gugagcaaagAuucaggugugu...............  8       1  T63
......................................................................gugagcaaaguuucagCugugu...............  16      1  T63
......................................................................guAagcaaaguuucaggugugu...............  8       1  T63
......................................................................gugagcaaaguuucaggAgugu...............  34      1  T63
......................................................................Cugagcaaaguuucaggugugu...............  10      1  T63
......................................................................gugagcaaaguuucagguguUu...............  56      1  T63
......................................................................gugagcaaaguuucaUgugugu...............  33      1  T63
......................................................................gugaAcaaaguuucaggugugu...............  27      1  T63
......................................................................guCagcaaaguuucaggugugu...............  5       1  T63
......................................................................gugagcaaaguuucaggugugG...............  83      1  T63
......................................................................gugagcaaaguuucaggCgugu...............  6       1  T63
......................................................................gugagcaGaguuucaggugugu...............  4       1  T63
......................................................................gugagcaaaguuucagguAugu...............  22      1  T63
......................................................................gAgagcaaaguuucaggugugu...............  7       1  T63
......................................................................gugagcaaaguuGcaggugugu...............  2       1  T63
......................................................................gCgagcaaaguuucaggugugu...............  11      1  T63
......................................................................gugagcaaaguuucagAugugu...............  22      1  T63
......................................................................gugagcaaagGuucaggugugu...............  7       1  T63
......................................................................gugagcaaaguuucaggGgugu...............  19      1  T63
......................................................................gugagcaaagCuucaggugugu...............  6       1  T63
```

```
                                Star                              Mature
uaucuccaucauuuucaugcgaaacuuucuggcaggccugaaucuuugucucaaccuucuguaaugaaaggugagcaaaguuucaggugugguuugggggacuucuuuu
......................................................................gugGgcaaaguuucaggugugu................  9     1   T63
......................................................................gugagcaaaguuucUggugugu................  99    1   T63
......................................................................gugagcaaaguuucagguUugu................  22    1   T63
......................................................................gugagcaaaguuucaCgugugu................  18    1   T63
......................................................................gugagcaaaUuuucaggugugu................  3     1   T63
......................................................................gugagcaaUguuucaggugugu................  79    1   T63
......................................................................gugagcaaaguuuGagguguguu...............  3     1   T63
......................................................................gugagcaaaguuucagguguguu...............  4561  0   T63
......................................................................gugagcaaaguuucaggugugAu...............  14    1   T63
......................................................................gCgagcaaaguuucagguguguu...............  3     1   T63
......................................................................gugagcaaaguuAcagguguguu...............  2     1   T63
......................................................................gugagUaaaguuucagguguguu...............  5     1   T63
......................................................................gugagcaaaguuCcagguguguu...............  1     1   T63
......................................................................gugagcaaaguuucaggugugGu...............  2     1   T63
......................................................................gugagcaUaguuucagguguguu...............  1     1   T63
......................................................................gugagcaaaguuucagguguguG...............  25    1   T63
......................................................................gugagcaaUguuucagguguguu...............  1     1   T63
......................................................................gugagcaaaguuucagUuguguu...............  3     1   T63
......................................................................gugagcaaaguuucagCuguguu...............  1     1   T63
......................................................................Augagcaaaguuucagguguguu...............  3     1   T63
......................................................................gugagcaaaguuucaggugAguu...............  2     1   T63
......................................................................gugagcaaaguuucagguUuguu...............  1     1   T63
......................................................................gugagcaaaguuucagguguguA...............  335   1   T63
......................................................................gugaUcaaaguuucagguguguu...............  1     1   T63
......................................................................gugagcaaaguuucUgguguguu...............  4     1   T63
......................................................................gugagcaaaguuucaCguguguu...............  3     1   T63
......................................................................gugagcaaagCuucagguguguu...............  1     1   T63
......................................................................gugagcaaaguuucagAuguguu...............  2     1   T63
......................................................................gugagcaaaguuucagguguAuu...............  1     1   T63
......................................................................gugUgcaaaguuucagguguguu...............  4     1   T63
......................................................................gugagcaaaguuucagguCuguu...............  1     1   T63
......................................................................gugagcaaaguuucaggugugCu...............  1     1   T63
......................................................................gugagcaaaCuuucagguguguu...............  3     1   T63
......................................................................gugagGaaaguuucagguguguu...............  1     1   T63
......................................................................gugGgcaaaguuucagguguguu...............  1     1   T63
......................................................................gugaAcaaaguuucagguguguu...............  1     1   T63
......................................................................gugaCcaaaguuucagguguguu...............  2     1   T63
......................................................................gugagAaaaguuucagguguguu...............  2     1   T63
......................................................................gugagcaaaguuucagguguguuG..............  5     1   T63
......................................................................gugagcaaaguuucagguguguuu..............  109   0   T63
......................................................................gugagcaaaguuuAagguguguuu..............  1     1   T63
......................................................................gugagcaaaguuucagguguguGu..............  3     1   T63
......................................................................gugagcaaaguuucaggugugAuu..............  1     1   T63
......................................................................gugagcaaaguuucagguguguuC..............  4     1   T63
......................................................................Augagcaaaguuucagguguguuu..............  1     1   T63
......................................................................gugagcaaaguuucagguguguuA..............  37    1   T63
......................................................................gugagcaaaguuucagguguguAu..............  16    1   T63
......................................................................gugagcaaaguuucagguguguuuU.............  12    1   T63
......................................................................gugagcaaaguuucagguguguAug.............  1     1   T63
......................................................................gugagcaaaguuucagguguguuuA.............  2     1   T63
.......................................................................ugagcaaaguuucaggugugA................  1     1   T63
.......................................................................ugagcaaaguuucaggugugu................  9     0   T63
.......................................................................ugagcaaaguuucagguguguu...............  1     0   T63
........................................................................gagcaaaguuucaggugu..................  2     0   T63
........................................................................gagcaaaguuucaggugug.................  1     0   T63
........................................................................gagcaaaguuucaggugugu................  22    0   T63
........................................................................gagcaaaguuucagguAugu................  1     1   T63
........................................................................gagcaaaguuucaggugugA................  2     1   T63
.........................................................................agcaaaguuucaggugugu................  9     0   T63
.........................................................................agcaaaguuucagguguguu...............  2     0   T63
..........................................................................gcaaaguuucaggugugu................  12    0   T63

................................Uaggccugaaucuuugucuca.......................................................  1     1   MOL
................................Aaggccugaaucuuugucucaa......................................................  1     1   MOL
................................Uaggccugaaucuuugucucaa......................................................  1     1   MOL
.................................aggccugaaucuuugucA.........................................................  1     1   MOL
.................................aggccugaaucuuugucu.........................................................  18    0   MOL
.................................Cggccugaaucuuugucuc........................................................  1     1   MOL
.................................aggccugaaCcuuugucuc........................................................  1     1   MOL
.................................aggccugaaucuuugucuc........................................................  245   0   MOL
```

Star Mature

```
uaucuccaucauuuucaugcgaaacuuucuggcaggccugaaucuuugucucaaccuucuguaaugaaaggugagcaaaguuucagguguguuugggggacuucuuuu
.................................aggccugaaucuuugucAca.......................................................  1     1  MOL
.................................aggccugaaucuuugucucG.......................................................  1     1  MOL
.................................aggccugaaCcuuugucuca.......................................................  1     1  MOL
.................................aggccugaaucuuAgucuca.......................................................  5     1  MOL
.................................agUccugaaucuuugucuca.......................................................  2     1  MOL
.................................aggccuCaaucuuugucuca.......................................................  1     1  MOL
.................................aggccugUaucuuugucuca.......................................................  1     1  MOL
.................................aggcUugaaucuuugucuca.......................................................  2     1  MOL
.................................aggccugaaucAuugucuca.......................................................  2     1  MOL
.................................aggUcugaaucuuugucuca.......................................................  1     1  MOL
.................................aggccugaaucCuugucuca.......................................................  3     1  MOL
.................................aggccugaaucuuugucuca.......................................................  3065  0  MOL
.................................aggccugaauUuuugucuca.......................................................  3     1  MOL
.................................aggccugaUucuuugucuca.......................................................  4     1  MOL
.................................aggccugaaucuuugAcuca.......................................................  2     1  MOL
.................................aggccugaaucuuugucuUa.......................................................  3     1  MOL
.................................aggccugaaucuuugucucU.......................................................  9     1  MOL
.................................aggccugaaucuuugucuAa.......................................................  3     1  MOL
.................................aggccGgaaucuuugucuca.......................................................  1     1  MOL
.................................aggccugaGucuuugucuca.......................................................  2     1  MOL
.................................aggccCgaaucuuugucuca.......................................................  2     1  MOL
.................................aggccugaaucuuAgucucaa......................................................  5     1  MOL
.................................aggAcugaaucuuugucucaa......................................................  2     1  MOL
.................................aggccugaaucuAugucucaa......................................................  1     1  MOL
.................................aggccugaaucCuugucucaa......................................................  1     1  MOL
.................................aCgccugaaucuuugucucaa......................................................  2     1  MOL
.................................aggUcugaaucuuugucucaa......................................................  1     1  MOL
.................................aggccugaaucuuCgucucaa......................................................  1     1  MOL
.................................aggccugaaucuuugAcucaa......................................................  2     1  MOL
.................................aggccugaaucuuugucucaC......................................................  1     1  MOL
.................................aggccugaauUuuugucucaa......................................................  1     1  MOL
.................................aggccugaaucuuugucuUaa......................................................  2     1  MOL
.................................aggccugaaucuuugucucaU......................................................  14    1  MOL
.................................aggccugUaucuuugucucaa......................................................  1     1  MOL
.................................aggccugaaucuuugucucaa......................................................  1006  0  MOL
.................................aggccugaaucuCugucucaa......................................................  1     1  MOL
.................................aggccugaaucuuugucucaUc.....................................................  1     1  MOL
.................................aggccugaUucuuugucucaac.....................................................  2     1  MOL
.................................aggccugaaucuCugucucaac.....................................................  1     1  MOL
.................................aggccugUaucuuugucucaac.....................................................  1     1  MOL
.................................aggccugaaucuuugucGcaac.....................................................  1     1  MOL
.................................aggccugaaucuuAgucucaac.....................................................  1     1  MOL
.................................aggUcugaaucuuugucucaac.....................................................  2     1  MOL
.................................aCgccugaaucuuugucucaac.....................................................  2     1  MOL
.................................aggccugaaucuuugucuUaac.....................................................  1     1  MOL
.................................aggccugaaucuuugucucaac.....................................................  1479  0  MOL
.................................aggccugaaucuAugucucaac.....................................................  1     1  MOL
.................................aggcAugaaucuuugucucaac.....................................................  1     1  MOL
.................................aggccugaaucuuuguAucaac.....................................................  1     1  MOL
.................................aggccugCaucuuugucucaac.....................................................  1     1  MOL
.................................aggccugaaucuuugucuAaac.....................................................  1     1  MOL
.................................aggccugaaucuuugucucaaA.....................................................  17    1  MOL
.................................aggccugaaucuuugucucaaU.....................................................  27    1  MOL
.................................aggccugaUucuuugucucaacc....................................................  1     1  MOL
.................................aggccugaaCcuuugucucaacc....................................................  1     1  MOL
.................................aggccugaaucuuugucucaacA....................................................  31    1  MOL
.................................aggccugGaucuuugucucaacc....................................................  1     1  MOL
.................................aggccugaaucuuugucucaaUc....................................................  1     1  MOL
.................................aggccugaaucAuugucucaacc....................................................  1     1  MOL
.................................aggccugaaucCuugucucaacc....................................................  1     1  MOL
.................................aggccugaaucuuugucucaaAc....................................................  1     1  MOL
.................................aggccugaaucuuugucucaacG....................................................  6     1  MOL
.................................aCgccugaaucuuugucucaacc....................................................  1     1  MOL
.................................aggccugaaucuuugucucaacU....................................................  36    1  MOL
.................................aggccAgaaucuuugucucaacc....................................................  1     1  MOL
.................................aggccugaaucuuugucucaacc....................................................  1140  0  MOL
.................................aggccugaaucuuugAcucaacc....................................................  1     1  MOL
.................................aggccugaaucuuugucAcaaccu...................................................  1     1  MOL
.................................aggccugaaucuuugucucaacUu...................................................  1     1  MOL
.................................aggccugaaucuuugucucaaccu...................................................  296   0  MOL
```

Star Mature

```
uaucuccaucauuuucaugcgaaacuuucuggcaggccugaaucuuugucucaaccuucuguaaugaaaggugagcaaaguuucagguguguuugggggacuucuuuu
.................................aggccugaaucuuugucucaacAu...................................................  2    1  MOL
.................................agUccugaaucuuugucucaaccu...................................................  1    1  MOL
.................................aggccugUaucuuugucucaaccu...................................................  1    1  MOL
.................................aggccugaaCcuuugucucaaccu...................................................  1    1  MOL
.................................aggccugaaucuuugucuUaaccu...................................................  1    1  MOL
.................................agAccugaaucuuugucucaaccu...................................................  2    1  MOL
.................................aggccugaaucuuugucucaaccC...................................................  1    1  MOL
.................................aggccugaaucuuugucucaaccA...................................................  13   1  MOL
.................................aggccugaaucuuugucucaaccG...................................................  2    1  MOL
.................................aggccugaaucuuugucucaaccuA..................................................  2    1  MOL
.................................aggccugaaucuuugucucaaccCu..................................................  2    1  MOL
.................................aggUcugaaucuuugucucaaccuu..................................................  2    1  MOL
.................................aggccugaaucuuugucucaacUuu..................................................  1    1  MOL
.................................Uggccugaaucuuugucucaaccuu..................................................  1    1  MOL
.................................aggccugaaucuuugucucaaccuu..................................................  217  0  MOL
.................................agUccugaaucuuugucucaaccuu..................................................  1    1  MOL
.................................aggccugaaucuuugucucaaccuuG.................................................  1    1  MOL
.................................aggccugaaucuuugucucaaccuuU.................................................  29   1  MOL
..................................ggccugaaucuuugucuca.......................................................  2    0  MOL
..................................ggccugaaucuuugucucaa......................................................  2    0  MOL
..................................ggccugaaucuuugucucaac.....................................................  3    0  MOL
..................................ggccugaaucuuugucucaacc....................................................  1    0  MOL
..................................ggccugaaucuuugucucaaccu...................................................  3    0  MOL
..................................ggccugaaucuuugucucaaccuu..................................................  3    0  MOL
....................................ccugaaucuuugucucaaccu...................................................  5    0  MOL
....................................ccugaaucuuugucucaaccuu..................................................  1    0  MOL
.....................................cugaaucuuugucucaaccu...................................................  1    0  MOL
......................................ugaaucuuugucucaacc....................................................  1    0  MOL
......................................ugaaucuuugucucaaccu...................................................  1    0  MOL
.................................................................gaaaAgugagcaaaguuucaggugugu................  1    1  MOL
.................................................................gGaaggugagcaaaguuucaggugugu................  8    1  MOL
.................................................................gaaaCgugagcaaaguuucaggugugu................  1    1  MOL
.................................................................gaaGggugagcaaaguuucaggugugu................  5    1  MOL
.................................................................gaGaggugagcaaaguuucaggugugu................  9    1  MOL
.................................................................gaaaggugagcaaaguuucaggugugu................  3    0  MOL
..................................................................aaGggugagcaaaguuucaggugug.................  1    1  MOL
..................................................................aaGggugagcaaaguuucaggugugu................  8    1  MOL
..................................................................Gaaggugagcaaaguuucaggugugu................  3    1  MOL
..................................................................aaaCgugagcaaaguuucaggugugu................  3    1  MOL
..................................................................aGaggugagcaaaguuucaggugugu................  2    1  MOL
..................................................................aaaggugagcaaaguuucaggugugu................  1    0  MOL
..................................................................aaaCgugagcaaaguuucagguguguu...............  1    1  MOL
...................................................................Gaggugagcaaaguuucaggugugu................  7    1  MOL
...................................................................aaCgugagcaaaguuucaggugugu................  1    1  MOL
...................................................................aGggugagcaaaguuucaggugugu................  9    1  MOL
....................................................................Uggugagcaaaguuucaggugugu................  1    1  MOL
....................................................................Gggugagcaaaguuucaggugugu................  3    1  MOL
.....................................................................ggugagcaaaguuucaggu....................  2    0  MOL
.....................................................................ggugagcaaaguuucaggug...................  1    0  MOL
.....................................................................ggugagcaaaguuucagguA...................  1    1  MOL
.....................................................................ggugagcaaaguuucaggugu..................  62   0  MOL
.....................................................................ggugagcaaaguuucaggugC..................  1    1  MOL
.....................................................................Ugugagcaaaguuucaggugu..................  1    1  MOL
.....................................................................ggugaAcaaaguuucaggugu..................  1    1  MOL
.....................................................................ggugagUaaaguuucaggugu..................  1    1  MOL
.....................................................................ggugagcaaaguuAcaggugu..................  1    1  MOL
.....................................................................ggugagcaaaguuucagguguA.................  1    1  MOL
.....................................................................ggugagcaaaguuucaggAgug.................  1    1  MOL
.....................................................................ggugagcaaaguuucaggugug.................  74   0  MOL
.....................................................................Ugugagcaaaguuucaggugug.................  6    1  MOL
.....................................................................ggugagcaaaguuucagguguU.................  14   1  MOL
.....................................................................ggugagcaaaguuucaggugugA................  54   1  MOL
.....................................................................ggugUgcaaaguuucaggugugu................  2    1  MOL
.....................................................................ggugCgcaaaguuucaggugugu................  2    1  MOL
.....................................................................ggugagcaaaguAucaggugugu................  1    1  MOL
.....................................................................ggugagcaaaguuucagguguUu................  4    1  MOL
.....................................................................Ugugagcaaaguuucaggugugu................  111  1  MOL
.....................................................................Agugagcaaaguuucaggugugu................  4    1  MOL
.....................................................................ggugagcaaaguuucagUugugu................  1    1  MOL
.....................................................................ggugagcaaaguuucaggugugG................  1    1  MOL
```

Star **Mature**

```
uaucuccaucauuuucaugcgaaacuuucuggcaggccugaaucuuugucucaaccuucuguaaugaaaggugagcaaaguuucagguguguuugggggacuucuuuu
.....................................................................ggugagcaaaguuucUggugugu................ 1      1    MOL
.....................................................................ggugagUaaaguuucaggugugu................ 1      1    MOL
.....................................................................ggugagcaUaguuucaggugugu................ 1      1    MOL
.....................................................................ggugagcaaaguuucagguUugu................ 1      1    MOL
.....................................................................ggugagcaaaguuucaggugugu................ 1199   0    MOL
.....................................................................ggugagcaaaguuucagAugugu................ 1      1    MOL
.....................................................................ggugagcaaaguuucaUgugugu................ 1      1    MOL
.....................................................................ggugagcaaaguuuGaggugugu................ 1      1    MOL
.....................................................................ggugagcaaaguuucaggugugC................ 4      1    MOL
.....................................................................Ugugagcaaaguuucagguguguu............... 1      1    MOL
.....................................................................ggugagcaaaguuucaggugugAu............... 1      1    MOL
.....................................................................ggugagcaaaguuucagguguguG............... 1      1    MOL
.....................................................................ggugagcaaaguuucagguguguu............... 12     0    MOL
.....................................................................ggugagcaaaguuucagguguguA............... 4      1    MOL
.....................................................................ggugagcaaaguuucagguguguuu.............. 5      0    MOL
......................................................................gugagcaaaguuucaggG.................... 1      1    MOL
......................................................................gugagcaaaguuucaggu.................... 87     0    MOL
......................................................................gugagcaaaguuucaggC.................... 2      1    MOL
......................................................................gugagcaaaguuucagguU................... 1      1    MOL
......................................................................gugagcaaaguuucaggug................... 215    0    MOL
......................................................................gugagcaaaguuucUggug................... 1      1    MOL
......................................................................gugagcaaaguAucaggug................... 1      1    MOL
......................................................................gugagcaaaguuucGggug................... 1      1    MOL
......................................................................gugagcaaaguuucagguC................... 3      1    MOL
......................................................................gugagcaaaguuucaggugu.................. 3431   0    MOL
......................................................................gugagcaaaguuucUggugu.................. 4      1    MOL
......................................................................gugagcaaaguuucaggGgu.................. 3      1    MOL
......................................................................gGgagcaaaguuucaggugu.................. 1      1    MOL
......................................................................gugagUaaaguuucaggugu.................. 1      1    MOL
......................................................................gugUgcaaaguuucaggugu.................. 1      1    MOL
......................................................................gugagcaaaguuucaAgugu.................. 1      1    MOL
......................................................................gugagcaaaCuuucaggugu.................. 1      1    MOL
......................................................................Cugagcaaaguuucaggugu.................. 1      1    MOL
......................................................................gugagcaaaguuucagAugu.................. 1      1    MOL
......................................................................gugGgcaaaguuucaggugu.................. 1      1    MOL
......................................................................guUagcaaaguuucaggugu.................. 1      1    MOL
......................................................................gAgagcaaaguuucaggugu.................. 1      1    MOL
......................................................................gugagcaaaguuAcaggugu.................. 5      1    MOL
......................................................................gugagcaaaguuucGggugu.................. 1      1    MOL
......................................................................gugagcaaaguuucaUgugu.................. 3      1    MOL
......................................................................gugaCcaaaguuucaggugu.................. 1      1    MOL
......................................................................gugagcaaaguuucaggugA.................. 6      1    MOL
......................................................................gugagcaaaguuucagguUu.................. 3      1    MOL
......................................................................gugagcaaaguAucaggugu.................. 1      1    MOL
......................................................................gugagGaaaguuucaggugu.................. 3      1    MOL
......................................................................gugaAcaaaguuucaggugu.................. 1      1    MOL
......................................................................gugagcaaagGuucaggugu.................. 2      1    MOL
......................................................................gugagcaaaguuucaggugC.................. 4      1    MOL
......................................................................gugagcaaaguuucagUugu.................. 1      1    MOL
......................................................................gCgagcaaaguuucaggugu.................. 2      1    MOL
......................................................................gugagcaaaUuuucaggugu.................. 1      1    MOL
......................................................................gugagcaaUguuucaggugu.................. 3      1    MOL
......................................................................gugagcaaaguuucaggAgu.................. 1      1    MOL
......................................................................gugagcaaaguuuUaggugu.................. 3      1    MOL
......................................................................gugagcaaaguuucUggugug................. 9      1    MOL
......................................................................gugagcGaaguuucaggugug................. 1      1    MOL
......................................................................gugagcaaaguCucaggugug................. 2      1    MOL
......................................................................gugagcaaaguuucaggugug................. 11001  0    MOL
......................................................................gugaUcaaaguuucaggugug................. 2      1    MOL
......................................................................gugagcaaaguuucaCgugug................. 1      1    MOL
......................................................................gugagcaaaguuuUaggugug................. 2      1    MOL
......................................................................gugagcaaaguAucaggugug................. 1      1    MOL
......................................................................gugagcaaaguuucaAgugug................. 3      1    MOL
......................................................................gugagcaaagGuucaggugug................. 3      1    MOL
......................................................................gugagcaaaAuuucaggugug................. 2      1    MOL
......................................................................gugCgcaaaguuucaggugug................. 1      1    MOL
......................................................................gugagcaaaguuucagguguA................. 19     1    MOL
......................................................................gugagcaaaguuucagUugug................. 4      1    MOL
......................................................................gugagcaaaguuucaUgugug................. 7      1    MOL
......................................................................gugagcaaaUuuucaggugug................. 2      1    MOL
```

```
                                 Star                                    Mature
uaucuccaucauuuucaugcgaaacuuucuggcaggccugaaucuuugucucaaccuucuguaaugaaaggugagcaaaguuucagguguguuugggggacuucuuuu
......................................................................gugagcaaaguuucagCugug.................   1       1       MOL
......................................................................gugagcaaaguuucagguCug.................   3       1       MOL
......................................................................gugagcaaaguuucaggugAg.................   4       1       MOL
......................................................................gugagcaaagCuucaggugug.................   3       1       MOL
......................................................................gugagcaaCguuucaggugug.................   4       1       MOL
......................................................................guUagcaaaguuucaggugug.................   2       1       MOL
......................................................................gCgagcaaaguuucaggugug.................   2       1       MOL
......................................................................gugagcaaaguuucaggGgug.................   6       1       MOL
......................................................................gugaCcaaaguuucaggugug.................   1       1       MOL
......................................................................gugUgcaaaguuucaggugug.................   6       1       MOL
......................................................................gugagcaaGguuucaggugug.................   3       1       MOL
......................................................................gugagcaaaCuucaggugug.................   7       1       MOL
......................................................................gugagcaaaguuucagguguU.................   299     1       MOL
......................................................................gugagcaUaguuucaggugug.................   3       1       MOL
......................................................................Cugagcaaaguuucaggugug.................   1       1       MOL
......................................................................guAagcaaaguuucaggugug.................   2       1       MOL
......................................................................gugagcaaaguuucaggAgug.................   5       1       MOL
......................................................................gugagGaaaguuucaggugug.................   1       1       MOL
......................................................................gugagcaaaguuucGggugug.................   4       1       MOL
......................................................................gugagcaaagAuucaggugug.................   1       1       MOL
......................................................................gugagcaaaguuucagguAug.................   7       1       MOL
......................................................................gugagcaaaguuucagguUug.................   5       1       MOL
......................................................................gugagcaaaguuuAaggugug.................   1       1       MOL
......................................................................gugagcaaaguuucaggugCg.................   4       1       MOL
......................................................................gugaAcaaaguuucaggugug.................   2       1       MOL
......................................................................gugagUaaaguuucaggugug.................   9       1       MOL
......................................................................gugagcaaUguuucaggugug.................   11      1       MOL
......................................................................gugagcaaaguuucaggugC.................   10      1       MOL
......................................................................gugagcaaaguuAcaggugug.................   6       1       MOL
......................................................................gugagcaaaguuGaggugug.................   3       1       MOL
......................................................................gugagAaaaguuucaggugug.................   1       1       MOL
......................................................................gugagcaaaguuucagAugug.................   2       1       MOL
......................................................................gugagcaaaguuucaggCgug.................   3       1       MOL
......................................................................gugagcaaaguuAcaggugugu................   111     1       MOL
......................................................................gugagcaaaguuucagguAugu................   66      1       MOL
......................................................................gugagUaaaguuucaggugugu................   153     1       MOL
......................................................................gugagcaaaguuucUggugugu................   279     1       MOL
......................................................................gugagcaaaguuucagAugugu................   94      1       MOL
......................................................................Cugagcaaaguuucaggugugu................   18      1       MOL
......................................................................gugagcaaaguuucGggugugu................   123     1       MOL
......................................................................gugagcaaaCuucaggugugu................   118     1       MOL
......................................................................gGgagcaaaguuucaggugugu................   47      1       MOL
......................................................................gugagcaaaUuucaggugugu................   8       1       MOL
......................................................................gugGgcaaaguuucaggugugu................   20      1       MOL
......................................................................gugagcaaaguuucaggugugu................   291869  0       MOL
......................................................................gugagAaaaguuucaggugugu................   57      1       MOL
......................................................................gugagcaaaguuucagguUugu................   49      1       MOL
......................................................................Uugagcaaaguuucaggugugu................   14      1       MOL
......................................................................gugagcaGaguuucaggugugu................   17      1       MOL
......................................................................gugagcaaaguuucagguguCu................   16      1       MOL
......................................................................gugagcaaaguuGcaggugugu................   17      1       MOL
......................................................................gugUgcaaaguuucaggugugu................   97      1       MOL
......................................................................gugagcaaaguuucagguguAu................   60      1       MOL
......................................................................gugagcaaaguCucaggugugu................   15      1       MOL
......................................................................gugagcaaaguuucagguCugu................   16      1       MOL
......................................................................gCgagcaaaguuucaggugugu................   47      1       MOL
......................................................................gugagcaUaguuucaggugugu................   17      1       MOL
......................................................................gugagcaaaguuuAaggugugu................   23      1       MOL
......................................................................gugagcaaagGuucaggugugu................   31      1       MOL
......................................................................gugagcaaaAuucaggugugu................   19      1       MOL
......................................................................gugagcaaaguuCcaggugugu................   30      1       MOL
......................................................................gugagcaaaguuucaggugugG................   133     1       MOL
......................................................................gugagcaaaguuuGaggugugu................   97      1       MOL
......................................................................gugCgcaaaguuucaggugugu................   14      1       MOL
......................................................................gugagcUaaguuucaggugugu................   19      1       MOL
......................................................................gugagcaaaguuucaggugAgu................   67      1       MOL
......................................................................gugagcaaaguGucaggugugu................   7       1       MOL
......................................................................gugagcaaaguuucaggGugu................   72      1       MOL
......................................................................gugagcaaUguuucaggugugu................   241     1       MOL
......................................................................gAgagcaaaguuucaggugugu................   13      1       MOL
```

```
                                        Star                                    Mature
uaucuccaucauuuucaugcgaaacuuucuggcaggccugaaucuuugucucaaccuucuguaaugaaaggugagcaaaguuucagguguguuugggggacuucuuuu
......................................................................gugagcaaaguuucaAgugugu................    59     1     MOL
......................................................................gugagcaaaguuucagguguUu................    100    1     MOL
......................................................................gugagcaaaguuucaCgugugu................    63     1     MOL
......................................................................gugagGaaaguuucaggugugu................    77     1     MOL
......................................................................gugagcCaaguuucaggugugu................    1      1     MOL
......................................................................gugagcaaaguuucaUgugugu................    72     1     MOL
......................................................................gugagcaaGguuucaggugugu................    42     1     MOL
......................................................................gugagcaaaguuucCggugugu................    7      1     MOL
......................................................................gugagcaaaguuucaggAgugu................    73     1     MOL
......................................................................gugaUcaaaguuucaggugugu................    23     1     MOL
......................................................................gugaAcaaaguuucaggugugu................    65     1     MOL
......................................................................gugagcaaaguuucaggugugC................    267    1     MOL
......................................................................guCagcaaaguuucaggugugu................    8      1     MOL
......................................................................gugagcaaaguuucagCugugu................    50     1     MOL
......................................................................gugagcaaagCuucaggugugu................    24     1     MOL
......................................................................gugagcaaaguuuUaggugugu................    180    1     MOL
......................................................................gugagcaaaguuucagUugugu................    123    1     MOL
......................................................................gugagcaaaguuucaggugGgu................    44     1     MOL
......................................................................guAagcaaaguuucaggugugu................    25     1     MOL
......................................................................gugagcGaaguuucaggugugu................    16     1     MOL
......................................................................gugagcaaaguuucaggugCgu................    15     1     MOL
......................................................................gugagcaaCguuucaggugugu................    18     1     MOL
......................................................................gugagcaaaguuucaggugugA................    6745   1     MOL
......................................................................gugagcaaaguAucaggugugu................    49     1     MOL
......................................................................gugagcaaagAuucaggugugu................    19     1     MOL
......................................................................gugaCcaaaguuucaggugugu................    30     1     MOL
......................................................................gugagcaaaguuucaggCgugu................    22     1     MOL
......................................................................guUagcaaaguuucaggugugu................    35     1     MOL
......................................................................gugagcaaaguuuGagguguguu...............    2      1     MOL
......................................................................gugagcaaaguuucagguguguA...............    1520   1     MOL
......................................................................gugagcaGaguuucagguguguu...............    2      1     MOL
......................................................................gugagcaaaguuucagguguUuu...............    2      1     MOL
......................................................................guAagcaaaguuucagguguguu...............    1      1     MOL
......................................................................gugagcaaaguuucUgguguguu...............    5      1     MOL
......................................................................gCgagcaaaguuucagguguguu...............    2      1     MOL
......................................................................gugagcaaaguuucaggGguguu...............    3      1     MOL
......................................................................gugaCcaaaguuucagguguguu...............    1      1     MOL
......................................................................gugagcaaaguuucagguguAuu...............    3      1     MOL
......................................................................gugagcaaagAuucagguguguu...............    1      1     MOL
......................................................................gugagcaaaguuucaAguguguu...............    2      1     MOL
......................................................................gugagcaaaguuAcagguguguu...............    6      1     MOL
......................................................................gugagcaaaguuucCgguguguu...............    1      1     MOL
......................................................................gugagcaaagGuucagguguguu...............    1      1     MOL
......................................................................gugaAcaaaguuucagguguguu...............    2      1     MOL
......................................................................gugagcaaaguuucagAuguguu...............    2      1     MOL
......................................................................gugagcaaaguuucagguAuguu...............    2      1     MOL
......................................................................gugagcaaaguAucagguguguu...............    1      1     MOL
......................................................................gugagcaaaguuucagguguguu...............    5487   0     MOL
......................................................................gugagcaaUguuucagguguguu...............    2      1     MOL
......................................................................gugagcaaaguuucagUuguguu...............    1      1     MOL
......................................................................gugagcaaaguuucaCguguguu...............    1      1     MOL
......................................................................gugagcaaaguuucaggugugAu...............    44     1     MOL
......................................................................gugagcaaaguuucaggugugCu...............    3      1     MOL
......................................................................gugagGaaaguuucagguguguu...............    2      1     MOL
......................................................................gugagcaaaguuCcagguguguu...............    2      1     MOL
......................................................................gugagcaaaguuucaggugAguu...............    1      1     MOL
......................................................................gugagcaaaguuucagguguguG...............    47     1     MOL
......................................................................guUagcaaaguuucagguguguu...............    1      1     MOL
......................................................................gugUgcaaaguuucagguguguu...............    5      1     MOL
......................................................................gugagAaaaguuucagguguguu...............    2      1     MOL
......................................................................gGgagcaaaguuucagguguguu...............    2      1     MOL
......................................................................guCagcaaaguuucagguguguu...............    1      1     MOL
......................................................................gugagcaaaguuucaggAguguu...............    2      1     MOL
......................................................................gugagUaaaguuucagguguguu...............    4      1     MOL
......................................................................gugagcaaaCuuucagguguguu...............    2      1     MOL
......................................................................gugGgcaaaguuucagguguguu...............    2      1     MOL
......................................................................gugagcaaaguuuUagguguguu...............    7      1     MOL
......................................................................gAgagcaaaguuucagguguguu...............    1      1     MOL
......................................................................gugagcaaaguuucaUguguguu...............    4      1     MOL
......................................................................gugagcaaaguuucagguguguAu..............    80     1     MOL
```

```
                                  Star                                Mature
uaucuccaucauuuucaugcgaaacuuucuggcaggccugaaucuuugucucaaccuucuguaaugaaaggugagcaaaguuucaggugugu uugggggacuucuuuu
......................................................................gugagcaaaguuucagguguguCu..............  2     1    MOL
......................................................................gugagcaaaguuucagguguguGu..............  2     1    MOL
......................................................................gugagcaaaguuucagguguguuu..............  231   0    MOL
......................................................................gugagcaaaguuucagguguguuC..............  15    1    MOL
......................................................................gugagcaaaCuuucagguguguuu..............  1     1    MOL
......................................................................gugagcaaaguuucagguguguuA..............  182   1    MOL
......................................................................gugagcaaaguuucagUuguguuu..............  1     1    MOL
......................................................................gugagcaaaguuucagguguguuG..............  6     1    MOL
......................................................................gugagcaaaguuucaggugugAuu..............  3     1    MOL
......................................................................gugagcaaaguuucagguguguuuU.............  18    1    MOL
......................................................................gugagcaaaguuucagguguguuug.............  1     0    MOL
......................................................................gugagcaaaguuucagguguguGuggg...........  1     1    MOL
.......................................................................ugagcaaaguuucaggugug.................  1     0    MOL
.......................................................................ugagcaaaguuucaggugugA................  2     1    MOL
.......................................................................Ggagcaaaguuucaggugugu................  2     1    MOL
.......................................................................ugagcaaaguuucaggugugu................  17    0    MOL
.......................................................................ugagcaaaUuuucaggugugu................  1     1    MOL
.......................................................................ugagcaaaguuucagguguguu...............  2     0    MOL
........................................................................gagcaaaguuucaggugug.................  3     0    MOL
........................................................................gagcaaaguuucaggugugu................  35    0    MOL
........................................................................gUgcaaaguuucaggugugu................  1     1    MOL
........................................................................gagcUaaguuucaggugugu................  1     1    MOL
........................................................................gagcaaaguuucagguguguu...............  4     0    MOL
.........................................................................agcaaaguuucaggugugu................  9     0    MOL
..........................................................................gcaaaguuucaggugugu................  8     0    MOL

................................Uaggccugaaucuuugucuc........................................................  2     1    T53
................................Uaggccugaaucuuugucuca.......................................................  1     1    T53
.................................aggccugaaucuuugucu.........................................................  74    0    T53
.................................aggccugaaucuuugucA.........................................................  19    1    T53
.................................aggccugaaucuuugucG.........................................................  1     1    T53
.................................aggcUugaaucuuugucuc........................................................  1     1    T53
.................................aggccugaaucCuugucuc........................................................  1     1    T53
.................................aggAcugaaucuuugucuc........................................................  1     1    T53
.................................aggccugaUucuuugucuc........................................................  1     1    T53
.................................aggccugaaucuuugucuc........................................................  1173  0    T53
.................................aggccugaaucuuugucuA........................................................  19    1    T53
.................................agUccugaaucuuugucuc........................................................  1     1    T53
.................................aggccugaaucuuugucCc........................................................  1     1    T53
.................................aggccugaaucuuugucuU........................................................  4     1    T53
.................................aggccugaaucuuAgucuca.......................................................  10    1    T53
.................................aggccugaaucuuugucAca.......................................................  1     1    T53
.................................aggccugaaucuuugucGca.......................................................  1     1    T53
.................................agAccugaaucuuugucuca.......................................................  1     1    T53
.................................aggccugaaucuuugucucU.......................................................  13    1    T53
.................................aCgccugaaucuuugucuca.......................................................  1     1    T53
.................................aggccuUaaucuuugucuca.......................................................  1     1    T53
.................................aggccugaaucuuugucCca.......................................................  4     1    T53
.................................aggccugaaucuuuUucuca.......................................................  1     1    T53
.................................aggcGugaaucuuugucuca.......................................................  1     1    T53
.................................aggccugaaucuuCgucuca.......................................................  2     1    T53
.................................aggccugaaucCuugucuca.......................................................  3     1    T53
.................................aggccuAaaucuuugucuca.......................................................  3     1    T53
.................................aggccugaaAcuuugucuca.......................................................  1     1    T53
.................................aggccugaaucuuugucuUa.......................................................  3     1    T53
.................................aggccCgaaucuuugucuca.......................................................  2     1    T53
.................................aggAcugaaucuuugucuca.......................................................  3     1    T53
.................................aggccugaaucuuuguUuca.......................................................  1     1    T53
.................................aggccugaauAuuugucuca.......................................................  1     1    T53
.................................aggccugaaucAuugucuca.......................................................  1     1    T53
.................................aggUcugaaucuuugucuca.......................................................  4     1    T53
.................................aggccugaaucuuugucuAa.......................................................  7     1    T53
.................................aggccugaUucuuugucuca.......................................................  4     1    T53
.................................Gggccugaaucuuugucuca.......................................................  1     1    T53
.................................aggccugaaCcuuugucuca.......................................................  2     1    T53
.................................aggccugaaucuuugucuca.......................................................  6941  0    T53
.................................aggccugaaucuuugCcuca.......................................................  1     1    T53
.................................aggccugaGucuuugucuca.......................................................  4     1    T53
.................................aggcUugaaucuuugucuca.......................................................  1     1    T53
.................................aggccugaaucuuuCucuca.......................................................  1     1    T53
```

```
                                 Star                           Mature
uaucuccaucauuuucaugcgaaacuuucuggcaggccugaaucuuugucucaaccuucuguaaugaaaggugagcaaaguuucagguguguuugggggacuucuuuu
.................................aggccugaaucuGugucuca.......................................................   1     1   T53
.................................aggccugUaucuuugucuca.......................................................   5     1   T53
.................................aggccugaaucuuugucucG.......................................................   1     1   T53
.................................aggccugaCucuuugucuca.......................................................   1     1   T53
.................................aggccugaaucuuugucucC.......................................................   1     1   T53
.................................aggccAgaaucuuugucuca.......................................................   3     1   T53
.................................aggccugaaucuuugAcuca.......................................................   4     1   T53
.................................aggccugaaucuuuguGuca.......................................................   2     1   T53
.................................aggccugaauUuuugucucaa......................................................   1     1   T53
.................................aggccugaaucuuugAcucaa......................................................   1     1   T53
.................................Gggccugaaucuuugucucaa......................................................   1     1   T53
.................................aggccugaaucuuAgucucaa......................................................   6     1   T53
.................................aggccugaaucuuCgucucaa......................................................   1     1   T53
.................................aggccugaUucuuugucucaa......................................................   2     1   T53
.................................aggAcugaaucuuugucucaa......................................................   2     1   T53
.................................aggccugaaucuuuguUucaa......................................................   4     1   T53
.................................aggccugaaucCuugucucaa......................................................   3     1   T53
.................................aggUcugaaucuuugucucaa......................................................   3     1   T53
.................................aggccugaaucuuugucucaC......................................................   1     1   T53
.................................aggccugaaucuuGgucucaa......................................................   1     1   T53
.................................aggccugUaucuuugucucaa......................................................   2     1   T53
.................................aggccugaaucuCugucucaa......................................................   1     1   T53
.................................aggccugaaucuuugucucUa......................................................   1     1   T53
.................................aggccugaaucuuugGcucaa......................................................   1     1   T53
.................................aggccGgaaucuuugucucaa......................................................   1     1   T53
.................................aggccCgaaucuuugucucaa......................................................   3     1   T53
.................................aggccugaaucuuugucucaa......................................................   3652  0   T53
.................................aggccugaaucuuugucuUaa......................................................   1     1   T53
.................................aggccugaaucuuugucucaU......................................................   51    1   T53
.................................aggccugaaucuAugucucaa......................................................   1     1   T53
.................................Uggccugaaucuuugucucaa......................................................   1     1   T53
.................................aggccugaaucuGugucucaac.....................................................   2     1   T53
.................................aggccuCaaucuuugucucaac.....................................................   1     1   T53
.................................aggccugaaucuuCgucucaac.....................................................   1     1   T53
.................................aggccugaaucuuuAucucaac.....................................................   2     1   T53
.................................agUccugaaucuuugucucaac.....................................................   3     1   T53
.................................aggccugUaucuuugucucaac.....................................................   3     1   T53
.................................aggccugaaucuuuguAucaac.....................................................   1     1   T53
.................................aggccAgaaucuuugucucaac.....................................................   2     1   T53
.................................aggccugaaucuuugucucaac.....................................................   3895  0   T53
.................................aggGcugaaucuuugucucaac.....................................................   1     1   T53
.................................aggccugaaucuuAgucucaac.....................................................   5     1   T53
.................................aggccCgaaucuuugucucaac.....................................................   3     1   T53
.................................aggccugaaucuuugucucaaA.....................................................   19    1   T53
.................................aggccugaaucuuugucucGac.....................................................   2     1   T53
.................................aggccugaaucuuugucucaaU.....................................................   154   1   T53
.................................aggccugaaucuuGgucucaac.....................................................   1     1   T53
.................................aggccugaaucuuuCucucaac.....................................................   1     1   T53
.................................Uggccugaaucuuugucucaac.....................................................   2     1   T53
.................................aggccugaaucuuugucuAaac.....................................................   1     1   T53
.................................aggccuAaaucuuugucucaac.....................................................   1     1   T53
.................................aggccugaaucuuugucucaaG.....................................................   3     1   T53
.................................aggccugaaucuuugAcucaac.....................................................   1     1   T53
.................................aggUcugaaucuuugucucaac.....................................................   2     1   T53
.................................aggAcugaaucuuugucucaac.....................................................   1     1   T53
.................................aggccugaaucCuugucucaac.....................................................   2     1   T53
.................................aggccugaaucuCugucucaac.....................................................   1     1   T53
.................................aggccuUaaucuuugucucaac.....................................................   1     1   T53
.................................aggccugaaucuuugucucaUc.....................................................   1     1   T53
.................................aggccugaUucuuugucucaac.....................................................   2     1   T53
.................................aggccugaaucuAugucucaacc....................................................   1     1   T53
.................................aggccugaaucuuAgucucaacc....................................................   1     1   T53
.................................aggccuAaaucuuugucucaacc....................................................   1     1   T53
.................................aggccugaaucuuugucucGacc....................................................   1     1   T53
.................................aggccugaaucuuugucucaacc....................................................   862   0   T53
.................................aggccugaaucuuugucuUaacc....................................................   3     1   T53
.................................aggccugaaucuuugucucaacG....................................................   6     1   T53
.................................aggccugaaucuuugCcucaacc....................................................   1     1   T53
.................................aggccugaaucuuugucucaacU....................................................   51    1   T53
.................................aggccugaaucuuuUucucaacc....................................................   1     1   T53
```

```
                                 Star                                Mature
uaucuccaucauuuucaugcgaaacuuucuggcaggccugaaucuuugucucaaccuucuguaaugaaaggugagcaaaguuucaggugugu uugggggacuucuuuu
.................................aggccugaaucuuugucucaacA....................................................  52   1  T53
.................................aUgccugaaucuuugucucaacc....................................................  1    1  T53
.................................aggccugaaucuuugucucaaUc....................................................  2    1  T53
.................................aggccugaaucuuugucucaacAu...................................................  6    1  T53
.................................agAccugaaucuuugucucaaccu...................................................  1    1  T53
.................................aggUcugaaucuuugucucaaccu...................................................  1    1  T53
.................................aggccugaaucuuugucucaaccu...................................................  250  0  T53
.................................aggccugaaucuuugucucaacUu...................................................  2    1  T53
.................................aggccuAaaucuuugucucaaccu...................................................  1    1  T53
.................................aggccugaaucuAugucucaaccu...................................................  1    1  T53
.................................aggccugaaucuuugucucGaccu...................................................  1    1  T53
.................................aggccugaaucuuugucucaaccA...................................................  6    1  T53
.................................aggccugaaucuuugucucaaccuC..................................................  1    1  T53
.................................aggccugaaucuuugucucaaccuA..................................................  1    1  T53
.................................aggccugaaucuuuguAucaaccuu..................................................  1    1  T53
.................................Gggccugaaucuuugucucaaccuu..................................................  1    1  T53
.................................aggccugaaucuuugucucaaccAu..................................................  1    1  T53
.................................aggccugaaucuuugucGcaaccuu..................................................  1    1  T53
.................................aggccugaaucuuugucucaaccuu..................................................  111  0  T53
.................................aggccugaaucuuugucucaaccuuc.................................................  1    0  T53
.................................aggccugaaucuuugucucaaccuuU.................................................  8    1  T53
..................................ggccugaaucuuugucuca.......................................................  3    0  T53
..................................ggccugaaucuuugucucaa......................................................  5    0  T53
..................................ggccugaaucuuugucucaac.....................................................  3    0  T53
..................................ggccugaauUuuugucucaac.....................................................  1    1  T53
..................................ggccugaaucuuugucucaaccu...................................................  8    0  T53
..................................ggccugaaucuuugucucaaccuu..................................................  1    0  T53
...................................gccugaaucuuugucucaaccuu..................................................  1    0  T53
....................................ccugaaucuuugucucaa......................................................  2    0  T53
....................................ccugaaucuuugucucaac.....................................................  2    0  T53
....................................ccugaaucuuugucucaaU.....................................................  2    1  T53
....................................ccugaaucuuugucucaacc....................................................  2    0  T53
....................................ccugaaucuuugucucaaccu...................................................  9    0  T53
.....................................cugaaucuuugucucaac.....................................................  1    0  T53
.....................................cugaaucuuugucucaacc....................................................  1    0  T53
.....................................cugaaucuuugucucaaccu...................................................  4    0  T53
.....................................cugaaucuuugucucaaccuu..................................................  1    0  T53
......................................ugaaucuuugucucaacc....................................................  1    0  T53
.................................................................gaaGggugagcaaaguuucaggugugu................  1    1  T53
..................................................................aaGggugagcaaaguuucaggugugu................  1    1  T53
...................................................................aGggugagcaaaguuucaggugugu................  4    1  T53
....................................................................aUgugagcaaaguuucaggugugu................  1    1  T53
....................................................................aggugagcaaaguuucaggugugu................  1    0  T53
....................................................................aAgugagcaaaguuucaggugugu................  1    1  T53
....................................................................Uggugagcaaaguuucaggugugu................  3    1  T53
.....................................................................ggugagcaaaguuucagg.....................  1    0  T53
.....................................................................Ugugagcaaaguuucaggugu..................  1    1  T53
.....................................................................ggugagcaaaguuucaggugu..................  15   0  T53
.....................................................................ggugagcaaaguuucagguguU.................  9    1  T53
.....................................................................Ugugagcaaaguuucaggugug.................  1    1  T53
.....................................................................ggugagcaaaguuucaggugug.................  24   0  T53
.....................................................................ggugagcaaaguuucaggugugu................  314  0  T53
.....................................................................Agugagcaaaguuucaggugugu................  3    1  T53
.....................................................................ggugagcaaaguuucagguguUu................  9    1  T53
.....................................................................ggugCgcaaaguuucaggugugu................  1    1  T53
.....................................................................ggugagcaaaguuucaggugugA................  15   1  T53
.....................................................................Ugugagcaaaguuucaggugugu................  74   1  T53
.....................................................................Cgugagcaaaguuucaggugugu................  1    1  T53
.....................................................................ggugagcaaaguuucUggugugu................  1    1  T53
.....................................................................ggugagcaaaguuucaUgugugu................  1    1  T53
.....................................................................ggugaAcaaaguuucagguguguu...............  1    1  T53
.....................................................................ggugagcaaaguuucagguguguu...............  5    0  T53
.....................................................................ggugagcaaaguuucaggugugA...............  1    1  T53
.....................................................................ggugagcaaaguuucaggugugAu...............  4    1  T53
.....................................................................Ugugagcaaaguuucagguguguu...............  2    1  T53
.....................................................................ggugagcaaaguuucagguguguuA..............  1    1  T53
.....................................................................ggugagcaaaguuucagguguguuu..............  2    0  T53
......................................................................gugagcaaaguuucaggA....................  2    1  T53
......................................................................gugagcaaaguuucaggu....................  79   0  T53
......................................................................gugagcaaaguuucaggug...................  72   0  T53
```

Star **Mature**

```
uaucuccaucauuuucaugcgaaacuuucuggcaggccugaaucuuugucucaaccuucuguaaugaaaggugagcaaaguuucagguguguuugggggacuucuuuu
......................................................................gugagcaaaCuuucaggug...................   1        1    T53
......................................................................gugagcaaaguuuUaggug...................   1        1    T53
......................................................................gugagcaaaguuucagguU...................   1        1    T53
......................................................................gugagcaaaguuucagguC...................   1        1    T53
......................................................................gugagcaaaguuucUggug...................   1        1    T53
......................................................................gugagcaaaguuucaggAg...................   1        1    T53
......................................................................gugUgcaaaguuucaggugu..................   2        1    T53
......................................................................gugagcaaaguCucaggugu..................   2        1    T53
......................................................................gugagcaaaguuucUggugu..................   3        1    T53
......................................................................gugagcaaaguuucaUgugu..................   1        1    T53
......................................................................gugagcaaaguuAcaggugu..................   1        1    T53
......................................................................gugagcaaaguuucaggugu..................   1510     0    T53
......................................................................gugagcaaaguuucaggGgu..................   2        1    T53
......................................................................gugagcaaaguuucagguAu..................   1        1    T53
......................................................................gugaAcaaaguuucaggugu..................   1        1    T53
......................................................................gugagcaaaguuucagguguC.................   6        1    T53
......................................................................gugagcaaaCuuucaggugug.................   1        1    T53
......................................................................gGgagcaaaguuucaggugug.................   1        1    T53
......................................................................gugagcaaUguuucaggugug.................   3        1    T53
......................................................................guUagcaaaguuucaggugug.................   1        1    T53
......................................................................gugagcaaaguuucagAugug.................   2        1    T53
......................................................................gugagcGaaguuucaggugug.................   1        1    T53
......................................................................gugUgcaaaguuucaggugug.................   1        1    T53
......................................................................gugagcaaaguuucaggugug.................   2786     0    T53
......................................................................gugagcaaaguuucaggugCg.................   3        1    T53
......................................................................gugagcaaaguuAcaggugug.................   2        1    T53
......................................................................gCgagcaaaguuucaggugug.................   1        1    T53
......................................................................gugagcaaaguuucagguguA.................   4        1    T53
......................................................................gugagcaaaguuucagguguU.................   271      1    T53
......................................................................gugagcaaaguuucaUgugug.................   1        1    T53
......................................................................gugagcaaaguuucaggCgug.................   2        1    T53
......................................................................gugagcaaaguuucaggugAg.................   1        1    T53
......................................................................gugagUaaaguuucaggugug.................   1        1    T53
......................................................................gugagcaaaguuucaggGgug.................   1        1    T53
......................................................................gugaAcaaaguuucaggugug.................   2        1    T53
......................................................................gugagcaaagCuucaggugug.................   1        1    T53
......................................................................gugagcaaaguuucUggugug.................   3        1    T53
......................................................................gugagAaaaguuucaggugug.................   1        1    T53
......................................................................gugagcaaaguuucagUugug.................   1        1    T53
......................................................................gugagcaaaguuuUaggugug.................   2        1    T53
......................................................................gugagcaaaAuuucaggugugu................   6        1    T53
......................................................................gugagcaaaUuuucaggugugu................   2        1    T53
......................................................................gugagcaaaguAucaggugugu................   24       1    T53
......................................................................gugagcaaaguuucaggugGgu................   24       1    T53
......................................................................gGgagcaaaguuucaggugugu................   22       1    T53
......................................................................gugagcaaaguuucaggugAgu................   47       1    T53
......................................................................gugUgcaaaguuucaggugugu................   75       1    T53
......................................................................gugagcaaaguuucaggugugA................   2688     1    T53
......................................................................gugagcaaaCuuucaggugugu................   82       1    T53
......................................................................gugagcaaaguuucaggugugu................   219630   0    T53
......................................................................gugagcaaaguuucUggugugu................   172      1    T53
......................................................................gugagcaaaguuucagguguCu................   14       1    T53
......................................................................gAgagcaaaguuucaggugugu................   8        1    T53
......................................................................gugagcaaaguuucaggugCgu................   7        1    T53
......................................................................guUagcaaaguuucaggugugu................   16       1    T53
......................................................................Cugagcaaaguuucaggugugu................   13       1    T53
......................................................................gugagcaaaguuucagguguAu................   37       1    T53
......................................................................gugagcaUaguuucaggugugu................   9        1    T53
......................................................................gugagcaaaguuucaUgugugu................   49       1    T53
......................................................................gugagcaaagCuucaggugugu................   10       1    T53
......................................................................gugagcaaaguuuAaggugugu................   15       1    T53
......................................................................gugagAaaaguuucaggugugu................   23       1    T53
......................................................................gugagcaaCguuucaggugugu................   11       1    T53
......................................................................gugagcaaaguuucagguguUu................   86       1    T53
......................................................................gugagcaaaguuucaAgugugu................   39       1    T53
......................................................................gugagGaaaguuucaggugugu................   57       1    T53
......................................................................gugagcaaGguuucaggugugu................   16       1    T53
......................................................................gugagcaaaguuucagCugugu................   24       1    T53
......................................................................gugagcaaaguGucaggugugu................   4        1    T53
......................................................................gugagcaGaguuucaggugugu................   10       1    T53
```

```
                                Star                     Mature
uaucuccaucauuuucaugcgaaacuuucuggcaggccugaaucuuugucucaaccuucuguaaugaaaggugagcaaaguuucagguguguuugggggacuucuuuu
......................................................................gugagcaaaguuuUaggugugu................   86     1     T53
......................................................................gugagcGaaguuucaggugugu................   8      1     T53
......................................................................gugagcUaaguuucaggugugu................   3      1     T53
......................................................................gugagcaaaguuucaggugugG................   91     1     T53
......................................................................gugagcaaaguuucagguAugu................   47     1     T53
......................................................................gugagcaaaguuucaggAgugu................   50     1     T53
......................................................................gugaCcaaaguuucaggugugu................   13     1     T53
......................................................................gugCgcaaaguuucaggugugu................   1      1     T53
......................................................................gCgagcaaaguuucaggugugu................   30     1     T53
......................................................................gugagcaaaguuGcaggugugu................   8      1     T53
......................................................................gugagcaaagAuucaggugugu................   5      1     T53
......................................................................gugagcaaaguuucagguCugu................   22     1     T53
......................................................................gugaUcaaaguuucaggugugu................   35     1     T53
......................................................................gugagcaaaguuucCggugugu................   15     1     T53
......................................................................gugagcaaaguuuGaggugugu................   54     1     T53
......................................................................gugagcaaaguuCcaggugugu................   10     1     T53
......................................................................gugagcaaaguCucaggugugu................   9      1     T53
......................................................................gugagcaaaguuucaCgugugu................   41     1     T53
......................................................................guCagcaaaguuucaggugugu................   6      1     T53
......................................................................gugagcaaUguuucaggugugu................   151    1     T53
......................................................................gugagUaaaguuucaggugugu................   111    1     T53
......................................................................gugagcaaaguuAcaggugugu................   73     1     T53
......................................................................Uugagcaaaguuucaggugugu................   13     1     T53
......................................................................gugagcaaaguuucagUugugu................   69     1     T53
......................................................................gugagcaaaguuucGggugugu................   123    1     T53
......................................................................gugagcaaagGuucaggugugu................   20     1     T53
......................................................................gugaAcaaaguuucaggugugu................   61     1     T53
......................................................................gugagcaaaguuucaggugugC................   138    1     T53
......................................................................gugagcaaaguuucaggCgugu................   9      1     T53
......................................................................guAagcaaaguuucaggugugu................   11     1     T53
......................................................................gugagcaaaguuucaggGgugu................   48     1     T53
......................................................................gugGgcaaaguuucaggugugu................   17     1     T53
......................................................................gugagcaaaguuucagguUugu................   32     1     T53
......................................................................gugagcaaaguuucagAugugu................   38     1     T53
......................................................................gugagcaaaguuucUgguguguu...............   9      1     T53
......................................................................gugagcaaaguuucaggugCguu...............   1      1     T53
......................................................................gugagcaaaguuucagguAuguu...............   3      1     T53
......................................................................gugagcaaaguuucagguguAuu...............   1      1     T53
......................................................................gugagcaaaguuucagAuguguu...............   5      1     T53
......................................................................gugagcaaaguuuGagguguguu...............   1      1     T53
......................................................................gugaAcaaaguuucagguguguu...............   1      1     T53
......................................................................gugagcaaaguuucagguUuguu...............   1      1     T53
......................................................................gugagcaaaguuucaggugugAu...............   17     1     T53
......................................................................gugagcaaaguuucaggAguguu...............   1      1     T53
......................................................................gugagcaaaCuuucagguguguu...............   6      1     T53
......................................................................gCgagcaaaguuucagguguguu...............   1      1     T53
......................................................................gugagcaaaguuucaggugugGu...............   2      1     T53
......................................................................gugagcaaaguuucaggGguguu...............   3      1     T53
......................................................................gugagGaaaguuucagguguguu...............   2      1     T53
......................................................................gugagcaaaguuucaggCguguu...............   1      1     T53
......................................................................Augagcaaaguuucagguguguu...............   2      1     T53
......................................................................gugaCcaaaguuucagguguguu...............   2      1     T53
......................................................................gugagcaaaguuucGgguguguu...............   4      1     T53
......................................................................gugagcaaaguuucagguguCuu...............   1      1     T53
......................................................................gugagAaaaguuucagguguguu...............   1      1     T53
......................................................................gugagcaaaguuucagCuguguu...............   2      1     T53
......................................................................gugagcaaaguuucagguguUuu...............   6      1     T53
......................................................................gugagcaaaguuucagUuguguu...............   3      1     T53
......................................................................gugagcaaaguuuUagguguguu...............   5      1     T53
......................................................................gugCgcaaaguuucagguguguu...............   1      1     T53
......................................................................gugUgcaaaguuucagguguguu...............   3      1     T53
......................................................................Cugagcaaaguuucagguguguu...............   1      1     T53
......................................................................gugagcaaaguuucagguguguu...............   7643   0     T53
......................................................................gugagcaaaguuAcagguguguu...............   2      1     T53
......................................................................gugagcaaaguuucaggugAguu...............   2      1     T53
......................................................................gugagcaaaguCucagguguguu...............   1      1     T53
......................................................................gugagcaaaguuucagguguguA...............   616    1     T53
......................................................................gugagcaaUguuucagguguguu...............   6      1     T53
......................................................................gugagcaaagCuucagguguguu...............   1      1     T53
......................................................................gugagUaaaguuucagguguguu...............   10     1     T53
```

Star **Mature**

```
uaucuccaucauuuucaugcgaaacuuucuggcaggccugaaucuuugucucaaccuucuguaaugaaaggugagcaaaguuucagguguguuugggggacuucuuuu
......................................................................gugagcaaaguuucaAgugugu................  3     1  T53
......................................................................gugagcaaaguuucaggugugG................  38    1  T53
......................................................................gugagcaaaguuucagguguguuG..............  10    1  T53
......................................................................gugagcaaaguuucagguguguuA..............  90    1  T53
......................................................................gugagcaaaguuucagAuguguuu..............  1     1  T53
......................................................................gugagcaaaguuucagguguguuu..............  250   0  T53
......................................................................gugagUaaaguuucagguguguuu..............  1     1  T53
......................................................................gugagcaaaguuucaggugugugCu..............  4     1  T53
......................................................................gugagcaaaguuucagguguguGu..............  6     1  T53
......................................................................gugagcaaaguuucagguguguAu..............  35    1  T53
......................................................................gugagcaaaguuucagguguguuC..............  6     1  T53
......................................................................gugagcaaaguuucagguguguuuA.............  1     1  T53
......................................................................gugagcaaaguuucagguguguuuU.............  16    1  T53
......................................................................gugagcaaaguuucagguguguuuC.............  1     1  T53
......................................................................gugagcaaaguuucagguguguuCg.............  3     1  T53
.......................................................................ugagcaaaguuucaggugug.................  1     0  T53
.......................................................................ugagcaaaguuucaggugugu................  21    0  T53
.......................................................................Ggagcaaaguuucaggugugu................  2     1  T53
.......................................................................ugagcaaaguuucaggugugA................  2     1  T53
.......................................................................ugagcaaaguuucagguguguu...............  1     0  T53
........................................................................gagcaaaguuucaggugugu................  55    0  T53
.........................................................................agcaaaguuucaggugug.................  1     0  T53
.........................................................................aAcaaaguuucaggugugu................  1     1  T53
.........................................................................agcaaaguuucaggugugu................  21    0  T53
.........................................................................agcaaaguuucagguguguGug.............  2     1  T53
..........................................................................gcaaaguuucaggugugu................  22    0  T53
..........................................................................gcaaaguuucGggugugu................  1     1  T53
..........................................................................gcaaaguuucagguguguu...............  1     0  T53
............................................................................aaaguuucagguguguuu..............  1     0  T53

.................................aggccugaaucuuugucu.........................................................  2     0  te1
.................................Gggccugaaucuuugucu.........................................................  1     1  te1
.................................Gggccugaaucuuugucuc........................................................  1     1  te1
.................................aggccugaaucuuugucuU........................................................  1     1  te1
.................................aUgccugaaucuuugucuc........................................................  1     1  te1
.................................aggccugaaucuuugucuc........................................................  39    0  te1
.................................Uggccugaaucuuugucuca.......................................................  2     1  te1
.................................aggcAugaaucuuugucuca.......................................................  2     1  te1
.................................aggccugaauUuuugucuca.......................................................  1     1  te1
.................................aggccugaauAuuugucuca.......................................................  1     1  te1
.................................aggccugaaucuuugucucU.......................................................  8     1  te1
.................................Cggccugaaucuuugucuca.......................................................  2     1  te1
.................................aggccugaaucuuugucuca.......................................................  1231  0  te1
.................................Gggccugaaucuuugucuca.......................................................  3     1  te1
.................................aggccugaaucuuugucCca.......................................................  1     1  te1
.................................aggccugaaucuuugGcuca.......................................................  1     1  te1
.................................aggUcugaaucuuugucucaa......................................................  1     1  te1
.................................Uggccugaaucuuugucucaa......................................................  1     1  te1
.................................aggccugaauUuuugucucaa......................................................  3     1  te1
.................................aggccugaaucuuugucuUaa......................................................  1     1  te1
.................................aggccugaaucuuugucucaa......................................................  209   0  te1
.................................aggccugaaucuuugucucaU......................................................  4     1  te1
.................................Uggccugaaucuuugucucaac.....................................................  2     1  te1
.................................Gggccugaaucuuugucucaac.....................................................  1     1  te1
.................................aggccugaaucuuugucucaaG.....................................................  1     1  te1
.................................aggcUugaaucuuugucucaac.....................................................  2     1  te1
.................................aAgccugaaucuuugucucaac.....................................................  1     1  te1
.................................aggccugaaucuuugucucaac.....................................................  527   0  te1
.................................aggccugaaucuuugGcucaac.....................................................  1     1  te1
.................................aggccugaaucuuuguUucaac.....................................................  1     1  te1
.................................aggccugaaucuuugucucaaA.....................................................  3     1  te1
.................................aggccugaaucuuugucucaaU.....................................................  14    1  te1
.................................aggccugaaucuuugucuUaac.....................................................  1     1  te1
.................................aggAcugaaucuuugucucaac.....................................................  1     1  te1
.................................aggccugaaucuuugucucaacc....................................................  584   0  te1
.................................aggccugaaucuuugucuUaacc....................................................  1     1  te1
.................................aggccugaaucuuugucucaaUc....................................................  2     1  te1
.................................Uggccugaaucuuugucucaacc....................................................  2     1  te1
.................................aggccugaaucuuuCucucaacc....................................................  1     1  te1
.................................aggccugaaucuuugucucaacU....................................................  19    1  te1
```

Star Mature

uaucuccaucauuuucaugcgaaacuuucuggcaggccugaaucuuugucucaaccuucuguaaugaaaggugagcaaaguuucagguguguuugggggacuucuuuu			
.................................aggccugaaucuuugucucaacA..	6	1	te1
.................................aAgccugaaucuuugucucaacc..	1	1	te1
.................................aggccugaaucuuugucucaaccA...	1	1	te1
.................................aggccugaaucuuugucCcaaccu...	1	1	te1
.................................aggccugaaucuuugucucaaccu...	101	0	te1
.................................Uggccugaaucuuugucucaaccu...	1	1	te1
.................................aggccugaaucuuugucucaacUu...	1	1	te1
.................................aggccugaaucuuugucucaaccuA..	1	1	te1
.................................aggccugaaucuuugucucaaUcuu..	1	1	te1
.................................Cggccugaaucuuugucucaaccuu..	1	1	te1
.................................aggccugaaucuuugucucaaccuu..	32	0	te1
.................................aggccugaaucuuugucucaaccuuU...	7	1	te1
..................................ggccugaaucuuugucucaac...	2	0	te1
..................................ggccugaaucuuugucucaacc..	1	0	te1
..................................ggccugaaucuuugucucaaccu...	4	0	te1
..................................ggccugaaucuuugucucaaccuu..	1	0	te1
....................................ccugaaucuuugucucaa..	1	0	te1
....................................ccugaaucuuugucucaacc..	1	0	te1
.....................................cugaaucuuugucucaaccu...	1	0	te1
......................................ugaaucuuugucucaaccu...	2	0	te1
....gaaaCgugagcaaaguuucaggugugu................	1	1	te1
....Gaaggugagcaaaguuucaggugugu................	1	1	te1
....aCggugagcaaaguuucaggugugu................	1	1	te1
....Gaggugagcaaaguuucaggugugu................	1	1	te1
....aGggugagcaaaguuucaggugugu................	2	1	te1
....Gggugagcaaaguuucaggugugu................	1	1	te1
....ggugagcaaaguuucagg.....................	2	0	te1
....ggugagcaaaguuucaggugu..................	30	0	te1
....Ugugagcaaaguuucaggugu..................	1	1	te1
....ggugagcaaaguuucaggugug.................	17	0	te1
....ggugagcaaaguuucagguguU.................	10	1	te1
....gAugagcaaaguuucaggugugu................	9	1	te1
....ggugagcaaaguuucaggugugA................	15	1	te1
....ggugagcaaaguuucaggugugG................	1	1	te1
....ggugagcaaaguuucaggugugu................	334	0	te1
....ggugagcaaaguuuUaggugugu................	1	1	te1
....Ugugagcaaaguuucaggugugu................	26	1	te1
....ggugagcaaaguuucagUugugu................	1	1	te1
....ggugagcaaaguuucagguguUu................	1	1	te1
....ggugagcaaaguuucaggugugAu...............	1	1	te1
....ggugagcaaaguuucagguguguu...............	4	0	te1
....ggugagcaaaguuucagguguguuu..............	1	0	te1
....gugagcaaaguuucaggu....................	59	0	te1
....gugagcaaaguuucagguU...................	1	1	te1
....gugagcaaaguuucagguC...................	1	1	te1
....gugagcaaagCuucaggug...................	1	1	te1
....gugagcaaaguuucaggug...................	45	0	te1
....gugagcaaaguuucaggGgu..................	2	1	te1
....gugagcaaagGuucaggugu..................	1	1	te1
....gugagcaaaguuuGaggugu..................	1	1	te1
....gugagAaaaguuucaggugu..................	1	1	te1
....gugagcaaaguuucaggCgu..................	1	1	te1
....Uugagcaaaguuucaggugu..................	1	1	te1
....gugagcaaaguuucaggugu..................	1121	0	te1
....gugagcaaCguuucaggugu..................	1	1	te1
....gugGgcaaaguuucaggugu..................	1	1	te1
....gugagcaaagCuucaggugu..................	1	1	te1
....gugaCcaaaguuucaggugu..................	1	1	te1
....gugagcaaaguuuUaggugu..................	1	1	te1
....gugagcaaaguuucaggugC..................	3	1	te1
....gugagcaaaguuucaggCgug.................	1	1	te1
....gugagcaaaguuucagguguC.................	2	1	te1
....gugagcaaaguuuUaggugug.................	1	1	te1
....gugagUaaaguuucaggugug.................	1	1	te1
....gugagcaaaguuuGaggugug.................	1	1	te1
....gugagcaaaguuucaggAgug.................	1	1	te1
....gugGgcaaaguuucaggugug.................	1	1	te1
....gugagcaaaguuGcaggugug.................	1	1	te1
....gugagcaaaguuucGggugug.................	1	1	te1
....gugagcaaaguCucaggugug.................	1	1	te1

Star **Mature**

```
uaucuccaucauuuucaugcgaaacuuucuggcaggccugaaucuuugucucaaccuucuguaaugaaaggugagcaaaguuucagguguguuugggggacuucuuuu
......................................................................gugagcaaaguuucagguguU.................  141     1   te1
......................................................................gugagcaaaguuucaggugug.................  1531    0   te1
......................................................................gugagcaaaguuucagguguA.................  1       1   te1
......................................................................gugagcaaaguAucaggugug.................  1       1   te1
......................................................................gugagcaaagGuucaggugug.................  2       1   te1
......................................................................gugagcaaaguuucagUugugu................  8       1   te1
......................................................................gAgagcaaaguuucaggugugu................  17      1   te1
......................................................................gugagcaaaguuucUggugugu................  6       1   te1
......................................................................gugagcaaagGuucaggugugu................  17      1   te1
......................................................................Cugagcaaaguuucaggugugu................  3       1   te1
......................................................................gugagcaaaguGucaggugugu................  8       1   te1
......................................................................gugagcaUaguuucaggugugu................  3       1   te1
......................................................................gugagcaaaguuucaggugCgu................  18      1   te1
......................................................................gugagcaaaguuucaCgugugu................  6       1   te1
......................................................................gugagcaaaguuucGggugugu................  31      1   te1
......................................................................gugagcaaaguuucagguAugu................  7       1   te1
......................................................................gCgagcaaaguuucaggugugu................  11      1   te1
......................................................................gugagcCaaguuucaggugugu................  5       1   te1
......................................................................gGgagcaaaguuucaggugugu................  14      1   te1
......................................................................gugagcaaaguuucagguguUu................  12      1   te1
......................................................................gugagcaaaguAucaggugugu................  18      1   te1
......................................................................gugagcUaaguuucaggugugu................  4       1   te1
......................................................................gugagUaaaguuucaggugugu................  36      1   te1
......................................................................gugagcaaaguuucaggugAgu................  1       1   te1
......................................................................gugagcaaaguuucagCugugu................  3       1   te1
......................................................................gugagcaaaAuucaggugugu................  1       1   te1
......................................................................gugagcaaagCuucaggugugu................  20      1   te1
......................................................................gugagcaaaguuucaAgugugu................  7       1   te1
......................................................................gugagcaaaguuucaggugGgu................  19      1   te1
......................................................................gugagcaaaguCucaggugugu................  10      1   te1
......................................................................gugagcaaaguuucaggugugA................  1381    1   te1
......................................................................gugagcaaaguuucaUgugugu................  5       1   te1
......................................................................gugagcaaaguuucCggugugu................  4       1   te1
......................................................................gugagcaaaguuAcaggugugu................  1       1   te1
......................................................................gugagcaaaguuucagAugugu................  4       1   te1
......................................................................gugagcaaaguuucaggugugu................  73965   0   te1
......................................................................gugagcaCaguuucaggugugu................  9       1   te1
......................................................................gugagcaaaguuCcaggugugu................  9       1   te1
......................................................................gugagGaaaguuucaggugugu................  7       1   te1
......................................................................gugagcaaaguuucagguCugu................  4       1   te1
......................................................................gugagcaaaguuucagguguAu................  3       1   te1
......................................................................Uugagcaaaguuucaggugugu................  18      1   te1
......................................................................gugagAaaaguuucaggugugu................  6       1   te1
......................................................................gugagcaaaguuucaggAgugu................  4       1   te1
......................................................................gugagcaaaUuuucaggugugu................  1       1   te1
......................................................................gugaUcaaaguuucaggugugu................  10      1   te1
......................................................................gugagcaaGguuucaggugugu................  2       1   te1
......................................................................gugagcaaaguuucaggCgugu................  25      1   te1
......................................................................guAagcaaaguuucaggugugu................  1       1   te1
......................................................................gugagcaGaguuucaggugugu................  7       1   te1
......................................................................gugagcaaaguuucagguUugu................  11      1   te1
......................................................................gugagcaaaguuucagguguCu................  1       1   te1
......................................................................gugagcaaaCuuucaggugugu................  1       1   te1
......................................................................gugagcGaaguuucaggugugu................  4       1   te1
......................................................................guUagcaaaguuucaggugugu................  6       1   te1
......................................................................gugUgcaaaguuucaggugugu................  14      1   te1
......................................................................gugagcaaCguuucaggugugu................  1       1   te1
......................................................................gugagcaaaguuucaggugugG................  178     1   te1
......................................................................gugagcaaagAuucaggugugu................  8       1   te1
......................................................................gugagcaaaguuucaggGgugu................  24      1   te1
......................................................................gugGgcaaaguuucaggugugu................  17      1   te1
......................................................................gugCgcaaaguuucaggugugu................  31      1   te1
......................................................................guCagcaaaguuucaggugugu................  3       1   te1
......................................................................gugagcaaaguuGcaggugugu................  1       1   te1
......................................................................gugaCcaaaguuucaggugugu................  11      1   te1
......................................................................gugagcaaaguuucaggugugC................  40      1   te1
......................................................................gugagcaaaguuuGaggugugu................  15      1   te1
......................................................................gugagcaaaguuuUaggugugu................  52      1   te1
......................................................................gugaAcaaaguuucaggugugu................  24      1   te1
......................................................................Augagcaaaguuucaggugugu u..............  3       1   te1
```

```
                                Star                                Mature
uaucuccaucauuuucaugcgaaacuuucuggcaggccugaaucuuugucucaaccuucuguaaugaaaggugagcaaaguuucagguguguuuggggacuucuuuu
......................................................................gugagcaaaguuucaggugugAu..............   4      1     te1
......................................................................gugagUaaaguuucagguguguu..............   1      1     te1
......................................................................gugagcaaaguuucagguguguu..............   1625   0     te1
......................................................................gugagcaaaguuucagguguguA..............   272    1     te1
......................................................................gugagcaaaguuucagguguUuu..............   2      1     te1
......................................................................gugagcaaaguuucaggCguguu..............   2      1     te1
......................................................................gugagcaaaguuucaggAguguu..............   1      1     te1
......................................................................gugagcaaagAuucagguguguu..............   1      1     te1
......................................................................gugagcaaaguuuUagguguguu..............   1      1     te1
......................................................................gugagcaaaguuucagguguguG..............   5      1     te1
......................................................................gugaCcaaaguuucagguguguu..............   1      1     te1
......................................................................gugagcaaaguuucagUuguguu..............   1      1     te1
......................................................................gugagcaaaguuucGgguguguu..............   1      1     te1
......................................................................gugagcaaaguuucaggugCguu..............   2      1     te1
......................................................................gugagcaaagCuucagguguguu..............   1      1     te1
......................................................................gugagcaaaguuucaggGguguu..............   1      1     te1
......................................................................gugagcaaGguuucagguguguu..............   1      1     te1
......................................................................gugagcaaaguuucagguguguCu.............   3      1     te1
......................................................................gugagcaaaguuucagguguguuA.............   31     1     te1
......................................................................gugagcaaaguuucagguguguAu.............   24     1     te1
......................................................................gugagcaaaguuucagguguguuG.............   2      1     te1
......................................................................gugagcaaaguuucagguguguuu.............   51     0     te1
......................................................................gugagcaaaguuucagguguguuuA............   1      1     te1
......................................................................gugagcaaaguuucagguguguuuU............   3      1     te1
......................................................................gugagcaaaguuucagguguguuAggg..........   1      1     te1
.......................................................................ugagcaaaguuucaggugug................   2      0     te1
.......................................................................ugagcaaaguCucaggugugu...............   1      1     te1
.......................................................................ugagcaaaguuucaggugugA...............   1      1     te1
.......................................................................ugagcaaaguuucaggugugu...............   67     0     te1
.......................................................................Ggagcaaaguuucaggugugu...............   1      1     te1
.......................................................................Agagcaaaguuucaggugugu...............   1      1     te1
.......................................................................ugagcaaaguuucaggugugG...............   1      1     te1
.......................................................................ugagcCaaguuucaggugugu...............   3      1     te1
.......................................................................ugagcaaaguuucagguguguu..............   1      0     te1
........................................................................gagcaaaguuucaggugugu...............   38     0     te1
........................................................................Aagcaaaguuucaggugugu...............   1      1     te1
........................................................................gagcaaaguuucagguguguGu.............   1      1     te1
.........................................................................agcaaaguuucaggugugu...............   8      0     te1
..........................................................................gcaaaguuucaggugugu...............   29     0     te1
..........................................................................gcaaaguuuUaggugugu...............   1      1     te1
..........................................................................gcaaaguuucagguguguu..............   1      0     te1
............................................................................aaaguuucagguguguuuA............   1      1     te1

...........uuuGcaugcgaaacuuucuggc..........................................................................   1      1     egg
...............caugcgaaacuuucuggc..........................................................................   1      0     egg
.................................aggcUugaaucuuugucu........................................................   1      1     egg
.................................aggccugaaucuuugucuca......................................................   5      0     egg
.................................aggccugaaucuuugucucaa.....................................................   12     0     egg
.................................aggccugaaucuCugucucaa.....................................................   1      1     egg
.................................aggUcugaaucuuugucucaa.....................................................   1      1     egg
.................................aggccugaaucCuugucucaa.....................................................   1      1     egg
.................................aggccugaaucuCugucucaac....................................................   2      1     egg
.................................aggccugGaucuuugucucaac....................................................   2      1     egg
.................................aggccugaaucuuugucucaaU....................................................   3      1     egg
.................................aggcGugaaucuuugucucaac....................................................   1      1     egg
.................................aggccugUaucuuugucucaac....................................................   1      1     egg
.................................aggccugaaucuuugucucaac....................................................   50     0     egg
.................................aggcUugaaucuuugucucaacc...................................................   1      1     egg
.................................aggccugaaucuuugucucaacc...................................................   43     0     egg
.................................Uggccugaaucuuugucucaacc...................................................   1      1     egg
.................................aggccugaaucuuugucucGacc...................................................   1      1     egg
.................................aggccugaaucuuugucCcaacc...................................................   1      1     egg
.................................aggccugaaucuuugucucaacU...................................................   3      1     egg
.................................aggccugaaucuuugucucaacA...................................................   5      1     egg
.................................aggccugaaucuuugucucaUcc...................................................   1      1     egg
.................................aggccugaaucuuugucucaGcc...................................................   1      1     egg
.................................aggccugaaucuuugucucaaccu..................................................   4      0     egg
.................................aggccugaaucuuugucucaaccA..................................................   2      1     egg
.................................Cggccugaaucuuugucucaaccu..................................................   8      1     egg
.................................aggccugaaucuuugCcucaaccu..................................................   1      1     egg
```

Star / Mature			
uaucuccaucauuuucaugcgaaacuuucuggcaggccugaaucuuugucucaaccuucuguaaugaaaggugagcaaaguuucagguguguuugggggacuucuuuu			
.................................aggccugaaucuuugucucaaccuu..	7	0	egg
.................................Cggccugaaucuuugucucaaccuu..	1	1	egg
.................................aggcUugaaucuuugucucaaccuu..	1	1	egg
....ggugagcaaaguuucaggugug.................	3	0	egg
....ggugagcaaaguuucaggCgugu................	1	1	egg
....ggugagcaaaguuucaggugugu................	6	0	egg
....Cgugagcaaaguuucaggugugu................	2	1	egg
....gCugagcaaaguuucaggugugu................	1	1	egg
....gugagcaaaguuucaggu....................	1	0	egg
....gugagcaaGguuucaggugu..................	1	1	egg
....gugagcaaaguuucaggugu..................	1	0	egg
....Nugagcaaaguuucaggugug.................	1	1	egg
....gugagcaaaguuucaggugug.................	49	0	egg
....gugagcaGaguuucaggugug.................	1	1	egg
....gugagcaUaguuucaggugugu................	2	1	egg
....gugagcaaaguuucaggCgugu................	4	1	egg
....gugagcaaaguuGcaggugugu................	1	1	egg
....Nugagcaaaguuucaggugugu................	2	1	egg
....gugaAcaaaguuucaggugugu................	8	1	egg
....gugagcaaaguuucaggGgugu................	2	1	egg
....gugagcaaaguCucaggugugu................	4	1	egg
....gugagcaaaguuucaggugCgu................	5	1	egg
....gugUgcaaaguuucaggugugu................	11	1	egg
....gugagcaaaguuucagAugugu................	2	1	egg
....gAgagcaaaguuucaggugugu................	4	1	egg
....gugagcaaaAuuucaggugugu................	2	1	egg
....gugaUcaaaguuucaggugugu................	42	1	egg
....gugagcaaaguuucaggugugA................	27	1	egg
....gugagcaaaguuucCggugugu................	3	1	egg
....gCgagcaaaguuucaggugugu................	4	1	egg
....gugagcaaaguuCcaggugugu................	5	1	egg
....gugagcaaaguuucaAgugugu................	2	1	egg
....gugagcaaagCuucaggugugu................	3	1	egg
....gugagcaaaguuucagguguUu................	2	1	egg
....gugagcaaaguuucaggugGgu................	1	1	egg
....gugagcaaaguuucagguguAu................	1	1	egg
....gugagcGaaguuucaggugugu................	2	1	egg
....gugaCcaaaguuucaggugugu................	2	1	egg
....gugagcaaaCuuucaggugugu................	1	1	egg
....Uugagcaaaguuucaggugugu................	4	1	egg
....guAagcaaaguuucaggugugu................	1	1	egg
....gugagcaaaguuucaUgugugu................	1	1	egg
....gugagcaaaguuucUggugugu................	1	1	egg
....gugagcaaGguuucaggugugu................	32	1	egg
....gugagcaaaguGucaggugugu................	1	1	egg
....gugagAaaaguuucaggugugu................	1	1	egg
....gugagGaaaguuucaggugugu................	2	1	egg
....gugagcaGaguuucaggugugu................	5	1	egg
....gugagcaaaguuucaggugugC................	90	1	egg
....gugagcaaaguuucaggugugG................	4	1	egg
....gugGgcaaaguuucaggugugu................	7	1	egg
....gugagcaaaguuucGggugugu................	1	1	egg
....gugagcaaUguuucaggugugu................	1	1	egg
....gugagcaaaguuucaggugugu................	1262	0	egg
....gugagUaaaguuucaggugugu................	1	1	egg
....gugagcaaaguuucagguAugu................	3	1	egg
....gugUgcaaaguuucagguguguu...............	1	1	egg
....gugagcaaaguuucagguguguu...............	41	0	egg
....gugaUcaaaguuucagguguguu...............	1	1	egg
....gugagcaaaguuucaggugugugA...............	14	1	egg
....gugagcaaaguuucaggugugugAu..............	2	1	egg
....gugagcaaaguuucagguguguuA..............	1	1	egg
....gugagcaaaguuucagguguguuug.............	1	0	egg
....ugagcaaaguuucaggugugu................	1	0	egg
................................Uaggccugaaucuuugucuca...	1	1	T6P
................................Uaggccugaaucuuugucucaacc..	2	1	T6P
.................................aggccugaaucuuugucuc..	25	0	T6P
.................................aggccugaaucuuugCcuca...	1	1	T6P
.................................agAccugaaucuuugucuca...	1	1	T6P

Star **Mature**

```
uaucuccaucauuuucaugcgaaacuuucuggcaggccugaaucuuugucucaaccuucuguaaugaaaggugagcaaaguuucaggugugu uugggggacuucuuuu
.................................aggccuCaaucuuugucuca.......................................................  1     1  T6P
.................................aggccugaaucuuCgucuca.......................................................  1     1  T6P
.................................aggccugaaucuuugucuca.......................................................  254   0  T6P
.................................aggccugaaucuCugucuca.......................................................  1     1  T6P
.................................aAgccugaaucuuugucuca.......................................................  1     1  T6P
.................................aggccugaaucuuugAcuca.......................................................  1     1  T6P
.................................aggccugaaucuuugucucaU......................................................  2     1  T6P
.................................aggccugaaucuuugucucaa......................................................  208   0  T6P
.................................aggccugaaucAuugucucaa......................................................  1     1  T6P
.................................agUccugaaucuuugucucaa......................................................  1     1  T6P
.................................aggccugaaucuuugCcucaac.....................................................  1     1  T6P
.................................aggccugGaucuuugucucaac.....................................................  1     1  T6P
.................................aggccugaaucuuuUucucaac.....................................................  1     1  T6P
.................................aggccugaauUuuugucucaac.....................................................  2     1  T6P
.................................aggccugaaucuuuguAucaac.....................................................  1     1  T6P
.................................aggccugaaucuuuCucucaac.....................................................  2     1  T6P
.................................aggUcugaaucuuugucucaac.....................................................  1     1  T6P
.................................aggccugaaucuuuguUucaac.....................................................  1     1  T6P
.................................aggccugaaucuuugucucaaU.....................................................  10    1  T6P
.................................aggccugaaucuuugucucaaA.....................................................  1     1  T6P
.................................aggccugaaucuuCgucucaac.....................................................  2     1  T6P
.................................aggccugaaucuuugucucaac.....................................................  1174  0  T6P
.................................aggccugUaucuuugucucaac.....................................................  1     1  T6P
.................................aggccugaaucuCugucucaac.....................................................  1     1  T6P
.................................aggccugaaucuuugAcucaac.....................................................  1     1  T6P
.................................aggccugaaucCuugucucaac.....................................................  1     1  T6P
.................................aggccCgaaucuuugucucaacc....................................................  1     1  T6P
.................................aggccugaaucuuugucuUaacc....................................................  1     1  T6P
.................................aggccugaaucuuuAucucaacc....................................................  1     1  T6P
.................................aggccugaaucuuuguUucaacc....................................................  1     1  T6P
.................................aggccugaaucuuugucucaacA....................................................  2     1  T6P
.................................aggccugaaucuuugucucaacc....................................................  1784  0  T6P
.................................aCgccugaaucuuugucucaacc....................................................  2     1  T6P
.................................aggccugaaucuuugGcucaacc....................................................  1     1  T6P
.................................aggccugaaucuuugucucaaUc....................................................  3     1  T6P
.................................aggccugGaucuuugucucaacc....................................................  1     1  T6P
.................................aggccuCaaucuuugucucaacc....................................................  1     1  T6P
.................................aggccugaaucuuugucucaacU....................................................  20    1  T6P
.................................aggccuUaaucuuugucucaacc....................................................  1     1  T6P
.................................aggccugaaucuuugucuAaacc....................................................  1     1  T6P
.................................aggccuAaaucuuugucucaacc....................................................  2     1  T6P
.................................aggccugaaucuuugucucaacUu...................................................  2     1  T6P
.................................aggccuAaaucuuugucucaaccu...................................................  1     1  T6P
.................................aggccugaaucuuugucuUaaccu...................................................  1     1  T6P
.................................aggccugaaucuuugucucaaccu...................................................  532   0  T6P
.................................aggccugaUucuuugucucaaccu...................................................  1     1  T6P
.................................aggccugaaucuuugucucaaccC...................................................  1     1  T6P
.................................aCgccugaaucuuugucucaaccu...................................................  1     1  T6P
.................................aggccugaaucuuugucucaGccu...................................................  1     1  T6P
.................................aggccugaauUuuugucucaaccu...................................................  2     1  T6P
.................................aUgccugaaucuuugucucaaccu...................................................  1     1  T6P
.................................aggccugaaucuuugGcucaaccu...................................................  1     1  T6P
.................................aggccugaaucuuugucucaUccu...................................................  1     1  T6P
.................................aggccugaaucuuugucucaaUcu...................................................  1     1  T6P
.................................aggUcugaaucuuugucucaaccu...................................................  2     1  T6P
.................................aggccugaaucuuugucucaaccA...................................................  2     1  T6P
.................................aggccugaaucuuugucucaaccuC..................................................  1     1  T6P
.................................aggccugaaucuuugucucaaccuA..................................................  7     1  T6P
.................................aggccugaaucuuugucucaaccuu..................................................  250   0  T6P
.................................aggccugaaucuuugucucaaccuuA.................................................  1     1  T6P
.................................aggccugaaucuuugucucaaccuuU.................................................  34    1  T6P
..................................ggccugaaucuuugucuca.......................................................  1     0  T6P
..................................ggccugaaucuuugucucaacc....................................................  7     0  T6P
..................................ggccugaaucuuugucucaaccu...................................................  6     0  T6P
..................................ggccugaaucuuugucucaaccuu..................................................  11    0  T6P
..................................ggccugaaucuuugucucaaccuuU.................................................  1     1  T6P
...................................gccugaaucuuugucucaaccu...................................................  1     0  T6P
....................................ccugaaucuuugucucaa......................................................  1     0  T6P
....................................ccugaaucuuugucucaaccu...................................................  3     0  T6P
.....................................cugaaucuuugucucaacc....................................................  1     0  T6P
```

Star **Mature**

uaucuccaucauuuucaugcgaaacuuucuggcaggccugaaucuuugucucaaccuucuguaaugaaaggugagcaaaguuucagguguguuugggggacuucuuuu			
.....................................cugaaucuuugucucaaccu...	1	0	T6P
......................................ugaaucuuugucucaaccu...	2	0	T6P
....gaaucuuugucucaaccu...	4	0	T6P
....gGaaggugagcaaaguuucaggugugu................	11	1	T6P
....gaaGggugagcaaaguuucaggugugu................	3	1	T6P
....aGaggugagcaaaguuucaggugugu................	1	1	T6P
....Gaaggugagcaaaguuucaggugugu................	2	1	T6P
....aaGggugagcaaaguuucaggugugu................	1	1	T6P
....aCggugagcaaaguuucaggugugu................	1	1	T6P
....aGggugagcaaaguuucaggugugu................	3	1	T6P
....Gaggugagcaaaguuucaggugugu................	1	1	T6P
....Gggugagcaaaguuucaggugugu................	1	1	T6P
....Uggugagcaaaguuucaggugugu................	2	1	T6P
....ggugagcaaaguuucaggu....................	1	0	T6P
....ggugagcaaaguuucaggug...................	1	0	T6P
....ggugagcaaaguuucaggugu..................	49	0	T6P
....ggugagcaaaguuucaggugug.................	93	0	T6P
....ggugagcaaaguuucagguguU.................	5	1	T6P
....ggCgagcaaaguuucaggugug.................	1	1	T6P
....ggugaAcaaaguuucaggugug.................	1	1	T6P
....ggugagGaaaguuucaggugugu................	2	1	T6P
....ggugagcaaaguuucaggugugu................	2162	0	T6P
....ggugagcaaaguuucaggugGgu................	1	1	T6P
....Cgugagcaaaguuucaggugugu................	2	1	T6P
....ggugagcaaaguuucUggugugu................	1	1	T6P
....ggugagcaaaguuucagCugugu................	2	1	T6P
....ggugagcaaaguuucagguguUu................	2	1	T6P
....ggugagcaaaAuuucaggugugu................	1	1	T6P
....ggugagcaaaguuucaggugCgu................	1	1	T6P
....gUugagcaaaguuucaggugugu................	6	1	T6P
....ggugagcaaaguuucaggugugG................	1	1	T6P
....ggCgagcaaaguuucaggugugu................	1	1	T6P
....gguUagcaaaguuucaggugugu................	1	1	T6P
....ggugagcaaaguuucaCgugugu................	1	1	T6P
....gAugagcaaaguuucaggugugu................	1	1	T6P
....ggugagcaaaguuucaUgugugu................	2	1	T6P
....Ugugagcaaaguuucaggugugu................	78	1	T6P
....gCugagcaaaguuucaggugugu................	2	1	T6P
....ggugagcaaaUuuucaggugugu................	1	1	T6P
....ggugagAaaaguuucaggugugu................	1	1	T6P
....ggugagcaaaguuucGggugugu................	2	1	T6P
....ggugagcaaaguuGcaggugugu................	1	1	T6P
....ggugagcaaaguuucagAugugu................	1	1	T6P
....ggugaCcaaaguuucaggugugu................	2	1	T6P
....ggugagUaaaguuucaggugugu................	1	1	T6P
....ggugagcaaaguuucaggugugA................	49	1	T6P
....ggugagcaaaguuucagguguguu...............	30	0	T6P
....ggugagcaaaguuucagguguguA...............	8	1	T6P
....Ugugagcaaaguuucagguguguu...............	2	1	T6P
....ggugagcaaaguuucaggugugAu...............	2	1	T6P
....ggugagcaaaguuucagguguguuu..............	3	0	T6P
....gugagcaaaguuucaUgu....................	1	1	T6P
....gugagcaaaguuucaggu....................	172	0	T6P
....gugagcaaaguuuUaggug...................	1	1	T6P
....gugagcaaaguuucaggug...................	151	0	T6P
....gugagcaaaguuucUggug...................	1	1	T6P
....gugagcaaaguuucagguC...................	3	1	T6P
....gugagcaaaguuucagguA...................	1	1	T6P
....gugagcaaaguuucUggugu..................	1	1	T6P
....gugagcaaaguuucaggugC..................	2	1	T6P
....gAgagcaaaguuucaggugu..................	1	1	T6P
....gugUgcaaaguuucaggugu..................	1	1	T6P
....gugagcCaaguuucaggugu..................	1	1	T6P
....gugagcaaaguuucGggugu..................	1	1	T6P
....gugagcaaaguuucaggugu..................	1326	0	T6P
....gugagcaaaguuucagguAu..................	1	1	T6P
....gugagcaaaguuucaggugA..................	1	1	T6P
....gugagcaaaguuucaggGgu..................	5	1	T6P
....guAagcaaaguuucaggugu..................	1	1	T6P
....gugagcaaaguuucaAgugu..................	1	1	T6P

Star   Mature

```
uaucuccaucauuuucaugcgaaacuuucuggcaggccugaaucuuugucucaaccuucuguaaugaaaggugagcaaaguuucaggugugu uugggggacuucuuuu
......................................................................gugagcaaaguuucaUgugu..................  2       1   T6P
......................................................................gugagcaaaguCucaggugu..................  2       1   T6P
......................................................................gugagUaaaguuucaggugu..................  1       1   T6P
......................................................................gugagUaaaguuucaggugug.................  3       1   T6P
......................................................................gugCgcaaaguuucaggugug.................  1       1   T6P
......................................................................gugagcaaaguGucaggugug.................  1       1   T6P
......................................................................guAagcaaaguuucaggugug.................  1       1   T6P
......................................................................gugagcaaaguuucagguguA.................  5       1   T6P
......................................................................Uugagcaaaguuucaggugug.................  2       1   T6P
......................................................................gugagcaaaguuuAaggugug.................  3       1   T6P
......................................................................gugagcaaaguuucaggugAg.................  1       1   T6P
......................................................................gugagcaaaguuCcaggugug.................  2       1   T6P
......................................................................guUagcaaaguuucaggugug.................  2       1   T6P
......................................................................gugagcaaaguuucagguguC.................  2       1   T6P
......................................................................gugaAcaaaguuucaggugug.................  1       1   T6P
......................................................................gugaUcaaaguuucaggugug.................  1       1   T6P
......................................................................gugagcaaaguuucagguCug.................  1       1   T6P
......................................................................gugagcaaaguuucaUgugug.................  4       1   T6P
......................................................................gugaCcaaaguuucaggugug.................  1       1   T6P
......................................................................gugagcaaaguuucagguguU.................  121     1   T6P
......................................................................gugagcGaaguuucaggugug.................  4       1   T6P
......................................................................gugagcaaaguuucaAgugug.................  1       1   T6P
......................................................................gugagcaaaguuuUaggugug.................  3       1   T6P
......................................................................Cugagcaaaguuucaggugug.................  2       1   T6P
......................................................................gugagcaaaguuucaggugCg.................  12      1   T6P
......................................................................gugagcaaaguuucGggugug.................  6       1   T6P
......................................................................gugagcaaaguuucaggCgug.................  2       1   T6P
......................................................................gugagcaaagGuucaggugug.................  1       1   T6P
......................................................................gugagcaaaUuuucaggugug.................  2       1   T6P
......................................................................gugagcaUaguuucaggugug.................  1       1   T6P
......................................................................gugagcaaaguuucagUugug.................  2       1   T6P
......................................................................gugagcaaaguuuGaggugug.................  2       1   T6P
......................................................................gugagcaaaguuucaCgugug.................  1       1   T6P
......................................................................gGgagcaaaguuucaggugug.................  1       1   T6P
......................................................................gugagcaaaguuucaggugug.................  5625    0   T6P
......................................................................gugUgcaaaguuucaggugug.................  7       1   T6P
......................................................................gugagcaaaAuuucaggugug.................  4       1   T6P
......................................................................gugagcaaaguuucaUgugugu................  160     1   T6P
......................................................................gugaAcaaaguuucaggugugu................  65      1   T6P
......................................................................gugagcaaaguAucaggugugu................  12      1   T6P
......................................................................gugagcaaaguuucagguUugu................  72      1   T6P
......................................................................gugagcGaaguuucaggugugu................  306     1   T6P
......................................................................gugagcaaaUuuucaggugugu................  67      1   T6P
......................................................................gugagcaaaCuuucaggugugu................  55      1   T6P
......................................................................gugagcaaCguuucaggugugu................  1       1   T6P
......................................................................gugagcaaaguuucagguCugu................  57      1   T6P
......................................................................gugagcaaaguGucaggugugu................  6       1   T6P
......................................................................gugagAaaaguuucaggugugu................  6       1   T6P
......................................................................gCgagcaaaguuucaggugugu................  23      1   T6P
......................................................................gugagcaaaguuucaggugAgu................  37      1   T6P
......................................................................gugagcaaUguuucaggugugu................  9       1   T6P
......................................................................gugagcaaaguuucaggAgugu................  16      1   T6P
......................................................................gugGgcaaaguuucaggugugu................  49      1   T6P
......................................................................gugagcaaaguuGcaggugugu................  5       1   T6P
......................................................................gugagcaaaguCucaggugugu................  30      1   T6P
......................................................................gugagcaaaguuucaggugugu................  339506  0   T6P
......................................................................gugagcaaGguuucaggugugu................  16      1   T6P
......................................................................gugagcaaagAuucaggugugu................  6       1   T6P
......................................................................guUagcaaaguuucaggugugu................  94      1   T6P
......................................................................gugagcaaaguuucagguAugu................  29      1   T6P
......................................................................Uugagcaaaguuucaggugugu................  99      1   T6P
......................................................................gGgagcaaaguuucaggugugu................  27      1   T6P
......................................................................gugagcaaaguuCcaggugugu................  52      1   T6P
......................................................................gugaUcaaaguuucaggugugu................  60      1   T6P
......................................................................gugagGaaaguuucaggugugu................  6       1   T6P
......................................................................guCagcaaaguuucaggugugu................  127     1   T6P
......................................................................gugagcaaaguuucaggCgugu................  29      1   T6P
......................................................................gugagcaaaguuucaAgugugu................  54      1   T6P
......................................................................gugCgcaaaguuucaggugugu................  29      1   T6P
......................................................................gugagcaaaguuucagUugugu................  152     1   T6P
```

Star   Mature

```
uaucuccaucauuuucaugcgaaacuuucuggcaggccugaaucuuugucucaaccuucuguaaugaaaggugagcaaaguuucagguguguuugggggacuucuuuu
......................................................................gugagUaaaguuucaggugugu................   138    1   T6P
......................................................................gugagcaaaguuucaggGgugu................   41     1   T6P
......................................................................gugagcaUaguuucaggugugu................   4      1   T6P
......................................................................gugagcaaaguuucGggugugu................   207    1   T6P
......................................................................gugagcaaaguuucagguguUu................   73     1   T6P
......................................................................guAagcaaaguuucaggugugu................   27     1   T6P
......................................................................gugagcaGaguuucaggugugu................   18     1   T6P
......................................................................gugagcaaaguuuAaggugugu................   28     1   T6P
......................................................................Cugagcaaaguuucaggugugu................   23     1   T6P
......................................................................gugagcaaaguuucagCugugu................   122    1   T6P
......................................................................gugagcaaaguuucaggugugG................   56     1   T6P
......................................................................gugagcaaagCuucaggugugu................   17     1   T6P
......................................................................gugUgcaaaguuucaggugugu................   525    1   T6P
......................................................................gugagcaaaguuuGaggugugu................   89     1   T6P
......................................................................gugagcaaaguuucaggugCgu................   37     1   T6P
......................................................................gugagcaaaguuucaggugugC................   448    1   T6P
......................................................................gugagcaaaguuAcaggugugu................   2      1   T6P
......................................................................gugagcCaaguuucaggugugu................   69     1   T6P
......................................................................gugagcaaaguuucagguguCu................   81     1   T6P
......................................................................gAgagcaaaguuucaggugugu................   17     1   T6P
......................................................................gugagcaaagGuucaggugugu................   37     1   T6P
......................................................................gugagcaaaguuucaCgugugu................   138    1   T6P
......................................................................gugagcaaaguuucaggugGgu................   27     1   T6P
......................................................................gugagcaaaguuuUaggugugu................   281    1   T6P
......................................................................gugagcUaaguuucaggugugu................   48     1   T6P
......................................................................gugagcaaaguuucCggugugu................   31     1   T6P
......................................................................gugagcaaaguuucagAugugu................   101    1   T6P
......................................................................gugagcaaaguuucUggugugu................   78     1   T6P
......................................................................gugagcaaaAuuucaggugugu................   88     1   T6P
......................................................................gugagcaaaguuucaggugugA................   1985   1   T6P
......................................................................gugagcaaaguuucagguguAu................   70     1   T6P
......................................................................gugaCcaaaguuucaggugugu................   46     1   T6P
......................................................................gugaAcaaaguuucagguguguu...............   3      1   T6P
......................................................................gugagcaaaguuucGgguguguu...............   6      1   T6P
......................................................................gugagcaaaguuucagguguCuu...............   2      1   T6P
......................................................................gugagcaaaguuucaUguguguu...............   4      1   T6P
......................................................................gugagcaaaguuucaCguguguu...............   3      1   T6P
......................................................................gugagcGaaguuucagguguguu...............   4      1   T6P
......................................................................gugagcaaaguuucagUuguguu...............   1      1   T6P
......................................................................gugagcaaaguuucaggugCguu...............   1      1   T6P
......................................................................Augagcaaaguuucagguguguu...............   2      1   T6P
......................................................................gugagcaaaguuucagguguguu...............   6032   0   T6P
......................................................................gugagcaaagGuucagguguguu...............   1      1   T6P
......................................................................gugagcaaaguuCcagguguguu...............   1      1   T6P
......................................................................gugagcaaaguuucagguUuguu...............   1      1   T6P
......................................................................gugagUaaaguuucagguguguu...............   1      1   T6P
......................................................................gugagcaaaguuucaggugGguu...............   2      1   T6P
......................................................................gugagcaaaAuuucagguguguu...............   1      1   T6P
......................................................................Uugagcaaaguuucagguguguu...............   1      1   T6P
......................................................................guUagcaaaguuucagguguguu...............   2      1   T6P
......................................................................gugagcaaaguuucaggugugAu...............   10     1   T6P
......................................................................gGgagcaaaguuucagguguguu...............   1      1   T6P
......................................................................gugagcaaaguuucaggAguguu...............   1      1   T6P
......................................................................gugUgcaaaguuucagguguguu...............   14     1   T6P
......................................................................gAgagcaaaguuucagguguguu...............   2      1   T6P
......................................................................gugagcaaaguuucaggCguguu...............   2      1   T6P
......................................................................gugagcaaaguuucaggugugCu...............   2      1   T6P
......................................................................gugagcaaaguuucaAguguguu...............   3      1   T6P
......................................................................gugagcaaaguuucagguguguA...............   991    1   T6P
......................................................................gugaUcaaaguuucagguguguu...............   2      1   T6P
......................................................................gugagcaaaguuucagCuguguu...............   5      1   T6P
......................................................................gugagcaaaguuucagguCuguu...............   2      1   T6P
......................................................................gugagcaaaguuuUagguguguu...............   6      1   T6P
......................................................................gugagcaaaguuucagguguUuu...............   5      1   T6P
......................................................................guCagcaaaguuucagguguguu...............   2      1   T6P
......................................................................gugagcaaaguuucagAuguguu...............   1      1   T6P
......................................................................gugagcaaaguuucagguguguG...............   36     1   T6P
......................................................................gugagcaaaguuucagguguguCu..............   1      1   T6P
......................................................................gugagcaaaCuuucagguguguuu..............   1      1   T6P
......................................................................gugagcaaaguuucagguguguuC..............   4      1   T6P
```

Star **Mature**

```
uaucuccaucauuuucaugcgaaacuuucuggcaggccugaaucuuugucucaaccuucuguaaugaaaggugagcaaaguuucaggugugu uuggggacuucuuuu
......................................................................gugagcaaaguuucaggugugu uG.............   16    1    T6P
......................................................................gugagcaaaguuucaggugugu Gu.............   2     1    T6P
......................................................................gugagcaaaguuucagguguUuuu.............   1     1    T6P
......................................................................gugagcaaaguuucagguguguAu.............   111   1    T6P
......................................................................gugagcaaaguuucagguguguuA.............   119   1    T6P
......................................................................gugagcaaaguuucagguguguuu.............   178   0    T6P
......................................................................gugagcaaaguuucagguguguAug............   1     1    T6P
......................................................................gugagcaaaguuucagguguguuuU............   14    1    T6P
......................................................................gugagcaaaguuucagguguguuuA............   2     1    T6P
......................................................................gugagcaaaguuucagguguguuGgg...........   1     1    T6P
.......................................................................ugagcaaaguuucaggugug................   1     0    T6P
.......................................................................uUagcaaaguuucaggugugu...............   2     1    T6P
.......................................................................Ggagcaaaguuucaggugugu...............   4     1    T6P
.......................................................................ugagcaaaguuucaggugugu...............   47    0    T6P
.......................................................................uCagcaaaguuucaggugugu...............   3     1    T6P
.......................................................................ugagcaaaguuucaggugugA...............   1     1    T6P
.......................................................................ugagcaaaguuucagguguguu..............   2     0    T6P
........................................................................gagcaaaguuucaggugu.................   1     0    T6P
........................................................................gagcaaaguuucaggugug................   3     0    T6P
........................................................................gagcaaaguuucaggugugu...............   143   0    T6P
........................................................................gagcaaaguuucaggGgugu...............   1     1    T6P
........................................................................Cagcaaaguuucaggugugu...............   21    1    T6P
........................................................................gUgcaaaguuucaggugugu...............   3     1    T6P
........................................................................Uagcaaaguuucaggugugu...............   4     1    T6P
........................................................................gagcaaaguuucagguguguu..............   5     0    T6P
........................................................................gagcaaaguuucagguguguAu.............   1     1    T6P
.........................................................................agcaaaguuucaggugugu...............   28    0    T6P
.........................................................................agcaaaguuucaggugugC...............   1     1    T6P
.........................................................................agcaaaguuucagguguguGug............   2     1    T6P
..........................................................................gcaaaguuucaggugugu...............   21    0    T6P
..........................................................................gcaaaguuucagguguguu..............   1     0    T6P
```

Provisional ID	:	Scaffold_1043_1003
Score total	:	567073.3
Score for star read(s)	:	3.9
Score for read counts	:	567063.3
Score for mfe	:	1.5
Score for randfold	:	1.6
Score for cons. seed	:	3
Total read count	:	1112282
Mature read count	:	1109528
Loop read count	:	1
Star read count	:	2753

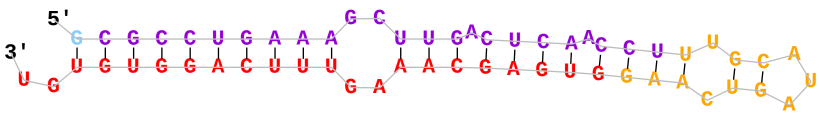

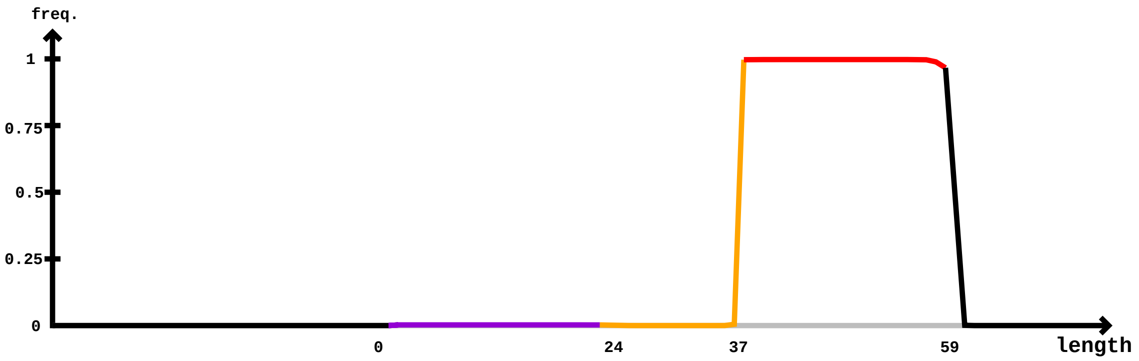


```
                                   Star                              Mature
5'- uuguaggucugugcauuuauacagcucuacuuggcgccugaaagcuugacucaaccuuugcauagucaaggugagcaaaguuucaggugugucagugggguuaaaaug   -3'    obs
    uuguaggucugugcauuuauacagcucuacuuggcgccugaaagcuugacucaaccuuugcauagucaaggugagcaaaguuucaggugugucagugggguuaaaaug          exp
    .(((((((......))))))).(((((((((..((((((((((..(((.((((.((((.((...)).)))))))))))..))))))))))...)))))))))......    reads     mm      sample
    ..................................cgccugaaagcuugacuca.......................................................    4         0       T63
    ..................................cgccugaaagcuugacucaa......................................................    1         0       T63
    ..................................cgccugaaagcuugacucaac.....................................................    1         0       T63
    ..................................cgccugaaagcuugaUucaacc....................................................    1         1       T63
    ..................................cgccugaaagcuugacucaacc....................................................    1         0       T63
    ..................................cgUcugaaagcuugacucaaccu...................................................    1         1       T63
    ..................................cgccugaaagcuugacucaaccu...................................................    44        0       T63
    ..................................cgccugaaagcuAgacucaaccu...................................................    1         1       T63
    ..................................cgccugaaagcuugUcucaaccu...................................................    1         1       T63
    ..................................cgccugaaagcuugacucaaccuu..................................................    3         0       T63
    ..................................cgccugaaagcuugacucaaccuuug................................................    1         0       T63
    ..................................................................Gaaggugagcaaaguuucaggugugu................    1         1       T63
    ....................................................................Gggugagcaaaguuucaggugugu................    1         1       T63
    .....................................................................Ugugagcaaaguuucaggu....................    1         1       T63
    .....................................................................ggugagcaaaguuucaggugu..................    14        0       T63
    .....................................................................ggugagcaaaguuucaggugugU.................    10        1       T63
    .....................................................................ggugagcaaaguuucaggugug.................    25        0       T63
    .....................................................................Cgugagcaaaguuucaggugugu................    1         1       T63
    .....................................................................ggugagcaaaguuucaggugugu................    186       0       T63
    .....................................................................ggugagcaaaguuucagguguUu................    6         1       T63
    .....................................................................Ugugagcaaaguuucaggugugu................    41        1       T63
    .....................................................................ggugagcaaaguuucGggugugu................    1         1       T63
    .....................................................................Agugagcaaaguuucaggugugu................    1         1       T63
    .....................................................................ggugagcaaaguuAcaggugugu................    1         1       T63
    .....................................................................ggCgagcaaaguuucaggugugu................    1         1       T63
    .....................................................................ggugagcaaaguuucaggugugA................    8         1       T63
    .....................................................................ggugagcaaaguuucagguguguAa..............    1         1       T63
    ......................................................................gugagcaaaguuucaggA....................    1         1       T63
    ......................................................................gugagcaaaguuucaggu....................    57        0       T63
    ......................................................................gugagcaaaguuucagguA...................    1         1       T63
    ......................................................................gugagcaaaguuucaggug...................    66        0       T63
    ......................................................................gugagcaaaguuucagguU...................    1         1       T63
    ......................................................................gugagcaaaguuucaggugA..................    3         1       T63
```

Star **Mature**

```
uuguaggucugugcauuuauacagcucuacuuggcgccugaaagcuugacucaaccuuugcauagucaaggugagcaaaguuucaggugugucagugggguuaaaaug
......................................................................gugaUcaaaguuucaggugu..................  2     1  T63
......................................................................gugagcaaaguuucaAgugu..................  1     1  T63
......................................................................gugagcaaaguuucaggugC..................  1     1  T63
......................................................................gugagcaaaguuucaggGgu..................  1     1  T63
......................................................................gugaAcaaaguuucaggugu..................  1     1  T63
......................................................................gugagcaaaguCucaggugu..................  1     1  T63
......................................................................gugagcaaagCuucaggugu..................  1     1  T63
......................................................................gugagcaaaguuucagUugu..................  1     1  T63
......................................................................gugUgcaaaguuucaggugu..................  1     1  T63
......................................................................gugagcaaaCuuucaggugu..................  1     1  T63
......................................................................gugagcaaaguuucaggugu..................  921   0  T63
......................................................................gugagcaaagAuucaggugug.................  1     1  T63
......................................................................gugUgcaaaguuucaggugug.................  1     1  T63
......................................................................gugagcaaUguuucaggugug.................  5     1  T63
......................................................................gugagcaaaguuucagCugug.................  1     1  T63
......................................................................gugagcaaaguuucaggGgug.................  3     1  T63
......................................................................gugagcaaaguuucagAugug.................  1     1  T63
......................................................................gugagcaaaguuucagguguU.................  286   1  T63
......................................................................gugagGaaaguuucaggugug.................  1     1  T63
......................................................................gugagcaaaguuucaggugAg.................  1     1  T63
......................................................................gugagcUaaguuucaggugug.................  1     1  T63
......................................................................gugagcaaaguuucaggugug.................  2084  0  T63
......................................................................gugagcaaaguuucagguguC.................  3     1  T63
......................................................................gugagcaaaguuucaggugGg.................  1     1  T63
......................................................................gugagcaaaguuucaggAgug.................  2     1  T63
......................................................................gugagcaaaguuucaCgugug.................  1     1  T63
......................................................................gugaAcaaaguuucaggugug.................  2     1  T63
......................................................................gugagcaaaguuucagguguA.................  2     1  T63
......................................................................gugagcaaaguuucagUugug.................  2     1  T63
......................................................................gugagcaaaguuucUggugug.................  2     1  T63
......................................................................guCagcaaaguuucaggugug.................  1     1  T63
......................................................................guUagcaaaguuucaggugug.................  1     1  T63
......................................................................gugagcaaaguuuUaggugug.................  2     1  T63
......................................................................gugagcaaaguuuUaggugugu................  40    1  T63
......................................................................gugagcaaaguCucaggugugu................  7     1  T63
......................................................................gugaUcaaaguuucaggugugu................  18    1  T63
......................................................................gugagcaaaguuucUggugugu................  99    1  T63
......................................................................gugagGaaaguuucaggugugu................  26    1  T63
......................................................................gugagcaaaguuucGggugugu................  68    1  T63
......................................................................gugagcaaaguuucagAugugu................  22    1  T63
......................................................................gugagcaaaguuucaggCgugu................  6     1  T63
......................................................................gugagcaaaUuuucaggugugu................  3     1  T63
......................................................................gugagcGaaguuucaggugugu................  5     1  T63
......................................................................gugagcaaaguuucagguguAu................  12    1  T63
......................................................................gugagcaaagGuucaggugugu................  7     1  T63
......................................................................gugagcaaaguuuAaggugugu................  15    1  T63
......................................................................gugUgcaaaguuucaggugugu................  36    1  T63
......................................................................gugagcaaaguuCcaggugugu................  9     1  T63
......................................................................gugagAaaaguuucaggugugu................  12    1  T63
......................................................................gugagcaaaguuucaggugAgu................  39    1  T63
......................................................................gugagcaaaguuucagCugugu................  16    1  T63
......................................................................gugagcaaaguuucaggGgugu................  19    1  T63
......................................................................gugCgcaaaguuucaggugugu................  5     1  T63
......................................................................gugagcaaaguuucaggAgugu................  34    1  T63
......................................................................gugaAcaaaguuucaggugugu................  27    1  T63
......................................................................gugagcaaaguuucaCgugugu................  18    1  T63
......................................................................gugaCcaaaguuucaggugugu................  11    1  T63
......................................................................gugagcaaCguuucaggugugu................  6     1  T63
......................................................................gugagcaaaguuucagguAugu................  22    1  T63
......................................................................gugagcaaGguuucaggugugu................  13    1  T63
......................................................................gugagcaaaguuucagguguUu................  56    1  T63
......................................................................gugagcaaaguAucaggugugu................  14    1  T63
......................................................................gCgagcaaaguuucaggugugu................  11    1  T63
......................................................................gugagcaUaguuucaggugugu................  8     1  T63
......................................................................gugagcaGaguuucaggugugu................  4     1  T63
......................................................................gugagcaaaguuucaUgugugu................  33    1  T63
......................................................................guAagcaaaguuucaggugugu................  8     1  T63
......................................................................gugagcaaaguuuGaggugugu................  34    1  T63
......................................................................Cugagcaaaguuucaggugugu................  10    1  T63
......................................................................gugagcaaaguuucaggugugC................  77    1  T63
```

```
                                  Star                                   Mature
uuguaggucugugcauuuauacagcucuacuuggcgccugaaagcuugacucaaccuuugcauagucaaggugagcaaaguuucaggugugucagugggguuaaaaug
......................................................................gugagcaaagCuucaggugugu................     6       1    T63
......................................................................gugagcaaaguuucaAgugugu................     27      1    T63
......................................................................gugagcaaaguGucaggugugu................     2       1    T63
......................................................................gugagcaaUguuucaggugugu................     79      1    T63
......................................................................gugagcaaaCuuucaggugugu................     41      1    T63
......................................................................gugagcaaaguuucagUugugu................     21      1    T63
......................................................................gugagcaaaguuucCggugugu................     10      1    T63
......................................................................gugagcaaaguuAcaggugugu................     34      1    T63
......................................................................gugagcaaaAuuucaggugugu................     10      1    T63
......................................................................gugagcaaaguuGcaggugugu................     2       1    T63
......................................................................gugagcaaaguuucagguguCu................     8       1    T63
......................................................................gugagcaaagAuucaggugugu................     8       1    T63
......................................................................gugagcaaaguuucaggugugA................     1441    1    T63
......................................................................guUagcaaaguuucaggugugu................     10      1    T63
......................................................................gugagcaaaguuucaggugCgu................     10      1    T63
......................................................................gGgagcaaaguuucaggugugu................     16      1    T63
......................................................................gugagcaaaguuucaggugugu................     110426  0    T63
......................................................................gugagcaaaguuucaggugugG................     83      1    T63
......................................................................gugagcaaaguuucagguCugu................     15      1    T63
......................................................................gugGgcaaaguuucaggugugu................     9       1    T63
......................................................................guCagcaaaguuucaggugugu................     5       1    T63
......................................................................gAgagcaaaguuucaggugugu................     7       1    T63
......................................................................gugagcUaaguuucaggugugu................     5       1    T63
......................................................................gugagUaaaguuucaggugugu................     69      1    T63
......................................................................gugagcaaaguuucagguUugu................     22      1    T63
......................................................................Uugagcaaaguuucaggugugu................     14      1    T63
......................................................................gugagcaaaguuucaggugGgu................     16      1    T63
......................................................................gugagcaaaguuucagguguguc...............     58      0    T63
......................................................................gugagcaaaguuucagguguguA...............     335     1    T63
......................................................................gugagcaaaguuucagguguguG...............     25      1    T63
......................................................................gugagcaaaguuucagguguguAa..............     40      1    T63
......................................................................gugagcaaaguuucagguguguUa..............     37      1    T63
......................................................................gugagcaaaguuucaggugugucG..............     4       1    T63
......................................................................gugagcaaaguuucagguguguGa..............     2       1    T63
......................................................................gugagcaaaguuucagguguguca..............     1       0    T63
.......................................................................ugagcaaaguuucaggugugu................     9       0    T63
.......................................................................ugagcaaaguuucaggugugA................     1       1    T63
........................................................................gagcaaaguuucaggugu..................     2       0    T63
........................................................................gagcaaaguuucaggugug.................     1       0    T63
........................................................................gagcaaaguuucaggugugu................     22      0    T63
........................................................................gagcaaaguuucaggugugA................     2       1    T63
........................................................................gagcaaaguuucagguAugu................     1       1    T63
.........................................................................agcaaaguuucaggugugu................     9       0    T63
..........................................................................gcaaaguuucaggugugu................     12      0    T63

...............................uggcgccugaaagcuugacucaaccu...................................................     1       0    MOL
...............................Gggcgccugaaagcuugacucaaccu...................................................     1       1    MOL
.................................Ucgccugaaagcuugacucaaccu...................................................     1       1    MOL
.................................gcgccugaaagcuugacucaaccu...................................................     1       0    MOL
..................................cgccugaaagcuugacuca.......................................................     3       0    MOL
..................................cgccugaaagcuugacucaa......................................................     2       0    MOL
..................................cgccugaaagcuugacucaaU.....................................................     1       1    MOL
..................................cCccugaaagcuugacucaac.....................................................     1       1    MOL
..................................cgccugaaagcuugacucaac.....................................................     18      0    MOL
..................................cgccugaaagcuugacucaacc....................................................     59      0    MOL
..................................cgccugaaagcuugacucaacA....................................................     4       1    MOL
..................................cgccugUaagcuugacucaacc....................................................     1       1    MOL
..................................cgccuAaaagcuugacucaaccu...................................................     1       1    MOL
..................................cgccugUaagcuugacucaaccu...................................................     2       1    MOL
..................................cgccugaaagcuugacucaaccC...................................................     1       1    MOL
..................................cgccugaaagcuugacucaaccA...................................................     2       1    MOL
..................................Ugccugaaagcuugacucaaccu...................................................     1       1    MOL
..................................cgccugGaagcuugacucaaccu...................................................     1       1    MOL
..................................cgccugaaagcuugUcucaaccu...................................................     2       1    MOL
..................................cgccugaaUgcuugacucaaccu...................................................     1       1    MOL
..................................cgccugCaagcuugacucaaccu...................................................     1       1    MOL
..................................cgccugaaagcuugacuAaaccu...................................................     1       1    MOL
..................................cgUcugaaagcuugacucaaccu...................................................     1       1    MOL
..................................cgccugaaagcuCgacucaaccu...................................................     1       1    MOL
..................................cgccugaaagcuugacucaaccu...................................................     1050    0    MOL
```

Star **Mature**

uuguaggucugugcauuuauacagcucuacuuggcgccugaaagcuugacucaaccuuugcauagucaaggugagcaaaguuucaggugugucagugggguuaaaaug			
..................................cgcUugaaagcuugacucaaccu...	1	1	MOL
..................................cgccAgaaagcuugacucaaccu...	1	1	MOL
..................................cgccugaaagcuugacucaaccuu..	92	0	MOL
..................................cgccugaaagcuugacucaaccuA..	1	1	MOL
..................................cgccugaaagcuugacucaaccuuu...	18	0	MOL
..................................cgccugaaagcuugacucaaccuuC...	1	1	MOL
..................................cgccugaaagcuugacucaaccuuug..	3	0	MOL
..................................cgccugaaagcuugacucaaccuuuU..	1	1	MOL
..................................cgccugaaagcuugacucaaccuuugc...	5	0	MOL
...................................gccugaaagcuugacucaacc..	1	0	MOL
...................................gccugaaagcuugacucaaccu...	3	0	MOL
...................................gccugaaagcuugacucaaAcu...	1	1	MOL
...................................gccugaaagcuugacucaaccuu..	1	0	MOL
......................................ugaaagcuugacucaaccu...	1	0	MOL
....uagucaaggugagcaaaguuuc........................	1	0	MOL
....caaggugagcaaaguuucaggugugu................	1	0	MOL
....Gaaggugagcaaaguuucaggugugu................	3	1	MOL
....caGgugagcaaaguuucaggugugu................	1	1	MOL
....Gaggugagcaaaguuucaggugugu................	7	1	MOL
....aGggugagcaaaguuucaggugugu................	9	1	MOL
....aaCgugagcaaaguuucaggugugu................	1	1	MOL
....Gggugagcaaaguuucaggugugu................	3	1	MOL
....Uggugagcaaaguuucaggugugu................	1	1	MOL
....ggugagcaaaguuucaggu....................	2	0	MOL
....ggugagcaaaguuucaggug...................	1	0	MOL
....ggugagcaaaguuucagguA...................	1	1	MOL
....Ugugagcaaaguuucaggugu..................	1	1	MOL
....ggugagcaaaguuAcaggugu..................	1	1	MOL
....ggugagcaaaguuucaggugC..................	1	1	MOL
....ggugaAcaaaguuucaggugu..................	1	1	MOL
....ggugagcaaaguuucaggugu..................	62	0	MOL
....ggugagUaaaguuucaggugu..................	1	1	MOL
....ggugagcaaaguuucaggugug.................	74	0	MOL
....ggugagcaaaguuucagguguU.................	14	1	MOL
....ggugagcaaaguuucagguguA.................	1	1	MOL
....Ugugagcaaaguuucaggugug.................	6	1	MOL
....ggugagcaaaguuucaggAgug.................	1	1	MOL
....ggugagcaaaguuuGaggugugu................	1	1	MOL
....ggugUgcaaaguuucaggugugu................	2	1	MOL
....ggugagUaaaguuucaggugugu................	1	1	MOL
....ggugagcaaaguuucUggugugu................	1	1	MOL
....Agugagcaaaguuucaggugugu................	4	1	MOL
....ggugagcaaaguuucagAugugu................	1	1	MOL
....ggugagcaaaguAucaggugugu................	1	1	MOL
....ggugagcaaaguuucaggugugG................	1	1	MOL
....ggugagcaaaguuucagUugugu................	1	1	MOL
....ggugagcaaaguuucaggugugC................	4	1	MOL
....ggugagcaaaguuucagguguUu................	4	1	MOL
....Ugugagcaaaguuucaggugugu................	111	1	MOL
....ggugagcaaaguuucagguUugu................	1	1	MOL
....ggugagcaUaguuucaggugugu................	1	1	MOL
....ggugCgcaaaguuucaggugugu................	2	1	MOL
....ggugagcaaaguuucaggugugA................	54	1	MOL
....ggugagcaaaguuucaUgugugu................	1	1	MOL
....ggugagcaaaguuucaggugugu................	1199	0	MOL
....ggugagcaaaguuucagguguguA...............	4	1	MOL
....ggugagcaaaguuucagguguguG...............	1	1	MOL
....ggugagcaaaguuucagguguguAa..............	1	1	MOL
....gugagcaaaguuucaggu....................	87	0	MOL
....gugagcaaaguuucaggC....................	2	1	MOL
....gugagcaaaguuucaggG....................	1	1	MOL
....gugagcaaaguuucagguC...................	3	1	MOL
....gugagcaaaguAucaggug...................	1	1	MOL
....gugagcaaaguuucaggug...................	215	0	MOL
....gugagcaaaguuucagguU...................	1	1	MOL
....gugagcaaaguuucUggug...................	1	1	MOL
....gugagcaaaguuucGggug...................	1	1	MOL
....gugagcaaaguAucaggugu..................	1	1	MOL
....Cugagcaaaguuucaggugu..................	1	1	MOL
....gugaCcaaaguuucaggugu..................	1	1	MOL

Star Mature

uuguaggucugugcauuuauacagcucuacuuggcgccugaaagcuugacucaaccuuugcauagucaaggugagcaaaguuucaggugugucaguggggguuaaaaug			
....gugagcaaaguuucaggugC...................	4	1	MOL
....gugGgcaaaguuucaggugu...................	1	1	MOL
....gugagcaaaguuucagUugu...................	1	1	MOL
....gugagcaaUguuucaggugu...................	3	1	MOL
....gugagcaaaguuAcaggugu...................	5	1	MOL
....gGgagcaaaguuucaggugu...................	1	1	MOL
....gAgagcaaaguuucaggugu...................	1	1	MOL
....gugagcaaaguuucagguUu...................	3	1	MOL
....gugagcaaaguuucaggugu...................	3431	0	MOL
....gugagcaaaUuuucaggugu...................	1	1	MOL
....gugagcaaaguuucaAgugu...................	1	1	MOL
....gugagcaaaguuucGggugu...................	1	1	MOL
....gugagcaaagGuucaggugu...................	2	1	MOL
....guUagcaaaguuucaggugu...................	1	1	MOL
....gugagcaaaguuucagAugu...................	1	1	MOL
....gugagcaaaguuucaggAgu...................	1	1	MOL
....gugagUaaaguuucaggugu...................	1	1	MOL
....gugaAcaaaguuucaggugu...................	1	1	MOL
....gugUgcaaaguuucaggugu...................	1	1	MOL
....gugagcaaaguuucaggGgu...................	3	1	MOL
....gCgagcaaaguuucaggugu...................	2	1	MOL
....gugagcaaaguuucaggugA...................	6	1	MOL
....gugagcaaaguuuUaggugu...................	3	1	MOL
....gugagGaaaguuucaggugu...................	3	1	MOL
....gugagcaaaguuucaUgugu...................	3	1	MOL
....gugagcaaaCuuucaggugu...................	1	1	MOL
....gugagcaaaguuucUggugu...................	4	1	MOL
....gugagcaaaCuuucaggugug..................	7	1	MOL
....gugagcaaGguuucaggugug..................	3	1	MOL
....gugagcaaagGuucaggugug..................	3	1	MOL
....gugagcaUaguuucaggugug..................	3	1	MOL
....gugagcaaCguuucaggugug..................	4	1	MOL
....gugagcaaaguuucagguguU..................	299	1	MOL
....gugagcaaaguuucagguguC..................	10	1	MOL
....gugagcaaaguuucaAgugug..................	3	1	MOL
....gugagcaaaguuuUaggugug..................	2	1	MOL
....gugCgcaaaguuucaggugug..................	1	1	MOL
....gugagcaaaguuucUggugug..................	9	1	MOL
....gugagcaaagCuucaggugug..................	3	1	MOL
....gugagcaaaguuucaggugug..................	11001	0	MOL
....gugagcaaaguuucGggugug..................	4	1	MOL
....gugagcaaaguuucagguCug..................	3	1	MOL
....gugagcaaUguuucaggugug..................	11	1	MOL
....gugagcaaaguuucaggugAg..................	4	1	MOL
....gugagcaaaguuucaCgugug..................	1	1	MOL
....gugaAcaaaguuucaggugug..................	2	1	MOL
....gugagGaaaguuucaggugug..................	1	1	MOL
....gugagcaaaguuuGaggugug..................	3	1	MOL
....gugagUaaaguuucaggugug..................	9	1	MOL
....gugagcaaaguAucaggugug..................	1	1	MOL
....gugUgcaaaguuucaggugug..................	6	1	MOL
....gugagcaaagAuucaggugug..................	1	1	MOL
....guAagcaaaguuucaggugug..................	2	1	MOL
....gugagcaaaguuucaggAgug..................	5	1	MOL
....gugagcaaaguuucaUgugug..................	7	1	MOL
....gugagcaaaguCucaggugug..................	2	1	MOL
....Cugagcaaaguuucaggugug..................	1	1	MOL
....gugaCcaaaguuucaggugug..................	1	1	MOL
....gugaUcaaaguuucaggugug..................	2	1	MOL
....gugagcaaaguuucagguguA..................	19	1	MOL
....gugagcaaaguuucaggugCg..................	4	1	MOL
....gugagcaaaguuAcaggugug..................	6	1	MOL
....gugagcaaaguuucagguUug..................	5	1	MOL
....gugagcaaaAuuucaggugug..................	2	1	MOL
....gugagcaaaguuucagCugug..................	1	1	MOL
....guUagcaaaguuucaggugug..................	2	1	MOL
....gugagcaaaUuuucaggugug..................	2	1	MOL
....gugagcaaaguuucaggGgug..................	6	1	MOL
....gugagcGaaguuucaggugug..................	1	1	MOL
....gugagcaaaguuuAaggugug..................	1	1	MOL

```
                                                  Star                              Mature
uuguaggucugugcauuuauacagcucuacuuggcgccugaaagcuugacucaaccuuugcauagucaaggugagcaaaguuucaggugugucagugggguuaaaaug
......................................................................gugagcaaaguuucagUugug.................   4       1    MOL
......................................................................gugagcaaaguuucagAugug.................   2       1    MOL
......................................................................gugagcaaaguuucaggCgug.................   3       1    MOL
......................................................................gugagAaaaguuucaggugug.................   1       1    MOL
......................................................................gCgagcaaaguuucaggugug.................   2       1    MOL
......................................................................gugagcaaaguuucagguAug.................   7       1    MOL
......................................................................gugagcaaaCuuucaggugugu................   118     1    MOL
......................................................................gugagcaaaguuucagguguCu................   16      1    MOL
......................................................................gugagcaaaguuucaggugGgu................   44      1    MOL
......................................................................gugagcaaUguuucaggugugu................   241     1    MOL
......................................................................gugagcaaaguCucaggugugu................   15      1    MOL
......................................................................gugagcaaaguuucaggGgugu................   72      1    MOL
......................................................................gugagcUaaguuucaggugugu................   19      1    MOL
......................................................................gugagcaUaguuucaggugugu................   17      1    MOL
......................................................................Cugagcaaaguuucaggugugu................   18      1    MOL
......................................................................gugagcaaaguAucaggugugu................   49      1    MOL
......................................................................gAgagcaaaguuucaggugugu................   13      1    MOL
......................................................................gugagcaaaguuucagguAugu................   66      1    MOL
......................................................................gugGgcaaaguuucaggugugu................   20      1    MOL
......................................................................gugagcaaaguuucaggugugA................   6745    1    MOL
......................................................................guAagcaaaguuucaggugugu................   25      1    MOL
......................................................................gugagcaaaguuucUggugugu................   279     1    MOL
......................................................................gugagcaaagCuucaggugugu................   24      1    MOL
......................................................................gugUgcaaaguuucaggugugu................   97      1    MOL
......................................................................gugagcaaaguuucagguguAu................   60      1    MOL
......................................................................gugagcaaaguuucaggugAgu................   67      1    MOL
......................................................................guCagcaaaguuucaggugugu................   8       1    MOL
......................................................................Uugagcaaaguuucaggugugu................   14      1    MOL
......................................................................gCgagcaaaguuucaggugugu................   47      1    MOL
......................................................................gugagcaaaAuuucaggugugu................   19      1    MOL
......................................................................gugagUaaaguuucaggugugu................   153     1    MOL
......................................................................gugagcGaaguuucaggugugu................   16      1    MOL
......................................................................gugaUcaaaguuucaggugugu................   23      1    MOL
......................................................................gugagcCaaguuucaggugugu................   1       1    MOL
......................................................................gugagAaaaguuucaggugugu................   57      1    MOL
......................................................................gugagcaaaguuucagguCugu................   16      1    MOL
......................................................................gugagcaaaguuucagCugugu................   50      1    MOL
......................................................................guUagcaaaguuucaggugugu................   35      1    MOL
......................................................................gugagcaaaguuucagguguUu................   100     1    MOL
......................................................................gugagcaaagAuucaggugugu................   19      1    MOL
......................................................................gugagcaaaguuucagUugugu................   123     1    MOL
......................................................................gugaAcaaaguuucaggugugu................   65      1    MOL
......................................................................gugagcaaaguuucaggugCgu................   15      1    MOL
......................................................................gugagcaaaguuucaggugugC................   267     1    MOL
......................................................................gugagcaaaguuucaCgugugu................   63      1    MOL
......................................................................gugagcaaaguuucagAugugu................   94      1    MOL
......................................................................gugagcaGaguuucaggugugu................   17      1    MOL
......................................................................gugagcaaaguuucaggCgugu................   22      1    MOL
......................................................................gugagcaaaguuucaggugugu................   291869  0    MOL
......................................................................gugagcaaCguuucaggugugu................   18      1    MOL
......................................................................gugCgcaaaguuucaggugugu................   14      1    MOL
......................................................................gugagGaaaguuucaggugugu................   77      1    MOL
......................................................................gugagcaaaguuucCggugugu................   7       1    MOL
......................................................................gugagcaaagGuucaggugugu................   31      1    MOL
......................................................................gugagcaaaguuuGaggugugu................   97      1    MOL
......................................................................gugagcaaaguuAcaggugugu................   111     1    MOL
......................................................................gugagcaaaguuucaggAgugu................   73      1    MOL
......................................................................gugagcaaaguGucaggugugu................   7       1    MOL
......................................................................gugagcaaaguuucaUgugugu................   72      1    MOL
......................................................................gugagcaaaguuuAaggugugu................   23      1    MOL
......................................................................gugagcaaaUuuucaggugugu................   8       1    MOL
......................................................................gGgagcaaaguuucaggugugu................   47      1    MOL
......................................................................gugagcaaaguuucagguUugu................   49      1    MOL
......................................................................gugagcaaaguuuUaggugugu................   180     1    MOL
......................................................................gugagcaaaguuucaAgugugu................   59      1    MOL
......................................................................gugagcaaaguuGcaggugugu................   17      1    MOL
......................................................................gugagcaaGguuucaggugugu................   42      1    MOL
......................................................................gugagcaaaguuucaggugugG................   133     1    MOL
......................................................................gugagcaaaguuucGggugugu................   123     1    MOL
......................................................................gugagcaaaguuCcaggugugu................   30      1    MOL
```

Star **Mature**

```
uuguaggucugugcauuuauacagcucuacuuggcgccugaaagcuugacucaaccuuugcauagucaaggugagcaaaguuucaggugugucagugggguuaaaaug
......................................................................gugaCcaaaguuucaggugugu................   30    1   MOL
......................................................................gugagcaaaguuucaggugugAc...............   8     1   MOL
......................................................................gugagcaaaguuucaggugugCc...............   1     1   MOL
......................................................................gugagcaaaguuucagguguguc...............   85    0   MOL
......................................................................gugagcaaaguuucagguguguA...............   1520  1   MOL
......................................................................gugagcaaaguuucaUguguguc...............   1     1   MOL
......................................................................gugagcaaaguuucagguguguG...............   47    1   MOL
......................................................................gugagcaaaguuucaggugugucG..............   5     1   MOL
......................................................................gugagcaaaguuucagguguguGa..............   12    1   MOL
......................................................................gugagcaaaguuucagguguguUa..............   182   1   MOL
......................................................................gugGgcaaaguuucaggugugucа..............   1     1   MOL
......................................................................gugagcaaaguuucaggugugAca..............   2     1   MOL
......................................................................gugagcaaaguuucagguguguAa..............   184   1   MOL
......................................................................gugagcaaaguuucaggugugucа..............   25    0   MOL
......................................................................gugagcaaaguuucaggugugucU..............   2     1   MOL
......................................................................gugagcaaaguuucaggugugucagA............   1     1   MOL
......................................................................gugagcaaaguuucagguguguGagu............   1     1   MOL
......................................................................gugagcaaaguuucagguguguGagug...........   1     1   MOL
.......................................................................ugagcaaaguuucaggugug.................   1     0   MOL
.......................................................................ugagcaaaUuucaggugugu................   1     1   MOL
.......................................................................Ggagcaaaguuucaggugugu................   2     1   MOL
.......................................................................ugagcaaaguuucaggugugA................   2     1   MOL
.......................................................................ugagcaaaguuucaggugugu................   17    0   MOL
........................................................................gagcaaaguuucaggugug.................   3     0   MOL
........................................................................gagcUaaguuucaggugugu................   1     1   MOL
........................................................................gagcaaaguuucaggugugu................   35    0   MOL
........................................................................gUgcaaaguuucaggugugu................   1     1   MOL
.........................................................................agcaaaguuucaggugugu................   9     0   MOL
..........................................................................gcaaaguuucaggugugu................   8     0   MOL

..............auuuauacagcucuacuugg..........................................................................   1     0   te1
...............................uggcgccugaaagcuugacucaaccuu..................................................   1     0   te1
.................................gcgccugaaagcuugacucaaccu...................................................   2     0   te1
..................................cgccugaaagcuugacuca.......................................................   1     0   te1
..................................cgccugaaagcuugacucaa......................................................   6     0   te1
..................................cgccugaaagcuugacucaac.....................................................   10    0   te1
..................................cgccugaaagcuugGcucaacc....................................................   1     1   te1
..................................cgccugaaagcuugacucaacc....................................................   17    0   te1
..................................cgccugaaagcuugacucaacUu...................................................   1     1   te1
..................................cgccugaaagcuugacucaaccu...................................................   233   0   te1
..................................cgccugaaagcuugacucaaccG...................................................   2     1   te1
..................................cgccugaaagcuugacucaacAu...................................................   1     1   te1
..................................cgccugaaagcuugacucaaccuu..................................................   12    0   te1
..................................cgccugaaagcuugacucaaccuuu.................................................   1     0   te1
..................................cgccugaaagcuugacucaaccuAu.................................................   1     1   te1
..................................cgccugaaagcuugacucaaccuuug................................................   1     0   te1
...................................gccugGaagcuugacucaaccu...................................................   1     1   te1
..................................................................Gaaggugagcaaaguuucaggugugu................   1     1   te1
...................................................................Gaggugagcaaaguuucaggugugu................   1     1   te1
...................................................................aCggugagcaaaguuucaggugugu................   1     1   te1
...................................................................aGggugagcaaaguuucaggugugu................   2     1   te1
....................................................................Gggugagcaaaguuucaggugugu................   1     1   te1
.....................................................................ggugagcaaaguuucagg.....................   2     0   te1
.....................................................................ggugagcaaaguuucaggugu..................   30    0   te1
.....................................................................Ugugagcaaaguuucaggugu..................   1     1   te1
.....................................................................ggugagcaaaguuucagguguU.................   10    1   te1
.....................................................................ggugagcaaaguuucaggugug.................   17    0   te1
.....................................................................ggugagcaaaguuuUaggugugu................   1     1   te1
.....................................................................ggugagcaaaguuucagguguUu................   1     1   te1
.....................................................................ggugagcaaaguuucaggugugA................   15    1   te1
.....................................................................ggugagcaaaguuucagUugugu................   1     1   te1
.....................................................................ggugagcaaaguuucaggugugG................   1     1   te1
.....................................................................gAugagcaaaguuucaggugugu................   9     1   te1
.....................................................................Ugugagcaaaguuucaggugugu................   26    1   te1
.....................................................................ggugagcaaaguuucaggugugu................   334   0   te1
......................................................................gugagcaaaguuucaggu....................   59    0   te1
......................................................................gugagcaaagCuucaggug...................   1     1   te1
......................................................................gugagcaaaguuucagguC...................   1     1   te1
......................................................................gugagcaaaguuucaggug...................   45    0   te1
......................................................................gugagcaaaguuucagguU...................   1     1   te1
```

Star Mature

```
uuguaggucugugcauuuauacagcucuacuuggcgccugaaagcuugacucaaccuuugcauagucaaggugagcaaaguuucaggugugucaguggggguuaaaaug
......................................................................gugagcaaaguuuUaggugu...................  1      1  te1
......................................................................Uugagcaaaguuucaggugu...................  1      1  te1
......................................................................gugagcaaagCuucaggugu...................  1      1  te1
......................................................................gugaCcaaaguuucaggugu...................  1      1  te1
......................................................................gugagcaaCguuucaggugu...................  1      1  te1
......................................................................gugagcaaagGuucaggugu...................  1      1  te1
......................................................................gugGgcaaaguuucaggugu...................  1      1  te1
......................................................................gugagcaaaguuucaggGgu...................  2      1  te1
......................................................................gugagcaaaguuucaggCgu...................  1      1  te1
......................................................................gugagcaaaguuuGaggugu...................  1      1  te1
......................................................................gugagAaaaguuucaggugu...................  1      1  te1
......................................................................gugagcaaaguuucaggugu...................  1121   0  te1
......................................................................gugagcaaaguuucaggugC...................  3      1  te1
......................................................................gugagcaaaguuucagguguC..................  2      1  te1
......................................................................gugagcaaaguuucagguguA..................  1      1  te1
......................................................................gugagcaaaguuuGaggugug..................  1      1  te1
......................................................................gugagcaaaguuucaggugug..................  1531   0  te1
......................................................................gugagcaaaguuucagguguU..................  141    1  te1
......................................................................gugagcaaaguuuUaggugug..................  1      1  te1
......................................................................gugagcaaagGuucaggugug..................  2      1  te1
......................................................................gugagcaaaguuGcaggugug..................  1      1  te1
......................................................................gugagcaaaguAucaggugug..................  1      1  te1
......................................................................gugGgcaaaguuucaggugug..................  1      1  te1
......................................................................gugagcaaaguuucaggCgug..................  1      1  te1
......................................................................gugagUaaaguuucaggugug..................  1      1  te1
......................................................................gugagcaaaguCucaggugug..................  1      1  te1
......................................................................gugagcaaaguuucGggugug..................  1      1  te1
......................................................................gugagcaaaguuucaggAgug..................  1      1  te1
......................................................................gugagcaaagGuucaggugugu.................  17     1  te1
......................................................................gugagcaaaguuucagguCugu.................  4      1  te1
......................................................................Uugagcaaaguuucaggugugu.................  18     1  te1
......................................................................gugagcaaCguuucaggugugu.................  1      1  te1
......................................................................gugagcaaaguuCcaggugugu.................  9      1  te1
......................................................................gugagcaaaguuucagAugugu.................  4      1  te1
......................................................................gugagcaaaguuucaggugugu.................  73965  0  te1
......................................................................gugagcaaaguuucaggugugC.................  40     1  te1
......................................................................gugagGaaaguuucaggugugu.................  7      1  te1
......................................................................gugagcaaaguuucCggugugu.................  4      1  te1
......................................................................gugCgcaaaguuucaggugugu.................  31     1  te1
......................................................................Cugagcaaaguuucaggugugu.................  3      1  te1
......................................................................gugagcaaaguuucagguguCu.................  1      1  te1
......................................................................gugagcaaaUuuucaggugugu.................  1      1  te1
......................................................................gugagcaaaguuucaggugAgu.................  1      1  te1
......................................................................gugagcaaaAuuucaggugugu.................  1      1  te1
......................................................................gugagcaaaguuuGaggugugu.................  15     1  te1
......................................................................gugagcaaaguuucaggugGgu.................  19     1  te1
......................................................................gugagUaaaguuucaggugugu.................  36     1  te1
......................................................................gugagcaaaguuucaggGgugu.................  24     1  te1
......................................................................gugagcaaaguuucagguguUu.................  12     1  te1
......................................................................gugagcaaaguuucaCgugugu.................  6      1  te1
......................................................................gugUgcaaaguuucaggugugu.................  14     1  te1
......................................................................gugagcaaaguuucaggugugA.................  1381   1  te1
......................................................................gugagcaaaguuucagguguAu.................  3      1  te1
......................................................................gugagcCaaguuucaggugugu.................  5      1  te1
......................................................................guAagcaaaguuucaggugugu.................  1      1  te1
......................................................................gugagcaaaguuucaggugugG.................  178    1  te1
......................................................................gCgagcaaaguuucaggugugu.................  11     1  te1
......................................................................gugagcaaaguuucagCugugu.................  3      1  te1
......................................................................gugagcaaaguuGcaggugugu.................  1      1  te1
......................................................................gugagcaaaguuuUaggugugu.................  52     1  te1
......................................................................gugagcaaaguAucaggugugu.................  18     1  te1
......................................................................gugagcaaaguuucaUgugugu.................  5      1  te1
......................................................................gAgagcaaaguuucaggugugu.................  17     1  te1
......................................................................gugaUcaaaguuucaggugugu.................  10     1  te1
......................................................................gugagcaaagAuucaggugugu.................  8      1  te1
......................................................................gugaCcaaaguuucaggugugu.................  11     1  te1
......................................................................gugagcGaaguuucaggugugu.................  4      1  te1
......................................................................gugagcaGaguuucaggugugu.................  7      1  te1
......................................................................gugagcaaaguuucUggugugu.................  6      1  te1
......................................................................gugGgcaaaguuucaggugugu.................  17     1  te1
```

Star **Mature**

uuguaggucugugcauuuauacagcucuacuuggcgccugaaagcuugacucaaccuuugcauagucaaggugagcaaaguuucaggugugucagugggguuaaaaug			
....gugagcaaGguuucaggugugu................	2	1	te1
....gugagcaaaguGucaggugugu................	8	1	te1
....gugagcaaaguuucaggugCgu................	18	1	te1
....gugagcaaaguuucaggCgugu................	25	1	te1
....gugagAaaaguuucaggugugu................	6	1	te1
....gugagcaaaguuucagUugugu................	8	1	te1
....gugagcaaaCuuucaggugugu................	1	1	te1
....gugaAcaaaguuucaggugugu................	24	1	te1
....guUagcaaaguuucaggugugu................	6	1	te1
....gugagcaUaguuucaggugugu................	3	1	te1
....gugagcaaaguuucagguAugu................	7	1	te1
....gugagcaaaguuucagguUugu................	11	1	te1
....gugagcaaaguuAcaggugugu................	1	1	te1
....gugagcaCaguuucaggugugu................	9	1	te1
....gugagcaaaguCucaggugugu................	10	1	te1
....guCagcaaaguuucaggugugu................	3	1	te1
....gugagcaaagCuucaggugugu................	20	1	te1
....gugagcaaaguuucaggAgugu................	4	1	te1
....gugagcUaaguuucaggugugu................	4	1	te1
....gGgagcaaaguuucaggugugu................	14	1	te1
....gugagcaaaguuucaAgugugu................	7	1	te1
....gugagcaaaguuucGgugugu................	31	1	te1
....gugagcaaaguuucaggugugc...............	8	0	te1
....gugagcaaaguuucagguguguG...............	5	1	te1
....gugagcaaaguuucagguguguA...............	272	1	te1
....gugagcaaaguuucagguguguUa..............	31	1	te1
....gugagcaaaguuucagguguguGa..............	1	1	te1
....gugagcaaaguuucagguguguAa..............	44	1	te1
....gugagcaaaguuucaggugugucU..............	3	1	te1
....gugagcaaaguuucaggugugucA..............	2	0	te1
....ugagcaaaguuucaggugug.................	2	0	te1
....Agagcaaaguuucaggugugu................	1	1	te1
....ugagcaaaguuucaggugugG................	1	1	te1
....ugagcaaaguuucaggugugu................	67	0	te1
....ugagcaaaguCucaggugugu................	1	1	te1
....ugagcCaaguuucaggugugu................	3	1	te1
....ugagcaaaguuucaggugugA................	1	1	te1
....Ggagcaaaguuucaggugugu................	1	1	te1
....gagcaaaguuucaggugugu................	38	0	te1
....Aagcaaaguuucaggugugu................	1	1	te1
....agcaaaguuucaggugugu................	8	0	te1
....gcaaaguuuUaggugugu................	1	1	te1
....gcaaaguuucaggugugu................	29	0	te1
...............................uggcgccugaaagcuugacu...	1	0	T6P
...............................uggcgccugaaagcuugacucaaccu...	1	0	T6P
.................................gcgccugaaagcuugacucaaccu...	4	0	T6P
..................................cgccugaaagcuugacuc..	1	0	T6P
..................................cgccugaaagcuugacucaa..	4	0	T6P
..................................cgccugaaagcuugacucaac...	17	0	T6P
..................................cgccugaaagcuugacucaacc..	29	0	T6P
..................................cgccugaaagcuugGcucaaccu...	2	1	T6P
..................................cgccugaaagcuugUcucaaccu...	3	1	T6P
..................................cgccugaaagcuugacucaaccA...	1	1	T6P
..................................cgccugaaagcuugacucaaccG...	1	1	T6P
..................................cgccugaaagcuugacucaaccC...	1	1	T6P
..................................cgccugaaagcuugacucaCccu...	1	1	T6P
..................................Ugccugaaagcuugacucaaccu...	2	1	T6P
..................................cgccugCaagcuugacucaaccu...	1	1	T6P
..................................cgcUugaaagcuugacucaaccu...	1	1	T6P
..................................cgccugaaagcuugCcucaaccu...	1	1	T6P
..................................cgccugaaagcuugaUucaaccu...	1	1	T6P
..................................cgccugaaagcuugacuUaaccu...	1	1	T6P
..................................cgccugaaagcuugacucaaccu...	635	0	T6P
..................................cgccugaaagcuugacucaaccuA..	2	1	T6P
..................................cgccugaaagcuugacucaaccuu..	60	0	T6P
..................................cgccugaaagcuugacucaaccuuu...	16	0	T6P
..................................cgccugaaagcuugacucaaccuuugc...	2	0	T6P
...................................gccugaaagcuugacucaaccu...	1	0	T6P
...................................gccugaaagcuugacucaaccuuug..	1	0	T6P

Star **Mature**

uuguaggucugugcauuuauacagcucuacuuggcgccugaaagcuugacucaaccuuugcauagucaaggugagcaaaguuucaggugugucagugggguuaaaaug			
....................................ccugaaagcuugacucaaccu...	1	0	T6P
....Gaaggugagcaaaguuucaggugugu................	2	1	T6P
....aGggugagcaaaguuucaggugugu................	3	1	T6P
....aCggugagcaaaguuucaggugugu................	1	1	T6P
....Gaggugagcaaaguuucaggugugu................	1	1	T6P
....Uggugagcaaaguuucaggugugu................	2	1	T6P
....Gggugagcaaaguuucaggugugu................	1	1	T6P
....ggugagcaaaguuucaggu....................	1	0	T6P
....ggugagcaaaguuucaggug...................	1	0	T6P
....ggugagcaaaguuucaggugu..................	49	0	T6P
....ggugaAcaaaguuucaggugug.................	1	1	T6P
....ggugagcaaaguuucagguguU.................	5	1	T6P
....ggCgagcaaaguuucaggugug.................	1	1	T6P
....ggugagcaaaguuucaggugug.................	93	0	T6P
....Cgugagcaaaguuucaggugugu................	2	1	T6P
....ggCgagcaaaguuucaggugugu................	1	1	T6P
....ggugagcaaaguuucagguguUu................	2	1	T6P
....ggugagUaaaguuucaggugugu................	1	1	T6P
....ggugagcaaaguuucGggugugu................	2	1	T6P
....Ugugagcaaaguuucaggugugu................	78	1	T6P
....ggugagcaaaguuucaCgugugu................	1	1	T6P
....ggugagcaaaguuucaggugCgu................	1	1	T6P
....gCugagcaaaguuucaggugugu................	2	1	T6P
....gAugagcaaaguuucaggugugu................	1	1	T6P
....ggugagcaaaguuucaggugGgu................	1	1	T6P
....ggugagcaaaUuuucaggugugu................	1	1	T6P
....ggugagGaaaguuucaggugugu................	2	1	T6P
....gUugagcaaaguuucaggugugu................	6	1	T6P
....ggugagcaaaguuGcaggugugu................	1	1	T6P
....ggugagcaaaguuucaggugugA................	49	1	T6P
....ggugagcaaaguuucagAugugu................	1	1	T6P
....gguUagcaaaguuucaggugugu................	1	1	T6P
....ggugaCcaaaguuucaggugugu................	2	1	T6P
....ggugagAaaaguuucaggugugu................	1	1	T6P
....ggugagcaaaguuucaggugugu................	2162	0	T6P
....ggugagcaaaguuucagCugugu................	2	1	T6P
....ggugagcaaaguuucaUgugugu................	2	1	T6P
....ggugagcaaaguuucUggugugu................	1	1	T6P
....ggugagcaaaguuucaggugugG................	1	1	T6P
....ggugagcaaaAuuucaggugugu................	1	1	T6P
....ggugagcaaaguuucagguguguA...............	8	1	T6P
....ggugagcaaaguuucaggugugucc...............	1	0	T6P
....ggugagcaaaguuucagguguguAa..............	1	1	T6P
....gugagcaaaguuucaUgu....................	1	1	T6P
....gugagcaaaguuucaggu....................	172	0	T6P
....gugagcaaaguuucagguA...................	1	1	T6P
....gugagcaaaguuucaggug...................	151	0	T6P
....gugagcaaaguuucUggug...................	1	1	T6P
....gugagcaaaguuuUaggug...................	1	1	T6P
....gugagcaaaguuucagguC...................	3	1	T6P
....gugUgcaaaguuucaggugu..................	1	1	T6P
....gugagcaaaguuucaggugA..................	1	1	T6P
....gugagcaaaguuucaggGgu..................	5	1	T6P
....gugagcaaaguuucaggugu..................	1326	0	T6P
....guAagcaaaguuucaggugu..................	1	1	T6P
....gugagUaaaguuucaggugu..................	1	1	T6P
....gugagcaaaguuucaUgugu..................	2	1	T6P
....gugagcCaaguuucaggugu..................	1	1	T6P
....gugagcaaaguuucaAgugu..................	1	1	T6P
....gAgagcaaaguuucaggugu..................	1	1	T6P
....gugagcaaaguuucGggugu..................	1	1	T6P
....gugagcaaaguuucaggugC..................	2	1	T6P
....gugagcaaaguuucagguAu..................	1	1	T6P
....gugagcaaaguCucaggugu..................	2	1	T6P
....gugagcaaaguuucUggugu..................	1	1	T6P
....gugUgcaaaguuucaggugug.................	7	1	T6P
....gugaUcaaaguuucaggugug.................	1	1	T6P
....guUagcaaaguuucaggugug.................	2	1	T6P
....gugagcaaaguuucaggCgug.................	2	1	T6P
....Cugagcaaaguuucaggugug.................	2	1	T6P

```
                                  Star                                Mature
uuguaggucugugcauuuauacagcucuacuuggcgccugaaagcuugacucaaccuuugcauagucaaggugagcaaaguuucaggugugucagugggguuaaaaug
......................................................................Uugagcaaaguuucaggugug.................   2        1    T6P
......................................................................gugagcaaaguuuUaggugug.................   3        1    T6P
......................................................................gugagcaaaguuCcaggugug.................   2        1    T6P
......................................................................gugagcaaaguuucGggugug.................   6        1    T6P
......................................................................gugagcGaaguuucaggugug.................   4        1    T6P
......................................................................gugagcaaaguuucaggugCg.................   12       1    T6P
......................................................................gugagcaaaguuucaggugAg.................   1        1    T6P
......................................................................gugagcaaaguuuGaggugug.................   2        1    T6P
......................................................................gugagcaaaAuuucaggugug.................   4        1    T6P
......................................................................gugagcaaaguuucagUugug.................   2        1    T6P
......................................................................gugagcaaaguuuAaggugug.................   3        1    T6P
......................................................................gugagcaUaguuucaggugug.................   1        1    T6P
......................................................................gGgagcaaaguuucaggugug.................   1        1    T6P
......................................................................gugaAcaaaguuucaggugug.................   1        1    T6P
......................................................................gugaCcaaaguuucaggugug.................   1        1    T6P
......................................................................gugagcaaaguuucagguguU.................   121      1    T6P
......................................................................gugagcaaaguuucagguCug.................   1        1    T6P
......................................................................gugagcaaaguuucagguguC.................   2        1    T6P
......................................................................gugagcaaaguuucaggugug.................   5625     0    T6P
......................................................................gugagcaaagGuucaggugug.................   1        1    T6P
......................................................................gugagcaaaguuucaAgugug.................   1        1    T6P
......................................................................gugagcaaaguGucaggugug.................   1        1    T6P
......................................................................gugagcaaaUuuucaggugug.................   2        1    T6P
......................................................................gugagcaaaguuucaUgugug.................   4        1    T6P
......................................................................gugagcaaaguuucaCgugug.................   1        1    T6P
......................................................................gugagcaaaguuucagguguA.................   5        1    T6P
......................................................................gugCgcaaaguuucaggugug.................   1        1    T6P
......................................................................guAagcaaaguuucaggugug.................   1        1    T6P
......................................................................gugagUaaaguuucaggugug.................   3        1    T6P
......................................................................gugagAaaaguuucaggugugu................   6        1    T6P
......................................................................gugagcaaaguuucagguguUu................   73       1    T6P
......................................................................gugagcaaaguuucaggGgugu................   41       1    T6P
......................................................................gugagcaaaguGucaggugugu................   6        1    T6P
......................................................................gugagcaaaguuucCggugugu................   31       1    T6P
......................................................................gugagcaaaguuAcaggugugu................   2        1    T6P
......................................................................guAagcaaaguuucaggugugu................   27       1    T6P
......................................................................gugagcaaaguuucagUugugu................   152      1    T6P
......................................................................gugagcGaaguuucaggugugu................   306      1    T6P
......................................................................gugagcaaaguuucagCugugu................   122      1    T6P
......................................................................gugagcaaaguuucagguCugu................   57       1    T6P
......................................................................gugagcaaaguuucaggugugu................   339506   0    T6P
......................................................................gugUgcaaaguuucaggugugu................   525      1    T6P
......................................................................gugagcaaaguuucaCgugugu................   138      1    T6P
......................................................................gugagcaaaguuuGaggugugu................   89       1    T6P
......................................................................gugagcUaaguuucaggugugu................   48       1    T6P
......................................................................gugagcaaagCuucaggugugu................   17       1    T6P
......................................................................gugagUaaaguuucaggugugu................   138      1    T6P
......................................................................gugagcaGaguuucaggugugu................   18       1    T6P
......................................................................gugCgcaaaguuucaggugugu................   29       1    T6P
......................................................................gugagcaaaguuucaggugugA................   1985     1    T6P
......................................................................gugagcaaaguAucaggugugu................   12       1    T6P
......................................................................guCagcaaaguuucaggugugu................   127      1    T6P
......................................................................gugagcaaaCuuucaggugugu................   55       1    T6P
......................................................................gugagcaaUguuucaggugugu................   9        1    T6P
......................................................................gCgagcaaaguuucaggugugu................   23       1    T6P
......................................................................gugagcCaaguuucaggugugu................   69       1    T6P
......................................................................gugagcaaaguuucGggugugu................   207      1    T6P
......................................................................gugagcaaaguuucaggugAgu................   37       1    T6P
......................................................................gugagcaaaguuucagguUugu................   72       1    T6P
......................................................................gugagcaaaguuucagAugugu................   101      1    T6P
......................................................................gugagcaaaguuucaggugugC................   448      1    T6P
......................................................................gugagcaaaAuuucaggugugu................   88       1    T6P
......................................................................gugagcaaaguuuUaggugugu................   281      1    T6P
......................................................................gugagcaUaguuucaggugugu................   4        1    T6P
......................................................................gGgagcaaaguuucaggugugu................   27       1    T6P
......................................................................gugagGaaaguuucaggugugu................   6        1    T6P
......................................................................guUagcaaaguuucaggugugu................   94       1    T6P
......................................................................gugagcaaaguCucaggugugu................   30       1    T6P
......................................................................gugagcaaaguuucaggCgugu................   29       1    T6P
......................................................................gugagcaaaguuGcaggugugu................   5        1    T6P
```

Star Mature

```
uuguaggucugugcauuuauacagcucuacuuggcgccugaaagcuugacucaaccuuugcauagucaaggugagcaaaguuucaggugugucagugggguuaaaaug
......................................................................gugagcaaaguuucaUgugugu................    160    1    T6P
......................................................................gugagcaaaguuucagguguAu................    70     1    T6P
......................................................................gugagcaaaguuCcaggugugu................    52     1    T6P
......................................................................gugagcaaaguuucaggugGgu................    27     1    T6P
......................................................................gugagcaaGguuucaggugugu................    16     1    T6P
......................................................................gAgagcaaaguuucaggugugu................    17     1    T6P
......................................................................gugagcaaaguuuAaggugugu................    28     1    T6P
......................................................................gugagcaaCguuucaggugugu................    1      1    T6P
......................................................................gugGgcaaaguuucaggugugu................    49     1    T6P
......................................................................gugagcaaaguuucaggugCgu................    37     1    T6P
......................................................................gugagcaaagGuucaggugugu................    37     1    T6P
......................................................................gugagcaaaguuucagguguCu................    81     1    T6P
......................................................................gugaCcaaaguuucaggugugu................    46     1    T6P
......................................................................gugagcaaaguuucaAgugugu................    54     1    T6P
......................................................................gugagcaaagAuucaggugugu................    6      1    T6P
......................................................................gugagcaaaUuuucaggugugu................    67     1    T6P
......................................................................Cugagcaaaguuucaggugugu................    23     1    T6P
......................................................................Uugagcaaaguuucaggugugu................    99     1    T6P
......................................................................gugaUcaaaguuucaggugugu................    60     1    T6P
......................................................................gugaAcaaaguuucaggugugu................    65     1    T6P
......................................................................gugagcaaaguuucUggugugu................    78     1    T6P
......................................................................gugagcaaaguuucaggugugG................    56     1    T6P
......................................................................gugagcaaaguuucagguAugu................    29     1    T6P
......................................................................gugagcaaaguuucaggAgugu................    16     1    T6P
......................................................................gugagcaaaguuucagguguguG...............    36     1    T6P
......................................................................gugagcaaaguuucagguguguA...............    991    1    T6P
......................................................................gugagcaaaguuucagguguguc...............    78     0    T6P
......................................................................gugagcaaaguuucaggugugAc...............    1      1    T6P
......................................................................gugagcaaaguuucaAguguguc...............    1      1    T6P
......................................................................gugagcaaaguuucagAuguguc...............    1      1    T6P
......................................................................gugagcaaaguuucagguguguUa..............    119    1    T6P
......................................................................gugagcaaaguuucagguguguGa..............    10     1    T6P
......................................................................gugagcaaaguuucagguguguAa..............    137    1    T6P
......................................................................gugagcaaaguuucaggugugucU..............    1      1    T6P
......................................................................gugagcaaaguuucaggugugucа..............    16     0    T6P
......................................................................gugagcaaaguuucaggugugucaA.............    1      1    T6P
......................................................................gugagcaaaguuucagguguguGagu............    1      1    T6P
.......................................................................ugagcaaaguuucaggugug.................    1      0    T6P
.......................................................................uUagcaaaguuucaggugugu................    2      1    T6P
.......................................................................Ggagcaaaguuucaggugugu................    4      1    T6P
.......................................................................uCagcaaaguuucaggugugu................    3      1    T6P
.......................................................................ugagcaaaguuucaggugugA................    1      1    T6P
.......................................................................ugagcaaaguuucaggugugu................    47     0    T6P
........................................................................gagcaaaguuucaggugu..................    1      0    T6P
........................................................................gagcaaaguuucaggugug.................    3      0    T6P
........................................................................gagcaaaguuucaggugugu................    143    0    T6P
........................................................................Uagcaaaguuucaggugugu................    4      1    T6P
........................................................................gUgcaaaguuucaggugugu................    3      1    T6P
........................................................................Cagcaaaguuucaggugugu................    21     1    T6P
........................................................................gagcaaaguuucaggGgugu................    1      1    T6P
.........................................................................agcaaaguuucaggugugu................    28     0    T6P
.........................................................................agcaaaguuucaggugugC................    1      1    T6P
..........................................................................gcaaaguuucaggugugu................    21     0    T6P
..........................................................................gcaaaguuucagguguguc...............    1      0    T6P

..............auuuauacagcucuacuugg..........................................................................    1      0    egg
..................................cUccugaaagcuugacucaaccu...................................................    1      1    egg
..................................cgccugaaagcuugGcucaaccu...................................................    1      1    egg
..................................cgccugaaagcuugacucaGccu...................................................    1      1    egg
..................................cgccugaaagcuugacucaaccu...................................................    29     0    egg
..................................cgccugaaagcuCgacucaaccu...................................................    2      1    egg
..................................cgccugaaagcCugacucaaccu...................................................    1      1    egg
..................................cgccugaaagcuugacucaaccC...................................................    1      1    egg
..................................cgccugaaagcuugacucaaccuu..................................................    3      0    egg
..................................cgccugaaagcuugacucaaccuA..................................................    1      1    egg
..................................cgccugaaagcuugacucaaccuuuA................................................    2      1    egg
.....................................................................ggugagcaaaguuucaggugug.................    3      0    egg
.....................................................................ggugagcaaaguuucaggCgugu................    1      1    egg
.....................................................................gCugagcaaaguuucaggugugu................    1      1    egg
.....................................................................Cgugagcaaaguuucaggugugu................    2      1    egg
```

Star **Mature**

```
uuguaggucugugcauuuauacagcucuacuuggcgccugaaagcuugacucaaccuuugcauagucaaggugagcaaaguuucaggugugucagugggguuaaaaug
.....................................................................ggugagcaaaguuucaggugugu................   6      0   egg
......................................................................gugagcaaaguuucaggu....................   1      0   egg
......................................................................gugagcaaaguuucaggugu..................   1      0   egg
......................................................................gugagcaaGguuucaggugu..................   1      1   egg
......................................................................Nugagcaaaguuucaggugug.................   1      1   egg
......................................................................gugagcaGaguuucaggugug.................   1      1   egg
......................................................................gugagcaaaguuucaggugug.................   49     0   egg
......................................................................guAagcaaaguuucaggugugu................   1      1   egg
......................................................................gugaCcaaaguuucaggugugu................   2      1   egg
......................................................................gugagcaaaguuGcaggugugu................   1      1   egg
......................................................................gugagcaaaguCucaggugugu................   4      1   egg
......................................................................gAgagcaaaguuucaggugugu................   4      1   egg
......................................................................gugagcaaaguuucaAgugugu................   2      1   egg
......................................................................gugagcaaaguuucaggGgugu................   2      1   egg
......................................................................gugagcaaUguuucaggugugu................   1      1   egg
......................................................................gugagcaaaguuCcaggugugu................   5      1   egg
......................................................................gugagcaaaguuucaggugGgu................   1      1   egg
......................................................................gugagcaaGguuucaggugugu................   32     1   egg
......................................................................gugaUcaaaguuucaggugugu................   42     1   egg
......................................................................gugagGaaaguuucaggugugu................   2      1   egg
......................................................................gCgagcaaaguuucaggugugu................   4      1   egg
......................................................................Nugagcaaaguuucaggugugu................   2      1   egg
......................................................................gugagcaaaguuucUggugugu................   1      1   egg
......................................................................gugagcaaagCuucaggugugu................   3      1   egg
......................................................................gugagcaaaguGucaggugugu................   1      1   egg
......................................................................gugagAaaaguuucaggugugu................   1      1   egg
......................................................................gugagcGaaguuucaggugugu................   2      1   egg
......................................................................gugagcaaaguuucaggugCgu................   5      1   egg
......................................................................gugagcaaaguuucaggugugu................   1262   0   egg
......................................................................gugagcaaaguuucaUgugugu................   1      1   egg
......................................................................Uugagcaaaguuucaggugugu................   4      1   egg
......................................................................gugagcaaaguuucagguAugu................   3      1   egg
......................................................................gugaAcaaaguuucaggugugu................   8      1   egg
......................................................................gugagcaaaguuucaggugugG................   4      1   egg
......................................................................gugagUaaaguuucaggugugu................   1      1   egg
......................................................................gugagcaaaguuucGgugugu................   1      1   egg
......................................................................gugagcaGaguuucaggugugu................   5      1   egg
......................................................................gugagcaaaCuuucaggugugu................   1      1   egg
......................................................................gugUgcaaaguuucaggugugu................   11     1   egg
......................................................................gugagcaUaguuucaggugugu................   2      1   egg
......................................................................gugagcaaaguuucagAugugu................   2      1   egg
......................................................................gugagcaaaAuuucaggugugu................   2      1   egg
......................................................................gugagcaaaguuucCggugugu................   3      1   egg
......................................................................gugagcaaaguuucaggugugA................   27     1   egg
......................................................................gugagcaaaguuucaggugugC................   90     1   egg
......................................................................gugagcaaaguuucagguguUu................   2      1   egg
......................................................................gugagcaaaguuucaggCgugu................   4      1   egg
......................................................................gugGgcaaaguuucaggugugu................   7      1   egg
......................................................................gugagcaaaguuucagguguAu................   1      1   egg
......................................................................gugagcaaaguuucagguguguA...............   14     1   egg
......................................................................gugagcaaaguuucagguguguUa..............   1      1   egg
.......................................................................ugagcaaaguuucaggugugu................   1      0   egg

...............................uggcgccugaaagcuugacuca.......................................................   1      0   T53
...............................uggcgccugaaagcuugacucaacU....................................................   1      1   T53
..................................cgccugaaagcuugacuc........................................................   1      0   T53
..................................cgccugaaagcuugacuca.......................................................   2      0   T53
..................................cgccugaaagcuugacucaa......................................................   2      0   T53
..................................cgccugaaagcuugacucaac.....................................................   4      0   T53
..................................cgccugaaagcuugacucaacG....................................................   1      1   T53
..................................cgccugaaagcuugacucaacc....................................................   24     0   T53
..................................cgccuCaaagcuugacucaaccu...................................................   1      1   T53
..................................cgccugaaagcuugacucaaccA...................................................   2      1   T53
..................................cgccugaaUgcuugacucaaccu...................................................   1      1   T53
..................................cgccugaaagcuugacucaaccu...................................................   228    0   T53
..................................cgccugaaagcuugUcucaaccu...................................................   1      1   T53
..................................cgccugaaagcuugacucaaccuu..................................................   9      0   T53
..................................cgccugaaagcuugacucaaccuuu.................................................   2      0   T53
..................................cgccugaaagcuugacucaaccuuug................................................   2      0   T53
.........................................................uugcauagucaaggugag.................................   1      0   T53
```

Star / Mature			
uuguaggucugugcauuuauacagcucuacuuggcgccugaaagcuugacucaaccuuugcauagucaaggugagcaaaguuucaggugugucagugggguuaaaaug			
....aGggugagcaaaguuucaggugugu................	4	1	T53
....aAgugagcaaaguuucaggugugu................	1	1	T53
....aggugagcaaaguuucaggugugu................	1	0	T53
....aUgugagcaaaguuucaggugugu................	1	1	T53
....Uggugagcaaaguuucaggugugu................	3	1	T53
....ggugagcaaaguuucagg.....................	1	0	T53
....ggugagcaaaguuucaggugu..................	15	0	T53
....Ugugagcaaaguuucaggugu..................	1	1	T53
....ggugagcaaaguuucagguguU.................	9	1	T53
....ggugagcaaaguuucaggugug.................	24	0	T53
....Ugugagcaaaguuucaggugug.................	1	1	T53
....ggugagcaaaguuucaggugugA................	15	1	T53
....Ugugagcaaaguuucaggugugu................	74	1	T53
....ggugagcaaaguuucaUgugugu................	1	1	T53
....ggugCgcaaaguuucaggugugu................	1	1	T53
....ggugagcaaaguuucaggugugu................	314	0	T53
....ggugagcaaaguuucagguguUu................	9	1	T53
....ggugagcaaaguuucUggugugu................	1	1	T53
....Agugagcaaaguuucaggugugu................	3	1	T53
....Cgugagcaaaguuucaggugugu................	1	1	T53
....ggugagcaaaguuucaggugugu A...............	1	1	T53
....ggugagcaaaguuucaggugugu Ua..............	1	1	T53
....gugagcaaaguuucaggA....................	2	1	T53
....gugagcaaaguuucaggu....................	79	0	T53
....gugagcaaaguuucUggug...................	1	1	T53
....gugagcaaaguuuUaggug...................	1	1	T53
....gugagcaaaguuucaggug...................	72	0	T53
....gugagcaaaguuucaggAg...................	1	1	T53
....gugagcaaaguuucagguC...................	1	1	T53
....gugagcaaaCuuucaggug...................	1	1	T53
....gugagcaaaguuucagguU...................	1	1	T53
....gugaAcaaaguuucaggugu..................	1	1	T53
....gugagcaaaguuucaUgugu..................	1	1	T53
....gugagcaaaguuucagguAu..................	1	1	T53
....gugagcaaaguuAcaggugu..................	1	1	T53
....gugagcaaaguuucUggugu..................	3	1	T53
....gugagcaaaguCucaggugu..................	2	1	T53
....gugagcaaaguuucaggugu..................	1510	0	T53
....gugUgcaaaguuucaggugu..................	2	1	T53
....gugagcaaaguuucaggGgu..................	2	1	T53
....gugagcaaaguuucagAugug.................	2	1	T53
....gugagcaaaguuucaUgugug.................	1	1	T53
....gugagcGaaguuucaggugug.................	1	1	T53
....gugagcaaaguuucaggugug.................	2786	0	T53
....gCgagcaaaguuucaggugug.................	1	1	T53
....gugagAaaaguuucaggugug.................	1	1	T53
....gugagcaaaguuuUaggugug.................	2	1	T53
....gugagcaaaguuucagguguC.................	6	1	T53
....gugagcaaaguuucaggugCg.................	3	1	T53
....gugagcaaaguuucaggCgug.................	2	1	T53
....gugUgcaaaguuucaggugug.................	1	1	T53
....gugagcaaaguuucagguguU.................	271	1	T53
....gugagcaaaCuuucaggugug.................	1	1	T53
....gugagUaaaguuucaggugug.................	1	1	T53
....gugagcaaUguuucaggugug.................	3	1	T53
....gGgagcaaaguuucaggugug.................	1	1	T53
....guUagcaaaguuucaggugug.................	1	1	T53
....gugaAcaaaguuucaggugug.................	2	1	T53
....gugagcaaaguuucagUugug.................	1	1	T53
....gugagcaaaguuucagguguA.................	4	1	T53
....gugagcaaaguuAcaggugug.................	2	1	T53
....gugagcaaaguuucaggugAg.................	1	1	T53
....gugagcaaaguuucUggugug.................	3	1	T53
....gugagcaaaguuucaggGgug.................	1	1	T53
....gugagcaaagCuucaggugug.................	1	1	T53
....gugagcaaaguAucaggugugu................	24	1	T53
....gugagcaaaguuuGaggugugu................	54	1	T53
....gugagcaaaguuucUggugugu................	172	1	T53
....gugagAaaaguuucaggugugu................	23	1	T53
....gugagcaaaguuucCggugugu................	15	1	T53

```
                                  Star                               Mature
uuguaggucugugcauuuauacagcucuacuuggcgccugaaagcuugacucaaccuuugcauagucaaggugagcaaaguuucaggugugucagugggguuaaaaug
......................................................................gugagcaaaguuuAaggugugu................   15       1    T53
......................................................................gugagcaaaguuucagguguUu................   86       1    T53
......................................................................gugagcUaaguuucaggugugu................   3        1    T53
......................................................................gugagcaaaUuuucaggugugu................   2        1    T53
......................................................................gugagcaaaAuuucaggugugu................   6        1    T53
......................................................................gugagcaaaguuucagUugugu................   69       1    T53
......................................................................gugagcaaUguuucaggugugu................   151      1    T53
......................................................................gugagcaaaguuucaggugGgu................   24       1    T53
......................................................................guCagcaaaguuucaggugugu................   6        1    T53
......................................................................gugagcaaaguuucagguguCu................   14       1    T53
......................................................................gugaUcaaaguuucaggugugu................   35       1    T53
......................................................................gugagUaaaguuucaggugugu................   111      1    T53
......................................................................gugagcaaaguuucaggugugA................   2688     1    T53
......................................................................gugagcaaaguuucaggugugC................   138      1    T53
......................................................................guUagcaaaguuucaggugugu................   16       1    T53
......................................................................gugagcaaaguuucaggCgugu................   9        1    T53
......................................................................gugagGaaaguuucaggugugu................   57       1    T53
......................................................................gugagcaUaguuucaggugugu................   9        1    T53
......................................................................gugagcaaaguCucaggugugu................   9        1    T53
......................................................................gugagcaGaguuucaggugugu................   10       1    T53
......................................................................Uugagcaaaguuucaggugugu................   13       1    T53
......................................................................gugagcaaGguuucaggugugu................   16       1    T53
......................................................................gCgagcaaaguuucaggugugu................   30       1    T53
......................................................................gugagcGaaguuucaggugugu................   8        1    T53
......................................................................gugagcaaaguuuUaggugugu................   86       1    T53
......................................................................gugCgcaaaguuucaggugugu................   1        1    T53
......................................................................gugGgcaaaguuucaggugugu................   17       1    T53
......................................................................gugagcaaaguuGcaggugugu................   8        1    T53
......................................................................gugUgcaaaguuucaggugugu................   75       1    T53
......................................................................gugagcaaaguuucagguAugu................   47       1    T53
......................................................................gugagcaaaguuucagguUugu................   32       1    T53
......................................................................gugagcaaaguuucaggugCgu................   7        1    T53
......................................................................gugagcaaaCuuucaggugugu................   82       1    T53
......................................................................gugagcaaagGuucaggugugu................   20       1    T53
......................................................................gugagcaaaguuucaggugugG................   91       1    T53
......................................................................gugagcaaaguuucaggugugu................   219630   0    T53
......................................................................gAgagcaaaguuucaggugugu................   8        1    T53
......................................................................gugagcaaaguuucagAugugu................   38       1    T53
......................................................................guAagcaaaguuucaggugugu................   11       1    T53
......................................................................gugagcaaaguuCcaggugugu................   10       1    T53
......................................................................Cugagcaaaguuucaggugugu................   13       1    T53
......................................................................gugagcaaaguuucaggAgugu................   50       1    T53
......................................................................gugaCcaaaguuucaggugugu................   13       1    T53
......................................................................gugagcaaaguuucaggugAgu................   47       1    T53
......................................................................gugagcaaaguGucaggugugu................   4        1    T53
......................................................................gugagcaaagCuucaggugugu................   10       1    T53
......................................................................gugagcaaaguuAcaggugugu................   73       1    T53
......................................................................gugagcaaaguuucGggugugu................   123      1    T53
......................................................................gugagcaaaguuucagguguAu................   37       1    T53
......................................................................gugagcaaaguuucaCgugugu................   41       1    T53
......................................................................gugaAcaaaguuucaggugugu................   61       1    T53
......................................................................gugagcaaCguuucaggugugu................   11       1    T53
......................................................................gugagcaaaguuucaAgugugu................   39       1    T53
......................................................................gugagcaaagAuucaggugugu................   5        1    T53
......................................................................gugagcaaaguuucaUgugugu................   49       1    T53
......................................................................gugagcaaaguuucagguCugu................   22       1    T53
......................................................................gGgagcaaaguuucaggugugu................   22       1    T53
......................................................................gugagcaaaguuucagCugugu................   24       1    T53
......................................................................gugagcaaaguuucaggGgugu................   48       1    T53
......................................................................gugagcaaaguuucagUuguguc...............   1        1    T53
......................................................................gugagcaaaguuucagguguguA...............   616      1    T53
......................................................................gugagcaaaguuucagguguguc...............   68       0    T53
......................................................................gugagcaaaguuucaggugugGc...............   1        1    T53
......................................................................gugagcaaaguuucagguguguG...............   38       1    T53
......................................................................gugagcaaaguuucagguguguUa..............   90       1    T53
......................................................................gugagcaaaguuucaggugugucG..............   12       1    T53
......................................................................gugagcaaaguuucaggugugucа..............   9        0    T53
......................................................................gugagcaaaguuucagguguguAa..............   85       1    T53
......................................................................gugagcaaaguuucaggugugucC..............   1        1    T53
......................................................................gugagcaaaguuucaggugugucU..............   4        1    T53
```

Star Mature

```
uuguaggucugugcauuuauacagcucuacuuggcgccugaaagcuugacucaaccuuugcauagucaaggugagcaaaguuucaggugugucagugggguuaaaaug
......................................................................gugagcaaaguuucagguguguGa..............	4	1	T53
......................................................................gugagcaaaguuucaggugugucCg.............	1	1	T53
.......................................................................ugagcaaaguuucaggugug.................	1	0	T53
.......................................................................ugagcaaaguuucaggugugA................	2	1	T53
.......................................................................ugagcaaaguuucaggugugu................	21	0	T53
.......................................................................Ggagcaaaguuucaggugugu................	2	1	T53
........................................................................gagcaaaguuucaggugugu................	55	0	T53
.........................................................................agcaaaguuucaggugug.................	1	0	T53
.........................................................................aAcaaaguuucaggugugu................	1	1	T53
.........................................................................agcaaaguuucaggugugu................	21	0	T53
..........................................................................gcaaaguuucaggugugu................	22	0	T53
..........................................................................gcaaaguuucGggugugu................	1	1	T53
```

Provisional ID : Scaffold_1043_1251
Score total : 2245281
Score for star read(s) : 3.9
Score for read counts : 2245272.5
Score for mfe : 0
Score for randfold : 1.6
Score for cons. seed : 3
Total read count : 4404017
Mature read count : 4354088
Loop read count : 11
Star read count : 49918

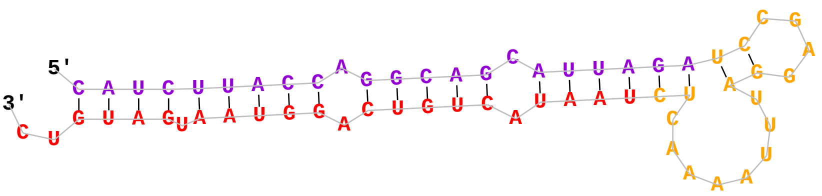

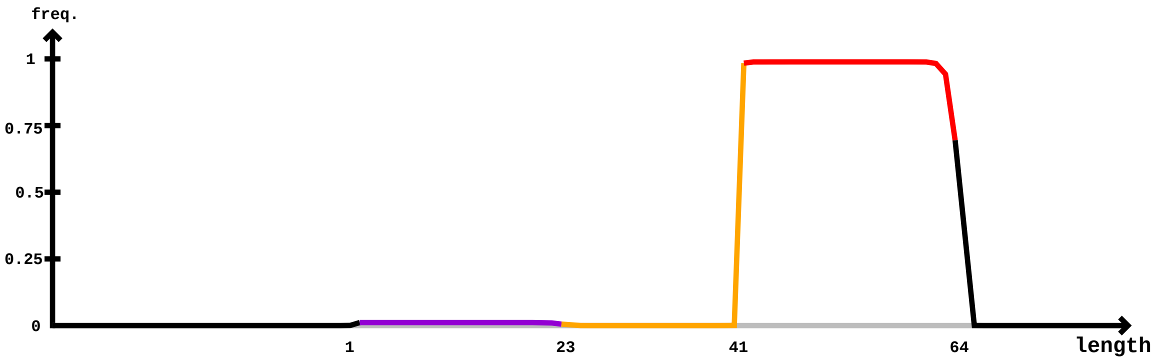


```
                                 Star                                    Mature
5'- uaacaaaaauuauauaauaaucuuuguagucaucuuaccaggcagcauuagauccgaggauuuaaaacucuaauacugucagguaaugaugucagcagaguuucguacaa  -3'  obs
    uaacaaaaauuauauaauaaucuuuguagucaucuuaccaggcagcauuagauccgaggauuuaaaacucuaauacugucagguaaugaugucagcagaguuucguacaa       exp
    ....................((((((.(.(((((((((.(((((.((((((((....))........)))))).))))).))))).)))).).))))))...........  reads  mm  sample
    ..........................uagGcaucuuaccaggcagcauuag...........................................................  1      1   T63
    ............................Uucaucuuaccaggcagcauuaga..........................................................  1      1   T63
    .............................ucaucuuaccaggcagcau..............................................................  1      0   T63
    .............................ucaucuuaccaggcagcaA..............................................................  1      1   T63
    .............................ucaucuuaccaggcagcauu.............................................................  87     0   T63
    .............................ucaucuuaccaggcaCcauu.............................................................  1      1   T63
    .............................ucaAcuuaccaggcagcauu.............................................................  1      1   T63
    .............................Gcaucuuaccaggcagcauu.............................................................  1      1   T63
    .............................ucaucuuacUaggcagcauu.............................................................  1      1   T63
    .............................ucaucuuaccaggcagcauua............................................................  57     0   T63
    .............................ucaucuuaccaggcagcauuU............................................................  2      1   T63
    .............................ucaucuuaccaggcagcauuag...........................................................  86     0   T63
    .............................ucaucuuaccaggcagcauuaA...........................................................  3      1   T63
    .............................ucaucuuacUaggcagcauuag...........................................................  1      1   T63
    .............................ucaucuuaccaggcagUauuag...........................................................  1      1   T63
    .............................ucaucuuaccaggcagcauuaga..........................................................  12     0   T63
    .............................ucaucuuaccaggcagcauuagU..........................................................  1      1   T63
    .............................ucaucuuaccaggcagcauuagUu.........................................................  2      1   T63
    ..............................caucuuaccaggcagcau..............................................................  19     0   T63
    ..............................caucuuaccaggcagcauu.............................................................  712    0   T63
    ..............................caucuuaccagAcagcauu.............................................................  1      1   T63
    ..............................caucuuaccaggcaUcauu.............................................................  1      1   T63
    ..............................caucuuaccGggcagcauu.............................................................  1      1   T63
    ..............................caucuuaccaggcagcaAu.............................................................  1      1   T63
    ..............................caucuuaccaggcagcauA.............................................................  3      1   T63
    ..............................caucuuacUaggcagcauu.............................................................  1      1   T63
    ..............................caucuuaccaggUagcauua............................................................  1      1   T63
    ..............................caucuuaccaggcagcauua............................................................  329    0   T63
    ..............................caucuCaccaggcagcauua............................................................  1      1   T63
    ..............................caucuuaccaggcagcauuU............................................................  7      1   T63
    ..............................caucuuacUaggcagcauuag...........................................................  1      1   T63
    ..............................caucuuaccaggcagcauuCg...........................................................  1      1   T63
    ..............................caucuuaccaggcagUauuag...........................................................  1      1   T63
```

```
                              Star                                       Mature
uaacaaaauuauauaauaaucuuuguagucaucuuaccaggcagcauuagauccgaggauuuaaaacucuaauacugucagguaaugaugucagcagaguuucguacaa
.............................caucuuaccGggcagcauuag...........................................................    13      1   T63
.............................caucuAaccaggcagcauuag...........................................................    2       1   T63
.............................caucuuaccaggcaAcauuag...........................................................    1       1   T63
.............................cCucuuaccaggcagcauuag...........................................................    1       1   T63
.............................caucuGaccaggcagcauuag...........................................................    1       1   T63
.............................caucuuaccaggcagcauuag...........................................................    2103    0   T63
.............................caucuuaccaggUagcauuag...........................................................    2       1   T63
.............................cGucuuaccaggcagcauuag...........................................................    1       1   T63
.............................caucuuaccaggcagcUuuag...........................................................    1       1   T63
.............................caucuuaccaggcagcauuaA...........................................................    16      1   T63
.............................caucuuaccaUgcagcauuag...........................................................    1       1   T63
.............................caucuuaccaggcCgcauuag...........................................................    1       1   T63
.............................caucuuaccaggcagcauuaU...........................................................    15      1   T63
.............................caucuuaccagAcagcauuag...........................................................    1       1   T63
.............................caucuuaccagCcagcauuaga..........................................................    1       1   T63
.............................caucuuaccaggcagcauuaAa..........................................................    2       1   T63
.............................caucuuaccaggUagcauuaga..........................................................    2       1   T63
.............................caucuuaccaggcagcauuagG..........................................................    5       1   T63
.............................caucuuaccaggcaAcauuaga..........................................................    2       1   T63
.............................caucuuaccaggcagcauuagU..........................................................    66      1   T63
.............................caucuuaccaUgcagcauuaga..........................................................    1       1   T63
.............................caucuuaccagAcagcauuaga..........................................................    1       1   T63
.............................caucuuaccaggcaCcauuaga..........................................................    1       1   T63
.............................caucuuaccGggcagcauuaga..........................................................    27      1   T63
.............................caucuuaccaggcagcauuaga..........................................................    1250    0   T63
.............................caucuuaccaggcagcauuagaA.........................................................    37      1   T63
.............................caucuuaccaggcagcauuagaC.........................................................    1       1   T63
.............................caucuuaccaggcagcauuagUu.........................................................    8       1   T63
.............................caucuuaccaggcagcauuagau.........................................................    8       0   T63
.............................caucuuaccaggcagcauuagauc........................................................    1       0   T63
.............................caucuuaccaggcagcauuagauU........................................................    1       1   T63
.............................caucuuaccaggcagcauuagaAc........................................................    5       1   T63
.............................caucuuaccaggcagcauuagaucc.......................................................    1       0   T63
.............................caucuuaccaggcagcauuagauccga.....................................................    1       0   T63
..............................aucuuaccaggcagcauu..............................................................    1       0   T63
..............................aucuuaccaggcagcauuag...........................................................    3       0   T63
..............................aucuuaccaggcagcauuaga..........................................................    3       0   T63
..............................aucuuaccaggcagcauuagau.........................................................    2       0   T63
..............................aucuuaccaggcagcauuagaA.........................................................    1       1   T63
..............................aucuuaccaggcagcauuagauc........................................................    2       0   T63
..............................aucuuaccaggcagcauuagauA........................................................    1       1   T63
...............................ucuuaccaggcagcauuaga..........................................................    1       0   T63
...............................ucuuaccaggcagcauuagau.........................................................    3       0   T63
...............................ucuuaccaggcagcauuagauc........................................................    61      0   T63
...............................ucuuaccaggcagcauuagauU........................................................    1       1   T63
...............................ucuuaccaggcagcauuagauA........................................................    3       1   T63
...............................ucuuaccaggcagcauuagaucA.......................................................    1       1   T63
...............................ucuuaccaggcagcauuagaucU.......................................................    1       1   T63
................................cuuaccaggcagcauuag...........................................................    4       0   T63
.................................uuaccaggcagcauuaga..........................................................    1       0   T63
...................................................uccgaggauuuaaaacuA........................................    2       1   T63
...................................................uccgaggauuuaaaacuc........................................    4       0   T63
.................................................................acuUuaauacugucagguaaugaug...................    1       1   T63
..................................................................cuUuaauacugucagguaaugau....................    3       1   T63
..................................................................cuUuaauacugucagguaaugaug...................    3       1   T63
..................................................................cuUuaauacugucagguaaugaugu..................    8       1   T63
..................................................................cuUuaauacugucagguaaugauguc.................    3       1   T63
...................................................................uUuaauacugucagguaauga.....................    1       1   T63
...................................................................ucuaauacugucagguaauga.....................    3       0   T63
...................................................................uUuaauacugucagguaaugau....................    2       1   T63
...................................................................uUuaauacugucagguaaugaug...................    11      1   T63
...................................................................uUuaauacugucagguaaugaugu..................    20      1   T63
...................................................................uAuaauacugucagguaaugaugu..................    1       1   T63
...................................................................uUuaauacugucagguaaugauguc.................    16      1   T63
...................................................................uUuaauacugucagguaaugauguca................    1       1   T63
....................................................................cuaauacugucagguaaugau....................    6       0   T63
....................................................................Guaauacugucagguaaugau....................    1       1   T63
....................................................................Uuaauacugucagguaaugau....................    1       1   T63
....................................................................cuaauacugucagguaaugaug...................    4       0   T63
....................................................................cuaauacugucagguaaugauU...................    1       1   T63
```

```
                              Star                                    Mature
uaacaaaauuauauaauaaucuuuguagucaucuuaccaggcagcauuagauccgaggauuuaaaacucuaauacugucagguaaugaugucagcagaguuucguacaa
....................................................................Uuaauacugucagguaaugaug...................    12      1    T63
....................................................................cuaauacugucagguaaugaCgu..................    1       1    T63
....................................................................Guaauacugucagguaaugaugu..................    2       1    T63
....................................................................cuaauacugucagguaaugaugu..................    13      0    T63
....................................................................Auaauacugucagguaaugaugu..................    2       1    T63
....................................................................Uuaauacugucagguaaugaugu..................    62      1    T63
....................................................................Guaauacugucagguaaugauguc.................    1       1    T63
....................................................................cuaauacugucagguaaugauguU.................    3       1    T63
....................................................................Auaauacugucagguaaugauguc.................    13      1    T63
....................................................................Uuaauacugucagguaaugauguc.................    173     1    T63
....................................................................cuaauacugucagguaaugauguc.................    6       0    T63
....................................................................Uuaauacugucagguaaugauguca................    2       1    T63
....................................................................cuaauacugucagguaaugaugucU................    3       1    T63
.....................................................................uaauacugucagguaauA......................    2       1    T63
.....................................................................uaauacugucagguaaug......................    72      0    T63
.....................................................................uaauacugucagguaauU......................    2       1    T63
.....................................................................uaauacAgucagguaauga.....................    1       1    T63
.....................................................................uaauacugucagguaauAa.....................    2       1    T63
.....................................................................uaauacugucagguaaugU.....................    14      1    T63
.....................................................................uaauacugucagguaauga.....................    168     0    T63
.....................................................................uaauacuAucagguaaugau....................    1       1    T63
.....................................................................uaauacugucagguUaugau....................    12      1    T63
.....................................................................uaauacugucagguaaugCu....................    1       1    T63
.....................................................................uaauacugucagguaaugUu....................    11      1    T63
.....................................................................uaauacugucagguaGugau....................    1       1    T63
.....................................................................Gaauacugucagguaaugau....................    1       1    T63
.....................................................................uaauaUugucagguaaugau....................    2       1    T63
.....................................................................uaauacugucagguaaugau....................    8372    0    T63
.....................................................................uaauacugucUgguaaugau....................    2       1    T63
.....................................................................uaauacugucaggAaaugau....................    9       1    T63
.....................................................................uaauacuguUagguaaugau....................    3       1    T63
.....................................................................uGauacugucagguaaugau....................    1       1    T63
.....................................................................uaauacuguAagguaaugau....................    1       1    T63
.....................................................................uaauacAgucagguaaugau....................    1       1    T63
.....................................................................uaGuacugucagguaaugau....................    1       1    T63
.....................................................................uaauacuguGagguaaugau....................    5       1    T63
.....................................................................uaauacugucaCguaaugau....................    2       1    T63
.....................................................................uaauacugAcagguaaugau....................    8       1    T63
.....................................................................uaaAacugucagguaaugau....................    7       1    T63
.....................................................................uaauacugucGgguaaugau....................    3       1    T63
.....................................................................uaauaAugucagguaaugau....................    1       1    T63
.....................................................................uaauacugucagguaaAgau....................    10      1    T63
.....................................................................Aaauacugucagguaaugau....................    6       1    T63
.....................................................................uaauaGugucagguaaugau....................    7       1    T63
.....................................................................uaauacuUucagguaaugau....................    2       1    T63
.....................................................................uaUuacugucagguaaugau....................    3       1    T63
.....................................................................uaauacugucagAuaaugau....................    1       1    T63
.....................................................................uaauacugucagguaauUau....................    1       1    T63
.....................................................................uaauacugucagguaaugaG....................    1       1    T63
.....................................................................uaauacugucagguaauCau....................    1       1    T63
.....................................................................uUauacugucagguaaugau....................    2       1    T63
.....................................................................uaauacugucagguaaugaC....................    2       1    T63
.....................................................................uaauacugucagguaauAau....................    3       1    T63
.....................................................................uaauacugucaggGaaugau....................    3       1    T63
.....................................................................uaauacugucagguaaCgau....................    1       1    T63
.....................................................................uaauacugucagguaaugaA....................    32      1    T63
.....................................................................uaauacugucagguaaugGu....................    3       1    T63
.....................................................................uaauacugucagguUaugaug...................    27      1    T63
.....................................................................uaGuacugucagguaaugaug...................    4       1    T63
.....................................................................uUauacugucagguaaugaug...................    4       1    T63
.....................................................................uaauacugucagguaaGgaug...................    4       1    T63
.....................................................................uaauacugucagguaGugaug...................    2       1    T63
.....................................................................uaauacugucagguaaugaug...................    46584   0    T63
.....................................................................Caauacugucagguaaugaug...................    8       1    T63
.....................................................................uaauacugucagguaaAgaug...................    62      1    T63
.....................................................................uaauacuCucagguaaugaug...................    2       1    T63
.....................................................................uaauacuguGagguaaugaug...................    13      1    T63
.....................................................................uaUuacugucagguaaugaug...................    17      1    T63
.....................................................................uaauacugucagguaaugUug...................    63      1    T63
.....................................................................uaauacugCcagguaaugaug...................    2       1    T63
```

```
                               Star                                    Mature
uaacaaaaauuauauaauaaucuuuguagucaucuuaccaggcagcauuagauccgaggauuuaaaacucuaauacugucagguaaugaugucagcagaguuucguacaa
......................................................................uaauGcugucagguaaugaug...................  1       1   T63
......................................................................uaauUcugucagguaaugaug...................  3       1   T63
......................................................................uaauacuAucagguaaugaug...................  2       1   T63
......................................................................uaauacugGcagguaaugaug...................  2       1   T63
......................................................................uaauacugucagCuaaugaug...................  4       1   T63
......................................................................uaauacugucagguaUugaug...................  5       1   T63
......................................................................uaCuacugucagguaaugaug...................  1       1   T63
......................................................................uaaCacugucagguaaugaug...................  9       1   T63
......................................................................uaauacuUucagguaaugaug...................  3       1   T63
......................................................................uaauacugucagguGaugaug...................  5       1   T63
......................................................................uaauacugucGgguaaugaug...................  11      1   T63
......................................................................uaauacugucagguaauCaug...................  3       1   T63
......................................................................uaauacugucagguaaugauA...................  2713    1   T63
......................................................................uaauacugucagguaaugaAg...................  4       1   T63
......................................................................uaauacugucagguaaugCug...................  2       1   T63
......................................................................uaauacugucagguaaugGug...................  12      1   T63
......................................................................uaauacugucCgguaaugaug...................  1       1   T63
......................................................................uaauaAugucagguaaugaug...................  5       1   T63
......................................................................uaauacugucaUguaaugaug...................  12      1   T63
......................................................................uaauacugucagguCaugaug...................  2       1   T63
......................................................................uaauacugucaggAaaugaug...................  74      1   T63
......................................................................uGauacugucagguaaugaug...................  3       1   T63
......................................................................uaauacugucagguaauAaug...................  11      1   T63
......................................................................uaauacugucagguaaugaGg...................  13      1   T63
......................................................................Gaauacugucagguaaugaug...................  2       1   T63
......................................................................uaauacugucagguaaugaCg...................  6       1   T63
......................................................................uaauacugucagUuaaugaug...................  9       1   T63
......................................................................uaauacCgucagguaaugaug...................  4       1   T63
......................................................................uaauacugucaggCaaugaug...................  3       1   T63
......................................................................uaaAacugucagguaaugaug...................  19      1   T63
......................................................................uaauacuguUagguaaugaug...................  6       1   T63
......................................................................uaauacugucagguaaCgaug...................  4       1   T63
......................................................................uaauacugucagAuaaugaug...................  6       1   T63
......................................................................uaauaGugucagguaaugaug...................  20      1   T63
......................................................................uaauacugucagguaauUaug...................  8       1   T63
......................................................................Aaauacugucagguaaugaug...................  36      1   T63
......................................................................uaauacugAcagguaaugaug...................  55      1   T63
......................................................................uaauacugucaAguaaugaug...................  11      1   T63
......................................................................uaaGacugucagguaaugaug...................  3       1   T63
......................................................................uaauacugucaCguaaugaug...................  5       1   T63
......................................................................uaauacuguAagguaaugaug...................  1       1   T63
......................................................................uaauacAgucagguaaugaug...................  14      1   T63
......................................................................uaauacugucagguaaugauC...................  87      1   T63
......................................................................uCauacugucagguaaugaug...................  7       1   T63
......................................................................uaauacugucagguaaugauU...................  393     1   T63
......................................................................uaauaUugucagguaaugaug...................  15      1   T63
......................................................................uaauacugucaggGaaugaug...................  5       1   T63
......................................................................uaauacugucUgguaaugaug...................  7       1   T63
......................................................................uaauacugucagguaCugaug...................  2       1   T63
......................................................................uaauacugucaggCaaugaugu..................  23      1   T63
......................................................................uaauaGugucagguaaugaugu..................  56      1   T63
......................................................................uaauacugucagguGaugaugu..................  7       1   T63
......................................................................uaauacugucagguaaGgaugu..................  20      1   T63
......................................................................uaauacugucaggGaaugaugu..................  27      1   T63
......................................................................uaauacugucCgguaaugaugu..................  3       1   T63
......................................................................Gaauacugucagguaaugaugu..................  12      1   T63
......................................................................uaauacugucagguaaCgaugu..................  21      1   T63
......................................................................uaauacuguAagguaaugaugu..................  5       1   T63
......................................................................uaauacugucaggAaaugaugu..................  186     1   T63
......................................................................uaauacugucagguaaugaugu..................  189449  0   T63
......................................................................uaauacugucagguaaugUugu..................  128     1   T63
......................................................................uaauacuguUagguaaugaugu..................  50      1   T63
......................................................................uaauacugGcagguaaugaugu..................  10      1   T63
......................................................................uaauacugucagguaaugCugu..................  12      1   T63
......................................................................uaauacugucagCuaaugaugu..................  14      1   T63
......................................................................uaauacugucagguaaugauAu..................  18      1   T63
......................................................................uUauacugucagguaaugaugu..................  20      1   T63
......................................................................uaauacugAcagguaaugaugu..................  174     1   T63
......................................................................uaauacugucagguaaugaugC..................  142     1   T63
......................................................................uaauacugucagguaauAaugu..................  40      1   T63
```

```
                              Star                                    Mature
uaacaaaaauuauauaauaaucuuuguagucaucuuaccaggcagcauuagauccgaggauuuaaaacucuaauacugucagguaaugaugucagcagaguuucguacaa
......................................................................uaauacugucGgguaaugaugu..................   62      1   T63
......................................................................uaauacugucagguUaugaugu..................   118     1   T63
......................................................................uaauacugucagAuaaugaugu..................   47      1   T63
......................................................................uaaCacugucagguaaugaugu..................   15      1   T63
......................................................................uaauacugucagguaaugaGgu..................   20      1   T63
......................................................................uaauacuAucagguaaugaugu..................   9       1   T63
......................................................................uaauacuUucagguaaugaugu..................   14      1   T63
......................................................................uaauaUugucagguaaugaugu..................   41      1   T63
......................................................................uaauacuCucagguaaugaugu..................   7       1   T63
......................................................................uaauacugucagguaGugaugu..................   8       1   T63
......................................................................uaauacugucagguaauCaugu..................   26      1   T63
......................................................................uaUuacugucagguaaugaugu..................   80      1   T63
......................................................................uaauaAugucagguaaugaugu..................   18      1   T63
......................................................................uaaGacugucagguaaugaugu..................   5       1   T63
......................................................................Caauacugucagguaaugaugu..................   29      1   T63
......................................................................uaauacGgucagguaaugaugu..................   3       1   T63
......................................................................uaauGcugucagguaaugaugu..................   13      1   T63
......................................................................uaauacugucagguaauUaugu..................   37      1   T63
......................................................................uCauacugucagguaaugaugu..................   21      1   T63
......................................................................uaauacCgucagguaaugaugu..................   8       1   T63
......................................................................uaauacugucagguaaugaAgu..................   33      1   T63
......................................................................uaauacugucagguaaugGugu..................   39      1   T63
......................................................................uaauacugucagguaaugauCu..................   11      1   T63
......................................................................uaauacugucagguaCugaugu..................   3       1   T63
......................................................................Aaauacugucagguaaugaugu..................   110     1   T63
......................................................................uaauacugucagguaaugaugG..................   61      1   T63
......................................................................uaauacugCcagguaaugaugu..................   11      1   T63
......................................................................uaauUcugucagguaaugaugu..................   22      1   T63
......................................................................uaauacugucaCguaaugaugu..................   18      1   T63
......................................................................uaauacugucUgguaaugaugu..................   7       1   T63
......................................................................uGauacugucagguaaugaugu..................   26      1   T63
......................................................................uaauacugucagUuaaugaugu..................   18      1   T63
......................................................................uaauacAgucagguaaugaugu..................   49      1   T63
......................................................................uaauacugucaUguaaugaugu..................   21      1   T63
......................................................................uaaAacugucagguaaugaugu..................   70      1   T63
......................................................................uaauacuguGagguaaugaugu..................   33      1   T63
......................................................................uaauacugucaAguaaugaugu..................   27      1   T63
......................................................................uaauCcugucagguaaugaugu..................   2       1   T63
......................................................................uaCuacugucagguaaugaugu..................   1       1   T63
......................................................................uaauacugucagguaaugaugA..................   599     1   T63
......................................................................uaauacugucagguaaugauUu..................   44      1   T63
......................................................................uaauacugucagguaaAgaugu..................   321     1   T63
......................................................................uaGuacugucagguaaugaugu..................   5       1   T63
......................................................................uaauacugucagguaUugaugu..................   21      1   T63
......................................................................uaauacugucagguCaugaugu..................   6       1   T63
......................................................................uaauacugucagguaaugaCgu..................   7       1   T63
......................................................................uaauacugucagguaGugauguc.................   21      1   T63
......................................................................uaauacugucagguaaugauCuc.................   77      1   T63
......................................................................uaauacugucaCguaaugauguc.................   38      1   T63
......................................................................uaauacugGcagguaaugauguc.................   25      1   T63
......................................................................uaauacugucagguaaCgauguc.................   63      1   T63
......................................................................uaauacugucagguaaugaGguc.................   60      1   T63
......................................................................uaauacugucGgguaaugauguc.................   137     1   T63
......................................................................uaauacugucagguaaugaugCc.................   374     1   T63
......................................................................uaauacugucagguaaugauguA.................   453     1   T63
......................................................................uaauacugucagguaaugGuguc.................   150     1   T63
......................................................................uaauGcugucagguaaugauguc.................   50      1   T63
......................................................................uaauacugucCgguaaugauguc.................   6       1   T63
......................................................................uaCuacugucagguaaugauguc.................   26      1   T63
......................................................................uaGuacugucagguaaugauguc.................   63      1   T63
......................................................................uaaGacugucagguaaugauguc.................   47      1   T63
......................................................................uaauacugucagguaCugauguc.................   22      1   T63
......................................................................uaauacuguAagguaaugauguc.................   16      1   T63
......................................................................uaauacugucagguaaugauguG.................   113     1   T63
......................................................................uaauCcugucagguaaugauguc.................   6       1   T63
......................................................................uaauacugucagguaaAgauguc.................   2129    1   T63
......................................................................uaauacugucagguCaugauguc.................   19      1   T63
......................................................................uaauaUugucagguaaugauguc.................   162     1   T63
......................................................................uaauacugucagguaaugUuguc.................   726     1   T63
......................................................................uaauacugucagguaaugauguc.................   694776  0   T63
```

Star Mature

```
uaacaaaauuauauaauaaucuuuguagucaucuuaccaggcagcauuagauccgaggauuuaaaacucuaauacugucagguaaugaugucagcagaguuucguacaa
.....................................................................uaauacugucagguaaugaCguc.................   27     1   T63
.....................................................................uaauacugucagguaaugaAguc.................   103    1   T63
.....................................................................uGauacugucagguaaugauguc.................   100    1   T63
.....................................................................uaauacugucagguaaugauAuc.................   56     1   T63
.....................................................................uaauacuguGagguaaugauguc.................   75     1   T63
.....................................................................uaaAacugucagguaaugauguc.................   231    1   T63
.....................................................................uaauacugucaggGaaugauguc.................   100    1   T63
.....................................................................uaauacugucagguaauUauguc.................   171    1   T63
.....................................................................uaauacugucagguaaugauUuc.................   130    1   T63
.....................................................................uaauacuguUagguaaugauguc.................   162    1   T63
.....................................................................uaauacugucagUuaaugauguc.................   66     1   T63
.....................................................................uaauacugucaggAaaugauguc.................   732    1   T63
.....................................................................Aaauacugucagguaaugauguc.................   394    1   T63
.....................................................................uaauacugucagAuaaugauguc.................   97     1   T63
.....................................................................uaauacugucagguGaugauguc.................   55     1   T63
.....................................................................uaauacugucagguaauAauguc.................   110    1   T63
.....................................................................uaauacugucagguaaugCuguc.................   38     1   T63
.....................................................................uaauacugucagguaaGgauguc.................   67     1   T63
.....................................................................uaaCacugucagguaaugauguc.................   57     1   T63
.....................................................................uCauacugucagguaaugauguc.................   67     1   T63
.....................................................................uaauacugCcagguaaugauguc.................   57     1   T63
.....................................................................uaauacGgucagguaaugauguc.................   16     1   T63
.....................................................................uaauacugAcagguaaugauguc.................   575    1   T63
.....................................................................uaauUcugucagguaaugauguc.................   52     1   T63
.....................................................................uaauacugucagguaaugauguU.................   4221   1   T63
.....................................................................uaauacCgucagguaaugauguc.................   28     1   T63
.....................................................................uaauacuCucagguaaugauguc.................   46     1   T63
.....................................................................uaauacugucagguaaugaugGc.................   142    1   T63
.....................................................................Caauacugucagguaaugauguc.................   55     1   T63
.....................................................................uaauacuAucagguaaugauguc.................   43     1   T63
.....................................................................uaauacugucUgguaaugauguc.................   50     1   T63
.....................................................................uUauacugucagguaaugauguc.................   111    1   T63
.....................................................................uaauacugucagCuaaugauguc.................   43     1   T63
.....................................................................uaauacugucaUguaaugauguc.................   53     1   T63
.....................................................................uaauaGugucagguaaugauguc.................   137    1   T63
.....................................................................uaauaAugucagguaaugauguc.................   69     1   T63
.....................................................................uaauacugucagguaauCauguc.................   121    1   T63
.....................................................................uaauacugucagguaaugaugAc.................   927    1   T63
.....................................................................uaUuacugucagguaaugauguc.................   406    1   T63
.....................................................................Gaauacugucagguaaugauguc.................   51     1   T63
.....................................................................uaauacuUucagguaaugauguc.................   32     1   T63
.....................................................................uaauacugucaggCaaugauguc.................   61     1   T63
.....................................................................uaauacugucagguaUugauguc.................   110    1   T63
.....................................................................uaauacugucaAguaaugauguc.................   68     1   T63
.....................................................................uaauacugucagguUaugauguc.................   423    1   T63
.....................................................................uaauacAgucagguaaugauguc.................   144    1   T63
.....................................................................uaauacugucagguUaugauguca................   5      1   T63
.....................................................................uaauacugucagguaaugauguca................   4834   0   T63
.....................................................................uaauacCgucagguaaugauguca................   3      1   T63
.....................................................................uaauacugucagguGaugauguca................   1      1   T63
.....................................................................uaauacugAcagguaaugauguca................   1      1   T63
.....................................................................uaauacugucagguaaugaugucC................   654    1   T63
.....................................................................uaauacugucagguaaugauUuca................   4      1   T63
.....................................................................uaauacugucagguaaAgauguca................   24     1   T63
.....................................................................uaauacugucagguaaugaugCca................   3      1   T63
.....................................................................uaauacugucagguaaugUuguca................   7      1   T63
.....................................................................uaauacugucaggGaaugauguca................   1      1   T63
.....................................................................uaauacugucagAuaaugauguca................   1      1   T63
.....................................................................uaauacugucagguaaugauguAa................   1      1   T63
.....................................................................uaauacuguUagguaaugauguca................   3      1   T63
.....................................................................Gaauacugucagguaaugauguca................   1      1   T63
.....................................................................uaauacAgucagguaaugauguca................   2      1   T63
.....................................................................uaauacugucagguaaugauguUa................   18     1   T63
.....................................................................uaauacugucagguaaugauguGa................   3      1   T63
.....................................................................uaauacugucagguaauCauguca................   2      1   T63
.....................................................................uaauacugucagguaaCgauguca................   3      1   T63
.....................................................................uGauacugucagguaaugauguca................   2      1   T63
.....................................................................uaauaGugucagguaaugauguca................   1      1   T63
.....................................................................uaauacugucagguaaugaugGca................   2      1   T63
.....................................................................uaauacugucagguaaugauCuca................   2      1   T63
```

Star Mature

```
uaacaaaaauuauauaauaaucuuuguagucaucuuaccaggcagcauuagauccgaggauuuaaaacucuaauacugucagguaaugaugucagcagaguuucguacaa
......................................................................uaauacugucagguaaugaugAca................	9	1	T63
......................................................................uaauacugucagguaauAauguca................	1	1	T63
......................................................................uaauacugucagguaaugaGguca................	1	1	T63
......................................................................uaauacugucagguaaugaugucU................	33134	1	T63
......................................................................uaauacGgucagguaaugauguca................	1	1	T63
......................................................................uaauacuCucagguaaugauguca................	1	1	T63
......................................................................uaUuacugucagguaaugauguca................	2	1	T63
......................................................................uaauacugucagguaaugaugucG................	606	1	T63
......................................................................uaauGcugucagguaaugauguca................	1	1	T63
......................................................................Aaauacugucagguaaugauguca................	1	1	T63
......................................................................uaauacugucagUuaaugauguca................	2	1	T63
......................................................................uaauacugucaggAaaugauguca................	7	1	T63
......................................................................uaauacugucagguaauUauguca................	2	1	T63
......................................................................uaauacugucagguaaugaugucaA...............	35	1	T63
......................................................................uaauacugucagguaaugaugucag...............	1	0	T63
......................................................................uaauacugucagguaaugaugucaC...............	16	1	T63
......................................................................uaauacugucagguaaugaugucaU...............	89	1	T63
......................................................................uaauacugucagguaaugaugucUg...............	2	1	T63
......................................................................uaauacugucagguaaugaugucGg...............	1	1	T63
......................................................................uaauacugucagguaaugaugucCg...............	1	1	T63
......................................................................uaauacugucagguaaugauguGag...............	1	1	T63
......................................................................uaauacugucagguaaugaugucUgc..............	1	1	T63
......................................................................uaauacugucagguaaugauguGagc..............	1	1	T63
......................................................................uaauacugucagguaaugaugucaAc..............	1	1	T63
......................................................................uaauacugucagguaaugaugucagU..............	1	1	T63
.......................................................................aauacugucagguaaugau....................	16	0	T63
.......................................................................aauacugucagguaaugauA...................	1	1	T63
.......................................................................aauacugucagguaaugaug...................	93	0	T63
.......................................................................aauacugucCgguaaugaug...................	1	1	T63
.......................................................................aauacugucaggGaaugaugu..................	1	1	T63
.......................................................................aauacugucagguaaAgaugu..................	1	1	T63
.......................................................................Uauacugucagguaaugaugu..................	3	1	T63
.......................................................................aauacuguAagguaaugaugu..................	2	1	T63
.......................................................................aauacugucagguaUugaugu..................	2	1	T63
.......................................................................aauacugucagguaaugaugu..................	367	0	T63
.......................................................................aauacugucagAuaaugaugu..................	1	1	T63
.......................................................................aCuacugucagguaaugaugu..................	1	1	T63
.......................................................................aauacugucagguaaugaugA..................	1	1	T63
.......................................................................aauacugucagguaaugauguc.................	3155	0	T63
.......................................................................aauacugucagguaaAgauguc.................	6	1	T63
.......................................................................aauacugucagguaUugauguc.................	2	1	T63
.......................................................................aauacuguAagguaaugauguc.................	4	1	T63
.......................................................................aauacugucagguaauUauguc.................	2	1	T63
.......................................................................aaAacugucagguaaugauguc.................	1	1	T63
.......................................................................aauacugucagguaCugauguc.................	1	1	T63
.......................................................................aauacugucagguaaugaGguc.................	1	1	T63
.......................................................................aauacugCcagguaaugauguc.................	1	1	T63
.......................................................................Uauacugucagguaaugauguc.................	10	1	T63
.......................................................................aGuacugucagguaaugauguc.................	2	1	T63
.......................................................................aUuacugucagguaaugauguc.................	3	1	T63
.......................................................................aauacugAcagguaaugauguc.................	4	1	T63
.......................................................................aauacugucagguaauAauguc.................	1	1	T63
.......................................................................aauacugucaggAaaugauguc.................	4	1	T63
.......................................................................aauacugucaCguaaugauguc.................	1	1	T63
.......................................................................aauacugucagguaaugUuguc.................	1	1	T63
.......................................................................aauacugucagguaaugauguU.................	19	1	T63
.......................................................................aauaUugucagguaaugauguc.................	1	1	T63
.......................................................................aauacugucagAuaaugauguc.................	3	1	T63
.......................................................................aauacugucagguaaugaugAc.................	2	1	T63
.......................................................................aauacugucagguaaugaugCc.................	3	1	T63
.......................................................................aauacugucagguaaugGuguc.................	1	1	T63
.......................................................................aauacugucagguaaugauguG.................	1	1	T63
.......................................................................aauacugucagguaaugauCuc.................	4	1	T63
.......................................................................aauacugucagguaaugauguA.................	1	1	T63
.......................................................................aauacugucCgguaaugauguc.................	1	1	T63
.......................................................................aauacuguUagguaaugauguc.................	3	1	T63
.......................................................................aauacugucagguaaugauguca................	41	0	T63
.......................................................................aauacugucagguaaAgauguca................	1	1	T63
.......................................................................aauacugucagguaaugaugucU................	605	1	T63
.......................................................................aauacugucagguaaugaugucC................	10	1	T63
```

```
                              Star                                  Mature
uaacaaaaauuauauaauaaucuuuguagucaucuuaccaggcagcauuagauccgaggauuuaaaacucuaauacugucagguaaugaugucagcagaguuucguacaa
.......................................................................aauacugucGgguaaugauguca................   1   1   T63
.......................................................................aauacugucagguaaugaugGca................   1   1   T63
.......................................................................aauacugucagUuaaugauguca................   1   1   T63
.......................................................................aauacugucagguaaugaugucG................   3   1   T63
.......................................................................aauacugucagguaaugaugucaU...............   1   1   T63
.......................................................................aauacugucagguaaugaugucaC...............   1   1   T63
.......................................................................aauacugucagguaaugaugucaA...............   1   1   T63
........................................................................auacugucagguaaugau....................   3   0   T63
........................................................................auacugucagguaaugaug...................   1   0   T63
........................................................................auacugucagguaaugaugu..................   9   0   T63
........................................................................Uuacugucagguaaugaugu..................   1   1   T63
........................................................................aCacugucagguaaugaugu..................   2   1   T63
........................................................................aAacugucagguaaugaugu..................   1   1   T63
........................................................................auacugucagguaaugauguc.................   49  0   T63
........................................................................aGacugucagguaaugauguc.................   1   1   T63
........................................................................Uuacugucagguaaugauguc.................   8   1   T63
........................................................................auacugucagguaaugaugucU................   5   1   T63
.........................................................................uacugucagguaaugauA...................   1   1   T63
.........................................................................uaAugucagguaaugaug...................   1   1   T63
.........................................................................uacugucagguaaugaugu..................   6   0   T63
.........................................................................uacugucagguaaugauguc.................   18  0   T63
.........................................................................uacugucagguaaugaugucU................   2   1   T63
..........................................................................acugucagguaaugaugu..................   15  0   T63
..........................................................................acugucagguaaugauguc.................   11  0   T63
..........................................................................acugucagguaaugaugucG................   1   1   T63
...........................................................................cugucagguaaugauguU.................   1   1   T63
...........................................................................cugucagguaaugauguc.................   50  0   T63
...........................................................................cugucaggAaaugauguc.................   1   1   T63
...........................................................................cugucagguaaugaugucU................   12  1   T63

..........uauauaauaaucuuuguagu................................................................................   1   0   MOL
...........................Ggucaucuuaccaggcagcauuag...........................................................   1   1   MOL
...........................agGcaucuuaccaggcagcauuaga..........................................................   3   1   MOL
............................Uucaucuuaccaggcagcauuaga..........................................................   2   1   MOL
.............................ucaucuuaccaggcagcau..............................................................   2   0   MOL
.............................ucaucuuaccaggcagcauu.............................................................   139 0   MOL
.............................Acaucuuaccaggcagcauu.............................................................   3   1   MOL
.............................ucaucuuaAcaggcagcauu.............................................................   1   1   MOL
.............................ucaucuAaccaggcagcauu.............................................................   1   1   MOL
.............................ucaucuuaccaggcagcauA.............................................................   1   1   MOL
.............................Ccaucuuaccaggcagcauu.............................................................   1   1   MOL
.............................ucaucuuaccaggcagcauuU............................................................   4   1   MOL
.............................ucaucuuacUaggcagcauua............................................................   1   1   MOL
.............................ucaucuuaccaggcagcauua............................................................   178 0   MOL
.............................ucaucuuaccaggUagcauua............................................................   1   1   MOL
.............................ucaucuuaccaggcagcauuC............................................................   1   1   MOL
.............................ucaucuuaccaggcagcauuaU...........................................................   1   1   MOL
.............................ucaucuuaccaggcagcauuaA...........................................................   3   1   MOL
.............................ucaucuuaccaggcagcauuag...........................................................   158 0   MOL
.............................Ccaucuuaccaggcagcauuaga..........................................................   1   1   MOL
.............................ucaucuuaccaggcagcauuagU..........................................................   5   1   MOL
.............................ucaucuuaccaggcagcauuaga..........................................................   39  0   MOL
.............................ucaucuuaccaggcagcauuagaA.........................................................   3   1   MOL
.............................ucaucuuaccaggcagcauuagauc........................................................   1   0   MOL
..............................caucuuaccGggcagcau..............................................................   1   1   MOL
..............................caucuuaccaggcagcau..............................................................   8   0   MOL
..............................Uaucuuaccaggcagcauu.............................................................   1   1   MOL
..............................caucuuaccagAcagcauu.............................................................   1   1   MOL
..............................caucuuaccaggcagcauu.............................................................   433 0   MOL
..............................cauUuuaccaggcagcauu.............................................................   1   1   MOL
..............................caucuuaccaggcagcauA.............................................................   1   1   MOL
..............................cUucuuaccaggcagcauu.............................................................   1   1   MOL
..............................caucuuUccaggcagcauua............................................................   1   1   MOL
..............................caucuuaccagUcagcauua............................................................   1   1   MOL
..............................caucuuaccaggcagcaAua............................................................   1   1   MOL
..............................caucuuaccaAgcagcauua............................................................   1   1   MOL
..............................caucuuaccagAcagcauua............................................................   2   1   MOL
..............................Uaucuuaccaggcagcauua............................................................   1   1   MOL
..............................cauUuuaccaggcagcauua............................................................   1   1   MOL
..............................caucuuaccGggcagcauua............................................................   1   1   MOL
```

```
                              Star                                    Mature
uaacaaaaauuauauaauaaucuuuguagucaucuuaccaggcagcauuagauccgaggauuuaaaacucuaauacugucagguaaugaugucagcagaguuucguacaa
..............................caucuuaccaggcagcauua............................................................    896     0    MOL
..............................caucuuaccaggcagcauuU............................................................    28      1    MOL
..............................caucuuaccaggcaCcauua............................................................    1       1    MOL
..............................caucuuaccaggcagGauuag...........................................................    1       1    MOL
..............................caucuuaccagAcagcauuag...........................................................    1       1    MOL
..............................caucuuaccaggcagcauuag...........................................................    4543    0    MOL
..............................caucuuaccaggUagcauuag...........................................................    2       1    MOL
..............................Uaucuuaccaggcagcauuag...........................................................    3       1    MOL
..............................caucuuaccaggcUgcauuag...........................................................    2       1    MOL
..............................cauUuuaccaggcagcauuag...........................................................    1       1    MOL
..............................caucuuaccaggcagAauuag...........................................................    1       1    MOL
..............................caucuuaccaggcagcauuaU...........................................................    25      1    MOL
..............................caucuuaccaCgcagcauuag...........................................................    1       1    MOL
..............................caucuAaccaggcagcauuag...........................................................    1       1    MOL
..............................caucuuaccagCcagcauuag...........................................................    1       1    MOL
..............................caucuuaccaggcagcaCuag...........................................................    1       1    MOL
..............................cGucuuaccaggcagcauuag...........................................................    2       1    MOL
..............................cauAuuaccaggcagcauuag...........................................................    1       1    MOL
..............................caucuuaccaggcagcauAag...........................................................    2       1    MOL
..............................caucuuaccaggcaAcauuag...........................................................    2       1    MOL
..............................caucuuaccaggcagcauuaC...........................................................    1       1    MOL
..............................caucuuaccaggcaCcauuag...........................................................    1       1    MOL
..............................caucuuaccGggcagcauuag...........................................................    5       1    MOL
..............................caucuuaccaggcagcauuaA...........................................................    60      1    MOL
..............................caucuCaccaggcagcauuag...........................................................    1       1    MOL
..............................caucuuacAaggcagcauuag...........................................................    2       1    MOL
..............................caucuuaccUggcagcauuag...........................................................    2       1    MOL
..............................caucuuaccCggcagcauuag...........................................................    2       1    MOL
..............................caucuuaccagUcagcauuag...........................................................    1       1    MOL
..............................caucuuaccaggcagcauCag...........................................................    1       1    MOL
..............................cUucuuaccaggcagcauuag...........................................................    4       1    MOL
..............................caucuuaccaggcagcGuuag...........................................................    1       1    MOL
..............................caucuuaccaggcagUauuag...........................................................    4       1    MOL
..............................caucuuaccaggcagcaAuag...........................................................    2       1    MOL
..............................caucuCaccaggcagcauuaga..........................................................    1       1    MOL
..............................caucuuaccaggcagUauuaga..........................................................    7       1    MOL
..............................caucuuaccagAcagcauuaga..........................................................    5       1    MOL
..............................caucuuaccaggcagAauuaga..........................................................    4       1    MOL
..............................cauUuuaccaggcagcauuaga..........................................................    3       1    MOL
..............................caucuuacAaggcagcauuaga..........................................................    3       1    MOL
..............................caucuuaccaggcagcauAaga..........................................................    1       1    MOL
..............................caucuuaccaggcagcGuuaga..........................................................    1       1    MOL
..............................caucuuaccaggcagcauuGga..........................................................    1       1    MOL
..............................caucuuaccaggcagcauuaCa..........................................................    7       1    MOL
..............................caucuGaccaggcagcauuaga..........................................................    1       1    MOL
..............................Uaucuuaccaggcagcauuaga..........................................................    2       1    MOL
..............................caGcuuaccaggcagcauuaga..........................................................    1       1    MOL
..............................caucuuaccaggcagcauuagC..........................................................    3       1    MOL
..............................cGucuuaccaggcagcauuaga..........................................................    5       1    MOL
..............................caucGuaccaggcagcauuaga..........................................................    1       1    MOL
..............................caucuuaccaggcagcauuagU..........................................................    111     1    MOL
..............................caucuuaccaCgcagcauuaga..........................................................    2       1    MOL
..............................caucuuaccagCcagcauuaga..........................................................    1       1    MOL
..............................caucuuaccaggcagGauuaga..........................................................    1       1    MOL
..............................caucuuaccaggcagcUuuaga..........................................................    1       1    MOL
..............................cCucuuaccaggcagcauuaga..........................................................    1       1    MOL
..............................caucuuaccaggUagcauuaga..........................................................    2       1    MOL
..............................caucuuaccaggcaCcauuaga..........................................................    1       1    MOL
..............................caucuuacUaggcagcauuaga..........................................................    1       1    MOL
..............................caucuuaccaggcagcauCaga..........................................................    1       1    MOL
..............................caucuuaccaggcaUcauuaga..........................................................    3       1    MOL
..............................caucuuaccaggcagcauuaga..........................................................    6789    0    MOL
..............................caucuuaccaggcaAcauuaga..........................................................    5       1    MOL
..............................caucuuUccaggcagcauuaga..........................................................    1       1    MOL
..............................caucuuaccaggcagcauuaAa..........................................................    16      1    MOL
..............................caucuuaccaggcagcauuaUa..........................................................    2       1    MOL
..............................caucuuaccGggcagcauuaga..........................................................    34      1    MOL
..............................caucuuaccCggcagcauuaga..........................................................    1       1    MOL
..............................caucuuaccaUgcagcauuaga..........................................................    2       1    MOL
..............................caucuuaccaggcagcauuagG..........................................................    7       1    MOL
```

```
                              Star                                      Mature
uaacaaaaauuauauaauaaucuuuguagucaucuuaccaggcagcauuagauccgaggauuuaaaacucuaauacugucagguaaugaugucagcagaguuucguacaa
..............................caucuuaccaggcUgcauuaga..........................................................   1    1   MOL
..............................caucuuaccaggcagcauuagUu.........................................................   13   1   MOL
..............................caucuuaccaggcagcauuagaC.........................................................   13   1   MOL
..............................caucuuaccaggcagcauuagau.........................................................   21   0   MOL
..............................caucuuaccaggcagcauuaUau.........................................................   1    1   MOL
..............................caucuuaccaggcagcauuagaA.........................................................   161  1   MOL
..............................caucuuaccaggcagcauuagCu.........................................................   1    1   MOL
..............................caucuuaccaggcagcauuaAau.........................................................   1    1   MOL
..............................caucuuaccaggcagcauuagaAc........................................................   1    1   MOL
..............................caucuuaccaggcagcauuagauA........................................................   6    1   MOL
..............................caucuuaccaggcagcauuagauc........................................................   1    0   MOL
..............................caucuuaccaggcagcauuagauU........................................................   4    1   MOL
..............................caucuuaccaggcagcauuagaucc.......................................................   5    0   MOL
..............................caucuuaccaggcagcauuagauccg......................................................   1    0   MOL
..............................caucuuaccaggcagcauuagauccga.....................................................   2    0   MOL
...............................aucuuaccaggcagcauuag...........................................................   3    0   MOL
...............................aucuuaccaggcagcauuaga..........................................................   10   0   MOL
...............................aucuuaccGggcagcauuaga..........................................................   1    1   MOL
...............................aucuuaccaggcagcauuagaA.........................................................   1    1   MOL
...............................aucuuaccaggcagcauuagau.........................................................   1    0   MOL
...............................aucuuaccaggcagcauuagauc........................................................   2    0   MOL
...............................aucuuaccaggcagcauuagaAc........................................................   1    1   MOL
...............................aucuuaccaggcagcauuagauccga.....................................................   1    0   MOL
................................ucuuaccaggcagcauuagau.........................................................   4    0   MOL
................................ucuuaccaggcagcauuagaA.........................................................   1    1   MOL
................................ucuuaccaggcagcauuagauc........................................................   38   0   MOL
................................ucuuaccagAcagcauuagauc........................................................   1    1   MOL
................................ucuuaccaggcagcauuagauA........................................................   2    1   MOL
.................................cuuaccaggcagcauuag...........................................................   3    0   MOL
.................................cuuaccaggcagcauuagauccg......................................................   1    0   MOL
..................................uuaccaggcagcauuaga..........................................................   1    0   MOL
..................................uuaccaggcagcauuagauccg......................................................   2    0   MOL
..................................uuaccaggcagcauuagauccgU.....................................................   1    1   MOL
..................................uuaccaggcagcauuagauccgag....................................................   1    0   MOL
....................................accaggcagcauuagauccgaggauuu...............................................   1    0   MOL
..................................................................acuUuaauacugucagguaaugaug...................   1    1   MOL
..................................................................acuUuaauacugucagguaaugaugu..................   2    1   MOL
..................................................................acucuaauacugucagguaaugauguc.................   1    0   MOL
..................................................................acuUuaauacugucagguaaugauguc.................   1    1   MOL
...................................................................Gucuaauacugucagguaaugaug...................   1    1   MOL
...................................................................cuUuaauacugucagguaaugaugu..................   12   1   MOL
...................................................................cuUuaauacugucagguaaugauguc.................   6    1   MOL
....................................................................uUuaauacugucagguaaugau....................   3    1   MOL
....................................................................uUuaauacugucagguaaugaug...................   6    1   MOL
....................................................................uUuaauacugucagguaaugaugu..................   35   1   MOL
....................................................................uAuaauacugucagguaaugauguc.................   1    1   MOL
....................................................................uUuaauacugucagguaaugauguc.................   31   1   MOL
....................................................................uUuaauacugucagguaaugauguca................   1    1   MOL
.....................................................................Uuaauacugucagguaaugau....................   3    1   MOL
.....................................................................cuaauacugucagguaaugaug...................   4    0   MOL
.....................................................................Uuaauacugucagguaaugaug...................   5    1   MOL
.....................................................................cuaauacugucagguaaugaugu..................   14   0   MOL
.....................................................................Uuaauacugucagguaaugaugu..................   63   1   MOL
.....................................................................Guaauacugucagguaaugaugu..................   2    1   MOL
.....................................................................Auaauacugucagguaaugaugu..................   1    1   MOL
.....................................................................Guaauacugucagguaaugauguc.................   3    1   MOL
.....................................................................cuaauacugucagguaaugauguU.................   5    1   MOL
.....................................................................cuaauacugucagguaaugauguc.................   7    0   MOL
.....................................................................Uuaauacugucagguaaugauguc.................   191  1   MOL
.....................................................................Auaauacugucagguaaugauguc.................   5    1   MOL
.....................................................................Guaauacugucagguaaugauguca................   1    1   MOL
.....................................................................Uuaauacugucagguaaugauguca................   5    1   MOL
.....................................................................cuaauacugucagguaaugaugucU................   5    1   MOL
......................................................................uaauacugucaggGaaug......................   1    1   MOL
......................................................................uaauacugucagguaauA......................   1    1   MOL
......................................................................uaauacugucagguaaug......................   30   0   MOL
......................................................................Aaauacugucagguaauga.....................   1    1   MOL
......................................................................uaauacugAcagguaauga.....................   1    1   MOL
......................................................................uaauacugucagguaaugC.....................   1    1   MOL
......................................................................uaauacugucagguaaAga.....................   1    1   MOL
```

```
                              Star                          Mature
uaacaaaaauuauauaauaaucuuuguagucaucuuaccaggcagcauuagauccgaggauuuaaaacucuaauacugucagguaaugaugucagcagaguuucguacaa
......................................................................uaauacugucagguaaugU.....................  8      1  MOL
......................................................................uaauacugucagguaauga.....................  274    0  MOL
......................................................................uaauacugucagguaaugGu....................  1      1  MOL
......................................................................uaUuacugucagguaaugau....................  1      1  MOL
......................................................................uaauacuguUagguaaugau....................  1      1  MOL
......................................................................uaaAacugucagguaaugau....................  1      1  MOL
......................................................................uaauacugucagguaaugaA....................  49     1  MOL
......................................................................uaauacugucagguaauCau....................  1      1  MOL
......................................................................uaauacugucagguaaugaG....................  2      1  MOL
......................................................................uaauacugAcagguaaugau....................  7      1  MOL
......................................................................uaauaGugucagguaaugau....................  1      1  MOL
......................................................................uaauacugucagguaaugUu....................  2      1  MOL
......................................................................uaauacugucagguaauUau....................  1      1  MOL
......................................................................uUauacugucagguaaugau....................  1      1  MOL
......................................................................uaauacugucaAguaaugau....................  1      1  MOL
......................................................................uaauacugucagguUaugau....................  1      1  MOL
......................................................................uaauacCgucagguaaugau....................  1      1  MOL
......................................................................uaauacugucagguaUugau....................  1      1  MOL
......................................................................uaauacugucaggAaaugau....................  6      1  MOL
......................................................................uaauacugucagguaaGgau....................  1      1  MOL
......................................................................Aaauacugucagguaaugau....................  1      1  MOL
......................................................................uaauacugucagguaaAgau....................  2      1  MOL
......................................................................uaauacAgucagguaaugau....................  2      1  MOL
......................................................................uaauacugucagAuaaugau....................  1      1  MOL
......................................................................uaauacugucagguaaugau....................  2761   0  MOL
......................................................................Gaauacugucagguaaugau....................  2      1  MOL
......................................................................uaauacugucaggCaaugau....................  1      1  MOL
......................................................................uaaCacugucagguaaugaug...................  3      1  MOL
......................................................................uaauacugucagguaaugaCg...................  3      1  MOL
......................................................................uaauacuguUagguaaugaug...................  8      1  MOL
......................................................................uaauacugucagguaaugaGg...................  2      1  MOL
......................................................................uaUuacugucagguaaugaug...................  20     1  MOL
......................................................................uaauacugGcagguaaugaug...................  2      1  MOL
......................................................................uaauacugucagguaaugauU...................  222    1  MOL
......................................................................uaauacugucagguaaAgaug...................  39     1  MOL
......................................................................uaauacugucagguaaugaAg...................  12     1  MOL
......................................................................uaauacugucagAuaaugaug...................  9      1  MOL
......................................................................uaauacugucagguaauUaug...................  7      1  MOL
......................................................................uCauacugucagguaaugaug...................  4      1  MOL
......................................................................uaauacugucagguaauCaug...................  3      1  MOL
......................................................................uaauacugucGgguaaugaug...................  10     1  MOL
......................................................................uaauacugucagguaaugGug...................  8      1  MOL
......................................................................uaauacugucagUuaaugaug...................  2      1  MOL
......................................................................uaauacugCcagguaaugaug...................  3      1  MOL
......................................................................uaauacuCucagguaaugaug...................  1      1  MOL
......................................................................uaauacugucaggAaaugaug...................  46     1  MOL
......................................................................uaauacugucagguaaugCug...................  1      1  MOL
......................................................................uaaAacugucagguaaugaug...................  13     1  MOL
......................................................................uaauacugucaggCaaugaug...................  4      1  MOL
......................................................................uaauacugucagguaUugaug...................  5      1  MOL
......................................................................uaauacuAucagguaaugaug...................  1      1  MOL
......................................................................uaauaGugucagguaaugaug...................  7      1  MOL
......................................................................uaauacugucUgguaaugaug...................  2      1  MOL
......................................................................uaauacugucagguaaugauC...................  77     1  MOL
......................................................................uaauacCgucagguaaugaug...................  1      1  MOL
......................................................................uaauaAugucagguaaugaug...................  3      1  MOL
......................................................................uaauacugucaCguaaugaug...................  4      1  MOL
......................................................................uaauacuguGagguaaugaug...................  6      1  MOL
......................................................................uaauacugucagCuaaugaug...................  5      1  MOL
......................................................................uaauacugucagguaGugaug...................  2      1  MOL
......................................................................uaauacugucagguaaGgaug...................  6      1  MOL
......................................................................uaauaUugucagguaaugaug...................  13     1  MOL
......................................................................uaCuacugucagguaaugaug...................  1      1  MOL
......................................................................uaaGacugucagguaaugaug...................  1      1  MOL
......................................................................uaauacugucagguaauAaug...................  19     1  MOL
......................................................................uaauUcugucagguaaugaug...................  9      1  MOL
......................................................................uaauacugAcagguaaugaug...................  34     1  MOL
......................................................................uaauacugucaUguaaugaug...................  2      1  MOL
......................................................................uaauacugucagguaaugaug...................  27103  0  MOL
......................................................................uaauacAgucagguaaugaug...................  10     1  MOL
```

```
                                Star                                       Mature
uaacaaaaauuauauaauaaucuuuguagucaucuuaccaggcagcauuagauccgaggauuuaaaacucuaauacugucagguaaugaugucagcagaguuucguacaa
......................................................................uaauacugucaAguaaugaug...................   2       1   MOL
......................................................................uaauacugucagguaaugauA...................   813     1   MOL
......................................................................uaauacugucagguaaugUug...................   14      1   MOL
......................................................................uaauacugucagguGaugaug...................   1       1   MOL
......................................................................uaauacuUucagguaaugaug...................   1       1   MOL
......................................................................Caauacugucagguaaugaug...................   4       1   MOL
......................................................................uaauacugucaggGaaugaug...................   3       1   MOL
......................................................................uaauacugucagguUaugaug...................   20      1   MOL
......................................................................Gaauacugucagguaaugaug...................   5       1   MOL
......................................................................Aaauacugucagguaaugaug...................   30      1   MOL
......................................................................uaauacuguAagguaaugaug...................   1       1   MOL
......................................................................uGauacugucagguaaugaug...................   2       1   MOL
......................................................................uaauacugucUgguaaugaugu..................   15      1   MOL
......................................................................uaauacugucagguaaugauUu..................   56      1   MOL
......................................................................uaauacuAucagguaaugaugu..................   9       1   MOL
......................................................................uaauacugucagAuaaugaugu..................   57      1   MOL
......................................................................uaauGcugucagguaaugaugu..................   13      1   MOL
......................................................................uaauaAugucagguaaugaugu..................   26      1   MOL
......................................................................uaauacuCucagguaaugaugu..................   13      1   MOL
......................................................................uaauacugucagCuaaugaugu..................   22      1   MOL
......................................................................uaauacugucagguaauAaugu..................   62      1   MOL
......................................................................Caauacugucagguaaugaugu..................   41      1   MOL
......................................................................uaauacugucagguaaGgaugu..................   35      1   MOL
......................................................................Aaauacugucagguaaugaugu..................   264     1   MOL
......................................................................uaauacugucagguaaugaugA..................   732     1   MOL
......................................................................uaauacugucGgguaaugaugu..................   39      1   MOL
......................................................................uaauacugucagguGaugaugu..................   17      1   MOL
......................................................................uaauacGgucagguaaugaugu..................   6       1   MOL
......................................................................uaauacuguAagguaaugaugu..................   14      1   MOL
......................................................................uaauacugucCgguaaugaugu..................   3       1   MOL
......................................................................uaauacugucagguaaugCugu..................   15      1   MOL
......................................................................uaauacugucaCguaaugaugu..................   19      1   MOL
......................................................................uaauacugucagguaaugaCgu..................   18      1   MOL
......................................................................uaauacugucagguaGugaugu..................   12      1   MOL
......................................................................uaauacugucagguCaugaugu..................   4       1   MOL
......................................................................uaaCacugucagguaaugaugu..................   29      1   MOL
......................................................................uaauacugucagUuaaugaugu..................   31      1   MOL
......................................................................uaauacugucagguaaugGugu..................   47      1   MOL
......................................................................uaauacAgucagguaaugaugu..................   79      1   MOL
......................................................................uaauacugucagguaaugauCu..................   24      1   MOL
......................................................................uaauUcugucagguaaugaugu..................   21      1   MOL
......................................................................uaauacugucaggAaaugaugu..................   308     1   MOL
......................................................................uaCuacugucagguaaugaugu..................   3       1   MOL
......................................................................uaauacugucagguaUugaugu..................   37      1   MOL
......................................................................uaauacugAcagguaaugaugu..................   249     1   MOL
......................................................................uaauacugucagguaaAgaugu..................   376     1   MOL
......................................................................uaauacuguGagguaaugaugu..................   41      1   MOL
......................................................................Gaauacugucagguaaugaugu..................   50      1   MOL
......................................................................uaauacugucagguaaugauAu..................   35      1   MOL
......................................................................uUauacugucagguaaugaugu..................   40      1   MOL
......................................................................uaauaUugucagguaaugaugu..................   49      1   MOL
......................................................................uaauacugGcagguaaugaugu..................   18      1   MOL
......................................................................uaauacCgucagguaaugaugu..................   14      1   MOL
......................................................................uaauacugucagguaCugaugu..................   13      1   MOL
......................................................................uaauacuguUagguaaugaugu..................   73      1   MOL
......................................................................uaauacugucagguaaugaugG..................   34      1   MOL
......................................................................uaauacugucaAguaaugaugu..................   35      1   MOL
......................................................................uaauacugucagguaaugUugu..................   244     1   MOL
......................................................................uaauacugucaUguaaugaugu..................   26      1   MOL
......................................................................uaaAacugucagguaaugaugu..................   88      1   MOL
......................................................................uaauCcugucagguaaugaugu..................   3       1   MOL
......................................................................uCauacugucagguaaugaugu..................   21      1   MOL
......................................................................uaauacugucagguaauUaugu..................   46      1   MOL
......................................................................uaauacugCcagguaaugaugu..................   32      1   MOL
......................................................................uaauacugucagguUaugaugu..................   112     1   MOL
......................................................................uaauacugucagguaaugaugu..................   235359  0   MOL
......................................................................uaauacugucagguaaugaugC..................   208     1   MOL
......................................................................uaauacugucaggCaaugaugu..................   19      1   MOL
......................................................................uaauaGugucagguaaugaugu..................   65      1   MOL
......................................................................uaGuacugucagguaaugaugu..................   5       1   MOL
```

Star Mature

uaacaaaaauuauauaauaaucuuuguagucaucuuaccaggcagcauuagauccgaggauuuaaaacucuaauacugucagguaaugaugucagcagaguuucguacaa			
....uaauacugucagguaaugaGgu..................	39	1	MOL
....uaauacugucagguaaCgaugu..................	43	1	MOL
....uaauacuUucagguaaugaugu..................	11	1	MOL
....uaauacugucaggGaaugaugu..................	21	1	MOL
....uaaGacugucagguaaugaugu..................	13	1	MOL
....uaUuacugucagguaaugaugu..................	89	1	MOL
....uaauacugucagguaaugaAgu..................	31	1	MOL
....uGauacugucagguaaugaugu..................	30	1	MOL
....uaauacugucagguaauCaugu..................	42	1	MOL
....uaauacugucagguaGugauguc.................	35	1	MOL
....uaauacugucagguaaugauCuc.................	117	1	MOL
....uaauacugucaggCaaugauguc.................	80	1	MOL
....uaauacugucaAguaaugauguc.................	103	1	MOL
....uaauacugucagguCaugauguc.................	22	1	MOL
....uaauacugucagCuaaugauguc.................	61	1	MOL
....uaauacugucagUuaaugauguc.................	151	1	MOL
....Caauacugucagguaaugauguc.................	129	1	MOL
....uaauGcugucagguaaugauguc.................	50	1	MOL
....uaUuacugucagguaaugauguc.................	602	1	MOL
....uaauacugCcagguaaugauguc.................	71	1	MOL
....uaauacAgucagguaaugauguc.................	192	1	MOL
....uaauacugucUgguaaugauguc.................	45	1	MOL
....uaauCcugucagguaaugauguc.................	6	1	MOL
....Gaauacugucagguaaugauguc.................	96	1	MOL
....uaCuacugucagguaaugauguc.................	60	1	MOL
....uaauacugucGgguaaugauguc.................	96	1	MOL
....uaauacugucagguaaugaugAc.................	1047	1	MOL
....uaGuacugucagguaaugauguc.................	104	1	MOL
....uaauacugucagguaaugaGguc.................	120	1	MOL
....uaauaAugucagguaaugauguc.................	58	1	MOL
....uaauacugucagguUaugauguc.................	408	1	MOL
....uaauacugucagguaaugGuguc.................	158	1	MOL
....Aaauacugucagguaaugauguc.................	769	1	MOL
....uUauacugucagguaaugauguc.................	118	1	MOL
....uGauacugucagguaaugauguc.................	109	1	MOL
....uaauacugucaggAaaugauguc.................	898	1	MOL
....uaauacugucagguaaugauguc.................	713177	0	MOL
....uaauacugucaUguaaugauguc.................	79	1	MOL
....uaauacGgucagguaaugauguc.................	15	1	MOL
....uaauacugucagguaaugCuguc.................	72	1	MOL
....uaauacugucagguaaugUuguc.................	795	1	MOL
....uaauaUugucagguaaugauguc.................	156	1	MOL
....uaauacugucagguaaugauUuc.................	184	1	MOL
....uaauacCgucagguaaugauguc.................	46	1	MOL
....uaauacuCucagguaaugauguc.................	41	1	MOL
....uaauacugucagguaaCgauguc.................	96	1	MOL
....uaauacugGcagguaaugauguc.................	37	1	MOL
....uaauacugucagguaaugaCguc.................	43	1	MOL
....uaauacugucagguaaugauguU.................	10347	1	MOL
....uaauacugucagguaaugauguA.................	803	1	MOL
....uaauacuAucagguaaugauguc.................	55	1	MOL
....uaauacugucCgguaaugauguc.................	3	1	MOL
....uaauacugucagguaaugauguG.................	109	1	MOL
....uaaCacugucagguaaugauguc.................	71	1	MOL
....uaauacugucagguaauCauguc.................	94	1	MOL
....uaauacuguGagguaaugauguc.................	74	1	MOL
....uaaGacugucagguaaugauguc.................	35	1	MOL
....uaauacugucagguGaugauguc.................	54	1	MOL
....uaauUcugucagguaaugauguc.................	73	1	MOL
....uaauacugAcagguaaugauguc.................	753	1	MOL
....uCauacugucagguaaugauguc.................	94	1	MOL
....uaauacugucagguaaGgauguc.................	95	1	MOL
....uaauacugucagguaCugauguc.................	44	1	MOL
....uaaAacugucagguaaugauguc.................	287	1	MOL
....uaauacuUucagguaaugauguc.................	31	1	MOL
....uaauacugucagguaauUauguc.................	133	1	MOL
....uaauacugucagguaaAgauguc.................	2221	1	MOL
....uaauacugucagguaaugaugGc.................	116	1	MOL
....uaauacugucagAuaaugauguc.................	133	1	MOL
....uaauacugucagguaaugaAguc.................	132	1	MOL

```
                               Star                                   Mature
uaacaaaaauuauauaauaaucuuuguagucaucuuaccaggcagcauuagauccgaggauuuaaaacucuaauacugucagguaaugaugucagcagaguuucguacaa
......................................................................uaauacugucagguaUugauguc.................   136     1    MOL
......................................................................uaauacugucagguaaugaugCc.................   297     1    MOL
......................................................................uaauaGugucagguaaugauguc.................   178     1    MOL
......................................................................uaauacuguUagguaaugauguc.................   127     1    MOL
......................................................................uaauacugucaggGaaugauguc.................   59      1    MOL
......................................................................uaauacugucaCguaaugauguc.................   71      1    MOL
......................................................................uaauacugucagguaauAauguc.................   129     1    MOL
......................................................................uaauacugucagguaaugauAuc.................   39      1    MOL
......................................................................uaauacuguAagguaaugauguc.................   21      1    MOL
......................................................................uaGuacugucagguaaugauguca................   4       1    MOL
......................................................................CaauacugucagguaaugaugUca................   4       1    MOL
......................................................................uaauacugucagguaaugaCguca................   1       1    MOL
......................................................................uaauacugucagguaUugauguca................   2       1    MOL
......................................................................uaauacugucagAuaaugauguca................   3       1    MOL
......................................................................uaauacugucagguaaugauguUa................   81      1    MOL
......................................................................uaauacuguAagguaaugauguca................   1       1    MOL
......................................................................uaaAacugucagguaaugauguca................   6       1    MOL
......................................................................uaauacugucagguaaAgauguca................   24      1    MOL
......................................................................uaauacugucaggCaaugauguca................   5       1    MOL
......................................................................uaauacuCucagguaaugauguca................   1       1    MOL
......................................................................uaaCacugucagguaaugauguca................   2       1    MOL
......................................................................uaauacugucaCguaaugauguca................   1       1    MOL
......................................................................uaauacugCcagguaaugauguca................   2       1    MOL
......................................................................uaauacugucagguaGugauguca................   2       1    MOL
......................................................................uaauacuAucagguaaugauguca................   2       1    MOL
......................................................................uaauacugucagguaaugauguca................   15783   0    MOL
......................................................................uaauacugucagguaauCauguca................   6       1    MOL
......................................................................uaauacugucagguaaugaGguca................   5       1    MOL
......................................................................uaauacugucagguaaugaAguca................   3       1    MOL
......................................................................uaauacugucagguaaugauguGa................   3       1    MOL
......................................................................uaauacugucagguaaugCuguca................   4       1    MOL
......................................................................uaauacugucagguaaugUuguca................   19      1    MOL
......................................................................uaauacugucagguUaugauguca................   12      1    MOL
......................................................................uaauacugucagguaaugaugGca................   6       1    MOL
......................................................................uaUuacugucagguaaugauguca................   10      1    MOL
......................................................................uaauUcugucagguaaugauguca................   2       1    MOL
......................................................................uaauaGugucagguaaugauguca................   3       1    MOL
......................................................................uaauacugGcagguaaugauguca................   1       1    MOL
......................................................................uaauacugucagguaaugauguAa................   15      1    MOL
......................................................................uaauacugAcagguaaugauguca................   14      1    MOL
......................................................................uaauacugucagUuaaugauguca................   3       1    MOL
......................................................................uaauacugucagguaaugaugucC................   643     1    MOL
......................................................................uaauacugucagguaaugauAuca................   2       1    MOL
......................................................................Aaauacugucagguaaugauguca................   14      1    MOL
......................................................................uaauacugucUgguaaugauguca................   1       1    MOL
......................................................................uaauacugucagguaauAauguca................   5       1    MOL
......................................................................uaauacugucagguaaugaugAca................   30      1    MOL
......................................................................uaauacugucagguaaugaugucG................   640     1    MOL
......................................................................uaauacugucaggAaaugauguca................   22      1    MOL
......................................................................uaauacuguUagguaaugauguca................   8       1    MOL
......................................................................Gaauacugucagguaaugauguca................   3       1    MOL
......................................................................uaauacugucagguaaugaugucU................   35994   1    MOL
......................................................................uaauacugucagguaaGgauguca................   1       1    MOL
......................................................................uGauacugucagguaaugauguca................   3       1    MOL
......................................................................uaauacugucagguGaugauguca................   1       1    MOL
......................................................................uaauacugucagguaaugaugCca................   5       1    MOL
......................................................................uaauacugucGgguaaugauguca................   2       1    MOL
......................................................................uaauacugucagCuaaugauguca................   3       1    MOL
......................................................................uaauacugucagguaaugauUuca................   5       1    MOL
......................................................................uaauacugucagguaaugGuguca................   2       1    MOL
......................................................................uaauacAgucagguaaugauguca................   4       1    MOL
......................................................................uaauacuguGagguaaugauguca................   3       1    MOL
......................................................................uaauacCgucagguaaugauguca................   1       1    MOL
......................................................................uCauacugucagguaaugauguca................   1       1    MOL
......................................................................uaauacugucagguaauUauguca................   5       1    MOL
......................................................................uaauaUugucagguaaugauguca................   5       1    MOL
......................................................................uaauacugucagguaaugaugucaA...............   69      1    MOL
......................................................................uaauacugucagguaaugaugucaU...............   125     1    MOL
......................................................................uaauacugucagguaaugaugucaC...............   19      1    MOL
......................................................................uaauacugucagguaaugUugucag...............   1       1    MOL
```

Star **Mature**

```
uaacaaaaauuauauaauaaucuuuguagucaucuuaccaggcagcauuagauccgaggauuuaaaacucuaauacugucagguaaugaugucagcagaguuucguacaa
......................................................................uaauacugucagguaaugaugucag...............  2     0  MOL
......................................................................uaauacugucagguaaugaugucUg...............  2     1  MOL
......................................................................uaauacugucagguaaugaugucagU..............  3     1  MOL
......................................................................uaauacugucagguaaugaugucGgc..............  3     1  MOL
......................................................................uaauacugucagguaaugauguGagc..............  1     1  MOL
......................................................................uaauacugucagguaaugaugucaAc..............  1     1  MOL
......................................................................uaauacugucagguaaugaugucagUa.............  1     1  MOL
.......................................................................aauacugucagguaauga.....................  2     0  MOL
.......................................................................aauacugucagguaaugau....................  3     0  MOL
.......................................................................Uauacugucagguaaugaug...................  1     1  MOL
.......................................................................aauacugucagguaaugaug...................  35    0  MOL
.......................................................................aauacugucaggAaaugaug...................  1     1  MOL
.......................................................................aauacugucagguaaugauA...................  1     1  MOL
.......................................................................aauacGgucagguaaugaugu..................  1     1  MOL
.......................................................................aauacugucaCguaaugaugu..................  1     1  MOL
.......................................................................aauacugucagCuaaugaugu..................  1     1  MOL
.......................................................................aUuacugucagguaaugaugu..................  1     1  MOL
.......................................................................aauacugucagguaaugGugu..................  1     1  MOL
.......................................................................aauacugucagguaGugaugu..................  1     1  MOL
.......................................................................Uauacugucagguaaugaugu..................  3     1  MOL
.......................................................................aauacugucagguaaugaugu..................  363   0  MOL
.......................................................................aauacugucagguaaugaugA..................  2     1  MOL
.......................................................................aauacugucagguaaugaGgu..................  1     1  MOL
.......................................................................aauacugucagguaaAgaugu..................  1     1  MOL
.......................................................................aauUcugucagguaaugauguc.................  1     1  MOL
.......................................................................aauacuguAagguaaugauguc.................  4     1  MOL
.......................................................................aauacugucagguaaugauguc.................  2634  0  MOL
.......................................................................aauacuUucagguaaugauguc.................  1     1  MOL
.......................................................................aauacugucaCguaaugauguc.................  1     1  MOL
.......................................................................aauacugucagAuaaugauguc.................  3     1  MOL
.......................................................................aauacugucagguaUugauguc.................  2     1  MOL
.......................................................................aUuacugucagguaaugauguc.................  1     1  MOL
.......................................................................aauacugucagguaaugaugAc.................  1     1  MOL
.......................................................................aauacugucagguaaCgauguc.................  1     1  MOL
.......................................................................aaAacugucagguaaugauguc.................  1     1  MOL
.......................................................................Uauacugucagguaaugauguc.................  37    1  MOL
.......................................................................aauacugucagguaCugauguc.................  1     1  MOL
.......................................................................aauacugucagguaaugauCuc.................  1     1  MOL
.......................................................................aauacugucagguaaugauUuc.................  1     1  MOL
.......................................................................aauacugucagguaauUauguc.................  1     1  MOL
.......................................................................aauacugucagguGaugauguc.................  1     1  MOL
.......................................................................aauacugucagguaaugauguA.................  5     1  MOL
.......................................................................aGuacugucagguaaugauguc.................  1     1  MOL
.......................................................................aauacugucagguaaugauAuc.................  1     1  MOL
.......................................................................aauacugucagguaaugGuguc.................  3     1  MOL
.......................................................................Gauacugucagguaaugauguc.................  2     1  MOL
.......................................................................aauacugucaAguaaugauguc.................  1     1  MOL
.......................................................................aauacugucagguaaugaugCc.................  1     1  MOL
.......................................................................aauacAgucagguaaugauguc.................  1     1  MOL
.......................................................................aauacugucagguaaAgauguc.................  5     1  MOL
.......................................................................aauacugucagguaaugUuguc.................  1     1  MOL
.......................................................................aCuacugucagguaaugauguc.................  4     1  MOL
.......................................................................aauacugucaggAaaugauguc.................  1     1  MOL
.......................................................................aauacugAcagguaaugauguc.................  4     1  MOL
.......................................................................aauacugucagguaaugauguU.................  22    1  MOL
.......................................................................aauacugCcagguaaugauguc.................  2     1  MOL
.......................................................................aauacugucCgguaaugauguc.................  1     1  MOL
.......................................................................aauacugucagguaaugauguca................  137   0  MOL
.......................................................................aauacuguAagguaaugauguca................  1     1  MOL
.......................................................................aauacugucagguaaugaugucU................  697   1  MOL
.......................................................................aauacugucagguaaugaugGca................  1     1  MOL
.......................................................................aauacugucagguaaugaugucC................  13    1  MOL
.......................................................................Uauacugucagguaaugauguca................  1     1  MOL
.......................................................................aauacugucagguaaugauguUa................  1     1  MOL
.......................................................................aauacugucagguaaugaugucG................  8     1  MOL
.......................................................................aauacugucaCguaaugauguca................  1     1  MOL
.......................................................................aauacugucagguaaugaugucaA...............  3     1  MOL
........................................................................auacugucagguaaugaugu..................  2     0  MOL
........................................................................auacugucagguaaugauguU.................  2     1  MOL
........................................................................aAacugucagguaaugauguc.................  1     1  MOL
```

```
                              Star                                Mature
uaacaaaaauuauauaauaaucuuuguagucaucuuaccaggcagcauuagauccgaggauuuaaaacucuaauacugucagguaaugaugucagcagaguuucguacaa
........................................................................auacugucagguaaAgauguc.................  1    1  MOL
........................................................................Uuacugucagguaaugauguc.................  7    1  MOL
........................................................................auacugucagguaaugauguc.................  56   0  MOL
........................................................................aGacugucagguaaugauguc.................  1    1  MOL
........................................................................auacugucagguaaugaugucG................  1    1  MOL
........................................................................auacugucagguaaugaugucU................  10   1  MOL
........................................................................auacugucagguaaugauguca................  3    0  MOL
........................................................................auacugucagguaaugaugucaU...............  2    1  MOL
.........................................................................uacugucagguaaugaug...................  2    0  MOL
.........................................................................uacugucagguaaugaugu..................  2    0  MOL
.........................................................................uacugucagguaaugauguc.................  11   0  MOL
.........................................................................uacugucagguaaugaugucU................  2    1  MOL
.........................................................................uacugucagguaaugauguca................  1    0  MOL
..........................................................................acugucagguaaugaugu..................  6    0  MOL
..........................................................................acugucagguaaugauguc.................  12   0  MOL
..........................................................................aUugucagguaaugauguc.................  1    1  MOL
..........................................................................acugucagguaaugauguU.................  1    1  MOL
..........................................................................acugucagguaaugauguca................  1    0  MOL
...........................................................................cugucagguaaugauguc.................  18   0  MOL
...........................................................................cugucagguaaugauguca................  2    0  MOL
...........................................................................cugucagguaaugaugucU................  1    1  MOL

...........................agGcaucuuaccaggcagcauuaga..........................................................  2    1  te1
............................Uucaucuuaccaggcagcauuag...........................................................  1    1  te1
............................gGcaucuuaccaggcagcauuaga..........................................................  1    1  te1
............................Uucaucuuaccaggcagcauuaga..........................................................  1    1  te1
.............................ucaucuuaccaggcagcau..............................................................  1    0  te1
.............................Ccaucuuaccaggcagcauu.............................................................  2    1  te1
.............................ucaucuCaccaggcagcauu.............................................................  1    1  te1
.............................Gcaucuuaccaggcagcauu.............................................................  1    1  te1
.............................ucaucuuaccaggcagcauu.............................................................  117  0  te1
.............................Ccaucuuaccaggcagcauua............................................................  2    1  te1
.............................ucauGuuaccaggcagcauua............................................................  1    1  te1
.............................ucaucuuaccaggcagcauuU............................................................  4    1  te1
.............................ucaucuuaccaggcagcauua............................................................  209  0  te1
.............................ucaucuuaccaggcagcUuua............................................................  1    1  te1
.............................ucaucuuaccaggcagcauuaA...........................................................  6    1  te1
.............................Gcaucuuaccaggcagcauuag...........................................................  2    1  te1
.............................Acaucuuaccaggcagcauuag...........................................................  2    1  te1
.............................ucaucuuaccaggUagcauuag...........................................................  1    1  te1
.............................ucaucuuaccaggcagcauuag...........................................................  215  0  te1
.............................ucaucuuaccaggcagcUuuag...........................................................  1    1  te1
.............................ucaucuuaccaggcagAauuag...........................................................  1    1  te1
.............................Ccaucuuaccaggcagcauuag...........................................................  2    1  te1
.............................ucaucuuaccaggcagcauuaU...........................................................  4    1  te1
.............................ucaucuuaccaggcagcauuaAa..........................................................  1    1  te1
.............................ucaucuuaccaggcagcauuaga..........................................................  36   0  te1
.............................ucaucuuaccaggcagcauuagU..........................................................  7    1  te1
.............................ucaucuuaccaggcagcauuagaA.........................................................  2    1  te1
.............................ucaucuuaccaggcagcauuagau.........................................................  3    0  te1
.............................ucaucuuaccaggcagcauuagUu.........................................................  1    1  te1
..............................caucuuaccaggcagcau..............................................................  1    0  te1
..............................caucuuaccaggcagcaG..............................................................  1    1  te1
..............................caucuuaccaggcagcauu.............................................................  175  0  te1
..............................caucuuaccaggcagcaCu.............................................................  1    1  te1
..............................caucuuaccaggcagcauA.............................................................  1    1  te1
..............................caucuuaccaggcagcauG.............................................................  1    1  te1
..............................caucuuaccaggcagUauua............................................................  2    1  te1
..............................cGucuuaccaggcagcauua............................................................  1    1  te1
..............................caucuuaccaggcagcauua............................................................  697  0  te1
..............................caucuuaccaggcagcauuU............................................................  13   1  te1
..............................caucuuacUaggcagcauua............................................................  1    1  te1
..............................caucuuaccaggcagcauGa............................................................  1    1  te1
..............................cUucuuaccaggcagcauua............................................................  2    1  te1
..............................caucuuUccaggcagcauuag...........................................................  1    1  te1
..............................caucuuaccUggcagcauuag...........................................................  1    1  te1
..............................caucuuaccaggcagcaGuag...........................................................  2    1  te1
..............................caucuuacUaggcagcauuag...........................................................  2    1  te1
..............................caucuuaccaCgcagcauuag...........................................................  2    1  te1
..............................caucuuaUcaggcagcauuag...........................................................  1    1  te1
```

```
                              Star                            Mature
uaacaaaaauuauauaauaaucuuuguagucaucuuaccaggcagcauuagauccgaggauuuaaaacucuaauacugucagguaaugaugucagcagaguuucguacaa
..............................caucuuaccaggcagcauuaA...........................................................    48      1    te1
..............................caucAuaccaggcagcauuag...........................................................    3       1    te1
..............................caucuuaccaggUagcauuag...........................................................    1       1    te1
..............................caucuuaccaggcaAcauuag...........................................................    3       1    te1
..............................caucuuaccaggcagcauuaC...........................................................    3       1    te1
..............................cauUuuaccaggcagcauuag...........................................................    1       1    te1
..............................caucuuaccaggcagcauuGg...........................................................    1       1    te1
..............................caucuuaccaggcGgcauuag...........................................................    3       1    te1
..............................Uaucuuaccaggcagcauuag...........................................................    3       1    te1
..............................caucuAaccaggcagcauuag...........................................................    1       1    te1
..............................caucuuaccaggcagcGuuag...........................................................    1       1    te1
..............................caGcuuaccaggcagcauuag...........................................................    1       1    te1
..............................caCcuuaccaggcagcauuag...........................................................    1       1    te1
..............................caucuCaccaggcagcauuag...........................................................    1       1    te1
..............................caucuuaccaggcagcaAuag...........................................................    1       1    te1
..............................cauGuuaccaggcagcauuag...........................................................    3       1    te1
..............................caucGuaccaggcagcauuag...........................................................    2       1    te1
..............................caucuuaccaAgcagcauuag...........................................................    2       1    te1
..............................cUucuuaccaggcagcauuag...........................................................    3       1    te1
..............................caucuuaccaggcagcauuaU...........................................................    36      1    te1
..............................caucuuaccGggcagcauuag...........................................................    2       1    te1
..............................cCucuuaccaggcagcauuag...........................................................    2       1    te1
..............................caucuuaccaggcagcauCag...........................................................    1       1    te1
..............................caucuGaccaggcagcauuag...........................................................    1       1    te1
..............................caucuuaccCggcagcauuag...........................................................    4       1    te1
..............................caucuuaccaggcagcauuag...........................................................    6349    0    te1
..............................caucuuaccaggcagUauuag...........................................................    1       1    te1
..............................caucuuaccaggcagcauGag...........................................................    1       1    te1
..............................caucuuaccagAcagcauuag...........................................................    3       1    te1
..............................caucuuaccaggcaCcauuag...........................................................    1       1    te1
..............................caucuuaccaggcagcauuaga..........................................................    5339    0    te1
..............................caucuuaccagCcagcauuaga..........................................................    1       1    te1
..............................caucuuaccaggUagcauuaga..........................................................    1       1    te1
..............................caucuuUccaggcagcauuaga..........................................................    1       1    te1
..............................caucuuaccaggcagcauuagC..........................................................    1       1    te1
..............................caucuuacUaggcagcauuaga..........................................................    2       1    te1
..............................caucuuaccaggcGgcauuaga..........................................................    1       1    te1
..............................caucuuaccaggcagcauCaga..........................................................    2       1    te1
..............................caucuuaccaCgcagcauuaga..........................................................    1       1    te1
..............................caucuuaccaggcagcauGaga..........................................................    2       1    te1
..............................caucuuaccaAgcagcauuaga..........................................................    1       1    te1
..............................caucuGaccaggcagcauuaga..........................................................    2       1    te1
..............................Uaucuuaccaggcagcauuaga..........................................................    2       1    te1
..............................caucuuaccaggcagcauuaUa..........................................................    1       1    te1
..............................cGucuuaccaggcagcauuaga..........................................................    3       1    te1
..............................caucuuaccaggcagcUuuaga..........................................................    1       1    te1
..............................caucuuaccaggcagcauuaAa..........................................................    6       1    te1
..............................caucuuaccaggcUgcauuaga..........................................................    1       1    te1
..............................caucuuaccGggcagcauuaga..........................................................    3       1    te1
..............................caucuuaccaggcagcauuagG..........................................................    96      1    te1
..............................caucGuaccaggcagcauuaga..........................................................    2       1    te1
..............................caucuuaccaggcaAcauuaga..........................................................    2       1    te1
..............................caucuuaccaggcagcCuuaga..........................................................    2       1    te1
..............................caucuuaccagAcagcauuaga..........................................................    5       1    te1
..............................caucuuaccaggcagUauuaga..........................................................    1       1    te1
..............................caucuuaccaggcagcaGuaga..........................................................    1       1    te1
..............................caucuuaccaggcagcauuagU..........................................................    138     1    te1
..............................caucuuaccaUgcagcauuaga..........................................................    1       1    te1
..............................caucuuacGaggcagcauuaga..........................................................    1       1    te1
..............................cUucuuaccaggcagcauuaga..........................................................    4       1    te1
..............................caucuuaccaggcaUcauuaga..........................................................    1       1    te1
..............................caucuuaccaggcagcauuagaG.........................................................    2       1    te1
..............................caucuuaccaggcagcauuagaA.........................................................    141     1    te1
..............................caucuuaccaggcagcauuagUu.........................................................    4       1    te1
..............................caucuuaccaggcagcauuagaC.........................................................    2       1    te1
..............................caucuuaccaggcagcauuagau.........................................................    23      0    te1
..............................caucuuaccaggcagcauuagauA........................................................    5       1    te1
..............................caucuuaccaggcagcauuagaAc........................................................    1       1    te1
..............................caucuuaccaggcagcauuagauU........................................................    4       1    te1
..............................caucuuaccaggcagcauuagaucc.......................................................    1       0    te1
```

Star Mature

uaacaaaaauuauauaauaaucuuuguagucaucuuaccaggcagcauuagauccgaggauuuaaaacucuaauacugucagguaaugaugucagcagaguuucguacaa			
...............................aucuuaccaggcagcauuag...	1	0	te1
...............................aucuuaccaggcagcauuaga..	12	0	te1
...............................aucuuaccaggcagcauuagaA...	1	1	te1
...............................aucuuaccaggcagcauuagauc..	1	0	te1
................................ucuuaccaggcagcauuaga..	3	0	te1
................................ucuuaccaggcagcauuagau...	3	0	te1
................................ucuuaccaggcagcauuagauc..	27	0	te1
................................ucuuaccaggUagcauuagauc..	1	1	te1
................................ucuuaccaggcagcauuagauA..	1	1	te1
.................................cuuaccaggcagcauuag...	2	0	te1
.................................cuuaccaggcagcauuaga..	1	0	te1
..................................uuaccaggcagcauuaga..	6	0	te1
..................................uuaccaggcagcauuagaA...	1	1	te1
....acuUuaauacugucagguaaugaugu..................	1	1	te1
....cuUuaauacugucagguaaugaugu..................	4	1	te1
....cuUuaauacugucagguaaugauguc.................	4	1	te1
....uUuaauacugucagguaaugaug...................	2	1	te1
....Acuaauacugucagguaaugaugu..................	1	1	te1
....uUuaauacugucagguaaugaugu..................	26	1	te1
....uUuaauacugucagguaaugauguc.................	12	1	te1
....Uuaauacugucagguaaugau....................	2	1	te1
....cuaauacugucagguaaugau....................	4	0	te1
....Uuaauacugucagguaaugaug...................	9	1	te1
....Uuaauacugucagguaaugaugu..................	48	1	te1
....cuaauacugucagguaaugaugu..................	12	0	te1
....Guaauacugucagguaaugaugu..................	1	1	te1
....Auaauacugucagguaaugaugu..................	7	1	te1
....Uuaauacugucagguaaugauguc.................	43	1	te1
....cuaauacugucagguaaugauguU.................	1	1	te1
....Auaauacugucagguaaugauguc.................	2	1	te1
....cuaauacugucagguaaugaugucU................	1	1	te1
....uaauacugucagguaaGg......................	1	1	te1
....uaauacugucagguaaug......................	18	0	te1
....uaauacugucagguaaugU.....................	2	1	te1
....uaauacugucagguaauga.....................	57	0	te1
....uaauacuCucagguaauga.....................	1	1	te1
....uaauacuguUagguaaugau....................	1	1	te1
....uaauacugucagUuaaugau....................	1	1	te1
....uaauaUugucagguaaugau....................	1	1	te1
....uaauacugucagguaCugau....................	1	1	te1
....Aaauacugucagguaaugau....................	1	1	te1
....uaauacugucagguaaugaA....................	19	1	te1
....uaCuacugucagguaaugau....................	1	1	te1
....uaauacuCucagguaaugau....................	2	1	te1
....uaauacugucagguaaugaG....................	2	1	te1
....uaauacugucagguaaugau....................	1748	0	te1
....uaauacuguGagguaaugau....................	1	1	te1
....uaauacugucaggCaaugau....................	1	1	te1
....uaauacugucagguaaugGu....................	1	1	te1
....uaauacugucagguaauCau....................	1	1	te1
....uUauacugucagguaaugau....................	2	1	te1
....uaauacugucagguaaugUu....................	5	1	te1
....uCauacugucagguaaugau....................	1	1	te1
....uaauGcugucagguaaugaug...................	6	1	te1
....uaauaUugucagguaaugaug...................	4	1	te1
....uaauacugucaggGaaugaug...................	4	1	te1
....uaauacugucagguaaGgaug...................	1	1	te1
....uaaAacugucagguaaugaug...................	1	1	te1
....uaauacugucaggAaaugaug...................	1	1	te1
....uaauacugucaggCaaugaug...................	4	1	te1
....uaauacugucagAuaaugaug...................	2	1	te1
....uaUuacugucagguaaugaug...................	1	1	te1
....uaaGacugucagguaaugaug...................	1	1	te1
....uUauacugucagguaaugaug...................	2	1	te1
....uaauacugucagguaaugaGg...................	35	1	te1
....uaauacuAucagguaaugaug...................	1	1	te1
....uaauacugucagguaaugauC...................	21	1	te1
....uaauacugucagguaaugCug...................	2	1	te1
....uaauacugCcagguaaugaug...................	6	1	te1
....uaauacuguGagguaaugaug...................	2	1	te1

```
                                   Star                                         Mature
uaacaaaaauuauauaauaaucuuuguagucaucuuaccaggcagcauuagauccgaggauuuaaaacucuaauacugucagguaaugaugucagcagaguuucguacaa
......................................................................uaauacGgucagguaaugaug...................   1      1    te1
......................................................................uaaCacugucagguaaugaug...................   3      1    te1
......................................................................uaauacugucCgguaaugaug...................   1      1    te1
......................................................................uaauacugucagguaGugaug...................   1      1    te1
......................................................................uaauacugGcagguaaugaug...................   3      1    te1
......................................................................uaauacugucagguaaugaAg...................   3      1    te1
......................................................................uaauacugucagguaaugauA...................   417    1    te1
......................................................................uaauacugucagCuaaugaug...................   1      1    te1
......................................................................uaauUcugucagguaaugaug...................   1      1    te1
......................................................................uCauacugucagguaaugaug...................   1      1    te1
......................................................................uaauacugucaCguaaugaug...................   1      1    te1
......................................................................uaauacugucagguaaugGug...................   9      1    te1
......................................................................uaauacugucagguaauCaug...................   6      1    te1
......................................................................uaauacugucagguUaugaug...................   1      1    te1
......................................................................uaauacugucagguGaugaug...................   1      1    te1
......................................................................uaauacugucagguaaugauU...................   95     1    te1
......................................................................Aaauacugucagguaaugaug...................   5      1    te1
......................................................................uaauacugucagguaaugUug...................   4      1    te1
......................................................................uaauacugucagguaaugaug...................   15311  0    te1
......................................................................uaauacugucagguaauAaug...................   3      1    te1
......................................................................uaGuacugucagguaaugaug...................   6      1    te1
......................................................................uaauacuCucagguaaugaug...................   3      1    te1
......................................................................uaauacugucagguaaugaCg...................   1      1    te1
......................................................................uaauaGugucagguaaugaug...................   1      1    te1
......................................................................Caauacugucagguaaugaug...................   14     1    te1
......................................................................uaauacugucUgguaaugaug...................   2      1    te1
......................................................................uaauacugucagguaUugaug...................   2      1    te1
......................................................................uaauacugucagguaaCgaug...................   3      1    te1
......................................................................uaauacugucaAguaaugaug...................   4      1    te1
......................................................................uaauacugucGgguaaugaug...................   5      1    te1
......................................................................Gaauacugucagguaaugaug...................   6      1    te1
......................................................................uGauacugucagguaaugaug...................   7      1    te1
......................................................................uaauacugucagguaauUaug...................   5      1    te1
......................................................................uaauacugucagguaaGgaugu..................   15     1    te1
......................................................................uaauacugucagguaaugaugG..................   159    1    te1
......................................................................uaauacugucagguaaugaAgu..................   1      1    te1
......................................................................uaauacuCucagguaaugaugu..................   29     1    te1
......................................................................uaauacuguUagguaaugaugu..................   39     1    te1
......................................................................uaauacAgucagguaaugaugu..................   2      1    te1
......................................................................uaauacugucagguaaugaGgu..................   27     1    te1
......................................................................Caauacugucagguaaugaugu..................   168    1    te1
......................................................................uaaGacugucagguaaugaugu..................   38     1    te1
......................................................................uGauacugucagguaaugaugu..................   86     1    te1
......................................................................uaauCcugucagguaaugaugu..................   14     1    te1
......................................................................uaauacugucagCuaaugaugu..................   9      1    te1
......................................................................uaauacugCcagguaaugaugu..................   39     1    te1
......................................................................uaauacugucagguaaugaCgu..................   10     1    te1
......................................................................uaauacuAucagguaaugaugu..................   7      1    te1
......................................................................uaUuacugucagguaaugaugu..................   14     1    te1
......................................................................uaaCacugucagguaaugaugu..................   26     1    te1
......................................................................uaauacugGcagguaaugaugu..................   31     1    te1
......................................................................uaauacuguAagguaaugaugu..................   1      1    te1
......................................................................uaauacugucagguaauUaugu..................   30     1    te1
......................................................................uaauUcugucagguaaugaugu..................   2      1    te1
......................................................................uaauaUugucagguaaugaugu..................   33     1    te1
......................................................................uaauacugucagguaGugaugu..................   19     1    te1
......................................................................uaauacugucagguaaugauAu..................   21     1    te1
......................................................................uaauacugucagguaauCaugu..................   23     1    te1
......................................................................uaauacugucaAguaaugaugu..................   27     1    te1
......................................................................uaauacugucaggGaaugaugu..................   39     1    te1
......................................................................uaauacugucGgguaaugaugu..................   43     1    te1
......................................................................uaauacugucCgguaaugaugu..................   5      1    te1
......................................................................uaauacugucagUuaaugaugu..................   8      1    te1
......................................................................uaauacCgucagguaaugaugu..................   3      1    te1
......................................................................uaauacugucagguaaugauCu..................   4      1    te1
......................................................................uaauacugAcagguaaugaugu..................   14     1    te1
......................................................................uaauacugucagguUaugaugu..................   1      1    te1
......................................................................Aaauacugucagguaaugaugu..................   164    1    te1
......................................................................uCauacugucagguaaugaugu..................   29     1    te1
......................................................................uaauacugucagguaaugUugu..................   105    1    te1
```

Star Mature

```
uaacaaaaauuauauaauaaucuuuguagucaucuuaccaggcagcauuagauccgaggauuuaaaacucuaauacugucagguaaugaugucagcagaguuucguacaa
......................................................................uaauacugucaggCaaugaugu..................  38      1  te1
......................................................................uaauaGugucagguaaugaugu..................  17      1  te1
......................................................................uaauacugucagguaaCgaugu..................  8       1  te1
......................................................................uaauacugucagguaaugCugu..................  44      1  te1
......................................................................uaauGcugucagguaaugaugu..................  44      1  te1
......................................................................uaauacugucaUguaaugaugu..................  8       1  te1
......................................................................uaauacugucagguaauAaugu..................  14      1  te1
......................................................................uaauacugucagguaaugaugC..................  115     1  te1
......................................................................uaauacugucagguaUugaugu..................  5       1  te1
......................................................................uaauacugucaCguaaugaugu..................  14      1  te1
......................................................................uaauacugucagguGaugaugu..................  10      1  te1
......................................................................Gaauacugucagguaaugaugu..................  76      1  te1
......................................................................uaCuacugucagguaaugaugu..................  9       1  te1
......................................................................uaauacugucagguaCugaugu..................  9       1  te1
......................................................................uaauacugucUgguaaugaugu..................  20      1  te1
......................................................................uaGuacugucagguaaugaugu..................  18      1  te1
......................................................................uaauacugucagguCaugaugu..................  6       1  te1
......................................................................uUauacugucagguaaugaugu..................  13      1  te1
......................................................................uaauacugucagguaaugaugA..................  262     1  te1
......................................................................uaauacugucaggAaaugaugu..................  19      1  te1
......................................................................uaauacugucagguaaugGugu..................  78      1  te1
......................................................................uaauacGgucagguaaugaugu..................  1       1  te1
......................................................................uaauacugucagAuaaugaugu..................  15      1  te1
......................................................................uaauaAugucagguaaugaugu..................  3       1  te1
......................................................................uaauacugucagguaaugaugu..................  165282  0  te1
......................................................................uaauacugucagguaaAgaugu..................  8       1  te1
......................................................................uaauacuguGagguaaugaugu..................  29      1  te1
......................................................................uaauacugucagguaaugaugGc.................  28      1  te1
......................................................................uaaAacugucagguaaugauguc.................  2       1  te1
......................................................................uaauaGugucagguaaugauguc.................  10      1  te1
......................................................................uaauacugucagguaCugauguc.................  11      1  te1
......................................................................uaauacugucagguaaugaugAc.................  19      1  te1
......................................................................uaauacugucagguaaugauUuc.................  6       1  te1
......................................................................uaauacugucagguaaugaGguc.................  20      1  te1
......................................................................uaauacugucGgguaaugauguc.................  38      1  te1
......................................................................uaauacugucagCuaaugauguc.................  10      1  te1
......................................................................uaauacugucagguUaugauguc.................  4       1  te1
......................................................................uaauacugucagguaaugGuguc.................  118     1  te1
......................................................................uaauacugucagguaaGgauguc.................  17      1  te1
......................................................................uaauacCgucagguaaugauguc.................  7       1  te1
......................................................................uaauacugucagguaauCauguc.................  23      1  te1
......................................................................uaauacugAcagguaaugauguc.................  11      1  te1
......................................................................uaaGacugucagguaaugauguc.................  28      1  te1
......................................................................Caauacugucagguaaugauguc.................  196     1  te1
......................................................................uaauacugucaCguaaugauguc.................  23      1  te1
......................................................................uaauacugCcagguaaugauguc.................  42      1  te1
......................................................................uaauacugucagguaUugauguc.................  6       1  te1
......................................................................uaauacugucagguaGugauguc.................  17      1  te1
......................................................................uaauacugucagguaaugaugCc.................  101     1  te1
......................................................................uaUuacugucagguaaugauguc.................  20      1  te1
......................................................................uaauacugGcagguaaugauguc.................  31      1  te1
......................................................................uaauacugucagguaaugauCuc.................  21      1  te1
......................................................................uaauacugucagguaaugaAguc.................  3       1  te1
......................................................................uaauCcugucagguaaugauguc.................  20      1  te1
......................................................................uaauacugucagguaaugauAuc.................  10      1  te1
......................................................................uUauacugucagguaaugauguc.................  18      1  te1
......................................................................uaauacugucagguaaugauguc.................  200263  0  te1
......................................................................uaauacugucaAguaaugauguc.................  41      1  te1
......................................................................uaauacAgucagguaaugauguc.................  1       1  te1
......................................................................uaauacugucaggAaaugauguc.................  14      1  te1
......................................................................uaauacugucagguaaugaugG.................  27      1  te1
......................................................................uaauaUugucagguaaugauguc.................  48      1  te1
......................................................................uaauacugucagguaaAgauguc.................  9       1  te1
......................................................................uGauacugucagguaaugauguc.................  134     1  te1
......................................................................uaauacuCucagguaaugauguc.................  35      1  te1
......................................................................uaauacuAucagguaaugauguc.................  10      1  te1
......................................................................uaauacuUucagguaaugauguc.................  3       1  te1
......................................................................Aaauacugucagguaaugauguc.................  188     1  te1
......................................................................uaauacugucaUguaaugauguc.................  11      1  te1
......................................................................uaauaAugucagguaaugauguc.................  1       1  te1
```

Star **Mature**

uaacaaaauuauauaauaaucuuuguagucaucuuaccaggcagcauuagauccgaggauuuaaaacucuaauacugucagguaaugaugucagcagaguuucguacaa			
....uaauacugucagguaauUauguc.................	31	1	te1
....uaauacugucagguaaugauguA.................	322	1	te1
....uaauacugucagguaaugCuguc.................	55	1	te1
....uaauacugucagguaauAauguc.................	16	1	te1
....uaauacugucagguGaugauguc.................	9	1	te1
....uaauUcugucagguaaugauguc.................	5	1	te1
....uaauacugucUgguaaugauguc.................	17	1	te1
....uaauacugucaggCaaugauguc.................	31	1	te1
....uaGuacugucagguaaugauguc.................	69	1	te1
....uaauacugucagAuaaugauguc.................	7	1	te1
....uaauacugucagguaaugaCguc.................	29	1	te1
....uaauacugucagguaaugauguU.................	3918	1	te1
....uaCuacugucagguaaugauguc.................	36	1	te1
....uaauacGgucagguaaugauguc.................	5	1	te1
....uaauacugucagguaaCgauguc.................	11	1	te1
....uaauacugucagguCaugauguc.................	5	1	te1
....uaaCacugucagguaaugauguc.................	28	1	te1
....uaauacugucagUuaaugauguc.................	13	1	te1
....Gaauacugucagguaaugauguc.................	85	1	te1
....uCauacugucagguaaugauguc.................	38	1	te1
....uaauacugucCgguaaugauguc.................	9	1	te1
....uaauacuguAagguaaugauguc.................	1	1	te1
....uaauacuguGagguaaugauguc.................	19	1	te1
....uaauacugucaggGaaugauguc.................	51	1	te1
....uaauacugucagguaaugUuguc.................	100	1	te1
....uaauGcugucagguaaugauguc.................	85	1	te1
....uaauacuguUagguaaugauguc.................	32	1	te1
....uaauacugucagguaaugaugCca................	4	1	te1
....uaauacuguGagguaaugauguca................	1	1	te1
....uaauacugucagguaaugaCguca................	1	1	te1
....uaauacuguUagguaaugauguca................	1	1	te1
....Caauacugucagguaaugauguca................	1	1	te1
....uGauacugucagguaaugauguca................	3	1	te1
....uaauacugucagguaaugaugucU................	10849	1	te1
....uaauacugucagguaaugauCuca................	1	1	te1
....uaauGcugucagguaaugauguca................	1	1	te1
....uaauacugucagguaaugauguAa................	7	1	te1
....uaauacugucagguaaugaGguca................	1	1	te1
....uaauacugucagguaaugauguca................	2091	0	te1
....uaauacugucagguaaugaugucC................	103	1	te1
....uaauacugucagguaaugauguUa................	24	1	te1
....uaauacugucagguaauUauguca................	2	1	te1
....Aaauacugucagguaaugauguca................	5	1	te1
....uaauacugucagguaaugGuguca................	3	1	te1
....uaauacugucaggGaaugauguca................	2	1	te1
....uaauacugGcagguaaugauguca................	1	1	te1
....uaauacugucagguaaugaugucG................	307	1	te1
....uaauacugucagguaaugaugucaA...............	12	1	te1
....uaauacugucagguaaugaugucUg...............	3	1	te1
....uaauacugucagguaaugaugucaU...............	29	1	te1
....uaauacugucagguaaugaugucaC...............	3	1	te1
....uaauacugucagguaaugaugucUgc..............	1	1	te1
....aauacugucagguaauga.....................	1	0	te1
....aauacugucagguaaugau....................	11	0	te1
....aauacugucagguaaugCug...................	1	1	te1
....aauacugucagguaaugaGg...................	1	1	te1
....Uauacugucagguaaugaug...................	3	1	te1
....aauacugucagguaaugaug...................	110	0	te1
....aauacugucagguaaugUug...................	1	1	te1
....aauacugucagguaaugaCg...................	1	1	te1
....aauCcugucagguaaugaug...................	1	1	te1
....aauacugucagguaaugauA...................	2	1	te1
....aauacugucagguaaugaugu..................	1475	0	te1
....aauacugucagguaaugaugG..................	1	1	te1
....aauacugucagguaaugGugu..................	1	1	te1
....Uauacugucagguaaugaugu..................	19	1	te1
....aGuacugucagguaaugaugu..................	2	1	te1
....aauacugucagguaaugaugA..................	3	1	te1
....aauacugucagguaaugauAu..................	1	1	te1
....aauacugucagguaaugaugC..................	2	1	te1

Star **Mature**

```
uaacaaaaauuauauaauaaucuuuguagucaucuuaccaggcagcauuagauccgaggauuuaaaacucuaauacugucagguaaugaugucagcagaguuucguacaa
.......................................................................aauacugucagAuaaugaugu..................   1      1    te1
.......................................................................aauacugucagguaaugUugu..................   2      1    te1
.......................................................................aauaGugucagguaaugaugu..................   2      1    te1
.......................................................................aauacuguUagguaaugauguc.................   2      1    te1
.......................................................................aauaGugucagguaaugauguc.................   1      1    te1
.......................................................................Uauacugucagguaaugauguc.................   27     1    te1
.......................................................................aauacugucagguaaugauguU.................   22     1    te1
.......................................................................aauacugucagguaaugauguc.................   1027   0    te1
.......................................................................aGuacugucagguaaugauguc.................   1      1    te1
.......................................................................aauCcugucagguaaugauguc.................   1      1    te1
.......................................................................aauUcugucagguaaugauguc.................   1      1    te1
.......................................................................aauacugucagguaaGgauguc.................   1      1    te1
.......................................................................aauacuCucagguaaugauguc.................   2      1    te1
.......................................................................aauacugucagguaaugauguA.................   4      1    te1
.......................................................................aauaUugucagguaaugauguc.................   1      1    te1
.......................................................................aauacugucagguaaugUuguc.................   2      1    te1
.......................................................................aCuacugucagguaaugauguc.................   1      1    te1
.......................................................................aauacugucaCguaaugauguc.................   1      1    te1
.......................................................................aauacugucagguaaugauguca................   18     0    te1
.......................................................................aauacugucagguaaugaugucU................   216    1    te1
.......................................................................aauacugucagguaaugaugucG................   3      1    te1
.......................................................................aauacugucagguaaugauguUa................   1      1    te1
.......................................................................aauacugucagguaaugaugucC................   4      1    te1
.......................................................................aauacugucagguaaugaugucaU...............   1      1    te1
........................................................................Uuacugucagguaaugaugu..................   1      1    te1
........................................................................auacugucagguaaugaugu..................   17     0    te1
........................................................................aAacugucagguaaugauguc.................   1      1    te1
........................................................................auacugucagguaaugauguc.................   32     0    te1
........................................................................Uuacugucagguaaugauguc.................   5      1    te1
........................................................................auacugucagguaaugauguca................   1      0    te1
........................................................................auacugucagguaaugaugucU................   2      1    te1
.........................................................................uacugucagguaaugaug...................   1      0    te1
.........................................................................uacugucagguaaugaugu..................   1      0    te1
.........................................................................uacugucagguaaugauguc.................   14     0    te1
.........................................................................uacugucagguaaugaugucU................   1      1    te1
..........................................................................acugucagguaaugaugu..................   8      0    te1
..........................................................................acugucagguaaugauguc.................   14     0    te1
..........................................................................Ucugucagguaaugauguc.................   1      1    te1
..........................................................................acugucagguaaugaugucU................   5      1    te1
..........................................................................acugucagguaaugaugucG................   2      1    te1
...........................................................................cugucagguaaugauguc.................   35     0    te1
...........................................................................cugucagguaaugaugucU................   2      1    te1
............................................................................ugucagguaaugaugucU................   1      1    te1
............................................................................ugucagguaaugauguca................   1      0    te1

...........................agGcaucuuaccaggcagcauuaga..........................................................   1      1    T6P
.............................ucaucuuaccaggcagcauu.............................................................   32     0    T6P
.............................ucaucuuaccaggcagcauua............................................................   37     0    T6P
.............................ucaucuGaccaggcagcauua............................................................   1      1    T6P
.............................ucaucuuaccaggcagcauuU............................................................   2      1    T6P
.............................ucaucuuaccaggcagcauuag...........................................................   50     0    T6P
.............................ucaucuuaccaggcagcauuaU...........................................................   1      1    T6P
.............................ucaucuuaccaggcagcauuaga..........................................................   12     0    T6P
.............................ucaucuuaccaggcagcauuagaA.........................................................   1      1    T6P
..............................caucuuaccaggcagcau..............................................................   1      0    T6P
..............................caucuuaccaggcaCcauu.............................................................   1      1    T6P
..............................caucuuaccaUgcagcauu.............................................................   1      1    T6P
..............................caucuuaccaggcagcauu.............................................................   218    0    T6P
..............................caucuuaccGggcagcauu.............................................................   1      1    T6P
..............................caucuuaccaggcagUauua............................................................   1      1    T6P
..............................caucuuaccaggcagcauua............................................................   426    0    T6P
..............................caucuuaccaggcagcauAa............................................................   1      1    T6P
..............................caucuuaccaggcagcauuU............................................................   2      1    T6P
..............................caucuuaccaggcagcauuaA...........................................................   22     1    T6P
..............................caucuuaccaggcaCcauuag...........................................................   1      1    T6P
..............................Uaucuuaccaggcagcauuag...........................................................   2      1    T6P
..............................caucuuaccagCcagcauuag...........................................................   1      1    T6P
..............................caucuuaccaggcagcauuaC...........................................................   3      1    T6P
..............................caucuuaccaggcagUauuag...........................................................   2      1    T6P
..............................caucuuaccaggcaUcauuag...........................................................   2      1    T6P
```

```
                              Star                    Mature
uaacaaaaauuauauaauaaucuuuguagucaucuuaccaggcagcauuagauccgaggauuuaaaacucuaauacugucagguaaugaugucagcagaguuucguacaa
..............................caucuuaccagAcagcauuag...........................................................   1      1    T6P
..............................caucuuacUaggcagcauuag...........................................................   1      1    T6P
..............................caucuuaccaggUagcauuag...........................................................   1      1    T6P
..............................caucuuaccaUgcagcauuag...........................................................   3      1    T6P
..............................caucuuaccaggcagcauuag...........................................................   2514   0    T6P
..............................Gaucuuaccaggcagcauuag...........................................................   1      1    T6P
..............................caucuuaccagUcagcauuag...........................................................   1      1    T6P
..............................caucuuaccaggcagcauuaU...........................................................   2      1    T6P
..............................caucuuaccGggcagcauuag...........................................................   11     1    T6P
..............................caucuuaccaggcagcaCuag...........................................................   1      1    T6P
..............................caucuuaccaggcagcUuuag...........................................................   1      1    T6P
..............................caucuuaccaggcagcauuGg...........................................................   1      1    T6P
..............................caucuuaccaggcCgcauuag...........................................................   2      1    T6P
..............................caucuuaccaggUagcauuaga..........................................................   3      1    T6P
..............................caucuuaUcaggcagcauuaga..........................................................   1      1    T6P
..............................caucuuaccaggcagcauuagG..........................................................   5      1    T6P
..............................caucuuaccaggcaUcauuaga..........................................................   1      1    T6P
..............................caucuuaccaUgcagcauuaga..........................................................   1      1    T6P
..............................caucuuaccaggcCgcauuaga..........................................................   2      1    T6P
..............................caucuuaccaggcagUauuaga..........................................................   6      1    T6P
..............................Uaucuuaccaggcagcauuaga..........................................................   2      1    T6P
..............................caucuuaccaggcagcauuagU..........................................................   28     1    T6P
..............................caucuuaccaggcagcUuuaga..........................................................   5      1    T6P
..............................cauUuuaccaggcagcauuaga..........................................................   4      1    T6P
..............................caucuuaccagAcagcauuaga..........................................................   3      1    T6P
..............................caucuuaccagUcagcauuaga..........................................................   1      1    T6P
..............................caucuuaccaggcagcauuaga..........................................................   5825   0    T6P
..............................caucuuaccaggcagcauuaAa..........................................................   3      1    T6P
..............................caucuuacUaggcagcauuaga..........................................................   1      1    T6P
..............................caucuuaccagCcagcauuaga..........................................................   3      1    T6P
..............................caucuuaccCggcagcauuaga..........................................................   1      1    T6P
..............................caAcuuaccaggcagcauuaga..........................................................   1      1    T6P
..............................caucuuaccaggcGgcauuaga..........................................................   2      1    T6P
..............................caucuuaccaggcaCcauuaga..........................................................   2      1    T6P
..............................caucuuGccaggcagcauuaga..........................................................   3      1    T6P
..............................caucuuaccaggcagcauuaCa..........................................................   2      1    T6P
..............................caucuuaccaggcagcCuuaga..........................................................   1      1    T6P
..............................caucuuaccGggcagcauuaga..........................................................   25     1    T6P
..............................caucuuaccaggcagcauuagaC.........................................................   6      1    T6P
..............................caucuuaccaggcagcauuagUu.........................................................   1      1    T6P
..............................caucuuaccaggcagcauuagau.........................................................   20     0    T6P
..............................caucuuaccaggcagcauuagaA.........................................................   125    1    T6P
..............................caucuuaccaggcagcauuagauA........................................................   2      1    T6P
..............................caucuuaccaggcagcauuagaAc........................................................   3      1    T6P
..............................caucuuaccaggcagcauuagauU........................................................   4      1    T6P
..............................caucuuaccaggcagcauuagaucc.......................................................   2      0    T6P
..............................caucuuaccaggcagcauuagauccga.....................................................   1      0    T6P
...............................aucuuaccaggcagcauuag...........................................................   1      0    T6P
...............................aucuuaccaggcagcauuaga..........................................................   10     0    T6P
...............................Cucuuaccaggcagcauuaga..........................................................   1      1    T6P
...............................aucuuaccaggcagcauuagaA.........................................................   1      1    T6P
...............................aucuuaccaggcagcauuagauU........................................................   1      1    T6P
...............................aucuuaccaggcagcauuagauccgag....................................................   1      0    T6P
................................ucuuaccaggcagcauuaga..........................................................   1      0    T6P
................................ucuuaccaggcagcauuagau.........................................................   3      0    T6P
................................ucuuaccaggcagcauuagauc........................................................   13     0    T6P
.................................cuuaccaggcagcauuag...........................................................   2      0    T6P
..................................uuaccaggcagcauuaga..........................................................   1      0    T6P
...................................................................cuUuaauacugucagguaaugaugu..................   1      1    T6P
...................................................................cuUuaauacugucagguaaugauguc.................   1      1    T6P
....................................................................uUuaauacugucagguaaugaug...................   1      1    T6P
....................................................................uUuaauacugucagguaaugaugu..................   8      1    T6P
....................................................................ucuaauacugucagguaaugaugu..................   1      0    T6P
....................................................................uUuaauacugucagguaaugauguc.................   3      1    T6P
.....................................................................cuaauacugucagguaaugau....................   2      0    T6P
.....................................................................cuaauacugucagguaaugaug...................   3      0    T6P
.....................................................................Uuaauacugucagguaaugaug...................   2      1    T6P
.....................................................................Auaauacugucagguaaugaugu..................   1      1    T6P
.....................................................................Uuaauacugucagguaaugaugu..................   42     1    T6P
.....................................................................cuaauacugucagguaaugaugu..................   7      0    T6P
```

Star **Mature**

uaacaaaaauuauauaauaaucuuuguagucaucuuaccaggcagcauuagauccgaggauuuaaaacucuaauacugucagguaaugaugucagcagaguuucguacaa

....cuaauacugucagguaaugauguU.................	1	1	T6P
....Uuaauacugucagguaaugauguc.................	31	1	T6P
....uaauacugucagguaaug......................	16	0	T6P
....uaauacugucagguaauA......................	2	1	T6P
....uaauacugucagguaaugU.....................	4	1	T6P
....uaauacugucagguaauga.....................	73	0	T6P
....uaauacugucagguaauAa.....................	1	1	T6P
....uaauacugucagguaaAga.....................	1	1	T6P
....uaauacugucaUguaaugau....................	1	1	T6P
....uaauacCgucagguaaugau....................	1	1	T6P
....uaauacugucagguaaugaC....................	1	1	T6P
....uaauacuguUagguaaugau....................	1	1	T6P
....uaauacugucagguaaugUu....................	2	1	T6P
....uaauacugucagguaaugaG....................	1	1	T6P
....uaauacugucagguaaAgau....................	7	1	T6P
....uaauacugucagguaaugCu....................	1	1	T6P
....uaauacugucagguaUugau....................	1	1	T6P
....uaauacuguAagguaaugau....................	1	1	T6P
....uaauacugucagUuaaugau....................	1	1	T6P
....uaauacugucagguaaugaA....................	14	1	T6P
....uaauacugucagguaaugau....................	1713	0	T6P
....uaauacugucagguaaugaCg...................	1	1	T6P
....uaauacugucaCguaaugaug...................	2	1	T6P
....uaauacugucGgguaaugaug...................	3	1	T6P
....uaGuacugucagguaaugaug...................	1	1	T6P
....uaauaAugucagguaaugaug...................	1	1	T6P
....uaauacugucagguaaugauA...................	165	1	T6P
....uaauacugucaUguaaugaug...................	3	1	T6P
....uaauacugucagguaaCgaug...................	1	1	T6P
....uaauacugucagguaaugCug...................	2	1	T6P
....uaauaUugucagguaaugaug...................	1	1	T6P
....uaUuacugucagguaaugaug...................	1	1	T6P
....uaauUcugucagguaaugaug...................	4	1	T6P
....uaauacugucagguaaAgaug...................	87	1	T6P
....uaauacugucagguaaugaGg...................	1	1	T6P
....uaauacugucagguaCugaug...................	1	1	T6P
....uaauacugucaggGaaugaug...................	4	1	T6P
....uaauacugucagguaGugaug...................	1	1	T6P
....uaauacGgucagguaaugaug...................	8	1	T6P
....uaauacugucagguaaugauU...................	73	1	T6P
....uaauacugucaggCaaugaug...................	3	1	T6P
....uaauacugucaggAaaugaug...................	3	1	T6P
....Aaauacugucagguaaugaug...................	1	1	T6P
....uaauacugucagCuaaugaug...................	1	1	T6P
....uaauacugucagguaauUaug...................	9	1	T6P
....uaCuacugucagguaaugaug...................	1	1	T6P
....uaaGacugucagguaaugaug...................	2	1	T6P
....uaauacuguAagguaaugaug...................	2	1	T6P
....uaauacugucagguaaugauC...................	8	1	T6P
....uaauacAgucagguaaugaug...................	1	1	T6P
....uaauacugucagguaauCaug...................	15	1	T6P
....uaauacugucUgguaaugaug...................	2	1	T6P
....uaauacugucagguaaugGug...................	2	1	T6P
....Caauacugucagguaaugaug...................	1	1	T6P
....uaauacugucagguaaGgaug...................	2	1	T6P
....uaauacugucagguGaugaug...................	2	1	T6P
....uaauacugucaAguaaugaug...................	2	1	T6P
....uaauacuguUagguaaugaug...................	2	1	T6P
....uaauacCgucagguaaugaug...................	3	1	T6P
....uaauacugucagguCaugaug...................	1	1	T6P
....uaauGcugucagguaaugaug...................	1	1	T6P
....uaauacugucagguaaugaug...................	15001	0	T6P
....uaauacugucagUuaaugaug...................	4	1	T6P
....uaauacugucagguaauAaug...................	1	1	T6P
....uaauacugCcagguaaugaug...................	1	1	T6P
....uaauacugucagguaaugUug...................	1	1	T6P
....uaauacugucaggAaaugaugu..................	63	1	T6P
....uaauCcugucagguaaugaugu..................	1	1	T6P
....uaauaAugucagguaaugaugu..................	5	1	T6P
....uaauacugucagguaaugauUu..................	28	1	T6P

```
                              Star                                      Mature
uaacaaaaauuauauaauaaucuuuguagucaucuuaccaggcagcauuagauccgaggauuuaaaacucuaauacugucagguaaugaugucagcagaguuucguacaa
......................................................................Caauacugucagguaaugaugu..................  10      1   T6P
......................................................................uaauacugucagguaaugaugA..................  152     1   T6P
......................................................................uaUuacugucagguaaugaugu..................  11      1   T6P
......................................................................uaauacugucagguaaCgaugu..................  3       1   T6P
......................................................................uaauUcugucagguaaugaugu..................  28      1   T6P
......................................................................uaauacugAcagguaaugaugu..................  9       1   T6P
......................................................................uaauacugucagguaaugaugu..................  230155  0   T6P
......................................................................uaauacugucagUuaaugaugu..................  53      1   T6P
......................................................................uaauacugucaggGaaugaugu..................  66      1   T6P
......................................................................uaauacugucagguaaugauCu..................  44      1   T6P
......................................................................uaauacuUucagguaaugaugu..................  14      1   T6P
......................................................................uaaAacugucagguaaugaugu..................  3       1   T6P
......................................................................uaauacugucagguaaugaAgu..................  4       1   T6P
......................................................................uaauacugucagguaauCaugu..................  100     1   T6P
......................................................................uaauacuguAagguaaugaugu..................  42      1   T6P
......................................................................uaauacugucagguaaugUugu..................  9       1   T6P
......................................................................uaauacugucagguaUugaugu..................  49      1   T6P
......................................................................Aaauacugucagguaaugaugu..................  17      1   T6P
......................................................................uaauacuCucagguaaugaugu..................  8       1   T6P
......................................................................uaauacuguGagguaaugaugu..................  9       1   T6P
......................................................................uaauacuAucagguaaugaugu..................  6       1   T6P
......................................................................uaauacugucUgguaaugaugu..................  56      1   T6P
......................................................................uaauacugucagguaCugaugu..................  2       1   T6P
......................................................................uaauacugucaCguaaugaugu..................  72      1   T6P
......................................................................uaauGcugucagguaaugaugu..................  28      1   T6P
......................................................................uaaCacugucagguaaugaugu..................  12      1   T6P
......................................................................uaauacugucagguaaugaugG..................  11      1   T6P
......................................................................uaauacugCcagguaaugaugu..................  15      1   T6P
......................................................................uaauacugucaUguaaugaugu..................  65      1   T6P
......................................................................uaauacugucagguGaugaugu..................  18      1   T6P
......................................................................uaaGacugucagguaaugaugu..................  15      1   T6P
......................................................................uaauacugucagguaaugGugu..................  33      1   T6P
......................................................................uaauacugucagguaauAaugu..................  41      1   T6P
......................................................................uaauacugucagguaaugauAu..................  60      1   T6P
......................................................................uaauaGugucagguaaugaugu..................  4       1   T6P
......................................................................uaGuacugucagguaaugaugu..................  11      1   T6P
......................................................................uaauacugucagAuaaugaugu..................  33      1   T6P
......................................................................uaauacugucagCuaaugaugu..................  36      1   T6P
......................................................................uaauacAgucagguaaugaugu..................  42      1   T6P
......................................................................uaauacugucagguUaugaugu..................  6       1   T6P
......................................................................uaauacugucagguCaugaugu..................  7       1   T6P
......................................................................uaauacugucagguaaugCugu..................  9       1   T6P
......................................................................uaauacugucagguaaugaugC..................  66      1   T6P
......................................................................uaCuacugucagguaaugaugu..................  18      1   T6P
......................................................................uaauacugGcagguaaugaugu..................  3       1   T6P
......................................................................uaauacugucagguaaugaGgu..................  8       1   T6P
......................................................................uaauacugucCgguaaugaugu..................  5       1   T6P
......................................................................uaauacugucGgguaaugaugu..................  45      1   T6P
......................................................................uaauacugucaAguaaugaugu..................  41      1   T6P
......................................................................uaauacCgucagguaaugaugu..................  53      1   T6P
......................................................................uGauacugucagguaaugaugu..................  9       1   T6P
......................................................................uUauacugucagguaaugaugu..................  5       1   T6P
......................................................................uaauacugucagguaauUaugu..................  121     1   T6P
......................................................................uaauacuguUagguaaugaugu..................  95      1   T6P
......................................................................uaauacugucagguaaGgaugu..................  10      1   T6P
......................................................................uaauaUugucagguaaugaugu..................  35      1   T6P
......................................................................uaauacugucagguaaugaCgu..................  11      1   T6P
......................................................................uaauacugucagguaGugaugu..................  6       1   T6P
......................................................................uaauacugucagguaaAgaugu..................  296     1   T6P
......................................................................uaauacGgucagguaaugaugu..................  116     1   T6P
......................................................................Gaauacugucagguaaugaugu..................  8       1   T6P
......................................................................uaauacugucaggCaaugaugu..................  75      1   T6P
......................................................................uaauacugucagguaaugUuguc.................  17      1   T6P
......................................................................Aaauacugucagguaaugauguc.................  8       1   T6P
......................................................................uaauacugucagguaaugauUuc.................  13      1   T6P
......................................................................uaauacugucagguaaugGuguc.................  20      1   T6P
......................................................................uaauacugucagguaaugauguU.................  5268    1   T6P
......................................................................uaauacugucagguaGugauguc.................  2       1   T6P
......................................................................uaGuacugucagguaaugauguc.................  15      1   T6P
......................................................................uaauacugucUgguaaugauguc.................  23      1   T6P
```

```
                              Star                                      Mature
uaacaaaaauuauauaauaaucuuuguagucaucuuaccaggcagcauuagauccgaggauuuaaaacucuaauacugucagguaaugaugucagcagaguuucguacaa
......................................................................uaauacugucagguaaugauguG.................    8       1     T6P
......................................................................uaaGacugucagguaaugauguc.................    13      1     T6P
......................................................................uaauacugucGgguaaugauguc.................    26      1     T6P
......................................................................uaauacugucaAguaaugauguc.................    23      1     T6P
......................................................................uaauacugucagUuaaugauguc.................    59      1     T6P
......................................................................uaauacuCucagguaaugauguc.................    4       1     T6P
......................................................................uaauacugucagguaaugaGguc.................    3       1     T6P
......................................................................uaauaUugucagguaaugauguc.................    21      1     T6P
......................................................................uaauacugCcagguaaugauguc.................    7       1     T6P
......................................................................uaauaAugucagguaaugauguc.................    1       1     T6P
......................................................................uaauacugucagguaaAgauguc.................    1509    1     T6P
......................................................................uaauacugucagguCaugauguc.................    5       1     T6P
......................................................................uaauacugucaCguaaugauguc.................    40      1     T6P
......................................................................uaauacugucagguaaugaCguc.................    5       1     T6P
......................................................................uaauacugucagguaaugauguA.................    316     1     T6P
......................................................................uaauacugucagguaaugaAguc.................    4       1     T6P
......................................................................uaUuacugucagguaaugauguc.................    16      1     T6P
......................................................................uaauacugucagguaaGgauguc.................    6       1     T6P
......................................................................Gaauacugucagguaaugauguc.................    2       1     T6P
......................................................................uaCuacugucagguaaugauguc.................    19      1     T6P
......................................................................uaaCacugucagguaaugauguc.................    5       1     T6P
......................................................................uaauacugucCgguaaugauguc.................    2       1     T6P
......................................................................uaauacugucagguaCugauguc.................    4       1     T6P
......................................................................uaauacugucagguaaCgauguc.................    12      1     T6P
......................................................................uaauacugucagCuaaugauguc.................    58      1     T6P
......................................................................uaauacuguAagguaaugauguc.................    22      1     T6P
......................................................................uaauacugucaggGaaugauguc.................    39      1     T6P
......................................................................uaauacugucagAuaaugauguc.................    20      1     T6P
......................................................................uaauacAgucagguaaugauguc.................    31      1     T6P
......................................................................Caauacugucagguaaugauguc.................    9       1     T6P
......................................................................uaaAacugucagguaaugauguc.................    6       1     T6P
......................................................................uaauacugucagguaUugauguc.................    24      1     T6P
......................................................................uaauacuguUagguaaugauguc.................    43      1     T6P
......................................................................uaauacugGcagguaaugauguc.................    1       1     T6P
......................................................................uaauacuUucagguaaugauguc.................    7       1     T6P
......................................................................uaauacugucagguaaugauguc.................    141707  0     T6P
......................................................................uaauacugucagguaauAauguc.................    15      1     T6P
......................................................................uaauUcugucagguaaugauguc.................    29      1     T6P
......................................................................uaauacuAucagguaaugauguc.................    2       1     T6P
......................................................................uCauacugucagguaaugauguc.................    2       1     T6P
......................................................................uGauacugucagguaaugauguc.................    5       1     T6P
......................................................................uaauacugucagguaaugaugGc.................    5       1     T6P
......................................................................uaauacugucagguaaugCuguc.................    6       1     T6P
......................................................................uaauacugucagguUaugauguc.................    14      1     T6P
......................................................................uaauacugucagguaaugauCuc.................    36      1     T6P
......................................................................uaauacugucagguaaugaugCc.................    35      1     T6P
......................................................................uaauCcugucagguaaugauguc.................    1       1     T6P
......................................................................uaauGcugucagguaaugauguc.................    30      1     T6P
......................................................................uaauacugucagguaauUauguc.................    211     1     T6P
......................................................................uUauacugucagguaaugauguc.................    9       1     T6P
......................................................................uaauacCgucagguaaugauguc.................    16      1     T6P
......................................................................uaauacugucagguGaugauguc.................    12      1     T6P
......................................................................uaauacugucaggCaaugauguc.................    34      1     T6P
......................................................................uaauacugucaUguaaugauguc.................    111     1     T6P
......................................................................uaauacGgucagguaaugauguc.................    50      1     T6P
......................................................................uaauacugucagguaaugaugAc.................    14      1     T6P
......................................................................uaauacugucagguaaugauAuc.................    20      1     T6P
......................................................................uaauacuguGagguaaugauguc.................    3       1     T6P
......................................................................uaauacugucagguaauCauguc.................    156     1     T6P
......................................................................uaauacugucaggAaaugauguc.................    36      1     T6P
......................................................................uaauacuguUagguaaugauguca................    1       1     T6P
......................................................................uaauacugucagguaauCauguca................    5       1     T6P
......................................................................uaauacugucagguaaugauguUa................    30      1     T6P
......................................................................uaauGcugucagguaaugauguca................    1       1     T6P
......................................................................uaauUcugucagguaaugauguca................    1       1     T6P
......................................................................uaauacugucGgguaaugauguca................    1       1     T6P
......................................................................uaauacugucagguaaugaCguca................    1       1     T6P
......................................................................uaauacAgucagguaaugauguca................    1       1     T6P
......................................................................uaauacugucagguaaugauguca................    2096    0     T6P
......................................................................uaauacugucagUuaaugauguca................    1       1     T6P
```

```
                                             Star                                        Mature
uaacaaaaauuauauaauaaucuuuguagucaucuuaccaggcagcauuagauccgaggauuuaaaacucuaauacugucagguaaugaugucagcagaguuucguacaa
......................................................................uaauacugucagguaaugaugucC................   117    1   T6P
......................................................................uaauacugucagguaaugaugucU................   5014   1   T6P
......................................................................uaauacugucaggCaaugauguca................   1      1   T6P
......................................................................uaauacugucagguaauUauguca................   4      1   T6P
......................................................................uaauacugucagguaaugCuguca................   1      1   T6P
......................................................................uaGuacugucagguaaugauguca................   1      1   T6P
......................................................................uaauacugucagguaaAgauguca................   10     1   T6P
......................................................................uaauacugucagAuaaugauguca................   1      1   T6P
......................................................................uaauaAugucagguaaugauguca................   1      1   T6P
......................................................................uaauacugucagguaaugaugCca................   6      1   T6P
......................................................................uaauacugucagguaaugaugucG................   57     1   T6P
......................................................................uaauacugucagCuaaugauguca................   1      1   T6P
......................................................................uaauacugucagguaaugaugAca................   1      1   T6P
......................................................................uaauacugucagguaaugauguAa................   2      1   T6P
......................................................................uaauacugucagguaaugauCuca................   1      1   T6P
......................................................................uaauacugucagguaaugaugucaA...............   7      1   T6P
......................................................................uaauacugucagguaaugaugucag...............   1      0   T6P
......................................................................uaauacugucagguaaugaugucaC...............   5      1   T6P
......................................................................uaauacugucagguaaugaugucaU...............   30     1   T6P
......................................................................uaauacugucagguaaugaugucagc..............   1      0   T6P
.......................................................................aauacugucagguaaugau....................   4      0   T6P
.......................................................................aauacugucagguaaugaug...................   38     0   T6P
.......................................................................Uauacugucagguaaugaug...................   2      1   T6P
.......................................................................aauacugucagguaaugauA...................   1      1   T6P
.......................................................................aauacugucagguaaugauU...................   1      1   T6P
.......................................................................aauacugucagguGaugaugu..................   1      1   T6P
.......................................................................aauacugucaUguaaugaugu..................   1      1   T6P
.......................................................................aauacugucagguaaugaAgu..................   1      1   T6P
.......................................................................aauacugucagguaaugauAu..................   1      1   T6P
.......................................................................aauacugucagguaaugaGgu..................   1      1   T6P
.......................................................................aauacugucagguaaugaugC..................   1      1   T6P
.......................................................................aauUcugucagguaaugaugu..................   2      1   T6P
.......................................................................aauacugucagguUaugaugu..................   1      1   T6P
.......................................................................aauacugucagguaaugaugu..................   649    0   T6P
.......................................................................aauacugucagguaaugauCu..................   1      1   T6P
.......................................................................aauacugucagguaUugaugu..................   1      1   T6P
.......................................................................Uauacugucagguaaugaugu..................   12     1   T6P
.......................................................................aauacuguUagguaaugauguc.................   1      1   T6P
.......................................................................aauacugucagguaaugauguA.................   1      1   T6P
.......................................................................aauacugucagUuaaugauguc.................   1      1   T6P
.......................................................................aauacugucGgguaaugauguc.................   1      1   T6P
.......................................................................aauacugucagguaUugauguc.................   1      1   T6P
.......................................................................aauacugucagguaauCauguc.................   2      1   T6P
.......................................................................aauacugucagguaaugauguc.................   789    0   T6P
.......................................................................aauacuUucagguaaugauguc.................   1      1   T6P
.......................................................................Uauacugucagguaaugauguc.................   9      1   T6P
.......................................................................aauacugucagguaaugauguU.................   24     1   T6P
.......................................................................aauacuCucagguaaugauguc.................   1      1   T6P
.......................................................................aauacugucagguGaugauguc.................   1      1   T6P
.......................................................................aauacugucagguaCugauguc.................   1      1   T6P
.......................................................................aauacugucagguaaAgauguc.................   7      1   T6P
.......................................................................aauacugucagguaaugaugucC................   10     1   T6P
.......................................................................aauacuguUagguaaugauguca................   1      1   T6P
.......................................................................aauacugucagguaaugaugucU................   150    1   T6P
.......................................................................aauacugucagguaaugauguca................   16     0   T6P
........................................................................auacugucagguaaAgaugu..................   1      1   T6P
........................................................................auacugucagguaaugaugu..................   7      0   T6P
........................................................................aAacugucagguaaugaugu..................   2      1   T6P
........................................................................Uuacugucagguaaugaugu..................   4      1   T6P
........................................................................aCacugucagguaaugauguc.................   2      1   T6P
........................................................................auacugucagguaaugauguU.................   3      1   T6P
........................................................................auacugucagguaaCgauguc.................   1      1   T6P
........................................................................auacugucagguaaugauguc.................   19     0   T6P
........................................................................Uuacugucagguaaugauguc.................   2      1   T6P
........................................................................auacugucagguaaugaugucU................   2      1   T6P
........................................................................auacugucagguaaugaugucaU...............   1      1   T6P
.........................................................................uacugucagguaaugaugu..................   5      0   T6P
.........................................................................uacugucaUguaaugauguc.................   1      1   T6P
.........................................................................uacugucGgguaaugauguc.................   1      1   T6P
.........................................................................uacugucagguaaugauguc.................   14     0   T6P
```

```
                              Star                                      Mature
uaacaaaaauuauauaauaaucuuuguagucaucuuaccaggcagcauuagauccgaggauuuaaaacucuaauacugucagguaaugaugucagcagaguuucguacaa
.........................................................................uacugucagguaaugaugucU................  2    1   T6P
..........................................................................acugucagguaaugaugu..................  6    0   T6P
..........................................................................acugucagguaaugauguc.................  10   0   T6P
..........................................................................acugucagguaaugaugucU................  1    1   T6P
...........................................................................cugucagguaaugauguc.................  22   0   T6P
...........................................................................cugucagguaaugaugucU................  5    1   T6P
...........................................................................cugucagguaaugauguca................  1    0   T6P
............................................................................ugucagguaaugauguca................  1    0   T6P

.............................ucaucuuaccaggcagcauuag...........................................................  1    0   egg
..............................caucuuaccaggcagcauu.............................................................  9    0   egg
..............................caucuuaccaggcagcGuuag...........................................................  1    1   egg
..............................caucuuaccaggcagcauuag...........................................................  28   0   egg
..............................caucuuaccGggcagcauuaga..........................................................  1    1   egg
..............................caucuuaccaggcagcGuuaga..........................................................  1    1   egg
..............................Uaucuuaccaggcagcauuaga..........................................................  1    1   egg
..............................caucuuaccaggcagcauuagU..........................................................  1    1   egg
..............................caucuuGccaggcagcauuaga..........................................................  1    1   egg
..............................caCcuuaccaggcagcauuaga..........................................................  1    1   egg
..............................caucuuaccaAgcagcauuaga..........................................................  1    1   egg
..............................caucuuaccaggcagcauuaga..........................................................  97   0   egg
..............................caucGuaccaggcagcauuaga..........................................................  5    1   egg
..............................caucCuaccaggcagcauuaga..........................................................  1    1   egg
..............................caucuuaccaggUagcauuaga..........................................................  2    1   egg
..............................caucuuaccaggcagcauuagau.........................................................  2    0   egg
..............................caucuuaccaggcagcauuagaA.........................................................  5    1   egg
..............................caucuuaccaggcagcauuagauU........................................................  1    1   egg
...............................aucuuaccaggcagcauuaga..........................................................  1    0   egg
............................................gcauuagauccgaggauuuaaaacuc........................................  4    0   egg
......................................................................uaauacugucagguaauga.....................  3    0   egg
......................................................................uaauacugucagguaaugaC....................  2    1   egg
......................................................................uaaGacugucagguaaugau....................  1    1   egg
......................................................................uaauacugucagguaaugau....................  4    0   egg
......................................................................uaauacugucagguaaugaug...................  61   0   egg
......................................................................uaauacugucaggCaaugaug...................  1    1   egg
......................................................................uaauacugucagguaaugauA...................  4    1   egg
......................................................................uaauUcugucagguaaugaug...................  1    1   egg
......................................................................uGauacugucagguaaugaug...................  1    1   egg
......................................................................uaauacugucagguaaugauC...................  1    1   egg
......................................................................uaauacugucagguaaugauU...................  1    1   egg
......................................................................uaauacugucagguaaAgaug...................  1    1   egg
......................................................................uaauacugucagguaaugGugu..................  1    1   egg
......................................................................uaauacugucagguaaugaugC..................  8    1   egg
......................................................................uaauacugucagguaaugaugu..................  82   0   egg
......................................................................uaaCacugucagguaaugaugu..................  1    1   egg
......................................................................uaauacugucagguaaAgaugu..................  7    1   egg
......................................................................uaaGacugucagguaaugaugu..................  2    1   egg
......................................................................uaauacugucagguaauAaugu..................  1    1   egg
......................................................................uaauacugucagguaaugaugA..................  1    1   egg
......................................................................uaauacugucagguaaugaCgu..................  2    1   egg
......................................................................uGauacugucagguaaugaugu..................  1    1   egg
......................................................................uaauacuAucagguaaugaugu..................  1    1   egg
......................................................................uaaAacugucagguaaugaugu..................  1    1   egg
......................................................................uaauUcugucagguaaugaugu..................  1    1   egg
......................................................................Naauacugucagguaaugaugu..................  1    1   egg
......................................................................uaauacCgucagguaaugauguc.................  1    1   egg
......................................................................uaaAacugucagguaaugauguc.................  1    1   egg
......................................................................uaauacugucagguaaugGuguc.................  2    1   egg
......................................................................uaauacugucagguCaugauguc.................  1    1   egg
......................................................................uaauacugucUgguaaugauguc.................  1    1   egg
......................................................................uaauacugucagguaaugaCguc.................  1    1   egg
......................................................................uaaCacugucagguaaugauguc.................  6    1   egg
......................................................................uaauacugucagguaaugauguU.................  16   1   egg
......................................................................uaauacugucGgguaaugauguc.................  1    1   egg
......................................................................uaauacugucagAuaaugauguc.................  1    1   egg
......................................................................Caauacugucagguaaugauguc.................  4    1   egg
......................................................................uaauacugucagguaaugauguc.................  395  0   egg
......................................................................uaauacugucagguaaugaugCc.................  1    1   egg
......................................................................uaauacugucagguaGugauguc.................  1    1   egg
......................................................................uaauacugucagguaaugauguA.................  4    1   egg
```

Star Mature

```
uaacaaaauuauauaauaaucuuuguagucaucuuaccaggcagcauuagauccgaggauuuaaaacucuaauacugucagguaaugaugucagcagaguuucguacaa
.....................................................................uaauacugucagguaauAauguc.................   3      1   egg
.....................................................................uaauaGugucagguaaugauguc.................   1      1   egg
.....................................................................uaauacugucagguaaugaugAc.................   1      1   egg
.....................................................................uaauUcugucagguaaugauguc.................   9      1   egg
.....................................................................uaauGcugucagguaaugauguc.................   1      1   egg
.....................................................................uaaGacugucagguaaugauguc.................   11     1   egg
.....................................................................Aaauacugucagguaaugauguc.................   2      1   egg
.....................................................................uaauacugucagguaaCgauguc.................   4      1   egg
.....................................................................uCauacugucagguaaugauguc.................   1      1   egg
.....................................................................uaauacugucagguGaugauguc.................   1      1   egg
.....................................................................uaauacugucaggCaaugauguc.................   1      1   egg
.....................................................................uaauacugucagguaaAgauguc.................   8      1   egg
.....................................................................uaauacugCcagguaaugauguc.................   3      1   egg
.....................................................................uaauacugucagguaaugaugucU................   20     1   egg
.....................................................................Naauacugucagguaaugauguca................   1      1   egg
.....................................................................uaauacugucagguaaugauguca................   40     0   egg
.....................................................................uaauacugucagguaaugaugCca................   1      1   egg
.....................................................................uaauacugucagguaaugaCguca................   1      1   egg
.....................................................................uaauacugucagguaaugaugucC................   19     1   egg
.....................................................................uaauaAugucagguaaugauguca................   1      1   egg
......................................................................Uauacugucagguaaugauguc.................   1      1   egg
.......................................................................auacugucagguaaugauguc.................   1      0   egg

..........auauaauaaucuuuguagu................................................................................   1      0   T53
...........................Uucaucuuaccaggcagcauu.............................................................   1      1   T53
...........................Uucaucuuaccaggcagcauuaga..........................................................   1      1   T53
............................ucaucuuaccaggcagcau..............................................................   1      0   T53
............................Acaucuuaccaggcagcauu.............................................................   2      1   T53
............................ucaucuuaccaggcagcauu.............................................................   162    0   T53
............................ucaucuuaccaCgcagcauu.............................................................   1      1   T53
............................ucaucuuaccaggcagcauua............................................................   90     0   T53
............................ucaucuuaccaggcagcauuU............................................................   3      1   T53
............................ucUucuuaccaggcagcauuag...........................................................   1      1   T53
............................ucaucuuaccaggcagcauuag...........................................................   129    0   T53
............................ucaucuuaccaggcagcauuaA...........................................................   2      1   T53
............................Acaucuuaccaggcagcauuag...........................................................   1      1   T53
............................ucaucuuaccaggcagcauuagU..........................................................   7      1   T53
............................ucauUuuaccaggcagcauuaga..........................................................   1      1   T53
............................ucaucuuaccaggcagcauuaAa..........................................................   2      1   T53
............................ucaucuuaccaggcagcauuaga..........................................................   15     0   T53
............................ucaucuuaccaggcagcauuagau.........................................................   1      0   T53
............................ucaucuuaccaggcagcauuagUu.........................................................   2      1   T53
............................ucaucuuaccaggcagcauuagaA.........................................................   4      1   T53
............................ucaucuuaccaggcagcauuagaucc.......................................................   1      0   T53
.............................caucuuaccaggcagcau..............................................................   15     0   T53
.............................caucuuaccaggcagcaA..............................................................   1      1   T53
.............................caucuAaccaggcagcauu.............................................................   1      1   T53
.............................Uaucuuaccaggcagcauu.............................................................   1      1   T53
.............................caucuuaccagAcagcauu.............................................................   1      1   T53
.............................caucuuacAaggcagcauu.............................................................   1      1   T53
.............................caucuuaUcaggcagcauu.............................................................   1      1   T53
.............................caucuuaccaggUagcauu.............................................................   2      1   T53
.............................caucuuaccGggcagcauu.............................................................   6      1   T53
.............................caCcuuaccaggcagcauu.............................................................   1      1   T53
.............................caucuuaccaggcagcauA.............................................................   3      1   T53
.............................caucuuaccaggcagcauu.............................................................   1191   0   T53
.............................caucuuaccaggcagcaAu.............................................................   1      1   T53
.............................cauAuuaccaggcagcauu.............................................................   1      1   T53
.............................caucuuaccaggcagcauua............................................................   476    0   T53
.............................Uaucuuaccaggcagcauua............................................................   2      1   T53
.............................cauUuuaccaggcagcauua............................................................   1      1   T53
.............................caucuuaccGggcagcauua............................................................   1      1   T53
.............................caucuuaccagUcagcauua............................................................   1      1   T53
.............................caucuuaccaggcagcauuU............................................................   26     1   T53
.............................caucuuaccaggcaAcauua............................................................   1      1   T53
.............................caucuuaccaggcagcauuaC...........................................................   2      1   T53
.............................caucuuaccaggcagcauuag...........................................................   2600   0   T53
.............................caucuuaccaggcUgcauuag...........................................................   1      1   T53
.............................cCucuuaccaggcagcauuag...........................................................   3      1   T53
.............................caucuuaccaggcagcauuaA...........................................................   21     1   T53
```

```
                               Star                                       Mature
uaacaaaaauuauauaauaaucuuuguagucaucuuaccaggcagcauuagauccgaggauuuaaaacucuaauacugucagguaaugaugucagcagaguuucguacaa
..............................cGucuuaccaggcagcauuag...........................................................   2      1    T53
..............................caucuuaccaggcagcaGuag...........................................................   1      1    T53
..............................caCcuuaccaggcagcauuag...........................................................   1      1    T53
..............................caucuuacAaggcagcauuag...........................................................   1      1    T53
..............................caucuuaccaggcagcauuaU...........................................................   14     1    T53
..............................caucuuaccaggcagcauAag...........................................................   1      1    T53
..............................caucuuaccCggcagcauuag...........................................................   2      1    T53
..............................caucuuaccaggcagUauuag...........................................................   3      1    T53
..............................caucuuaccaggcGgcauuag...........................................................   1      1    T53
..............................caucuuacUaggcagcauuag...........................................................   2      1    T53
..............................caucuuaccaggcaCcauuag...........................................................   1      1    T53
..............................caucuuaccagCcagcauuag...........................................................   1      1    T53
..............................caucuuaccaggcagcaAuag...........................................................   1      1    T53
..............................Uaucuuaccaggcagcauuag...........................................................   3      1    T53
..............................caucuGaccaggcagcauuag...........................................................   1      1    T53
..............................caucuuaccaggcagGauuag...........................................................   1      1    T53
..............................caucuuaccaUgcagcauuag...........................................................   1      1    T53
..............................caucuuaccagAcagcauuag...........................................................   3      1    T53
..............................caucuuaccaggUagcauuag...........................................................   4      1    T53
..............................caucuuaccaCgcagcauuag...........................................................   1      1    T53
..............................caucuuaccaggcaUcauuag...........................................................   2      1    T53
..............................caucuuaUcaggcagcauuag...........................................................   2      1    T53
..............................caucuuaccGggcagcauuag...........................................................   16     1    T53
..............................caucuuUccaggcagcauuaga..........................................................   1      1    T53
..............................caucuuaccUggcagcauuaga..........................................................   1      1    T53
..............................caucuuaccaggcagcauuagG..........................................................   2      1    T53
..............................caucuuaccaggcagcaGuaga..........................................................   1      1    T53
..............................caucuuaccGggcagcauuaga..........................................................   47     1    T53
..............................caucuuaccaggcagcauuaCa..........................................................   1      1    T53
..............................caucuuacAaggcagcauuaga..........................................................   1      1    T53
..............................caucuuaccaggcagcauAaga..........................................................   1      1    T53
..............................caucuuaccaggUagcauuaga..........................................................   1      1    T53
..............................caucuuacUaggcagcauuaga..........................................................   1      1    T53
..............................caucuuaccaggcagcauuaAa..........................................................   3      1    T53
..............................caucuuaccaggcagcauGaga..........................................................   1      1    T53
..............................caucuuaccaggcaCcauuaga..........................................................   1      1    T53
..............................caucuuaccaUgcagcauuaga..........................................................   1      1    T53
..............................caCcuuaccaggcagcauuaga..........................................................   1      1    T53
..............................caucuuaccaggcagcauuagU..........................................................   115    1    T53
..............................caucuuaccaggcagcauuaga..........................................................   2217   0    T53
..............................cUucuuaccaggcagcauuaga..........................................................   2      1    T53
..............................caucuuaccagCcagcauuaga..........................................................   2      1    T53
..............................cCucuuaccaggcagcauuaga..........................................................   1      1    T53
..............................caucuuaccaggcagUauuaga..........................................................   6      1    T53
..............................caucuGaccaggcagcauuaga..........................................................   1      1    T53
..............................caucuuaccaggcaUcauuaga..........................................................   3      1    T53
..............................caucuuaccCggcagcauuaga..........................................................   1      1    T53
..............................caucuuaUcaggcagcauuaga..........................................................   1      1    T53
..............................caucuuaccaggcagcauuagaG.........................................................   1      1    T53
..............................caucuuaccaggcagcauuagau.........................................................   8      0    T53
..............................caucuuaccaggcagcauuagaA.........................................................   49     1    T53
..............................caucuuaccaggcagcauuagUu.........................................................   10     1    T53
..............................caucuuaccaggcagcauuagauc........................................................   2      0    T53
..............................caucuuaccaggcagcauuagaAc........................................................   2      1    T53
..............................caucuuaccaggcagcauuagauA........................................................   6      1    T53
..............................caucuuaccaggcagcauuagauU........................................................   1      1    T53
..............................caucuuaccaggcagcauuagaucc.......................................................   1      0    T53
..............................caucuuaccaggcagcauuagauccga.....................................................   3      0    T53
...............................aucuuaccaggcagcauu.............................................................   3      0    T53
...............................aucuuaccaggcagcauuag...........................................................   6      0    T53
...............................aucuuaccaggcagcauuaga..........................................................   7      0    T53
...............................aucuuaccaggcagcauuagauc........................................................   1      0    T53
...............................aucuuaccaggcagcauuagauU........................................................   1      1    T53
...............................aucuuaccaggcagcauuagauccgag....................................................   1      0    T53
................................ucuuaccaggcagcauuagau.........................................................   2      0    T53
................................ucuuaccaggcagcauuagauc........................................................   65     0    T53
................................ucuuaccaggcagcauuagauA........................................................   2      1    T53
.................................cuuaccaggcagcauuag...........................................................   1      0    T53
.................................cuuaccaggcagcauuagau.........................................................   1      0    T53
..................................uuaccaggcagcauuaga..........................................................   1      0    T53
```

Star **Mature**

```
uaacaaaaauuauauaauaaucuuuguagucaucuuaccaggcagcauuagauccgaggauuuaaaacucuaauacugucagguaaugaugucagcagaguuucguacaa
....................................................uccgaggauuuaaaacuc........................................   3     0   T53
....................................................uccgaggauuuaaaacuA........................................   2     1   T53
..................................................................acucuaauacugucagguaau.......................   2     0   T53
..................................................................acuUuaauacugucagguaaugauguc.................   1     1   T53
...................................................................cuUuaauacugucagguaaugau....................   3     1   T53
...................................................................cuUuaauacugucagguaaugaug...................   7     1   T53
...................................................................cuUuaauacugucagguaaugaugu..................   16    1   T53
...................................................................cuUuaauacugucagguaaugauguc.................   9     1   T53
....................................................................uUuaauacugucagguaaugau....................   7     1   T53
....................................................................ucuaauacugucagguaaugau....................   3     0   T53
....................................................................Gcuaauacugucagguaaugaug...................   2     1   T53
....................................................................uUuaauacugucagguaaugaug...................   10    1   T53
....................................................................uAuaauacugucagguaaugaugu..................   1     1   T53
....................................................................uUuaauacugucagguaaugaugu..................   49    1   T53
....................................................................Gcuaauacugucagguaaugaugu..................   1     1   T53
....................................................................uUuaauacugucagguaaugauguc.................   19    1   T53
.....................................................................cuaauacugucagguaaugau....................   13    0   T53
.....................................................................Uuaauacugucagguaaugau....................   5     1   T53
.....................................................................Auaauacugucagguaaugau....................   1     1   T53
.....................................................................Guaauacugucagguaaugaug...................   1     1   T53
.....................................................................Auaauacugucagguaaugaug...................   2     1   T53
.....................................................................cuaauacugucagguaaugaug...................   3     0   T53
.....................................................................cuaauacugucagguaaugauA..................   1     1   T53
.....................................................................Uuaauacugucagguaaugaug...................   28    1   T53
.....................................................................Uuaauacugucagguaaugaugu..................   78    1   T53
.....................................................................Auaauacugucagguaaugaugu..................   9     1   T53
.....................................................................Guaauacugucagguaaugaugu..................   4     1   T53
.....................................................................cuaauacugucagguaaugaugu..................   20    0   T53
.....................................................................Uuaauacugucagguaaugauguc.................   210   1   T53
.....................................................................cuaauacugucagguaaugauguU.................   5     1   T53
.....................................................................cuaauacugucagguaaugauguc.................   8     0   T53
.....................................................................Guaauacugucagguaaugauguc.................   4     1   T53
.....................................................................Auaauacugucagguaaugauguc.................   18    1   T53
.....................................................................cuaauacugucagguaaugaugucU................   4     1   T53
......................................................................uaauacugucagguaaAg......................   1     1   T53
......................................................................uaauacugucagguaauC......................   2     1   T53
......................................................................uaauacugucagguaaug......................   86    0   T53
......................................................................uaauacugucagguaauA......................   3     1   T53
......................................................................uaaGacugucagguaaug......................   1     1   T53
......................................................................uaauaUugucagguaaug......................   2     1   T53
......................................................................uaauacugucagguaauU......................   1     1   T53
......................................................................uaauacugucagguaauga.....................   204   0   T53
......................................................................uaauacugucUgguaauga.....................   1     1   T53
......................................................................uaauacugucagguaaAga.....................   2     1   T53
......................................................................uaauacugucagguaaugU.....................   17    1   T53
......................................................................uaauacugAcagguaauga.....................   1     1   T53
......................................................................uaauacugucagguaauAa.....................   2     1   T53
......................................................................Aaauacugucagguaauga.....................   1     1   T53
......................................................................uaauaUugucagguaauga.....................   1     1   T53
......................................................................uaauacugucagguaauUau....................   3     1   T53
......................................................................uaauacugucagguaaugUu....................   15    1   T53
......................................................................uaauacugucGgguaaugau....................   2     1   T53
......................................................................uaauacugucagguaUugau....................   3     1   T53
......................................................................uaauacuguUagguaaugau....................   2     1   T53
......................................................................uaauacugucagguaaugau....................   9862  0   T53
......................................................................uaauacugucagUuaaugau....................   1     1   T53
......................................................................uaauacugucagguaauAau....................   2     1   T53
......................................................................uaauacugucagguaCugau....................   2     1   T53
......................................................................Gaauacugucagguaaugau....................   4     1   T53
......................................................................uaauacAgucagguaaugau....................   4     1   T53
......................................................................uaauacugucagguaauCau....................   2     1   T53
......................................................................uUauacugucagguaaugau....................   2     1   T53
......................................................................uaauacugucaCguaaugau....................   1     1   T53
......................................................................uaauaGugucagguaaugau....................   5     1   T53
......................................................................uaauacugucaggCaaugau....................   1     1   T53
......................................................................uaauacugucagCuaaugau....................   1     1   T53
......................................................................uaauacugucagguaaugGu....................   2     1   T53
......................................................................Aaauacugucagguaaugau....................   19    1   T53
......................................................................uaauacuAucagguaaugau....................   1     1   T53
......................................................................uaauacugucagguaaCgau....................   1     1   T53
```

```
                              Star                          Mature
uaacaaaaauuauauaauaaucuuuguagucaucuuaccaggcagcauuagauccgaggauuuaaaacucuaauacugucagguaaugaugucagcagaguuucguacaa
......................................................................uaauUcugucagguaaugau....................  2    1  T53
......................................................................uaauaUugucagguaaugau....................  2    1  T53
......................................................................uaauacugucaggAaaugau....................  17   1  T53
......................................................................uaauGcugucagguaaugau....................  1    1  T53
......................................................................uCauacugucagguaaugau....................  1    1  T53
......................................................................uaauacugucagAuaaugau....................  2    1  T53
......................................................................uaaCacugucagguaaugau....................  3    1  T53
......................................................................uaaGacugucagguaaugau....................  1    1  T53
......................................................................uaauacugucagguGaugau....................  1    1  T53
......................................................................uaaAacugucagguaaugau....................  2    1  T53
......................................................................uaUuacugucagguaaugau....................  6    1  T53
......................................................................uaauacugucaggGaaugau....................  2    1  T53
......................................................................uaauacugucagguUaugau....................  7    1  T53
......................................................................uaauacugucagguaaugaG....................  10   1  T53
......................................................................Caauacugucagguaaugau....................  3    1  T53
......................................................................uaauacugucagguaaugaC....................  3    1  T53
......................................................................uaauacugucagguaaugaA....................  38   1  T53
......................................................................uaauacugucagguaaAgau....................  5    1  T53
......................................................................uaauacugAcagguaaugau....................  11   1  T53
......................................................................Caauacugucagguaaugaug...................  15   1  T53
......................................................................uaauacugucCgguaaugaug...................  1    1  T53
......................................................................uaauacugCcagguaaugaug...................  3    1  T53
......................................................................uaauacugucagguaaGgaug...................  7    1  T53
......................................................................uaauaAugucagguaaugaug...................  3    1  T53
......................................................................uaauacugucagguaaCgaug...................  4    1  T53
......................................................................uaauacugucagCuaaugaug...................  2    1  T53
......................................................................uaauacugucagguGaugaug...................  1    1  T53
......................................................................uaauacugucaAguaaugaug...................  13   1  T53
......................................................................uaauacugucagguaaugaCg...................  23   1  T53
......................................................................Aaauacugucagguaaugaug...................  57   1  T53
......................................................................uaauacugucGgguaaugaug...................  18   1  T53
......................................................................uaauacugucagguaaugauC...................  105  1  T53
......................................................................uaauacugucagguaCugaug...................  1    1  T53
......................................................................uaauGcugucagguaaugaug...................  2    1  T53
......................................................................uaaGacugucagguaaugaug...................  4    1  T53
......................................................................uaauacugucUgguaaugaug...................  3    1  T53
......................................................................uaauaGugucagguaaugaug...................  9    1  T53
......................................................................uCauacugucagguaaugaug...................  3    1  T53
......................................................................uaauacugucagguaaugaAg...................  19   1  T53
......................................................................uaauacuAucagguaaugaug...................  5    1  T53
......................................................................uaaCacugucagguaaugaug...................  10   1  T53
......................................................................uaauacugucagguaaugUug...................  63   1  T53
......................................................................uaauacugAcagguaaugaug...................  75   1  T53
......................................................................uaauacugucagguaUugaug...................  9    1  T53
......................................................................uaauacugucagguaauUaug...................  9    1  T53
......................................................................uaauaUugucagguaaugaug...................  15   1  T53
......................................................................uaauacAgucagguaaugaug...................  23   1  T53
......................................................................uaauacugucagUuaaugaug...................  12   1  T53
......................................................................uaauacugGcagguaaugaug...................  7    1  T53
......................................................................uaauacugucagguaauCaug...................  8    1  T53
......................................................................uaauacugucaggCaaugaug...................  6    1  T53
......................................................................uaauacugucaCguaaugaug...................  4    1  T53
......................................................................uaauacugucagguaaugauU...................  545  1  T53
......................................................................uaauacugucagAuaaugaug...................  18   1  T53
......................................................................uaauUcugucagguaaugaug...................  3    1  T53
......................................................................uaauacGgucagguaaugaug...................  3    1  T53
......................................................................uaauacugucagguaaugCug...................  4    1  T53
......................................................................uaauacugucaUguaaugaug...................  6    1  T53
......................................................................uUauacugucagguaaugaug...................  8    1  T53
......................................................................uaauacugucagguaauAaug...................  28   1  T53
......................................................................uaauacugucagguaaugaGg...................  14   1  T53
......................................................................uaauacuguGagguaaugaug...................  10   1  T53
......................................................................uaaAacugucagguaaugaug...................  29   1  T53
......................................................................uaauacuCucagguaaugaug...................  5    1  T53
......................................................................uaauacugucaggAaaugaug...................  96   1  T53
......................................................................uaauacugucagguaaugGug...................  5    1  T53
......................................................................uaauacuguAagguaaugaug...................  2    1  T53
......................................................................uGauacugucagguaaugaug...................  8    1  T53
......................................................................uaauacuguUagguaaugaug...................  14   1  T53
......................................................................uaauacugucagguUaugaug...................  28   1  T53
```

Star Mature

```
uaacaaaaauuauauaauaaucuuuguagucaucuuaccaggcagcauuagauccgaggauuuaaaacucuaauacugucagguaaugaugucagcagaguuucguacaa
......................................................................uaUuacugucagguaaugaug...................  30      1  T53
......................................................................Gaauacugucagguaaugaug...................  11      1  T53
......................................................................uaauacugucagguaaAgaug...................  81      1  T53
......................................................................uaauacugucaggGaaugaug...................  7       1  T53
......................................................................uaauacugucagguaaugaug...................  59992   0  T53
......................................................................uaauacugucagguaaugauA...................  3938    1  T53
......................................................................uaauacugCcagguaaugaugu..................  15      1  T53
......................................................................uaauacugucUgguaaugaugu..................  21      1  T53
......................................................................uaauacugucagguaaAgaugu..................  421     1  T53
......................................................................uaauacugucaCguaaugaugu..................  17      1  T53
......................................................................uaauacugucagguaaugUugu..................  205     1  T53
......................................................................uaauacugucagguaauCaugu..................  64      1  T53
......................................................................uaauacugucagguaaugauUu..................  78      1  T53
......................................................................Aaauacugucagguaaugaugu..................  277     1  T53
......................................................................uaauacugucagguaaugaugA..................  849     1  T53
......................................................................uaauacugucagguaaugGugu..................  36      1  T53
......................................................................uaauacugucaggAaaugaugu..................  321     1  T53
......................................................................uaauacugucagguGaugaugu..................  18      1  T53
......................................................................uaauacugucagguaauAaugu..................  62      1  T53
......................................................................uUauacugucagguaaugaugu..................  33      1  T53
......................................................................uaauacugucagguaaugaugu..................  249972  0  T53
......................................................................uaauUcugucagguaaugaugu..................  25      1  T53
......................................................................uaauacugucCgguaaugaugu..................  1       1  T53
......................................................................uaaGacugucagguaaugaugu..................  19      1  T53
......................................................................uaauacuguUagguaaugaugu..................  79      1  T53
......................................................................uaauCcugucagguaaugaugu..................  1       1  T53
......................................................................uaauacuUucagguaaugaugu..................  7       1  T53
......................................................................uaUuacugucagguaaugaugu..................  120     1  T53
......................................................................uaGuacugucagguaaugaugu..................  12      1  T53
......................................................................uaauacuAucagguaaugaugu..................  13      1  T53
......................................................................uaauacugucaUguaaugaugu..................  34      1  T53
......................................................................uaaAacugucagguaaugaugu..................  92      1  T53
......................................................................uaauacugucagguUaugaugu..................  143     1  T53
......................................................................uaauacugucagguaGugaugu..................  11      1  T53
......................................................................uaaCacugucagguaaugaugu..................  19      1  T53
......................................................................uaauaAugucagguaaugaugu..................  26      1  T53
......................................................................uaauacugucagguCaugaugu..................  8       1  T53
......................................................................uaauacugucaggCaaugaugu..................  25      1  T53
......................................................................uaauacugucagguaCugaugu..................  6       1  T53
......................................................................uaauacugucagCuaaugaugu..................  27      1  T53
......................................................................uaauacugucagguaaGgaugu..................  40      1  T53
......................................................................uaauacugucaAguaaugaugu..................  39      1  T53
......................................................................uaauacugucagguaaugaugC..................  200     1  T53
......................................................................uaauacugucagguaaugaAgu..................  42      1  T53
......................................................................uaauacugucagguaaCgaugu..................  23      1  T53
......................................................................uaauacugucagAuaaugaugu..................  67      1  T53
......................................................................uaauacugucagguaaugCugu..................  15      1  T53
......................................................................Caauacugucagguaaugaugu..................  52      1  T53
......................................................................uaauacuCucagguaaugaugu..................  18      1  T53
......................................................................uCauacugucagguaaugaugu..................  21      1  T53
......................................................................uaauacugucagguaaugauAu..................  31      1  T53
......................................................................uaauacugucagguaaugaCgu..................  20      1  T53
......................................................................uaauacuguAagguaaugaugu..................  5       1  T53
......................................................................uaauacugucGgguaaugaugu..................  106     1  T53
......................................................................uaauacugGcagguaaugaugu..................  12      1  T53
......................................................................uaauacugucagguaaugaGgu..................  19      1  T53
......................................................................uaauacugAcagguaaugaugu..................  227     1  T53
......................................................................uaauacugucagguaauUaugu..................  63      1  T53
......................................................................uaauacCgucagguaaugaugu..................  8       1  T53
......................................................................uaauGcugucagguaaugaugu..................  19      1  T53
......................................................................uaauacugucagUuaaugaugu..................  37      1  T53
......................................................................uaauaGugucagguaaugaugu..................  62      1  T53
......................................................................uaauacAgucagguaaugaugu..................  71      1  T53
......................................................................uaauacugucagguaaugauCu..................  22      1  T53
......................................................................uaauaUugucagguaaugaugu..................  63      1  T53
......................................................................uaauacugucagguaaugaugG..................  48      1  T53
......................................................................Gaauacugucagguaaugaugu..................  43      1  T53
......................................................................uGauacugucagguaaugaugu..................  36      1  T53
......................................................................uaauacuguGagguaaugaugu..................  26      1  T53
......................................................................uaCuacugucagguaaugaugu..................  2       1  T53
```

Star **Mature**

uaacaaaaauuauauaauaaucuuuguagucaucuuaccaggcagcauuagauccgaggauuuaaaacucuaauacugucagguaaugaugucagcagaguuucguacaa			
....uaauacGgucagguaaugaugu..................	10	1	T53
....uaauacugucagguaUugaugu..................	49	1	T53
....uaauacugucaggGaaugaugu..................	21	1	T53
....uaauacugucagguaaugaCguc.................	68	1	T53
....uaauacugucagguaaugauUuc.................	244	1	T53
....Gaauacugucagguaaugauguc.................	143	1	T53
....uaauacugucagguaaugCuguc.................	44	1	T53
....uaauacugucagguaaugaugGc.................	152	1	T53
....uaauacugucagguaCugauguc.................	38	1	T53
....uaauacuUucagguaaugauguc.................	51	1	T53
....uaauacugucagguaUugauguc.................	228	1	T53
....uaauacugucUgguaaugauguc.................	82	1	T53
....uaauacugucagguaaCgauguc.................	113	1	T53
....uaauacCgucagguaaugauguc.................	63	1	T53
....uaauGcugucagguaaugauguc.................	85	1	T53
....uaauacugucagguaaugaugCc.................	588	1	T53
....uaauacugucagguaaugaGguc.................	135	1	T53
....uaauacugucagguaaugaugAc.................	1689	1	T53
....uGauacugucagguaaugauguc.................	176	1	T53
....uaauUcugucagguaaugauguc.................	109	1	T53
....uaauacugucagguaaugauguU.................	6658	1	T53
....uaauacugucaggAaaugauguc.................	1325	1	T53
....uaauacugucagguCaugauguc.................	24	1	T53
....uaauacAgucagguaaugauguc.................	297	1	T53
....uaGuacugucagguaaugauguc.................	329	1	T53
....uCauacugucagguaaugauguc.................	136	1	T53
....uaauacugucagAuaaugauguc.................	222	1	T53
....Aaauacugucagguaaugauguc.................	1036	1	T53
....uaauacugucagguaGugauguc.................	53	1	T53
....uaauacugucagguaaGgauguc.................	157	1	T53
....uaauacGgucagguaaugauguc.................	17	1	T53
....uaaAacugucagguaaugauguc.................	372	1	T53
....uaauaAugucagguaaugauguc.................	86	1	T53
....uaauacugucagguGaugauguc.................	78	1	T53
....uaCuacugucagguaaugauguc.................	160	1	T53
....uaauCcugucagguaaugauguc.................	8	1	T53
....uaauacugucagguaaugauCuc.................	133	1	T53
....uaauacugucaggCaaugauguc.................	116	1	T53
....uaauacuAucagguaaugauguc.................	98	1	T53
....uaauacuCucagguaaugauguc.................	58	1	T53
....uaauacuguAagguaaugauguc.................	18	1	T53
....uaauacugucagguaauCauguc.................	185	1	T53
....Caauacugucagguaaugauguc.................	194	1	T53
....uaaGacugucagguaaugauguc.................	60	1	T53
....uaauacugucCgguaaugauguc.................	6	1	T53
....uaauacugucagguaaugUuguc.................	1162	1	T53
....uaauacugucagguaaugauAuc.................	77	1	T53
....uaauacugCcagguaaugauguc.................	77	1	T53
....uaauacugucagguaauAauguc.................	223	1	T53
....uaauacugAcagguaaugauguc.................	1109	1	T53
....uaauacugucagguaaAgauguc.................	3330	1	T53
....uaauacugucGgguaaugauguc.................	173	1	T53
....uaUuacugucagguaaugauguc.................	1162	1	T53
....uaauacugucaggGaaugauguc.................	55	1	T53
....uaauacugGcagguaaugauguc.................	45	1	T53
....uaauacugucagguaaugGuguc.................	169	1	T53
....uaaCacugucagguaaugauguc.................	101	1	T53
....uaauacugucagCuaaugauguc.................	103	1	T53
....uaauacugucaCguaaugauguc.................	89	1	T53
....uaauaUugucagguaaugauguc.................	240	1	T53
....uUauacugucagguaaugauguc.................	175	1	T53
....uaauacugucagguaauUauguc.................	263	1	T53
....uaauacuguUagguaaugauguc.................	260	1	T53
....uaauacugucagguUaugauguc.................	564	1	T53
....uaauacuguGagguaaugauguc.................	88	1	T53
....uaauacugucagUuaaugauguc.................	193	1	T53
....uaauaGugucagguaaugauguc.................	206	1	T53
....uaauacugucagguaaugauguA.................	623	1	T53
....uaauacugucagguaaugauguc.................	1023937	0	T53
....uaauacugucaUguaaugauguc.................	110	1	T53

```
                             Star                                        Mature
uaacaaaaauuauauaauaaucuuuguagucaucuuaccaggcagcauuagauccgaggauuuaaaacucuaauacugucagguaaugaugucagcagaguuucguacaa
......................................................................uaauacugucaAguaaugauguc.................    153     1    T53
......................................................................uaauacugucagguaaugauguG.................    116     1    T53
......................................................................uaauacugucagguaaugaAguc.................    189     1    T53
......................................................................uaauacugucagguaaugaugGca................    5       1    T53
......................................................................uaauGcugucagguaaugauguca................    1       1    T53
......................................................................uCauacugucagguaaugauguca................    1       1    T53
......................................................................Gaauacugucagguaaugauguca................    1       1    T53
......................................................................uaauacugucagguaaugaugCca................    2       1    T53
......................................................................uaauaGugucagguaaugauguca................    6       1    T53
......................................................................uaauacugucaCguaaugauguca................    3       1    T53
......................................................................uaauacugucagguaauCauguca................    3       1    T53
......................................................................uaauacugucagguaauAauguca................    4       1    T53
......................................................................uaUuacugucagguaaugauguca................    6       1    T53
......................................................................uaauacuguAagguaaugauguca................    1       1    T53
......................................................................uaauacugucagguaaugauguca................    12040   0    T53
......................................................................uaauacugucagguaaugaGguca................    2       1    T53
......................................................................uaauacugucagAuaaugauguca................    1       1    T53
......................................................................uaauacugucagguaaugauguGa................    2       1    T53
......................................................................uaauacuguUagguaaugauguca................    2       1    T53
......................................................................uaauacGgucagguaaugauguca................    1       1    T53
......................................................................uaauacugucaggAaaugauguca................    14      1    T53
......................................................................uaauacugucGgguaaugauguca................    3       1    T53
......................................................................uUauacugucagguaaugauguca................    3       1    T53
......................................................................uaauacugucagguGaugauguca................    1       1    T53
......................................................................uaauacugucagguaaugauCuca................    2       1    T53
......................................................................uaauacugucagguaaCgauguca................    2       1    T53
......................................................................uaauacugucagguaaugaugucU................    57352   1    T53
......................................................................uaauacugucagguaaugauguUa................    33      1    T53
......................................................................uaauacuAucagguaaugauguca................    1       1    T53
......................................................................uaauacugucagUuaaugauguca................    1       1    T53
......................................................................uaauUcugucagguaaugauguca................    4       1    T53
......................................................................Caauacugucagguaaugauguca................    3       1    T53
......................................................................uaauacAgucagguaaugauguca................    3       1    T53
......................................................................uaauacugucagguaaugaugucC................    1354    1    T53
......................................................................uaauacugAcagguaaugauguca................    13      1    T53
......................................................................uaauacugucagguUaugauguca................    4       1    T53
......................................................................uaauacugucagguaaugauUuca................    2       1    T53
......................................................................Aaauacugucagguaaugauguca................    6       1    T53
......................................................................uaauacugucagguaaugaAguca................    1       1    T53
......................................................................uaauaAugucagguaaugauguca................    1       1    T53
......................................................................uaauacugucaggCaaugauguca................    3       1    T53
......................................................................uaauacugucaUguaaugauguca................    1       1    T53
......................................................................uaauacugucaAguaaugauguca................    1       1    T53
......................................................................uaaGacugucagguaaugauguca................    2       1    T53
......................................................................uaauacuCucagguaaugauguca................    3       1    T53
......................................................................uaaCacugucagguaaugauguca................    3       1    T53
......................................................................uaauacugCcagguaaugauguca................    3       1    T53
......................................................................uaauacugucagguaaugauguAa................    15      1    T53
......................................................................uaaAacugucagguaaugauguca................    3       1    T53
......................................................................uaauacugucagguaaugaugucG................    1594    1    T53
......................................................................uaauacugucagguaaugUuguca................    13      1    T53
......................................................................uaauacugucagguaUugauguca................    2       1    T53
......................................................................uaauaUugucagguaaugauguca................    5       1    T53
......................................................................uaGuacugucagguaaugauguca................    2       1    T53
......................................................................uaauacugucagguaaugaugAca................    26      1    T53
......................................................................uaauacugucagguaauUauguca................    2       1    T53
......................................................................uaauacugucagguaaAgauguca................    28      1    T53
......................................................................uaauacugucagguaaugaugucCg...............    10      1    T53
......................................................................uaauacugucagguaaugaugucUg...............    6       1    T53
......................................................................uaauacugucagguaaugaugucaC...............    16      1    T53
......................................................................uaauacugucagguaaugaugucaA...............    72      1    T53
......................................................................uaauacugucagguaaugaugucGg...............    2       1    T53
......................................................................uaauacugucagguaaugaugucaU...............    193     1    T53
......................................................................uaauacugucagguaaugaugucaUc..............    1       1    T53
......................................................................uaauacugucagguaaugaugucagc..............    1       0    T53
......................................................................uaauacugucagguaaugaugucUgc..............    3       1    T53
......................................................................uaauacugucagguaaugaugucGgc..............    2       1    T53
......................................................................uaauacugucagguaaugaugucaAc..............    4       1    T53
......................................................................uaauacugucagguaaugaugucaAca.............    1       1    T53
.......................................................................aauacugucagguaaugau....................    27      0    T53
```

```
                              Star                                   Mature
uaacaaaaauuauauaauaaucuuuguagucaucuuaccaggcagcauuagauccgaggauuuaaaacucuaauacugucagguaaugaugucagcagaguuucguacaa
.......................................................................aauacugucagguaaugUu....................    1      1    T53
.......................................................................aauacugucagguaaugGu....................    1      1    T53
.......................................................................aauacugucagguaaugauA...................    4      1    T53
.......................................................................Uauacugucagguaaugaug...................    3      1    T53
.......................................................................aauacugucagguaaugaug...................    118    0    T53
.......................................................................Uauacugucagguaaugaugu..................    4      1    T53
.......................................................................aauacugucagCuaaugaugu..................    1      1    T53
.......................................................................aauacuguGagguaaugaugu..................    1      1    T53
.......................................................................aauacugucCgguaaugaugu..................    1      1    T53
.......................................................................aauacugucagguaaugaugu..................    552    0    T53
.......................................................................aUuacugucagguaaugauguc.................    12     1    T53
.......................................................................aauacugucagguaauUauguc.................    2      1    T53
.......................................................................aCuacugucagguaaugauguc.................    6      1    T53
.......................................................................aauacugucagguaaugauguG.................    1      1    T53
.......................................................................aauacugucagguaaugaugGc.................    1      1    T53
.......................................................................aauacugucagguaaugaAguc.................    1      1    T53
.......................................................................aaAacugucagguaaugauguc.................    3      1    T53
.......................................................................aauacugucagguaaugaugCc.................    2      1    T53
.......................................................................aauacugucagguUaugauguc.................    1      1    T53
.......................................................................aauacugucagguaauCauguc.................    1      1    T53
.......................................................................aauacugucagguaaAgauguc.................    3      1    T53
.......................................................................aauacugucagguaaugauguc.................    4678   0    T53
.......................................................................aauacuguAagguaaugauguc.................    1      1    T53
.......................................................................aauacugucagguaaugaugAc.................    3      1    T53
.......................................................................aauacugucagUuaaugauguc.................    1      1    T53
.......................................................................aauacugucagguaaugGuguc.................    5      1    T53
.......................................................................aauacugucaggAaaugauguc.................    2      1    T53
.......................................................................aauacuguUagguaaugauguc.................    2      1    T53
.......................................................................aauacugucaggCaaugauguc.................    1      1    T53
.......................................................................aauacugucagguaaugauguA.................    1      1    T53
.......................................................................Uauacugucagguaaugauguc.................    19     1    T53
.......................................................................aGuacugucagguaaugauguc.................    5      1    T53
.......................................................................aauacugucaAguaaugauguc.................    1      1    T53
.......................................................................aauacugucagguaaugauguU.................    19     1    T53
.......................................................................aauacugucagguaaugauAuc.................    1      1    T53
.......................................................................aauacugucagguaUugauguc.................    3      1    T53
.......................................................................aauacugucagguaaugUuguc.................    2      1    T53
.......................................................................aauacugucGgguaaugauguc.................    2      1    T53
.......................................................................aauacugucagAuaaugauguc.................    2      1    T53
.......................................................................aauacugucagguaaugauCuc.................    1      1    T53
.......................................................................Gauacugucagguaaugauguc.................    1      1    T53
.......................................................................aauacugucaCguaaugauguc.................    1      1    T53
.......................................................................aauacuAucagguaaugauguc.................    1      1    T53
.......................................................................aauaUugucagguaaugauguc.................    1      1    T53
.......................................................................aauacCgucagguaaugauguc.................    2      1    T53
.......................................................................aauacugAcagguaaugauguc.................    7      1    T53
.......................................................................aauacAgucagguaaugauguc.................    2      1    T53
.......................................................................aauacugucagguaaugaugucG................    14     1    T53
.......................................................................aauacugucagguaaugauguca................    115    0    T53
.......................................................................aauacugucagguaaugauguAa................    1      1    T53
.......................................................................aauacugucagguaaugaugucC................    27     1    T53
.......................................................................aauacugucagguaaugaugucU................    1021   1    T53
.......................................................................aauacugucagguaaugaugucUg...............    1      1    T53
.......................................................................aauacugucagguaaugaugucaU...............    3      1    T53
........................................................................auacugucagguaaugauA...................    1      1    T53
........................................................................auacugucagguaaugaug...................    3      0    T53
........................................................................auacugucagguaaugaugu..................    7      0    T53
........................................................................Uuacugucagguaaugaugu..................    1      1    T53
........................................................................aAacugucagguaaugauguc.................    1      1    T53
........................................................................aGacugucagguaaugauguc.................    1      1    T53
........................................................................Uuacugucagguaaugauguc.................    10     1    T53
........................................................................auacugucagguaaugauguc.................    73     0    T53
........................................................................auacugucaUguaaugauguc.................    1      1    T53
........................................................................auacugucagguaaugaugucU................    9      1    T53
........................................................................auacugucagguaaugauguca................    5      0    T53
........................................................................auacugucagguaaugaugucC................    1      1    T53
........................................................................auacugucagguaaugaugucaU...............    1      1    T53
.........................................................................uacugucagguaaugaug...................    3      0    T53
.........................................................................uacugucagguaaugaugu..................    7      0    T53
.........................................................................Aacugucagguaaugauguc.................    1      1    T53
```

```
                              Star                                    Mature
uaacaaaaauuauauaauaaucuuuguagucaucuuaccaggcagcauuagauccgaggauuuaaaacucuaauacugucagguaaugaugucagcagaguuucguacaa
.........................................................................uacugucagguaaugauguc.................   26   0   T53
.........................................................................uacugucagguaaugaugucU................   3    1   T53
..........................................................................acugucagguaaugaugu..................   16   0   T53
..........................................................................acugucagguaaugaugA..................   1    1   T53
..........................................................................acugucagguaaugauguc.................   22   0   T53
...........................................................................cugucagguaaugauguc.................   69   0   T53
...........................................................................cuguUagguaaugauguc.................   1    1   T53
...........................................................................cugucagguaaugauguU.................   1    1   T53
...........................................................................cugucaggAaaugauguc.................   1    1   T53
...........................................................................cugucagguaaugauguA.................   2    1   T53
...........................................................................cugucagguaaugaugucU................   5    1   T53
...........................................................................cugucagguaaugauguca................   1    0   T53
...........................................................................cugucagguaaugaugucaA...............   1    1   T53
............................................................................ugucagguaaugaugucU................   2    1   T53
```

Provisional ID	:	Scaffold_1043_1596
Score total	:	31916.1
Score for star read(s)	:	3.9
Score for read counts	:	31911.6
Score for mfe	:	-0.3
Score for randfold	:	1.6
Score for cons. seed	:	-0.6
Total read count	:	62605
Mature read count	:	50950
Loop read count	:	0
Star read count	:	11655

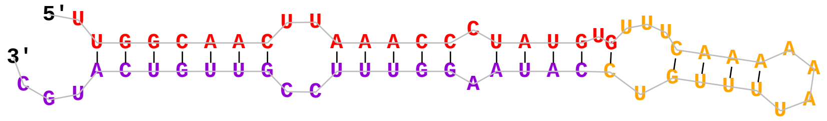

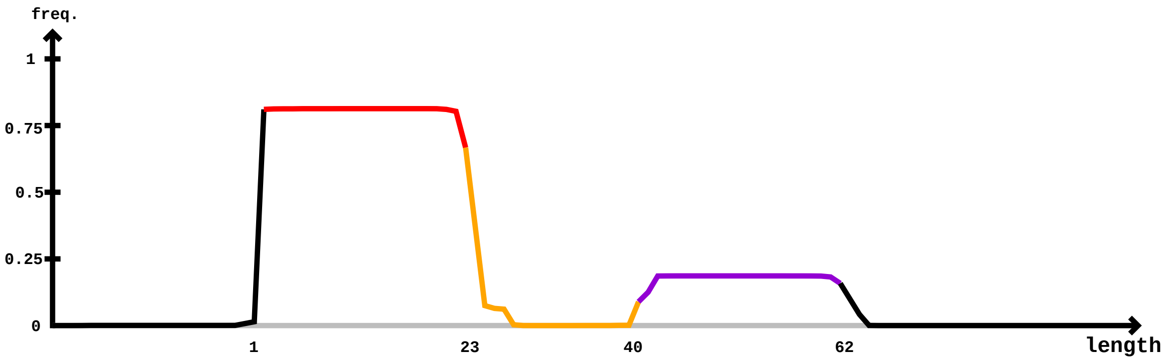


```
                    Mature                                 Star
5'- uucaccuacugacuguuguuuuggcaacuuaaacccuauguguuucaaaaaaauuuugucacauaagguuuccguugucaugccaacaauuagucacaacaaccacaacaaac  -3'   obs
    uucaccuacugacuguuguuuuggcaacuuaaacccuauguguuucaaaaaaauuuugucacauaagguuuccguugucaugccaacaauuagucacaacaaccacaacaaac         exp
    .......(((((.(((((...(((((((..(((((.((((.(...((((....)))).))))).))))).)))))))...))))).)))))....................    reads    mm    sample
    uucaccuacugacuguuguu............................................................................................    1        0     te1
    ..caccuacugacuguuguu............................................................................................    1        0     te1
    ................Uuuuuggcaacuuaaacccuau..........................................................................    1        1     te1
    ................Uuuuuggcaacuuaaacccuaug.........................................................................    2        1     te1
    .................uuuuggcaacuuaaacccua...........................................................................    2        0     te1
    .................uuuuggcaacuuaaaccUuau..........................................................................    1        1     te1
    .................uuuuggcaacuuaaacccuau..........................................................................    43       0     te1
    .................Auuuggcaacuuaaacccuaug.........................................................................    1        1     te1
    .................uuuuggcaacuuaaacccuauU.........................................................................    1        1     te1
    .................uuuuggcaacuuaaacccuaug.........................................................................    66       0     te1
    .................uuuuggcaacuuaaacccuaugA........................................................................    27       1     te1
    .................uuuuggcaacuuaaacccuaugu........................................................................    4        0     te1
    .................uuuuggcaacuuaaaUccuaugu........................................................................    1        1     te1
    .................uuuuggcaacuuaaacccuaugugA......................................................................    1        1     te1
    .................uuuuggcaacuuaaacccuauguAu......................................................................    1        1     te1
    ..................uuuggcaacuuaaacccuau..........................................................................    1        0     te1
    ..................uuuggcaacuuaaacccuaug.........................................................................    3        0     te1
    ..................uuuggcaacuuaaaccUuaugu........................................................................    1        1     te1
    ..................uuuggcaacuuaaacccuaugA........................................................................    3        1     te1
    ..................uuCggcaacuuaaacccuaugu........................................................................    1        1     te1
    ..................uuuggcaacuuaaacccuaugu........................................................................    51       0     te1
    ..................uuuggUaacuuaaacccuaugu........................................................................    1        1     te1
    ..................uuuggcaacuuaaacccuaugug.......................................................................    4        0     te1
    ..................uuuggcaacuuaaacccuauguA.......................................................................    2        1     te1
    ..................uuuggcaacuuaaacccuaugugA......................................................................    2        1     te1
    ..................uuuggcaacuuaaacccuauguUu......................................................................    1        1     te1
    ..................uuuggcaacuuaaacccuauguguuuc...................................................................    1        0     te1
    ....................uuggcaacuuaaacccua..........................................................................    3        0     te1
    ....................uuggcaacuuaaacccuau.........................................................................    8        0     te1
    ....................uuggcaacuuaaacccuaug........................................................................    34       0     te1
    ....................uuggcaacuuaaacccuauA........................................................................    2        1     te1
    ....................uuggcaacuuaaacccuGug........................................................................    1        1     te1
    ....................uuggcaacuuaaacccuauU........................................................................    1        1     te1
```

Mature Star

```
uucaccuacugacuguuguuuuggcaacuuaaacccuauguguuucaaaaaauuuuguccauaagguuuccguugucaugccaacaauuagucacaacaaccacaacaaac
....................uugAcaacuuaaacccuaugu......................................................................  1     1  te1
....................uuggcaacuuaaacccuaugA......................................................................  22    1  te1
....................uuggcaacuuaaacccCaugu......................................................................  1     1  te1
....................uuggcaacuuaaaUccuaugu......................................................................  2     1  te1
....................uuggUaacuuaaacccuaugu......................................................................  1     1  te1
....................Cuggcaacuuaaacccuaugu......................................................................  4     1  te1
....................uAggcaacuuaaacccuaugu......................................................................  1     1  te1
....................uuggcaacuuaaaccUuaugu......................................................................  2     1  te1
....................uuggcaacCuaaacccuaugu......................................................................  1     1  te1
....................uuUgcaacuuaaacccuaugu......................................................................  1     1  te1
....................uuggcaacuuaaacccuaugu......................................................................  1753  0  te1
....................uuggcaacuuaaacccuaugG......................................................................  2     1  te1
....................uuggcaacuuaaacccuUugu......................................................................  2     1  te1
....................uuggcaUcuuaaacccuaugu......................................................................  2     1  te1
....................uuggcaacuuaCacccuaugu......................................................................  1     1  te1
....................Auggcaacuuaaacccuaugu......................................................................  5     1  te1
....................Guggcaacuuaaacccuaugu......................................................................  4     1  te1
....................uuggcaacuuCaacccuaugu......................................................................  1     1  te1
....................uuggcaGcuuaaacccuaugug.....................................................................  1     1  te1
....................uuggcaacuuCaacccuaugug.....................................................................  1     1  te1
....................uGggcaacuuaaacccuaugug.....................................................................  1     1  te1
....................uuggcaacuuaaacccuauguA.....................................................................  134   1  te1
....................uuggcaacuuaaacccuaugug.....................................................................  2703  0  te1
....................uuggcaacuuaaacccuaGgug.....................................................................  1     1  te1
....................Auggcaacuuaaacccuaugug.....................................................................  11    1  te1
....................uuggcaCcuuaaacccuaugug.....................................................................  2     1  te1
....................Cuggcaacuuaaacccuaugug.....................................................................  6     1  te1
....................uCggcaacuuaaacccuaugug.....................................................................  3     1  te1
....................uuggcaacuuaaacccuauguU.....................................................................  85    1  te1
....................uuggcaacuuaaaGccuaugug.....................................................................  1     1  te1
....................uuggcaacuuaaacccuaugCg.....................................................................  1     1  te1
....................uuggcaacCuaaacccuaugug.....................................................................  1     1  te1
....................uuggcaacuuaaacccCaugug.....................................................................  1     1  te1
....................uuggcaacuuaaaccUuaugug.....................................................................  2     1  te1
....................uuggcaUcuuaaacccuaugug.....................................................................  2     1  te1
....................uuggcaacuuaaacccuauguC.....................................................................  4     1  te1
....................uuggcaacuuaaaUccuaugug.....................................................................  1     1  te1
....................Guggcaacuuaaacccuaugug.....................................................................  5     1  te1
....................uuggUaacuuaaacccuaugug.....................................................................  1     1  te1
....................uuggcaacuuaaacccuUugug.....................................................................  2     1  te1
....................uuggcaacuuaaacUcuaugug.....................................................................  2     1  te1
....................uuggcaacuuaaacccuaugugu....................................................................  153   0  te1
....................uuggcaacuuaaacccuauguAu....................................................................  24    1  te1
....................uuggcaacuuaaacccuauguUu....................................................................  10    1  te1
....................uuggcaacuuaaacccuaugugC....................................................................  3     1  te1
....................uuggcaacuuaaacccuaugugG....................................................................  72    1  te1
....................uuggcaacuuaaacccuaugugA....................................................................  4183  1  te1
....................uGggcaacuuaaacccuaugugu....................................................................  1     1  te1
....................uuggcaacuuaaacccuauguguG...................................................................  1     1  te1
....................uuggcaacuuaaacccuauguUuu...................................................................  2     1  te1
....................uuggcaacuuaaacccuauguguu...................................................................  11    0  te1
....................uuggcaacuuaaacccuauguguA...................................................................  5     1  te1
....................uuggcaacuuaaacccuaugugAu...................................................................  50    1  te1
....................uuggcaacuuaaacccuauguguuu..................................................................  2     0  te1
....................uuggcaacuuaaacccuauguguuA..................................................................  1     1  te1
....................uuggcaacuuaaacccuauguguuuc.................................................................  55    0  te1
....................uuggcaacuuaaacccuauguguuuA.................................................................  1     1  te1
....................uuggcaacuuaaacccuauguguuuca................................................................  7     0  te1
.....................uggcaacuuaaacccuaug.......................................................................  1     0  te1
.....................uggcaacuuaaacccuaugu......................................................................  3     0  te1
.....................uggcaacuuaaacccuaugug.....................................................................  10    0  te1
.....................Gggcaacuuaaacccuaugug.....................................................................  1     1  te1
.....................uggcaacuuaaacccuaugugA....................................................................  17    1  te1
.....................uggcaacuuaaacccuauguguuuca................................................................  1     0  te1
......................ggcaacuuaaacccuaugu......................................................................  2     0  te1
......................ggcaacuuaaacccuaugug.....................................................................  4     0  te1
......................ggcaacuuaaacccuaugugu....................................................................  1     0  te1
......................ggcaacuuaaacccuaugugA....................................................................  3     1  te1
.......................gcaGcuuaaacccuaugug.....................................................................  1     1  te1
........................caacuuaaacccuaugugA....................................................................  8     1  te1
```

Mature **Star**

```
uucaccuacugacuguuguuuuggcaacuuaaacccuauguguuucaaaaaauuuuguccauaagguuuccguugucaugccaacaauuagucacaacaaccacaacaaac
........................caacuuaaacccuauguUu....................................................................  1    1  te1
........................caacuuaaacccuauguguuuc.................................................................  1    0  te1
.........................aacuuaaacccuaugugA....................................................................  2    1  te1
..........................................uuucaaaaaauuuuguccauaag..............................................  1    0  te1
.........................................................uccauaagguuuccguugucaugc..............................  1    0  te1
...........................................................cauaagguuuccguugucau................................  17   0  te1
...........................................................cauaagguuuccguugucaGg...............................  2    1  te1
...........................................................cauaagguuCccguugucaug...............................  1    1  te1
...........................................................cauaGgguuuccguugucaug...............................  1    1  te1
...........................................................cauaagguuuccguugucaug...............................  134  0  te1
...........................................................cauaagguuuccguugucaugA..............................  4    1  te1
...........................................................cauaagguuuccguugucaugc..............................  213  0  te1
...........................................................cauaaAguuuccguugucaugc..............................  1    1  te1
...........................................................cauaagguuuccguugucaugcU.............................  4    1  te1
...........................................................cauaagguuuccguugucaugcA.............................  29   1  te1
...........................................................cauaagguuuccguugucaugcG.............................  2    1  te1
...........................................................cauaagguuuccguugucaugcca............................  1    0  te1
...........................................................cauaagguuuccguugucaugcUa............................  2    1  te1
............................................................auaagguuuccguugucaug...............................  18   0  te1
............................................................auaagguuuccguugucaugA..............................  2    1  te1
............................................................auaagguuuccguugucaugc..............................  43   0  te1
............................................................auaagguuuccguugCcaugc..............................  1    1  te1
............................................................auaagguuuccguugucaugcA.............................  17   1  te1
............................................................auaagguuuccguugucaugcU.............................  45   1  te1
............................................................auaagguuuccguugucaugcc.............................  21   0  te1
............................................................auaagguuuccguugucaugcG.............................  1    1  te1
............................................................auaagguuuccguugucaugcAa............................  4    1  te1
............................................................auaagguuuccguugucaugccU............................  3    1  te1
............................................................auaagguuuccguugucaugcca............................  5    0  te1
............................................................auaagguuuccguugucaugcUa............................  10   1  te1
.............................................................uaagguuuccguugucaug...............................  1    0  te1
.............................................................uaagguuuccguugucaugA..............................  1    1  te1
.............................................................uaagguuuccguugucaugc..............................  22   0  te1
.............................................................uaagguuuccguugucaugcU.............................  18   1  te1
.............................................................uaagguuuccguugucaugcc.............................  15   0  te1
.............................................................uaagguuuccguugucaugcA.............................  2    1  te1
.............................................................uaagguuuccguugucaugccU............................  8    1  te1
.............................................................uaagguuuccguugucaugccG............................  1    1  te1
.............................................................uaagguuuccguugucaugcca............................  112  0  te1
.............................................................uaagguuuccguugucaugcAa............................  3    1  te1
.............................................................uaagguuuccguugucaugcUa............................  7    1  te1
.............................................................uaagguuuccguugucaugccaU...........................  1    1  te1
...............................................................agguuuccguugucaugccaa...........................  1    0  te1

..caccuCcugacuguuguu...........................................................................................  1    1  egg
..caccuacugacuguuguu...........................................................................................  1    0  egg
..................uuuuggcaacuuaaacccuaug.......................................................................  1    0  egg
....................uuggcaacuuaaacccuGugu......................................................................  1    1  egg
....................uuggcCacuuaaacccuaugu......................................................................  1    1  egg
....................uuggcaacuuaaacccuaugu......................................................................  12   0  egg
....................uuggcaacuCaaacccuaugug.....................................................................  1    1  egg
....................uuggcaacuuaaacccuaugug.....................................................................  48   0  egg
....................uuggUaacuuaaacccuaugug.....................................................................  1    1  egg
....................uuggcaacuuaaacccuaugugA....................................................................  3    1  egg
....................uuggcaacuuaaacccuaugugu....................................................................  4    0  egg
...........................................................cauaagguuuccguugucaugc..............................  1    0  egg

uucaccuacugacuguuguu...........................................................................................  1    0  T6P
.................Cuuuuggcaacuuaaacccuaugug.....................................................................  1    1  T6P
..................uuuuggcaacuuaaacccuau........................................................................  13   0  T6P
..................uuuuggcaacuuaaacccuaug.......................................................................  27   0  T6P
..................Auuuggcaacuuaaacccuaug.......................................................................  1    1  T6P
..................uuuuggcaacuuaaacccuaugu......................................................................  5    0  T6P
..................uuuuggcaacuuaaacccuauguU.....................................................................  1    1  T6P
..................uuuuggcaacuuaaacccuaugug.....................................................................  1    0  T6P
..................uuuuggcaacuuaaacccuaugugA....................................................................  1    1  T6P
...................uuuggcaacuuaaacccuaug.......................................................................  2    0  T6P
...................uuuggUaacuuaaacccuaugu......................................................................  1    1  T6P
...................uuuggcaacuuaaacccuaugu......................................................................  75   0  T6P
...................uuuggcaacuuaaacccuauguA.....................................................................  4    1  T6P
```

Mature **Star**

uucaccuacugacuguuguuuuggcaacuuaaacccuaugugguuucaaaaaauuuuguccauaagguuuccguugucaugccaacaauuagucacaacaaccacaacaaac			
...................uuuggcaacuuaaacccuaugug...	5	0	T6P
...................uuuggcaacuuaaacccuaugugA..	2	1	T6P
...................uuuggcaacuuaaacccuauguguuuc...	1	0	T6P
....................uuggcaacuuaaacccua...	1	0	T6P
....................uuggcaacuuaaacccuau..	1	0	T6P
....................uuggcaacuuaaacccuaug...	15	0	T6P
....................uuAgcaacuuaaacccuaugu..	1	1	T6P
....................uuggcaacuuaaaccUuaugu..	1	1	T6P
....................uuggcaacuuaaacccuaugA..	2	1	T6P
....................uuggUaacuuaaacccuaugu..	3	1	T6P
....................uuggcaacuuaaaUccuaugu..	1	1	T6P
....................uugCcaacuuaaacccuaugu..	1	1	T6P
....................uugAcaacuuaaacccuaugu..	1	1	T6P
....................uuggcaacuuaaacccuaugu..	1179	0	T6P
....................uuggcaUcuuaaacccuaugug...	2	1	T6P
....................uuggcaacuuaaacccuUugug...	1	1	T6P
....................uuggUaacuuaaacccuaugug...	10	1	T6P
....................uuggcaacuuaaacccuauguU...	44	1	T6P
....................uuggcaacuuaaGcccuaugug...	2	1	T6P
....................uuggcGacuuaaacccuaugug...	1	1	T6P
....................uuggcaacuuaaUcccuaugug...	1	1	T6P
....................uuggcaacuuaaacccuaAgug...	1	1	T6P
....................uuggGaacuuaaacccuaugug...	1	1	T6P
....................uAggcaacuuaaacccuaugug...	1	1	T6P
....................Cuggcaacuuaaacccuaugug...	1	1	T6P
....................uuggcaacuuaaacUcuaugug...	5	1	T6P
....................uuCgcaacuuaaacccuaugug...	1	1	T6P
....................uuggcaacuuaaaccUuaugug...	7	1	T6P
....................uGggcaacuuaaacccuaugug...	1	1	T6P
....................uuggcaacuuaaacccuauguC...	3	1	T6P
....................uuUgcaacuuaaacccuaugug...	2	1	T6P
....................uuggcaacuuaaacccuaugug...	5005	0	T6P
....................uuggcaacuuaaaUccuaugug...	4	1	T6P
....................uuggcaacuuaaacccuauguA...	73	1	T6P
....................Auggcaacuuaaacccuaugug...	2	1	T6P
....................uuggcaacuuUaacccuaugug...	1	1	T6P
....................uugUcaacuuaaacccuaugug...	3	1	T6P
....................uugAcaacuuaaacccuaugug...	1	1	T6P
....................uuggcaacuuaaaGccuaugug...	1	1	T6P
....................uuggcaacuuaaacccuaugugA..	2681	1	T6P
....................uuggcaacuuaaacccuauguAu..	14	1	T6P
....................uuggcaacuuaaacccuaugugG..	8	1	T6P
....................uuggcaacuuaaacccuaugugu..	214	0	T6P
....................uuggcaacuuaaacccuauguUu..	7	1	T6P
....................uuggcaacuuaaacccuaugugC..	3	1	T6P
....................uuggcaacuuaaacccuaugugAu...	38	1	T6P
....................uuggcaacuuaaacccuauguguu...	15	0	T6P
....................uuggcaacuuaaacccuauguguA...	3	1	T6P
....................uuggcaacuuaaacccuauguUuu...	1	1	T6P
....................uuggcaacuuaaacccuauguguuu..	4	0	T6P
....................uuggcaaUuuaaacccuauguguuu..	2	1	T6P
....................uuggcaacuuaaacccuauguguAu..	1	1	T6P
....................uuggcaacuuaaacccuauguguuA..	2	1	T6P
....................uuggcaacuuaaacccCauguguuuc...	1	1	T6P
....................uuggcaacuuaaacccuGuguguuuc...	1	1	T6P
....................uuggcaacuCaaacccuauguguuuc...	1	1	T6P
....................uuggcaacuuaaacccuauguguuuc...	82	0	T6P
....................uuggcaacuuaaacccuauguguuuca..	8	0	T6P
....................uuggcaacuuaaacccuauguguuucU..	1	1	T6P
.....................uggcaacuuaaacccuaugu..	1	0	T6P
.....................uggcaacuuaaacccuaugug...	9	0	T6P
.....................uggcaacuuaaacccuauguA...	1	1	T6P
.....................uggcaacuuaaacccuaugugu..	2	0	T6P
.....................uggcaacuuaaacccuaugugA..	4	1	T6P
......................ggcaacuuaaacccuaugug...	2	0	T6P
........................caacuuaaacccuaugugA..	1	1	T6P
....uuguccauaagguuuccguugucaugc..............................	1	0	T6P
....uguccauaagguuuccguugucaugc..............................	1	0	T6P
....uccauaagguuuccguugucau................................	1	0	T6P
....uccauaagguuuccguugucaugc..............................	2	0	T6P

```
                    Mature                                 Star
uucaccuacugacuguuguuuuggcaacuuaaacccuaugugguuucaaaaaauuuuguccauaagguuuccguugucaugccaacaauuagucacaacaaccacaacaaac
...........................................................cauaagguuuccguugucau................................    2     0    T6P
...........................................................cauaagguuuccguugucaug...............................    12    0    T6P
...........................................................cauaagguuuccguugucaugU..............................    1     1    T6P
...........................................................cauaagguuuccguugucaAgc..............................    1     1    T6P
...........................................................cauaagCuuuccguugucaugc..............................    1     1    T6P
...........................................................cauaagguuuccguugucaugc..............................    81    0    T6P
...........................................................cauaagguuuccguugucaugcA.............................    4     1    T6P
............................................................auaagguuuccguugucau................................    1     0    T6P
............................................................auaagguuuccguugucaug...............................    2     0    T6P
............................................................auaagguuuccguugucaugc..............................    18    0    T6P
............................................................auaagguuuccguugucaugcc.............................    9     0    T6P
............................................................auaagguuuccguugucaugcU.............................    13    1    T6P
............................................................auaagguuuccguugucaugcA.............................    2     1    T6P
............................................................auaagguuuccguugucaugcUa............................    1     1    T6P
............................................................auaagguuuccguugucaugcca............................    4     0    T6P
............................................................auaagguuuccguugucaugcAa............................    2     1    T6P
.............................................................uaagguuuccguugucaugc..............................    7     0    T6P
.............................................................uaagguuuccguugucaugcc.............................    20    0    T6P
.............................................................uaagguuuccguugucaugcA.............................    1     1    T6P
.............................................................uaagguuuccguugucaugcU.............................    6     1    T6P
.............................................................uaagguuuccguugucaugcca............................    74    0    T6P
.............................................................uaagguuuccguugucaugcUa............................    5     1    T6P
...............................................................agguuuccguugucaugcca............................    1     0    T6P
.................................................................................caacaauuagucacaacaaccac.......    1     0    T6P

.ucaccuacugacuguuguu...........................................................................................    1     0    T53
..caccuacugacuguuguu...........................................................................................    9     0    T53
.................Uuuuuggcaacuuaaacccuaug.......................................................................    1     1    T53
..................uuuuggcaacuuaaacccuau........................................................................    14    0    T53
..................uuuuggcaacuuaaacccuaug.......................................................................    59    0    T53
..................uuuuggcaacuuaaUcccuaug.......................................................................    1     1    T53
..................uuuuggcaacuuaaacccuauU.......................................................................    2     1    T53
..................uuuuggcaacuuaaacccuaugu......................................................................    16    0    T53
..................uuuuggcaacuuaaacccuaugA......................................................................    5     1    T53
..................uuuuggcaacuuaaacccuauguA.....................................................................    1     1    T53
..................uuuuggcaacuuaaacccuauguU.....................................................................    4     1    T53
..................uuuuggcaacuuaaacccuauguUu....................................................................    1     1    T53
..................uuuuggcaacuuaaacccuauguCu....................................................................    1     1    T53
..................uuuuggcaacuuaaacccuauguUuu...................................................................    1     1    T53
..................uuuuggcaacuuaaacccuauguguuu..................................................................    1     0    T53
...................uuuggcaacuuaaacccuau........................................................................    1     0    T53
...................uuuggcaacuuaaacccuaug.......................................................................    6     0    T53
...................uuuggcaacuuaaacccuaugA......................................................................    2     1    T53
...................uuuggcaacuuaaacccuaugu......................................................................    72    0    T53
...................uuuggUaacuuaaacccuaugu......................................................................    1     1    T53
...................uuuggcaacuuaaacccuaugug.....................................................................    2     0    T53
...................uuuggcaacuuaaacccuauguA.....................................................................    4     1    T53
...................uuuggcaacuuaaacccuauguU.....................................................................    5     1    T53
...................uuuggcaacuuaaacccuaugugA....................................................................    5     1    T53
...................uuuggcaacuuaaacccuauguguuuc.................................................................    13    0    T53
....................uuggcaacuuaaacccua.........................................................................    1     0    T53
....................uuggcaacuuaUacccuau........................................................................    1     1    T53
....................uuggcaacuuaaacccuau........................................................................    21    0    T53
....................uuggcaacuuaaaccAuaug.......................................................................    1     1    T53
....................uuggcaacuuaaacccuauA.......................................................................    1     1    T53
....................uuggcaacuuaaacccuauU.......................................................................    3     1    T53
....................uuggcaacuuaaacccuaug.......................................................................    54    0    T53
....................uuggcaacuuaaacccuaugA......................................................................    14    1    T53
....................uuggcaacuuaaacccAaugu......................................................................    1     1    T53
....................uugCcaacuuaaacccuaugu......................................................................    1     1    T53
....................Guggcaacuuaaacccuaugu......................................................................    3     1    T53
....................uugAcaacuuaaacccuaugu......................................................................    2     1    T53
....................uuggcaacuuaaacccuauUu......................................................................    1     1    T53
....................uuggcCacuuaaacccuaugu......................................................................    1     1    T53
....................uuggcaacuCaaacccuaugu......................................................................    1     1    T53
....................uuggcaacuuaaaUccuaugu......................................................................    1     1    T53
....................uuggcaacuuaaacccuaugu......................................................................    2153  0    T53
....................uuggcaacuuaaacccuaugC......................................................................    1     1    T53
....................uuggcaaAuuaaacccuaugu......................................................................    1     1    T53
....................uuggcaacCuaaacccuaugu......................................................................    1     1    T53
```

```
                    Mature                                 Star
uucaccuacugacuguuguuuuggcaacuuaaacccuaugugguuucaaaaaauuuuguccauaagguuuccguugucaugccaacaauuagucacaacaaccacaacaaac
....................uuggcGacuuaaacccuaugu......................................................................   1      1      T53
....................uuggcaacuuaaacUcuaugu......................................................................   1      1      T53
....................uCggcaacuuaaacccuaugu......................................................................   1      1      T53
....................Auggcaacuuaaacccuaugu......................................................................   1      1      T53
....................uuggUaacuuaaacccuaugu......................................................................   7      1      T53
....................uugUcaacuuaaacccuaugu......................................................................   2      1      T53
....................uuggcaacuuaaaccUuaugu......................................................................   1      1      T53
....................Auggcaacuuaaacccuaugug.....................................................................   4      1      T53
....................uuAgcaacuuaaacccuaugug.....................................................................   1      1      T53
....................uuggcaacuAaaacccuaugug.....................................................................   1      1      T53
....................uuggcUacuuaaacccuaugug.....................................................................   1      1      T53
....................uuggcaacuuaaacccuaugCg.....................................................................   1      1      T53
....................uuggcaacuuaaacccuauCug.....................................................................   1      1      T53
....................uuggcaacuuaaaccAuaugug.....................................................................   1      1      T53
....................uuggcGacuuaaacccuaugug.....................................................................   1      1      T53
....................Cuggcaacuuaaacccuaugug.....................................................................   1      1      T53
....................Guggcaacuuaaacccuaugug.....................................................................   1      1      T53
....................uuggcaacuuaaacccuaugAg.....................................................................   1      1      T53
....................uuggcaacuuaaacccuauguC.....................................................................   14     1      T53
....................uuggcaGcuuaaacccuaugug.....................................................................   1      1      T53
....................uuggcaacuuaaacccCaugug.....................................................................   3      1      T53
....................uuggcaacuuaaaGccuaugug.....................................................................   1      1      T53
....................uuggUaacuuaaacccuaugug.....................................................................   9      1      T53
....................uuggcaacuuaaacccuGugug.....................................................................   1      1      T53
....................uuggcaacuuaaacUcuaugug.....................................................................   2      1      T53
....................uuggcaacuuaaacccuauguA.....................................................................   189    1      T53
....................uuggcaacuuaaacccuauguU.....................................................................   429    1      T53
....................uuggcaacuuaUacccuaugug.....................................................................   1      1      T53
....................uuUgcaacuuaaacccuaugug.....................................................................   1      1      T53
....................uuggcaacuuaaacccuaugug.....................................................................   3485   0      T53
....................uugCcaacuuaaacccuaugug.....................................................................   1      1      T53
....................uCggcaacuuaaacccuaugug.....................................................................   1      1      T53
....................uuggcaaUuuaaacccuaugug.....................................................................   1      1      T53
....................uugAcaacuuaaacccuaugug.....................................................................   5      1      T53
....................uuggcaacuuaaaccUuaugug.....................................................................   1      1      T53
....................uuggcaacuuaaaAccuaugug.....................................................................   1      1      T53
....................uuggcaacAuaaacccuaugug.....................................................................   1      1      T53
....................uuggcaacuuaaacccuauguAu....................................................................   59     1      T53
....................uuggcaacuuaaacccuauguUu....................................................................   49     1      T53
....................uuggcaacuuaaacccuaugugC....................................................................   4      1      T53
....................uuggcaacuuaaacccuaugugG....................................................................   28     1      T53
....................uuggcaacuuaaacUcuaugugu....................................................................   1      1      T53
....................uuggcaacuuaaacccuauguCu....................................................................   3      1      T53
....................uuggcaacuuaaacccuaugugA....................................................................   3537   1      T53
....................uugCcaacuuaaacccuaugugu....................................................................   1      1      T53
....................uuggcaacuuaaacccuaAgugu....................................................................   1      1      T53
....................uuggcGacuuaaacccuaugugu....................................................................   1      1      T53
....................Guggcaacuuaaacccuaugugu....................................................................   1      1      T53
....................uuggcaacuuaaacccuaugugu....................................................................   361    0      T53
....................uuggcaacuuaaacccuauguguu...................................................................   138    0      T53
....................uugAcaacuuaaacccuauguguu...................................................................   1      1      T53
....................uuggcaacuuaaacccuauguguA...................................................................   4      1      T53
....................uuggcaacuuaaacccuauguAuu...................................................................   2      1      T53
....................uuggcaacuuaaacccuauguUuu...................................................................   2      1      T53
....................uuggcaacuuaaacccuaugugAu...................................................................   128    1      T53
....................uuggcaacuuaaacccuauguguuA..................................................................   5      1      T53
....................uuggcaaUuuaaacccuauguguuu..................................................................   1      1      T53
....................uuggcaacuuaaacccuaugugAuu..................................................................   7      1      T53
....................uuggcaacuuaaacccuauguUuuu..................................................................   1      1      T53
....................uuggcaacuuaaacccuauguguAu..................................................................   3      1      T53
....................uuggcaacuuaaacccuauguguuC..................................................................   1      1      T53
....................uuggcaacuuaaacccuauguguuu..................................................................   48     0      T53
....................uuggcaacuuaaacccuauguUuuuc.................................................................   1      1      T53
....................uugUcaacuuaaacccuauguguuuc.................................................................   1      1      T53
....................uuggcaacuuaaacccAauguguuuc.................................................................   1      1      T53
....................uuggcaacCuaaacccuauguguuuc.................................................................   1      1      T53
....................uuUgcaacuuaaacccuauguguuuc.................................................................   1      1      T53
....................uCggcaacuuaaacccuauguguuuc.................................................................   1      1      T53
....................uuggcaacuuaaacUcuauguguuuc.................................................................   1      1      T53
....................uuggcaacuuaaaUccuauguguuuc.................................................................   1      1      T53
```

```
                    Mature                                    Star
uucaccuacugacuguuguuuuggcaacuuaaacccuauguguuucaaaaaauuuuguccauaagguuuccguugucaugccaacaauuagucacaacaaccacaacaaac
....................uuggcaacuCaaacccuauguguuuc.................................................................    1     1   T53
....................uuggcaacuuaaacccuauguguuuc.................................................................    1201  0   T53
....................Cuggcaacuuaaacccuauguguuuc.................................................................    1     1   T53
....................uugAcaacuuaaacccuauguguuuc.................................................................    1     1   T53
....................uuggcaacuuaaaccUuauguguuuc.................................................................    1     1   T53
....................uuggcaacuuaaacccuauguAuuuc.................................................................    1     1   T53
....................uuggcaacuuaaacccuauguguuuU.................................................................    1     1   T53
....................uuggcaacuuaaacccuauguguAuc.................................................................    1     1   T53
....................Auggcaacuuaaacccuauguguuuc.................................................................    1     1   T53
....................uuggcaacuuaaacccuGuguguuuc.................................................................    1     1   T53
....................uuggUaacuuaaacccuauguguuuc.................................................................    4     1   T53
....................Guggcaacuuaaacccuauguguuuc.................................................................    1     1   T53
....................uuggcaacuuaaaUccuauguguuuca................................................................    1     1   T53
....................uuggcaacuuaaacccuauguguuucU................................................................    1     1   T53
....................uuggcaacuuaaacccuauguguuuca................................................................    59    0   T53
.....................uggcaacuuaaacccuaugu......................................................................    5     0   T53
.....................uggcaacuuaaacccuaugug.....................................................................    6     0   T53
.....................uggcaacuuaaacccuaugugA....................................................................    8     1   T53
.....................uggcaacuuaaacccuaugugu....................................................................    1     0   T53
.....................uggcaacuuaaacccuauguguuuc.................................................................    1     0   T53
......................ggcaacuuaaacccuaugu......................................................................    1     0   T53
......................ggcaacuuaaacccCaugug.....................................................................    1     1   T53
......................ggcaacuuaaacccuauguguu...................................................................    2     0   T53
........................caacuuaaacccuaugug.....................................................................    2     0   T53
........................caacuuaaacccuaugugA....................................................................    5     1   T53
........................caacuuaaacccuauguguuuc.................................................................    1     0   T53
.....................................................uuuguccauaagguuuccguu.....................................    1     0   T53
.....................................................uuuguccauaagguuuccguug....................................    1     0   T53
......................................................uuguccauaagguuuccguugucaugc..............................    2     0   T53
.......................................................uguccauaagguuuccguugucaugcc.............................    1     0   T53
.........................................................uccauaagguuuccguuguc..................................    2     0   T53
.........................................................uccauaagguuuccguugucU.................................    1     1   T53
.........................................................uccauaagguuuccguuguca.................................    3     0   T53
.........................................................uccauaagguuuccguugucau................................    4     0   T53
.........................................................uccauaagguuuccguugucaugc..............................    4     0   T53
.........................................................uccauaagguuuccguugucaugcU.............................    1     1   T53
..........................................................ccauaagguuuccguugucau................................    1     0   T53
..........................................................Ucauaagguuuccguugucaugc..............................    2     1   T53
...........................................................cauaagguuuccguuguc..................................    2     0   T53
...........................................................cauaagguuuccgAuguc..................................    1     1   T53
...........................................................cauaagguuuccguugucU.................................    3     1   T53
...........................................................cauaagguuuccguuguca.................................    2     0   T53
...........................................................cauaagguuuccguugucau................................    67    0   T53
...........................................................cauaGgguuuccguugucaug...............................    2     1   T53
...........................................................cauaagguuuccguugucauA...............................    3     1   T53
...........................................................cauaagAuuuccguugucaug...............................    1     1   T53
...........................................................cauaaggAuuccguugucaug...............................    2     1   T53
...........................................................cauaagguuuccguugucauU...............................    5     1   T53
...........................................................cauaagguuuccguugucauC...............................    1     1   T53
...........................................................cauaagguuuccguugucaug...............................    584   0   T53
...........................................................cauaagguuuccguugAcaug...............................    2     1   T53
...........................................................cauaagguuuccguugucUug...............................    1     1   T53
...........................................................cauaGgguuuccguugucaugc..............................    9     1   T53
...........................................................cauaagguuuccguugucaAgc..............................    1     1   T53
...........................................................cauaagguuuccguugucaugA..............................    9     1   T53
...........................................................Uauaagguuuccguugucaugc..............................    1     1   T53
...........................................................cauaagguuuccguugucaugU..............................    11    1   T53
...........................................................cauaUgguuuccguugucaugc..............................    1     1   T53
...........................................................cauaagguuuccguuguUaugc..............................    1     1   T53
...........................................................cauaagguuucUguugucaugc..............................    1     1   T53
...........................................................cauaagguuuUcguugucaugc..............................    1     1   T53
...........................................................cauaagguuuccguugucaugc..............................    1594  0   T53
...........................................................cauaagguuuccgAugucaugc..............................    6     1   T53
...........................................................cauaagguuucGguugucaugc..............................    1     1   T53
...........................................................cauaagguuuccguugGcaugc..............................    1     1   T53
...........................................................cauaagguuuccguugAcaugc..............................    2     1   T53
...........................................................cauaagguuuccAuugucaugc..............................    1     1   T53
...........................................................cauaagguuuccguugucaugcU.............................    32    1   T53
...........................................................cauaagguuuccguugucaugcc.............................    3     0   T53
...........................................................cauaagguuuccguugucaugcG.............................    2     1   T53
```

Mature **Star**

```
uucaccuacugacuguuguuuuggcaacuuaaacccuaugugguuucaaaaaauuuugucccauaagguuuccguugucaugccaacaauuagucacaacaaccacaacaaac
...........................................................cauaagguuuccguugucaugcA.............................  159  1  T53
...........................................................cauaagguuuccguugucaugcca............................  3    0  T53
...........................................................cauaagguuuccguugucaugcAa............................  11   1  T53
...........................................................cauaagguuuccguugucaugcUa............................  2    1  T53
............................................................auaagguuuccguugucau................................  13   0  T53
............................................................auaGgguuuccguugucau................................  1    1  T53
............................................................auaagguuuccguugCcaug...............................  1    1  T53
............................................................auaagguuuccguugucaug...............................  51   0  T53
............................................................auaagguuuccguugucauA...............................  1    1  T53
............................................................auaGgguuuccguugucaug...............................  1    1  T53
............................................................auaagguuuccguugucaugA..............................  2    1  T53
............................................................auaagguuuccguugucauAc..............................  4    1  T53
............................................................auaagguAuccguugucaugc..............................  1    1  T53
............................................................auaagguuuccguAgucaugc..............................  1    1  T53
............................................................auaGgguuuccguugucaugc..............................  1    1  T53
............................................................auGagguuuccguugucaugc..............................  2    1  T53
............................................................auaagguuuccguugucaugc..............................  259  0  T53
............................................................auaagguuuUcguugucaugc..............................  1    1  T53
............................................................auaagguuuccguugucaugcA.............................  96   1  T53
............................................................auaagguuuccgAugucaugcc.............................  1    1  T53
............................................................auaGgguuuccguugucaugcc.............................  1    1  T53
............................................................auaagguuucUguugucaugcc.............................  1    1  T53
............................................................auaagguuuccguugucaugcG.............................  1    1  T53
............................................................auaagguuuccguugucaugcU.............................  230  1  T53
............................................................auaagguuuccguugucaugcc.............................  121  0  T53
............................................................auaagguuuccguugucaugcAa............................  10   1  T53
............................................................auaagguuuccguugucaugccU............................  14   1  T53
............................................................auaGgguuuccguugucaugcca............................  2    1  T53
............................................................auaagguuuccguugucaugcUa............................  52   1  T53
............................................................auaagguuuccguugucaugcca............................  44   0  T53
............................................................auaagguuuccguugucauAcca............................  1    1  T53
............................................................auaagguuuccguugucaugccC............................  2    1  T53
............................................................auaagguuuccguugucaugcGa............................  1    1  T53
............................................................auaagguuuccguugucaugcAaa...........................  2    1  T53
............................................................auaagguuuccguugucaugcUaa...........................  1    1  T53
.............................................................uaagguuuccguugucau................................  5    0  T53
.............................................................uaagguuuccguugucaug...............................  11   0  T53
.............................................................uaagguuuccguugucauA...............................  1    1  T53
.............................................................uaagguuuccguugucaugc..............................  193  0  T53
.............................................................uaGgguuuccguugucaugc..............................  4    1  T53
.............................................................uaagguuuccguugucaugA..............................  5    1  T53
.............................................................uaagguuuccguugucaugcc.............................  275  0  T53
.............................................................Aaagguuuccguugucaugcc.............................  1    1  T53
.............................................................uaagCuuuccguugucaugcc.............................  1    1  T53
.............................................................uaagguuuccguugucaugcU.............................  115  1  T53
.............................................................uaGgguuuccguugucaugcc.............................  7    1  T53
.............................................................uaagguuuccguugucaugcA.............................  19   1  T53
.............................................................uaagguuuccguugucaugUc.............................  1    1  T53
.............................................................uaagguuuccguuguUaugcc.............................  2    1  T53
.............................................................uaagguuuccguugucauAcc.............................  1    1  T53
.............................................................uaagguuuccguAgucaugcca............................  1    1  T53
.............................................................uGagguuuccguugucaugcca............................  2    1  T53
.............................................................uaagguuuccguuguUaugcca............................  1    1  T53
.............................................................uaagguuucUguugucaugcca............................  1    1  T53
.............................................................uaaggGuuccguugucaugcca............................  1    1  T53
.............................................................uaaAguuuccguugucaugcca............................  1    1  T53
.............................................................uaagguuuccguugucaugcUa............................  57   1  T53
.............................................................uaagguuuccguugucaugcAa............................  16   1  T53
.............................................................uaagguuuccguugucaugccU............................  66   1  T53
.............................................................uaagCuuuccguugucaugcca............................  1    1  T53
.............................................................uaagAuuuccguugucaugcca............................  1    1  T53
.............................................................uaagguuuccguugucauAcca............................  1    1  T53
.............................................................uaagguAuccguugucaugcca............................  1    1  T53
.............................................................uCagguuuccguugucaugcca............................  1    1  T53
.............................................................uaagguuuccguugucaugUca............................  3    1  T53
.............................................................uaaCguuuccguugucaugcca............................  1    1  T53
.............................................................uaagguuuccguugucaugcca............................  891  0  T53
.............................................................uaGgguuuccguugucaugcca............................  31   1  T53
.............................................................uaagguuuccguugAcaugcca............................  2    1  T53
.............................................................uaagguuuccguugucaugcUaa...........................  1    1  T53
```

```
                    Mature                                      Star
uucaccuacugacuguuguuuuggcaacuuaaacccuauguguuucaaaaaauuuuguccauaagguuuccguugucaugccaacaauuagucacaacaaccacaacaaac
.............................................................uaagguuuccguugucaugccaU...........................   8      1      T53
..............................................................aagguuuccguugucaugcU.............................   1      1      T53
..............................................................aagguuuccguugucaugcUa............................   1      1      T53
..............................................................aagguuuccguugucaugcca............................   6      0      T53
...............................................................agguuuccguugucaugc..............................   2      0      T53
...............................................................agguuuccguugucaugccaa...........................   1      0      T53

.ucaccuacugacuguuguu...........................................................................................   1      0      MOL
..caccuacugacuguuguu...........................................................................................   1      0      MOL
.................Uuuuuggcaacuuaaacccuaug.......................................................................   1      1      MOL
..................uuuuggcaacuuaaacccuau........................................................................   5      0      MOL
..................uuuuggcaacuuaaacccuaug.......................................................................   18     0      MOL
..................uuuuggcaacuuaaacccuaugA......................................................................   4      1      MOL
..................uuuuggcaacuuaaacccuaugu......................................................................   5      0      MOL
..................uuuuggcaacuuaaacccuaugugA....................................................................   1      1      MOL
..................uuuuggcaacuuaaacccuauguUu....................................................................   1      1      MOL
..................uuuuggcaacuuaaacccuauguguuu..................................................................   1      0      MOL
...................uuuggcaacuuaaacccua.........................................................................   1      0      MOL
...................uuuggcaacuuaaacccuaug.......................................................................   3      0      MOL
...................uuuggcaacuuaaacccuaugu......................................................................   78     0      MOL
...................uuuggcaacuuaaacccuaugug.....................................................................   2      0      MOL
...................Auuggcaacuuaaacccuaugug.....................................................................   1      1      MOL
...................uuuggcaacuuaaacccuauguU.....................................................................   5      1      MOL
...................uuuggcaacuuaaacccuauguA.....................................................................   1      1      MOL
...................uuuggcaacuuaaacccuaugugA....................................................................   2      1      MOL
...................uuuggcaacuuaaacccuauguguuuc.................................................................   1      0      MOL
....................uuggcaacuuaaacccua.........................................................................   1      0      MOL
....................uuggcaacuuaaacccuau........................................................................   2      0      MOL
....................uuggcaacuuaaacccuaug.......................................................................   33     0      MOL
....................uuggcaacuuaaaccUuaugu......................................................................   1      1      MOL
....................uuggcaacuuaaacccuaugA......................................................................   3      1      MOL
....................uuggcaacuuaaacccAaugu......................................................................   2      1      MOL
....................Cuggcaacuuaaacccuaugu......................................................................   1      1      MOL
....................Auggcaacuuaaacccuaugu......................................................................   4      1      MOL
....................uuggcaacuuaaacccuaugu......................................................................   1361   0      MOL
....................uuggcaacuuaaaUccuaugu......................................................................   3      1      MOL
....................uuggcaacuuaaacccuaCgu......................................................................   1      1      MOL
....................uuggcaacuuaUacccuaugu......................................................................   2      1      MOL
....................uuggcaaUuuaaacccuaugu......................................................................   1      1      MOL
....................uuggcaacuuaaacccuauAu......................................................................   2      1      MOL
....................uugAcaacuuaaacccuaugu......................................................................   1      1      MOL
....................uCggcaacuuaaacccuaugug.....................................................................   1      1      MOL
....................uuggcaacuuaaacccuaAgug.....................................................................   1      1      MOL
....................uuggcaacuuaaacccuauAug.....................................................................   1      1      MOL
....................uuggcaacuuaaacccuauUug.....................................................................   1      1      MOL
....................uuggcaacuuaaaccUuaugug.....................................................................   4      1      MOL
....................uuggcaacuuaUacccuaugug.....................................................................   1      1      MOL
....................uuggcaUcuuaaacccuaugug.....................................................................   2      1      MOL
....................uuggcaacCuaaacccuaugug.....................................................................   1      1      MOL
....................uuggcUacuuaaacccuaugug.....................................................................   1      1      MOL
....................Cuggcaacuuaaacccuaugug.....................................................................   1      1      MOL
....................uuggcaacuuaaUcccuaugug.....................................................................   3      1      MOL
....................uugUcaacuuaaacccuaugug.....................................................................   1      1      MOL
....................Guggcaacuuaaacccuaugug.....................................................................   5      1      MOL
....................uuggcaacuuaaacccuaugGg.....................................................................   1      1      MOL
....................uuggcaacuuaaacccuaCgug.....................................................................   1      1      MOL
....................uuggUaacuuaaacccuaugug.....................................................................   5      1      MOL
....................uuggcaacuuaaacccuauguU.....................................................................   203    1      MOL
....................uuggcaacuuaaacccuaugAg.....................................................................   1      1      MOL
....................uuggcaacuuaaacccuauguA.....................................................................   104    1      MOL
....................uuggcaacuuaaacccuauguC.....................................................................   8      1      MOL
....................uuggcaacuuaaacAcuaugug.....................................................................   2      1      MOL
....................uuggcaacuuaaacccuGugug.....................................................................   1      1      MOL
....................uuggcaacuCaaacccuaugug.....................................................................   2      1      MOL
....................uuggcaacAuaaacccuaugug.....................................................................   2      1      MOL
....................Auggcaacuuaaacccuaugug.....................................................................   6      1      MOL
....................uuggcaacuuaaacccuaugug.....................................................................   3300   0      MOL
....................uuggcGacuuaaacccuaugug.....................................................................   1      1      MOL
....................uuggcaacCuaaacccuaugugu....................................................................   1      1      MOL
....................uuggcaacuuaaacccuaugugu....................................................................   381    0      MOL
```

```
                    Mature                                      Star
uucaccuacugacuguuguuuuggcaacuuaaacccuaugugguuucaaaaaauuuuguccauaagguuuccguugucaugccaacaauuagucacaacaaccacaacaaac
....................uuggcGacuuaaacccuaugugu....................................................................  1     1   MOL
....................uuggcaacuuaaacccuaugugC....................................................................  6     1   MOL
....................uuggcaacuuaaacccuaugugG....................................................................  10    1   MOL
....................uuggcaacuuaaacccuauguUu....................................................................  30    1   MOL
....................uuggcaacuuaaacccuauguAu....................................................................  61    1   MOL
....................uuggcaacuuGaacccuaugugu....................................................................  1     1   MOL
....................uuggcaacuuaaacccuaugugA....................................................................  2855  1   MOL
....................uugAcaacuuaaacccuaugugu....................................................................  1     1   MOL
....................Auggcaacuuaaacccuaugugu....................................................................  1     1   MOL
....................uuggcaacuuaaacccuauguguG...................................................................  1     1   MOL
....................uuggcaacuuaaacccuauguguA...................................................................  2     1   MOL
....................uuggcaacuuaaacccuaugugAu...................................................................  57    1   MOL
....................uuggcaacuuaaacccuauguUuu...................................................................  5     1   MOL
....................uuggcaacuuaaacccuauguguu...................................................................  31    0   MOL
....................uuggcaacuuaaacccuauguguAu..................................................................  2     1   MOL
....................uuggcaacuuaaacccuaugugAuu..................................................................  3     1   MOL
....................uuggcaacuuaaacccuauguguuu..................................................................  25    0   MOL
....................uuggcaUcuuaaacccuauguguuu..................................................................  1     1   MOL
....................Cuggcaacuuaaacccuauguguuuc.................................................................  1     1   MOL
....................uuggcaacuuaaacccuaCguguuuc.................................................................  1     1   MOL
....................uugAcaacuuaaacccuauguguuuc.................................................................  1     1   MOL
....................uuggcaacuGaaacccuauguguuuc.................................................................  1     1   MOL
....................uuggcaacAuaaacccuauguguuuc.................................................................  1     1   MOL
....................uuggcaacuuaaacccuauUuguuuc.................................................................  1     1   MOL
....................uuggcaacuuaaacccuauguguuuc.................................................................  277   0   MOL
....................uuggcaacuuaaacccuGuguguuuc.................................................................  1     1   MOL
....................uuggcaacuCaaacccuauguguuuc.................................................................  1     1   MOL
....................uuggcaacuuaaacccuauguAuuuc.................................................................  1     1   MOL
....................uuggcaacuuaaacccuauguAuuuca................................................................  1     1   MOL
....................Auggcaacuuaaacccuauguguuuca................................................................  1     1   MOL
....................uuggcaacuuaaacccuauguCuuuca................................................................  1     1   MOL
....................uuggcaacuuaaacccuauguguuuca................................................................  39    0   MOL
.....................uggcaacuuaaacccuaugu......................................................................  3     0   MOL
.....................uggcaacuuaaacccuaugug.....................................................................  2     0   MOL
.....................uggcaacuuaaacccuaugugA....................................................................  4     1   MOL
.....................uggcaacuuaaacccuaugugAu...................................................................  1     1   MOL
.....................uggcaacuuaaacccuauguguuuca................................................................  1     0   MOL
......................ggcaacuuaaacccuaugug.....................................................................  1     0   MOL
......................ggcaacuuaaacccuaugugA....................................................................  2     1   MOL
......................ggcaacuuaaacccuauguguuuc.................................................................  1     0   MOL
........................caacuuaaacccuaugug.....................................................................  3     0   MOL
........................caacuuaaacccuaugugA....................................................................  3     1   MOL
........................caacuuaaacccuauguguuucaa...............................................................  1     0   MOL
.............................uaaacccuauguguuucaa...............................................................  1     0   MOL
..........................................uuucaaaaaauuuuguccauaCgguu...........................................  1     1   MOL
.......................................................uguccauaagguuuccguugucaugc..............................  1     0   MOL
.........................................................uccauaagguuuccguuguca.................................  1     0   MOL
.........................................................uccauaagguuuccguugucau................................  3     0   MOL
..........................................................ccauaagguuuccguugucaugc..............................  1     0   MOL
...........................................................cauaagguuuccguuguca.................................  2     0   MOL
...........................................................cauaGgguuuccguugucau................................  1     1   MOL
...........................................................cauaagguuuccguugucau................................  18    0   MOL
...........................................................cauaagguuuccguCgucaug..............................  1     1   MOL
...........................................................cauaagguuuccguugucaGg..............................  1     1   MOL
...........................................................cauaagguuuccguugucaug..............................  104   0   MOL
...........................................................cauaGgguuuccguugucaug..............................  1     1   MOL
...........................................................cauCagguuuccguugucaug..............................  1     1   MOL
...........................................................cauaagguuuccguugucauU..............................  1     1   MOL
...........................................................cauGagguuuccguugucaug..............................  1     1   MOL
...........................................................cauaaggAuuccguugucaug..............................  1     1   MOL
...........................................................cauaagguuuccguugucaugA.............................  3     1   MOL
...........................................................cauaagguuuccguugucaugc.............................  175   0   MOL
...........................................................cauaagguuuccguugucaugU.............................  1     1   MOL
...........................................................cauaGgguuuccguugucaugc.............................  2     1   MOL
...........................................................cauaagguuuccguugucaugcA............................  23    1   MOL
...........................................................cauaagguuuccguugucaugcU............................  2     1   MOL
...........................................................cauaagguuuccguugucaugcca...........................  1     0   MOL
............................................................auaagguuuccguuguca.................................  1     0   MOL
............................................................auaagguuuccguugucau................................  5     0   MOL
............................................................auaagguuuccguugucaug...............................  10    0   MOL
```

```
                    Mature                                 Star
uucaccuacugacuguuguuuuggcaacuuaaacccuauguguuucaaaaaauuuuguccauaagguuuccguugucaugccaacaauuagucacaacaaccacaacaaac
............................................................auaagguuuccguAgucaugc..............................  1    1  MOL
............................................................auaagguuuccguugucaugc..............................  32   0  MOL
............................................................auaagCuuuccguugucaugc..............................  1    1  MOL
............................................................auaagguuuccguugucaugA..............................  1    1  MOL
............................................................auaagguuuccguugucaugcc.............................  24   0  MOL
............................................................auaagguuuccguugucaugcA.............................  14   1  MOL
............................................................auaagguuuUcguugucaugcc.............................  1    1  MOL
............................................................auaagguuuccguugucaugcU.............................  47   1  MOL
............................................................auaagguuuccguugucaugccU............................  1    1  MOL
............................................................auaagguuuccguugucaugcca............................  10   0  MOL
............................................................auaagguuuccguugucaugcAa............................  3    1  MOL
............................................................auaagguuuccguugucaugcUa............................  5    1  MOL
.............................................................uaagguuuccguugucaug...............................  1    0  MOL
.............................................................uaagguuuccguugucaugc..............................  23   0  MOL
.............................................................uaagguuuccguugucauCcc.............................  1    1  MOL
.............................................................uaagguuuccguugucaugcU.............................  14   1  MOL
.............................................................uaagguuuccguugucaugcc.............................  22   0  MOL
.............................................................uaagguuuccguugucaugcA.............................  4    1  MOL
.............................................................uaagguuuccguugucUugcca............................  1    1  MOL
.............................................................uaagguuuccgGugucaugcca............................  1    1  MOL
.............................................................uaGgguuuccguugucaugcca............................  4    1  MOL
.............................................................uaagguuuccguugucaugcAa............................  3    1  MOL
.............................................................Caagguuuccguugucaugcca............................  1    1  MOL
.............................................................uaagguuuccguAgucaugcca............................  1    1  MOL
.............................................................uaagguuuccguugucaugcca............................  169  0  MOL
.............................................................uaagguuuccguugucauAcca............................  1    1  MOL
.............................................................uaagguuuccguugucaugcUa............................  5    1  MOL
.............................................................uaagguuuccguugucaugccU............................  2    1  MOL
.............................................................uaagguuuccguugucaugccaU...........................  1    1  MOL
..............................................................aagguuuccguugucaugc..............................  1    0  MOL
..............................................................aagguuuccguugucaugcU.............................  1    1  MOL

.ucaccuacugacuguuguu...........................................................................................  4    0  T63
..caccuacugacuguuguu...........................................................................................  9    0  T63
.................Uuuuuggcaacuuaaacccuaug.......................................................................  2    1  T63
..................uuuuggcaacuuaaacccu..........................................................................  1    0  T63
..................uuuuggcaacuuaaacccua.........................................................................  1    0  T63
..................uuuuggcaacuuaaacccuau........................................................................  20   0  T63
..................uuuuggcaacuuaaacccuauU.......................................................................  1    1  T63
..................uuuuggcaacuuaaacccuaug.......................................................................  45   0  T63
..................uuuuggcaacuuaaUcccuaug.......................................................................  1    1  T63
..................Auuuggcaacuuaaacccuaugu......................................................................  1    1  T63
..................uuuuggcaacuuaaacccuaugA......................................................................  3    1  T63
..................uuuuggcaacuuaaacccuaugu......................................................................  6    0  T63
..................uuuuggcaacuuaaacccuauguU.....................................................................  3    1  T63
..................uuuuggcaacuuaaacccuauguA.....................................................................  3    1  T63
..................uuuuggcaacuuaaacccuauguUu....................................................................  3    1  T63
..................uuuuggcaacuuaaacccuauguguu...................................................................  1    0  T63
...................uuuggcaacuuaaacccuau........................................................................  2    0  T63
...................uuuggcaacuuaaacccuaug.......................................................................  2    0  T63
...................uuuggcaacuuaaacccuaugu......................................................................  47   0  T63
...................Auuggcaacuuaaacccuaugu......................................................................  1    1  T63
...................uuuggcaacuuaaacccuaugug.....................................................................  2    0  T63
...................uuuggcaacuuaaacccuauguU.....................................................................  1    1  T63
...................uuuggcaacuuaaacccuaugugA....................................................................  1    1  T63
...................uuuggcaacuuaaacccuauguguuuc.................................................................  7    0  T63
....................uuggcaacuuaaacccua.........................................................................  2    0  T63
....................uuggcaacuuaaacccuaA........................................................................  1    1  T63
....................uuggcaacuuaaacccuau........................................................................  18   0  T63
....................uuggcaacuuaaacccuaug.......................................................................  59   0  T63
....................uuggcaacuuaaacccuauU.......................................................................  2    1  T63
....................uuggcaacuuaaaUccuaug.......................................................................  1    1  T63
....................Auggcaacuuaaacccuaug.......................................................................  1    1  T63
....................uugUcaacuuaaacccuaugu......................................................................  2    1  T63
....................Auggcaacuuaaacccuaugu......................................................................  2    1  T63
....................uuggcaacuuaaacGcuaugu......................................................................  1    1  T63
....................uuggcaacuuaaaccUuaugu......................................................................  3    1  T63
....................uuggcaacuuaaacccuaugA......................................................................  10   1  T63
....................uuggcaacuuaaacccuauUu......................................................................  1    1  T63
....................uuggcaacuCaaacccuaugu......................................................................  1    1  T63
```

```
                    Mature                                      Star
uucaccuacugacuguuguuuuggcaacuuaaacccuauguguuucaaaaaauuuuguccauaagguuuccguugucaugccaacaauuagucacaacaaccacaacaaac
....................uuggcaacuuaCacccuaugu......................................................................    1      1    T63
....................uuggcaacuuaaaGccuaugu......................................................................    1      1    T63
....................Guggcaacuuaaacccuaugu......................................................................    1      1    T63
....................uuggcaacuuaaacccAaugu......................................................................    1      1    T63
....................uuggcaacuuaaacccuaugu......................................................................    1494   0    T63
....................uuggcaacCuaaacccuaugu......................................................................    1      1    T63
....................uuggcaacuuaaaccAuaugug.....................................................................    1      1    T63
....................uuggcaacuuaaacccuauguU.....................................................................    291    1    T63
....................uuggcaacuuaaacccuaCgug.....................................................................    1      1    T63
....................Guggcaacuuaaacccuaugug.....................................................................    1      1    T63
....................uuggcaaUuuaaacccuaugug.....................................................................    1      1    T63
....................uugCcaacuuaaacccuaugug.....................................................................    1      1    T63
....................uuggcaacuuaaacccuaugug.....................................................................    3367   0    T63
....................Cuggcaacuuaaacccuaugug.....................................................................    1      1    T63
....................uuggUaacuuaaacccuaugug.....................................................................    5      1    T63
....................uuggcaacuuaaacccCaugug.....................................................................    2      1    T63
....................uuggcaacuuaaaUccuaugug.....................................................................    4      1    T63
....................uugAcaacuuaaacccuaugug.....................................................................    2      1    T63
....................uuggcaacuuGaacccuaugug.....................................................................    1      1    T63
....................uuggcaacuuaaaccUuaugug.....................................................................    1      1    T63
....................uuggcaacAuaaacccuaugug.....................................................................    3      1    T63
....................uuggcaacuuaaUcccuaugug.....................................................................    1      1    T63
....................uuggcaacuuaaacccuauCug.....................................................................    1      1    T63
....................uuggcaacuuaUacccuaugug.....................................................................    1      1    T63
....................uuggcaacuuaaacccuUugug.....................................................................    1      1    T63
....................uuggcaacuuaaacccAaugug.....................................................................    1      1    T63
....................uuUgcaacuuaaacccuaugug.....................................................................    1      1    T63
....................uuggcaacuuaaacccuauguC.....................................................................    16     1    T63
....................Auggcaacuuaaacccuaugug.....................................................................    3      1    T63
....................uuggcaacuuaaacccuauUug.....................................................................    1      1    T63
....................uuAgcaacuuaaacccuaugug.....................................................................    1      1    T63
....................uuggcaacuuaaacccuauguA.....................................................................    109    1    T63
....................uuggcaacuuaaacccGaugug.....................................................................    2      1    T63
....................uuggcaacuuaaacUcuaugug.....................................................................    2      1    T63
....................uuggcaacuuaaacccuaugugG....................................................................    17     1    T63
....................uuggcaacuuaaacccuaugugA....................................................................    2019   1    T63
....................Auggcaacuuaaacccuaugugu....................................................................    2      1    T63
....................uuggcaacuuaaacccuaugugu....................................................................    187    0    T63
....................uuggcaacuuaaacccuauguUu....................................................................    39     1    T63
....................uuggUaacuuaaacccuaugugu....................................................................    1      1    T63
....................uuggcaacuuaaacccuaugAgu....................................................................    1      1    T63
....................uuggcaacuuaaacccuauguAu....................................................................    39     1    T63
....................uuggcaacuuaaacccuaugugC....................................................................    4      1    T63
....................uuggcaacuuaaacccuauguguu...................................................................    82     0    T63
....................uuggcaacuuaaacccuauguguA...................................................................    3      1    T63
....................uuggcaacuCaaacccuauguguu...................................................................    1      1    T63
....................uuggcaacuuaaacccuauguUuu...................................................................    5      1    T63
....................uuggcaacuuaaacccuaugugAu...................................................................    53     1    T63
....................Auggcaacuuaaacccuauguguuu..................................................................    1      1    T63
....................uuggcaacuuaaacccuauguguuA..................................................................    4      1    T63
....................uuggcaacuuaaacccuaugugAuu..................................................................    8      1    T63
....................uuggcaacuuaaacccuauguguAu..................................................................    3      1    T63
....................uuggcaacuuaaacccuauguguuu..................................................................    55     0    T63
....................uuggcaacuuaaacccuauCuguuuc.................................................................    1      1    T63
....................uuggcaacuuaaacccuauguguuuc.................................................................    1957   0    T63
....................uuggUaacuuaaacccuauguguuuc.................................................................    4      1    T63
....................uuggcaacAuaaacccuauguguuuc.................................................................    1      1    T63
....................uuggcaacuuaUacccuauguguuuc.................................................................    2      1    T63
....................uuggcaacuuaaacccuauUuguuuc.................................................................    1      1    T63
....................uuggcGacuuaaacccuauguguuuc.................................................................    2      1    T63
....................uuggcaacuuaaacccuauguguuuU.................................................................    1      1    T63
....................uuggcaacuuaaacccuauguguAuc.................................................................    1      1    T63
....................uuggcaacuuaaacccuaugGguuuc.................................................................    1      1    T63
....................uuggcaacuuaaacccuGuguguuuc.................................................................    1      1    T63
....................uuCgcaacuuaaacccuauguguuuc.................................................................    1      1    T63
....................uuggcaacuuaaacUcuauguguuuc.................................................................    1      1    T63
....................uuggcaacuuaaacccuaugugAuuc.................................................................    1      1    T63
....................uuggcaacuuaaacccAauguguuuc.................................................................    1      1    T63
....................uuggcaacuuaaacccuaugCguuuc.................................................................    1      1    T63
....................uuggcaacuuaaacccuauguAuuuc.................................................................    2      1    T63
```

```
                    Mature                                      Star
uucaccuacugacuguuguuuuggcaacuuaaacccuauguguuucaaaaaauuuuguccauaagguuuccguugucaugccaacaauuagucacaacaaccacaacaaac
....................uugCcaacuuaaacccuauguguuuc.................................................................   1     1   T63
....................Cuggcaacuuaaacccuauguguuuc.................................................................   2     1   T63
....................uuggcaacuuaGacccuauguguuuc.................................................................   1     1   T63
....................uuggcaacuCaaacccuauguguuuc.................................................................   1     1   T63
....................uuggcaacuuaaacccuauguguuuca................................................................   63    0   T63
.....................uggcaacuuaaacccuaugu......................................................................   2     0   T63
.....................uggcaacuuaaacccuaugug.....................................................................   8     0   T63
.....................uggcaacuuaaacccuauguU.....................................................................   1     1   T63
.....................Cggcaacuuaaacccuaugug.....................................................................   1     1   T63
.....................uggcaacuuaaacccuaugugA....................................................................   6     1   T63
.....................uggcaacuuaaacccuauguguuuc.................................................................   5     0   T63
.......................gcaacuuaaacccuaugugA....................................................................   1     1   T63
........................caacuuaaacccuaugug.....................................................................   3     0   T63
........................caacuuaaacccuauguguu...................................................................   3     0   T63
........................caacuuaaacccuauguguuuc.................................................................   1     0   T63
............................uuaaacccuauguguuuc.................................................................   1     0   T63
....................................................uuuuguccauaagguuuccguug....................................   1     0   T63
......................................................uuguccauaagguuuccguugucaugc..............................   2     0   T63
........................................................gGccauaagguuuccguugucaug................................   1     1   T63
.........................................................uccauaagguuuccguuguc..................................   1     0   T63
.........................................................uccauaagguuuccguuguca.................................   4     0   T63
.........................................................uccauaagguuuccguuguUa.................................   1     1   T63
.........................................................uccauaagguuuccguugucau................................   6     0   T63
.........................................................Accauaagguuuccguugucaugc..............................   1     1   T63
.........................................................uccauaagguuuccguugucaugc..............................   4     0   T63
..........................................................Ucauaagguuuccguugucaugc..............................   2     1   T63
...........................................................cauaagguuuccguuguc..................................   3     0   T63
...........................................................cauaagguuuccguuguca.................................   4     0   T63
...........................................................cauaagguAuccguuguca.................................   1     1   T63
...........................................................cauaagguuuccguuAucau................................   1     1   T63
...........................................................cauaagguuuccguugucaC................................   1     1   T63
...........................................................cauaagguuuccguugucau................................   52    0   T63
...........................................................cauaagguuucAguugucaug...............................   1     1   T63
...........................................................cauaagguuuccguAgucaug...............................   1     1   T63
...........................................................cauaagguuuccguugucauA...............................   1     1   T63
...........................................................cauaaCguuuccguugucaug...............................   2     1   T63
...........................................................cauaagguuuccguugucaAg...............................   1     1   T63
...........................................................cauaagguuuccguugucaug...............................   459   0   T63
...........................................................cauaagguuuccguugucauU...............................   1     1   T63
...........................................................cauaGgguuuccguugucaug...............................   4     1   T63
...........................................................cauaagguuuccguugucauUc..............................   2     1   T63
...........................................................cauGagguuuccguugucaugc..............................   1     1   T63
...........................................................cauaagguuuccguAgucaugc..............................   1     1   T63
...........................................................cauaagguuuccguugGcaugc..............................   2     1   T63
...........................................................cauaGgguuuccguugucaugc..............................   11    1   T63
...........................................................cauaagguuucUguugucaugc..............................   1     1   T63
...........................................................cauaagguuuccguugucauAc..............................   2     1   T63
...........................................................cauaagguAuccguugucaugc..............................   1     1   T63
...........................................................cauaagguuuccguugAcaugc..............................   3     1   T63
...........................................................cauaUgguuuccguugucaugc..............................   1     1   T63
...........................................................Uauaagguuuccguugucaugc..............................   1     1   T63
...........................................................cauaagguuuccgAugucaugc..............................   1     1   T63
...........................................................cauaagguuuccAuugucaugc..............................   2     1   T63
...........................................................cauaagguuuccguugucaugA..............................   6     1   T63
...........................................................caAaagguuuccguugucaugc..............................   1     1   T63
...........................................................cauaagguuuccguugucGugc..............................   1     1   T63
...........................................................cauaagguuuccguugucaugU..............................   5     1   T63
...........................................................cauaagguuuccguugucaAgc..............................   1     1   T63
...........................................................cauaagguuuccguugucaugc..............................   1451  0   T63
...........................................................cauaaUguuuccguugucaugc..............................   2     1   T63
...........................................................cauaagguuuccguugucaugcA.............................   104   1   T63
...........................................................cauaagguuuccguugucaugcc.............................   1     0   T63
...........................................................cauaagguuuccguugucaugcG.............................   1     1   T63
...........................................................cauaagguuuccguugucaugcU.............................   12    1   T63
...........................................................cauaagguuuccguugucaugcca............................   1     0   T63
...........................................................cauaagguuuccguugucaugcUa............................   2     1   T63
...........................................................cauaagguuuccguugucaugcAa............................   2     1   T63
............................................................auaagguuuccguAgucau................................   1     1   T63
............................................................auaagguuuccguugucau................................   11    0   T63
............................................................auaagguuuccguugucaug...............................   60    0   T63
```

Mature **Star**

uucaccuacugacuguuguuuuggcaacuuaaacccuaugugguuucaaaaaauuuugucauaagguuuccguugucaugccaacaauuagucacaacaaccacaacaaac

....auaagguuuccguugucaugU..............................	1	1	T63
....auaGgguuuccguugucaugc..............................	2	1	T63
....auaagguuuccguuguUaugc..............................	1	1	T63
....auaagguuuccguugucaugc..............................	318	0	T63
....auaagguuuccguugAcaugc..............................	1	1	T63
....auaagguuuccguAgucaugc..............................	1	1	T63
....auaagguuuccguugucaugA..............................	3	1	T63
....auaGgguuuccguugucaugcc.............................	2	1	T63
....auaagguuuccguugucaugcA.............................	78	1	T63
....auaagguuuccguugucaugcc.............................	120	0	T63
....auaagguuuccguugucaugcU.............................	289	1	T63
....auaagguuuccguugucaugcca............................	24	0	T63
....auaagguuuccguugucaugcAa............................	1	1	T63
....auaGgguuuccguugucaugcca............................	1	1	T63
....auaagguuuccguugucaugccU............................	8	1	T63
....auaagguuuccguugucaugcUa............................	24	1	T63
....auaagguuuccguugucaugcAaa...........................	1	1	T63
....auaagguuuccguugucaugccaU...........................	1	1	T63
....uaagguuuccguugucau................................	3	0	T63
....uaagguuuccguugucaug...............................	10	0	T63
....uaagguuuccguugucaugA..............................	2	1	T63
....uaagguuuccguugucauAc..............................	1	1	T63
....uaagguuuccguugucUugc..............................	1	1	T63
....uaagguuuccguugucaugc..............................	216	0	T63
....uaagguuuAcguugucaugc..............................	1	1	T63
....uaagguuuccguugucaugcc.............................	298	0	T63
....uaagguuuccguugucaugcG.............................	2	1	T63
....uaagguuuccguugucUugcc.............................	1	1	T63
....uaagguuuccguugucaugcA.............................	32	1	T63
....uaagguuucUguugucaugcc.............................	1	1	T63
....uaagguuuccguugucaugUc.............................	1	1	T63
....uaGgguuuccguugucaugcc.............................	3	1	T63
....uaagguuuccguugucaugcU.............................	96	1	T63
....uaGgguuuccguugucaugcca............................	8	1	T63
....uaagguuuccguugucauAcca............................	4	1	T63
....uaagguuuccguugucaugcGa............................	1	1	T63
....uaagguuuccguugucaugcUa............................	37	1	T63
....uaagguuuccguugucaugccU............................	60	1	T63
....uaagguuuccguugucaAgcca............................	1	1	T63
....uaagguuuccguugucaugcca............................	728	0	T63
....uaagguuuccguugucaugUca............................	2	1	T63
....uaagguAuccguugucaugcca............................	1	1	T63
....uaagguuuccguugucaugcAa............................	9	1	T63
....uaagguuuccguugucaugccaU...........................	2	1	T63
....aagguuuccguugucaugcc.............................	3	0	T63
....aagguuuccguugucaugcca............................	2	0	T63
....aagguuuccguugucaugcUa............................	1	1	T63
....agguuuccguugucaugcU.............................	1	1	T63
....agguuuccguugucaugcca............................	1	0	T63
....agguuuccguugucaugccaa...........................	1	0	T63

Provisional ID	:	Scaffold_1043_2834
Score total	:	778854.8
Score for star read(s)	:	3.9
Score for read counts	:	778844.7
Score for mfe	:	1.6
Score for randfold	:	1.6
Score for cons. seed	:	3
Total read count	:	1527682
Mature read count	:	1525308
Loop read count	:	0
Star read count	:	2374

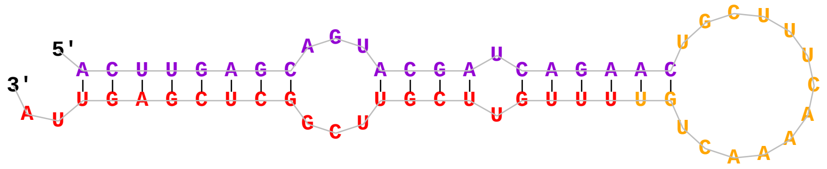

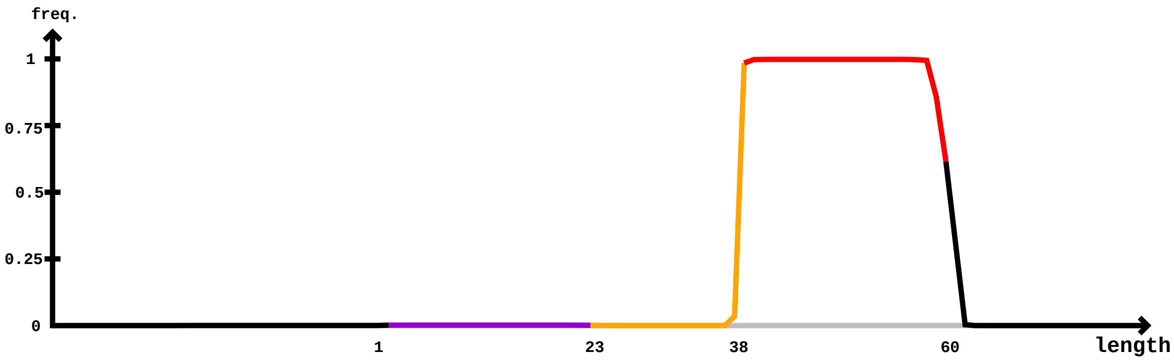

Star   Mature

```
5'- gaaggauacugucuuuauaagacuagugugguaacuugagcaguacgaucagaacugcuuucaaaacuguuuuguucguucggcucgaguuaucaaguggucaaaaugacuu   -3'    obs
    gaaggauacugucuuuauaagacuagugugguaacuugagcaguacgaucagaacugcuuucaaaacuguuuuguucguucggcucgaguuaucaaguggucaaaaugacuu          exp
    .((((((...))))))....(((((.(.(((((((((((((...((((.((((((.............)))))).))))...)))))))))))))).))))).......... reads  mm  sample
    ...........ucuuuauaagacuagugugguа............................................................................... 2      0   MOL
    ............cuuuauaagacuagugugguа............................................................................... 9      0   MOL
    .............uuuauaagacuagugugguа............................................................................... 13     0   MOL
    ................................aacuugagcaguacgaucaga........................................................... 1      0   MOL
    ................................aacuugagcaguacgaucagaa.......................................................... 1      0   MOL
    ................................aacuugagcaguacgaucagaac......................................................... 1      0   MOL
    ................................aacuugagcaguacgaucagaacu........................................................ 1      0   MOL
    .................................acuugagcaguacgaucaga........................................................... 18     0   MOL
    .................................acuugagcaguacgaucagaa.......................................................... 40     0   MOL
    .................................acuugagcaguacgaGcagaa.......................................................... 1      1   MOL
    .................................acuugagcaguacgaAcagaa.......................................................... 1      1   MOL
    .................................acuugagcaguacgaucagGac......................................................... 1      1   MOL
    .................................acuugagcaguacgaucagaaA......................................................... 5      1   MOL
    .................................acuugagcaguacgaucagaac......................................................... 71     0   MOL
    .................................acuugagcUguacgaucagaac......................................................... 1      1   MOL
    .................................acuugagcaguacgaucagaacA........................................................ 9      1   MOL
    .................................acuugagcaguacgaucagaacu........................................................ 6      0   MOL
    .................................acuugagcaguacgaucagaacuA....................................................... 2      1   MOL
    ..................................cuugagcaguacgaucagaa.......................................................... 1      0   MOL
    ..................................cuugagcaguacgaucagaac......................................................... 4      0   MOL
    ..................................cuugagcaguacgaucagaaA......................................................... 2      1   MOL
    ..................................cuugagcaguacgaucagaacu........................................................ 2      0   MOL
    ...................................................................uguuuuguucguucggcucgagu...................... 1      0   MOL
    ....................................................................Uuuuuguucguucggcucgagu...................... 7      1   MOL
    ....................................................................guuuuguucguucggcucgagu...................... 1      0   MOL
    ....................................................................Uuuuuguucguucggcucgaguu..................... 8      1   MOL
    ....................................................................guuuuguucguucggcucgaguu..................... 1      0   MOL
    ....................................................................guuuuguucguucggcucgaguAa.................... 1      1   MOL
    ....................................................................Uuuuuguucguucggcucgaguua.................... 11     1   MOL
    ....................................................................guuuuguucguucggcucgaguuau................... 1      0   MOL
    ....................................................................guuuuguucguucggcucgaguuCu................... 1      1   MOL
    ....................................................................Uuuuuguucguucggcucgaguuau................... 1      1   MOL
    .....................................................................Guuuguucguucggcucg......................... 1      1   MOL
```

Star Mature

```
gaaggauacugucuuuauaagacuagugugguaacuugagcaguacgaucagaacugcuuucaaaacuguuuuguucguucggcucgaguuaucaaguggucaaaaugacuu
......................................................................uuuuguucguucggcucg.........................   10     0   MOL
......................................................................uuuuguucguucggcucga........................   38     0   MOL
......................................................................Guuuguucguucggcucga........................   1      1   MOL
......................................................................uuuuguucguucggcucgaA.......................   4      1   MOL
......................................................................uuuuguucguucggcucgaU.......................   1      1   MOL
......................................................................uuuuguucguucggcucgag.......................   86     0   MOL
......................................................................uuuuguucguucggcucgagG......................   1      1   MOL
......................................................................uuuuguucguucggcucgUgu......................   2      1   MOL
......................................................................uuuuguucguucggcucgaCu......................   1      1   MOL
......................................................................uuuuguucguucggcucgagu......................   1004   0   MOL
......................................................................uuuuguucguucggUucgagu......................   1      1   MOL
......................................................................Auuuguucguucggcucgagu......................   1      1   MOL
......................................................................uuuuguucguucggcucgagC......................   2      1   MOL
......................................................................uuAuguucguucggcucgagu......................   1      1   MOL
......................................................................uuuuguucguucggcucgaUu......................   5      1   MOL
......................................................................uuuugCucguucggcucgagu......................   2      1   MOL
......................................................................uuuuguucAuucggcucgagu......................   3      1   MOL
......................................................................uuuuguucgCucggcucgagu......................   2      1   MOL
......................................................................uuuuguuUguucggcucgagu......................   1      1   MOL
......................................................................Cuuuguucguucggcucgagu......................   1      1   MOL
......................................................................uuuuguucguucggcucgagA......................   53     1   MOL
......................................................................uuuuguucguucgAcucgagu......................   1      1   MOL
......................................................................uuuuguucguuUggcucgaguu.....................   1      1   MOL
......................................................................uuuuguucguucggcucgaguu.....................   1440   0   MOL
......................................................................uuuuguucguucgAcucgaguu.....................   3      1   MOL
......................................................................uuuuguucAuucggcucgaguu.....................   2      1   MOL
......................................................................uuuuguucguucggcAcgaguu.....................   2      1   MOL
......................................................................uuuuguucguucggcuUgaguu.....................   1      1   MOL
......................................................................uuuuguucgCucggcucgaguu.....................   1      1   MOL
......................................................................uuuuguucguucggcucgagGu.....................   1      1   MOL
......................................................................uuuugGucguucggcucgaguu.....................   1      1   MOL
......................................................................Cuuuguucguucggcucgaguu.....................   1      1   MOL
......................................................................uuuuguucguucggcucgaguG.....................   1      1   MOL
......................................................................Auuuguucguucggcucgaguu.....................   3      1   MOL
......................................................................uuuuguucguucggcucgaguC.....................   1      1   MOL
......................................................................uuuuguucguucggcucgaAuu.....................   1      1   MOL
......................................................................uuuuguucguucggcucgaguA.....................   50     1   MOL
......................................................................Guuuguucguucggcucgaguu.....................   2      1   MOL
......................................................................uuuuguucguucggcucgUguu.....................   4      1   MOL
......................................................................uuuuguucguuUggcucgaguua....................   2      1   MOL
......................................................................uuuuguuUguucggcucgaguua....................   1      1   MOL
......................................................................uuuuguucguucggcucgaguAa....................   81     1   MOL
......................................................................Auuuguucguucggcucgaguua....................   5      1   MOL
......................................................................Guuuguucguucggcucgaguua....................   2      1   MOL
......................................................................uuuuguucguucggcucgaguua....................   1344   0   MOL
......................................................................uuuugCucguucggcucgaguua....................   2      1   MOL
......................................................................uuuuguucguucggcucgCguua....................   1      1   MOL
......................................................................uuuuguucguucggcucgaguuC....................   38     1   MOL
......................................................................uuuuguucguucggcucgaUuua....................   1      1   MOL
......................................................................uuuuguucguucggcucgaguuU....................   888    1   MOL
......................................................................uuuuguucguucggcuUgaguua....................   1      1   MOL
......................................................................uuuuguucguucggcucgaguGa....................   2      1   MOL
......................................................................uuuuguucguucgAcucgaguua....................   2      1   MOL
......................................................................uuuuguucguucggcucgaguCa....................   1      1   MOL
......................................................................uuuuguucguucggcucgagAua....................   1      1   MOL
......................................................................uuuuguucguucggcucgaguuG....................   7      1   MOL
......................................................................uuuuguucguucggcucgUguua....................   3      1   MOL
......................................................................uuuuguucguucggcucgUguuau...................   3      1   MOL
......................................................................uuuuguucguucggcucgaguuaC...................   6      1   MOL
......................................................................uuuuguucguucggcAcgaguuau...................   1      1   MOL
......................................................................uuuuguucguucggcucgaguuaG...................   4      1   MOL
......................................................................uuuuguucguucggcucgaguuCu...................   3      1   MOL
......................................................................uuuuguucguucggcucgaguuau...................   242    0   MOL
......................................................................uuuuguucguucggcucgaguAau...................   4      1   MOL
......................................................................uuuuguucguucggcucgaguuUu...................   61     1   MOL
......................................................................uuuuCuucguucggcucgaguuau...................   1      1   MOL
......................................................................Auuuguucguucggcucgaguuau...................   1      1   MOL
......................................................................uuuuguucguucgCcucgaguuau...................   1      1   MOL
......................................................................Guuuguucguucggcucgaguuau...................   1      1   MOL
......................................................................uuuuguucguucggcucgaguuaA...................   194    1   MOL
```

Star **Mature**

```
gaaggauacugucuuuauaagacuaguguggua acuugagcaguacgaucagaac ugcuuucaaaacugu uuuguucguucggcucgaguua ucaaguggucaaaaugacuu
.....................................................................uuuguucguucggcucgaguuauc...................   1      0   MOL
.....................................................................uuuguucguucggcucgaguuauA...................   8      1   MOL
.....................................................................uuuguucguucggcucgaguuauU...................   12     1   MOL
.....................................................................uuuguucguucggcucgaguuaucU..................   1      1   MOL
......................................................................uuuguucguucggcucga........................   72     0   MOL
......................................................................uuuguucguucggcucgaU.......................   2      1   MOL
......................................................................uuuguucguucggcucgaA.......................   9      1   MOL
......................................................................uuuguucguucggcucgag.......................   225    0   MOL
......................................................................uuuguucguucggcucgUg.......................   1      1   MOL
......................................................................uGuguucguucggcucgagu......................   1      1   MOL
......................................................................Cuuguucguucggcucgagu......................   6      1   MOL
......................................................................uuuguucguAcggcucgagu......................   1      1   MOL
......................................................................uuuguucguucggcucgaUu......................   16     1   MOL
......................................................................uuuguucguucggcAcgagu......................   2      1   MOL
......................................................................Guuguucguucggcucgagu......................   4      1   MOL
......................................................................uuuguucguucggUucgagu......................   12     1   MOL
......................................................................uuuguucgCucggcucgagu......................   3      1   MOL
......................................................................uuuguucguucggcuUgagu......................   3      1   MOL
......................................................................uuuUuucguucggcucgagu......................   2      1   MOL
......................................................................uuuguucguCcggcucgagu......................   1      1   MOL
......................................................................uuuguucguucggcucgGgu......................   2      1   MOL
......................................................................uuuguucguucggcucgaAu......................   14     1   MOL
......................................................................uuuguucguucggcucgagu......................   8377   0   MOL
......................................................................uuuguucguucggcucgaCu......................   1      1   MOL
......................................................................uuuguucguucggcucgagG......................   3      1   MOL
......................................................................uuuguucguucggcucAagu......................   1      1   MOL
......................................................................uuuguucguucgUcucgagu......................   1      1   MOL
......................................................................uuuguucguucggcucgagC......................   9      1   MOL
......................................................................uCuguucguucggcucgagu......................   1      1   MOL
......................................................................uuuguucguucgAcucgagu......................   7      1   MOL
......................................................................uuuguucguucggcuGgagu......................   1      1   MOL
......................................................................uuuguucguucggcucgagA......................   268    1   MOL
......................................................................uuuguucguucggcCcgagu......................   1      1   MOL
......................................................................uuuguucguucggGucgagu......................   1      1   MOL
......................................................................uuuguucgAucggcucgagu......................   9      1   MOL
......................................................................uuuguucguucAgcucgagu......................   1      1   MOL
......................................................................uuuguucguucggcucgUgu......................   10     1   MOL
......................................................................uuuguucguucggcucgCgu......................   8      1   MOL
......................................................................uuuguucguucUgcucgagu......................   2      1   MOL
......................................................................Auuguucguucggcucgagu......................   19     1   MOL
......................................................................uuuguCcguucggcucgagu......................   2      1   MOL
......................................................................uuuguucguuUggcucgagu......................   5      1   MOL
......................................................................uuuguucguucggAucgagu......................   2      1   MOL
......................................................................uuuguucgCucggcucgaguu.....................   3      1   MOL
......................................................................uuuguucgAucggcucgaguu.....................   26     1   MOL
......................................................................uuuguucguucggcucgGguu.....................   16     1   MOL
......................................................................Cuuguucguucggcucgaguu.....................   14     1   MOL
......................................................................uuuguucguucgCcucgaguu.....................   2      1   MOL
......................................................................uuuguucAuucggcucgaguu.....................   1      1   MOL
......................................................................uuuguucguCcggcucgaguu.....................   8      1   MOL
......................................................................uuuguucguucggcGcgaguu.....................   3      1   MOL
......................................................................uGuguucguucggcucgaguu.....................   2      1   MOL
......................................................................uuuguucguucggcCcgaguu.....................   6      1   MOL
......................................................................uuuguucguucggAucgaguu.....................   4      1   MOL
......................................................................uuuguucguucggcucgaUuu.....................   6      1   MOL
......................................................................uuGguucguucggcucgaguu.....................   1      1   MOL
......................................................................uuuguuGguucggcucgaguu.....................   2      1   MOL
......................................................................uAuguucguucggcucgaguu.....................   1      1   MOL
......................................................................uuuguucguucggcucUaguu.....................   3      1   MOL
......................................................................uuuguucguucggcucgaguA.....................   430    1   MOL
......................................................................uuuguucguucggcuGgaguu.....................   3      1   MOL
......................................................................uuuguuAguucggcucgaguu.....................   1      1   MOL
......................................................................uuuguucguucggcucgaAuu.....................   1      1   MOL
......................................................................uuugCucguucggcucgaguu.....................   3      1   MOL
......................................................................uuuguucguucgUcucgaguu.....................   4      1   MOL
......................................................................Guuguucguucggcucgaguu.....................   18     1   MOL
......................................................................uuuguCcguucggcucgaguu.....................   7      1   MOL
......................................................................uuuguucguucggcucgaguC.....................   6      1   MOL
......................................................................uuuguucguAcggcucgaguu.....................   2      1   MOL
......................................................................uuuguucguucggcucgaCuu.....................   4      1   MOL
```

Star **Mature**

```
gaaggauacugucuuuauaagacuaguguggua acuugagcaguacgaucagaac ugcuuucaaaacugu uuuguucguucggcucgaguua ucaaguggucaaaaugacuu
......................................................................uuuguuUguucggcucgaguu.....................  4      1  MOL
......................................................................uuuguucguuUggcucgaguu.....................  6      1  MOL
......................................................................uuuguucguucggcAcgaguu.....................  6      1  MOL
......................................................................uuuguucguucCgcucgaguu.....................  2      1  MOL
......................................................................uuuguucguucggcucgUguu.....................  24     1  MOL
......................................................................uuuguucguucggcucgaguu.....................  22430  0  MOL
......................................................................uuuguucguucggcuUgaguu.....................  16     1  MOL
......................................................................uuuguGcguucggcucgaguu.....................  4      1  MOL
......................................................................uuuAuucguucggcucgaguu.....................  2      1  MOL
......................................................................uuuguAcguucggcucgaguu.....................  5      1  MOL
......................................................................uuuguucguucggcucAaguu.....................  2      1  MOL
......................................................................uuuguucUuucggcucgaguu.....................  2      1  MOL
......................................................................uuuguucCuucggcucgaguu.....................  1      1  MOL
......................................................................uuuguucguucggGucgaguu.....................  2      1  MOL
......................................................................uuuguucguucUgcucgaguu.....................  6      1  MOL
......................................................................uCuguucguucggcucgaguu.....................  5      1  MOL
......................................................................uuuguucguucggcucgagGu.....................  1      1  MOL
......................................................................uuuguucguucggcucgaguG.....................  9      1  MOL
......................................................................uuuguucguucggcucgagCu.....................  3      1  MOL
......................................................................uuuguucguucggcucgCguu.....................  109    1  MOL
......................................................................uuuguucguucggUucgaguu.....................  19     1  MOL
......................................................................Auuguucguucggcucgaguu.....................  57     1  MOL
......................................................................uuAguucguucggcucgaguu.....................  4      1  MOL
......................................................................uuuguucgGucggcucgaguu.....................  4      1  MOL
......................................................................uuuguucguucggcucgagAu.....................  17     1  MOL
......................................................................uuuguucguucAgcucgaguu.....................  1      1  MOL
......................................................................uuuUuucguucggcucgaguu.....................  3      1  MOL
......................................................................uuuguucguucgAcucgaguu.....................  23     1  MOL
......................................................................uuuguucguucggcuAgaguu.....................  1      1  MOL
......................................................................uuuguucguuAggcucgaguu.....................  1      1  MOL
......................................................................uuuguucguucgCcucgaguua....................  2      1  MOL
......................................................................uuuguucguucggcucgaguCa....................  11     1  MOL
......................................................................uuuguucguucggcCcgaguua....................  3      1  MOL
......................................................................uuuguucguucggcuUgaguua....................  24     1  MOL
......................................................................uuuguucguuUggcucgaguua....................  29     1  MOL
......................................................................uuuguucguucggUucgaguua....................  15     1  MOL
......................................................................uuuguucAuucggcucgaguua....................  5      1  MOL
......................................................................uuuguucguucggcucgaguuG....................  237    1  MOL
......................................................................uuuguucguucggcucgagCua....................  2      1  MOL
......................................................................uuAguucguucggcucgaguua....................  11     1  MOL
......................................................................uuuguucguucggcucgGguua....................  22     1  MOL
......................................................................uuuguucgGucggcucgaguua....................  2      1  MOL
......................................................................uuuguucguuAggcucgaguua....................  1      1  MOL
......................................................................uuuguuAguucggcucgaguua....................  2      1  MOL
......................................................................uuuUuucguucggcucgaguua....................  3      1  MOL
......................................................................uuuguucguucggcGcgaguua....................  1      1  MOL
......................................................................uuuguucguucggcucgaguAa....................  1502   1  MOL
......................................................................Cuuguucguucggcucgaguua....................  38     1  MOL
......................................................................uuuguuUguucggcucgaguua....................  19     1  MOL
......................................................................uuuguucguucgAcucgaguua....................  68     1  MOL
......................................................................uuuguucguucggcucgaguuC....................  1364   1  MOL
......................................................................uuuguucguucggcucgagAua....................  18     1  MOL
......................................................................uuugAucguucggcucgaguua....................  4      1  MOL
......................................................................uuuguucUuucggcucgaguua....................  9      1  MOL
......................................................................uuuguucgAucggcucgaguua....................  46     1  MOL
......................................................................uuuguCcguucggcucgaguua....................  12     1  MOL
......................................................................uuuguucguucggcuAgaguua....................  5      1  MOL
......................................................................uuuguucguCcggcucgaguua....................  15     1  MOL
......................................................................uuuguucguucCgcucgaguua....................  1      1  MOL
......................................................................uuCguucguucggcucgaguua....................  5      1  MOL
......................................................................uuuguucguucggcucgUguua....................  37     1  MOL
......................................................................uuuguucguuGggcucgaguua....................  1      1  MOL
......................................................................uuuguucguucggcucgaCuua....................  14     1  MOL
......................................................................Guuguucguucggcucgaguua....................  41     1  MOL
......................................................................uuuguucguucggcucgCguua....................  260    1  MOL
......................................................................uGuguucguucggcucgaguua....................  2      1  MOL
......................................................................uuuguAcguucggcucgaguua....................  11     1  MOL
......................................................................uuuguucguucggcucgaguua....................  56492  0  MOL
......................................................................uuuguucguucUgcucgaguua....................  6      1  MOL
......................................................................uuuguucguucggcucgaUuua....................  23     1  MOL
```

```
                                  Star                                     Mature
gaaggauacugucuuuauaagacuaguguggua acuugagcaguacgaucagaac ugcuuucaaaacugu uuuguucguucggcucgaguua ucaaguggucaaaaugacuu
......................................................................Auuguucguucggcucgaguua....................  123    1  MOL
......................................................................uCuguucguucggcucgaguua....................  9      1  MOL
......................................................................uuuAuucguucggcucgaguua....................  2      1  MOL
......................................................................uuuguucguucggcucCaguua....................  9      1  MOL
......................................................................uuuguucguAcggcucgaguua....................  5      1  MOL
......................................................................uuuguucguucggcucUaguua....................  6      1  MOL
......................................................................uuuguucguucggcucgaguGa....................  15     1  MOL
......................................................................uuuguucguucggcucgaAuua....................  9      1  MOL
......................................................................uuuguucguucAgcucgaguua....................  6      1  MOL
......................................................................uuuCuucguucggcucgaguua....................  3      1  MOL
......................................................................uuuguGcguucggcucgaguua....................  8      1  MOL
......................................................................uuuguucguucggGucgaguua....................  4      1  MOL
......................................................................uuuguucguucggcucgaguuU....................  16401  1  MOL
......................................................................uuuguucguucggcucAaguua....................  7      1  MOL
......................................................................uuuguucguucggcuGgaguua....................  5      1  MOL
......................................................................uuuguucgCucggcucgaguua....................  8      1  MOL
......................................................................uuGguucguucggcucgaguua....................  3      1  MOL
......................................................................uuuguucguucgUcucgaguua....................  17     1  MOL
......................................................................uuuguucguucggAucgaguua....................  5      1  MOL
......................................................................uuuguuGguucggcucgaguua....................  5      1  MOL
......................................................................uuuguucguucggcucgagGua....................  4      1  MOL
......................................................................uuugCucguucggcucgaguua....................  3      1  MOL
......................................................................uuuguucguucggcAcgaguua....................  12     1  MOL
......................................................................uuuguucguucggcucgaguGau...................  1      1  MOL
......................................................................uuuguucUuucggcucgaguuau...................  1      1  MOL
......................................................................uuuguucgGucggcucgaguuau...................  1      1  MOL
......................................................................uuuguucguucggcAcgaguuau...................  4      1  MOL
......................................................................uuuguucguuUggcucgaguuau...................  5      1  MOL
......................................................................uuuguucguucggcuUgaguuau...................  7      1  MOL
......................................................................Auuguucguucggcucgaguuau...................  11     1  MOL
......................................................................uuuguucguucggcucgGguuau...................  5      1  MOL
......................................................................uuuguucguucggUucgaguuau...................  4      1  MOL
......................................................................uuuguucguucggcuGgaguuau...................  1      1  MOL
......................................................................uuAguucguucggcucgaguuau...................  2      1  MOL
......................................................................uuuguucguCcggcucgaguuau...................  5      1  MOL
......................................................................uuuguucguucggAucgaguuau...................  1      1  MOL
......................................................................uuuguucguucgAcucgaguuau...................  8      1  MOL
......................................................................uuugCucguucggcucgaguuau...................  1      1  MOL
......................................................................uuuguucguucggcucgaguuGu...................  14     1  MOL
......................................................................uuugAucguucggcucgaguuau...................  1      1  MOL
......................................................................uGuguucguucggcucgaguuau...................  1      1  MOL
......................................................................uuuguucguucggcucgaguuau...................  6897   0  MOL
......................................................................uuuguucguucggcucgaguuaA...................  10934  1  MOL
......................................................................uuuguucguucggcucgaguCau...................  4      1  MOL
......................................................................uuuguucguucggcucgUguuau...................  4      1  MOL
......................................................................uuuguucgCucggcucgaguuau...................  2      1  MOL
......................................................................uuuguucguucggcucgaguuaC...................  533    1  MOL
......................................................................Cuuguucguucggcucgaguuau...................  5      1  MOL
......................................................................uuuguucguucggcucAaguuau...................  1      1  MOL
......................................................................uuuguucguucggcucgaguAau...................  34     1  MOL
......................................................................Guuguucguucggcucgaguuau...................  9      1  MOL
......................................................................uuuguCcguucggcucgaguuau...................  3      1  MOL
......................................................................uuuCuucguucggcucgaguuau...................  1      1  MOL
......................................................................uuGguucguucggcucgaguuau...................  1      1  MOL
......................................................................uuuguucguucggcucgaCuuau...................  2      1  MOL
......................................................................uuuguucguucggcucgaAuuau...................  1      1  MOL
......................................................................uuuguucguucggcucgCguuau...................  33     1  MOL
......................................................................uuuguucguucAgcucgaguuau...................  2      1  MOL
......................................................................uCuguucguucggcucgaguuau...................  1      1  MOL
......................................................................uuuguucgAucggcucgaguuau...................  6      1  MOL
......................................................................uuuguucguucggcucgaUuuau...................  2      1  MOL
......................................................................uuuguucguucggcucgaguuUu...................  879    1  MOL
......................................................................uuuguucguucgUcucgaguuau...................  3      1  MOL
......................................................................uuuguucguucggcucgaguuaG...................  285    1  MOL
......................................................................uuuguucguucggcucCaguuau...................  1      1  MOL
......................................................................uuuguucguucggcucgagCuau...................  3      1  MOL
......................................................................uuuguucguucggcucgaguuCu...................  185    1  MOL
......................................................................uuuguucguucggcucgaguuauG..................  5      1  MOL
......................................................................uuuguucguucggcucgaguuaAc..................  30     1  MOL
......................................................................uuuguucguucggcucgaguuauA..................  66     1  MOL
```

Star **Mature**

```
gaaggauacugucuuuauaagacuaguguggua acuugagcaguacgaucagaac ugcuuucaaaacugu uuuguucguucggcucgaguua ucaaguggucaaaaugacuu
......................................................................uuuguucguucggcucgaguuauc..................	12	0	MOL
......................................................................uuuguucguucggcucgaguuUuc..................	1	1	MOL
......................................................................uuuguucguucggcucgaguuaCc..................	2	1	MOL
......................................................................uuuguucguucggcucgaguuauU..................	232	1	MOL
......................................................................uuuguucguucggcucgaguuaAca.................	3	1	MOL
......................................................................uuuguucguucggcucgaguuauUa.................	3	1	MOL
......................................................................uuuguucguucggcucgaguuauAa.................	2	1	MOL
......................................................................uuuguucguucggcucgaguuaucU.................	1	1	MOL
.......................................................................uuguucguucggcucgaU.......................	1	1	MOL
.......................................................................uuguucguucggcucgagu......................	38	0	MOL
.......................................................................uuguucguucggcucgagA......................	1	1	MOL
.......................................................................uuguucguucggcucgUgu......................	1	1	MOL
.......................................................................uuguucguucggcucgaguu.....................	148	0	MOL
.......................................................................Guguucguucggcucgaguu.....................	1	1	MOL
.......................................................................Auguucguucggcucgaguu.....................	1	1	MOL
.......................................................................uuCuucguucggcucgaguu.....................	1	1	MOL
.......................................................................uuUuucguucggcucgaguu.....................	1	1	MOL
.......................................................................Cuguucguucggcucgaguua....................	2	1	MOL
.......................................................................Auguucguucggcucgaguua....................	3	1	MOL
.......................................................................Guguucguucggcucgaguua....................	1	1	MOL
.......................................................................uuguucguucggcucgaCuua....................	1	1	MOL
.......................................................................uuguucguAcggcucgaguua....................	1	1	MOL
.......................................................................uugAucguucggcucgaguua....................	1	1	MOL
.......................................................................uuguucguuUggcucgaguua....................	1	1	MOL
.......................................................................uuguucguucggcucgaguuC....................	25	1	MOL
.......................................................................uuguucguucggcucgaguAa....................	4	1	MOL
.......................................................................uuguucguucggcAcgaguua....................	1	1	MOL
.......................................................................uuguucguucggcucgGguua....................	1	1	MOL
.......................................................................uuguucguucggcucgCguua....................	5	1	MOL
.......................................................................uuUuucguucggcucgaguua....................	2	1	MOL
.......................................................................uuguucguucggcucgaguuU....................	103	1	MOL
.......................................................................uuguucguucggcucgaguuG....................	3	1	MOL
.......................................................................uuguucguucggcucgaguua....................	619	0	MOL
.......................................................................uuUuucguucggcucgaguuau...................	1	1	MOL
.......................................................................uuguucguucggcucgaguuau...................	49	0	MOL
.......................................................................uuguucguucggcucgaguuUu...................	4	1	MOL
.......................................................................uuguucguucggcucgaguuaA...................	64	1	MOL
.......................................................................uuguucguucggcucgaguuCu...................	2	1	MOL
.......................................................................uuguucguucggcucgCguuau...................	4	1	MOL
.......................................................................uuguucguucggcucgaguuaC...................	4	1	MOL
.......................................................................uuguucguucggcucgaguuauU..................	2	1	MOL
.......................................................................uuguucguucggcucgaguuauA..................	1	1	MOL
........................................................................uguucguucggcucgagu......................	4	0	MOL
........................................................................uUuucguucggcucgagu......................	2	1	MOL
........................................................................uUuucguucggcucgaguu.....................	1	1	MOL
........................................................................uguucguucggcucgCguu.....................	1	1	MOL
........................................................................uguucguucggcucgaguu.....................	5	0	MOL
........................................................................uguucguucggcucgaguuG....................	1	1	MOL
........................................................................uguucguucggcucgaguuU....................	8	1	MOL
........................................................................uUuucguucggcucgaguua....................	2	1	MOL
........................................................................uguucguucggcucgaguua....................	26	0	MOL
........................................................................uguucguucggcucgaguuC....................	2	1	MOL
........................................................................uguucguucggcucgaguuaA...................	1	1	MOL
........................................................................uguucguucggcucgaguuau...................	3	0	MOL
........................................................................uguucguucggcucgaguuaC...................	1	1	MOL
.........................................................................guucguucggcucgaguu.....................	3	0	MOL
.........................................................................guucguucggcucgaguua....................	3	0	MOL
.........................................................................Uuucguucggcucgaguua....................	1	1	MOL
.........................................................................guucguucggcucgaguuU....................	1	1	MOL
.........................................................................Cuucguucggcucgaguuau...................	1	1	MOL
.........................................................................guucguucggcucgaguuaA...................	3	1	MOL
.........................................................................Uuucguucggcucgaguuau...................	1	1	MOL
.........................................................................guucguucggcucgaguuau...................	1	0	MOL
..........................................................................uuUguucggcucgaguua....................	1	1	MOL

............cuuuauaagacuaguguggua...............................................................................	14	0	T63
.............Auuauaagacuaguguggua...............................................................................	1	1	T63
.............uuuauaagacuaguguggua...............................................................................	19	0	T63
..............uuauaagacuaguguggua...............................................................................	4	0	T63
................................aacuugagcaguacgaucaga...........................................................	1	0	T63
```

Star Mature

```
gaaggauacugucuuuauaagacuaguguggua acuugagcaguacgaucagaac ugcuuucaaaacugu uuuguucguucggcucgaguua ucaaguggucaaaaugacuu
.................................acuugagcaguacgaucag............................................................ 1 0 T63
.................................acuugagcaguacgaucaga........................................................... 11 0 T63
.................................acuugagcaguacgaucagaa.......................................................... 7 0 T63
.................................acuugagcaguacgaucagaaA......................................................... 2 1 T63
.................................acuugagcaguacgaucagaac......................................................... 31 0 T63
.................................acuugagcaguacgaucagaacA........................................................ 2 1 T63
.................................acuugagcaguacgaucagaacC........................................................ 3 1 T63
..................................cuugagcaguacgaucagaaA......................................................... 1 1 T63
..................................cuugagcaguacgaucagaac......................................................... 2 0 T63
..................................cuugagcaguacgaucagaacu........................................................ 1 0 T63
..................................cuugagcaguacgaucagaacuU....................................................... 2 1 T63
...........................................................uucaaaacuguuuuguucguucgg............................. 1 0 T63
....................................................................Auuuuguucguucggcucgagu...................... 1 1 T63
....................................................................Uuuuuguucguucggcucgagu...................... 6 1 T63
....................................................................Uuuuuguucguucggcucgaguu..................... 7 1 T63
....................................................................Uuuuuguucguucggcucgaguua.................... 3 1 T63
.....................................................................uuuguucguucggcucg.......................... 43 0 T63
.....................................................................uuuguucguucggcucga......................... 68 0 T63
.....................................................................uuuguucguucggcAcga......................... 1 1 T63
.....................................................................uuuguucguucggcucgag........................ 45 0 T63
.....................................................................uuuguucguucgAcucgag........................ 1 1 T63
.....................................................................Cuuguucguucggcucgagu....................... 1 1 T63
.....................................................................uuuguucAuucggcucgagu....................... 1 1 T63
.....................................................................uuugCucguucggcucgagu....................... 1 1 T63
.....................................................................uuuguucguucggUucgagu....................... 3 1 T63
.....................................................................uuuUuucguucggcucgagu....................... 1 1 T63
.....................................................................uuuguucguucggcucgaUu....................... 3 1 T63
.....................................................................uuuguucguucggcucgagG....................... 18 1 T63
.....................................................................uuAguucguucggcucgagu....................... 3 1 T63
.....................................................................uuuguucguucggcuGgagu....................... 1 1 T63
.....................................................................uuuguucguucggcucgagu....................... 2804 0 T63
.....................................................................Guuguucguucggcucgagu....................... 1 1 T63
.....................................................................uuuguucgAucggcucgagu....................... 1 1 T63
.....................................................................uuuguuUguucggcucgagu....................... 1 1 T63
.....................................................................uuuguucUuucggcucgagu....................... 1 1 T63
.....................................................................uuuguucguucggcucgUgu....................... 3 1 T63
.....................................................................uAuguucguucggcucgagu....................... 1 1 T63
.....................................................................uuuguucguucggcucgGgu....................... 1 1 T63
.....................................................................uuuguucguucgAcucgagu....................... 2 1 T63
.....................................................................uuuguAcguucggcucgagu....................... 3 1 T63
.....................................................................uuuguucguucggcucAagu....................... 1 1 T63
.....................................................................Auuguucguucggcucgagu....................... 10 1 T63
.....................................................................uuAguucguucggcucgagu....................... 3 1 T63
.....................................................................uuugAucguucggcucgagu....................... 1 1 T63
.....................................................................uuuguucguUggcucgagu........................ 3 1 T63
.....................................................................uuuguucguucggcAcgagu....................... 2 1 T63
.....................................................................uuuguuGguucggcucgagu....................... 1 1 T63
.....................................................................uuuguucguucggcucgagA....................... 71 1 T63
.....................................................................Auuguucguucggcucgaguu...................... 3 1 T63
.....................................................................uuuguucguucggcucUaguu...................... 1 1 T63
.....................................................................uuuguucguucggcucgaguu...................... 1652 0 T63
.....................................................................uuuguucguucggcAcgaguu...................... 1 1 T63
.....................................................................uuAguucguucggcucgaguu...................... 1 1 T63
.....................................................................uuuguucguucUgcucgaguu...................... 1 1 T63
.....................................................................uuuguucguuUggcucgaguu...................... 1 1 T63
.....................................................................uuuguucguucggcucgaguA...................... 34 1 T63
.....................................................................uuuUuucguucggcucgaguu...................... 1 1 T63
.....................................................................uuuguucguucggcucgGguu...................... 2 1 T63
.....................................................................uuuguucguucggcucgaguC...................... 1 1 T63
.....................................................................uuuguucguCcggcucgaguu...................... 1 1 T63
.....................................................................uuuguucguucggcucgagCu...................... 1 1 T63
.....................................................................uuuguucAuucggcucgaguu...................... 1 1 T63
.....................................................................uuuguucguucgAcucgaguu...................... 1 1 T63
.....................................................................Cuuguucguucggcucgaguu...................... 2 1 T63
.....................................................................uuuguucguucggcucgaAuu...................... 1 1 T63
.....................................................................uuugGucguucggcucgaguu...................... 1 1 T63
.....................................................................uuuguucguucggcucgUguu...................... 4 1 T63
.....................................................................uuuguucguucggcCcgaguu...................... 2 1 T63
.....................................................................uuuguucguucggcucgaguua..................... 359 0 T63
.....................................................................uuuguucguucggcucgaguuG..................... 26 1 T63
```

```
                                 Star                               Mature
gaaggauacugucuuuauaagacuaguguggua acuugagcaguacgaucagaac ugcuuucaaaacugu uuuguucguucggcucgaguua ucaaguggucaaaaugacuu
.....................................................................uuuuguucguucggcucgaguCa....................    1       1   T63
.....................................................................uuuuguucguucgAcucgaguua....................    2       1   T63
.....................................................................uuuuguucAuucggcucgaguua....................    1       1   T63
.....................................................................uuuuguucguucggcucgaguuC....................    60      1   T63
.....................................................................uuuuguucguucggcucgaguuU....................    1121    1   T63
.....................................................................Auuuguucguucggcucgaguua....................    2       1   T63
.....................................................................uuuuguucguucggAucgaguua....................    1       1   T63
.....................................................................uuuuAuucguucggcucgaguua....................    1       1   T63
.....................................................................uuuuguucguucggcucgaguAa....................    41      1   T63
.....................................................................uuuuguucguucggcucgaguuCu...................    6       1   T63
.....................................................................uuuuguucguucggcucgaguuaG...................    4       1   T63
.....................................................................Auuuguucguucggcucgaguuau...................    1       1   T63
.....................................................................uuuuguucguucggcucgaguuaC...................    2       1   T63
.....................................................................uuuuguucguucggcucgaguuau...................    201     0   T63
.....................................................................Cuuuguucguucggcucgaguuau...................    1       1   T63
.....................................................................uuuuguucguucggcucgaguuUu...................    84      1   T63
.....................................................................uuuuguucgCucggcucgaguuau...................    1       1   T63
.....................................................................uuuuguucguucggcucgaguAau...................    2       1   T63
.....................................................................uuuuguucguucggcucgaguuaA...................    78      1   T63
.....................................................................uuuuguucguucggcucgaguuauU..................    28      1   T63
.....................................................................uuuuguucguucggcucgaguuauA..................    1       1   T63
.....................................................................uuuuguucguucggcucgaguuaucU.................    1       1   T63
......................................................................uuuguucguucggcucgC........................    1       1   T63
......................................................................uuuguucguucggcucga........................    342     0   T63
......................................................................uuuguucguucggcucAa........................    1       1   T63
......................................................................uuuguucgAucggcucga........................    2       1   T63
......................................................................uuuguucguucggcucgU........................    2       1   T63
......................................................................uuuguucguucgAcucga........................    1       1   T63
......................................................................uuuguucgCucggcucgag.......................    1       1   T63
......................................................................uuuguucguucggcucgUg.......................    1       1   T63
......................................................................uuuguucguucAgcucgag.......................    1       1   T63
......................................................................uuuguucguucggcucgCg.......................    2       1   T63
......................................................................uuuguucguucggcucgaA.......................    27      1   T63
......................................................................uuuguucguucggcucgaC.......................    2       1   T63
......................................................................uuuguucguucggcucgaU.......................    14      1   T63
......................................................................uuuguucguucggcucgag.......................    333     0   T63
......................................................................Auuguucguucggcucgag.......................    1       1   T63
......................................................................uuuguucguucgAcucgag.......................    1       1   T63
......................................................................uuuguucguucggcucgaAu......................    22      1   T63
......................................................................uuuguucguucggcucgagu......................    25868   0   T63
......................................................................uuugAucguucggcucgagu......................    1       1   T63
......................................................................uuuguAcguucggcucgagu......................    4       1   T63
......................................................................uuuguucguucggcAcgagu......................    4       1   T63
......................................................................uuugCucguucggcucgagu......................    5       1   T63
......................................................................uuuUuucguucggcucgagu......................    3       1   T63
......................................................................uuuguucgAucggcucgagu......................    19      1   T63
......................................................................uuuguucguucggcucgCgu......................    11      1   T63
......................................................................uuuguucguucggcucgGgu......................    12      1   T63
......................................................................uuuguuAguucggcucgagu......................    1       1   T63
......................................................................uuuguCcguucggcucgagu......................    2       1   T63
......................................................................uuuguucguucggcucgaUu......................    36      1   T63
......................................................................uuuguucguucggcucgagG......................    124     1   T63
......................................................................uuuguucAuucggcucgagu......................    4       1   T63
......................................................................uuuguucguucAgcucgagu......................    4       1   T63
......................................................................uuuguuUguucggcucgagu......................    8       1   T63
......................................................................Cuuguucguucggcucgagu......................    13      1   T63
......................................................................uuuguucguucggcucgagA......................    486     1   T63
......................................................................uuuguucguucggcucCagu......................    4       1   T63
......................................................................uuuguucguuUggcucgagu......................    16      1   T63
......................................................................uCuguucguucggcucgagu......................    2       1   T63
......................................................................uuuguucguucgUcucgagu......................    4       1   T63
......................................................................uuuguucguCcggcucgagu......................    7       1   T63
......................................................................uuuguucguucCgcucgagu......................    1       1   T63
......................................................................uuuguucguucggcucUagu......................    1       1   T63
......................................................................uuuguucguucggcucgagC......................    24      1   T63
......................................................................uuuguucguucUgcucgagu......................    6       1   T63
......................................................................uuuguucguuAggcucgagu......................    1       1   T63
......................................................................Auuguucguucggcucgagu......................    33      1   T63
......................................................................uuuguucguucggcCcgagu......................    2       1   T63
......................................................................uuuguucgGucggcucgagu......................    2       1   T63
```

```
                                 Star                                 Mature
gaaggauacugucuuuauaagacuagugugguaacuugagcaguacgaucagaacugcuuucaaaacuguuuuguucguucggcucgaguuaucaaguggucaaaaugacuu
......................................................................uuuguuGguucggcucgagu......................   1       1   T63
......................................................................uuuguucguucggcuUgagu......................   13      1   T63
......................................................................uuuguucguucggcucAagu......................   5       1   T63
......................................................................uuuguucguucggUucgagu......................   19      1   T63
......................................................................uuCguucguucggcucgagu......................   3       1   T63
......................................................................uuuguucguucggcucgaCu......................   9       1   T63
......................................................................uuuguucguucgAcucgagu......................   21      1   T63
......................................................................uuuguucguucggcucgUgu......................   23      1   T63
......................................................................uuuAuucguucggcucgagu......................   2       1   T63
......................................................................uuuCuucguucggcucgagu......................   1       1   T63
......................................................................uuuguucgCucggcucgagu......................   4       1   T63
......................................................................uuuguGcguucggcucgagu......................   4       1   T63
......................................................................Guuguucguucggcucgagu......................   11      1   T63
......................................................................uuuguucguucgCcucgagu......................   4       1   T63
......................................................................uuuguucguuGggcucgagu......................   1       1   T63
......................................................................uuuguucguucggAucgagu......................   1       1   T63
......................................................................uuuguucguucggcucCaguu.....................   6       1   T63
......................................................................uuuguCcguucggcucgaguu.....................   7       1   T63
......................................................................uuuguucguAcggcucgaguu.....................   3       1   T63
......................................................................uuuguucguucggcucgaguG.....................   12      1   T63
......................................................................Cuuguucguucggcucgaguu.....................   25      1   T63
......................................................................uuuguucguucggcucgagAu.....................   18      1   T63
......................................................................uGuguucguucggcucgaguu.....................   3       1   T63
......................................................................uuuUuucguucggcucgaguu.....................   8       1   T63
......................................................................uuuguucguucggcucgaguu.....................   31516   0   T63
......................................................................uuuguucguGcggcucgaguu.....................   1       1   T63
......................................................................uuuCuucguucggcucgaguu.....................   3       1   T63
......................................................................uuuguucguucggcucgaAuu.....................   15      1   T63
......................................................................uuuguucguCcggcucgaguu.....................   16      1   T63
......................................................................uuuguucguucgAcucgaguu.....................   49      1   T63
......................................................................uuuAuucguucggcucgaguu.....................   2       1   T63
......................................................................uuugGucguucggcucgaguu.....................   1       1   T63
......................................................................Auuguucguucggcucgaguu.....................   47      1   T63
......................................................................uuuguucguuAggcucgaguu.....................   2       1   T63
......................................................................uuuguucguucggcuGgaguu.....................   5       1   T63
......................................................................uCuguucguucggcucgaguu.....................   3       1   T63
......................................................................uuuguucguucggcucgCguu.....................   95      1   T63
......................................................................uuuguuGguucggcucgaguu.....................   1       1   T63
......................................................................uuCguucguucggcucgaguu.....................   3       1   T63
......................................................................uuuguucguucggUucgaguu.....................   17      1   T63
......................................................................uuuguucguucggcuUgaguu.....................   8       1   T63
......................................................................uuuguucguucggcucgaguA.....................   536     1   T63
......................................................................uuuguucguucAgcucgaguu.....................   1       1   T63
......................................................................uuuguAcguucggcucgaguu.....................   4       1   T63
......................................................................uuuguucgAucggcucgaguu.....................   26      1   T63
......................................................................uuuguucguucggcucgaUuu.....................   5       1   T63
......................................................................Guuguucguucggcucgaguu.....................   14      1   T63
......................................................................uuuguucguuGggcucgaguu.....................   1       1   T63
......................................................................uuuguucguucggcCcgaguu.....................   2       1   T63
......................................................................uuuguucguucggcucgaCuu.....................   5       1   T63
......................................................................uuAguucguucggcucgaguu.....................   3       1   T63
......................................................................uuuguucguuUggcucgaguu.....................   24      1   T63
......................................................................uuuguucguucgUcucgaguu.....................   8       1   T63
......................................................................uuuguucguucCgcucgaguu.....................   1       1   T63
......................................................................uuuguucguucggAucgaguu.....................   3       1   T63
......................................................................uuGguucguucggcucgaguu.....................   1       1   T63
......................................................................uuuguGcguucggcucgaguu.....................   5       1   T63
......................................................................uuuguucUuucggcucgaguu.....................   3       1   T63
......................................................................uuuguucAuucggcucgaguu.....................   3       1   T63
......................................................................uuuguucguucggcucgGguu.....................   17      1   T63
......................................................................uuuguucgGucggcucgaguu.....................   7       1   T63
......................................................................uuuguuUguucggcucgaguu.....................   11      1   T63
......................................................................uuuguucguucggcucAaguu.....................   5       1   T63
......................................................................uuuguucguucggcucgUguu.....................   17      1   T63
......................................................................uuuguucguucUgcucgaguu.....................   5       1   T63
......................................................................uuuguucguucgCcucgaguu.....................   9       1   T63
......................................................................uuuguucguucggcAcgaguu.....................   5       1   T63
......................................................................uuuguucguucggcucgagCu.....................   2       1   T63
......................................................................uuuguucguucggcucgaguC.....................   8       1   T63
......................................................................uuuguucguucggcucUaguu.....................   4       1   T63
```

Star **Mature**

gaaggauacugucuuuauaagacuaguguggua**acuugagcaguacgaucagaac**ugcuuucaaaacugu**uuuguucguucggcucgaguua**ucaaguggucaaaaugacuu			
....uuugAucguucggcucgaguu.....................	1	1	T63
....uuuguucguucggcuAgaguu.....................	3	1	T63
....uuuguucguucggGucgaguu.....................	6	1	T63
....uAuguucguucggcucgaguu.....................	1	1	T63
....uuuguucguucggcucgagGu.....................	1	1	T63
....uuugCucguucggcucgaguu.....................	1	1	T63
....uuuguucguucggcucUaguua....................	1	1	T63
....uuuguucguucggcucgUguua....................	7	1	T63
....uuuCuucguucggcucgaguua....................	1	1	T63
....uuuguucguucggcucgaguuU....................	20566	1	T63
....uuuguucguucggcucgaAuua....................	1	1	T63
....uuuguucguucggcucgaCuua....................	4	1	T63
....uuuguucguucggcucgaguCa....................	2	1	T63
....uuuguucguucggUucgaguua....................	4	1	T63
....uuuguucguucggcucgaUuua....................	4	1	T63
....uuuguucguucggcuUgaguua....................	6	1	T63
....uuuguucguucggcucgaguAa....................	716	1	T63
....uuuguucguucggcucgagGua....................	2	1	T63
....uuuguucguucggcucgCguua....................	185	1	T63
....Cuuguucguucggcucgaguua....................	11	1	T63
....uuuguuUguucggcucgaguua....................	9	1	T63
....uuuguucguucggcucgagAua....................	6	1	T63
....uuuguucguucggcucgGguua....................	5	1	T63
....uuuguucguucggcAcgaguua....................	4	1	T63
....uuAguucguucggcucgaguua....................	5	1	T63
....uuuguucguucCgcucgaguua....................	1	1	T63
....uuuguucguucggcucgaguua....................	19757	0	T63
....uuuguucguucggcucgaguGa....................	2	1	T63
....uuuguucguuUggcucgaguua....................	37	1	T63
....uuuguucgCucggcucgaguua....................	3	1	T63
....uuuguucguuGggcucgaguua....................	3	1	T63
....uuuguucguGcggcucgaguua....................	1	1	T63
....uuugCucguucggcucgaguua....................	1	1	T63
....uuuguucguucggcGcgaguua....................	1	1	T63
....Guuguucguucggcucgaguua....................	10	1	T63
....uuuguucguucggcucgaguuC....................	1805	1	T63
....uuuguucAuucggcucgaguua....................	3	1	T63
....uuuguCcguucggcucgaguua....................	3	1	T63
....uuuguucgGucggcucgaguua....................	1	1	T63
....uuCguucguucggcucgaguua....................	1	1	T63
....Auuguucguucggcucgaguua....................	20	1	T63
....uuuguucUuucggcucgaguua....................	1	1	T63
....uuuguucguucggcucgaguuG....................	569	1	T63
....uuuguucguucggcCcgaguua....................	2	1	T63
....uuugAucguucggcucgaguua....................	2	1	T63
....uuuguucguucggcucAaguua....................	2	1	T63
....uuuguucguCcggcucgaguua....................	3	1	T63
....uuuguucguucgUcucgaguua....................	2	1	T63
....uuuguAcguucggcucgaguua....................	1	1	T63
....uuuguucguucggcuGgaguua....................	1	1	T63
....uuuguucguucAgcucgaguua....................	3	1	T63
....uuuguucguucUgcucgaguua....................	4	1	T63
....uuuguucguucggcucCaguua....................	1	1	T63
....uuuguucgAucggcucgaguua....................	13	1	T63
....uuuguucguucgAcucgaguua....................	20	1	T63
....uuuguucguucggAucgaguuau...................	1	1	T63
....uuuguCcguucggcucgaguuau...................	2	1	T63
....uuuguucguucggcucgaguuGu...................	16	1	T63
....uuuguucgAucggcucgaguuau...................	7	1	T63
....uuuguucguucggcucgaguGau...................	4	1	T63
....uuuguucguucggcucgaguuau...................	7147	0	T63
....uuuguucguucggcucgaguAau...................	10	1	T63
....uuuguucguucggUucgaguuau...................	1	1	T63
....uAuguucguucggcucgaguuau...................	1	1	T63
....uuuguucguucggcucgaguuCu...................	239	1	T63
....uuuguucguucggcucgaguCau...................	1	1	T63
....uuuguucguucggcuAgaguuau...................	1	1	T63
....uuuguucguucggcucgaAuuau...................	2	1	T63
....uuuCuucguucggcucgaguuau...................	1	1	T63
....uuugCucguucggcucgaguuau...................	1	1	T63

```
                                 Star                              Mature
gaaggauacugucuuuauaagacuaguguggua acuugagcaguacgaucagaac ugcuuucaaaacugu uuuguucguucggcucgaguua ucaaguggucaaaaugacuu
......................................................................uuuguucguucggcucCaguuau...................    5     1   T63
......................................................................Cuuguucguucggcucgaguuau...................    3     1   T63
......................................................................uuuguucguucggcuUgaguuau...................    4     1   T63
......................................................................uuuguucguucggcucgagGuau...................    1     1   T63
......................................................................uuuguucguucggcuGgaguuau...................    2     1   T63
......................................................................uuuguucguucgAcucgaguuau...................    14    1   T63
......................................................................Guuguucguucggcucgaguuau...................    1     1   T63
......................................................................uuuguucguucggcucAaguuau...................    1     1   T63
......................................................................uuuguucguucggcucgCguuau...................    35    1   T63
......................................................................uCuguucguucggcucgaguuau...................    1     1   T63
......................................................................Auuguucguucggcucgaguuau...................    8     1   T63
......................................................................uuuguuUguucggcucgaguuau...................    2     1   T63
......................................................................uuuguucguucggcucgaguuaA...................    4905  1   T63
......................................................................uuuguucguuUggcucgaguuau...................    15    1   T63
......................................................................uuuguucguucggcucUaguuau...................    2     1   T63
......................................................................uuAguucguucggcucgaguuau...................    1     1   T63
......................................................................uuuguucguucggcCcgaguuau...................    1     1   T63
......................................................................uuuguucguuGggcucgaguuau...................    1     1   T63
......................................................................uuuguucguucggcucgUguuau...................    4     1   T63
......................................................................uuuguucguucggcucgaUuuau...................    4     1   T63
......................................................................uuuguucguucggcucgGguuau...................    5     1   T63
......................................................................uuuguucguucCgcucgaguuau...................    1     1   T63
......................................................................uuuguucguucggcucgaguuUu...................    820   1   T63
......................................................................uuuguucguucggcucgagAuau...................    1     1   T63
......................................................................uuuguucgCucggcucgaguuau...................    2     1   T63
......................................................................uuuguucguCcggcucgaguuau...................    1     1   T63
......................................................................uuuUuucguucggcucgaguuau...................    2     1   T63
......................................................................uuuguucguucggcucgaguuaC...................    411   1   T63
......................................................................uuuguucguucggcucgaguuaG...................    581   1   T63
......................................................................uuuguucguucggcAcgaguuau...................    1     1   T63
......................................................................uuCguucguucggcucgaguuau...................    2     1   T63
......................................................................uuuguucguucggcucgaCuuau...................    3     1   T63
......................................................................uuuguGcguucggcucgaguuau...................    1     1   T63
......................................................................uuuguucguucggcucgaguuauG..................    9     1   T63
......................................................................uuuguucguucggcucgaguuauc..................    10    0   T63
......................................................................uuuguucguucggcucgaguuaAc..................    25    1   T63
......................................................................uuuguucguucggcucgaguuauU..................    276   1   T63
......................................................................uuuguucguucggcucgaguuaCc..................    1     1   T63
......................................................................uuuguucguucggcucgaguuUuc..................    2     1   T63
......................................................................uuuguucguucggcucgaguuauA..................    48    1   T63
......................................................................uuuguucguucggcucgaguuauAa.................    2     1   T63
......................................................................uuuguucguucggcucgaguuauUa.................    11    1   T63
......................................................................uuuguucguucggcucgaguuauca.................    1     0   T63
......................................................................uuuguucguucggcucgaguuaAca.................    2     1   T63
......................................................................uuuguucguucggcucgaguuaucU.................    6     1   T63
.......................................................................uuguucguucggcucgag.......................    2     0   T63
.......................................................................uuguucguucggcucgagu......................    115   0   T63
.......................................................................Guguucguucggcucgagu......................    2     1   T63
.......................................................................uuguucguucggcucgagG......................    1     1   T63
.......................................................................uuguucguucggcucgGgu......................    1     1   T63
.......................................................................uuUuucguucggcucgagu......................    4     1   T63
.......................................................................uuguucguucggcucgagA......................    6     1   T63
.......................................................................Cuguucguucggcucgaguu.....................    1     1   T63
.......................................................................uuguucguucggcucgaguu.....................    190   0   T63
.......................................................................uuUuucguucggcucgaguu.....................    2     1   T63
.......................................................................uuguucguucggcucgaguA.....................    2     1   T63
.......................................................................uuguucguucggcucAaguu.....................    1     1   T63
.......................................................................uuguucguucggUucgaguua....................    1     1   T63
.......................................................................uuguucguucggcuUgaguua....................    1     1   T63
.......................................................................uuguucguucggcucgaguAa....................    3     1   T63
.......................................................................uuguucguucggcucgaguuU....................    116   1   T63
.......................................................................uuguucguucggcucgaguuG....................    3     1   T63
.......................................................................Auguucguucggcucgaguua....................    2     1   T63
.......................................................................uuguucguuUggcucgaguua....................    1     1   T63
.......................................................................uuUuucguucggcucgaguua....................    2     1   T63
.......................................................................uuguucguucggcucgaUuua....................    1     1   T63
.......................................................................uuguucguucggcucgCguua....................    4     1   T63
.......................................................................uuguucguucggcucgaguuC....................    47    1   T63
.......................................................................uuguucguucggcucgaguua....................    309   0   T63
.......................................................................uuguucguucggcucgaguuUu...................    5     1   T63
```

Star **Mature**

gaaggauacugucuuuauaagacuaguguggua**acuugagcaguacgaucagaac**ugcuuucaaaacugu**uuguucguucggcucgaguua**ucaaguggucaaaaugacuu			
....Auguucguucggcucgaguuau...................	1	1	T63
....uuguucguucggcucgaguuaC...................	5	1	T63
....uuguucguucggcucgaguuaG...................	4	1	T63
....uuguucguucggcucgaguuCu...................	2	1	T63
....uuguucguucggcucgaguuaA...................	20	1	T63
....uuguucguucggcucgaguuau...................	52	0	T63
....uuguucguucggcCcgaguuau...................	1	1	T63
....uuguucguucggcucgaguuaCc..................	3	1	T63
....uuguucguucggcucgaguuauU..................	2	1	T63
....uuguucguucggcucgaguuauA..................	1	1	T63
....uUuucguucggcucgagu......................	1	1	T63
....uguucguucggcucgagu......................	5	0	T63
....uguCcguucggcucgaguu.....................	1	1	T63
....uguuUguucggcucgaguu.....................	1	1	T63
....uUuucguucggcucgaguu.....................	2	1	T63
....uguucguucggcucgaguu.....................	12	0	T63
....uguucguucggcucgaguuU....................	8	1	T63
....uguucguucggcucgCguua....................	1	1	T63
....uguucguucggcucgaguua....................	4	0	T63
....uguucguucggcucgaguuau...................	5	0	T63
....uguucguucggcucgaguuaA...................	1	1	T63
....guucguucggcucgaguu.....................	3	0	T63
....guucguucggcucgaguuU....................	2	1	T63
....guucguucggcucgaguua....................	5	0	T63
....guucguucggcucgaguuG....................	2	1	T63
....uucguucggcucgaguuU....................	2	1	T63
....uucguucggcucgaguua....................	1	0	T63
....ucguucggcucgaguuaA...................	1	1	T63
...........ucuuuauaagacuaguguggua..	24	0	te1
...........ucCuuauaagacuaguguggua..	1	1	te1
...........ucuuGauaagacuaguguggua..	2	1	te1
............cuuuauaagacuagugug...	1	0	te1
............cuuGauaagacuaguguggua..	1	1	te1
............cuuuauaagacuaguguggua..	154	0	te1
.............Auuauaagacuaguguggua..	1	1	te1
.............uuuauaagacuaguguggua..	147	0	te1
.............Cuuauaagacuaguguggua..	1	1	te1
.............uuGauaagacuaguguggua..	3	1	te1
..............uuauaagacuaguguggua..	13	0	te1
................................aacuugagcaguacgaucag...	3	0	te1
................................aacuugagcaguacgaucaga..	26	0	te1
................................Uacuugagcaguacgaucaga..	1	1	te1
................................aacuugagcaguacgaucagaa...	8	0	te1
................................aacuugagcaguacgaucagaaA..	1	1	te1
................................aacuugagcaguacgaucagaac..	4	0	te1
................................aacuugagcaguacgaucagaaU..	3	1	te1
.................................acuugagcCguacgauca..	1	1	te1
.................................acuugagcaguacgaucag...	8	0	te1
.................................acuugagcaguaUgaucaga..	1	1	te1
.................................acuugagcaguacgaucaga..	307	0	te1
.................................acuGgagcaguacgaucaga..	1	1	te1
.................................aGuugagcaguacgaucaga..	1	1	te1
.................................acuugagcaguacgaucagG..	3	1	te1
.................................acuugagUaguacgaucaga..	1	1	te1
.................................acuugagcaguacgauGagaa...	1	1	te1
.................................acuugagUaguacgaucagaa...	1	1	te1
.................................acuugagcaguacgGucagaa...	1	1	te1
.................................Gcuugagcaguacgaucagaa...	1	1	te1
.................................acuuUagcaguacgaucagaa...	1	1	te1
.................................acuugagcaguacAaucagaa...	1	1	te1
.................................acuugagcaguacgaucagaa...	449	0	te1
.................................acuugCgcaguacgaucagaac..	1	1	te1
.................................acuCgagcaguacgaucagaac..	1	1	te1
.................................Ucuugagcaguacgaucagaac..	1	1	te1
.................................acuugagcaguacgaucagaaA..	90	1	te1
.................................acuugagcaguacgaucagaac..	707	0	te1
.................................acuugagcaguacgaucagaaU..	6	1	te1
.................................acuugGgcaguacgaucagaac..	1	1	te1
.................................acuugagcagGacgaucagaac..	1	1	te1

Star **Mature**

```
gaaggauacugucuuuauaagacuaguguggua acuugagcaguacgaucagaac ugcuuucaaaacugu uuuguucguucggcucgaguua ucaaguggucaaaaugacuu
.................................acuugagcaguacgaucUgaac.........................................................  1    1  te1
.................................acuugagcaguacgaucagaacA........................................................  54   1  te1
.................................acuugagcaguacgaucagaacC........................................................  3    1  te1
.................................acuugagcaguacgaucagaacu........................................................  32   0  te1
.................................acuugagcaguacgaucagaacuU.......................................................  1    1  te1
.................................acuugagcaguacgaucagaacuA.......................................................  4    1  te1
..................................cuugagcaguacgaucaga...........................................................  2    0  te1
..................................cuugagcaguacgaucagG...........................................................  1    1  te1
..................................cuugagcaguacgaucagaa..........................................................  5    0  te1
..................................cuugagcaguacgaucagaac.........................................................  28   0  te1
..................................cuugagcaguacgaucagaaA.........................................................  5    1  te1
..................................cuugagcaguacgaucagaaU.........................................................  1    1  te1
..................................cuugagcaguacgaucagaacA........................................................  2    1  te1
..................................cuugagcaguacgaucagaacu........................................................  18   0  te1
...................................uugagcaguacgaucagaac.........................................................  1    0  te1
...................................uugagcaguacgaucagaaA.........................................................  1    1  te1
...................................uugagcaguacgaucagaacu........................................................  6    0  te1
....................................ugagcaguacgaucagaa..........................................................  2    0  te1
....................................Ggagcaguacgaucagaac.........................................................  1    1  te1
....................................ugagcaguacgaucagaac.........................................................  2    0  te1
....................................ugagcaguacgaucagaacu........................................................  1    0  te1
......................................................cugcuuucaaaacuguuuuguuc...................................  1    0  te1
.......................................................ugcuuucaaaacuguuuuguucguu................................  1    0  te1
...........................................................uucaaaacuguuuuguucguucggcuc..........................  1    0  te1
.................................................................acuguuuuguucguucggcucgagu......................  1    0  te1
..................................................................cuguuuuguucguucggcucgaguuU....................  2    1  te1
...................................................................uUuuuuguucguucggcucgag.......................  1    1  te1
...................................................................uguuuuguucguucggcucgagu......................  1    0  te1
...................................................................uUuuuuguucguucggcucgaguu.....................  1    1  te1
...................................................................uCuuuuguucguucggcucgaguua....................  1    1  te1
...................................................................uUuuuuguucguucggcucgaguua....................  2    1  te1
....................................................................Uuuuuguucguucggcucg.........................  1    1  te1
....................................................................Uuuuuguucguucggcucgag.......................  4    1  te1
....................................................................gCuuuguucguucggcucgag.......................  1    1  te1
....................................................................Cuuuuguucguucggcucgagu......................  1    1  te1
....................................................................Uuuuuguucguucggcucgagu......................  32   1  te1
....................................................................Auuuuguucguucggcucgagu......................  1    1  te1
....................................................................Uuuuuguucguucggcucgaguu.....................  45   1  te1
....................................................................guuuuguucguucggcucgaguu.....................  2    0  te1
....................................................................guuuuguucguucggcucgaguuU....................  2    1  te1
....................................................................Uuuuuguucguucggcucgaguua....................  51   1  te1
....................................................................guuuuguucguucggcucgaguuUu...................  1    1  te1
....................................................................Uuuuuguucguucggcucgaguuau...................  15   1  te1
.....................................................................uuuAguucguucggcucg.........................  1    1  te1
.....................................................................uuuuguucguucggcucg.........................  78   0  te1
.....................................................................uuuuguucguucggcuGg.........................  1    1  te1
.....................................................................Cuuuguucguucggcucga........................  1    1  te1
.....................................................................uuuuguucguucggcucga........................  170  0  te1
.....................................................................Cuuuguucguucggcucgag.......................  1    1  te1
.....................................................................uuuuguucguucggcucgaC.......................  1    1  te1
.....................................................................uuuuguucguucggcucAag.......................  1    1  te1
.....................................................................uuuuguucguucggcucgaA.......................  19   1  te1
.....................................................................uuuuguucguucggUucgag.......................  2    1  te1
.....................................................................uGuuguucguucggcucgag.......................  1    1  te1
.....................................................................uuuuguuGguucggcucgag.......................  1    1  te1
.....................................................................Guuuguucguucggcucgag.......................  1    1  te1
.....................................................................uuuuguucguucggcucgUg.......................  1    1  te1
.....................................................................uuuuguucguucggcucUag.......................  1    1  te1
.....................................................................uuuuguucguuAggcucgag.......................  1    1  te1
.....................................................................uuuuguucguucggcuUgag.......................  2    1  te1
.....................................................................uuuuguucguucggcucgaU.......................  27   1  te1
.....................................................................uuuuguucguucggcucgag.......................  959  0  te1
.....................................................................uuuuguucguuUggcucgag.......................  1    1  te1
.....................................................................uuuuguuUguucggcucgag.......................  1    1  te1
.....................................................................Auuuguucguucggcucgag.......................  2    1  te1
.....................................................................uuuugGucguucggcucgagu......................  3    1  te1
.....................................................................uuuuAuucguucggcucgagu......................  5    1  te1
.....................................................................uuuuguucguucggcucgagA......................  476  1  te1
.....................................................................uuuuguucguucgCcucgagu......................  1    1  te1
.....................................................................uCuuguucguucggcucgagu......................  3    1  te1
```

```
                                 Star                                  Mature
gaaggauacugucuuuauaagacuagugugguaacuugagcaguacgaucagaacugcuuucaaaacuguuuuguucguucggcucgaguuaucaaguggucaaaaugacuu
.....................................................................uuuuguucguucgUcucgagu......................	1	1	te1
.....................................................................uuuuguucguucggcucgGgu......................	1	1	te1
.....................................................................uuuuguucguAcggcucgagu......................	1	1	te1
.....................................................................uGuuguucguucggcucgagu......................	18	1	te1
.....................................................................uuuuguucguucggcuGgagu......................	1	1	te1
.....................................................................uuuAguucguucggcucgagu......................	8	1	te1
.....................................................................uuuuguucguucggcuUgagu......................	9	1	te1
.....................................................................uuuuguucCuucggcucgagu......................	1	1	te1
.....................................................................uuuuguuUguucggcucgagu......................	1	1	te1
.....................................................................uuuuguucguucggcGcgagu......................	1	1	te1
.....................................................................uuuuguucguucggUucgagu......................	7	1	te1
.....................................................................uuuuguucguucggcuAgagu......................	1	1	te1
.....................................................................uuuuguucguucggcucUagu......................	1	1	te1
.....................................................................uuAuguucguucggcucgagu......................	2	1	te1
.....................................................................uuuuguucguucggcucgagG......................	490	1	te1
.....................................................................uuuuguucguucggcucgUgu......................	4	1	te1
.....................................................................Auuuguucguucggcucgagu......................	16	1	te1
.....................................................................uuuuguucguucggcCcgagu......................	3	1	te1
.....................................................................uuuCguucguucggcucgagu......................	4	1	te1
.....................................................................Cuuuguucguucggcucgagu......................	13	1	te1
.....................................................................uuuGguucguucggcucgagu......................	1	1	te1
.....................................................................uuuuguucguucggcucgaCu......................	1	1	te1
.....................................................................uuuuguucguucUgcucgagu......................	4	1	te1
.....................................................................uuuuguucguucggcucAagu......................	1	1	te1
.....................................................................uuuuguucguucggcucgagu......................	7154	0	te1
.....................................................................uuuuguucguCcggcucgagu......................	1	1	te1
.....................................................................uuuuguucguucggcucgCgu......................	3	1	te1
.....................................................................uuuuguucguucggcucgaUu......................	6	1	te1
.....................................................................Guuuguucguucggcucgagu......................	8	1	te1
.....................................................................uuuuguucguucggcucgagC......................	11	1	te1
.....................................................................uuuuguucguuAggcucgagu......................	1	1	te1
.....................................................................uuuuguucguucggGucgagu......................	1	1	te1
.....................................................................uuGuguucguucggcucgagu......................	2	1	te1
.....................................................................uAuuguucguucggcucgagu......................	8	1	te1
.....................................................................uuuuguucguGcggcucgagu......................	1	1	te1
.....................................................................uuuuguucguuUggcucgagu......................	3	1	te1
.....................................................................uuuuguuGguucggcucgagu......................	1	1	te1
.....................................................................uuuuguucguucggcucgaAu......................	3	1	te1
.....................................................................uuuuguucguucgAcucgagu......................	4	1	te1
.....................................................................uuuAguucguucggcucgaguu.....................	11	1	te1
.....................................................................uuuuAuucguucggcucgaguu.....................	2	1	te1
.....................................................................uuuuguucguucggcucgagAu.....................	6	1	te1
.....................................................................uuuuguucguucggcuUgaguu.....................	3	1	te1
.....................................................................uCuuguucguucggcucgaguu.....................	1	1	te1
.....................................................................uuuuguucguucggcucgaguG.....................	5	1	te1
.....................................................................uuuuguucguucgAcucgaguu.....................	13	1	te1
.....................................................................uuuuguucguucggcuGgaguu.....................	1	1	te1
.....................................................................uuuuCuucguucggcucgaguu.....................	1	1	te1
.....................................................................uuGuguucguucggcucgaguu.....................	10	1	te1
.....................................................................Auuuguucguucggcucgaguu.....................	13	1	te1
.....................................................................uuuuguucguucggcucgaUuu.....................	1	1	te1
.....................................................................uuuuguucguucggcucgagGu.....................	1	1	te1
.....................................................................uuuuguucguucggcucgagCu.....................	1	1	te1
.....................................................................Guuuguucguucggcucgaguu.....................	5	1	te1
.....................................................................uuCuguucguucggcucgaguu.....................	2	1	te1
.....................................................................uuuuUuucguucggcucgaguu.....................	1	1	te1
.....................................................................uuuuguucguucggcucAaguu.....................	4	1	te1
.....................................................................uuuuguucguGcggcucgaguu.....................	2	1	te1
.....................................................................Cuuuguucguucggcucgaguu.....................	10	1	te1
.....................................................................uuuuguucguucUgcucgaguu.....................	1	1	te1
.....................................................................uuuuguucguuAggcucgaguu.....................	1	1	te1
.....................................................................uuuugCucguucggcucgaguu.....................	1	1	te1
.....................................................................uuuuguucguucggcucgGguu.....................	3	1	te1
.....................................................................uuAuguucguucggcucgaguu.....................	4	1	te1
.....................................................................uuuuguucguucggcucgaguC.....................	1	1	te1
.....................................................................uAuuguucguucggcucgaguu.....................	16	1	te1
.....................................................................uuuuguucguucggcucgaguu.....................	4922	0	te1
.....................................................................uuuuguucguucggcCcgaguu.....................	1	1	te1
.....................................................................uGuuguucguucggcucgaguu.....................	23	1	te1
.....................................................................uuuuguucgCucggcucgaguu.....................	1	1	te1
```

```
                                 Star                            Mature
gaaggauacugucuuuauaagacuaguguggua acuugagcaguacgaucagaac ugcuuucaaaacugu uuuguucguucggcucgaguua ucaaguggucaaaaugacuu
.....................................................................uuuGguucguucggcucgaguu.....................	1	1	te1
.....................................................................uuuuguucguuUggcucgaguu.....................	1	1	te1
.....................................................................uuuuguucguucgCcucgaguu.....................	2	1	te1
.....................................................................uuuCguucguucggcucgaguu.....................	4	1	te1
.....................................................................uuuuguucguucggcucgaguA.....................	81	1	te1
.....................................................................uuuuguucguucggcucgUguu.....................	4	1	te1
.....................................................................uuuuguuUguucggcucgaguua....................	6	1	te1
.....................................................................uuuuguucguucggcucgagCua....................	1	1	te1
.....................................................................uuCuguucguucggcucgaguua....................	7	1	te1
.....................................................................uuuuguucguucggcucgaguuC....................	104	1	te1
.....................................................................Auuuguucguucggcucgaguua....................	13	1	te1
.....................................................................uuuuguucguuAggcucgaguua....................	1	1	te1
.....................................................................uuAuguucguucggcucgaguua....................	5	1	te1
.....................................................................uuuuUuucguucggcucgaguua....................	2	1	te1
.....................................................................uuuuguucguucggUucgaguua....................	1	1	te1
.....................................................................uuuuguucguAcggcucgaguua....................	2	1	te1
.....................................................................uuuuguucguucggcucUaguua....................	1	1	te1
.....................................................................uuuCguucguucggcucgaguua....................	11	1	te1
.....................................................................uuuuguucguucggcucgaCuua....................	1	1	te1
.....................................................................uuuAguucguucggcucgaguua....................	12	1	te1
.....................................................................uuuuguucguucggcucgaguAa....................	145	1	te1
.....................................................................uuuuguucguucggcucgagAua....................	2	1	te1
.....................................................................Guuuguucguucggcucgaguua....................	4	1	te1
.....................................................................uuuuguucguucgAcucgaguua....................	5	1	te1
.....................................................................uuuuguucguucggcucgaguuG....................	41	1	te1
.....................................................................uuuuguucguucggcucgaguua....................	3065	0	te1
.....................................................................uuuugGucguucggcucgaguua....................	1	1	te1
.....................................................................Cuuuguucguucggcucgaguua....................	6	1	te1
.....................................................................uuuuguucguucggcucgaguuU....................	5776	1	te1
.....................................................................uuuuguucguucggcCcgaguua....................	1	1	te1
.....................................................................uuuuguucguGcggcucgaguua....................	2	1	te1
.....................................................................uGuuguucguucggcucgaguua....................	24	1	te1
.....................................................................uAuuguucguucggcucgaguua....................	17	1	te1
.....................................................................uuuuguucguucggcuUgaguua....................	2	1	te1
.....................................................................uuuuguucguuUggcucgaguua....................	1	1	te1
.....................................................................uCuuguucguucggcucgaguua....................	1	1	te1
.....................................................................uuGuguucguucggcucgaguua....................	14	1	te1
.....................................................................uuuGguucguucggcucgaguua....................	1	1	te1
.....................................................................uuuuguucguucggcucgUguua....................	2	1	te1
.....................................................................uAuuguucguucggcucgaguuau...................	5	1	te1
.....................................................................uuuuguucguucggcucgaguuaC...................	13	1	te1
.....................................................................uuAuguucguucggcucgaguuau...................	1	1	te1
.....................................................................uuuuguucguucggcucgaguAau...................	6	1	te1
.....................................................................uuuuguucguucggcucgaguuUu...................	569	1	te1
.....................................................................uuuuguucAuucggcucgaguuau...................	1	1	te1
.....................................................................uuuuCuucguucggcucgaguuau...................	1	1	te1
.....................................................................Auuuguucguucggcucgaguuau...................	5	1	te1
.....................................................................uuuCguucguucggcucgaguuau...................	1	1	te1
.....................................................................uuuuguucguucggcucgaCuuau...................	2	1	te1
.....................................................................uuuuguucguucggcucgaguuaA...................	900	1	te1
.....................................................................uGuuguucguucggcucgaguuau...................	3	1	te1
.....................................................................uuuugGucguucggcucgaguuau...................	1	1	te1
.....................................................................uuuuguucguucggcucgaguuGu...................	7	1	te1
.....................................................................uuuuguucguucggcucgaguuCu...................	18	1	te1
.....................................................................uCuuguucguucggcucgaguuau...................	1	1	te1
.....................................................................uuuAguucguucggcucgaguuau...................	4	1	te1
.....................................................................uuGuguucguucggcucgaguuau...................	3	1	te1
.....................................................................uuuuguucguuUggcucgaguuau...................	1	1	te1
.....................................................................uuuuguucguucggcucgUguuau...................	2	1	te1
.....................................................................uuuGguucguucggcucgaguuau...................	1	1	te1
.....................................................................uuuuguucguucggcucgaguuau...................	1775	0	te1
.....................................................................uuuugCucguucggcucgaguuau...................	1	1	te1
.....................................................................uuuuguucguucggcuUgaguuau...................	2	1	te1
.....................................................................uuuuguucguucgAcucgaguuau...................	2	1	te1
.....................................................................Cuuuguucguucggcucgaguuau...................	3	1	te1
.....................................................................uuCuguucguucggcucgaguuau...................	1	1	te1
.....................................................................Guuuguucguucggcucgaguuau...................	1	1	te1
.....................................................................uuuuguucguucggcucgaguuaG...................	18	1	te1
.....................................................................uuuuguuUguucggcucgaguuau...................	2	1	te1
.....................................................................uuuuAuucguucggcucgaguuau...................	1	1	te1
```

Star **Mature**

```
gaaggauacugucuuuauaagacuaguguggua acuugagcaguacgaucagaac ugcuuucaaaacugu uuuguucguucggcucgaguua ucaaguggucaaaaugacuu
.....................................................................uuuuguucguucggcucgaguuUuc..................	1	1	te1
.....................................................................uuuuguucguucggcucgaguuauU..................	167	1	te1
.....................................................................uuuuguucguucggcucgaguuauA..................	8	1	te1
.....................................................................uuuuguucguucggcucgaguuaAc..................	1	1	te1
.....................................................................uuuuguucguucggcucgaguuauG..................	1	1	te1
.....................................................................uuuuguucguucggcucgaguuauUa.................	1	1	te1
.....................................................................uuuuguucguucggcucgaguuaucU.................	1	1	te1
.....................................................................uuuuguucguucggcucgaguuauAa.................	1	1	te1
......................................................................uuuguucguucggcuUga........................	1	1	te1
......................................................................uuuguucguucggcucgU........................	2	1	te1
......................................................................uuuguucguucggcucga........................	191	0	te1
......................................................................uuuguucguucgAcucga........................	1	1	te1
......................................................................uuuguucguuUggcucga........................	1	1	te1
......................................................................uuuguucCuucggcucga........................	1	1	te1
......................................................................uuuguucguucggcucgC........................	1	1	te1
......................................................................uuuguuUguucggcucgag.......................	1	1	te1
......................................................................uuuguucguucggUucgag.......................	1	1	te1
......................................................................Cuuguucguucggcucgag.......................	2	1	te1
......................................................................uuuguucguucgAcucgag.......................	2	1	te1
......................................................................Guuguucguucggcucgag.......................	2	1	te1
......................................................................uuuguucguucggcucgaU.......................	22	1	te1
......................................................................uuuguucguucgCcucgag.......................	1	1	te1
......................................................................uCuguucguucggcucgag.......................	1	1	te1
......................................................................uuuguucguucggcucAag.......................	2	1	te1
......................................................................uuuUuucguucggcucgag.......................	1	1	te1
......................................................................uAuguucguucggcucgag.......................	1	1	te1
......................................................................Auuguucguucggcucgag.......................	6	1	te1
......................................................................uuuguucguucggcucgaA.......................	20	1	te1
......................................................................uuuguucgGucggcucgag.......................	1	1	te1
......................................................................uuuguucguucggcucgUg.......................	5	1	te1
......................................................................uuugCucguucggcucgag.......................	1	1	te1
......................................................................uuuguucguucggcuUgag.......................	2	1	te1
......................................................................uuuguucguucggcucgGg.......................	2	1	te1
......................................................................uuuguucguucggcucgag.......................	1380	0	te1
......................................................................uuuguucguucggcucgCg.......................	1	1	te1
......................................................................uuuguAcguucggcucgagu......................	1	1	te1
......................................................................uuuguucguucggcCcgagu......................	16	1	te1
......................................................................uuuguucguucggcuUgagu......................	46	1	te1
......................................................................uuuguucguucCgcucgagu......................	3	1	te1
......................................................................uuuguucguucgUcucgagu......................	8	1	te1
......................................................................uuuguucgAucggcucgagu......................	1	1	te1
......................................................................uuuguucguucgAcucgagu......................	66	1	te1
......................................................................uuuguucguucggcucgaAu......................	36	1	te1
......................................................................uuuguucguucggcucgagu......................	98364	0	te1
......................................................................uuCguucguucggcucgagu......................	5	1	te1
......................................................................uuuguucguCcggcucgagu......................	7	1	te1
......................................................................uuugGucguucggcucgagu......................	20	1	te1
......................................................................uuuguucguucggcucgaCu......................	13	1	te1
......................................................................uuuguucguucggcucUagu......................	2	1	te1
......................................................................uuuguuAguucggcucgagu......................	1	1	te1
......................................................................uuuguucguucgCcucgagu......................	14	1	te1
......................................................................uuuguucguucggcucgGgu......................	16	1	te1
......................................................................uuuguucguAcggcucgagu......................	1	1	te1
......................................................................uuuguuUguucggcucgagu......................	20	1	te1
......................................................................uuuguucguucggUucgagu......................	64	1	te1
......................................................................uuugCucguucggcucgagu......................	42	1	te1
......................................................................uuuAuucguucggcucgagu......................	4	1	te1
......................................................................uuuguucCuucggcucgagu......................	3	1	te1
......................................................................uuugAucguucggcucgagu......................	37	1	te1
......................................................................uuuguucguuAggcucgagu......................	9	1	te1
......................................................................uuuguCcguucggcucgagu......................	19	1	te1
......................................................................uuuguucguuUggcucgagu......................	59	1	te1
......................................................................uuuguucguucggcucgUgu......................	81	1	te1
......................................................................uuuguucguucggcucgagA......................	2475	1	te1
......................................................................uuuguucguGcggcucgagu......................	2	1	te1
......................................................................uuuguucguucAgcucgagu......................	22	1	te1
......................................................................Guuguucguucggcucgagu......................	183	1	te1
......................................................................uuuguuGguucggcucgagu......................	4	1	te1
......................................................................uuuguucguucggcucgCgu......................	11	1	te1
......................................................................uuuguucguucggcGcgagu......................	1	1	te1
```

Star **Mature**

gaaggauacugucuuuauaagacuagugugguaacuugagcaguacgaucagaacugcuuucaaaacuguuuuguucguucggcucgaguuaucaaguggucaaaaugacuu			
....uuGguucguucggcucgagu......................	5	1	te1
....uuuguucguucggcucgagC......................	166	1	te1
....uuuCuucguucggcucgagu......................	17	1	te1
....uuuUuucguucggcucgagu......................	20	1	te1
....Auuguucguucggcucgagu......................	205	1	te1
....uuuguucguucggcucCagu......................	2	1	te1
....Cuuguucguucggcucgagu......................	257	1	te1
....uuuguucguucggcuGgagu......................	6	1	te1
....uGuguucguucggcucgagu......................	25	1	te1
....uuuguucUuucggcucgagu......................	13	1	te1
....uuuguucguuGggcucgagu......................	4	1	te1
....uuuguGcguucggcucgagu......................	11	1	te1
....uuuguucAuucggcucgagu......................	11	1	te1
....uAuguucguucggcucgagu......................	14	1	te1
....uuuguucguucUgcucgagu......................	9	1	te1
....uuuguucguucggcucAagu......................	18	1	te1
....uuuguucguucggcucgagG......................	6647	1	te1
....uuuguucgGucggcucgagu......................	15	1	te1
....uuuguucguucggcucgaUu......................	100	1	te1
....uuuguucguucggAucgagu......................	5	1	te1
....uuuguucgCucggcucgagu......................	16	1	te1
....uuuguucguucggGucgagu......................	4	1	te1
....uCuguucguucggcucgagu......................	35	1	te1
....uuuguucgGucggcucgaguu.....................	31	1	te1
....uuuguucguucggcuGgaguu.....................	21	1	te1
....uuuguucguucggcucgagAu.....................	49	1	te1
....uuuguucguucggcucgagGu.....................	52	1	te1
....uuuguucguucgAcucgaguu.....................	220	1	te1
....uuuguucguucggcCcgaguu.....................	18	1	te1
....uuuguucguucUgcucgaguu.....................	20	1	te1
....Cuuguucguucggcucgaguu.....................	680	1	te1
....uuuguucUuucggcucgaguu.....................	46	1	te1
....uuugGucguucggcucgaguu.....................	31	1	te1
....uuuguCcguucggcucgaguu.....................	28	1	te1
....uuAguucguucggcucgaguu.....................	2	1	te1
....uuuguucguucggcucgagCu.....................	39	1	te1
....uuuguucguucggcucgaAuu.....................	13	1	te1
....uuuguucguGcggcucgaguu.....................	5	1	te1
....uuuguucguucggcucgaguC.....................	37	1	te1
....uuuguucguucAgcucgaguu.....................	57	1	te1
....uuuguucguucggcucAaguu.....................	14	1	te1
....uuuguucgCucggcucgaguu.....................	46	1	te1
....Guuguucguucggcucgaguu.....................	403	1	te1
....uuuguucguucggcucgaUuu.....................	31	1	te1
....uAuguucguucggcucgaguu.....................	23	1	te1
....uuuguucguucggcuAgaguu.....................	2	1	te1
....uuugCucguucggcucgaguu.....................	71	1	te1
....uuuguucguucggcucCaguu.....................	17	1	te1
....uuuguucguucggAucgaguu.....................	12	1	te1
....uuuguucguucggcucgCguu.....................	38	1	te1
....uuuguucguucggcuUgaguu.....................	149	1	te1
....uuugAucguucggcucgaguu.....................	47	1	te1
....uuuguucguCcggcucgaguu.....................	10	1	te1
....uuuguuUguucggcucgaguu.....................	45	1	te1
....uuuguucguucggcAcgaguu.....................	3	1	te1
....uuuguucguucggcGcgaguu.....................	5	1	te1
....uuuAuucguucggcucgaguu.....................	9	1	te1
....uuuguucguuUggcucgaguu.....................	135	1	te1
....uuuguAcguucggcucgaguu.....................	1	1	te1
....uuuUuucguucggcucgaguu.....................	20	1	te1
....uuuguucguucggcucgGguu.....................	55	1	te1
....uuuguucguuAggcucgaguu.....................	29	1	te1
....uuuguucguucggGucgaguu.....................	11	1	te1
....uuCguucguucggcucgaguu.....................	17	1	te1
....uuuguGcguucggcucgaguu.....................	31	1	te1
....uuuguucguucggcucgaCuu.....................	26	1	te1
....uCuguucguucggcucgaguu.....................	53	1	te1
....uuuguucCuucggcucgaguu.....................	4	1	te1
....uuuguucguucggUucgaguu.....................	142	1	te1
....uuuguuGguucggcucgaguu.....................	4	1	te1

Star   Mature

```
gaaggauacugucuuuauaagacuaguguggua acuugagcaguacgaucagaac ugcuuucaaaacugu uuuguucguucggcucgaguua ucaaguggucaaaaugacuu
......................................................................uuuguucgAucggcucgaguu.....................  1       1  te1
......................................................................uuuguucguucggcucUaguu.....................  7       1  te1
......................................................................uuuguucAuucggcucgaguu.....................  17      1  te1
......................................................................Auuguucguucggcucgaguu.....................  551     1  te1
......................................................................uGuguucguucggcucgaguu.....................  60      1  te1
......................................................................uuuguucguucggcucgaguG.....................  79      1  te1
......................................................................uuuguucguucggcucgaguu.....................  215813  0  te1
......................................................................uuuguucguucgCcucgaguu.....................  16      1  te1
......................................................................uuuguuAguucggcucgaguu.....................  1       1  te1
......................................................................uuuguucguucCgcucgaguu.....................  16      1  te1
......................................................................uuGguucguucggcucgaguu.....................  18      1  te1
......................................................................uuuguucguucgUcucgaguu.....................  20      1  te1
......................................................................uuuguucguuGggcucgaguu.....................  22      1  te1
......................................................................uuuCuucguucggcucgaguu.....................  33      1  te1
......................................................................uuuguucguucggcucgUguu.....................  188     1  te1
......................................................................uuuguucguucggcucgaguA.....................  2190    1  te1
......................................................................uuGguucguucggcucgaguua....................  26      1  te1
......................................................................uuuguucguuAggcucgaguua....................  33      1  te1
......................................................................uuuguucguucggUucgaguua....................  124     1  te1
......................................................................uuuguucguuUggcucgaguua....................  234     1  te1
......................................................................uuuguuUguucggcucgaguua....................  77      1  te1
......................................................................uuuguucguucUgcucgaguua....................  8       1  te1
......................................................................uuuguucguucggcucgaguuU....................  158496  1  te1
......................................................................uuuguucguCcggcucgaguua....................  14      1  te1
......................................................................uuuguucguucggcucgCguua....................  52      1  te1
......................................................................uuuUuucguucggcucgaguua....................  28      1  te1
......................................................................Auuguucguucggcucgaguua....................  722     1  te1
......................................................................uuuguucUuucggcucgaguua....................  67      1  te1
......................................................................uuuguucguucggcucgaguGa....................  25      1  te1
......................................................................uuuguucguuGggcucgaguua....................  23      1  te1
......................................................................uuuguucguucggcucgagGua....................  73      1  te1
......................................................................uuuguCcguucggcucgaguua....................  47      1  te1
......................................................................uAuguucguucggcucgaguua....................  34      1  te1
......................................................................uuuguucguucggcCcgaguua....................  20      1  te1
......................................................................uuuguuGguucggcucgaguua....................  13      1  te1
......................................................................uuuguucguucggGucgaguua....................  5       1  te1
......................................................................uuuguucguucggcucgaguAa....................  3375    1  te1
......................................................................uuuguAcguucggcucgaguua....................  1       1  te1
......................................................................uuuguucguucggcuGgaguua....................  30      1  te1
......................................................................uuuguucguucggcucgaguuG....................  1114    1  te1
......................................................................uuuguucAuucggcucgaguua....................  22      1  te1
......................................................................Cuuguucguucggcucgaguua....................  863     1  te1
......................................................................uuuguucguucggcucgagCua....................  58      1  te1
......................................................................uuuguucguucggcucgaUuua....................  62      1  te1
......................................................................uuuguucguucggcucgaguCa....................  35      1  te1
......................................................................uuuguucguucAgcucgaguua....................  51      1  te1
......................................................................uuuguucguucggcucUaguua....................  22      1  te1
......................................................................uuuguucguucggcAcgaguua....................  4       1  te1
......................................................................uuuguucguucggcuAgaguua....................  1       1  te1
......................................................................uuugCucguucggcucgaguua....................  114     1  te1
......................................................................uuuguucguucggcucCaguua....................  25      1  te1
......................................................................uuuguuAguucggcucgaguua....................  1       1  te1
......................................................................uuuguucgCucggcucgaguua....................  42      1  te1
......................................................................uCuguucguucggcucgaguua....................  68      1  te1
......................................................................uuugAucguucggcucgaguua....................  86      1  te1
......................................................................uuuguucguucggAucgaguua....................  10      1  te1
......................................................................uuuguucgAucggcucgaguua....................  2       1  te1
......................................................................uuugGucguucggcucgaguua....................  52      1  te1
......................................................................uuuAuucguucggcucgaguua....................  17      1  te1
......................................................................uuuguGcguucggcucgaguua....................  44      1  te1
......................................................................uuuguucguGcggcucgaguua....................  6       1  te1
......................................................................uuuCuucguucggcucgaguua....................  41      1  te1
......................................................................uuCguucguucggcucgaguua....................  18      1  te1
......................................................................uuuguucguucCgcucgaguua....................  6       1  te1
......................................................................uuuguucguucggcucgaCuua....................  26      1  te1
......................................................................uuuguucguucgCcucgaguua....................  12      1  te1
......................................................................uuuguucguucggcucgaguua....................  281957  0  te1
......................................................................uuuguucguucggcucAaguua....................  10      1  te1
......................................................................uuuguucCuucggcucgaguua....................  10      1  te1
......................................................................uuuguucguucggcucgagAua....................  26      1  te1
```

Star / Mature			
gaaggauacugucuuuauaagacuaguguggua**acuugagcaguacgaucagaac**ugcuuucaaaacugu**uuuguucguucggcucgaguua**ucaaguggucaaaaugacuu			
....uGuguucguucggcucgaguua....................	75	1	te1
....uuuguucguucggcucgGguua....................	56	1	te1
....uuuguucguucggcGcgaguua....................	7	1	te1
....uuuguucguucgUcucgaguua....................	8	1	te1
....uuAguucguucggcucgaguua....................	7	1	te1
....uuuguucguucggcuUgaguua....................	157	1	te1
....uuuguucguucggcucgUguua....................	237	1	te1
....uuuguucguucggcucgaguuC....................	6122	1	te1
....uuuguucguucgAcucgaguua....................	304	1	te1
....uuuguucgGucggcucgaguua....................	30	1	te1
....Guuguucguucggcucgaguua....................	569	1	te1
....uuuguucguucggcucgaAuua....................	12	1	te1
....uuuguuUguucggcucgaguuau...................	24	1	te1
....uuuguucguGcggcucgaguuau...................	4	1	te1
....uuuguucguuUggcucgaguuau...................	76	1	te1
....uuuguucguucggcuAgaguuau...................	1	1	te1
....uCuguucguucggcucgaguuau...................	25	1	te1
....uuuguucguucggcucgGguuau...................	34	1	te1
....uuuguucguucggcucUaguuau...................	2	1	te1
....uuuguucguucggcucgaguuaG...................	1750	1	te1
....uuuguucgGucggcucgaguuau...................	5	1	te1
....uuuguGcguucggcucgaguuau...................	15	1	te1
....uuuguucguuGggcucgaguuau...................	9	1	te1
....uuuguucguucggcucAaguuau...................	2	1	te1
....uuGguucguucggcucgaguuau...................	15	1	te1
....uuuguucguuAggcucgaguuau...................	8	1	te1
....Guuguucguucggcucgaguuau...................	144	1	te1
....uuuguucguucggcucgaguCau...................	14	1	te1
....uuuguucguucggcucgaguuGu...................	71	1	te1
....uuuguucguucgCcucgaguuau...................	3	1	te1
....uuuguucguucggcuGgaguuau...................	11	1	te1
....uuugGucguucggcucgaguuau...................	12	1	te1
....uuuguucCuucggcucgaguuau...................	2	1	te1
....uuuguucguucggUucgaguuau...................	30	1	te1
....uuuguucguCcggcucgaguuau...................	3	1	te1
....uuuguucguucggcucgaguGau...................	5	1	te1
....uuuCuucguucggcucgaguuau...................	13	1	te1
....uuuguucguucggcuUgaguuau...................	38	1	te1
....uuuguucguucggcucgagGuau...................	25	1	te1
....uuuguucAuucggcucgaguuau...................	7	1	te1
....uuuguucguucggcucgaguAau...................	56	1	te1
....uuuguucguucggcucgaguuaC...................	1882	1	te1
....uuuguucguucggcucgaguuaA...................	49835	1	te1
....uuuguucguucggcucgaAuuau...................	8	1	te1
....uuugCucguucggcucgaguuau...................	43	1	te1
....uuuguucguucggcCcgaguuau...................	5	1	te1
....Auuguucguucggcucgaguuau...................	177	1	te1
....uuuguucguucggcucgCguuau...................	15	1	te1
....uAuguucguucggcucgaguuau...................	7	1	te1
....Cuuguucguucggcucgaguuau...................	255	1	te1
....uuuguucgAucggcucgaguuau...................	2	1	te1
....uuuguuAguucggcucgaguuau...................	1	1	te1
....uuuguucguucCgcucgaguuau...................	3	1	te1
....uuuguucguucgAcucgaguuau...................	81	1	te1
....uuuguucguucggAucgaguuau...................	3	1	te1
....uuuguucguucggGucgaguuau...................	2	1	te1
....uuuguucguucggcucgagCuau...................	24	1	te1
....uuuUuucguucggcucgaguuau...................	8	1	te1
....uuuguCcguucggcucgaguuau...................	8	1	te1
....uuugAucguucggcucgaguuau...................	27	1	te1
....uuuAuucguucggcucgaguuau...................	8	1	te1
....uuuguucguucggcucgaguuau...................	79379	0	te1
....uuuguucguucggcucgaCuuau...................	11	1	te1
....uuuguucguucggcucgUguuau...................	72	1	te1
....uuuguuGguucggcucgaguuau...................	4	1	te1
....uuuguucUuucggcucgaguuau...................	18	1	te1
....uuuguAcguucggcucgaguuau...................	1	1	te1
....uuCguucguucggcucgaguuau...................	3	1	te1
....uuuguucguucggcucCaguuau...................	10	1	te1
....uuuguucgCucggcucgaguuau...................	15	1	te1

Star   Mature

```
gaaggauacugucuuuauaagacuaguguggua acuugagcaguacgaucagaac ugcuuucaaaacugu uuuguucguucggcucgaguua ucaaguggucaaaaugacuu
......................................................................uuuguucguucggcGcgaguuau................... 1 1 te1
......................................................................uuuguucguucAgcucgaguuau................... 35 1 te1
......................................................................uuuguucguucggcucgagAuau................... 11 1 te1
......................................................................uuuguucguucggcucgaUuuau................... 23 1 te1
......................................................................uuuguucguucggcucgaguuUu................... 8389 1 te1
......................................................................uuuguucguucUgcucgaguuau................... 3 1 te1
......................................................................uGuguucguucggcucgaguuau................... 16 1 te1
......................................................................uuuguucguucggcucgaguuCu................... 570 1 te1
......................................................................uuuguucguucggcucgaguuauG.................. 37 1 te1
......................................................................uuuguucguucggcucgaguuauU.................. 2381 1 te1
......................................................................Cuuguucguucggcucgaguuauc.................. 1 1 te1
......................................................................uuuguucguucggcucgaguuaCc.................. 2 1 te1
......................................................................uuuguucguucggcucgaguuUuc.................. 6 1 te1
......................................................................uuuguucguucggcucgaguuauA.................. 484 1 te1
......................................................................uuuguucguucggcucgaguuaAc.................. 48 1 te1
......................................................................uuuguucguucggcucgaguuCuc.................. 2 1 te1
......................................................................uuuguucguucggcucgaguuauc.................. 35 0 te1
......................................................................uuuguucguucggcucgaguuaGc.................. 2 1 te1
......................................................................uuuguucguucggcucgaguuaAca................. 3 1 te1
......................................................................uuuguucguucggcucgaguuaucG................. 1 1 te1
......................................................................uuuguucguucggcucgaguuauUa................. 56 1 te1
......................................................................uuuguucguucggcucgaguuauGa................. 3 1 te1
......................................................................uuuguucguucggcucgaguuauAa................. 24 1 te1
......................................................................uuuguucguucggcucgaguuauca................. 2 0 te1
......................................................................uuuguucguucggcucgaguuaucU................. 10 1 te1
......................................................................uuuguucguucggcucgaguuauUaa................ 2 1 te1
......................................................................uuuguucguucggcucgaguuaucUa................ 2 1 te1
......................................................................uuuguucguucggcucgaguuauAaa................ 7 1 te1
.......................................................................uuUuucguucggcucgag....................... 1 1 te1
.......................................................................uuguucguucggcucgag....................... 5 0 te1
.......................................................................uuguucguucggcucgagC...................... 1 1 te1
.......................................................................uCguucguucggcucgagu...................... 3 1 te1
.......................................................................uugCucguucggcucgagu...................... 1 1 te1
.......................................................................Guguucguucggcucgagu...................... 3 1 te1
.......................................................................uuUuucguucggcucgagu...................... 15 1 te1
.......................................................................uuguucguucggcucgagA...................... 12 1 te1
.......................................................................uuguucguucggcucgagu...................... 360 0 te1
.......................................................................Cuguucguucggcucgagu...................... 5 1 te1
.......................................................................uuCuucguucggcucgagu...................... 1 1 te1
.......................................................................Auguucguucggcucgagu...................... 2 1 te1
.......................................................................uuguucguucggcucgGgu...................... 1 1 te1
.......................................................................uuguucguucggcucgagG...................... 18 1 te1
.......................................................................uuguucguucggcucgaUu...................... 1 1 te1
.......................................................................uuguucguucggcucgaCuu..................... 1 1 te1
.......................................................................uuguucguucgAcucgaguu..................... 1 1 te1
.......................................................................uuUuucguucggcucgaguu..................... 12 1 te1
.......................................................................uuguucguucggcucgaguA..................... 19 1 te1
.......................................................................Cuguucguucggcucgaguu..................... 16 1 te1
.......................................................................uuguucguucggcucgaAuu..................... 1 1 te1
.......................................................................uuguGcguucggcucgaguu..................... 1 1 te1
.......................................................................uuguucguucggcucgaguC..................... 1 1 te1
.......................................................................uugCucguucggcucgaguu..................... 1 1 te1
.......................................................................Auguucguucggcucgaguu..................... 10 1 te1
.......................................................................uuguucguucggcucgaguu..................... 2309 0 te1
.......................................................................uuguucguucggcuUgaguu..................... 2 1 te1
.......................................................................uuguucguucUgcucgaguu..................... 1 1 te1
.......................................................................Guguucguucggcucgaguu..................... 13 1 te1
.......................................................................uuguucguCcggcucgaguu..................... 1 1 te1
.......................................................................uCguucguucggcucgaguu..................... 2 1 te1
.......................................................................uuguucguucgCcucgaguu..................... 1 1 te1
.......................................................................uuguucguucggcucgUguu..................... 1 1 te1
.......................................................................uuguucguucggcucgaguG..................... 1 1 te1
.......................................................................uuguCcguucggcucgaguu..................... 1 1 te1
.......................................................................uugAucguucggcucgaguua.................... 2 1 te1
.......................................................................uuguucguucggcucgaguua.................... 9647 0 te1
.......................................................................uuguucguucggcucgUguua.................... 3 1 te1
.......................................................................uuCuucguucggcucgaguua.................... 3 1 te1
.......................................................................uuguucguucggcucgGguua.................... 6 1 te1
.......................................................................uuguucAuucggcucgaguua.................... 1 1 te1
.......................................................................uuguucguucggUucgaguua.................... 1 1 te1
```

Star **Mature**

gaaggauacugucuuuauaagacuagugugguaacuugagcaguacgaucagaacugcuuucaaaacuguuuguucguucggcucgaguuaucaaguggucaaaaugacuu

....Guguucguucggcucgaguua....................	27	1	te1
....Auguucguucggcucgaguua....................	33	1	te1
....uuguucguucggcucgaguuG....................	6	1	te1
....uuguucguucggcucgaguAa....................	14	1	te1
....uuguucguucggcucgaguuU....................	1319	1	te1
....uuguucCuucggcucgaguua....................	1	1	te1
....uuguucguuGggcucgaguua....................	1	1	te1
....uuguucguucggcuUgaguua....................	7	1	te1
....uuguAcguucggcucgaguua....................	2	1	te1
....uuUuucguucggcucgaguua....................	19	1	te1
....uuguucguucggcucgaguuC....................	382	1	te1
....uuguucguucggcucgagCua....................	3	1	te1
....uuguucguucggcucgCguua....................	6	1	te1
....uuguGcguucggcucgaguua....................	1	1	te1
....uuguucgGucggcucgaguua....................	1	1	te1
....uugCucguucggcucgaguua....................	1	1	te1
....uuguucguucggcucgaCuua....................	2	1	te1
....uuguucguucggcucgaguGa....................	3	1	te1
....uuguucguucgAcucgaguua....................	12	1	te1
....uAguucguucggcucgaguua....................	2	1	te1
....uuguucguucAgcucgaguua....................	1	1	te1
....uGguucguucggcucgaguua....................	3	1	te1
....uuguucguucggcucgaguCa....................	1	1	te1
....uuguucguucggcucgagGua....................	3	1	te1
....Cuguucguucggcucgaguua....................	40	1	te1
....uuguucgCucggcucgaguua....................	3	1	te1
....uCguucguucggcucgaguua....................	4	1	te1
....uuguucguuUggcucgaguua....................	15	1	te1
....uuguucguucggcucgaUuua....................	1	1	te1
....uuguuUguucggcucgaguua....................	3	1	te1
....uuguucguucggcucgagAua....................	2	1	te1
....uuguucguCcggcucgaguua....................	2	1	te1
....uuAuucguucggcucgaguua....................	1	1	te1
....uuUuucguucggcucgaguuau...................	7	1	te1
....uuguucguucggcucgagCuau...................	1	1	te1
....uuguucguucggcucgaguuaG...................	12	1	te1
....uuguucguucggcucgaguuUu...................	53	1	te1
....Auguucguucggcucgaguuau...................	4	1	te1
....Guguucguucggcucgaguuau...................	1	1	te1
....uuCuucguucggcucgaguuau...................	1	1	te1
....uuguucgCucggcucgaguuau...................	1	1	te1
....Cuguucguucggcucgaguuau...................	2	1	te1
....uuguucguucgAcucgaguuau...................	1	1	te1
....uuguucguucggcucgaguuCu...................	18	1	te1
....uugCucguucggcucgaguuau...................	1	1	te1
....uuguucguucggcucgaguuaA...................	572	1	te1
....uuguucguucggcucgaguuGu...................	2	1	te1
....uuguGcguucggcucgaguuau...................	1	1	te1
....uuguucguucggcucgaguuaC...................	33	1	te1
....uuguucguucggcucgaguuau...................	775	0	te1
....uuguucguucggcucgUguuau...................	1	1	te1
....uuguucguucggcCcgaguuau...................	1	1	te1
....uuguucguucggcucgaguuauc..................	2	0	te1
....uuguucguucggcucgaguuauU..................	32	1	te1
....uuguucguucggcucgaguuauA..................	8	1	te1
....uuguucguucggcucgaguuauAaa................	1	1	te1
....Cguucguucggcucgagu......................	2	1	te1
....uguucguucggcucgagA......................	2	1	te1
....uguucguucgUcucgagu......................	1	1	te1
....uUuucguucggcucgagu......................	8	1	te1
....uguucguucggcucgagG......................	2	1	te1
....uguucguucggcucgagu......................	47	0	te1
....Aguucguucggcucgaguu.....................	2	1	te1
....uguucguucggcucgaguA.....................	3	1	te1
....uUuucguucggcucgaguu.....................	27	1	te1
....uguucguucggcucgaguu.....................	129	0	te1
....uguucguuUggcucgaguu.....................	1	1	te1
....Gguucguucggcucgaguu.....................	3	1	te1
....Cguucguucggcucgaguu.....................	1	1	te1
....uguucguucggcucgaguuC....................	9	1	te1

```
                                 Star                                    Mature
gaaggauacugucuuuauaagacuaguguggua acuugagcaguacgaucagaac ugcuuucaaaacugu uuuguucguucggcucgaguua ucaagugguucaaaaugacuu
........................................................................uCuucguucggcucgaguua.....................	1	1	te1
........................................................................uUuucguucggcucgaguua.....................	36	1	te1
........................................................................Gguucguucggcucgaguua.....................	3	1	te1
........................................................................Cguucguucggcucgaguua.....................	1	1	te1
........................................................................uguucguucggcucgaguuG.....................	2	1	te1
........................................................................Aguucguucggcucgaguua.....................	3	1	te1
........................................................................uguucguucggcucgaguua.....................	282	0	te1
........................................................................uguucguucggcucgaguuU.....................	130	1	te1
........................................................................uguucguucggcucgaguuUu....................	9	1	te1
........................................................................Aguucguucggcucgaguuau....................	1	1	te1
........................................................................uguucguucggcucgaguuaA....................	38	1	te1
........................................................................Cguucguucggcucgaguuau....................	1	1	te1
........................................................................uguucguucggcucgaguuaG....................	3	1	te1
........................................................................uguucguucggcucgaguuau....................	76	0	te1
........................................................................uUuucguucggcucgaguuau....................	10	1	te1
........................................................................uguucguucggcucgaguuaC....................	4	1	te1
........................................................................Gguucguucggcucgaguuau....................	1	1	te1
........................................................................uguucguucggcucgaguuauU...................	1	1	te1
........................................................................uguucguucggcucgaguuauA...................	1	1	te1
.........................................................................guucguucggcucgaguu......................	38	0	te1
.........................................................................Uuucguucggcucgaguu......................	10	1	te1
.........................................................................guucguucggcucgaguuC.....................	2	1	te1
.........................................................................Uuucguucggcucgaguua.....................	12	1	te1
.........................................................................guucguucggcucgaguAa.....................	1	1	te1
.........................................................................guucguucggcucgaguuU.....................	52	1	te1
.........................................................................guucguucggcucgaguua.....................	78	0	te1
.........................................................................guucguucggcucgaguGa.....................	1	1	te1
.........................................................................guucguucggcucgaguuaG....................	2	1	te1
.........................................................................Uuucguucggcucgaguuau....................	4	1	te1
.........................................................................guucguucggcucgaguuau....................	35	0	te1
.........................................................................guucguucggcucgaguuUu....................	4	1	te1
.........................................................................guucguucggcucgaguuaA....................	18	1	te1
.........................................................................guucguucggcucgaguuauU...................	1	1	te1
.........................................................................guucguucggcucgaguuaucU..................	1	1	te1
.........................................................................guucguucggcucgaguuauUa..................	1	1	te1
..........................................................................uucguucggcucgaguuU.....................	6	1	te1
..........................................................................uucguucggcucgaguua.....................	13	0	te1
..........................................................................uuUguucggcucgaguua.....................	8	1	te1
..........................................................................uucguucggcucgaguuUu....................	1	1	te1
..........................................................................uucguucggcucgaguuau....................	6	0	te1
..........................................................................uucguucggcucgaguuaA....................	2	1	te1
..........................................................................uuUguucggcucgaguuau....................	3	1	te1
...........................................................................ucguucggcucgaguuau....................	9	0	te1
...........................................................................ucguucggcucgaguuaA....................	2	1	te1

.............uuuauaagacuaguguggua................................................................................	1	0	egg
.................................acuugagcaguacgaucagaac..........................................................	35	0	egg
.................................acuuUagcaguacgaucagaac..........................................................	2	1	egg
.................................Ucuugagcaguacgaucagaac..........................................................	1	1	egg
.................................acuugCgcaguacgaucagaac..........................................................	1	1	egg
.................................acuugagcGguacgaucagaac..........................................................	1	1	egg
.................................acuugagcaguacgaucagaacu.........................................................	1	0	egg
.....................................................................uuuuguuUguucggcucgagu.......................	1	1	egg
.....................................................................uuuuguucguucggcucgagu.......................	4	0	egg
.....................................................................uuuuguucguucggcucgaguu......................	19	0	egg
.....................................................................uuuuguucguucggcucgaguA......................	1	1	egg
.....................................................................uuuuguucguucggcucgGguu......................	1	1	egg
.....................................................................uCuuguucguucggcucgaguu......................	1	1	egg
.....................................................................uuuuguucguucggcucgaguuU.....................	4	1	egg
.....................................................................uuuuguucguucggcucgaguua.....................	8	0	egg
.....................................................................uuuuguucguucggcucgaguuau....................	2	0	egg
.....................................................................uuuuguucguucggcucgaguuaC....................	1	1	egg
.....................................................................uuuuguucguucggcucgaguuaA....................	1	1	egg
......................................................................uuuguucguucggcucgag........................	3	0	egg
......................................................................uuugGucguucggcucgagu.......................	1	1	egg
......................................................................uuuguucguucggcucgagu.......................	29	0	egg
......................................................................uuuguucguucggcucgagA.......................	1	1	egg
......................................................................uuuUuucguucggcucgagu.......................	1	1	egg
......................................................................uuuguucgCucggcucgagu.......................	1	1	egg
......................................................................uuuguucguucggcucgagC.......................	2	1	egg
```

Star Mature

```
gaaggauacugucuuuauaagacuagugugguaacuugagcaguacgaucagaacugcuuucaaaacuguuuuguucguucggcucgaguuaucaaguggucaaaaugacuu
......................................................................uuugAucguucggcucgaguu.....................   2     1   egg
......................................................................uuuguucguucggcucgaguA.....................   1     1   egg
......................................................................uuugGucguucggcucgaguu.....................   6     1   egg
......................................................................uuuguucguucggcucgaguu.....................   225   0   egg
......................................................................uuuUuucguucggcucgaguu.....................   5     1   egg
......................................................................uuugCucguucggcucgaguu.....................   3     1   egg
......................................................................uuuguucguucggcucgaguC.....................   9     1   egg
......................................................................uAuguucguucggcucgaguu.....................   1     1   egg
......................................................................uuuguucAuucggcucgaguu.....................   1     1   egg
......................................................................uuuguucguucggcucUaguu.....................   1     1   egg
......................................................................uuuguucguucgAcucgaguu.....................   1     1   egg
......................................................................uuuguucguucggcucgaguGa....................   1     1   egg
......................................................................uuuguuUguucggcucgaguua....................   2     1   egg
......................................................................uuuguCcguucggcucgaguua....................   2     1   egg
......................................................................uuuguucguucggcGcgaguua....................   1     1   egg
......................................................................uuuguucguucggcucgagAua....................   1     1   egg
......................................................................uuuguucguucggcucgaguCa....................   1     1   egg
......................................................................uuugCucguucggcucgaguua....................   3     1   egg
......................................................................uuuguucguucggcuAgaguua....................   1     1   egg
......................................................................uuuguucCuucggcucgaguua....................   1     1   egg
......................................................................uuuguucguucggcCcgaguua....................   1     1   egg
......................................................................uuugAucguucggcucgaguua....................   3     1   egg
......................................................................uuugGucguucggcucgaguua....................   15    1   egg
......................................................................uuuguucguucggcAcgaguua....................   1     1   egg
......................................................................uuuguucgCucggcucgaguua....................   1     1   egg
......................................................................uuuguucAuucggcucgaguua....................   1     1   egg
......................................................................uuuguucguAcggcucgaguua....................   1     1   egg
......................................................................uCuguucguucggcucgaguua....................   3     1   egg
......................................................................uuCguucguucggcucgaguua....................   2     1   egg
......................................................................uuuUuucguucggcucgaguua....................   1     1   egg
......................................................................uuuguucguucggcucgaguua....................   346   0   egg
......................................................................uuuguucguucggcucgaguuG....................   4     1   egg
......................................................................uuuguucguucggcucgGguua....................   1     1   egg
......................................................................uuuguucguucggcucgaguuU....................   24    1   egg
......................................................................uuuguucguucggcucgagCua....................   2     1   egg
......................................................................uuuguucguucggcucgaguuaA...................   10    1   egg
......................................................................uuuguucguucggcucgaguuau...................   14    0   egg
......................................................................uuuguucguucggcucgaguuUu...................   1     1   egg
......................................................................uCuguucguucggcucgaguuau...................   1     1   egg
......................................................................uuuguucguucggcucgaguuauU..................   4     1   egg
.......................................................................uuguucguucggcucgaguua....................   1     0   egg

...........ucuuuauaagacuagugugguа...............................................................................   3     0   T6P
............cuuuauaagacuagugugguа...............................................................................   7     0   T6P
.............uuuauaagacuagugugguа...............................................................................   4     0   T6P
..............uuauaagacuagugugguа...............................................................................   1     0   T6P
...............................uaacuugagcaguacgaucagaa..........................................................   1     0   T6P
................................aacuugagcaguacgaucagaa..........................................................   2     0   T6P
................................aacuugagcaguacgaucagaac.........................................................   1     0   T6P
.................................acuugagcaguacgaucaga...........................................................   17    0   T6P
.................................acuugagcaguacgaucagaa..........................................................   34    0   T6P
.................................acuugagcaguacgaucagaaU.........................................................   3     1   T6P
.................................acuugaCcaguacgaucagaac.........................................................   1     1   T6P
.................................acuugagcaguacgaucagaaA.........................................................   5     1   T6P
.................................acuugagcaguaUgaucagaac.........................................................   1     1   T6P
.................................acuugagcaguacgaucagaac.........................................................   87    0   T6P
.................................acuugagUaguacgaucagaac.........................................................   1     1   T6P
.................................acuugagcaguacgaucagaacu........................................................   2     0   T6P
.................................acuugagcaguacgaucagaacC........................................................   1     1   T6P
.................................acuugagcaguacgaucagaacA........................................................   3     1   T6P
.................................acuugagcaguacgaucagaacuU.......................................................   2     1   T6P
..................................cuugagcaguacgaucaga...........................................................   1     0   T6P
..................................cuugagcaguacgaucagaa..........................................................   1     0   T6P
..................................cuugagcaguacgaucagaac.........................................................   1     0   T6P
..................................cuugagcaguacgaucaUaac.........................................................   1     1   T6P
...................................uugagcaguacgaucagaacu........................................................   1     0   T6P
....................................ugagcaguacgaucagaa..........................................................   2     0   T6P
.....................................................................Uuuuguucguucggcucgagu......................   2     1   T6P
.....................................................................Uuuuguucguucggcucgaguu.....................   2     1   T6P
.....................................................................Uuuuguucguucggcucgaguua....................   1     1   T6P
```

Star Mature

```
gaaggauacugucuuuauaagacuagugugguaacuugagcaguacgaucagaacugcuuucaaaacuguuuuguucguucggcucgaguuaucaaguggucaaaaugacuu
```

```
gaaggauacugucuuuauaagacuagugugguaacuugagcaguacgaucagaacugcuuucaaaacuguuuuguucguucggcucgaguuaucaaguggucaaaaugacuu
....................................................................guuuuguucguucggcucgaguua....................    1     0    T6P
....................................................................Uuuuuguucguucggcucgaguuau...................    1     1    T6P
.....................................................................uuuuguucguucggcucg.........................    5     0    T6P
.....................................................................Auuuguucguucggcucga........................    1     1    T6P
.....................................................................uuuuguucguucggcucga........................    11    0    T6P
.....................................................................uuuuguucguucggcucAa........................    1     1    T6P
.....................................................................uuuuguucguucggcucgag.......................    7     0    T6P
.....................................................................uuuuguucguucggcucgagu......................    436   0    T6P
.....................................................................uuuuguucguucggcucgaUu......................    1     1    T6P
.....................................................................uuuuguucguucggcucgagA......................    12    1    T6P
.....................................................................uuuuguucUuucggcucgagu......................    1     1    T6P
.....................................................................uuuuguucguucggcucgagG......................    1     1    T6P
.....................................................................uuuuguCcguucggcucgaguu.....................    1     1    T6P
.....................................................................uuuuguucguucUgcucgaguu.....................    1     1    T6P
.....................................................................uuCuguucguucggcucgaguu.....................    1     1    T6P
.....................................................................uuuuguucguucggcucgaCuu.....................    1     1    T6P
.....................................................................uuuuguGcguucggcucgaguu.....................    2     1    T6P
.....................................................................uuuuguucguucggcucgaguA.....................    12    1    T6P
.....................................................................uuuuguucguucgCcucgaguu.....................    1     1    T6P
.....................................................................uuuuguucgAucggcucgaguu.....................    1     1    T6P
.....................................................................uuuCguucguucggcucgaguu.....................    1     1    T6P
.....................................................................uuuuguuUguucggcucgaguu.....................    1     1    T6P
.....................................................................uuuuguucguucgAcucgaguu.....................    3     1    T6P
.....................................................................uuuuguucguucggcucgaguu.....................    1072  0    T6P
.....................................................................uuuuguucguucggcucgaguuC....................    31    1    T6P
.....................................................................uuuuguucguucggcucCaguua....................    1     1    T6P
.....................................................................uuuuguucguucggcucgaguAa....................    10    1    T6P
.....................................................................uuuuguucguucggcucgaguuG....................    13    1    T6P
.....................................................................uuCuguucguucggcucgaguua....................    2     1    T6P
.....................................................................uuuuguucguucgCcucgaguua....................    1     1    T6P
.....................................................................uuuuguucguucggcucgaguuU....................    544   1    T6P
.....................................................................uuuuguucguucggcucgaguua....................    375   0    T6P
.....................................................................uuuuguucguucggcucgaguuUu...................    32    1    T6P
.....................................................................uuuuguucguucggcucgaguuaA...................    56    1    T6P
.....................................................................uuuuguucguucggcucgaguuCu...................    1     1    T6P
.....................................................................uuuuguucguucggcucgaguAau...................    1     1    T6P
.....................................................................uuuuguucguucggcucgaguuau...................    210   0    T6P
.....................................................................uuuuguucguucggcuUgaguuau...................    2     1    T6P
.....................................................................uuuuguucguucggcucgaguCau...................    1     1    T6P
.....................................................................uuuuguucCuucggcucgaguuau...................    1     1    T6P
.....................................................................uuuuguucguucggcucgaguuauU..................    27    1    T6P
......................................................................uuuguucguucggcucga........................    13    0    T6P
......................................................................uuuguucguucggcucgag.......................    81    0    T6P
......................................................................uuuguucguucggcucgCg.......................    1     1    T6P
......................................................................uuuguucguucggcucgaA.......................    3     1    T6P
......................................................................uuuguucguucggcucgaU.......................    3     1    T6P
......................................................................uuuguucguGcggcucgagu......................    1     1    T6P
......................................................................uuuguucCuucggcucgagu......................    1     1    T6P
......................................................................uuuguucguucCgcucgagu......................    1     1    T6P
......................................................................uuuguucguuUggcucgagu......................    4     1    T6P
......................................................................uuCguucguucggcucgagu......................    1     1    T6P
......................................................................uuuguucguucggcucgagG......................    3     1    T6P
......................................................................uuuguucguucggcucUagu......................    1     1    T6P
......................................................................uuuUuucguucggcucgagu......................    7     1    T6P
......................................................................uuuguucguucggcucgagC......................    4     1    T6P
......................................................................uuuguucguucggcucgaAu......................    2     1    T6P
......................................................................uuuguuAguucggcucgagu......................    1     1    T6P
......................................................................uuuguucguucggcucgUgu......................    1     1    T6P
......................................................................uuuguucguucggcuUgagu......................    2     1    T6P
......................................................................uuuguucguucggcucgagu......................    3635  0    T6P
......................................................................uuuguucguucggUucgagu......................    3     1    T6P
......................................................................uuuguucguucUgcucgagu......................    2     1    T6P
......................................................................uuuguucguucggcucCagu......................    1     1    T6P
......................................................................uuuguucAuucggcucgagu......................    2     1    T6P
......................................................................uuuguucguucggcucgaUu......................    2     1    T6P
......................................................................uuuguucgCucggcucgagu......................    1     1    T6P
......................................................................uuuguucguucgAcucgagu......................    3     1    T6P
......................................................................uuuguucguucggcucgaCu......................    1     1    T6P
......................................................................uuuguucguucggGucgagu......................    1     1    T6P
......................................................................uuuguucguucggcCcgagu......................    1     1    T6P
```

Star / Mature

```
gaaggauacugucuuuauaagacuaguguggua acuugagcaguacgaucagaac ugcuuucaaaacugu uuuguucguucggcucgaguua ucaaguggucaaaaugacuu
```

```
gaaggauacugucuuuauaagacuagugugguaacuugagcaguacgaucagaacugcuuucaaaacuguuuuguucguucggcucgaguuaucaaguggucaaaaugacuu
......................................................................uuuguAcguucggcucgagu......................   1      1   T6P
......................................................................uuuguucguucgUcucgagu......................   1      1   T6P
......................................................................uuuguucguucggcucgagA......................   51     1   T6P
......................................................................uuugGucguucggcucgagu......................   3      1   T6P
......................................................................Auuguucguucggcucgagu......................   2      1   T6P
......................................................................uuuguuUguucggcucgagu......................   1      1   T6P
......................................................................uuuguucguucgCcucgagu......................   3      1   T6P
......................................................................uuuguuGguucggcucgagu......................   1      1   T6P
......................................................................uuuguuUguucggcucgaguu.....................   5      1   T6P
......................................................................Cuuguucguucggcucgaguu.....................   6      1   T6P
......................................................................uuuguucguucggcucgaUuu.....................   1      1   T6P
......................................................................uuuguucguucggGucgaguu.....................   4      1   T6P
......................................................................uuuguucguucggcucgaguC.....................   4      1   T6P
......................................................................uuuguucguucggcuAgaguu.....................   1      1   T6P
......................................................................uuuguucguuUggcucgaguu.....................   15     1   T6P
......................................................................uuuguucguucggcucgGguu.....................   4      1   T6P
......................................................................uuuguucguucggcucgaAuu.....................   4      1   T6P
......................................................................uuugGucguucggcucgaguu.....................   2      1   T6P
......................................................................uuuAuucguucggcucgaguu.....................   1      1   T6P
......................................................................Guuguucguucggcucgaguu.....................   2      1   T6P
......................................................................uuuguucguGcggcucgaguu.....................   2      1   T6P
......................................................................uuuguucguucggcucgaguG.....................   2      1   T6P
......................................................................uuuguuGguucggcucgaguu.....................   17     1   T6P
......................................................................uuuguucguucggcucgagAu.....................   5      1   T6P
......................................................................uuuguucguucggcucCaguu.....................   3      1   T6P
......................................................................uuuguucguucggcucgCguu.....................   4      1   T6P
......................................................................uuuguucguucgCcucgaguu.....................   8      1   T6P
......................................................................uuuguucguuGgcucgaguu......................   2      1   T6P
......................................................................uuuguucguucggcucgagGu.....................   3      1   T6P
......................................................................uuuguucguucUgcucgaguu.....................   16     1   T6P
......................................................................uuuguucguAcggcucgaguu.....................   5      1   T6P
......................................................................uuuguucguucggcucAaguu.....................   2      1   T6P
......................................................................uuugAucguucggcucgaguu.....................   1      1   T6P
......................................................................uuugCucguucggcucgaguu.....................   2      1   T6P
......................................................................uuuguucguucggcucgUguu.....................   14     1   T6P
......................................................................uuuguucguucgUcucgaguu.....................   6      1   T6P
......................................................................uuGguucguucggcucgaguu.....................   2      1   T6P
......................................................................uuuguucguucggcucgaguA.....................   105    1   T6P
......................................................................uuuguucguucAgcucgaguu.....................   3      1   T6P
......................................................................uuuguucguucggcuUgaguu.....................   14     1   T6P
......................................................................uuuguucguucggcucUaguu.....................   9      1   T6P
......................................................................uuuguucguucggcucgagCu.....................   5      1   T6P
......................................................................uuuUuucguucggcucgaguu.....................   8      1   T6P
......................................................................uuuguucgGucggcucgaguu.....................   2      1   T6P
......................................................................uuuguucguucggcucgaguu.....................   20844  0   T6P
......................................................................uuCguucguucggcucgaguu.....................   6      1   T6P
......................................................................Auuguucguucggcucgaguu.....................   4      1   T6P
......................................................................uuuguucguucgAcucgaguu.....................   24     1   T6P
......................................................................uuuguucgCucggcucgaguu.....................   5      1   T6P
......................................................................uuuguucguucggAucgaguu.....................   3      1   T6P
......................................................................uuuguucCuucggcucgaguu.....................   1      1   T6P
......................................................................uuuguucguucCgcucgaguu.....................   4      1   T6P
......................................................................uuuguucAuucggcucgaguu.....................   2      1   T6P
......................................................................uuuguucguucggcuGgaguu.....................   1      1   T6P
......................................................................uuuguuAguucggcucgaguu.....................   4      1   T6P
......................................................................uuuguucguucggUucgaguu.....................   17     1   T6P
......................................................................uuuguucguucggcCcgaguu.....................   2      1   T6P
......................................................................uuuguucguucggcucgaCuu.....................   5      1   T6P
......................................................................uuuguCcguucggcucgaguu.....................   3      1   T6P
......................................................................uuuguucUuucggcucgaguu.....................   5      1   T6P
......................................................................uuuguucguucggcAcgaguu.....................   1      1   T6P
......................................................................uuugAucguucggcucgaguua....................   3      1   T6P
......................................................................uuuguucguucggcucgCguua....................   1      1   T6P
......................................................................uuuguucguucggcucgaCuua....................   31     1   T6P
......................................................................uuuguuUguucggcucgaguua....................   8      1   T6P
......................................................................uuuguucguucggcucAaguua....................   4      1   T6P
......................................................................uuCguucguucggcucgaguua....................   4      1   T6P
......................................................................uuuguucguucggcucgGguua....................   3      1   T6P
......................................................................uuuguucguucggcCcgaguua....................   2      1   T6P
......................................................................uuuguucguucggcAcgaguua....................   1      1   T6P
```

Star Mature

```
gaaggauacugucuuuauaagacuagugugguaacuugagcaguacgaucagaacugcuuucaaaacuguuuuguucguucggcucgaguuaucaaguggucaaaaugacuu
......................................................................uuuguucguucggcucgagAua....................	3	1	T6P
......................................................................uuuguucguucgUcucgaguua....................	3	1	T6P
......................................................................uuuguucguucggcucgaAuua....................	2	1	T6P
......................................................................Guuguucguucggcucgaguua....................	4	1	T6P
......................................................................uuuguucguucUgcucgaguua....................	7	1	T6P
......................................................................uuuguucAuucggcucgaguua....................	2	1	T6P
......................................................................uuuguuGguucggcucgaguua....................	6	1	T6P
......................................................................Auuguucguucggcucgaguua....................	17	1	T6P
......................................................................uuuguucguucggcucgagGua....................	3	1	T6P
......................................................................uuuguucguucggcuUgaguua....................	16	1	T6P
......................................................................uuuguucguucCgcucgaguua....................	3	1	T6P
......................................................................uuuguucguucggcucgaguCa....................	4	1	T6P
......................................................................Cuuguucguucggcucgaguua....................	4	1	T6P
......................................................................uuuguucguucggGucgaguua....................	3	1	T6P
......................................................................uuuUuucguucggcucgaguua....................	5	1	T6P
......................................................................uuuguucguucggcucgaguuC....................	633	1	T6P
......................................................................uuuguucguucgCcucgaguua....................	3	1	T6P
......................................................................uuuguucUuucggcucgaguua....................	7	1	T6P
......................................................................uuuguucguucgAcucgaguua....................	21	1	T6P
......................................................................uuuguucguucggcucCaguua....................	7	1	T6P
......................................................................uuuguucgCucggcucgaguua....................	6	1	T6P
......................................................................uuuguucguucggcucgUguua....................	7	1	T6P
......................................................................uuAguucguucggcucgaguua....................	3	1	T6P
......................................................................uuuguucguucggcucgaguuU....................	8807	1	T6P
......................................................................uuuguucguucggcucUaguua....................	12	1	T6P
......................................................................uuugCucguucggcucgaguua....................	2	1	T6P
......................................................................uuugGucguucggcucgaguua....................	1	1	T6P
......................................................................uuuguucguuUggcucgaguua....................	14	1	T6P
......................................................................uuuguucguucAgcucgaguua....................	3	1	T6P
......................................................................uuuguucguucggcucgaguAa....................	194	1	T6P
......................................................................uuuguucguCcggcucgaguua....................	1	1	T6P
......................................................................uuuguucguucggcucgaguua....................	20102	0	T6P
......................................................................uuuguucguucggUucgaguua....................	13	1	T6P
......................................................................uuuguuAguucggcucgaguua....................	8	1	T6P
......................................................................uuuguucguucggcucgagCua....................	2	1	T6P
......................................................................uuuguucguucggcucgaguGa....................	4	1	T6P
......................................................................uuuguucCuucggcucgaguua....................	4	1	T6P
......................................................................uuuguucguucggcucgaguuG....................	104	1	T6P
......................................................................uuuCuucguucggcucgaguua....................	5	1	T6P
......................................................................uuuguucguucggcucgaUuua....................	8	1	T6P
......................................................................uuuguucguucggcGcgaguuau...................	1	1	T6P
......................................................................uuugAucguucggcucgaguuau...................	1	1	T6P
......................................................................uuuguucguucggUucgaguuau...................	1	1	T6P
......................................................................uuuguucguucgCcucgaguuau...................	1	1	T6P
......................................................................uuuguuGguucggcucgaguuau...................	4	1	T6P
......................................................................uuuCuucguucggcucgaguuau...................	1	1	T6P
......................................................................uuuguucguucggcucgaAuuau...................	1	1	T6P
......................................................................uuuguuUguucggcucgaguuau...................	1	1	T6P
......................................................................uuuguucguGcggcucgaguuau...................	1	1	T6P
......................................................................Auuguucguucggcucgaguuau...................	3	1	T6P
......................................................................uuuguucguucggcucgUguuau...................	2	1	T6P
......................................................................uuuguucguucggcucgaguuaA...................	2645	1	T6P
......................................................................uuuguucguucggcucgaguuCu...................	31	1	T6P
......................................................................uuuguucguucggcucgaguuau...................	4637	0	T6P
......................................................................uuuguucCuucggcucgaguuau...................	1	1	T6P
......................................................................uuuguucguuGggcucgaguuau...................	1	1	T6P
......................................................................uuuguucguucggcucgaguuUu...................	313	1	T6P
......................................................................uuuguucguucggcucgaUuuau...................	3	1	T6P
......................................................................uuuguucguucggcucgaguAau...................	5	1	T6P
......................................................................uuuguucguucggcucgaguuaC...................	182	1	T6P
......................................................................uuuUuucguucggcucgaguuau...................	6	1	T6P
......................................................................uuuguucguAcggcucgaguuau...................	2	1	T6P
......................................................................uuuguucguucggcucgaguGau...................	1	1	T6P
......................................................................uuuguucguucggcucgaguuGu...................	5	1	T6P
......................................................................uuuguucUuucggcucgaguuau...................	2	1	T6P
......................................................................uuuguucguuUggcucgaguuau...................	2	1	T6P
......................................................................uuuguucAuucggcucgaguuau...................	2	1	T6P
......................................................................uuuguucguucggcucgaguuaG...................	85	1	T6P
......................................................................uuuguucguucggcAcgaguuau...................	1	1	T6P
......................................................................uuuguucguucggcucgaCuuau...................	2	1	T6P
```

```
                                 Star                                   Mature
gaaggauacugucuuuauaagacuaguguggua acuugagcaguacgaucagaac ugcuuucaaaacugu uuuguucguucggcucgaguua ucaaguggucaaaaugacuu
......................................................................uuuguucguucggcucUaguuau...................   2     1   T6P
......................................................................uuuguucguucggcucgaguCau...................   1     1   T6P
......................................................................uuuguCcguucggcucgaguuau...................   1     1   T6P
......................................................................uuuguucguucggcucgagGuau...................   1     1   T6P
......................................................................uuuguuAguucggcucgaguuau...................   2     1   T6P
......................................................................uuuguucguucggcuUgaguuau...................   4     1   T6P
......................................................................uuuguucguucggcucCaguuau...................   1     1   T6P
......................................................................uuuguucguucgAcucgaguuau...................   5     1   T6P
......................................................................uuuguucguucggcucgagCuau...................   1     1   T6P
......................................................................uuuguucguucggcucgaguuAc...................   4     1   T6P
......................................................................uuuguucguucggcucgaguuauA..................   16    1   T6P
......................................................................uuuguucguucggcucgaguuauc..................   8     0   T6P
......................................................................uuuguucguucggcucgaguuauG..................   4     1   T6P
......................................................................uuuguucguucggcucgaguuauU..................   153   1   T6P
......................................................................uuuguucguucggcucgaguuauAa.................   1     1   T6P
......................................................................uuuguucguucggcucgaguuauUa.................   1     1   T6P
......................................................................uuuguucguucggcucgaguuaGca.................   1     1   T6P
......................................................................uuuguucguucggcucgaguuaucU.................   1     1   T6P
.......................................................................uuguucguucggcucgagu......................   11    0   T6P
.......................................................................uuCuucguucggcucgagu......................   1     1   T6P
.......................................................................uuUuucguucggcucgagu......................   2     1   T6P
.......................................................................uuguucguucggcucgaguu.....................   110   0   T6P
.......................................................................uuUuucguucggcucgaguu.....................   4     1   T6P
.......................................................................Auguucguucggcucgaguu.....................   2     1   T6P
.......................................................................uuCuucguucggcucgaguu.....................   2     1   T6P
.......................................................................uuguucguucggcucgagAu.....................   1     1   T6P
.......................................................................uuguucguucggcucgaguAa....................   1     1   T6P
.......................................................................uuguucguucggcucgaguuC....................   8     1   T6P
.......................................................................uuguucguucggcucgaguua....................   143   0   T6P
.......................................................................uuUuucguucggcucgaguua....................   1     1   T6P
.......................................................................uuguucguucggcucgaguuG....................   1     1   T6P
.......................................................................uuguucguucggcucgagCua....................   1     1   T6P
.......................................................................uuguucguucggcucgaguuU....................   50    1   T6P
.......................................................................uuguucguucggcucgaguuaA...................   14    1   T6P
.......................................................................uuguucguucggcucgaguuau...................   32    0   T6P
.......................................................................uuUuucguucggcucgaguuau...................   3     1   T6P
.......................................................................uuguucguucggcucgaguuaC...................   4     1   T6P
........................................................................uguucguucggcucgagu......................   6     0   T6P
........................................................................uguucguucgCcucgagu......................   1     1   T6P
........................................................................uguucguucggcucgaguu.....................   14    0   T6P
........................................................................uUuucguucggcucgaguu.....................   4     1   T6P
........................................................................uguucguucggcucgaguuU....................   3     1   T6P
........................................................................uguucguucggcucgaguuC....................   1     1   T6P
........................................................................uguucguucggcucgaguua....................   15    0   T6P
........................................................................uguucguucggcucgaUuua....................   1     1   T6P
........................................................................uUuucguucggcucgaguuau...................   3     1   T6P
........................................................................uguucguucggcucgaguuaA...................   2     1   T6P
........................................................................uCuucguucggcucgaguuau...................   1     1   T6P
........................................................................uguucguucggcucgaguuau...................   2     0   T6P
........................................................................uguucguucggcucgaguuUu...................   1     1   T6P
.........................................................................guucguucggcucgaguu.....................   1     0   T6P
.........................................................................guucguucggcucgaguuU....................   3     1   T6P
.........................................................................guucguucggcucgaguua....................   5     0   T6P
.........................................................................Uuucguucggcucgaguua....................   1     1   T6P
.........................................................................guucguucggcucgaguuau...................   4     0   T6P
.........................................................................Uuucguucggcucgaguuau...................   1     1   T6P
.........................................................................guucguucggcucgaguuaA...................   2     1   T6P
..........................................................................uucguucggcucgaguuau...................   1     0   T6P
...........................................................................ucguucggcucgaguuau...................   2     0   T6P

............cuuuauaagacuaguguggu................................................................................   1     0   T53
............cuuuauaagacuaguguggua...............................................................................   23    0   T53
.............uuuauaagacuaguguggua...............................................................................   28    0   T53
..............uuauaagacuaguguggua...............................................................................   3     0   T53
................................aacuugagcaguacgaucaga...........................................................   1     0   T53
.................................acuugagcaguacgaucU.............................................................   1     1   T53
.................................acuugagcaguacgaucag............................................................   1     0   T53
.................................acuugagcaguacgaucaga...........................................................   17    0   T53
.................................acuugagcaguacgaucagaa..........................................................   25    0   T53
.................................acuugagcaguacgaucagaac.........................................................   59    0   T53
```

```
                                  Star                                Mature
gaaggauacugucuuuauaagacuaguguggua acuugagcaguacgaucagaac ugcuuucaaaacugu uuuguucguucggcucgaguua ucaaguggucaaaaugacuu
```

```
.................................acuugagcaguacgaucagaaA.........................................................   6     1   T53
.................................acuugagcaguacgaucagaacA........................................................   4     1   T53
.................................acuugagcaguacgaucagaacu........................................................   4     0   T53
.................................acuugagcaguacgaucagaacC........................................................   4     1   T53
..................................cuugagcaguacgaucagaaA.........................................................   1     1   T53
..................................cuugagcaguacgaucagaac.........................................................   4     0   T53
..................................cuugagcaguacgaucagaacu........................................................   1     0   T53
..................................cuugagcaguacgaucagaacuA.......................................................   1     1   T53
...................................uugagcaguacgaucagaac.........................................................   1     0   T53
.................................................................acuguuuuguucguucggcucgag.......................   1     0   T53
.................................................................acuguuuuguucguucggcucgaguuC....................   1     1   T53
..................................................................cuguuuuguucguucggcucgagA......................   1     1   T53
...................................................................uguuuuguucguucggcucgag.......................   1     0   T53
...................................................................Aguuuuguucguucggcucgagu......................   1     1   T53
....................................................................Uuuuuguucguucggcucgagu......................   17    1   T53
....................................................................Uuuuuguucguucggcucgaguu.....................   16    1   T53
....................................................................Cuuuuguucguucggcucgaguu.....................   1     1   T53
....................................................................guuuuguucguucggcucCaguu.....................   1     1   T53
....................................................................Auuuuguucguucggcucgaguu.....................   2     1   T53
....................................................................Uuuuuguucguucggcucgaguua....................   12    1   T53
....................................................................Uuuuuguucguucggcucgaguuau...................   1     1   T53
.....................................................................uuuuguucguucggcucg.........................   44    0   T53
.....................................................................uuuuguucguucggcuGg.........................   1     1   T53
.....................................................................uuuuguucguucggcuUga........................   1     1   T53
.....................................................................uuuuguucguucggcucgU........................   1     1   T53
.....................................................................uuuuguucguuUggcucga........................   1     1   T53
.....................................................................uuuuguucguucggcucga........................   102   0   T53
.....................................................................uuuuguucguucggcucAa........................   1     1   T53
.....................................................................uuuuguucguucggcucgaU.......................   7     1   T53
.....................................................................uuuuguucguucggcucgag.......................   120   0   T53
.....................................................................Auuuguucguucggcucgag.......................   1     1   T53
.....................................................................uuuuCuucguucggcucgag.......................   1     1   T53
.....................................................................uuuuguucguucggcucgaA.......................   5     1   T53
.....................................................................uuuuguucAuucggcucgagu......................   3     1   T53
.....................................................................uuuuguucguucggcucgagu......................   3761  0   T53
.....................................................................uuuuguucguucggcucUagu......................   3     1   T53
.....................................................................uuuuguucguucggcucgagG......................   11    1   T53
.....................................................................uuuuguucguucgUcucgagu......................   4     1   T53
.....................................................................uuuuguucguucgAcucgagu......................   1     1   T53
.....................................................................uuuuguucguucggcucgaAu......................   1     1   T53
.....................................................................uuuuguucguucggcucgagA......................   122   1   T53
.....................................................................uuuugAucguucggcucgagu......................   1     1   T53
.....................................................................uuuuguAcguucggcucgagu......................   2     1   T53
.....................................................................uuuuguucguucggcucgUgu......................   3     1   T53
.....................................................................uuuGguucguucggcucgagu......................   2     1   T53
.....................................................................uuuuguucguucUgcucgagu......................   2     1   T53
.....................................................................uuuuguucguucggcAcgagu......................   2     1   T53
.....................................................................uuuuguucguucgCcucgagu......................   1     1   T53
.....................................................................uuuuUuucguucggcucgagu......................   2     1   T53
.....................................................................uuuuguucguucggcucCagu......................   2     1   T53
.....................................................................uuuuguucguuUggcucgagu......................   2     1   T53
.....................................................................uuuuguucguucggUucgagu......................   3     1   T53
.....................................................................uuuAguucguucggcucgagu......................   1     1   T53
.....................................................................uuuuguuAguucggcucgagu......................   1     1   T53
.....................................................................uuuuguucgCucggcucgagu......................   2     1   T53
.....................................................................uuuuguucguucggcucgaUu......................   4     1   T53
.....................................................................uuuuguucguucggcuGgagu......................   1     1   T53
.....................................................................Cuuuguucguucggcucgagu......................   3     1   T53
.....................................................................Guuuguucguucggcucgagu......................   8     1   T53
.....................................................................uuuuguucguucggcucgGgu......................   1     1   T53
.....................................................................uuuCguucguucggcucgagu......................   1     1   T53
.....................................................................Auuuguucguucggcucgagu......................   9     1   T53
.....................................................................uuuuguucguucggcucgagC......................   5     1   T53
.....................................................................uuuuguucguucggAucgagu......................   1     1   T53
.....................................................................uuuuguucguucggcucgaCu......................   2     1   T53
.....................................................................uuuuguucUuucggcucgaguu.....................   1     1   T53
.....................................................................uuuuguucguucggcucgaguA.....................   66    1   T53
.....................................................................Auuuguucguucggcucgaguu.....................   10    1   T53
.....................................................................uuuuguAcguucggcucgaguu.....................   2     1   T53
.....................................................................uuuCguucguucggcucgaguu.....................   1     1   T53
```

Star Mature

gaaggauacugucuuuauaagacuaguguggua**acuugagcaguacgaucagaac**ugcuuucaaaacugu**uuuguucguucggcucgaguua**ucaaguggucaaaaugacuu			
....uCuuguucguucggcucgaguu.....................	1	1	T53
....Cuuuguucguucggcucgaguu.....................	3	1	T53
....Guuuguucguucggcucgaguu.....................	2	1	T53
....uuuuguucgCucggcucgaguu.....................	2	1	T53
....uuuuguucguucggcucgaUuu.....................	1	1	T53
....uuuuguucguuUggcucgaguu.....................	3	1	T53
....uuuuguucguucgAcucgaguu.....................	6	1	T53
....uuuuguucguucggcucCaguu.....................	1	1	T53
....uuAuguucguucggcucgaguu.....................	1	1	T53
....uuCuguucguucggcucgaguu.....................	1	1	T53
....uuuuguucguucggcucUaguu.....................	3	1	T53
....uuuuguucguucUgcucgaguu.....................	1	1	T53
....uuuuguucguucggUucgaguu.....................	3	1	T53
....uuuuguucguucgUcucgaguu.....................	1	1	T53
....uuuuguucguucgCcucgaguu.....................	1	1	T53
....uuuuguucguucggcAcgaguu.....................	1	1	T53
....uuuuguucguucggcucgagAu.....................	2	1	T53
....uuuuguucguucggcucgaAuu.....................	1	1	T53
....uuuuguucCuucggcucgaguu.....................	1	1	T53
....uuuuguucguucggcuUgaguu.....................	1	1	T53
....uuuuguucguucggcucgaguu.....................	2473	0	T53
....uuuuguucguucggcucgaguG.....................	2	1	T53
....uAuuguucguucggcucgaguu.....................	1	1	T53
....Cuuuguucguucggcucgaguua....................	1	1	T53
....uuuuAuucguucggcucgaguua....................	1	1	T53
....uuuuguucguucggcucgaguuC....................	101	1	T53
....uuuuguucguucggcucgaguuG....................	32	1	T53
....uuuuguucguucggcucgaCuua....................	1	1	T53
....uuuuguucguucggcucgCguua....................	1	1	T53
....uuuuguucguucggcucgaguua....................	638	0	T53
....uuuuguucgCucggcucgaguua....................	1	1	T53
....Auuuguucguucggcucgaguua....................	2	1	T53
....uuuuguucguucgAcucgaguua....................	3	1	T53
....uuuuguucAuucggcucgaguua....................	1	1	T53
....uuuuguucguucggcucgaguAa....................	75	1	T53
....uuuuguucguucggcucgaguuU....................	1446	1	T53
....Guuuguucguucggcucgaguua....................	2	1	T53
....uuuuguucguucggcucgaguuau...................	317	0	T53
....uuuuguucguucggcucgaguuGu...................	2	1	T53
....uuuuguucguucgAcucgaguuau...................	4	1	T53
....uuuuguucguucggcucgaguAau...................	4	1	T53
....uuuuguucguucggcucgaguuaC...................	12	1	T53
....uuuuguucguucggcucgaguuUu...................	117	1	T53
....uuuuguucguucggcucgaguuaG...................	10	1	T53
....uuuuguucguucggUucgaguuau...................	1	1	T53
....uuuuguucguucggcucgaguuaA...................	266	1	T53
....uuuugGucguucggcucgaguuau...................	1	1	T53
....uuuuguucguucggcucgaguuCu...................	11	1	T53
....uuuuguucguucggcucgaguuauG..................	5	1	T53
....uuuuguucguucggcucgaguuauU..................	41	1	T53
....uuuuguucguucggcucgaguuauA..................	2	1	T53
....uuuuguucguucggcucgaguuaucU.................	3	1	T53
....uuuuguucguucggcucgaguuauAa.................	2	1	T53
....Cuuguucguucggcucga........................	1	1	T53
....uuuguucguucggcucga........................	829	0	T53
....uuuguucguucggcucUa........................	1	1	T53
....uuuguucguucggcucgU........................	7	1	T53
....uGuguucguucggcucga........................	1	1	T53
....uuuguucguucggcAcga........................	1	1	T53
....uuuguucguucAgcucga........................	1	1	T53
....uuuguucguucgAcucga........................	4	1	T53
....Auuguucguucggcucga........................	1	1	T53
....uuuguucguucggAucga........................	1	1	T53
....uuuguuUguucggcucga........................	1	1	T53
....uuuguucguucggcucgaA.......................	60	1	T53
....uuuguucguucggcucgag.......................	561	0	T53
....uuuguucguucggcucgGg.......................	1	1	T53
....uuuguucguucggcucgaC.......................	4	1	T53
....uuuguucAuucggcucgag.......................	1	1	T53
....uCuguucguucggcucgag.......................	1	1	T53

Star Mature

```
gaaggauacugucuuuauaagacuaguguggua acuugagcaguacgaucagaac ugcuuucaaaacugu uuuguucguucggcucgaguua ucaaguggucaaaaugacuu
......................................................................uuuguucguucgAcucgag.......................   1      1   T53
......................................................................uuuguucguucggcucgaU.......................   35     1   T53
......................................................................uuuguucguucggcAcgagu......................   3      1   T53
......................................................................Guuguucguucggcucgagu......................   17     1   T53
......................................................................uuCguucguucggcucgagu......................   3      1   T53
......................................................................uuuguAcguucggcucgagu......................   8      1   T53
......................................................................uuuguucguucggcucgagG......................   91     1   T53
......................................................................uuuguucguucggAucgagu......................   1      1   T53
......................................................................uuuguucguucCgcucgagu......................   6      1   T53
......................................................................uuuguucguuGggcucgagu......................   2      1   T53
......................................................................uuuguucguucggcucUagu......................   2      1   T53
......................................................................uuuguCcguucggcucgagu......................   7      1   T53
......................................................................uuuguucguucggUucgagu......................   34     1   T53
......................................................................uuuguucguucggcuAgagu......................   2      1   T53
......................................................................uuuguuAguucggcucgagu......................   2      1   T53
......................................................................Auuguucguucggcucgagu......................   54     1   T53
......................................................................uuuguucguucggcucgUgu......................   18     1   T53
......................................................................uuuguucguucggcucAagu......................   12     1   T53
......................................................................uuuguucguucAgcucgagu......................   3      1   T53
......................................................................uuuUuucguucggcucgagu......................   5      1   T53
......................................................................uuuguGcguucggcucgagu......................   2      1   T53
......................................................................uuuguucguucgAcucgagu......................   42     1   T53
......................................................................uGuguucguucggcucgagu......................   2      1   T53
......................................................................uuuguucguAcggcucgagu......................   2      1   T53
......................................................................uuuCuucguucggcucgagu......................   1      1   T53
......................................................................uuuguucguucggGucgagu......................   1      1   T53
......................................................................uuuguucguucUgcucgagu......................   2      1   T53
......................................................................uuuguucguucggcucgaCu......................   13     1   T53
......................................................................uuuguucguucggcucgCgu......................   19     1   T53
......................................................................uuuguucguuUggcucgagu......................   26     1   T53
......................................................................uuuguucgGucggcucgagu......................   3      1   T53
......................................................................uuuguucUuucggcucgagu......................   4      1   T53
......................................................................uuuguucgCucggcucgagu......................   5      1   T53
......................................................................uuuguucguucggcucgagu......................   41660  0   T53
......................................................................uuuguucguucggcucgaAu......................   41     1   T53
......................................................................uuuguucguucggcucgGgu......................   11     1   T53
......................................................................Cuuguucguucggcucgagu......................   16     1   T53
......................................................................uuugCucguucggcucgagu......................   2      1   T53
......................................................................uuugGucguucggcucgagu......................   1      1   T53
......................................................................uuuguucguucggcucgaUu......................   45     1   T53
......................................................................uuuguucguucggcucgagA......................   1207   1   T53
......................................................................uuuguucguucgCcucgagu......................   6      1   T53
......................................................................uuuguucguucggcCcgagu......................   1      1   T53
......................................................................uuuguucguucggcuUgagu......................   15     1   T53
......................................................................uuugAucguucggcucgagu......................   3      1   T53
......................................................................uuuguucguCcggcucgagu......................   20     1   T53
......................................................................uuuguucguucggcuGgagu......................   1      1   T53
......................................................................uuuguucCuucggcucgagu......................   4      1   T53
......................................................................uAuguucguucggcucgagu......................   2      1   T53
......................................................................uCuguucguucggcucgagu......................   5      1   T53
......................................................................uuuguuUguucggcucgagu......................   18     1   T53
......................................................................uuuguucgAucggcucgagu......................   17     1   T53
......................................................................uuuguucguucgUcucgagu......................   8      1   T53
......................................................................uuAguucguucggcucgagu......................   3      1   T53
......................................................................uuuguucguucggcucgagC......................   38     1   T53
......................................................................uuuguucAuucggcucgagu......................   10     1   T53
......................................................................uuGguucguucggcucgagu......................   1      1   T53
......................................................................uuuCuucguucggcucgaguu.....................   4      1   T53
......................................................................uuuguucguucggcCcgaguu.....................   5      1   T53
......................................................................uuCguucguucggcucgaguu.....................   2      1   T53
......................................................................uuuguucguucggcucgagGu.....................   2      1   T53
......................................................................uuuguucguucggcucgaguA.....................   1103   1   T53
......................................................................uuuguucguucAgcucgaguu.....................   8      1   T53
......................................................................uuuguucguucggcuGgaguu.....................   6      1   T53
......................................................................uAuguucguucggcucgaguu.....................   3      1   T53
......................................................................Auuguucguucggcucgaguu.....................   91     1   T53
......................................................................uuuguucguucggcuAgaguu.....................   6      1   T53
......................................................................uuuguucguAcggcucgaguu.....................   2      1   T53
......................................................................uuuguucguucggcucgaguC.....................   18     1   T53
......................................................................uuuguucguucggcuUgaguu.....................   31     1   T53
```

Star / Mature			
gaaggauacugucuuuauaagacuagugugguaacuugagcaguacgaucagaacugcuuucaaaacuguuuuguucguucggcucgaguuaucaaguggucaaaaugacuu			
....uuuguucguuUggcucgaguu.....................	47	1	T53
....uuuguucguucggcAcgaguu.....................	7	1	T53
....Cuuguucguucggcucgaguu.....................	22	1	T53
....uuuguucguucgCcucgaguu.....................	6	1	T53
....uuuguucgAucggcucgaguu.....................	36	1	T53
....uuuguucguGcggcucgaguu.....................	1	1	T53
....uuuguucUuucggcucgaguu.....................	9	1	T53
....uuuAuucguucggcucgaguu.....................	4	1	T53
....uuuguucguucggcucUaguu.....................	11	1	T53
....uuuguucguucggcucgaguG.....................	19	1	T53
....uuuguucguucggcucAaguu.....................	9	1	T53
....uuuguucguucgUcucgaguu.....................	10	1	T53
....uuuguucguucggcucCaguu.....................	9	1	T53
....uuuguucAuucggcucgaguu.....................	6	1	T53
....uuuguucguucggcucgaAuu.....................	31	1	T53
....uuuguucguucggUucgaguu.....................	19	1	T53
....uuuguucCuucggcucgaguu.....................	4	1	T53
....uuugCucguucggcucgaguu.....................	1	1	T53
....uuuguucguuAggcucgaguu.....................	1	1	T53
....uuuguucguCcggcucgaguu.....................	17	1	T53
....uuuguucguucggcucgagCu.....................	8	1	T53
....Guuguucguucggcucgaguu.....................	23	1	T53
....uuuguAcguucggcucgaguu.....................	11	1	T53
....uuuguuGguucggcucgaguu.....................	1	1	T53
....uuAguucguucggcucgaguu.....................	6	1	T53
....uuuUuucguucggcucgaguu.....................	12	1	T53
....uCuguucguucggcucgaguu.....................	6	1	T53
....uuuguucguucggcGcgaguu.....................	1	1	T53
....uuuguuAguucggcucgaguu.....................	4	1	T53
....uuuguucguucUgcucgaguu.....................	11	1	T53
....uuuguucguucggcucgaCuu.....................	15	1	T53
....uuuguucguucggcucgCguu.....................	109	1	T53
....uuuguucguucggcucgaUuu.....................	12	1	T53
....uuuguucguucCgcucgaguu.....................	2	1	T53
....uuuguuUguucggcucgaguu.....................	22	1	T53
....uuuguucguucggGucgaguu.....................	5	1	T53
....uuuguucguucgAcucgaguu.....................	81	1	T53
....uuuguucguucggcucgUguu.....................	28	1	T53
....uuuguGcguucggcucgaguu.....................	6	1	T53
....uuuguucgCucggcucgaguu.....................	10	1	T53
....uuuguucgGucggcucgaguu.....................	2	1	T53
....uGuguucguucggcucgaguu.....................	4	1	T53
....uuuguucguucggcucgagAu.....................	25	1	T53
....uuugGucguucggcucgaguu.....................	1	1	T53
....uuugAucguucggcucgaguu.....................	1	1	T53
....uuuguCcguucggcucgaguu.....................	6	1	T53
....uuuguucguucggcucgGguu.....................	7	1	T53
....uuuguucguucggcucgaguu.....................	52505	0	T53
....uuuguucguucggcuGgaguua....................	5	1	T53
....uuuguucguuGggcucgaguua....................	1	1	T53
....uuuguucguucgAcucgaguua....................	85	1	T53
....uuCguucguucggcucgaguua....................	3	1	T53
....uuuguucgGucggcucgaguua....................	2	1	T53
....uuuguucAuucggcucgaguua....................	3	1	T53
....uuuguucguucgCcucgaguua....................	2	1	T53
....uuuguucguucggcucCaguua....................	2	1	T53
....uuuguucguucgUcucgaguua....................	4	1	T53
....uuuguucguucggcucgUguua....................	20	1	T53
....uuuguucguucggcucUaguua....................	7	1	T53
....uuuguucguucggcucgGguua....................	12	1	T53
....uuuguucguucggcucgaguGa....................	10	1	T53
....uuuUuucguucggcucgaguua....................	4	1	T53
....uuAguucguucggcucgaguua....................	6	1	T53
....uuuguucguucggcucgagCua....................	1	1	T53
....uuugCucguucggcucgaguua....................	4	1	T53
....Auuguucguucggcucgaguua....................	64	1	T53
....uuuguAcguucggcucgaguua....................	2	1	T53
....uuuguucguucggUucgaguua....................	20	1	T53
....uuuguucCuucggcucgaguua....................	2	1	T53
....uuuguucguucAgcucgaguua....................	4	1	T53

Star **Mature**

gaaggauacugucuuuauaagacuaguguggua**acuugagcaguacgaucagaac**ugcuuucaaaacugu**uuuguucguucggcucgaguua**ucaagugguucaaaaugacuu			
....uuuguucguucggcAcgaguua....................	8	1	T53
....uuuguuAguucggcucgaguua....................	4	1	T53
....uuuguucguucggcGcgaguua....................	1	1	T53
....uuuguucguAcggcucgaguua....................	1	1	T53
....uGuguucguucggcucgaguua....................	4	1	T53
....uuuguucgCucggcucgaguua....................	3	1	T53
....Guuguucguucggcucgaguua....................	25	1	T53
....Cuuguucguucggcucgaguua....................	31	1	T53
....uuuguucguucggcucgagAua....................	19	1	T53
....uuuguucguuUggcucgaguua....................	61	1	T53
....uuuguucguucggcucgaCuua....................	7	1	T53
....uuuguucguucggcuUgaguua....................	15	1	T53
....uuuguucguucggcucgaguuG....................	714	1	T53
....uuuguucUuucggcucgaguua....................	7	1	T53
....uuugAucguucggcucgaguua....................	3	1	T53
....uuuguucguucggcucgaguuC....................	2809	1	T53
....uuuguucguucggcucgaguCa....................	14	1	T53
....uuuguucguucCgcucgaguua....................	5	1	T53
....uuuguucguucggcucgaAuua....................	5	1	T53
....uuuguucguucggcucgaguAa....................	1754	1	T53
....uuuguucguucUgcucgaguua....................	5	1	T53
....uuuguucguucggcucgagGua....................	2	1	T53
....uAuguucguucggcucgaguua....................	1	1	T53
....uuuAuucguucggcucgaguua....................	5	1	T53
....uuuguuUguucggcucgaguua....................	5	1	T53
....uuuguucguucggcucgaguua....................	39142	0	T53
....uuuguucguucggcucgaguuU....................	28913	1	T53
....uuuCuucguucggcucgaguua....................	1	1	T53
....uuuguucguucggcucgaUuua....................	9	1	T53
....uuuguucguucggcucAaguua....................	13	1	T53
....uuuguucguucggcCcgaguua....................	6	1	T53
....uuuguucguucggcuAgaguua....................	1	1	T53
....uuuguucguucggcucgCguua....................	275	1	T53
....uuugGucguucggcucgaguua....................	2	1	T53
....uuuguucguCcggcucgaguua....................	15	1	T53
....uuuguucguucggAucgaguua....................	2	1	T53
....uuuguGcguucggcucgaguua....................	4	1	T53
....uuuguucgAucggcucgaguua....................	33	1	T53
....uuuguCcguucggcucgaguua....................	3	1	T53
....uuuguucgAucggcucgaguuau...................	7	1	T53
....uuuguucguucggcucgUguuau...................	2	1	T53
....uuuguucguCcggcucgaguuau...................	5	1	T53
....uuuguucguucggcuUgaguuau...................	4	1	T53
....uuuguAcguucggcucgaguuau...................	1	1	T53
....uuuguucguucggcucgGguuau...................	2	1	T53
....uuuguucguucggcCcgaguuau...................	7	1	T53
....uuuguucguucggcucgaguuaA...................	12124	1	T53
....Guuguucguucggcucgaguuau...................	8	1	T53
....uuuguucguucggcucUaguuau...................	2	1	T53
....uuuguucgGucggcucgaguuau...................	1	1	T53
....uuuguucguucggcucgaguuCu...................	350	1	T53
....uuuguucguucggcucgaAuuau...................	2	1	T53
....uuuguucguucCgcucgaguuau...................	1	1	T53
....uuuguucguucggcucgaguuau...................	12825	0	T53
....uuuguucguucggcucgCguuau...................	50	1	T53
....uuuguuUguucggcucgaguuau...................	4	1	T53
....uuAguucguucggcucgaguuau...................	3	1	T53
....uuuguucguucggUucgaguuau...................	7	1	T53
....uuuguucAuucggcucgaguuau...................	1	1	T53
....uuuguucguucggcucgaguuaG...................	1339	1	T53
....uuuguucguucggcucgaUuuau...................	4	1	T53
....uuuguucguucggcucgaguuaC...................	798	1	T53
....Cuuguucguucggcucgaguuau...................	7	1	T53
....uuuguucguucggcucgagAuau...................	6	1	T53
....uuuguucguucggcucgaguAau...................	38	1	T53
....uuuguucguucggcucgaguuGu...................	14	1	T53
....uuuguucguuUggcucgaguuau...................	29	1	T53
....uuuguucCuucggcucgaguuau...................	1	1	T53
....uuuguucUuucggcucgaguuau...................	3	1	T53
....uuuguucguucggcucAaguuau...................	1	1	T53

Star **Mature**

```
gaaggauacugucuuuauaagacuaguguggua acuugagcaguacgaucagaac ugcuuucaaaacugu uuuguucguucggcucgaguua ucaaguggucaaaaugacuu
......................................................................uuuUuucguucggcucgaguuau...................	1	1	T53
......................................................................uuuguucguucggcAcgaguuau...................	7	1	T53
......................................................................uuuguucguucggcucgaguGau...................	2	1	T53
......................................................................uuuguucguucggcuGgaguuau...................	4	1	T53
......................................................................uuugCucguucggcucgaguuau...................	4	1	T53
......................................................................uuuguucguucggcucgagGuau...................	1	1	T53
......................................................................uuuguucguucggcucgaCuuau...................	6	1	T53
......................................................................uuuguucguucggcucgaguCau...................	3	1	T53
......................................................................Auuguucguucggcucgaguuau...................	13	1	T53
......................................................................uuuguucgCucggcucgaguuau...................	1	1	T53
......................................................................uuuguucguucggcucCaguuau...................	4	1	T53
......................................................................uuuguGcguucggcucgaguuau...................	4	1	T53
......................................................................uuuguucguucggcucgaguuUu...................	1266	1	T53
......................................................................uuuguCcguucggcucgaguuau...................	3	1	T53
......................................................................uuuguucguucgUcucgaguuau...................	3	1	T53
......................................................................uuuguucguAcggcucgaguuau...................	1	1	T53
......................................................................uuuguucguucgAcucgaguuau...................	19	1	T53
......................................................................uuuguucguucggcucgaguuUuc..................	1	1	T53
......................................................................uuuguucguucggcucgaguuGuc..................	1	1	T53
......................................................................uuuguucguucggcucgaguuauU..................	473	1	T53
......................................................................uuuguucguucggcucgaguuauA..................	87	1	T53
......................................................................uuuguucguucggcucgaguuaGc..................	1	1	T53
......................................................................uuuguucguucggcucgaguuaCc..................	2	1	T53
......................................................................uuuguucguucggcucgaguuauc..................	16	0	T53
......................................................................uuuguucguucggcucgaguuauG..................	15	1	T53
......................................................................uuuguucguucggcucgaguuaAc..................	78	1	T53
......................................................................uuuguucguucggcucgaguuauUa.................	2	1	T53
......................................................................uuuguucguucggcucgaguuaucU.................	9	1	T53
......................................................................uuuguucguucggcucgaguuauAa.................	1	1	T53
......................................................................uuuguucguucggcucgaguuauca.................	2	0	T53
......................................................................uuuguucguucggcucgaguuaAca.................	10	1	T53
......................................................................uuuguucguucggcucgaguuauUaa................	1	1	T53
.......................................................................uuguucguucggcucgag.......................	1	0	T53
.......................................................................Auguucguucggcucgagu......................	1	1	T53
.......................................................................uuguucguucggcucgUgu......................	1	1	T53
.......................................................................uuguucguucggcucgagu......................	163	0	T53
.......................................................................uuguucguucggcucgagG......................	1	1	T53
.......................................................................uuguucguucggcucgagA......................	4	1	T53
.......................................................................uuguucguucggcucgaguG.....................	1	1	T53
.......................................................................uuguucguucggcucgaguA.....................	3	1	T53
.......................................................................uuUuucguucggcucgaguu.....................	1	1	T53
.......................................................................uuguucguucggcucgCguu.....................	1	1	T53
.......................................................................uuguucguucggcuUgaguu.....................	1	1	T53
.......................................................................Auguucguucggcucgaguu.....................	5	1	T53
.......................................................................uuguucguucggcucgaguu.....................	252	0	T53
.......................................................................uuguucguucggUucgaguua....................	1	1	T53
.......................................................................uuguucgAucggcucgaguua....................	1	1	T53
.......................................................................Guguucguucggcucgaguua....................	1	1	T53
.......................................................................uuguucguucggcucgaguAa....................	3	1	T53
.......................................................................uuguucguucggcucgUguua....................	1	1	T53
.......................................................................Cuguucguucggcucgaguua....................	1	1	T53
.......................................................................uuguGcguucggcucgaguua....................	1	1	T53
.......................................................................uuUuucguucggcucgaguua....................	4	1	T53
.......................................................................uuguucguucggcucgaguuG....................	2	1	T53
.......................................................................Auguucguucggcucgaguua....................	1	1	T53
.......................................................................uuguucguucggcucgaguua....................	554	0	T53
.......................................................................uuguucguucggcucgGguua....................	1	1	T53
.......................................................................uuguucguuUggcucgaguua....................	1	1	T53
.......................................................................uuguucguucggcucgaguuC....................	35	1	T53
.......................................................................uuguucguucggcucgaguuU....................	139	1	T53
.......................................................................uuguucguucggcucgaguuCu...................	1	1	T53
.......................................................................uuguucguucggcucgaguuUu...................	3	1	T53
.......................................................................uuguucguucggcucgaguuau...................	84	0	T53
.......................................................................uuguucguucggcucgaguuaG...................	2	1	T53
.......................................................................uuguucguuUggcucgaguuau...................	1	1	T53
.......................................................................uuUuucguucggcucgaguuau...................	1	1	T53
.......................................................................uuguucguucggcucgaguuaA...................	54	1	T53
.......................................................................Auguucguucggcucgaguuau...................	2	1	T53
.......................................................................uuguucguucggcucgaguuaC...................	13	1	T53
.......................................................................uuguucguucggcucgaguuaCc..................	2	1	T53
```

```
                                 Star                                 Mature
gaaggauacugucuuuauaagacuaguguggua acuugagcaguacgaucagaac ugcuuucaaaacugu uuuguucguucggcucgaguua ucaaguggucaaaaugacuu
```

Star: acuugagcaguacgaucagaac — Mature: uuuguucguucggcucgaguua

```
gaaggauacugucuuuauaagacuaguguggua acuugagcaguacgaucagaacugcuuucaaaacuguuuuguucguucggcucgaguuaucaaguggucaaaaugacuu
.......................................................................uuguucguucggcucgaguuauU..................   4   1   T53
........................................................................uguucguAcggcucgagu......................   1   1   T53
........................................................................uCuucguucggcucgagu......................   1   1   T53
........................................................................uUuucguucggcucgagu......................   2   1   T53
........................................................................uguucguucggcucgagu......................   10  0   T53
........................................................................uUuucguucggcucgaguu.....................   2   1   T53
........................................................................uguucguucggcucgaguu.....................   12  0   T53
........................................................................uguucguucggcucgaguuU....................   12  1   T53
........................................................................uguucguucggcucgaguua....................   14  0   T53
........................................................................uUuucguucggcucgaguua....................   3   1   T53
........................................................................uguucguucggcucgaguuaA...................   5   1   T53
........................................................................uguucguucggcucgaguuaC...................   1   1   T53
........................................................................uguucguucggcucgaguuau...................   4   0   T53
........................................................................uUuucguucggcucgaguuau...................   2   1   T53
........................................................................uguucguucggcucgaguuauU..................   1   1   T53
.........................................................................guucguucgAcucgaguu.....................   1   1   T53
.........................................................................guucguucggcucgaguu.....................   4   0   T53
.........................................................................guucguucggcucgaguuC....................   1   1   T53
.........................................................................guucguucggcucgaguuU....................   4   1   T53
.........................................................................guucguucggcucgCguua....................   2   1   T53
.........................................................................guucguucggcucgaguAa....................   1   1   T53
.........................................................................Uuucguucggcucgaguua....................   3   1   T53
.........................................................................guucguucggcucgaguua....................   3   0   T53
.........................................................................guucguucggcucgaguuau...................   1   0   T53
.........................................................................Uuucguucggcucgaguuau...................   2   1   T53
.........................................................................guucguucggcucgaguuaA...................   1   1   T53
..........................................................................uucguucggcucgaguua....................   1   0   T53
..........................................................................uucguucggcucgaguuU....................   1   1   T53
..........................................................................uucguucggcucgaguuUu...................   1   1   T53
..........................................................................uucguucggcucgaguuauA..................   1   1   T53
..........................................................................uucguucggcucgaguuauca.................   1   0   T53
```

Provisional ID	:	Scaffold_1043_2937
Score total	:	1648663.3
Score for star read(s)	:	3.9
Score for read counts	:	1648653.2
Score for mfe	:	1.5
Score for randfold	:	1.6
Score for cons. seed	:	3
Total read count	:	3233774
Mature read count	:	3165464
Loop read count	:	1
Star read count	:	68309

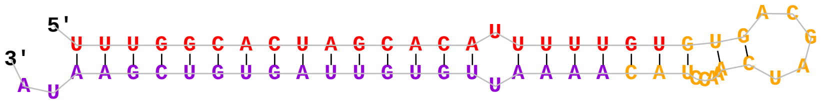

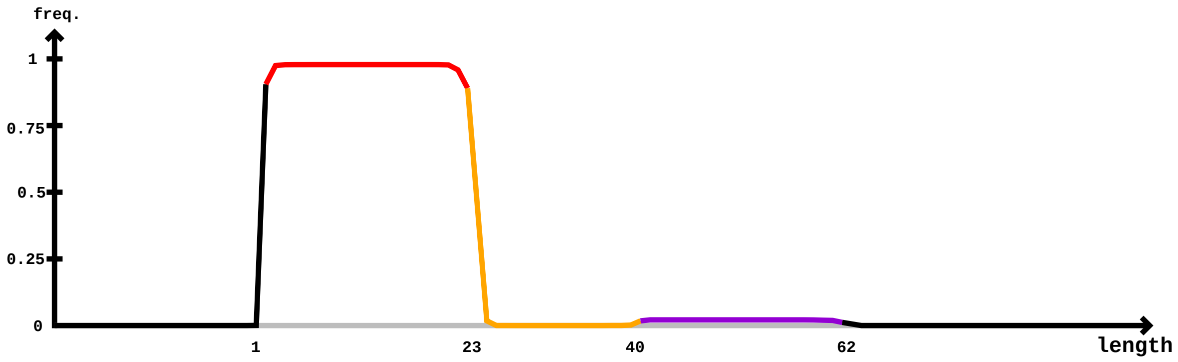

Mature Star

```
5'- gcuaguccucuucucuguucuuuggcacuagcacauuuuuguggugacgaucaaaccuacaaaauuguguuagugucgaauaauaugggaagacauucgucauaaaucauaca   -3'   obs
    gcuaguccucuucucuguucuuuggcacuagcacauuuuuguggugacgaucaaaccuacaaaauuguguuagugucgaauaauaugggaagacauucgucauaaaucauaca         exp
    ........((((((((((.((((((((((((((.(((((((((.....))....))))))).)))))))))))))).)))).))))))).......................   reads   mm   sample
    .................uuUuuuggcacuagcacauuuuugu......................................................................   1       1    T63
    ..................uUuuuggcacuagcacauuuuugu......................................................................   23      1    T63
    ...................Uuuuggcacuagcacauuu..........................................................................   1       1    T63
    ...................Uuuuggcacuagcacauuuuu........................................................................   8       1    T63
    ...................cuuuggcacuagcacauuuuu........................................................................   9       0    T63
    ...................Uuuuggcacuagcacauuuuug.......................................................................   6       1    T63
    ...................cuuuggcacuagcacauuuuug.......................................................................   2       0    T63
    ...................cuuuggcacuagcacauuuuugu......................................................................   33      0    T63
    ...................Auuuggcacuagcacauuuuugu......................................................................   15      1    T63
    ...................Uuuuggcacuagcacauuuuugu......................................................................   279     1    T63
    ...................Guuuggcacuagcacauuuuugu......................................................................   1       1    T63
    ...................cuuuggcacuagcacaAuuuugu......................................................................   1       1    T63
    ....................uuuggcacuagcacauuu..........................................................................   46      0    T63
    ....................uuuggcacuagcacauGu..........................................................................   4       1    T63
    ....................uuuggcacuagcacauuA..........................................................................   2       1    T63
    ....................uuuggcacuagcaUauuu..........................................................................   1       1    T63
    ....................uuuggcacuagcacauuG..........................................................................   1       1    T63
    ....................uuuggcacuagcacauAu..........................................................................   1       1    T63
    ....................uuuggcacuaAcacauuuu.........................................................................   3       1    T63
    ....................uuuggcacuagcacauuAu.........................................................................   1       1    T63
    ....................uuuggUacuagcacauuuu.........................................................................   3       1    T63
    ....................uuuggcacuagcacUuuuu.........................................................................   2       1    T63
    ....................Cuuggcacuagcacauuuu.........................................................................   1       1    T63
    ....................uuuggcacuagcacauuuu.........................................................................   994     0    T63
    ....................Guuggcacuagcacauuuu.........................................................................   1       1    T63
    ....................uuugCcacuagcacauuuu.........................................................................   1       1    T63
    ....................uuuCgcacuagcacauuuu.........................................................................   1       1    T63
    ....................uuuggcacuagcacauuuG.........................................................................   11      1    T63
    ....................uuuggcacuCgcacauuuu.........................................................................   1       1    T63
    ....................uuuggcacuagcacauuGu.........................................................................   8       1    T63
    ....................uCuggcacuagcacauuuu.........................................................................   1       1    T63
    ....................uuuggcacuagcacauuuA.........................................................................   4       1    T63
    ....................uuugAcacuagcacauuuu.........................................................................   1       1    T63
```

```
                    Mature                   Star
gcuaguccucuucucuguucuuuggcacuagcacauuuuugugugacgaucaaaccuacaaaauuguguuagugucgaauaauaugggaagacauucgucauaaaucauaca
....................uAuggcacuagcacauuuu.........................................................................   1       1   T63
....................Auuggcacuagcacauuuu.........................................................................   1       1   T63
....................uuuggcacuagcaAauuuu.........................................................................   1       1   T63
....................uuuggcacuagcacauCuuu........................................................................   3       1   T63
....................uuuggcacuGgcacauuuuu........................................................................   1       1   T63
....................uuuggAacuagcacauuuuu........................................................................   1       1   T63
....................uuuggGacuagcacauuuuu........................................................................   1       1   T63
....................uuuggcacuagcacauuuuA........................................................................   13      1   T63
....................uuuggcacuagcUcauuuuu........................................................................   2       1   T63
....................uCuggcacuagcacauuuuu........................................................................   2       1   T63
....................uuuggcacuagcacauuuGu........................................................................   61      1   T63
....................uuuggcacuagcacaAuuuu........................................................................   15      1   T63
....................uuuggcacuagcacauuuAu........................................................................   1       1   T63
....................Auuggcacuagcacauuuuu........................................................................   10      1   T63
....................uuuggcacuagcacauAuuu........................................................................   3       1   T63
....................uuuUgcacuagcacauuuuu........................................................................   7       1   T63
....................uuuggcaAuagcacauuuuu........................................................................   2       1   T63
....................uuugAcacuagcacauuuuu........................................................................   8       1   T63
....................uuuggcacuagcacauuuuG........................................................................   12      1   T63
....................uuAggcacuagcacauuuuu........................................................................   4       1   T63
....................uuuggcacuagcacUuuuuu........................................................................   8       1   T63
....................uuuggcUcuagcacauuuuu........................................................................   3       1   T63
....................uuugUcacuagcacauuuuu........................................................................   1       1   T63
....................uuuggcacuagcGcauuuuu........................................................................   1       1   T63
....................Cuuggcacuagcacauuuuu........................................................................   5       1   T63
....................uuuggcacuagUacauuuuu........................................................................   7       1   T63
....................uuuggcacuaCcacauuuuu........................................................................   1       1   T63
....................uuuggcGcuagcacauuuuu........................................................................   1       1   T63
....................uuuggcacuagcacauuGuu........................................................................   4       1   T63
....................uuuggcacuagGacauuuuu........................................................................   5       1   T63
....................uuCggcacuagcacauuuuu........................................................................   3       1   T63
....................uuuggcacuaAcacauuuuu........................................................................   19      1   T63
....................Guuggcacuagcacauuuuu........................................................................   1       1   T63
....................uuuggcacuagcacauuuuC........................................................................   1       1   T63
....................uuuggcacuCgcacauuuuu........................................................................   2       1   T63
....................uuuCgcacuagcacauuuuu........................................................................   2       1   T63
....................uuuggcacuagcacauuuuu........................................................................   12748   0   T63
....................uuuggcacuagcacauuCuu........................................................................   1       1   T63
....................uuuggcaUuagcacauuuuu........................................................................   3       1   T63
....................uuuggcacAagcacauuuuu........................................................................   6       1   T63
....................uuGggcacuagcacauuuuu........................................................................   2       1   T63
....................uuuggUacuagcacauuuuu........................................................................   34      1   T63
....................uuugCcacuagcacauuuuu........................................................................   1       1   T63
....................uuuggcacuagcacauuAuu........................................................................   3       1   T63
....................uuuggcacuagcaUauuuuu........................................................................   12      1   T63
....................uuuggcacuUgcacauuuuug.......................................................................   3       1   T63
....................uuuggcacuagUacauuuuug.......................................................................   10      1   T63
....................uuuggcacuCgcacauuuuug.......................................................................   10      1   T63
....................uuuggcacCagcacauuuuug.......................................................................   2       1   T63
....................uuCggcacuagcacauuuuug.......................................................................   3       1   T63
....................uuuggcacuagcacauCuuug.......................................................................   6       1   T63
....................uuuggcaAuagcacauuuuug.......................................................................   3       1   T63
....................uAuggcacuagcacauuuuug.......................................................................   2       1   T63
....................uuuggcacuagcacauAuuug.......................................................................   25      1   T63
....................Guuggcacuagcacauuuuug.......................................................................   6       1   T63
....................uuuggcacAagcacauuuuug.......................................................................   14      1   T63
....................uuuggcaUuagcacauuuuug.......................................................................   5       1   T63
....................uuuggcacuGgcacauuuuug.......................................................................   4       1   T63
....................uuuUgcacuagcacauuuuug.......................................................................   6       1   T63
....................uuuggcacuagcacauuCuug.......................................................................   2       1   T63
....................uuuggcacuagcacauuuuuA.......................................................................   97      1   T63
....................uuuggcacuaUcacauuuuug.......................................................................   1       1   T63
....................uuuggcacuagcacauuuCug.......................................................................   1       1   T63
....................Auuggcacuagcacauuuuug.......................................................................   39      1   T63
....................uuuggcacuaAcacauuuuug.......................................................................   38      1   T63
....................uuuggcacuagAacauuuuug.......................................................................   4       1   T63
....................Cuuggcacuagcacauuuuug.......................................................................   12      1   T63
....................uuuAgcacuagcacauuuuug.......................................................................   2       1   T63
....................uuuggcacuagcacauuuuuU.......................................................................   309     1   T63
....................uuAggcacuagcacauuuuug.......................................................................   4       1   T63
```

Mature **Star**

gcuaguccucuucucuguucuuuggcacuagcacauuuuugugugacgaucaaaccuacaaaauuguguuagugucgaauaauaugggaagacauucgucauaaaucauaca			
....................uuuggcacuagcacauGuuug...	4	1	T63
....................uuuggcUcuagcacauuuuug...	2	1	T63
....................uuuggcacGagcacauuuuug...	1	1	T63
....................uuuggcacuagcacauuuuuC...	7	1	T63
....................uuuggcacuaCcacauuuuug...	3	1	T63
....................uuugCcacuagcacauuuuug...	10	1	T63
....................uuuggcacuagcacGuuuuug...	1	1	T63
....................uuuggcacuagcacauuuuGg...	2	1	T63
....................uuuggUacuagcacauuuuug...	76	1	T63
....................uuuCgcacuagcacauuuuug...	2	1	T63
....................uuuggcacuagcacauuuuug...	29913	0	T63
....................uuuggcacuagcacauuuuCg...	1	1	T63
....................uuGggcacuagcacauuuuug...	8	1	T63
....................uuugAcacuagcacauuuuug...	16	1	T63
....................uuuggcacuagcacaAuuuug...	25	1	T63
....................uuuggcacuagcacaGuuuug...	1	1	T63
....................uuuggcacuagcaUauuuuug...	16	1	T63
....................uuuggcacuagcacCuuuuug...	3	1	T63
....................uuuggcacuagcaAauuuuug...	4	1	T63
....................uuuggcacuagcacauuuAug...	3	1	T63
....................uuuggAacuagcacauuuuug...	2	1	T63
....................uuuggGacuagcacauuuuug...	4	1	T63
....................uuuggcacuagcUcauuuuug...	1	1	T63
....................uuugUcacuagcacauuuuug...	2	1	T63
....................uuuggcacuagcacUuuuuug...	30	1	T63
....................uuuggcacuagcGcauuuuug...	3	1	T63
....................uuuggcacuagcacauuAuug...	4	1	T63
....................uGuggcacuagcacauuuuug...	3	1	T63
....................uuuggcacuagcacauuGuug...	5	1	T63
....................uCuggcacuagcacauuuuug...	7	1	T63
....................uuuggcacuagGacauuuuug...	5	1	T63
....................uuuggcacuagcacauuGuugu..	84	1	T63
....................uuuggcUcuagcacauuuuugu..	59	1	T63
....................uuuggcacuGgcacauuuuugu..	92	1	T63
....................uuuggcacuagcaGauuuuugu..	42	1	T63
....................Auuggcacuagcacauuuuugu..	789	1	T63
....................uuuggcaGuagcacauuuuugu..	21	1	T63
....................uuuggcacuagcacauuuuugC..	263	1	T63
....................uuuggcacuagcacauuuAugu..	114	1	T63
....................uuuggcacuagcacGuuuuugu..	39	1	T63
....................uuuggcacuagcacauuuuAgu..	109	1	T63
....................uuuggcacuagcacauuuuugu..	819008	0	T63
....................uuuggcacuagcaAauuuuugu..	46	1	T63
....................uuGggcacuagcacauuuuugu..	190	1	T63
....................uAuggcacuagcacauuuuugu..	21	1	T63
....................uuuggcGcuagcacauuuuugu..	20	1	T63
....................uuuggcacuagcacaCuuuugu..	61	1	T63
....................uuuggcaAuagcacauuuuugu..	18	1	T63
....................uuugAcacuagcacauuuuugu..	392	1	T63
....................uuuggcacuagcacauuuuuAu..	53	1	T63
....................uuuggUacuagcacauuuuugu..	1723	1	T63
....................Guuggcacuagcacauuuuugu..	182	1	T63
....................uuuggcacuaUcacauuuuugu..	32	1	T63
....................uuuggcacuagcacaGuuuugu..	14	1	T63
....................uuuggcacuagcacauuAuugu..	116	1	T63
....................uuuggcacAagcacauuuuugu..	392	1	T63
....................uuCggcacuagcacauuuuugu..	57	1	T63
....................uuuggcacuagcacauuuuugA..	4825	1	T63
....................Cuuggcacuagcacauuuuugu..	250	1	T63
....................uuugCcacuagcacauuuuugu..	114	1	T63
....................uuuggcacuagcacUuuuuugu..	493	1	T63
....................uuuggcacuagcGcauuuuugu..	35	1	T63
....................uuuggcacuagcacauGuuugu..	60	1	T63
....................uuuggcacuCgcacauuuuugu..	241	1	T63
....................uGuggcacuagcacauuuuugu..	42	1	T63
....................uuuggcacuagcacauuuGugu..	28	1	T63
....................uuuggcacuUgcacauuuuugu..	50	1	T63
....................uuuggcacuagcacauAuuugu..	349	1	T63
....................uuuggcacuaAcacauuuuugu..	828	1	T63
....................uuuggcCcuagcacauuuuugu..	7	1	T63

```
                    Mature                                     Star
gcuaguccucuucucuguucuuuggcacuagcacauuuuugugugacgaucaaaccuacaaaauuguguuagugucgaauaauaugggaagacauucgucauaaaucauaca
....................uuuggcacuagcacauuuuGgu......................................................................  38    1  T63
....................uuuggcacuagAacauuuuugu......................................................................  88    1  T63
....................uuuggcacuagUacauuuuugu......................................................................  201   1  T63
....................uuuggcacuagcacauuuuuCu......................................................................  121   1  T63
....................uuuggcacGagcacauuuuugu......................................................................  22    1  T63
....................uuuggcacuagcacauuCuugu......................................................................  48    1  T63
....................uuuggcacuagcacauCuuugu......................................................................  96    1  T63
....................uuuggcacuagcacCuuuuugu......................................................................  21    1  T63
....................uuuggcacuagcacauuuCugu......................................................................  78    1  T63
....................uuuggcacuagGacauuuuugu......................................................................  153   1  T63
....................uuuggcacuagcaUauuuuugu......................................................................  135   1  T63
....................uuuCgcacuagcacauuuuugu......................................................................  43    1  T63
....................uuugUcacuagcacauuuuugu......................................................................  79    1  T63
....................uuuggcacuaCcacauuuuugu......................................................................  84    1  T63
....................uuuggcaUuagcacauuuuugu......................................................................  150   1  T63
....................uuuggcacuagcacauuuuCgu......................................................................  38    1  T63
....................uuuggcacuagcacauuuuugG......................................................................  325   1  T63
....................uuuggcacuagcUcauuuuugu......................................................................  21    1  T63
....................uuuggAacuagcacauuuuugu......................................................................  83    1  T63
....................uuuggcacuagcacaAuuuugu......................................................................  266   1  T63
....................uuuggcacuagcacauuuuuUu......................................................................  230   1  T63
....................uCuggcacuagcacauuuuugu......................................................................  65    1  T63
....................uuuUgcacuagcacauuuuugu......................................................................  119   1  T63
....................uuAggcacuagcacauuuuugu......................................................................  87    1  T63
....................uuuggcacCagcacauuuuugu......................................................................  46    1  T63
....................uuuAgcacuagcacauuuuugu......................................................................  43    1  T63
....................uuuggGacuagcacauuuuugu......................................................................  125   1  T63
....................uuuggcacuagcacauuuuuguA.....................................................................  9053  1  T63
....................uuuAgcacuagcacauuuuugug.....................................................................  1     1  T63
....................uuuUgcacuagcacauuuuugug.....................................................................  1     1  T63
....................uuuggcacuagcacaAuuuugug.....................................................................  1     1  T63
....................uuuggcacuGgcacauuuuugug.....................................................................  14    1  T63
....................uuuggcacuagcacauuuuuguU.....................................................................  9638  1  T63
....................uuuggUacuagcacauuuuugug.....................................................................  1     1  T63
....................uuuggcacuagcacUuuuuugug.....................................................................  1     1  T63
....................uuuggcacuagcacauuuuugug.....................................................................  1109  0  T63
....................uuugUcacuagcacauuuuugug.....................................................................  1     1  T63
....................uuuggcacuagcacauuuuugAg.....................................................................  11    1  T63
....................uuGggcacuagcacauuuuugug.....................................................................  1     1  T63
....................uuuggcacuagcacauuuuuUug.....................................................................  2     1  T63
....................uuuggcacuaAcacauuuuugug.....................................................................  2     1  T63
....................uuugAcacuagcacauuuuugug.....................................................................  1     1  T63
....................Auuggcacuagcacauuuuugug.....................................................................  1     1  T63
....................uuuggcacAagcacauuuuugug.....................................................................  1     1  T63
....................uGuggcacuagcacauuuuugug.....................................................................  2     1  T63
....................uuuggcacuagcacauuuuuguC.....................................................................  174   1  T63
....................uuuggcacuagcacauuuuugugu....................................................................  61    0  T63
....................uuuggcacuagcacauuuuugugG....................................................................  11    1  T63
....................uuuggcacuagcacauuuuugugA....................................................................  1441  1  T63
....................uuuggcacuagcacauuuuuUugu....................................................................  5     1  T63
....................uuuggcacuagcacauuuuugugC....................................................................  1     1  T63
....................uuuggcacuagcacauuuuugGgu....................................................................  1     1  T63
....................uuuggcacuagcacauuuuGgugu....................................................................  1     1  T63
....................uuuggcacuagcacauuuuuguAu....................................................................  1700  1  T63
....................uuuggcacuagcacauuuuuguCu....................................................................  9     1  T63
....................uuuggcacuagcacauuuuuguUu....................................................................  498   1  T63
....................uuuggcacuagcacauuuuugugug...................................................................  1     0  T63
....................uuuggcacuagcacauuuuuguAug...................................................................  1     1  T63
....................uuuggcacuagcacauuuuugugAga..................................................................  1     1  T63
....................uuuggcacuagcacauuuuuguguga..................................................................  8     0  T63
....................uuuggcacuagcacauuuuugugugac.................................................................  43    0  T63
....................uGuggcacuagcacauuuuugugugac.................................................................  1     1  T63
....................uuuggcacuagcacauuuuugAgugac.................................................................  1     1  T63
....................uuuggcacuagcacauuuuugugugaU.................................................................  1     1  T63
.....................uuggcacuaAcacauuuu.........................................................................  1     1  T63
.....................uuggcacuagcacauuuu.........................................................................  55    0  T63
.....................uuggcacuagcacaAuuuu........................................................................  1     1  T63
.....................uuggcacuagcacauuAuu........................................................................  1     1  T63
.....................uuggcacuagcacauuuGu........................................................................  4     1  T63
.....................uuUgcacuagcacauuuuu........................................................................  2     1  T63
```

```
                    Mature                                 Star
gcuaguccucuucucuguucuuuggcacuagcacauuuuugugugacgaucaaaccuacaaaauuguguuagugucgaauaauaugggaagacauucgucauaaaucauaca
.....................uuggcacuagcacauuuuu........................................................................	259	0	T63
.....................uuggcacuaAcacauuuuu........................................................................	1	1	T63
.....................uuggcacuagcacauuAuug.......................................................................	2	1	T63
.....................uuggcacuagcacauuuAug.......................................................................	1	1	T63
.....................uuggcacuagcacauAuuug.......................................................................	1	1	T63
.....................uuggcacuUgcacauuuuug.......................................................................	4	1	T63
.....................uugAcacuagcacauuuuug.......................................................................	1	1	T63
.....................uuggcacuagcaUauuuuug.......................................................................	1	1	T63
.....................uuggcacuagcacauuuuuU.......................................................................	6	1	T63
.....................uuggcacuagcacauuuuuA.......................................................................	5	1	T63
.....................uGggcacuagcacauuuuug.......................................................................	1	1	T63
.....................uuggcacuagcGcauuuuug.......................................................................	1	1	T63
.....................uuggcacuagcacauuuGug.......................................................................	1	1	T63
.....................uuggUacuagcacauuuuug.......................................................................	1	1	T63
.....................uuggcacuagUacauuuuug.......................................................................	1	1	T63
.....................uuggcacuagAacauuuuug.......................................................................	1	1	T63
.....................uuggcacuagcacauuuuug.......................................................................	687	0	T63
.....................Auggcacuagcacauuuuug.......................................................................	1	1	T63
.....................uuggcacuaAcacauuuuug.......................................................................	1	1	T63
.....................Guggcacuagcacauuuuug.......................................................................	1	1	T63
.....................Cuggcacuagcacauuuuugu......................................................................	23	1	T63
.....................uuggcaAuagcacauuuuugu......................................................................	4	1	T63
.....................uuggcacuagcacaCuuuugu......................................................................	2	1	T63
.....................uuggcacuagcacauuuuugG......................................................................	24	1	T63
.....................uuggcacAagcacauuuuugu......................................................................	36	1	T63
.....................uuggcacuagcacauuuAugu......................................................................	16	1	T63
.....................uuggcacuagcGcauuuuugu......................................................................	2	1	T63
.....................uuggcacuagcacauuuuAgu......................................................................	8	1	T63
.....................uuggcacuagcacauuuuugC......................................................................	23	1	T63
.....................uuggcacuagcacauuuuuCu......................................................................	11	1	T63
.....................Guggcacuagcacauuuuugu......................................................................	26	1	T63
.....................uuggcacuagcacauuuuuUu......................................................................	6	1	T63
.....................uuggcacuagcacaGuuuugu......................................................................	1	1	T63
.....................uuggcUcuagcacauuuuugu......................................................................	8	1	T63
.....................uuggcacuagcacauGuuugu......................................................................	1	1	T63
.....................uuggcacGagcacauuuuugu......................................................................	4	1	T63
.....................uuUgcacuagcacauuuuugu......................................................................	14	1	T63
.....................uuggcacuagGacauuuuugu......................................................................	11	1	T63
.....................uuggcacuagcacaAuuuugu......................................................................	26	1	T63
.....................uuggcacuaUcacauuuuugu......................................................................	8	1	T63
.....................uuggcacuagcacauuuuugu......................................................................	58891	0	T63
.....................uuggcacuagcacauuuCugu......................................................................	4	1	T63
.....................uugAcacuagcacauuuuugu......................................................................	15	1	T63
.....................uuggcGcuagcacauuuuugu......................................................................	3	1	T63
.....................uuggcacuagcacauuAuugu......................................................................	27	1	T63
.....................uuggcacuagcacUuuuuugu......................................................................	12	1	T63
.....................uuggGacuagcacauuuuugu......................................................................	3	1	T63
.....................uuggcacuagcacauCuuugu......................................................................	7	1	T63
.....................uGggcacuagcacauuuuugu......................................................................	10	1	T63
.....................uuggcacuaCcacauuuuugu......................................................................	29	1	T63
.....................uuggAacuagcacauuuuugu......................................................................	3	1	T63
.....................Auggcacuagcacauuuuugu......................................................................	80	1	T63
.....................uuggcCcuagcacauuuuugu......................................................................	2	1	T63
.....................uuggcacuagcacauuuuugA......................................................................	108	1	T63
.....................uuggcacuagcacauuuuCgu......................................................................	1	1	T63
.....................uuggcacuagcaAauuuuugu......................................................................	8	1	T63
.....................uuggcacuagcaGauuuuugu......................................................................	1	1	T63
.....................uuggcacuagcacauuuuuAu......................................................................	10	1	T63
.....................uuggcacuagAacauuuuugu......................................................................	7	1	T63
.....................uuggcacuagcUcauuuuugu......................................................................	6	1	T63
.....................uuggcacuUgcacauuuuugu......................................................................	35	1	T63
.....................uuggcacuagcacauuuGugu......................................................................	6	1	T63
.....................uuAgcacuagcacauuuuugu......................................................................	3	1	T63
.....................uuggcacuGgcacauuuuugu......................................................................	4	1	T63
.....................uuggcacuaAcacauuuuugu......................................................................	40	1	T63
.....................uuggcacuagcaUauuuuugu......................................................................	12	1	T63
.....................uuggcacuagcacauuCuugu......................................................................	6	1	T63
.....................uuggcaUuagcacauuuuugu......................................................................	11	1	T63
.....................uugCcacuagcacauuuuugu......................................................................	9	1	T63
.....................uCggcacuagcacauuuuugu......................................................................	6	1	T63
```

```
                    Mature                                      Star
gcuaguccucuucucuguucuuuggcacuagcacauuuuuguguugacgaucaaaccuacaaaauuguguuagugucgaauaauaugggaagacauucgucauaaaucauaca
.....................uuCgcacuagcacauuuuugu......................................................................    8      1    T63
.....................uuggcacuagcacauAuuugu......................................................................    20     1    T63
.....................uuggUacuagcacauuuuugu......................................................................    112    1    T63
.....................uuggcacuagcacauuGuugu......................................................................    2      1    T63
.....................uuggcacuagUacauuuuugu......................................................................    21     1    T63
.....................uugUcacuagcacauuuuugu......................................................................    19     1    T63
.....................uuggcacCagcacauuuuugu......................................................................    11     1    T63
.....................uuggcacuagcacauuuAugug.....................................................................    1      1    T63
.....................uuggcacuGgcacauuuuugug.....................................................................    3      1    T63
.....................uuggcGcuagcacauuuuugug.....................................................................    1      1    T63
.....................uuggcacuagcacauuuuuguA.....................................................................    1452   1    T63
.....................uuggcacuagcacauuuuugGg.....................................................................    1      1    T63
.....................uuggcacuagcacauuAuugug.....................................................................    2      1    T63
.....................uuggcCcuagcacauuuuugug.....................................................................    1      1    T63
.....................uuggcacuagcacauuuuAgug.....................................................................    1      1    T63
.....................uuggcacCagcacauuuuugug.....................................................................    1      1    T63
.....................uuggcacuagcacauuuuugug.....................................................................    5681   0    T63
.....................uuggcacuagcacauuuuuUug.....................................................................    1      1    T63
.....................uuCgcacuagcacauuuuugug.....................................................................    1      1    T63
.....................uuggcacuagcacauuuuugAg.....................................................................    2      1    T63
.....................uuUgcacuagcacauuuuugug.....................................................................    3      1    T63
.....................uuggcacuagcacauuuuCgug.....................................................................    1      1    T63
.....................uuggcacuagcacauuuuugCg.....................................................................    1      1    T63
.....................uuggcaGuagcacauuuuugug.....................................................................    1      1    T63
.....................uuggcacuagcacauuuGugug.....................................................................    2      1    T63
.....................uuggcacuagcacauuuuuguU.....................................................................    558    1    T63
.....................uuggcUcuagcacauuuuugug.....................................................................    2      1    T63
.....................uuggcacuagGacauuuuugug.....................................................................    2      1    T63
.....................uuggcacuagcacauuuuuguC.....................................................................    19     1    T63
.....................uuggcacuagcacauuuuuAug.....................................................................    1      1    T63
.....................uuAgcacuagcacauuuuugug.....................................................................    1      1    T63
.....................uuggcacuagcUcauuuuugug.....................................................................    1      1    T63
.....................uugAcacuagcacauuuuugug.....................................................................    2      1    T63
.....................uuggcacuagcaUauuuuugug.....................................................................    1      1    T63
.....................uuggUacuagcacauuuuugug.....................................................................    8      1    T63
.....................Auggcacuagcacauuuuugug.....................................................................    6      1    T63
.....................uugUcacuagcacauuuuugug.....................................................................    1      1    T63
.....................uuggcacuagcacauuuuuCug.....................................................................    3      1    T63
.....................uuggcacuaCcacauuuuugug.....................................................................    3      1    T63
.....................uuggcacuagcacauCuuugug.....................................................................    1      1    T63
.....................uuggcacuagcacauAuuugug.....................................................................    2      1    T63
.....................uuggcacAagcacauuuuugug.....................................................................    2      1    T63
.....................uuggcacuagcacaCuuuugug.....................................................................    1      1    T63
.....................uuggcacuagcacauuuCugug.....................................................................    2      1    T63
.....................uuggcacuUgcacauuuuugug.....................................................................    3      1    T63
.....................uuggcacuagUacauuuuugug.....................................................................    2      1    T63
.....................uuggcacuaAcacauuuuugug.....................................................................    8      1    T63
.....................uuggcacuagcacauuuuugugC....................................................................    14     1    T63
.....................uuggcacuagcacauuuuuguUu....................................................................    40     1    T63
.....................uuggcacuagcacauuuuugugG....................................................................    138    1    T63
.....................uuggcaUuagcacauuuuugugu....................................................................    1      1    T63
.....................uuggcacuagcacauuuuuguAu....................................................................    320    1    T63
.....................uuggcacuagcacauuuuugugA....................................................................    10155  1    T63
.....................Auggcacuagcacauuuuugugu....................................................................    2      1    T63
.....................uuggcacuagcacauuuuugugu....................................................................    186    0    T63
.....................uuggcacuaCcacauuuuugugu....................................................................    1      1    T63
.....................uuggcacuagcacauuuuuguguA...................................................................    2      1    T63
.....................uuggcacuagcacauuuuugugAg...................................................................    5      1    T63
.....................uuggcacuagcacauuuuugugug...................................................................    3      0    T63
.....................uuggcacuagcacauuuuuguguU...................................................................    1      1    T63
.....................uuggcacuagcacauuuuugugAga..................................................................    2      1    T63
.....................uuggcacuagcacauuuuuguguAa..................................................................    1      1    T63
.....................uuggcacuagcacauuuuugugugac.................................................................    3      0    T63
.....................uuggcacuagcacauuuuugugugacA................................................................    1      1    T63
......................uggcacuagcacauuuuu........................................................................    3      0    T63
......................uggcacuagcacauuuuug.......................................................................    11     0    T63
......................uggcacuagcacauuuuugA......................................................................    1      1    T63
......................uggcacuGgcacauuuuugu......................................................................    1      1    T63
......................uggcacuagGacauuuuugu......................................................................    3      1    T63
......................uUgcacuagcacauuuuugu......................................................................    1      1    T63
```

```
                    Mature                                 Star
gcuaguccucuucucuguucuuuggcacuagcacauuuuuguguugacgaucaaaccuacaaaauuguguuagugucgaauaauaugggaagacauucgucauaaaucauaca
......................uggcacuagcacauuuuugu......................................................................    1325    0    T63
......................Gggcacuagcacauuuuugu......................................................................    5       1    T63
......................uggcaUuagcacauuuuugu......................................................................    1       1    T63
......................uggcacuagcacauuuGugu......................................................................    1       1    T63
......................uggUacuagcacauuuuugu......................................................................    2       1    T63
......................ugUcacuagcacauuuuugu......................................................................    1       1    T63
......................uggcacAagcacauuuuugu......................................................................    1       1    T63
......................uggcUcuagcacauuuuugu......................................................................    2       1    T63
......................uggcacuaAcacauuuuugu......................................................................    5       1    T63
......................Aggcacuagcacauuuuugu......................................................................    2       1    T63
......................uggcacuagcacauuuuAgu......................................................................    1       1    T63
......................uggcacuagcacauuuuugug.....................................................................    247     0    T63
......................uggcacuagcacauuuuuguU.....................................................................    20      1    T63
......................uggcacuagcaGauuuuugug.....................................................................    1       1    T63
......................uggcacuagcacauuuuuguA.....................................................................    18      1    T63
......................uUgcacuagcacauuuuugug.....................................................................    2       1    T63
......................Aggcacuagcacauuuuugug.....................................................................    2       1    T63
......................uCgcacuagcacauuuuugug.....................................................................    1       1    T63
......................uggcacuaAcacauuuuugug.....................................................................    1       1    T63
......................uggcacuaUcacauuuuugugu....................................................................    1       1    T63
......................uggcacuagcacauuuuugugA....................................................................    660     1    T63
......................uggcacuaAcacauuuuugugu....................................................................    1       1    T63
......................uggcacuagcacauuuuuguAu....................................................................    9       1    T63
......................uggcacuagcacauuuuugugu....................................................................    393     0    T63
......................uggcacuagcacauuuuuAugu....................................................................    1       1    T63
......................uggcacuagcacauuuuugGgu....................................................................    1       1    T63
......................uggcacuagcacauuuuuguUu....................................................................    1       1    T63
......................Aggcacuagcacauuuuugugu....................................................................    3       1    T63
......................uggcacuagcacauuuuugugG....................................................................    5       1    T63
......................Gggcacuagcacauuuuugugu....................................................................    2       1    T63
......................uggcacuagcacauuuuugugAg...................................................................    1       1    T63
......................uggcacuagcacauuuuuguguU...................................................................    2       1    T63
......................uggcacuagcacauuuuugugug...................................................................    3       0    T63
......................uggcacuagcacauuuuuguguA...................................................................    7       1    T63
......................uggcacuagcacauuuuuguguga..................................................................    1       0    T63
.......................ggcacuagcacauuuuug.......................................................................    2       0    T63
.......................Ugcacuagcacauuuuugu......................................................................    1       1    T63
.......................ggcacuagcacauuuuugu......................................................................    71      0    T63
.......................ggcaUuagcacauuuuugu......................................................................    1       1    T63
.......................ggcacuagcacauuuuugA......................................................................    2       1    T63
.......................ggcacuagcacauuuuuguU.....................................................................    2       1    T63
.......................ggcacuagcacauuuuuguA.....................................................................    1       1    T63
.......................ggcacuagcacauuuuugug.....................................................................    2       0    T63
.......................ggcacuagcacauuuuugugA....................................................................    2       1    T63
........................gcacuagcacauuuuugu......................................................................    24      0    T63
........................gcacuagcacauuuuuguU.....................................................................    1       1    T63
........................gcacuagcacauuuuuguA.....................................................................    1       1    T63
........................gcacuagcacauuuuuguAu....................................................................    1       1    T63
.........................cacuagcacauuuuugug.....................................................................    1       0    T63
.........................cacuagcacauuuuuguU.....................................................................    2       1    T63
.........................cacuagcacauuuuuguA.....................................................................    24      1    T63
.........................cacuagcacauuuuugugA....................................................................    1       1    T63
.......................................................cuacaaaauuguguuaguguc....................................    1       0    T63
........................................................uacaaaauuguguuaguguc....................................    12      0    T63
........................................................uacaaaauuguguuagugucC...................................    1       1    T63
........................................................uacaaaauuguguuagugucg...................................    195     0    T63
........................................................uacaaaauuguguuagugucA...................................    1       1    T63
........................................................uacaaaauuguguuagugucgU..................................    8       1    T63
........................................................uacaaaauuguguuagugucga..................................    16      0    T63
........................................................uacaaaauuguguuagugucgaa.................................    2       0    T63
........................................................uacaaaauuguguuagugucgaau................................    3       0    T63
........................................................uacaaaauuguguuagugucgaaua...............................    1       0    T63
.........................................................acaaaauuguguuagugucg...................................    4       0    T63
.........................................................acaaaauuguguuagugucUa..................................    1       1    T63
.........................................................acaaaauuguguuagugucga..................................    17      0    T63
.........................................................acaaaauuguguuagugucgaa.................................    8       0    T63
.........................................................acaaaauuguguuagugucgaU.................................    1       1    T63
.........................................................acaaaauuguguuagugucgaau................................    3       0    T63
..........................................................caaaauuguguuaguguc....................................    3       0    T63
..........................................................caaaauuguguuagugucg...................................    8       0    T63
```

Mature Star

gcuaguccucuucucuguucuuuggcacuagcacauuuuugugugacgaucaaaccuacaaaauuguguuagugucgaauaauaugggaagacauucgucauaaaucauaca			
....caaaauugAguuagugucga..................................	1	1	T63
....caaaauuguguuagugucAa..................................	1	1	T63
....caaaauuguguuagugucga..................................	159	0	T63
....caaaauuguguuagugucgGa.................................	1	1	T63
....caaaauuguguuagugucgaa.................................	73	0	T63
....caaaauuguguuagugucgaC.................................	1	1	T63
....caaaauuguguuagugucCaau................................	1	1	T63
....caaaauuCuguuagugucgaau................................	1	1	T63
....caaaauuguguuagugCcgaau................................	1	1	T63
....caaaauuguguuagugucgaUu................................	4	1	T63
....Aaaaauuguguuagugucgaau................................	1	1	T63
....caaGauuguguuagugucgaau................................	1	1	T63
....caaaauuguguuaguUucgaau................................	1	1	T63
....Gaaaauuguguuagugucgaau................................	3	1	T63
....caaaauuguguuagugucgUau................................	2	1	T63
....caaaauuguguuagugucgaaA................................	33	1	T63
....caaaauuguguuagugucgaaG................................	4	1	T63
....caaaauuguguuagugucgaaC................................	9	1	T63
....Uaaaauuguguuagugucgaau................................	2	1	T63
....caaaauugAguuagugucgaau................................	1	1	T63
....caaaauuguguuagugucgaau................................	695	0	T63
....caaaUuuguguuagugucgaau................................	1	1	T63
....caaaauuguguuagugucgaauG...............................	1	1	T63
....caaaauuguguuagugucgaaua...............................	73	0	T63
....caaaauuguguuagugucgaauU...............................	48	1	T63
....caaaauuguguuagugucgaaAa...............................	9	1	T63
....caaaauuguguuagugucgaauC...............................	2	1	T63
....caaaauuguguuagugucgaaCa...............................	1	1	T63
....Uaaaauuguguuagugucgaaua...............................	2	1	T63
....caaaauuguguuagugucgaauUa..............................	3	1	T63
....caaaauuguguuagugucgaauaC..............................	1	1	T63
....caaaauuguguuagugucgaaAaa..............................	1	1	T63
....aaaauuguguuagugucg...................................	49	0	T63
....aaaauuguguuagugucA...................................	2	1	T63
....aaaauugAguuagugucg...................................	1	1	T63
....aaaauuguguuagAgucg...................................	1	1	T63
....aaUauuguguuagugucga..................................	2	1	T63
....aaaauuguguuagugucAa..................................	1	1	T63
....aUaauuguguuagugucga..................................	1	1	T63
....aaaauugCguuagugucga..................................	1	1	T63
....aaaauuguguuagugucga..................................	681	0	T63
....aaaauuguguuagAgucga..................................	1	1	T63
....aaaauuguguuagugAcga..................................	1	1	T63
....aaaauuguAuuagugucga..................................	2	1	T63
....aaaauuguguuGgugucgaa.................................	1	1	T63
....aaaauuguguuaguguUgaa.................................	1	1	T63
....aaaauuguguuagugucgGa.................................	2	1	T63
....aaaauuguguuagugucgaG.................................	1	1	T63
....aaaauuguguuagugucgUa.................................	4	1	T63
....aaaauuguguuagugucgaa.................................	387	0	T63
....aaaauugugCuagugucgaa.................................	1	1	T63
....aaaauuguguuagugucgaU.................................	13	1	T63
....aaaauuguguuaCugucgaau................................	1	1	T63
....aaaauuguguuagugucgaau................................	3777	0	T63
....aaaauuAuguuagugucgaau................................	1	1	T63
....aaaUuuguguuagugucgaau................................	4	1	T63
....aaaauuguguuagugucgaaG................................	9	1	T63
....aaaauAguguuagugucgaau................................	1	1	T63
....aaaauugugCuagugucgaau................................	2	1	T63
....aaaauuguguuUgugucgaau................................	2	1	T63
....aaaauuUuguuagugucgaau................................	2	1	T63
....aaaauuguguuGgugucgaau................................	2	1	T63
....aaaauuguguuagugucgUau................................	1	1	T63
....aaaaGuguguuagugucgaau................................	1	1	T63
....aaaauuguguuaguguGgaau................................	3	1	T63
....aaaauGguguuagugucgaau................................	1	1	T63
....aaaauuguguuagugucgaaA................................	126	1	T63
....aaaGuuguguuagugucgaau................................	1	1	T63
....aaaauuguguuagugucgGau................................	2	1	T63
....aaaauuguguuagugucAaau................................	4	1	T63

```
                    Mature                                     Star
gcuaguccucuucucuguucuuuggcacuagcacauuuuugugugacgaucaaaccuacaaaauuguguuagugucgaauaauaugggaagacauucgucauaaaucauaca
...........................................................aUaauuguguuagugucgaau......................................................	1	1	T63
...........................................................aaaauugugAuagugucgaau......................................................	1	1	T63
...........................................................aaaauuguguuagugucgaaC......................................................	45	1	T63
...........................................................aaaauuguguuagugucUaau......................................................	2	1	T63
...........................................................aaaauuguguuagGgucgaau......................................................	1	1	T63
...........................................................aaaauuguAuuagugucgaau......................................................	1	1	T63
...........................................................aaaauuguguuaAugucgaau......................................................	1	1	T63
...........................................................aaaauugAguuagugucgaau......................................................	2	1	T63
...........................................................aaUauuguguuagugucgaau......................................................	3	1	T63
...........................................................aaaauuguguuagugucgaUu......................................................	6	1	T63
...........................................................aaaauuguguuagAgucgaau......................................................	7	1	T63
...........................................................aUaauuguguuagugucgaaua.....................................................	3	1	T63
...........................................................aaaauuguguuagugucAaaua.....................................................	2	1	T63
...........................................................aaaauugAguuagugucgaaua.....................................................	4	1	T63
...........................................................aaaauuguguuaguCucgaaua.....................................................	1	1	T63
...........................................................aaaauuguguuagugAcgaaua.....................................................	2	1	T63
...........................................................Gaaauuguguuagugucgaaua.....................................................	1	1	T63
...........................................................aaaauuguguuagugucgUaua.....................................................	1	1	T63
...........................................................aaaauuguguuaguAucgaaua.....................................................	1	1	T63
...........................................................aaaauuguguuagAgucgaaua.....................................................	6	1	T63
...........................................................aaaauuguguuagugucgaaGa.....................................................	5	1	T63
...........................................................aaaauuguguuaguguUgaaua.....................................................	1	1	T63
...........................................................aaaauuguCuuagugucgaaua.....................................................	1	1	T63
...........................................................aaaauuguguuaguguGgaaua.....................................................	1	1	T63
...........................................................aaaauuguguuagugucgaaCa.....................................................	74	1	T63
...........................................................aaaUuuguguuagugucgaaua.....................................................	3	1	T63
...........................................................aGaauuguguuagugucgaaua.....................................................	1	1	T63
...........................................................aaaauuguguCagugucgaaua.....................................................	2	1	T63
...........................................................aaaauuguguuagugucgaaua.....................................................	3742	0	T63
...........................................................aaaauuguUuuagugucgaaua.....................................................	1	1	T63
...........................................................aaaauuguguuUgugucgaaua.....................................................	1	1	T63
...........................................................aaaauuUuguuagugucgaaua.....................................................	1	1	T63
...........................................................aaaauuguguuagugucgaauU.....................................................	261	1	T63
...........................................................aaaauuguguuagugucgaUua.....................................................	1	1	T63
...........................................................aaaauuguguuagugucgaauC.....................................................	22	1	T63
...........................................................aaaauCguguuagugucgaaua.....................................................	1	1	T63
...........................................................aaaauuguguuagugucgaauG.....................................................	29	1	T63
...........................................................aaaauAguguuagugucgaaua.....................................................	2	1	T63
...........................................................aaaauuguguuagugucgaaAa.....................................................	72	1	T63
...........................................................aaaauugugAuagugucgaaua.....................................................	1	1	T63
...........................................................aaaauuguguuagugucUaaua.....................................................	1	1	T63
...........................................................aaaauuguAuuagugucgaaua.....................................................	3	1	T63
...........................................................aaaauuguguuagugucgaaAaa....................................................	3	1	T63
...........................................................aaaauuguguuagugucgaauGa....................................................	1	1	T63
...........................................................aaaauuguguuagugucgaauUa....................................................	6	1	T63
...........................................................aaaauuguguuagugucgaauaa....................................................	17	0	T63
...........................................................aaaauuguguuagugucgaauaG....................................................	1	1	T63
...........................................................aaaauuguguuagugucgaauaU....................................................	20	1	T63
...........................................................aaaauuguguuagugucgaauaC....................................................	10	1	T63
...........................................................aaaauuguguuagugucgaauaCu...................................................	1	1	T63
...........................................................aaaauuguguuagAgucgaauaau...................................................	1	1	T63
...........................................................aaaauuguguuagugucgaauaaC...................................................	1	1	T63
...........................................................aaaauuguguuagugucgaauaaA...................................................	1	1	T63
...........................................................aaaauuguguuagugucgaauaau...................................................	3	0	T63
...........................................................aaaauuguguuagugucgaauaauC..................................................	1	1	T63
............................................................aaauuguguuagugucga........................................................	1	0	T63
............................................................aaauuguguuagugucgaa.......................................................	1	0	T63
............................................................aaauuguguuagugucgaaA......................................................	1	1	T63
............................................................aaauuguguuaguAucgaau......................................................	1	1	T63
............................................................aaauuguguuagugucgaau......................................................	88	0	T63
............................................................aaauugugAuagugucgaaua.....................................................	3	1	T63
............................................................aaauuguguuagugucgaauG.....................................................	1	1	T63
............................................................aaauuAuguuagugucgaaua.....................................................	1	1	T63
............................................................aaauuguguuagugucgaaua.....................................................	924	0	T63
............................................................aaaCuguguuagugucgaaua.....................................................	1	1	T63
............................................................aaauuguguuGgugucgaaua.....................................................	1	1	T63
............................................................aaauuguguuaguAucgaaua.....................................................	1	1	T63
............................................................aUauuguguuagugucgaaua.....................................................	1	1	T63
............................................................aaauugCguuagugucgaaua.....................................................	1	1	T63
............................................................aaauuguguuaguguUgaaua.....................................................	2	1	T63
```

```
                    Mature                                    Star
gcuaguccucuucucuguucuuuggcacuagcacauuuuugugugacgaucaaaccuacaaaauuguguuagugucgaauaauaugggaagacauucgucauaaaucauaca
............................................................aaauuguguuagugucgaauU...............................  12   1  T63
............................................................aaauuguguCagugucgaaua...............................  1    1  T63
............................................................aaaAuguguuagugucgaaua...............................  1    1  T63
............................................................aaauuguguuaguUucgaaua...............................  1    1  T63
............................................................aaauuCuguuagugucgaaua...............................  2    1  T63
............................................................aaauuguguuagugucgaaAa...............................  1    1  T63
............................................................aaauuguguuagugucgaauaU..............................  10   1  T63
............................................................aaauuguguuagugucgaauaa..............................  4    0  T63
............................................................aaauuguguuagugucgaauaC..............................  3    1  T63
............................................................aaauuguguuagugucgaauUa..............................  2    1  T63
............................................................aaauuguguuagugucgaauaaA.............................  1    1  T63
.............................................................aauuguguuagugucgaau................................  2    0  T63
.............................................................aauuguguuagugucgaaua...............................  6    0  T63
..............................................................auuguguuagugucgaau................................  1    0  T63

.................uAcuuuggcacuagcacauuuuugu......................................................................  1    1  MOL
..................ucuuuggcacuagcacauuuu.........................................................................  1    0  MOL
..................ucuuuggcacuagcacauuuuu........................................................................  2    0  MOL
..................Gcuuuggcacuagcacauuuuu........................................................................  1    1  MOL
..................ucuuuggcacuagcacauuuuug.......................................................................  2    0  MOL
..................uUuuuggcacuagcacauuuuug.......................................................................  2    1  MOL
..................uUuuuggcacuagcacauuuuugu......................................................................  13   1  MOL
...................cuuuggcacuagcacauuuu.........................................................................  1    0  MOL
...................Uuuuggcacuagcacauuuu.........................................................................  2    1  MOL
...................Auuuggcacuagcacauuuuu........................................................................  1    1  MOL
...................Uuuuggcacuagcacauuuuu........................................................................  7    1  MOL
...................cuuuggcacuagcacauuuuu........................................................................  17   0  MOL
...................cuuuggcacuagcacauuuuug.......................................................................  13   0  MOL
...................Uuuuggcacuagcacauuuuug.......................................................................  20   1  MOL
...................Auuuggcacuagcacauuuuug.......................................................................  1    1  MOL
...................Auuuggcacuagcacauuuuugu......................................................................  5    1  MOL
...................cuuuggcacuagcacauuuuugA......................................................................  11   1  MOL
...................Guuuggcacuagcacauuuuugu......................................................................  1    1  MOL
...................cuuuggcacuagcacauuuuugu......................................................................  28   0  MOL
...................Uuuuggcacuagcacauuuuugu......................................................................  100  1  MOL
....................uuuggcacuagcacauuG..........................................................................  2    1  MOL
....................uuuggcacuagcacauuu..........................................................................  23   0  MOL
....................uuuggcacuagcacauGu..........................................................................  2    1  MOL
....................uuuggUacuagcacauuuu.........................................................................  1    1  MOL
....................uuuggcacuagcacauuuu.........................................................................  554  0  MOL
....................uuGggcacuagcacauuuu.........................................................................  1    1  MOL
....................uuuggcacuagcacUuuuu.........................................................................  1    1  MOL
....................Cuuggcacuagcacauuuu.........................................................................  1    1  MOL
....................uuuggcacuagcacauuuG.........................................................................  9    1  MOL
....................uuuggcacuagcacauuuA.........................................................................  1    1  MOL
....................uuuggcacuaAcacauuuu.........................................................................  2    1  MOL
....................uuuggcacuagcacaCuuu.........................................................................  1    1  MOL
....................uuuggcacuagcacauuGu.........................................................................  18   1  MOL
....................Auuggcacuagcacauuuu.........................................................................  2    1  MOL
....................uuuggcacuagcaAauuuu.........................................................................  3    1  MOL
....................Cuuggcacuagcacauuuuu........................................................................  3    1  MOL
....................Auuggcacuagcacauuuuu........................................................................  18   1  MOL
....................uuuggcacuagcUcauuuuu........................................................................  1    1  MOL
....................uuuggcacuagcacauuuGu........................................................................  47   1  MOL
....................uuuggcaUuagcacauuuuu........................................................................  3    1  MOL
....................uuuAgcacuagcacauuuuu........................................................................  1    1  MOL
....................uuuggcacuagcacauuCuu........................................................................  1    1  MOL
....................Guuggcacuagcacauuuuu........................................................................  5    1  MOL
....................uuuggcacuagcacauuuAu........................................................................  4    1  MOL
....................uuuggUacuagcacauuuuu........................................................................  6    1  MOL
....................uuuggcacuaCcacauuuuu........................................................................  2    1  MOL
....................uuuggcacAagcacauuuuu........................................................................  7    1  MOL
....................uuuggcacuagcacauCuuu........................................................................  2    1  MOL
....................uuuggcGcuagcacauuuuu........................................................................  1    1  MOL
....................uuuggAacuagcacauuuuu........................................................................  1    1  MOL
....................uuuggcaAuagcacauuuuu........................................................................  1    1  MOL
....................uuuggcacuagcacauuuuG........................................................................  25   1  MOL
....................uuuggcacuagcacauGuuu........................................................................  1    1  MOL
....................uuugAcacuagcacauuuuu........................................................................  5    1  MOL
....................uuuggcacuGgcacauuuuu........................................................................  1    1  MOL
```

```
                    Mature                                       Star
gcuaguccucuucucuguucuuuggcacuagcacauuuuugugugacgaucaaaccuacaaaauuguguuagugucgaauaauaugggaagacauucgucauaaaucauaca
....................uuuggcacuagcacauuuuu........................................................................ 8892 0 MOL
....................uuuggcacuagcacauAuuu........................................................................ 9 1 MOL
....................uuuggcacuCgcacauuuuu........................................................................ 4 1 MOL
....................uuuggcacuagcacUuuuuu........................................................................ 5 1 MOL
....................uuuggcacuagcacauuuuA........................................................................ 21 1 MOL
....................uuuggcacuagcacaGuuuu........................................................................ 2 1 MOL
....................uuuggcacuagcacaAuuuu........................................................................ 7 1 MOL
....................uuuggcacuagcaUauuuuu........................................................................ 4 1 MOL
....................uuuggcacuagcacauuAuu........................................................................ 1 1 MOL
....................uuugCcacuagcacauuuuu........................................................................ 4 1 MOL
....................uuAggcacuagcacauuuuu........................................................................ 4 1 MOL
....................uuuUgcacuagcacauuuuu........................................................................ 2 1 MOL
....................uCuggcacuagcacauuuuu........................................................................ 1 1 MOL
....................uuuggcacuagAacauuuuu........................................................................ 2 1 MOL
....................uuuggcacuaAcacauuuuu........................................................................ 5 1 MOL
....................uuuggcacuagcacaCuuuu........................................................................ 1 1 MOL
....................uuuCgcacuagcacauuuuu........................................................................ 1 1 MOL
....................uuuggcacuagUacauuuuu........................................................................ 5 1 MOL
....................uuugUcacuagcacauuuuug....................................................................... 14 1 MOL
....................uuuggAacuagcacauuuuug....................................................................... 4 1 MOL
....................Cuuggcacuagcacauuuuug....................................................................... 25 1 MOL
....................uuuggUacuagcacauuuuug....................................................................... 60 1 MOL
....................uuuggcacuagcUcauuuuug....................................................................... 1 1 MOL
....................uuuggcacuagcacauuAuug....................................................................... 7 1 MOL
....................uuugCcacuagcacauuuuug....................................................................... 9 1 MOL
....................uuuggcacuagcacCuuuuug....................................................................... 1 1 MOL
....................uuuggcacuagcacGuuuuug....................................................................... 2 1 MOL
....................uuuAgcacuagcacauuuuug....................................................................... 5 1 MOL
....................uuuggcUcuagcacauuuuug....................................................................... 4 1 MOL
....................uuuggcacuGgcacauuuuug....................................................................... 1 1 MOL
....................uuuggcacuagcacauuuuAg....................................................................... 7 1 MOL
....................uuuggcacuagcaAauuuuug....................................................................... 3 1 MOL
....................uuuggcacuagcaUauuuuug....................................................................... 10 1 MOL
....................Auuggcacuagcacauuuuug....................................................................... 77 1 MOL
....................uuuggcacuagGacauuuuug....................................................................... 9 1 MOL
....................uuuggcacuagcacauuuuGg....................................................................... 2 1 MOL
....................uuuggcacuCgcacauuuuug....................................................................... 16 1 MOL
....................uuuggcacuagcGcauuuuug....................................................................... 1 1 MOL
....................uuuggcacuagUacauuuuug....................................................................... 9 1 MOL
....................uuGggcacuagcacauuuuug....................................................................... 9 1 MOL
....................uuuCgcacuagcacauuuuug....................................................................... 4 1 MOL
....................uuuggcacuagcacauGuuug....................................................................... 4 1 MOL
....................uuuggcacuagcacauuuuuU....................................................................... 237 1 MOL
....................uAuggcacuagcacauuuuug....................................................................... 1 1 MOL
....................uuuggcacuagcacauuGuug....................................................................... 6 1 MOL
....................uuuggcaUuagcacauuuuug....................................................................... 13 1 MOL
....................uCuggcacuagcacauuuuug....................................................................... 5 1 MOL
....................uuuggcacuagcacauuuuCg....................................................................... 2 1 MOL
....................uuuggcGcuagcacauuuuug....................................................................... 2 1 MOL
....................uuuggcacAagcacauuuuug....................................................................... 17 1 MOL
....................uuuggcacuagcacaGuuuug....................................................................... 1 1 MOL
....................uuuggcacuagcacauuuuuC....................................................................... 9 1 MOL
....................uuuggcacuagcacauuuuuA....................................................................... 264 1 MOL
....................uuuggcacCagcacauuuuug....................................................................... 1 1 MOL
....................uuuggcacuagcacauuuAug....................................................................... 12 1 MOL
....................uuuggcacuaCcacauuuuug....................................................................... 4 1 MOL
....................uuuggcacuagAacauuuuug....................................................................... 3 1 MOL
....................uuuggcacuagcacaCuuuug....................................................................... 4 1 MOL
....................uuuggcacuagcacauuuGug....................................................................... 2 1 MOL
....................uuuggGacuagcacauuuuug....................................................................... 6 1 MOL
....................uuCggcacuagcacauuuuug....................................................................... 4 1 MOL
....................uuuggcacuagcacaAuuuug....................................................................... 20 1 MOL
....................uuuggcacuagcacUuuuuug....................................................................... 16 1 MOL
....................uuuggcacuagcacauAuuug....................................................................... 28 1 MOL
....................uuuggcacuagcaGauuuuug....................................................................... 4 1 MOL
....................uuuggcaGuagcacauuuuug....................................................................... 2 1 MOL
....................uuuggcacuagcacauuuCug....................................................................... 5 1 MOL
....................uuuggcacuaUcacauuuuug....................................................................... 2 1 MOL
....................uuuggcacuagcacauCuuug....................................................................... 7 1 MOL
....................uuugAcacuagcacauuuuug....................................................................... 15 1 MOL
```

```
                    Mature                                 Star
gcuaguccucuucucuguucuuuggcacuagcacauuuuugugugacgaucaaaccuacaaaauuguguuagugucgaauaauaugggaagacauucgucauaaaucauaca
....................uuuggcacuaAcacauuuuug.......................................................................    26      1    MOL
....................uuAggcacuagcacauuuuug.......................................................................    12      1    MOL
....................Guuggcacuagcacauuuuug.......................................................................    26      1    MOL
....................uGuggcacuagcacauuuuug.......................................................................    2       1    MOL
....................uuuggcacuagcacauuuuug.......................................................................    39622   0    MOL
....................uuuUgcacuagcacauuuuug.......................................................................    5       1    MOL
....................uuuggcacuagcacauCuuugu......................................................................    57      1    MOL
....................uuuggcacuagcacauuCuugu......................................................................    19      1    MOL
....................uGuggcacuagcacauuuuugu......................................................................    23      1    MOL
....................uuuggcacuCgcacauuuuugu......................................................................    91      1    MOL
....................uuuggcacuagcacCuuuuugu......................................................................    6       1    MOL
....................uuuggcacuaCcacauuuuugu......................................................................    17      1    MOL
....................uuuggcacuagcacaGuuuugu......................................................................    18      1    MOL
....................uuuggcacuagcacauuAuugu......................................................................    53      1    MOL
....................uuugCcacuagcacauuuuugu......................................................................    108     1    MOL
....................uuuggcacuagcacauuuuugG......................................................................    118     1    MOL
....................uuuggcacuagcaAauuuuugu......................................................................    32      1    MOL
....................uuuggcacuagcaUauuuuugu......................................................................    59      1    MOL
....................uuuggcCcuagcacauuuuugu......................................................................    1       1    MOL
....................uuuggcacuagcacUuuuuugu......................................................................    116     1    MOL
....................uuugUcacuagcacauuuuugu......................................................................    49      1    MOL
....................uuuggAacuagcacauuuuugu......................................................................    38      1    MOL
....................uuuggcacuagcacauuuuuCu......................................................................    57      1    MOL
....................uuuggcacuagcacaCuuuugu......................................................................    26      1    MOL
....................uuuggcacuagcacauuuuCgu......................................................................    28      1    MOL
....................uuuggcacuagcacauuuuugu......................................................................    285243  0    MOL
....................uuuggcacuagcacauuuuugC......................................................................    166     1    MOL
....................uuuUgcacuagcacauuuuugu......................................................................    37      1    MOL
....................uuAggcacuagcacauuuuugu......................................................................    46      1    MOL
....................uuuggcacuagcacauuuuugA......................................................................    6545    1    MOL
....................uuuggcacuagcUcauuuuugu......................................................................    6       1    MOL
....................uuGggcacuagcacauuuuugu......................................................................    63      1    MOL
....................uuuggcacuagcacauuuuuAu......................................................................    72      1    MOL
....................uuuggcacuUgcacauuuuugu......................................................................    16      1    MOL
....................Guuggcacuagcacauuuuugu......................................................................    154     1    MOL
....................uuuggcacuagcacauuuuGgu......................................................................    34      1    MOL
....................uuuggcacuagcacaAuuuugu......................................................................    98      1    MOL
....................uuuggcacuagGacauuuuugu......................................................................    50      1    MOL
....................uuugAcacuagcacauuuuugu......................................................................    151     1    MOL
....................uuuggcUcuagcacauuuuugu......................................................................    22      1    MOL
....................uuuggcaGuagcacauuuuugu......................................................................    7       1    MOL
....................uuuggcacuagcacauuuCugu......................................................................    25      1    MOL
....................uuuggcacuaAcacauuuuugu......................................................................    185     1    MOL
....................uuuggUacuagcacauuuuugu......................................................................    461     1    MOL
....................uuuggcacuagcacauuGuugu......................................................................    26      1    MOL
....................uuuggcacAagcacauuuuugu......................................................................    151     1    MOL
....................uuuggcacuGgcacauuuuugu......................................................................    18      1    MOL
....................uuuggcaUuagcacauuuuugu......................................................................    79      1    MOL
....................uAuggcacuagcacauuuuugu......................................................................    8       1    MOL
....................uuuggcacuagcacauGuuugu......................................................................    36      1    MOL
....................Cuuggcacuagcacauuuuugu......................................................................    153     1    MOL
....................uuuggcGcuagcacauuuuugu......................................................................    5       1    MOL
....................uuuggcacuagcacauuuuuUu......................................................................    120     1    MOL
....................uuCggcacuagcacauuuuugu......................................................................    27      1    MOL
....................uuuggcacuaUcacauuuuugu......................................................................    17      1    MOL
....................uuuggcacuagcacauuuGugu......................................................................    10      1    MOL
....................uuuggcacuagcacauuuuAgu......................................................................    77      1    MOL
....................uuuggcacuagcaGauuuuugu......................................................................    10      1    MOL
....................uuuggGacuagcacauuuuugu......................................................................    46      1    MOL
....................uuuggcacuagUacauuuuugu......................................................................    59      1    MOL
....................uuuggcacuagAacauuuuugu......................................................................    36      1    MOL
....................uuuggcacCagcacauuuuugu......................................................................    17      1    MOL
....................uuuCgcacuagcacauuuuugu......................................................................    13      1    MOL
....................uuuAgcacuagcacauuuuugu......................................................................    33      1    MOL
....................uuuggcaAuagcacauuuuugu......................................................................    14      1    MOL
....................Auuggcacuagcacauuuuugu......................................................................    549     1    MOL
....................uCuggcacuagcacauuuuugu......................................................................    31      1    MOL
....................uuuggcacuagcacGuuuuugu......................................................................    9       1    MOL
....................uuuggcacuagcacauAuuugu......................................................................    185     1    MOL
....................uuuggcacuagcacauuuAugu......................................................................    56      1    MOL
```

Mature Star

gcuaguccucuucucuguucuuuggcacuagcacauuuuugugugacgaucaaaccuacaaaauuguguuagugucgaauaauaugggaagacauucgucauaaaucauaca			
....................uuuggcacuagcGcauuuuugu..	7	1	MOL
....................uuuggcacGagcacauuuuugu..	5	1	MOL
....................uuuggcacuagcacUuuuuugug...	1	1	MOL
....................uuuggcacuagcacauCuuugug...	2	1	MOL
....................uuuggcacuagcacauuGuugug...	2	1	MOL
....................uuuggcacuaAcacauuuuugug...	2	1	MOL
....................uuuggcacuagcaUauuuuugug...	2	1	MOL
....................Guuggcacuagcacauuuuugug...	2	1	MOL
....................uuuggcacuagcacauuuuuguU...	1469	1	MOL
....................uuuggcacuagcacauAuuugug...	1	1	MOL
....................uuuggcacuagcacauuuuuguA...	3310	1	MOL
....................uuuggcacuagcacauuuuugCg...	1	1	MOL
....................uuuggcacuagcacauuuuugGg...	2	1	MOL
....................uuuggUacuagcacauuuuugug...	2	1	MOL
....................uuuggcacuagcacauuuuugug...	1443	0	MOL
....................uuuggcacuagcacauuuuuUug...	1	1	MOL
....................uuuggcacuagcacauuuuuguC...	95	1	MOL
....................uuuggcacuagcacauuuuugAg...	3	1	MOL
....................Auuggcacuagcacauuuuugug...	6	1	MOL
....................Cuuggcacuagcacauuuuugug...	1	1	MOL
....................uuuggcacAagcacauuuuugug...	1	1	MOL
....................uuuggcacuagcacauuuuugugA..	1360	1	MOL
....................uuuggcacuagcacauuuuuguCu..	9	1	MOL
....................uuuggcacuagcacauuuuuguUu..	52	1	MOL
....................uuuggcacuagcacauuuuugugC..	1	1	MOL
....................uuuggcacuagcacauuuuuUugu..	2	1	MOL
....................uuuggcacuagcacauuuuugugG..	15	1	MOL
....................uuuggcacuagcacauuuuugugu..	63	0	MOL
....................uuuggcacuagcacauuuuuguAu..	407	1	MOL
....................uuuggcacuagcacauuuuuguguA...	2	1	MOL
....................uuuggcacuagcacauuuuuguguga..	3	0	MOL
....................uuuggcacuagcacauuuuugugugac...	26	0	MOL
.....................uuggcacuagcacauuuu...	8	0	MOL
.....................uuggcacuagcacauuGu...	1	1	MOL
.....................uuggcacuGgcacauuuuu..	1	1	MOL
.....................uuggcacuagcacauuuGu..	3	1	MOL
.....................uuggcacuagcacauuuuC..	1	1	MOL
.....................uuggcacuagcacauuuuu..	62	0	MOL
.....................uuggcacuagcacauuuuG..	1	1	MOL
.....................uuggcacuagcaUauuuuug...	1	1	MOL
.....................uuggcacuagcacauuuuuA...	1	1	MOL
.....................Cuggcacuagcacauuuuug...	1	1	MOL
.....................Auggcacuagcacauuuuug...	2	1	MOL
.....................uuggcacuagcacauuuuuU...	2	1	MOL
.....................uugAcacuagcacauuuuug...	1	1	MOL
.....................uuggcacuagcacauuuuug...	474	0	MOL
.....................uuggcacuagcacaAuuuug...	1	1	MOL
.....................uuggcacuagcacauuuGug...	2	1	MOL
.....................uuggcacuagcacauuuuugu..	10121	0	MOL
.....................uuCgcacuagcacauuuuugu..	3	1	MOL
.....................uuggcacuagcacauuuuugC..	3	1	MOL
.....................uuggcacuagcacauuuuCgu..	1	1	MOL
.....................uuggcacuagcacauuuuugA..	99	1	MOL
.....................uuggcacuaCcacauuuuugu..	4	1	MOL
.....................uuggcaGuagcacauuuuugu..	2	1	MOL
.....................uCggcacuagcacauuuuugu..	2	1	MOL
.....................uuggcacuagcacauuuGugu..	2	1	MOL
.....................uugCcacuagcacauuuuugu..	1	1	MOL
.....................uuggcacuCgcacauuuuugu..	1	1	MOL
.....................uuggcacuagGacauuuuugu..	1	1	MOL
.....................uuggUacuagcacauuuuugu..	22	1	MOL
.....................uuggcacAagcacauuuuugu..	10	1	MOL
.....................uugAcacuagcacauuuuugu..	8	1	MOL
.....................uuggcacuagcacauGuuugu..	1	1	MOL
.....................Guggcacuagcacauuuuugu..	9	1	MOL
.....................uuggcacuagcacauuuuuAu..	2	1	MOL
.....................uuggcacGagcacauuuuugu..	1	1	MOL
.....................uuggcacuagcacaAuuuugu..	2	1	MOL
.....................uuggcacuagcacauuuuugG..	7	1	MOL
.....................uuggcacuagUacauuuuugu..	1	1	MOL

Mature **Star**

gcuaguccucuucucuguucuuuggcacuagcacauuuuugugugacgaucaaaccuacaaaauuguguuagugucgaauaauaugggaagacauucgucauaaaucauaca

.....................uuggcGcuagcacauuuuugu..	2	1	MOL
.....................uuggcacuagcacauAuuugu..	5	1	MOL
.....................Auggcacuagcacauuuuugu..	36	1	MOL
.....................uGggcacuagcacauuuuugu..	4	1	MOL
.....................uuggcacuagcacauuuuuUu..	2	1	MOL
.....................uuggcacuagcacauuuuuCu..	3	1	MOL
.....................uuAgcacuagcacauuuuugu..	1	1	MOL
.....................uugUcacuagcacauuuuugu..	2	1	MOL
.....................uuggcUcuagcacauuuuugu..	2	1	MOL
.....................uuggcacuagcacauuuuAgu..	2	1	MOL
.....................uuggcaUuagcacauuuuugu..	3	1	MOL
.....................Cuggcacuagcacauuuuugu..	10	1	MOL
.....................uuggcacuUgcacauuuuugu..	4	1	MOL
.....................uuggcacuagcacUuuuuugu..	6	1	MOL
.....................uuggcacCagcacauuuuugu..	1	1	MOL
.....................uuggcacuaAcacauuuuugu..	8	1	MOL
.....................uuUgcacuagcacauuuuugu..	4	1	MOL
.....................uuggcacuagcaAauuuuugu..	3	1	MOL
.....................uuggcacuagcUcauuuuugu..	1	1	MOL
.....................uuggcacuagcacauuuAugu..	3	1	MOL
.....................uuggcacuagcacauCuuugu..	2	1	MOL
.....................uuggcacuagcaUauuuuugu..	2	1	MOL
.....................uuggcacuagcacauuAuugu..	8	1	MOL
.....................uuggcacuagcacauuuuuguC...	5	1	MOL
.....................uuggcacuagcacauuuuAgug...	2	1	MOL
.....................uuggcacuagcacauuuuuCug...	2	1	MOL
.....................uuggcacuagcacUuuuuugug...	1	1	MOL
.....................uGggcacuagcacauuuuugug...	1	1	MOL
.....................uuggcacuagcacauuuuGgug...	2	1	MOL
.....................uuggcacuagcacauuuuuguA...	239	1	MOL
.....................uuggcacuaAcacauuuuugug...	4	1	MOL
.....................uuggcacuaCcacauuuuugug...	2	1	MOL
.....................uuggcacuagcacauuuAugug...	1	1	MOL
.....................uuggcacuagcaGauuuuugug...	1	1	MOL
.....................uuggcUcuagcacauuuuugug...	3	1	MOL
.....................uuggcacAagcacauuuuugug...	3	1	MOL
.....................uuggcacuagcacauuuuugAg...	1	1	MOL
.....................Cuggcacuagcacauuuuugug...	2	1	MOL
.....................uuggcacuagcaAauuuuugug...	2	1	MOL
.....................uuggcaUuagcacauuuuugug...	2	1	MOL
.....................uuggcacuagcGcauuuuugug...	1	1	MOL
.....................uuggcacuUgcacauuuuugug...	5	1	MOL
.....................uuggcCcuagcacauuuuugug...	2	1	MOL
.....................uuggcacuagcacauuuGugug...	1	1	MOL
.....................Guggcacuagcacauuuuugug...	2	1	MOL
.....................uuUgcacuagcacauuuuugug...	1	1	MOL
.....................uugCcacuagcacauuuuugug...	1	1	MOL
.....................uuggcGcuagcacauuuuugug...	1	1	MOL
.....................uuggcacuagcacauuuuuguU...	56	1	MOL
.....................Auggcacuagcacauuuuugug...	15	1	MOL
.....................uuggcacuagcacauuAuugug...	6	1	MOL
.....................uugAcacuagcacauuuuugug...	2	1	MOL
.....................uuggcacuagcacauCuuugug...	1	1	MOL
.....................uuggcacuagUacauuuuugug...	3	1	MOL
.....................uuggcacuagcacauuuuuUug...	4	1	MOL
.....................uuggcacuagcacauuuuugug...	5672	0	MOL
.....................uuggUacuagcacauuuuugug...	8	1	MOL
.....................uuggcacCagcacauuuuugug...	1	1	MOL
.....................uuggcacuagcacauAuuugug...	3	1	MOL
.....................uuggcacuagcacauuuuugugu..	128	0	MOL
.....................uuggcacuagcacauuuuuguUu..	3	1	MOL
.....................uuggcacuagcacauuuuuguAu..	45	1	MOL
.....................uuggcacuagcacauuuuugugA..	6197	1	MOL
.....................uuggcacuagcacaCuuuugugu..	1	1	MOL
.....................uuggcacuagcacauuuuugugG..	73	1	MOL
.....................uuggcacuagcacauuuuugugC..	6	1	MOL
.....................uuggcacuagcacauuuuuUugu..	1	1	MOL
.....................uuggcacuagcacauuuuugugAg...	1	1	MOL
.....................uuggcacuagcacauuuuuguguA...	2	1	MOL
.....................uuggcacuagcacauuuuugugug...	2	0	MOL

Mature **Star**

gcuaguccucuucucuguucuuggcacuagcacauuuuugugugacgaucaaaccuacaaaauuguguuagugucgaauaauaugggaagacauucgucauaaaucauaca

....................uuggcacuagcacauuuuuguguU...	1	1	MOL
....................uuggcacuagcacauuuuugugugU..	1	1	MOL
....................uuggcacuagcacauuuuugugAga..	2	1	MOL
....................uuggcacuagcacauuuuuguguga..	3	0	MOL
....................uuggcacuagcUcauuuuugugugac...	1	1	MOL
.....................uggcacuagcacauuuuu..	1	0	MOL
.....................uUgcacuagcacauuuuug...	1	1	MOL
.....................uggcacuagcacauuuuug...	21	0	MOL
.....................uCgcacuagcacauuuuugu..	1	1	MOL
.....................Gggcacuagcacauuuuugu..	1	1	MOL
.....................uggcacuagcacUuuuuugu..	1	1	MOL
.....................uggcacuagcacauuuuugC..	1	1	MOL
.....................uggcacuagcacauuuAugu..	1	1	MOL
.....................Aggcacuagcacauuuuugu..	3	1	MOL
.....................uggcacuagcacauuuuugu..	300	0	MOL
.....................uggcacAagcacauuuuugu..	1	1	MOL
.....................uggcacuagcacauuuuugA..	2	1	MOL
.....................Cggcacuagcacauuuuugug...	2	1	MOL
.....................Aggcacuagcacauuuuugug...	1	1	MOL
.....................uggcacuagcacauuuuuguA...	15	1	MOL
.....................uUgcacuagcacauuuuugug...	1	1	MOL
.....................ugUcacuagcacauuuuugug...	1	1	MOL
.....................uggcacuagcacauuuuGgug...	1	1	MOL
.....................uggcacuagcacauuuuuguU...	3	1	MOL
.....................uggcacuagcacauuuuugug...	264	0	MOL
.....................Gggcacuagcacauuuuugug...	1	1	MOL
.....................uggcacuaAcacauuuuugug...	1	1	MOL
.....................uggcacuagcacauuuuugugu..	158	0	MOL
.....................uggcacuagcacauuuuugugC..	1	1	MOL
.....................uggcacuagcacauGuuugugu..	1	1	MOL
.....................uggcacuagcacauuuuugugG..	6	1	MOL
.....................uggcacuagcacauuuuuguAu..	3	1	MOL
.....................uggcacuagcacauuuuugugA..	418	1	MOL
.....................uggcacuagcacauuuuuguUu..	1	1	MOL
.....................ugUcacuagcacauuuuugugu..	1	1	MOL
.....................uggcacuagcacauuuuugugug...	3	0	MOL
.....................uggcacuagcacauuuuugugAg...	2	1	MOL
.....................uggcacuagcacauuuuuguguAa..	2	1	MOL
.....................uggcacuagcacauuuuuguguUa..	1	1	MOL
......................ggcacuagcacauuuuug...	7	0	MOL
......................ggcacuagcacauuuuugu..	57	0	MOL
......................ggcacuagcacUuuuuugu..	1	1	MOL
......................ggcacuagcacauuuuuguA...	2	1	MOL
......................ggcacuagcacauuuuugug...	4	0	MOL
......................ggcacuagcacauuuuuguU...	2	1	MOL
......................ggcacuagcacauuuuugugA..	2	1	MOL
.......................gcacuagcacauuuuugu..	16	0	MOL
........................cacuagcacauuuuuguU...	2	1	MOL
........................cacuagcacauuuuuguA...	8	1	MOL
........................cacuagcacauuuuugug...	1	0	MOL
........................cacuagcacauuuuugugA..	3	1	MOL
........................cacuagcacauuuuugugu..	1	0	MOL
.........................acuagcacauuuuugugA..	1	1	MOL
....aaccuacaaaauuguguuagugucgaa.................................	1	0	MOL
....aaccuacaaaauuguguuagugucgUa.................................	1	1	MOL
....ccuacaaaauuguguuagugucg...................................	1	0	MOL
....uacaaaauuguguuaguguc....................................	2	0	MOL
....uacaaaauuguguAagugucg...................................	1	1	MOL
....uacaaaauuguguuagugucg...................................	83	0	MOL
....uacaaaauuguguuUgugucg...................................	1	1	MOL
....uacaaaauuguguuagugucga..................................	15	0	MOL
....uacaaaauuguguuagugucgU..................................	8	1	MOL
....uacaaaauuguguuagugucgaaA................................	1	1	MOL
....uacaaaauuguguuagugucgaau................................	2	0	MOL
....uacaaaauuguguuagugucgaauU...............................	1	1	MOL
....acaaaauuguguuagugucg...................................	2	0	MOL
....acaaaauuguguuagugucga..................................	10	0	MOL
....acaaaauuguguuagugucgaa.................................	2	0	MOL
....acaaaauuguguuagugucgaau................................	4	0	MOL
....caaaauuguguuagugucg...................................	3	0	MOL

Mature Star

```
gcuaguccucuucucuguucuuuggcacuagcacauuuuugugugacgaucaaaccuacaaaauuguguuagugucgaauaauaugggaagacauucgucauaaaucauaca
..........................................................caaaauuguguuagugucCa..................................  1     1  MOL
..........................................................caaaauuguguuagugucga..................................  66    0  MOL
..........................................................caaaauAguguuagugucga..................................  1     1  MOL
..........................................................caaaauugAguuagugucga..................................  1     1  MOL
..........................................................caaaauuguguuagugucgaa.................................  51    0  MOL
..........................................................Aaaaauuguguuagugucgaa.................................  1     1  MOL
..........................................................caaaauuguCuuagugucgaa.................................  1     1  MOL
..........................................................caaaauugAguuagugucgaau................................  1     1  MOL
..........................................................caaaauuguguuagugucgaaC................................  6     1  MOL
..........................................................caaaauuguguuagugucgaaG................................  1     1  MOL
..........................................................caaaauuguguuaguCucgaau................................  1     1  MOL
..........................................................caaaauuguguuaUugucgaau................................  1     1  MOL
..........................................................caaaauuguguuagugucgaaA................................  16    1  MOL
..........................................................caaaauuguguuagugucgaau................................  283   0  MOL
..........................................................caaaauuguguuagugucgaUu................................  1     1  MOL
..........................................................caaaauuguguuagugucgaaua...............................  33    0  MOL
..........................................................caaaauuguguuagugucgGaua...............................  1     1  MOL
..........................................................caaaauuguguuagugucgaaAa...............................  4     1  MOL
..........................................................caaaauuguguuagugucgaaCa...............................  1     1  MOL
..........................................................caaaauuguguuagugucgaauU...............................  11    1  MOL
..........................................................caaaauuguguuagugucgaauC...............................  3     1  MOL
..........................................................caaaauuguguuaguAucgaaua...............................  1     1  MOL
..........................................................caaaauuguguuagugucgaauaU..............................  1     1  MOL
..........................................................caaaauuguguuagugucgaauaa..............................  1     0  MOL
..........................................................caaaauuguguuagugucgaauUa..............................  1     1  MOL
...........................................................aaaauuguguuagugucg...................................  10    0  MOL
...........................................................aaaauuguguuagugucAa..................................  1     1  MOL
...........................................................aaaauuguguuagugucga..................................  333   0  MOL
...........................................................aaaauuguguuUgugucga..................................  1     1  MOL
...........................................................aaaauuguguuaguguGga..................................  1     1  MOL
...........................................................aaaauGguguuagugucga..................................  1     1  MOL
...........................................................aaaauugAguuagugucga..................................  1     1  MOL
...........................................................aaaauuguguuagugucgGa.................................  2     1  MOL
...........................................................aaaauuguguuagugucgaa.................................  306   0  MOL
...........................................................aaaauuguguuagugucgaU.................................  8     1  MOL
...........................................................aaaauuguUuuagugucgaau................................  1     1  MOL
...........................................................aaaauuguguuagCgucgaau................................  1     1  MOL
...........................................................aaaauuguguuagugucAaau................................  2     1  MOL
...........................................................aaaauuguguuagugucgaaG................................  1     1  MOL
...........................................................aaaauuguguuagugucgaau................................  1550  0  MOL
...........................................................aCaauuguguuagugucgaau................................  1     1  MOL
...........................................................aaaauuguguuaguguGgaau................................  2     1  MOL
...........................................................aaaauuguguuaUugucgaau................................  1     1  MOL
...........................................................aaaauuguguuagugucUaau................................  1     1  MOL
...........................................................aaaauuguguuagugucgUau................................  2     1  MOL
...........................................................aUaauuguguuagugucgaau................................  2     1  MOL
...........................................................aaaauuguguuaguguUgaau................................  1     1  MOL
...........................................................aaaauuguguuagugucgaaA................................  110   1  MOL
...........................................................aaaauuguguuagugucgaaC................................  23    1  MOL
...........................................................aaaauuguguuaguUucgaau................................  1     1  MOL
...........................................................aaaauuguguuagAgucgaau................................  2     1  MOL
...........................................................aaaauuguguuagugucUaaua...............................  1     1  MOL
...........................................................aUaauuguguuagugucgaaua...............................  3     1  MOL
...........................................................aaaauuguguuagugucgaauC...............................  5     1  MOL
...........................................................aaaauuguguuagugAcgaaua...............................  2     1  MOL
...........................................................aaaauuguguuagugucgaauU...............................  75    1  MOL
...........................................................aaUauuguguuagugucgaaua...............................  5     1  MOL
...........................................................aaaCuuguguuagugucgaaua...............................  1     1  MOL
...........................................................aaaauuguguuagugucgaaAa...............................  56    1  MOL
...........................................................aaaauuguguuagugucgaauG...............................  7     1  MOL
...........................................................aaaauuguAuuagugucgaaua...............................  2     1  MOL
...........................................................aaaauugugCuagugucgaaua...............................  2     1  MOL
...........................................................aaaauGguguuagugucgaaua...............................  2     1  MOL
...........................................................aaaauuguUuuagugucgaaua...............................  1     1  MOL
...........................................................aaaauuguguuagCgucgaaua...............................  1     1  MOL
...........................................................aGaauuguguuagugucgaaua...............................  1     1  MOL
...........................................................aaaauuguguuagGgucgaaua...............................  1     1  MOL
...........................................................aaaauuguguuagugucgaaGa...............................  2     1  MOL
...........................................................aaaauuguguuagAgucgaaua...............................  3     1  MOL
...........................................................aaaauuguguuagugucgUaua...............................  2     1  MOL
```

```
                    Mature                                      Star
gcuaguccucuucucuguucuuuggcacuagcacauuuuugugugacgaucaaaccuacaaaauuguguuagugucgaauaauaugggaagacauucgucauaaaucauaca
...........................................................aaaauuguguuagugucgaaCa...............................	32	1	MOL
...........................................................aaaauuguguuagugucgaaua...............................	1248	0	MOL
...........................................................aaaauugAguuagugucgaaua...............................	2	1	MOL
...........................................................aaaauuguguuagugucgaauaU..............................	12	1	MOL
...........................................................aaaauuguguuagugucgaauaa..............................	22	0	MOL
...........................................................aaaauuguguuagugucgaauCa..............................	2	1	MOL
...........................................................aaaauuguguuagugucgaauaC..............................	4	1	MOL
...........................................................aaaauuguguuagugucgaaAaa..............................	5	1	MOL
...........................................................aaaauuguguuagugucgaauGa..............................	1	1	MOL
...........................................................aaaauuguguuagugucgaauaG..............................	1	1	MOL
...........................................................aaaauuguguuagugucgaauUa..............................	7	1	MOL
...........................................................aaaauuguguuagugucgaauaaA.............................	1	1	MOL
...........................................................aaaauuguguuagugucgaauGau.............................	1	1	MOL
...........................................................aaaauuguguuagugucgaauaUu.............................	2	1	MOL
...........................................................aaaauuguguuagugucgaauaaC.............................	1	1	MOL
............................................................aaauuguguuagugucgaa.................................	3	0	MOL
............................................................aaauuguguuagugucgaau................................	55	0	MOL
............................................................aaauuguguuagugucgaUu................................	1	1	MOL
............................................................aaauuguguuagugucgaaA................................	4	1	MOL
............................................................aaauuguguuagugucgaauU...............................	3	1	MOL
............................................................aaauuguguuagugucgaaAa...............................	2	1	MOL
............................................................aaauuguguuagugCcgaaua...............................	1	1	MOL
............................................................aGauuguguuagugucgaaua...............................	1	1	MOL
............................................................aaauuguCuuagugucgaaua...............................	1	1	MOL
............................................................aaauuguguuagugucgaaCa...............................	1	1	MOL
............................................................aaauugAguuagugucgaaua...............................	1	1	MOL
............................................................aaauuguguuagugucgaauC...............................	1	1	MOL
............................................................aaauuguguuagugucgaaua...............................	426	0	MOL
............................................................aaauuguguuagAgucgaaua...............................	1	1	MOL
............................................................aaauuguguuagugucgaauaU..............................	5	1	MOL
............................................................aaauuguguuagugucgaauaa..............................	8	0	MOL
............................................................aaauuguguuagugucgaauaC..............................	4	1	MOL
............................................................aaauuguguuagugucgaauaUu.............................	1	1	MOL
.............................................................aauuguguuagugucgaauU...............................	1	1	MOL
.............................................................aauuguguuagugucgaaua...............................	2	0	MOL
.............................................................aauuguguuagugucgaauaU..............................	1	1	MOL
..............................................................auuguguuagugucgaaua...............................	1	0	MOL

..................uUuuuggcacuagcacauuuu.........................................................................	1	1	te1
..................uUuuuggcacuagcacauuuuu........................................................................	2	1	te1
..................ucuuuggcacuagcacauuuuug.......................................................................	1	0	te1
..................uUuuuggcacuagcacauuuuug.......................................................................	6	1	te1
..................ucuuuggcacuagcacauuuuugu......................................................................	1	0	te1
..................Gcuuuggcacuagcacauuuuugu......................................................................	1	1	te1
..................uUuuuggcacuagcacauuuuugu......................................................................	49	1	te1
..................uGuuuggcacuagcacauuuuugu......................................................................	1	1	te1
...................cuuuggcacuagcacauuuu.........................................................................	3	0	te1
...................cuuuggcacuagcacauuuuu........................................................................	57	0	te1
...................Uuuuggcacuagcacauuuuu........................................................................	12	1	te1
...................Auuuggcacuagcacauuuuug.......................................................................	1	1	te1
...................cuuuggcacuagcacauuuuuU.......................................................................	4	1	te1
...................Uuuuggcacuagcacauuuuug.......................................................................	61	1	te1
...................cuuuggcacuagcacauuuuug.......................................................................	21	0	te1
...................Guuuggcacuagcacauuuuug.......................................................................	2	1	te1
...................cuuuggcacuagcacauuuuuA.......................................................................	1	1	te1
...................cuuuCgcacuagcacauuuuugu......................................................................	1	1	te1
...................Guuuggcacuagcacauuuuugu......................................................................	4	1	te1
...................cuuuggcacuagcacauuuuugA......................................................................	8	1	te1
...................Uuuuggcacuagcacauuuuugu......................................................................	299	1	te1
...................cuuuggcacuagcacauuuuugu......................................................................	46	0	te1
...................Auuuggcacuagcacauuuuugu......................................................................	6	1	te1
...................cuuuggcacuagcacauuuCugu......................................................................	1	1	te1
...................cuuuggcacuagcacauuuuuUu......................................................................	1	1	te1
...................Guuuggcacuagcacauuuuugug.....................................................................	1	1	te1
...................cuuuggcacuagcacauuuuuguA.....................................................................	1	1	te1
...................cuuuggcacuagcacauuuuuguU.....................................................................	1	1	te1
...................Uuuuggcacuagcacauuuuugug.....................................................................	1	1	te1
...................Uuuuggcacuagcacauuuuugugu....................................................................	1	1	te1
....................uuuggcacuagcacauuG..........................................................................	14	1	te1
....................uuuggcacuagcacauuu..........................................................................	20	0	te1
```

```
                    Mature                                 Star
gcuaguccucuucucuguucuuuggcacuagcacauuuuugugugacgaucaaaccuacaaaauuguguuagugucgaauaauaugggaagacauucgucauaaaucauaca
....................uuuggcacuagcacauGu..........................................................................    34      1   te1
....................uuuggcaUuagcacauuuu.........................................................................    1       1   te1
....................uuugCcacuagcacauuuu.........................................................................    1       1   te1
....................uuGggcacuagcacauuuu.........................................................................    1       1   te1
....................uuuggcacuagcacauuuu.........................................................................    384     0   te1
....................uuuggcacuagcacauuGu.........................................................................    117     1   te1
....................uuuggUacuagcacauuuu.........................................................................    2       1   te1
....................Cuuggcacuagcacauuuu.........................................................................    2       1   te1
....................uuuggcacuagcacauuuG.........................................................................    155     1   te1
....................uuuggcacuagcacGuuuuu........................................................................    2       1   te1
....................Auuggcacuagcacauuuuu........................................................................    34      1   te1
....................uuuggcacuagcacauuuuA........................................................................    15      1   te1
....................uuuggcacuagcaUauuuuu........................................................................    11      1   te1
....................uuuggcacuagcacauuAuu........................................................................    1       1   te1
....................uuuggcacuagcacauGuuu........................................................................    1       1   te1
....................uuuggcacuUgcacauuuuu........................................................................    2       1   te1
....................uCuggcacuagcacauuuuu........................................................................    5       1   te1
....................uuuggcacuagcacauuCuu........................................................................    2       1   te1
....................uuuggcacAagcacauuuuu........................................................................    1       1   te1
....................uuuggcacuCgcacauuuuu........................................................................    2       1   te1
....................uuuggcacuagcacauuuGu........................................................................    493     1   te1
....................uuGggcacuagcacauuuuu........................................................................    8       1   te1
....................uuugAcacuagcacauuuuu........................................................................    10      1   te1
....................uuuggcUcuagcacauuuuu........................................................................    2       1   te1
....................uuuggcacuagcacauuuuu........................................................................    18047   0   te1
....................uuuggcacuagcaGauuuuu........................................................................    1       1   te1
....................uuuCgcacuagcacauuuuu........................................................................    4       1   te1
....................Guuggcacuagcacauuuuu........................................................................    27      1   te1
....................uuuUgcacuagcacauuuuu........................................................................    1       1   te1
....................uuugUcacuagcacauuuuu........................................................................    5       1   te1
....................uuAggcacuagcacauuuuu........................................................................    1       1   te1
....................uuuggcacuagcacauuuuG........................................................................    155     1   te1
....................uuuggcacuagcUcauuuuu........................................................................    1       1   te1
....................uuuggcacuaAcacauuuuu........................................................................    10      1   te1
....................uuuggcacuagcacaAuuuu........................................................................    12      1   te1
....................uuuggcacuGgcacauuuuu........................................................................    3       1   te1
....................Cuuggcacuagcacauuuuu........................................................................    39      1   te1
....................uuuggcaUuagcacauuuuu........................................................................    4       1   te1
....................uuuggcacCagcacauuuuu........................................................................    2       1   te1
....................uuuggcacuagcacUuuuuu........................................................................    1       1   te1
....................uuCggcacuagcacauuuuu........................................................................    1       1   te1
....................uuuggcacuagGacauuuuu........................................................................    1       1   te1
....................uuuggcacuaCcacauuuuu........................................................................    2       1   te1
....................uuuggcacuagAacauuuuu........................................................................    6       1   te1
....................uuuggcacuagcacauuuuC........................................................................    1       1   te1
....................uuuggcacuagcacauuGuu........................................................................    3       1   te1
....................uuuggcGcuagcacauuuuu........................................................................    1       1   te1
....................uuuggUacuagcacauuuuu........................................................................    34      1   te1
....................uuuggcacuagUacauuuuu........................................................................    15      1   te1
....................uuugCcacuagcacauuuuu........................................................................    4       1   te1
....................uAuggcacuagcacauuuuu........................................................................    2       1   te1
....................uuuggcacuagcacauuuAu........................................................................    2       1   te1
....................uuuggcacuagcacaCuuuu........................................................................    1       1   te1
....................uGuggcacuagcacauuuuu........................................................................    8       1   te1
....................uuuggcacuagcacauAuuu........................................................................    1       1   te1
....................uuuggcacAagcacauuuuug.......................................................................    1       1   te1
....................Cuuggcacuagcacauuuuug.......................................................................    250     1   te1
....................uuuggcacuUgcacauuuuug.......................................................................    2       1   te1
....................uuuggcacuGgcacauuuuug.......................................................................    16      1   te1
....................uuCggcacuagcacauuuuug.......................................................................    12      1   te1
....................uuuggcacuagcacauuuuuU.......................................................................    443     1   te1
....................uuugCcacuagcacauuuuug.......................................................................    24      1   te1
....................uuuggcacuagcacauuuuGg.......................................................................    13      1   te1
....................uuuggcacuagcacauuuuAg.......................................................................    1       1   te1
....................uGuggcacuagcacauuuuug.......................................................................    36      1   te1
....................uuuggGacuagcacauuuuug.......................................................................    1       1   te1
....................uuuggcacuagcacauuuuuA.......................................................................    180     1   te1
....................uuuggcacuagcacauuGuug.......................................................................    16      1   te1
....................uuuggcacuagcacUuuuuug.......................................................................    16      1   te1
....................uuugAcacuagcacauuuuug.......................................................................    36      1   te1
```

Mature **Star**

gcuaguccucuucucuguucuuuggcacuagcacauuuuugugugacgaucaaaccuacaaaauuguguuagugucgaauaauaugggaagacauucgucauaaaucauaca			
....................uuuCgcacuagcacauuuuug...	27	1	te1
....................uuuggcacuagcacauCuuug...	9	1	te1
....................uAuggcacuagcacauuuuug...	17	1	te1
....................Auuggcacuagcacauuuuug...	197	1	te1
....................uuuggcacuagGacauuuuug...	22	1	te1
....................uuuggcacuagcacauuAuug...	4	1	te1
....................uuuggcacuagAacauuuuug...	32	1	te1
....................uuuggcacuaAcacauuuuug...	69	1	te1
....................uuuggcacuagcaAauuuuug...	1	1	te1
....................uuuggcacuagcacauAuuug...	8	1	te1
....................uuuggcacGagcacauuuuug...	1	1	te1
....................uuuggcaUuagcacauuuuug...	19	1	te1
....................uuuggUacuagcacauuuuug...	158	1	te1
....................uuuggcacuagcacaAuuuug...	33	1	te1
....................uuuggcacuagcaGauuuuug...	10	1	te1
....................uuuggcGcuagcacauuuuug...	6	1	te1
....................uuuggcacuagcUcauuuuug...	3	1	te1
....................uuuggcacuagcacauuuuug...	100372	0	te1
....................uuuggcacuaCcacauuuuug...	22	1	te1
....................uuuggcaGuagcacauuuuug...	7	1	te1
....................uuuggcacuagcaUauuuuug...	24	1	te1
....................uuuggcacuagcacaGuuuug...	1	1	te1
....................uuuggcacuagcacauuuGug...	6	1	te1
....................uuuggcCcuagcacauuuuug...	3	1	te1
....................uCuggcacuagcacauuuuug...	19	1	te1
....................uuuggcacuagcacauuuuuC...	13	1	te1
....................Guuggcacuagcacauuuuug...	146	1	te1
....................uuuggcacuaUcacauuuuug...	7	1	te1
....................uuugUcacuagcacauuuuug...	23	1	te1
....................uuuggcacuagcacauuuAug...	1	1	te1
....................uuuggcacCagcacauuuuug...	2	1	te1
....................uuuggcacuagcacauuuuCg...	10	1	te1
....................uuuggcacuagcacauuCuug...	22	1	te1
....................uuuggcUcuagcacauuuuug...	5	1	te1
....................uuuggcacuCgcacauuuuug...	7	1	te1
....................uuuggcacuagUacauuuuug...	46	1	te1
....................uuuggcacuagcacauGuuug...	3	1	te1
....................uuuUgcacuagcacauuuuug...	4	1	te1
....................uuuggcacuagcacaCuuuug...	7	1	te1
....................uuAggcacuagcacauuuuug...	7	1	te1
....................uuGggcacuagcacauuuuug...	43	1	te1
....................uuuggAacuagcacauuuuug...	2	1	te1
....................uuuggcacuagcacGuuuuug...	2	1	te1
....................uuuggcacuagcacauuuCug...	10	1	te1
....................uuuAgcacuagcacauuuuug...	9	1	te1
....................uuuggcacuagcacaCuuuugu..	39	1	te1
....................uuuggGacuagcacauuuuugu..	7	1	te1
....................uuuggUacuagcacauuuuugu..	819	1	te1
....................uuugAcacuagcacauuuuugu..	346	1	te1
....................uuuggcacuagcacauuGuugu..	33	1	te1
....................uuuggcacuagcacCuuuuugu..	6	1	te1
....................uuuggcacuaUcacauuuuugu..	44	1	te1
....................uuuggcacuagcacauuuGugu..	158	1	te1
....................uuuggcacuagcacauuCuugu..	71	1	te1
....................uuuggcacuUgcacauuuuugu..	10	1	te1
....................uuuggcacuagcacauuuuGgu..	51	1	te1
....................uuugCcacuagcacauuuuugu..	118	1	te1
....................uuuggcacuagcacUuuuuugu..	59	1	te1
....................uuuggcacuagcacauuuuuCu..	44	1	te1
....................uuugUcacuagcacauuuuugu..	84	1	te1
....................uuuggcacuagcacauAuuugu..	47	1	te1
....................uuuggcaGuagcacauuuuugu..	50	1	te1
....................uuuggcaAuagcacauuuuugu..	3	1	te1
....................uuuggcacuagcacauGuuugu..	29	1	te1
....................uuuggcacuagcaUauuuuugu..	121	1	te1
....................uuuggcacuagcacauuuuugA..	13922	1	te1
....................uuuggcacuagcacauuuuugu..	577875	0	te1
....................Auuggcacuagcacauuuuugu..	1358	1	te1
....................uuuggcacuagcacauuuCugu..	164	1	te1
....................uuuggcacuagcacauuuuuAu..	39	1	te1

```
                    Mature                                  Star
gcuaguccucuucucuguucuuuggcacuagcacauuuuugugugacgaucaaaccuacaaaauuguguuagugucgaauaauaugggaagacauucgucauaaaucauaca
....................uuuggcacuaAcacauuuuugu......................................................................    475     1   te1
....................uuuggcacuagcacauuuuuUu......................................................................    60      1   te1
....................uuuggcacuagcacaGuuuugu......................................................................    9       1   te1
....................uuuggcCcuagcacauuuuugu......................................................................    18      1   te1
....................uuuggcacAagcacauuuuugu......................................................................    1       1   te1
....................uuAggcacuagcacauuuuugu......................................................................    13      1   te1
....................uuuggcacuagcUcauuuuugu......................................................................    17      1   te1
....................uuuggcacuagGacauuuuugu......................................................................    73      1   te1
....................uuuggcacuagcacauuuuCgu......................................................................    58      1   te1
....................uuuggcacuagUacauuuuugu......................................................................    212     1   te1
....................uGuggcacuagcacauuuuugu......................................................................    184     1   te1
....................uuuggcacuagcaGauuuuugu......................................................................    24      1   te1
....................uuuggcacuagcacauuuuAgu......................................................................    4       1   te1
....................uuuUgcacuagcacauuuuugu......................................................................    46      1   te1
....................uuuggcacuagcacauuuAugu......................................................................    40      1   te1
....................uuuggcacuagAacauuuuugu......................................................................    193     1   te1
....................uuuggcacuagcGcauuuuugu......................................................................    5       1   te1
....................uCuggcacuagcacauuuuugu......................................................................    157     1   te1
....................uuuggAacuagcacauuuuugu......................................................................    2       1   te1
....................uuuggcacuaCcacauuuuugu......................................................................    89      1   te1
....................uuuggcUcuagcacauuuuugu......................................................................    26      1   te1
....................uuuggcacuagcacauuuuugG......................................................................    664     1   te1
....................uuuggcacCagcacauuuuugu......................................................................    29      1   te1
....................uAuggcacuagcacauuuuugu......................................................................    70      1   te1
....................uuuggcacuagcacauuuuugC......................................................................    240     1   te1
....................uuuggcacuagcacaAuuuugu......................................................................    59      1   te1
....................uuuggcacuGgcacauuuuugu......................................................................    74      1   te1
....................uuuAgcacuagcacauuuuugu......................................................................    40      1   te1
....................uuuggcacuagcacGuuuuugu......................................................................    9       1   te1
....................Guuggcacuagcacauuuuugu......................................................................    956     1   te1
....................uuuggcaUuagcacauuuuugu......................................................................    150     1   te1
....................uuuggcacuagcacauuAuugu......................................................................    6       1   te1
....................Cuuggcacuagcacauuuuugu......................................................................    1645    1   te1
....................uuuggcacGagcacauuuuugu......................................................................    9       1   te1
....................uuGggcacuagcacauuuuugu......................................................................    179     1   te1
....................uuuggcGcuagcacauuuuugu......................................................................    52      1   te1
....................uuuCgcacuagcacauuuuugu......................................................................    119     1   te1
....................uuuggcacuagcacauCuuugu......................................................................    43      1   te1
....................uuuggcacuagcaAauuuuugu......................................................................    2       1   te1
....................uuuggcacuCgcacauuuuugu......................................................................    15      1   te1
....................uuCggcacuagcacauuuuugu......................................................................    58      1   te1
....................uuuggcacuagcacauuuuuguC.....................................................................    55      1   te1
....................uuuggcacuagcacauuuuugCg.....................................................................    1       1   te1
....................uuuggcacuagcacauuuuCgug.....................................................................    1       1   te1
....................uuuggcacuagcacauuuuuguA.....................................................................    6414    1   te1
....................uuuggcacuagcacauuCuugug.....................................................................    1       1   te1
....................uuuggcacuaAcacauuuuugug.....................................................................    1       1   te1
....................uuuggcacuagcacauuuuGgug.....................................................................    1       1   te1
....................uuuggcacuaUcacauuuuugug.....................................................................    1       1   te1
....................uGuggcacuagcacauuuuugug.....................................................................    1       1   te1
....................Cuuggcacuagcacauuuuugug.....................................................................    3       1   te1
....................uuuggcacuagcacauuuuugug.....................................................................    783     0   te1
....................uuGggcacuagcacauuuuugug.....................................................................    1       1   te1
....................Auuggcacuagcacauuuuugug.....................................................................    1       1   te1
....................uuuggcacuagcacauuuuuguU.....................................................................    5303    1   te1
....................uuuggcacuagcacauuuuugAg.....................................................................    8       1   te1
....................uuuggUacuagcacauuuuugug.....................................................................    1       1   te1
....................Cuuggcacuagcacauuuuugugu....................................................................    1       1   te1
....................uuuggcacuagcacauuuuugugG....................................................................    16      1   te1
....................uuuggcacuagcacauuuuuguCu....................................................................    6       1   te1
....................uuuggcacuagcacauuuuugugC....................................................................    4       1   te1
....................uuuggcacuagcacauuuuugugA....................................................................    1211    1   te1
....................uuuggcacuagcacauuGuugugu....................................................................    1       1   te1
....................uuuggcacuagcacauuuuuguAu....................................................................    1213    1   te1
....................uGuggcacuagcacauuuuugugu....................................................................    1       1   te1
....................uuuggcacuagcacauuuuuguUu....................................................................    246     1   te1
....................uuuggcacuagcacauuuuugugu....................................................................    163     0   te1
....................uuuggcacuagcacauuuuuUugu....................................................................    5       1   te1
....................uuuggcacuagcacauuuuugugug...................................................................    2       0   te1
....................uuuggcacuagcacauuuuugugCg...................................................................    1       1   te1
```

Mature **Star**

```
gcuaguccucuucucuguucuuuggcacuagcacauuuuugugugacgaucaaaccuacaaaauuguguuagugucgaauaauaugggaagacauucgucauaaaucauaca
....................uuuggcacuagcacauuuuuguguA...................................................................  2     1  te1
....................uuuggcacuagcacauuuuuguUug...................................................................  1     1  te1
....................uuuCgcacuagcacauuuuuguguga..................................................................  1     1  te1
....................uuuggcacuagcacauuuuugugugU..................................................................  1     1  te1
....................uuuggcacuagcacauuuuuguguAa..................................................................  1     1  te1
....................uuuggcacuagcacauuuuuguguga..................................................................  6     0  te1
....................Auuggcacuagcacauuuuugugugac.................................................................  1     1  te1
....................uuuggcacuagcacauuuuugugugac.................................................................  51    0  te1
....................uuuggcacuagcacauuuuugugugaA.................................................................  1     1  te1
.....................uuggcacuagcacauuGu.........................................................................  7     1  te1
.....................uuggcacuagcacauuuG.........................................................................  3     1  te1
.....................uuggcacuagcacauuuu.........................................................................  12    0  te1
.....................uuggcacuagcacauuuuGu.......................................................................  16    1  te1
.....................uuggcacuagcacauuuuuG.......................................................................  3     1  te1
.....................uuggcacuagcaUauuuuu........................................................................  1     1  te1
.....................Guggcacuagcacauuuuu........................................................................  2     1  te1
.....................Cuggcacuagcacauuuuu........................................................................  2     1  te1
.....................uuggcacuagcacauuuuu........................................................................  86    0  te1
.....................Auggcacuagcacauuuuu........................................................................  2     1  te1
.....................uuggcacuagcacauuuuuA.......................................................................  1     1  te1
.....................Cuggcacuagcacauuuuug.......................................................................  7     1  te1
.....................uuggcacuagcacauuuuug.......................................................................  1234  0  te1
.....................uuggUacuagcacauuuuug.......................................................................  1     1  te1
.....................uuggcacuCgcacauuuuug.......................................................................  1     1  te1
.....................uugAcacuagcacauuuuug.......................................................................  1     1  te1
.....................uuggAacuagcacauuuuug.......................................................................  1     1  te1
.....................uuggcacuGgcacauuuuug.......................................................................  1     1  te1
.....................uuUgcacuagcacauuuuug.......................................................................  1     1  te1
.....................uuggcaUuagcacauuuuug.......................................................................  2     1  te1
.....................uuggGacuagcacauuuuug.......................................................................  1     1  te1
.....................Auggcacuagcacauuuuug.......................................................................  9     1  te1
.....................uuggcacuagcacauuuuuU.......................................................................  4     1  te1
.....................Guggcacuagcacauuuuug.......................................................................  7     1  te1
.....................uuggAacuagcacauuuuugu......................................................................  7     1  te1
.....................uuggcacuagcacaAuuuugu......................................................................  7     1  te1
.....................uuggcacuagcacauuuuCgu......................................................................  2     1  te1
.....................uuggcacuagUacauuuuugu......................................................................  7     1  te1
.....................uuggcacuagcaGauuuuugu......................................................................  3     1  te1
.....................uuggcacuagcacauuuuugA......................................................................  227   1  te1
.....................uuggcacuagcacauuAuugu......................................................................  2     1  te1
.....................uuggGacuagcacauuuuugu......................................................................  2     1  te1
.....................uugCcacuagcacauuuuugu......................................................................  1     1  te1
.....................uuggcacuCgcacauuuuugu......................................................................  1     1  te1
.....................uuggcacuagcacaCuuuugu......................................................................  4     1  te1
.....................uuUgcacuagcacauuuuugu......................................................................  15    1  te1
.....................uGggcacuagcacauuuuugu......................................................................  7     1  te1
.....................uuggcaUuagcacauuuuugu......................................................................  8     1  te1
.....................uuggcacuagcacauuuuuUu......................................................................  3     1  te1
.....................uuggcacuagcaAauuuuugu......................................................................  1     1  te1
.....................uAggcacuagcacauuuuugu......................................................................  4     1  te1
.....................uuggcacuagcacauCuuugu......................................................................  1     1  te1
.....................uuggcacuagGacauuuuugu......................................................................  5     1  te1
.....................uuggcacuagcaUauuuuugu......................................................................  8     1  te1
.....................uuggcacuagcacauGuuugu......................................................................  1     1  te1
.....................uuggcacuaCcacauuuuugu......................................................................  1     1  te1
.....................uuggcacuaAcacauuuuugu......................................................................  31    1  te1
.....................Auggcacuagcacauuuuugu......................................................................  103   1  te1
.....................uuggcUcuagcacauuuuugu......................................................................  2     1  te1
.....................uuggcacuGgcacauuuuugu......................................................................  5     1  te1
.....................uuggcacuagcGcauuuuugu......................................................................  1     1  te1
.....................uuggcacGagcacauuuuugu......................................................................  3     1  te1
.....................uuggcacuagcacauuuuuAu......................................................................  1     1  te1
.....................uuggcacuagcacauuGuugu......................................................................  3     1  te1
.....................uuggcacuagcacauAuuugu......................................................................  1     1  te1
.....................uugAcacuagcacauuuuugu......................................................................  15    1  te1
.....................uuggcacuagcacauuuAugu......................................................................  2     1  te1
.....................uuggUacuagcacauuuuugu......................................................................  29    1  te1
.....................uuggcaGuagcacauuuuugu......................................................................  2     1  te1
.....................uugUcacuagcacauuuuugu......................................................................  6     1  te1
.....................uuCgcacuagcacauuuuugu......................................................................  3     1  te1
```

Mature **Star**

gcuaguccucuucucuguucuuuggcacuagcacauuuuugugugacgaucaaaccuacaaaauuguguuagugucgaauaauaugggaagacauucgucauaaaucauaca			
.....................uuggcacuagcacauuuuGgu..	4	1	te1
.....................Guggcacuagcacauuuuugu..	103	1	te1
.....................uuggcCcuagcacauuuuugu..	1	1	te1
.....................Cuggcacuagcacauuuuugu..	145	1	te1
.....................uuggcacuagcacauuuuuCu..	2	1	te1
.....................uuggcacuagcacauuuCugu..	2	1	te1
.....................uuggcacuagcacauuuuugG..	26	1	te1
.....................uuggcacuaUcacauuuuugu..	2	1	te1
.....................uuggcGcuagcacauuuuugu..	1	1	te1
.....................uuAgcacuagcacauuuuugu..	2	1	te1
.....................uuggcacuagcacauuuuugC..	12	1	te1
.....................uuggcacuagcacauuuGugu..	11	1	te1
.....................uCggcacuagcacauuuuugu..	6	1	te1
.....................uuggcacuagAacauuuuugu..	2	1	te1
.....................uuggcacuagcacUuuuuugu..	4	1	te1
.....................uuggcacuUgcacauuuuugu..	1	1	te1
.....................uuggcacuagcacauuuuugu..	26321	0	te1
.....................uuggcacuagcacauuuuuguA...	593	1	te1
.....................uuggcacuagcacauuuuCgug...	4	1	te1
.....................Cuggcacuagcacauuuuugug...	12	1	te1
.....................uuggcacuagcacauuuuugAg...	1	1	te1
.....................uuggcacuagcacauuuuuUug...	1	1	te1
.....................uuggcacuagcacauuuAugug...	1	1	te1
.....................uCggcacuagcacauuuuugug...	1	1	te1
.....................uuggUacuagcacauuuuugug...	3	1	te1
.....................Auggcacuagcacauuuuugug...	6	1	te1
.....................uGggcacuagcacauuuuugug...	3	1	te1
.....................uuggcacuagcacauuuGgug..	4	1	te1
.....................uuggcUcuagcacauuuuugug...	1	1	te1
.....................uuggcacuagGacauuuuugug...	1	1	te1
.....................uuggcacuagcacauuuCugug...	2	1	te1
.....................uugAcacuagcacauuuuugug...	3	1	te1
.....................uuggcacuagcacauuuuugGg...	2	1	te1
.....................uuggcacuagcacauuuuugCg...	2	1	te1
.....................uuggcacuagcacauuuuuguC...	8	1	te1
.....................uuggcacuagAacauuuuugug...	1	1	te1
.....................uuggcacuagcacauGuuugug...	1	1	te1
.....................uuggcacuagcacauuuuuguU...	196	1	te1
.....................uuggcacuaUcacauuuuugug...	1	1	te1
.....................uuggAacuagcacauuuuugug...	1	1	te1
.....................uuCgcacuagcacauuuuugug...	1	1	te1
.....................uuggcacuagcacauuGuugug...	1	1	te1
.....................uuggcacuaAcacauuuuugug...	3	1	te1
.....................uuggcacuagcacauuuuugug...	4136	0	te1
.....................uuggcacuagUacauuuuugug...	2	1	te1
.....................Guggcacuagcacauuuuugug...	11	1	te1
.....................uuggcacuagcacauuuuuUugu..	1	1	te1
.....................uuggcacuagcacauuuuGgugu..	1	1	te1
.....................uuggcacuagcacauuuuugugG..	198	1	te1
.....................uuggcacuagcacauuuuugugu..	600	0	te1
.....................uuggcacuagcacauuuuuguCu..	1	1	te1
.....................uuggcacuagcacUuuuuugugu..	1	1	te1
.....................Cuggcacuagcacauuuuugugu..	4	1	te1
.....................uuggcacuagcacauuuuuguAu..	105	1	te1
.....................uuggcacuagcacauuuuuguUu..	12	1	te1
.....................uuggcacuagcacauuuuugugC..	22	1	te1
.....................uuggcacuaAcacauuuuugugu..	1	1	te1
.....................Guggcacuagcacauuuuugugu..	1	1	te1
.....................uuggcacuCgcacauuuuugugu..	1	1	te1
.....................uuggcacuagcacauuuuugugA..	10161	1	te1
.....................uuggcacuagcacauuuuugugAg...	9	1	te1
.....................uuggcacuagcacauuuuuguguU...	2	1	te1
.....................uuggcacuagcacauuuuugugCg...	1	1	te1
.....................uuggcacuagcacauuuuugugGg...	1	1	te1
.....................uuggcacuagcacauuuuugugug...	1	0	te1
.....................uuggcacuagcacauuuuuguguA...	3	1	te1
.....................uuggcacuagcacauuuuuguguAa..	3	1	te1
.....................uuggcacuagcacauuuuugugAga..	6	1	te1
.....................uuggcacuagcacauuuuugugugac...	1	0	te1
......................uggcacuagcacauuuuu..	4	0	te1

```
                    Mature                                Star
gcuaguccucuucucuguucuuggcacuagcacauuuuugugugacgaucaaaccuacaaaauuguguuagugucgaauaauaugggaagacauucgucauaaaucauaca
.....................uUgcacuagcacauuuuug.......................................................................   1      1   te1
.....................uggcacuagcacauuuuug.......................................................................   73     0   te1
.....................Cggcacuagcacauuuuug.......................................................................   1      1   te1
.....................Gggcacuagcacauuuuug.......................................................................   2      1   te1
.....................Aggcacuagcacauuuuug.......................................................................   1      1   te1
.....................Cggcacuagcacauuuuugu......................................................................   13     1   te1
.....................uggcacuagcacauuuuugu......................................................................   1075   0   te1
.....................uggcacuagcacUuuuuugu......................................................................   1      1   te1
.....................uggcacuagcaGauuuuugu......................................................................   1      1   te1
.....................ugUcacuagcacauuuuugu......................................................................   1      1   te1
.....................uAgcacuagcacauuuuugu......................................................................   1      1   te1
.....................Aggcacuagcacauuuuugu......................................................................   13     1   te1
.....................uUgcacuagcacauuuuugu......................................................................   2      1   te1
.....................uggcacuagcacauuuuugC......................................................................   2      1   te1
.....................uggcacuaAcacauuuuugu......................................................................   2      1   te1
.....................Gggcacuagcacauuuuugu......................................................................   14     1   te1
.....................uggcacuagcacauuuuugA......................................................................   8      1   te1
.....................uggcacuagcacauuuuugug.....................................................................   400    0   te1
.....................Gggcacuagcacauuuuugug.....................................................................   2      1   te1
.....................Aggcacuagcacauuuuugug.....................................................................   2      1   te1
.....................Cggcacuagcacauuuuugug.....................................................................   1      1   te1
.....................uggcacuagcacauuuuuguU.....................................................................   12     1   te1
.....................uggcacuagcacauuuuuguC.....................................................................   1      1   te1
.....................uggcacuagcacauuuuuguA.....................................................................   21     1   te1
.....................uggUacuagcacauuuuugugu....................................................................   1      1   te1
.....................uggcacuagcacauuuuugugA....................................................................   1202   1   te1
.....................uggcacuagcacauuuuuguAu....................................................................   8      1   te1
.....................uggcacuagUacauuuuugugu....................................................................   1      1   te1
.....................ugAcacuagcacauuuuugugu....................................................................   1      1   te1
.....................uggcacuagcacauuuuuguUu....................................................................   1      1   te1
.....................uggcacuagcacauuuuCgugu....................................................................   1      1   te1
.....................Cggcacuagcacauuuuugugu....................................................................   2      1   te1
.....................uAgcacuagcacauuuuugugu....................................................................   1      1   te1
.....................uggcacuagcacauuuuugugG....................................................................   19     1   te1
.....................uggcacuagcacauuuuAgugu....................................................................   1      1   te1
.....................uggcacuagcacauAuuugugu....................................................................   1      1   te1
.....................uggcacuagcGcauuuuugugu....................................................................   1      1   te1
.....................uggcGcuagcacauuuuugugu....................................................................   2      1   te1
.....................Gggcacuagcacauuuuugugu....................................................................   3      1   te1
.....................uggcacuagcacauuuuugugu....................................................................   1286   0   te1
.....................uggcaUuagcacauuuuugugu....................................................................   1      1   te1
.....................Aggcacuagcacauuuuugugu....................................................................   8      1   te1
.....................uggcacuagcaAauuuuugugu....................................................................   1      1   te1
.....................uggcacuaAcacauuuuugugu....................................................................   2      1   te1
.....................uggcacuagcacauuuuugugug...................................................................   7      0   te1
.....................uggcacuagcacauuuuuguguU...................................................................   6      1   te1
.....................uggcacuagcacauuuuugugAg...................................................................   3      1   te1
.....................uggcacuagcacauuuuuguguA...................................................................   14     1   te1
.....................uggcacuagcacauuuuuguguAa..................................................................   6      1   te1
.....................uggcacuagcacauuuuuguguUa..................................................................   3      1   te1
.....................uggcacuagcacauuuuuguguga..................................................................   6      0   te1
......................ggcacuagcacauuuuug.......................................................................   34     0   te1
......................Ugcacuagcacauuuuugu......................................................................   3      1   te1
......................ggcacuagcacauuuuGgu......................................................................   1      1   te1
......................ggGacuagcacauuuuugu......................................................................   1      1   te1
......................Agcacuagcacauuuuugu......................................................................   2      1   te1
......................ggcacuagUacauuuuugu......................................................................   1      1   te1
......................ggcacuGgcacauuuuugu......................................................................   1      1   te1
......................ggcacuagcacauuuuugA......................................................................   6      1   te1
......................ggUacuagcacauuuuugu......................................................................   1      1   te1
......................ggcacuagcacauuuuugu......................................................................   503    0   te1
......................ggcacuagcacauuuuugG......................................................................   1      1   te1
......................ggcacuagcacauuuuugug.....................................................................   8      0   te1
......................ggcacuagcacauuuuuguA.....................................................................   3      1   te1
......................ggcacuagcacauuuuuguU.....................................................................   2      1   te1
......................ggcacuagcacauuuuugugA....................................................................   14     1   te1
......................ggcacuagcacauuuuuguAu....................................................................   1      1   te1
......................ggcacuagcacauuuuugugu....................................................................   4      0   te1
......................ggcacuagcacauuuuuguUu....................................................................   1      1   te1
......................ggcacuagcacauuuuugugug...................................................................   1      0   te1
```

```
                    Mature                                      Star
gcuaguccucuucucuguucuuuggcacuagcacauuuuugugugacgaucaaaccuacaaaauuguguuagugucgaauaauaugggaagacauucgucauaaaucauaca
........................gcacuagcacauuuuugA......................................................................    3     1   te1
........................gcacuagcacauuuuugu......................................................................    138   0   te1
........................gcacuagcacauuuuuguU.....................................................................    2     1   te1
........................gcacuagcacauuuuuguA.....................................................................    2     1   te1
........................gcacuagcacauuuuugugA....................................................................    1     1   te1
.........................cacuagcacauuuuuguA.....................................................................    22    1   te1
.........................cacuagcacauuuuuguU.....................................................................    10    1   te1
.........................cacuagcacauuuuugug.....................................................................    2     0   te1
.........................cacuagcacauuuuuguUu....................................................................    1     1   te1
.........................cacuagcacauuuuugugu....................................................................    4     0   te1
.........................cacuagcacauuuuugugA....................................................................    8     1   te1
.........................cacuagcacauuuuuguAu....................................................................    8     1   te1
..........................acuagcacauuuuuguUu....................................................................    1     1   te1
.....................................................accuacaaaauuguguuagugucgaa.................................    1     0   te1
........................................................uacaaaauuguguuaguguc....................................    2     0   te1
........................................................uacaaaauuguguuaguguU....................................    1     1   te1
........................................................uaUaaaauuguguuagugucg...................................    1     1   te1
........................................................uacaaaauuguguuagugucA...................................    1     1   te1
........................................................uacaaaauuguguuagugucg...................................    202   0   te1
........................................................uacaaaauuguguuagugucga..................................    12    0   te1
........................................................uacaaaauuguguuagugucgU..................................    4     1   te1
........................................................uacaaaauuguguuagugucgaa.................................    1     0   te1
........................................................uacaaaauuguguuagugucgaau................................    4     0   te1
.........................................................acaaaauuguguuagugucg...................................    3     0   te1
.........................................................acaaaauCguguuagugucg...................................    1     1   te1
.........................................................acaaaauuguguuagugucga..................................    9     0   te1
.........................................................acaaaauuguguuagugucgaa.................................    4     0   te1
.........................................................Ucaaaauuguguuagugucgaau................................    1     1   te1
.........................................................acaaaauuguguuagugucgaaA................................    2     1   te1
.........................................................acaaaauuguguuagugucgaau................................    5     0   te1
..........................................................caaaauuguguuaguguc....................................    1     0   te1
..........................................................caaaauuguguuagugucg...................................    2     0   te1
..........................................................caaaauuCguuagugucga..................................    1     1   te1
..........................................................caaaCuuguguuagugucga..................................    1     1   te1
..........................................................caaaauuguguuagugucga..................................    131   0   te1
..........................................................Uaaaauuguguuagugucgaa.................................    1     1   te1
..........................................................cUaaauuguguuagugucgaa.................................    1     1   te1
..........................................................caaaauuguguuUgugucgaa.................................    1     1   te1
..........................................................caaaauuguguuagugucgUa.................................    2     1   te1
..........................................................caaaauuguguuagugucgaa.................................    98    0   te1
..........................................................caaaauuguguuagugucgaU.................................    1     1   te1
..........................................................caaaGuuguguuagugucgaau................................    2     1   te1
..........................................................caaaauuguguuagugucUaau................................    1     1   te1
..........................................................caaaauugugAuagugucgaau................................    1     1   te1
..........................................................caGaauuguguuagugucgaau................................    1     1   te1
..........................................................caaaauuguguuaguUucgaau................................    1     1   te1
..........................................................caaaauuguguuagugucgaaC................................    7     1   te1
..........................................................caaaauuguguuaguguGgaau................................    1     1   te1
..........................................................caaaauuguguuagugucgUau................................    1     1   te1
..........................................................caaaauuguguuagugucAaau................................    2     1   te1
..........................................................Aaaaauuguguuagugucgaau................................    2     1   te1
..........................................................caaaauuguguAagugucgaau................................    1     1   te1
..........................................................caaaauuguguuagugucgaau................................    1695  0   te1
..........................................................caaaauuguguuagugucgaGu................................    1     1   te1
..........................................................caaaauuguguuaguAucgaau................................    1     1   te1
..........................................................caaaauugCguuagugucgaau................................    1     1   te1
..........................................................caaaauuguguuagugucgaaA................................    60    1   te1
..........................................................caaaauuguguuagugucgaUu................................    1     1   te1
..........................................................caaaauuguguuagugucgaaG................................    3     1   te1
..........................................................caaaauuguguuaAugucgaau................................    1     1   te1
..........................................................caaaauuguguuaguguUgaau................................    2     1   te1
..........................................................caaGauuguguuagugucgaau................................    1     1   te1
..........................................................caaaauuguguuagugucgaaua...............................    47    0   te1
..........................................................caaaauuguguuagugucgaauU...............................    118   1   te1
..........................................................caaaauuguguuagugucgaauC...............................    1     1   te1
..........................................................Uaaaauuguguuagugucgaaua...............................    2     1   te1
..........................................................caaaauuguguuagugucgaaAa...............................    23    1   te1
..........................................................caaaauuguguuagugucgaaGa...............................    1     1   te1
..........................................................caaaauuguguuagugucgaauG...............................    4     1   te1
..........................................................caaaauuguguuagugucgaauGa..............................    1     1   te1
```

Mature **Star**

gcuaguccucuucucuguucuuuggcacuagcacauuuuugugugacgaucaaaccuacaaaauuguguuagugucgaauaauaugggaagacauucgucauaaaucauaca			
....caaaauuguguuagugucgaauaU..............................	2	1	te1
....caaaauuguguuagugucgaauUa..............................	6	1	te1
....caaaauuguguuagugucgaaGaa..............................	2	1	te1
....caaaauuguguuagugucgaaAaa..............................	3	1	te1
....caaaauuguguuagugucgaauaa..............................	2	0	te1
....caaaauuguguuagugucgaauaUu.............................	4	1	te1
....caaaauuguguuagugucgaauaau.............................	1	0	te1
....aaaauuguguuagugucg...................................	4	0	te1
....Uaaauuguguuagugucga..................................	1	1	te1
....aaaauuguguuagugucga..................................	280	0	te1
....aaaauuguguuagugAcga..................................	1	1	te1
....aaaauuguguuagugucgaU.................................	23	1	te1
....aaaauuguguuagugucgaa.................................	621	0	te1
....aaaauuguguuagugucgGa.................................	1	1	te1
....aaaauuguguuaguguGgaa.................................	2	1	te1
....aaaauuguguuagCgucgaa.................................	1	1	te1
....aaaauuguguuagAgucgaa.................................	1	1	te1
....aaaauuguguuaUugucgaa.................................	1	1	te1
....aaaauuguguuaguguUgaa.................................	2	1	te1
....aaaauugGguuagugucgaau................................	1	1	te1
....aUaauuguguuagugucgaau................................	1	1	te1
....aaaauuguguuagugucgCau................................	1	1	te1
....aaaauuguguCagugucgaau................................	1	1	te1
....aaaauuguguuagugucgaUu................................	6	1	te1
....aaaauuUuguuagugucgaau................................	1	1	te1
....aaaauuguguuagugucCaau................................	1	1	te1
....aaaauuguguuagugucgaaA................................	409	1	te1
....aaaauuguguuaCugucgaau................................	1	1	te1
....aaaauuguguuagugucgaau................................	7395	0	te1
....aGaauuguguuagugucgaau................................	4	1	te1
....aaaGuuguguuagugucgaau................................	2	1	te1
....aaaauugugAuagugucgaau................................	1	1	te1
....aaaauuguguuagugCcgaau................................	2	1	te1
....aaaauuguguuGgugucgaau................................	1	1	te1
....aaaauuguguuUgugucgaau................................	1	1	te1
....aaaauuCuguuagugucgaau................................	1	1	te1
....aaaauuguguuagugucgaaC................................	29	1	te1
....aaaauugCguuagugucgaau................................	1	1	te1
....aaaauuguguuaguguAgaau................................	1	1	te1
....aaaauuguguuaguguUgaau................................	4	1	te1
....aaaauuguguuagugucgGau................................	4	1	te1
....aaGauuguguuagugucgaau................................	2	1	te1
....aaaauuguguuagugucgaCu................................	4	1	te1
....aaaauuguguGagugucgaau................................	1	1	te1
....aaaauuguUuuagugucgaau................................	2	1	te1
....aaaauuguguuagugucgUau................................	3	1	te1
....Uaaauuguguuagugucgaau................................	3	1	te1
....aaaauuguguuaguguGgaau................................	4	1	te1
....aaaauuguguuagugucgaaG................................	22	1	te1
....aCaauuguguuagugucgaau................................	3	1	te1
....aaaauuguguuagugGcgaau................................	1	1	te1
....aaaauuguguuaguCucgaau................................	1	1	te1
....aaaCuuguguuagugucgaau................................	3	1	te1
....aaaauuguguuagCgucgaaua...............................	1	1	te1
....aCaauuguguuagugucgaaua...............................	1	1	te1
....aaaauuguguuaguguGgaaua...............................	15	1	te1
....aaaauCguguuagugucgaaua...............................	1	1	te1
....aaaauuUuguuagugucgaaua...............................	1	1	te1
....aaaauuguguuagugucUaaua...............................	2	1	te1
....aaaauuguguCagugucgaaua...............................	2	1	te1
....aaaauuguguuagugucgaaua...............................	13009	0	te1
....aaaauuguguGagugucgaaua...............................	2	1	te1
....aaaauuguguuagugucgaauU...............................	283	1	te1
....aaaauuguguuGgugucgaaua...............................	3	1	te1
....aaaauugugCuagugucgaaua...............................	2	1	te1
....aaaGuuguguuagugucgaaua...............................	4	1	te1
....aaaauuguguuaguCucgaaua...............................	1	1	te1
....aaaauuguguuagugucgaaGa...............................	26	1	te1
....aaaauuguguuagugucgaUua...............................	2	1	te1
....aaaauuguguuagugucgUaua...............................	6	1	te1

Mature Star

```
gcuaguccucuucucuguucuuuggcacuagcacauuuuugugugacgaucaaaccuacaaaauuguguuagugucgaauaauaugggaagacauucgucauaaaucauaca
...........................................................aaaauuguguuaUugucgaaua...............................  1     1   te1
...........................................................aaaauuguguuagugucCaaua...............................  1     1   te1
...........................................................aaaauuguguuagugucgaauG...............................  54    1   te1
...........................................................aaaauuguguuagugucAaaua...............................  2     1   te1
...........................................................aaaauuguguuaguguUgaaua...............................  1     1   te1
...........................................................aaaCuuguguuagugucgaaua...............................  3     1   te1
...........................................................aaaauuguguuagugucgCaua...............................  7     1   te1
...........................................................aaaauuguguuagugucgGaua...............................  8     1   te1
...........................................................Uaaauuguguuagugucgaaua...............................  11    1   te1
...........................................................aaUauuguguuagugucgaaua...............................  3     1   te1
...........................................................aaGauuguguuagugucgaaua...............................  3     1   te1
...........................................................aaaauuguguuagugucgaauC...............................  17    1   te1
...........................................................aaaauuguguuagugucgaaAa...............................  378   1   te1
...........................................................aaaauuguguuagugGcgaaua...............................  3     1   te1
...........................................................aaaaAuguguuagugucgaaua...............................  1     1   te1
...........................................................aaCauuguguuagugucgaaua...............................  1     1   te1
...........................................................Gaaauuguguuagugucgaaua...............................  2     1   te1
...........................................................aaaauuguguuagugucgaaCa...............................  44    1   te1
...........................................................aaaauuguguuaguUucgaaua...............................  1     1   te1
...........................................................aaaaGuguguuagugucgaaua...............................  3     1   te1
...........................................................aaaauGguguuagugucgaaua...............................  1     1   te1
...........................................................aGaauuguguuagugucgaaua...............................  3     1   te1
...........................................................aaaaCuguguuagugucgaaua...............................  4     1   te1
...........................................................aaaauugCguuagugucgaaua...............................  2     1   te1
...........................................................aaaauuguguuagGgucgaaua...............................  1     1   te1
...........................................................aUaauuguguuagugucgaaua...............................  1     1   te1
...........................................................aaaauuguguuCgugucgaaua...............................  1     1   te1
...........................................................aaaauugGguuagugucgaaua...............................  4     1   te1
...........................................................aaaauuguguuaguguGgaauaa..............................  1     1   te1
...........................................................aaaauuguguuagugucgaauaa..............................  188   0   te1
...........................................................aaaauuguguuagugucgaauCa..............................  2     1   te1
...........................................................aaaauuguguuagugucgaaGaa..............................  2     1   te1
...........................................................aaaauuguguuagugucgaauaG..............................  1     1   te1
...........................................................aaaauuguguuagugucgaauaU..............................  91    1   te1
...........................................................aaaauuguguuagugucgaauGa..............................  7     1   te1
...........................................................aaaauuguguuagugucgaauUa..............................  39    1   te1
...........................................................aaaauuguguuagugucgaaAaa..............................  46    1   te1
...........................................................aaaauuguguuagugucgaauaC..............................  25    1   te1
...........................................................Uaaauuguguuagugucgaauaa..............................  1     1   te1
...........................................................aaaauuguguuaUugucgaauaa..............................  1     1   te1
...........................................................aaaauuguguuagugucgaauaau.............................  1     0   te1
...........................................................aaaauuguguuagugucgaauaaA.............................  4     1   te1
...........................................................aaaauuguguuagugucgaauaUu.............................  8     1   te1
...........................................................aaaauuguguuagugucgaauaaua............................  1     0   te1
...........................................................aaaauuguguuagugucgaauaaGa............................  1     1   te1
............................................................aaauuguguuagugucga..................................  2     0   te1
............................................................aaauuguguuagugucgaa.................................  6     0   te1
............................................................aaauuguguuaguguGgaau................................  1     1   te1
............................................................aaauuguguuagugucgaaC................................  1     1   te1
............................................................aaauuguguuagugucgaau................................  525   0   te1
............................................................aaauuguguuagugucAaau................................  1     1   te1
............................................................Uaauuguguuagugucgaau................................  1     1   te1
............................................................aaauuguguuagugucgaaA................................  11    1   te1
............................................................aaauuguguuGgugucgaau................................  1     1   te1
............................................................aaauuguguuagugucgUau................................  2     1   te1
............................................................aaauuguguuagugucgaaGa...............................  1     1   te1
............................................................aaCuuguguuagugucgaaua...............................  1     1   te1
............................................................aaauuguguuagAgucgaaua...............................  1     1   te1
............................................................aaauuguguuagugucgaauU...............................  16    1   te1
............................................................aaauuguguuagugucgGaua...............................  1     1   te1
............................................................aaauuguCuuagugucgaaua...............................  7     1   te1
............................................................aaauugugAuagugucgaaua...............................  1     1   te1
............................................................aaauuguguuagugucgaaua...............................  8662  0   te1
............................................................aaauuguguuaguguAgaaua...............................  1     1   te1
............................................................aaauuguguCagugucgaaua...............................  1     1   te1
............................................................aaauuguguuagugCcgaaua...............................  1     1   te1
............................................................aaauuguguuaguguUgaaua...............................  3     1   te1
............................................................aaauuguguuagugucgaUua...............................  6     1   te1
............................................................aaauuguguuagugucgCaua...............................  1     1   te1
............................................................aaauuguguuaCugucgaaua...............................  2     1   te1
```

Mature **Star**

gcuaguccucuucucuguucuuuggcacuagcacauuuuugugugacgaucaaaccuacaaaauuguguuagugucgaauaauaugggaagacauucgucauaaaucauaca			
....aaauuguguuagugucgUaua...............................	7	1	te1
....aaaCuguguuagugucgaaua...............................	2	1	te1
....aaauGguguuagugucgaaua...............................	3	1	te1
....aaauuguguuagugucUaaua...............................	2	1	te1
....aaauuguguuagugucgaaCa...............................	1	1	te1
....aaGuguguuagugucgaaua................................	1	1	te1
....aaauugCguuagugucgaaua...............................	2	1	te1
....aaauuguguuagugucgaauG...............................	3	1	te1
....aaaGuguguuagugucgaaua...............................	3	1	te1
....aaauuguguuaguguGgaaua...............................	4	1	te1
....aaauuguguuaAugucgaaua...............................	1	1	te1
....aUauuguguuagugucgaaua...............................	2	1	te1
....aaauuguguuagugucAaaua...............................	1	1	te1
....aaUuguguuagugucgaaua................................	1	1	te1
....Uaauuguguuagugucgaaua...............................	12	1	te1
....aaauuguguuagugucgaaAa...............................	12	1	te1
....aaauugugCuagugucgaaua...............................	6	1	te1
....aaauuCuguuagugucgaaua...............................	1	1	te1
....aaauuguguuGgugucgaaua...............................	1	1	te1
....aCauuguguuagugucgaaua...............................	3	1	te1
....aGauuguguuagugucgaaua...............................	1	1	te1
....aaaAuguguuagugucgaaua...............................	2	1	te1
....aaauugugGuagugucgaaua...............................	1	1	te1
....aaauuguguuagugucgaauaU..............................	18	1	te1
....aaauuguguuagugucgaauUa..............................	2	1	te1
....aaauuguguuagugucgaauaG..............................	1	1	te1
....aaauuguguuagugucgaauaa..............................	61	0	te1
....aaauuguguuagugucgaaAaa..............................	1	1	te1
....aaauuguguuagugucgaauaC..............................	8	1	te1
....aaauuguguuagugucgaauaaA.............................	6	1	te1
....aaauuguguuagugucgaauaau.............................	1	0	te1
....aaauuguguuagugucgaauaUu.............................	1	1	te1
....aaauuguguuagugucgaauaaua............................	1	0	te1
....aaauuguguuagugucgaauaauUu...........................	1	1	te1
....aauuguguuagugucgaau................................	3	0	te1
....aauuguguuagugucgaaua...............................	12	0	te1
....aauuguguuagugucgaauaU..............................	2	1	te1
....auguguuagugucgaaua................................	2	0	te1
....uguguuagugucgaaua................................	8	0	te1
....uguguuagugucgaauU................................	1	1	te1
....uguguuagugucgaaAaa...............................	1	1	te1
...................Auuggcacuagcacauuuuugu...	1	1	egg
....................uuuggcacuagcacauuuuug...	7	0	egg
....................uuuggcacuagcacauuuuuA...	1	1	egg
....................uuugUcacuagcacauuuuug...	1	1	egg
....................Cuuggcacuagcacauuuuugu..	1	1	egg
....................uuugUcacuagcacauuuuugu..	1	1	egg
....................uuuggcacuagcGcauuuuugu..	1	1	egg
....................uuuggcacuagcacauuuuugA..	1	1	egg
....................uuuUgcacuagcacauuuuugu..	1	1	egg
....................uuuggcacuGgcacauuuuugu..	2	1	egg
....................uuuggcacuagcacauuuuugu..	45	0	egg
....................uuAggcacuagcacauuuuugu..	1	1	egg
....................uuuggcacuagcacauuuuugC..	5	1	egg
....................uuuggcacuagcacauuuuuguU...	9	1	egg
....................uuuggcacuagcacauuuuuguA...	6	1	egg
....................uuuggcacuagcacauuuuuguC...	1	1	egg
.....................uuggcacuagcacauuuuugug...	2	0	egg
.....................uuggcacuagcacauuuuugugA..	1	1	egg
....caaaauuguguuagugucga..................................	1	0	egg
....caaaauuguguuagugucgaau................................	8	0	egg
....caaaauuguguuagugucgaaua...............................	1	0	egg
....aaaauuguguuagugucga..................................	1	0	egg
....aaaauuguguuagugucgaa.................................	1	0	egg
....aaaauuguguuagugucgaaA................................	1	1	egg
....aaaaGuguguuagugucgaau................................	2	1	egg
....aaaauuguguuaguguUgaau................................	1	1	egg
....aaaauuguguuagugucgaaC................................	1	1	egg
....aaaauuguguuagugucgaau................................	9	0	egg

```
                   Mature                                 Star
gcuaguccucuucucuguucuuuggcacuagcacauuuuugugugacgaucaaaccuacaaaauuguguuagugucgaauaauaugggaagacauucgucauaaaucauaca
...........................................................Naaauuguguuagugucgaau................................   1     1     egg
...........................................................aaaauuguguuagugucgaaua...............................   4     0     egg
...........................................................aaaaGuguguuagugucgaaua...............................   1     1     egg
............................................................aaauuguguuagugucgaau................................   2     0     egg

..................ucuuuggcacuagcacauuuuu........................................................................   1     0     T6P
...................cuuuggcacuagcacauuuuu........................................................................   8     0     T6P
...................Uuuuggcacuagcacauuuuu........................................................................   1     1     T6P
...................cuuuggcacuagcacauuuuug.......................................................................   1     0     T6P
...................Uuuuggcacuagcacauuuuug.......................................................................   1     1     T6P
...................Uuuuggcacuagcacauuuuugu......................................................................   26    1     T6P
...................cuuuggcacuagcacauuuuugA......................................................................   2     1     T6P
...................cuuuggcacuagcacauuuuugu......................................................................   2     0     T6P
...................Uuuuggcacuagcacauuuuugug.....................................................................   1     1     T6P
....................uuuggcacuagcacauuu..........................................................................   1     0     T6P
....................uuuggcacuagcacauGu..........................................................................   1     1     T6P
....................uuuggcacuagcacauuuu.........................................................................   44    0     T6P
....................uuCggcacuagcacauuuu.........................................................................   1     1     T6P
....................uuuggcacuagcacauuuG.........................................................................   1     1     T6P
....................uuuggcacuagcacauuGu.........................................................................   12    1     T6P
....................uuuggcacuagcacaAuuuu........................................................................   1     1     T6P
....................uuuggcacuagcaUauuuuu........................................................................   3     1     T6P
....................uuuUgcacuagcacauuuuu........................................................................   3     1     T6P
....................uuuggcacuagGacauuuuu........................................................................   2     1     T6P
....................Auuggcacuagcacauuuuu........................................................................   1     1     T6P
....................uuuggcacuagcacauuuGu........................................................................   24    1     T6P
....................uuuggcacuagcacauuuuA........................................................................   1     1     T6P
....................uuugAcacuagcacauuuuu........................................................................   1     1     T6P
....................uuuggcacuCgcacauuuuu........................................................................   1     1     T6P
....................uuuggcGcuagcacauuuuu........................................................................   1     1     T6P
....................uuGggcacuagcacauuuuu........................................................................   1     1     T6P
....................uAuggcacuagcacauuuuu........................................................................   1     1     T6P
....................uuuggcacuagcacauuuuG........................................................................   3     1     T6P
....................uuuggcacuagcacauuuuu........................................................................   1833  0     T6P
....................uuuggcacuagcUcauuuuu........................................................................   1     1     T6P
....................uuuggcacuagcacauuuCug.......................................................................   2     1     T6P
....................uuGggcacuagcacauuuuug.......................................................................   2     1     T6P
....................uuuggcacuagcacauuuuug.......................................................................   8102  0     T6P
....................uuuggcacuagcaUauuuuug.......................................................................   3     1     T6P
....................uuuggcacuagcacUuuuuug.......................................................................   2     1     T6P
....................uuuggcaUuagcacauuuuug.......................................................................   3     1     T6P
....................uuugCcacuagcacauuuuug.......................................................................   1     1     T6P
....................uuuggcacuagcacauCuuug.......................................................................   2     1     T6P
....................uuuggcacuagcacaCuuuug.......................................................................   1     1     T6P
....................uuuUgcacuagcacauuuuug.......................................................................   5     1     T6P
....................Guuggcacuagcacauuuuug.......................................................................   1     1     T6P
....................uuuggcacuagcacauuuuCg.......................................................................   1     1     T6P
....................uuugAcacuagcacauuuuug.......................................................................   5     1     T6P
....................uuuggcacuagcacGuuuuug.......................................................................   1     1     T6P
....................uuuggcacuagcacaAuuuug.......................................................................   1     1     T6P
....................uuuggcacuagcacauuAuug.......................................................................   1     1     T6P
....................uuuggcacuagcacauuuuuC.......................................................................   1     1     T6P
....................uuuggcacuaCcacauuuuug.......................................................................   3     1     T6P
....................uuuggcacuagUacauuuuug.......................................................................   3     1     T6P
....................uuuggcacuagcacauAuuug.......................................................................   1     1     T6P
....................uuuggUacuagcacauuuuug.......................................................................   16    1     T6P
....................uuuCgcacuagcacauuuuug.......................................................................   1     1     T6P
....................uuCggcacuagcacauuuuug.......................................................................   2     1     T6P
....................uuuggcacuagcacauuuuuA.......................................................................   11    1     T6P
....................uuuggcaAuagcacauuuuug.......................................................................   1     1     T6P
....................uuuggcacuUgcacauuuuug.......................................................................   1     1     T6P
....................uuuggcacuGgcacauuuuug.......................................................................   1     1     T6P
....................uuuggcGcuagcacauuuuug.......................................................................   4     1     T6P
....................uuugUcacuagcacauuuuug.......................................................................   2     1     T6P
....................uuuggcacuagcacauuuuuU.......................................................................   12    1     T6P
....................uuuAgcacuagcacauuuuug.......................................................................   1     1     T6P
....................Cuuggcacuagcacauuuuug.......................................................................   4     1     T6P
....................Auuggcacuagcacauuuuug.......................................................................   3     1     T6P
....................uuuggcacCagcacauuuuug.......................................................................   2     1     T6P
....................uuuggcacuaAcacauuuuug.......................................................................   9     1     T6P
```

```
                    Mature                                 Star
gcuaguccucuucucuguucuuuggcacuagcacauuuuugugugacgaucaaaccuacaaaauuguguuagugucgaauaauaugggaagacauucgucauaaaucauaca
....................uuuggcacuaUcacauuuuug.......................................................................    6       1    T6P
....................uuuggcacuUgcacauuuuugu......................................................................    5       1    T6P
....................uuuAgcacuagcacauuuuugu......................................................................    3       1    T6P
....................uuuggcacuagcacauuuuAgu......................................................................    2       1    T6P
....................AuuggcacuagcacauuuuugU......................................................................    30      1    T6P
....................uuuggcacuaCcacauuuuugu......................................................................    4       1    T6P
....................uuuggcacuagcacauuuAugu......................................................................    1       1    T6P
....................uuuggcacuagcacauuuuuUu......................................................................    23      1    T6P
....................uuuggcacuagcacauuuuuCu......................................................................    18      1    T6P
....................uuuggcacuagcacauCuuugu......................................................................    8       1    T6P
....................uuuggcacuagcUcauuuuugu......................................................................    2       1    T6P
....................uuuggcacuagcacauuuuugu......................................................................    81369   0    T6P
....................uuuggcacuagcacauuuCugu......................................................................    15      1    T6P
....................uuuggcacuagcacaGuuuugu......................................................................    6       1    T6P
....................uuuggcacuagcacaAuuuugu......................................................................    22      1    T6P
....................uuuggcacuagcacauAuuugu......................................................................    4       1    T6P
....................uuuggcacuagcacauGuuugu......................................................................    1       1    T6P
....................uuuUgcacuagcacauuuuugu......................................................................    56      1    T6P
....................uuuggcacuagcacaCuuuugu......................................................................    13      1    T6P
....................uuuggcacAagcacauuuuugu......................................................................    1       1    T6P
....................uuuggUacuagcacauuuuugu......................................................................    141     1    T6P
....................uuugUcacuagcacauuuuugu......................................................................    28      1    T6P
....................uAuggcacuagcacauuuuugu......................................................................    3       1    T6P
....................uuuggcacuagcaAauuuuugu......................................................................    5       1    T6P
....................uuuggcacuagcacGuuuuugu......................................................................    5       1    T6P
....................uuuggcacuagcaGauuuuugu......................................................................    10      1    T6P
....................uuuggcacuagcacauuuuugC......................................................................    16      1    T6P
....................uuuggcUcuagcacauuuuugu......................................................................    10      1    T6P
....................uuuCgcacuagcacauuuuugu......................................................................    3       1    T6P
....................uuuggcacuaAcacauuuuugu......................................................................    103     1    T6P
....................uuuggcacCagcacauuuuugu......................................................................    7       1    T6P
....................uuuggcacuagcacauuuuuAu......................................................................    8       1    T6P
....................uuuggcGcuagcacauuuuugu......................................................................    50      1    T6P
....................uuuggcaGuagcacauuuuugu......................................................................    3       1    T6P
....................uuuggcacuagcacauuuGugu......................................................................    1       1    T6P
....................uuuggcacuagcacUuuuuugu......................................................................    9       1    T6P
....................uuuggcacuagcacauuuuCgu......................................................................    11      1    T6P
....................uCuggcacuagcacauuuuugu......................................................................    14      1    T6P
....................uuuggcCcuagcacauuuuugu......................................................................    6       1    T6P
....................uuuggcaUuagcacauuuuugu......................................................................    16      1    T6P
....................Guuggcacuagcacauuuuugu......................................................................    14      1    T6P
....................uuuggcacuCgcacauuuuugu......................................................................    3       1    T6P
....................Cuuggcacuagcacauuuuugu......................................................................    24      1    T6P
....................uuuggcacuagcaUauuuuugu......................................................................    23      1    T6P
....................uuugAcacuagcacauuuuugu......................................................................    34      1    T6P
....................uuugCcacuagcacauuuuugu......................................................................    8       1    T6P
....................uuAggcacuagcacauuuuugu......................................................................    3       1    T6P
....................uuCggcacuagcacauuuuugu......................................................................    17      1    T6P
....................uuuggcacuagUacauuuuugu......................................................................    32      1    T6P
....................uuuggcacuagcacauuuuGgu......................................................................    1       1    T6P
....................uuuggcacuagcGcauuuuugu......................................................................    2       1    T6P
....................uuGggcacuagcacauuuuugu......................................................................    25      1    T6P
....................uuuggcacuaUcacauuuuugu......................................................................    10      1    T6P
....................uuuggcacuagcacauuCuugu......................................................................    12      1    T6P
....................uuuggcacuagcacauuuuugG......................................................................    8       1    T6P
....................uuuggcacuGgcacauuuuugu......................................................................    9       1    T6P
....................uuuggcacuagcacauuAuugu......................................................................    2       1    T6P
....................uuuggcacuagAacauuuuugu......................................................................    13      1    T6P
....................uuuggcacuagcacauuuuugA......................................................................    926     1    T6P
....................uuuggcacuagGacauuuuugu......................................................................    28      1    T6P
....................uuuggcacuagcacauuuuuguC.....................................................................    9       1    T6P
....................uuuggcGcuagcacauuuuugug.....................................................................    1       1    T6P
....................uuuggcacuagcacauuuuuguA.....................................................................    868     1    T6P
....................uuuggcacuagcacauuuuuguU.....................................................................    448     1    T6P
....................Cuuggcacuagcacauuuuugug.....................................................................    1       1    T6P
....................uuuggcacuagcacauuuuugug.....................................................................    202     0    T6P
....................uuuggUacuagcacauuuuugug.....................................................................    1       1    T6P
....................uuuggcacuagcaAauuuuugug.....................................................................    1       1    T6P
....................uuuUgcacuagcacauuuuugug.....................................................................    1       1    T6P
....................uuuggcacuagcacauuuuuguCu....................................................................    1       1    T6P
```

Mature Star

gcuaguccucuucucuguucuuuggcacuagcacauuuuugugugacgaucaaaccuacaaaauuguguuagugucgaauaauaugggaagacauucgucauaaaucauaca

....................uuuggcacuagcacauuuuuguUu..	18	1	T6P
....................uuuggcacuagcacauuuuuguAu..	111	1	T6P
....................uuuggcacuagcacauuuuugugu..	23	0	T6P
....................uuuggcacuagcacauuuuugugA..	185	1	T6P
....................uuuggcacuagcacauuuuuguUug...	1	1	T6P
....................uuuggcacuagcacauuuuuguguU...	1	1	T6P
....................uuuggcacuagcacauuuuuguguga..	2	0	T6P
....................uuuggcacuagcacauuuuugugugac...	5	0	T6P
.....................uuggcacuagcacauuuu...	2	0	T6P
.....................uuggcacuagcacauuuGu..	1	1	T6P
.....................uuggcacuagcacauuuuu..	17	0	T6P
.....................uuCgcacuagcacauuuuu..	1	1	T6P
.....................uugAcacuagcacauuuuug...	1	1	T6P
.....................uuggcacuagcacauuuuug...	111	0	T6P
.....................uuggcacuagcacauuuuugA..	9	1	T6P
.....................uuggcacuagcacauuuuCgu..	1	1	T6P
.....................uuggcacuagcacauuuuuUu..	2	1	T6P
.....................Cuggcacuagcacauuuuugu..	2	1	T6P
.....................uuggcacuagcaGauuuuugu..	1	1	T6P
.....................uuUgcacuagcacauuuuugu..	5	1	T6P
.....................Guggcacuagcacauuuuugu..	1	1	T6P
.....................uCggcacuagcacauuuuugu..	1	1	T6P
.....................uuggcaAuagcacauuuuugu..	1	1	T6P
.....................uuggcacuagcacauGuuugu..	1	1	T6P
.....................uuggcacuagUacauuuuugu..	1	1	T6P
.....................uuggcaGuagcacauuuuugu..	3	1	T6P
.....................uuggcacuaAcacauuuuugu..	4	1	T6P
.....................uuggcacuaUcacauuuuugu..	1	1	T6P
.....................uugCcacuagcacauuuuugu..	1	1	T6P
.....................uGggcacuagcacauuuuugu..	1	1	T6P
.....................uuggcacuagcacauuuuugG..	2	1	T6P
.....................uuggUacuagcacauuuuugu..	6	1	T6P
.....................uuggcacuaCcacauuuuugu..	2	1	T6P
.....................Auggcacuagcacauuuuugu..	1	1	T6P
.....................uugUcacuagcacauuuuugu..	5	1	T6P
.....................uuggcacuagcacauuuuugu..	4056	0	T6P
.....................uuUgcacuagcacauuuuugug...	2	1	T6P
.....................uuggcacuagcacauuuuuguA...	84	1	T6P
.....................uuggcacuagcacauuuuugAg...	1	1	T6P
.....................uuggcacuagcacauuuuugug...	1704	0	T6P
.....................uuggcacuagcacauuuuuguU...	13	1	T6P
.....................uuggcacuagcCcauuuuugug...	1	1	T6P
.....................uugUcacuagcacauuuuugug...	1	1	T6P
.....................Auggcacuagcacauuuuugug...	1	1	T6P
.....................uuggUacuagcacauuuuugug...	2	1	T6P
.....................uAggcacuagcacauuuuugug...	1	1	T6P
.....................uugAcacuagcacauuuuugug...	1	1	T6P
.....................uuggcacuaAcacauuuuugug...	3	1	T6P
.....................uuggcacuagcacauuuuuUug...	1	1	T6P
.....................uuggcacuagUacauuuuugug...	1	1	T6P
.....................uuggcacuaUcacauuuuugug...	1	1	T6P
.....................uuggcacuagcacauuuuugugC..	2	1	T6P
.....................uuggcacuagcacauuuuuguUu..	1	1	T6P
.....................uuggcacuagcacauuuuugugA..	1858	1	T6P
.....................uuggcacuagcacauuuuugugG..	20	1	T6P
.....................uuggcacuagcacauuuuugugu..	36	0	T6P
.....................uuggcacuagcacauuuuuguAu..	9	1	T6P
.....................uuggcacuagcacauuuuuguguA...	1	1	T6P
.....................uuggcacuagcacauuuuuguguU...	2	1	T6P
.....................uuggcacuagcacauuuuugugAg...	1	1	T6P
......................uggcacuagcacauuuuu..	1	0	T6P
......................uggcacuagcacauuuuug...	6	0	T6P
......................uggcacuagcacauuuuugA..	1	1	T6P
......................uUgcacuagcacauuuuugu..	2	1	T6P
......................uggcacuagcacauuuuugC..	1	1	T6P
......................ugUcacuagcacauuuuugu..	1	1	T6P
......................uggcacuagcacauuuuugu..	194	0	T6P
......................uggcacuagcacauuGuugu..	1	1	T6P
......................uggUacuagcacauuuuugug...	1	1	T6P
......................uggcacuagcacauuuuuguA...	3	1	T6P

```
                    Mature                                     Star
gcuaguccucuucucuguucuuuggcacuagcacauuuuugugugacgaucaaaccuacaaaauuguguuagugucgaauaauaugggaagacauucgucauaaaucauaca
......................uggcacuagcacauuuuugug.....................................................................    117    0    T6P
......................uggcacuagcacauuuuuguU.....................................................................    2      1    T6P
......................uggcacuagcacauuuuugugA....................................................................    192    1    T6P
......................uggcacuagcacauuuuuUugu....................................................................    1      1    T6P
......................uggcacuagcacauuuuugugu....................................................................    86     0    T6P
......................uggcacuagcacauuuuuguguA...................................................................    1      1    T6P
......................uggcacuagcacauuuuugugug...................................................................    1      0    T6P
.......................ggcacuagcacauuuuug.......................................................................    4      0    T6P
.......................ggcacuagcacauuuuuUu......................................................................    1      1    T6P
.......................ggcacuagcacauuuuugu......................................................................    92     0    T6P
.......................ggcacuagcacauuuuuguA.....................................................................    1      1    T6P
.......................ggcacuagcacauuuuugugA....................................................................    1      1    T6P
........................gcacuagcacauuuuugu......................................................................    6      0    T6P
........................Ucacuagcacauuuuugu......................................................................    1      1    T6P
.........................cacuagcacauuuuuguA.....................................................................    4      1    T6P
.........................cacuagcacauuuuuguC.....................................................................    2      1    T6P
........................................................uacaaaauuguguuagugucg...................................    7      0    T6P
........................................................uacaaaauuguguuagugucga..................................    2      0    T6P
........................................................uacaaaauuguguuagugucgaaA................................    1      1    T6P
..........................................................caaaauuguguuagugucga..................................    7      0    T6P
..........................................................caaaauuguguuagugucgaa.................................    9      0    T6P
..........................................................caaaauuguguuagugucgaaA................................    3      1    T6P
..........................................................caaaauuguUuuagugucgaau................................    1      1    T6P
..........................................................caaaauuguguuagugucgaau................................    59     0    T6P
..........................................................caaaauuguguuagugucgaaC................................    1      1    T6P
..........................................................caaaauuguguuagugucgaaAa...............................    1      1    T6P
..........................................................caaaauuguguuagugucgaauU...............................    1      1    T6P
...........................................................aaaauuguguuagugucg...................................    3      0    T6P
...........................................................aaaauuguguuagugucga..................................    46     0    T6P
...........................................................aaaauuguguuaguAucga..................................    1      1    T6P
...........................................................aaaauuguguuagugucgaU.................................    1      1    T6P
...........................................................aaaauuguguuagugucgaa.................................    93     0    T6P
...........................................................aaaauuguguuaguAucgaau................................    1      1    T6P
...........................................................aaaauuguguuagugucgaaC................................    2      1    T6P
...........................................................aaCauuguguuagugucgaau................................    1      1    T6P
...........................................................aaaauuguguuaguCucgaau................................    1      1    T6P
...........................................................aaaauuguguuagugucgaaG................................    2      1    T6P
...........................................................aaaauuguguuagugucCaau................................    1      1    T6P
...........................................................aaaauuguguuaguguUgaau................................    1      1    T6P
...........................................................aaaauuguguuagugucgaau................................    679    0    T6P
...........................................................aaaauuguguuagCgucgaau................................    1      1    T6P
...........................................................aaaauuguguuagugucgaaA................................    43     1    T6P
...........................................................aaaauuguguuaUugucgaaua...............................    1      1    T6P
...........................................................aaaauuguguuagugucgaauC...............................    3      1    T6P
...........................................................aaaauuguguuagugucgaauU...............................    20     1    T6P
...........................................................aaaauuguguuaCugucgaaua...............................    2      1    T6P
...........................................................aaaauuguguuagugucgaaCa...............................    5      1    T6P
...........................................................aaaauuguguuagugucgaauG...............................    5      1    T6P
...........................................................aaaauuAuguuagugucgaaua...............................    1      1    T6P
...........................................................aaaauuguguuagugucCaaua...............................    1      1    T6P
...........................................................aaaauuguguuagugucgaaua...............................    495    0    T6P
...........................................................aaaauuguguuagugucgaaAa...............................    11     1    T6P
...........................................................aaaauuguguuGgugucgaaua...............................    1      1    T6P
...........................................................aaaauuguguuagugucgaauaa..............................    8      0    T6P
...........................................................aaaauuguguuagugucgaauaU..............................    4      1    T6P
...........................................................aaaauuguguuagugucgaauUa..............................    1      1    T6P
............................................................aaauuguguuagugucgaaA................................    1      1    T6P
............................................................aaauuguguuagugucgaau................................    7      0    T6P
............................................................aaauuguguuagugucgaaua...............................    42     0    T6P
............................................................aaauuguguuagugucgaauaU..............................    3      1    T6P
.............................................................aauuguguuagugucgaaua...............................    1      0    T6P

..................ucuuuggcacuagcacauuu..........................................................................    1      0    T53
..................uUuuuggcacuagcacauuuuug.......................................................................    2      1    T53
..................Gcuuuggcacuagcacauuuuugu......................................................................    2      1    T53
..................uUuuuggcacuagcacauuuuugu......................................................................    31     1    T53
...................cuuuggcacuagcacauuuu.........................................................................    1      0    T53
...................Uuuuggcacuagcacauuuu.........................................................................    1      1    T53
...................cuuuggcacuagcacauuuuu........................................................................    7      0    T53
...................Uuuuggcacuagcacauuuuu........................................................................    5      1    T53
```

```
                  Mature                                    Star
gcuaguccucuucucuguucuuuggcacuagcacauuuuugugugacgaucaaaccuacaaaauuguguuagugucgaauaauaugggaagacauucgucauaaaucauaca
...................cuuuggcacuagcacauuuuug.......................................................................    1       0    T53
...................Uuuuggcacuagcacauuuuug.......................................................................    14      1    T53
...................cuuuggcacuagcacauuuuugu......................................................................    17      0    T53
...................Auuuggcacuagcacauuuuugu......................................................................    9       1    T53
...................Guuuggcacuagcacauuuuugu......................................................................    4       1    T53
...................Uuuuggcacuagcacauuuuugu......................................................................    239     1    T53
...................cuuuggcacuagcacauuuuuguU.....................................................................    2       1    T53
...................cuuuggcacuagcacauuuuugug.....................................................................    1       0    T53
....................uuuggcacuagcacauuu..........................................................................    43      0    T53
....................uuuggcacuagcacauGu..........................................................................    5       1    T53
....................uuuggcacuagcacauuG..........................................................................    1       1    T53
....................uuuggcacuagcacauAu..........................................................................    1       1    T53
....................uuAggcacuagcacauuuu.........................................................................    1       1    T53
....................uuuggcacuaAcacauuuu.........................................................................    1       1    T53
....................uuuggcacuagUacauuuu.........................................................................    1       1    T53
....................uuuggcacuagcacauuAu.........................................................................    1       1    T53
....................uuuggcacuagcacauuuA.........................................................................    2       1    T53
....................uuuggcacuagcacaAuuu.........................................................................    1       1    T53
....................uuuggUacuagcacauuuu.........................................................................    4       1    T53
....................uuuggcacuagcacauuGu.........................................................................    9       1    T53
....................Cuuggcacuagcacauuuu.........................................................................    1       1    T53
....................uuuggcacuagcaAauuuu.........................................................................    3       1    T53
....................uuGggcacuagcacauuuu.........................................................................    1       1    T53
....................uuuggcacuagcacauuuu.........................................................................    1064    0    T53
....................uuuggcacuagcacauuuG.........................................................................    8       1    T53
....................Auuggcacuagcacauuuu.........................................................................    1       1    T53
....................uuuggcacuagAacauuuu.........................................................................    1       1    T53
....................uuuggcacuagcacauAuu.........................................................................    2       1    T53
....................uuuggcacuagcacUuuuu.........................................................................    2       1    T53
....................uuugCcacuagcacauuuu.........................................................................    1       1    T53
....................uuuggcUcuagcacauuuuu........................................................................    2       1    T53
....................uuuggcacuagUacauuuuu........................................................................    8       1    T53
....................uuuggcacuagAacauuuuu........................................................................    5       1    T53
....................uuuggcacuagcacGuuuuu........................................................................    3       1    T53
....................Cuuggcacuagcacauuuuu........................................................................    6       1    T53
....................uuuggUacuagcacauuuuu........................................................................    35      1    T53
....................uuGggcacuagcacauuuuu........................................................................    6       1    T53
....................uuugUcacuagcacauuuuu........................................................................    2       1    T53
....................uuuggcacuagcUcauuuuu........................................................................    4       1    T53
....................uuuggcaUuagcacauuuuu........................................................................    6       1    T53
....................uuuggcacuagcacauuuuG........................................................................    15      1    T53
....................uuuggcacuagcCcauuuuu........................................................................    2       1    T53
....................uuuggcacuagcacaAuuuu........................................................................    12      1    T53
....................uuuggcacuagcacauuuAu........................................................................    2       1    T53
....................uuuggcacuagcacauuGuu........................................................................    4       1    T53
....................uuugCcacuagcacauuuuu........................................................................    5       1    T53
....................uuuggcacuagcacauuuGu........................................................................    64      1    T53
....................uuAggcacuagcacauuuuu........................................................................    5       1    T53
....................uuuggcacuagcacauuuuA........................................................................    17      1    T53
....................uuuggGacuagcacauuuuu........................................................................    3       1    T53
....................Guuggcacuagcacauuuuu........................................................................    5       1    T53
....................uuuggcacuGgcacauuuuu........................................................................    1       1    T53
....................uuuggcacuagcacauGuuu........................................................................    3       1    T53
....................uuuAgcacuagcacauuuuu........................................................................    1       1    T53
....................uCuggcacuagcacauuuuu........................................................................    2       1    T53
....................uuuggcacuagcacauAuuu........................................................................    9       1    T53
....................uuuggcacuagcacauuuuC........................................................................    3       1    T53
....................uuuggcacuagcacauCuuu........................................................................    6       1    T53
....................uuuggcacuagcacauuAuu........................................................................    1       1    T53
....................uuuggcacuaAcacauuuuu........................................................................    11      1    T53
....................uuuggcacuagcacauuuuu........................................................................    16532   0    T53
....................uuuggcacuaCcacauuuuu........................................................................    2       1    T53
....................uuuggcacuagcacauuCuu........................................................................    1       1    T53
....................uuuggcacuagGacauuuuu........................................................................    5       1    T53
....................uuuggcGcuagcacauuuuu........................................................................    2       1    T53
....................uuuggcacuagcacUuuuuu........................................................................    13      1    T53
....................uuuggcacAagcacauuuuu........................................................................    10      1    T53
....................uuuCgcacuagcacauuuuu........................................................................    1       1    T53
....................Auuggcacuagcacauuuuu........................................................................    23      1    T53
....................uuuggcacuagcaAauuuuu........................................................................    1       1    T53
```

Mature Star

```
gcuaguccucuucucuguucuuuggcacuagcacauuuuugugugacgaucaaaccuacaaaauuguguuagugucgaauaauaugggaagacauucgucauaaaucauaca
....................uuuggAacuagcacauuuuu........................................................................ 2 1 T53
....................uuuggcacuCgcacauuuuu........................................................................ 2 1 T53
....................uuCggcacuagcacauuuuu........................................................................ 4 1 T53
....................uAuggcacuagcacauuuuu........................................................................ 1 1 T53
....................uuuggcacuagcaUauuuuu........................................................................ 13 1 T53
....................uuugAcacuagcacauuuuu........................................................................ 12 1 T53
....................uuuggcacuUgcacauuuuu........................................................................ 1 1 T53
....................uuuUgcacuagcacauuuuu........................................................................ 1 1 T53
....................uuuggcacGagcacauuuuug....................................................................... 3 1 T53
....................uuuggcacuaAcacauuuuug....................................................................... 40 1 T53
....................uuuggcacuagcacaGuuuug....................................................................... 1 1 T53
....................uuuggcacuagUacauuuuug....................................................................... 8 1 T53
....................uuuggcacuagcacauuuCug....................................................................... 1 1 T53
....................uuuggcacuagcacauuuuCg....................................................................... 5 1 T53
....................uuuggcacuCgcacauuuuug....................................................................... 12 1 T53
....................uuuAgcacuagcacauuuuug....................................................................... 3 1 T53
....................uuuggcaAuagcacauuuuug....................................................................... 1 1 T53
....................uuuggcacuagAacauuuuug....................................................................... 4 1 T53
....................uuuggcacAagcacauuuuug....................................................................... 18 1 T53
....................Guuggcacuagcacauuuuug....................................................................... 11 1 T53
....................uuuggcacuaCcacauuuuug....................................................................... 6 1 T53
....................uuuggcacuagcUcauuuuug....................................................................... 5 1 T53
....................uuuggcacuagcacauuuuuC....................................................................... 14 1 T53
....................uuCggcacuagcacauuuuug....................................................................... 2 1 T53
....................uuGggcacuagcacauuuuug....................................................................... 12 1 T53
....................Cuuggcacuagcacauuuuug....................................................................... 22 1 T53
....................uuuggcacuagcacauuuuuU....................................................................... 383 1 T53
....................uuuggcacuagcacauuuAug....................................................................... 12 1 T53
....................uuuggcUcuagcacauuuuug....................................................................... 1 1 T53
....................uuuggcacCagcacauuuuug....................................................................... 3 1 T53
....................uuuggcacuagGacauuuuug....................................................................... 7 1 T53
....................uuuggcacuagcaUauuuuug....................................................................... 15 1 T53
....................uuuggcacuagcacauGuuug....................................................................... 1 1 T53
....................uuuggcacuUgcacauuuuug....................................................................... 3 1 T53
....................uuuggcacuagcacUuuuuug....................................................................... 28 1 T53
....................uuuggcacuagcacauuuuAg....................................................................... 12 1 T53
....................uuuggcacuagcacauuAuug....................................................................... 5 1 T53
....................uuuggcacuagcacauCuuug....................................................................... 7 1 T53
....................uuuggcacuGgcacauuuuug....................................................................... 3 1 T53
....................uuuggcacuaUcacauuuuug....................................................................... 4 1 T53
....................uuuggcaUuagcacauuuuug....................................................................... 6 1 T53
....................Auuggcacuagcacauuuuug....................................................................... 46 1 T53
....................uuuggcacuagcacauuuuuA....................................................................... 179 1 T53
....................uuugAcacuagcacauuuuug....................................................................... 17 1 T53
....................uuugCcacuagcacauuuuug....................................................................... 5 1 T53
....................uuuggcacuagcacaCuuuug....................................................................... 2 1 T53
....................uuuggUacuagcacauuuuug....................................................................... 78 1 T53
....................uuuggcacuagcacauAuuug....................................................................... 27 1 T53
....................uuuggGacuagcacauuuuug....................................................................... 12 1 T53
....................uuuggcacuagcacauuCuug....................................................................... 2 1 T53
....................uuuggAacuagcacauuuuug....................................................................... 2 1 T53
....................uuAggcacuagcacauuuuug....................................................................... 4 1 T53
....................uuuggcacuagcacauuGuug....................................................................... 4 1 T53
....................uCuggcacuagcacauuuuug....................................................................... 1 1 T53
....................uAuggcacuagcacauuuuug....................................................................... 1 1 T53
....................uuuUgcacuagcacauuuuug....................................................................... 2 1 T53
....................uuuggcacuagcaAauuuuug....................................................................... 3 1 T53
....................uuuggcacuagcacauuuuug....................................................................... 35122 0 T53
....................uuuggcacuagcaGauuuuug....................................................................... 3 1 T53
....................uuuggcacuagcacGuuuuug....................................................................... 7 1 T53
....................uGuggcacuagcacauuuuug....................................................................... 4 1 T53
....................uuuggcacuagcacaAuuuug....................................................................... 34 1 T53
....................uuugUcacuagcacauuuuug....................................................................... 6 1 T53
....................Cuuggcacuagcacauuuuugu...................................................................... 297 1 T53
....................Guuggcacuagcacauuuuugu...................................................................... 257 1 T53
....................uuuggcGcuagcacauuuuugu...................................................................... 21 1 T53
....................uuugAcacuagcacauuuuugu...................................................................... 395 1 T53
....................uuuggcacuagcacaAuuuugu...................................................................... 340 1 T53
....................uuuggcacuUgcacauuuuugu...................................................................... 21 1 T53
....................uuuggcacuaUcacauuuuugu...................................................................... 31 1 T53
```

```
                    Mature                                  Star
gcuaguccucuucucuguucuuuggcacuagcacauuuuugugugacgaucaaaccuacaaaauuguguuagugucgaauaauaugggaagacauucgucauaaaucauaca
....................uuuggcacuagGacauuuuugu......................................................................   143      1   T53
....................uuuggcacuagcacauuuuugC......................................................................   321      1   T53
....................uuuggcacuagcacauuuuCgu......................................................................   53       1   T53
....................uuuggcacGagcacauuuuugu......................................................................   12       1   T53
....................uuuggcaAuagcacauuuuugu......................................................................   28       1   T53
....................uCuggcacuagcacauuuuugu......................................................................   85       1   T53
....................uuugCcacuagcacauuuuugu......................................................................   124      1   T53
....................uuuAgcacuagcacauuuuugu......................................................................   60       1   T53
....................uAuggcacuagcacauuuuugu......................................................................   40       1   T53
....................uuuggcacuagcacaGuuuugu......................................................................   36       1   T53
....................uuuggcacuagcacGuuuuugu......................................................................   34       1   T53
....................uuuggcaGuagcacauuuuugu......................................................................   13       1   T53
....................uuuggUacuagcacauuuuugu......................................................................   1501     1   T53
....................uuuggcacuagcacauuCuugu......................................................................   43       1   T53
....................uuuggcacAagcacauuuuugu......................................................................   405      1   T53
....................Auuggcacuagcacauuuuugu......................................................................   1034     1   T53
....................uuuggcacuagcacaCuuuugu......................................................................   75       1   T53
....................uuGggcacuagcacauuuuugu......................................................................   172      1   T53
....................uuuggcacuagAacauuuuugu......................................................................   128      1   T53
....................uuuUgcacuagcacauuuuugu......................................................................   97       1   T53
....................uuuggcUcuagcacauuuuugu......................................................................   51       1   T53
....................uuuggcacuagcUcauuuuugu......................................................................   16       1   T53
....................uuuggcacuagcacauAuuugu......................................................................   470      1   T53
....................uuuggcacuGgcacauuuuugu......................................................................   49       1   T53
....................uuuggcacuagcacauuuuugG......................................................................   250      1   T53
....................uuuggcacuagcacauuuGugu......................................................................   33       1   T53
....................uuAggcacuagcacauuuuugu......................................................................   112      1   T53
....................uuuggcacuagcacUuuuuugu......................................................................   419      1   T53
....................uuuggcacuagcaAauuuuugu......................................................................   64       1   T53
....................uuuggcacuagcacauuuuuCu......................................................................   159      1   T53
....................uuuggcacuaCcacauuuuugu......................................................................   96       1   T53
....................uuuggcacuCgcacauuuuugu......................................................................   239      1   T53
....................uuuggcacuagcacauuuuuUu......................................................................   251      1   T53
....................uuuggcacuagcacauuuuugA......................................................................   6074     1   T53
....................uuuggcacCagcacauuuuugu......................................................................   35       1   T53
....................uuuggGacuagcacauuuuugu......................................................................   108      1   T53
....................uuuggcaUuagcacauuuuugu......................................................................   154      1   T53
....................uuuggcacuagcacauuuuuAu......................................................................   127      1   T53
....................uuuggcacuagUacauuuuugu......................................................................   241      1   T53
....................uuuggcacuagcacauuuuAgu......................................................................   121      1   T53
....................uuuggcacuagcacauCuuugu......................................................................   139      1   T53
....................uuuggcacuagcacauGuuugu......................................................................   95       1   T53
....................uuuggcacuagcCcauuuuugu......................................................................   1        1   T53
....................uuuggcacuagcaGauuuuugu......................................................................   46       1   T53
....................uuuggAacuagcacauuuuugu......................................................................   87       1   T53
....................uuCggcacuagcacauuuuugu......................................................................   59       1   T53
....................uuuggcacuaAcacauuuuugu......................................................................   766      1   T53
....................uuuggcacuagcaUauuuuugu......................................................................   149      1   T53
....................uuuggcCcuagcacauuuuugu......................................................................   1        1   T53
....................uuuggcacuagcacauuAuugu......................................................................   139      1   T53
....................uuuggcacuagcacCuuuuugu......................................................................   20       1   T53
....................uuuggcacuagcacauuuAugu......................................................................   113      1   T53
....................uuugUcacuagcacauuuuugu......................................................................   77       1   T53
....................uuuggcacuagcacauuGuugu......................................................................   102      1   T53
....................uuuCgcacuagcacauuuuugu......................................................................   58       1   T53
....................uuuggcacuagcGcauuuuugu......................................................................   23       1   T53
....................uuuggcacuagcacauuuuGgu......................................................................   62       1   T53
....................uGuggcacuagcacauuuuugu......................................................................   50       1   T53
....................uuuggcacuagcacauuuuugu......................................................................   740530   0   T53
....................uuuggcacuagcacauuuCugu......................................................................   66       1   T53
....................uuuggUacuagcacauuuuugug.....................................................................   1        1   T53
....................uuGggcacuagcacauuuuugug.....................................................................   1        1   T53
....................uuuggcacuagcacauuuuuguU.....................................................................   8578     1   T53
....................uuuggcacuagcacauuuuugug.....................................................................   747      0   T53
....................uuuggcacuagAacauuuuugug.....................................................................   1        1   T53
....................uuugCcacuagcacauuuuugug.....................................................................   1        1   T53
....................uuuggcacuagcacauuuuuguC.....................................................................   133      1   T53
....................uuuggcacuaAcacauuuuugug.....................................................................   2        1   T53
....................uuCggcacuagcacauuuuugug.....................................................................   1        1   T53
....................uuuggcaUuagcacauuuuugug.....................................................................   1        1   T53
```

Mature **Star**

gcuaguccucuucucuguucuuuggcacuagcacauuuuugugugacgaucaaaccuacaaaauuguguuagugucgaauaauaugggaagacauucgucauaaaucauaca			
....................uuuggcacuagcacauuuCugug...	1	1	T53
....................uuuggcacuagcacauuuuugCg...	1	1	T53
....................Auuggcacuagcacauuuuugug...	1	1	T53
....................Cuuggcacuagcacauuuuugug...	2	1	T53
....................uuuggcacuagcacauuuuuUug...	1	1	T53
....................uuuggcacuagcacauuuuuguA...	8934	1	T53
....................uuuggcacuagcacauuuuCgug...	1	1	T53
....................uuuggcacuagcacauuuuugAg...	19	1	T53
....................uuuggcacuagcacauuuuugugG..	10	1	T53
....................uuuggcacuagcacauuuuugAgu..	1	1	T53
....................uuuggcacuagcacauuuuuUugu..	4	1	T53
....................uuuggcacuagcacauuuuuCugu..	1	1	T53
....................uuuggcacuagcacauuuuugugA..	1046	1	T53
....................uuuggcacuagcacauuuuuguUu..	454	1	T53
....................uuuggcacuagcacauuuuugugu..	36	0	T53
....................uuuggcacuagcacauuuuuguCu..	10	1	T53
....................Guuggcacuagcacauuuuugugu..	1	1	T53
....................uuuggcacuagcacauuuuuguAu..	1902	1	T53
....................uuuggcacuagcacauuuuugugC..	2	1	T53
....................uuuggcacuagcacauuuuugugug...	1	0	T53
....................uuuggcacuagcacauuuuuguAug...	1	1	T53
....................uuuggcacuagcacauuuuuguguA...	1	1	T53
....................uuuggcacuagcacauuuuuguguga..	2	0	T53
....................uuGggcacuagcacauuuuuguguga..	1	1	T53
....................uuuggcacuagcacauuuuugugugac...	21	0	T53
....................uuuggcacuagcacauuuuugugugUc...	1	1	T53
.....................uuggcacuagcacauuGu...	3	1	T53
.....................uuggcacuagcacauuuu...	40	0	T53
.....................Auggcacuagcacauuuuu..	1	1	T53
.....................uuggcacuagcacauuuuu..	319	0	T53
.....................uuggcacuagcacauuuGu..	5	1	T53
.....................uuggcacuagcacauuCuu..	1	1	T53
.....................uugUcacuagcacauuuuu..	1	1	T53
.....................uuggcacuagcacauAuuug...	1	1	T53
.....................uuggcacuagcacauuuuug...	772	0	T53
.....................uAggcacuagcacauuuuug...	1	1	T53
.....................uuggcacuagcacauuAuug...	1	1	T53
.....................uuggcacuagcacauuuuuU...	8	1	T53
.....................uuggcacuagGacauuuuug...	1	1	T53
.....................uuggcacuagcacauuuuuA...	6	1	T53
.....................uuggcacuUgcacauuuuug...	1	1	T53
.....................uuggcGcuagcacauuuuug...	1	1	T53
.....................Cuggcacuagcacauuuuug...	1	1	T53
.....................uuggUacuagcacauuuuug...	2	1	T53
.....................uuggcacuaAcacauuuuug...	2	1	T53
.....................uuggcacuagcaUauuuuugu..	12	1	T53
.....................uuggcacuagcacCuuuuugu..	1	1	T53
.....................uuAgcacuagcacauuuuugu..	4	1	T53
.....................uuggcacuagcacauuuuCgu..	5	1	T53
.....................uuggcacuaCcacauuuuugu..	23	1	T53
.....................uuggcacuagcacauuuuugA..	102	1	T53
.....................uuggcacuagcacauuCuugu..	3	1	T53
.....................uuggcacuagcacUuuuuugu..	6	1	T53
.....................uuUgcacuagcacauuuuugu..	13	1	T53
.....................uuggcacuagcacaAuuuugu..	13	1	T53
.....................uuggcacuagGacauuuuugu..	12	1	T53
.....................uuggcacuagcacauAuuugu..	23	1	T53
.....................uuggAacuagcacauuuuugu..	1	1	T53
.....................uuggcacuagcGcauuuuugu..	4	1	T53
.....................uuggcacuagcaAauuuuugu..	3	1	T53
.....................uuggcCcuagcacauuuuugu..	3	1	T53
.....................uAggcacuagcacauuuuugu..	5	1	T53
.....................uuggcacuaUcacauuuuugu..	3	1	T53
.....................uuggcacuagcacauuuuAgu..	8	1	T53
.....................uuggcaUuagcacauuuuugu..	14	1	T53
.....................uuggcacuagcacauCuuugu..	4	1	T53
.....................uuggcGcuagcacauuuuugu..	6	1	T53
.....................uuggcacAagcacauuuuugu..	29	1	T53
.....................uuggcacuagcacauuuuuUu..	15	1	T53
.....................uGggcacuagcacauuuuugu..	12	1	T53

```
                    Mature                                  Star
gcuaguccucuucucuguucuuuggcacuagcacauuuuugugugacgaucaaaccuacaaaauuguguuagugucgaauaauaugggaagacauucgucauaaaucauaca
.....................uuggcacuagcacauuuCugu......................................................................   3       1   T53
.....................uuCgcacuagcacauuuuugu......................................................................   3       1   T53
.....................uuggcacuagUacauuuuugu......................................................................   24      1   T53
.....................uuggcacuagcacauuuuugG......................................................................   7       1   T53
.....................Cuggcacuagcacauuuuugu......................................................................   24      1   T53
.....................uuggcacuagcacauuuGugu......................................................................   6       1   T53
.....................uuggcacuagcacauuuAugu......................................................................   10      1   T53
.....................uuggcacuagcacauuuuugu......................................................................   53300   0   T53
.....................uuggcaGuagcacauuuuugu......................................................................   1       1   T53
.....................uCggcacuagcacauuuuugu......................................................................   9       1   T53
.....................uuggcacGagcacauuuuugu......................................................................   2       1   T53
.....................uuggcacuagcacauuAuugu......................................................................   33      1   T53
.....................uuggcacuagcacauuuuugC......................................................................   14      1   T53
.....................uuggcacuGgcacauuuuugu......................................................................   3       1   T53
.....................uuggcacuagcaGauuuuugu......................................................................   5       1   T53
.....................uuggcacuagcacauuuuuCu......................................................................   15      1   T53
.....................uuggcUcuagcacauuuuugu......................................................................   7       1   T53
.....................uugCcacuagcacauuuuugu......................................................................   5       1   T53
.....................uuggcacuagcacaGuuuugu......................................................................   3       1   T53
.....................uuggcacuagAacauuuuugu......................................................................   5       1   T53
.....................uuggUacuagcacauuuuugu......................................................................   88      1   T53
.....................uuggcacuagcacauGuuugu......................................................................   2       1   T53
.....................uuggcacuCgcacauuuuugu......................................................................   1       1   T53
.....................uuggcacuagcacauuuuuAu......................................................................   6       1   T53
.....................Guggcacuagcacauuuuugu......................................................................   24      1   T53
.....................uuggcacuUgcacauuuuugu......................................................................   19      1   T53
.....................uugUcacuagcacauuuuugu......................................................................   9       1   T53
.....................uuggcacuaAcacauuuuugu......................................................................   60      1   T53
.....................uuggcacuagcacaCuuuugu......................................................................   6       1   T53
.....................uuggcaAuagcacauuuuugu......................................................................   2       1   T53
.....................uuggcacCagcacauuuuugu......................................................................   2       1   T53
.....................uuggcacuagcUcauuuuugu......................................................................   5       1   T53
.....................uugAcacuagcacauuuuugu......................................................................   20      1   T53
.....................Auggcacuagcacauuuuugu......................................................................   121     1   T53
.....................uuggcacuagcacauuGuugu......................................................................   2       1   T53
.....................uuggcacuagcacauuuuugug.....................................................................   3414    0   T53
.....................uuggcacuagcacauuuuuguA.....................................................................   1721    1   T53
.....................uCggcacuagcacauuuuugug.....................................................................   3       1   T53
.....................uuggcacuagcacauuuuugAg.....................................................................   3       1   T53
.....................uuAgcacuagcacauuuuugug.....................................................................   1       1   T53
.....................uuggcacuagcacauuuuuguC.....................................................................   13      1   T53
.....................uuggcacuagcaAauuuuugug.....................................................................   1       1   T53
.....................uuggUacuagcacauuuuugug.....................................................................   6       1   T53
.....................uuggcacuagcacauuuuGgug.....................................................................   3       1   T53
.....................uuggcacuagcacauuAuugug.....................................................................   1       1   T53
.....................uuggcacuagcacaAuuuugug.....................................................................   1       1   T53
.....................uuggcacuagUacauuuuugug.....................................................................   1       1   T53
.....................uugAcacuagcacauuuuugug.....................................................................   1       1   T53
.....................uuggcacuagcacauuuuugCg.....................................................................   2       1   T53
.....................uugUcacuagcacauuuuugug.....................................................................   1       1   T53
.....................uuggcacuagcacauuuuuguU.....................................................................   563     1   T53
.....................uuggcacuagcGcauuuuugug.....................................................................   1       1   T53
.....................Auggcacuagcacauuuuugug.....................................................................   5       1   T53
.....................uuggcacuagcacaCuuuugug.....................................................................   1       1   T53
.....................uuggcacuaAcacauuuuugug.....................................................................   4       1   T53
.....................uuggcGcuagcacauuuuugug.....................................................................   2       1   T53
.....................Guggcacuagcacauuuuugug.....................................................................   1       1   T53
.....................uuggcacAagcacauuuuugug.....................................................................   1       1   T53
.....................uuggcaAuagcacauuuuugug.....................................................................   1       1   T53
.....................uuggcacuagcacaGuuuugug.....................................................................   1       1   T53
.....................uuggcacuagcacauuuGugug.....................................................................   1       1   T53
.....................uugAcacuagcacauuuuugugu....................................................................   1       1   T53
.....................uuggcacuagcacauuuuugugu....................................................................   115     0   T53
.....................uuggcacuagcacauuuuuguAu....................................................................   555     1   T53
.....................uuggcacuagcacauuuuuUugu....................................................................   2       1   T53
.....................uuggcacuagcacauuuuuguUu....................................................................   46      1   T53
.....................uuggcacuagcacauuuuugugC....................................................................   4       1   T53
.....................uuggcacuagcacauuuuugugA....................................................................   8137    1   T53
.....................uuggcacuagcacauuuuugugG....................................................................   89      1   T53
.....................uugCcacuagcacauuuuugugu....................................................................   1       1   T53
```

Mature Star

```
gcuaguccucuucucuguucuuuggcacuagcacauuuuuguguguacgaucaaaccuacaaaauuguguuagugucgaauaauaugggaagacauucgucauaaaucauaca
```

.....................uuggcacuagcacauuuuuguCu..	2	1	T53
.....................uuggcacuagcacauuuuuguguAa..	1	1	T53
.....................uuggcacuagcacauuuuuguAuga..	1	1	T53
......................uggcacuagcacauuuuu..	6	0	T53
......................uggcacuagcacauuuuug...	15	0	T53
......................ugAcacuagcacauuuuugu..	1	1	T53
......................uggUacuagcacauuuuugu..	1	1	T53
......................Aggcacuagcacauuuuugu..	3	1	T53
......................uggcacuagcacauuuuAgu..	1	1	T53
......................Gggcacuagcacauuuuugu..	3	1	T53
......................uggcacuagUacauuuuugu..	1	1	T53
......................uggcacuaAcacauuuuugu..	2	1	T53
......................uggcacuagcacauuuuugu..	1148	0	T53
......................uggcacuagcacauuuuugG..	1	1	T53
......................uggcaGuagcacauuuuugu..	1	1	T53
......................ugUcacuagcacauuuuugu..	1	1	T53
......................uggcacuagcacaAuuuugu..	3	1	T53
......................uggcacuagcacauuuuugug...	134	0	T53
......................uggcacuagcacauuuuuguU...	19	1	T53
......................uggcacuagcacauuuuuguA...	25	1	T53
......................uggcacuagcacaAuuuugugu..	1	1	T53
......................uggcacuagcacauuuuugugG..	5	1	T53
......................Aggcacuagcacauuuuugugu..	1	1	T53
......................uggcacuagcacauuuuugugu..	195	0	T53
......................uggcacuagcacauuuuugugC..	1	1	T53
......................uggcacuagcacauuuuugugA..	615	1	T53
......................uggcacuagcacauuAuugugu..	1	1	T53
......................uggUacuagcacauuuuugugu..	1	1	T53
......................uggcacuagcacauuuuuguUu..	1	1	T53
......................uggcaUuagcacauuuuugugu..	1	1	T53
......................uggcacuagcacauuuuuguAu..	20	1	T53
......................uggcacuagcacauuuuugugAg...	3	1	T53
......................uggcacuagcacauuuuuguguA...	5	1	T53
......................uggcacuagcacauuuuugugug...	1	0	T53
......................uggcacuagcacauuuuuguguUa..	1	1	T53
.......................ggcacuagcacauuuuug...	3	0	T53
.......................ggcacuagcacauuuuugu..	68	0	T53
.......................ggcacuagcacauuuuugA..	1	1	T53
.......................Ugcacuagcacauuuuugu..	1	1	T53
.......................ggcacuagcacauAuuugu..	1	1	T53
.......................ggcacuagcacauuuuuguU...	1	1	T53
.......................ggcacuagcacauuuuugug...	1	0	T53
........................gcacuagcacauuuuugu..	30	0	T53
........................gcacuagcacauuuuuguU...	1	1	T53
........................gcacuagcacauuuuuguA...	2	1	T53
........................gcacuagcacauuuuugugA..	1	1	T53
.........................cacuagcacauuuuugug...	2	0	T53
.........................cacuagcacauuuuuguU...	8	1	T53
.........................cacuagcacauuuuuguC...	1	1	T53
.........................cacuagcacauuuuuguA...	28	1	T53
.........................cacuagcacauuuuuguUu..	2	1	T53
.........................cacuagcacauuuuuguAu..	1	1	T53
.........................cacuagcacauuuuugugA..	2	1	T53
....gugacgaucaaaccuaca..	1	0	T53
....ccuacaaaauuguguuagugucgaau................................	2	0	T53
....uacaaaauuguguuagugu.....................................	2	0	T53
....uacaaaauuguguuaguguc....................................	8	0	T53
....uaAaaaauuguguuagugucg...................................	1	1	T53
....uacaaaauuguguuagugucg...................................	311	0	T53
....uacaaaauuguguCagugucg...................................	1	1	T53
....uacaaaauuguguAagugucg...................................	2	1	T53
....uacaaaauuguguGagugucg...................................	1	1	T53
....uacaaaauAguguuagugucg...................................	1	1	T53
....Aacaaaauuguguuagugucg...................................	2	1	T53
....uacaaaauuguguuagugucgU..................................	2	1	T53
....uacaaaauuguguuagugucga..................................	16	0	T53
....uacaaaauuguguuagugucgaU.................................	1	1	T53
....uacaaaauuguguuagugucgaa.................................	3	0	T53
....uacaaaauuguguuagugucgaau................................	5	0	T53
....uacaaaauuguguuagugucgaaA................................	1	1	T53

```
                    Mature                                Star
gcuaguccucuucucuguucuuuggcacuagcacauuuuugugugacgaucaaaccuacaaaauuguguuagugucgaauaauaugggaagacauucgucauaaaucauaca
.........................................................acaaaauuguguuagugucg...................................    3     0    T53
.........................................................acaaaauuguguuagugucga..................................    17    0    T53
.........................................................acUaaauuguguuagugucga..................................    1     1    T53
.........................................................acaaaauuguguuagugucgU..................................    1     1    T53
.........................................................acaaaauuguguuagugGcga..................................    1     1    T53
.........................................................acaaaauuguguuagugucgaU.................................    1     1    T53
.........................................................acaaaauuguguuagugucgaa.................................    4     0    T53
.........................................................acaaaauuguguuagugucgaau................................    5     0    T53
.........................................................aAaaaauuguguuagugucgaau................................    1     1    T53
..........................................................caaaauuguguuaguguc....................................    1     0    T53
..........................................................caaaauuguguuagugucg...................................    14    0    T53
..........................................................Uaaaauuguguuagugucga..................................    1     1    T53
..........................................................caaaauAguguuagugucga..................................    1     1    T53
..........................................................caUaauuguguuagugucga..................................    1     1    T53
..........................................................caaaauuguguuagugucga..................................    309   0    T53
..........................................................caaaUuuguguuagugucga..................................    1     1    T53
..........................................................caaaauuguguuagugucgG..................................    1     1    T53
..........................................................caaaauugAguuagugucga..................................    1     1    T53
..........................................................caaaauuguguuaguAucga..................................    2     1    T53
..........................................................caaaauuguguuagugucgaa.................................    94    0    T53
..........................................................caaaauuguguuagugucgaU.................................    1     1    T53
..........................................................caaaauuguguuagugucgGa.................................    1     1    T53
..........................................................caaaauuguguuagugucgaUu................................    1     1    T53
..........................................................caaaauugGguuagugucgaau................................    1     1    T53
..........................................................caaaauuguguuagugucAaau................................    1     1    T53
..........................................................caaaauuguguuagugucgaaC................................    6     1    T53
..........................................................caaaauuguguuagugucgUau................................    3     1    T53
..........................................................caaaaAuguguuagugucgaau................................    1     1    T53
..........................................................caaaauuguguuagugucUaau................................    1     1    T53
..........................................................caaaauuguguuagugucgaaG................................    4     1    T53
..........................................................caaaauuguguuagugucgaau................................    852   0    T53
..........................................................caaaauuguguuagugAcgaau................................    1     1    T53
..........................................................caGaauuguguuagugucgaau................................    1     1    T53
..........................................................caaaauuguguuagugucgaaA................................    50    1    T53
..........................................................caaaauuguguAagugucgaau................................    1     1    T53
..........................................................caaaauuguguuagugucgUaua...............................    1     1    T53
..........................................................caaaauuguguuagugucgaaua...............................    108   0    T53
..........................................................caaaauuguguuagugucgaaGa...............................    2     1    T53
..........................................................caaaauuguguuagugucgaauC...............................    6     1    T53
..........................................................caaaauuguguuagugucgaauU...............................    50    1    T53
..........................................................caaaauuguguuagugucgaaAa...............................    13    1    T53
..........................................................caaaauuguguuagugucgaaCa...............................    2     1    T53
..........................................................caaaauuguguuagugucgaauG...............................    3     1    T53
..........................................................caaaauuguguuagugucgaauGa..............................    1     1    T53
..........................................................caaaauuguguuagugucgaaAaa..............................    4     1    T53
..........................................................caaaauuguguuagugucgaauUa..............................    2     1    T53
..........................................................caaaauuguguuagugucgaauaa..............................    1     0    T53
...........................................................aaUauuguguuagugucg...................................    1     1    T53
...........................................................aaaauuguguuaguguUg...................................    1     1    T53
...........................................................aaaauuguguuagugucA...................................    1     1    T53
...........................................................aaaauuguguuagugucg...................................    70    0    T53
...........................................................aGaauuguguuagugucga..................................    1     1    T53
...........................................................aCaauuguguuagugucga..................................    1     1    T53
...........................................................aaUauuguguuagugucga..................................    2     1    T53
...........................................................aaaauugugCuagugucga..................................    2     1    T53
...........................................................aaaauuguguuagugucAa..................................    2     1    T53
...........................................................aaaauuguguuagAgucga..................................    3     1    T53
...........................................................aaaauuguguuagugucgU..................................    4     1    T53
...........................................................aaaauAguguuagugucga..................................    1     1    T53
...........................................................aaaauuguguuaCugucga..................................    1     1    T53
...........................................................aaaauuguguuagugucga..................................    1152  0    T53
...........................................................aUaauuguguuagugucga..................................    1     1    T53
...........................................................aaaauuUuguuagugucga..................................    2     1    T53
...........................................................aaaauuguguuagugucgGa.................................    3     1    T53
...........................................................aaaauuguguuagugucgaC.................................    1     1    T53
...........................................................aUaauuguguuagugucgaa.................................    2     1    T53
...........................................................aaaauugGguuagugucgaa.................................    1     1    T53
...........................................................aaaauuguguuagugucgaU.................................    32    1    T53
...........................................................aaaauuguguuagugucgUa.................................    1     1    T53
...........................................................aaaauugugCuagugucgaa.................................    1     1    T53
```

```
                    Mature                                    Star
gcuagucucuucucuguucuuuggcacuagcacauuuuugugugacgaucaaaccuacaaaauuguguuagugucgaauaauaugggaagacauucgucauaaaucauaca
..........................................................aaaauuguguuaguUucgaa.................................   1      1     T53
..........................................................aaaauuguguuagugucgaa.................................   536    0     T53
..........................................................aaUauuguguuagugucgaau................................   2      1     T53
..........................................................aaaUuuguguuagugucgaau................................   1      1     T53
..........................................................aaaauuAuguuagugucgaau................................   1      1     T53
..........................................................aaaauuguguuGgugucgaau................................   3      1     T53
..........................................................aaaauuguguuaguAucgaau................................   1      1     T53
..........................................................aaaauGguguuagugucgaau................................   1      1     T53
..........................................................aaaauuguguuagugucgaUu................................   7      1     T53
..........................................................aaaauuguguuagugucgaaG................................   18     1     T53
..........................................................aaaauuguguuaguguGgaau................................   2      1     T53
..........................................................aaGauuguguuagugucgaau................................   1      1     T53
..........................................................aCaauuguguuagugucgaau................................   1      1     T53
..........................................................aaaauuguguuagugucgUau................................   10     1     T53
..........................................................aaaauuguguuagugucAaau................................   3      1     T53
..........................................................aaaauuguguuaCugucgaau................................   1      1     T53
..........................................................aaaauuguguAagugucgaau................................   1      1     T53
..........................................................aUaauuguguuagugucgaau................................   2      1     T53
..........................................................aaaauuguguuagugCcgaau................................   1      1     T53
..........................................................aaaauugugAuagugucgaau................................   1      1     T53
..........................................................aaaauAguguuagugucgaau................................   1      1     T53
..........................................................aaaauuguguuagugucgaaC................................   50     1     T53
..........................................................aaaauuguguuagugAcgaau................................   2      1     T53
..........................................................aaaauuguguuagAgucgaau................................   5      1     T53
..........................................................aaaauugGguuagugucgaau................................   1      1     T53
..........................................................aaaauuguguCagugucgaau................................   3      1     T53
..........................................................aaaauuguguuagCgucgaau................................   1      1     T53
..........................................................aaaauuguAuuagugucgaau................................   2      1     T53
..........................................................aaaauuguguuagugucgaaA................................   260    1     T53
..........................................................aGaauuguguuagugucgaau................................   2      1     T53
..........................................................aaaauugAguuagugucgaau................................   10     1     T53
..........................................................aaaauuguguuagugucgaau................................   4572   0     T53
..........................................................aaaauuguguuaguguUgaau................................   1      1     T53
..........................................................aaaauugCguuagugucgaau................................   2      1     T53
..........................................................aaaauuguguuagugucgaauU...............................   387    1     T53
..........................................................aaaauuguguuagugucgaauG...............................   35     1     T53
..........................................................aaaauuguguuagugucgaaGa...............................   7      1     T53
..........................................................aaaauGguguuagugucgaaua...............................   1      1     T53
..........................................................aaaauuguguuaguUucgaaua...............................   2      1     T53
..........................................................aaaauuguguuagugGcgaaua...............................   1      1     T53
..........................................................aaaauAguguuagugucgaaua...............................   3      1     T53
..........................................................aGaauuguguuagugucgaaua...............................   1      1     T53
..........................................................aaaauuguguuagugucgaaAa...............................   95     1     T53
..........................................................aaaauCguguuagugucgaaua...............................   2      1     T53
..........................................................aaaauuguguuagugucgaaua...............................   4813   0     T53
..........................................................aaaauuguguuagGgucgaaua...............................   1      1     T53
..........................................................aaaauuguguAagugucgaaua...............................   1      1     T53
..........................................................aUaauuguguuagugucgaaua...............................   2      1     T53
..........................................................aaaauuguguuUgugucgaaua...............................   1      1     T53
..........................................................aaaauuguguuagugucgaaCa...............................   73     1     T53
..........................................................aaaauuguguuaguguUgaaua...............................   2      1     T53
..........................................................aaUauuguguuagugucgaaua...............................   10     1     T53
..........................................................aaaauuguCuuagugucgaaua...............................   1      1     T53
..........................................................aaaauuguguuGgugucgaaua...............................   2      1     T53
..........................................................aaaauuguguuagugucgaauC...............................   30     1     T53
..........................................................aaaauuguguuagugucgUaua...............................   9      1     T53
..........................................................aaaauuguguuaguguGgaaua...............................   1      1     T53
..........................................................aaaauuguguuagugAcgaaua...............................   2      1     T53
..........................................................aaaauuguguuagugucUaaua...............................   1      1     T53
..........................................................aaaauuguguuaguAucgaaua...............................   2      1     T53
..........................................................aaaauuguguuagAgucgaaua...............................   4      1     T53
..........................................................aaaauuguguuagugucAaaua...............................   3      1     T53
..........................................................aaaauuguguuagugucgaUua...............................   1      1     T53
..........................................................aaaauuguguuagugucgaGua...............................   2      1     T53
..........................................................aaaauugAguuagugucgaaua...............................   8      1     T53
..........................................................aaaauuguAuuagugucgaaua...............................   5      1     T53
..........................................................aaaauuguguuagugCcgaaua...............................   2      1     T53
..........................................................aaaauugugCuagugucgaaua...............................   2      1     T53
..........................................................aaaauuguguuagugucCaaua...............................   1      1     T53
..........................................................aaaauuguguuagugucgaaGaa..............................   1      1     T53
```

```
                    Mature                                      Star
gcuaguccucuucucuguucuuuggcacuagcacauuuuugugugacgaucaaaccuacaaaauuguguuagugucgaauaauaugggaagacauucgucauaaaucauaca
...........................................................aaaauuguguuagugucgaauUa..............................    16    1    T53
...........................................................aaaauuguguuagugucgaauCa..............................    1     1    T53
...........................................................aaaauuguguuagugucgaauaU..............................    42    1    T53
...........................................................aaaauuguguuagugucgaauGa..............................    3     1    T53
...........................................................aaaauuguguuagugucgaauaC..............................    21    1    T53
...........................................................aaaauuguguuagugucgaaAaa..............................    7     1    T53
...........................................................aaaauuguguuagugucgaauaa..............................    33    0    T53
...........................................................aaaauuguguuagugucgaauaau.............................    2     0    T53
...........................................................aaaauuguguuagugucgaaGaau.............................    2     1    T53
...........................................................aaaauuguguuagugucgaaAaau.............................    1     1    T53
............................................................aaauuguguuagugucga..................................    6     0    T53
............................................................aaauuguguuagugucgaa.................................    3     0    T53
............................................................aaauuguguuagugucgaaA................................    1     1    T53
............................................................aaauuguguuagugucgaaG................................    1     1    T53
............................................................aaauuguguuagugucgaau................................    108   0    T53
............................................................aCauuguguuagugucgaaua...............................    1     1    T53
............................................................aaauuguUuuagugucgaaua...............................    1     1    T53
............................................................aaauugCguuagugucgaaua...............................    1     1    T53
............................................................aaauuguguuagugucgUaua...............................    1     1    T53
............................................................aaauuguguuagugucgaauU...............................    23    1    T53
............................................................aaauuguguuagugucgaaCa...............................    1     1    T53
............................................................aaauuguAuuagugucgaaua...............................    1     1    T53
............................................................aaauuguguuagugucgaaua...............................    936   0    T53
............................................................aaaCuguguuagugucgaaua...............................    1     1    T53
............................................................aaauuguguuagugucgaaAa...............................    3     1    T53
............................................................aaUuuguguuagugucgaaua...............................    2     1    T53
............................................................aaauuguguuagugucAaaua...............................    1     1    T53
............................................................aaauugAguuagugucgaaua...............................    1     1    T53
............................................................aaauuguguuagCgucgaaua...............................    1     1    T53
............................................................aaauuguguuaguguUaaua...............................    1     1    T53
............................................................aaauuguguuagugucgaauC...............................    2     1    T53
............................................................aaauuguguuagugucgaauaa..............................    9     0    T53
............................................................aaauuguguuagugucgaauaC..............................    5     1    T53
............................................................aaauuguguuagugucgaauaU..............................    8     1    T53
............................................................aaauuguguuagugucgaauaau.............................    1     0    T53
.............................................................aauuguguuagugucgaa.................................    1     0    T53
.............................................................aauuguguuagugucgaaua...............................    4     0    T53
.............................................................aauuguguuagugucgaauaUu.............................    1     1    T53
...............................................................uuguguuagugucgaaCa...............................    1     1    T53
```

Provisional ID	:	Scaffold_1043_2939
Score total	:	4.1
Score for star read(s)	:	-1.3
Score for read counts	:	0
Score for mfe	:	0.8
Score for randfold	:	1.6
Score for cons. seed	:	3
Total read count	:	506476
Mature read count	:	501559
Loop read count	:	14
Star read count	:	4903

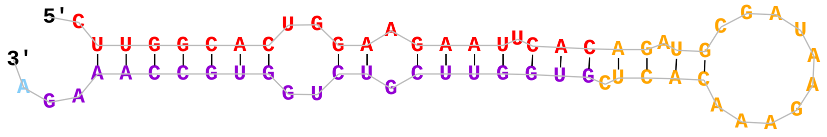

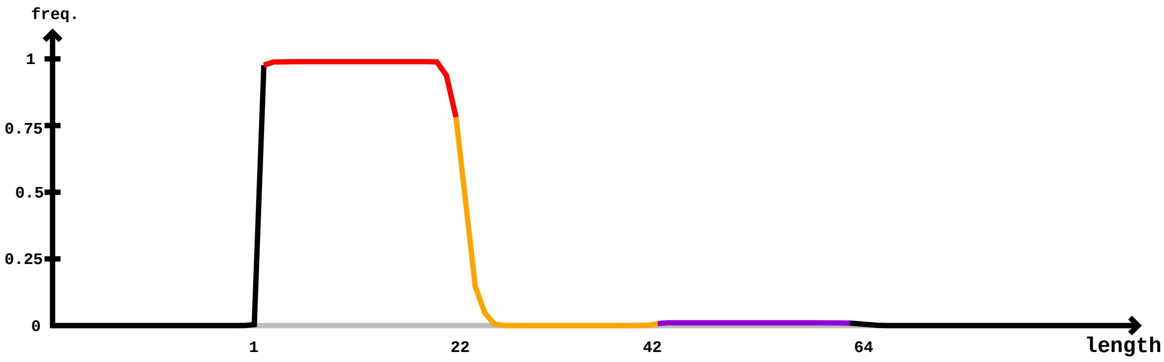

Mature Star

```
5'- acaauuggccuuaccuuauccuuggcacuggaagaauucacagaugcgauaagaaacacucgugguucgucuggugccaaagauaaacgcaagguauacacucugaacuuu  -3'  obs
    acaauuggccuuaccuuauccuuggcacuggaagaauucacagaugcgauaagaaacacucgugguucgucuggugccaaagauaaacgcaagguauacacucugaacuuu        exp
    .......(((((.(.(((((.(((((((..((.((((.(((((.((..........)))).))))))).))..))))))).)))))..).)))))................  reads  mm  sample
    ..................uUcuuggcacuggaagaauuc........................................................................  2  1  T63
    ..................uUcuuggcacuggaagaauucac......................................................................  4  1  T63
    ..................uUcuuggcacuggaagaauucaca.....................................................................  3  1  T63
    ...................ccuuggcacuggaagaauu.........................................................................  1  0  T63
    ...................ccuuggUacuggaagaauuc........................................................................  2  1  T63
    ...................Acuuggcacuggaagaauuc........................................................................  2  1  T63
    ...................Gcuuggcacuggaagaauuc........................................................................  1  1  T63
    ...................ccuuggcacuggaagaauuA........................................................................  1  1  T63
    ...................Ucuuggcacuggaagaauuc........................................................................  2  1  T63
    ...................ccuuggcacuggaagaauuc........................................................................  47  0  T63
    ...................ccuuggcacuggaagGauuc........................................................................  1  1  T63
    ...................Ucuuggcacuggaagaauuca.......................................................................  2  1  T63
    ...................ccuuggcacuggaagaauuca.......................................................................  25  0  T63
    ...................ccuuggcacuggaagaauucU.......................................................................  1  1  T63
    ...................Acuuggcacuggaagaauuca.......................................................................  2  1  T63
    ...................ccuuggcacuggaagaauucac......................................................................  23  0  T63
    ...................Acuuggcacuggaagaauucac......................................................................  1  1  T63
    ...................Ucuuggcacuggaagaauucac......................................................................  1  1  T63
    ...................ccuuggcacuggaagaauucaA.....................................................................  1  1  T63
    ...................ccuuggcacuggaagaauucaca.....................................................................  1  0  T63
    ....................cuuggcacuggaagaauA.........................................................................  2  1  T63
    ....................cuuggcacuggaagaaAu.........................................................................  1  1  T63
    ....................cuuggcacuggaagaauu.........................................................................  157  0  T63
    ....................cuGggcacuggaagaauu.........................................................................  1  1  T63
    ....................cuuggcacuggaagaauC.........................................................................  1  1  T63
    ....................cuugAcacuggaagaauuc........................................................................  2  1  T63
    ....................cuuggcacuggaagGauuc........................................................................  8  1  T63
    ....................cuuggcacAggaagaauuc........................................................................  1  1  T63
    ....................cuuggcacuggUagaauuc........................................................................  8  1  T63
    ....................cuuggcacuggaagaauuc........................................................................  5125  0  T63
    ....................cuugUcacuggaagaauuc........................................................................  1  1  T63
    ....................cuuggcacuggGagaauuc........................................................................  4  1  T63
    ....................cuuggcacuggaagaauGc........................................................................  1  1  T63
```

Mature   Star

```
acaauuggccuuaccuuauccuuggcacuggaagaauucacagaugcgauaagaaacacucgugguucgucuggugccaaagauaaacgcaagguauacacucugaacuuu
....................cuuggcacuggaagaaCuc........................................................................  2     1  T63
....................cuuggcacCggaagaauuc........................................................................  2     1  T63
....................cuuUgcacuggaagaauuc........................................................................  1     1  T63
....................cuuggcacuggaaCaauuc........................................................................  1     1  T63
....................cuuggcacuggaagaUuuc........................................................................  2     1  T63
....................cuuggcacuggaagaauuA........................................................................  83    1  T63
....................cuCggcacuggaagaauuc........................................................................  1     1  T63
....................cuuggcaUuggaagaauuc........................................................................  2     1  T63
....................cuuggcacuggaagCauuc........................................................................  1     1  T63
....................cuuggcacuggaagaauAc........................................................................  1     1  T63
....................cuuggcaGuggaagaauuc........................................................................  1     1  T63
....................cuuggGacuggaagaauuc........................................................................  1     1  T63
....................cGuggcacuggaagaauuc........................................................................  1     1  T63
....................cuuggcacuggCagaauuc........................................................................  1     1  T63
....................cuuggcacugUaagaauuc........................................................................  1     1  T63
....................cuuggcacuggaaUaauuc........................................................................  2     1  T63
....................cuuggcacuggaagaaGuc........................................................................  2     1  T63
....................cuuggcacuggaagaauuU........................................................................  18    1  T63
....................cuuggcacuggaagaauCc........................................................................  2     1  T63
....................cCuggcacuggaagaauuc........................................................................  1     1  T63
....................Uuuggcacuggaagaauuc........................................................................  4     1  T63
....................cuuggUacuggaagaauuc........................................................................  3     1  T63
....................cuuggcacuggaagUauuc........................................................................  11    1  T63
....................cuuggcacuggaagaauucG.......................................................................  8     1  T63
....................cuuggcacuggaagaauucU.......................................................................  21    1  T63
....................cuuggAacuggaagaauuca.......................................................................  1     1  T63
....................cuuggcacuAgaagaauuca.......................................................................  1     1  T63
....................cuuUgcacuggaagaauuca.......................................................................  1     1  T63
....................cuuAgcacuggaagaauuca.......................................................................  1     1  T63
....................cuuggcacuUgaagaauuca.......................................................................  1     1  T63
....................cuuggcacAggaagaauuca.......................................................................  5     1  T63
....................cuuggcacuggaagaauuAa.......................................................................  33    1  T63
....................cuuggcacuggGagaauuca.......................................................................  3     1  T63
....................cuuggcacuggaagaauuca.......................................................................  4527  0  T63
....................cuuggcGcuggaagaauuca.......................................................................  1     1  T63
....................cuuggcaAuggaagaauuca.......................................................................  1     1  T63
....................cuuggcacuggaaUaauuca.......................................................................  3     1  T63
....................Uuuggcacuggaagaauuca.......................................................................  3     1  T63
....................cuuggcacuggUagaauuca.......................................................................  2     1  T63
....................cuugCcacuggaagaauuca.......................................................................  1     1  T63
....................cuuggcacugAaagaauuca.......................................................................  1     1  T63
....................cuuggcacuggaagCauuca.......................................................................  2     1  T63
....................cuuggcacuggaagGauuca.......................................................................  6     1  T63
....................cuugUcacuggaagaauuca.......................................................................  2     1  T63
....................cuuggcaUuggaagaauuca.......................................................................  1     1  T63
....................cuuggcacuggaagUauuca.......................................................................  12    1  T63
....................cuuggcacuggaagaaAuca.......................................................................  2     1  T63
....................cuuggcacugCaagaauuca.......................................................................  1     1  T63
....................cCuggcacuggaagaauuca.......................................................................  2     1  T63
....................cuuggcacuggaaCaauuca.......................................................................  3     1  T63
....................Auuggcacuggaagaauuca.......................................................................  2     1  T63
....................cuuggcacuggaagaUuuca.......................................................................  2     1  T63
....................cuuggUacuggaagaauuca.......................................................................  7     1  T63
....................cuuggcacuggaagaauuUa.......................................................................  2     1  T63
....................cuuggcacuggaaAaauuca.......................................................................  1     1  T63
....................Uuuggcacuggaagaauucac......................................................................  4     1  T63
....................cuuggcacuggaagaaGucac......................................................................  2     1  T63
....................cuuggcacuggaagaCuucac......................................................................  1     1  T63
....................cuuggcacuggaGgaauucac......................................................................  1     1  T63
....................Auuggcacuggaagaauucac......................................................................  1     1  T63
....................cuuggcacuggUagaauucac......................................................................  9     1  T63
....................cuuggcacuggaagaauucac......................................................................  8873  0  T63
....................cuuggcacuCgaagaauucac......................................................................  4     1  T63
....................cuuggcacuggaagaauGcac......................................................................  1     1  T63
....................cuuggcacAggaagaauucac......................................................................  8     1  T63
....................cuugAcacuggaagaauucac......................................................................  2     1  T63
....................cuuggcacuggaagaauucaG......................................................................  1     1  T63
....................cuuggcaAuggaagaauucac......................................................................  1     1  T63
....................cuuggcacuggaagaUuucac......................................................................  8     1  T63
....................cuuggcacuAgaagaauucac......................................................................  1     1  T63
```

Mature **Star**

```
acaauuggccuuaccuuauccuuggcacuggaagaauucacagaugcgauaagaaacacucgugguucgucuggugccaaagauaaacgcaagguauacacucugaacuuu
....................cuuggcacuggaagUauucac......................................................................   13    1  T63
....................cuuggcacuggaagaauuUac......................................................................   6     1  T63
....................cuuggcacGgaagaauucac......................................................................   1     1  T63
....................cuuggcacuggaagGauucac......................................................................   22    1  T63
....................cuuggcacuggaagaauucaU......................................................................   52    1  T63
....................cuuggGacuggaagaauucac......................................................................   1     1  T63
....................cuuggcacugUaagaauucac......................................................................   2     1  T63
....................cuuggcacuggaUgaauucac......................................................................   1     1  T63
....................cuuggcacuggGagaauucac......................................................................   6     1  T63
....................cuCggcacuggaagaauucac......................................................................   3     1  T63
....................cuuggcacuUgaagaauucac......................................................................   1     1  T63
....................cuuggcacuggaagaauAcac......................................................................   5     1  T63
....................cuuggcacuggaaAaauucac......................................................................   2     1  T63
....................cuuggcacuggaagCauucac......................................................................   2     1  T63
....................cuuggcacugAaagaauucac......................................................................   1     1  T63
....................cuuggUacuggaagaauucac......................................................................   10    1  T63
....................cuugUcacuggaagaauucac......................................................................   1     1  T63
....................cuuggcacuggaagaaAucac......................................................................   11    1  T63
....................cuuggcacuggaaUaauucac......................................................................   2     1  T63
....................cuuggcacuggaagaauCcac......................................................................   2     1  T63
....................cuuggcacuggaagaauucaA......................................................................   382   1  T63
....................cuuggAacuggaagaauucac......................................................................   1     1  T63
....................cuuggcacCggaagaauucac......................................................................   2     1  T63
....................cCuggcacuggaagaauucac......................................................................   1     1  T63
....................cuuggcaUuggaagaauucac......................................................................   1     1  T63
....................cuuggcaUuggaagaauucaca.....................................................................   2     1  T63
....................cuuggcacuggaagaauucaca.....................................................................   4463  0  T63
....................cuuggcacuggaagaUuucaca.....................................................................   2     1  T63
....................cCuggcacuggaagaauucaca.....................................................................   1     1  T63
....................cuuggcacuggaagGauucaca.....................................................................   7     1  T63
....................cuugCcacuggaagaauucaca.....................................................................   1     1  T63
....................cuuggcacuggaUgaauucaca.....................................................................   1     1  T63
....................cuuggcacuggaagaaAucaca.....................................................................   6     1  T63
....................cuuggcacuggaagaauuGaca.....................................................................   1     1  T63
....................cuuggcacuggaagaauuUaca.....................................................................   1     1  T63
....................cuuggcacuAgaagaauucaca.....................................................................   1     1  T63
....................cuuggUacuggaagaauucaca.....................................................................   8     1  T63
....................cuuggcacuCgaagaauucaca.....................................................................   2     1  T63
....................cuuggcacCggaagaauucaca.....................................................................   1     1  T63
....................cuuggcacuggaagaauucacC.....................................................................   2     1  T63
....................cuuggcacuggaagaauucacU.....................................................................   242   1  T63
....................cuuggcacugUaagaauucaca.....................................................................   2     1  T63
....................cuuggcacuggaagaauucaUa.....................................................................   2     1  T63
....................cuuggGacuggaagaauucaca.....................................................................   1     1  T63
....................cuuggcacuggUagaauucaca.....................................................................   4     1  T63
....................cuuggcacuggaagaauucaAa.....................................................................   47    1  T63
....................cuugAcacuggaagaauucaca.....................................................................   2     1  T63
....................cuuCgcacuggaagaauucaca.....................................................................   1     1  T63
....................cuCggcacuggaagaauucaca.....................................................................   1     1  T63
....................cuuggcacuggCagaauucaca.....................................................................   2     1  T63
....................cuuggcacuggaagUauucaca.....................................................................   9     1  T63
....................cuuggcacuggGagaauucaca.....................................................................   3     1  T63
....................cGuggcacuggaagaauucaca.....................................................................   2     1  T63
....................cuuggcacuggaaAaauucaca.....................................................................   1     1  T63
....................cuuggcacuggaagaauuAaca.....................................................................   1     1  T63
....................cuuggcacuggaagaauucacG.....................................................................   10    1  T63
....................cuuggcacuggaagaauucacaA....................................................................   62    1  T63
....................cuuggUacuggaagaauucacag....................................................................   2     1  T63
....................cuuggcacuggaagaaAucacag....................................................................   1     1  T63
....................cuuggcacuggUagaauucacag....................................................................   1     1  T63
....................cuuggcacuggaagaauucaUag....................................................................   1     1  T63
....................cuuggcacuAgaagaauucacag....................................................................   1     1  T63
....................cuuggcacuggaUgaauucacag....................................................................   1     1  T63
....................cuuggcacuggaagaauucacaU....................................................................   74    1  T63
....................cuuggcacuggaagUauucacag....................................................................   2     1  T63
....................cuuggcacAggaagaauucacag....................................................................   1     1  T63
....................cuuggcacuggaagaauucacag....................................................................   1226  0  T63
....................cuuggcacugAaagaauucacag....................................................................   1     1  T63
....................cuuggcacuggaagaauucacaC....................................................................   46    1  T63
....................cuuggcacuggaagaauucacGg....................................................................   2     1  T63
```

```
                    Mature                                   Star
acaauuggccuuaccuuauccuuggcacuggaagaauucacagaugcgauaagaaacacucgugguucgucuggugccaaagauaaacgcaagguauacacucugaacuuu
....................cuCggcacuggaagaauucacag.................................................................... 1 1 T63
....................cuuggcacuggaagaauucacCg.................................................................... 1 1 T63
....................cuuggcacuggaagUauucacaga................................................................... 1 1 T63
....................cuuggcacuggaagaauucacaUa................................................................... 1 1 T63
....................cuuggcacuggaagaauucacagG................................................................... 3 1 T63
....................cuuggcacuCgaagaauucacaga................................................................... 1 1 T63
....................cuuggcacuggGagaauucacaga................................................................... 1 1 T63
....................cuugUcacuggaagaauucacaga................................................................... 1 1 T63
....................cuuggcacuggaagaauucacagC................................................................... 1 1 T63
....................cuuggcacuggaagaauucacaCa................................................................... 2 1 T63
....................cuuggcacuggaagaauucacUga................................................................... 1 1 T63
....................cuuggcacuggaagaauucacagU................................................................... 29 1 T63
....................cuuggcacuggaaAaauucacaga................................................................... 2 1 T63
....................cuuggcacuggaagaauucacaAa................................................................... 3 1 T63
....................cuuggcacuggaagaauucacaga................................................................... 535 0 T63
....................cuuggcacuggaagaauucacagUu.................................................................. 2 1 T63
....................cuuggcacuggaagaauucacagaA.................................................................. 23 1 T63
....................cuuggcacuggaagaauucacagau.................................................................. 31 0 T63
....................cuuggcacuggaagaauucacagaC.................................................................. 1 1 T63
....................cuuggcacuggUagaauucacagau.................................................................. 1 1 T63
....................cuuggcacuggaagaauucacagauU................................................................. 1 1 T63
....................cuuggcacuggaagaauucacagauA................................................................. 2 1 T63
.....................uuggcacuggaagaauuc........................................................................ 114 0 T63
.....................uuggcacuggaagUauuc........................................................................ 2 1 T63
.....................uuggcacuggaagaauuUa....................................................................... 1 1 T63
.....................uuggcacuggaagaauuca....................................................................... 97 0 T63
.....................uuggcacAggaagaauucac...................................................................... 1 1 T63
.....................uuggcacuggaagaauucaA...................................................................... 2 1 T63
.....................Cuggcacuggaagaauucac...................................................................... 1 1 T63
.....................uuggcacuggaagaauucac...................................................................... 179 0 T63
.....................uuggcacuggaagaauucacU..................................................................... 7 1 T63
.....................uugAcacuggaagaauucaca..................................................................... 1 1 T63
.....................uuggcacuggaagaauucaca..................................................................... 141 0 T63
.....................uuggcacuggaUgaauucaca..................................................................... 1 1 T63
.....................uuggcacuggaagaauucacC..................................................................... 1 1 T63
.....................uuggcacuggaaAaauucacag.................................................................... 1 1 T63
.....................uuggcacuggaagaauucacag.................................................................... 34 0 T63
.....................uuggcacuggaagaauucacaU.................................................................... 3 1 T63
.....................uuggcacuggaagaauucacaA.................................................................... 1 1 T63
.....................uuggcacuggaagaauucacaga................................................................... 6 0 T63
.....................uuggcacuggaagaauucacagauU................................................................. 1 1 T63
......................uggcacuggaagaauucac...................................................................... 5 0 T63
.......................ggcacuggaagaauucac...................................................................... 12 0 T63
.......................ggcacuggaagaauucaA...................................................................... 1 1 T63
.......................ggcacuggaagaauucaca..................................................................... 4 0 T63
.......................ggcacuggaagaauucacag.................................................................... 2 0 T63
.......................ggcacuggaagaauucacaga................................................................... 1 0 T63
........................gcacuggaagaauucacU..................................................................... 1 1 T63
.........................cacuggaagaauucacaC.................................................................... 1 1 T63
.........................cacuggaagaauucacaga................................................................... 1 0 T63
............................................ugcgauaagaaacacucguggu............................................. 1 0 T63
............................................ugcgauaagaaacacucgugguuc........................................... 1 0 T63
............................................ugcgauaagaaacacucgugguucg.......................................... 1 0 T63
...........................................................ucgugguucgucuggugcca................................ 5 0 T63
...........................................................ucgugguucgucuggugccaa............................... 1 0 T63
...........................................................ucgugguucgucuggugccaaa.............................. 3 0 T63
...........................................................ucgugguucgucuggugccaaaU............................. 3 1 T63
.............................................................gugguucgucuggugcca................................ 1 0 T63
.............................................................gugguucgucuggugccaa............................... 1 0 T63
.............................................................gugguucgucuggugccaGa.............................. 1 1 T63
.............................................................gugguucgucuggugccaaa.............................. 14 0 T63
.............................................................gugguucgucuggugccaaaU............................. 1 1 T63
.............................................................gugguucgucuggugccaaGg............................. 18 1 T63
.............................................................gugguucgucuggugccaaag............................. 90 0 T63
.............................................................gugguucgucuggugccaaaC............................. 1 1 T63
.............................................................gugguucgucuggugccaaagU............................ 3 1 T63
.............................................................gugguucgucuggugccaaaga............................ 121 0 T63
.............................................................gugguucgucugguCccaaaga............................ 3 1 T63
.............................................................gugguucgucuggugccaaagC............................ 1 1 T63
.............................................................gugguucgucuggugccaGaga............................ 1 1 T63
```

```
                    Mature                          Star
acaauuggccuuaccuuaucuuggcacuggaagaauucacagaugcgauaagaaacacucgugguucgucuggugccaaagauaaacgcaagguauacacucugaacuuu
.............................................................gugguucgucuggugccaaagG............................   1     1     T63
.............................................................gugguucgucuggugccaaagaA...........................   1     1     T63
.............................................................gugguucgucuggugccaaagaCa..........................   1     1     T63
.............................................................gugguucgucuggugccaaagaua..........................   3     0     T63
..............................................................ugguucgucuggugccaU...............................   1     1     T63
..............................................................ugguucgucuggugccaa...............................   3     0     T63
..............................................................ugguucgucuggugccaaa..............................   10    0     T63
..............................................................ugguucgucuggugccaaU..............................   2     1     T63
..............................................................ugguucguUuggugccaaa..............................   1     1     T63
..............................................................ugguucgucuggugccaaGg.............................   6     1     T63
..............................................................ugguucgucuggugccaaag.............................   27    0     T63
..............................................................ugguucgucuggugccaaagU............................   1     1     T63
..............................................................ugguucgucuggugccaaaga............................   30    0     T63
..............................................................ugguucgucuggugccaaagau...........................   7     0     T63
..............................................................ugguucgucuggugccaaagaA...........................   1     1     T63
..............................................................ugguucgucuggugccaaagaAa..........................   1     1     T63
..............................................................ugguucgucuggugccaaagauC..........................   1     1     T63
..............................................................ugguucgucuggugccaaagauU..........................   33    1     T63
..............................................................ugguucgucuggugccaaagauG..........................   1     1     T63
..............................................................ugguucgucuggugccaaagaua..........................   14    0     T63
..............................................................ugguucgucuggugccaaagaCa..........................   1     1     T63

.................aGccuuggcacuggaagaauucac......................................................................   1     1     MOL
..................uUcuuggcacuggaagaauuc........................................................................   1     1     MOL
..................uUcuuggcacuggaagaauuca.......................................................................   2     1     MOL
..................uUcuuggcacuggaagaauucac......................................................................   9     1     MOL
..................uUcuuggcacuggaagaauucaca.....................................................................   9     1     MOL
..................uUcuuggcacuggaagaauucacag....................................................................   1     1     MOL
...................ccuuggcacuggaagaauu.........................................................................   3     0     MOL
...................ccuuggcacuggaagaauuc........................................................................   63    0     MOL
...................ccuuggcacuggaagaauuA........................................................................   2     1     MOL
...................ccuuggcacuggaagaauuca.......................................................................   73    0     MOL
...................Ucuuggcacuggaagaauuca.......................................................................   1     1     MOL
...................Ucuuggcacuggaagaauucac......................................................................   2     1     MOL
...................ccuuggUacuggaagaauucac......................................................................   1     1     MOL
...................ccuuggcacuggaagaauucac......................................................................   162   0     MOL
...................cUuuggcacuggaagaauucac......................................................................   1     1     MOL
...................ccuugUcacuggaagaauucac......................................................................   1     1     MOL
...................ccuuggcacuggaagaauucaA......................................................................   4     1     MOL
...................ccuuggcacuggaagaauucaca.....................................................................   41    0     MOL
...................ccuuggcacuggaagaauucacU.....................................................................   8     1     MOL
...................Ucuuggcacuggaagaauucaca.....................................................................   6     1     MOL
...................ccuuggcacuggaagaauucacaU....................................................................   1     1     MOL
...................ccuuggcacuggaagaauucacaA....................................................................   1     1     MOL
...................ccuuggcacuggaagaauucacag....................................................................   1     0     MOL
...................Ucuuggcacuggaagaauucacaga...................................................................   2     1     MOL
...................ccuuggcacuggaagaauucacaga...................................................................   1     0     MOL
....................cuuggcacuggaagaauu.........................................................................   43    0     MOL
....................cuuggcacuggaagaauC.........................................................................   1     1     MOL
....................cuuggcacuggaaCaauuc........................................................................   1     1     MOL
....................cuuggcacuAgaagaauuc........................................................................   1     1     MOL
....................cuuggcacuggaagaauuA........................................................................   65    1     MOL
....................cuAggcacuggaagaauuc........................................................................   1     1     MOL
....................cuuUgcacuggaagaauuc........................................................................   1     1     MOL
....................cuuggcacuggaagaaAuc........................................................................   3     1     MOL
....................cuuggcacuggaaAaauuc........................................................................   2     1     MOL
....................cuuggcacCggaagaauuc........................................................................   1     1     MOL
....................cuCggcacuggaagaauuc........................................................................   2     1     MOL
....................cuuggUacuggaagaauuc........................................................................   3     1     MOL
....................cuuggcacuCgaagaauuc........................................................................   1     1     MOL
....................cuuggcacuggaagCauuc........................................................................   1     1     MOL
....................cuuggcacuggaagaauuc........................................................................   2825  0     MOL
....................cuuggcacuggaagGauuc........................................................................   2     1     MOL
....................cuuggcacuggCagaauuc........................................................................   1     1     MOL
....................cuuggcaUuggaagaauuc........................................................................   3     1     MOL
....................Auuggcacuggaagaauuc........................................................................   2     1     MOL
....................Uuuggcacuggaagaauuc........................................................................   2     1     MOL
....................cuuggcacuggaagaauuU........................................................................   8     1     MOL
....................cuuggcacuggaagUauuc........................................................................   5     1     MOL
....................cuuggcacuggGagaauuc........................................................................   1     1     MOL
```

```
                    Mature                                   Star
acaauuggccuuaccuuauccuuggcacuggaagaauucacagaugcgauaagaaacacucgugguucgucuggugccaaagauaaacgcaagguauacacucugaacuuu
....................cuuggcacuggaagaauAc........................................................................    4          1          MOL
....................cuugAcacuggaagaauuc........................................................................    1          1          MOL
....................cuuggcacuggUagaauuc........................................................................    4          1          MOL
....................cuuggcacuggaagGauuca.......................................................................    4          1          MOL
....................cuuggcacugUaagaauuca.......................................................................    4          1          MOL
....................cuuggcacCggaagaauuca.......................................................................    1          1          MOL
....................cuuggcacuggUagaauuca.......................................................................    6          1          MOL
....................cuuUgcacuggaagaauuca.......................................................................    1          1          MOL
....................cuuggcacuggaagaauucU.......................................................................    23         1          MOL
....................cuuggcacuggaagaauuAa.......................................................................    45         1          MOL
....................cCuggcacuggaagaauuca.......................................................................    2          1          MOL
....................cuuggcacuggaagaauCca.......................................................................    2          1          MOL
....................cuuggcacuggGagaauuca.......................................................................    4          1          MOL
....................cuuggGacuggaagaauuca.......................................................................    3          1          MOL
....................cuuggcacuggaaCaauuca.......................................................................    2          1          MOL
....................cuuggcacuCgaagaauuca.......................................................................    3          1          MOL
....................cuuggUacuggaagaauuca.......................................................................    11         1          MOL
....................cuuggcacuggaaUaauuca.......................................................................    1          1          MOL
....................cuuggcacAggaagaauuca.......................................................................    6          1          MOL
....................cuuggcacuggaagaGuuca.......................................................................    2          1          MOL
....................cuuggcacuggaagaUuuca.......................................................................    1          1          MOL
....................cuCggcacuggaagaauuca.......................................................................    2          1          MOL
....................cuuggcacuggaagaauAca.......................................................................    2          1          MOL
....................cAuggcacuggaagaauuca.......................................................................    2          1          MOL
....................cuugUcacuggaagaauuca.......................................................................    1          1          MOL
....................cuuggcacugAaagaauuca.......................................................................    2          1          MOL
....................cuuggcaUuggaagaauuca.......................................................................    2          1          MOL
....................cuuggcacuggaagaauGca.......................................................................    1          1          MOL
....................cuuggcacuggaagaauuGa.......................................................................    1          1          MOL
....................Uuuggcacuggaagaauuca.......................................................................    5          1          MOL
....................cuugAcacuggaagaauuca.......................................................................    1          1          MOL
....................cuuggcacuggaagaaAuca.......................................................................    10         1          MOL
....................cuuggcacuggaagaaCuca.......................................................................    1          1          MOL
....................cuuggcacuggaagUauuca.......................................................................    21         1          MOL
....................cuuggcacuggaaAaauuca.......................................................................    1          1          MOL
....................cuuggcacuggaUgaauuca.......................................................................    2          1          MOL
....................cuuggcacuggaagaauuca.......................................................................    7906       0          MOL
....................cuAggcacuggaagaauuca.......................................................................    1          1          MOL
....................cuuggcacuggaagaauucG.......................................................................    7          1          MOL
....................cuuggcacuggaGgaauuca.......................................................................    2          1          MOL
....................cuugUcacuggaagaauucac......................................................................    2          1          MOL
....................cuuggcacuggaagUauucac......................................................................    47         1          MOL
....................cCuggcacuggaagaauucac......................................................................    5          1          MOL
....................cuuggcacugCaagaauucac......................................................................    4          1          MOL
....................cuuggcacAggaagaauucac......................................................................    16         1          MOL
....................cuuggcacuCgaagaauucac......................................................................    7          1          MOL
....................cuuggcacuggaagaaGucac......................................................................    1          1          MOL
....................cuuggcacuggaagaUuucac......................................................................    5          1          MOL
....................cuuAgcacuggaagaauucac......................................................................    1          1          MOL
....................cuuggcacugUaagaauucac......................................................................    6          1          MOL
....................cuugCcacuggaagaauucac......................................................................    1          1          MOL
....................cuuUgcacuggaagaauucac......................................................................    3          1          MOL
....................cuuggcacuggaagGauucac......................................................................    11         1          MOL
....................cuuggcacuAgaagaauucac......................................................................    3          1          MOL
....................cGuggcacuggaagaauucac......................................................................    1          1          MOL
....................cuCggcacuggaagaauucac......................................................................    3          1          MOL
....................cuuggAacuggaagaauucac......................................................................    6          1          MOL
....................cuuggcaUuggaagaauucac......................................................................    9          1          MOL
....................cuuggcacuggCagaauucac......................................................................    7          1          MOL
....................cuuggUacuggaagaauucac......................................................................    24         1          MOL
....................cuuggcacuggaaAaauucac......................................................................    10         1          MOL
....................cuuggcacCggaagaauucac......................................................................    1          1          MOL
....................cuuggcacuggaagaauGcac......................................................................    1          1          MOL
....................cuuggcacuggaagaauAcac......................................................................    4          1          MOL
....................cuuggcacuggaaCaauucac......................................................................    4          1          MOL
....................cuuggcacuggaagaaAucac......................................................................    13         1          MOL
....................cuuggcacuggaaUaauucac......................................................................    6          1          MOL
....................cuuggcacuggaagaauucac......................................................................    22079      0          MOL
....................Auuggcacuggaagaauucac......................................................................    3          1          MOL
....................cuuggcUcuggaagaauucac......................................................................    1          1          MOL
```

```
                    Mature                      Star
acaauuggccuuaccuuauccuuggcacuggaagaauucacagaugcgauaagaaacacucgugguucgucuggugccaaagauaaacgcaagguauacacucugaacuuu
....................cuuggcacuggaagaaCucac......................................................................    5      1   MOL
....................cuuggcacuggaagaauuUac......................................................................    12     1   MOL
....................cuuggcacuggaUgaauucac......................................................................    1      1   MOL
....................cuAggcacuggaagaauucac......................................................................    6      1   MOL
....................cuuggcacuggaagaauucaG......................................................................    2      1   MOL
....................cuuggcacuggaagaauCcac......................................................................    9      1   MOL
....................cuuggcacuggaagaauucaU......................................................................    85     1   MOL
....................cuuggcacuggUagaauucac......................................................................    25     1   MOL
....................cuuggcaAuggaagaauucac......................................................................    3      1   MOL
....................cuugAcacuggaagaauucac......................................................................    4      1   MOL
....................cuuggcacugAaagaauucac......................................................................    10     1   MOL
....................cuuggcacuggaagaauuAac......................................................................    5      1   MOL
....................Uuuggcacuggaagaauucac......................................................................    9      1   MOL
....................cuuggcacuggaagaauucaA......................................................................    640    1   MOL
....................cuuggGacuggaagaauucac......................................................................    2      1   MOL
....................cuuggcacuggaagaauucGc......................................................................    1      1   MOL
....................Guuggcacuggaagaauucac......................................................................    1      1   MOL
....................cuuggcacuggGagaauucac......................................................................    21     1   MOL
....................cuuggcGcuggaagaauucac......................................................................    1      1   MOL
....................cuuggcacuggaagCauucac......................................................................    6      1   MOL
....................cuuggcacuggaagaauuGac......................................................................    4      1   MOL
....................cuuggGacuggaagaauucaca.....................................................................    1      1   MOL
....................Guuggcacuggaagaauucaca.....................................................................    1      1   MOL
....................cuugAcacuggaagaauucaca.....................................................................    9      1   MOL
....................cuuggcacugCaagaauucaca.....................................................................    6      1   MOL
....................cuuggcacuggaAaauucaca......................................................................    2      1   MOL
....................cuuggcacuCgaagaauucaca.....................................................................    8      1   MOL
....................cuuggcacuggaagCauucaca.....................................................................    6      1   MOL
....................cuuggcacuggaagaauucacC.....................................................................    4      1   MOL
....................cuuUgcacuggaagaauucaca.....................................................................    2      1   MOL
....................cuuggcaUuggaagaauucaca.....................................................................    3      1   MOL
....................cuuggcacuggUagaauucaca.....................................................................    25     1   MOL
....................cuuggcacuggaagaauuGaca.....................................................................    1      1   MOL
....................cuuggcacuggaagaaCucaca.....................................................................    3      1   MOL
....................cuuggcacCggaagaauucaca.....................................................................    2      1   MOL
....................cuuggcacuggaagGauucaca.....................................................................    20     1   MOL
....................cuugCcacuggaagaauucaca.....................................................................    2      1   MOL
....................cuAggcacuggaagaauucaca.....................................................................    4      1   MOL
....................cuuggcacuggaagaauGcaca.....................................................................    4      1   MOL
....................cuuggcacuggaagaauCcaca.....................................................................    5      1   MOL
....................cuuggcacuggaagaaAucaca.....................................................................    11     1   MOL
....................cuuggcacuggaagaauucaca.....................................................................    21370  0   MOL
....................cuuggcacuggaagaauucaAa.....................................................................    26     1   MOL
....................cuuCgcacuggaagaauucaca.....................................................................    1      1   MOL
....................cuuggAacuggaagaauucaca.....................................................................    2      1   MOL
....................cuuggcacuggaCgaauucaca.....................................................................    1      1   MOL
....................cuGggcacuggaagaauucaca.....................................................................    1      1   MOL
....................cuuggcacuggaagaauucacG.....................................................................    58     1   MOL
....................cuuggcaGuggaagaauucaca.....................................................................    1      1   MOL
....................cuuggcacuggaagUauucaca.....................................................................    35     1   MOL
....................cuuggcacAggaagaauucaca.....................................................................    14     1   MOL
....................cuuggUacuggaagaauucaca.....................................................................    25     1   MOL
....................cuuggcacugAaagaauucaca.....................................................................    4      1   MOL
....................cuuggcacuggaagaaGucaca.....................................................................    2      1   MOL
....................Uuuggcacuggaagaauucaca.....................................................................    7      1   MOL
....................cuuggcacuggaagaauucaUa.....................................................................    11     1   MOL
....................cuuggcacuggaagaauucacU.....................................................................    332    1   MOL
....................cCuggcacuggaagaauucaca.....................................................................    2      1   MOL
....................cuuggcacuggaagaauuAaca.....................................................................    3      1   MOL
....................cuuggcacuAgaagaauucaca.....................................................................    4      1   MOL
....................cuuAgcacuggaagaauucaca.....................................................................    1      1   MOL
....................cuuggcacuggGagaauucaca.....................................................................    14     1   MOL
....................cuuggcacuggaUgaauucaca.....................................................................    4      1   MOL
....................cuuggcacugUaagaauucaca.....................................................................    8      1   MOL
....................cuuggcacuggaagaauAcaca.....................................................................    6      1   MOL
....................cuugUcacuggaagaauucaca.....................................................................    1      1   MOL
....................cuuggcacuggaagaauucaGa.....................................................................    1      1   MOL
....................cuuggcacuggCagaauucaca.....................................................................    4      1   MOL
....................cuuggcacuggaaCaauucaca.....................................................................    3      1   MOL
....................cuuggcacuggaagaauucUca.....................................................................    2      1   MOL
```

Mature Star

```
acaauuggccuuaccuuauccuuggcacuggaagaauucacagaugcgauaaagaaacacucgugguucgucuggugccaaagauaaacgcaagguauacacucugaacuuu
....................cuuggcacuggaagaauuUaca......................................................................  6     1  MOL
....................cGuggcacuggaagaauucaca......................................................................  4     1  MOL
....................cuuggcacuggaagaUuucaca......................................................................  7     1  MOL
....................Auuggcacuggaagaauucaca......................................................................  1     1  MOL
....................cuuggcacuggaagaauGcacag.....................................................................  2     1  MOL
....................cuuggcacuggaagaauuUacag.....................................................................  3     1  MOL
....................cuuggcacuggaagaauucUcag.....................................................................  1     1  MOL
....................Uuuggcacuggaagaauucacag.....................................................................  1     1  MOL
....................cuAggcacuggaagaauucacag.....................................................................  1     1  MOL
....................cuuggcacuggaagGauucacag.....................................................................  3     1  MOL
....................cuuggcaUuggaagaauucacag.....................................................................  2     1  MOL
....................cuuggcacuggUagaauucacag.....................................................................  4     1  MOL
....................cuuggcacuggaagaauuGacag.....................................................................  1     1  MOL
....................cuuggcacuggaagaGuucacag.....................................................................  1     1  MOL
....................cuuggcacuggaagaauucacUg.....................................................................  3     1  MOL
....................cuuggcacuggaagaauucacaC.....................................................................  171   1  MOL
....................cuCggcacuggaagaauucacag.....................................................................  1     1  MOL
....................cuugCcacuggaagaauucacag.....................................................................  1     1  MOL
....................cuuggcacuggaagaauucacag.....................................................................  4773  0  MOL
....................cuugAcacuggaagaauucacag.....................................................................  1     1  MOL
....................cuuggcacuggaagaauucacaA.....................................................................  395   1  MOL
....................cuuggcacugUaagaauucacag.....................................................................  1     1  MOL
....................cuuggcacuggaagaaCucacag.....................................................................  1     1  MOL
....................cuuggcacuggaagUauucacag.....................................................................  10    1  MOL
....................cuuggUacuggaagaauucacag.....................................................................  2     1  MOL
....................cuuggcacuggaUgaauucacag.....................................................................  2     1  MOL
....................cuuggcacuggaagaauucacaU.....................................................................  262   1  MOL
....................cuuggcacugAaagaauucacag.....................................................................  1     1  MOL
....................cuuggcacuggaagaauucGcag.....................................................................  1     1  MOL
....................cuuggGacuggaagaauucacag.....................................................................  1     1  MOL
....................cuuggcacuggaaUaauucacag.....................................................................  1     1  MOL
....................cuuggcacuggGagaauucacag.....................................................................  3     1  MOL
....................cuuggcacuggaGgaauucacag.....................................................................  3     1  MOL
....................cuuggcacuggaagaauucacGg.....................................................................  23    1  MOL
....................cuuggcacuggaagaauucacCg.....................................................................  2     1  MOL
....................cuuggcacuCgaagaauucacag.....................................................................  1     1  MOL
....................cuuggAacuggaagaauucacag.....................................................................  2     1  MOL
....................cuuggcacuggaagaauucaUag.....................................................................  3     1  MOL
....................cuuggcacAggaagaauucacag.....................................................................  2     1  MOL
....................cuuggcacuggaagaauucacaCa....................................................................  7     1  MOL
....................cuuUgcacuggaagaauucacaga....................................................................  2     1  MOL
....................cuuggcacuggaagaauuAacaga....................................................................  1     1  MOL
....................cuuggcacuggaagaauuGacaga....................................................................  2     1  MOL
....................cuuggcacuggaagaauucacagG....................................................................  2     1  MOL
....................cuuggcacuggaagaauucacaga....................................................................  1982  0  MOL
....................cuuggcacuggaagaauucacagU....................................................................  85    1  MOL
....................cuuggcacuggaagaauucacaUa....................................................................  6     1  MOL
....................cuuggcacuggaagaauucacaAa....................................................................  12    1  MOL
....................cuuggcacuCgaagaauucacaga....................................................................  1     1  MOL
....................cuuggcacAggaagaauucacaga....................................................................  2     1  MOL
....................cuuggcacuggaagaauucacGga....................................................................  2     1  MOL
....................cuuggcacuggaagaauucacUga....................................................................  3     1  MOL
....................cuuggcacuggCagaauucacaga....................................................................  1     1  MOL
....................cuugAcacuggaagaauucacaga....................................................................  1     1  MOL
....................cuuggcacuggaagaaCucacaga....................................................................  1     1  MOL
....................cuuggcacuggaagUauucacaga....................................................................  3     1  MOL
....................cuAggcacuggaagaauucacaga....................................................................  2     1  MOL
....................cuuggcacuggaagaauucacagC....................................................................  4     1  MOL
....................cuuggcacuggaagaauucaUaga....................................................................  2     1  MOL
....................cuuggcaUuggaagaauucacaga....................................................................  1     1  MOL
....................Uuuggcacuggaagaauucacaga....................................................................  2     1  MOL
....................cuuggUacuggaagaauucacaga....................................................................  1     1  MOL
....................cuuggcacuggaagaauucacagUu...................................................................  11    1  MOL
....................cuuggcacuggaagaauucacagaA...................................................................  105   1  MOL
....................cuuggcacugAaagaauucacagau...................................................................  1     1  MOL
....................cuuggcacuggaagaauucacaCau...................................................................  1     1  MOL
....................cuuggcacuggaagaauucacaAau...................................................................  1     1  MOL
....................cuuggcacuggaagaauucacGgau...................................................................  1     1  MOL
....................cuuggcacuggaagaauucacaUau...................................................................  2     1  MOL
....................cuuAgcacuggaagaauucacagau...................................................................  1     1  MOL
```

Mature Star

```
acaauuggccuuaccuuauccuuggcacuggaagaauucacagaugcgauaaagaaacacucgugguucgucuggugccaaagauaaacgcaagguauacacucugaacuuu
....................cuuggcacuggaagUauucacagau...................................................................	1	1	MOL
....................cuuggcacuCgaagaauucacagau...................................................................	1	1	MOL
....................cuuggcacuggaagaauucacagaC...................................................................	4	1	MOL
....................cuuggcacuggaagaauucacagCu...................................................................	2	1	MOL
....................cuuggcacuggaagaauucacagau...................................................................	194	0	MOL
....................cuuggcacuggaagaauucacagauC..................................................................	1	1	MOL
....................cuuggcacuggaagaauucacagauA..................................................................	4	1	MOL
....................cuuggcacuggaagaauucacagauU..................................................................	26	1	MOL
....................cuuggcacuggaagaauucacagaug..................................................................	1	0	MOL
....................cuuggcacuggaagaauucacagaugc.................................................................	1	0	MOL
....................cuuggcacuggaagaauucacagaugA.................................................................	1	1	MOL
.....................uuggcacuggaagaauuc.........................................................................	8	0	MOL
.....................uuggcacuggaagaauuca........................................................................	29	0	MOL
.....................uuggcacuggaagaauucac.......................................................................	138	0	MOL
.....................uuggcacuggaagaauucaU.......................................................................	2	1	MOL
.....................Auggcacuggaagaauucaca......................................................................	2	1	MOL
.....................uuggcacugCaagaauucaca......................................................................	1	1	MOL
.....................uuggcacuggaagaauucaca......................................................................	192	0	MOL
.....................uuggcacuggaagaauucacU......................................................................	2	1	MOL
.....................uuggcacuggaagaauucacaA.....................................................................	2	1	MOL
.....................uuggcacuggaagaauucacaU.....................................................................	7	1	MOL
.....................uuggcacuggaagaauucaAag.....................................................................	1	1	MOL
.....................uuggcacuggaagaauucacaC.....................................................................	2	1	MOL
.....................uuggcacuggaagaauucacag.....................................................................	83	0	MOL
.....................uuggcacuggaagaauucacagC....................................................................	1	1	MOL
.....................uuggcacuggaagUauucacaga....................................................................	1	1	MOL
.....................uuggcacuggaagaauucacaga....................................................................	65	0	MOL
.....................uuggcacuggaagaauucacagaA...................................................................	5	1	MOL
.....................uuggcacuggaagaauucacagau...................................................................	3	0	MOL
......................uggcacuggaagaauuca........................................................................	2	0	MOL
......................uggcacuggaagaauucac.......................................................................	3	0	MOL
......................uggcacuggaagaauucacag.....................................................................	1	0	MOL
......................uggcacuggaagaauucacagau...................................................................	1	0	MOL
.......................ggcacuggaagaauucac.......................................................................	13	0	MOL
.......................ggcacuggaagaauucaca......................................................................	13	0	MOL
.......................ggcacuggaagaauucacag.....................................................................	4	0	MOL
.......................ggcacuggaagaauucacaga....................................................................	1	0	MOL
............................................ugcgauaaagaaacacucguggu.............................................	1	0	MOL
............................................ugcgauaaagaaacacucgugguu............................................	2	0	MOL
............................................ugcgauaaagaaacacucgugguuc...........................................	1	0	MOL
............................................ugcgauaaagaaacacucgugguucguc........................................	2	0	MOL
.............................................gcgauaaagaaacacucguggu.............................................	1	0	MOL
..........................................................acucgugguucgucuggugccaaaga............................	1	0	MOL
...........................................................cucgugguucgucuggugcca................................	1	0	MOL
...........................................................cucgugguucgucuggugccaaag.............................	1	0	MOL
............................................................ucgugguucgucuggugcc.................................	8	0	MOL
............................................................ucgugguucgucuggugccU................................	3	1	MOL
............................................................ucgugguucgucuggugcca................................	67	0	MOL
............................................................ucgugguucgucuggugccaa...............................	5	0	MOL
............................................................ucgugguucgucuggugccaaG..............................	1	1	MOL
............................................................ucgugguucgucuggugccaaU..............................	4	1	MOL
............................................................ucgugguucgucuggugccaaC..............................	1	1	MOL
............................................................ucgugguucgucuggugccaaa..............................	9	0	MOL
............................................................ucgugguucgucuggugccaaag.............................	3	0	MOL
............................................................ucgugguucgucuggugccaaaga............................	11	0	MOL
............................................................ucgugguucgucuggugccaaagauU..........................	1	1	MOL
..............................................................gugguucgucuggugccaa...............................	4	0	MOL
..............................................................gugguucgucuggugccaaa..............................	18	0	MOL
..............................................................gugguucgucuggugUcaaa..............................	1	1	MOL
..............................................................gugguucgucuggugccaaG..............................	1	1	MOL
..............................................................gugguucgucuggugccaaU..............................	1	1	MOL
..............................................................gugguucgucuggugccaaag.............................	159	0	MOL
..............................................................gugguucguGuggugccaaag.............................	1	1	MOL
..............................................................gugguucguUuggugccaaag.............................	1	1	MOL
..............................................................gugguucgucuggAgccaaag.............................	1	1	MOL
..............................................................gugguucgucuggugcUaaag.............................	1	1	MOL
..............................................................gugguucgucuggugccaaGg.............................	1	1	MOL
..............................................................gugguucgucugguAccaaaga............................	2	1	MOL
..............................................................gugguucgucuggugccaaagU............................	6	1	MOL
..............................................................gugguucgucuggugccaaaga............................	237	0	MOL
```

```
                    Mature                                   Star
acaauuggccuuaccuuauccuuggcacuggaagaauucacagaugcgauaagaaacacucgugguucgucuggugccaaagauaaacgcaagguauacacucugaacuuu
.............................................................gugUuucgucuggugccaaaga............................   1    1    MOL
.............................................................gugguucgucuggugccaaaAa............................   1    1    MOL
.............................................................gugguucgucuggugccaaagaA...........................   1    1    MOL
.............................................................gugguucgucuggugccaaagaua..........................   1    0    MOL
.............................................................gugguucgucuggugccaaagaCa..........................   3    1    MOL
.............................................................gugguucgucuggugccaaagaAa..........................   1    1    MOL
..............................................................ugguucgucuggugccaaa..............................   8    0    MOL
..............................................................ugguucgucuggugccaaaA.............................   1    1    MOL
..............................................................ugguucgucuggugccaaag.............................   72   0    MOL
..............................................................ugguucgucuggugccaaGg.............................   2    1    MOL
..............................................................ugguucgucuggugccaaaC.............................   1    1    MOL
..............................................................ugguucgucuggugccaaaU.............................   1    1    MOL
..............................................................Agguucgucuggugccaaaga............................   3    1    MOL
..............................................................ugguucgucuggugccaaGga............................   2    1    MOL
..............................................................ugguucgucCggugccaaaga............................   1    1    MOL
..............................................................ugAuucgucuggugccaaaga............................   1    1    MOL
..............................................................ugguucgucuggugccaaagU............................   7    1    MOL
..............................................................Ggguucgucuggugccaaaga............................   1    1    MOL
..............................................................ugguucgucuggugccaaaga............................   244  0    MOL
..............................................................ugguucgucuggugccaaagG............................   1    1    MOL
..............................................................ugguucgucuggugccaaagC............................   1    1    MOL
..............................................................ugguucguAuggugccaaaga............................   2    1    MOL
..............................................................ugguucgucuggugccaaagaA...........................   8    1    MOL
..............................................................ugguucgucuggugccaaagaG...........................   1    1    MOL
..............................................................ugguucgucuggugccaaagaC...........................   4    1    MOL
..............................................................ugguucgucuggugccaaagau...........................   27   0    MOL
..............................................................ugguucgucuggugccaaagauG..........................   5    1    MOL
..............................................................ugguucgucuggugccaaagaCa..........................   5    1    MOL
..............................................................ugguucgucuggugccaaagauC..........................   1    1    MOL
..............................................................ugguucgucuggugccaaagaua..........................   27   0    MOL
..............................................................ugguucgucuggugccaaagauU..........................   45   1    MOL
..............................................................ugguucgucuggugccaaagaAa..........................   3    1    MOL
..............................................................ugguucgucuggugccaaagauaU.........................   1    1    MOL

..................uUcuuggcacuggaagaauuca.......................................................................   1    1    T6P
..................uAcuuggcacuggaagaauuca.......................................................................   1    1    T6P
..................uUcuuggcacuggaagaauucac......................................................................   5    1    T6P
..................uUcuuggcacuggaagaauucaca.....................................................................   1    1    T6P
..................uUcuuggcacuggaagaauucacaga...................................................................   1    1    T6P
...................ccuuggcacuggaagaauu.........................................................................   1    0    T6P
...................ccuuggcacuggaagaauuc........................................................................   27   0    T6P
...................Ucuuggcacuggaagaauuc........................................................................   2    1    T6P
...................ccuuggcacuggaagaauuca.......................................................................   50   0    T6P
...................Acuuggcacuggaagaauuca.......................................................................   1    1    T6P
...................Ucuuggcacuggaagaauuca.......................................................................   3    1    T6P
...................Ucuuggcacuggaagaauucac......................................................................   3    1    T6P
...................ccuuggGacuggaagaauucac......................................................................   1    1    T6P
...................ccuuggcacuggaagaauucac......................................................................   34   0    T6P
...................ccuuggcacuggaagaauucaA......................................................................   1    1    T6P
...................ccuuggcacuggaagaauucacU.....................................................................   1    1    T6P
...................ccuuggcacuggaagaauucaca.....................................................................   8    0    T6P
...................Ucuuggcacuggaagaauucaca.....................................................................   8    1    T6P
...................ccuuggcacuggaagaauucacaA....................................................................   1    1    T6P
....................cuuggcacuggaagaauu.........................................................................   7    0    T6P
....................cuuggcacuggaagaauA.........................................................................   1    1    T6P
....................cuuggcacuggaagGauuc........................................................................   1    1    T6P
....................cuuggcacugUaagaauuc........................................................................   2    1    T6P
....................cuuggcacuggaagaauAc........................................................................   8    1    T6P
....................cuuggcacuggaUgaauuc........................................................................   1    1    T6P
....................cuuggcacuggaagaauuc........................................................................   581  0    T6P
....................cuuggcacuggaagUauuc........................................................................   1    1    T6P
....................cuuggcacuggGagaauuc........................................................................   1    1    T6P
....................cCuggcacuggaagaauuc........................................................................   1    1    T6P
....................cuuggcacuggaagaauuU........................................................................   1    1    T6P
....................cuuggcacuggaaCaauuc........................................................................   3    1    T6P
....................cuuggcacuggaagaUuuc........................................................................   1    1    T6P
....................cuuggcacuggaagaauuA........................................................................   13   1    T6P
....................cuuggUacuggaagaauuc........................................................................   2    1    T6P
....................cuuggcacuggaagUauuca.......................................................................   2    1    T6P
....................cuuggcacuggaagaauucG.......................................................................   1    1    T6P
```

```
                    Mature                Star
acaauuggccuuaccuuauccuuggcacuggaagaauucacagaugcgauaagaaacacucgugguucgucuggugccaaagauaaacgcaagguauacacucugaacuuu
....................cuuggUacuggaagaauuca.......................................................................   1      1      T6P
....................cuuggcacuggaagaauucU.......................................................................   1      1      T6P
....................cuugUcacuggaagaauuca.......................................................................   2      1      T6P
....................cuuggcacuggaagaauuca.......................................................................   2155   0      T6P
....................cuuggcacuggaaUaauuca.......................................................................   4      1      T6P
....................Uuuggcacuggaagaauuca.......................................................................   1      1      T6P
....................cuuggcacugCaagaauuca.......................................................................   1      1      T6P
....................cuuggcacuggaagGauuca.......................................................................   1      1      T6P
....................cuuggcacuggaagaauCca.......................................................................   1      1      T6P
....................cuuggcacuggaUgaauuca.......................................................................   1      1      T6P
....................cuuggcacugUaagaauuca.......................................................................   3      1      T6P
....................cuugAcacuggaagaauuca.......................................................................   1      1      T6P
....................cuuggcaUuggaagaauuca.......................................................................   2      1      T6P
....................cuuggcGcuggaagaauuca.......................................................................   1      1      T6P
....................cuuggcacuCgaagaauuca.......................................................................   1      1      T6P
....................cuuggcacuggaaCaauuca.......................................................................   6      1      T6P
....................cuuggcacuUgaagaauuca.......................................................................   1      1      T6P
....................cuuggcacuggaaAaauuca.......................................................................   2      1      T6P
....................cuuggcacuggGagaauuca.......................................................................   1      1      T6P
....................cuuggcacuggaagaauuUa.......................................................................   1      1      T6P
....................cuuggcacuggaagaauuAa.......................................................................   5      1      T6P
....................cuuggcacuggCagaauucac......................................................................   1      1      T6P
....................cuuggcacuggaagUauucac......................................................................   3      1      T6P
....................cuuUgcacuggaagaauucac......................................................................   5      1      T6P
....................cuuggcacuggaagaauucac......................................................................   7317   0      T6P
....................cuuggcacuggaagaaCucac......................................................................   1      1      T6P
....................cuuggcacuggaaAaauucac......................................................................   2      1      T6P
....................cuuggcacuggaaCaauucac......................................................................   9      1      T6P
....................cuuggcacugCaagaauucac......................................................................   5      1      T6P
....................cuuggcacuCgaagaauucac......................................................................   1      1      T6P
....................cuuggcGcuggaagaauucac......................................................................   3      1      T6P
....................cuuggcacuggaagCauucac......................................................................   1      1      T6P
....................cuuggcacuUgaagaauucac......................................................................   2      1      T6P
....................cuuggcacuggaagaauucaU......................................................................   9      1      T6P
....................cuuggcacuggaagaUuucac......................................................................   2      1      T6P
....................cuuggcacuggaaUaauucac......................................................................   13     1      T6P
....................Guuggcacuggaagaauucac......................................................................   1      1      T6P
....................cuuggcacuggaagGauucac......................................................................   3      1      T6P
....................cuuggcaGuggaagaauucac......................................................................   1      1      T6P
....................cuuggcacuggaagaauucaG......................................................................   2      1      T6P
....................cuugAcacuggaagaauucac......................................................................   3      1      T6P
....................cuuggcacuggaagaauucaA......................................................................   169    1      T6P
....................cuuggcacuggaUgaauucac......................................................................   3      1      T6P
....................cuugUcacuggaagaauucac......................................................................   2      1      T6P
....................cuuggcacuggaagaGuucac......................................................................   1      1      T6P
....................cuuggcUcuggaagaauucac......................................................................   1      1      T6P
....................cuuggcacuggGagaauucac......................................................................   2      1      T6P
....................cuuggcCcuggaagaauucac......................................................................   4      1      T6P
....................cuuggcacuAgaagaauucac......................................................................   2      1      T6P
....................cAuggcacuggaagaauucac......................................................................   1      1      T6P
....................cuuggcacuggaagaauCcac......................................................................   2      1      T6P
....................cuuggcacugUaagaauucac......................................................................   4      1      T6P
....................Uuuggcacuggaagaauucac......................................................................   8      1      T6P
....................cuuggcacuggaagaauuUac......................................................................   12     1      T6P
....................cuuggUacuggaagaauucac......................................................................   8      1      T6P
....................cuuggcacuggaagaauAcaca.....................................................................   1      1      T6P
....................cuuggcacuggaaUaauucaca.....................................................................   4      1      T6P
....................cuugCcacuggaagaauucaca.....................................................................   1      1      T6P
....................cuuggcacuggaagaauucGca.....................................................................   2      1      T6P
....................cuuggcacuggaagCauucaca.....................................................................   2      1      T6P
....................cuuAgcacuggaagaauucaca.....................................................................   1      1      T6P
....................cuuggcacugAaagaauucaca.....................................................................   3      1      T6P
....................cuuggcacuggaagGauucaca.....................................................................   1      1      T6P
....................cuuggcacuggaagaauucaAa.....................................................................   6      1      T6P
....................cuuggcacuggaaCaauucaca.....................................................................   9      1      T6P
....................cuuggcacuggaagaauuGaca.....................................................................   1      1      T6P
....................cuuggcacuggaagUauucaca.....................................................................   3      1      T6P
....................cuuggcacuggaagaauucacU.....................................................................   106    1      T6P
....................cuCggcacuggaagaauucaca.....................................................................   1      1      T6P
....................cuuggcacuggCagaauucaca.....................................................................   2      1      T6P
```

Mature **Star**

```
acaauuggccuuaccuuauccuuggcacuggaagaauucacagaugcgauaaagaaacacucgugguucgucuggugccaaagauaaacgcaagguauacacucugaacuuu
....................cAuggcacuggaagaauucaca......................................................................   1     1   T6P
....................cuuggcacuggaagaauucacG.......................................................................   17    1   T6P
....................cuGggcacuggaagaauucaca......................................................................   1     1   T6P
....................cuuggcacuggaagaUuucaca......................................................................   1     1   T6P
....................Uuuggcacuggaagaauucaca......................................................................   2     1   T6P
....................cuuggcacuUgaagaauucaca......................................................................   2     1   T6P
....................cuuggcacuggaUgaauucaca......................................................................   2     1   T6P
....................cuuggcacugUaagaauucaca......................................................................   6     1   T6P
....................cuuggcacuggGagaauucaca......................................................................   2     1   T6P
....................cuuggUacuggaagaauucaca......................................................................   12    1   T6P
....................cuugAcacuggaagaauucaca......................................................................   1     1   T6P
....................cuuggcacuCgaagaauucaca......................................................................   2     1   T6P
....................cuuggcacCggaagaauucaca......................................................................   1     1   T6P
....................cuuggcacuggaagaauCcaca......................................................................   3     1   T6P
....................cuuggcacAggaagaauucaca......................................................................   1     1   T6P
....................cuuggcacuggaagaauucaca......................................................................   7564  0   T6P
....................cuuggcacuggaagaauucCca......................................................................   1     1   T6P
....................cuuggcacugCaagaauucaca......................................................................   7     1   T6P
....................cuuggcacuggaagaauuUaca......................................................................   2     1   T6P
....................cuuggcacuggaagaauucaUa......................................................................   5     1   T6P
....................cuuggcacuggaagaauucacC......................................................................   3     1   T6P
....................cuuggcGcuggaagaauucaca......................................................................   6     1   T6P
....................cuuCgcacuggaagaauucaca......................................................................   1     1   T6P
....................cuuUgcacuggaagaauucaca......................................................................   4     1   T6P
....................cuuggcacugUaagaauucacag.....................................................................   1     1   T6P
....................cuuggcacuggaagaauucacaC.....................................................................   55    1   T6P
....................cuuggcacuggGagaauucacag.....................................................................   1     1   T6P
....................cuuggcacugCaagaauucacag.....................................................................   1     1   T6P
....................cuuggcacuggaagaauucacaU.....................................................................   104   1   T6P
....................cuuggcacuggaagaauucacGg.....................................................................   4     1   T6P
....................cuuUgcacuggaagaauucacag.....................................................................   1     1   T6P
....................cuuggcacuggaagaauucacaA.....................................................................   160   1   T6P
....................cuugCcacuggaagaauucacag.....................................................................   1     1   T6P
....................cuuggcacugAaagaauucacag.....................................................................   1     1   T6P
....................cuuggcacuggaagaauucacag.....................................................................   1336  0   T6P
....................cuuggUacuggaagaauucacag.....................................................................   2     1   T6P
....................cuuggcacuggaaCaauucacag.....................................................................   2     1   T6P
....................cuuggcacuggaagaUuucacaga....................................................................   1     1   T6P
....................cuuggcacuggaagaauucacaUa....................................................................   2     1   T6P
....................cuuggcacuggaaCaauucacaga....................................................................   1     1   T6P
....................cuuggcacugUaagaauucacaga....................................................................   2     1   T6P
....................cuuggcacuggaagaauCcacaga....................................................................   4     1   T6P
....................cuuggcacuggaaAaauucacaga....................................................................   1     1   T6P
....................cuuggcacuggaagaauucacagC....................................................................   2     1   T6P
....................cuuggcacugCaagaauucacaga....................................................................   2     1   T6P
....................cuCggcacuggaagaauucacaga....................................................................   1     1   T6P
....................cuuggcacuggaagGauucacaga....................................................................   1     1   T6P
....................cuuggcacuggaagaauucacaga....................................................................   1165  0   T6P
....................cuuggcacuggaagaauuUacaga....................................................................   1     1   T6P
....................cuuggUacuggaagaauucacaga....................................................................   2     1   T6P
....................cuuggcacuggaagaauucacagU....................................................................   46    1   T6P
....................cuuggcacuggaagaauucacaAa....................................................................   5     1   T6P
....................cuuggcGcuggaagaauucacaga....................................................................   1     1   T6P
....................cuuggcacuggaagaauucacaCa....................................................................   4     1   T6P
....................cuuggcacuggaagaauucacaUau...................................................................   1     1   T6P
....................cuuggcacuggaagaauucacagau...................................................................   80    0   T6P
....................cuuggcacuggaagaauucacagUu...................................................................   9     1   T6P
....................cuuggcacuggaagaauucacagaC...................................................................   1     1   T6P
....................cuuggcacuggaagaauucacagaA...................................................................   71    1   T6P
....................cuuggcacuggaagaauucacagUug..................................................................   1     1   T6P
....................cuuggcacuggaagaauucacagauA..................................................................   1     1   T6P
....................cuuggcacuggaagaauucacagauU..................................................................   3     1   T6P
....................cuuggcacuggaagaauucacagaugc.................................................................   1     0   T6P
.....................uuggcacuggaagaauuc.........................................................................   1     0   T6P
.....................uuggcacuggaagaauuca........................................................................   33    0   T6P
.....................uuggcacuggaagaauucac.......................................................................   214   0   T6P
.....................uuggcacuggaagaauuGac.......................................................................   1     1   T6P
.....................uuggcacuggaaAauucac.......................................................................   1     1   T6P
.....................uuggcacugUaagaauucaca......................................................................   1     1   T6P
.....................uuggcacuggaagaauucaca......................................................................   264   0   T6P
```

```
                    Mature                                  Star
acaauuggccuuaccuuauccuuggcacuggaagaauucacagaugcgauaagaaacacucgugguucgucuggugccaaagauaaacgcaagguauacacucugaacuuu
.....................uugCcacuggaagaauucaca.....................................................................    1    1    T6P
.....................Cuggcacuggaagaauucaca.....................................................................    1    1    T6P
.....................uuggcacuggaagaauucacU.....................................................................    2    1    T6P
.....................uuggcacuggaagaaAucaca.....................................................................    1    1    T6P
.....................uuggcacugAaagaauucaca.....................................................................    1    1    T6P
.....................uuggcacugCaagaauucaca.....................................................................    1    1    T6P
.....................uuggcacuggaagaauucacaU....................................................................    3    1    T6P
.....................uuggcacuggaagaauucacag....................................................................    49   0    T6P
.....................uuggcacuggaagaauucacaA....................................................................    2    1    T6P
.....................uuggcacuggaagaauucacaga...................................................................    23   0    T6P
.....................uuggcacuggaagaauucacagU...................................................................    2    1    T6P
.....................uuggcacuggaagaauucacagau..................................................................    1    0    T6P
.....................uuggcacuggaagaauucacagaA..................................................................    3    1    T6P
......................uggcacuggaagaauuca.......................................................................    1    0    T6P
......................uggcacuggaagaauucac......................................................................    5    0    T6P
......................uggcacuggaagaauucaca.....................................................................    3    0    T6P
......................uggcacuggaagaauucacag....................................................................    4    0    T6P
......................uggcacuggaagaauucacaga...................................................................    2    0    T6P
.......................ggcacuggaagaauucac......................................................................    23   0    T6P
.......................ggcacuggaagaauucacU.....................................................................    1    1    T6P
.......................ggcacuggaagaauucaca.....................................................................    16   0    T6P
.......................ggcacuggaagaauucacag....................................................................    4    0    T6P
.......................ggcacuggaagaauucacaC....................................................................    1    1    T6P
.......................ggcacuggaagaauucacaU....................................................................    2    1    T6P
.......................ggcacuggaagaauucacagU...................................................................    1    1    T6P
.......................ggcacuggaagaauucacaga...................................................................    2    0    T6P
........................gcacuggaagaauucaca.....................................................................    2    0    T6P
.........................cacuggaagaauucacaA....................................................................    1    1    T6P
.........................cacuggaagaauucacaC....................................................................    1    1    T6P
............................................ugcgauaagaaacacucguggu.............................................    3    0    T6P
............................................ugcgauaagaaacacucgugguu............................................    2    0    T6P
............................................ugcgauaagaaacacucgugguucguc........................................    2    0    T6P
.........................................................acucgugguucgucuggugcca................................    1    0    T6P
...........................................................ucgugguucgucuggugcc.................................    1    0    T6P
...........................................................ucgugguucgucuggugccU................................    1    1    T6P
...........................................................ucgugguucgucuggugcca................................    35   0    T6P
...........................................................ucgugguucgucuggugccaa...............................    1    0    T6P
...........................................................ucgugguucgucuggugUcaa...............................    1    1    T6P
...........................................................ucgugguucgucuggugccaU...............................    1    1    T6P
...........................................................ucgugguucgucuggugccaaa..............................    5    0    T6P
...........................................................ucgugguucgucuggugccaaU..............................    2    1    T6P
...........................................................ucgugguucgucuggugccaaaga............................    1    0    T6P
...........................................................ucgugguucgucuggugccaaaAau...........................    1    1    T6P
............................................................cgugguucgucuggugcca................................    1    0    T6P
.............................................................gugguucgucuggugccaa...............................    2    0    T6P
.............................................................gugguucgucuggugccaaa..............................    5    0    T6P
.............................................................gugguucgucuggugccaaag.............................    25   0    T6P
.............................................................gugguucgucuggugccaaGg.............................    1    1    T6P
.............................................................gugguucgucuggugccaaagU............................    1    1    T6P
.............................................................gugguucgucuggugccaaaga............................    79   0    T6P
.............................................................gugguucgucugguAccaaaga............................    1    1    T6P
.............................................................gugguucgucuggugccaaaAa............................    1    1    T6P
.............................................................gugguucgucuggugccaaagaC...........................    1    1    T6P
.............................................................gugguucgucuggugccaaagUu...........................    1    1    T6P
.............................................................gugguucgucuggugccaaagauU..........................    1    1    T6P
..............................................................ugguucgucuggugccaaG..............................    1    1    T6P
..............................................................ugguucgucuggugccaaU..............................    2    1    T6P
..............................................................ugguucgucuggugccaaa..............................    23   0    T6P
..............................................................ugguucgucuggugccaaag.............................    41   0    T6P
..............................................................ugguucgucugguAccaaag.............................    1    1    T6P
..............................................................ugguucgucuggugccaaGg.............................    2    1    T6P
..............................................................ugguucgucuggugccaaaU.............................    1    1    T6P
..............................................................ugguucgucuggugccaaaga............................    42   0    T6P
..............................................................ugguucgucuggugccaaagU............................    2    1    T6P
..............................................................ugguucgucuggugccaaagau...........................    9    0    T6P
..............................................................ugguucgucuggugccaaagauU..........................    9    1    T6P
..............................................................ugAuucgucuggugccaaagaua..........................    1    1    T6P
..............................................................ugguucgucuggugccaaagaua..........................    2    0    T6P

...................ccuuggcacuggaagaauuc........................................................................    1    0    egg
```

```
                   Mature                  Star
acaauuggccuuaccuuauccuuggcacuggaagaauucacagaugcgauaagaaacacucgugguucgucuggugccaaagauaaacgcaagguauacacucugaacuuu
...................ccuuggcacuggaagaauucac......................................................................   2      0      egg
...................ccuugUcacuggaagaauucaca.....................................................................   1      1      egg
...................ccuGggcacuggaagaauucaca.....................................................................   1      1      egg
...................ccuuggcacuggaagaauucaca.....................................................................   11     0      egg
....................cuuUgcacuggaagaauuc........................................................................   1      1      egg
....................cuuggcacuggaagaauuc........................................................................   36     0      egg
....................cuuggcacCggaagaauuc........................................................................   2      1      egg
....................cuuUgcacuggaagaauuca.......................................................................   1      1      egg
....................cuuggcacuUgaagaauuca.......................................................................   4      1      egg
....................cuugUcacuggaagaauuca.......................................................................   4      1      egg
....................cuuggcacuggaagaauuca.......................................................................   57     0      egg
....................cuuggcacuggaagaGuuca.......................................................................   1      1      egg
....................Nuuggcacuggaagaauucac......................................................................   1      1      egg
....................cuuggcacuggaagaauucac......................................................................   500    0      egg
....................cuuggcacuggaagaauucGc......................................................................   2      1      egg
....................cuuAgcacuggaagaauucac......................................................................   1      1      egg
....................cuuggcacuggaagaauAcac......................................................................   1      1      egg
....................cuuggcacuggaagaGuucac......................................................................   1      1      egg
....................cuuggUacuggaagaauucac......................................................................   1      1      egg
....................cuugAcacuggaagaauucac......................................................................   5      1      egg
....................cuuggAacuggaagaauucac......................................................................   1      1      egg
....................cuugUcacuggaagaauucac......................................................................   13     1      egg
....................cuuggcacuggaagaauucaA......................................................................   7      1      egg
....................cuuggcacCggaagaauucac......................................................................   1      1      egg
....................cuuggcacuggaaAaauucac......................................................................   1      1      egg
....................cuuggcacAggaagaauucac......................................................................   1      1      egg
....................cuuggcacuggGagaauucac......................................................................   1      1      egg
....................cuuggcacuggaagGauucac......................................................................   2      1      egg
....................cuCggcacuggaagaauucac......................................................................   2      1      egg
....................cuuggcaUuggaagaauucac......................................................................   2      1      egg
....................cuuggcacuggaagaauucaU......................................................................   24     1      egg
....................cuuggcacuggaagaauCcac......................................................................   1      1      egg
....................cuuggcacugAaagaauucac......................................................................   4      1      egg
....................cuuUgcacuggaagaauucac......................................................................   1      1      egg
....................cuugCcacuggaagaauucac......................................................................   3      1      egg
....................cuuggcacCggaagaauucaca.....................................................................   5      1      egg
....................cuuggcacuggaUgaauucaca.....................................................................   2      1      egg
....................cuuggcacuggaGgaauucaca.....................................................................   5      1      egg
....................cuuggcacuggGagaauucaca.....................................................................   3      1      egg
....................cAuggcacuggaagaauucaca.....................................................................   1      1      egg
....................cuuUgcacuggaagaauucaca.....................................................................   2      1      egg
....................Uuuggcacuggaagaauucaca.....................................................................   1      1      egg
....................cuuggcacAggaagaauucaca.....................................................................   3      1      egg
....................cuuggcacuggaagaauuUaca.....................................................................   2      1      egg
....................cuuggcacuggaagaauuAaca.....................................................................   1      1      egg
....................cuuAgcacuggaagaauucaca.....................................................................   1      1      egg
....................cuuggcacuggaagaauucGca.....................................................................   5      1      egg
....................cuuggcaUuggaagaauucaca.....................................................................   1      1      egg
....................cuuggcacuggaagaauCcaca.....................................................................   7      1      egg
....................cuuggcacugAaagaauucaca.....................................................................   2      1      egg
....................cuuggcacuCgaagaauucaca.....................................................................   1      1      egg
....................cuugCcacuggaagaauucaca.....................................................................   6      1      egg
....................cuuggcacuggaagaauAcaca.....................................................................   3      1      egg
....................cuAggcacuggaagaauucaca.....................................................................   2      1      egg
....................cCuggcacuggaagaauucaca.....................................................................   4      1      egg
....................Nuuggcacuggaagaauucaca.....................................................................   6      1      egg
....................cuuggcacuUgaagaauucaca.....................................................................   1      1      egg
....................cuCggcacuggaagaauucaca.....................................................................   2      1      egg
....................cuuggcacuggaaAaauucaca.....................................................................   1      1      egg
....................cuugAcacuggaagaauucaca.....................................................................   5      1      egg
....................cuuggcacuggaagaauucacG.....................................................................   4      1      egg
....................cuuggcacuggaagaauucUca.....................................................................   1      1      egg
....................cuuggcacuggaagaaCucaca.....................................................................   3      1      egg
....................cuuggcacuggaagGauucaca.....................................................................   5      1      egg
....................cuuggcacuggaagaGuucaca.....................................................................   4      1      egg
....................cuuggcacuggaagaauGcaca.....................................................................   1      1      egg
....................cuuggcacuggaagaauucacU.....................................................................   9      1      egg
....................cuuggcacuggaagaauucaca.....................................................................   1224   0      egg
....................cuuggcacuAgaagaauucaca.....................................................................   4      1      egg
....................cuugUcacuggaagaauucaca.....................................................................   23     1      egg
```

Mature **Star**

acaauuggccuuaccuuauccuuggcacuggaagaauucacagaugcgauaaagaaacacucgugguucgucugguggccaaagauaaacgcaagguauacacucugaacuuu			
....................cuuggcacuggaagaauucacC...	8	1	egg
....................cuGggcacuggaagaauucaca...	1	1	egg
....................cuuggcGcuggaagaauucaca...	4	1	egg
....................cuuggcacuggaagaaAucaca...	1	1	egg
....................cuuggcacuggaagaauucaUa...	1	1	egg
....................cuuggcUcuggaagaauucaca...	2	1	egg
....................cuuggcacuggaagaauucGcag..	1	1	egg
....................cuuggcacuggaagaauucacCg..	1	1	egg
....................Uuuggcacuggaagaauucacag..	1	1	egg
....................cuuggcGcuggaagaauucacag..	3	1	egg
....................cuuggcacuggaGgaauucacag..	4	1	egg
....................cuuggcacuggaagGauucacag..	2	1	egg
....................cuuggcacuggaagaaCucacag..	1	1	egg
....................cuuggcacCggaagaauucacag..	1	1	egg
....................cuuggcacuggGagaauucacag..	2	1	egg
....................cuuggcacugUaagaauucacag..	1	1	egg
....................cuuggcacuggaagaauucacUg..	1	1	egg
....................cuuggcacuggaagaauucacag..	653	0	egg
....................cuuUgcacuggaagaauucacag..	1	1	egg
....................cuuAgcacuggaagaauucacag..	3	1	egg
....................cuuggcacuggaagaauucacGg..	1	1	egg
....................cuugAcacuggaagaauucacag..	4	1	egg
....................Nuuggcacuggaagaauucacag..	1	1	egg
....................cuuggcacuggaagaauucacaA..	26	1	egg
....................cuuggcacuggaagaauGcacag..	2	1	egg
....................cuuggcacuggaagaGuucacag..	2	1	egg
....................cuCggcacuggaagaauucacag..	1	1	egg
....................cCuggcacuggaagaauucacag..	1	1	egg
....................cuugCcacuggaagaauucacag..	2	1	egg
....................cuuggcacuggaagaauucacaC..	9	1	egg
....................cuuggcacuggaagaauucaUag..	2	1	egg
....................cuuggcacuggaagaauCcacag..	3	1	egg
....................cuuggcacuggaagaauAcacag..	2	1	egg
....................cuugUcacuggaagaauucacag..	20	1	egg
....................cuuggcacugAaagaauucacag..	1	1	egg
....................cuuggcacuggaagaauucacaU..	9	1	egg
....................cuAggcacuggaagaauucacag..	2	1	egg
....................Nuuggcacuggaagaauucacaga...	5	1	egg
....................cuuggcacuggaagaauucacagG...	2	1	egg
....................cuuggcacuggGagaauucacaga...	8	1	egg
....................cuuggcUcuggaagaauucacaga...	1	1	egg
....................cuuggcacuggaagaaCucacaga...	1	1	egg
....................cuuggcacuggaagGauucacaga...	3	1	egg
....................cuuggcacuggaagaauucacaga...	796	0	egg
....................Uuuggcacuggaagaauucacaga...	2	1	egg
....................cuuUgcacuggaagaauucacaga...	6	1	egg
....................cuuggcacuggaagaauucacaCa...	2	1	egg
....................cCuggcacuggaagaauucacaga...	4	1	egg
....................cuugCcacuggaagaauucacaga...	3	1	egg
....................cuuggcacCggaagaauucacaga...	4	1	egg
....................cuuggcacuggaaUaauucacaga...	1	1	egg
....................cuuggcacAggaagaauucacaga...	2	1	egg
....................cuuggcacuggaagaGuucacaga...	4	1	egg
....................cuuggcaUuggaagaauucacaga...	1	1	egg
....................cuuggcacuggaagaauCcacaga...	5	1	egg
....................cuuggcacuggaagaauucacagC...	6	1	egg
....................cuuggUacuggaagaauucacaga...	2	1	egg
....................cuuggcacuggaagaaGucacaga...	1	1	egg
....................cuuggcacuggaagaauucGcaga...	1	1	egg
....................cuugAcacuggaagaauucacaga...	3	1	egg
....................cuuggcGcuggaagaauucacaga...	5	1	egg
....................cuuggcacuggaagaauucacGga...	2	1	egg
....................cuuggcacuggaagaauucacagU...	11	1	egg
....................cuCggcacuggaagaauucacaga...	5	1	egg
....................cuuggcaAuggaagaauucacaga...	1	1	egg
....................cuugUcacuggaagaauucacaga...	30	1	egg
....................cuuggcacuggaagaauAcacaga...	1	1	egg
....................cuuAgcacuggaagaauucacaga...	2	1	egg
....................cuuggcacugCaagaauucacaga...	1	1	egg
....................cuuggcacuggaagaauuAacaga...	1	1	egg

Mature Star

```
acaauuggccuuaccuuauccuuggcacuggaagaauucacagaugcgauaaagaaacacucgugguucgucuggugccaaagauaaacgcaagguauacacucugaacuuu
....................cuuggcacuggaagaauucaUaga....................................................................    1    1    egg
....................cuuggcacuggaagaauGcacaga....................................................................    1    1    egg
....................cuuggcacuggaagaauucacagau..................................................................    21   0    egg
....................cuuggcacuggaGgaauucacagau..................................................................    1    1    egg
....................cuuggcacuggaagaauucacagaA..................................................................    10   1    egg
....................cuugUcacuggaagaauucacagau..................................................................    1    1    egg
....................cuuAgcacuggaagaauucacagau..................................................................    1    1    egg
....................cuuggcacuggaagaauucacagaC..................................................................    7    1    egg
....................cuuggcacuggaagaauucacagaCg.................................................................    2    1    egg
....................cuuggcacuggaagaauucacagauC.................................................................    5    1    egg
....................cuuggcacuggaagaauucacagauU.................................................................    28   1    egg
....................cuuggcacuggaagaauucacagauA.................................................................    14   1    egg
....................cuuggcacuggaagaauucacagauUc................................................................    2    1    egg
.....................uuggcacuggaagaauucaU.......................................................................    1    1    egg
.....................uuggcacuggaagaauucac.......................................................................    6    0    egg
.....................uuggcCcuggaagaauucac.......................................................................    1    1    egg
.....................uuggGacuggaagaauucaca......................................................................    1    1    egg
.....................uuggcacuggaagaauucaca......................................................................    23   0    egg
.....................uuggcacuggaagaauucacU......................................................................    1    1    egg
.....................uuggcacuggaagaauucGca......................................................................    1    1    egg
.....................uuggcacuggaagaaCucacag.....................................................................    1    1    egg
.....................uugCcacuggaagaauucacag.....................................................................    1    1    egg
.....................uuAgcacuggaagaauucacag.....................................................................    1    1    egg
.....................uuggcacuggaagaauucacaA.....................................................................    4    1    egg
.....................uugUcacuggaagaauucacag.....................................................................    1    1    egg
.....................uuggcacuggaagaauucacag.....................................................................    48   0    egg
.....................uuggcacuggaagaaGucacag.....................................................................    1    1    egg
.....................Auggcacuggaagaauucacag.....................................................................    1    1    egg
.....................uCggcacuggaagaauucacag.....................................................................    1    1    egg
.....................uuggcacuggaagaauucGcag.....................................................................    1    1    egg
.....................uuggcaUuggaagaauucacag.....................................................................    1    1    egg
.....................uuggcacuggaagaauucacagU....................................................................    1    1    egg
.....................uuggcCcuggaagaauucacaga....................................................................    1    1    egg
.....................uuggcacuggaagaauucacGga....................................................................    2    1    egg
.....................uuggcacuggaagaauCcacaga....................................................................    2    1    egg
.....................uuggcacuggaagaauucacaga....................................................................    119  0    egg
.....................uuggcacuggaagaauucacagG....................................................................    3    1    egg
.....................uuggcacuggaGgaauucacaga....................................................................    1    1    egg
.....................uuggcacugAaagaauucacaga....................................................................    1    1    egg
.....................uugUcacuggaagaauucacaga....................................................................    1    1    egg
.....................uuggcacuggaagaauucGcaga....................................................................    1    1    egg
.....................Nuggcacuggaagaauucacaga....................................................................    1    1    egg
.....................uuggUacuggaagaauucacaga....................................................................    1    1    egg
.....................uuggcacuggaCgaauucacaga....................................................................    1    1    egg
.....................uuggcacuggaagaauucacagaA...................................................................    2    1    egg
.....................uuggcacuggaagaauucacagau...................................................................    1    0    egg
.....................uuggcacuggaagaauucacagauU..................................................................    1    1    egg
.....................uuggcacuggaagaauucacagauC..................................................................    1    1    egg
.....................uuggcacuggaagaauucacagauA..................................................................    1    1    egg
......................uggcacuggaagaauucacaga....................................................................    1    0    egg
......................uggcacuggaagaauucacagau...................................................................    2    0    egg
......................uggcacuggaagaauucacagaug..................................................................    3    0    egg
............................................................ucgugguucgucuggugcc.................................    2    0    egg
..............................................................gugguucgucuggugccaaaga............................    10   0    egg
..............................................................gugguucgucuggugccaaagaA...........................    1    1    egg
..............................................................gugguucgucuggugccaaagaua..........................    1    0    egg
...............................................................ugguucgucuggugccaaaga............................    2    0    egg
...............................................................ugguucgucuggugccaaagauU..........................    4    1    egg

acaauuggccuuaccuuauc............................................................................................    1    0    te1
..................uUcuuggcacuggaagaauuc.........................................................................    4    1    te1
..................Cccuuggcacuggaagaauuca........................................................................    1    1    te1
..................uUcuuggcacuggaagaauuca........................................................................    19   1    te1
..................uUcuuggcacuggaagaauucac.......................................................................    38   1    te1
..................uUcuuggcacuggaagaauucaca......................................................................    9    1    te1
..................uGcuuggcacuggaagaauucaca......................................................................    1    1    te1
..................uUcuuggcacuggaagaauucacag.....................................................................    2    1    te1
..................uUcuuggcacuggaagaauucacaga....................................................................    2    1    te1
...................ccuuggcacuggaagaauu..........................................................................    2    0    te1
...................cUuuggcacuggaagaauuc.........................................................................    1    1    te1
```

```
                    Mature                                  Star
acaauuggccuuaccuuauccuuggcacuggaagaauucacagaugcgauaagaaacacucgugguucgucuggugccaaagauaaacgcaagguauacacucugaacuuu
...................Acuuggcacuggaagaauuc........................................................................    1      1    te1
...................ccuuggcacuggaagaauuA........................................................................    3      1    te1
...................ccuuggcacuggaagaauuc........................................................................    223    0    te1
...................Ucuuggcacuggaagaauuc........................................................................    2      1    te1
...................ccuuggcacuggGagaauuca.......................................................................    1      1    te1
...................ccuuggcacuggaagaauuca.......................................................................    289    0    te1
...................Ucuuggcacuggaagaauuca.......................................................................    22     1    te1
...................cGuuggcacuggaagaauuca.......................................................................    5      1    te1
...................cAuuggcacuggaagaauuca.......................................................................    2      1    te1
...................ccuuggcacuCgaagaauucac......................................................................    1      1    te1
...................Ucuuggcacuggaagaauucac......................................................................    68     1    te1
...................Acuuggcacuggaagaauucac......................................................................    2      1    te1
...................ccuuggcacuggaagaauucaA......................................................................    6      1    te1
...................ccuuggcacuggaagaauucac......................................................................    144    0    te1
...................cAuuggcacuggaagaauucac......................................................................    13     1    te1
...................ccuuggcacuggaagaauucaU......................................................................    2      1    te1
...................cGuuggcacuggaagaauucac......................................................................    10     1    te1
...................cUuuggcacuggaagaauucac......................................................................    3      1    te1
...................ccuuggcacuggaagaauucacU.....................................................................    1      1    te1
...................cGuuggcacuggaagaauucaca.....................................................................    7      1    te1
...................ccuuggcacuggaagaauucaca.....................................................................    21     0    te1
...................Ucuuggcacuggaagaauucaca.....................................................................    42     1    te1
...................cAuuggcacuggaagaauucaca.....................................................................    6      1    te1
...................Gcuuggcacuggaagaauucacag....................................................................    1      1    te1
...................cGuuggcacuggaagaauucacag....................................................................    2      1    te1
...................ccuuggcacuggaagaauucacaA....................................................................    1      1    te1
...................cAuuggcacuggaagaauucacag....................................................................    3      1    te1
...................Ucuuggcacuggaagaauucacag....................................................................    8      1    te1
...................ccuuggcacuggaagaauucacaU....................................................................    5      1    te1
...................cGuuggcacuggaagaauucacaga...................................................................    2      1    te1
...................cAuuggcacuggaagaauucacaga...................................................................    1      1    te1
...................Gcuuggcacuggaagaauucacaga...................................................................    1      1    te1
...................Ucuuggcacuggaagaauucacaga...................................................................    3      1    te1
...................ccuuggcacuggaagaauucacaga...................................................................    2      0    te1
....................cuuggcacuggaagaauu.........................................................................    68     0    te1
....................cuuggcacuggGagaauu.........................................................................    1      1    te1
....................cuuggcGcuggaagaauu.........................................................................    1      1    te1
....................cuuggcacuggaagaauA.........................................................................    4      1    te1
....................cuuggcaUuggaagaauuc........................................................................    1      1    te1
....................cuuggcacuggaagaauAc........................................................................    17     1    te1
....................cuuggcGcuggaagaauuc........................................................................    1      1    te1
....................cuGggcacuggaagaauuc........................................................................    1      1    te1
....................cuuggcacuggaagaaGuc........................................................................    1      1    te1
....................cuuggcacuCgaagaauuc........................................................................    1      1    te1
....................cuuggcacCggaagaauuc........................................................................    1      1    te1
....................cuuggcacuggaagaauuU........................................................................    45     1    te1
....................Auuggcacuggaagaauuc........................................................................    1      1    te1
....................cuuggcacuggaagaauuG........................................................................    3      1    te1
....................Uuuggcacuggaagaauuc........................................................................    5      1    te1
....................cuuggcacuggaagaauuA........................................................................    246    1    te1
....................Guuggcacuggaagaauuc........................................................................    5      1    te1
....................cuuggcacuggUagaauuc........................................................................    1      1    te1
....................cuuggcacuggaGgaauuc........................................................................    1      1    te1
....................cuuggcCcuggaagaauuc........................................................................    1      1    te1
....................cuuggcacugAaagaauuc........................................................................    2      1    te1
....................cuuggcacuggaagaauuc........................................................................    7313   0    te1
....................cuuggcacugCaagaauuc........................................................................    4      1    te1
....................cuuCgcacuggaagaauuc........................................................................    4      1    te1
....................cuuggcacuggaagGauuc........................................................................    7      1    te1
....................cuugAcacuggaagaauuc........................................................................    2      1    te1
....................cuuggcacuggCagaauuc........................................................................    5      1    te1
....................cuuggcacuggaagUauuc........................................................................    1      1    te1
....................cuuggcacuggaagaUuuc........................................................................    1      1    te1
....................cuuggUacuggaagaauuc........................................................................    3      1    te1
....................cuugCcacuggaagaauuc........................................................................    2      1    te1
....................cuuggcacuggaagaaAuc........................................................................    1      1    te1
....................cuuggcacuggGagaauuc........................................................................    16     1    te1
....................cuuggcacAggaagaauuc........................................................................    1      1    te1
....................cuuggGacuggaagaauuc........................................................................    1      1    te1
....................cuuggcacuggaCgaauuc........................................................................    1      1    te1
```

```
                    Mature                                    Star
acaauuggccuuaccuuauccuuggcacuggaagaauucacagaugcgauaaagaaacacucgugguucgucuggugccaaagauaaacgcaagguauacacucugaacuuu
....................Guuggcacuggaagaauuca........................................................................ 57    1 te1
....................cGuggcacuggaagaauuca........................................................................ 16    1 te1
....................cuuggcacuggaGgaauuca........................................................................ 10    1 te1
....................cuuggcacugUaagaauuca........................................................................ 11    1 te1
....................cuuggcacuggaagGauuca........................................................................ 45    1 te1
....................cuuggcaGuggaagaauuca........................................................................ 5     1 te1
....................cuuggcacuUgaagaauuca........................................................................ 3     1 te1
....................Auuggcacuggaagaauuca........................................................................ 13    1 te1
....................cuuggcacuggaagaauuUa........................................................................ 44    1 te1
....................cuuggcacuggaagaauucU........................................................................ 155   1 te1
....................cuCggcacuggaagaauuca........................................................................ 5     1 te1
....................cuuggcacuggaagaCuuca........................................................................ 4     1 te1
....................cuuCgcacuggaagaauuca........................................................................ 21    1 te1
....................cuuggcacuggaagaaGuca........................................................................ 5     1 te1
....................cuuggcacuggaagaauGca........................................................................ 13    1 te1
....................cuuggcacuggaagaauuAa........................................................................ 188   1 te1
....................cuuggGacuggaagaauuca........................................................................ 1     1 te1
....................cuuggUacuggaagaauuca........................................................................ 46    1 te1
....................cuuggcacuAgaagaauuca........................................................................ 4     1 te1
....................cuuggcacCggaagaauuca........................................................................ 10    1 te1
....................cuuUgcacuggaagaauuca........................................................................ 2     1 te1
....................cuGggcacuggaagaauuca........................................................................ 7     1 te1
....................cCuggcacuggaagaauuca........................................................................ 1     1 te1
....................cuuggcacuggaagaGuuca........................................................................ 6     1 te1
....................cuugAcacuggaagaauuca........................................................................ 22    1 te1
....................cuuggcacuggaagaUuuca........................................................................ 1     1 te1
....................cuugCcacuggaagaauuca........................................................................ 16    1 te1
....................cuuggcacuggaagaauCca........................................................................ 20    1 te1
....................cuugUcacuggaagaauuca........................................................................ 12    1 te1
....................cuuggcacuggaagaauuca........................................................................ 55270 0 te1
....................cuuggcacuggUagaauuca........................................................................ 5     1 te1
....................cuuggcacuggGagaauuca........................................................................ 84    1 te1
....................cuuggcacuggaagaauucC........................................................................ 4     1 te1
....................cuuggcacugCaagaauuca........................................................................ 26    1 te1
....................cuuggcacuggaagaauuGa........................................................................ 2     1 te1
....................cuuAgcacuggaagaauuca........................................................................ 10    1 te1
....................cuuggcacuggaaCaauuca........................................................................ 3     1 te1
....................cuuggcacuggaagaauucG........................................................................ 27    1 te1
....................cuuggcacuggaaUaauuca........................................................................ 4     1 te1
....................cuuggcacuggaaAaauuca........................................................................ 3     1 te1
....................cuuggAacuggaagaauuca........................................................................ 1     1 te1
....................cuuggcacuggaagaaAuca........................................................................ 3     1 te1
....................cuuggcacuggaCgaauuca........................................................................ 1     1 te1
....................cuuggcacGggaagaauuca........................................................................ 1     1 te1
....................cuuggcaUuggaagaauuca........................................................................ 27    1 te1
....................cuuggcCcuggaagaauuca........................................................................ 3     1 te1
....................Uuuggcacuggaagaauuca........................................................................ 27    1 te1
....................cAuggcacuggaagaauuca........................................................................ 7     1 te1
....................cuuggcacAggaagaauuca........................................................................ 1     1 te1
....................cuuggcacuCgaagaauuca........................................................................ 4     1 te1
....................cuuggcacuggaagUauuca........................................................................ 9     1 te1
....................cuuggcacuggaagaaCuca........................................................................ 4     1 te1
....................cuuggcacugAaagaauuca........................................................................ 17    1 te1
....................cuuggcGcuggaagaauuca........................................................................ 1     1 te1
....................cuuggcacuggaagCauuca........................................................................ 3     1 te1
....................cuuggcacuggaUgaauuca........................................................................ 6     1 te1
....................cuuggcacuggCagaauuca........................................................................ 11    1 te1
....................cuuggcUcuggaagaauucac....................................................................... 6     1 te1
....................cuugCcacuggaagaauucac....................................................................... 16    1 te1
....................cuuAgcacuggaagaauucac....................................................................... 7     1 te1
....................cuuUgcacuggaagaauucac....................................................................... 10    1 te1
....................cuuggcacugUaagaauucac....................................................................... 22    1 te1
....................cuuggcacuggUagaauucac....................................................................... 17    1 te1
....................cuuggcacuggaagaaGucac....................................................................... 9     1 te1
....................cuuggcacGggaagaauucac....................................................................... 4     1 te1
....................cuuggcacuggaagaauuGac....................................................................... 9     1 te1
....................cuuggcacuggaagCauucac....................................................................... 15    1 te1
....................cuuggcCcuggaagaauucac....................................................................... 9     1 te1
....................cuuggcacuggaagaauGcac....................................................................... 18    1 te1
....................cuuggcacuggaaAaauucac....................................................................... 10    1 te1
```

Mature **Star**

```
acaauuggccuuaccuuauccuuggcacuggaagaauucacagaugcgauaagaaacacucgugguucgucuggugccaaagauaaacgcaagguauacacucugaacuuu
....................cuuggcacuggaagUauucac...................................................................... 6 1 te1
....................cuuCgcacuggaagaauucac...................................................................... 30 1 te1
....................cCuggcacuggaagaauucac...................................................................... 7 1 te1
....................cuuggcacuggaagaauucaG...................................................................... 30 1 te1
....................cuuggcacuggaagaauucaU...................................................................... 542 1 te1
....................cuuggcacuggaagaaAucac...................................................................... 20 1 te1
....................cuuggGacuggaagaauucac...................................................................... 4 1 te1
....................cGuggcacuggaagaauucac...................................................................... 28 1 te1
....................Guuggcacuggaagaauucac...................................................................... 89 1 te1
....................cuuggcacuAgaagaauucac...................................................................... 2 1 te1
....................cuuggcaAuggaagaauucac...................................................................... 2 1 te1
....................cuuggcacuggGagaauucac...................................................................... 195 1 te1
....................cuuggcacugCaagaauucac...................................................................... 47 1 te1
....................cuuggUacuggaagaauucac...................................................................... 110 1 te1
....................cuuggcaGuggaagaauucac...................................................................... 13 1 te1
....................cuuggcacuggaagaauucaA...................................................................... 3516 1 te1
....................cuuggcacuUgaagaauucac...................................................................... 12 1 te1
....................cuuggcacuggaagaGuucac...................................................................... 13 1 te1
....................cuuggcacuggaaUaauucac...................................................................... 8 1 te1
....................Auuggcacuggaagaauucac...................................................................... 15 1 te1
....................cuuggcacuggaagGauucac...................................................................... 63 1 te1
....................cuuggcacuggaagaauuUac...................................................................... 124 1 te1
....................cuuggcacuggaagaauucac...................................................................... 116607 0 te1
....................cuugAcacuggaagaauucac...................................................................... 46 1 te1
....................cuuggcacuggaGgaauucac...................................................................... 12 1 te1
....................cuuggcacuggaaCaauucac...................................................................... 10 1 te1
....................cuuggcacuggaagaauAcac...................................................................... 15 1 te1
....................cuCggcacuggaagaauucac...................................................................... 10 1 te1
....................cuuggcacuggaCgaauucac...................................................................... 4 1 te1
....................cuuggcacuggaagaUuucac...................................................................... 7 1 te1
....................cuuggcacuggaagaauCcac...................................................................... 57 1 te1
....................cuuggcacuggaagaauucGc...................................................................... 5 1 te1
....................cuuggcacuCgaagaauucac...................................................................... 9 1 te1
....................cuuggcacugAaagaauucac...................................................................... 32 1 te1
....................cuuggcacAggaagaauucac...................................................................... 2 1 te1
....................cuGggcacuggaagaauucac...................................................................... 15 1 te1
....................cuuggcacuggaagaauuAac...................................................................... 1 1 te1
....................cuuggAacuggaagaauucac...................................................................... 1 1 te1
....................cuuggcacuggaagaaCucac...................................................................... 9 1 te1
....................cuAggcacuggaagaauucac...................................................................... 1 1 te1
....................Uuuggcacuggaagaauucac...................................................................... 62 1 te1
....................cuuggcacuggaagaauucUc...................................................................... 10 1 te1
....................cuuggcaUuggaagaauucac...................................................................... 37 1 te1
....................cAuggcacuggaagaauucac...................................................................... 20 1 te1
....................cuugUcacuggaagaauucac...................................................................... 13 1 te1
....................cuuggcacuggaagaCuucac...................................................................... 3 1 te1
....................cuuggcacuggaagaauucCc...................................................................... 3 1 te1
....................cuuggcGcuggaagaauucac...................................................................... 8 1 te1
....................cuuggcacuggaUgaauucac...................................................................... 4 1 te1
....................cuuggcacuggCagaauucac...................................................................... 41 1 te1
....................cuuggcacCggaagaauucac...................................................................... 14 1 te1
....................cuuggcUcuggaagaauucaca..................................................................... 2 1 te1
....................cuuggcacuggaagaauAcaca..................................................................... 8 1 te1
....................cuuggcacuggaagaauucacG..................................................................... 432 1 te1
....................cuuAgcacuggaagaauucaca..................................................................... 6 1 te1
....................cuuggcacuggUagaauucaca..................................................................... 13 1 te1
....................cuuggcacuggaagaauucaca..................................................................... 96916 0 te1
....................cuuggcaGuggaagaauucaca..................................................................... 8 1 te1
....................cuuggcacuggaagaauuAaca..................................................................... 1 1 te1
....................cuuggcacuggaagaGuucaca..................................................................... 12 1 te1
....................cuuggcacuggaagaaCucaca..................................................................... 3 1 te1
....................cuuggcacuggaaAaauucaca..................................................................... 3 1 te1
....................cuuUgcacuggaagaauucaca..................................................................... 4 1 te1
....................cuuggcacuggaagaauucUca..................................................................... 5 1 te1
....................cuuggcacugAaagaauucaca..................................................................... 28 1 te1
....................cuuggcacuggaagaauucacU..................................................................... 2496 1 te1
....................cuuggGacuggaagaauucaca..................................................................... 2 1 te1
....................cuuggcacuggaagaaGucaca..................................................................... 4 1 te1
....................cuuggcacuggaagaauucaUa..................................................................... 35 1 te1
....................cuCggcacuggaagaauucaca..................................................................... 6 1 te1
```

Mature Star

```
acaauuggccuuaccuuauccuuggcacuggaagaauucacagaugcgauaagaaacacucgugguucgucuggugccaaagauaaacgcaagguauacacucugaacuuu
....................cuuggcacuggaagaauGcaca.....................................................................  17    1  te1
....................cuuggcacuggaUgaauucaca.....................................................................  4     1  te1
....................cuuggcacuggaagaauucaGa.....................................................................  7     1  te1
....................cuuggcacugCaagaauucaca.....................................................................  34    1  te1
....................cuuggcacuggaagaauucCca.....................................................................  3     1  te1
....................cuuggcacGggaagaauucaca.....................................................................  3     1  te1
....................cuuggcacuggaagaauuGaca.....................................................................  8     1  te1
....................cuuggcacCggaagaauucaca.....................................................................  14    1  te1
....................cuuggcacuggaagaUuucaca.....................................................................  5     1  te1
....................cuuggcacuggaagaaAucaca.....................................................................  5     1  te1
....................cuuggcacuCgaagaauucaca.....................................................................  1     1  te1
....................cuuggcacuggaagCauucaca.....................................................................  9     1  te1
....................cuuggcacuggaaCaauucaca.....................................................................  5     1  te1
....................cuGggcacuggaagaauucaca.....................................................................  10    1  te1
....................cuugAcacuggaagaauucaca.....................................................................  41    1  te1
....................cuuggcacuggaaUaauucaca.....................................................................  5     1  te1
....................cuuggcacuggaagaauCcaca.....................................................................  27    1  te1
....................cuuggcacuggCagaauucaca.....................................................................  45    1  te1
....................cCuggcacuggaagaauucaca.....................................................................  9     1  te1
....................cuuggcGcuggaagaauucaca.....................................................................  5     1  te1
....................cuuggcCcuggaagaauucaca.....................................................................  5     1  te1
....................cuuggcacuggGagaauucaca.....................................................................  151   1  te1
....................cuugUcacuggaagaauucaca.....................................................................  10    1  te1
....................cuAggcacuggaagaauucaca.....................................................................  4     1  te1
....................cuuggcacugUaagaauucaca.....................................................................  14    1  te1
....................cuuggcacuggaagaCuucaca.....................................................................  3     1  te1
....................cuuggcacuggaagaauucacC.....................................................................  36    1  te1
....................Uuuggcacuggaagaauucaca.....................................................................  48    1  te1
....................cuuggcacuggaCgaauucaca.....................................................................  2     1  te1
....................cuuggcaAuggaagaauucaca.....................................................................  2     1  te1
....................cGuggcacuggaagaauucaca.....................................................................  17    1  te1
....................cuuggcacuAgaagaauucaca.....................................................................  7     1  te1
....................cuuggcacuggaagaauuUaca.....................................................................  65    1  te1
....................cuuggcaUuggaagaauucaca.....................................................................  19    1  te1
....................cuuggcacuUgaagaauucaca.....................................................................  5     1  te1
....................Auuggcacuggaagaauucaca.....................................................................  3     1  te1
....................cuuggcacuggaagUauucaca.....................................................................  5     1  te1
....................cAuggcacuggaagaauucaca.....................................................................  11    1  te1
....................cuuggcacuggaagaauucaAa.....................................................................  183   1  te1
....................Guuggcacuggaagaauucaca.....................................................................  61    1  te1
....................cuuCgcacuggaagaauucaca.....................................................................  18    1  te1
....................cuuggcacuggaagGauucaca.....................................................................  40    1  te1
....................cuuggcacuggaagaauucGca.....................................................................  7     1  te1
....................cuugCcacuggaagaauucaca.....................................................................  15    1  te1
....................cuuggcacuggaGgaauucaca.....................................................................  8     1  te1
....................cuuggUacuggaagaauucaca.....................................................................  100   1  te1
....................Uuuggcacuggaagaauucacag....................................................................  21    1  te1
....................cuuggcacuggaagGauucacag....................................................................  10    1  te1
....................cuuAgcacuggaagaauucacag....................................................................  1     1  te1
....................cuuggcacuggaagaauucUcag....................................................................  4     1  te1
....................cuuggcacuggaagaauucacUg....................................................................  13    1  te1
....................cuugUcacuggaagaauucacag....................................................................  2     1  te1
....................Guuggcacuggaagaauucacag....................................................................  18    1  te1
....................cuuggcacuggaagUauucacag....................................................................  2     1  te1
....................cuGggcacuggaagaauucacag....................................................................  6     1  te1
....................cuuggcacuggaagaauucacaU....................................................................  1917  1  te1
....................cuuggcacuggCagaauucacag....................................................................  17    1  te1
....................cuuggcacuggaagaauucaUag....................................................................  22    1  te1
....................cuuggcacuggaUgaauucacag....................................................................  1     1  te1
....................cuuggcacuggGagaauucacag....................................................................  35    1  te1
....................cuuCgcacuggaagaauucacag....................................................................  4     1  te1
....................cuuggcCcuggaagaauucacag....................................................................  1     1  te1
....................cuuggcacuggUagaauucacag....................................................................  4     1  te1
....................cuuggcacuggaaCaauucacag....................................................................  1     1  te1
....................cuuggcacuggaagaauCcacag....................................................................  3     1  te1
....................cuuggcacCggaagaauucacag....................................................................  2     1  te1
....................cuuggcacuggaagaGuucacag....................................................................  4     1  te1
....................cuuggcaGuggaagaauucacag....................................................................  3     1  te1
....................cAuggcacuggaagaauucacag....................................................................  8     1  te1
....................cuuggcacuggaagaaGucacag....................................................................  4     1  te1
```

```
                    Mature                   Star
acaauuggccuuaccuuauccuuggcacuggaagaauucacagaugcgauaaagaaacacucgugguucgucuggugccaaagauaaacgcaagguauacacucugaacuuu
....................cuuggcacuggaagaauuUacag.....................................................................    18      1    te1
....................cuuggcacuggaagaaCucacag.....................................................................    1       1    te1
....................cuuggcacuggaagaauGcacag.....................................................................    6       1    te1
....................cuuggcacugUaagaauucacag.....................................................................    6       1    te1
....................cCuggcacuggaagaauucacag.....................................................................    1       1    te1
....................cuuggcacuggaCgaauucacag.....................................................................    3       1    te1
....................cuuggcacuggaagaauucGcag.....................................................................    4       1    te1
....................cGuggcacuggaagaauucacag.....................................................................    2       1    te1
....................cuuggcacuggaagaauucacGg.....................................................................    30      1    te1
....................cuCggcacuggaagaauucacag.....................................................................    2       1    te1
....................cuuUgcacuggaagaauucacag.....................................................................    2       1    te1
....................cuuggcacuggaagaUuucacag.....................................................................    4       1    te1
....................cuuggcacuggaGgaauucacag.....................................................................    4       1    te1
....................cuuggcacuggaagCauucacag.....................................................................    1       1    te1
....................cuuggcacuggaagaauAcacag.....................................................................    1       1    te1
....................cuuggcGcuggaagaauucacag.....................................................................    3       1    te1
....................cuugAcacuggaagaauucacag.....................................................................    22      1    te1
....................cuuggcacuggaagaauucacCg.....................................................................    3       1    te1
....................cuuggcacuggaagaauucacag.....................................................................    33433   0    te1
....................cuuggcacuUgaagaauucacag.....................................................................    1       1    te1
....................cuuggcacuggaaAaauucacag.....................................................................    1       1    te1
....................cuuggGacuggaagaauucacag.....................................................................    1       1    te1
....................cuuggcacuggaagaauuGacag.....................................................................    5       1    te1
....................cuuggcacuggaaUaauucacag.....................................................................    1       1    te1
....................cuuggcacuggaagaauucacaC.....................................................................    1590    1    te1
....................cuuggcacugAaagaauucacag.....................................................................    11      1    te1
....................cuuggUacuggaagaauucacag.....................................................................    25      1    te1
....................cuugCcacuggaagaauucacag.....................................................................    3       1    te1
....................cuuggcacuAgaagaauucacag.....................................................................    4       1    te1
....................cuAggcacuggaagaauucacag.....................................................................    2       1    te1
....................cuuggcacugCaagaauucacag.....................................................................    11      1    te1
....................cuuggAacuggaagaauucacag.....................................................................    1       1    te1
....................cuuggcaUuggaagaauucacag.....................................................................    6       1    te1
....................cuuggcacuggaagaauucacaA.....................................................................    1388    1    te1
....................cuuggcacuggaagaauuGacaga....................................................................    1       1    te1
....................cuuggcacuggaagaauucacaAa....................................................................    67      1    te1
....................cuCggcacuggaagaauucacaga....................................................................    2       1    te1
....................cuAggcacuggaagaauucacaga....................................................................    1       1    te1
....................cuuggUacuggaagaauucacaga....................................................................    18      1    te1
....................cuuggcacuggaGgaauucacaga....................................................................    1       1    te1
....................Auuggcacuggaagaauucacaga....................................................................    2       1    te1
....................cuuCgcacuggaagaauucacaga....................................................................    2       1    te1
....................cuuggcacuggaagaauuUacaga....................................................................    2       1    te1
....................cuuggcacuggaagaauucacagC....................................................................    15      1    te1
....................cCuggcacuggaagaauucacaga....................................................................    2       1    te1
....................cuuggcCcuggaagaauucacaga....................................................................    2       1    te1
....................cuuggcacuUgaagaauucacaga....................................................................    1       1    te1
....................cuuggcacuggaagaauucacUga....................................................................    4       1    te1
....................cuuggcacuggaagGauucacaga....................................................................    11      1    te1
....................cuuggcacuggaaAaauucacaga....................................................................    1       1    te1
....................cuuggcacuggaagaauucaGaga....................................................................    1       1    te1
....................cuuggcacugCaagaauucacaga....................................................................    2       1    te1
....................cuugCcacuggaagaauucacaga....................................................................    4       1    te1
....................cuuggcacugAaagaauucacaga....................................................................    2       1    te1
....................cuuggcacuggaagaauucacaCa....................................................................    83      1    te1
....................Uuuggcacuggaagaauucacaga....................................................................    5       1    te1
....................cuuggcacuggaagaauAcacaga....................................................................    2       1    te1
....................cuuggcacuggaUgaauucacaga....................................................................    1       1    te1
....................cuuggcacuggaagaauucacagU....................................................................    802     1    te1
....................cuuggcacuggaagaauucacagG....................................................................    88      1    te1
....................cuuggcacuggaagaaCucacaga....................................................................    5       1    te1
....................cuuggcacuggCagaauucacaga....................................................................    6       1    te1
....................cGuggcacuggaagaauucacaga....................................................................    3       1    te1
....................cuuggcacuggaagaauucacGga....................................................................    3       1    te1
....................cuuggcacuggaaUaauucacaga....................................................................    2       1    te1
....................cuuggcacuggaagaauGcacaga....................................................................    5       1    te1
....................cuuggcacugUaagaauucacaga....................................................................    2       1    te1
....................cuugUcacuggaagaauucacaga....................................................................    1       1    te1
....................cuuggcacuggaagaauucacaga....................................................................    13653   0    te1
....................cuuggcacuggaagUauucacaga....................................................................    1       1    te1
```

Mature **Star**

```
acaauuggccuuaccuuauccuuggcacuggaagaauucacagaugcgauaaagaaacacucgugguucgucuggugccaaagauaaacgcaagguauacacucugaacuuu
....................cuGggcacuggaagaauucacaga....................................................................  2     1  te1
....................Guuggcacuggaagaauucacaga....................................................................  10    1  te1
....................cuuggcacuggaCgaauucacaga....................................................................  1     1  te1
....................cuuggcacuggGagaauucacaga....................................................................  14    1  te1
....................cuuggcacuggaagaauucaUaga....................................................................  12    1  te1
....................cuuggcacuggaagaauCcacaga....................................................................  2     1  te1
....................cuuggcacuggaagaaGucacaga....................................................................  1     1  te1
....................cuuggcacuggaagaauucacaUa....................................................................  23    1  te1
....................cuuggcacCggaagaauucacaga....................................................................  2     1  te1
....................cuuggcaUuggaagaauucacaga....................................................................  4     1  te1
....................cuugAcacuggaagaauucacaga....................................................................  6     1  te1
....................cAuggcacuggaagaauucacaga....................................................................  5     1  te1
....................cuuggcacuggaagaauucacagaC...................................................................  11    1  te1
....................cuuggcacuggaagGauucacagau...................................................................  1     1  te1
....................cuuggcacuggaagaauucacagUu...................................................................  75    1  te1
....................cuuggcacuggaagaauucacGgau...................................................................  1     1  te1
....................cuuggcacuggaagaauucacagGu...................................................................  1     1  te1
....................cuuggcacuggaagaauucacaCau...................................................................  2     1  te1
....................cuuggcacuggaagaauucacagaA...................................................................  688   1  te1
....................Uuuggcacuggaagaauucacagau...................................................................  3     1  te1
....................cuuggcacuggaagaauucacagau...................................................................  1052  0  te1
....................cuuggcacuggaaAaauucacagau...................................................................  1     1  te1
....................cuuggcacuggGagaauucacagau...................................................................  3     1  te1
....................cuuggcacuggaagaauucacaAau...................................................................  3     1  te1
....................cuuggcacuggaagaauuUacagau...................................................................  1     1  te1
....................cuuggcacuggaagaauucacagCu...................................................................  1     1  te1
....................cuuggcacuggaagaauucaUagau...................................................................  3     1  te1
....................cuuggcacuggaagaauucacagaG...................................................................  5     1  te1
....................cuuggUacuggaagaauucacagau...................................................................  1     1  te1
....................cuuggcacuggaagaauucacaUau...................................................................  15    1  te1
....................cuuggcacuggaagaauucacagaAg..................................................................  1     1  te1
....................cuuggcacuggaagaauucacagaug..................................................................  4     0  te1
....................cuuggcacuggaagaauucacagauA..................................................................  30    1  te1
....................cuuggcacuggaagaauucacagauU..................................................................  16    1  te1
....................cuuggcacuggaagaauucacagaugA.................................................................  1     1  te1
....................cuuggcacuggaagaauucacagaugU.................................................................  1     1  te1
.....................uuggcacuggaagaauuc.........................................................................  10    0  te1
.....................uuUgcacuggaagaauuca........................................................................  1     1  te1
.....................uuggcacuggaagaauuca........................................................................  149   0  te1
.....................Cuggcacuggaagaauuca........................................................................  3     1  te1
.....................uuggcacuggaagaauuAa........................................................................  1     1  te1
.....................uuggcacuggaagaauuUac.......................................................................  1     1  te1
.....................uugAcacuggaagaauucac.......................................................................  1     1  te1
.....................Cuggcacuggaagaauucac.......................................................................  8     1  te1
.....................uuggcacuggaagaauucUc.......................................................................  1     1  te1
.....................uuggcacuggaagaauucac.......................................................................  919   0  te1
.....................uuggUacuggaagaauucac.......................................................................  3     1  te1
.....................uuggcacuggaagaauucaA.......................................................................  6     1  te1
.....................uuggcacuggaagaauucaU.......................................................................  5     1  te1
.....................uuggcacuggaagaauCcac.......................................................................  1     1  te1
.....................uuggcaUuggaagaauucac.......................................................................  1     1  te1
.....................Guggcacuggaagaauucac.......................................................................  3     1  te1
.....................uuggcacuggaUgaauucaca......................................................................  1     1  te1
.....................uuggcacuggaagUauucaca......................................................................  1     1  te1
.....................uuggGacuggaagaauucaca......................................................................  1     1  te1
.....................Auggcacuggaagaauucaca......................................................................  11    1  te1
.....................uuggcacuggaagaauucacU......................................................................  23    1  te1
.....................uuggcacuggaagaauucaca......................................................................  1270  0  te1
.....................uuggUacuggaagaauucaca......................................................................  3     1  te1
.....................uuggcacuggaagaauucUca......................................................................  1     1  te1
.....................Cuggcacuggaagaauucaca......................................................................  11    1  te1
.....................uuggcacuggaagaauuGaca......................................................................  1     1  te1
.....................uuggcacuggaagaauucacG......................................................................  7     1  te1
.....................Guggcacuggaagaauucaca......................................................................  1     1  te1
.....................uuggcacuggUagaauucaca......................................................................  1     1  te1
.....................uuggcaUuggaagaauucaca......................................................................  1     1  te1
.....................uuggcacGgaagaauucaca......................................................................  1     1  te1
.....................uuggcacuggaagaauucacaA.....................................................................  10    1  te1
.....................uuggcacuggaagaauucacCg.....................................................................  1     1  te1
.....................Guggcacuggaagaauucacag.....................................................................  1     1  te1
```

```
                    Mature                                  Star
acaauuggccuuaccuuauccuuggcacuggaagaauucacagaugcgauaagaaacacucgugguucgucuggugccaaagauaaacgcaagguauacacucugaacuuu
.....................uuggUacuggaagaauucacag....................................................................   1     1    te1
.....................uugAcacuggaagaauucacag....................................................................   1     1    te1
.....................Auggcacuggaagaauucacag....................................................................   4     1    te1
.....................uuggcacuggaagaauucacag....................................................................   437   0    te1
.....................uuggcacuggaagaauucacaU....................................................................   18    1    te1
.....................uuggcacuggaagaauucacGg....................................................................   1     1    te1
.....................uuggcacuggaagaauucaUag....................................................................   2     1    te1
.....................Cuggcacuggaagaauucacag....................................................................   2     1    te1
.....................uuggcacuggaagaauucUcag....................................................................   1     1    te1
.....................uuggcacuggaagaauucacaC....................................................................   5     1    te1
.....................uuggcacuggaagaauucacaga...................................................................   268   0    te1
.....................uuggcacuggaagaauucacagU...................................................................   6     1    te1
.....................Guggcacuggaagaauucacaga...................................................................   1     1    te1
.....................Auggcacuggaagaauucacaga...................................................................   1     1    te1
.....................uuggcacuAgaagaauucacaga...................................................................   1     1    te1
.....................uuggcacuggaagaauucacagau..................................................................   27    0    te1
.....................uuggcacuggaagaauucacagaA..................................................................   32    1    te1
.....................uuggcacuggaagaauucacagauU.................................................................   6     1    te1
......................uggcacuggaagaauuca.......................................................................   12    0    te1
......................uggcacuggaagaauucac......................................................................   66    0    te1
......................uggcacuggaagaauucaU......................................................................   1     1    te1
......................uggcacuggUagaauucaca.....................................................................   1     1    te1
......................Gggcacuggaagaauucaca.....................................................................   1     1    te1
......................uggcacuggaagaauucaca.....................................................................   56    0    te1
......................uggcaGuggaagaauucaca.....................................................................   1     1    te1
......................uggcacuggaaAaauucaca.....................................................................   1     1    te1
......................uggcacuggaagaauucacaA....................................................................   2     1    te1
......................uggcacuggaagaauucacaC....................................................................   1     1    te1
......................uggcacuggaagaauucacag....................................................................   20    0    te1
......................uggcacuggaagaauucacaU....................................................................   1     1    te1
......................uggcaGuggaagaauucacaga...................................................................   1     1    te1
......................uggcacuggaagaauucacaga...................................................................   16    0    te1
......................uggcacuggaagaauucacagaA..................................................................   1     1    te1
......................uggcacuggaagaauucacagau..................................................................   9     0    te1
......................uggcacuggaagCauucacagau..................................................................   1     1    te1
.......................ggUacuggaagaauucac......................................................................   1     1    te1
.......................ggcacuggaagaauucaA......................................................................   3     1    te1
.......................ggcacuggaagaauucac......................................................................   151   0    te1
.......................ggcacuggGagaauucac......................................................................   1     1    te1
.......................ggcacuggGagaauucaca.....................................................................   1     1    te1
.......................ggcacuggaagaauucacU.....................................................................   2     1    te1
.......................ggcacuggaagaauucaca.....................................................................   95    0    te1
.......................ggUacuggaagaauucaca.....................................................................   2     1    te1
.......................ggcacuggaagaauucacaC....................................................................   1     1    te1
.......................ggcacuggaagaauucacaA....................................................................   2     1    te1
.......................ggcacuggaagaauucacag....................................................................   26    0    te1
.......................ggcacuggaagaauucacaU....................................................................   2     1    te1
.......................ggGacuggaagaauucacaga...................................................................   1     1    te1
.......................ggcacuggaagaauucacaga...................................................................   9     0    te1
.......................ggcacuggaagaauucacagU...................................................................   2     1    te1
.......................ggcacuggaagaauucacGgau..................................................................   1     1    te1
.......................ggcacuggaagaauucacagaA..................................................................   2     1    te1
........................gcacuggaagaauucaca.....................................................................   8     0    te1
........................gcacuggaagaauucacU.....................................................................   1     1    te1
.........................cacuggaagaauucacag....................................................................   7     0    te1
.........................cacuggaagaauucacaU....................................................................   1     1    te1
.........................cacuggaagaauucacaA....................................................................   1     1    te1
.........................cacuggaagaauucacGga...................................................................   1     1    te1
.........................cacuggaagaauucacaga...................................................................   3     0    te1
.........................cacuggaagaauucacGgau..................................................................   1     1    te1
.........................cacuggaagaauucacagaugcga..............................................................   1     0    te1
..........................acuggaagaauucacGga...................................................................   1     1    te1
..........................acuggaagaauucacGgau..................................................................   2     1    te1
...........................cuggaagaauucacagUu..................................................................   1     1    te1
...........................cuggaagaauucacagau..................................................................   1     0    te1
............................................ugcgauaagaaacacucguggu.............................................   1     0    te1
............................................ugcgauaagaaacacucgugguu............................................   1     0    te1
............................................ugcgauaagaaacacucgugguucguc........................................   2     0    te1
.........................................................acucgugguucgucuggugccaU...............................   1     1    te1
..........................................................Uucgugguucgucuggugcca................................   1     1    te1
```

```
                    Mature                            Star
acaauuggccuuaccuuauccuuggcacuggaagaauucacagaugcgauaagaaacacucgugguucgucuggugccaaagauaaacgcaagguauacacucugaacuuu
..........................................................cucgugguucgucuggugcca................................   1     0    te1
..........................................................cucgugguucgucuggugccaaU..............................   1     1    te1
..........................................................cucgugguucgucuggugccaaa..............................   1     0    te1
...........................................................ucgugguucgucuggugcc.................................   3     0    te1
...........................................................ucgugguGcgucuggugcca................................   1     1    te1
...........................................................ucgugguCcgucuggugcca................................   1     1    te1
...........................................................ucgugguucguUuggugcca................................   1     1    te1
...........................................................ucgugguucgucuggugcca................................   54    0    te1
...........................................................ucgugguucgucuggugccU................................   3     1    te1
...........................................................ucgugguucgucuggugccaa...............................   2     0    te1
...........................................................ucgugguucgucuggugccaU...............................   3     1    te1
...........................................................ucgugguucgucuggugccaUa..............................   1     1    te1
...........................................................ucgugguucgucuggugccaaa..............................   6     0    te1
...........................................................ucgugguucgucuggugccaaU..............................   4     1    te1
...........................................................ucgugguucgucuggugccaaC..............................   1     1    te1
...........................................................ucgugguucgucuggugccaaGg.............................   13    1    te1
...........................................................ucgugguucgucuggugccaaaga............................   1     0    te1
............................................................cgugguucgucuggugccaU...............................   1     1    te1
............................................................cgugguucgucuggugccaaa..............................   1     0    te1
............................................................cgugguucgucuggugccaaGg.............................   3     1    te1
............................................................cgugguucgucuggugccaaagauU..........................   1     1    te1
.............................................................gugguucgucuggugcca................................   3     0    te1
.............................................................gugguucgucuggugccaa...............................   5     0    te1
.............................................................gugguucgucuggugccaUa..............................   2     1    te1
.............................................................gugguucgucuggugccaaG..............................   2     1    te1
.............................................................gugguucgucuggugccaaa..............................   68    0    te1
.............................................................gugguucgucuggugccaaaC.............................   1     1    te1
.............................................................gugguucgucuggugccaaUg.............................   1     1    te1
.............................................................gugguucgucuggugccaaag.............................   217   0    te1
.............................................................gugguucgucuggugccaaaA.............................   2     1    te1
.............................................................gugguucgucuggugccaaaU.............................   1     1    te1
.............................................................gugguucgucuggugccaaGg.............................   1395  1    te1
.............................................................gugguucgucuggugccaaCg.............................   1     1    te1
.............................................................gAgguucgucuggugccaaaga............................   1     1    te1
.............................................................gugguucgucuggugccaaagU............................   9     1    te1
.............................................................gugguucgucuggugUcaaaga............................   2     1    te1
.............................................................gugguucgucuggugccaaaga............................   496   0    te1
.............................................................gugguucgucuggugccaaGga............................   2     1    te1
.............................................................guggCucgucuggugccaaaga............................   1     1    te1
.............................................................gugguucgucuggugccaUaga............................   1     1    te1
.............................................................gugguucgucuggugccaaagC............................   7     1    te1
.............................................................gugguucgucuAgugccaaaga............................   1     1    te1
.............................................................gugguucgucuggugccaaagG............................   130   1    te1
.............................................................gugAuucgucuggugccaaaga............................   1     1    te1
.............................................................gugguucgucuggugccaaagCu...........................   1     1    te1
.............................................................gugguucgucuggugccaaagaA...........................   2     1    te1
.............................................................gugguucgucuggugccaaagau...........................   3     0    te1
.............................................................gugguucgucuggugccaaagaC...........................   1     1    te1
.............................................................gugguucgucuggugccaaagauU..........................   3     1    te1
.............................................................gugguucgucuggugccaaagaua..........................   3     0    te1
.............................................................gugguucgucuggugccaaagaGa..........................   1     1    te1
.............................................................gugguucgucuggugccaaagaAa..........................   4     1    te1
.............................................................gugguucgucuggugccaaagaCa..........................   6     1    te1
.............................................................Uugguucgucuggugccaaagaua..........................   1     1    te1
.............................................................gugguucgucuggugccaaagauaa.........................   1     0    te1
..............................................................ugguucgucuggugccaa...............................   2     0    te1
..............................................................ugguucgucuggugccaaU..............................   1     1    te1
..............................................................ugguucgucuggugccaaa..............................   3     0    te1
..............................................................ugguucgucuggugccaaag.............................   38    0    te1
..............................................................ugguucgucuggugccaaaA.............................   1     1    te1
..............................................................ugguucgucuggugccaaGg.............................   294   1    te1
..............................................................ugguucgucuggugccCaag.............................   1     1    te1
..............................................................ugguucgucuggugccaaaga............................   140   0    te1
..............................................................ugguucgucuggugccaaagG............................   33    1    te1
..............................................................Ggguucgucuggugccaaaga............................   1     1    te1
..............................................................ugguucgucuggugccaaagU............................   4     1    te1
..............................................................ugguucgucuggugccaaagau...........................   21    0    te1
..............................................................ugguucgucuggugccaaagaA...........................   7     1    te1
..............................................................ugguucgucuggugccaaagaG...........................   1     1    te1
..............................................................ugguucgucuggugccaaagaua..........................   22    0    te1
```

Mature **Star**

```
acaauuggccuuaccuuauccuuggcacuggaagaauucacagaugcgauaagaaacacucgugguucgucuggugccaaagauaaacgcaagguauacacucugaacuuu
..............................................................ugguucgucuggugccaaagaCa..........................  3     1  te1
..............................................................ugguucgucuggugccaaagauG..........................  12    1  te1
..............................................................ugguucgucuggugccaaagaAa..........................  7     1  te1
..............................................................ugguucgucuggugccaaagauU..........................  44    1  te1
...............................................................gguucgucuggugccaaGg.............................  1     1  te1
...............................................................gguucgucuggugccaaagG............................  1     1  te1
................................................................guucgucuggugccaaGg.............................  1     1  te1
.................................................................uucgucuggugccaaagaAa..........................  1     1  te1
...................................................................cgucuggugccaaagaGa..........................  1     1  te1
...................................................................................uaaacgcaagguauacacucug......  1     0  te1

.caauuggccuuaccuuauc...........................................................................................  1     0  T53
..................uUcuuggcacuggaagaauuc........................................................................  1     1  T53
..................uUcuuggcacuggaagaauucac......................................................................  3     1  T53
...................ccuuggcacuggaagaauu.........................................................................  3     0  T53
...................ccuuggcacuggaagaauuc........................................................................  55    0  T53
...................Ucuuggcacuggaagaauuc........................................................................  1     1  T53
...................Acuuggcacuggaagaauuc........................................................................  1     1  T53
...................ccuuggcacuggaagaauuca.......................................................................  31    0  T53
...................ccuuggcacuggaagaauucC.......................................................................  2     1  T53
...................ccuuggcacuggaagaauCca.......................................................................  1     1  T53
...................cUuuggcacuggaagaauucac......................................................................  2     1  T53
...................Ucuuggcacuggaagaauucac......................................................................  3     1  T53
...................ccuuggcacuggaagaauucac......................................................................  12    0  T53
...................ccuuggcacuggaagaauucaA......................................................................  1     1  T53
...................Ucuuggcacuggaagaauucaca.....................................................................  4     1  T53
....................cuuggcacuggaagaauC.........................................................................  1     1  T53
....................cuuggcacuggUagaauu.........................................................................  1     1  T53
....................cuuggcacuggaagaaAu.........................................................................  1     1  T53
....................cuuggcGcuggaagaauu.........................................................................  1     1  T53
....................cuuggcacuggaagaauu.........................................................................  198   0  T53
....................cuuggUacuggaagaauu.........................................................................  1     1  T53
....................cuuggcacuggCagaauuc........................................................................  1     1  T53
....................cuuAgcacuggaagaauuc........................................................................  2     1  T53
....................cuuggcGcuggaagaauuc........................................................................  1     1  T53
....................cuuggAacuggaagaauuc........................................................................  1     1  T53
....................cuuggcacuggaagUauuc........................................................................  13    1  T53
....................cuuggcacuggaagCauuc........................................................................  2     1  T53
....................cuuggcacuggaagaauuG........................................................................  1     1  T53
....................cuuggcacuggaagaUuuc........................................................................  4     1  T53
....................cuuggGacuggaagaauuc........................................................................  2     1  T53
....................cuuggcacuAgaagaauuc........................................................................  1     1  T53
....................cuuggcacuggaGgaauuc........................................................................  1     1  T53
....................cuuggcacugAaagaauuc........................................................................  2     1  T53
....................cuuggcacuggaUgaauuc........................................................................  1     1  T53
....................cuugCcacuggaagaauuc........................................................................  1     1  T53
....................cuuggcacuggaagaauAc........................................................................  2     1  T53
....................cuuggcacuggUagaauuc........................................................................  6     1  T53
....................cuuggcacuggaaAaauuc........................................................................  2     1  T53
....................cuAggcacuggaagaauuc........................................................................  1     1  T53
....................cuuggUacuggaagaauuc........................................................................  11    1  T53
....................cuuggcacuggaagGauuc........................................................................  1     1  T53
....................cuCggcacuggaagaauuc........................................................................  2     1  T53
....................cuuggcacuggaagaauuU........................................................................  22    1  T53
....................cuuggcacuggGagaauuc........................................................................  8     1  T53
....................cuuggcacuCgaagaauuc........................................................................  1     1  T53
....................cuuggcacuggaagaauuA........................................................................  135   1  T53
....................cuugAcacuggaagaauuc........................................................................  1     1  T53
....................Uuuggcacuggaagaauuc........................................................................  3     1  T53
....................cuuggcaUuggaagaauuc........................................................................  1     1  T53
....................cuuggcacuggaagaaAuc........................................................................  8     1  T53
....................cuuggcacuggaagaauuc........................................................................  8080  0  T53
....................cuuggcacuggaaUaauuc........................................................................  2     1  T53
....................cCuggcacuggaagaauuc........................................................................  1     1  T53
....................cuuggcacAggaagaauuc........................................................................  5     1  T53
....................cuuggcacugCaagaauuc........................................................................  3     1  T53
....................cuuggcacuggaagUauuca.......................................................................  12    1  T53
....................cuuggcacuggaagaaAuca.......................................................................  6     1  T53
....................cuuggcGcuggaagaauuca.......................................................................  1     1  T53
....................cuuggcacuggaaUaauuca.......................................................................  2     1  T53
```

```
                    Mature                                   Star
acaauuggccuuaccuuauccuuggcacuggaagaauucacagaugcgauaagaaacacucgugguucgucuggugccaaagauaaacgcaagguauacacucugaacuuu
....................cuuggcaUuggaagaauuca.......................................................................    1     1    T53
....................cuugCcacuggaagaauuca.......................................................................    1     1    T53
....................cuuggcacuggUagaauuca.......................................................................    8     1    T53
....................cuuggcacuggaagaauAca.......................................................................    3     1    T53
....................cuuggUacuggaagaauuca.......................................................................    10    1    T53
....................Guuggcacuggaagaauuca.......................................................................    1     1    T53
....................cuuggGacuggaagaauuca.......................................................................    1     1    T53
....................cuugAcacuggaagaauuca.......................................................................    2     1    T53
....................cuuggcacuggaagaauucC.......................................................................    1     1    T53
....................cuuggcacuggaagaauGca.......................................................................    2     1    T53
....................cuuggcacAggaagaauuca.......................................................................    6     1    T53
....................Uuuggcacuggaagaauuca.......................................................................    2     1    T53
....................cuuggcacugUaagaauuca.......................................................................    3     1    T53
....................Auuggcacuggaagaauuca.......................................................................    1     1    T53
....................cuuggcacuggaaAaauuca.......................................................................    1     1    T53
....................cuuggcacugCaagaauuca.......................................................................    2     1    T53
....................cuuggcaGuggaagaauuca.......................................................................    1     1    T53
....................cuuAgcacuggaagaauuca.......................................................................    1     1    T53
....................cGuggcacuggaagaauuca.......................................................................    1     1    T53
....................cuuggcacuggaagaGuuca.......................................................................    1     1    T53
....................cuuggcacuggCagaauuca.......................................................................    1     1    T53
....................cuuggcacuCgaagaauuca.......................................................................    3     1    T53
....................cuuggcacuggaagaauuca.......................................................................    6931  0    T53
....................cuuggcacuUgaagaauuca.......................................................................    1     1    T53
....................cuuggcacuggaagaaGuca.......................................................................    1     1    T53
....................cuuggcacuggaagaauuAa.......................................................................    51    1    T53
....................cuuggcacuggaagaauCca.......................................................................    1     1    T53
....................cuuggcacuggaagaauucU.......................................................................    28    1    T53
....................cuuggcacuggGagaauuca.......................................................................    4     1    T53
....................cuAggcacuggaagaauuca.......................................................................    1     1    T53
....................cuuggAacuggaagaauuca.......................................................................    1     1    T53
....................cuuggcacuggaGgaauuca.......................................................................    1     1    T53
....................cuuggcacuAgaagaauuca.......................................................................    3     1    T53
....................cCuggcacuggaagaauuca.......................................................................    2     1    T53
....................cuuggcacuggaUgaauuca.......................................................................    2     1    T53
....................cuuggcacuggaagaauuUa.......................................................................    6     1    T53
....................cuuggcacuggaagCauuca.......................................................................    1     1    T53
....................cuuggcacuggaagaauucG.......................................................................    16    1    T53
....................cuuggcacuggaagGauuca.......................................................................    7     1    T53
....................cuuggcacuggaagaauCcac......................................................................    1     1    T53
....................cuuggcacuggaagaaGucac......................................................................    1     1    T53
....................cuuggcacuggaagaGuucac......................................................................    2     1    T53
....................Auuggcacuggaagaauucac......................................................................    1     1    T53
....................cuuggcacuggaagaauuUac......................................................................    3     1    T53
....................cuuggcacuggaagaauucaA......................................................................    696   1    T53
....................cuCggcacuggaagaauucac......................................................................    2     1    T53
....................cuuggcacuggaagaauucaU......................................................................    100   1    T53
....................Uuuggcacuggaagaauucac......................................................................    1     1    T53
....................cuuggAacuggaagaauucac......................................................................    2     1    T53
....................cuuggcacugCaagaauucac......................................................................    6     1    T53
....................cuuggcacuCgaagaauucac......................................................................    5     1    T53
....................cuuAgcacuggaagaauucac......................................................................    1     1    T53
....................cuuggcacuggaagaauucac......................................................................    9866  0    T53
....................cCuggcacuggaagaauucac......................................................................    2     1    T53
....................cuuggcacugAaagaauucac......................................................................    3     1    T53
....................cuuggcacuggaagaauucaG......................................................................    3     1    T53
....................cuuggcacuggaagaaAucac......................................................................    7     1    T53
....................cuuggcaGuggaagaauucac......................................................................    1     1    T53
....................cuuggcacuggaagaauuGac......................................................................    1     1    T53
....................cuuggcacuggaagGauucac......................................................................    2     1    T53
....................cuuggcacuggaagaUuucac......................................................................    2     1    T53
....................cuuggcacuggaagaaCucac......................................................................    2     1    T53
....................cuuggcaUuggaagaauucac......................................................................    2     1    T53
....................cuuggcacuggaagaauucUc......................................................................    4     1    T53
....................cuuggcacuggaagaauAcac......................................................................    1     1    T53
....................cuugAcacuggaagaauucac......................................................................    4     1    T53
....................cuuggcacuggUagaauucac......................................................................    16    1    T53
....................cuuggcacuggaaAaauucac......................................................................    1     1    T53
....................cuugUcacuggaagaauucac......................................................................    1     1    T53
....................cuuUgcacuggaagaauucac......................................................................    1     1    T53
```

Mature **Star**

```
acaauuggccuuaccuuauccuuggcacuggaagaauucacagaugcgauaaagaaacacucgugguucgucuggugccaaagauaaacgcaagguauacacucugaacuuu
....................cuuggcacuggaaUaauucac.......................................................................  2     1  T53
....................cuuggcacuggaagUauucac.......................................................................  16    1  T53
....................cuuggcacuggaagaauGcac.......................................................................  2     1  T53
....................cuuggUacuggaagaauucac.......................................................................  12    1  T53
....................cuuggcacuggaagaauuAac.......................................................................  3     1  T53
....................cuuggcacAggaagaauucac.......................................................................  11    1  T53
....................cuuggcacuggGagaauucac.......................................................................  6     1  T53
....................cuuggcaUuggaagaauucaca......................................................................  1     1  T53
....................Uuuggcacuggaagaauucaca......................................................................  1     1  T53
....................cuuggcacuggGagaauucaca......................................................................  8     1  T53
....................cuuggcacuggaagaauucaAa......................................................................  69    1  T53
....................cCuggcacuggaagaauucaca......................................................................  2     1  T53
....................cuuggcacuggaagUauucaca......................................................................  9     1  T53
....................cuuggGacuggaagaauucaca......................................................................  1     1  T53
....................cuuggcacuggUagaauucaca......................................................................  8     1  T53
....................cuuggcacuggaagaauuUaca......................................................................  1     1  T53
....................cuuggcacuggaagaauGcaca......................................................................  2     1  T53
....................cuuggcacuggaagaUuucaca......................................................................  2     1  T53
....................cuuggcacuggaagaaAucaca......................................................................  1     1  T53
....................cuugAcacuggaagaauucaca......................................................................  1     1  T53
....................Auuggcacuggaagaauucaca......................................................................  1     1  T53
....................cuuggcacuggaGgaauucaca......................................................................  1     1  T53
....................cuuggcacuggCagaauucaca......................................................................  2     1  T53
....................cuuggcacuggaagaauucacC......................................................................  8     1  T53
....................cGuggcacuggaagaauucaca......................................................................  1     1  T53
....................cuuggcacuggaAaauucaca.......................................................................  1     1  T53
....................cuuggcacGggaagaauucaca......................................................................  1     1  T53
....................cuuggcacuggaagaauucacG......................................................................  12    1  T53
....................cuuggcacuggaaUaauucaca......................................................................  4     1  T53
....................cuuggcacugAaagaauucaca......................................................................  1     1  T53
....................cuuggcUcuggaagaauucaca......................................................................  2     1  T53
....................cuuggUacuggaagaauucaca......................................................................  12    1  T53
....................cuuggcacuggaagaauucaGa......................................................................  3     1  T53
....................cuuggcacuggaagaauucaca......................................................................  5194  0  T53
....................cuuggcacuggaagaauucaUa......................................................................  3     1  T53
....................cuuggcacuggaagaauCcaca......................................................................  1     1  T53
....................cuuggcacuggaagGauucaca......................................................................  2     1  T53
....................cuuggcacuggaagaauucacU......................................................................  347   1  T53
....................cuuggcacuCgaagaauucaca......................................................................  2     1  T53
....................cuuggAacuggaagaauucaca......................................................................  1     1  T53
....................cuuUgcacuggaagaauucaca......................................................................  1     1  T53
....................cuuggcacAggaagaauucaca......................................................................  1     1  T53
....................cuuggcacAggaagaauucacag.....................................................................  1     1  T53
....................cuuUgcacuggaagaauucacag.....................................................................  1     1  T53
....................cuuggcacuggaagaauAcacag.....................................................................  2     1  T53
....................cuuggcacuggUagaauucacag.....................................................................  3     1  T53
....................cuuggcacuggaagaauucacag.....................................................................  1202  0  T53
....................cuuggcaUuggaagaauucacag.....................................................................  1     1  T53
....................cuuggcacuggaagUauucacag.....................................................................  4     1  T53
....................cuuggcacuggaagaaAucacag.....................................................................  1     1  T53
....................cuuggcacuggaagaauucacaA.....................................................................  85    1  T53
....................Auuggcacuggaagaauucacag.....................................................................  1     1  T53
....................cuuggcacuggaagaauucacaC.....................................................................  61    1  T53
....................cuuggcacuggaagaauGcacag.....................................................................  1     1  T53
....................cuuggcacuggaagaauucacaU.....................................................................  115   1  T53
....................cuuggcacAggaagaauucacaga....................................................................  1     1  T53
....................cuuggcacuggaagUauucacaga....................................................................  2     1  T53
....................cuuggUacuggaagaauucacaga....................................................................  2     1  T53
....................cuuggcacuggaagaauucacaAa....................................................................  2     1  T53
....................cuuggcacuggaagCauucacaga....................................................................  1     1  T53
....................cuuggcacuggaagaauucacaga....................................................................  631   0  T53
....................cuuggcacuggaagaauucacaCa....................................................................  1     1  T53
....................cuuggcacuggaagaauucacaUa....................................................................  1     1  T53
....................cuugAcacuggaagaauucacaga....................................................................  1     1  T53
....................cuuggcacuggaagaaAucacaga....................................................................  1     1  T53
....................cuuggcacuggaagaauucacUga....................................................................  1     1  T53
....................cuuggcacuggaagaauucacagU....................................................................  26    1  T53
....................cuuggcacuCgaagaauucacaga....................................................................  1     1  T53
....................cuuggcacuggaagaauucacagaA...................................................................  32    1  T53
....................cuuggcacuggaagaauucacagUu...................................................................  4     1  T53
```

Mature / Star

```
acaauuggccuuaccuuauccuuggcacuggaagaauucacagaugcgauaaagaaacacucgugguucgucuggugccaaagauaaacgcaagguauacacucugaacuuu
....................cuuggcacuggaagaauucacaCau...................................................................	1	1	T53
....................cuuggcacuggaagaauucacagau...................................................................	47	0	T53
....................cuuggcacuggaagaauucacagaC...................................................................	1	1	T53
....................cuuggcacuggaagaauucacagauA..................................................................	1	1	T53
....................cuuggcacuggaagaauucacagauU..................................................................	8	1	T53
.....................uuggcacuggaagaauuA.........................................................................	2	1	T53
.....................uuggcacuggaagaauuc.........................................................................	138	0	T53
.....................uuggUacuggaagaauuc.........................................................................	1	1	T53
.....................uuggcacuggaagaauuU.........................................................................	1	1	T53
.....................uuggcacuggaagaauuAa........................................................................	1	1	T53
.....................uuggcacuggaagaauuca........................................................................	133	0	T53
.....................uuggUacuggaagaauuca........................................................................	1	1	T53
.....................uuggUacuggaagaauucac.......................................................................	1	1	T53
.....................uugAcacuggaagaauucac.......................................................................	1	1	T53
.....................uuggcacuggaagaauucac.......................................................................	168	0	T53
.....................uuggcacuggaagaauucacU......................................................................	3	1	T53
.....................uuggcacuggaagaauucacC......................................................................	1	1	T53
.....................uuggcacuggaagaauucaca......................................................................	134	0	T53
.....................uuggcacuggaagaauucacaU.....................................................................	3	1	T53
.....................uuggcacuggaagaaAucacag.....................................................................	1	1	T53
.....................uuggcacuggaagaauucacag.....................................................................	33	0	T53
.....................uuggcacuggaagaauucacaga....................................................................	15	0	T53
.....................uuggcacuggaagaauucacagC....................................................................	2	1	T53
.....................uuggcacuggaagaauucacagU....................................................................	2	1	T53
.....................uuggcacuggaagaauucacagau...................................................................	1	0	T53
.....................uuggcacuggaagaauucacagauA..................................................................	1	1	T53
......................uggcacuggaagaauuca........................................................................	3	0	T53
......................uggcacuggaagaauucac.......................................................................	7	0	T53
......................uggcacuggaagaauucaca......................................................................	2	0	T53
......................uggcacuggaagaauucacag.....................................................................	1	0	T53
.......................ggcacuggaagaauucac.......................................................................	13	0	T53
.......................ggcacuggaagaauucaA.......................................................................	1	1	T53
.......................ggcacuggaagaauucaU.......................................................................	1	1	T53
.......................ggcacuggaagaauucacU......................................................................	2	1	T53
.......................ggcacuggaagaaAucaca......................................................................	1	1	T53
.......................ggcacuggaagaauucaca......................................................................	3	0	T53
.......................ggcacuggaagaauucacag.....................................................................	1	0	T53
.........................cacuggaagaauucacaga....................................................................	2	0	T53
...........................................................cucgugguucgucuggugcc.................................	2	0	T53
............................................................ucgugguucgucuggugcc.................................	3	0	T53
............................................................ucgugguucgucuggugcca................................	3	0	T53
............................................................ucgugguucgucuggugccU................................	1	1	T53
............................................................ucgugguucgucuggugccaa...............................	3	0	T53
............................................................ucgugguucgucuggugccaaU..............................	2	1	T53
............................................................ucgugguucgucuggugccaaa..............................	5	0	T53
............................................................ucgugguucgucuggugccaaGg.............................	1	1	T53
............................................................ucgugguucgucuggugccaaaU.............................	1	1	T53
..............................................................gugguucgucuggugccaa...............................	2	0	T53
..............................................................gugguucgucuggugccaaa..............................	10	0	T53
..............................................................gugguucgucuggugccaaag.............................	37	0	T53
..............................................................gugguucgucuggugccaaGg.............................	5	1	T53
..............................................................gugguucgucuggAgccaaag.............................	1	1	T53
..............................................................gugguucgucuggugccaaaU.............................	2	1	T53
..............................................................gugguucgucuggugccaaGga............................	2	1	T53
..............................................................gugguucgucuggugccaaaga............................	107	0	T53
..............................................................gugguucguGuggugccaaaga............................	1	1	T53
..............................................................gugguucgucuggugccaaagU............................	2	1	T53
..............................................................gugguucgucuggugccaaagC............................	1	1	T53
..............................................................gugguucgucuUgugccaaaga............................	1	1	T53
..............................................................gugguucgucuggugccaaagau...........................	1	0	T53
..............................................................gugguucgucuggugccaaagaC...........................	1	1	T53
..............................................................gugguucgucuggugccaaagaCa..........................	2	1	T53
..............................................................gugguucgucuggugccaaagaua..........................	2	0	T53
..............................................................gugguucgucuggugccaaagauC..........................	1	1	T53
...............................................................ugguucgucuggugccaUa..............................	1	1	T53
...............................................................ugguucgucuggugccaaU..............................	6	1	T53
...............................................................ugguucgucuggugccaaa..............................	10	0	T53
...............................................................ugguucgucuggugccaaaA.............................	1	1	T53
...............................................................ugguucgucuggugccaaag.............................	12	0	T53
...............................................................ugguucgucuggugccaaaga............................	38	0	T53
```

Mature / Star			
acaauuggccuuaccuuauccuuggcacuggaagaauucacagaugcgauaagaaacacucgugguucgucuggugccaaagauaaacgcaagguauacacucugaacuuu			
....ugguucgucuggugccaaagU............................	2	1	T53
....ugguucgucuggugccaaagaC...........................	1	1	T53
....ugguucgucuggAgccaaagau...........................	1	1	T53
....ugguucgucuggugccaaagaA...........................	2	1	T53
....ugguucgucuggugccaaagau...........................	11	0	T53
....ugguucgucuggugccaaagauU..........................	19	1	T53
....ugguucgucuggugccaaagaAa..........................	1	1	T53
....ugguucgucuggugccaaagaua..........................	10	0	T53
....ugguucgucuggugccaaagauG..........................	4	1	T53

Provisional ID : Scaffold_1043_2941
Score total : 165764.5
Score for star read(s) : 3.9
Score for read counts : 165754
Score for mfe : 2.1
Score for randfold : 1.6
Score for cons. seed : 3
Total read count : 325131
Mature read count : 323745
Loop read count : 0
Star read count : 1386

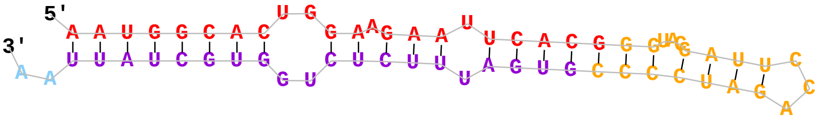

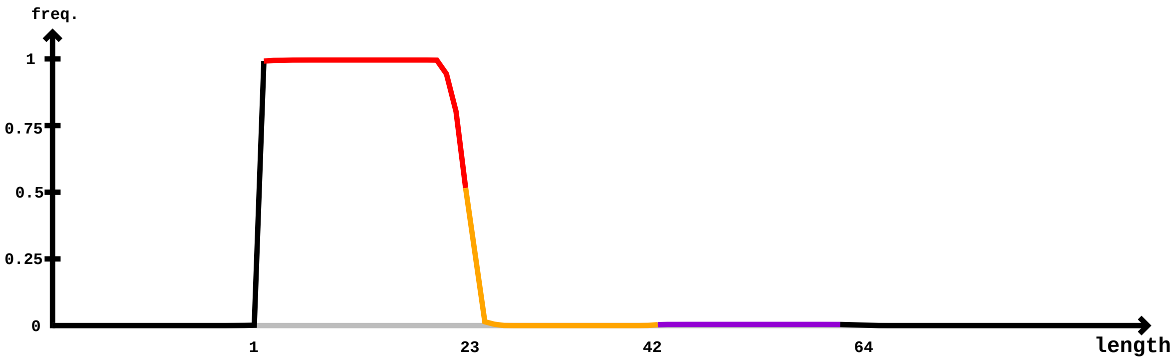

Mature Star

```
5'- gccuuuuccuggugacacuuaauggcacuggaagaauucacgggguacgauuccagauccccgugauuucucuggugcuauuaagaguugcugugggacaaaucugcuuuaaa   -3'   obs
    gccuuuuccuggugacacuuaauggcacuggaagaauucacgggguacgauuccagauccccgugauuucucuggugcuauuaagaguugcugugggacaaaucugcuuuaaa         exp
    .....((((((..((.(((((((((((..((.(((.(((((((...((((...))))))))))).)))))..))))))))))).))..))).))))................   reads   mm   sample
    ..cuuuuccuggugacacuu............................................................................................   1       0    T63
    .................cuuaauggcacuggaagaauuc.........................................................................   1       0    T63
    .................cuuaauggcacuggaagaauucacg......................................................................   1       0    T63
    ..................uuaauggcacuggaagaauuc.........................................................................   3       0    T63
    ..................uuaauggcacuggaagaauucac.......................................................................   2       0    T63
    ..................uAaauggcacuggaagaauucac.......................................................................   1       1    T63
    ..................uuaauggcacuggaagaauucacg......................................................................   2       0    T63
    ...................Aaauggcacuggaagaauuc.........................................................................   1       1    T63
    ...................uaauggcacuggaagaauuc.........................................................................   3       0    T63
    ...................Gaauggcacuggaagaauucac.......................................................................   1       1    T63
    ...................uaauggcacuggaagaauucac.......................................................................   3       0    T63
    ...................uaauggcacuggaagaauucacgg.....................................................................   3       0    T63
    ....................aauggcacuggaagaauu..........................................................................   48      0    T63
    ....................aauggcacuggaagaauuU.........................................................................   5       1    T63
    ....................aauggcacAggaagaauuc.........................................................................   2       1    T63
    ....................aaugAcacuggaagaauuc.........................................................................   1       1    T63
    ....................aauggcaUuggaagaauuc.........................................................................   2       1    T63
    ....................aauggcacuggaagUauuc.........................................................................   3       1    T63
    ....................aauggAacuggaagaauuc.........................................................................   2       1    T63
    ....................aauggcacuggUagaauuc.........................................................................   4       1    T63
    ....................aauggcacuCgaagaauuc.........................................................................   2       1    T63
    ....................aauggcacuggaagaauuA.........................................................................   8       1    T63
    ....................aauggcacuggGagaauuc.........................................................................   1       1    T63
    ....................aauggcacuggaaUaauuc.........................................................................   2       1    T63
    ....................aauggcacuggaagaCuuc.........................................................................   1       1    T63
    ....................aauggcacuggaaAaauuc.........................................................................   2       1    T63
    ....................aauggcacugAaagaauuc.........................................................................   1       1    T63
    ....................aGuggcacuggaagaauuc.........................................................................   3       1    T63
    ....................aauggcacuggaagaaAuc.........................................................................   3       1    T63
    ....................aauggcacuggaagaauuc.........................................................................   3765    0    T63
    ....................aauUgcacuggaagaauuc.........................................................................   1       1    T63
    ....................aauggUacuggaagaauuc.........................................................................   6       1    T63
    ....................aauggcacuggaagCauuc.........................................................................   2       1    T63
```

```
                    Mature                                  Star
gccuuuuccuggugacacuuaauggcacuggaagaauucacggguacgauuccagauccccgugauuucucuggugcuauuaagaguugcugugggacaaaucugcuuuaaa
....................aauggcacuggaaCaauuc.........................................................................   1     1   T63
....................aauggcacuggaagaauucC........................................................................   1     1   T63
....................aauggcacuggaagaauGca........................................................................   1     1   T63
....................aauggcacuggaagaauuAa........................................................................   2     1   T63
....................aauggcacuggaagCauuca........................................................................   1     1   T63
....................aauggcUcuggaagaauuca........................................................................   1     1   T63
....................aauggcacuggaagaauucG........................................................................   4     1   T63
....................aauggcacuggaagaauucU........................................................................   20    1   T63
....................aauggcacuggaagaaAuca........................................................................   2     1   T63
....................aauggcacuggaGgaauuca........................................................................   1     1   T63
....................aaCggcacuggaagaauuca........................................................................   1     1   T63
....................aauggcacuggaagaauuca........................................................................   3259  0   T63
....................aauggcacuggCagaauuca........................................................................   1     1   T63
....................aauggcacuggaagUauuca........................................................................   9     1   T63
....................aGuggcacuggaagaauuca........................................................................   1     1   T63
....................aaugAcacuggaagaauuca........................................................................   1     1   T63
....................aauggcacuggaUgaauuca........................................................................   2     1   T63
....................aauggcacuggaagaUuuca........................................................................   1     1   T63
....................aauggcacuggaagaCuuca........................................................................   2     1   T63
....................aauggcacuggaagaauuUa........................................................................   1     1   T63
....................aauggcacuggGagaauuca........................................................................   2     1   T63
....................aauggcacuggaagGauuca........................................................................   2     1   T63
....................aauggcacuggUagaauuca........................................................................   4     1   T63
....................aaugUcacuggaagaauuca........................................................................   1     1   T63
....................aauggUacuggaagaauuca........................................................................   1     1   T63
....................aUuggcacuggaagaauuca........................................................................   2     1   T63
....................aauggcacugCaagaauucac.......................................................................   1     1   T63
....................aauggcacuggaagaauuUac.......................................................................   13    1   T63
....................aauggcacAggaagaauucac.......................................................................   2     1   T63
....................aauggcacuggUagaauucac.......................................................................   4     1   T63
....................aauggAacuggaagaauucac.......................................................................   2     1   T63
....................aaugAcacuggaagaauucac.......................................................................   3     1   T63
....................aauggcacCggaagaauucac.......................................................................   2     1   T63
....................aauggcacuggaagGauucac.......................................................................   2     1   T63
....................aauggcacuggaagUauucac.......................................................................   13    1   T63
....................aauggcacuggaagaauucaA.......................................................................   28    1   T63
....................aauggcacuggaagaauAcac.......................................................................   2     1   T63
....................aCuggcacuggaagaauucac.......................................................................   1     1   T63
....................aauggcacuCgaagaauucac.......................................................................   2     1   T63
....................aauggcacugAaagaauucac.......................................................................   1     1   T63
....................aauggcacuAgaagaauucac.......................................................................   2     1   T63
....................aauggcacuggaagaaCucac.......................................................................   1     1   T63
....................aauggcacuggaagaauucaU.......................................................................   12    1   T63
....................aauggUacuggaagaauucac.......................................................................   11    1   T63
....................aaCggcacuggaagaauucac.......................................................................   2     1   T63
....................aauggcacuggaagaauucac.......................................................................   6617  0   T63
....................aauggcacuggaagaauCcac.......................................................................   1     1   T63
....................aauggcacuggaagCauucac.......................................................................   1     1   T63
....................aauggcacuggaaCaauucac.......................................................................   2     1   T63
....................aUuggcacuggaagaauucac.......................................................................   1     1   T63
....................aGuggcacuggaagaauucac.......................................................................   1     1   T63
....................aauggcacuggGagaauucac.......................................................................   2     1   T63
....................aauggcacugUaagaauucac.......................................................................   1     1   T63
....................aauggcacuggaaAaauucac.......................................................................   1     1   T63
....................aauggcacuggaaUaauucac.......................................................................   1     1   T63
....................aauggcacuggaUgaauucac.......................................................................   1     1   T63
....................aauggcacuggaagaaAucac.......................................................................   3     1   T63
....................aauggcaUuggaagaauucac.......................................................................   4     1   T63
....................aauUgcacuggaagaauucacg......................................................................   1     1   T63
....................aauggcacuggaaAaauucacg......................................................................   1     1   T63
....................aauggcacugAaagaauucacg......................................................................   4     1   T63
....................aaCggcacuggaagaauucacg......................................................................   1     1   T63
....................aauggcacuggaagGauucacg......................................................................   2     1   T63
....................aauggcacuggaagaauucacA......................................................................   245   1   T63
....................aauggcacuggaagaauucacg......................................................................   4290  0   T63
....................aauggcacuggaaCaauucacg......................................................................   2     1   T63
....................Gauggcacuggaagaauucacg......................................................................   1     1   T63
....................aauggcacuggaagCauucacg......................................................................   1     1   T63
....................aauggcacuCgaagaauucacg......................................................................   4     1   T63
....................aauggcacuggUagaauucacg......................................................................   1     1   T63
```

Mature **Star**

```
gccuuuuccuggugacacuuaauggcacuggaagaauucacggguacgauuccagauccccgugauuucucuggugcuauuaagaguugcugugggacaaaucugcuuuaaa
....................aauggcacuggaGgaauucacg......................................................................   1     1   T63
....................aauggcacuggaagUauucacg......................................................................   7     1   T63
....................aauggcacuggaagaauucacC......................................................................   5     1   T63
....................aauggAacuggaagaauucacg......................................................................   1     1   T63
....................aauggcacuggaagaauucaUg......................................................................   6     1   T63
....................aauggcacuggaagaauuUacg......................................................................   3     1   T63
....................aauggcacuggaagaaAucacg......................................................................   1     1   T63
....................aauggcacuggaaUaauucacg......................................................................   1     1   T63
....................aauggUacuggaagaauucacg......................................................................   4     1   T63
....................aauggcacCggaagaauucacg......................................................................   2     1   T63
....................aauggcacAggaagaauucacg......................................................................   1     1   T63
....................aUuggcacuggaagaauucacg......................................................................   1     1   T63
....................aCuggcacuggaagaauucacg......................................................................   1     1   T63
....................aauggcaUuggaagaauucacg......................................................................   2     1   T63
....................aauggcacuggGagaauucacg......................................................................   1     1   T63
....................aauggcacuggaagaUuucacg......................................................................   2     1   T63
....................aauggcacuggaagaauuGacg......................................................................   1     1   T63
....................aGuggcacuggaagaauucacg......................................................................   3     1   T63
....................aauggcacuggaagaauucacU......................................................................   111   1   T63
....................aauggcacugAaagaauucacgg.....................................................................   2     1   T63
....................aCuggcacuggaagaauucacgg.....................................................................   1     1   T63
....................aauggcacuggaagaauucacAg.....................................................................   1     1   T63
....................aauggcacuggaagGauucacgg.....................................................................   1     1   T63
....................aauggUacuggaagaauucacgg.....................................................................   1     1   T63
....................aauggcacuggaagaauucacUg.....................................................................   1     1   T63
....................aaugUcacuggaagaauucacgg.....................................................................   2     1   T63
....................aauggcacuggaagaauucGcgg.....................................................................   1     1   T63
....................aauggcacuggaagaauucaUgg.....................................................................   1     1   T63
....................aauggcacuggaagaauCcacgg.....................................................................   1     1   T63
....................aauggcacAggaagaauucacgg.....................................................................   1     1   T63
....................aauggcacuggaagaauucacgA.....................................................................   14    1   T63
....................aauggcacuggaagaauucacgU.....................................................................   16    1   T63
....................aauggcacuggaagaauucacgC.....................................................................   6     1   T63
....................aauggcacuggUagaauucacgg.....................................................................   1     1   T63
....................aauggcacuggaagaauucacgg.....................................................................   767   0   T63
....................aauggcacuggaagaauucacggg....................................................................   17    0   T63
....................aauggcacuggaagaauucacggU....................................................................   9     1   T63
....................aauggcacuggaagaauucacggA....................................................................   13    1   T63
....................aauggcacuggaagaauucacggUu...................................................................   4     1   T63
....................aauggcacuggaagaauucacgggu...................................................................   8     0   T63
....................aauggcacuggaagaauucacgggA...................................................................   6     1   T63
....................aauggcacuggaagaauucacggAu...................................................................   25    1   T63
....................aauggcacuggaagaauucacggguU..................................................................   2     1   T63
.....................auggcacuggaagaauuc.........................................................................   1     0   T63
.....................auggcacuggUagaauuca........................................................................   2     1   T63
.....................Cuggcacuggaagaauucac.......................................................................   1     1   T63
.....................auggcacuggaagaauuUac.......................................................................   1     1   T63
.....................auggcacuggaagaauucac.......................................................................   5     0   T63
.....................auggcacuggaagaauucacg......................................................................   12    0   T63
.....................auggcacuggaagaauucacgg.....................................................................   4     0   T63
.....................auggcacuggaagaauucacggg....................................................................   1     0   T63
.....................auggcacuggaagaauucacgggu...................................................................   1     0   T63
.....................auggcacuggaagaauucacggAu...................................................................   1     1   T63
......................uggcacuggaagaauucac.......................................................................   5     0   T63
......................uggcacuggaagaauucacgggA...................................................................   2     1   T63
.......................ggcacuggaagaauucac.......................................................................   12    0   T63
.......................ggcacuggaagaauucaA.......................................................................   1     1   T63
.......................ggcacuggaagaauucacg......................................................................   1     0   T63
........................gcacuggaagaauucacU......................................................................   1     1   T63
........................gcacuggaagaauucacg......................................................................   3     0   T63
............................................................cgugauuucucuggugcuau................................   1     0   T63
............................................................cgugauuucucuggugcuauu...............................   2     0   T63
............................................................cgugauuucucuggugcuauua..............................   2     0   T63
.............................................................gugauuucucuggugcuau................................   11    0   T63
.............................................................gugauuucucuggugcuauu...............................   27    0   T63
.............................................................gugauuucucuggugcuauua..............................   7     0   T63
.............................................................gugauuucucuggugcuauuaC.............................   2     1   T63
.............................................................gugauuucucuggugcuauuaa.............................   1     0   T63
.............................................................gugauuucucuggugcuauuaaA............................   2     1   T63
..............................................................ugauuucucuggugcuau................................   4     0   T63
```

```
                   Mature                                  Star
gccuuuuccuggugacacuuaauggcacuggaagaauucacgggguacgauuccagauccccgugauuucucuggugcuauuaagaguugcugugggacaaaucugcuuuaaa
..............................................................ugauuucucuggugcuauu...............................    8      0     T63
..............................................................ugauuucucuggugcuauuU..............................    1      1     T63
..............................................................ugUuuucucuggugcuauua..............................    1      1     T63
..............................................................ugauuucucuggugcuauua..............................    2      0     T63
..............................................................ugauuucucuggugcuauuaa.............................    1      0     T63
..............................................................ugauuucucuggugcuauuaaU............................    1      1     T63

...............Gacuuaauggcacuggaagaauucacg......................................................................    1      1     MOL
................acuuaauggcacuggaagaauuca........................................................................    2      0     MOL
.................cuuaauggcacuggaagaauuc.........................................................................    2      0     MOL
.................cuuaauggcacuggaagaauuca........................................................................    2      0     MOL
.................cuuaauggcacuggaagaauucacg......................................................................    4      0     MOL
..................uuaauggcacuggaagaauuc.........................................................................    2      0     MOL
..................uuaauggcacuggaagaauuca........................................................................    4      0     MOL
..................uuaauggcacuggaagaauucac.......................................................................    4      0     MOL
..................uuaauggcacuggaagaauucacC......................................................................    1      1     MOL
..................uuaauggcacuggaagaauucacg......................................................................    6      0     MOL
..................uuaauggcacuggaagaauucacgg.....................................................................    2      0     MOL
..................uuaauggcacuggaagaauucacggA....................................................................    1      1     MOL
...................uaauggcacuggaagaauuca........................................................................    6      0     MOL
...................Aaauggcacuggaagaauuca........................................................................    1      1     MOL
...................uaauggcacuggaagaauucac.......................................................................    3      0     MOL
...................Caauggcacuggaagaauucacg......................................................................    1      1     MOL
...................uaauggcacuggaagaauucacg......................................................................    9      0     MOL
...................uaauggcacuggaagaauucacgg.....................................................................    8      0     MOL
...................uaauggcacuggaagaauucacggg....................................................................    1      0     MOL
....................aauggcacuggaagaauu..........................................................................    25     0     MOL
....................aauggcacuggaagaauA..........................................................................    1      1     MOL
....................aauggGacuggaagaauuc.........................................................................    1      1     MOL
....................aauggcacuggaagaauuU.........................................................................    1      1     MOL
....................aauggcacuggaagaaCuc.........................................................................    2      1     MOL
....................aauggcacuggaaAaauuc.........................................................................    1      1     MOL
....................aauggcacuggaagaUuuc.........................................................................    1      1     MOL
....................aauggcGcuggaagaauuc.........................................................................    1      1     MOL
....................aUuggcacuggaagaauuc.........................................................................    2      1     MOL
....................aauggcaGuggaagaauuc.........................................................................    1      1     MOL
....................aauggcacuggaagaGuuc.........................................................................    1      1     MOL
....................aauggcacuggaagaauuA.........................................................................    10     1     MOL
....................aauggcacAggaagaauuc.........................................................................    1      1     MOL
....................aauggcacuggaagaauuc.........................................................................    2099   0     MOL
....................aauggcacuggaagaCuuc.........................................................................    1      1     MOL
....................aauggcacuggaagUauuc.........................................................................    4      1     MOL
....................aauggcacuggaagaauCc.........................................................................    1      1     MOL
....................aGuggcacuggaagaauuc.........................................................................    3      1     MOL
....................aauggcacugUaagaauuc.........................................................................    2      1     MOL
....................aauggAacuggaagaauuc.........................................................................    2      1     MOL
....................aGuggcacuggaagaauuca........................................................................    6      1     MOL
....................aauggcacuggaagaauCca........................................................................    1      1     MOL
....................aauggcacuggaaUaauuca........................................................................    2      1     MOL
....................aauggcacAggaagaauuca........................................................................    1      1     MOL
....................aauggcacuggUagaauuca........................................................................    3      1     MOL
....................aauggcacugAaagaauuca........................................................................    2      1     MOL
....................aauggcacuggaagaauAca........................................................................    2      1     MOL
....................aauUgcacuggaagaauuca........................................................................    1      1     MOL
....................aauggcacuggaagaauuGa........................................................................    2      1     MOL
....................aCuggcacuggaagaauuca........................................................................    5      1     MOL
....................aauggcacuggaagCauuca........................................................................    1      1     MOL
....................Cauggcacuggaagaauuca........................................................................    2      1     MOL
....................aauggcacuggaagaauucG........................................................................    5      1     MOL
....................Uauggcacuggaagaauuca........................................................................    1      1     MOL
....................aauggcacuggaagaauuca........................................................................    5756   0     MOL
....................aauggcaUuggaagaauuca........................................................................    1      1     MOL
....................aaAggcacuggaagaauuca........................................................................    2      1     MOL
....................aauggAacuggaagaauuca........................................................................    2      1     MOL
....................aauggcacuCgaagaauuca........................................................................    4      1     MOL
....................aauggcacuggaagaUuuca........................................................................    1      1     MOL
....................aauggcacuggaagaCuuca........................................................................    2      1     MOL
....................aauggcacuggaaAaauuca........................................................................    1      1     MOL
....................aauggcacuggaagaauGca........................................................................    1      1     MOL
....................aauggcacuggaagaaAuca........................................................................    3      1     MOL
```

Mature **Star**

gccuuuuccuggugacacuuaauggcacuggaagaauucacggguacgauuccagauccccgugauuucucuggugcuauuaagaguugcugugggacaaaucugcuuuaaa			
....................aauggcacuggaagUauuca..	11	1	MOL
....................aauggcacuggaagaauucU..	5	1	MOL
....................aauggcacuggaagaauuAa..	8	1	MOL
....................aauggcacuggaagaGuuca..	1	1	MOL
....................aaugAcacuggaagaauuca..	1	1	MOL
....................aauggGacuggaagaauuca..	2	1	MOL
....................aauggcacuggaagaauuUa..	3	1	MOL
....................aauggcacugUaagaauuca..	3	1	MOL
....................aauggUacuggaagaauuca..	5	1	MOL
....................aauggcacuggaUgaauuca..	1	1	MOL
....................aauggcacuggaagaauucaG...	1	1	MOL
....................aauggcacuggaagaauuAac...	1	1	MOL
....................aauggcaAuggaagaauucac...	1	1	MOL
....................aauggcaUuggaagaauucac...	2	1	MOL
....................aauggcacuggaagaauucaU...	12	1	MOL
....................aauggcacuggaagUauucac...	12	1	MOL
....................aUuggcacuggaagaauucac...	3	1	MOL
....................aauggcacuggaaAaauucac...	1	1	MOL
....................aauggcacAggaagaauucac...	7	1	MOL
....................aauggcacugAaagaauucac...	5	1	MOL
....................aauggcacuggaagGauucac...	2	1	MOL
....................aauggcacuggaagaauucac...	9210	0	MOL
....................Uauggcacuggaagaauucac...	1	1	MOL
....................aauggcacuggaagaauucaA...	99	1	MOL
....................aauggcacuggUagaauucac...	4	1	MOL
....................aaugUcacuggaagaauucac...	1	1	MOL
....................aCuggcacuggaagaauucac...	1	1	MOL
....................aauggcacuggaagaauuUac...	9	1	MOL
....................aauggcacuggaagaCuucac...	1	1	MOL
....................aauggcacuggaagaauGcac...	2	1	MOL
....................aauCgcacuggaagaauucac...	1	1	MOL
....................aauggcacuggaagaauuGac...	2	1	MOL
....................aaugAcacuggaagaauucac...	2	1	MOL
....................aaAggcacuggaagaauucac...	1	1	MOL
....................aauggcacuggaagaauCcac...	1	1	MOL
....................aauggUacuggaagaauucac...	3	1	MOL
....................aauggcacuggaagaUuucac...	1	1	MOL
....................aauggcacugUaagaauucac...	1	1	MOL
....................aauggcacuggCagaauucac...	1	1	MOL
....................aauggcacuCgaagaauucac...	5	1	MOL
....................aauggcacuggaUgaauucac...	1	1	MOL
....................aauggcacuggaagaaAucac...	3	1	MOL
....................aauggcacuggGagaauucac...	3	1	MOL
....................aauggcacCggaagaauucac...	1	1	MOL
....................aauAgcacuggaagaauucac...	1	1	MOL
....................aauggcacuUgaagaauucac...	1	1	MOL
....................aauggcacuggaaUaauucac...	3	1	MOL
....................aGuggcacuggaagaauucac...	3	1	MOL
....................aauggcacuggaaCaauucac...	2	1	MOL
....................aauggcacuggaagCauucac...	1	1	MOL
....................aauggcacuggaagaauAcac...	2	1	MOL
....................aauggGacuggaagaauucac...	5	1	MOL
....................aGuggcacuggaagaauucacg..	6	1	MOL
....................aauggcacuggGagaauucacg..	15	1	MOL
....................aauggcacuggUagaauucacg..	15	1	MOL
....................aauggcGcuggaagaauucacg..	1	1	MOL
....................aauggcacugUaagaauucacg..	4	1	MOL
....................aauggcacuggaagCauucacg..	3	1	MOL
....................aauggcUcuggaagaauucacg..	2	1	MOL
....................aauggcacuggaagaauucacC..	4	1	MOL
....................aauggGacuggaagaauucacg..	2	1	MOL
....................aauggcacuggaagaauCcacg..	5	1	MOL
....................aauggcacugCaagaauucacg..	2	1	MOL
....................aauggcaUuggaagaauucacg..	4	1	MOL
....................aauggcacuggaagGauucacg..	8	1	MOL
....................aCuggcacuggaagaauucacg..	4	1	MOL
....................aauggcacuAgaagaauucacg..	3	1	MOL
....................aauggcacuggaagaauuUacg..	11	1	MOL
....................aauggcacuggaagaauucacA..	330	1	MOL
....................aauggcacugAaagaauucacg..	6	1	MOL

Mature Star

gccuuuuccuggugacacuuaauggcacuggaagaauucacggguacgauuccagauccccgugauuucucuggugcuauuaagaguugcugugggacaaaucugcuuuaaa			
....................aaugUcacuggaagaauucacg..	3	1	MOL
....................aauggcacGggaagaauucacg..	1	1	MOL
....................aauggcacCggaagaauucacg..	4	1	MOL
....................Uauggcacuggaagaauucacg..	1	1	MOL
....................aauAgcacuggaagaauucacg..	3	1	MOL
....................aauggcacuggaagaaAucacg..	5	1	MOL
....................aauggcacuggaaAaauucacg..	1	1	MOL
....................aauggcacuggaagaauucGcg..	1	1	MOL
....................aauggcacuggaagaauucaUg..	16	1	MOL
....................aauggcacuCgaagaauucacg..	7	1	MOL
....................aauggcacAggaagaauucacg..	13	1	MOL
....................aUuggcacuggaagaauucacg..	2	1	MOL
....................aauggcacuggaaUaauucacg..	3	1	MOL
....................aaugCcacuggaagaauucacg..	1	1	MOL
....................aauggcacuggaagaauucacg..	18606	0	MOL
....................aauggcacuggaagaauucacU..	94	1	MOL
....................aauggcacuggaagaauAcacg..	4	1	MOL
....................aauggcacuggaagaauuGacg..	2	1	MOL
....................aauUgcacuggaagaauucacg..	3	1	MOL
....................aaAggcacuggaagaauucacg..	1	1	MOL
....................aaugAcacuggaagaauucacg..	5	1	MOL
....................aauggcacuggaaCaauucacg..	3	1	MOL
....................aauggcaAuggaagaauucacg..	1	1	MOL
....................aauggcacuggaGgaauucacg..	1	1	MOL
....................aauggcacuggaagaauuAacg..	2	1	MOL
....................aauggcacuggaagUauucacg..	23	1	MOL
....................aauggcacuggaagaaCucacg..	1	1	MOL
....................aauggUacuggaagaauucacg..	11	1	MOL
....................aauggcacuggCagaauucacg..	2	1	MOL
....................aaCggcacuggaagaauucacg..	2	1	MOL
....................aauggcacuggaagaUuucacg..	3	1	MOL
....................aauggAacuggaagaauucacg..	2	1	MOL
....................aauggcacuggaUgaauucacg..	1	1	MOL
....................aauggcacuggUagaauucacgg...	4	1	MOL
....................aUuggcacuggaagaauucacgg...	2	1	MOL
....................aauggcacuggaagaUuucacgg...	1	1	MOL
....................aauggcacAggaagaauucacgg...	2	1	MOL
....................aauggcacuggaagaauucacgg...	6912	0	MOL
....................aaugUcacuggaagaauucacgg...	1	1	MOL
....................aauggcaGuggaagaauucacgg...	1	1	MOL
....................aauggcacuggaagaauucacgC...	30	1	MOL
....................aauggcacugAaagaauucacgg...	5	1	MOL
....................aauggcacuggaagaaAucacgg...	1	1	MOL
....................aGuggcacuggaagaauucacgg...	2	1	MOL
....................aauggcacuCgaagaauucacgg...	1	1	MOL
....................aauggcacuggGagaauucacgg...	3	1	MOL
....................aaugAcacuggaagaauucacgg...	5	1	MOL
....................aauggcacuggaagaauuUacgg...	3	1	MOL
....................aauggcacuggaagaauucacUg...	3	1	MOL
....................aauggcacuggaagaauucacgA...	134	1	MOL
....................aauggUacuggaagaauucacgg...	4	1	MOL
....................aauggcacuggaagaauucacgU...	57	1	MOL
....................aauggcacuggaagaauGcacgg...	1	1	MOL
....................aauggcacuggaagaaCucacgg...	1	1	MOL
....................aauggcacuggaagaaGucacgg...	1	1	MOL
....................aauggcacuggaagaauuGacgg...	2	1	MOL
....................aauggcacuggCagaauucacgg...	1	1	MOL
....................aauggcacuggaagUauucacgg...	8	1	MOL
....................aauggcacugCaagaauucacgg...	2	1	MOL
....................aauggcUcuggaagaauucacgg...	1	1	MOL
....................aauggcacuggaagaauucaUgg...	8	1	MOL
....................aauggGacuggaagaauucacgg...	3	1	MOL
....................aauggcacuggaagaauCcacgg...	1	1	MOL
....................aauggcacuggaagaauucacCg...	2	1	MOL
....................aaugCcacuggaagaauucacgg...	2	1	MOL
....................aauggcacuggaagaauAcacgg...	2	1	MOL
....................aauggcacuggaagGauucacgg...	2	1	MOL
....................aauggcaUuggaagaauucacgg...	2	1	MOL
....................aauggcacuggaagaauucacAg...	13	1	MOL
....................aauggcacuggaagaauucacggU..	59	1	MOL

Mature Star

```
gccuuuuccuggugacacuuaauggcacuggaagaauucacggguacgauuccagauccccgugauuucucuggugcuauuaagaguugcugugggacaaaucugcuuuaaa
....................aauggcacuggaagaauucacggA....................................................................    499    1    MOL
....................aauggcacugAaagaauucacggg....................................................................    2      1    MOL
....................aauggcacuggaagaauucacggg....................................................................    132    0    MOL
....................aauggcacuggaagaauucacggC....................................................................    1      1    MOL
....................aauggcacuggaagUauucacgggu...................................................................    1      1    MOL
....................aauggcacuggaagaauucacgggu...................................................................    60     0    MOL
....................aauggcacuggaagaauucacgAgu...................................................................    4      1    MOL
....................aauggcacugAaagaauucacgggu...................................................................    1      1    MOL
....................aauggcacuggaagaauucacggAu...................................................................    139    1    MOL
....................aauggcacuggaagaauucacgggA...................................................................    90     1    MOL
....................aauggcacuggaagaauucacgggG...................................................................    1      1    MOL
....................aauggcacuggaagaauucacggUu...................................................................    34     1    MOL
....................aauggcacuggaagaauucacggguU..................................................................    15     1    MOL
....................aauggcacuggaagaauucacgggua..................................................................    1      0    MOL
....................aauggcacuggaagaauucacggAua..................................................................    1      1    MOL
....................aauggcacuggaagaauucacggguUc.................................................................    1      1    MOL
.....................auggcacuggaagaauuc.........................................................................    2      0    MOL
.....................auggcacuggaagaauuca........................................................................    2      0    MOL
.....................auggcacuggaagaauucac.......................................................................    7      0    MOL
.....................auggcacuggaagaauucacA......................................................................    2      1    MOL
.....................auggUacuggaagaauucacg......................................................................    1      1    MOL
.....................auggcacuggaagaauucacg......................................................................    17     0    MOL
.....................auggcacuggaagaauucacU......................................................................    1      1    MOL
.....................auggcacuggaagaauucacgg.....................................................................    13     0    MOL
.....................augAcacuggaagaauucacggg....................................................................    1      1    MOL
.....................auggcacuggaagaauucacggA....................................................................    1      1    MOL
.....................auggcacuggaagaauucacggg....................................................................    5      0    MOL
.....................auggcacuggaagaauucacgggu...................................................................    3      0    MOL
.....................auggcacuggaagaauucacgggA...................................................................    1      1    MOL
.....................auggcacuggaagaauucacggguU..................................................................    1      1    MOL
......................uggcacuggaagaauuca........................................................................    2      0    MOL
......................uggcacuggaagaauucac.......................................................................    3      0    MOL
.......................ggcacuggaagaauucac.......................................................................    13     0    MOL
.......................ggcacuggaagaauucacg......................................................................    2      0    MOL
........................gcacuggaagaauucacg......................................................................    3      0    MOL
........................gcacuggaagaauucacgg.....................................................................    1      0    MOL
.........................cacuggaagaauucacgg.....................................................................    4      0    MOL
............................................................cgugauuucucuggugcu..................................    1      0    MOL
............................................................cgugauuucucuggugcuau................................    2      0    MOL
............................................................cgugauuucucuggugcuauu...............................    6      0    MOL
............................................................cgugauuucucuggugcuauua..............................    10     0    MOL
............................................................cgugauuucucuggugcuauuU..............................    1      1    MOL
............................................................cgugauuucucuggugcuauuaU.............................    1      1    MOL
............................................................cgugauuucucuggugcuauuaa.............................    1      0    MOL
............................................................cgugauuucucuggugcuauuaaAa...........................    1      1    MOL
.............................................................gugauuucucuggugcua.................................    1      0    MOL
.............................................................gugauuucucuggugcuau................................    7      0    MOL
.............................................................gugauuucucuggugcuauu...............................    54     0    MOL
.............................................................gugauuucucuggugcuauuU..............................    2      1    MOL
.............................................................gugauuucucuggugcuauua..............................    74     0    MOL
.............................................................gugauuucucuggugcuauuaU.............................    3      1    MOL
.............................................................gugauuucucuggugcuauuaa.............................    7      0    MOL
.............................................................gugauuucucuggugcuauuaaU............................    1      1    MOL
..............................................................ugauuucucuggugcuau................................    1      0    MOL
..............................................................ugauuucucuggugcuauu...............................    11     0    MOL
..............................................................uUauuucucuggugcuauu...............................    1      1    MOL
..............................................................uCauuucucuggugcuauu...............................    1      1    MOL
..............................................................ugauuucucuggugcuauua..............................    19     0    MOL
..............................................................ugauuucucuggugcuauuU..............................    1      1    MOL
..............................................................ugauuucucuggugcuauuaC.............................    2      1    MOL
..............................................................ugauuucucuggugcuauuaa.............................    6      0    MOL
..............................................................ugauuucucuggugcuauuaU.............................    1      1    MOL
..............................................................ugauuucucuggugcuauuaaU............................    2      1    MOL
..............................................................ugauuucucuggugcuauuaaA............................    3      1    MOL
...............................................................gauuucucuggugcuauua..............................    1      0    MOL
...............................................................gauuucucuggugcuauuaC.............................    1      1    MOL
................................................................auuucucuggugcuauuaaga...........................    1      0    MOL

...............Gacuuaauggcacuggaagaauucac........................................................................    1      1    te1
...............Gacuuaauggcacuggaagaauucacg.......................................................................    4      1    te1
```

```
                Mature                                    Star
gccuuuuccuggugacacuuaauggcacuggaagaauucacgguacgauuccagaucccccgugauuucucuggugcuauuaagaguugcugugggacaaaucugcuuuaaa
.................cuuaauggcacuggaaga.............................................................................    1     0     te1
.................cuuaauggcacuggaagaauuca........................................................................    2     0     te1
.................cuuaauggcacuggaagaauucac.......................................................................    7     0     te1
.................cuuaauggcacuggaagaauucacU......................................................................    1     1     te1
.................cuuaauggcacuggaagaauucacg......................................................................    10    0     te1
.................cuuaauggcacuggaagaauucacgg.....................................................................    3     0     te1
..................uuaauggcacuggaagaauuc.........................................................................    4     0     te1
..................uuaauggcacuggaagaauuca........................................................................    8     0     te1
..................uuaauggcacuggaagaauucac.......................................................................    20    0     te1
..................uuaauggcacuggaagaauucacg......................................................................    35    0     te1
..................uuaauggcacuggaagaauucacA......................................................................    1     1     te1
..................uuaauggcacuggaagaauucacU......................................................................    1     1     te1
..................uuaauggcacuggaagaauucacgg.....................................................................    2     0     te1
...................uaauggcacuggaagaauuc.........................................................................    3     0     te1
...................uaauggUacuggaagaauuc.........................................................................    1     1     te1
...................uaauggcacuggaagaauuca........................................................................    22    0     te1
...................Gaauggcacuggaagaauucac.......................................................................    1     1     te1
...................Caauggcacuggaagaauucac.......................................................................    1     1     te1
...................uaauggcacuggaagaauucac.......................................................................    59    0     te1
...................Aaauggcacuggaagaauucac.......................................................................    1     1     te1
...................uaauggcacuggaagaauucacA......................................................................    3     1     te1
...................uaauggcacuggaagaauucacg......................................................................    80    0     te1
...................uUauggcacuggaagaauucacg......................................................................    1     1     te1
...................uaGuggcacuggaagaauucacg......................................................................    1     1     te1
...................Aaauggcacuggaagaauucacg......................................................................    1     1     te1
...................Caauggcacuggaagaauucacg......................................................................    1     1     te1
...................uaauggcacuggaagaauucacU......................................................................    1     1     te1
...................uaauggcacuggaagaauucacgg.....................................................................    9     0     te1
...................Caauggcacuggaagaauucacgg.....................................................................    1     1     te1
...................uaauggcacuggaagaauucacgC.....................................................................    1     1     te1
...................uaauggcacuggaagaauucacggA....................................................................    2     1     te1
...................uaauggcacuggaagaauucacggAu...................................................................    1     1     te1
....................aauggcacuggaagaauu..........................................................................    58    0     te1
....................aauggcacuggaagaauA..........................................................................    2     1     te1
....................aauggcacuggaagaauuc.........................................................................    5159  0     te1
....................aauggcacuggaagaauuA.........................................................................    29    1     te1
....................aCuggcacuggaagaauuc.........................................................................    1     1     te1
....................aaugUcacuggaagaauuc.........................................................................    2     1     te1
....................aauggcacuggGagaauuc.........................................................................    4     1     te1
....................aauggcacuggaagaauuU.........................................................................    22    1     te1
....................aauggcacuggCagaauuc.........................................................................    1     1     te1
....................aauCgcacuggaagaauuc.........................................................................    1     1     te1
....................Gauggcacuggaagaauuc.........................................................................    3     1     te1
....................aauggcacuggaagaauGc.........................................................................    2     1     te1
....................aUuggcacuggaagaauuc.........................................................................    1     1     te1
....................aauggcacugCaagaauuc.........................................................................    3     1     te1
....................aauggcacCggaagaauuc.........................................................................    1     1     te1
....................aaugAcacuggaagaauuc.........................................................................    4     1     te1
....................aauggcacuggaaAaauuc.........................................................................    1     1     te1
....................aauggcacuggaagaauuG.........................................................................    4     1     te1
....................aauggcaGuggaagaauuc.........................................................................    1     1     te1
....................aauggcacuggaagGauuc.........................................................................    2     1     te1
....................aauggcacuggaagCauuc.........................................................................    4     1     te1
....................aGuggcacuggaagaauuc.........................................................................    4     1     te1
....................aauggcaUuggaagaauuc.........................................................................    2     1     te1
....................aauggUacuggaagaauuc.........................................................................    3     1     te1
....................aauggcacugUaagaauuc.........................................................................    3     1     te1
....................aauggcacuggaGgaauuc.........................................................................    2     1     te1
....................aaCggcacuggaagaauuc.........................................................................    1     1     te1
....................aauggcacugAaagaauuc.........................................................................    1     1     te1
....................aauggcacuggaagaauAc.........................................................................    9     1     te1
....................Uauggcacuggaagaauuc.........................................................................    7     1     te1
....................aauggcacuggaagaUuuc.........................................................................    2     1     te1
....................aauggcacuggUagaauuca........................................................................    5     1     te1
....................aauggcacuggaagaauucC........................................................................    3     1     te1
....................aauggcGcuggaagaauuca........................................................................    1     1     te1
....................aauggcacGggaagaauuca........................................................................    2     1     te1
....................aauggcacuggaagaUuuca........................................................................    2     1     te1
....................aauggcacuggCagaauuca........................................................................    10    1     te1
....................aauggcacuggaaAaauuca........................................................................    2     1     te1
```

Mature **Star**

```
gccuuuuccuggugacacuuaauggcacuggaagaauucacgggguacgauuccagauccccgugauuucucuggugcuauuaagaguugcuguggg acaaaucugcuuuaaa
```

```
gccuuuuccuggugacacuuaauggcacuggaagaauucacgguacgauuccagauccccgugauuucucuggugcuauuaagaguugcugugggacaaaucugcuuuaaa
....................aauAgcacuggaagaauuca........................................................................    2      1    te1
....................aauggcacuggaagaaAuca........................................................................    4      1    te1
....................aauggcacuggaagaauGca........................................................................    12     1    te1
....................Gauggcacuggaagaauuca........................................................................    12     1    te1
....................aauggcacuggaaCaauuca........................................................................    1      1    te1
....................aauggcacuggaagaauuGa........................................................................    3      1    te1
....................aauUgcacuggaagaauuca........................................................................    3      1    te1
....................aaCggcacuggaagaauuca........................................................................    2      1    te1
....................aauggcacuggaagaauuca........................................................................    27915  0    te1
....................aaugCcacuggaagaauuca........................................................................    7      1    te1
....................aauggUacuggaagaauuca........................................................................    14     1    te1
....................aauggcacuggaagaauucG........................................................................    23     1    te1
....................aauggcacuggaagCauuca........................................................................    6      1    te1
....................aauggcacuggaagaaGuca........................................................................    2      1    te1
....................aUuggcacuggaagaauuca........................................................................    13     1    te1
....................Uauggcacuggaagaauuca........................................................................    20     1    te1
....................aauggcacuggGagaauuca........................................................................    19     1    te1
....................aauggcacugUaagaauuca........................................................................    8      1    te1
....................aauggcacuggaagaauucU........................................................................    45     1    te1
....................aauCgcacuggaagaauuca........................................................................    8      1    te1
....................aauggcacuggaagUauuca........................................................................    3      1    te1
....................aauggcacugCaagaauuca........................................................................    16     1    te1
....................Cauggcacuggaagaauuca........................................................................    7      1    te1
....................aauggcacugAaagaauuca........................................................................    5      1    te1
....................aauggcacuggaagaauCca........................................................................    10     1    te1
....................aaGggcacuggaagaauuca........................................................................    1      1    te1
....................aauggcacuggaagaGuuca........................................................................    1      1    te1
....................aaugAcacuggaagaauuca........................................................................    12     1    te1
....................aauggcacuggaagaauuUa........................................................................    17     1    te1
....................aauggcaGuggaagaauuca........................................................................    5      1    te1
....................aauggcaUuggaagaauuca........................................................................    6      1    te1
....................aauggcacuCgaagaauuca........................................................................    1      1    te1
....................aauggcacuggaaUaauuca........................................................................    1      1    te1
....................aGuggcacuggaagaauuca........................................................................    18     1    te1
....................aauggcacuggaCgaauuca........................................................................    1      1    te1
....................aauggcacuggaagaCuuca........................................................................    5      1    te1
....................aauggcacuggaagaaCuca........................................................................    5      1    te1
....................aauggcacuggaagGauuca........................................................................    10     1    te1
....................aauggcacAggaagaauuca........................................................................    1      1    te1
....................aauggcUcuggaagaauuca........................................................................    2      1    te1
....................aaugUcacuggaagaauuca........................................................................    1      1    te1
....................aauggGacuggaagaauuca........................................................................    1      1    te1
....................aauggcacCggaagaauuca........................................................................    1      1    te1
....................aauggcacuUgaagaauuca........................................................................    2      1    te1
....................aCuggcacuggaagaauuca........................................................................    7      1    te1
....................aauggcacuggaagaauuAa........................................................................    18     1    te1
....................aauggcacuggaGgaauuca........................................................................    6      1    te1
....................aauggcacuggaagaauAca........................................................................    2      1    te1
....................aauggcCcuggaagaauucac.......................................................................    3      1    te1
....................aauggcacuggGagaauucac.......................................................................    34     1    te1
....................aauggcacuggaagaauucGc.......................................................................    1      1    te1
....................Cauggcacuggaagaauucac.......................................................................    20     1    te1
....................aauggcacuUgaagaauucac.......................................................................    1      1    te1
....................aauggcacuggaagaauucCc.......................................................................    1      1    te1
....................aauggcacuggaagaauuGac.......................................................................    8      1    te1
....................Uauggcacuggaagaauucac.......................................................................    47     1    te1
....................aauggcacuggaaAaauucac.......................................................................    2      1    te1
....................aGuggcacuggaagaauucac.......................................................................    42     1    te1
....................aauggcacGggaagaauucac.......................................................................    1      1    te1
....................aaGggcacuggaagaauucac.......................................................................    4      1    te1
....................aauggcacuggaagaaAucac.......................................................................    5      1    te1
....................aauggcGcuggaagaauucac.......................................................................    3      1    te1
....................aauggcacuCgaagaauucac.......................................................................    3      1    te1
....................aauggcacCggaagaauucac.......................................................................    2      1    te1
....................aauggcaUuggaagaauucac.......................................................................    10     1    te1
....................aaCggcacuggaagaauucac.......................................................................    3      1    te1
....................aauggcacuggaagaGuucac.......................................................................    9      1    te1
....................aauggcacuggaagaauuUac.......................................................................    53     1    te1
....................aaugAcacuggaagaauucac.......................................................................    29     1    te1
....................aUuggcacuggaagaauucac.......................................................................    15     1    te1
```

```
                    Mature                                    Star
gccuuuuccuggugacacuuaauggcacuggaagaauucacgagguacgauuccagauccccgugauuucucuggugcuauuaagaguugcugugggacaaaucugcuuuaaa
....................aauggcacugCaagaauucac.......................................................................   23      1   te1
....................aauAgcacuggaagaauucac.......................................................................   2       1   te1
....................aauggcacuggaagaauucaG.......................................................................   2       1   te1
....................aaAggcacuggaagaauucac.......................................................................   1       1   te1
....................aauggcacuggaagGauucac.......................................................................   15      1   te1
....................aauggcacuggaagaauucaA.......................................................................   417     1   te1
....................Gauggcacuggaagaauucac.......................................................................   18      1   te1
....................aauggcacuggaagaaCucac.......................................................................   5       1   te1
....................aauggcacuggaagaUuucac.......................................................................   3       1   te1
....................aauggUacuggaagaauucac.......................................................................   43      1   te1
....................aauggcacuggaagaauucUc.......................................................................   9       1   te1
....................aaugCcacuggaagaauucac.......................................................................   7       1   te1
....................aauggcacuggaaCaauucac.......................................................................   4       1   te1
....................aauCgcacuggaagaauucac.......................................................................   10      1   te1
....................aauggcacuggaagaCuucac.......................................................................   2       1   te1
....................aauggcUcuggaagaauucac.......................................................................   2       1   te1
....................aauggcacuggaagCauucac.......................................................................   7       1   te1
....................aauggGacuggaagaauucac.......................................................................   1       1   te1
....................aauggcacuggaagaauCcac.......................................................................   23      1   te1
....................aauggcacuggaagaauGcac.......................................................................   12      1   te1
....................aauggcacuggaagaaGucac.......................................................................   5       1   te1
....................aauggcacuggaagaauucac.......................................................................   57451   0   te1
....................aauggcacuggaagaauucaU.......................................................................   64      1   te1
....................aauggcacugUaagaauucac.......................................................................   14      1   te1
....................aCuggcacuggaagaauucac.......................................................................   16      1   te1
....................aauggcacugAaagaauucac.......................................................................   17      1   te1
....................aauggcacuggUagaauucac.......................................................................   5       1   te1
....................aaugUcacuggaagaauucac.......................................................................   8       1   te1
....................aauUgcacuggaagaauucac.......................................................................   1       1   te1
....................aauggcacuggaGgaauucac.......................................................................   7       1   te1
....................aauggcaGuggaagaauucac.......................................................................   6       1   te1
....................aauggcacuggaCgaauucac.......................................................................   2       1   te1
....................aauggcacuggaUgaauucac.......................................................................   3       1   te1
....................aauggcacuAgaagaauucac.......................................................................   2       1   te1
....................aauggcacuggCagaauucac.......................................................................   18      1   te1
....................aauggcacuggaaUaauucac.......................................................................   6       1   te1
....................aauggcacuggaagaauAcac.......................................................................   2       1   te1
....................aauggcacuggaagUauucac.......................................................................   4       1   te1
....................aaAggcacuggaagaauucacg......................................................................   1       1   te1
....................aCuggcacuggaagaauucacg......................................................................   22      1   te1
....................aauggcacuggaGgaauucacg......................................................................   13      1   te1
....................aauggcacGggaagaauucacg......................................................................   1       1   te1
....................aauggcacuggaagaaAucacg......................................................................   7       1   te1
....................aauggcacuggaagaauucacU......................................................................   675     1   te1
....................aauggcacuggaagaCuucacg......................................................................   3       1   te1
....................aauggcacuggaagaauUacg.......................................................................   108     1   te1
....................aauggcaAuggaagaauucacg......................................................................   1       1   te1
....................aGuggcacuggaagaauucacg......................................................................   56      1   te1
....................aauggcacuggaagaaGucacg......................................................................   4       1   te1
....................aauggcacuggaUgaauucacg......................................................................   6       1   te1
....................aauggcacuggaagaUuucacg......................................................................   8       1   te1
....................aauggcacuggaagCauucacg......................................................................   10      1   te1
....................aauggcGcuggaagaauucacg......................................................................   3       1   te1
....................aauggUacuggaagaauucacg......................................................................   47      1   te1
....................aauggcacuggaagaauucaAg......................................................................   14      1   te1
....................aauggcacuCgaagaauucacg......................................................................   5       1   te1
....................aauggcaUuggaagaauucacg......................................................................   22      1   te1
....................aauggcacugUaagaauucacg......................................................................   17      1   te1
....................aaugCcacuggaagaauucacg......................................................................   15      1   te1
....................aauggcacuggaagaauucacA......................................................................   3405    1   te1
....................aauggcacuggUagaauucacg......................................................................   13      1   te1
....................aauggcacuggaagaauucaGg......................................................................   1       1   te1
....................aauggcacugCaagaauucacg......................................................................   20      1   te1
....................aauggcacuggaagaauucGcg......................................................................   6       1   te1
....................aauggcacuggaagaGuucacg......................................................................   8       1   te1
....................aauggcacuggaagaauuGacg......................................................................   6       1   te1
....................aauggcacuggaagaauAcacg......................................................................   5       1   te1
....................aauggcaGuggaagaauucacg......................................................................   10      1   te1
....................aauggcacuggaagaauucaUg......................................................................   46      1   te1
....................aauggAacuggaagaauucacg......................................................................   1       1   te1
```

Mature Star

```
gccuuuuccuggugacacuuaauggcacuggaagaauucacggguacgauuccagauccccgugauuucucuggugcuauuaagaguugcugugggacaaaucugcuuuaaa
....................aauggcacuggGagaauucacg......................................................................   80      1   te1
....................aauggcacuggaCgaauucacg......................................................................   1       1   te1
....................aauggcacuggaagaauucacg......................................................................   77087   0   te1
....................aaugUcacuggaagaauucacg......................................................................   10      1   te1
....................aauggcacuggaagaauGcacg......................................................................   14      1   te1
....................aauggcacuggaagaauCcacg......................................................................   32      1   te1
....................aauggcacuggaagGauucacg......................................................................   21      1   te1
....................Cauggcacuggaagaauucacg......................................................................   1       1   te1
....................aauCgcacuggaagaauucacg......................................................................   14      1   te1
....................aauggcUcuggaagaauucacg......................................................................   1       1   te1
....................aauggcacuUgaagaauucacg......................................................................   7       1   te1
....................aaugAcacuggaagaauucacg......................................................................   38      1   te1
....................Gauggcacuggaagaauucacg......................................................................   49      1   te1
....................aauUgcacuggaagaauucacg......................................................................   3       1   te1
....................aaGggcacuggaagaauucacg......................................................................   10      1   te1
....................aauggcacuggaagUauucacg......................................................................   5       1   te1
....................aauAgcacuggaagaauucacg......................................................................   4       1   te1
....................aauggcacuAgaagaauucacg......................................................................   1       1   te1
....................aauggcacugAaagaauucacg......................................................................   17      1   te1
....................aauggcacuggaagaaCucacg......................................................................   6       1   te1
....................aauggcacuggaaUaauucacg......................................................................   6       1   te1
....................aauggcacuggCagaauucacg......................................................................   29      1   te1
....................aauggcacCggaagaauucacg......................................................................   7       1   te1
....................aauggGacuggaagaauucacg......................................................................   5       1   te1
....................aauggcCcuggaagaauucacg......................................................................   1       1   te1
....................aUuggcacuggaagaauucacg......................................................................   14      1   te1
....................aaCggcacuggaagaauucacg......................................................................   7       1   te1
....................Uauggcacuggaagaauucacg......................................................................   58      1   te1
....................aauggcacuggaaCaauucacg......................................................................   7       1   te1
....................aauggcacuggaaAaauucacg......................................................................   3       1   te1
....................aauggcacuggaagaauucacC......................................................................   17      1   te1
....................aauggcacuggaagaauucUcg......................................................................   6       1   te1
....................aauggcacuggaagaauucacgg.....................................................................   13571   0   te1
....................aauggcacuggaaAaauucacgg.....................................................................   1       1   te1
....................aauggcacugAaagaauucacgg.....................................................................   2       1   te1
....................aauggcacuggaagaauucacgC.....................................................................   164     1   te1
....................aauggcacuggaagaauCcacgg.....................................................................   8       1   te1
....................aauggcacuggaagaauuUacgg.....................................................................   9       1   te1
....................aauggcacuggaUgaauucacgg.....................................................................   1       1   te1
....................aauggUacuggaagaauucacgg.....................................................................   11      1   te1
....................aauggcacuggaagaauucacgA.....................................................................   454     1   te1
....................aauggcacuggaGgaauucacgg.....................................................................   2       1   te1
....................aaCggcacuggaagaauucacgg.....................................................................   1       1   te1
....................aauggcacuggaagUauucacgg.....................................................................   1       1   te1
....................aauggcacuggaagaauucUcgg.....................................................................   1       1   te1
....................aaugAcacuggaagaauucacgg.....................................................................   12      1   te1
....................aauggcacuCgaagaauucacgg.....................................................................   1       1   te1
....................aaGggcacuggaagaauucacgg.....................................................................   3       1   te1
....................aauAgcacuggaagaauucacgg.....................................................................   2       1   te1
....................aauggcacuggaagaaGucacgg.....................................................................   2       1   te1
....................aauggcacuggUagaauucacgg.....................................................................   4       1   te1
....................aauggcacuggaagaauucaAgg.....................................................................   23      1   te1
....................aauggcacugCaagaauucacgg.....................................................................   5       1   te1
....................aauggcacuggGagaauucacgg.....................................................................   7       1   te1
....................aauggcacuggaagaaAucacgg.....................................................................   1       1   te1
....................aauggcacuggaagaauGcacgg.....................................................................   5       1   te1
....................aauggcacuggaaCaauucacgg.....................................................................   2       1   te1
....................aauUgcacuggaagaauucacgg.....................................................................   1       1   te1
....................Gauggcacuggaagaauucacgg.....................................................................   11      1   te1
....................aauggcacuggaagaauucacUg.....................................................................   3       1   te1
....................aauggcacuggaagaauucacgU.....................................................................   226     1   te1
....................aauggcacuggCagaauucacgg.....................................................................   3       1   te1
....................aauggcacuggaagaaCucacgg.....................................................................   1       1   te1
....................aauggcaGuggaagaauucacgg.....................................................................   1       1   te1
....................aauggcacuggaagGauucacgg.....................................................................   5       1   te1
....................aauggcaUuggaagaauucacgg.....................................................................   2       1   te1
....................aaugCcacuggaagaauucacgg.....................................................................   4       1   te1
....................aGuggcacuggaagaauucacgg.....................................................................   22      1   te1
....................aauggcacuUgaagaauucacgg.....................................................................   2       1   te1
....................Uauggcacuggaagaauucacgg.....................................................................   16      1   te1
```

Mature Star

```
gccuuuuccuggugacacuuaauggcacuggaagaauucacggguacgauuccagaucccccgugauuucucuggugcuauuaagaguugcugugggacaaaucugcuuuaaa
....................aauggcacuggaagCauucacgg.....................................................................  1    1  te1
....................aCuggcacuggaagaauucacgg.....................................................................  3    1  te1
....................aauggcacuggaagaGuucacgg.....................................................................  2    1  te1
....................aauggcacuggaagaauucacCg.....................................................................  2    1  te1
....................aauggcacuggaagaauucacAg.....................................................................  19   1  te1
....................aauggcacuggaagaauucaGgg.....................................................................  2    1  te1
....................aUuggcacuggaagaauucacgg.....................................................................  1    1  te1
....................aauggcacuggaagaauucaUgg.....................................................................  25   1  te1
....................aauggcacugUaagaauucacgg.....................................................................  5    1  te1
....................aauggcacuggaagaauucacggU....................................................................  121  1  te1
....................aauggcacuggaagaauucacggg....................................................................  203  0  te1
....................aauggcacuggaagaauucacggC....................................................................  1    1  te1
....................aauggcacuggaagaauucacAgg....................................................................  1    1  te1
....................aauggcacuggaagaauucacggA....................................................................  854  1  te1
....................aauggcacuggaagaauuUacggg....................................................................  1    1  te1
....................aauggcacuggaagaauucacggAu...................................................................  302  1  te1
....................aauggcacuggaagaauucacggCu...................................................................  1    1  te1
....................aauggcacuggaagaauucacgggG...................................................................  2    1  te1
....................aauggcacuggaagaauucacgggA...................................................................  136  1  te1
....................aauggcacuggaagaauucacgggu...................................................................  60   0  te1
....................aauggcacuggaagaauucacggUu...................................................................  147  1  te1
....................aauggcacuggaagaauucacggAua..................................................................  14   1  te1
....................aauggcacuggaagaauucacggguU..................................................................  14   1  te1
....................aauggcacuggaagaauucacgggua..................................................................  3    0  te1
....................aauggcacuggaagaauucacgggAa..................................................................  2    1  te1
....................aauggcacuggaagaauucacggUua..................................................................  3    1  te1
....................aauggcacuggaagaauucacggguaU.................................................................  1    1  te1
.....................auggcacuggaagaauuc.........................................................................  1    0  te1
.....................Cuggcacuggaagaauuca........................................................................  3    1  te1
.....................auggcacuggaagaauuca........................................................................  12   0  te1
.....................Cuggcacuggaagaauucac.......................................................................  8    1  te1
.....................auggcacuggaagaauucaU.......................................................................  1    1  te1
.....................auggcacuggUagaauucac.......................................................................  2    1  te1
.....................auggcacuggaagaauucac.......................................................................  100  0  te1
.....................Guggcacuggaagaauucac.......................................................................  3    1  te1
.....................auggcacuggaagGauucac.......................................................................  1    1  te1
.....................auggcacuggaagaauucaA.......................................................................  2    1  te1
.....................auggAacuggaagaauucacg......................................................................  1    1  te1
.....................Uuggcacuggaagaauucacg......................................................................  7    1  te1
.....................auggcacuggaagaauucacg......................................................................  246  0  te1
.....................aAggcacuggaagaauucacg......................................................................  1    1  te1
.....................auggcacuggUagaauucacg......................................................................  1    1  te1
.....................auggcacuggaagaauucacU......................................................................  2    1  te1
.....................Guggcacuggaagaauucacg......................................................................  1    1  te1
.....................auggcacuggaagaauucacA......................................................................  11   1  te1
.....................auggUacuggaagaauucacgg.....................................................................  1    1  te1
.....................auggcacuggaagaauucacAg.....................................................................  4    1  te1
.....................Uuggcacuggaagaauucacgg.....................................................................  1    1  te1
.....................auggcacuggaagaauucacgA.....................................................................  2    1  te1
.....................auggcacuggaagaauucacgC.....................................................................  1    1  te1
.....................auggcacuggaagaauuUacgg.....................................................................  1    1  te1
.....................auggcacuggaagaauucacgg.....................................................................  86   0  te1
.....................auggcacuggaagaauucacggg....................................................................  7    0  te1
.....................auggcacuggaagaauucacggA....................................................................  6    1  te1
.....................auggcacuggaagaauucacggU....................................................................  2    1  te1
.....................auggcacuggaagaauucacggAu...................................................................  5    1  te1
.....................auggcacuggaagaauucacgggA...................................................................  4    1  te1
.....................auggcacuggaagaauucacggUu...................................................................  2    1  te1
.....................auggcacuggaagaauucacgggu...................................................................  10   0  te1
.....................auggcacuggaagaauucacggguU..................................................................  4    1  te1
......................uggcacuggaagaauuca........................................................................  12   0  te1
......................uggcacuggaagaauucaU.......................................................................  1    1  te1
......................uggcacuggaagaauucac.......................................................................  66   0  te1
......................uggcacuggaagaauucacg......................................................................  11   0  te1
......................uggcacuggaagaauucacgg.....................................................................  5    0  te1
......................uggcacuggaagaauucacggg....................................................................  1    0  te1
......................uggcacuggaagaauucacgggA...................................................................  1    1  te1
......................uggcacuggaagaauucacgggu...................................................................  5    0  te1
......................uggcacuggaagaauucacggguU..................................................................  2    1  te1
.......................ggcacuggaagaauucaA.......................................................................  3    1  te1
```

Mature Star

gccuuuuccuggugacacuuaauggcacuggaagaauucacggguacgauuccagauccccgugauuucucuggugcuauuaagaguugcugugggacaaaucugcuuuaaa			
.......................ggcacuggGagaauucac...	1	1	te1
.......................ggUacuggaagaauucac...	1	1	te1
.......................ggcacuggaagaauucac...	151	0	te1
.......................ggcacuggaagaauucacU..	2	1	te1
.......................ggcacuggaagaauucacg..	31	0	te1
.......................ggcacuggaagaauucacgg...	11	0	te1
.......................ggcacuggaagaauucacggAu...	1	1	te1
........................gcacuggaagaauucacg..	34	0	te1
........................gcacuggaagaauucacU..	1	1	te1
........................gcacuggaagaauCcacg..	1	1	te1
........................gcacuggaagaauucacgg...	9	0	te1
.........................cacuggaagaauucacgg...	15	0	te1
.........................cacuggaagaauucaUgg...	1	1	te1
.........................cacuggaagaauucacggA..	1	1	te1
.........................cacuggaagaauucacggAu...	1	1	te1
.........................cacuggaagaauucacggUu...	1	1	te1
..........................acuggaagaauucacggA..	1	1	te1
..........................acuggaagaauucacggAu...	2	1	te1
...........................cuggaagaauucacgggu...	1	0	te1
....cgugauuucucuggugcu..................................	2	0	te1
....cgugauuucucuggugcuau................................	1	0	te1
....cgugauuucuUuggugcuau................................	1	1	te1
....cgugauuucucuggugcuauu...............................	36	0	te1
....cgugauuucucuggugcuauua..............................	55	0	te1
....cgugauuucucuggugcuauuU..............................	4	1	te1
....cgugauuucucuggugcuauuaU.............................	2	1	te1
....gugauuucucuggugcuau................................	16	0	te1
....gugauuucucuggugcuauA...............................	1	1	te1
....gugauuucucuggugcuauu...............................	222	0	te1
....gugauuucucuggugcuauuG..............................	2	1	te1
....gugauuucuUuggugcuauua..............................	1	1	te1
....gugauuucucuggugcuauua..............................	186	0	te1
....gugauuucucuggugcuauuU..............................	20	1	te1
....gugaAuucucuggugcuauua..............................	1	1	te1
....gugauuucucuggugcuauuUa.............................	1	1	te1
....gugUuuucucuggugcuauuaa.............................	1	1	te1
....gugauuucucuggugcuauuaC.............................	12	1	te1
....gugauuucucuggugcuauuaa.............................	18	0	te1
....gugauuucucuggugcuauuaU.............................	10	1	te1
....gugauuucucuggugcuauuaaA............................	4	1	te1
....gugauuucucuggugcuauuaag............................	2	0	te1
....gugauuucucuggugcuauuaaU............................	6	1	te1
....ugauuCcucuggugcuauu...............................	1	1	te1
....ugauuucucuggugcuauA...............................	1	1	te1
....ugauuucucuggugcuauu...............................	20	0	te1
....ugauuucucuggugcuauua..............................	59	0	te1
....ugauuucucuggugcuauuG..............................	1	1	te1
....Agauuucucuggugcuauua..............................	1	1	te1
....ugauuucucuggugcuauuU..............................	8	1	te1
....ugauuucucuggugcuauAaa.............................	1	1	te1
....ugauuucucuggugcuauuaU.............................	11	1	te1
....ugauuucucuggugcuauuaC.............................	28	1	te1
....ugauuucucuggugcuauuaa.............................	9	0	te1
....ugauuucucuggugcuauuaCg............................	1	1	te1
....ugauuucucuggugcuauuaaA............................	4	1	te1
....ugauuucucuggugcuauuaaU............................	5	1	te1
....ugauuucucuggugcuauuaaUa...........................	1	1	te1
....ugauuucucuggugcuauuaaAa...........................	1	1	te1
....auuucucuggugcuauuaaA............................	1	1	te1
....auuucucuggugcuauuaag............................	1	0	te1
....auuucucuggugcuauuaaAa...........................	4	1	te1
....auuucucuggugcuauuaaga...........................	1	0	te1
....auuucucuggugcuauuaaCa...........................	1	1	te1
....auuucucuggugcuauuaagaA..........................	1	1	te1
....auuucucuggugcuauuaagaCu.........................	1	1	te1
...................Caauggcacuggaagaauucacg..	1	1	egg
...................Caauggcacuggaagaauucacgg...	1	1	egg
....................aauggcacuggaagaauu..	1	0	egg
....................aauggcacuggaagaauuc...	11	0	egg

Mature **Star**

```
gccuuuuccuggugacacuuaauggcacuggaagaauucacggguacgauuccagauccccgugauuucucuggugcuauuaagaguugcugugggacaaaucugcuuuaaa
....................aaugAcacuggaagaauuc.........................................................................  1    1  egg
....................aauggcacuggaagGauuc.........................................................................  1    1  egg
....................aaugUcacuggaagaauuc.........................................................................  1    1  egg
....................aaugCcacuggaagaauuca........................................................................  1    1  egg
....................aauggcacuggaagaauuca........................................................................  22   0  egg
....................aauggcacuggaagaauucG........................................................................  1    1  egg
....................aauggcacuggaagaauucC........................................................................  1    1  egg
....................aauUgcacuggaagaauuca........................................................................  1    1  egg
....................aaugUcacuggaagaauucac.......................................................................  5    1  egg
....................Gauggcacuggaagaauucac.......................................................................  1    1  egg
....................aauggcGcuggaagaauucac.......................................................................  1    1  egg
....................aaCggcacuggaagaauucac.......................................................................  1    1  egg
....................aaugAcacuggaagaauucac.......................................................................  4    1  egg
....................aauggcacuggaagGauucac.......................................................................  1    1  egg
....................aauggcacuggGagaauucac.......................................................................  1    1  egg
....................aauggcacuggaagaGuucac.......................................................................  2    1  egg
....................aauggcacuggaagaauucaU.......................................................................  11   1  egg
....................aaugCcacuggaagaauucac.......................................................................  1    1  egg
....................aauggcacuggaagaauucac.......................................................................  256  0  egg
....................aauggcacuggaGgaauucac.......................................................................  2    1  egg
....................aGuggcacuggaagaauucac.......................................................................  1    1  egg
....................aCuggcacuggaagaauucac.......................................................................  1    1  egg
....................aauggcUcuggaagaauucac.......................................................................  1    1  egg
....................aauggcacuggaagaauucaA.......................................................................  3    1  egg
....................aauggcacAggaagaauucac.......................................................................  1    1  egg
....................Uauggcacuggaagaauucac.......................................................................  4    1  egg
....................aauggcacuggUagaauucac.......................................................................  1    1  egg
....................aauggcacuggaaAaauucacg......................................................................  1    1  egg
....................aauggcacuggaagaauucaUg......................................................................  3    1  egg
....................aauggcacuggaUgaauucacg......................................................................  1    1  egg
....................aauggcacuggaagaauucaAg......................................................................  1    1  egg
....................aauggcacuggaagaauucacA......................................................................  85   1  egg
....................aauUgcacuggaagaauucacg......................................................................  9    1  egg
....................aauggcacuggaagaGuucacg......................................................................  8    1  egg
....................aaugUcacuggaagaauucacg......................................................................  47   1  egg
....................aauggcacuggaagaauCcacg......................................................................  6    1  egg
....................aGuggcacuggaagaauucacg......................................................................  1    1  egg
....................aauggcacuggaagaauuUacg......................................................................  2    1  egg
....................aauggcacuggaagaaAucacg......................................................................  1    1  egg
....................aauggcUcuggaagaauucacg......................................................................  2    1  egg
....................aaugAcacuggaagaauucacg......................................................................  9    1  egg
....................aauggcacuAgaagaauucacg......................................................................  2    1  egg
....................aauggcacuggaagaUuucacg......................................................................  1    1  egg
....................aauggcacugAaagaauucacg......................................................................  1    1  egg
....................aauggcaGuggaagaauucacg......................................................................  1    1  egg
....................aauggcacuggaagGauucacg......................................................................  13   1  egg
....................aauggcacuggaagaauucacU......................................................................  5    1  egg
....................aauggcacuggaGgaauucacg......................................................................  10   1  egg
....................aauggcacuggaagaaCucacg......................................................................  8    1  egg
....................aauggcacuggaaUaauucacg......................................................................  1    1  egg
....................aaugCcacuggaagaauucacg......................................................................  6    1  egg
....................aCuggcacuggaagaauucacg......................................................................  2    1  egg
....................aauggcacuggGagaauucacg......................................................................  11   1  egg
....................aaAggcacuggaagaauucacg......................................................................  1    1  egg
....................aauggcacuggaagaauAcacg......................................................................  3    1  egg
....................aauggcCcuggaagaauucacg......................................................................  1    1  egg
....................aauggcacuCgaagaauucacg......................................................................  2    1  egg
....................aauggcacugCaagaauucacg......................................................................  1    1  egg
....................aauggcaUuggaagaauucacg......................................................................  3    1  egg
....................aauggcacuggUagaauucacg......................................................................  3    1  egg
....................aauggcacCggaagaauucacg......................................................................  2    1  egg
....................aauggUacuggaagaauucacg......................................................................  4    1  egg
....................Uauggcacuggaagaauucacg......................................................................  12   1  egg
....................aauAgcacuggaagaauucacg......................................................................  5    1  egg
....................aauggcacuggaagaauucGcg......................................................................  5    1  egg
....................Gauggcacuggaagaauucacg......................................................................  6    1  egg
....................aauggcacuggaCgaauucacg......................................................................  1    1  egg
....................aauggcacAggaagaauucacg......................................................................  1    1  egg
....................aauggcacugUaagaauucacg......................................................................  1    1  egg
....................Nauggcacuggaagaauucacg......................................................................  10   1  egg
```

Mature **Star**

```
gccuuuuccuggugacacuuaauggcacuggaagaauucacggguacgauuccagauccccgugauuucucuggugcuauuaagaguugcugugggacaaaucugcuuuaaa
....................aauggcacuggaagaauucacC......................................................................  8     1  egg
....................aauggcGcuggaagaauucacg......................................................................  6     1  egg
....................aaCggcacuggaagaauucacg......................................................................  3     1  egg
....................aauggcacuggaagaauucacg......................................................................  1825  0  egg
....................aauggcacuggaagaaAucacgg.....................................................................  1     1  egg
....................Gauggcacuggaagaauucacgg.....................................................................  6     1  egg
....................aauggcacuAgaagaauucacgg.....................................................................  3     1  egg
....................aauggcacuggaaAaauucacgg.....................................................................  1     1  egg
....................aauggcacuggaagaauuAacgg.....................................................................  1     1  egg
....................aauggcacuggaagaauucUcgg.....................................................................  1     1  egg
....................aaGggcacuggaagaauucacgg.....................................................................  1     1  egg
....................aauggcacuggaagUauucacgg.....................................................................  3     1  egg
....................aauggcacuggaagaauucCcgg.....................................................................  1     1  egg
....................aauggcacugUaagaauucacgg.....................................................................  1     1  egg
....................aauggcacuggaagaauucacAg.....................................................................  2     1  egg
....................aaugAcacuggaagaauucacgg.....................................................................  15    1  egg
....................aauggcacGggaagaauucacgg.....................................................................  1     1  egg
....................aauggcacAggaagaauucacgg.....................................................................  3     1  egg
....................aaAggcacuggaagaauucacgg.....................................................................  2     1  egg
....................aauggcacuggaagaauucacUg.....................................................................  1     1  egg
....................aauggcacuggaagaauucacgA.....................................................................  89    1  egg
....................aauggcacuggaagaUuucacgg.....................................................................  2     1  egg
....................aauggcacuggaagaaCucacgg.....................................................................  10    1  egg
....................aauAgcacuggaagaauucacgg.....................................................................  4     1  egg
....................aauggcaUuggaagaauucacgg.....................................................................  4     1  egg
....................aauggcacuggaaUaauucacgg.....................................................................  1     1  egg
....................aauggcacuggaagaauucacgC.....................................................................  18    1  egg
....................aauggcacuggaagaauucacgg.....................................................................  2405  0  egg
....................aauggcacuggaagaauuUacgg.....................................................................  9     1  egg
....................aGuggcacuggaagaauucacgg.....................................................................  10    1  egg
....................aauggcacuggaagaGuucacgg.....................................................................  10    1  egg
....................aauggcacuggaagGauucacgg.....................................................................  5     1  egg
....................aauggcacCggaagaauucacgg.....................................................................  4     1  egg
....................Cauggcacuggaagaauucacgg.....................................................................  1     1  egg
....................aCuggcacuggaagaauucacgg.....................................................................  4     1  egg
....................aauggcGcuggaagaauucacgg.....................................................................  10    1  egg
....................aauggcacuggGagaauucacgg.....................................................................  10    1  egg
....................aaCggcacuggaagaauucacgg.....................................................................  8     1  egg
....................aauggcaAuggaagaauucacgg.....................................................................  1     1  egg
....................aauggcUcuggaagaauucacgg.....................................................................  4     1  egg
....................aauggcacuggaagaauCcacgg.....................................................................  12    1  egg
....................aauggcacugAaagaauucacgg.....................................................................  1     1  egg
....................aauggcacuggaagaauucaUgg.....................................................................  4     1  egg
....................aaugUcacuggaagaauucacgg.....................................................................  63    1  egg
....................aauggcacugCaagaauucacgg.....................................................................  1     1  egg
....................aauggcacuggaGgaauucacgg.....................................................................  14    1  egg
....................aauggcacuggaagaauucGcgg.....................................................................  12    1  egg
....................Uauggcacuggaagaauucacgg.....................................................................  9     1  egg
....................aUuggcacuggaagaauucacgg.....................................................................  2     1  egg
....................aauUgcacuggaagaauucacgg.....................................................................  14    1  egg
....................aauggcacuggaagaauGcacgg.....................................................................  3     1  egg
....................aauggUacuggaagaauucacgg.....................................................................  4     1  egg
....................aauggcacuggaagaauucacgU.....................................................................  40    1  egg
....................aaugCcacuggaagaauucacgg.....................................................................  11    1  egg
....................aauggcacuggaagaauAcacgg.....................................................................  2     1  egg
....................Nauggcacuggaagaauucacgg.....................................................................  9     1  egg
....................aauUgcacuggaagaauucacggg....................................................................  1     1  egg
....................aauggcacCggaagaauucacggg....................................................................  1     1  egg
....................aauggcacuggaagaauucacggC....................................................................  60    1  egg
....................aauggcacuggaagaauucacgAg....................................................................  1     1  egg
....................aGuggcacuggaagaauucacggg....................................................................  1     1  egg
....................aauggcacuggGagaauucacggg....................................................................  2     1  egg
....................aauggcacuggaagaauucaUggg....................................................................  1     1  egg
....................aauggcCcuggaagaauucacggg....................................................................  1     1  egg
....................aauggcacuggaagaauucacggU....................................................................  188   1  egg
....................aauggcacuggaagaGuucacggg....................................................................  1     1  egg
....................Gauggcacuggaagaauucacggg....................................................................  1     1  egg
....................aauggcacuggaagaauucacggA....................................................................  138   1  egg
....................aCuggcacuggaagaauucacggg....................................................................  1     1  egg
....................aaugUcacuggaagaauucacggg....................................................................  4     1  egg
```

```
                    Mature                                  Star
gccuuuuccuggugacacuuaauggcacuggaagaauucacggguacgauuccagauccccgugauuucucuggugcuauuaagaguugcugugggacaaaucugcuuuaaa
....................aauggcacuggaagaauucacggg....................................................................  291   0  egg
....................aauggcacuggaagaauCcacggg....................................................................  3     1  egg
....................aauggcacuggaagaauucacAgg....................................................................  1     1  egg
....................aauggcacuggaGgaauucacggg....................................................................  5     1  egg
....................aaugAcacuggaagaauucacggg....................................................................  3     1  egg
....................aauggcacuggaagGauucacggg....................................................................  1     1  egg
....................aGuggcacuggaagaauucacgggu...................................................................  1     1  egg
....................aauggcacuggaagaauucacggCu...................................................................  1     1  egg
....................aauggcacuggaagaauucacgggC...................................................................  1     1  egg
....................aauggcacuggaagaauucacggAu...................................................................  11    1  egg
....................aauggcacuggaagaauucacgggu...................................................................  55    0  egg
....................aauggcacuggaagaauucacggUu...................................................................  64    1  egg
....................aauggcacuggaagaauucacgggA...................................................................  37    1  egg
....................aauggcacuggaGgaauucacgggu...................................................................  1     1  egg
....................aauggcacugAaagaauucacgggu...................................................................  1     1  egg
....................aauggcacuggaagaauucacgggAa..................................................................  1     1  egg
....................aauggcacuggaagaauucacggguU..................................................................  15    1  egg
....................aauggcacuggaagaauucacggAua..................................................................  3     1  egg
....................aauggcacuggaagaauucacgggua..................................................................  4     0  egg
....................aauggcacuggaagaauucacggguUc.................................................................  1     1  egg
.....................auggcacuggaagaauucacg......................................................................  1     0  egg
.....................auggcacuggaagaauucacAg.....................................................................  1     1  egg
.....................auggcacuggaagaauucacgg.....................................................................  5     0  egg
.....................auggcacuggaagaauucacggg....................................................................  3     0  egg
.....................auggcacuggaagaauucacggU....................................................................  1     1  egg
.....................auggcCcuggaagaauucacgggu...................................................................  1     1  egg
.....................auggcacuggaagaauucacggAu...................................................................  1     1  egg
.....................auggcacuggaagaauucacgggu...................................................................  1     0  egg
.....................auggcacuggaagaauucacggguU..................................................................  1     1  egg
......................uggcacuggaagaauucacg......................................................................  1     0  egg
......................uggcacuggaagaauucacggg....................................................................  4     0  egg
.........................cacuggaagaauucacggguaU.................................................................  2     1  egg
............................................................cgugauuucucuggugcuauuaaU............................  1     1  egg
............................................................cgugauuucucuggugcuauuaaAa...........................  2     1  egg
..............................................................ugauuucucuggugcuauuaa.............................  4     0  egg

.................cuuaauggcacuggaagaauucac.......................................................................  1     0  T6P
..................uuaauggcacuggaagaauuc.........................................................................  1     0  T6P
..................uuaauggcacuggaagaauucac.......................................................................  1     0  T6P
..................uuaauggcacuggaagaauucacg......................................................................  9     0  T6P
..................uuaauggcacuggaagaauucacgg.....................................................................  1     0  T6P
..................uuaauggcacuggaagaauucacgC.....................................................................  1     1  T6P
...................uaauggcacuggaagaauuc.........................................................................  1     0  T6P
...................uaauggcacuggaagaauuca........................................................................  1     0  T6P
...................uaauAgcacuggaagaauucac.......................................................................  1     1  T6P
...................uaauggcacuggaagaauucac.......................................................................  10    0  T6P
...................uaauggcacuggaagaauucacg......................................................................  5     0  T6P
...................uaauggcacugUaagaauucacg......................................................................  1     1  T6P
...................uaauggcacuggaagaauucacgg.....................................................................  2     0  T6P
....................aauggcacuggaagaauu..........................................................................  14    0  T6P
....................aauggcacuggaagaauuA.........................................................................  6     1  T6P
....................aauggcacuggaagGauuc.........................................................................  3     1  T6P
....................aauggcacCggaagaauuc.........................................................................  1     1  T6P
....................aauUgcacuggaagaauuc.........................................................................  1     1  T6P
....................aauggcacuggaagaauuc.........................................................................  895   0  T6P
....................aGuggcacuggaagaauuc.........................................................................  1     1  T6P
....................aauggcacugAaagaauuc.........................................................................  1     1  T6P
....................aauggcacuggaaUaauuc.........................................................................  1     1  T6P
....................aauggcacugUaagaauuc.........................................................................  1     1  T6P
....................aauggcacuggaagUauuc.........................................................................  1     1  T6P
....................aauggcacuggGagaauuc.........................................................................  1     1  T6P
....................aauggUacuggaagaauuca........................................................................  3     1  T6P
....................aauggcacuggaagaauucC........................................................................  1     1  T6P
....................aauggcacuggaagaauuUa........................................................................  3     1  T6P
....................aaugUcacuggaagaauuca........................................................................  1     1  T6P
....................aauggcacuggaagaauCca........................................................................  1     1  T6P
....................aauggcacuggUagaauuca........................................................................  1     1  T6P
....................aauggcacuggaagGauuca........................................................................  4     1  T6P
....................aauggcacugAaagaauuca........................................................................  2     1  T6P
....................aauggcacuggaagaauuca........................................................................  3895  0  T6P
```

```
                    Mature                                      Star
gccuuuuccuggugacacuuaauggcacuggaagaauucacgggu acgauuccagauccccgugauuucucuggugcuauuaagaguugcugugggacaaaucugcuuuaaa
....................aauggcacuggaaAaauuca........................................................................ 2     1  T6P
....................aauggcacuCgaagaauuca........................................................................ 1     1  T6P
....................aauggcacuggaagCauuca........................................................................ 1     1  T6P
....................aaugCcacuggaagaauuca........................................................................ 2     1  T6P
....................aauggcacuggaagaauuAa........................................................................ 2     1  T6P
....................aauggcacuggaaUaauuca........................................................................ 2     1  T6P
....................aauggcacuggaagaaCuca........................................................................ 1     1  T6P
....................aauggcacuAgaagaauuca........................................................................ 1     1  T6P
....................aGuggcacuggaagaauuca........................................................................ 1     1  T6P
....................aauggcacuggaagaauucU........................................................................ 2     1  T6P
....................aaAggcacuggaagaauuca........................................................................ 1     1  T6P
....................aauggcacuggaagUauuca........................................................................ 1     1  T6P
....................aauggcacugUaagaauuca........................................................................ 2     1  T6P
....................aauggcacuggaaCaauuca........................................................................ 2     1  T6P
....................aauggcacuggaagCauucac....................................................................... 4     1  T6P
....................aauggcacuUgaagaauucac....................................................................... 3     1  T6P
....................aauUgcacuggaagaauucac....................................................................... 4     1  T6P
....................aauggcacuggaaAaauucac....................................................................... 2     1  T6P
....................aauggcacuggaagaauucaU....................................................................... 5     1  T6P
....................aaugUcacuggaagaauucac....................................................................... 7     1  T6P
....................aauggcacugUaagaauucac....................................................................... 11    1  T6P
....................aGuggcacuggaagaauucac....................................................................... 1     1  T6P
....................aauggcaUuggaagaauucac....................................................................... 1     1  T6P
....................aauggcacuggaagaauucac....................................................................... 11756 0  T6P
....................aauggcacuggaagaauucaA....................................................................... 48    1  T6P
....................aauggcacuggaagGauucac....................................................................... 3     1  T6P
....................aauggcacuggaagaUuucac....................................................................... 1     1  T6P
....................Cauggcacuggaagaauucac....................................................................... 1     1  T6P
....................aauggcGcuggaagaauucac....................................................................... 11    1  T6P
....................aauggcacuggaagaauuUac....................................................................... 7     1  T6P
....................aauggcacugAaagaauucac....................................................................... 2     1  T6P
....................aauggcacuggaGgaauucac....................................................................... 1     1  T6P
....................aauggcacuggaagaauAcac....................................................................... 1     1  T6P
....................aauggcacuggUagaauucac....................................................................... 1     1  T6P
....................aauggcacuggaaCaauucac....................................................................... 10    1  T6P
....................Uauggcacuggaagaauucac....................................................................... 4     1  T6P
....................aaugAcacuggaagaauucac....................................................................... 3     1  T6P
....................aauggcacuggaagaaCucac....................................................................... 2     1  T6P
....................aauggcacugCaagaauucac....................................................................... 9     1  T6P
....................aauggcacuggGagaauucac....................................................................... 3     1  T6P
....................aauggcCcuggaagaauucac....................................................................... 2     1  T6P
....................aauggcacuggaaUaauucac....................................................................... 8     1  T6P
....................aaAggcacuggaagaauucac....................................................................... 2     1  T6P
....................aauggcacuggaagUauucac....................................................................... 2     1  T6P
....................aauggcacuggaagaGuucac....................................................................... 2     1  T6P
....................aauggUacuggaagaauucac....................................................................... 5     1  T6P
....................aaugCcacuggaagaauucac....................................................................... 1     1  T6P
....................aauggcacuggaagaauCcac....................................................................... 5     1  T6P
....................aauggcacuggCagaauucac....................................................................... 2     1  T6P
....................Gauggcacuggaagaauucac....................................................................... 2     1  T6P
....................aauggcacuAgaagaauucac....................................................................... 11    1  T6P
....................aauggcacuCgaagaauucac....................................................................... 4     1  T6P
....................aauggcacuggaagaaGucac....................................................................... 1     1  T6P
....................aauCgcacuggaagaauucac....................................................................... 2     1  T6P
....................aauggcacuggaagaauucaGg...................................................................... 1     1  T6P
....................aauggcacuggaagaGuucacg...................................................................... 1     1  T6P
....................aauggcacuggaagaauucGcg...................................................................... 2     1  T6P
....................aauggcacuggaagGauucacg...................................................................... 6     1  T6P
....................aauggcGcuggaagaauucacg...................................................................... 12    1  T6P
....................aGuggcacuggaagaauucacg...................................................................... 1     1  T6P
....................aauggcacuggaagUauucacg...................................................................... 12    1  T6P
....................aauggcacuggGagaauucacg...................................................................... 2     1  T6P
....................Gauggcacuggaagaauucacg...................................................................... 3     1  T6P
....................aauggcacuggaaCaauucacg...................................................................... 9     1  T6P
....................aauggcacugUaagaauucacg...................................................................... 5     1  T6P
....................aauggcacuggaagaauucacg...................................................................... 18113 0  T6P
....................aauggcacuggaagaauucacC...................................................................... 14    1  T6P
....................aauggcacuggaagaauuUacg...................................................................... 6     1  T6P
....................aauCgcacuggaagaauucacg...................................................................... 3     1  T6P
....................aauggcacuggaagCauucacg...................................................................... 1     1  T6P
```

Mature **Star**

gccuuuuccuggugacacuuaauggcacuggaagaauucacggguacgauuccagaucccgugauuucucuggugcuauuaagaguugcugugggacaaaucugcuuuaaa			
....................aauggcacugCaagaauucacg..	4	1	T6P
....................aauggcacuggaagaaAucacg..	1	1	T6P
....................aaugCcacuggaagaauucacg..	1	1	T6P
....................aauggUacuggaagaauucacg..	16	1	T6P
....................aauggcacuggaagaUuucacg..	4	1	T6P
....................aauggcacuAgaagaauucacg..	3	1	T6P
....................aauggcacugAaagaauucacg..	4	1	T6P
....................Uauggcacuggaagaauucacg..	6	1	T6P
....................aauUgcacuggaagaauucacg..	14	1	T6P
....................aauggcacuggaagaauGcacg..	1	1	T6P
....................aauggcacuCgaagaauucacg..	9	1	T6P
....................aaugUcacuggaagaauucacg..	6	1	T6P
....................aaugAcacuggaagaauucacg..	9	1	T6P
....................aauggcacuggaagaCuucacg..	1	1	T6P
....................aauggcacuggaaUaauucacg..	10	1	T6P
....................aauggcCcuggaagaauucacg..	1	1	T6P
....................aaAggcacuggaagaauucacg..	2	1	T6P
....................aauggcacuggaaAaauucacg..	5	1	T6P
....................aauggcacuggaagaauucacU..	84	1	T6P
....................aauggcacuUgaagaauucacg..	5	1	T6P
....................aauggcacuggaagaauucaAg..	3	1	T6P
....................aauggcacuggaagaauucaUg..	11	1	T6P
....................aauggcacuggaagaauucacA..	351	1	T6P
....................aUuggcacuggaagaauucacg..	2	1	T6P
....................aauggcacuggaagaaCucacg..	2	1	T6P
....................aauggcaUuggaagaauucacg..	5	1	T6P
....................aauggcUcuggaagaauucacg..	1	1	T6P
....................aaCggcacuggaagaauucacg..	6	1	T6P
....................aauggcacuggaagaauCcacg..	9	1	T6P
....................aauggcacuggaagaaGucacgg...	1	1	T6P
....................aauggcacuggaagaauucacUg...	2	1	T6P
....................aUuggcacuggaagaauucacgg...	1	1	T6P
....................aauggcCcuggaagaauucacgg...	2	1	T6P
....................aauggUacuggaagaauucacgg...	1	1	T6P
....................aauggcacuggaagaauucacgC...	17	1	T6P
....................aauggcacuggaagaaCucacgg...	2	1	T6P
....................aauggcacugAaagaauucacgg...	2	1	T6P
....................aaAggcacuggaagaauucacgg...	1	1	T6P
....................aaugUcacuggaagaauucacgg...	1	1	T6P
....................aaugCcacuggaagaauucacgg...	1	1	T6P
....................aauggcGcuggaagaauucacgg...	4	1	T6P
....................aauggcacuggaagaauucacgU...	44	1	T6P
....................aauggcacugCaagaauucacgg...	5	1	T6P
....................aauggcacuggUagaauucacgg...	1	1	T6P
....................aauggcacuggaagaGuucacgg...	1	1	T6P
....................aauggcacuggaagaauucaUgg...	3	1	T6P
....................aauggcacuggaagaauucacCg...	2	1	T6P
....................aauggcacuggaagUauucacgg...	1	1	T6P
....................aauggcacuggaagaauucacAg...	8	1	T6P
....................aaugAcacuggaagaauucacgg...	1	1	T6P
....................aauggcacuUgaagaauucacgg...	1	1	T6P
....................aauggcacuggaagaUuucacgg...	2	1	T6P
....................aauggcacuggaagGauucacgg...	4	1	T6P
....................aauggcacuggaagCauucacgg...	3	1	T6P
....................aauggcacuggaaUaauucacgg...	2	1	T6P
....................aauggcacugUaagaauucacgg...	1	1	T6P
....................Uauggcacuggaagaauucacgg...	1	1	T6P
....................aauggcacuggaaAaauucacgg...	2	1	T6P
....................aauggcacuggaaCaauucacgg...	1	1	T6P
....................aauggcacuggaagaauucacgA...	86	1	T6P
....................aauggcacuggaUgaauucacgg...	1	1	T6P
....................aauggcacuggGagaauucacgg...	1	1	T6P
....................aauUgcacuggaagaauucacgg...	3	1	T6P
....................aaCggcacuggaagaauucacgg...	2	1	T6P
....................aauggcacuggaagaauucacgg...	3908	0	T6P
....................aauggcacuggaagaauucacggg..	106	0	T6P
....................aauggcacuggaagaauucacggA..	199	1	T6P
....................aauggcacuggaagaauucaUggg..	1	1	T6P
....................aauggcacuggaagaauucacggU..	41	1	T6P
....................aauggcacugAaagaauucacgggu...	1	1	T6P

```
                    Mature                                   Star
gccuuuuccuggugacacuuaauggcacuggaagaauucacggguacgauuccagauccccgugauuucucuggugcuauuaagaguugcugugggacaaaucugcuuuaaa
....................aauggcacuggaagaauucacgggA...................................................................    23    1    T6P
....................aauggcacuggaagaauucacgAgu...................................................................    1     1    T6P
....................aauggcacuggaagaauucacggAu...................................................................    86    1    T6P
....................aauggcacuggaagaauucacgggu...................................................................    44    0    T6P
....................aauggcacuggaagaauucacggUu...................................................................    35    1    T6P
....................aauggcacuggaagaauucacggguU..................................................................    14    1    T6P
.....................auggcacuggaagaauuc.........................................................................    1     0    T6P
.....................auggcacuggaagaauuca........................................................................    2     0    T6P
.....................aAggcacuggaagaauucac.......................................................................    1     1    T6P
.....................auggcacuggaagaauucac.......................................................................    8     0    T6P
.....................auggcacuggaagaauucacg......................................................................    11    0    T6P
.....................auggcacuggaagaauucacU......................................................................    1     1    T6P
.....................auggcacuggaagaauucacgg.....................................................................    16    0    T6P
.....................auggcacuggaagaauucacggg....................................................................    2     0    T6P
.....................auggcacuggaagaauucacggA....................................................................    1     1    T6P
.....................auggcacuggaagaauucacgggu...................................................................    2     0    T6P
.....................auggcacuggaagaauucacggAu...................................................................    3     1    T6P
.....................auggcacuggaagaauucacggAua..................................................................    1     1    T6P
......................uggcacuggaagaauuca........................................................................    1     0    T6P
......................uggcacuggaagaauucac.......................................................................    5     0    T6P
......................uggcacuggaagaauucacg......................................................................    2     0    T6P
......................uggcacuggaagaauucacgg.....................................................................    1     0    T6P
.......................ggcacuggaagaauucac.......................................................................    23    0    T6P
.......................ggcacuggaagaauucacg......................................................................    1     0    T6P
.......................ggcacuggaagaauucacU......................................................................    1     1    T6P
........................gcacuggaagaauucacg......................................................................    1     0    T6P
........................gcacuggaagaauucacgg.....................................................................    1     0    T6P
.........................cacuggaagaauucacgg.....................................................................    3     0    T6P
............................................................cgugauuucucuggugcuauu...............................    3     0    T6P
............................................................cgugauuucucuggugcuauuU..............................    2     1    T6P
............................................................cgugauuucucuggugcuauua..............................    7     0    T6P
.............................................................gugauuucucuggugcuau................................    13    0    T6P
.............................................................gugauuucucuggugcuauu...............................    93    0    T6P
.............................................................gugauuucucuggugcuauua..............................    54    0    T6P
.............................................................gugauuucucuggugcuauuaa.............................    9     0    T6P
.............................................................gugauuucucuggugcuauuaC.............................    1     1    T6P
.............................................................gugauuucucuggugcuauuaU.............................    1     1    T6P
.............................................................gugauuucucuggugcuauuaUg............................    1     1    T6P
.............................................................gugauuucucuggugcuauuaaU............................    2     1    T6P
..............................................................ugauuucucuggugcuauu...............................    31    0    T6P
..............................................................ugauuucucuggugcuauua..............................    13    0    T6P
..............................................................ugauuucucuggugcuauuaa.............................    1     0    T6P
..............................................................ugauuucucuggugcuauuaaga...........................    1     0    T6P
.................................................................uuucucuggugcuauuaaAa...........................    1     1    T6P
...................................................................................gaguugcugugggacaaaucugcuA....    1     1    T6P

..cuuuuccuggugacacuu............................................................................................    1     0    T53
.................cuuaauggcacuggaagaauuc.........................................................................    1     0    T53
.................cuuaauggcacuggaagaauucac.......................................................................    6     0    T53
.................cuuaauggcacuggaagaauucacg......................................................................    1     0    T53
..................uGaauggcacuggaagaauuca........................................................................    1     1    T53
..................uuaauggcacuggaagaauucac.......................................................................    4     0    T53
...................Aaauggcacuggaagaauuc.........................................................................    1     1    T53
...................uaauggcacuggaagaauuc.........................................................................    1     0    T53
...................Caauggcacuggaagaauuca........................................................................    1     1    T53
...................uaauggcacuggaagaauuca........................................................................    3     0    T53
...................uaauggcacuggaagaauucac.......................................................................    4     0    T53
...................uaauggcacuggaagaauucacg......................................................................    1     0    T53
....................aauggcacuggaagaauu..........................................................................    36    0    T53
....................aaugAcacuggaagaauuc.........................................................................    3     1    T53
....................aauggcacuggaaCaauuc.........................................................................    1     1    T53
....................aauggcacuggaagaauuA.........................................................................    24    1    T53
....................aauggcacuggaagaauAc.........................................................................    1     1    T53
....................aauggcacuggUagaauuc.........................................................................    4     1    T53
....................aauggcaGuggaagaauuc.........................................................................    1     1    T53
....................aauggcacAggaagaauuc.........................................................................    2     1    T53
....................aaAggcacuggaagaauuc.........................................................................    1     1    T53
....................aauggcacuggaagaUuuc.........................................................................    1     1    T53
....................aauggcacugUaagaauuc.........................................................................    1     1    T53
....................aauggcacuggaagUauuc.........................................................................    5     1    T53
```

Mature **Star**

gccuuuuccuggugacacuuaauggcacuggaagaauucacggguacgauuccagauccccgugauuucucuggugcuauuaagaguugcugugggacaaaucugcuuuaaa			
....................aauggcacuUgaagaauuc...	1	1	T53
....................aauggcacuCgaagaauuc...	1	1	T53
....................aauggcacuggaaAaauuc...	2	1	T53
....................aauggUacuggaagaauuc...	8	1	T53
....................aaugUcacuggaagaauuc...	1	1	T53
....................aGuggcacuggaagaauuc...	1	1	T53
....................aauggGacuggaagaauuc...	1	1	T53
....................aaugCcacuggaagaauuc...	1	1	T53
....................aauggcacuggaagaauuU...	2	1	T53
....................aauggcacuggaagaaGuc...	1	1	T53
....................aauggcacuggaagaauCc...	1	1	T53
....................aauggcacuggaagaaAuc...	1	1	T53
....................aauggcUcuggaagaauuc...	1	1	T53
....................aauggcaUuggaagaauuc...	1	1	T53
....................aauggcacuggaagaauuc...	4432	0	T53
....................aauggcacuggaagGauuc...	1	1	T53
....................aGuggcacuggaagaauuca..	1	1	T53
....................aauggcacuggaaUaauuca..	1	1	T53
....................aauggcacuggaagaauucU..	16	1	T53
....................aauggcacuggaagaauCca..	1	1	T53
....................aauggcacuggaagaaAuca..	1	1	T53
....................aUuggcacuggaagaauuca..	1	1	T53
....................aauggcacuggaagUauuca..	6	1	T53
....................aauggcacuCgaagaauuca..	4	1	T53
....................aauggcacugAaagaauuca..	2	1	T53
....................aauggcacuggGagaauuca..	2	1	T53
....................aauggcacuggaagaauuAa..	6	1	T53
....................aauggcacuggaagCauuca..	2	1	T53
....................aauggGacuggaagaauuca..	1	1	T53
....................aaAggcacuggaagaauuca..	1	1	T53
....................aauggcacugCaagaauuca..	2	1	T53
....................aauggcacugUaagaauuca..	2	1	T53
....................aaCggcacuggaagaauuca..	2	1	T53
....................aauggcacuggaagaauuUa..	2	1	T53
....................aauggcacAggaagaauuca..	2	1	T53
....................aauggcacuggaagaUuuca..	1	1	T53
....................aauggcacuggaagGauuca..	1	1	T53
....................aauggcacuggaagaauuGa..	1	1	T53
....................aauggcacuggaagaauuca..	4211	0	T53
....................aCuggcacuggaagaauuca..	1	1	T53
....................aauggcacCggaagaauuca..	2	1	T53
....................aaugAcacuggaagaauuca..	2	1	T53
....................aaugUcacuggaagaauuca..	1	1	T53
....................aauggcacuggaagaCuuca..	1	1	T53
....................aauggcaUuggaagaauuca..	1	1	T53
....................aauggcacuggaagaauAca..	4	1	T53
....................aauggUacuggaagaauuca..	3	1	T53
....................aauggcacuggaagaauucG..	9	1	T53
....................aauAgcacuggaagaauucac...	1	1	T53
....................aGuggcacuggaagaauucac...	1	1	T53
....................Uauggcacuggaagaauucac...	1	1	T53
....................aauggcacuggaaCaauucac...	1	1	T53
....................aauggcacugAaagaauucac...	3	1	T53
....................aauggcacuggaagaauuGac...	1	1	T53
....................aauggcacuggaagaCuucac...	1	1	T53
....................aauggcacuggaagGauucac...	2	1	T53
....................aauggcacuggaagaaAucac...	4	1	T53
....................aauggcacuAgaagaauucac...	1	1	T53
....................aaAggcacuggaagaauucac...	1	1	T53
....................aCuggcacuggaagaauucac...	1	1	T53
....................aauggcacuggaagaauuUac...	3	1	T53
....................aauggcacuggaaAaauucac...	2	1	T53
....................aauggcacAggaagaauucac...	4	1	T53
....................aauggcacuggaagaUuucac...	3	1	T53
....................aauggcacuggaagaauAcac...	1	1	T53
....................aauggcacuCgaagaauucac...	3	1	T53
....................aauggcacuggGagaauucac...	3	1	T53
....................aauggcacuggaaUaauucac...	1	1	T53
....................aauggcacuggaagUauucac...	9	1	T53
....................aauggcacuggaagaauucaA..	44	1	T53

Mature Star

```
gccuuuuccuggugacacuuaauggcacuggaagaauucacgggguacgauuccagauccccgugauuucucuggugcuauuaagaguugcugugggacaaaucugcuuuaaa
....................aauggcacuggaUgaauucac.......................................................................	1	1	T53
....................aaugUcacuggaagaauucac.......................................................................	2	1	T53
....................aauggcacuggaagCauucac.......................................................................	1	1	T53
....................aauggcacugUaagaauucac.......................................................................	1	1	T53
....................aUuggcacuggaagaauucac.......................................................................	1	1	T53
....................aaugAcacuggaagaauucac.......................................................................	2	1	T53
....................aauggcacuggaagaauCcac.......................................................................	1	1	T53
....................aauggcacuggUagaauucac.......................................................................	4	1	T53
....................aauggUacuggaagaauucac.......................................................................	4	1	T53
....................aauggcacuggaagaauucaU.......................................................................	7	1	T53
....................aauggcacuggaagaauucac.......................................................................	5764	0	T53
....................aauggcacuggaagaauGcac.......................................................................	1	1	T53
....................aauggcacuggaagaGuucac.......................................................................	1	1	T53
....................aUuggcacuggaagaauucacg......................................................................	3	1	T53
....................aauggcacuggaagaauucacU......................................................................	155	1	T53
....................aauggcacugCaagaauucacg......................................................................	1	1	T53
....................aauggcacuggaagaauucacg......................................................................	3990	0	T53
....................aaugAcacuggaagaauucacg......................................................................	3	1	T53
....................aauggcacuggaagaauucaUg......................................................................	2	1	T53
....................aauggcacugUaagaauucacg......................................................................	1	1	T53
....................aCuggcacuggaagaauucacg......................................................................	1	1	T53
....................aauggcacAggaagaauucacg......................................................................	1	1	T53
....................aauggcacCggaagaauucacg......................................................................	1	1	T53
....................aauggcacuggaagUauucacg......................................................................	3	1	T53
....................aauggcacuggGagaauucacg......................................................................	4	1	T53
....................aauggcacuggaagaauuAacg......................................................................	1	1	T53
....................aauggcacuggaagaauucacC......................................................................	7	1	T53
....................aauggcaUuggaagaauucacg......................................................................	1	1	T53
....................aauggcacuggaagGauucacg......................................................................	4	1	T53
....................aauggcacuggaagaauucUcg......................................................................	1	1	T53
....................aauggcacuggaagCauucacg......................................................................	1	1	T53
....................aauggcacugAaagaauucacg......................................................................	1	1	T53
....................aauggcacuggaagaauuGacg......................................................................	2	1	T53
....................aauggcacuggUagaauucacg......................................................................	2	1	T53
....................aauggcacuggaagaaAucacg......................................................................	2	1	T53
....................aauggcacuCgaagaauucacg......................................................................	3	1	T53
....................aauggGacuggaagaauucacg......................................................................	2	1	T53
....................aauggcacuggaaCaauucacg......................................................................	1	1	T53
....................aauggcacuggaaUaauucacg......................................................................	2	1	T53
....................aauggcacuggaagaauucacA......................................................................	331	1	T53
....................aauggcacuggaaAaauucacg......................................................................	3	1	T53
....................aauggcacuggaagaaCucacg......................................................................	2	1	T53
....................aauggUacuggaagaauucacg......................................................................	2	1	T53
....................aauggcacuCgaagaauucacgg.....................................................................	1	1	T53
....................aauggcUcuggaagaauucacgg.....................................................................	2	1	T53
....................aauggcacuggaagaauucacgC.....................................................................	12	1	T53
....................aauggcacuggaagaauucacCg.....................................................................	2	1	T53
....................aaugAcacuggaagaauucacgg.....................................................................	1	1	T53
....................aauggcacuggaagaauucacgU.....................................................................	18	1	T53
....................aauggcacuggaagaauucaGgg.....................................................................	1	1	T53
....................aauggcacuggaagaauucacgA.....................................................................	11	1	T53
....................aauggcacugAaagaauucacgg.....................................................................	2	1	T53
....................aauggcacuggaagaauucaUgg.....................................................................	2	1	T53
....................aauggcacuggaagaauucacgg.....................................................................	953	0	T53
....................aauggcacuggaagGauucacgg.....................................................................	1	1	T53
....................aauggcacuggaagaauucacggA....................................................................	33	1	T53
....................aauggcacuggaagaauucacggU....................................................................	16	1	T53
....................aauggcacuggaagaauucacggg....................................................................	9	0	T53
....................aauggcacugAaagaauucacggg....................................................................	2	1	T53
....................aauggcacuggaagaauucacgggA...................................................................	3	1	T53
....................aauggcacuggaagaauucacggUu...................................................................	10	1	T53
....................aauggcacuggaagaauucacgggu...................................................................	6	0	T53
....................aauggcacuggaagaauucacggAu...................................................................	24	1	T53
....................aauggcacuggaagaauucacgggAa..................................................................	1	1	T53
.....................auggcacuggaagaauuc.........................................................................	4	0	T53
.....................auggcacuggaagaauuca........................................................................	2	0	T53
.....................auggcacuggaagaauucac.......................................................................	5	0	T53
.....................auggcacuggaagaauucacU......................................................................	1	1	T53
.....................auggcacuggaagaauucacg......................................................................	18	0	T53
.....................auggcacuggaagaauucacgg.....................................................................	6	0	T53
```

Mature   Star

```
gccuuuuccuggugacacuuaauggcacuggaagaauucacggguacgauuccagauccccgugauuucucuggugcuauuaagaguugcugugggacaaaucugcuuuaaa
.....................auggcacuggaagaauucacgC.....................................................................     2    1    T53
.....................auggcacuggaagaauucacggg....................................................................     1    0    T53
.....................auggcacuggaagaauucacggAu...................................................................     2    1    T53
......................uggcacuggaagaauuca........................................................................     3    0    T53
......................uggcacuggaagaauucac.......................................................................     7    0    T53
.......................ggcacuggaagaauucaU.......................................................................     1    1    T53
.......................ggcacuggaagaauucac.......................................................................    13    0    T53
.......................ggcacuggaagaauucaA.......................................................................     1    1    T53
.......................ggcacuggaagaauucacU......................................................................     2    1    T53
.......................ggcacuggaagaauucacg......................................................................     2    0    T53
.......................ggcacuggaagaauucacgg.....................................................................     2    0    T53
.......................ggcacuggaagaauucacgggu...................................................................     1    0    T53
........................gcacuggaagaauucacg......................................................................     2    0    T53
........................gcacuggaagaauucacgg.....................................................................     1    0    T53
............................................................cgugauuucucuggugcuauu...............................     3    0    T53
............................................................cgugauuucucuggugcuauua..............................     3    0    T53
............................................................cgugauuucucuggugcuauuU..............................     1    1    T53
............................................................cgugauuucucuggugcuauuUa.............................     1    1    T53
.............................................................gugauuucucuggugcuau................................     7    0    T53
.............................................................gugauuucucuggugcuauu...............................    33    0    T53
.............................................................gugauuucucuggugcuauua..............................    14    0    T53
.............................................................gugauuucucuggugcuauuU..............................     3    1    T53
.............................................................gugauuucucuggugcuauuaa.............................     2    0    T53
.............................................................gugauuucucuggugcuauuaaUa...........................     1    1    T53
..............................................................ugauuucucuggugcuau................................     1    0    T53
..............................................................ugauuucucuggugcuauu...............................     8    0    T53
..............................................................ugauuucucuggugcuauua..............................     3    0    T53
..............................................................ugauuucucuggugcuauuaU.............................     1    1    T53
..............................................................ugauuucucuggugcuauuaC.............................     3    1    T53
```

Provisional ID	:	Scaffold_1043_2989
Score total	:	155867.8
Score for star read(s)	:	3.9
Score for read counts	:	155857.2
Score for mfe	:	2
Score for randfold	:	1.6
Score for cons. seed	:	3
Total read count	:	305719
Mature read count	:	300410
Loop read count	:	0
Star read count	:	5309

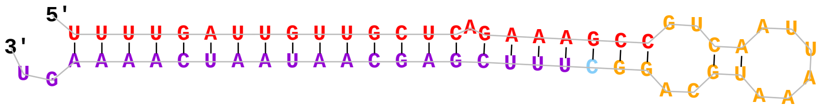

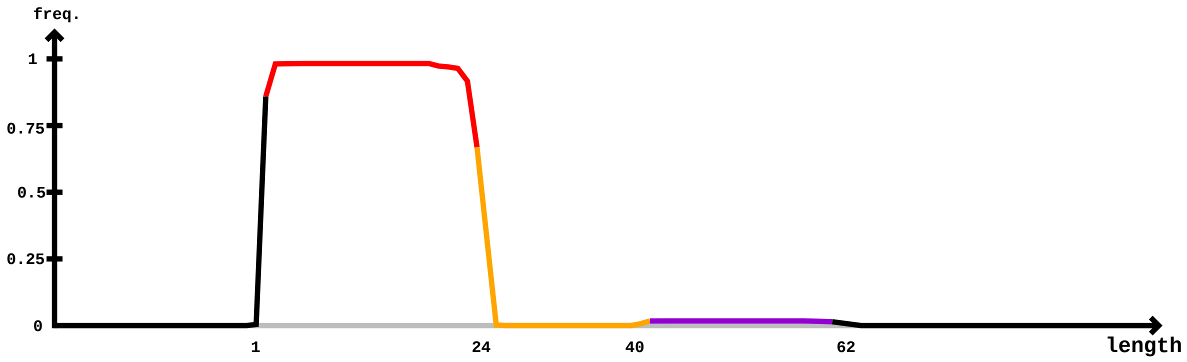

Mature Star

```
5'- uuuaaauguguccucucuacuuuugauuguugcucagaaagccgucaauuaaaaugcaggcuuucgagcaauaaaucaaaaguugagggucgacaaauaaaaucgucuguauaau  -3'  obs
    uuuaaauguguccucucuacuuuugauuguugcucagaaagccgucaauuaaaaugcaggcuuucgagcaauaaaucaaaaguugagggucgacaaauaaaaucgucuguauaau        exp
    ........(((((((((.((((((((((((((((((.(((((((..((......))..))))))))))))))))))))))))).)))))..)))).................... reads  mm  sample
    ...................Uuuuugauuguugcucaga............................................................................. 3     1   T63
    ...................Uuuuugauuguugcucagaa............................................................................ 1     1   T63
    ...................Uuuuugauuguugcucagaaa........................................................................... 4     1   T63
    ...................Uuuuugauuguugcucagaaag.......................................................................... 9     1   T63
    ...................Uuuuugauuguugcucagaaagc......................................................................... 74    1   T63
    ...................Uuuuugauuguugcucagaaagcc........................................................................ 310   1   T63
    ...................cuuuugauuguugcucagaaagcc........................................................................ 1     0   T63
    ....................uuuugaGuguugcucaga............................................................................. 1     1   T63
    ....................uuuugauuguugcucagG............................................................................. 4     1   T63
    ....................uuuugauuguugcuUaga............................................................................. 1     1   T63
    ....................uuuugauuguugcucaga............................................................................. 1061  0   T63
    ....................uuuugauuguugcucaAa............................................................................. 1     1   T63
    ....................uuuugauuguugcucagU............................................................................. 16    1   T63
    ....................uuuugCuuguugcucaga............................................................................. 1     1   T63
    ....................Auuugauuguugcucaga............................................................................. 1     1   T63
    ....................uuuugaAuguugcucaga............................................................................. 1     1   T63
    ....................uuuugauuguugcucagaC............................................................................ 1     1   T63
    ....................Guuugauuguugcucagaa............................................................................ 1     1   T63
    ....................uuuugauuguugcucagaG............................................................................ 1     1   T63
    ....................uuuugauuguugcucagaa............................................................................ 143   0   T63
    ....................uuuugauuguugcucagaU............................................................................ 25    1   T63
    ....................uuuugauuguugcucagaaU........................................................................... 33    1   T63
    ....................uuuugauuguugcucagaaG........................................................................... 6     1   T63
    ....................uuuugauuguugcucagaUa........................................................................... 17    1   T63
    ....................uuuugauuguugcucagaaC........................................................................... 24    1   T63
    ....................uuuugauuguugcucagaaa........................................................................... 280   0   T63
    ....................uuuugCuuguugcucagaaag.......................................................................... 1     1   T63
    ....................uuuugauuguugcucagaaaA.......................................................................... 110   1   T63
    ....................Auuugauuguugcucagaaag.......................................................................... 3     1   T63
    ....................uuuugauugCugcucagaaag.......................................................................... 1     1   T63
    ....................uuuAgauuguugcucagaaag.......................................................................... 3     1   T63
    ....................uuuugauuguugcucagaaag.......................................................................... 3358  0   T63
    ....................Guuugauuguugcucagaaag.......................................................................... 2     1   T63
```

Mature Star			
uuuaaauguguccucucuacuuuugauuguugcucagaaagccgucaauuaaaugcaggcuuucgagcaauaaucaaaaguugagggucgacaaauaaaaucgucuguauaau			
....................uuuugGuuguugcucagaaag..	3	1	T63
....................uuuugauuUuugcucagaaag..	1	1	T63
....................Cuuugauuguugcucagaaag..	4	1	T63
....................uuuugauugAugcucagaaag..	2	1	T63
....................uuuugauuguugcuUagaaag..	3	1	T63
....................uuuugauuAuugcucagaaag..	1	1	T63
....................uuuugauuguGgcucagaaag..	1	1	T63
....................uuuugauuguugcucagaaaC..	5	1	T63
....................uuuugauuguugcucUgaaag..	2	1	T63
....................uuuugaAuguugcucagaaag..	1	1	T63
....................uuuugauuguugcucagUaag..	1	1	T63
....................uuAugauuguugcucagaaag..	1	1	T63
....................uuuugauuguugcucaAaaag..	1	1	T63
....................uuuugauAguugcucagaaag..	1	1	T63
....................uuuugauuguugcucagaaaU..	26	1	T63
....................uuuugauuguugUucagaaag..	1	1	T63
....................uuuugauuguugcucagaUag..	1	1	T63
....................uuuugauuguugcCcagaaag..	1	1	T63
....................uuuugauuguugcucagaaGg..	19	1	T63
....................uuuugauuguugcucaUaaag..	1	1	T63
....................uuuugauuguugcucagGaag..	1	1	T63
....................uuuugauuguugcucagaaCg..	1	1	T63
....................uuuugauuguugcucagaaagU...	177	1	T63
....................Cuuugauuguugcucagaaagc...	5	1	T63
....................uuuugauuguugcuUagaaagc...	1	1	T63
....................uuuugauuguugcCcagaaagc...	1	1	T63
....................uuuugauuguugcucagaGagc...	2	1	T63
....................uuuugauuguugcucagaaaAc...	3	1	T63
....................uuuugauuguuUcucagaaagc...	1	1	T63
....................uuuugauuguugcucGgaaagc...	1	1	T63
....................uuuugauuguugcucUgaaagc...	4	1	T63
....................uuuugauuguugcucaCaaagc...	3	1	T63
....................uuuugCuuguugcucagaaagc...	1	1	T63
....................uuuAgauuguugcucagaaagc...	5	1	T63
....................uuuugauuguugUucagaaagc...	7	1	T63
....................uuuugauuAuugcucagaaagc...	5	1	T63
....................uuuugauuguugcucagaaagc...	11133	0	T63
....................uuuugauuguugcucagaaagA...	532	1	T63
....................uuuugaAuguugcucagaaagc...	1	1	T63
....................uuuugauuguugcGcagaaagc...	2	1	T63
....................uuuugauuguugcucagaaagG...	37	1	T63
....................uuuugauuguugcucagaUagc...	5	1	T63
....................uuuugauuguugcucagGaagc...	10	1	T63
....................Guuugauuguugcucagaaagc...	4	1	T63
....................uuuugauuguugcucaUaaagc...	1	1	T63
....................uuuugauugCugcucagaaagc...	2	1	T63
....................uuuugauuguugcucagaaaCc...	2	1	T63
....................Auuugauuguugcucagaaagc...	18	1	T63
....................uuuugauuguugcucagaaUgc...	1	1	T63
....................uCuugauuguugcucagaaagc...	3	1	T63
....................uuuugUuuguugcucagaaagc...	6	1	T63
....................uuuuCauuguugcucagaaagc...	2	1	T63
....................uuuugauuguugcAcagaaagc...	5	1	T63
....................uuuugauuUuugcucagaaagc...	1	1	T63
....................uuuugauuguugcucagUaagc...	8	1	T63
....................uuuugauuguugcucagaaaUc...	2	1	T63
....................uuuugauuguuCcucagaaagc...	1	1	T63
....................uuuugauuguugcucagaaGgc...	22	1	T63
....................uuuugGuuguugcucagaaagc...	4	1	T63
....................uuuugauuguugcuGagaaagc...	1	1	T63
....................uuuugauAguugcucagaaagc...	3	1	T63
....................uuuugauuguugcucagaaCgc...	1	1	T63
....................uuuugauGguugcucagaaagcc..	6	1	T63
....................uuuugauuguuCcucagaaagcc..	2	1	T63
....................uuCugauuguugcucagaaagcc..	3	1	T63
....................uuuugauuguugcucagaaagcc..	47744	0	T63
....................uuuugauuguugcucagaaUgcc..	7	1	T63
....................uuuuCauuguugcucagaaagcc..	2	1	T63
....................uuuugauuguugcucagaaaUcc..	10	1	T63
....................uuuugauuguCgcucagaaagcc..	2	1	T63

Mature Star

```
uuuaaauguguccucucuacuuuugauuguugcucagaaagccgucaauuaaaugcaggcuuucgagcaauaaucaaaaguugagggucgacaaauaaaaucgucuguauaau
....................uuuugauuguugcucagaaagAc......................................................................	4	1	T63
....................Cuuugauuguugcucagaaagcc......................................................................	36	1	T63
....................uuuugauCguugcucagaaagcc......................................................................	2	1	T63
....................uuuugauuguugUucagaaagcc......................................................................	15	1	T63
....................uuuugauuguugcucagaGagcc......................................................................	17	1	T63
....................uuuugauuguugcucUgaaagcc......................................................................	11	1	T63
....................uuuGgauuguugcucagaaagcc......................................................................	3	1	T63
....................uuuugauuguugcucagUaagcc......................................................................	18	1	T63
....................uuuugauugCugcucagaaagcc......................................................................	18	1	T63
....................uuuugauuguuAcucagaaagcc......................................................................	11	1	T63
....................uuuugauuguugcucaAaaagcc......................................................................	4	1	T63
....................uuuugauuguugcucagaaagcU......................................................................	277	1	T63
....................uuuugaCuguugcucagaaagcc......................................................................	1	1	T63
....................uuuCgauuguugcucagaaagcc......................................................................	3	1	T63
....................uuuugauuguAgcucagaaagcc......................................................................	1	1	T63
....................uuuugauuguuUcucagaaagcc......................................................................	1	1	T63
....................uuuuUauuguugcucagaaagcc......................................................................	3	1	T63
....................uuuugauuguugcCcagaaagcc......................................................................	7	1	T63
....................uuuugaAuguugcucagaaagcc......................................................................	11	1	T63
....................uuuugGuuguugcucagaaagcc......................................................................	21	1	T63
....................uAuugauuguugcucagaaagcc......................................................................	2	1	T63
....................uuuugauuguugcuUagaaagcc......................................................................	12	1	T63
....................uuuugauugGugcucagaaagcc......................................................................	1	1	T63
....................uuuugauuguugGucagaaagcc......................................................................	3	1	T63
....................uuuugauuguugcAcagaaagcc......................................................................	19	1	T63
....................uuuugauuguugcucagaaagUc......................................................................	82	1	T63
....................uuuugauuAuugcucagaaagcc......................................................................	16	1	T63
....................uuuugCuuguugcucagaaagcc......................................................................	9	1	T63
....................uCuugauuguugcucagaaagcc......................................................................	3	1	T63
....................uuuAgauuguugcucagaaagcc......................................................................	19	1	T63
....................uuuugauuguugcucaUaaagcc......................................................................	6	1	T63
....................uuuugauuguugcucagaaCgcc......................................................................	2	1	T63
....................Guuugauuguugcucagaaagcc......................................................................	17	1	T63
....................uuuugauugAugcucagaaagcc......................................................................	3	1	T63
....................uuAugauuguugcucagaaagcc......................................................................	5	1	T63
....................uuuugauuguugcucagGaagcc......................................................................	41	1	T63
....................uuuugauuguugcucaCaaagcc......................................................................	8	1	T63
....................uuuugauuguugcucagaaaCcc......................................................................	6	1	T63
....................uuuugauuguugcucagaaGgcc......................................................................	144	1	T63
....................Auuugauuguugcucagaaagcc......................................................................	78	1	T63
....................uuuugauuguugcucagaaagcG......................................................................	58	1	T63
....................uuuugauuguugcucagaaagGc......................................................................	5	1	T63
....................uuuugauuguugcucagaaagcA......................................................................	631	1	T63
....................uuuugauuguugcucagaaaAcc......................................................................	8	1	T63
....................uuuugauuCuugcucagaaagcc......................................................................	3	1	T63
....................uuuugauuguugcuGagaaagcc......................................................................	1	1	T63
....................uGuugauuguugcucagaaagcc......................................................................	1	1	T63
....................uuuugauAguugcucagaaagcc......................................................................	14	1	T63
....................uuuugauuUuugcucagaaagcc......................................................................	3	1	T63
....................uuuugauuguugcucagCaagcc......................................................................	4	1	T63
....................uuuugUuuguugcucagaaagcc......................................................................	12	1	T63
....................uuuuAauuguugcucagaaagcc......................................................................	1	1	T63
....................uuuugauuguugcucGgaaagcc......................................................................	6	1	T63
....................uuuugauuguugGcagaaagcc.......................................................................	3	1	T63
....................uuuugauuguugcucagaUagcc......................................................................	19	1	T63
....................uuuugauuguugcucagaaagccA.....................................................................	792	1	T63
....................uuuugauuguugcucagaaagccU.....................................................................	464	1	T63
....................uuuugauuguugcucagaaagccC.....................................................................	17	1	T63
....................uuuugauuguugcucagaaagccg.....................................................................	34	0	T63
....................uuuugauuguugcucagaaagccUu....................................................................	4	1	T63
....................uuuugauuguugcucagaaagccgu....................................................................	3	0	T63
....................uuuugauuguugcucagaaagccAu....................................................................	20	1	T63
.....................uuugauuguugcucagaa..........................................................................	14	0	T63
.....................uuugauuguugcucagaC..........................................................................	1	1	T63
.....................uuugauuguugcucagaaa.........................................................................	21	0	T63
.....................uuugauuguugcucagaUa.........................................................................	2	1	T63
.....................uuugauugAugcucagaaa.........................................................................	1	1	T63
.....................uuugauuguugGucagaaa.........................................................................	1	1	T63
.....................uuugauuguugcucagaaU.........................................................................	2	1	T63
.....................uuugauuguugcucagaaaA........................................................................	3	1	T63
```

Mature Star

```
uuuaaauguguccucucuacuuugauuguugcucagaaagccgucaauuaaaugcaggcuuucgagcaauaaucaaaaguugagggucgacaaauaaaaucgucuguauaau
....................uuugauuguugcucagaaUg........................................................................ 1 1 T63
....................uuugauuguugcucUgaaag........................................................................ 1 1 T63
....................uuugauuguugcucagaaag........................................................................ 92 0 T63
....................uuugUuuguugcucagaaag........................................................................ 1 1 T63
....................uuugauuguugcucagaaagG....................................................................... 2 1 T63
....................uuugauuguugcucaCaaagc....................................................................... 1 1 T63
....................uuugauuguugcucagaaGgc....................................................................... 2 1 T63
....................uuugauuguugcucagaUagc....................................................................... 1 1 T63
....................uuugauuguugcuUagaaagc....................................................................... 3 1 T63
....................Auugauuguugcucagaaagc....................................................................... 1 1 T63
....................uuugauuguugcucagaaagc....................................................................... 765 0 T63
....................uuugauuguugcucUgaaagc....................................................................... 1 1 T63
....................Cuugauuguugcucagaaagc....................................................................... 2 1 T63
....................uCugauuguugcucagaaagc....................................................................... 1 1 T63
....................uuugauuguugcucagaaagA....................................................................... 18 1 T63
....................uuugauuguAgcucagaaagcc...................................................................... 1 1 T63
....................uuugauAguugcucagaaagcc...................................................................... 1 1 T63
....................uuugauuguugcucagaaUgcc...................................................................... 3 1 T63
....................uuugauuguugUucagaaagcc...................................................................... 2 1 T63
....................uuugaCuguugcucagaaagcc...................................................................... 1 1 T63
....................uuugaAuguugcucagaaagcc...................................................................... 2 1 T63
....................uuugauuguugcucagaaGgcc...................................................................... 21 1 T63
....................uuugauuguugcucagaaaAcc...................................................................... 3 1 T63
....................uuugGuuguugcucagaaagcc...................................................................... 2 1 T63
....................uuugauuguugcucagaaaCcc...................................................................... 1 1 T63
....................uuugauuguugcucagaaagUc...................................................................... 13 1 T63
....................uuugauuguugcucagGaagcc...................................................................... 2 1 T63
....................uuugauuguugcucagUaagcc...................................................................... 4 1 T63
....................Auugauuguugcucagaaagcc...................................................................... 5 1 T63
....................uuugauuguugcucagaaagcc...................................................................... 4606 0 T63
....................uuugauuguugcucagaaagcU...................................................................... 15 1 T63
....................uuugaGuguugcucagaaagcc...................................................................... 1 1 T63
....................uuugauuguCgcucagaaagcc...................................................................... 2 1 T63
....................uuugUuuguugcucagaaagcc...................................................................... 3 1 T63
....................uuugauuguugcucagaaagcG...................................................................... 4 1 T63
....................uuugauugAugcucagaaagcc...................................................................... 1 1 T63
....................Cuugauuguugcucagaaagcc...................................................................... 4 1 T63
....................uuugauuguugcucCgaaagcc...................................................................... 1 1 T63
....................uuugauuguugcucagaGagcc...................................................................... 3 1 T63
....................uuugauuguugcucUgaaagcc...................................................................... 5 1 T63
....................uuugauuguugcucagaaagcA...................................................................... 62 1 T63
....................uuugauuguugcucaUaaagcc...................................................................... 1 1 T63
....................uuugauuguugcucagCaagcc...................................................................... 1 1 T63
....................uuugauuguugcucagaaUgccg..................................................................... 1 1 T63
....................Cuugauuguugcucagaaagccg..................................................................... 1 1 T63
....................uuugauuguugcuGagaaagccg..................................................................... 1 1 T63
....................uuugauuguugcucagGaagccg..................................................................... 2 1 T63
....................uuugauuguugcucagaaGgccg..................................................................... 3 1 T63
....................uuugauuguugcucUgaaagccg..................................................................... 1 1 T63
....................uuugauuguugcucagaaagccC..................................................................... 5 1 T63
....................Guugauuguugcucagaaagccg..................................................................... 1 1 T63
....................uuugauuguugcucagaaagccU..................................................................... 51 1 T63
....................uuugauuguugcucagaaagccg..................................................................... 726 0 T63
....................uuugauuguugcucagaaagccA..................................................................... 73 1 T63
....................uuugauuguugcucagaaagccgA.................................................................... 1 1 T63
....................uuugauuguugcucagaaagccAu.................................................................... 2 1 T63
....................uuugauuguugcucagaaagccUu.................................................................... 3 1 T63
....................uuugauuguugcucagaaagccgu.................................................................... 8 0 T63
....................uuugauuguugcucagaaagccguU................................................................... 6 1 T63
.....................uugauuguugcucagaaag........................................................................ 1 0 T63
.....................uugauuguugcucagaaagc....................................................................... 5 0 T63
.....................Cugauuguugcucagaaagcc...................................................................... 1 1 T63
.....................uugauuguugcucagaaagcc...................................................................... 36 0 T63
.....................Augauuguugcucagaaagcc...................................................................... 1 1 T63
.....................uugauuguugcucagaaagccg..................................................................... 3 0 T63
......................ugauuguugcucagaaag........................................................................ 2 0 T63
......................ugauuguugcucagaaagc....................................................................... 3 0 T63
......................ugauuguugcucagaaagcc...................................................................... 24 0 T63
......................ugauuguugcucagaaagcA...................................................................... 1 1 T63
......................ugauuguugcucagaaagccU..................................................................... 1 1 T63
```

```
                    Mature                 Star
uuuaaauguguccucucuacuuuugauuguugcucagaaagccgucaauuaaaugcaggcuuucgagcaauaaucaaaaguugagggucgacaaauaaaaucgucuguauaau
........................gauuguugcucagaaagc.......................................................................  3    0  T63
........................gauuguugcucagaaagcc......................................................................  20   0  T63
........................gauuguugcucagaaagccg.....................................................................  1    0  T63
........................gauuguugcucagaaagccA.....................................................................  1    1  T63
.........................auuguugcucagaaagcc......................................................................  4    0  T63
...........................................................cuuucUagcaauaaucaa....................................  1    1  T63
...........................................................cuuucgagcaauaaucaa....................................  117  0  T63
...........................................................cuuucgagcaauaaucaaU...................................  1    1  T63
...........................................................cuGucgagcaauaaucaaa...................................  1    1  T63
...........................................................cuuucCagcaauaaucaaa...................................  1    1  T63
...........................................................cuuucgagcaauaaucaaa...................................  126  0  T63
...........................................................cuuucgagcaauaaucaaaC..................................  1    1  T63
...........................................................cuuucgagcaauaaucUaaa..................................  1    1  T63
...........................................................cuuucgagcaauaaucaaaa..................................  72   0  T63
...........................................................cuuucgagcaauaaucaaaaA.................................  24   1  T63
...........................................................cuuucgagcaauaaucaaaaC.................................  2    1  T63
...........................................................cuuucgagcaauaaucaaaaU.................................  1    1  T63
...........................................................cuuucgagcaauaaucaaaag.................................  3    0  T63
...........................................................cuuucgagcaauaaucaaaGgu................................  4    1  T63
...........................................................cuuucgagcaauaaucaaaaAu................................  2    1  T63
...........................................................Uuuucgagcaauaaucaaaagu................................  1    1  T63
...........................................................cuuucgagcaauaaucaaGagu................................  2    1  T63
...........................................................cuuucgagcaauaaucaaaagu................................  69   0  T63
...........................................................cuuucgagcaauaaucaUaagu................................  1    1  T63
...........................................................cuuucgagcaauaaucaaaagA................................  3    1  T63
...........................................................cuuucgagcaauaaucaaaaguu...............................  1    0  T63
............................................................uuucgagcaauaaucaaaag.................................  26   0  T63
............................................................Cuucgagcaauaaucaaaagu................................  1    1  T63
............................................................uuucgagcaauaaCcaaaagu................................  1    1  T63
............................................................uuucgagcaauaaucaaaagu................................  960  0  T63
............................................................uuucgagcaauaaucUaaagu................................  1    1  T63
............................................................uuucgaAcaauaaucaaaagu................................  1    1  T63
............................................................uuuAgagcaauaaucaaaagu................................  1    1  T63
............................................................Auucgagcaauaaucaaaagu................................  1    1  T63
............................................................uuucgagcGauaaucaaaagu................................  1    1  T63
............................................................uuucgagcUauaaucaaaagu................................  1    1  T63
............................................................uuucgagcaauaaucaaaUgu................................  1    1  T63
............................................................uuucgagcaauaaucaaaagA................................  11   1  T63
............................................................uuucCagcaauaaucaaaagu................................  1    1  T63
............................................................uuucgagcaauaaucaaaaguu...............................  4    0  T63
............................................................uuucgagcaauaaucaaaaguA...............................  3    1  T63
.............................................................uucgagcaauaaucaaaagu................................  9    0  T63
..............................................................ucgagcaauaaucaaaagu................................  1    0  T63

...................Uuuuugauuguugcucagaaa.........................................................................  2    1  MOL
...................Uuuuugauuguugcucagaaag........................................................................  5    1  MOL
...................Uuuuugauuguugcucagaaagc.......................................................................  21   1  MOL
...................cuuuugauuguugcucagaaagA.......................................................................  1    1  MOL
...................cuuuugauuguugcucagaaagc.......................................................................  1    0  MOL
...................Uuuuugauuguugcucagaaagcc......................................................................  71   1  MOL
....................uuuugauuguugcucaga...........................................................................  33   0  MOL
....................uuuugauuguugcucagU...........................................................................  1    1  MOL
....................Auuugauuguugcucagaa..........................................................................  1    1  MOL
....................uuuugauuguugcucagaU..........................................................................  4    1  MOL
....................uuuugauuguugcucagaa..........................................................................  76   0  MOL
....................Cuuugauuguugcucagaa..........................................................................  1    1  MOL
....................uuuugauuguugcucagaaG.........................................................................  12   1  MOL
....................Auuugauuguugcucagaaa.........................................................................  1    1  MOL
....................uuuugauuguugcucagaaC.........................................................................  6    1  MOL
....................uuuugauuguugcucagaaU.........................................................................  13   1  MOL
....................uuuugauuguugcucagaGa.........................................................................  1    1  MOL
....................uuuugauuguugcucagaaa.........................................................................  160  0  MOL
....................uuuugauuguugcucagaUa.........................................................................  2    1  MOL
....................uuuAgauuguugcucagaaa.........................................................................  1    1  MOL
....................uuuugauAguugcucagaaag........................................................................  1    1  MOL
....................uuuugauuguugUucagaaag........................................................................  2    1  MOL
....................uuuugaCuguugcucagaaag........................................................................  1    1  MOL
....................uuuugauuguugGucagaaag........................................................................  1    1  MOL
....................uuuugauuguugcucagaaaC........................................................................  2    1  MOL
....................Cuuugauuguugcucagaaag........................................................................  1    1  MOL
```

```
                    Mature                                 Star
uuuaaauguguccucucuacuuuugauuguugcucagaaagccgucaauuaaaugcaggcuuucgagcaauaaucaaaaguugagggucgacaaauaaaaucgucuguauaau
....................uuuugauuguugcucagaUag.......................................................................    2      1    MOL
....................Guuugauuguugcucagaaag.......................................................................    1      1    MOL
....................uuuugauuguugcucagaaag.......................................................................    775    0    MOL
....................uuuugauuguugcucagaaGg.......................................................................    3      1    MOL
....................uuuugauuguugcucagaaaU.......................................................................    7      1    MOL
....................uuuugauuguugcuGagaaag.......................................................................    1      1    MOL
....................uuuugauuguugcucagaaaA.......................................................................    50     1    MOL
....................Auuugauuguugcucagaaag.......................................................................    4      1    MOL
....................uuuugauuguugcucagaaaAc......................................................................    3      1    MOL
....................uuuugauuguugGucagaaagc......................................................................    1      1    MOL
....................uuuugauuguugcucagaaaCc......................................................................    2      1    MOL
....................uuuugauuguugcucagCaagc......................................................................    1      1    MOL
....................Cuuugauuguugcucagaaagc......................................................................    6      1    MOL
....................uuuugauuguugcucaAaaagc......................................................................    1      1    MOL
....................uuuugauuguugcucagaaagA......................................................................    181    1    MOL
....................uuuugauuguugcuUagaaagc......................................................................    1      1    MOL
....................uuuugauuguugcAcagaaagc......................................................................    4      1    MOL
....................uuuugauuguugcucagaaGgc......................................................................    7      1    MOL
....................uuuugauugCugcucagaaagc......................................................................    2      1    MOL
....................uuuugauuguugcucagUaagc......................................................................    2      1    MOL
....................uuuugauuguugUucagaaagc......................................................................    6      1    MOL
....................uuuugauAguugcucagaaagc......................................................................    1      1    MOL
....................uCuugauuguugcucagaaagc......................................................................    1      1    MOL
....................uuuugauuguugcuGagaaagc......................................................................    1      1    MOL
....................Auuugauuguugcucagaaagc......................................................................    10     1    MOL
....................uuuugaAuguugcucagaaagc......................................................................    2      1    MOL
....................uuuugauuAuugcucagaaagc......................................................................    1      1    MOL
....................uuuuCauuguugcucagaaagc......................................................................    1      1    MOL
....................uuuugauuguugcucagGaagc......................................................................    4      1    MOL
....................uuuugauuguugcucagaaagc......................................................................    3580   0    MOL
....................uuuugauuguugcucagaaUgc......................................................................    2      1    MOL
....................uuuugauuguugcucagaaagU......................................................................    33     1    MOL
....................uuuugauuguugcucaUaaagc......................................................................    1      1    MOL
....................Guuugauuguugcucagaaagc......................................................................    1      1    MOL
....................uuuugauGguugcucagaaagcc.....................................................................    2      1    MOL
....................uuuugauuguugcuGagaaagcc.....................................................................    2      1    MOL
....................Guuugauuguugcucagaaagcc.....................................................................    10     1    MOL
....................uuuugauuguugcucagaUagcc.....................................................................    4      1    MOL
....................uuuugauuguugcucagGaagcc.....................................................................    10     1    MOL
....................Cuuugauuguugcucagaaagcc.....................................................................    14     1    MOL
....................uuuugauuguuAcucagaaagcc.....................................................................    1      1    MOL
....................uuuugauuguugcucaUaaagcc.....................................................................    4      1    MOL
....................uuuugauuguugcucagaaagcG.....................................................................    21     1    MOL
....................uuuugauuguCgcucagaaagcc.....................................................................    1      1    MOL
....................uuuugGuuguugcucagaaagcc.....................................................................    2      1    MOL
....................uuuugauuguugcucagaaaAcc.....................................................................    3      1    MOL
....................uuuugauuguugcucaCaaagcc.....................................................................    1      1    MOL
....................uuuugauuguugcucagaaagcc.....................................................................    9571   0    MOL
....................uuuugUuuguugcucagaaagcc.....................................................................    2      1    MOL
....................uuuugauuguugUucagaaagcc.....................................................................    5      1    MOL
....................uCuugauuguugcucagaaagcc.....................................................................    1      1    MOL
....................uuuugauuguugcuAagaaagcc.....................................................................    1      1    MOL
....................uuuugauuguuCcucagaaagcc.....................................................................    2      1    MOL
....................uuuugauuguugcucagaaGgcc.....................................................................    21     1    MOL
....................uuuugauuguuUcucagaaagcc.....................................................................    1      1    MOL
....................uuuugCuuguugcucagaaagcc.....................................................................    1      1    MOL
....................uuuugauuguugcucagaaagcA.....................................................................    582    1    MOL
....................uuuugaCuguugcucagaaagcc.....................................................................    1      1    MOL
....................uuuugauuguugcucagaCagcc.....................................................................    1      1    MOL
....................uuuugauugAugcucagaaagcc.....................................................................    1      1    MOL
....................uuuugauuAuugcucagaaagcc.....................................................................    4      1    MOL
....................uuuugauuguugcucaAaaagcc.....................................................................    1      1    MOL
....................uuuuCauuguugcucagaaagcc.....................................................................    3      1    MOL
....................uuuAgauuguugcucagaaagcc.....................................................................    6      1    MOL
....................uuAugauuguugcucagaaagcc.....................................................................    1      1    MOL
....................uuuugauuguugcGcagaaagcc.....................................................................    1      1    MOL
....................uuuugauuguugcAcagaaagcc.....................................................................    6      1    MOL
....................uuuugauuguugcucagaaagGc.....................................................................    1      1    MOL
....................uuuugauuguugcucagaGagcc.....................................................................    2      1    MOL
....................uuuugauuguugcucGgaaagcc.....................................................................    1      1    MOL
```

Mature Star

```
uuuaaauguguccucucuacuuuugauuguugcucagaaagccgucaauuaaaugcaggcuuucgagcaauaaucaaaaguugagggucgacaaauaaaaucgucuguauaau
....................uuuugauuguugcucagUaagcc......................................................................  9    1  MOL
....................uuuugauCguugcucagaaagcc......................................................................  1    1  MOL
....................uuuuUauuguugcucagaaagcc......................................................................  1    1  MOL
....................uuuugauuguugcucagaaagUc......................................................................  13   1  MOL
....................uuuugauuguugcucagaaaUcc......................................................................  2    1  MOL
....................Auuugauuguugcucagaaagcc......................................................................  23   1  MOL
....................uuuugauuCuugcucagaaagcc......................................................................  1    1  MOL
....................uuuugauuguugcucagaaUgcc......................................................................  4    1  MOL
....................uuuugaAuguugcucagaaagcc......................................................................  2    1  MOL
....................uuuugauuguugcuUagaaagcc......................................................................  6    1  MOL
....................uuuCgauuguugcucagaaagcc......................................................................  1    1  MOL
....................uuuugauuguugcucagaaagAc......................................................................  3    1  MOL
....................uuuugauAguugcucagaaagcc......................................................................  5    1  MOL
....................uuuugauuguugcucagaaagcU......................................................................  107  1  MOL
....................uuuugauuguugcucagUaagccg.....................................................................  1    1  MOL
....................uuuugauuguugcucagaaagccA.....................................................................  315  1  MOL
....................uuuugauuguugcucagaaagccC.....................................................................  3    1  MOL
....................uuuugauuguugcucagaaagccg.....................................................................  19   0  MOL
....................Cuuugauuguugcucagaaagccg.....................................................................  1    1  MOL
....................uuuugauuguugcucagaaagccU.....................................................................  182  1  MOL
....................uuuugauuguugcucagGaagccg.....................................................................  1    1  MOL
....................uuuugauuguugcucagaaagccUu....................................................................  3    1  MOL
....................uuuugauuguugcucagaaagccAu....................................................................  11   1  MOL
.....................uuugauuguugcucagaaU.........................................................................  2    1  MOL
.....................uuugauuguugcucagaaa.........................................................................  5    0  MOL
.....................uuugauuguugcucagaUa.........................................................................  1    1  MOL
.....................uuugauuguugcucagaaag........................................................................  27   0  MOL
.....................Auugauuguugcucagaaag........................................................................  1    1  MOL
.....................uuugauuguugcucagaUagc.......................................................................  1    1  MOL
.....................uuugauuguugcucagaaagA.......................................................................  10   1  MOL
.....................uuugauuguugcucagaaagc.......................................................................  242  0  MOL
.....................uuugauuguugcucagaaGgc.......................................................................  1    1  MOL
.....................uuugauuguugcucagaaagG.......................................................................  1    1  MOL
.....................uuugauuguugcucagaaagU.......................................................................  1    1  MOL
.....................uuCgauuguugcucagaaagc.......................................................................  1    1  MOL
.....................uuugauuguugcucaCaaagc.......................................................................  1    1  MOL
.....................uuugauuguugcucagaaaCcc......................................................................  1    1  MOL
.....................Cuugauuguugcucagaaagcc......................................................................  5    1  MOL
.....................uuugauuguugcucagGaagcc......................................................................  1    1  MOL
.....................uuugauuguugcucagaaagUc......................................................................  2    1  MOL
.....................uuugauuguugcucagaaagcc......................................................................  888  0  MOL
.....................uuugauuguugcucagaaagcU......................................................................  8    1  MOL
.....................uuugauuguugcucagUaagcc......................................................................  1    1  MOL
.....................uuugauugAugcucagaaagcc......................................................................  1    1  MOL
.....................Auugauuguugcucagaaagcc......................................................................  2    1  MOL
.....................uuugauuguugcucagaaGgcc......................................................................  1    1  MOL
.....................uuugauuguugcucagaaagcA......................................................................  38   1  MOL
.....................uuugauuguugcucagaaagccU.....................................................................  20   1  MOL
.....................uuugauugAugcucagaaagccg.....................................................................  2    1  MOL
.....................uuugUuuguugcucagaaagccg.....................................................................  1    1  MOL
.....................uuugauuguugcucagaaGgccg.....................................................................  1    1  MOL
.....................uuugauugGugcucagaaagccg.....................................................................  1    1  MOL
.....................uuugauuguugcucagaaagccg.....................................................................  556  0  MOL
.....................Auugauuguugcucagaaagccg.....................................................................  1    1  MOL
.....................uuugauuguugcucagaaagUcg.....................................................................  1    1  MOL
.....................uuugauuguugcucagaaagccA.....................................................................  39   1  MOL
.....................uuugauuguugcucagaaagccUu....................................................................  2    1  MOL
.....................uuugauuguugcucagaaagccgC....................................................................  1    1  MOL
.....................uuugauuguugcucagaaagccgu....................................................................  23   0  MOL
.....................uuugauuguugcucagaaagccAu....................................................................  3    1  MOL
.....................uuugauuguugcucagaaagccguU...................................................................  2    1  MOL
......................uugauuguugcucagaaagc.......................................................................  3    0  MOL
......................uugauuguugcucagaaagcc......................................................................  8    0  MOL
......................uugauuguugcucagaaagcA......................................................................  1    1  MOL
......................uugauuguugcucagaaagccg.....................................................................  2    0  MOL
......................uugauuguugcucagaaagccU.....................................................................  1    1  MOL
......................uugauuguugcucagaaagccgu....................................................................  1    0  MOL
.......................ugauuguugcucagaaagc.......................................................................  1    0  MOL
.......................ugauuguugcucagaaagcc......................................................................  7    0  MOL
.......................ugauuguugcucagaaagccU.....................................................................  1    1  MOL
```

```
                    Mature                                  Star
uuuaaauguguccucucuacuuuugauuguugcucagaaagccgucaauuaaaugcaggcuuucgagcaauaaucaaaaguugagggucgacaaauaaaaucgucuguauaau
........................gauuguugcucagaaagc.......................................................................	1	0	MOL
........................gauuguugcucagaaagcc......................................................................	4	0	MOL
........................gauuguugcucagaGagcc......................................................................	1	1	MOL
........................gauuguugcucagaaagcU......................................................................	1	1	MOL
........................gauuguugcucagaaagccA.....................................................................	1	1	MOL
...........................................................cuuucgagcaauaaucaaa...................................	14	0	MOL
...........................................................cuuucgagcaauaaucaaaa..................................	36	0	MOL
...........................................................cuuucgagcaauaaucaaaaA.................................	8	1	MOL
...........................................................cuuucgagcaauaaucaaaag.................................	5	0	MOL
...........................................................cuuucgagcaauaaucaaaagu................................	61	0	MOL
...........................................................cuuucgagcaauaaucaaaaAu................................	1	1	MOL
...........................................................cuuucgagcaauaaucaaaagA................................	3	1	MOL
...........................................................cuuucgagcaauaaucaaaaguA...............................	1	1	MOL
............................................................uuucgagcaauaaucaaa...................................	1	0	MOL
............................................................uuucgagcaauaaucaaaag.................................	10	0	MOL
............................................................uuucgagcaauaaucaaaagu................................	110	0	MOL
............................................................uuucgagcaauaaucaaaagA................................	3	1	MOL
............................................................Auucgagcaauaaucaaaagu................................	1	1	MOL
............................................................uuucgagcaauaaucaaaaguu...............................	2	0	MOL
............................................................uuucgagcaauaaucaaaaguA...............................	5	1	MOL
.............................................................uucgagcaauaaucaaaag.................................	1	0	MOL
.............................................................uucgagcaauaaucaaaagu................................	5	0	MOL
..............................................................ucgagcaauaaucaaaagu................................	1	0	MOL

...................Uuuuugauuguugcucagaaa.........................................................................	1	1	te1
...................Uuuuugauuguugcucagaaag........................................................................	1	1	te1
...................cuuuugauuguugcucagaaag........................................................................	1	0	te1
...................Uuuuugauuguugcucagaaagc.......................................................................	14	1	te1
...................Uuuuugauuguugcucagaaagcc......................................................................	7	1	te1
....................uuuugauuguugcucaga...........................................................................	10	0	te1
....................uuuugauuguugcucagaa..........................................................................	25	0	te1
....................uuuugauuguugcucagaU..........................................................................	2	1	te1
....................uuuugauuguugcucagaG..........................................................................	1	1	te1
....................uuuugauuguugcucagaaa.........................................................................	72	0	te1
....................uuuugauuguugcucagaUa.........................................................................	3	1	te1
....................uuuugauuguugcucagaaG.........................................................................	7	1	te1
....................Cuuugauuguugcucagaaa.........................................................................	1	1	te1
....................uuuugauuguugcucagaaU.........................................................................	8	1	te1
....................uuuugauuguugcucagaaC.........................................................................	3	1	te1
....................uuuugauuguCgcucagaaag........................................................................	1	1	te1
....................Auuugauuguugcucagaaag........................................................................	1	1	te1
....................Cuuugauuguugcucagaaag........................................................................	2	1	te1
....................Guuugauuguugcucagaaag........................................................................	1	1	te1
....................uuuugauuguugGucagaaag........................................................................	1	1	te1
....................uuuugauuguugcucagaaag........................................................................	910	0	te1
....................uuuugauuguugcucagaaaA........................................................................	50	1	te1
....................uuuugauuguugcucagaaaC........................................................................	1	1	te1
....................uuuugauuguugcucagaaaU........................................................................	6	1	te1
....................uuuugauCguugcucagaaag........................................................................	1	1	te1
....................uuuugauuguugcucagaaGg........................................................................	13	1	te1
....................uuuugauuguugcucagUaag........................................................................	2	1	te1
....................uuuugauuguugcucaUaaagc.......................................................................	1	1	te1
....................uuuugauuguugcucagUaagc.......................................................................	2	1	te1
....................Cuuugauuguugcucagaaagc.......................................................................	3	1	te1
....................uuuugauuguugcucagaaagU.......................................................................	23	1	te1
....................uuuugauuguugcucagaUagc.......................................................................	1	1	te1
....................uuuugauuguugcucagaaagc.......................................................................	1824	0	te1
....................uuuugauuAuugcucagaaagc.......................................................................	1	1	te1
....................uuuugauuguugcucagaaagG.......................................................................	62	1	te1
....................uuuugauuguugcucagaaagA.......................................................................	220	1	te1
....................uGuugauuguugcucagaaagc.......................................................................	1	1	te1
....................Auuugauuguugcucagaaagc.......................................................................	3	1	te1
....................Guuugauuguugcucagaaagc.......................................................................	1	1	te1
....................uuuugauuguugcuUagaaagc.......................................................................	2	1	te1
....................uuuugauuguugcucagaaGgc.......................................................................	6	1	te1
....................uuuugauuguugcucagGaagc.......................................................................	1	1	te1
....................uuuugauuguugcuUagaaagcc......................................................................	1	1	te1
....................Auuugauuguugcucagaaagcc......................................................................	2	1	te1
....................uuuugauuguugcucagaaagcG......................................................................	23	1	te1
....................uuuugauuguugcucCgaaagcc......................................................................	1	1	te1
```

Mature Star

```
uuuaaaugugucc ucucuacuuuugauuguugcucagaaagccgucaauuaaaugcaggcuuucgagcaauaaucaaaaguugagggucgacaaauaaaaucgucuguauaau
....................uuuugauuguugcucagaaaAcc......................................................................  1     1  te1
....................uuuugauuguugcucagaaagcc......................................................................  1625  0  te1
....................uuuugauuguugcucagaaGgcc......................................................................  13    1  te1
....................uuuugauuguugcucagUaagcc......................................................................  2     1  te1
....................uuuugauuguugcucagaaagcU......................................................................  35    1  te1
....................uuuAgauuguugcucagaaagcc......................................................................  1     1  te1
....................uuuugauuguugcucaAaaagcc......................................................................  1     1  te1
....................uuuugauuguugcucagGaagcc......................................................................  2     1  te1
....................uuGugauuguugcucagaaagcc......................................................................  1     1  te1
....................uuuugauuguugcucagaaagcA......................................................................  443   1  te1
....................uCuugauuguugcucagaaagcc......................................................................  1     1  te1
....................Cuuugauuguugcucagaaagcc......................................................................  1     1  te1
....................uuuCgauuguugcucagaaagcc......................................................................  3     1  te1
....................uuuugauuguugcucagaUagcc......................................................................  1     1  te1
....................uuuugauuguugcucagaaagccU.....................................................................  33    1  te1
....................uuuugauuguugcucagaaagccA.....................................................................  60    1  te1
....................uuuugauuguugcucagaaagccg.....................................................................  3     0  te1
....................uuuugauuguugcucagaaagccAu....................................................................  3     1  te1
.....................uuugauuguugcucagaa..........................................................................  2     0  te1
.....................uuugauuguugcucagaaa.........................................................................  1     0  te1
.....................uuugauuguugcucagaaaA........................................................................  1     1  te1
.....................uuugauuguugcucagaaag........................................................................  45    0  te1
.....................uuugGuuguugcucagaaagc.......................................................................  1     1  te1
.....................uuugauuguugcucagaaGgc.......................................................................  1     1  te1
.....................uuugauuguugcucagaaagc.......................................................................  140   0  te1
.....................uuugauuguugcucagaaagA.......................................................................  9     1  te1
.....................uuugauuguugcucagaaagcA......................................................................  29    1  te1
.....................uuugauuguugcucagaaagcU......................................................................  2     1  te1
.....................Auugauuguugcucagaaagcc......................................................................  1     1  te1
.....................uuugauuguugcucagaaagcc......................................................................  433   0  te1
.....................uuugauuguugcucagaaGgcc......................................................................  1     1  te1
.....................uuugauuguugcucagaaagUc......................................................................  1     1  te1
.....................uuugauuguugcucagaaagcG......................................................................  1     1  te1
.....................uuugauuguuAcucagaaagcc......................................................................  1     1  te1
.....................uuugauuguGgcucagaaagcc......................................................................  1     1  te1
.....................uuugauuguugcucagaaagccU.....................................................................  8     1  te1
.....................Cuugauuguugcucagaaagccg.....................................................................  1     1  te1
.....................uuugauuguugcucagaaagUcg.....................................................................  1     1  te1
.....................uuugaGuguugcucagaaagccg.....................................................................  1     1  te1
.....................uuugauuguugcucagaaagccA.....................................................................  13    1  te1
.....................uuugauuguugcucagaaagccg.....................................................................  119   0  te1
.....................uuugauuguugcucagaaagccC.....................................................................  1     1  te1
.....................uuugauuguugcucagaaagccgu....................................................................  3     0  te1
.....................uuugauuguugcucagaaagccguU...................................................................  1     1  te1
......................uugauuguugcucagaaagc.......................................................................  8     0  te1
......................uuUauuguugcucagaaagcc......................................................................  1     1  te1
......................uugauuguugcucagaaagcc......................................................................  6     0  te1
.......................ugauuguugcucagaaag........................................................................  1     0  te1
.......................ugauuguugcucagaaagc.......................................................................  2     0  te1
.......................ugauuguugcucagaaagcc......................................................................  7     0  te1
........................gauuguugcucagaaGgc.......................................................................  1     1  te1
........................gauuguugcucagaaagcc......................................................................  3     0  te1
...........................................................cuuucgagcaauaaucaa....................................  1     0  te1
...........................................................cuuucgagcaauaaucaaa...................................  3     0  te1
...........................................................cuuucgagcaauaaucaaaa..................................  63    0  te1
...........................................................cuuucgagUaauaaucaaaa..................................  1     1  te1
...........................................................cuuucgagcaauaaucaaaaA.................................  26    1  te1
...........................................................cuuucgagcaauaaucaaaaU.................................  1     1  te1
...........................................................cuuucgagcaauaaucaaaagA................................  4     1  te1
...........................................................cuuucgagcaauaaucaaaagG................................  6     1  te1
...........................................................cuuucgagcaauaaucaaaGgu................................  1     1  te1
...........................................................cuuucgagcaauaaucaaaagu................................  133   0  te1
...........................................................cuuucgagcaauaaucaaaaguA...............................  5     1  te1
............................................................uuucgagcaauaaucaaa...................................  1     0  te1
............................................................uuucgagcaauaaucaaaag.................................  22    0  te1
............................................................Guucgagcaauaaucaaaag.................................  1     1  te1
............................................................uuucgagcaauaaucaaaagA................................  41    1  te1
............................................................uuucgagcaauGaucaaaagu................................  1     1  te1
............................................................uuucgagUaauaaucaaaagu................................  2     1  te1
............................................................uuucgagcaauaaucaaaagu................................  845   0  te1
```

```
                   Mature                                    Star
uuuaaaugugucucucuacuuuugauuguugcucagaaagccgucaauuaaaugcaggcuuucgagcaauaaucaaaaguugagggucgacaaauaaaaucgucuguauaau
...........................................................uuucgagcaauaaucaaaUgu................................  1    1   te1
...........................................................uuucgagAaauaaucaaaagu................................  1    1   te1
...........................................................Auucgagcaauaaucaaaagu................................  2    1   te1
...........................................................uuucgaCcaauaaucaaaagu................................  1    1   te1
...........................................................Guucgagcaauaaucaaaagu................................  3    1   te1
...........................................................uuucgagcaauaaucaaaagG................................  16   1   te1
...........................................................uuucgagcaauaaucaaUagu................................  1    1   te1
...........................................................Cuucgagcaauaaucaaaagu................................  4    1   te1
...........................................................uuucgagcaauaaucaaaaguA...............................  14   1   te1
...........................................................uuucgagcaauaaucaaaaguu...............................  1    0   te1
............................................................uucgagcaauaaucaaaagu................................  3    0   te1
............................................................uucgagcaauaaucaaaagG................................  1    1   te1
.............................................................ucgagcaauaaucaaaagu................................  1    0   te1
..............................................................cgagcaauaaucaaaagu................................  1    0   te1
...............................................................................uugagggucgacaaauaaaaucg..........  1    0   te1

..................Uuuugauuguugcucagaaagc.......................................................................  4    1   egg
..................cuuuugauuguugcucagaaagc.......................................................................  14   0   egg
..................cuuuGgauuguugcucagaaagc.......................................................................  1    1   egg
..................cuuuugauuguugcucagaaagA.......................................................................  1    1   egg
..................cuuuugauuguuAcucagaaagcc......................................................................  1    1   egg
..................cuuuugauuguugcucagaaagcc......................................................................  5    0   egg
..................cuuuugaCuguugcucagaaagccg.....................................................................  1    1   egg
..................cuuuugauuguugcucagGaagccg.....................................................................  1    1   egg
..................cuuuugauuguugcucagaaagccA.....................................................................  2    1   egg
...................uuuugauuguugcucagG...........................................................................  2    1   egg
...................uuuugauuguugcCcaga...........................................................................  1    1   egg
...................uuuugauuguugcucaga...........................................................................  103  0   egg
...................uuuCgauuguugcucaga...........................................................................  1    1   egg
...................uuuGgauuguugcucaga...........................................................................  4    1   egg
...................uuuugauuguuAcucaga...........................................................................  1    1   egg
...................uuuugCuuguugcucaga...........................................................................  1    1   egg
...................uCuugauuguugcucaga...........................................................................  1    1   egg
...................uuuAgauuguugcucaga...........................................................................  1    1   egg
...................uuuugauugCugcucaga...........................................................................  1    1   egg
...................uuuugaCuguugcucaga...........................................................................  1    1   egg
...................uuuuUauuguugcucaga...........................................................................  3    1   egg
...................uuuugaGuguugcucaga...........................................................................  1    1   egg
...................uuuuCauuguugcucaga...........................................................................  1    1   egg
...................uuuugGuuguugcucagaa..........................................................................  2    1   egg
...................uuuugauuguugcGcagaa..........................................................................  1    1   egg
...................uuuugauuguugcucagaa..........................................................................  167  0   egg
...................Cuuugauuguugcucagaa..........................................................................  2    1   egg
...................uCuugauuguugcucagaa..........................................................................  2    1   egg
...................uuuugauCguugcucagaa..........................................................................  1    1   egg
...................uuuuAauuguugcucagaa..........................................................................  2    1   egg
...................uuuAgauuguugcucagaa..........................................................................  1    1   egg
...................uAuugauuguugcucagaa..........................................................................  1    1   egg
...................uuuGgauuguugcucagaa..........................................................................  7    1   egg
...................uuuugauuguugcucCgaa..........................................................................  1    1   egg
...................uuuugCuuguugcucagaa..........................................................................  2    1   egg
...................uuuugauuguugcucagaG..........................................................................  2    1   egg
...................uuuugauuguugcCcagaa..........................................................................  1    1   egg
...................uuuugauuguugcucagGa..........................................................................  2    1   egg
...................uuuugauAguugcucagaa..........................................................................  1    1   egg
...................uuuugauuguugcucGgaa..........................................................................  4    1   egg
...................uuuugauuguGgcucagaa..........................................................................  1    1   egg
...................uuuugauuguugcucagaU..........................................................................  1    1   egg
...................uuuuUauuguugcucagaa..........................................................................  8    1   egg
...................uuuugauuguuAcucagaa..........................................................................  1    1   egg
...................uuuugauuguugcuAagaaa.........................................................................  1    1   egg
...................Cuuugauuguugcucagaaa.........................................................................  1    1   egg
...................uuuGgauuguugcucagaaa.........................................................................  2    1   egg
...................uuuugauuguugcucagaaa.........................................................................  81   0   egg
...................uuuugauuAuugcucagaaa.........................................................................  1    1   egg
...................uuuugauuguugcucagaCa.........................................................................  1    1   egg
...................uuuugUuuguugcucagaaa.........................................................................  1    1   egg
...................uuuugGuuguugcucagaaa.........................................................................  1    1   egg
...................uuuAgauuguugcucagaaa.........................................................................  1    1   egg
...................uuuugauuguugcucagUaa.........................................................................  1    1   egg
```

```
                    Mature                                  Star
uuuaaauguguccucucuacuuuugauuguugcucagaaagccgucaauuaaaugcaggcuuucgagcaauaaucaaaaguugagggucgacaaauaaaaucgucuguauaau
....................uuuugauuguugcucagaUa.........................................................................  2    1  egg
....................uuuuUauuguugcucagaaa.........................................................................  5    1  egg
....................uuuugauuguugcucagaaG.........................................................................  1    1  egg
....................uuuugauuguugcuUagaaa.........................................................................  1    1  egg
....................uuAugauuguugcucagaaag........................................................................  1    1  egg
....................uuuugCuuguugcucagaaag........................................................................  12   1  egg
....................uuuugauugAugcucagaaag........................................................................  1    1  egg
....................uuuugauuguugcucagGaag........................................................................  4    1  egg
....................uuuugauuguGgcucagaaag........................................................................  1    1  egg
....................uuuuCauuguugcucagaaag........................................................................  5    1  egg
....................uuuugauuguugcucagaaaA........................................................................  50   1  egg
....................uuuugUuuguugcucagaaag........................................................................  1    1  egg
....................uuuugauuAuugcucagaaag........................................................................  1    1  egg
....................uuuugauuguugcucagaGag........................................................................  8    1  egg
....................uuuGgauuguugcucagaaag........................................................................  16   1  egg
....................uuuugauuguugcucGgaaag........................................................................  1    1  egg
....................uuuuUauuguugcucagaaag........................................................................  28   1  egg
....................uuuugGuuguugcucagaaag........................................................................  3    1  egg
....................uuuugauuguugUucagaaag........................................................................  3    1  egg
....................uuuugauuguugcuUagaaag........................................................................  1    1  egg
....................uuuugaGuguugcucagaaag........................................................................  4    1  egg
....................uuuugauuguuAcucagaaag........................................................................  3    1  egg
....................uCuugauuguugcucagaaag........................................................................  3    1  egg
....................uuuuAauuguugcucagaaag........................................................................  2    1  egg
....................uuuugauuguugcucUgaaag........................................................................  1    1  egg
....................uuuugauCguugcucagaaag........................................................................  1    1  egg
....................uuuugauuguuCcucagaaag........................................................................  1    1  egg
....................uuuugauuguugcucagaaaC........................................................................  1    1  egg
....................Cuuugauuguugcucagaaag........................................................................  6    1  egg
....................uuuugauGguugcucagaaag........................................................................  2    1  egg
....................uuuugauuguugcucagaaaU........................................................................  3    1  egg
....................uuuugauugGugcucagaaag........................................................................  1    1  egg
....................Nuuugauuguugcucagaaag........................................................................  2    1  egg
....................uuuugauuguCgcucagaaag........................................................................  1    1  egg
....................uuuugauuguugcucagaaag........................................................................  793  0  egg
....................uuuCgauuguugcucagaaag........................................................................  8    1  egg
....................uuuugauuguugcCcagaaag........................................................................  8    1  egg
....................uuuugauuguugcucagaaGg........................................................................  6    1  egg
....................uuuugaCuguugcucagaaag........................................................................  3    1  egg
....................uuuugauuguugcucagaUag........................................................................  1    1  egg
....................uuCugauuguugcucagaaag........................................................................  6    1  egg
....................uuuugauuguugcucagaaagU.......................................................................  857  1  egg
....................uuuugUuuguugcucagaaagc.......................................................................  27   1  egg
....................Nuuugauuguugcucagaaagc.......................................................................  39   1  egg
....................uuuuUauuguugcucagaaagc.......................................................................  584  1  egg
....................uuuugauuguuAcucagaaagc.......................................................................  34   1  egg
....................uuuCgauuguugcucagaaagc.......................................................................  135  1  egg
....................uuuugauuguugcucagaaaAc.......................................................................  27   1  egg
....................uCuugauuguugcucagaaagc.......................................................................  83   1  egg
....................uuuAgauuguugcucagaaagc.......................................................................  109  1  egg
....................uuuugaGuguugcucagaaagc.......................................................................  19   1  egg
....................uuuugauuguugcucagaaaUc.......................................................................  2    1  egg
....................uuuugauGguugcucagaaagc.......................................................................  6    1  egg
....................uuuuAauuguugcucagaaagc.......................................................................  63   1  egg
....................uuuugauugCugcucagaaagc.......................................................................  79   1  egg
....................uuuugCuuguugcucagaaagc.......................................................................  358  1  egg
....................uuGugauuguugcucagaaagc.......................................................................  2    1  egg
....................uuuugauuguugcAcagaaagc.......................................................................  14   1  egg
....................uuuugauuguugcucagaGagc.......................................................................  84   1  egg
....................uuuugauuguuCcucagaaagc.......................................................................  6    1  egg
....................uuuugauuguugcucagUaagc.......................................................................  16   1  egg
....................uuuugauuguugcucaUaaagc.......................................................................  6    1  egg
....................uuuugauuguugcucGgaaagc.......................................................................  59   1  egg
....................uuuugauuguugcucagaaCgc.......................................................................  5    1  egg
....................uuuugauuguugcucagGaagc.......................................................................  79   1  egg
....................uuuugauuguugAucagaaagc.......................................................................  2    1  egg
....................uuuugauuguGgcucagaaagc.......................................................................  6    1  egg
....................uuuugauuguugcucaCaaagc.......................................................................  1    1  egg
....................uuuugauuguugcucagaaaCc.......................................................................  2    1  egg
....................Guuugauuguugcucagaaagc.......................................................................  10   1  egg
```

```
                    Mature                                  Star
uuuaaauguguccucucuacuuuugauuguugcucagaaagccgucaauuaaaugcaggcuuucgagcaauaaucaaaaguugagggucgacaaauaaaaucgucuguauaau
....................uuuugauuAuugcucagaaagc.......................................................................   25      1   egg
....................Auuugauuguugcucagaaagc.......................................................................   16      1   egg
....................uuAugauuguugcucagaaagc.......................................................................   12      1   egg
....................uGuugauuguugcucagaaagc.......................................................................   3       1   egg
....................uuuugauuguugcuGagaaagc.......................................................................   2       1   egg
....................uuuugauuguugcucagaaagA.......................................................................   310     1   egg
....................uuuugauuguugcCcagaaagc.......................................................................   91      1   egg
....................uuuugauuguugcucagaCagc.......................................................................   5       1   egg
....................uuuugauCguugcucagaaagc.......................................................................   57      1   egg
....................uuuugauuguugcucCgaaagc.......................................................................   4       1   egg
....................uuuugauuguugcucagaaUgc.......................................................................   13      1   egg
....................uuuuCauuguugcucagaaagc.......................................................................   32      1   egg
....................uuuugauuguugcGcagaaagc.......................................................................   8       1   egg
....................uuuugauuguCgcucagaaagc.......................................................................   56      1   egg
....................uuuugauuguugcuAagaaagc.......................................................................   3       1   egg
....................uAuugauuguugcucagaaagc.......................................................................   8       1   egg
....................uuuugauugGugcucagaaagc.......................................................................   5       1   egg
....................uuuugauuguugcucagaUagc.......................................................................   17      1   egg
....................uuuugaCuguugcucagaaagc.......................................................................   58      1   egg
....................uuuugauuguugcuUagaaagc.......................................................................   21      1   egg
....................uuuugauAguugcucagaaagc.......................................................................   21      1   egg
....................Cuuugauuguugcucagaaagc.......................................................................   104     1   egg
....................uuuugauuguugcucagCaagc.......................................................................   12      1   egg
....................uuuGgauuguugcucagaaagc.......................................................................   364     1   egg
....................uuuugauuguugcucagaaagc.......................................................................   19716   0   egg
....................uuuugauuguugcucUgaaagc.......................................................................   20      1   egg
....................uuuugauuguAgcucagaaagc.......................................................................   4       1   egg
....................uuuugauuguuUcucagaaagc.......................................................................   18      1   egg
....................uuuugauuguugcucagaaGgc.......................................................................   80      1   egg
....................uuCugauuguugcucagaaagc.......................................................................   70      1   egg
....................uuuugGuuguugcucagaaagc.......................................................................   91      1   egg
....................uuuugauuguugcucagaaagG.......................................................................   8       1   egg
....................uuuugaAuguugcucagaaagc.......................................................................   4       1   egg
....................uuuugauuguugUucagaaagc.......................................................................   29      1   egg
....................uuuugauuguugcucaAaaagc.......................................................................   16      1   egg
....................uuuugauugAugcucagaaagc.......................................................................   11      1   egg
....................uuuugGuuguugcucagaaagcc......................................................................   62      1   egg
....................uuCugauuguugcucagaaagcc......................................................................   44      1   egg
....................uuuugauAguugcucagaaagcc......................................................................   16      1   egg
....................uCuugauuguugcucagaaagcc......................................................................   36      1   egg
....................uuuugauuguCgcucagaaagcc......................................................................   57      1   egg
....................uuuugauuguugcAcagaaagcc......................................................................   17      1   egg
....................uuuugauuguugcucagGaagcc......................................................................   54      1   egg
....................uuuAgauuguugcucagaaagcc......................................................................   64      1   egg
....................uuuugauuguugcucagaaUgcc......................................................................   7       1   egg
....................uuuugauuguugcucagUaagcc......................................................................   13      1   egg
....................uuuugauuguugcucagaCagcc......................................................................   1       1   egg
....................Auuugauuguugcucagaaagcc......................................................................   6       1   egg
....................uuuugauuguugcucagaaagcc......................................................................   13783   0   egg
....................uuuugaCuguugcucagaaagcc......................................................................   46      1   egg
....................Nuuugauuguugcucagaaagcc......................................................................   20      1   egg
....................uuuugauuguuCcucagaaagcc......................................................................   7       1   egg
....................uuuugauuguAgcucagaaagcc......................................................................   6       1   egg
....................uuuugauuguugcucagCaagcc......................................................................   9       1   egg
....................uuuugauugGugcucagaaagcc......................................................................   2       1   egg
....................uuuugauuguugUucagaaagcc......................................................................   27      1   egg
....................uuuugauuUuugcucagaaagcc......................................................................   2       1   egg
....................uuuugauuguugGucagaaagcc......................................................................   2       1   egg
....................uuuugauuguugcuUagaaagcc......................................................................   13      1   egg
....................uuAugauuguugcucagaaagcc......................................................................   10      1   egg
....................uuuugCuuguugcucagaaagcc......................................................................   258     1   egg
....................uuuugauuguugcucUgaaagcc......................................................................   16      1   egg
....................uuuugauuguugcucaAaaagcc......................................................................   16      1   egg
....................uuuugauuguugcucagaaagUc......................................................................   13      1   egg
....................uuuugauuguugcucagaaagcA......................................................................   1179    1   egg
....................uAuugauuguugcucagaaagcc......................................................................   14      1   egg
....................uuuugauuguugcCcagaaagcc......................................................................   63      1   egg
....................uuuugauuguugcucagaUagcc......................................................................   12      1   egg
....................uuuugauuguugcucagaaagcG......................................................................   31      1   egg
....................uuuugUuuguugcucagaaagcc......................................................................   32      1   egg
```

```
                    Mature                                   Star
uuuaaauguguccucucuacuuuugauuguugcucagaaagccgucaauuaaaugcaggcuuucgagcaauaaucaaaaguugagggucgacaaauaaaaucgucuguauaau
....................uuuugauCguugcucagaaagcc......................................................................    46     1    egg
....................uuuugauuAuugcucagaaagcc......................................................................    10     1    egg
....................uuuuUauuguugcucagaaagcc......................................................................    386    1    egg
....................Guuugauuguugcucagaaagcc......................................................................    4      1    egg
....................uuuugauuguugcucagaaCgcc......................................................................    3      1    egg
....................uuuugauuCuugcucagaaagcc......................................................................    1      1    egg
....................uuuugauuguugcucagaaagAc......................................................................    3      1    egg
....................uuuugauuguugcucagaaaCcc......................................................................    1      1    egg
....................uuuugauuguuUcucagaaagcc......................................................................    22     1    egg
....................uuuugauugAugcucagaaagcc......................................................................    6      1    egg
....................uuuugauuguugcucCgaaagcc......................................................................    2      1    egg
....................uuuugauuguuAcucagaaagcc......................................................................    32     1    egg
....................uuuugauuguugcucaUaaagcc......................................................................    4      1    egg
....................uuuuCauuguugcucagaaagcc......................................................................    9      1    egg
....................uuuugauuguugcucagaaagcU......................................................................    940    1    egg
....................uuuugauuguugcuAagaaagcc......................................................................    2      1    egg
....................uGuugauuguugcucagaaagcc......................................................................    4      1    egg
....................uuuugauuguugcuGagaaagcc......................................................................    2      1    egg
....................uuuugauuguGgcucagaaagcc......................................................................    8      1    egg
....................uuuGgauuguugcucagaaagcc......................................................................    269    1    egg
....................uuuugaAuguugcucagaaagcc......................................................................    6      1    egg
....................uuuugauuguugcucagaaGgcc......................................................................    75     1    egg
....................uuuugauugCugcucagaaagcc......................................................................    55     1    egg
....................uuuugauuguugAucagaaagcc......................................................................    3      1    egg
....................uuuugauuguugcucagaGagcc......................................................................    51     1    egg
....................uuuugauuguugcGcagaaagcc......................................................................    1      1    egg
....................uuuugaGuguugcucagaaagcc......................................................................    15     1    egg
....................uuuugauuguugcucGgaaagcc......................................................................    59     1    egg
....................Cuuugauuguugcucagaaagcc......................................................................    79     1    egg
....................uuuugauuguugcucagaaagGc......................................................................    4      1    egg
....................uuuugauuguugcucagaaaAcc......................................................................    15     1    egg
....................uuuugauGguugcucagaaagcc......................................................................    6      1    egg
....................uuuugauuguugcucagaaaUcc......................................................................    4      1    egg
....................uuuCgauuguugcucagaaagcc......................................................................    94     1    egg
....................uuuuAauuguugcucagaaagcc......................................................................    39     1    egg
....................uuuugauuguugcucagaaagccA.....................................................................    756    1    egg
....................Cuuugauuguugcucagaaagccg.....................................................................    1      1    egg
....................uuuugaCuguugcucagaaagccg.....................................................................    2      1    egg
....................uuuGgauuguugcucagaaagccg.....................................................................    1      1    egg
....................uuuugauuguCgcucagaaagccg.....................................................................    1      1    egg
....................uuuugGuuguugcucagaaagccg.....................................................................    1      1    egg
....................uuuugauuguugcucagaaagccU.....................................................................    191    1    egg
....................uuuugUuuguugcucagaaagccg.....................................................................    1      1    egg
....................uuuuUauuguugcucagaaagccg.....................................................................    4      1    egg
....................uuuugauuguugcucagaaagcAg.....................................................................    1      1    egg
....................uCuugauuguugcucagaaagccg.....................................................................    1      1    egg
....................uuuugauuguugcucagaaGgccg.....................................................................    2      1    egg
....................uuuugauuguugcucagaaagccC.....................................................................    34     1    egg
....................uuuugauuguugcucagaaagccg.....................................................................    79     0    egg
....................uuuuUauuguugcucagaaagccgu....................................................................    2      1    egg
....................uuuugauuguugcucagaaagccUu....................................................................    10     1    egg
....................uuuugauuguugcucagaaagccgC....................................................................    1      1    egg
....................uuuugauuguugcucagaaagccgu....................................................................    20     0    egg
....................uuuugCuuguugcucagaaagccgu....................................................................    1      1    egg
....................uuuugauuguugcucagaaagccAu....................................................................    8      1    egg
.....................uuugauuguugcucagaa..........................................................................    13     0    egg
.....................uuugUuuguugcucagaa..........................................................................    1      1    egg
.....................uuugauuguugcucagaUa.........................................................................    1      1    egg
.....................uuugauuguugcucagaaG.........................................................................    1      1    egg
.....................uuugauuguugcucagaaa.........................................................................    1      0    egg
.....................uuugGuuguugcucagaaag........................................................................    1      1    egg
.....................uuugUuuguugcucagaaag........................................................................    2      1    egg
.....................uuugauuguugcucagaaGg........................................................................    1      1    egg
.....................uuugauuUuugcucagaaag........................................................................    1      1    egg
.....................uuugauuguugcucagaaag........................................................................    19     0    egg
.....................uuugauuguuAcucagaaagc.......................................................................    6      1    egg
.....................uuugauuguugcucagaaCgc.......................................................................    3      1    egg
.....................uuugauuguugcucagaaGgc.......................................................................    22     1    egg
.....................uuugauuguugcuGagaaagc.......................................................................    1      1    egg
.....................uuugauuguugcucagaaagU.......................................................................    206    1    egg
```

Mature **Star**

uuuaaauguguccucucuacuuuugauuguugcucagaaagccgucaauuaaaugcaggcuuucgagcaauaaucaaaaguugagggucgacaaauaaaaucgucuguauaau			
.....................Cuugauuguugcucagaaagc...	17	1	egg
.....................uuugauuguAgcucagaaagc...	4	1	egg
.....................Guugauuguugcucagaaagc...	4	1	egg
.....................uuugauuguugUucagaaagc...	9	1	egg
.....................uuugauuguugcucagaaagG...	4	1	egg
.....................uuugauuguGgcucagaaagc...	1	1	egg
.....................uuugauugCugcucagaaagc...	21	1	egg
.....................uuugauuguCgcucagaaagc...	20	1	egg
.....................uuugauuguugcGcagaaagc...	2	1	egg
.....................uuugauuCuugcucagaaagc...	1	1	egg
.....................uuugauugAugcucagaaagc...	4	1	egg
.....................uuugauuguuUcucagaaagc...	1	1	egg
.....................uuugauuguugcucagaUagc...	3	1	egg
.....................uuugauCguugcucagaaagc...	19	1	egg
.....................uuugauuguugcucCgaaagc...	1	1	egg
.....................Nuugauuguugcucagaaagc...	5	1	egg
.....................uuugauuguugcucUgaaagc...	1	1	egg
.....................uuuAauuguugcucagaaagc...	6	1	egg
.....................uuGgauuguugcucagaaagc...	1	1	egg
.....................uuugauuguugcuAagaaagc...	1	1	egg
.....................uuugCuuguugcucagaaagc...	1	1	egg
.....................uuugauuguugcucagUaagc...	3	1	egg
.....................uuCgauuguugcucagaaagc...	13	1	egg
.....................uuugauuguugcAcagaaagc...	6	1	egg
.....................uCugauuguugcucagaaagc...	17	1	egg
.....................uuugauuguugcucagGaagc...	17	1	egg
.....................uuugauuguugcucagaaAc...	4	1	egg
.....................uuuCauuguugcucagaaagc...	1	1	egg
.....................uuugauuguugcucagaCagc...	3	1	egg
.....................uuuUauuguugcucagaaagc...	22	1	egg
.....................uuugauuguugAucagaaagc...	1	1	egg
.....................uuugauAguugcucagaaagc...	8	1	egg
.....................uuugaCuguugcucagaaagc...	16	1	egg
.....................uuugauuguugGucagaaagc...	1	1	egg
.....................uuugauuAuugcucagaaagc...	3	1	egg
.....................uuugaAuguugcucagaaagc...	1	1	egg
.....................Auugauuguugcucagaaagc...	5	1	egg
.....................uuugUuuguugcucagaaagc...	88	1	egg
.....................uuugauuguugcucagaGagc...	26	1	egg
.....................uuugauGguugcucagaaagc...	4	1	egg
.....................uGugauuguugcucagaaagc...	5	1	egg
.....................uAugauuguugcucagaaagc...	5	1	egg
.....................uuugauuguugcuUagaaagc...	5	1	egg
.....................uuugaGuguugcucagaaagc...	1	1	egg
.....................uuugGuuguugcucagaaagc...	60	1	egg
.....................uuugauuguugcucagaaUgc...	1	1	egg
.....................uuugauuguugcCcagaaagc...	23	1	egg
.....................uuugauuguugcucagaaagc...	4888	0	egg
.....................uuugauuguugcucagaaagA...	75	1	egg
.....................uuugauuguuCcucagaaagc...	1	1	egg
.....................uuugauuguugcucagCaagc...	1	1	egg
.....................uuugauuguugcucaAaaagc...	3	1	egg
.....................uuAgauuguugcucagaaagc...	2	1	egg
.....................uuugauuguugcucGgaaagc...	17	1	egg
.....................uuugauuguugcucaUaaagc...	1	1	egg
.....................uuugauuUuugcucagaaagc...	3	1	egg
.....................uuugauCguugcucagaaagcc..	10	1	egg
.....................Nuugauuguugcucagaaagcc..	3	1	egg
.....................uuugGuuguugcucagaaagcc..	40	1	egg
.....................uuugauuguugcCcagaaagcc..	12	1	egg
.....................uuugauuguugGucagaaagcc..	1	1	egg
.....................uGugauuguugcucagaaagcc..	2	1	egg
.....................uuugauuguugcucagaCagcc..	1	1	egg
.....................uuugauuguugcucagaaagcA..	85	1	egg
.....................uuugaCuguugcucagaaagcc..	10	1	egg
.....................uuugauuguuAcucagaaagcc..	5	1	egg
.....................Cuugauuguugcucagaaagcc..	8	1	egg
.....................uuuUauuguugcucagaaagcc..	9	1	egg
.....................uuugauuguugcucaAaaagcc..	3	1	egg
.....................uuugauuguugcucagaaagcU..	164	1	egg

Mature **Star**

uuuaaauguguccucucuacuuugauuguugcucagaaagccgucaauuaaaugcaggcuuucgagcaauaaucaaaaguugagggucgacaaauaaaaucgucuguauaau			
....................uCugauuguugcucagaaagcc..	17	1	egg
....................uuugauuguugcucagaaagGc..	1	1	egg
....................uuugauuguugcucUgaaagcc..	2	1	egg
....................uuugauuguugcucCgaaagcc..	1	1	egg
....................uuugauuguugcucagUaagcc..	3	1	egg
....................uuCgauuguugcucagaaagcc..	10	1	egg
....................uuugauuguugcucagaaCgcc..	1	1	egg
....................uuugauugCugcucagaaagcc..	15	1	egg
....................uuugauuguugcucagaaaAcc..	1	1	egg
....................uuugauuguugUucagaaagcc..	2	1	egg
....................uuugauGguugcucagaaagcc..	6	1	egg
....................uuugUuuguugcucagaaagcc..	58	1	egg
....................uuugauuguuCcucagaaagcc..	3	1	egg
....................uuugauuguugcucagaaagcG..	1	1	egg
....................uuugauuAuugcucagaaagcc..	1	1	egg
....................uuugauuguugcucagaUagcc..	2	1	egg
....................uuugauugAugcucagaaagcc..	2	1	egg
....................uuugauuguugcucagaaGgcc..	7	1	egg
....................uuugauuguugcucagaaUgcc..	3	1	egg
....................uuugauuguugcucagaaagcc..	2418	0	egg
....................uAugauuguugcucagaaagcc..	1	1	egg
....................uuuAauuguugcucagaaagcc..	1	1	egg
....................uuugauuguugcuUagaaagcc..	1	1	egg
....................uuugauuguAgcucagaaagcc..	1	1	egg
....................uuugCuuguugcucagaaagcc..	1	1	egg
....................uuugauuguCgcucagaaagcc..	6	1	egg
....................uuugauuguugcucagaaagUc..	4	1	egg
....................uuugauuguugcucagaaaUcc..	1	1	egg
....................uuugauuguugcAcagaaagcc..	3	1	egg
....................uuugauuguugcucagaGagcc..	9	1	egg
....................uuugauuguugcucGgaaagcc..	5	1	egg
....................uuugauuguugcucagGaagcc..	6	1	egg
....................uuugauuguugAucagaaagcc..	1	1	egg
....................Auugauuguugcucagaaagcc..	2	1	egg
....................uuugauuguugcucagaaGgccg...	29	1	egg
....................uGugauuguugcucagaaagccg...	2	1	egg
....................Guugauuguugcucagaaagccg...	3	1	egg
....................uuAgauuguugcucagaaagccg...	1	1	egg
....................uuugauCguugcucagaaagccg...	23	1	egg
....................uuugauuguugcCcagaaagccg...	23	1	egg
....................uuugauuguugcucagaaagccg...	5620	0	egg
....................uuugauuguGgcucagaaagccg...	1	1	egg
....................uuugauuguugcucagaaagccC...	17	1	egg
....................uuugauGguugcucagaaagccg...	3	1	egg
....................uuugauuguugcucagaaagUcg...	9	1	egg
....................uuugauuguugcucagaGagccg...	15	1	egg
....................uCugauuguugcucagaaagccg...	32	1	egg
....................uuugauuguugcucagaaagccU...	13	1	egg
....................uuuAauuguugcucagaaagccg...	5	1	egg
....................uuugauuguugcucagaaagccA...	298	1	egg
....................uuugauuguugcucagaaagcUg...	9	1	egg
....................uuugauuguugGucagaaagccg...	1	1	egg
....................uAugauuguugcucagaaagccg...	4	1	egg
....................uuugauugAugcucagaaagccg...	6	1	egg
....................uuuUauuguugcucagaaagccg...	21	1	egg
....................Auugauuguugcucagaaagccg...	1	1	egg
....................Nuugauuguugcucagaaagccg...	8	1	egg
....................uuugauuguugcucagGaagccg...	18	1	egg
....................uuugauuguugcucagaaagcGg...	1	1	egg
....................uuugauuguugcucGgaaagccg...	20	1	egg
....................uuugauuguugcAcagaaagccg...	10	1	egg
....................uuugauuAuugcucagaaagccg...	2	1	egg
....................uuugauuguuAcucagaaagccg...	9	1	egg
....................uuugauuguuUcucagaaagccg...	2	1	egg
....................uuugauuguugcucagUaagccg...	6	1	egg
....................uuGgauuguugcucagaaagccg...	1	1	egg
....................uuugauAguugcucagaaagccg...	8	1	egg
....................uuugauuguAgcucagaaagccg...	2	1	egg
....................uuugauuguugcucagaaaUccg...	2	1	egg
....................uuugauuguugcucagaUagccg...	5	1	egg

```
                    Mature                                  Star
uuuaaauguguccucucuacuuuugauuguugcucagaaagccgucaauuaaaugcaggcuuucgagcaauaaucaaaaguugagggucgacaaauaaaaucgucuguauaau
.....................uuugaGuguugcucagaaagccg.....................................................................    2     1    egg
.....................uuCgauuguugcucagaaagccg.....................................................................    19    1    egg
.....................uuugauuguugcucCgaaagccg.....................................................................    1     1    egg
.....................Cuugauuguugcucagaaagccg.....................................................................    29    1    egg
.....................uuugauuguugcucaAaaagccg.....................................................................    4     1    egg
.....................uuugauuguCgcucagaaagccg.....................................................................    15    1    egg
.....................uuugGuuguugcucagaaagccg.....................................................................    64    1    egg
.....................uuugauuguugcucagCaagccg.....................................................................    1     1    egg
.....................uuugauuguugcuUagaaagccg.....................................................................    5     1    egg
.....................uuugauuguugUucagaaagccg.....................................................................    9     1    egg
.....................uuugaAuguugcucagaaagccg.....................................................................    1     1    egg
.....................uuugauuguugcucUgaaagccg.....................................................................    3     1    egg
.....................uuugauuguugcucagaCagccg.....................................................................    2     1    egg
.....................uuugauugCugcucagaaagccg.....................................................................    24    1    egg
.....................uuugUuuguugcucagaaagccg.....................................................................    102   1    egg
.....................uuugaCuguugcucagaaagccg.....................................................................    8     1    egg
.....................uuugauuguugcucagaaUgccg.....................................................................    4     1    egg
.....................uuugauuguugcucagaaaAccg.....................................................................    12    1    egg
.....................uuugGuuguugcucagaaagccgu....................................................................    2     1    egg
.....................uuugauugCugcucagaaagccgu....................................................................    1     1    egg
.....................uuugauuguugcucagaaagccAu....................................................................    3     1    egg
.....................uuugauuguugUucagaaagccgu....................................................................    1     1    egg
.....................uuugauuguugcucagaaagccgC....................................................................    12    1    egg
.....................uuugauuguugcucagaaGgccgu....................................................................    3     1    egg
.....................uuugauAguugcucagaaagccgu....................................................................    1     1    egg
.....................uuugauuguugcucagaaagAcgu....................................................................    1     1    egg
.....................uuugauuguCgcucagaaagccgu....................................................................    2     1    egg
.....................uuugauuguugcucagaaagccgu....................................................................    312   0    egg
.....................uuugauuguugcucagUaagccgu....................................................................    1     1    egg
.....................uuugauuguugcucagaaagccUu....................................................................    2     1    egg
.....................uuugauuguugcucagaaaUccgu....................................................................    2     1    egg
.....................uuugauuguugcucagaaagccgG....................................................................    1     1    egg
.....................uuugauuguugcucagaaaAccgu....................................................................    1     1    egg
.....................Cuugauuguugcucagaaagccgu....................................................................    1     1    egg
.....................uuugauuguugcucagaaagcUgu....................................................................    2     1    egg
.....................uuugauuguugcuUagaaagccgu....................................................................    1     1    egg
.....................uuugauuguugcCcagaaagccgu....................................................................    2     1    egg
.....................uuugauuguugcucagaaagccgA....................................................................    17    1    egg
.....................uuugUuuguugcucagaaagccgu....................................................................    8     1    egg
.....................uuuUauuguugcucagaaagccgu....................................................................    1     1    egg
.....................uuCgauuguugcucagaaagccgu....................................................................    2     1    egg
.....................uuugaCuguugcucagaaagccgu....................................................................    1     1    egg
.....................uuugauuguugcucagaCagccgu....................................................................    1     1    egg
.....................uuugauuguugcucagaaagccguc...................................................................    2     0    egg
.....................uuugauuguugcucagaaagccguG...................................................................    6     1    egg
.....................uuugauuguugcucagaaagccguU...................................................................    10    1    egg
......................uugauuguugcucagaaagc.......................................................................    10    0    egg
......................uugauuguugcucagaaGgc.......................................................................    1     1    egg
......................uugauuguugcucagaaagU.......................................................................    1     1    egg
......................uugauuguugcucagaaagcc......................................................................    4     0    egg
......................uugauuguugcucagaaagccA.....................................................................    5     1    egg
......................uugauuguugcucagaaagccg.....................................................................    4     0    egg
.......................ugauuguugcucagaaagU.......................................................................    1     1    egg
.......................ugauuAuugcucagaaagcc......................................................................    1     1    egg
.............................................................uucgagcaauaaucaaaagu................................    1     0    egg

...................Uuuuugauuguugcucagaaag........................................................................    4     1    T6P
...................Uuuuugauuguugcucagaaagc.......................................................................    35    1    T6P
...................Uuuuugauuguugcucagaaagcc......................................................................    59    1    T6P
....................uuuugauuguugcucaga...........................................................................    32    0    T6P
....................uuuugauuguugcucagaU..........................................................................    6     1    T6P
....................uuuugaCuguugcucagaa..........................................................................    1     1    T6P
....................uuuugaGuguugcucagaa..........................................................................    1     1    T6P
....................uuuugauuguugcucagaa..........................................................................    196   0    T6P
....................uuuugauuguugcucagaG..........................................................................    1     1    T6P
....................uuuugauuguugcucagaaC.........................................................................    1     1    T6P
....................uuuugaCuguugcucagaaa.........................................................................    1     1    T6P
....................uuuugauuguugcucagaUa.........................................................................    7     1    T6P
....................uuuuUauuguugcucagaaa.........................................................................    1     1    T6P
....................uuuugauuguugcucagaaa.........................................................................    131   0    T6P
```

```
                    Mature                                  Star
uuuaaauguguccucucuacuuuugauuguugcucagaaagccgucaauuaaaugcaggcuuucgagcaauaaucaaaaguugagggucgacaaauaaaaucgucuguauaau
....................uuuugauuguugcucagUaa.........................................................................   1     1   T6P
....................uuuugauuguugcucagaaG.........................................................................   5     1   T6P
....................uuuugauuguugcucagaaU.........................................................................   24    1   T6P
....................uuuugUuuguugcucagaaag........................................................................   1     1   T6P
....................uuuuUauuguugcucagaaag........................................................................   1     1   T6P
....................uuuugauuguugcucagaaaA........................................................................   32    1   T6P
....................uuuugauuguugcucagUaag........................................................................   1     1   T6P
....................uuuugauuguugcCcagaaag........................................................................   2     1   T6P
....................uuuugauuguugcucagaaag........................................................................   1591  0   T6P
....................uuuugauugAugcucagaaag........................................................................   1     1   T6P
....................uuuugauuguugUucagaaag........................................................................   1     1   T6P
....................uuuugauuguugcucagaaUg........................................................................   1     1   T6P
....................Guuugauuguugcucagaaag........................................................................   1     1   T6P
....................uuuugauuguuUcucagaaag........................................................................   2     1   T6P
....................uuuugauuCuugcucagaaag........................................................................   1     1   T6P
....................uuuugauuguugcucagaaaU........................................................................   21    1   T6P
....................uuuuAauuguugcucagaaag........................................................................   1     1   T6P
....................uuuugauuguugcucagGaag........................................................................   2     1   T6P
....................uuuugauuguugcucaUaaag........................................................................   3     1   T6P
....................Cuuugauuguugcucagaaag........................................................................   1     1   T6P
....................uuuugauuguugcucagaaaC........................................................................   3     1   T6P
....................uuuugauuguugcucagGaagc.......................................................................   1     1   T6P
....................uuuugauuguugcucagaaagU.......................................................................   40    1   T6P
....................uuAugauuguugcucagaaagc.......................................................................   1     1   T6P
....................Guuugauuguugcucagaaagc.......................................................................   2     1   T6P
....................uuuuUauuguugcucagaaagc.......................................................................   1     1   T6P
....................uuuugGuuguugcucagaaagc.......................................................................   4     1   T6P
....................uuuugauuguugcucagaaagA.......................................................................   238   1   T6P
....................Auuugauuguugcucagaaagc.......................................................................   1     1   T6P
....................uuuugauuguugcucaUaaagc.......................................................................   3     1   T6P
....................uuuugauuguugcucGgaaagc.......................................................................   3     1   T6P
....................uuuugaGuguugcucagaaagc.......................................................................   2     1   T6P
....................uuuugauuguugcuUagaaagc.......................................................................   3     1   T6P
....................uuuugauugCugcucagaaagc.......................................................................   1     1   T6P
....................uuuugaAuguugcucagaaagc.......................................................................   1     1   T6P
....................uuuugauuguugcucaCaaagc.......................................................................   3     1   T6P
....................uuuugauuguugUucagaaagc.......................................................................   11    1   T6P
....................uuuugauuguugcucagaaGgc.......................................................................   3     1   T6P
....................uuuugauuguugcucagCaagc.......................................................................   1     1   T6P
....................uuuugauuguugcucUgaaagc.......................................................................   2     1   T6P
....................uuuugauuguugcCcagaaagc.......................................................................   2     1   T6P
....................uuuugauuguugcucagaaagc.......................................................................   4258  0   T6P
....................uuuugauuAuugcucagaaagc.......................................................................   1     1   T6P
....................uuuugauugGugcucagaaagc.......................................................................   2     1   T6P
....................Cuuugauuguugcucagaaagc.......................................................................   2     1   T6P
....................uuuugauuguugcucagUaagc.......................................................................   3     1   T6P
....................uuuugauuguugcucagaaagG.......................................................................   3     1   T6P
....................uuuugauuguugcGcagaaagc.......................................................................   1     1   T6P
....................uuGugauuguugcucagaaagc.......................................................................   1     1   T6P
....................uuuugauCguugcucagaaagc.......................................................................   1     1   T6P
....................uuuugauuguugcucaAaaagc.......................................................................   3     1   T6P
....................uuuugauuguuUcucagaaagc.......................................................................   1     1   T6P
....................uuuugauuguugcucagaaaCc.......................................................................   2     1   T6P
....................uuuugauuguugcucagaaaUc.......................................................................   1     1   T6P
....................uuuugauGguugcucagaaagcc......................................................................   1     1   T6P
....................uuuugauuguugcucagaaaUcc......................................................................   2     1   T6P
....................uuuugauuguugcucagaaagcA......................................................................   602   1   T6P
....................uuuugUuuguugcucagaaagcc......................................................................   1     1   T6P
....................uuuugauuguugcucagaaagcU......................................................................   110   1   T6P
....................uuuugauuguugcucagaaagcG......................................................................   15    1   T6P
....................uuuugauuguugcucagaaagcc......................................................................   7872  0   T6P
....................Auuugauuguugcucagaaagcc......................................................................   8     1   T6P
....................uuuugauuguugcucaUaaagcc......................................................................   8     1   T6P
....................uuuCgauuguugcucagaaagcc......................................................................   1     1   T6P
....................uuuugauuguuUcucagaaagcc......................................................................   2     1   T6P
....................uuuuAauuguugcucagaaagcc......................................................................   1     1   T6P
....................uuuugauuguugcucagGaagcc......................................................................   6     1   T6P
....................uuuugauuguugcuGagaaagcc......................................................................   1     1   T6P
....................uuuugaGuguugcucagaaagcc......................................................................   6     1   T6P
....................uuuugauuAuugcucagaaagcc......................................................................   1     1   T6P
```

```
                    Mature                  Star
uuuaaauguguccucucuacuuuugauuguugcucagaaagccgucaauuaaaugcaggcuuucgagcaauaaucaaaaguugagggucgacaaauaaaaucgucuguauaau
....................uuuugauuguugcucagaaUgcc......................................................................   3     1    T6P
....................uuuugauuguugcCcagaaagcc......................................................................   1     1    T6P
....................uuuugGuuguugcucagaaagcc......................................................................   2     1    T6P
....................uuuugauuguugcucagaaagUc......................................................................   10    1    T6P
....................uuuugauuguugcuUagaaagcc......................................................................   8     1    T6P
....................uuuGgauuguugcucagaaagcc......................................................................   1     1    T6P
....................Cuuugauuguugcucagaaagcc......................................................................   7     1    T6P
....................uuuugauuguugcucagaUagcc......................................................................   1     1    T6P
....................uuuugauCguugcucagaaagcc......................................................................   1     1    T6P
....................uuuugauuguugcucagUaagcc......................................................................   4     1    T6P
....................uuuugauuguugcucagaaaAcc......................................................................   6     1    T6P
....................uuuugauugAugcucagaaagcc......................................................................   3     1    T6P
....................uuuugauuguuAcucagaaagcc......................................................................   2     1    T6P
....................uuuugauuguugcucagaaaCcc......................................................................   2     1    T6P
....................uAuugauuguugcucagaaagcc......................................................................   1     1    T6P
....................uuuugauuguugcucGgaaagcc......................................................................   3     1    T6P
....................uuuugauugGugcucagaaagcc......................................................................   1     1    T6P
....................uuuugaCuguugcucagaaagcc......................................................................   2     1    T6P
....................uuuugauuguugcucaCaaagcc......................................................................   3     1    T6P
....................Guuugauuguugcucagaaagcc......................................................................   7     1    T6P
....................uuuugauuguugcucUgaaagcc......................................................................   2     1    T6P
....................uuuugauuguugUucagaaagcc......................................................................   6     1    T6P
....................uuuugauuguugcucagaaGgcc......................................................................   13    1    T6P
....................uuuugauugCugcucagaaagcc......................................................................   2     1    T6P
....................uuuugauuguugcucagaaagcAg.....................................................................   2     1    T6P
....................uuuugauuguugcucagaaagccA.....................................................................   288   1    T6P
....................uuuugauuguugcucagaaagccU.....................................................................   121   1    T6P
....................uuuugauuguugcucagaaagcGg.....................................................................   1     1    T6P
....................uuuugauuguugcucagaaagccC.....................................................................   3     1    T6P
....................uuuugauuguugcucagaaagccg.....................................................................   10    0    T6P
....................uuuugauuguugcucagaaagccgu....................................................................   1     0    T6P
....................uuuugauuguugcucagaaagccAu....................................................................   8     1    T6P
.....................uugauuguugcucagaa...........................................................................   1     0    T6P
.....................uugauuguugcucagaUa..........................................................................   1     1    T6P
.....................uugauuguugcucagaaa..........................................................................   6     0    T6P
.....................Augauuguugcucagaaa..........................................................................   1     1    T6P
.....................uugauuguugcucagaaU..........................................................................   1     1    T6P
.....................uugauuguugcucagaaG..........................................................................   1     1    T6P
.....................uugauuguugcucagaaag.........................................................................   37    0    T6P
.....................uugauuguugcucagGaag.........................................................................   1     1    T6P
.....................uugauuguugcucagaaagA........................................................................   14    1    T6P
.....................uugauuguugUucagaaagc........................................................................   1     1    T6P
.....................uugauuguugcucagaaagc........................................................................   266   0    T6P
.....................uugauuguuAcucagaaagc........................................................................   1     1    T6P
.....................uugauuguugcucagaaaUc........................................................................   1     1    T6P
.....................uugauuguugcucagaaagG........................................................................   1     1    T6P
.....................uugauuguugcucagaaagcA.......................................................................   53    1    T6P
.....................uugauuguugcucagGaagcc.......................................................................   1     1    T6P
.....................uugauuguAgcucagaaagcc.......................................................................   1     1    T6P
.....................uugauuguugcucUgaaagcc.......................................................................   1     1    T6P
.....................uugauuguugcucagaaagcc.......................................................................   700   0    T6P
.....................uugauuguugUucagaaagcc.......................................................................   1     1    T6P
.....................uugauuguugcucagaaGgcc.......................................................................   2     1    T6P
.....................uugauuguugcucagaaagcU.......................................................................   7     1    T6P
.....................uugauuguugcucagaaagUc.......................................................................   3     1    T6P
.....................uugauuguugcuUagaaagcc.......................................................................   2     1    T6P
.....................uugauuguuAcucagaaagcc.......................................................................   1     1    T6P
.....................uugauuguugcucagUaagcc.......................................................................   1     1    T6P
.....................uugauuguugcucagaaagccA......................................................................   22    1    T6P
.....................Augauuguugcucagaaagccg......................................................................   1     1    T6P
.....................uugauuguugcuUagaaagccg......................................................................   1     1    T6P
.....................uugauuguugcucagaaagccU......................................................................   14    1    T6P
.....................uugauuguugcucagaaagccg......................................................................   246   0    T6P
.....................uugauuguugcucagaaagUcg......................................................................   1     1    T6P
.....................uugauuguugcucagaaagGcg......................................................................   1     1    T6P
.....................uugauuguugcucagaaagccgu.....................................................................   6     0    T6P
.....................uugauuguugcucagaaagccUu.....................................................................   1     1    T6P
......................ugauuguugcucagaaa..........................................................................   1     0    T6P
......................uuAauuguugcucagaaag........................................................................   1     1    T6P
......................ugauuguugcucagaaagA........................................................................   1     1    T6P
```

```
                    Mature                                  Star
uuuaaauguguccucucuacuuuugauuguugcucagaaagccgucaauuaaaugcagggcuuucgagcaauaaucaaaaguugagggucgacaaauaaaaucgucuguauaau
......................uugauuguugcucagaaagc.......................................................................   4     0   T6P
......................uugauuguugcucagaaagcc......................................................................   19    0   T6P
......................uugauuguugcucagaaagcG......................................................................   1     1   T6P
......................uugauuguugcucagaaagccg.....................................................................   3     0   T6P
......................uugauuguugcucagaaagAcg.....................................................................   1     1   T6P
.......................ugauuguugcucagaaag........................................................................   2     0   T6P
.......................ugauuguugcucagaaagc.......................................................................   18    0   T6P
.......................ugauuguugcucagaaagcc......................................................................   34    0   T6P
.......................ugauuguugcucagaaagccA.....................................................................   1     1   T6P
........................gauuguugcucagaaagc.......................................................................   6     0   T6P
........................gauuguugcucagaaagcc......................................................................   17    0   T6P
........................gauuguugcucagaaagccA.....................................................................   2     1   T6P
........................gauuguugcucagaaagccU.....................................................................   1     1   T6P
.........................auuguugcucagaaagcc......................................................................   1     0   T6P
...........................................................cuuucgagcaauaaucaa....................................   2     0   T6P
...........................................................cuuucgagcaauaaucaaa...................................   29    0   T6P
...........................................................cuuucgagcaauaaucaaaC..................................   1     1   T6P
...........................................................cuuucCagcaauaaucaaaa..................................   1     1   T6P
...........................................................cuAucgagcaauaaucaaaa..................................   1     1   T6P
...........................................................cuuucgagcaauaaucaaaa..................................   86    0   T6P
...........................................................cuuucgagcaauaaucaaaaA.................................   14    1   T6P
...........................................................cuuucgagcaauaaucaaaag.................................   4     0   T6P
...........................................................cuuucgCgcaauaaucaaaagu................................   1     1   T6P
...........................................................cuuucgagcaauaaucaaaagu................................   479   0   T6P
...........................................................Uuuucgagcaauaaucaaaagu................................   1     1   T6P
...........................................................cuuucgagcaauaaucaUaagu................................   1     1   T6P
...........................................................cuuucgaAcaauaaucaaaagu................................   1     1   T6P
...........................................................cuuucgagcaauaaucaaaagA................................   7     1   T6P
...........................................................cuuucUagcaauaaucaaaagu................................   1     1   T6P
...........................................................cuuucgagcaauaaucaaaagAu...............................   1     1   T6P
...........................................................cuuucgagcaauaaucaaaaguA...............................   5     1   T6P
............................................................uuucgagcaauaaucaaaag.................................   5     0   T6P
............................................................uuucgagcaauaaucaaaaUu................................   1     1   T6P
............................................................uuucgagcaauaaucaaaagu................................   173   0   T6P
............................................................uuucgagcaauaaucaaaagA................................   6     1   T6P
............................................................uuucgagcaauaaucaaaaguA...............................   1     1   T6P
.............................................................uucgUgcaauaaucaaaagu................................   1     1   T6P
.............................................................uucgagcaauaaucaaaagu................................   14    0   T6P
.............................................................Aucgagcaauaaucaaaagu................................   1     1   T6P
.................................................................................ugagggucgacaaauaaaau............   1     0   T6P
.................................................................................ugagggucgacaaauaaaaucgucugu.....   1     0   T6P

...................Uuuuugauuguugcucaga...........................................................................   4     1   T53
...................Uuuuugauuguugcucagaa..........................................................................   1     1   T53
...................Uuuuugauuguugcucagaaa.........................................................................   3     1   T53
...................Uuuuugauuguugcucagaaag........................................................................   22    1   T53
...................Uuuuugauuguugcucagaaagc.......................................................................   112   1   T53
...................Auuuugauuguugcucagaaagc.......................................................................   1     1   T53
...................cuuuugauuguugcucagaaagcc......................................................................   2     0   T53
...................Uuuuugauuguugcucagaaagcc......................................................................   482   1   T53
....................uuuugauuguugcucagU...........................................................................   33    1   T53
....................uuuugauuguugcuUaga...........................................................................   1     1   T53
....................uuuugauuguugcCcaga...........................................................................   1     1   T53
....................uuuAgauuguugcucaga...........................................................................   1     1   T53
....................uuuugauuguugcucaga...........................................................................   1565  0   T53
....................uuuugauuguuCcucaga...........................................................................   1     1   T53
....................uuuugauuguugcucagC...........................................................................   1     1   T53
....................uuuugauuguugcucaUa...........................................................................   1     1   T53
....................uuuugauuAuugcucaga...........................................................................   1     1   T53
....................Auuugauuguugcucaga...........................................................................   4     1   T53
....................uuuugauAguugcucaga...........................................................................   1     1   T53
....................uuuugUuuguugcucaga...........................................................................   1     1   T53
....................uuuuUauuguugcucaga...........................................................................   2     1   T53
....................uuuugauuguugcAcaga...........................................................................   1     1   T53
....................uuuugauuguugcucaAa...........................................................................   2     1   T53
....................Guuugauuguugcucaga...........................................................................   2     1   T53
....................uuuugauuguugcGcaga...........................................................................   1     1   T53
....................uuuugauugCugcucaga...........................................................................   1     1   T53
....................uuuugauuguugcucagG...........................................................................   3     1   T53
....................uuuugauuguugcucagaa..........................................................................   207   0   T53
```

Mature **Star**

uuuaaauguguccucucuacuuuugauuguugcucagaaagccgucaauuaaaugcaggcuuucgagcaauaaucaaaaguugagggucgacaaauaaaaucgucuguauaau			
....................uuuugauuguugcucagaC..	3	1	T53
....................uuuugauuguugcucagUa..	2	1	T53
....................uuuugauuguugcucagGa..	4	1	T53
....................uuuugauuguugcucagaU..	31	1	T53
....................uuuAgauuguugcucagaaa...	1	1	T53
....................uuuugauuguugcucagaaG...	22	1	T53
....................Cuuugauuguugcucagaaa...	1	1	T53
....................uuuugauuguugcucagaaa...	472	0	T53
....................uuuugauuguugcucagaaC...	29	1	T53
....................uuuugauuguugcucagGaa...	1	1	T53
....................Auuugauuguugcucagaaa...	1	1	T53
....................uuuugauuguugcucUgaaa...	1	1	T53
....................uuuugauuguugcucagaGa...	1	1	T53
....................uuuugauuguugcucagaCa...	7	1	T53
....................uuuugauuguugcucagaUa...	42	1	T53
....................uuuugauuguugcucagaaU...	97	1	T53
....................Guuugauuguugcucagaaa...	1	1	T53
....................uuuugauuguugcAcagaaa...	1	1	T53
....................uuuugauuguugUucagaaa...	1	1	T53
....................uuuugauuAuugcucagaaa...	1	1	T53
....................Auuugauuguugcucagaaag..	23	1	T53
....................uuuugaCuguugcucagaaag..	1	1	T53
....................uuuugauuguugcuGagaaag..	1	1	T53
....................uuuugauuguugUucagaaag..	4	1	T53
....................uuuugauuguugcucagaUag..	2	1	T53
....................uuuugauuguugcucagUaag..	3	1	T53
....................uuuugauuguuAcucagaaag..	2	1	T53
....................uuuugauuAuugcucagaaag..	7	1	T53
....................uuuugauuguugGucagaaag..	1	1	T53
....................uuuugauuguugcuUagaaag..	3	1	T53
....................uuuugauuguugcAcagaaag..	2	1	T53
....................Cuuugauuguugcucagaaag..	4	1	T53
....................uuuugauAguugcucagaaag..	1	1	T53
....................uuuugauuguugcucagaaaU..	59	1	T53
....................uuuugauuguugcucagaaCg..	1	1	T53
....................uuuugauuguugcucagaaaA..	204	1	T53
....................uuuugauuguugcucaAaaag..	1	1	T53
....................uuuugauuguugcucagaaGg..	16	1	T53
....................uuuugauuguugcucagaaag..	5734	0	T53
....................uuuugUuuguugcucagaaag..	1	1	T53
....................uuuugauCguugcucagaaag..	1	1	T53
....................uCuugauuguugcucagaaag..	1	1	T53
....................uuuugauGguugcucagaaag..	1	1	T53
....................uuuugauuguugcucagaGag..	2	1	T53
....................uuuugauuguugcCcagaaag..	1	1	T53
....................uuuugauuguugcucagGaag..	1	1	T53
....................uuuAgauuguugcucagaaag..	1	1	T53
....................uuuugaAuguugcucagaaag..	2	1	T53
....................uuuugCuuguugcucagaaag..	1	1	T53
....................Guuugauuguugcucagaaag..	5	1	T53
....................uuuugauugAugcucagaaag..	1	1	T53
....................uuuugauuguugcucaCaaag..	1	1	T53
....................uuuugauuguugcucagaaaC..	14	1	T53
....................uuuugauugCugcucagaaag..	2	1	T53
....................uuuugauuguAgcucagaaagc...	1	1	T53
....................uuuugauuguugcAcagaaagc...	12	1	T53
....................uuuugauuCuugcucagaaagc...	4	1	T53
....................uuuugauuguugcucagaaaAc...	3	1	T53
....................uuuugauuguugcucaAaaagc...	2	1	T53
....................uuuugGuuguugcucagaaagc...	4	1	T53
....................uuuugauuguugcucagaaGgc...	26	1	T53
....................uuuugauuguCgcucagaaagc...	1	1	T53
....................uuuugauuguugcucagaaagG...	37	1	T53
....................uuAugauuguugcucagaaagc...	3	1	T53
....................uuuugauuguuUcucagaaagc...	1	1	T53
....................uuuugauuguugcuAagaaagc...	2	1	T53
....................uuuugauuguugcucaCaaagc...	2	1	T53
....................uuuugaAuguugcucagaaagc...	3	1	T53
....................uuuuUauuguugcucagaaagc...	1	1	T53
....................uuuugauuguugGucagaaagc...	1	1	T53

```
                    Mature                                 Star
uuuaaauguguccucucuacuuuugauuguugcucagaaagccgucaauuaaaugcaggcuuucgagcaauaaucaaaaguugagggucgacaaauaaaaucgucuguauaau
....................uuuugauuguugUucagaaagc.......................................................................  12     1  T53
....................uuuugauuguugcucagUaagc.......................................................................  5      1  T53
....................uuuugauuguugcucagaaaCc.......................................................................  3      1  T53
....................Guuugauuguugcucagaaagc.......................................................................  16     1  T53
....................uuuugauuAuugcucagaaagc.......................................................................  13     1  T53
....................uuuugauuguugcucagaaagU.......................................................................  299    1  T53
....................uuuugauuguugcucaUaaagc.......................................................................  2      1  T53
....................uuuugUuuguugcucagaaagc.......................................................................  1      1  T53
....................uuuugauuguugcucagGaagc.......................................................................  9      1  T53
....................Cuuugauuguugcucagaaagc.......................................................................  15     1  T53
....................uuuugauuguugcucagaaUgc.......................................................................  4      1  T53
....................uuuugauuguugcucagaaagA.......................................................................  1042   1  T53
....................uuuugauuguuCcucagaaagc.......................................................................  2      1  T53
....................uuuugauAguugcucagaaagc.......................................................................  14     1  T53
....................uuuugauuguugAucagaaagc.......................................................................  1      1  T53
....................uGuugauuguugcucagaaagc.......................................................................  1      1  T53
....................uCuugauuguugcucagaaagc.......................................................................  1      1  T53
....................uuuugauuguugcucagaUagc.......................................................................  19     1  T53
....................Auuugauuguugcucagaaagc.......................................................................  46     1  T53
....................uuuugauugCugcucagaaagc.......................................................................  7      1  T53
....................uuuuCauuguugcucagaaagc.......................................................................  2      1  T53
....................uuuugauuguugcuUagaaagc.......................................................................  4      1  T53
....................uuuugaCuguugcucagaaagc.......................................................................  3      1  T53
....................uuuugauuguugcucagCaagc.......................................................................  1      1  T53
....................uuuugauuguuAcucagaaagc.......................................................................  8      1  T53
....................uuuuAauuguugcucagaaagc.......................................................................  2      1  T53
....................uuuugauuguugcucagaGagc.......................................................................  2      1  T53
....................uuuCgauuguugcucagaaagc.......................................................................  2      1  T53
....................uuuugauuguugcucagaaaUc.......................................................................  6      1  T53
....................uuuugCuuguugcucagaaagc.......................................................................  3      1  T53
....................uuuugauGguugcucagaaagc.......................................................................  2      1  T53
....................uuuugauuguugcucagaCagc.......................................................................  3      1  T53
....................uuuAgauuguugcucagaaagc.......................................................................  8      1  T53
....................uuuugauuguugcCcagaaagc.......................................................................  3      1  T53
....................uAuugauuguugcucagaaagc.......................................................................  1      1  T53
....................uuuugauuguugcucagaaCgc.......................................................................  1      1  T53
....................uuuugauuguugcucUgaaagc.......................................................................  3      1  T53
....................uuuugauuguugcucagaaagc.......................................................................  18695  0  T53
....................uuuugUuuguugcucagaaagcc......................................................................  15     1  T53
....................uuuugauuguugcucagaaagcU......................................................................  445    1  T53
....................uuuugauuguuCcucagaaagcc......................................................................  5      1  T53
....................uAuugauuguugcucagaaagcc......................................................................  2      1  T53
....................uuuugauuguugcucagaaaUcc......................................................................  21     1  T53
....................uuuugauuguugcGcagaaagcc......................................................................  4      1  T53
....................uuuugauuguugcucagaaUgcc......................................................................  71     1  T53
....................uuuugauuguugGucagaaagcc......................................................................  3      1  T53
....................uuuugauAguugcucagaaagcc......................................................................  21     1  T53
....................uuuugauuguugcucagaaagcG......................................................................  115    1  T53
....................uuuugauuguugcucagaGagcc......................................................................  10     1  T53
....................uuuugauuguugcuGagaaagcc......................................................................  2      1  T53
....................uuuugauuguugcuAagaaagcc......................................................................  1      1  T53
....................uuuugauuguugcCcagaaagcc......................................................................  18     1  T53
....................uuuugauuUuugcucagaaagcc......................................................................  3      1  T53
....................uuuugauuguCgcucagaaagcc......................................................................  3      1  T53
....................uuuugauuguugcucaAaaagcc......................................................................  9      1  T53
....................uuuugauuguugcucagaCagcc......................................................................  6      1  T53
....................uuuugauuguugcucagUaagcc......................................................................  34     1  T53
....................uuuugauuguugcucagaaGgcc......................................................................  366    1  T53
....................uuuugauuguugcucagGaagcc......................................................................  55     1  T53
....................uuuugaGuguugcucagaaagcc......................................................................  3      1  T53
....................uuuugauuguugcucagaaagcc......................................................................  76728  0  T53
....................uuuugCuuguugcucagaaagcc......................................................................  6      1  T53
....................uuuugauuAuugcucagaaagcc......................................................................  35     1  T53
....................uuuAgauuguugcucagaaagcc......................................................................  28     1  T53
....................uuuugauuguugUucagaaagcc......................................................................  15     1  T53
....................uuuuCauuguugcucagaaagcc......................................................................  4      1  T53
....................uuCugauuguugcucagaaagcc......................................................................  2      1  T53
....................uuuugauuguugcucaUaaagcc......................................................................  7      1  T53
....................uuuugauuguugcucUgaaagcc......................................................................  10     1  T53
....................uuuugauuguGgcucagaaagcc......................................................................  1      1  T53
```

```
                    Mature                 Star
uuuaaaugugucccucucuacuuuugauuguugcucagaaagccgucaauuaaaugcaggcuuucgagcaauaaucaaaaguugagggucgacaaauaaaaucgucuguauaau
....................uuuugauuguugcucagaaCgcc......................................................................  22    1  T53
....................uuuugauuguugcucagaaagAc......................................................................  9     1  T53
....................Cuuugauuguugcucagaaagcc......................................................................  49    1  T53
....................uuuuAauuguugcucagaaagcc......................................................................  1     1  T53
....................uuuugauuguAgcucagaaagcc......................................................................  1     1  T53
....................uuuGgauuguugcucagaaagcc......................................................................  6     1  T53
....................uuuugauuguugcuUagaaagcc......................................................................  30    1  T53
....................uuuugauugAugcucagaaagcc......................................................................  1     1  T53
....................uuuugauuguugcucagCaagcc......................................................................  2     1  T53
....................uuuugaCuguugcucagaaagcc......................................................................  5     1  T53
....................uuuugauugCugcucagaaagcc......................................................................  30    1  T53
....................uuuugauuCuugcucagaaagcc......................................................................  3     1  T53
....................uuAugauuguugcucagaaagcc......................................................................  4     1  T53
....................uuuugauGguugcucagaaagcc......................................................................  3     1  T53
....................uGuugauuguugcucagaaagcc......................................................................  1     1  T53
....................uuuugauuguugcucagaUagcc......................................................................  32    1  T53
....................uuuugaAuguugcucagaaagcc......................................................................  8     1  T53
....................uuuugauuguugcucaCaaagcc......................................................................  7     1  T53
....................uuuugauuguugcucagaaagGc......................................................................  2     1  T53
....................uuuugauuguuAcucagaaagcc......................................................................  19    1  T53
....................uuuugGuuguugcucagaaagcc......................................................................  19    1  T53
....................Auuugauuguugcucagaaagcc......................................................................  98    1  T53
....................uuuugauuguugcucagaaagcA......................................................................  1204  1  T53
....................uuuuUauuguugcucagaaagcc......................................................................  4     1  T53
....................uuuugauCguugcucagaaagcc......................................................................  9     1  T53
....................uuuugauuguugcucagaaaCcc......................................................................  11    1  T53
....................uuuugauuguugAucagaaagcc......................................................................  3     1  T53
....................uuuugauuguugcucagaaaAcc......................................................................  42    1  T53
....................uCuugauuguugcucagaaagcc......................................................................  4     1  T53
....................Guuugauuguugcucagaaagcc......................................................................  54    1  T53
....................uuuugauuguugcucagaaagUc......................................................................  142   1  T53
....................uuuCgauuguugcucagaaagcc......................................................................  3     1  T53
....................uuuugauuguugcAcagaaagcc......................................................................  23    1  T53
....................uuuugauuguuUcucagaaagcc......................................................................  1     1  T53
....................uuuugauuguugcucagaaagccU.....................................................................  975   1  T53
....................Guuugauuguugcucagaaagccg.....................................................................  1     1  T53
....................uuuugauuguugcucagaaagccA.....................................................................  1189  1  T53
....................uuuugauuguugcucagaaagcGg.....................................................................  1     1  T53
....................uuuugauuguugcucagaaagcUg.....................................................................  1     1  T53
....................uuuugauuguugcucagaaagccC.....................................................................  24    1  T53
....................uuuugauuguugcucagaaagccg.....................................................................  54    0  T53
....................uuuugauuguugcucagaaagcAg.....................................................................  1     1  T53
....................uuuugauuguugcucagaaagccAu....................................................................  26    1  T53
....................uuuugauuguugcucagaaagccUu....................................................................  6     1  T53
....................uuuugauuguugcucagaaagccCu....................................................................  4     1  T53
....................uuuugauuguugcucagaaagccgu....................................................................  2     0  T53
....................uuuugauuAuugcucagaaagccgu....................................................................  1     1  T53
....................uuuugauuguugcucagaaagccguU...................................................................  1     1  T53
....................uuuugauuguugcucagaaagccUuc...................................................................  1     1  T53
.....................uuugauuguugcucagaa..........................................................................  6     0  T53
.....................uuugauuguugcucagCa..........................................................................  1     1  T53
.....................uuugauuguugcucagaGa.........................................................................  1     1  T53
.....................uuugauuguugcucagaaC.........................................................................  3     1  T53
.....................uuugauuguugcucagaaU.........................................................................  5     1  T53
.....................uuugauuguugcucagaaa.........................................................................  34    0  T53
.....................uuugauuguugcucagaCa.........................................................................  1     1  T53
.....................uuugauuguugcucagaUa.........................................................................  2     1  T53
.....................uuugauuguugcucUgaaag........................................................................  1     1  T53
.....................uuugauuguugcucagaaaC........................................................................  1     1  T53
.....................uuugauuguugcucagaaag........................................................................  146   0  T53
.....................uuugauuguugcucagaaaA........................................................................  5     1  T53
.....................Guugauuguugcucagaaag........................................................................  1     1  T53
.....................uuugauuguugcucagaaaU........................................................................  2     1  T53
.....................uAugauuguugcucagaaagc.......................................................................  1     1  T53
.....................uuugauuguugcucagaGagc.......................................................................  1     1  T53
.....................uuugauuguugcucagaaagU.......................................................................  6     1  T53
.....................uuugauuguugcucUgaaagc.......................................................................  1     1  T53
.....................uuugauuguugcucagaaGgc.......................................................................  3     1  T53
.....................uuugauuguugcucagaaagc.......................................................................  1131  0  T53
.....................uuugauuguugcucagaaUgc.......................................................................  1     1  T53
```

```
                    Mature                                  Star
uuuaaauguguccucucuacuuuugauuguugcucagaaagccgucaauuaaaugcaggcuuucgagcaauaaucaaaaguugagggucgacaaauaaaaucgucuguauaau
.....................Auugauuguugcucagaaagc.......................................................................    4     1    T53
.....................uuugauuguugcucagaaagG.......................................................................    1     1    T53
.....................uuugauuguugcucagaaagA.......................................................................    22    1    T53
.....................uuugauuguugcucagUaagc.......................................................................    1     1    T53
.....................uuugauuguugcuAagaaagcc......................................................................    1     1    T53
.....................uuugauuguuCcucagaaagcc......................................................................    2     1    T53
.....................uCugauuguugcucagaaagcc......................................................................    2     1    T53
.....................uuugauuguugcucagaaaAcc......................................................................    1     1    T53
.....................Auugauuguugcucagaaagcc......................................................................    8     1    T53
.....................Cuugauuguugcucagaaagcc......................................................................    5     1    T53
.....................uuugaGuguugcucagaaagcc......................................................................    1     1    T53
.....................uuugauuguCgcucagaaagcc......................................................................    2     1    T53
.....................uuugauuguugcucagaaagcG......................................................................    8     1    T53
.....................uuugauuguugcucagaaagcU......................................................................    28    1    T53
.....................uuugauuCuugcucagaaagcc......................................................................    1     1    T53
.....................uuugauuguuAcucagaaagcc......................................................................    2     1    T53
.....................uuugauuguugcucagaaagcc......................................................................    7447  0    T53
.....................uuuUauuguugcucagaaagcc......................................................................    3     1    T53
.....................uuugaCuguugcucagaaagcc......................................................................    2     1    T53
.....................uuugauuguugcucagaaGgcc......................................................................    16    1    T53
.....................uuugauuguugcucagaaCgcc......................................................................    1     1    T53
.....................uuugauuguugcucagUaagcc......................................................................    5     1    T53
.....................uuugauuguugcucagaaagGc......................................................................    2     1    T53
.....................uuugauuguugcucUgaaagcc......................................................................    6     1    T53
.....................uuugUuuguugcucagaaagcc......................................................................    1     1    T53
.....................uuugaAuguugcucagaaagcc......................................................................    1     1    T53
.....................uuugauuguugcucagGaagcc......................................................................    10    1    T53
.....................uuugauuguugcucagaaUgcc......................................................................    15    1    T53
.....................uuugauuguugcucagaaaUcc......................................................................    1     1    T53
.....................uuugauuguugcucagaUagcc......................................................................    1     1    T53
.....................uuugauugAugcucagaaagcc......................................................................    8     1    T53
.....................uuugauuguugcucagCaagcc......................................................................    2     1    T53
.....................uuAgauuguugcucagaaagcc......................................................................    1     1    T53
.....................uuugGuuguugcucagaaagcc......................................................................    1     1    T53
.....................uuugauAguugcucagaaagcc......................................................................    2     1    T53
.....................uuugauuguugcucGgaaagcc......................................................................    1     1    T53
.....................uuugauuguugcucagaaagUc......................................................................    10    1    T53
.....................uuugauuguugcucagaaagcA......................................................................    82    1    T53
.....................uuugauuguugcucagaaaCcc......................................................................    1     1    T53
.....................uuugauuguugUucagaaagcc......................................................................    2     1    T53
.....................Guugauuguugcucagaaagcc......................................................................    1     1    T53
.....................uuugauuguugcucagaaagcGg.....................................................................    1     1    T53
.....................uuugauugAugcucagaaagccg.....................................................................    1     1    T53
.....................uuugauuguugcucagaaaAccg.....................................................................    1     1    T53
.....................uuugauAguugcucagaaagccg.....................................................................    1     1    T53
.....................uuugauuguugcucagCaagccg.....................................................................    1     1    T53
.....................uuugaCuguugcucagaaagccg.....................................................................    1     1    T53
.....................uuugauuguugcucagaaagccC.....................................................................    4     1    T53
.....................Auugauuguugcucagaaagccg.....................................................................    2     1    T53
.....................uuugauuCuugcucagaaagccg.....................................................................    1     1    T53
.....................uuugauuguugcCcagaaagccg.....................................................................    1     1    T53
.....................uuugauuguugcucagaaagUcg.....................................................................    4     1    T53
.....................uuugaGuguugcucagaaagccg.....................................................................    1     1    T53
.....................uuugauuguugcucagUaagccg.....................................................................    1     1    T53
.....................uuugauuguugcAcagaaagccg.....................................................................    1     1    T53
.....................uuugauuAuugcucagaaagccg.....................................................................    1     1    T53
.....................uuugauuguugcucagaaUgccg.....................................................................    3     1    T53
.....................uuugauuguugcucagaaagccA.....................................................................    170   1    T53
.....................uuugauuguugcucagaaagccU.....................................................................    103   1    T53
.....................uuugauuguugcucagaaGgccg.....................................................................    2     1    T53
.....................uuugauuguugcucagaaagccg.....................................................................    1518  0    T53
.....................uuCgauuguugcucagaaagccg.....................................................................    1     1    T53
.....................uuugauuguugcuAagaaagccg.....................................................................    1     1    T53
.....................Cuugauuguugcucagaaagccg.....................................................................    1     1    T53
.....................uuugUuuguugcucagaaagccg.....................................................................    1     1    T53
.....................uuugauuguugcucagaaagccAu....................................................................    9     1    T53
.....................uuugauuguugcucagaaagccUu....................................................................    8     1    T53
.....................uuugauuguugcucagaaagccgA....................................................................    3     1    T53
.....................uuugauuguugcucagaaagccgu....................................................................    37    0    T53
.....................uuugauuguugcucagaaagccguU...................................................................    15    1    T53
```

```
                    Mature                                  Star
uuuaaauguguccucucuacuuuugauuguugcucagaaagccgucaauuaaaugcaggcuuucgagcaauaaucaaaaguugagggucgacaaauaaaaucgucuguauaau
......................uugauuguugcucagaaag........................................................................  2    0  T53
......................uugauuguugcucagaaagc.......................................................................  10   0  T53
......................uugauuguugcucagaaagA.......................................................................  3    1  T53
......................Augauuguugcucagaaagc.......................................................................  1    1  T53
......................uugauuguugcucagaaagcc......................................................................  61   0  T53
......................uugauuguugcucagaaagcA......................................................................  2    1  T53
......................uugauuguugcucagaaagccU.....................................................................  3    1  T53
......................uugauuguugcucagaaagccA.....................................................................  3    1  T53
......................uugauuguugcucagaaagccg.....................................................................  5    0  T53
......................uugauuguugcucagaaagccC.....................................................................  4    1  T53
.......................ugauuguugcucagaaagc.......................................................................  4    0  T53
.......................Agauuguugcucagaaagc.......................................................................  1    1  T53
.......................ugauuguugcucagaaagcc......................................................................  43   0  T53
.......................ugauuguugcucagaaagcU......................................................................  1    1  T53
.......................ugauuguugcucagaaagccA.....................................................................  1    1  T53
........................gauuguugcucagaaagc.......................................................................  4    0  T53
........................gauuguugcucagaaagcc......................................................................  12   0  T53
........................gauuguugcuUagaaagcc......................................................................  1    1  T53
........................gauuguugcucagaaagccU.....................................................................  1    1  T53
.........................auuguugcucagaaagcc......................................................................  1    0  T53
...........................................................cuuucgagcaauaaucaa....................................  126  0  T53
...........................................................cuuucgGgcaauaaucaa....................................  1    1  T53
...........................................................cuuucgagcaauaaucaaa...................................  157  0  T53
...........................................................cuuucgagcaauaaucaaaU..................................  3    1  T53
...........................................................cuuucgagcaauaaucaaaa..................................  97   0  T53
...........................................................cuuucgagcaauaaucaaaaC.................................  1    1  T53
...........................................................cuuucgagcaauaaucaaaaU.................................  1    1  T53
...........................................................cuuucgagcaauaaucaaaaA.................................  25   1  T53
...........................................................cuuucgagcaauaaucaaaag.................................  3    0  T53
...........................................................cuuucgagcaauaaucaaaGgu................................  1    1  T53
...........................................................cuuucgagcaauaaucaaaaAu................................  1    1  T53
...........................................................cuuucgagcaauaaucaaaagA................................  5    1  T53
...........................................................cuuucgagcaauaaucaaaagu................................  149  0  T53
...........................................................cuuucgagcaauaaucaaaaguu...............................  2    0  T53
...........................................................cuuucgagcaauaaucaaaaguA...............................  4    1  T53
............................................................uuucgagcaauaaucaaaag.................................  17   0  T53
............................................................uuucgagcaauaUucaaaagu................................  1    1  T53
............................................................uuucgagcaauaauGaaaagu................................  1    1  T53
............................................................uuucgagUaauaaucaaaagu................................  1    1  T53
............................................................uuucgagcaauaaucaaaagA................................  6    1  T53
............................................................uuucgagcaauaaucaaaUgu................................  1    1  T53
............................................................Guucgagcaauaaucaaaagu................................  1    1  T53
............................................................uuucgaAcaauaaucaaaagu................................  1    1  T53
............................................................uuucgagcaauaaAcaaaagu................................  1    1  T53
............................................................uuucgUgcaauaaucaaaagu................................  1    1  T53
............................................................uuucgagcaauaaucaaaagG................................  1    1  T53
............................................................uuucgagcaauaaucaGaagu................................  1    1  T53
............................................................uuucgagcaauaaucaaaagu................................  910  0  T53
............................................................Cuucgagcaauaaucaaaagu................................  1    1  T53
............................................................uuucgagcaauaaucaaaaguu...............................  6    0  T53
............................................................uuucgagcaauaaucaaaaguA...............................  6    1  T53
.............................................................uucgagcaauaaucaaaagu................................  4    0  T53
..............................................................ucgagcaauaaucaaaagu................................  2    0  T53
.................................................................................uugagggucgacaaauaaa.............  1    0  T53
```

Provisional ID	:	Scaffold_1043_3131
Score total	:	456.8
Score for star read(s)	:	3.9
Score for read counts	:	446.7
Score for mfe	:	1.6
Score for randfold	:	1.6
Score for cons. seed	:	3
Total read count	:	888
Mature read count	:	873
Loop read count	:	0
Star read count	:	15

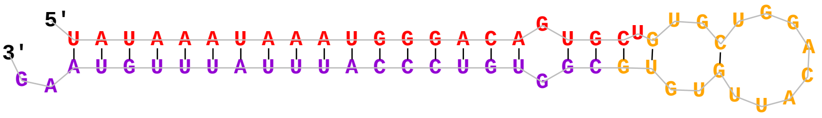

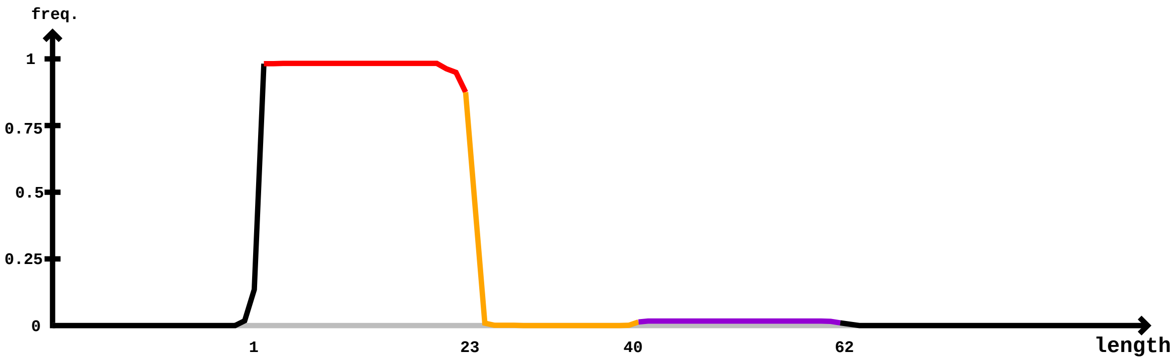


```
                       Mature                           Star
5'- auucaguucuuuuauaauuguauaaauaaaugggacagugcugugcuggacauugugugcggugucccauuuauuuguaagauuauaauuugagacacacaucgaaauacuu  -3'   obs
    auucaguucuuuuauaauuguauaaauaaaugggacagugcugugcuggacauugugugcggugucccauuuauuuguaagauuauaauuugagacacacaucgaaauacuu        exp
    .....((((((((((((((.(((((((((((((((((.(((.(..(........)..)))).))))))))))))))))).))))))))...)))).))..............  reads  mm  sample
    ..................uguauaaauaaaugggacagu.........................................................................  3      0   T6P
    ...................guauaaauaaaugggacagug........................................................................  1      0   T6P
    ...................guauaaauaaaugggacagugc.......................................................................  5      0   T6P
    ...................guauaaauaaaugggacagugcu......................................................................  9      0   T6P
    ....................uauaaauaaaugggacagu.........................................................................  1      0   T6P
    ....................uauaaauaaaugggacagugc.......................................................................  4      0   T6P
    ....................uauaaauaaGugggacagugcu......................................................................  2      1   T6P
    ....................uauaaauaaauggAacagugcu......................................................................  1      1   T6P
    ....................uauaaauaaaugggacagugcA......................................................................  1      1   T6P
    ....................uauaaauGaaugggacagugcu......................................................................  4      1   T6P
    ....................Cauaaauaaaugggacagugcu......................................................................  1      1   T6P
    ....................uauaaaGaaaugggacagugcu......................................................................  1      1   T6P
    ....................uauaaauaaaugggacagugcu......................................................................  64     0   T6P
    ....................uauaaauaGaugggacagugcu......................................................................  1      1   T6P
    ....................uauaaauaaaugggacagugcuU.....................................................................  1      1   T6P
    ......................uaaauaaaugggacagugcu......................................................................  1      0   T6P
    ...........................................................cggugucccauuuauuuguaa................................  1      0   T6P
    ...........................................................cggugucccauuuauuuguaag...............................  1      0   T6P
    ...........................................................cggugucccauuuauuuguaaga..............................  1      0   T6P
    ............................................................ggugucccauuuauuuguaaga..............................  1      0   T6P

    ..................uguauaaauaaaugggacagug........................................................................  1      0   egg
    ..................uguauaaauaaaugggacagugc.......................................................................  1      0   egg
    ..................uguauaaauaaauggUacagugc.......................................................................  1      1   egg
    ...................guauaaauaaaugggacagu.........................................................................  3      0   egg
    ...................guauaaauaaaugggacagugc.......................................................................  5      0   egg
    ...................guauaaauaaaugggacagugcu......................................................................  1      0   egg
    ....................uauaaauaaaugggacagugc.......................................................................  1      0   egg
    ....................uauaaCuaaaugggacagugcu......................................................................  1      1   egg
    ....................uauaaauaaaugggacaCugcu......................................................................  1      1   egg
    ....................uauaaauaaaugggacaguAcu......................................................................  1      1   egg
    ....................uauaaauaaaugggacagugcu......................................................................  43     0   egg
    ....................uauaaauaaaugggacagugcA......................................................................  1      1   egg
```

Mature **Star**

```
auucaguucuuuuauaauuguauaaauaaaugggacagugcugugcuggacauugugugcggugucccauuuauuuguaagauuauaauuugagacacacaucgaaauacuu
....................uauaaauaaaugggacagugcC......................................................................   2    1   egg
....................uauaaauaaaugggacagugcuA.....................................................................   2    1   egg

..................uguauaaauaaaugggacaguU........................................................................   1    1   te1
...................guauaaauaaaugggacagu.........................................................................   3    0   te1
...................guauaaauaaaugggacagug........................................................................   3    0   te1
...................guauaaauaaaugggacagugc.......................................................................   3    0   te1
...................guauaaauaGaugggacagugcu......................................................................   1    1   te1
...................guauaaauaaaugggacagugcu......................................................................   18   0   te1
...................guauaaauaaaugggacagugcuU.....................................................................   2    1   te1
...................guauaaauaaaugggacagugcuA.....................................................................   1    1   te1
....................uauaaauaaaugggacagu.........................................................................   1    0   te1
....................uauaaauaaGugggacagug........................................................................   1    1   te1
....................uauaaauaaaugggacagug........................................................................   1    0   te1
....................uauaaauaaaugggacagugc.......................................................................   9    0   te1
....................uauaaauaaaugggacagugcu......................................................................   162  0   te1
....................Gauaaauaaaugggacagugcu......................................................................   2    1   te1
....................uauaGauaaaugggacagugcu......................................................................   1    1   te1
....................uauaaauaGaugggacagugcu......................................................................   5    1   te1
....................uauaaauaaaugAgacagugcu......................................................................   1    1   te1
....................Cauaaauaaaugggacagugcu......................................................................   2    1   te1
....................uauaaauaaGugggacagugcu......................................................................   4    1   te1
....................uauaaauGaaugggacagugcu......................................................................   5    1   te1
....................uauaaauaaaugggacagugcuU.....................................................................   6    1   te1
....................uauaaauaaaugggacagugcuA.....................................................................   3    1   te1
....................uauaaauaaaugggacagugcuAu....................................................................   1    1   te1
..........................................................gcggugucccauuuGuuuguaa................................   1    1   te1
...........................................................cggugucccauuuauuuguaa................................   3    0   te1
...........................................................cggugucccauuuauuuguaag...............................   2    0   te1
...........................................................cggugucccauuuauuuguaaga..............................   1    0   te1
............................................................ggugucccauuuauuugua.................................   1    0   te1
............................................................ggugucccauuuauuuguaaga..............................   1    0   te1

..................uguauaaauaaaugggacagu.........................................................................   2    0   T53
...................guauaaauaaaugggacaguA........................................................................   1    1   T53
...................guauaaauaaaugggacagugU.......................................................................   1    1   T53
...................guauaaauaaaugggacagugc.......................................................................   3    0   T53
...................guauaaauaaaugggacagugcu......................................................................   12   0   T53
...................guauaaauaaaugggacagugcuU.....................................................................   1    1   T53
...................guauaaauaaaugggacagugcuAu....................................................................   1    1   T53
...................guauaaauaaaugggacagugcugu....................................................................   1    0   T53
....................uauaaauaaaugggacagug........................................................................   2    0   T53
....................uauaaauaaaugggacagugc.......................................................................   10   0   T53
....................uauaaauaaGugggacagugc.......................................................................   1    1   T53
....................uauaaauaaaugggacagugcu......................................................................   125  0   T53
....................uauaaauGaaugggacagugcu......................................................................   8    1   T53
....................uauaaauaGaugggacagugcu......................................................................   1    1   T53
....................uauaaauaaaugggacagugcuU.....................................................................   3    1   T53
....................uauaaauaaaugggacagugcuUu....................................................................   2    1   T53

..................uguauaaauaaaugggacagu.........................................................................   2    0   MOL
..................uguauaaauaaaugggacagugc.......................................................................   2    0   MOL
..................uguauaaauaaaugggacagugcug.....................................................................   1    0   MOL
...................guauaaauaaGugggacagug........................................................................   1    1   MOL
...................guauaaauaaaugggacagugc.......................................................................   6    0   MOL
...................guauaaauaaaugggacagugcu......................................................................   7    0   MOL
...................guauaaauaaaugggacagugcuA.....................................................................   2    1   MOL
....................uauaaauaaaugggacagugc.......................................................................   5    0   MOL
....................uauaaauaaGugggacagugcu......................................................................   2    1   MOL
....................uauaaauaaaugggGcagugcu......................................................................   1    1   MOL
....................uauaaauaaaugggacagugcu......................................................................   81   0   MOL
....................uauaaauaaaugggacagugcuA.....................................................................   2    1   MOL
....................uauaaauaaaugggacagugcuU.....................................................................   4    1   MOL
....................uauaaauaaaugggacagugcuAu....................................................................   1    1   MOL
....................uauaaauaaaugggacagugcuUu....................................................................   1    1   MOL
....................uauaaauaaaugggacagugcugugcu.................................................................   1    0   MOL
...........................................................cggugucccauuuauuuguaag...............................   1    0   MOL
...........................................................cggugucccauuuauuuguaaga..............................   1    0   MOL

..................uguauaaauaaGugggacagu.........................................................................   1    1   T63
```

Mature Star

```
auucaguucuuuuauaauuguauaaauaaaugggacagugcugugcuggacauugugugcggugucccauuuauuuguaagauuauaauuugagacacacaucgaaauacuu
..................uguauaaauaaGugggacagugc.......................................................................	1	1	T63
...................guauaaauaaaugggacagu.........................................................................	1	0	T63
...................guauaaauaaaugggacagugcu......................................................................	12	0	T63
....................uauaaauaaaugggacagu.........................................................................	1	0	T63
....................uauaaauaaaugggacagugc.......................................................................	8	0	T63
....................Aauaaauaaaugggacagugcu......................................................................	4	1	T63
....................uauaaauaaaugggacagugcG......................................................................	1	1	T63
....................uauaaauaaaugggacaguAcu......................................................................	1	1	T63
....................uauaaauGaaugggacagugcu......................................................................	3	1	T63
....................uauaaauaaaugggacagugcu......................................................................	141	0	T63
....................uauaaauaaaugggacagugcuU.....................................................................	6	1	T63
```

Provisional ID	:	Scaffold_4228_7041
Score total	:	294237.3
Score for star read(s)	:	3.9
Score for read counts	:	294227.3
Score for mfe	:	1.5
Score for randfold	:	1.6
Score for cons. seed	:	3
Total read count	:	577126
Mature read count	:	573640
Loop read count	:	0
Star read count	:	3486

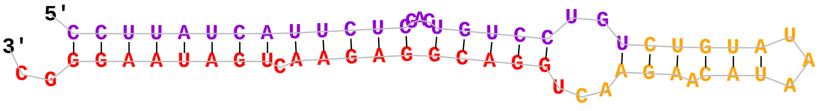

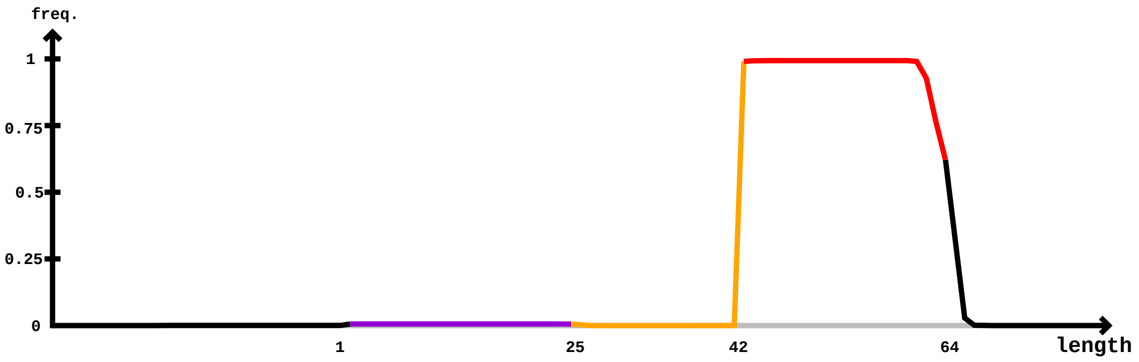

Star Mature

```
5'- gugaagaagaaggaaugguuuuuacauguccuuaucauucucgacuguccugucuguauaaauacaagaacuggacggagaacugauaagggccugugaaaauucaaua  -3'   obs
    gugaagaagaaggaaugguuuuuacauguccuuaucauucucgacuguccugucuguauaaauacaagaacuggacggagaacugauaagggccugugaaaauucaaua         exp
    ...............((((((((((.((((((((((((((...(((((..((((((...))).)))...)))))))))).)))))))))).)))))))).)))...   reads   mm   sample
    ........gaaggaaugguuuuuacaugu................................................................................   3       0    T63
    .........aaggaaugguuuuuacaugu................................................................................   13      0    T63
    ..........aggaaugguuuuuacaugu................................................................................   23      0    T63
    ...........ggaaugguuuuuacaugu................................................................................   11      0    T63
    ............................uccuuaucauucucgacugucc...........................................................   1       0    T63
    .............................ccuuaucauucucgacugucc...........................................................   3       0    T63
    .............................ccuuaucauucucgacuguccu..........................................................   13      0    T63
    .............................ccuuaucauucucgacuguccuU.........................................................   3       1    T63
    .............................ccuuaucauucucgacuguccug.........................................................   12      0    T63
    .............................ccuuaucauucucgaGuguccugu........................................................   1       1    T63
    .............................ccuuaucauucucgacuguccuUu........................................................   3       1    T63
    .............................Ucuuaucauucucgacuguccugu........................................................   1       1    T63
    .............................ccuuaucauucucgacuguccAgu........................................................   1       1    T63
    .............................ccuuaucauucucgacuguccugC........................................................   1       1    T63
    .............................ccuuaucauCcucgacuguccugu........................................................   1       1    T63
    .............................ccuuaucauucucgacuguccugu........................................................   226     0    T63
    .............................ccuuaucauucucgUcuguccugu........................................................   1       1    T63
    .............................ccuuaGcauucucgacuguccugu........................................................   1       1    T63
    .............................ccuuaucauuUucgacuguccugu........................................................   1       1    T63
    .............................ccuuaucauucucgacuguccuguU.......................................................   49      1    T63
    .............................ccuuaucauucucgacuguccuguA.......................................................   5       1    T63
    .............................ccuuaucauucucgacuguccuguc.......................................................   3       0    T63
    .............................ccuuaucauucucgacuguccuguUu......................................................   6       1    T63
    .............................ccuuaucauucucgacuguccugucu......................................................   2       0    T63
    ..............................cuuaucauucucgacuguccugu........................................................   2       0    T63
    ...............................uuaucauucucgacuguccugu........................................................   4       0    T63
    ...............................Cuaucauucucgacuguccugu........................................................   1       1    T63
    ...............................uuaucauucucgacuguccuguU.......................................................   1       1    T63
    ...................................................................gaGcuggacggagaacugauaagg..................   1       1    T63
    ....................................................................aacuggacggagaacugauaagg..................   1       0    T63
    ....................................................................aacuggacggagaacugauaaggU.................   1       1    T63
    ....................................................................aacuggacggagaacugauaagggcU...............   1       1    T63
    .....................................................................acuggacggagaacugauaaggg.................   1       0    T63
```

Star **Mature**

```
gugaagaagaaggaaugguuuuuacauguccuuaucauucucgacuguccugucuguauaaauacaagaacuggacggagaacugauaagggccugugaaaauucaaua
.....................................................................Uuggacggagaacugauaag....................  1      1   T63
.....................................................................Guggacggagaacugauaagg...................  1      1   T63
.....................................................................Auggacggagaacugauaagg...................  2      1   T63
.....................................................................Uuggacggagaacugauaagg...................  2      1   T63
.....................................................................Uuggacggagaacugauaaggg..................  3      1   T63
.....................................................................Auggacggagaacugauaagggc.................  1      1   T63
.....................................................................Uuggacggagaacugauaagggc.................  7      1   T63
.....................................................................cuggacggagaacugauaagggc.................  1      0   T63
......................................................................uggacggagUacugauaa.....................  1      1   T63
......................................................................uggacggagGacugauaa.....................  1      1   T63
......................................................................uggacggagaacugauGa.....................  6      1   T63
......................................................................uggacggagaacugauaa.....................  573    0   T63
......................................................................uggacggagCacugauaa.....................  2      1   T63
......................................................................uggacggagaacugaAaa.....................  3      1   T63
......................................................................uggacggagaacugauaU.....................  6      1   T63
......................................................................uggacggagaacugauUa.....................  4      1   T63
......................................................................uggacggagaacugauaUg....................  1      1   T63
......................................................................uggacggagGacugauaag....................  3      1   T63
......................................................................uggacggagaacugauUag....................  2      1   T63
......................................................................uggacggagaacugauaaU....................  24     1   T63
......................................................................Aggacggagaacugauaag....................  9      1   T63
......................................................................uggacggagaaUugauaag....................  3      1   T63
......................................................................uggacggagaacugauaaA....................  112    1   T63
......................................................................uggacggGgaacugauaag....................  3      1   T63
......................................................................uggacggaAaacugauaag....................  3      1   T63
......................................................................uCgacggagaacugauaag....................  2      1   T63
......................................................................ugUacggagaacugauaag....................  1      1   T63
......................................................................Gggacggagaacugauaag....................  2      1   T63
......................................................................uggacggagaacugUuaag....................  16     1   T63
......................................................................uggacgAagaacugauaag....................  2      1   T63
......................................................................uggUcggagaacugauaag....................  2      1   T63
......................................................................uggacggUgaacugauaag....................  1      1   T63
......................................................................uggacggaUaacugauaag....................  1      1   T63
......................................................................uggaGggagaacugauaag....................  3      1   T63
......................................................................uggacggagaacugaGaag....................  2      1   T63
......................................................................ugCacggagaacugauaag....................  1      1   T63
......................................................................uggGcggagaacugauaag....................  4      1   T63
......................................................................ugAacggagaacugauaag....................  1      1   T63
......................................................................uggacggagaacAgauaag....................  3      1   T63
......................................................................uggacggagaacuUauaag....................  1      1   T63
......................................................................uggacggagCacugauaag....................  7      1   T63
......................................................................uggacggagaaGugauaag....................  4      1   T63
......................................................................uAgacggagaacugauaag....................  1      1   T63
......................................................................uggacggagUacugauaag....................  2      1   T63
......................................................................uggacggagaacugGuaag....................  3      1   T63
......................................................................uggacCgagaacugauaag....................  2      1   T63
......................................................................uggacggagaacugauaaC....................  15     1   T63
......................................................................uggacggagaacugauaGg....................  4      1   T63
......................................................................uggacggagaacCgauaag....................  4      1   T63
......................................................................uggacggagaacugauaag....................  10075  0   T63
......................................................................uggaUggagaacugauaag....................  2      1   T63
......................................................................uggacggagaaAugauaagg...................  1      1   T63
......................................................................uggacggGgaacugauaagg...................  5      1   T63
......................................................................uCgacggagaacugauaagg...................  5      1   T63
......................................................................uggGcggagaacugauaagg...................  6      1   T63
......................................................................ugAacggagaacugauaagg...................  2      1   T63
......................................................................uggaUggagaacugauaagg...................  13     1   T63
......................................................................uggacggUgaacugauaagg...................  1      1   T63
......................................................................uggCcggagaacugauaagg...................  1      1   T63
......................................................................ugCacggagaacugauaagg...................  1      1   T63
......................................................................uggacggaAaacugauaagg...................  13     1   T63
......................................................................uggacggagaacCgauaagg...................  7      1   T63
......................................................................uggacggagaacugauaaUg...................  3      1   T63
......................................................................uggacgAagaacugauaagg...................  5      1   T63
......................................................................uggacggagUacugauaagg...................  1      1   T63
......................................................................uggacggagaacugauaUgg...................  1      1   T63
......................................................................uggacggagaacugauaagC...................  30     1   T63
......................................................................uggaGggagaacugauaagg...................  6      1   T63
......................................................................uggacggagaacugCuaagg...................  2      1   T63
......................................................................uggaAggagaacugauaagg...................  2      1   T63
```

Star Mature

```
gugaagaagaaggaaugguuuuuacauguccuuaucauucucgacuguccugucuguauaaauacaagaacuggacggagaacugauaagggccugugaaaauucaaua
.......................................................................uggacggagaacugauaGgg..................  4      1  T63
.......................................................................uggacggagaacuAauaagg..................  2      1  T63
.......................................................................uggacggagaacAgauaagg..................  3      1  T63
.......................................................................Cggacggagaacugauaagg..................  1      1  T63
.......................................................................uggacAgagaacugauaagg..................  4      1  T63
.......................................................................Gggacggagaacugauaagg..................  8      1  T63
.......................................................................uggacggagaacugaCaagg..................  5      1  T63
.......................................................................uggacggagaUcugauaagg..................  2      1  T63
.......................................................................ugUacggagaacugauaagg..................  3      1  T63
.......................................................................uggacggagCacugauaagg..................  6      1  T63
.......................................................................uggacggagaacuUauaagg..................  3      1  T63
.......................................................................uggacggagaacugauaaCg..................  1      1  T63
.......................................................................uggacggagaacugauaaAg..................  5      1  T63
.......................................................................uggacggagaacugauaagg..................  21473  0  T63
.......................................................................uUgacggagaacugauaagg..................  1      1  T63
.......................................................................uggacggagaacugauGagg..................  1      1  T63
.......................................................................uggacgUagaacugauaagg..................  2      1  T63
.......................................................................uggacUgagaacugauaagg..................  4      1  T63
.......................................................................uggacggagaaUugauaagg..................  1      1  T63
.......................................................................uggacggagaacugGuaagg..................  8      1  T63
.......................................................................uggUcggagaacugauaagg..................  3      1  T63
.......................................................................uAgacggagaacugauaagg..................  2      1  T63
.......................................................................uggacggCgaacugauaagg..................  1      1  T63
.......................................................................uggacggaUaacugauaagg..................  5      1  T63
.......................................................................Aggacggagaacugauaagg..................  17     1  T63
.......................................................................uggacggagaacugaAaagg..................  4      1  T63
.......................................................................uggacCgagaacugauaagg..................  7      1  T63
.......................................................................uggacggagaacugauaagA..................  740    1  T63
.......................................................................uggacggagGacugauaagg..................  5      1  T63
.......................................................................uggacggagaacugUuaagg..................  23     1  T63
.......................................................................uggacggagaacugauaagU..................  71     1  T63
.......................................................................uggacggagaaGugauaagg..................  2      1  T63
.......................................................................uggacggagaacugauaUggg.................  2      1  T63
.......................................................................uggaUggagaacugauaaggg.................  1      1  T63
.......................................................................Aggacggagaacugauaaggg.................  7      1  T63
.......................................................................uggacggagUacugauaaggg.................  2      1  T63
.......................................................................ugUacggagaacugauaaggg.................  2      1  T63
.......................................................................uggacAgagaacugauaaggg.................  2      1  T63
.......................................................................uggacggagaacugauaaggg.................  7468   0  T63
.......................................................................uggacggagaacugCuaaggg.................  1      1  T63
.......................................................................uggacggagaacugauGaggg.................  4      1  T63
.......................................................................uggacggagaacugaAaaggg.................  2      1  T63
.......................................................................uggaGggagaacugauaaggg.................  4      1  T63
.......................................................................uggacggagCacugauaaggg.................  7      1  T63
.......................................................................uggacggagGacugauaaggg.................  4      1  T63
.......................................................................uggacggagaacugauaagUg.................  3      1  T63
.......................................................................uggacgAagaacugauaaggg.................  1      1  T63
.......................................................................uggacggagaacugauaGggg.................  1      1  T63
.......................................................................uggacggUgaacugauaaggg.................  1      1  T63
.......................................................................uggGcggagaacugauaaggg.................  3      1  T63
.......................................................................uggUcggagaacugauaaggg.................  4      1  T63
.......................................................................uCgacggagaacugauaaggg.................  3      1  T63
.......................................................................uggaAggagaacugauaaggg.................  1      1  T63
.......................................................................uggacCgagaacugauaaggg.................  2      1  T63
.......................................................................uggacggagaacugauaaAgg.................  20     1  T63
.......................................................................uggacggagaacugauaaggC.................  62     1  T63
.......................................................................uggacUgagaacugauaaggg.................  2      1  T63
.......................................................................uggacggagaacGgauaaggg.................  1      1  T63
.......................................................................uggacggagaacugauCaggg.................  1      1  T63
.......................................................................uggacggaAaacugauaaggg.................  2      1  T63
.......................................................................uggacggagaacugauaaggU.................  285    1  T63
.......................................................................uggacggaCaacugauaaggg.................  1      1  T63
.......................................................................Cggacggagaacugauaaggg.................  2      1  T63
.......................................................................uggacggagaacugUuaaggg.................  6      1  T63
.......................................................................uggacggagaacugGuaaggg.................  4      1  T63
.......................................................................uggacggagaacuAauaaggg.................  1      1  T63
.......................................................................uggacggagaacugauaaggA.................  2973   1  T63
.......................................................................Gggacggagaacugauaaggg.................  3      1  T63
.......................................................................uggacggaUaacugauaagggc................  1      1  T63
.......................................................................uggacUgagaacugauaagggc................  7      1  T63
```

```
                          Star                                      Mature
gugaagaagaaggaaugguuuuuacauguccuuaucauucucgacuguccuguCuguauaaauacaagaacuggacggagaacugauaagggccugugaaaauucaaua
.......................................................................uggacggagaacugUuaagggc................ 31     1   T63
.......................................................................uggacggagaacugauaagggG................ 34     1   T63
.......................................................................uggacCgagaacugauaagggc................ 3      1   T63
.......................................................................uggacggagaacugauCagggc................ 1      1   T63
.......................................................................uggacggagaacuUauaagggc................ 1      1   T63
.......................................................................uggacggGgaacugauaagggc................ 2      1   T63
.......................................................................uggacggaAaacugauaagggc................ 13     1   T63
.......................................................................uggacggagaacugauaagggU................ 1074   1   T63
.......................................................................uggacggagaacugauaGgggc................ 9      1   T63
.......................................................................uggacggagaacugauUagggc................ 1      1   T63
.......................................................................uggacggagaacugauaUgggc................ 1      1   T63
.......................................................................uggacggagaacuAauaagggc................ 5      1   T63
.......................................................................uggacggagaacugauaaggAc................ 176    1   T63
.......................................................................uggacggagaacugauaagggA................ 6294   1   T63
.......................................................................uggacggagaacugauaagUgc................ 7      1   T63
.......................................................................uggacggagaacCgauaagggc................ 4      1   T63
.......................................................................Gggacggagaacugauaagggc................ 10     1   T63
.......................................................................uggacggagaaGugauaagggc................ 4      1   T63
.......................................................................uggacggagaacAgauaagggc................ 3      1   T63
.......................................................................uggacggagaacugauaagAgc................ 1      1   T63
.......................................................................uggacggagaacugauaagggc................ 26945  0   T63
.......................................................................uggacggagaacugaAaagggc................ 3      1   T63
.......................................................................uggacggagaacugauaaggCc................ 3      1   T63
.......................................................................uggaGggagaacugauaagggc................ 1      1   T63
.......................................................................uggacggagCacugauaagggc................ 13     1   T63
.......................................................................Cggacggagaacugauaagggc................ 3      1   T63
.......................................................................uggacggagaGcugauaagggc................ 2      1   T63
.......................................................................uggacggagaacugauGagggc................ 8      1   T63
.......................................................................uggacAgagaacugauaagggc................ 3      1   T63
.......................................................................uUgacggagaacugauaagggc................ 3      1   T63
.......................................................................uggacggCgaacugauaagggc................ 1      1   T63
.......................................................................uggacggagaCcugauaagggc................ 1      1   T63
.......................................................................uggaAggagaacugauaagggc................ 1      1   T63
.......................................................................Aggacggagaacugauaagggc................ 21     1   T63
.......................................................................uggaUggagaacugauaagggc................ 15     1   T63
.......................................................................uggacggagaacugauaaggUc................ 10     1   T63
.......................................................................ugUacggagaacugauaagggc................ 2      1   T63
.......................................................................uggUcggagaacugauaagggc................ 7      1   T63
.......................................................................uggacggagaacugauaaAggc................ 3      1   T63
.......................................................................uggacggagUacugauaagggc................ 8      1   T63
.......................................................................uggacggagaacugauaaUggc................ 7      1   T63
.......................................................................uggacggagaaUugauaagggc................ 7      1   T63
.......................................................................uggacggagaacugCuaagggc................ 3      1   T63
.......................................................................uggacggagaacugauaagCgc................ 3      1   T63
.......................................................................uggacggagGacugauaagggc................ 6      1   T63
.......................................................................uggGcggagaacugauaagggc................ 6      1   T63
.......................................................................uggacggUgaacugauaagggc................ 7      1   T63
.......................................................................uggacggagaacugaCaagggc................ 1      1   T63
.......................................................................uCgacggagaacugauaagggc................ 8      1   T63
.......................................................................uggacgUagaacugauaagggc................ 2      1   T63
.......................................................................uggacggagaacugGuaagggc................ 21     1   T63
.......................................................................uggacggagaacugauaagggcU............... 4491   1   T63
.......................................................................Aggacggagaacugauaagggcc............... 1      1   T63
.......................................................................uggacggagaacugauaagggAc............... 87     1   T63
.......................................................................uggacggagaacugauaagggcc............... 497    0   T63
.......................................................................uggacggagaacugauaaggAcc............... 1      1   T63
.......................................................................uggaUggagaacugauaagggcc............... 1      1   T63
.......................................................................uggacggagaacugauaagggcG............... 145    1   T63
.......................................................................uggacggagaacugUuaagggcc............... 1      1   T63
.......................................................................uggacggagaacugauaagggUc............... 2      1   T63
.......................................................................uggacggagaacugauaagggcA............... 2486   1   T63
.......................................................................uggacggagaacugauaagggccu.............. 154    0   T63
.......................................................................uggacggagaacugauaagggAcu.............. 1      1   T63
.......................................................................uggacggagaacugauaagggccA.............. 94     1   T63
.......................................................................uggacggagaacugauaagggccC.............. 2      1   T63
.......................................................................uggacggagaacugauaagggcUu.............. 858    1   T63
.......................................................................uggacggagaacugauaagggcAu.............. 322    1   T63
.......................................................................uggacggagaacugauaagggcGu.............. 12     1   T63
.......................................................................uggacggagaacugauaagggcUug............. 1      1   T63
.......................................................................uggacggagaacugauaagggcAug............. 2      1   T63
```

Star **Mature**

```
gugaagaagaaggaaugguuuuuacauguccuuaucauucucgacuguccugucuguauaaauacaagaacuggacggagaacugauaagggccugugaaaauucaaua
.......................................................................uggacggagaacugauaagggccuU.............   39    1   T63
.......................................................................uggacggagaacugauaagggccuUu............   54    1   T63
........................................................................ggacggagaacugauaag...................   8     0   T63
........................................................................ggacggagaacugauaagg..................   18    0   T63
........................................................................ggacggagUacugauaagg..................   1     1   T63
........................................................................ggacggagaacugauaaggg.................   4     0   T63
........................................................................ggacggagaacugauaaggA.................   4     1   T63
........................................................................ggacggagaacugauaagggA................   10    1   T63
........................................................................ggacggagaacugauaagggc................   36    0   T63
........................................................................ggacggagaacugauaagggU................   1     1   T63
........................................................................Ugacggagaacugauaagggc................   2     1   T63
........................................................................ggaGggagaacugauaagggc................   1     1   T63
........................................................................ggacggagaacugauaagggcU...............   5     1   T63
........................................................................ggacggagaacugauaagggcA...............   4     1   T63
........................................................................ggacggagaacugauaagggcUu..............   4     1   T63
.........................................................................gacggagaacugauaagg..................   4     0   T63
.........................................................................gacggagaacugauaaggg.................   2     0   T63
.........................................................................gacggagaacugauaagggc................   7     0   T63
.........................................................................gacggagaacugauaagggU................   1     1   T63
.........................................................................gacggagaacugauaagggcU...............   1     1   T63
..........................................................................acggagaacugauaagggc................   12    0   T63
..........................................................................acggagaacugauaagggA................   1     1   T63
..........................................................................acggagaacugauaagggcU...............   3     1   T63
..........................................................................acggagaacugauaagggcc...............   1     0   T63
..........................................................................acggagaacugauaagggcA...............   3     1   T63
...........................................................................cggagaacugauaagggA................   1     1   T63
...........................................................................cggagaacugauaagggcA...............   2     1   T63
...........................................................................cggagaacugauaagggAc...............   1     1   T63
............................................................................ggagaacugauaagggcU...............   2     1   T63

........gaaggaaugguuuuuacaugu................................................................................   2     0   MOL
.........aaggaaugguuuuuacaugu................................................................................   8     0   MOL
..........aggaaugguuuuuacaugu................................................................................   20    0   MOL
...........ggaaugguuuuuacaugu................................................................................   1     0   MOL
............................uccuuaucauucucgacugucc...........................................................   1     0   MOL
............................uccuuaucauucucgacuguccu..........................................................   1     0   MOL
.............................ccuuaucauucucgacugucc...........................................................   8     0   MOL
.............................ccuuaucauucucgacuguccu..........................................................   13    0   MOL
.............................ccuuaAcauucucgacuguccug.........................................................   1     1   MOL
.............................ccuuaucauucucgacuguccuU.........................................................   7     1   MOL
.............................ccuuaucauucucgacuguccug.........................................................   53    0   MOL
.............................cUuuaucauucucgacuguccug.........................................................   1     1   MOL
.............................ccuuaucauucucgacuguccuUu........................................................   3     1   MOL
.............................ccuuaucauucucgacuguccugu........................................................   419   0   MOL
.............................ccuCaucauucucgacuguccugu........................................................   1     1   MOL
.............................ccuuaucauucucgacuguccugC........................................................   1     1   MOL
.............................ccuuaucauucucgUcuguccugu........................................................   1     1   MOL
.............................Ucuuaucauucucgacuguccugu........................................................   1     1   MOL
.............................ccuuaucauucucgacuguccAgu........................................................   1     1   MOL
.............................ccuuaucaAucucgacuguccugu........................................................   3     1   MOL
.............................ccuuaucauucucgacuguccugA........................................................   2     1   MOL
.............................ccuuaucauucucgacuguccuguA.......................................................   11    1   MOL
.............................ccuuaucauucucgacuguccuguU.......................................................   61    1   MOL
.............................ccuuaucauucucgacuguccuguc.......................................................   4     0   MOL
.............................ccuuaucauucucgacuguccuguUu......................................................   4     1   MOL
.............................ccuuaucauucucgacuguccuguAu......................................................   2     1   MOL
.............................ccuuaucauucucgacuguccugucu......................................................   4     0   MOL
.............................ccuuaucauucucgacuguccugucug.....................................................   10    0   MOL
..............................cuuaucauucucgacuguccugu........................................................   2     0   MOL
...............................uuaucauucucgacuguccug.........................................................   1     0   MOL
...............................uuaucauucucgacuguccugu........................................................   1     0   MOL
...............................uuaucauucucgacuguccugucugu....................................................   1     0   MOL
................................uaucauucucgacuguccugu........................................................   2     0   MOL
................................uaucauucucgacuguccuguU.......................................................   1     1   MOL
................................uaucauucucgacuguccuguUu......................................................   1     1   MOL
.................................aucauucucgacuguccuguU.......................................................   1     1   MOL
.................................aucauucucgacuguccugucuguaua.................................................   1     0   MOL
.....................................Gucucgacuguccugucuguau..................................................   1     1   MOL
.....................................Cucucgacuguccugucuguau..................................................   1     1   MOL
...................................................................gaGcuggacggagaacugauaagg..................   1     1   MOL
```

Star	Mature			
gugaagaagaaggaaugguuuuuacaugu	ccuuaucauucucgacuguccugu cuguauaaauacaagaac uggacggagaacugauaagggc cugugaaaauucaaua			

gugaagaagaaggaaugguuuuuacauguccuuaucauucucgacuguccugucuguauaaauacaagaacuggacggagaacugauaagggccugugaaaauucaaua			
....gaGcuggacggagaacugauaaggg.................	2	1	MOL
....gaGcuggacggagaacugauaagggc................	2	1	MOL
....aacuggacggagaacugauaagggc................	1	0	MOL
....cuggacggagaacugauaag...................	1	0	MOL
....Uuggacggagaacugauaagg..................	1	1	MOL
....Auggacggagaacugauaaggg.................	1	1	MOL
....cuggacggagaacugauaaggg.................	2	0	MOL
....Uuggacggagaacugauaagggc................	3	1	MOL
....cuggacggagaacugauaagggcAu..............	1	1	MOL
....uggacggagaacugauaa....................	234	0	MOL
....uggacggagaacugauUa....................	2	1	MOL
....uAgacggagaacugauaa....................	1	1	MOL
....Gggacggagaacugauaa....................	1	1	MOL
....uggacggagaacugauaG....................	2	1	MOL
....uggacgCagaacugauaa....................	1	1	MOL
....uggacggagaacugUuaag...................	4	1	MOL
....uggacggagaaUugauaag...................	1	1	MOL
....uggacggagUacugauaag...................	1	1	MOL
....Aggacggagaacugauaag...................	6	1	MOL
....Gggacggagaacugauaag...................	4	1	MOL
....uggacggagaacugGuaag...................	3	1	MOL
....uggaUggagaacugauaag...................	1	1	MOL
....ugAacggagaacugauaag...................	2	1	MOL
....uAgacggagaacugauaag...................	2	1	MOL
....uggacgAagaacugauaag...................	1	1	MOL
....uggacggagaacAgauaag...................	1	1	MOL
....uggacUgagaacugauaag...................	1	1	MOL
....uggacggGgaacugauaag...................	1	1	MOL
....uggacggaAaacugauaag...................	1	1	MOL
....uggacggagaacugauaCg...................	1	1	MOL
....uggGcggagaacugauaag...................	2	1	MOL
....uUgacggagaacugauaag...................	1	1	MOL
....uggacggagaacugauUag...................	1	1	MOL
....uggacggagaacugauaag...................	4688	0	MOL
....uggacggagaacugauaGg...................	2	1	MOL
....Cggacggagaacugauaag...................	1	1	MOL
....uggacggagCacugauaag...................	6	1	MOL
....uggacggagaacugauaaU...................	16	1	MOL
....uggaGggagaacugauaag...................	4	1	MOL
....ugUacggagaacugauaag...................	1	1	MOL
....uggacggagaacugauaaA...................	79	1	MOL
....uggacggagaGcugauaag...................	1	1	MOL
....uggacggagaacugauaaC...................	1	1	MOL
....uggacgUagaacugauaagg..................	1	1	MOL
....uggacggagaacuAauaagg..................	1	1	MOL
....uggacggaAaacugauaagg..................	1	1	MOL
....Aggacggagaacugauaagg..................	15	1	MOL
....uggacggagaacugauaagC..................	9	1	MOL
....uggacggagaacugauaagU..................	26	1	MOL
....uggacggagaacugauGagg..................	2	1	MOL
....uCgacggagaacugauaagg..................	2	1	MOL
....uggacggagaaUugauaagg..................	2	1	MOL
....uggacUgagaacugauaagg..................	2	1	MOL
....uggacggagGacugauaagg..................	1	1	MOL
....uggacAgagaacugauaagg..................	1	1	MOL
....uggacggUgaacugauaagg..................	1	1	MOL
....ugAacggagaacugauaagg..................	2	1	MOL
....uggacggagaacugCuaagg..................	1	1	MOL
....uggacggCgaacugauaagg..................	1	1	MOL
....uggaGggagaacugauaagg..................	1	1	MOL
....uggGcggagaacugauaagg..................	1	1	MOL
....uggaUggagaacugauaagg..................	5	1	MOL
....Gggacggagaacugauaagg..................	2	1	MOL
....uggacggagaacugauaagg..................	7916	0	MOL
....Cggacggagaacugauaagg..................	1	1	MOL
....uggacggagaacugUuaagg..................	16	1	MOL
....uggacggagaacugauaaUg..................	1	1	MOL
....uggacggagaacAgauaagg..................	4	1	MOL
....uggacggagaacCgauaagg..................	1	1	MOL
....uggacggagaacugaAaagg..................	1	1	MOL

Star Mature

gugaagaagaaggaaugguuuuuacauguccuuaucauucucgacuguccugucuguauaaauacaagaacuggacggagaacugauaagggccugugaaaauucaaua			
....uggacggagaacugauaagA..................	724	1	MOL
....uggacggagaacugauaaCg..................	1	1	MOL
....uggacggagaacugGuaagg..................	6	1	MOL
....uggacggagaacugaCaagg..................	3	1	MOL
....ugUacggagaacugauaagg..................	1	1	MOL
....uggacggagaacugauaaAg..................	1	1	MOL
....uAgacggagaacugauaagg..................	1	1	MOL
....uggacggagCacugauaagg..................	4	1	MOL
....uggacgAagaacugauaagg..................	2	1	MOL
....uggUcggagaacugauaagg..................	4	1	MOL
....uggacggagaGcugauaagg..................	1	1	MOL
....uggaGggagaacugauaaggg.................	2	1	MOL
....uggacgCagaacugauaaggg.................	2	1	MOL
....uggUcggagaacugauaaggg.................	1	1	MOL
....uggacggagaacugUuaaggg.................	14	1	MOL
....Aggacggagaacugauaaggg.................	8	1	MOL
....uggacggagaacugauaGggg.................	1	1	MOL
....uUgacggagaacugauaaggg.................	1	1	MOL
....uggacggGgaacugauaaggg.................	2	1	MOL
....Gggacggagaacugauaaggg.................	2	1	MOL
....uggacgAagaacugauaaggg.................	1	1	MOL
....uggacggaAaacugauaaggg.................	1	1	MOL
....ugCacggagaacugauaaggg.................	1	1	MOL
....uggacggagaacugaCaaggg.................	1	1	MOL
....uggacggagaacugGuaaggg.................	4	1	MOL
....uggGcggagaacugauaaggg.................	1	1	MOL
....uggacggagaacugauaaggA.................	2673	1	MOL
....uggacggagaacugauaaAgg.................	9	1	MOL
....uggacAgagaacugauaaggg.................	1	1	MOL
....ugAacggagaacugauaaggg.................	1	1	MOL
....uggacggagaacugauaaggC.................	24	1	MOL
....uggacggagaacugauaagAg.................	4	1	MOL
....uggacggagaacugauaaggg.................	5214	0	MOL
....uggacggagCacugauaaggg.................	2	1	MOL
....uggacggagaacugauGaggg.................	3	1	MOL
....uggacggagaacugaAaaggg.................	1	1	MOL
....uggacggagaacugauaaggU.................	84	1	MOL
....uggacggagaUcugauaaggg.................	1	1	MOL
....uggacggagaacugauaaCgg.................	3	1	MOL
....uggacggagaacugauaagCg.................	1	1	MOL
....uggacCgagaacugauaaggg.................	1	1	MOL
....uggacggagaacugCuaaggg.................	1	1	MOL
....ugUacggagaacugauaaggg.................	3	1	MOL
....uggacggaCaacugauaaggg.................	1	1	MOL
....uggaUggagaacugauaaggg.................	3	1	MOL
....uggacggagaacugauaagUg.................	1	1	MOL
....uggacggagaaUugauaagggc................	10	1	MOL
....uggacggagaacugaAaagggc................	1	1	MOL
....uggacggagaacugaCaagggc................	3	1	MOL
....uggUcggagaacugauaagggc................	1	1	MOL
....uggacgAagaacugauaagggc................	1	1	MOL
....uggacggCgaacugauaagggc................	1	1	MOL
....uggCcggagaacugauaagggc................	1	1	MOL
....uggacggagaacugauaUgggc................	2	1	MOL
....uggaUggagaacugauaagggc................	12	1	MOL
....uggacggagaacugauCagggc................	1	1	MOL
....ugUacggagaacugauaagggc................	3	1	MOL
....uCgacggagaacugauaagggc................	5	1	MOL
....uggacggagaacugauaagggU................	611	1	MOL
....uggacggagaacugauaaggCc................	6	1	MOL
....uggacggagaacCgauaagggc................	3	1	MOL
....Aggacggagaacugauaagggc................	28	1	MOL
....uggacggagaacugauGagggc................	4	1	MOL
....uAgacggagaacugauaagggc................	4	1	MOL
....uggacCgagaacugauaagggc................	3	1	MOL
....uggacggagaacugauaGgggc................	5	1	MOL
....uggGcggagaacugauaagggc................	9	1	MOL
....uggacgUagaacugauaagggc................	2	1	MOL
....uggacggagaacugauaaggUc................	7	1	MOL
....uggaGggagaacugauaagggc................	2	1	MOL

```
                             Star                                      Mature
gugaagaagaaggaaugguuuuuacauguccuuaucauucucgacuguccugucuguauaaauacaagaacuggacggagaacugauaagggccugugaaaauucaaua
.......................................................................ugAacggagaacugauaagggc................ 5      1  MOL
.......................................................................uggacggagaacugGuaagggc................ 12     1  MOL
.......................................................................uggacggagaaGugauaagggc................ 3      1  MOL
.......................................................................Gggacggagaacugauaagggc................ 5      1  MOL
.......................................................................uggacggagaacugauaaggAc................ 88     1  MOL
.......................................................................uggacggaUaacugauaagggc................ 2      1  MOL
.......................................................................uggacggagaacugauaagUgc................ 5      1  MOL
.......................................................................uggacggaCaacugauaagggc................ 2      1  MOL
.......................................................................uggacggagaacugauaagCgc................ 2      1  MOL
.......................................................................Cggacggagaacugauaagggc................ 7      1  MOL
.......................................................................uggacggagGacugauaagggc................ 2      1  MOL
.......................................................................uggacUgagaacugauaagggc................ 2      1  MOL
.......................................................................uggacggagaacuUauaagggc................ 4      1  MOL
.......................................................................uggacggagCacugauaagggc................ 10     1  MOL
.......................................................................uggacggagaacugauaagggc................ 14914  0  MOL
.......................................................................uggacggagaacuAauaagggc................ 2      1  MOL
.......................................................................uggacggagaacugauaaUggc................ 2      1  MOL
.......................................................................uggacggagaacugauaagggG................ 26     1  MOL
.......................................................................uggacggagaacugauaagggA................ 6536   1  MOL
.......................................................................uggacggagaacugauaaAggc................ 1      1  MOL
.......................................................................uggacAgagaacugauaagggc................ 10     1  MOL
.......................................................................uggacggagaacugCuaagggc................ 6      1  MOL
.......................................................................uggacggUgaacugauaagggc................ 3      1  MOL
.......................................................................uggacggagaacugUuaagggc................ 26     1  MOL
.......................................................................uggacggaAaacugauaagggc................ 3      1  MOL
.......................................................................uggacggagaacugauaagAgc................ 3      1  MOL
.......................................................................uUgacggagaacugauaagggc................ 1      1  MOL
.......................................................................uggacggagUacugauaagggc................ 2      1  MOL
.......................................................................uggacggGgaacugauaagggc................ 5      1  MOL
.......................................................................uggacggagaUcugauaagggc................ 1      1  MOL
.......................................................................uggacggagaGcugauaagggc................ 1      1  MOL
.......................................................................uggacggagaaUugauaagggcc............... 1      1  MOL
.......................................................................uggacggagaacugauaagggcU............... 4321   1  MOL
.......................................................................uggacggagaacugauaagggcG............... 125    1  MOL
.......................................................................uggacggagaacugauaagggcc............... 575    0  MOL
.......................................................................uggacggagaacugauaagggAc............... 67     1  MOL
.......................................................................uggacggagaacugauaaAggcc............... 1      1  MOL
.......................................................................uggacggagaacugauGagggcc............... 1      1  MOL
.......................................................................uggacggagaacugauaagggcA............... 4295   1  MOL
.......................................................................uggacgAagaacugauaagggcc............... 1      1  MOL
.......................................................................uggacggagaacugauaagggUc............... 4      1  MOL
.......................................................................uggacggagaacugauGagggccu.............. 1      1  MOL
.......................................................................uggacggagaacugauaagggcAu.............. 705    1  MOL
.......................................................................uggacggagaacugauaagggAcu.............. 3      1  MOL
.......................................................................uggacggagGacugauaagggccu.............. 1      1  MOL
.......................................................................uggacggagaacugauaagggUcu.............. 1      1  MOL
.......................................................................uggacggagaacAgauaagggccu.............. 1      1  MOL
.......................................................................uggacggagaacugauaagggcGu.............. 9      1  MOL
.......................................................................uggacggagaacugauaagggccC.............. 1      1  MOL
.......................................................................uggacggGgaacugauaagggccu.............. 2      1  MOL
.......................................................................Aggacggagaacugauaagggccu.............. 2      1  MOL
.......................................................................Cggacggagaacugauaagggccu.............. 1      1  MOL
.......................................................................uggacggagaacugauaagggccu.............. 150    0  MOL
.......................................................................uggacggagaacugauaagggcUu.............. 1226   1  MOL
.......................................................................uggacggagaacugauaagggccA.............. 377    1  MOL
.......................................................................uggacggagaacugauaagggccuU............. 68     1  MOL
.......................................................................uggacggagaacugauaagggccuA............. 1      1  MOL
.......................................................................uggacggagaacugauaagggcUug............. 3      1  MOL
.......................................................................uggacggagaacugauaagggccuUu............ 73     1  MOL
........................................................................ggacggagaacugauaag................... 4      0  MOL
........................................................................ggacggagaacugauaagA.................. 1      1  MOL
........................................................................ggacggagaacugauaagg.................. 10     0  MOL
........................................................................ggacggagaacugauaaggg................. 5      0  MOL
........................................................................Ugacggagaacugauaaggg................. 1      1  MOL
........................................................................ggacggagaacugauaaAgg................. 1      1  MOL
........................................................................ggacggagaacugauaaggA................. 3      1  MOL
........................................................................ggacggagaacugauaagggc................ 20     0  MOL
........................................................................ggacggagaacugauaagggA................ 9      1  MOL
........................................................................ggacggagaacugauaagggcc............... 2      0  MOL
........................................................................ggacggagaacugauaagggcA............... 13     1  MOL
```

Star Mature

```
gugaagaagaaggaaugguuuuuacaugu ccuuaucauucucgacuguccugu cuguauaaauacaagaac uggacggagaacugauaagggc cugugaaaauucaaua
```

```
gugaagaagaaggaaugguuuuuacauguccuuaucauucucgacuguccugucuguauaaauacaagaacuggacggagaacugauaagggccugugaaaauucaaua
........................................................................ggacggagaacugauaagggcU...............  5    1  MOL
........................................................................ggacggagaacugauaagggccu..............  1    0  MOL
........................................................................ggacggagaacugauaagggcUu..............  1    1  MOL
.........................................................................gacggagaacugauaagg..................  1    0  MOL
.........................................................................gacggagaacugauaaggU.................  1    1  MOL
.........................................................................gacggagaacugauaagggc................  9    0  MOL
.........................................................................gacggagaacugauaagggA................  1    1  MOL
.........................................................................gacggagaacugauaagggcA...............  1    1  MOL
..........................................................................acggagaacugauaagggA................  1    1  MOL
..........................................................................acggagaacugauaagggc................  7    0  MOL
..........................................................................acggagaacugauaagggcc...............  1    0  MOL
..........................................................................acggagaacugauaagggcA...............  1    1  MOL
..........................................................................acggagaacugauaagggccu..............  1    0  MOL

........gaaggaaugguuuuuacaugu................................................................................  3    0  T53
.........aaggaaugguuuuuacaugu................................................................................  25   0  T53
..........aggaaugguuuuuacaugu................................................................................  52   0  T53
...........ggaaugguuuuuacaugu................................................................................  8    0  T53
............................Uccuuaucauucucgacuguccug.........................................................  1    1  T53
............................uccuuaucauucucgacugucc...........................................................  1    0  T53
............................uccuuaucauucucgacuguccu..........................................................  1    0  T53
.............................ccuuaucauucucgacugAcc...........................................................  1    1  T53
.............................ccuuaucauucucgacugucc...........................................................  6    0  T53
.............................ccuuaucauucucgacuguccu..........................................................  16   0  T53
.............................ccuuaucauucucgacuguccA..........................................................  1    1  T53
.............................ccuuaucauucucgacuguccuC.........................................................  1    1  T53
.............................ccuuaucauucucgacuguccuU.........................................................  6    1  T53
.............................ccuuaucauucucgacuguccug.........................................................  26   0  T53
.............................ccuuaucauuUucgacuguccugu........................................................  1    1  T53
.............................ccuuaucauucucgacuguccuUu........................................................  4    1  T53
.............................ccuuaucauucucgacuguccugu........................................................  456  0  T53
.............................ccuuaucauucucgaUuguccugu........................................................  1    1  T53
.............................ccuuaucauucucgacAguccugu........................................................  1    1  T53
.............................ccuuaucauucucgacuguccugC........................................................  1    1  T53
.............................ccuuaCcauucucgacuguccugu........................................................  1    1  T53
.............................ccuuaucauucucgacuguccAgu........................................................  2    1  T53
.............................ccuuaucauucucgacuguccuguU.......................................................  82   1  T53
.............................ccuuaucauucucgacuguccuguA.......................................................  10   1  T53
.............................ccuuaucauucucgacuguccuguc.......................................................  5    0  T53
.............................ccuuaucauucucgacuguccuguUu......................................................  4    1  T53
.............................ccuuaucauucucgacuguccuguAu......................................................  2    1  T53
.............................ccuuaucauucucgacuguccugucu......................................................  4    0  T53
.............................ccuuaucauucucgacuguccugucA......................................................  1    1  T53
.............................ccuuaucauucucgacuguccugucug.....................................................  3    0  T53
...............................uuaucauucucgacuguccug.........................................................  2    0  T53
...............................uuaucauucucgacuguccugu........................................................  4    0  T53
...............................uuaucauucucgacuguccuguU.......................................................  1    1  T53
................................uaucauucucgacuguccugucu......................................................  1    0  T53
..................................................................agaGcuggacggagaacugaua.....................  1    1  T53
...................................................................gaGcuggacggagaacugauaagg..................  1    1  T53
...................................................................gaaGuggacggagaacugauaagggc................  1    1  T53
...................................................................gaGcuggacggagaacugauaagggc................  2    1  T53
....................................................................aacuggacggagaacugauaagg..................  1    0  T53
....................................................................Gacuggacggagaacugauaagggc................  1    1  T53
....................................................................aacuggacggagaacugauaagggcU...............  2    1  T53
......................................................................cuggacggagaacugauaag...................  1    0  T53
......................................................................Uuggacggagaacugauaag...................  2    1  T53
......................................................................Uuggacggagaacugauaagg..................  4    1  T53
......................................................................cuggacggagaacugauaagg..................  1    0  T53
......................................................................Uuggacggagaacugauaaggg.................  3    1  T53
......................................................................Uuggacggagaacugauaagggc................  4    1  T53
......................................................................cuggacggagaacugauaagggc................  1    0  T53
......................................................................cuggacggagaacugauaagggcA...............  1    1  T53
.......................................................................uggacggagaacugauUa....................  2    1  T53
.......................................................................uggacggagCacugauaa....................  2    1  T53
.......................................................................uggacggagaacugauGa....................  2    1  T53
.......................................................................uggGcggagaacugauaa....................  1    1  T53
.......................................................................uggacggagaacugauaa....................  594  0  T53
.......................................................................Aggacggagaacugauaa....................  1    1  T53
.......................................................................uggaUggagaacugauaa....................  1    1  T53
```

Star   Mature

```
gugaagaagaaggaaugguuuuuacauguccuuaucauucucgacuguccugucuguauaaauacaagaacuggacggagaacugauaagggccugugaaaauucaaua
.......................................................................uggacggGgaacugauaa....................  1      1  T53
.......................................................................uggacggaUaacugauaa....................  1      1  T53
.......................................................................uggacggagaacugUuaa....................  2      1  T53
.......................................................................uggacggagaacugauaU....................  7      1  T53
.......................................................................uggacCgagaacugauaa....................  1      1  T53
.......................................................................uggacggagaacugaAaa....................  1      1  T53
.......................................................................Aggacggagaacugauaag...................  5      1  T53
.......................................................................uggacggagaaUugauaag...................  1      1  T53
.......................................................................uggacggagaacugCuaag...................  2      1  T53
.......................................................................uggGcggagaacugauaag...................  2      1  T53
.......................................................................uggacggagaacugauaaC...................  9      1  T53
.......................................................................uggacUgagaacugauaag...................  1      1  T53
.......................................................................uggacggagaCcugauaag...................  1      1  T53
.......................................................................uggacggagaacugauaaA...................  120    1  T53
.......................................................................ugUacggagaacugauaag...................  2      1  T53
.......................................................................Cggacggagaacugauaag...................  4      1  T53
.......................................................................uggacggagUacugauaag...................  2      1  T53
.......................................................................uggacggagaaGugauaag...................  1      1  T53
.......................................................................uggacggagaacugauaaU...................  29     1  T53
.......................................................................uggacggGgaacugauaag...................  2      1  T53
.......................................................................uggacggagaacugauaag...................  8286   0  T53
.......................................................................uggUcggagaacugauaag...................  2      1  T53
.......................................................................uggacggagaacuAauaag...................  1      1  T53
.......................................................................uggacggagaacugauaUg...................  1      1  T53
.......................................................................uggacggagaacugauGag...................  2      1  T53
.......................................................................uggacggagaacuUauaag...................  2      1  T53
.......................................................................uCgacggagaacugauaag...................  2      1  T53
.......................................................................uggacggagaacugGuaag...................  3      1  T53
.......................................................................uggacggaAaacugauaag...................  2      1  T53
.......................................................................uggacggagCacugauaag...................  4      1  T53
.......................................................................uggacggagaacugauaGg...................  1      1  T53
.......................................................................uggaUggagaacugauaag...................  3      1  T53
.......................................................................uggacggagaacugaAaag...................  1      1  T53
.......................................................................uggacggagaacCgauaag...................  1      1  T53
.......................................................................uggacggagaacAgauaag...................  2      1  T53
.......................................................................uggacggUgaacugauaag...................  1      1  T53
.......................................................................uggacggagaacugUuaag...................  14     1  T53
.......................................................................uggacggagaUcugauaag...................  1      1  T53
.......................................................................uUgacggagaacugauaag...................  1      1  T53
.......................................................................uggacCgagaacugauaag...................  1      1  T53
.......................................................................ugCacggagaacugauaag...................  1      1  T53
.......................................................................uggacggaUaacugauaag...................  1      1  T53
.......................................................................uAgacggagaacugauaag...................  1      1  T53
.......................................................................uggacggagaacugaAaagg..................  2      1  T53
.......................................................................uggacgAagaacugauaagg..................  2      1  T53
.......................................................................uggacggagaaUugauaagg..................  6      1  T53
.......................................................................uggacgCagaacugauaagg..................  1      1  T53
.......................................................................uggacggagaacugauaagU..................  87     1  T53
.......................................................................uggacggagaacugauaGgg..................  2      1  T53
.......................................................................uggacgUagaacugauaagg..................  2      1  T53
.......................................................................uggacggagaacAgauaagg..................  1      1  T53
.......................................................................uggacggagaacugauaagC..................  18     1  T53
.......................................................................uggaGggagaacugauaagg..................  4      1  T53
.......................................................................uAgacggagaacugauaagg..................  2      1  T53
.......................................................................uggacAgagaacugauaagg..................  2      1  T53
.......................................................................uggacggagaacuUauaagg..................  2      1  T53
.......................................................................uggacggagaacugUuaagg..................  14     1  T53
.......................................................................uggacggagaacugaCaagg..................  2      1  T53
.......................................................................Gggacggagaacugauaagg..................  4      1  T53
.......................................................................uggacggagaacuAauaagg..................  1      1  T53
.......................................................................uggacggaAaacugauaagg..................  6      1  T53
.......................................................................uggaAggagaacugauaagg..................  2      1  T53
.......................................................................Cggacggagaacugauaagg..................  3      1  T53
.......................................................................uggacggagaacugauaCgg..................  1      1  T53
.......................................................................uggacggagaacugauaagg..................  15200  0  T53
.......................................................................uggacCgagaacugauaagg..................  2      1  T53
.......................................................................uggacggaUaacugauaagg..................  1      1  T53
.......................................................................uggGcggagaacugauaagg..................  4      1  T53
.......................................................................uggacggagaacugauaaAg..................  3      1  T53
.......................................................................uggacggGgaacugauaagg..................  1      1  T53
```

Star Mature

```
gugaagaagaaggaaugguuuuuacauguccuuaucauucucgacuguccugucuguauaaauacaagaacuggacggagaacugauaagggccugugaaaauucaaua
.......................................................................uggacggUgaacugauaagg..................  2     1  T53
.......................................................................uggacggagaacugauUagg..................  2     1  T53
.......................................................................uggacggagGacugauaagg..................  3     1  T53
.......................................................................Aggacggagaacugauaagg..................  22    1  T53
.......................................................................uggacggagCacugauaagg..................  5     1  T53
.......................................................................uggacUgagaacugauaagg..................  6     1  T53
.......................................................................uggacggagaacugGuaagg..................  13    1  T53
.......................................................................uggacggagaacugauGagg..................  4     1  T53
.......................................................................uggacggagUacugauaagg..................  4     1  T53
.......................................................................uggacggagaacugauaaCg..................  1     1  T53
.......................................................................ugUacggagaacugauaagg..................  3     1  T53
.......................................................................uggacggCgaacugauaagg..................  2     1  T53
.......................................................................uCgacggagaacugauaagg..................  3     1  T53
.......................................................................uggacggagaacugauaaUg..................  1     1  T53
.......................................................................uggaUggagaacugauaagg..................  2     1  T53
.......................................................................uggacggagaacugauaagA..................  767   1  T53
.......................................................................uggacggagaacugauaUgg..................  1     1  T53
.......................................................................uggacUgagaacugauaaggg.................  2     1  T53
.......................................................................uggacggagaacugauaUggg.................  1     1  T53
.......................................................................uggacggagaacugauaaggA.................  2525  1  T53
.......................................................................uggaUggagaacugauaaggg.................  4     1  T53
.......................................................................uggacggagaacugauaaAgg.................  2     1  T53
.......................................................................uggacggagaacugUuaaggg.................  11    1  T53
.......................................................................uggacggagaacugauaagAg.................  5     1  T53
.......................................................................uggacggagaacugauaaggU.................  180   1  T53
.......................................................................uggacggagaacugauGaggg.................  2     1  T53
.......................................................................uggacggagGacugauaaggg.................  1     1  T53
.......................................................................uggaGggagaacugauaaggg.................  1     1  T53
.......................................................................uggacgCagaacugauaaggg.................  1     1  T53
.......................................................................uggacgUagaacugauaaggg.................  3     1  T53
.......................................................................ugCacggagaacugauaaggg.................  1     1  T53
.......................................................................uggacggagaacugaCaaggg.................  1     1  T53
.......................................................................uggacggagaacugauaGggg.................  4     1  T53
.......................................................................uggacggagCacugauaaggg.................  2     1  T53
.......................................................................uggacAgagaacugauaaggg.................  4     1  T53
.......................................................................uggacggUgaacugauaaggg.................  1     1  T53
.......................................................................uggacggagaacuUauaaggg.................  1     1  T53
.......................................................................uggacggaUaacugauaaggg.................  2     1  T53
.......................................................................uggacCgagaacugauaaggg.................  1     1  T53
.......................................................................uggacggaAaacugauaaggg.................  1     1  T53
.......................................................................uggacgAagaacugauaaggg.................  4     1  T53
.......................................................................uggacggagaacugauaaggg.................  6425  0  T53
.......................................................................uggacggagaacugauUaggg.................  1     1  T53
.......................................................................uggUcggagaacugauaaggg.................  1     1  T53
.......................................................................uUgacggagaacugauaaggg.................  1     1  T53
.......................................................................uCgacggagaacugauaaggg.................  1     1  T53
.......................................................................uggacggagaaUugauaaggg.................  1     1  T53
.......................................................................uggacggagaacugaAaaggg.................  1     1  T53
.......................................................................ugUacggagaacugauaaggg.................  1     1  T53
.......................................................................uggacggagUacugauaaggg.................  1     1  T53
.......................................................................uggacggagaacugCuaaggg.................  2     1  T53
.......................................................................uggacggagaacugauaaggC.................  30    1  T53
.......................................................................uggacggagaacugauaagUg.................  1     1  T53
.......................................................................Aggacggagaacugauaaggg.................  6     1  T53
.......................................................................ugAacggagaacugauaaggg.................  1     1  T53
.......................................................................uggacggagaacugGuaaggg.................  9     1  T53
.......................................................................Cggacggagaacugauaaggg.................  3     1  T53
.......................................................................uggacggagaacugauaagCgc................  4     1  T53
.......................................................................uggacggagaacugCuaagggc................  3     1  T53
.......................................................................uggacggagaacugauaagAgc................  2     1  T53
.......................................................................uggacggGgaacugauaagggc................  3     1  T53
.......................................................................uggacggagaacugauaGgggc................  7     1  T53
.......................................................................uggacAgagaacugauaagggc................  4     1  T53
.......................................................................uggacggagaacugaCaagggc................  3     1  T53
.......................................................................uggacggagaacugaAaagggc................  1     1  T53
.......................................................................uggacggagCacugauaagggc................  11    1  T53
.......................................................................ugAacggagaacugauaagggc................  2     1  T53
.......................................................................uggacggagaaGugauaagggc................  4     1  T53
.......................................................................uggacggagaacCgauaagggc................  2     1  T53
.......................................................................uggacggagaacugauaagggA................  5374  1  T53
```

Star **Mature**

```
gugaagaagaaggaaugguuuuuacaugucc uuaucauucucgacuguccugucuguauaaauacaagaacuggacggagaacugauaagggccugugaaaauucaaua
```

```
gugaagaagaaggaaugguuuuuacauguccuuaucauucucgacuguccugucuguauaaauacaagaacuggacggagaacugauaagggccugugaaaauucaaua
.......................................................................Aggacggagaacugauaagggc................ 21 1 T53
.......................................................................uggacggagaCcugauaagggc................ 1 1 T53
.......................................................................uggacggUgaacugauaagggc................ 5 1 T53
.......................................................................uggacggagaacugauGagggc................ 18 1 T53
.......................................................................uggacggagaaUugauaagggc................ 9 1 T53
.......................................................................uggacggagGacugauaagggc................ 4 1 T53
.......................................................................uCgacggagaacugauaagggc................ 5 1 T53
.......................................................................uggacggaAaacugauaagggc................ 10 1 T53
.......................................................................uggacggagaGcugauaagggc................ 1 1 T53
.......................................................................uUgacggagaacugauaagggc................ 2 1 T53
.......................................................................uggacgAagaacugauaagggc................ 1 1 T53
.......................................................................uggCcggagaacugauaagggc................ 2 1 T53
.......................................................................Gggacggagaacugauaagggc................ 12 1 T53
.......................................................................uggaGggagaacugauaagggc................ 1 1 T53
.......................................................................uggacggagaacugauaaUggc................ 6 1 T53
.......................................................................uggacggagaacugauaaggCc................ 4 1 T53
.......................................................................uggacgUagaacugauaagggc................ 1 1 T53
.......................................................................uggaUggagaacugauaagggc................ 8 1 T53
.......................................................................uggacggagaacugauaaAggc................ 2 1 T53
.......................................................................uggacggagaacugauaaggAc................ 115 1 T53
.......................................................................uggacggagaacugUuaagggc................ 27 1 T53
.......................................................................uggacggagaacAgauaagggc................ 5 1 T53
.......................................................................ugUacggagaacugauaagggc................ 4 1 T53
.......................................................................uggacggagaacugauaagggU................ 853 1 T53
.......................................................................uggacggagaUcugauaagggc................ 1 1 T53
.......................................................................uggacggagUacugauaagggc................ 1 1 T53
.......................................................................uggacggagaacugauaaggUc................ 11 1 T53
.......................................................................uggacggagaacugauaUgggc................ 2 1 T53
.......................................................................uggacggagaacugauUagggc................ 1 1 T53
.......................................................................Cggacggagaacugauaagggc................ 3 1 T53
.......................................................................uggacggagaacugauaagggG................ 24 1 T53
.......................................................................uggacggagaacugauaagUgc................ 8 1 T53
.......................................................................uggacUgagaacugauaagggc................ 2 1 T53
.......................................................................uggacCgagaacugauaagggc................ 3 1 T53
.......................................................................uggacggagaacuUauaagggc................ 1 1 T53
.......................................................................uggacggaUaacugauaagggc................ 3 1 T53
.......................................................................uggacggagaacugGuaagggc................ 26 1 T53
.......................................................................uggacggagaacugauaagggc................ 20818 0 T53
.......................................................................uggUcggagaacugauaagggc................ 5 1 T53
.......................................................................uggGcggagaacugauaagggc................ 3 1 T53
.......................................................................uggacggagaacugUuaagggcc............... 5 1 T53
.......................................................................uggacggagaacugauaagggcG............... 135 1 T53
.......................................................................uggacggagaacugauaagggcc............... 786 0 T53
.......................................................................uggacggagaacugauaagggUc............... 4 1 T53
.......................................................................uggacggaAaacugauaagggcc............... 1 1 T53
.......................................................................uggUcggagaacugauaagggcc............... 1 1 T53
.......................................................................uggacggagaacugauaaggAcc............... 3 1 T53
.......................................................................uCgacggagaacugauaagggcc............... 1 1 T53
.......................................................................uggacggagCacugauaagggcc............... 1 1 T53
.......................................................................uggacgAagaacugauaagggcc............... 1 1 T53
.......................................................................uggaUggagaacugauaagggcc............... 3 1 T53
.......................................................................Aggacggagaacugauaagggcc............... 2 1 T53
.......................................................................uggacggagaacugauaagggcA............... 2569 1 T53
.......................................................................uggacggagaacugauaagggcU............... 4199 1 T53
.......................................................................uggacggagaacugauaagggAc............... 64 1 T53
.......................................................................uggacggagaacugauaagggccC.............. 2 1 T53
.......................................................................uCgacggagaacugauaagggccu.............. 1 1 T53
.......................................................................uggacggagaacugauaagggccu.............. 213 0 T53
.......................................................................uggacggagaacugauaagggccA.............. 216 1 T53
.......................................................................uggacggagaacugUuaagggccu.............. 1 1 T53
.......................................................................uggacggagaacugauaagggcAu.............. 367 1 T53
.......................................................................uggacggagaacugauaagggccG.............. 1 1 T53
.......................................................................uggacggagaacugauaagggcUu.............. 899 1 T53
.......................................................................uggacggagaacugauaagggAcu.............. 9 1 T53
.......................................................................uggacggagaacugauaagggcGu.............. 9 1 T53
.......................................................................uggacggagaacugauaagggcAug............. 1 1 T53
.......................................................................uggacggagaacugauaagggccuU............. 37 1 T53
.......................................................................uggacggagaacugauaagggccuA............. 1 1 T53
.......................................................................uggacggagaacugauaagggccuC............. 1 1 T53
.......................................................................uggacggagaacugauaagggccuUu............ 61 1 T53
```

```
                              Star                                     Mature
gugaagaagaaggaaugguuuuuacauguccuuaucauucucgacuguccugucuguauaaauacaagaacuggacggagaacugauaagggccugugaaaauucaaua
........................................................................ggacggagaacugauaag...................   6     0     T53
........................................................................ggacggagaCcugauaagg..................   1     1     T53
........................................................................ggacggagaacugauaagg..................   25    0     T53
........................................................................Ugacggagaacugauaagg..................   1     1     T53
........................................................................ggacggagaacugauaaggg.................   6     0     T53
........................................................................ggacggagaacugauaaggA.................   3     1     T53
........................................................................ggacggagaacugauaagggc................   25    0     T53
........................................................................ggacggagaacugauaagggA................   11    1     T53
........................................................................ggacggagaacugauaagggcA...............   3     1     T53
........................................................................ggacggagaacugauaagggAc...............   1     1     T53
........................................................................ggacggagaacugauaagggcU...............   4     1     T53
........................................................................ggacggagaacugauaagggcc...............   2     0     T53
........................................................................ggacggagaacugauaagggcAu..............   2     1     T53
........................................................................ggacggagaacugauaagggccu..............   1     0     T53
........................................................................ggacggagaacugauaagggccA..............   1     1     T53
........................................................................ggacggagaacugauaagggcUu..............   1     1     T53
.........................................................................gacggagaacugauaagg..................   4     0     T53
.........................................................................gacggagaacugauaagggc................   3     0     T53
.........................................................................gacggagaacugauaagggA................   1     1     T53
.........................................................................gacggagaacugauaagggcU...............   1     1     T53
.........................................................................gacggagaacugauaagggcG...............   1     1     T53
..........................................................................acggagaacugauaaggg.................   1     0     T53
..........................................................................acggagaacugauaagggc................   10    0     T53
..........................................................................acgCagaacugauaagggc................   1     1     T53
...........................................................................cggagaacugauaagggc................   2     0     T53
...........................................................................cggagaacugauaagggA................   1     1     T53
...........................................................................cggagaacugauaagggcA...............   1     1     T53

..........aggaaugguuuuuacaugu................................................................................   13    0     te1
...........ggaaugguuuuuacaugu................................................................................   1     0     te1
.............................ccuuaucauucucgacuguccu..........................................................   3     0     te1
.............................ccuuaucauucucgacuguccug.........................................................   7     0     te1
.............................ccuuaucauucucgacuguccuU.........................................................   1     1     te1
.............................ccuuaucauucucgacCguccugu........................................................   1     1     te1
.............................ccuuaucauucucgacuguccugu........................................................   43    0     te1
.............................ccuuaucauucucgGcuguccugu........................................................   1     1     te1
.............................ccuuaucauucucgacuguccuguU.......................................................   6     1     te1
.............................ccuuaucauucucgacuguccugucug.....................................................   1     0     te1
..............................cuuaucauucucgacuguccu..........................................................   1     0     te1
..............................cuuaucauucucgacuguccug.........................................................   2     0     te1
...............................uuaucauucucgacuguccugu........................................................   2     0     te1
...............................uuaucauucucgacuguccuguU.......................................................   1     1     te1
................................uaucauucucgacuguccugu........................................................   1     0     te1
.................................aucauucucgacuguccugu........................................................   2     0     te1
......................................................................Uuggacggagaacugauaagg..................   1     1     te1
......................................................................cuggacggagaacugauaagg..................   1     0     te1
.......................................................................uggacggagaacugauaa....................   21    0     te1
.......................................................................Gggacggagaacugauaag...................   5     1     te1
.......................................................................uUgacggagaacugauaag...................   1     1     te1
.......................................................................uggacggagaacugauaaA...................   25    1     te1
.......................................................................uggacggagaacugauaaC...................   3     1     te1
.......................................................................uggacggagaacugGuaag...................   1     1     te1
.......................................................................uggacggagaGcugauaag...................   1     1     te1
.......................................................................uggUcggagaacugauaag...................   1     1     te1
.......................................................................uggacggagaacugauUag...................   1     1     te1
.......................................................................uggaGggagaacugauaag...................   1     1     te1
.......................................................................uggacggagaacugauaUg...................   4     1     te1
.......................................................................uggacggagaacugUuaag...................   1     1     te1
.......................................................................uggacggagaaUugauaag...................   1     1     te1
.......................................................................uggacggagaacugCuaag...................   1     1     te1
.......................................................................Cggacggagaacugauaag...................   4     1     te1
.......................................................................uggGcggagaacugauaag...................   2     1     te1
.......................................................................uggacgAagaacugauaag...................   1     1     te1
.......................................................................uggacggagaacugauaaU...................   7     1     te1
.......................................................................uggacggagaacugauaag...................   1762  0     te1
.......................................................................Aggacggagaacugauaag...................   3     1     te1
.......................................................................uggacggagaacugauaGg...................   3     1     te1
.......................................................................uggacggagaacugauaaUg..................   1     1     te1
.......................................................................uggCcggagaacugauaagg..................   3     1     te1
.......................................................................Cggacggagaacugauaagg..................   11    1     te1
```

Star Mature

gugaagaagaaggaaugguuuuuacauguccuuaucauucucgacuguccugucuguauaaauacaagaacuggacggagaacugauaagggccugugaaaauucaaua			
....uggacggagaGcugauaagg..................	2	1	te1
....Gggacggagaacugauaagg..................	32	1	te1
....ugAacggagaacugauaagg..................	1	1	te1
....ugCacggagaacugauaagg..................	1	1	te1
....uggacggUgaacugauaagg..................	2	1	te1
....uggacggagaaAugauaagg..................	3	1	te1
....uggacggagaUcugauaagg..................	1	1	te1
....uggaGggagaacugauaagg..................	3	1	te1
....uggacggagGacugauaagg..................	4	1	te1
....uggacggagaaGugauaagg..................	1	1	te1
....uggacggagaacugauaagg..................	6985	0	te1
....uggacggagaacCgauaagg..................	2	1	te1
....uggacggagCacugauaagg..................	4	1	te1
....uggacggagaacugauaagC..................	7	1	te1
....uggacggagaacugGuaagg..................	4	1	te1
....uggacggagaacugauGagg..................	1	1	te1
....uggacggagaaUugauaagg..................	7	1	te1
....uggacgUagaacugauaagg..................	1	1	te1
....uggacggagaacugauaagU..................	14	1	te1
....Aggacggagaacugauaagg..................	21	1	te1
....uggacggagaacugauaGgg..................	3	1	te1
....uggacggagaacuUauaagg..................	1	1	te1
....uggacggagaCcugauaagg..................	1	1	te1
....uggacggagaacugauaUgg..................	5	1	te1
....uggacggagaacugaCaagg..................	18	1	te1
....uggacUgagaacugauaagg..................	2	1	te1
....uggGcggagaacugauaagg..................	4	1	te1
....uggacggGgaacugauaagg..................	4	1	te1
....ugUacggagaacugauaagg..................	1	1	te1
....uUgacggagaacugauaagg..................	2	1	te1
....uggacggagaacugaGaagg..................	1	1	te1
....uggacggagaacugauaagA..................	305	1	te1
....uggaAggagaacugauaagg..................	1	1	te1
....uCgacggagaacugauaagg..................	2	1	te1
....uggacggagaacugUuaagg..................	1	1	te1
....uggacggagaacugauaaggU.................	48	1	te1
....uggacggUgaacugauaaggg.................	1	1	te1
....uggacggagGacugauaaggg.................	1	1	te1
....Gggacggagaacugauaaggg.................	1	1	te1
....uggacggagaacugauaagUg.................	1	1	te1
....uUgacggagaacugauaaggg.................	1	1	te1
....uggacggagaacugauaaggg.................	1355	0	te1
....Aggacggagaacugauaaggg.................	7	1	te1
....uggacggagaacugauaaggA.................	2668	1	te1
....uggacggagaacugauaaggC.................	10	1	te1
....uggacggagaacugauaUggg.................	1	1	te1
....Cggacggagaacugauaaggg.................	3	1	te1
....uggacggagaacugauaGggg.................	1	1	te1
....uggacggagaacugauaagAg.................	1	1	te1
....uggGcggagaacugauaaggg.................	2	1	te1
....uggacggagaacugauaaAgg.................	5	1	te1
....uggacggagaacugauGaggg.................	1	1	te1
....uggacggagCacugauaaggg.................	1	1	te1
....uggacggagGacugauaagggc................	1	1	te1
....ugAacggagaacugauaagggc................	1	1	te1
....uggacggagaacCgauaagggc................	1	1	te1
....uggacggagUacugauaagggc................	4	1	te1
....uggacggagaacugauaagggc................	1335	0	te1
....uggacggagaacugauaagUgc................	1	1	te1
....uggacUgagaacugauaagggc................	1	1	te1
....Cggacggagaacugauaagggc................	4	1	te1
....uggacggagaacugauaagggU................	234	1	te1
....uggacggagaacugauaGgggc................	1	1	te1
....uggacggagaacugauaagggG................	7	1	te1
....Gggacggagaacugauaagggc................	5	1	te1
....Aggacggagaacugauaagggc................	4	1	te1
....uggacggagaacugauaaggUc................	3	1	te1
....uggacggagaacugUuaagggc................	1	1	te1
....uggacggagaacugauaagCgc................	1	1	te1
....uggacggagaacugaCaagggc................	1	1	te1

```
                               Star                                    Mature
gugaagaagaaggaaugguuuuuacaugucuuaucauucucgacuguccugucuguauaaauacaagaacuggacggagaacugauaagggccugugaaaauucaaua
......................................................................uggacggagaacugauaagggA.................  1886  1  te1
......................................................................uggacggagCacugauaagggc.................  1     1  te1
......................................................................uggacggagaacugauaaggAc.................  71    1  te1
......................................................................uggGcggagaacugauaagggc.................  1     1  te1
......................................................................uggacggaCaacugauaagggc.................  1     1  te1
......................................................................uggacggGgaacugauaagggc.................  1     1  te1
......................................................................uggGcggagaacugauaagggcc................  1     1  te1
......................................................................uggacggagaacugauaagggcG................  8     1  te1
......................................................................uggacggagaacugauGagggcc................  1     1  te1
......................................................................uggacggagUacugauaagggcc................  1     1  te1
......................................................................uggacggagaacugauaagggcU................  894   1  te1
......................................................................uggacggagaacugauaagggcc................  116   0  te1
......................................................................uggacggagaacugauaagggAc................  31    1  te1
......................................................................uggacggagaacugauaagggcA................  693   1  te1
......................................................................uggacggagaacugauaagggAcu...............  2     1  te1
......................................................................uggacggagaacugauaagggcAu...............  53    1  te1
......................................................................uggacggagaacugauaagggccu...............  17    0  te1
......................................................................uggacggagaacugauaagggcUu...............  343   1  te1
......................................................................uggacggagaacugauaagggccA...............  53    1  te1
......................................................................uggacggagaacugauaagggcUug..............  1     1  te1
......................................................................uggacggagaacugauaagggccuU..............  19    1  te1
......................................................................uggacggagaacugauaagggccuUu.............  12    1  te1
.......................................................................ggacggagaacugauaagg...................  15    0  te1
.......................................................................ggacggagaacugauaagC...................  1     1  te1
.......................................................................ggacggagaacugauaagA...................  3     1  te1
.......................................................................ggacggagaacugauaaggA..................  3     1  te1
.......................................................................ggacggagaacugauaaggg..................  4     0  te1
.......................................................................ggacggagaacugauaagggA.................  2     1  te1
.......................................................................ggacggagaacugauaagggc.................  4     0  te1
.......................................................................ggacggagaacugauaagggU.................  1     1  te1
.......................................................................ggacggagaacugauaagggcA................  1     1  te1
.......................................................................ggacggagaacugauaagggAc................  1     1  te1
.......................................................................ggacggagaacugauaagggcU................  5     1  te1
.......................................................................ggacggagaacugauaagggcUu...............  2     1  te1
........................................................................gacggagaacugauaagggc.................  1     0  te1
.........................................................................acggagaacugauaaggA..................  1     1  te1

........gCaggaaugguuuuuacaugu................................................................................  1     1  egg
........gaaggaaugguuuuuacaugu................................................................................  1     0  egg
............................Cccuuaucauucucgacuguccugu........................................................  1     1  egg
.............................ccuuaucauucucgacugucc...........................................................  1     0  egg
.............................ccuGaucauucucgacuguccu..........................................................  1     1  egg
.............................ccuuaucauucucgacuguccu..........................................................  16    0  egg
.............................ccuuaucauCcucgacuguccug.........................................................  1     1  egg
.............................ccuuaucauucucgacuguccug.........................................................  61    0  egg
.............................Ncuuaucauucucgacuguccug.........................................................  1     1  egg
.............................ccuuaucauucucgacuguccuU.........................................................  4     1  egg
.............................ccuGaucauucucgacuguccug.........................................................  4     1  egg
.............................ccuuaucauucucgacuguccAg.........................................................  1     1  egg
.............................ccuuUucauucucgacuguccug.........................................................  1     1  egg
.............................ccuuaucauucucgacuguccuA.........................................................  3     1  egg
.............................ccuuaucauucucgacGguccug.........................................................  1     1  egg
.............................ccuuaCcauucucgacuguccugu........................................................  4     1  egg
.............................ccuuaucGuucucgacuguccugu........................................................  2     1  egg
.............................ccuuaAcauucucgacuguccugu........................................................  3     1  egg
.............................ccuuaucauucucgacuguccugC........................................................  58    1  egg
.............................ccuuaucUuucucgacuguccugu........................................................  2     1  egg
.............................cUuuaucauucucgacuguccugu........................................................  1     1  egg
.............................ccuuaucauucucgacuguccugu........................................................  1108  0  egg
.............................ccuuaucauucucgacuguccCgu........................................................  7     1  egg
.............................ccuuaucauCcucgacuguccugu........................................................  1     1  egg
.............................Ucuuaucauucucgacuguccugu........................................................  3     1  egg
.............................ccuuaucauGcucgacuguccugu........................................................  1     1  egg
.............................ccCuaucauucucgacuguccugu........................................................  1     1  egg
.............................ccuuaucauucucgCcuguccugu........................................................  1     1  egg
.............................ccuuaucauucucgacugAccugu........................................................  1     1  egg
.............................ccuuaucauucucgacugCccugu........................................................  3     1  egg
.............................ccAuaucauucucgacuguccugu........................................................  2     1  egg
.............................ccuuaucauucucUacuguccugu........................................................  1     1  egg
.............................cGuuaucauucucgacuguccugu........................................................  1     1  egg
```

```
                             Star                                          Mature
gugaagaagaaggaaugguuuuuacauguccuuaucauucucgacuguccugucuguauaaauacaagaacuggacggagaacugauaagggccugugaaaauucaaua
.............................Ncuuaucauucucgacuguccugu........................................................     3     1     egg
.............................ccuuaucauucucgacuguccGgu........................................................     1     1     egg
.............................ccuuCucauucucgacuguccugu........................................................     1     1     egg
.............................ccuuaucauucucgacCguccugu........................................................     2     1     egg
.............................ccuAaucauucucgacuguccugu........................................................     2     1     egg
.............................ccuuaucauucucgacuguccuAu........................................................     1     1     egg
.............................ccuuaucauucuUgacuguccugu........................................................     2     1     egg
.............................ccuuaucauucucgacAguccugu........................................................     2     1     egg
.............................ccuuaucaCucucgacuguccugu........................................................     2     1     egg
.............................ccuuaucauucGcgacuguccugu........................................................     1     1     egg
.............................ccuuaucauucucgaUuguccugu........................................................     1     1     egg
.............................ccuuUucauucucgacuguccugu........................................................     16    1     egg
.............................ccuGaucauucucgacuguccugu........................................................     24    1     egg
.............................ccuCaucauucucgacuguccugu........................................................     6     1     egg
.............................ccuuaucauucucgacugucUugu........................................................     3     1     egg
.............................ccuuaucauucucgacuguccuUu........................................................     4     1     egg
.............................ccuuGucauucucgacuguccugu........................................................     9     1     egg
.............................ccuuaucauucucgacuguccugA........................................................     18    1     egg
.............................ccuuaucauucucgGcuguccugu........................................................     2     1     egg
.............................ccuuaucauucucgacuguUcugu........................................................     1     1     egg
.............................ccuuaucauucCcgacuguccugu........................................................     4     1     egg
.............................ccuuaucauucucgacuguccAgu........................................................     2     1     egg
.............................ccuuaucauAcucgacuguccugu........................................................     1     1     egg
.............................ccuuaucauucAcgacuguccugu........................................................     2     1     egg
.............................cAuuaucauucucgacuguccugu........................................................     1     1     egg
.............................ccuuaucauucucgacuguccuguc.......................................................     17    0     egg
.............................ccuuaucauucucgacuguccuguU.......................................................     106   1     egg
.............................ccuuaucauucucgacCguccuguc.......................................................     1     1     egg
.............................ccuuaucauucucgacuguccuguA.......................................................     92    1     egg
.............................ccCuaucauucucgacuguccugucu......................................................     1     1     egg
.............................ccuuaucauucucgacuguccuguAu......................................................     2     1     egg
.............................ccuCaucauucucgacuguccugucu......................................................     1     1     egg
.............................ccuuaucauucucgacuguccugucA......................................................     1     1     egg
.............................ccuuaucauucucgGcuguccugucu......................................................     1     1     egg
.............................ccuuaucauucucgacuguccugucC......................................................     1     1     egg
.............................ccuuUucauucucgacuguccugucu......................................................     1     1     egg
.............................ccuuaucauucucgacuguccugucu......................................................     13    0     egg
.............................ccuuaucauucucgacuguccuguUu......................................................     12    1     egg
.............................ccuuaucauucucgacuguccugucG......................................................     1     1     egg
.............................ccuuaucauucucgacuguccugucug.....................................................     5     0     egg
.............................ccuuaucauucucgacuguccugucuA.....................................................     2     1     egg
.............................ccuuUucauucucgacuguccugucug.....................................................     1     1     egg
..............................cuuaucauucucgacuguccugu........................................................     2     0     egg
...............................uCaucauucucgacuguccugu........................................................     1     1     egg
...............................uuaucauucucgacuguccugu........................................................     1     0     egg
...............................uuaucauucucgacuguccuguU.......................................................     2     1     egg
...............................uuaucauucucgacuguccuguA.......................................................     1     1     egg
...............................uuaucauucucgacuguccugucA......................................................     2     1     egg
...............................uuaucauucucgacuguccugucC......................................................     1     1     egg
...............................uuaucauucucgacuguccugucu......................................................     1     0     egg
......................................................................cuggacggagaacugauaagg..................     2     0     egg
......................................................................cuggacggagaacugauaaggg.................     1     0     egg
......................................................................cuggacggagaacugauaaggA.................     1     1     egg
......................................................................cuggacggagaacugaAaagggc................     1     1     egg
......................................................................cuggacggagaacugauaagggU................     3     1     egg
......................................................................cuggacggagaacugauaagggA................     1     1     egg
......................................................................cuggacggagaacugauaagggc................     10    0     egg
......................................................................cuggacggagaacugauaagggcA...............     6     1     egg
......................................................................cuggacggagaacugauaagggccA..............     1     1     egg
.......................................................................uggacggagaacugGuaa....................     1     1     egg
.......................................................................uggGcggagaacugauaa....................     1     1     egg
.......................................................................uggacggagGacugauaa....................     1     1     egg
.......................................................................uggaUggagaacugauaa....................     2     1     egg
.......................................................................uggacggagaGcugauaa....................     1     1     egg
.......................................................................uggacggagaacugauaG....................     6     1     egg
.......................................................................uggaAggagaacugauaa....................     1     1     egg
.......................................................................uggacggagaacugauGa....................     1     1     egg
.......................................................................uggUcggagaacugauaa....................     1     1     egg
.......................................................................Nggacggagaacugauaa....................     2     1     egg
.......................................................................uggacggagaacugauaa....................     132   0     egg
```

```
                              Star                                        Mature
gugaagaagaaggaaugguuuuuacauguccuuaucauucucgacuguccugucuguauaaauacaagaacuggacggagaacugauaagggccugugaaaauucaaua
.......................................................................uggacggagaacugauaaA...................  28     1  egg
.......................................................................uggGcggagaacugauaag...................  2      1  egg
.......................................................................uAgacggagaacugauaag...................  3      1  egg
.......................................................................uggCcggagaacugauaag...................  1      1  egg
.......................................................................uggaGggagaacugauaag...................  3      1  egg
.......................................................................uggacggagaacugauaag...................  1180   0  egg
.......................................................................uggacggagaacAgauaag...................  2      1  egg
.......................................................................uggaUggagaacugauaag...................  13     1  egg
.......................................................................uggacggagaUcugauaag...................  1      1  egg
.......................................................................uggacggaAaacugauaag...................  1      1  egg
.......................................................................uggacggagGacugauaag...................  3      1  egg
.......................................................................uggacggagaacCgauaag...................  6      1  egg
.......................................................................uggacggagaacGgauaag...................  1      1  egg
.......................................................................uggUcggagaacugauaag...................  15     1  egg
.......................................................................uggacggagaacugauaUg...................  2      1  egg
.......................................................................uUgacggagaacugauaag...................  1      1  egg
.......................................................................uggacggagaacuAauaag...................  5      1  egg
.......................................................................ugAacggagaacugauaag...................  2      1  egg
.......................................................................uggacggUgaacugauaag...................  3      1  egg
.......................................................................Cggacggagaacugauaag...................  4      1  egg
.......................................................................uggacggagaacugGuaag...................  3      1  egg
.......................................................................uggacggagUacugauaag...................  3      1  egg
.......................................................................uggacggagaacugauaaU...................  2      1  egg
.......................................................................uggacggagaGcugauaag...................  3      1  egg
.......................................................................uggacggagaacugauaGg...................  27     1  egg
.......................................................................uggacggagaacugauaaC...................  3      1  egg
.......................................................................Nggacggagaacugauaag...................  2      1  egg
.......................................................................uggacggagaacugauGag...................  3      1  egg
.......................................................................Aggacggagaacugauaag...................  3      1  egg
.......................................................................uggacggagaacugUuaag...................  1      1  egg
.......................................................................uggacggGgaacugauaag...................  4      1  egg
.......................................................................uggacggagCacugauaag...................  1      1  egg
.......................................................................Gggacggagaacugauaag...................  1      1  egg
.......................................................................uggacggagaacugauGagg..................  27     1  egg
.......................................................................uggacggaAaacugauaagg..................  10     1  egg
.......................................................................uggacggagaacAgauaagg..................  14     1  egg
.......................................................................uggacggagaCcugauaagg..................  1      1  egg
.......................................................................uggacggagaacugaGaagg..................  2      1  egg
.......................................................................ugAacggagaacugauaagg..................  10     1  egg
.......................................................................Nggacggagaacugauaagg..................  19     1  egg
.......................................................................uggacggagaacugauaagg..................  10230  0  egg
.......................................................................uggaGggagaacugauaagg..................  33     1  egg
.......................................................................uggacggagaaAugauaagg..................  2      1  egg
.......................................................................uggacggagaacuAauaagg..................  7      1  egg
.......................................................................uggacggagUacugauaagg..................  18     1  egg
.......................................................................uggaUggagaacugauaagg..................  87     1  egg
.......................................................................uAgacggagaacugauaagg..................  13     1  egg
.......................................................................uggacggagaacGgauaagg..................  6      1  egg
.......................................................................uggacggagaacugauaaAg..................  7      1  egg
.......................................................................uggacggagaaGugauaagg..................  3      1  egg
.......................................................................uggacUgagaacugauaagg..................  2      1  egg
.......................................................................uggacggagaacugauaUgg..................  19     1  egg
.......................................................................uCgacggagaacugauaagg..................  12     1  egg
.......................................................................Cggacggagaacugauaagg..................  33     1  egg
.......................................................................uggacggagGacugauaagg..................  58     1  egg
.......................................................................uggacggagaaUugauaagg..................  19     1  egg
.......................................................................uggacggagaacugauaaUg..................  2      1  egg
.......................................................................uggacggagaacugauUagg..................  10     1  egg
.......................................................................uggacggagaacuUauaagg..................  3      1  egg
.......................................................................uggacggagaacugauaGgg..................  254    1  egg
.......................................................................uggacggagCacugauaagg..................  2      1  egg
.......................................................................Gggacggagaacugauaagg..................  3      1  egg
.......................................................................Aggacggagaacugauaagg..................  18     1  egg
.......................................................................uUgacggagaacugauaagg..................  7      1  egg
.......................................................................uggacAgagaacugauaagg..................  22     1  egg
.......................................................................uggacggagaacugauaagC..................  31     1  egg
.......................................................................uggacggagaacugUuaagg..................  8      1  egg
.......................................................................uggacggUgaacugauaagg..................  42     1  egg
.......................................................................uggacggagaacCgauaagg..................  37     1  egg
.......................................................................uggUcggagaacugauaagg..................  105    1  egg
```

```
                                      Star                                     Mature
gugaagaagaaggaaugguuuuuacauguccuuaucauucucgacuguccuguCuguauaaauacaagaacuggacggagaacugauaagggccugugaaaauucaaua
.......................................................................ugCacggagaacugauaagg..................  3      1   egg
.......................................................................uggaAggagaacugauaagg..................  1      1   egg
.......................................................................uggacggagaacugauaagU..................  5      1   egg
.......................................................................uggGcggagaacugauaagg..................  56     1   egg
.......................................................................uggacggCgaacugauaagg..................  3      1   egg
.......................................................................uggacggagaacugauaCgg..................  5      1   egg
.......................................................................uggacggagaGcugauaagg..................  40     1   egg
.......................................................................uggacCgagaacugauaagg..................  2      1   egg
.......................................................................uggacggagaUcugauaagg..................  3      1   egg
.......................................................................uggacggagaacugaCaagg..................  12     1   egg
.......................................................................uggacggGgaacugauaagg..................  34     1   egg
.......................................................................uggacggagaacugaAaagg..................  1      1   egg
.......................................................................uggacggagaacugGuaagg..................  46     1   egg
.......................................................................ugUacggagaacugauaagg..................  3      1   egg
.......................................................................uggacgAagaacugauaagg..................  5      1   egg
.......................................................................uggacggaUaacugauaagg..................  3      1   egg
.......................................................................uggacgCagaacugauaagg..................  1      1   egg
.......................................................................uggacggagaacugauaagA..................  415    1   egg
.......................................................................uAgacggagaacugauaaggg.................  27     1   egg
.......................................................................uggacggagaacAgauaaggg.................  15     1   egg
.......................................................................ugUacggagaacugauaaggg.................  2      1   egg
.......................................................................ugAacggagaacugauaaggg.................  9      1   egg
.......................................................................uggacggagaaUugauaaggg.................  17     1   egg
.......................................................................uggacggagaacCgauaaggg.................  54     1   egg
.......................................................................uggacggagaacugauaGggg.................  167    1   egg
.......................................................................uggacggagUacugauaaggg.................  20     1   egg
.......................................................................uggacggUgaacugauaaggg.................  36     1   egg
.......................................................................uggacggagaacugauUaggg.................  8      1   egg
.......................................................................uggacggagaCcugauaaggg.................  2      1   egg
.......................................................................uggacggagaacugauaCggg.................  7      1   egg
.......................................................................uggacggagaUcugauaaggg.................  7      1   egg
.......................................................................uggacgAagaacugauaaggg.................  12     1   egg
.......................................................................uggacggagaacugauaagCg.................  4      1   egg
.......................................................................uggacggagaacugGuaaggg.................  55     1   egg
.......................................................................uggacggagaacugauaUggg.................  17     1   egg
.......................................................................uggacggagaaGugauaaggg.................  5      1   egg
.......................................................................uggacggagaacugauGaggg.................  49     1   egg
.......................................................................uggUcggagaacugauaaggg.................  119    1   egg
.......................................................................uggacggagaacugaCaaggg.................  35     1   egg
.......................................................................uggacggGgaacugauaaggg.................  58     1   egg
.......................................................................uggacAgagaacugauaaggg.................  26     1   egg
.......................................................................uggacggagaacugaGaaggg.................  2      1   egg
.......................................................................uggCcggagaacugauaaggg.................  3      1   egg
.......................................................................uggacggagaacugauaaAgg.................  14     1   egg
.......................................................................uggacggagaacugauaaUgg.................  3      1   egg
.......................................................................uggacggagaacugauaaggA.................  1225   1   egg
.......................................................................uggacggagaacugCuaaggg.................  2      1   egg
.......................................................................uggacggagaacugauaagAg.................  18     1   egg
.......................................................................uggaUggagaacugauaaggg.................  123    1   egg
.......................................................................uggacggagaacugauaaggg.................  13593  0   egg
.......................................................................uggacggagaacugauaagUg.................  5      1   egg
.......................................................................uggacggagaacugUuaaggg.................  10     1   egg
.......................................................................uggacggaCaacugauaaggg.................  1      1   egg
.......................................................................Cggacggagaacugauaaggg.................  45     1   egg
.......................................................................uggacggagaacGgauaaggg.................  9      1   egg
.......................................................................Nggacggagaacugauaaggg.................  30     1   egg
.......................................................................uggacggaAaacugauaaggg.................  10     1   egg
.......................................................................uggacggaUaacugauaaggg.................  2      1   egg
.......................................................................uggacggagCacugauaaggg.................  4      1   egg
.......................................................................uggacggagaacuAauaaggg.................  11     1   egg
.......................................................................uUgacggagaacugauaaggg.................  8      1   egg
.......................................................................uggacgUagaacugauaaggg.................  4      1   egg
.......................................................................uggacggagaacuUauaaggg.................  4      1   egg
.......................................................................uggacggCgaacugauaaggg.................  2      1   egg
.......................................................................uggacggagaGcugauaaggg.................  50     1   egg
.......................................................................uggacggagGacugauaaggg.................  54     1   egg
.......................................................................uggacCgagaacugauaaggg.................  1      1   egg
.......................................................................Aggacggagaacugauaaggg.................  25     1   egg
.......................................................................Gggacggagaacugauaaggg.................  5      1   egg
.......................................................................uggacUgagaacugauaaggg.................  3      1   egg
```

Star **Mature**

gugaagaagaaggaaugguuuuuacaugu**ccuuaucauucucgacuguccugu**cuguauaaauacaagaac**uggacggagaacugauaagggc**cugugaaaauucaaua			
....uggacggagaacugauaaggC.................	65	1	egg
....uggGcggagaacugauaaggg.................	69	1	egg
....uggaGggagaacugauaaggg.................	48	1	egg
....uggacggagaaAugauaaggg.................	3	1	egg
....uggacggagaacugauaaggU.................	58	1	egg
....uCgacggagaacugauaaggg.................	16	1	egg
....uggaAggagaacugauaaggg.................	2	1	egg
....uggacggagaacugauCaggg.................	2	1	egg
....uggacggagaacugaAaaggg.................	4	1	egg
....uggacggagaacugauaagCgc................	2	1	egg
....uUgacggagaacugauaagggc................	15	1	egg
....uggacggagaacugaGaagggc................	5	1	egg
....uAgacggagaacugauaagggc................	51	1	egg
....uggacggagGacugauaagggc................	193	1	egg
....uggacggagaacugCuaagggc................	4	1	egg
....uggacggagaUcugauaagggc................	15	1	egg
....uggacggagaacugauaaAggc................	28	1	egg
....uggacggagaacugauaagUgc................	16	1	egg
....uggacggagaacuUauaagggc................	6	1	egg
....uggacggagaacugGuaagggc................	161	1	egg
....uggacggagaacugauUagggc................	18	1	egg
....uggacggagaacugauaagggc................	35910	0	egg
....ugUacggagaacugauaagggc................	5	1	egg
....uggacgAagaacugauaagggc................	28	1	egg
....Cggacggagaacugauaagggc................	150	1	egg
....uggGcggagaacugauaagggc................	183	1	egg
....uggacggUgaacugauaagggc................	121	1	egg
....uggacggagaaAugauaagggc................	5	1	egg
....uggacggagaacugauaaggAc................	64	1	egg
....uggacggagaacuAauaagggc................	34	1	egg
....uggaAggagaacugauaagggc................	9	1	egg
....uggacggagaacugauCagggc................	2	1	egg
....uggacggaCaacugauaagggc................	1	1	egg
....uCgacggagaacugauaagggc................	37	1	egg
....uggacggagaaUugauaagggc................	40	1	egg
....uggacggagaacugauaGgggc................	162	1	egg
....uggacggagaacugauaaggCc................	8	1	egg
....uggacggagCacugauaagggc................	26	1	egg
....uggacgUagaacugauaagggc................	8	1	egg
....uggUcggagaacugauaagggc................	339	1	egg
....uggaUggagaacugauaagggc................	297	1	egg
....uggacggagaacugauaagggA................	4906	1	egg
....uggacggagaacugaAaagggc................	8	1	egg
....uggacggaUaacugauaagggc................	7	1	egg
....uggacggagaacugauaaggUc................	5	1	egg
....ugAacggagaacugauaagggc................	33	1	egg
....uggacggagaacugauaUgggc................	54	1	egg
....uggacggagaaGugauaagggc................	17	1	egg
....uggacggCgaacugauaagggc................	7	1	egg
....ugCacggagaacugauaagggc................	2	1	egg
....uggacggagaacugaCaagggc................	77	1	egg
....uggacggagaacugauGagggc................	87	1	egg
....Nggacggagaacugauaagggc................	66	1	egg
....uggacAgagaacugauaagggc................	68	1	egg
....uggaGggagaacugauaagggc................	132	1	egg
....uggacggagUacugauaagggc................	60	1	egg
....uggacggagaGcugauaagggc................	138	1	egg
....uggacggagaacAgauaagggc................	29	1	egg
....uggCcggagaacugauaagggc................	5	1	egg
....uggacggagaacugauaagggG................	70	1	egg
....uggacggagaCcugauaagggc................	4	1	egg
....uggacggagaacCgauaagggc................	115	1	egg
....Aggacggagaacugauaagggc................	61	1	egg
....uggacggGgaacugauaagggc................	176	1	egg
....uggacggagaacugauaagggU................	2961	1	egg
....uggacggagaacugUuaagggc................	42	1	egg
....uggacgCagaacugauaagggc................	1	1	egg
....Gggacggagaacugauaagggc................	14	1	egg
....uggacUgagaacugauaagggc................	21	1	egg
....uggacggaAaacugauaagggc................	34	1	egg

```
                               Star                                         Mature
gugaagaagaaggaaugguuuuuacaugu ccuuaucauucucgacuguccugu cuguauaaauacaagaac uggacggagaacugauaagggc cugugaaaauucaaua
.......................................................................uggacggagaacGgauaagggc................  17    1  egg
.......................................................................uggacggagaacugauaagAgc................  32    1  egg
.......................................................................uggacggagaacugauaaUggc................  8     1  egg
.......................................................................uggacggagaacugauaCgggc................  17    1  egg
.......................................................................uggacCgagaacugauaagggc................  12    1  egg
.......................................................................uggacggagaacugauaaCggc................  1     1  egg
.......................................................................uggacggagaacuAauaagggcc...............  5     1  egg
.......................................................................uggacggagaacugauUagggcc...............  2     1  egg
.......................................................................uUgacggagaacugauaagggcc...............  2     1  egg
.......................................................................uggacggagaacugauaagggcA...............  3392  1  egg
.......................................................................uggacggagaacugauCagggcc...............  1     1  egg
.......................................................................uggacggagaacugauaaAggcc...............  3     1  egg
.......................................................................uAgacggagaacugauaagggcc...............  9     1  egg
.......................................................................uggacggagaacugauaCgggcc...............  3     1  egg
.......................................................................uggacggagaacAgauaagggcc...............  3     1  egg
.......................................................................uggacggagaacugGuaagggcc...............  18    1  egg
.......................................................................uggacgUagaacugauaagggcc...............  2     1  egg
.......................................................................uggacggagaaUugauaagggcc...............  4     1  egg
.......................................................................Cggacggagaacugauaagggcc...............  14    1  egg
.......................................................................uggacAgagaacugauaagggcc...............  10    1  egg
.......................................................................uggacggagaacuUauaagggcc...............  1     1  egg
.......................................................................uggacggagaacugauaaggCcc...............  2     1  egg
.......................................................................uggacUgagaacugauaagggcc...............  3     1  egg
.......................................................................uggacggagaacugauaaUggcc...............  1     1  egg
.......................................................................uggacggGgaacugauaagggcc...............  20    1  egg
.......................................................................uggacCgagaacugauaagggcc...............  3     1  egg
.......................................................................uggacggagaacugauaUgggcc...............  5     1  egg
.......................................................................uggacggUgaacugauaagggcc...............  11    1  egg
.......................................................................ugAacggagaacugauaagggcc...............  3     1  egg
.......................................................................uggacggagaacugauaagggAc...............  46    1  egg
.......................................................................uggacggagCacugauaagggcc...............  7     1  egg
.......................................................................uggacggagaacGgauaagggcc...............  4     1  egg
.......................................................................uggacggaAaacugauaagggcc...............  3     1  egg
.......................................................................Gggacggagaacugauaagggcc...............  7     1  egg
.......................................................................uggacggagaacugaCaagggcc...............  11    1  egg
.......................................................................uggacggagaacugauaGgggcc...............  14    1  egg
.......................................................................uCgacggagaacugauaagggcc...............  7     1  egg
.......................................................................uggacgAagaacugauaagggcc...............  6     1  egg
.......................................................................uggacggagaacugauaagggUc...............  19    1  egg
.......................................................................uggacggagaacugauaagAgcc...............  3     1  egg
.......................................................................uggacggaUaacugauaagggcc...............  1     1  egg
.......................................................................uggacggagaacugauaagggcc...............  4428  0  egg
.......................................................................uggaUggagaacugauaagggcc...............  37    1  egg
.......................................................................uggacggagaacugUuaagggcc...............  4     1  egg
.......................................................................uggacggagaaGugauaagggcc...............  1     1  egg
.......................................................................uggacggagaUcugauaagggcc...............  3     1  egg
.......................................................................uggacggagaacugauaagggcG...............  96    1  egg
.......................................................................uggaGggagaacugauaagggcc...............  16    1  egg
.......................................................................uggacggagaGcugauaagggcc...............  15    1  egg
.......................................................................ugCacggagaacugauaagggcc...............  1     1  egg
.......................................................................uggacggagaacugauGagggcc...............  12    1  egg
.......................................................................uggacggagaacugauaagggcU...............  3912  1  egg
.......................................................................uggacggagGacugauaagggcc...............  22    1  egg
.......................................................................uggUcggagaacugauaagggcc...............  43    1  egg
.......................................................................uggacggagaacCgauaagggcc...............  19    1  egg
.......................................................................Nggacggagaacugauaagggcc...............  6     1  egg
.......................................................................uggacggagaacugaGaagggcc...............  1     1  egg
.......................................................................uggGcggagaacugauaagggcc...............  24    1  egg
.......................................................................uggacggagUacugauaagggcc...............  3     1  egg
.......................................................................Aggacggagaacugauaagggcc...............  3     1  egg
.......................................................................uggacggagaacugauaaggAcc...............  9     1  egg
.......................................................................uggacggagaacugauaagggcUu..............  193   1  egg
.......................................................................uCgacggagaacugauaagggccu..............  1     1  egg
.......................................................................uggacggagaacugauaCgggccu..............  1     1  egg
.......................................................................uggacggagaacugauaagggcAu..............  508   1  egg
.......................................................................uggacgAagaacugauaagggccu..............  1     1  egg
.......................................................................uggacggagaacugauaagggUcu..............  4     1  egg
.......................................................................Cggacggagaacugauaagggccu..............  1     1  egg
.......................................................................uggacggagaacCgauaagggccu..............  3     1  egg
.......................................................................uggacggagaacugGuaagggccu..............  1     1  egg
```

```
                             Star                                      Mature
gugaagaagaaggaaugguuuuuacauguccuuaucauucucgacuguccugucuguauaaauacaagaacuggacggagaacugauaagggccugugaaaauucaaua
.......................................................................uggacggagaacugauaagggccC..............   35     1    egg
.......................................................................uggUcggagaacugauaagggccu..............   4      1    egg
.......................................................................uggacggagaacugauaagggccG..............   15     1    egg
.......................................................................uggaUggagaacugauaagggccu..............   1      1    egg
.......................................................................uggacggagaacugUuaagggccu..............   1      1    egg
.......................................................................uggacggagaacugauaUgggccu..............   1      1    egg
.......................................................................uggacggagaacugauaaggAccu..............   1      1    egg
.......................................................................uggacggagaacugauaagggcGu..............   6      1    egg
.......................................................................uggacggagCacugauaagggccu..............   1      1    egg
.......................................................................ugAacggagaacugauaagggccu..............   2      1    egg
.......................................................................uggacggagaacGgauaagggccu..............   1      1    egg
.......................................................................uggacggagGacugauaagggccu..............   4      1    egg
.......................................................................uggGcggagaacugauaagggccu..............   1      1    egg
.......................................................................uggacggGgaacugauaagggccu..............   2      1    egg
.......................................................................uggacggagaacugauaaggUccu..............   1      1    egg
.......................................................................uggacggagaacugauGagggccu..............   1      1    egg
.......................................................................uggacggagaacugaGaagggccu..............   1      1    egg
.......................................................................uggacggagaaUugauaagggccu..............   1      1    egg
.......................................................................uggacggagaacugauaagggccu..............   419    0    egg
.......................................................................uggacggagaacugauaGgggccu..............   1      1    egg
.......................................................................uggacggagaacugauaaAggccu..............   1      1    egg
.......................................................................uggacggagaacugauaagAgccu..............   1      1    egg
.......................................................................uggacggagaGcugauaagggccu..............   2      1    egg
.......................................................................uggacgUagaacugauaagggccu..............   1      1    egg
.......................................................................uAgacggagaacugauaagggccu..............   1      1    egg
.......................................................................uggacggagaacugauaagUgccu..............   1      1    egg
.......................................................................uggacggagaacugaCaagggccu..............   2      1    egg
.......................................................................uggacggagaacugauaagggccA..............   3497   1    egg
.......................................................................uggacggagaacugauaagggAcu..............   10     1    egg
.......................................................................uggacggagaacugauaagggccuU.............   42     1    egg
.......................................................................uggacggagaacugauaagggccuC.............   3      1    egg
.......................................................................uggacggagaacugauaagggccuA.............   5      1    egg
.......................................................................uggacggagaacugauaagggccAg.............   1      1    egg
.......................................................................uggacggagaacugauaagggccuUu............   72     1    egg
.......................................................................uggacggagaacugauaagggccuAu............   3      1    egg
.......................................................................uggacggagaacugauaagggccugA............   3      1    egg
........................................................................Ugacggagaacugauaagg..................   3      1    egg
........................................................................ggaUggagaacugauaagg..................   1      1    egg
........................................................................ggacggagaacugauaagg..................   4      0    egg
........................................................................Ugacggagaacugauaaggg.................   7      1    egg
........................................................................ggacggagaacugauaaggg.................   3      0    egg
........................................................................ggacUgagaacugauaagggc................   2      1    egg
........................................................................Ugacggagaacugauaagggc................   21     1    egg
........................................................................ggacggagaacugauaagggc................   16     0    egg
........................................................................ggacggagaacugauaagggA................   2      1    egg
........................................................................ggacggagaacugauaagggU................   2      1    egg
........................................................................ggacggagaacugauaagggcU...............   2      1    egg
........................................................................ggacggagaacugauaagggcc...............   3      0    egg
........................................................................Ugacggagaacugauaagggcc...............   2      1    egg
........................................................................ggacggagaacugauaagggcUu..............   1      1    egg
........................................................................ggacggagaacugauaagggccC..............   1      1    egg
........................................................................ggacggagaacugauaagggccu..............   1      0    egg
.........................................................................gacggagaacugauaagggcU...............   4      1    egg
.........................................................................gacggagaacugauaagggccA..............   1      1    egg
..........................................................................acggagaacugauaaggg.................   1      0    egg
..........................................................................acggagaacugauaagggA................   1      1    egg
...........................................................................cggagaacugauaagggc................   1      0    egg

.......agaaggaaugguuuuuacaugu................................................................................   3      0    T6P
........gaaggaaugguuuuuacaugu................................................................................   6      0    T6P
.........aaggaaugguuuuuacaugu................................................................................   5      0    T6P
..........aggaaugguuuuuacaugu................................................................................   82     0    T6P
..........aggaaugguuCuuacaugu................................................................................   1      1    T6P
...........ggaaugguuuuuacaugu................................................................................   5      0    T6P
.............................ccuuaucauucucgacugucc...........................................................   4      0    T6P
.............................ccuuaucauucucgacuguccu..........................................................   12     0    T6P
.............................ccuuaucauucucgacuguccuU.........................................................   2      1    T6P
.............................ccuuaucauucucgacuguccug.........................................................   8      0    T6P
.............................ccuuaucauucucgacuguccugu........................................................   72     0    T6P
.............................ccuuaucauucucgUcuguccugu........................................................   1      1    T6P
```

Star Mature

```
gugaagaagaaggaaugguuuuuacauguccuuaucauucucgacuguccugucuguauaaauacaagaacuggacggagaacugauaagggccugugaaaauucaaua
.............................ccuuaucauucucgacuguccuguA.......................................................   1      1     T6P
.............................ccuuaucauucucgacuguccuguU.......................................................   14     1     T6P
.............................ccuuaucauucucgacuguccuguGu......................................................   1      1     T6P
.............................ccuuaucauucucgacuguccugucug.....................................................   4      0     T6P
..............................cuuaucauucucgacuguccugu........................................................   2      0     T6P
...............................uuaucauucucgacuguccug.........................................................   1      0     T6P
...............................uuaucauucucgacuguccugu........................................................   2      0     T6P
................................uaucauucucgacuguccugu........................................................   2      0     T6P
.................................aucauucucgacuguccuguU.......................................................   1      1     T6P
.................................................................aagaGcuggacggagaacugauaagg..................   1      1     T6P
..................................................................agaacuggacggagaacugauaagg..................   1      0     T6P
..................................................................agaGcuggacggagaacugauaaggg.................   1      1     T6P
..................................................................agaGcuggacggagaacugauaagggc................   4      1     T6P
..................................................................agaaGuggacggagaacugauaagggc................   1      1     T6P
...................................................................gaGcuggacggagaacugaua.....................   1      1     T6P
...................................................................gaGcuggacggagaacugauaag...................   1      1     T6P
...................................................................gaGcuggacggagaacugauaagg..................   3      1     T6P
...................................................................gaGcuggacggagaacugauaaggg.................   5      1     T6P
...................................................................gaGcuggacggagaacugauaagggc................   9      1     T6P
....................................................................aacuggacggagaacugauaag...................   1      0     T6P
....................................................................aacuggacggagaacugauaagg..................   2      0     T6P
....................................................................aGcuggacggagaacugauaaggg.................   1      1     T6P
....................................................................aaUuggacggagaacugauaagggc................   1      1     T6P
....................................................................aacuggacggagaacugauaagggc................   1      0     T6P
....................................................................aGcuggacggagaacugauaagggc................   2      1     T6P
.....................................................................Gcuggacggagaacugauaagg..................   1      1     T6P
......................................................................Uuggacggagaacugauaag...................   2      1     T6P
......................................................................cuggacggagaacugauaagg..................   2      0     T6P
......................................................................Uuggacggagaacugauaagg..................   4      1     T6P
......................................................................Uuggacggagaacugauaaggg.................   6      1     T6P
......................................................................cuggUcggagaacugauaaggg.................   1      1     T6P
......................................................................cuggacggagaacugauaaggg.................   3      0     T6P
......................................................................cuggacggagaacugauaagggc................   4      0     T6P
......................................................................Uuggacggagaacugauaagggc................   25     1     T6P
......................................................................Uuggacggagaacugauaagggcc...............   2      1     T6P
.......................................................................uggacggagaacugauaU....................   1      1     T6P
.......................................................................Cggacggagaacugauaa....................   1      1     T6P
.......................................................................uggacggagaacugauaa....................   149    0     T6P
.......................................................................uggacggagaacugauaGg...................   2      1     T6P
.......................................................................uggacggaUaacugauaag...................   5      1     T6P
.......................................................................uggGcggagaacugauaag...................   2      1     T6P
.......................................................................Aggacggagaacugauaag...................   5      1     T6P
.......................................................................uggacgCagaacugauaag...................   5      1     T6P
.......................................................................uggacggagaacuCauaag...................   3      1     T6P
.......................................................................ugUacggagaacugauaag...................   1      1     T6P
.......................................................................uggacggagaaUugauaag...................   4      1     T6P
.......................................................................uggacggagaacugauaaC...................   4      1     T6P
.......................................................................uggacggagaGcugauaag...................   1      1     T6P
.......................................................................ugCacggagaacugauaag...................   4      1     T6P
.......................................................................uggacggagaacugaAaag...................   1      1     T6P
.......................................................................uggacggagaacugaGaag...................   1      1     T6P
.......................................................................uggacggagaUcugauaag...................   2      1     T6P
.......................................................................uggaGggagaacugauaag...................   3      1     T6P
.......................................................................uggacggagaacugGuaag...................   2      1     T6P
.......................................................................uggacgAagaacugauaag...................   3      1     T6P
.......................................................................uggacggagaacugauaUg...................   5      1     T6P
.......................................................................uggacggagaacugauaaU...................   18     1     T6P
.......................................................................uggacggaCaacugauaag...................   3      1     T6P
.......................................................................uggacggagaCcugauaag...................   1      1     T6P
.......................................................................uggacggagUacugauaag...................   2      1     T6P
.......................................................................uggacggagaacuAauaag...................   3      1     T6P
.......................................................................uggUcggagaacugauaag...................   11     1     T6P
.......................................................................uggacggagaacugauaCg...................   2      1     T6P
.......................................................................uggacggagaacuUauaag...................   6      1     T6P
.......................................................................Gggacggagaacugauaag...................   1      1     T6P
.......................................................................uggacgUagaacugauaag...................   4      1     T6P
.......................................................................uUgacggagaacugauaag...................   2      1     T6P
.......................................................................uggacggagGacugauaag...................   3      1     T6P
.......................................................................uCgacggagaacugauaag...................   1      1     T6P
.......................................................................uggacggagaacugauaag...................   9165   0     T6P
```

Star Mature

```
gugaagaagaaggaaugguuuuuacauguccuuaucauucucgacuguccugucuguauaaauacaagaacuggacggagaacugauaagggccugugaaaauucaaua
.......................................................................uggacCgagaacugauaag...................    1      1    T6P
.......................................................................uggacggagaacugauUag...................    1      1    T6P
.......................................................................ugAacggagaacugauaag...................    1      1    T6P
.......................................................................uggacggagCacugauaag...................    2      1    T6P
.......................................................................uggacggGgaacugauaag...................    2      1    T6P
.......................................................................uggacggagaacugaCaag...................    3      1    T6P
.......................................................................uggaUggagaacugauaag...................    7      1    T6P
.......................................................................uggacggagaacugauaaA...................    69     1    T6P
.......................................................................uggacggagaacugauGag...................    1      1    T6P
.......................................................................uggacggagaacugCuaag...................    1      1    T6P
.......................................................................uggUcggagaacugauaagg..................    45     1    T6P
.......................................................................Aggacggagaacugauaagg..................    15     1    T6P
.......................................................................Gggacggagaacugauaagg..................    3      1    T6P
.......................................................................uggacgCagaacugauaagg..................    17     1    T6P
.......................................................................uggacggagGacugauaagg..................    2      1    T6P
.......................................................................uggacAgagaacugauaagg..................    4      1    T6P
.......................................................................uUgacggagaacugauaagg..................    3      1    T6P
.......................................................................uggacggagaaUugauaagg..................    20     1    T6P
.......................................................................uggacggagaacugaAaagg..................    1      1    T6P
.......................................................................uggacggagaacugauaUgg..................    6      1    T6P
.......................................................................uggacCgagaacugauaagg..................    1      1    T6P
.......................................................................uggacggagaacugaGaagg..................    2      1    T6P
.......................................................................uggacggagaacuUauaagg..................    16     1    T6P
.......................................................................ugUacggagaacugauaagg..................    5      1    T6P
.......................................................................uggacggagaacugaCaagg..................    9      1    T6P
.......................................................................uggacggagaacugCuaagg..................    2      1    T6P
.......................................................................uggacggagaacugauaaUg..................    10     1    T6P
.......................................................................uggacggagaaAugauaagg..................    2      1    T6P
.......................................................................uggacggaUaacugauaagg..................    5      1    T6P
.......................................................................uggacggagaacugauaagU..................    40     1    T6P
.......................................................................uggacggagaacugauaagg..................    26376  0    T6P
.......................................................................Cggacggagaacugauaagg..................    3      1    T6P
.......................................................................uggaGggagaacugauaagg..................    7      1    T6P
.......................................................................uggacggagaUcugauaagg..................    2      1    T6P
.......................................................................uggacggagaacugGuaagg..................    8      1    T6P
.......................................................................uggacggaCaacugauaagg..................    4      1    T6P
.......................................................................uggacggagaGcugauaagg..................    1      1    T6P
.......................................................................uggacggagaacuAauaagg..................    3      1    T6P
.......................................................................uggacggagCacugauaagg..................    4      1    T6P
.......................................................................uggacggagUacugauaagg..................    5      1    T6P
.......................................................................uggacggagaacugauaCgg..................    3      1    T6P
.......................................................................uggacggagaacugauaagC..................    20     1    T6P
.......................................................................uggacggagaaGugauaagg..................    1      1    T6P
.......................................................................uCgacggagaacugauaagg..................    8      1    T6P
.......................................................................uggacggagaacugauGagg..................    3      1    T6P
.......................................................................uggacggagaacugauaaCg..................    6      1    T6P
.......................................................................uggaUggagaacugauaagg..................    12     1    T6P
.......................................................................uggacggGgaacugauaagg..................    4      1    T6P
.......................................................................uggacggagaacCgauaagg..................    5      1    T6P
.......................................................................uggacggagaacugauaaAg..................    3      1    T6P
.......................................................................uggacgUagaacugauaagg..................    19     1    T6P
.......................................................................uggacggagaacugUuaagg..................    4      1    T6P
.......................................................................ugAacggagaacugauaagg..................    1      1    T6P
.......................................................................uggacggagaacuCauaagg..................    30     1    T6P
.......................................................................uggacUgagaacugauaagg..................    3      1    T6P
.......................................................................uggacgAagaacugauaagg..................    27     1    T6P
.......................................................................uggGcggagaacugauaagg..................    10     1    T6P
.......................................................................uggacggagaacugauaGgg..................    8      1    T6P
.......................................................................ugCacggagaacugauaagg..................    10     1    T6P
.......................................................................uggCcggagaacugauaagg..................    3      1    T6P
.......................................................................uggacggagaacugauaagA..................    427    1    T6P
.......................................................................uggUcggagaacugauaaggg.................    41     1    T6P
.......................................................................uggacggagaacugauaGggg.................    8      1    T6P
.......................................................................uggacggagCacugauaaggg.................    1      1    T6P
.......................................................................ugUacggagaacugauaaggg.................    2      1    T6P
.......................................................................uggacggagaacugCuaaggg.................    5      1    T6P
.......................................................................uggacggagaacugauaaggA.................    4278   1    T6P
.......................................................................uggacggGgaacugauaaggg.................    3      1    T6P
.......................................................................uggacggagaaUugauaaggg.................    9      1    T6P
.......................................................................uggacgCagaacugauaaggg.................    22     1    T6P
```

Star Mature

gugaagaagaaggaaugguuuuuacaugu**ccuuaucauucucgacuguccugu**cuguauaaauacaagaac**uggacggagaacugauaagggc**cugugaaaauucaaua

....uggaGggagaacugauaaggg.................	10	1	T6P
....uggacggagGacugauaaggg.................	7	1	T6P
....uggCcggagaacugauaaggg.................	2	1	T6P
....uggacggagaacugGuaaggg.................	6	1	T6P
....uggacggagaacugauaaUgg.................	12	1	T6P
....uggacggagaacugaAaaggg.................	3	1	T6P
....uggacAgagaacugauaaggg.................	3	1	T6P
....uggacggagaacCgauaaggg.................	3	1	T6P
....uCgacggagaacugauaaggg.................	6	1	T6P
....ugAacggagaacugauaaggg.................	1	1	T6P
....uggacggagaCcugauaaggg.................	1	1	T6P
....uggacggagaacugauaaCgg.................	12	1	T6P
....uggacggagaacuUauaaggg.................	24	1	T6P
....uggacggagaacugauaagCg.................	3	1	T6P
....Gggacggagaacugauaaggg.................	4	1	T6P
....uggacggagaacuCauaaggg.................	23	1	T6P
....uggacggagaacugaGaaggg.................	1	1	T6P
....uggacgUagaacugauaaggg.................	17	1	T6P
....uUgacggagaacugauaaggg.................	7	1	T6P
....uAgacggagaacugauaaggg.................	1	1	T6P
....uggacggagUacugauaaggg.................	13	1	T6P
....uggacggagaacugaCaaggg.................	6	1	T6P
....uggacggaCaacugauaaggg.................	12	1	T6P
....Cggacggagaacugauaaggg.................	4	1	T6P
....uggacggagaacugauaaggC.................	149	1	T6P
....uggacggaAaacugauaaggg.................	1	1	T6P
....uggacggagaacuAauaaggg.................	6	1	T6P
....uggacggagaacugauaaAgg.................	16	1	T6P
....uggacCgagaacugauaaggg.................	3	1	T6P
....uggacggaUaacugauaaggg.................	10	1	T6P
....uggacggagaacugauUaggg.................	4	1	T6P
....uggaUggagaacugauaaggg.................	16	1	T6P
....uggacggagaacugauaaggU.................	150	1	T6P
....uggacggagaacugauaUggg.................	7	1	T6P
....uggacgAagaacugauaaggg.................	23	1	T6P
....uggacUgagaacugauaaggg.................	4	1	T6P
....uggacggagaacugauaaggg.................	29317	0	T6P
....uggacggagaacugauaagUg.................	7	1	T6P
....uggacggagaGcugauaaggg.................	2	1	T6P
....uggacggagaacugauaagAg.................	4	1	T6P
....uggGcggagaacugauaaggg.................	20	1	T6P
....ugCacggagaacugauaaggg.................	16	1	T6P
....uggacggagaacugauCaggg.................	1	1	T6P
....Aggacggagaacugauaaggg.................	16	1	T6P
....uggacggagaacugauGaggg.................	4	1	T6P
....uggacggUgaacugauaaggg.................	3	1	T6P
....uggacggagaacugauaCggg.................	1	1	T6P
....uggacggagaacugUuaaggg.................	9	1	T6P
....uggacggagaacugGuaagggc................	44	1	T6P
....uggacggaUaacugauaagggc................	34	1	T6P
....uggacggagaacugUuaagggc................	47	1	T6P
....uggacgCagaacugauaagggc................	104	1	T6P
....uggacAgagaacugauaagggc................	9	1	T6P
....uggacggagGacugauaagggc................	21	1	T6P
....uggacggagaacugauaaUggc................	82	1	T6P
....uggaAggagaacugauaagggc................	1	1	T6P
....uggacggagaacugauaagggA................	15961	1	T6P
....ugAacggagaacugauaagggc................	14	1	T6P
....uggacggagaacugauaagAgc................	15	1	T6P
....uUgacggagaacugauaagggc................	23	1	T6P
....uggacggagaacAgauaagggc................	2	1	T6P
....uggacggagaacuCauaagggc................	85	1	T6P
....uggacgUagaacugauaagggc................	72	1	T6P
....uggacggagaacugauaaggAc................	190	1	T6P
....Cggacggagaacugauaagggc................	14	1	T6P
....uggacggCgaacugauaagggc................	4	1	T6P
....uggacggagaacugauaagCgc................	57	1	T6P
....uggacggagaacugauaaCggc................	49	1	T6P
....uggacggagaacugauaCgggc................	6	1	T6P
....uggacggagaacugaAaagggc................	10	1	T6P

```
                              Star                                        Mature
gugaagaagaaggaaugguuuuuacauguccuuaucauucucgacuguccugucuguauaaauacaagaacuggacggagaacugauaagggccugugaaaauucaaua
.......................................................................uggacggagaacugauaagUgc................    56       1    T6P
.......................................................................uggaGggagaacugauaagggc................    28       1    T6P
.......................................................................uCgacggagaacugauaagggc................    44       1    T6P
.......................................................................uggacggaAaacugauaagggc................    7        1    T6P
.......................................................................uggacggagaacugauaagggU................    1556     1    T6P
.......................................................................ugUacggagaacugauaagggc................    29       1    T6P
.......................................................................uggacggagaacugaCaagggc................    16       1    T6P
.......................................................................uggacggagaacugauCagggc................    4        1    T6P
.......................................................................Gggacggagaacugauaagggc................    32       1    T6P
.......................................................................uggacggagCacugauaagggc................    10       1    T6P
.......................................................................uggacggagaacugauaagggc................    118951   0    T6P
.......................................................................uggacggagaacuUauaagggc................    73       1    T6P
.......................................................................uggacggGgaacugauaagggc................    18       1    T6P
.......................................................................uggacggagaacGgauaagggc................    1        1    T6P
.......................................................................uggacggUgaacugauaagggc................    7        1    T6P
.......................................................................uggacggagaaAugauaagggc................    7        1    T6P
.......................................................................uAgacggagaacugauaagggc................    2        1    T6P
.......................................................................uggacCgagaacugauaagggc................    39       1    T6P
.......................................................................uggacggagaacuAauaagggc................    30       1    T6P
.......................................................................uggacUgagaacugauaagggc................    40       1    T6P
.......................................................................uggacggagaacugauaaggCc................    57       1    T6P
.......................................................................uggacggagaacCgauaagggc................    20       1    T6P
.......................................................................uggGcggagaacugauaagggc................    44       1    T6P
.......................................................................uggacggagaacugauGagggc................    28       1    T6P
.......................................................................uggacggagaGcugauaagggc................    11       1    T6P
.......................................................................uggacggagaacugauaaggUc................    55       1    T6P
.......................................................................uggUcggagaacugauaagggc................    209      1    T6P
.......................................................................uggacggagaacugauUagggc................    14       1    T6P
.......................................................................uggacggagaacugauaGgggc................    20       1    T6P
.......................................................................uggaUggagaacugauaagggc................    68       1    T6P
.......................................................................uggacggagaaUugauaagggc................    57       1    T6P
.......................................................................uggacggagUacugauaagggc................    37       1    T6P
.......................................................................uggacggagaUcugauaagggc................    11       1    T6P
.......................................................................uggacggagaacugauaaAggc................    15       1    T6P
.......................................................................Aggacggagaacugauaagggc................    93       1    T6P
.......................................................................uggacgAagaacugauaagggc................    81       1    T6P
.......................................................................uggacggagaacugauaUgggc................    34       1    T6P
.......................................................................uggacggaCaacugauaagggc................    50       1    T6P
.......................................................................uggacggagaCcugauaagggc................    1        1    T6P
.......................................................................uggacggagaacugaGaagggc................    13       1    T6P
.......................................................................ugCacggagaacugauaagggc................    65       1    T6P
.......................................................................uggCcggagaacugauaagggc................    9        1    T6P
.......................................................................uggacggagaacugauaagggG................    127      1    T6P
.......................................................................uggacggagaaGugauaagggc................    4        1    T6P
.......................................................................uggacggagaacugCuaagggc................    24       1    T6P
.......................................................................uggacggagaacugaAaagggcc...............    1        1    T6P
.......................................................................uggacggagaacugauGagggcc...............    2        1    T6P
.......................................................................uggacUgagaacugauaagggcc...............    1        1    T6P
.......................................................................uggacggagaacugauaagggcU...............    7574     1    T6P
.......................................................................uggacgUagaacugauaagggcc...............    2        1    T6P
.......................................................................uggaUggagaacugauaagggcc...............    1        1    T6P
.......................................................................uggacggagaacugauaagggGc...............    1        1    T6P
.......................................................................uggacggaUaacugauaagggcc...............    1        1    T6P
.......................................................................ugCacggagaacugauaagggcc...............    2        1    T6P
.......................................................................uggacggagaacugauaagggAc...............    101      1    T6P
.......................................................................uggGcggagaacugauaagggcc...............    1        1    T6P
.......................................................................uggacggagaacuUauaagggcc...............    2        1    T6P
.......................................................................uggUcggagaacugauaagggcc...............    3        1    T6P
.......................................................................uggacggagaacugUuaagggcc...............    1        1    T6P
.......................................................................Cggacggagaacugauaagggcc...............    1        1    T6P
.......................................................................uUgacggagaacugauaagggcc...............    1        1    T6P
.......................................................................Aggacggagaacugauaagggcc...............    1        1    T6P
.......................................................................uggacggagaacugauaagggUc...............    5        1    T6P
.......................................................................uCgacggagaacugauaagggcc...............    1        1    T6P
.......................................................................uggacggagaacugauaagggcA...............    17241    1    T6P
.......................................................................uggacggagaacugGuaagggcc...............    3        1    T6P
.......................................................................ugAacggagaacugauaagggcc...............    1        1    T6P
.......................................................................uggacggagaacugauaaAggcc...............    1        1    T6P
.......................................................................uggacggagaacuCauaagggcc...............    2        1    T6P
.......................................................................uggacgCagaacugauaagggcc...............    2        1    T6P
```

Star **Mature**

```
gugaagaagaaggaaugguuuuuacauguccuuaucauucucgacuguccugucuguauaaauacaagaacuggacggagaacugauaagggccugugaaaauucaaua
.......................................................................uggacggagaacugauaaggAcc...............	1	1	T6P
.......................................................................uggacggagaacugauaaggCcc...............	1	1	T6P
.......................................................................uggacggagaacugauaaggUcc...............	1	1	T6P
.......................................................................uggCcggagaacugauaagggcc...............	1	1	T6P
.......................................................................uggacggagaacugauaagggcG...............	948	1	T6P
.......................................................................uggacggagaacugauaagggcc...............	1533	0	T6P
.......................................................................uggacggagaacuUauaagggccu..............	1	1	T6P
.......................................................................uggacggagaacugauaaggCccu..............	1	1	T6P
.......................................................................uggGcggagaacugauaagggccu..............	1	1	T6P
.......................................................................uggacggagaacugauaagggccC..............	2	1	T6P
.......................................................................uggacggagaacugauaagggcGu..............	43	1	T6P
.......................................................................uggacggagaacugauaagggccu..............	264	0	T6P
.......................................................................uggacggagaacugauaagggccA..............	321	1	T6P
.......................................................................uggacggagaacugauaagggcUu..............	2234	1	T6P
.......................................................................uggacggagaacugauaagggAcu..............	1	1	T6P
.......................................................................uggacggagaacugGuaagggccu..............	1	1	T6P
.......................................................................uggacggagaacugauaaggAccu..............	1	1	T6P
.......................................................................uggacggagaacugauaagggccG..............	1	1	T6P
.......................................................................uggacggagaacugauaagggcAu..............	1994	1	T6P
.......................................................................uggacggagCacugauaagggccu..............	1	1	T6P
.......................................................................uggacggagaacugauaagggccuA.............	1	1	T6P
.......................................................................uggacggagaacugauaagggcAug.............	2	1	T6P
.......................................................................uggacggagaacugauaagggccuU.............	55	1	T6P
.......................................................................uggacggagaacugauaagggccug.............	1	0	T6P
.......................................................................uggacggagaacugauaagggcUugu............	1	1	T6P
.......................................................................uggacggagaacugauaagggccAgu............	1	1	T6P
.......................................................................uggacggagaacugauaagggccuUu............	60	1	T6P
.......................................................................uggacggagaacugauaagggccuUug...........	1	1	T6P
........................................................................Ugacggagaacugauaag...................	1	1	T6P
........................................................................Cgacggagaacugauaag...................	1	1	T6P
........................................................................ggacggagaacugauaag...................	29	0	T6P
........................................................................ggacggagaacugGuaagg..................	1	1	T6P
........................................................................ggacggagaacugaCaagg..................	1	1	T6P
........................................................................Ugacggagaacugauaagg..................	6	1	T6P
........................................................................ggacggagaacugauaaAg..................	1	1	T6P
........................................................................ggaAggagaacugauaagg..................	1	1	T6P
........................................................................ggacggagaacugauaagg..................	71	0	T6P
........................................................................ggacgAagaacugauaaggg.................	1	1	T6P
........................................................................ggacggagaacugauaaggA.................	9	1	T6P
........................................................................ggacggaUaacugauaaggg.................	1	1	T6P
........................................................................Ugacggagaacugauaaggg.................	9	1	T6P
........................................................................ggacggagaacugauaaggg.................	72	0	T6P
........................................................................Cgacggagaacugauaaggg.................	2	1	T6P
........................................................................ggaGggagaacugauaaggg.................	1	1	T6P
........................................................................ggacggagaacugauaagggU................	4	1	T6P
........................................................................ggacggagaacugauaaggAc................	1	1	T6P
........................................................................ggacgCagaacugauaagggc................	2	1	T6P
........................................................................gCacggagaacugauaagggc................	3	1	T6P
........................................................................ggacggagaacugauaagggA................	50	1	T6P
........................................................................ggacggagaacugauaagggG................	1	1	T6P
........................................................................ggacggagaacuCauaagggc................	1	1	T6P
........................................................................ggaUggagaacugauaagggc................	1	1	T6P
........................................................................Agacggagaacugauaagggc................	1	1	T6P
........................................................................Ugacggagaacugauaagggc................	40	1	T6P
........................................................................gUacggagaacugauaagggc................	1	1	T6P
........................................................................Cgacggagaacugauaagggc................	2	1	T6P
........................................................................ggacggagGacugauaagggc................	2	1	T6P
........................................................................ggacggagUacugauaagggc................	1	1	T6P
........................................................................ggacggGgaacugauaagggc................	4	1	T6P
........................................................................ggacggagaacugauaagggc................	373	0	T6P
........................................................................ggacggagaacugauaagggcA...............	36	1	T6P
........................................................................ggacggagaacugauaagggcc...............	7	0	T6P
........................................................................ggacggagaacugauaagggcU...............	19	1	T6P
........................................................................ggacggagaacugauaagggcG...............	3	1	T6P
........................................................................ggacggagaacugauaagggcAu..............	3	1	T6P
........................................................................ggacggagaacugauaagggccA..............	3	1	T6P
........................................................................ggacggagaacugauaagggcUu..............	4	1	T6P
........................................................................ggacggagaacugauaagggccu..............	1	0	T6P
.........................................................................gacggagaacugauaagg..................	17	0	T6P
.........................................................................gacggagaacugauaaggA.................	2	1	T6P
```

Star Mature

gugaagaagaaggaaugguuuuuacauguccuuaucauucucgacuguccugucuguauaaauacaagaacuggacggagaacugauaagggccugugaaaauucaaua			
....gacggagaacugauaaggg.................	14	0	T6P
....gUcggagaacugauaagggc................	1	1	T6P
....gacggagaacugauaagggc................	89	0	T6P
....gacggagaacugauaagggA................	15	1	T6P
....Uacggagaacugauaagggc................	2	1	T6P
....gacggagaacugauaagggU................	3	1	T6P
....gacggagaacugauaagggcU...............	9	1	T6P
....gacggagaacugauaagggcA...............	11	1	T6P
....gacggagaacugauaagggccA..............	1	1	T6P
....gacggagaacugauaagggcUu..............	1	1	T6P
....gacggagaacugauaagggcAu..............	2	1	T6P
....acggagaacugauaaggg.................	2	0	T6P
....acggagaacugauaaggA.................	1	1	T6P
....acggagaacugauaaggC.................	1	1	T6P
....acggagaacugauaagggA................	1	1	T6P
....acggagaacugauaagggc................	63	0	T6P
....acggagaacugauaGgggc................	1	1	T6P
....acggagaacugauaagggcA...............	9	1	T6P
....acggagaacugauaagggcU...............	1	1	T6P
....cggagaacugauaagggU................	1	1	T6P
....cggagaacugauaagggc................	5	0	T6P
....cggagaacugauaagggcA...............	3	1	T6P
....cggagaacugauaagggcU...............	3	1	T6P
....cggagaacugauaagggcAu..............	1	1	T6P
....Uggagaacugauaagggccu..............	1	1	T6P

Provisional ID	:	Scaffold_4228_7048
Score total	:	290898.4
Score for star read(s)	:	3.9
Score for read counts	:	290888
Score for mfe	:	1.9
Score for randfold	:	1.6
Score for cons. seed	:	3
Total read count	:	570576
Mature read count	:	463862
Loop read count	:	0
Star read count	:	106714

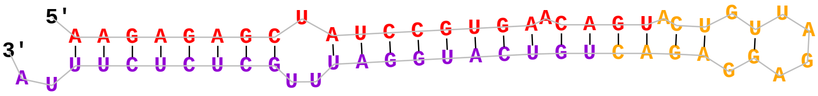

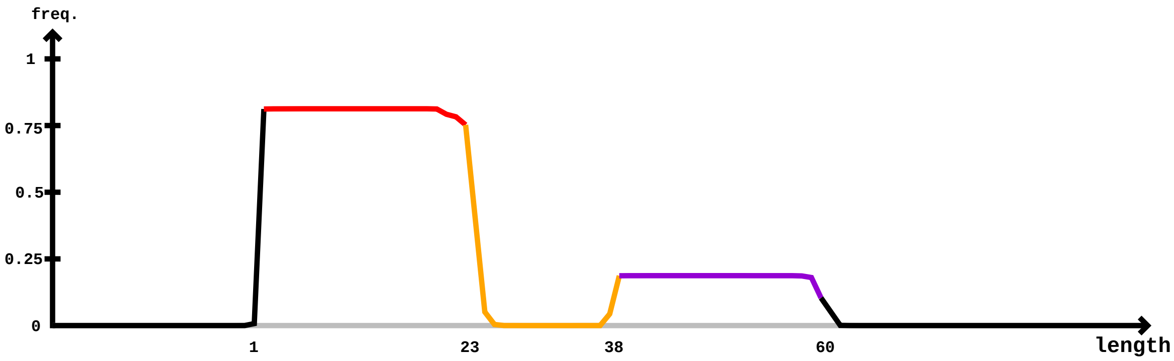

Mature **Star**

```
5'- uucaauggucaguagcuauaaagagagcuauccgugaacaguacuguuagaggagacugucauggauuugcucucuuuauuacacuggucaaaaucaucagguagcaacuac  -3'   obs
    uucaauggucaguagcuauaaagagagcuauccgugaacaguacuguuagaggagacugucauggauuugcucucuuuauuacacuggucaaaaucaucagguagcaacuac         exp
    .....((..((((.(..((((((((((.((((((((.((((.((.(....).))))))))))))))..))))))))))..)))))..))......................   reads   mm   sample
    uucaauggucaguagcuaua............................................................................................   1       0    T63
    ..................uaaagagagcuauccgugaac.........................................................................   3       0    T63
    ..................uaaagagagcuauccgugaaca........................................................................   1       0    T63
    ..................uaaagagagcuauccgugaacagu......................................................................   3       0    T63
    ..................uUaagagagcuauccgugaacagu......................................................................   10      1    T63
    ..................uUaagagagcuauccgugaacagua.....................................................................   3       1    T63
    ...................aaagagagcuauccgugaa..........................................................................   4       0    T63
    ...................aaagagagcuauccgugaac.........................................................................   71      0    T63
    ...................aaagagagcuauccgugGac.........................................................................   1       1    T63
    ...................aaagagagcuauccgugaUc.........................................................................   1       1    T63
    ...................aaagagagcuauccgugaGc.........................................................................   1       1    T63
    ...................Uaagagagcuauccgugaaca........................................................................   1       1    T63
    ...................aaagagagcuaucAgugaaca........................................................................   1       1    T63
    ...................aaagagagcuauccgugaaca........................................................................   39      0    T63
    ...................aGagagagcuauccgugaacag.......................................................................   1       1    T63
    ...................aaagagagcuauccgugaacaU.......................................................................   1       1    T63
    ...................aaagagagcuauccgugaacag.......................................................................   53      0    T63
    ...................Gaagagagcuauccgugaacagu......................................................................   1       1    T63
    ...................aaagagagcuauccgugaacagG......................................................................   1       1    T63
    ...................aaagagGgcuauccgugaacagu......................................................................   1       1    T63
    ...................aaagaCagcuauccgugaacagu......................................................................   1       1    T63
    ...................aGagagagcuauccgugaacagu......................................................................   2       1    T63
    ...................aaagagagcuauccgugaacagA......................................................................   7       1    T63
    ...................aaagagagUuauccgugaacagu......................................................................   2       1    T63
    ...................Uaagagagcuauccgugaacagu......................................................................   29      1    T63
    ...................aaagagagcuauccgugaacagu......................................................................   716     0    T63
    ...................aaagagGgcuauccgugaacagua.....................................................................   1       1    T63
    ...................Uaagagagcuauccgugaacagua.....................................................................   4       1    T63
    ...................aaagagagcuauccgugaacaguU.....................................................................   45      1    T63
    ...................aaagagagcuauccgugaacaguG.....................................................................   1       1    T63
    ...................aaagagagcuauccgugaacagua.....................................................................   36      0    T63
    ...................aaagagagcuauccgugaacaguaU....................................................................   57      1    T63
    ...................aaagagagcuauccgugaacaguaAu...................................................................   2       1    T63
```

```
                   Mature                              Star
uucaauggucaguagcuauaaagagagcuauccgugaacaguacuguuagaggagacugucauggauuugcucucuuuauuacacuggucaaaaucaucagguagcaacuac
...................aaagagagcuauccgugaacaguaUu...................................................................   1      1   T63
....................aagagagcuauccgugaa..........................................................................   175    0   T63
....................aagagagcuauccgugaG..........................................................................   1      1   T63
....................aagagagcuauccgugaU..........................................................................   6      1   T63
....................aagagagUuauccgugaac.........................................................................   4      1   T63
....................aagagagcAauccgugaac.........................................................................   3      1   T63
....................aagagagcuauccgCgaac.........................................................................   1      1   T63
....................aagagagcuaAccgugaac.........................................................................   1      1   T63
....................aUgagagcuauccgugaac.........................................................................   2      1   T63
....................aagagagcuauUcgugaac.........................................................................   1      1   T63
....................aagagagcuauccAugaac.........................................................................   1      1   T63
....................aagagagcuauccgugaGc.........................................................................   2      1   T63
....................aagagagcuauccguCaac.........................................................................   2      1   T63
....................aagaCagcuauccgugaac.........................................................................   1      1   T63
....................aagagagcuauccgugaUc.........................................................................   1      1   T63
....................aagagagcuauccUugaac.........................................................................   2      1   T63
....................aagagagcuauccgugUac.........................................................................   1      1   T63
....................aagagagcuCuccgugaac.........................................................................   1      1   T63
....................aagagagcuauccgugaaA.........................................................................   27     1   T63
....................aagagUgcuauccgugaac.........................................................................   2      1   T63
....................aagUgagcuauccgugaac.........................................................................   1      1   T63
....................aagagagcuauccgugaaG.........................................................................   4      1   T63
....................aGgagagcuauccgugaac.........................................................................   1      1   T63
....................aagagagcuaucUgugaac.........................................................................   1      1   T63
....................aagagagcuauccguAaac.........................................................................   2      1   T63
....................aagagagcuauccgugaaU.........................................................................   15     1   T63
....................aagagagcuauccgugaac.........................................................................   3708   0   T63
....................aagagagUuauccgugaaca........................................................................   1      1   T63
....................aagagagcuauccgugaaca........................................................................   1157   0   T63
....................aagagagcuauccgugaacG........................................................................   1      1   T63
....................aagagagcuauAcgugaaca........................................................................   1      1   T63
....................aCgagagcuauccgugaaca........................................................................   1      1   T63
....................aagagagcuauccgugaacU........................................................................   10     1   T63
....................aagagagcAauccgugaaca........................................................................   1      1   T63
....................aUgagagcuauccgugaaca........................................................................   1      1   T63
....................aagagaUcuauccgugaacag.......................................................................   1      1   T63
....................aagagagcuauccgugaGcag.......................................................................   1      1   T63
....................Gagagagcuauccgugaacag.......................................................................   1      1   T63
....................aagagagcuauccguAaacag.......................................................................   2      1   T63
....................aaUagagcuauccgugaacag.......................................................................   2      1   T63
....................aagagagcuauccgugaUcag.......................................................................   2      1   T63
....................aUgagagcuauccgugaacag.......................................................................   1      1   T63
....................aagagagcuauccgugaacag.......................................................................   3690   0   T63
....................aagagagcuauccgugaaUag.......................................................................   1      1   T63
....................aagagagcuauccgugUacag.......................................................................   3      1   T63
....................aagUgagcuauccgugaacag.......................................................................   1      1   T63
....................aagagagcuauccCugaacag.......................................................................   1      1   T63
....................aagagagUuauccgugaacag.......................................................................   5      1   T63
....................aagagagcuauccgugaacaU.......................................................................   11     1   T63
....................aagagagcuauccAugaacag.......................................................................   1      1   T63
....................aagagagcuauccgAgaacag.......................................................................   5      1   T63
....................aagagagcuauccgugaacaA.......................................................................   8      1   T63
....................aGgagagcuauccgugaacag.......................................................................   3      1   T63
....................aagagUgcuauccgugaacag.......................................................................   1      1   T63
....................aagagagcAauccgugaacag.......................................................................   3      1   T63
....................aagagCgcuauccgugaacag.......................................................................   1      1   T63
....................aagagagcuauccgugCacagu......................................................................   6      1   T63
....................aagGgagcuauccgugaacagu......................................................................   9      1   T63
....................aagagUgcuauccgugaacagu......................................................................   38     1   T63
....................aagagagcuauccgugaacaAu......................................................................   7      1   T63
....................aagagagcuauccgugaacagu......................................................................   96564  0   T63
....................aagagagcuauccAugaacagu......................................................................   45     1   T63
....................Uagagagcuauccgugaacagu......................................................................   1      1   T63
....................aagaAagcuauccgugaacagu......................................................................   4      1   T63
....................aagagagcuaAccgugaacagu......................................................................   5      1   T63
....................aagagagcuauccgugaacaCu......................................................................   14     1   T63
....................aagagaUcuauccgugaacagu......................................................................   7      1   T63
....................aagagagcuaucAgugaacagu......................................................................   6      1   T63
....................aagagagUuauccgugaacagu......................................................................   87     1   T63
....................aagagagcuauccgugGacagu......................................................................   35     1   T63
```

Mature Star

```
uucaauggucaguagcuauaaagagagcuauccgugaacaguacuguuagaggagacugucauggauuugcucucuuuauuacacuggucaaaaucaucagguagcaacuac
....................aagagagcGauccgugaacagu......................................................................	2	1	T63
....................Gagagagcuauccgugaacagu......................................................................	3	1	T63
....................aagagGgcuauccgugaacagu......................................................................	34	1	T63
....................aagagagcAauccgugaacagu......................................................................	53	1	T63
....................aagagCgcuauccgugaacagu......................................................................	20	1	T63
....................aagUgagcuauccgugaacagu......................................................................	32	1	T63
....................aagagagcuauccgugaacUgu......................................................................	10	1	T63
....................aagagagcuauccCugaacagu......................................................................	9	1	T63
....................aagagaCcuauccgugaacagu......................................................................	7	1	T63
....................Cagagagcuauccgugaacagu......................................................................	1	1	T63
....................aagaCagcuauccgugaacagu......................................................................	3	1	T63
....................aagagagcuauccgugaacagG......................................................................	150	1	T63
....................aagagagcuauccgugaacagC......................................................................	92	1	T63
....................aagagagcuauUcgugaacagu......................................................................	32	1	T63
....................aaAagagcuauccgugaacagu......................................................................	10	1	T63
....................aagagagcuGuccgugaacagu......................................................................	3	1	T63
....................aagagagAuauccgugaacagu......................................................................	6	1	T63
....................aagagagcuauccgugaGcagu......................................................................	13	1	T63
....................aagagagcuauccgugaacaUu......................................................................	25	1	T63
....................aagagagcuauccgugaUcagu......................................................................	7	1	T63
....................aagagagcuauAcgugaacagu......................................................................	2	1	T63
....................aagagagcuauGcgugaacagu......................................................................	6	1	T63
....................aaUagagcuauccgugaacagu......................................................................	6	1	T63
....................aagagagcuauccgCgaacagu......................................................................	8	1	T63
....................aagagagcuauccguAaacagu......................................................................	22	1	T63
....................aagagagcuCuccgugaacagu......................................................................	24	1	T63
....................aagagagcuauccgugaaGagu......................................................................	3	1	T63
....................aagagagcCauccgugaacagu......................................................................	5	1	T63
....................aagaUagcuauccgugaacagu......................................................................	8	1	T63
....................aagagagcuauccgugaaUagu......................................................................	27	1	T63
....................aagagagcuauccguUaacagu......................................................................	13	1	T63
....................aagagaAcuauccgugaacagu......................................................................	21	1	T63
....................aagagagcuauccgugaacagA......................................................................	465	1	T63
....................aagagagcuaGccgugaacagu......................................................................	1	1	T63
....................aagagagcuauccgugaacGgu......................................................................	12	1	T63
....................aagagagcuauccUugaacagu......................................................................	6	1	T63
....................aagagagcuaCccgugaacagu......................................................................	3	1	T63
....................aGgagagcuauccgugaacagu......................................................................	27	1	T63
....................aagagagcuUuccgugaacagu......................................................................	1	1	T63
....................aagagagGuauccgugaacagu......................................................................	3	1	T63
....................aagagagcuauccgGgaacagu......................................................................	10	1	T63
....................aagagagcuaucGgugaacagu......................................................................	8	1	T63
....................aaCagagcuauccgugaacagu......................................................................	5	1	T63
....................aCgagagcuauccgugaacagu......................................................................	16	1	T63
....................aUgagagcuauccgugaacagu......................................................................	35	1	T63
....................aagagagcuaucUgugaacagu......................................................................	23	1	T63
....................aagagagcuauccgAgaacagu......................................................................	24	1	T63
....................aagagagcuauccgugaaAagu......................................................................	10	1	T63
....................aagagagcuauccguCaacagu......................................................................	15	1	T63
....................aagagagcuauccgugUacagu......................................................................	53	1	T63
....................aagagagUuauccgugaacagua.....................................................................	10	1	T63
....................aagagagcAauccgugaacagua.....................................................................	6	1	T63
....................aagagagcuauUcgugaacagua.....................................................................	2	1	T63
....................aagagagcuauccgugaacaguC.....................................................................	21	1	T63
....................aagagagcuaucGgugaacagua.....................................................................	1	1	T63
....................aagagaAcuauccgugaacagua.....................................................................	1	1	T63
....................aagagagcuauccAugaacagua.....................................................................	5	1	T63
....................aCgagagcuauccgugaacagua.....................................................................	5	1	T63
....................aagagagcuauccgugaacaguG.....................................................................	14	1	T63
....................aagagagcuauccgAgaacagua.....................................................................	3	1	T63
....................aagagUgcuauccgugaacagua.....................................................................	2	1	T63
....................aagagagcuCuccgugaacagua.....................................................................	4	1	T63
....................aagagagcuauccgCgaacagua.....................................................................	1	1	T63
....................aaUagagcuauccgugaacagua.....................................................................	1	1	T63
....................aGgagagcuauccgugaacagua.....................................................................	3	1	T63
....................aagagGgcuauccgugaacagua.....................................................................	3	1	T63
....................aagagagcuauccgugaacaguU.....................................................................	4826	1	T63
....................Gagagagcuauccgugaacagua.....................................................................	1	1	T63
....................aUgagagcuauccgugaacagua.....................................................................	4	1	T63
....................aagUgagcuauccgugaacagua.....................................................................	2	1	T63
```

```
                    Mature                                    Star
uucaauggucaguagcuauaaagagagcuauccgugaacaguacuguuagaggagacugucauggauuugcucucuuuauuacacuggucaaaaucaucagguagcaacuac
....................aagagagcuauccgugaacagua.....................................................................  9548  0  T63
....................aagagagcuauccgugaaUagua.....................................................................  1     1  T63
....................aagagagcuauccgugGacagua.....................................................................  2     1  T63
....................aagagagcuaucAgugaacagua.....................................................................  1     1  T63
....................aagagagcuauccUugaacagua.....................................................................  1     1  T63
....................aagagagcuauccgGgaacagua.....................................................................  3     1  T63
....................aagagagcuaucUgugaacagua.....................................................................  3     1  T63
....................aagagagcuauccCugaacagua.....................................................................  1     1  T63
....................aagagagcuauccgugUacagua.....................................................................  7     1  T63
....................aagagagcuauccgugaUcagua.....................................................................  3     1  T63
....................aagagagcuauccgugaacagAa.....................................................................  22    1  T63
....................aagagagcuauccgugaGcagua.....................................................................  2     1  T63
....................aagagagcuauccgugaacagCa.....................................................................  1     1  T63
....................aagagagcuauccgugaacaCua.....................................................................  1     1  T63
....................aagagagcuauccgugaacaAua.....................................................................  2     1  T63
....................aagagagcuaCccgugaacagua.....................................................................  1     1  T63
....................aaAagagcuauccgugaacagua.....................................................................  3     1  T63
....................aagagagcuauccgugaacaUua.....................................................................  2     1  T63
....................aagagagcuauccgugaacaguaG....................................................................  15    1  T63
....................aagagagcuauccgugaacaguUc....................................................................  2     1  T63
....................aagagagcuauccgugaacaguaU....................................................................  6653  1  T63
....................aagagagcuauccgugaacaguaA....................................................................  346   1  T63
....................aagagagcuauccgugaacaguGc....................................................................  1     1  T63
....................aagagagcuauccgugaacaguac....................................................................  21    0  T63
....................aagagagcuauccgugaacaguacu...................................................................  9     0  T63
....................aagagagcuauccgugaacaguacA...................................................................  2     1  T63
....................aagagagcuauccgugaacaguaUu...................................................................  269   1  T63
....................aagagagcuauccgugaacaguaAu...................................................................  239   1  T63
....................aagagagcuauccgugaacaguaGu...................................................................  2     1  T63
....................aagagagcuauccgugaacaguacugu.................................................................  3     0  T63
.....................agagagcuauccgugaaca........................................................................  2     0  T63
.....................agagagcuauccgugaacag.......................................................................  2     0  T63
.....................aAagagcuauccgugaacagu......................................................................  1     1  T63
.....................agagagcuauccgAgaacagu......................................................................  1     1  T63
.....................agagagcuauccgugaacagu......................................................................  73    0  T63
.....................agagagcuauccgugaacaCu......................................................................  1     1  T63
.....................agagagcuauccgugaacaAu......................................................................  1     1  T63
.....................agaUagcuauccgugaacagu......................................................................  1     1  T63
.....................agagagcuauccgugaacagua.....................................................................  16    0  T63
.....................agagagcuauccgCgaacagua.....................................................................  1     1  T63
.....................agagagcuauccgugaacaguU.....................................................................  4     1  T63
.....................agagagcuauccgugaacaguaU....................................................................  6     1  T63
.....................agagagcuauccgugaacaguaA....................................................................  1     1  T63
.....................agagagcuauccgugaacaguaAu...................................................................  1     1  T63
.....................agagagcuauccgugaacaguacuguu................................................................  2     0  T63
......................gagagcuauccgugaacagu......................................................................  8     0  T63
......................Aagagcuauccgugaacagu......................................................................  5     1  T63
......................gagagcuauccgugaacagua.....................................................................  1     0  T63
......................gagagcuauccgugaacaguU.....................................................................  1     1  T63
.......................agagcuauccgugaacagu......................................................................  13    0  T63
.......................agagcuauccgugaacagua.....................................................................  1     0  T63
........................gagcuauccgugaacagu......................................................................  3     0  T63
........................gagcuauccgugaacaguU.....................................................................  1     1  T63
.........................agcuauccgugaacaguaU....................................................................  1     1  T63
.............................auccgugaacaguacugu.................................................................  1     0  T63
..........................................acuguuagaggagacugucaugg...............................................  1     0  T63
............................................uguuagaggagacugucauggauu............................................  1     0  T63
............................................uguuagaggagacugucauggauuugc.........................................  2     0  T63
.......................................................acugucauggauuugcucucu....................................  2     0  T63
.......................................................acugucauggauuugcucucuu...................................  3     0  T63
.......................................................acugucauggauuugcucucuAu..................................  1     1  T63
.......................................................acugucauggauuugcucucuuu..................................  9     0  T63
.......................................................acugucauggauuugcucucuuua.................................  1     0  T63
........................................................cugucauggauuugcucu......................................  4     0  T63
........................................................cugucauggauuugcucuc.....................................  1     0  T63
........................................................cugucauggauuugcucuA.....................................  1     1  T63
........................................................cugucauggauuugcucucu....................................  50    0  T63
........................................................cugucauggauuugcucucA....................................  1     1  T63
........................................................cugucauggauuugcucuUu....................................  2     1  T63
........................................................cugucauggauuugcucCcuu...................................  1     1  T63
```

```
                    Mature                              Star
uucaauggucaguagcuauaaagagagcuauccgugaacaguacuguuagaggagacugucauggauuugcucucuuuauuacacuggucaaaaucaucagguagcaacuac
........................................................cugucauggGuuugcucucuu...................................   2     1   T63
........................................................cugucaAggauuugcucucuu...................................   1     1   T63
........................................................cugucauggauuugcucucuu...................................   277   0   T63
........................................................cugucauggauuugcucucuG...................................   1     1   T63
........................................................cugucauggauuuAcucucuu...................................   1     1   T63
........................................................cugucauggauuugcAcucuu...................................   1     1   T63
........................................................Uugucauggauuugcucucuu...................................   1     1   T63
........................................................cugucauggauuuAcucucuuu..................................   5     1   T63
........................................................Uugucauggauuugcucucuuu..................................   2     1   T63
........................................................cugucauggauAugcucucuuu..................................   1     1   T63
........................................................cugucauggauuugcAcucuuu..................................   2     1   T63
........................................................cugGcauggauuugcucucuuu..................................   1     1   T63
........................................................cugucaugCauuugcucucuuu..................................   2     1   T63
........................................................cugAcauggauuugcucucuuu..................................   3     1   T63
........................................................cugucauggauuugcGcucuuu..................................   1     1   T63
........................................................cugucauggCuuugcucucuuu..................................   1     1   T63
........................................................cugucauggauuugcucucuuC..................................   5     1   T63
........................................................cugucauggauuugcucuUuuu..................................   4     1   T63
........................................................cugucauggauuugUucucuuu..................................   3     1   T63
........................................................cugucauggauuugcucucuuu..................................   3183  0   T63
........................................................cugucaugUauuugcucucuuu..................................   1     1   T63
........................................................cugucauggauuugcucucuGu..................................   1     1   T63
........................................................cugucauggauuugcucucCuu..................................   1     1   T63
........................................................cugucauggauuugcucucuuA..................................   11    1   T63
........................................................cuCucauggauuugcucucuuu..................................   1     1   T63
........................................................cCgucauggauuugcucucuuu..................................   1     1   T63
........................................................cugucauggGuuugcucucuuu..................................   7     1   T63
........................................................cugucGuggauuugcucucuuu..................................   1     1   T63
........................................................Augucauggauuugcucucuuu..................................   1     1   T63
........................................................cugucauggauuugcuUucuuu..................................   1     1   T63
........................................................cugucauggauuugcucucuuuU.................................   167   1   T63
........................................................cugucauggGuuugcucucuuua.................................   2     1   T63
........................................................cugucauggauuuCcucucuuua.................................   1     1   T63
........................................................cugucauggauuugcucucuuuG.................................   2     1   T63
........................................................cugucaugAauuugcucucuuua.................................   1     1   T63
........................................................Uugucauggauuugcucucuuua.................................   3     1   T63
........................................................cugucauggauuugcucucuuua.................................   2253  0   T63
........................................................cugucauggauuugcucGcuuua.................................   1     1   T63
........................................................cuCucauggauuugcucucuuua.................................   1     1   T63
........................................................cugucauggauuAgcucucuuua.................................   1     1   T63
........................................................cugucauggauuuAcucucuuua.................................   6     1   T63
........................................................cugucauggauuugcAcucuuua.................................   1     1   T63
........................................................cugucauggCuuugcucucuuua.................................   4     1   T63
........................................................cugucauggUuuugcucucuuua.................................   1     1   T63
........................................................cugucauggauuugcucucuuCa.................................   1     1   T63
........................................................cugAcauggauuugcucucuuua.................................   1     1   T63
........................................................cugucGuggauuugcucucuuua.................................   1     1   T63
........................................................cugucauggaGuugcucucuuua.................................   2     1   T63
........................................................cugucauggauuugcucucuuAa.................................   7     1   T63
........................................................cugucauggauuugcucucuGua.................................   1     1   T63
........................................................cugucCuggauuugcucucuuua.................................   1     1   T63
........................................................cugucauggauuugcucuUuuua.................................   2     1   T63
........................................................cugucauggauuugcucucuuuC.................................   5     1   T63
........................................................cugucauggaGuugcucucuuuau................................   3     1   T63
........................................................cugucauggauuugcucucuuuau................................   92    0   T63
........................................................cugucauggauuugcucucuuuaG................................   1     1   T63
........................................................cugucauggauuugcucucuuuaA................................   29    1   T63
........................................................cugucauggauuugcucucuuuaC................................   15    1   T63
........................................................cugucauggauuugcucucuuuUu................................   35    1   T63
........................................................cugucauAgauuugcucucuuuau................................   1     1   T63
........................................................cugucauggauuugcucucuuuCu................................   1     1   T63
........................................................cugucauggauuugcucucuuuauu...............................   4     0   T63
........................................................cugucauggauuugcucucuuuaAu...............................   5     1   T63
........................................................cugucauggauuugcucucuuuaCu...............................   1     1   T63
........................................................cugucauggauuugcucucuuuUuu...............................   6     1   T63
.........................................................ugucauggauuugcucuA.....................................   1     1   T63
.........................................................ugucauggauuugcucuc.....................................   7     0   T63
.........................................................uguUauggauuugcucuc.....................................   1     1   T63
.........................................................ugucauggauuugcucuG.....................................   1     1   T63
.........................................................ugucauggauuuAcucucu....................................   1     1   T63
```

```
                    Mature                               Star
uucaauggucaguagcuauaaagagagcuauccgugaacaguacuguuagaggagacugucauggauuugcucucuuuauuacacuggucaaaaucaucagguagcaacuac
.........................................................ugucauggauuugcucucu....................................    87      0    T63
.........................................................ugucauggauuugcucAcuu...................................    1       1    T63
.........................................................ugucauggauuugcucucuA...................................    2       1    T63
.........................................................ugucauggauuugcucucuu...................................    576     0    T63
.........................................................ugucauggauuugcucCcuu...................................    1       1    T63
.........................................................ugucauggauuGgcucucuu...................................    1       1    T63
.........................................................Agucauggauuugcucucuu...................................    2       1    T63
.........................................................ugucauggaCuugcucucuu...................................    1       1    T63
.........................................................ugucauggauuugcucucuuu..................................    7992    0    T63
.........................................................ugucaGggauuugcucucuuu..................................    2       1    T63
.........................................................ugucaugCauuugcucucuuu..................................    1       1    T63
.........................................................ugucauggauuuAcucucuuu..................................    9       1    T63
.........................................................ugucauggauuugcucucCuu..................................    4       1    T63
.........................................................ugAcauggauuugcucucuuu..................................    1       1    T63
.........................................................ugucaAggauuugcucucuuu..................................    1       1    T63
.........................................................ugucauggUuuugcucucuuu..................................    18      1    T63
.........................................................uAucauggauuugcucucuuu..................................    2       1    T63
.........................................................Agucauggauuugcucucuuu..................................    22      1    T63
.........................................................ugucauggauuugcucAcuuu..................................    5       1    T63
.........................................................ugucauggauuugcucucuuG..................................    2       1    T63
.........................................................ugucauggCuuugcucucuuu..................................    2       1    T63
.........................................................ugucauggauuuUcucucuuu..................................    4       1    T63
.........................................................uCucauggauuugcucucuuu..................................    2       1    T63
.........................................................ugucUuggauuugcucucuuu..................................    1       1    T63
.........................................................ugucauggauuugUucucuuu..................................    3       1    T63
.........................................................uUucauggauuugcucucuuu..................................    2       1    T63
.........................................................uguUauggauuugcucucuuu..................................    4       1    T63
.........................................................ugucauggauuCgcucucuuu..................................    1       1    T63
.........................................................Cgucauggauuugcucucuuu..................................    9       1    T63
.........................................................ugucauggaGuugcucucuuu..................................    10      1    T63
.........................................................ugucauggauuugcucuUuuu..................................    15      1    T63
.........................................................ugCcauggauuugcucucuuu..................................    1       1    T63
.........................................................ugucauggauuugcuUucuuu..................................    1       1    T63
.........................................................Ggucauggauuugcucucuuu..................................    6       1    T63
.........................................................ugucauggaCuugcucucuuu..................................    4       1    T63
.........................................................ugucauggauuugcAcucuuu..................................    1       1    T63
.........................................................ugucauggGuuugcucucuuu..................................    1       1    T63
.........................................................ugucaugAauuugcucucuuu..................................    1       1    T63
.........................................................ugucauggauuugcCcucuuu..................................    1       1    T63
.........................................................ugucaugUauuugcucucuuu..................................    1       1    T63
.........................................................ugucauggauuugcucuGuuu..................................    2       1    T63
.........................................................ugucauggauuugcucucuuA..................................    19      1    T63
.........................................................ugucaCggauuugcucucuuu..................................    2       1    T63
.........................................................ugucauggauuugcucucAuua.................................    1       1    T63
.........................................................ugucaCggauuugcucucuuua.................................    1       1    T63
.........................................................ugucauggauuugcucucuuua.................................    8030    0    T63
.........................................................ugucauggauuugcuGucuuua.................................    1       1    T63
.........................................................ugucauggauuugcucucuuuG.................................    6       1    T63
.........................................................Cgucauggauuugcucucuuua.................................    4       1    T63
.........................................................ugucauggauuugcucAcuuua.................................    4       1    T63
.........................................................ugucauggauuugcucuAuuua.................................    1       1    T63
.........................................................ugucauggauuugcucuUuuua.................................    21      1    T63
.........................................................ugucauggauuuCcucucuuua.................................    1       1    T63
.........................................................ugucauggauuugcuUucuuua.................................    2       1    T63
.........................................................ugucauggauuugcucucCuua.................................    3       1    T63
.........................................................ugGcauggauuugcucucuuua.................................    1       1    T63
.........................................................ugucauggauuAgcucucuuua.................................    1       1    T63
.........................................................ugucauggauuugcucGcuuua.................................    1       1    T63
.........................................................ugucUuggauuugcucucuuua.................................    2       1    T63
.........................................................ugucauggauuugcucuGuuua.................................    1       1    T63
.........................................................ugucauggauuugcucucuuGa.................................    1       1    T63
.........................................................ugucauggauuugcucucuuuU.................................    619     1    T63
.........................................................ugucaugAauuugcucucuuua.................................    1       1    T63
.........................................................ugucauggCuuugcucucuuua.................................    1       1    T63
.........................................................uUucauggauuugcucucuuua.................................    3       1    T63
.........................................................ugucauggUuuugcucucuuua.................................    11      1    T63
.........................................................uguUauggauuugcucucuuua.................................    3       1    T63
.........................................................ugucaugUauuugcucucuuua.................................    1       1    T63
.........................................................ugCcauggauuugcucucuuua.................................    1       1    T63
.........................................................uCucauggauuugcucucuuua.................................    4       1    T63
```

Mature **Star**

```
uucaauggucaguagcuauaaagagagcuauccgugaacaguacuguuagaggagacugucauggauuugcucucuuuauuacacuggucaaaaucaucagguagcaacuac
.........................................................ugucauggauuugcAcucuuua.................................	1	1	T63
.........................................................ugucauggauuugGucucuuua.................................	1	1	T63
.........................................................ugucaGggauuugcucucuuua.................................	1	1	T63
.........................................................uguAauggauuugcucucuuua.................................	1	1	T63
.........................................................Agucauggauuugcucucuuua.................................	25	1	T63
.........................................................ugucauggGuuugcucucuuua.................................	2	1	T63
.........................................................ugucauggauuuAcucucuuua.................................	17	1	T63
.........................................................uguGauggauuugcucucuuua.................................	1	1	T63
.........................................................ugucauggauuugcucucuuuC.................................	12	1	T63
.........................................................ugucauggauuugcuAucuuua.................................	1	1	T63
.........................................................ugucauggauuugcucucuuAa.................................	1	1	T63
.........................................................ugucauggauuugUucucuuua.................................	5	1	T63
.........................................................Ggucauggauuugcucucuuua.................................	8	1	T63
.........................................................ugucauggaCuugcucucuuua.................................	7	1	T63
.........................................................ugucauggauuugcucucuuuaC................................	62	1	T63
.........................................................ugucauggaGuugcucucuuuau................................	1	1	T63
.........................................................ugucauggauuugcucucuuuau................................	217	0	T63
.........................................................ugucauggauuugcucucuuuaG................................	1	1	T63
.........................................................Ggucauggauuugcucucuuuau................................	1	1	T63
.........................................................ugucauggauuugcucucuuuaA................................	50	1	T63
.........................................................ugucauggUuuugcucucuuuau................................	1	1	T63
.........................................................ugucauggauuugcucucuuuUu................................	44	1	T63
.........................................................Agucauggauuugcucucuuuau................................	1	1	T63
.........................................................ugucauggauuugcucucuuuauA...............................	5	1	T63
.........................................................ugucauggauuugcucucuuuauu...............................	32	0	T63
.........................................................ugucauggauuugcucucuuuUuu...............................	3	1	T63
.........................................................ugucauggauuugcucucuuuaAu...............................	4	1	T63
.........................................................ugucauggauuugcucucuuuauG...............................	1	1	T63
.........................................................ugucauggauuugcucucuuuaCu...............................	1	1	T63
.........................................................ugucauggauuugcucucuuuauuU..............................	2	1	T63
.........................................................ugucauggauuugcucucuuuaAua..............................	2	1	T63
..........................................................gucauggauuugcucucuu...................................	3	0	T63
..........................................................gucauggaGuugcucucuuu..................................	1	1	T63
..........................................................gucauggauuugcucucuuu..................................	32	0	T63
..........................................................gucauggauuugcucucuuuG.................................	2	1	T63
..........................................................gucauggauuugcucucuuua.................................	13	0	T63
..........................................................gucauggauuugcucucuuuU.................................	1	1	T63
..........................................................gucauggauuugcucucuuuau................................	1	0	T63
..........................................................gucauggauuugcucucuuuauA...............................	1	1	T63
...........................................................ucauggauuugcucucuuu..................................	1	0	T63
...........................................................ucauggauuugcucucuuuU.................................	1	1	T63
...........................................................ucauggauuugcucucuuua.................................	2	0	T63
............................................................cauggauuugcucucuuu..................................	1	0	T63
.............................................................auggauuugcucucuuua.................................	1	0	T63
...............................................................................uuacacuggucaaaaucauc.............	8	0	T63
...............................................................................uuacacuggucaaaaucauca............	3	0	T63
................................................................................uacacuggucaaaaucauc.............	1	0	T63

.................Uuaaagagagcuauccgugaacagu......................................................................	1	1	MOL
..................uaaagagagcuauccgugaac.........................................................................	2	0	MOL
..................uUaagagagcuauccgugaaca........................................................................	1	1	MOL
..................uaaagagagcuauccgugaaca........................................................................	3	0	MOL
..................uaaagagagcuauccgugaacag.......................................................................	1	0	MOL
..................uaaagagagcuauccgugaacagu......................................................................	3	0	MOL
..................uUaagagagcuauccgugaacagu......................................................................	1	1	MOL
..................uUaagagagcuauccgugaacagua.....................................................................	1	1	MOL
...................aaagagagcuauccgugaa..........................................................................	4	0	MOL
...................aaagagagcuauccgugaac.........................................................................	56	0	MOL
...................aaagagagcuauccgugaaA.........................................................................	3	1	MOL
...................aaagagagcuauccgugaacU........................................................................	2	1	MOL
...................aaagagagcuauccgugaaca........................................................................	67	0	MOL
...................aaagCgagcuauccgugaaca........................................................................	1	1	MOL
...................aaagagagcuauccgugaacag.......................................................................	90	0	MOL
...................aaagaAagcuauccgugaacag.......................................................................	1	1	MOL
...................aaagagagcuauccgugaacagu......................................................................	440	0	MOL
...................Uaagagagcuauccgugaacagu......................................................................	14	1	MOL
...................aaagagagcuauccgugaacagA......................................................................	15	1	MOL
...................aaagagagcuauccgugaacagC......................................................................	2	1	MOL
...................Gaagagagcuauccgugaacagu......................................................................	2	1	MOL
...................aaagagUgcuauccgugaacagu......................................................................	2	1	MOL
```

Mature / Star			
uucaauggucaguagcuauaaagagagcuauccgugaacaguacuguuagaggagacugucauggauuugcucucuuuauuacacuggucaaaaucaucagguagcaacuac			
...................aaagagagcuauccgugaacaguU...	16	1	MOL
...................Uaagagagcuauccgugaacagua...	2	1	MOL
...................aaagagagcuauccgugaacaguG...	1	1	MOL
...................aaagagagcuauccgugaacaguC...	1	1	MOL
...................aaagagagcuauccgugaacagua...	42	0	MOL
...................aaagagagcuauccgugaacaguaU..	25	1	MOL
...................aaagagagcuauccgugaacaguaA..	1	1	MOL
...................aaagagagcuauccgugaacaguaAu...	3	1	MOL
...................aaagagagcuauccgugaacaguaUu...	2	1	MOL
....................aagagagcuauccgugaa..	43	0	MOL
....................aGgagagcuauccgugaac...	2	1	MOL
....................aagagagcuauccguAaac...	1	1	MOL
....................aagagagcuauccgugCac...	1	1	MOL
....................aagagagcuauUcgugaac...	1	1	MOL
....................aagagagcuauccgAgaac...	1	1	MOL
....................aagagagcuauccgugaUc...	1	1	MOL
....................aagagagcuauccgugaaA...	15	1	MOL
....................aagagagcuauccgugaaU...	2	1	MOL
....................aagagagAuauccgugaac...	1	1	MOL
....................aagagagcuauccgugaac...	1177	0	MOL
....................aagagagcuauccgugUac...	1	1	MOL
....................aagaUagcuauccgugaac...	2	1	MOL
....................aagagagUuauccgugaac...	3	1	MOL
....................aCgagagcuauccgugaac...	1	1	MOL
....................aagagaAcuauccgugaac...	1	1	MOL
....................aagagagcuauccgugaacG..	1	1	MOL
....................aagagagcuauccgugaaca..	1363	0	MOL
....................aagagagcCauccgugaaca..	1	1	MOL
....................aagagagcuauccguCaaca..	1	1	MOL
....................aagagagcuauccgugaaAa..	4	1	MOL
....................aagaUagcuauccgugaaca..	1	1	MOL
....................aagagagcuauccgugCaca..	4	1	MOL
....................aagagGgcuauccgugaaca..	1	1	MOL
....................aagagagcuauUcgugaaca..	2	1	MOL
....................aagagagcuauccgugaacU..	6	1	MOL
....................aagagagcuauUcgugaacag...	4	1	MOL
....................aagagagcuauccAugaacag...	2	1	MOL
....................aagagagcuauccgugaacUg...	1	1	MOL
....................aagagagcuauccgugaaAag...	1	1	MOL
....................aagagagcuauccgugaacaA...	25	1	MOL
....................aagagagcuCuccgugaacag...	1	1	MOL
....................aagaUagcuauccgugaacag...	2	1	MOL
....................aUgagagcuauccgugaacag...	1	1	MOL
....................aCgagagcuauccgugaacag...	2	1	MOL
....................aagagagcuauccgugaaUag...	2	1	MOL
....................aagagagcuauccgugaacGg...	2	1	MOL
....................aagaCagcuauccgugaacag...	1	1	MOL
....................aGgagagcuauccgugaacag...	3	1	MOL
....................aagagagcuauccgugUacag...	3	1	MOL
....................aagagagcuaucGgugaacag...	2	1	MOL
....................aagagagcuauccgugCacag...	1	1	MOL
....................aagagagcuauccgugaacaU...	18	1	MOL
....................aagagagcuauccgugaacag...	4308	0	MOL
....................aagagagcuauccgugaacaC...	1	1	MOL
....................aagagGgcuauccgugaacag...	3	1	MOL
....................aagagagUuauccgugaacag...	5	1	MOL
....................aagagagcAauccgugaacag...	4	1	MOL
....................aagagUgcuauccgugaacag...	1	1	MOL
....................aagGgagcuauccgugaacag...	1	1	MOL
....................aagagCgcuauccgugaacag...	3	1	MOL
....................aagagagcuauccgugGacag...	4	1	MOL
....................aagagagcuauccgugaUcag...	1	1	MOL
....................aaCagagcuauccgugaacag...	1	1	MOL
....................aagagagcuauccguAaacag...	1	1	MOL
....................aagagagcuauAcgugaacag...	1	1	MOL
....................aagagagcuauccgCgaacag...	2	1	MOL
....................Gagagagcuauccgugaacagu..	4	1	MOL
....................aaUagagcuauccgugaacagu..	7	1	MOL
....................aagaCagcuauccgugaacagu..	3	1	MOL
....................aagagaAcuauccgugaacagu..	13	1	MOL

Mature **Star**

```
uucaauggucaguagcuauaaagagagcuauccgugaacaguacuguuagaggagacugucauggauuugcucucuuuauuacacuggucaaaaucaucagguagcaacuac
....................aagagagcuUuccgugaacagu......................................................................   1       1   MOL
....................aaCagagcuauccgugaacagu......................................................................   2       1   MOL
....................aagagagcuauccgugaacagu......................................................................   48799   0   MOL
....................aagagagcuauccgugaaGagu......................................................................   3       1   MOL
....................aagagaUcuauccgugaacagu......................................................................   5       1   MOL
....................aagagagcuauccguCaacagu......................................................................   12      1   MOL
....................aagagagcuauccgugaacagC......................................................................   55      1   MOL
....................aagaAagcuauccgugaacagu......................................................................   1       1   MOL
....................aagagagcuauccgugaGcagu......................................................................   7       1   MOL
....................aagagagcuaCccgugaacagu......................................................................   1       1   MOL
....................aGgagagcuauccgugaacagu......................................................................   12      1   MOL
....................aagagagcuauccUugaacagu......................................................................   1       1   MOL
....................aagagagcuauUcgugaacagu......................................................................   24      1   MOL
....................aagagagcuauccgCgaacagu......................................................................   7       1   MOL
....................aagagagcuauccgugCacagu......................................................................   4       1   MOL
....................aUgagagcuauccgugaacagu......................................................................   23      1   MOL
....................aagagagcuaucGgugaacagu......................................................................   1       1   MOL
....................aagagagcuaAccgugaacagu......................................................................   2       1   MOL
....................aagagagcuauccgugaaAagu......................................................................   5       1   MOL
....................Uagagagcuauccgugaacagu......................................................................   3       1   MOL
....................aagagagcuauccguUaacagu......................................................................   8       1   MOL
....................aagagagcuGuccgugaacagu......................................................................   1       1   MOL
....................aagagCgcuauccgugaacagu......................................................................   3       1   MOL
....................aagagagcuauccgugaacagG......................................................................   10      1   MOL
....................aagagagcuauccgugaacGgu......................................................................   1       1   MOL
....................aagagagcCauccgugaacagu......................................................................   2       1   MOL
....................aagagagcuauAcgugaacagu......................................................................   1       1   MOL
....................aagagagcuauccgugUacagu......................................................................   23      1   MOL
....................aagGgagcuauccgugaacagu......................................................................   3       1   MOL
....................aagagagcuCuccgugaacagu......................................................................   9       1   MOL
....................aagagGgcuauccgugaacagu......................................................................   20      1   MOL
....................aagagagAuauccgugaacagu......................................................................   1       1   MOL
....................aagagagcuauccgugaacaCu......................................................................   2       1   MOL
....................aagagagcuauccgugaacaUu......................................................................   14      1   MOL
....................aagagagcuauccgugaaUagu......................................................................   21      1   MOL
....................aaAagagcuauccgugaacagu......................................................................   4       1   MOL
....................aagagUgcuauccgugaacagu......................................................................   19      1   MOL
....................aagagagcuauccgugaUcagu......................................................................   7       1   MOL
....................aagagagcuauccAugaacagu......................................................................   11      1   MOL
....................aagagaCcuauccgugaacagu......................................................................   1       1   MOL
....................aagagagcuauccguAaacagu......................................................................   10      1   MOL
....................aagagagcuauccgugaacagA......................................................................   499     1   MOL
....................aagagagcuaucUgugaacagu......................................................................   18      1   MOL
....................aagagagcGauccgugaacagu......................................................................   5       1   MOL
....................aagagagcuauccgugGacagu......................................................................   16      1   MOL
....................aagagagcAauccgugaacagu......................................................................   27      1   MOL
....................aagagagcuauccgGgaacagu......................................................................   5       1   MOL
....................aagaUagcuauccgugaacagu......................................................................   3       1   MOL
....................aagagagUuauccgugaacagu......................................................................   41      1   MOL
....................aagagagGuauccgugaacagu......................................................................   3       1   MOL
....................aagUgagcuauccgugaacagu......................................................................   20      1   MOL
....................aCgagagcuauccgugaacagu......................................................................   12      1   MOL
....................aagagagcuauGcgugaacagu......................................................................   3       1   MOL
....................aagagagcuauccgugaacUgu......................................................................   2       1   MOL
....................aagagagcuauccgAgaacagu......................................................................   7       1   MOL
....................aagagagcuauccgugaacaAu......................................................................   7       1   MOL
....................aagagagcuauccAugaacagua.....................................................................   3       1   MOL
....................aagagagcuauUcgugaacagua.....................................................................   4       1   MOL
....................aagagagcuauccgugaacaUua.....................................................................   2       1   MOL
....................aagagagcuauccgugaaGagua.....................................................................   1       1   MOL
....................aagagagcuauccCugaacagua.....................................................................   1       1   MOL
....................aagUgagcuauccgugaacagua.....................................................................   2       1   MOL
....................aagagagcuauccgCgaacagua.....................................................................   1       1   MOL
....................aagagagcuauccgGgaacagua.....................................................................   3       1   MOL
....................aagagagcuauccgugaacaguU.....................................................................   1701    1   MOL
....................aagagagcuauccgugaacaguC.....................................................................   10      1   MOL
....................aagagagcuauccgugaacagCa.....................................................................   2       1   MOL
....................aagagagcuauccgugUacagua.....................................................................   5       1   MOL
....................aagagaCcuauccgugaacagua.....................................................................   1       1   MOL
....................aagGgagcuauccgugaacagua.....................................................................   1       1   MOL
```

**Mature** / **Star**			
uucaauggucaguagcuauaaagagagcuauccgugaacaguacuguuagaggagacugucauggauuugcucucuuuauuacacuggucaaaaucaucagguagcaacuac			
....................aagagagcuauccgugaacaCua...	1	1	MOL
....................aagagagcuauAcgugaacagua...	1	1	MOL
....................aagagagcuCuccgugaacagua...	1	1	MOL
....................aagagagcuaucUgugaacagua...	1	1	MOL
....................aagaUagcuauccgugaacagua...	1	1	MOL
....................aagagagcuauccgugaacaguG...	29	1	MOL
....................aagagagcuauccgugCacagua...	1	1	MOL
....................aagagagcuauccgugaacagAa...	25	1	MOL
....................aagagagcuauccgAgaacagua...	3	1	MOL
....................Gagagagcuauccgugaacagua...	2	1	MOL
....................aagagagcuauccgugaacGgua...	2	1	MOL
....................aagagUgcuauccgugaacagua...	3	1	MOL
....................aagagagcuauccgugaGcagua...	2	1	MOL
....................aagagagcuauccgugaacagua...	8534	0	MOL
....................aGgagagcuauccgugaacagua...	2	1	MOL
....................aagagagcuauccguCaacagua...	1	1	MOL
....................aagagaAcuauccgugaacagua...	3	1	MOL
....................Uagagagcuauccgugaacagua...	1	1	MOL
....................aagagagUuauccgugaacagua...	3	1	MOL
....................aagagagcuauccgugaacagGa...	2	1	MOL
....................aCgagagcuauccgugaacagua...	3	1	MOL
....................aagagagGuauccgugaacagua...	1	1	MOL
....................aagagagcuauccgugGacagua...	7	1	MOL
....................aagagagcuauccgugaaUagua...	4	1	MOL
....................aagagagcAauccgugaacagua...	3	1	MOL
....................aUgagagcuauccgugaacagua...	7	1	MOL
....................aagagagcuauUcgugaacaguac..	1	1	MOL
....................aagagagcuauccgugaacaguaU..	2178	1	MOL
....................aagagagcuauccgugaacaguac..	51	0	MOL
....................aagagagcuauccgugaacaguUc..	3	1	MOL
....................aagagagcuauccgugaacaguaG..	34	1	MOL
....................aagagagcuauccgugaacaguaA..	676	1	MOL
....................aagagagcuauccgugaacaguaUu...	129	1	MOL
....................aagagagcuauccgugaacaguaAu...	223	1	MOL
....................aagagagcuauccgugaacaguacA...	9	1	MOL
....................aagagagcuauccgugaacaguacG...	1	1	MOL
....................aagagagcuauccgugaacaguaGu...	3	1	MOL
....................aagagagcuauccgugaacaguacu...	12	0	MOL
....................aagagagcuauccgugaacaguUcu...	1	1	MOL
....................aagagagcuauccgugaacaguacugu...	1	0	MOL
.....................agagagcuauccgugaacag...	1	0	MOL
.....................agagagcuauccgugaacagu..	29	0	MOL
.....................agagagcuauccguAaacagu..	1	1	MOL
.....................agagagcuauccgugaacagua...	26	0	MOL
.....................agagagcuauccgugaacaguU...	1	1	MOL
.....................agagagcuauccgugaacaguaA..	3	1	MOL
.....................agagagcuauccgugaacaguaU..	2	1	MOL
.....................agagagcuauccgugaacaguac..	3	0	MOL
.....................agagagcuauccgugaacaguacuguu..	1	0	MOL
......................Uagagcuauccgugaacagu..	1	1	MOL
......................gagagcuauccgugaacagu..	2	0	MOL
......................gagagcuauccgugaacaguaU..	1	1	MOL
......................gagagcuauccgugaacaguacuguua...	1	0	MOL
.......................agagcuauccgugaacagu..	5	0	MOL
.......................agagcuauAcgugaacagu..	1	1	MOL
........................gagcuauccgugaacagu..	2	0	MOL
........................gagcuauccgugaacagua...	1	0	MOL
....uguuagaggagacugucauggauuugc...	3	0	MOL
....uuagaggagacugucauggauuugc...	1	0	MOL
....ggagaGugucauggauuugcucucuuu..................................	1	1	MOL
....ggagCcugucauggauuugcucucuuu..................................	1	1	MOL
....gaAacugucauggauuugcucucuuu..................................	1	1	MOL
....gagGcugucauggauuugcucucuuu..................................	1	1	MOL
....gGgacugucauggauuugcucucuuu..................................	1	1	MOL
....agaGugucauggauuugcucucu....................................	1	1	MOL
....agaGugucauggauuugcucucuuua.................................	3	1	MOL
....gaGugucauggauuugcucucuuu..................................	1	1	MOL
....gGcugucauggauuugcucucuuua.................................	1	1	MOL
....gaGugucauggauuugcucucuuua.................................	3	1	MOL
....acugucauggauuugcucucuu...................................	1	0	MOL

```
                    Mature                             Star
uucaauggucaguagcuauaaagagagcuauccgugaacaguacuguuagaggagacugucauggauuugcucucuuuauuacacuggucaaaaucaucagguagcaacuac
.......................................................acugucauggauuugcucucuuuU.................................    2      1    MOL
.......................................................Ucugucauggauuugcucucuuua.................................    2      1    MOL
........................................................cugucauggauuugcucu......................................    1      0    MOL
........................................................cugucauggauuugcucuc.....................................    4      0    MOL
........................................................cugucauggauuugcucucu....................................    40     0    MOL
........................................................cugucaugAauuugcucucu....................................    2      1    MOL
........................................................cugucauggauuuUcucucu....................................    1      1    MOL
........................................................cugucauggauuugcucucuu...................................    215    0    MOL
........................................................cugucauggauuugcucucuG...................................    1      1    MOL
........................................................cugucauggauuugcucuUuu...................................    1      1    MOL
........................................................cugucauggauuugUucucuu...................................    1      1    MOL
........................................................cugucauggauuugcucucuA...................................    1      1    MOL
........................................................cugucauggauCugcucucuu...................................    1      1    MOL
........................................................cugAcauggauuugcucucuu...................................    1      1    MOL
........................................................Gugucauggauuugcucucuu...................................    1      1    MOL
........................................................cugucauggaGuugcucucuu...................................    2      1    MOL
........................................................cugucaugAauuugcucucuu...................................    1      1    MOL
........................................................cugucauggauuugcucucuuA..................................    26     1    MOL
........................................................cugucauggauuugcuAucuuu..................................    1      1    MOL
........................................................cugGcauggauuugcucucuuu..................................    1      1    MOL
........................................................cugucauggauuugcuUucuuu..................................    1      1    MOL
........................................................cAgucauggauuugcucucuuu..................................    1      1    MOL
........................................................cugucauggauuuAcucucuuu..................................    2      1    MOL
........................................................cugucauggauuAgcucucuuu..................................    1      1    MOL
........................................................cugucauggauuugcucucuuC..................................    1      1    MOL
........................................................cugucauggauuugcucucuuu..................................    1403   0    MOL
........................................................Uugucauggauuugcucucuuu..................................    3      1    MOL
........................................................cugucauggCuuugcucucuuu..................................    1      1    MOL
........................................................cuCucauggauuugcucucuuu..................................    1      1    MOL
........................................................cugucauggaGuugcucucuuu..................................    3      1    MOL
........................................................cugucauggauuugcucuUuuu..................................    2      1    MOL
........................................................cugucaugAauuugcucucuuu..................................    2      1    MOL
........................................................cugucauggauuugcucucuuG..................................    1      1    MOL
........................................................cugucauggauuCgcucucuuu..................................    2      1    MOL
........................................................cugucauggGuuugcucucuuu..................................    1      1    MOL
........................................................cugucauggauuugcCcucuuu..................................    1      1    MOL
........................................................cugucauggauuugcucucCuu..................................    1      1    MOL
........................................................Gugucauggauuugcucucuuua.................................    1      1    MOL
........................................................cugucauggauuugcucucuuuU.................................    108    1    MOL
........................................................cugucauggauuugAucucuuua.................................    1      1    MOL
........................................................cugucauggauuugcucucuuGa.................................    1      1    MOL
........................................................cugucauggauuugcucucuuAa.................................    3      1    MOL
........................................................cugucauggCuuugcucucuuua.................................    1      1    MOL
........................................................cugucauggauuugcuAucuuua.................................    1      1    MOL
........................................................cugucauggGuuugcucucuuua.................................    5      1    MOL
........................................................cugucauggUuuugcucucuuua.................................    1      1    MOL
........................................................cugucauggauuuAcucucuuua.................................    5      1    MOL
........................................................cugucauggauuugUucucuuua.................................    1      1    MOL
........................................................cugucauggauuugcucucuuuC.................................    2      1    MOL
........................................................cugucauggauuugcucucuuua.................................    1886   0    MOL
........................................................cugucauggauuugcucuUuuua.................................    1      1    MOL
........................................................cugucauggauuugcucucuuuG.................................    10     1    MOL
........................................................cugucauggauuugcucucuuCa.................................    1      1    MOL
........................................................cugucaugUauuugcucucuuua.................................    1      1    MOL
........................................................cugucauggauuCgcucucuuua.................................    3      1    MOL
........................................................cugucauggauuugcucucuAua.................................    2      1    MOL
........................................................cugucauggauuAgcucucuuua.................................    1      1    MOL
........................................................Uugucauggauuugcucucuuua.................................    5      1    MOL
........................................................cugucauggauuugcucucuuuaA................................    37     1    MOL
........................................................cugucauggauuugcucuUuuuau................................    1      1    MOL
........................................................cugucauggaGuugcucucuuuau................................    1      1    MOL
........................................................cugucaugAauuugcucucuuuau................................    1      1    MOL
........................................................cugucauggauuugcuAucuuuau................................    1      1    MOL
........................................................cugucauggauuugcucucuuuCu................................    1      1    MOL
........................................................cugucauggauuuUcucucuuuau................................    1      1    MOL
........................................................Gugucauggauuugcucucuuuau................................    1      1    MOL
........................................................cugucauggauuugcucucuuuaC................................    27     1    MOL
........................................................cugucauggauuugcucucuuuUu................................    15     1    MOL
........................................................cugucauggauuugcucucuuuau................................    43     0    MOL
........................................................cugucauggauuugcucucuuuauu...............................    5      0    MOL
```

```
                    Mature                                    Star
uucaauggucaguagcuauaaagagagcuauccgugaacaguacuguuagaggagacugucauggauuugcucucuuuauuacacuggucaaaaucaucagguagcaacuac
........................................................cugucauggauuugcucucuuuauA...............................   3     1   MOL
........................................................cugucauggauuugcucucuuuUuu...............................   2     1   MOL
........................................................cugucauggauuugcucucuuuauuU..............................   1     1   MOL
.........................................................ugucauggauuugcucuc.....................................   4     0   MOL
.........................................................ugucauggauuugcucuUu....................................   1     1   MOL
.........................................................ugucauggauuugcucucu....................................   54    0   MOL
.........................................................ugucauggauuugcucucuu...................................   556   0   MOL
.........................................................ugucauggauuCgcucucuu...................................   1     1   MOL
.........................................................uguUauggauuugcucucuu...................................   1     1   MOL
.........................................................Ggucauggauuugcucucuu...................................   1     1   MOL
.........................................................ugucauggUuuugcucucuu...................................   2     1   MOL
.........................................................ugucauggauuugcucucuC...................................   1     1   MOL
.........................................................ugucauggauuugcucucuA...................................   1     1   MOL
.........................................................ugucauggaGuugcucucuu...................................   1     1   MOL
.........................................................ugucauggauAugcucucuu...................................   1     1   MOL
.........................................................ugucauggauuugcucuAuu...................................   1     1   MOL
.........................................................ugucauggGuuugcucucuu...................................   1     1   MOL
.........................................................ugucauggauuuAcucucuu...................................   2     1   MOL
.........................................................Agucauggauuugcucucuu...................................   1     1   MOL
.........................................................ugucauAgauuugcucucuuu..................................   1     1   MOL
.........................................................ugCcauggauuugcucucuuu..................................   1     1   MOL
.........................................................ugucauggaGuugcucucuuu..................................   3     1   MOL
.........................................................ugucauggCuuugcucucuuu..................................   1     1   MOL
.........................................................ugucauggauuugcCcucuuu..................................   1     1   MOL
.........................................................ugucauggauuugcucCcuuu..................................   1     1   MOL
.........................................................ugucauggauuugcucucuuC..................................   1     1   MOL
.........................................................ugucauggauuugcAcucuuu..................................   1     1   MOL
.........................................................ugucauggUuuugcucucuuu..................................   14    1   MOL
.........................................................ugucaAggauuugcucucuuu..................................   1     1   MOL
.........................................................ugucauggauuugcucucuuA..................................   32    1   MOL
.........................................................ugucauggauCugcucucuuu..................................   2     1   MOL
.........................................................ugucaugAauuugcucucuuu..................................   3     1   MOL
.........................................................uguUauggauuugcucucuuu..................................   1     1   MOL
.........................................................Ggucauggauuugcucucuuu..................................   4     1   MOL
.........................................................ugucaugUauuugcucucuuu..................................   1     1   MOL
.........................................................ugucauggGuuugcucucuuu..................................   1     1   MOL
.........................................................uCucauggauuugcucucuuu..................................   6     1   MOL
.........................................................ugucauggauuugcucuUuuu..................................   10    1   MOL
.........................................................ugucaugCauuugcucucuuu..................................   1     1   MOL
.........................................................ugucauggauuugcucucAuu..................................   1     1   MOL
.........................................................ugucauggauuugAucucuuu..................................   1     1   MOL
.........................................................ugucauggauuugcucucuuu..................................   5807  0   MOL
.........................................................Cgucauggauuugcucucuuu..................................   2     1   MOL
.........................................................ugucauggauuugcuUucuuu..................................   2     1   MOL
.........................................................Agucauggauuugcucucuuu..................................   23    1   MOL
.........................................................ugucauggauuugcucAcuuu..................................   1     1   MOL
.........................................................ugucauggauuuAcucucuuu..................................   9     1   MOL
.........................................................uUucauggauuugcucucuuu..................................   2     1   MOL
.........................................................ugGcauggauuugcucucuuu..................................   1     1   MOL
.........................................................ugucauggauuugcucucCuu..................................   1     1   MOL
.........................................................ugucUuggauuugcucucuuu..................................   1     1   MOL
.........................................................ugucauggauuugUucucuuu..................................   1     1   MOL
.........................................................ugucauggauuuUcucucuuu..................................   1     1   MOL
.........................................................ugucauggaCuugcucucuuu..................................   1     1   MOL
.........................................................ugucauggauuugcuGucuuu..................................   1     1   MOL
.........................................................ugucauggGuuugcucucuuua.................................   6     1   MOL
.........................................................ugucaCggauuugcucucuuua.................................   1     1   MOL
.........................................................ugucauggauuugcucCcuuua.................................   1     1   MOL
.........................................................ugucauggauuugcuUucuuua.................................   9     1   MOL
.........................................................ugucauggauuugcucucuuuU.................................   398   1   MOL
.........................................................ugucauggauuugUucucuuua.................................   6     1   MOL
.........................................................ugucauggauuugcucucuAua.................................   1     1   MOL
.........................................................ugucauggCuuugcucucuuua.................................   2     1   MOL
.........................................................ugucauggauCugcucucuuua.................................   1     1   MOL
.........................................................Ggucauggauuugcucucuuua.................................   6     1   MOL
.........................................................ugucauggauuuCcucucuuua.................................   1     1   MOL
.........................................................ugucaAggauuugcucucuuua.................................   3     1   MOL
.........................................................Agucauggauuugcucucuuua.................................   35    1   MOL
.........................................................ugucauggauuuAcucucuuua.................................   15    1   MOL
.........................................................ugCcauggauuugcucucuuua.................................   1     1   MOL
```

```
                    Mature                                    Star
uucaauggucaguagcuauaaagagagcuauccgugaacaguacuguuagaggagacugucauggauuugcucucuuuauuacacuggucaaaaucaucagguagcaacuac
.........................................................ugucauggauuugAucucuuua.................................   2      1      MOL
.........................................................ugucauggauuGgcucucuuua.................................   1      1      MOL
.........................................................ugucauggauuugcucucuuGa.................................   1      1      MOL
.........................................................ugAcauggauuugcucucuuua.................................   3      1      MOL
.........................................................uguUauggauuugcucucuuua.................................   3      1      MOL
.........................................................ugucUuggauuugcucucuuua.................................   2      1      MOL
.........................................................ugucauCgauuugcucucuuua.................................   1      1      MOL
.........................................................ugucauggauuugcucucGuua.................................   1      1      MOL
.........................................................ugucauggauuugcucAcuuua.................................   3      1      MOL
.........................................................ugucauggaCuugcucucuuua.................................   4      1      MOL
.........................................................ugucauggauuugcucuAuuua.................................   1      1      MOL
.........................................................ugucaugUauuugcucucuuua.................................   1      1      MOL
.........................................................ugucauggauuugcucucuuuG.................................   4      1      MOL
.........................................................ugucauggauuugcuAucuuua.................................   3      1      MOL
.........................................................ugucauggauuugcucucCuua.................................   2      1      MOL
.........................................................ugucauggauuugcucucuuCa.................................   2      1      MOL
.........................................................ugucauggauuugcucucuuAa.................................   4      1      MOL
.........................................................ugucaugAauuugcucucuuua.................................   1      1      MOL
.........................................................ugucauggauuugcGcucuuua.................................   1      1      MOL
.........................................................ugucauggauuugcucucuCua.................................   1      1      MOL
.........................................................uCucauggauuugcucucuuua.................................   1      1      MOL
.........................................................ugucauggauuugcucucuuua.................................   8248   0      MOL
.........................................................ugucauggaGuugcucucuuua.................................   6      1      MOL
.........................................................ugucauggUuuugcucucuuua.................................   25     1      MOL
.........................................................Cgucauggauuugcucucuuua.................................   5      1      MOL
.........................................................ugucaGggauuugcucucuuua.................................   2      1      MOL
.........................................................ugucauggauuugcucucuuuC.................................   4      1      MOL
.........................................................uguGauggauuugcucucuuua.................................   3      1      MOL
.........................................................ugucaugCauuugcucucuuua.................................   1      1      MOL
.........................................................ugucauggauuuUcucucuuua.................................   1      1      MOL
.........................................................ugucauggauuugcucuUuuua.................................   6      1      MOL
.........................................................uAucauggauuugcucucuuua.................................   1      1      MOL
.........................................................Cgucauggauuugcucucuuuau................................   1      1      MOL
.........................................................ugucauggauuugcucucuuuCu................................   2      1      MOL
.........................................................ugucauggauuugcucucuuuau................................   166    0      MOL
.........................................................ugucauggauuugcucucuuuaA................................   115    1      MOL
.........................................................ugucauggauuugcucucuuuUu................................   27     1      MOL
.........................................................ugucauggauuugcucucuuuaG................................   5      1      MOL
.........................................................Ggucauggauuugcucucuuuau................................   1      1      MOL
.........................................................ugucauggaGuugcucucuuuau................................   2      1      MOL
.........................................................ugucauggauuugcucucuuuaC................................   69     1      MOL
.........................................................ugucauggauuugcucucuuuaAu...............................   2      1      MOL
.........................................................ugucauggauuugcucucuuuauA...............................   8      1      MOL
.........................................................ugucauggauuugcucucuuuauu...............................   9      0      MOL
.........................................................ugucauggauuugcucucuuuaCu...............................   1      1      MOL
.........................................................ugucauggauuugcucucuuuUuu...............................   1      1      MOL
.........................................................ugucauggauuugcucucuuuaGu...............................   1      1      MOL
.........................................................ugucauggauuugcucucuuuauAa..............................   1      1      MOL
.........................................................ugucauggauuugcucucuuuaAua..............................   2      1      MOL
.........................................................ugucauggauuugcucucuuuauuU..............................   1      1      MOL
..........................................................gucauggauuugcucucuu...................................   2      0      MOL
..........................................................gucauggauuugcucucuuu..................................   14     0      MOL
..........................................................gucauggauuugcucucuuuU.................................   2      1      MOL
..........................................................gucauggauuugcucucuuua.................................   19     0      MOL
...........................................................ucauggauuugcucucuuu..................................   1      0      MOL
...........................................................ucauggauuugcucucuuua.................................   1      0      MOL
...............................................................................uuacacuggucaaaaucauc.............   4      0      MOL

..................uUaagagagcuauccgugaac.........................................................................   1      1      T6P
..................uaaagagagcuauccgugaac.........................................................................   2      0      T6P
..................uaaagagagcuauccgugaaca........................................................................   1      0      T6P
..................uaaagagagcuauccgugaacag.......................................................................   1      0      T6P
..................uUaagagagcuauccgugaacagu......................................................................   3      1      T6P
...................aaagagagcuauccgugaa..........................................................................   4      0      T6P
...................aaagagagcuauccgugaaA.........................................................................   1      1      T6P
...................aaagagaCcuauccgugaac.........................................................................   1      1      T6P
...................aaagagagcuauccgugaac.........................................................................   24     0      T6P
...................aaagagagcuauccgugaaca........................................................................   21     0      T6P
...................aaagagagcuauccgugaacU........................................................................   1      1      T6P
...................aaagagagcuauccgugaacag.......................................................................   64     0      T6P
```

```
                    Mature                Star
uucaauggucaguagcuauaaagagagcuauccgugaacaguacuguuagaggagacugucauggauuugcucucuuuauuacacuggucaaaaucaucagguagcaacuac
...................aaagagagcuauccgugaacagu......................................................................   400    0   T6P
...................aaagCgagcuauccgugaacagu......................................................................   1      1   T6P
...................aaaUagagcuauccgugaacagu......................................................................   1      1   T6P
...................Uaagagagcuauccgugaacagu......................................................................   7      1   T6P
...................aaagagagcuauUcgugaacagu......................................................................   1      1   T6P
...................aGagagagcuauccgugaacagu......................................................................   1      1   T6P
...................aaagagUgcuauccgugaacagu......................................................................   1      1   T6P
...................aaagagagUuauccgugaacagu......................................................................   2      1   T6P
...................aaagagagcuauccgugUacagu......................................................................   1      1   T6P
...................aaagagagcuauccgugaacagA......................................................................   3      1   T6P
...................Uaagagagcuauccgugaacagua.....................................................................   1      1   T6P
...................aaagagagcuauccgugaacaguU.....................................................................   29     1   T6P
...................aaagagagcuauccgugaacagua.....................................................................   17     0   T6P
...................aaagagagcuauccgugaacaguaU....................................................................   53     1   T6P
...................aaagagagcuauccgugaacaguaUu...................................................................   6      1   T6P
...................aaagagagcuauccgugaacaguaAu...................................................................   3      1   T6P
....................aagagagcuauccgugaC..........................................................................   1      1   T6P
....................aagagagcuauccgugGa..........................................................................   1      1   T6P
....................aagagagcuauccgugaa..........................................................................   8      0   T6P
....................aagagagcuauccgugGac.........................................................................   1      1   T6P
....................aaCagagcuauccgugaac.........................................................................   1      1   T6P
....................aagUgagcuauccgugaac.........................................................................   2      1   T6P
....................aagagaCcuauccgugaac.........................................................................   2      1   T6P
....................aagagaAcuauccgugaac.........................................................................   2      1   T6P
....................aagagagcuauccgugaac.........................................................................   1122   0   T6P
....................aagagagcuauccCugaac.........................................................................   2      1   T6P
....................aagagagcCauccgugaac.........................................................................   1      1   T6P
....................aagagagcuauccgugaaA.........................................................................   9      1   T6P
....................aagagagUuauccgugaac.........................................................................   1      1   T6P
....................aagagagcuauccgugaaca........................................................................   772    0   T6P
....................aaUagagcuauccgugaaca........................................................................   1      1   T6P
....................aagCgagcuauccgugaaca........................................................................   1      1   T6P
....................aaCagagcuauccgugaaca........................................................................   1      1   T6P
....................aagagagcuauccgugaacU........................................................................   10     1   T6P
....................aagagaCcuauccgugaaca........................................................................   1      1   T6P
....................aagagagcuauccAugaaca........................................................................   1      1   T6P
....................aagagagcuauccCugaaca........................................................................   1      1   T6P
....................aagagagcuauccguCaacag.......................................................................   2      1   T6P
....................aagagagcuauccgugaUcag.......................................................................   1      1   T6P
....................aagagagcuaCccgugaacag.......................................................................   1      1   T6P
....................aagagagcuauccgugUacag.......................................................................   2      1   T6P
....................aagagagcuauccgugaacaC.......................................................................   1      1   T6P
....................aagUgagcuauccgugaacag.......................................................................   7      1   T6P
....................aagagagcuauccgugaaUag.......................................................................   3      1   T6P
....................aagagagcuauccguUaacag.......................................................................   3      1   T6P
....................aagagagcuauccCugaacag.......................................................................   2      1   T6P
....................aagagagUuauccgugaacag.......................................................................   8      1   T6P
....................aagagagcuauAcgugaacag.......................................................................   1      1   T6P
....................aagagagcuauccgugaacaU.......................................................................   12     1   T6P
....................aagaUagcuauccgugaacag.......................................................................   2      1   T6P
....................aaCagagcuauccgugaacag.......................................................................   1      1   T6P
....................aagagagcuauccgugaacaA.......................................................................   8      1   T6P
....................aagagagcuauccgugaCcag.......................................................................   1      1   T6P
....................aagagagcuauccgugaacGg.......................................................................   1      1   T6P
....................aagagagcCauccgugaacag.......................................................................   1      1   T6P
....................aagagagcuauccgCgaacag.......................................................................   1      1   T6P
....................aagagagcuauccgugaacag.......................................................................   2904   0   T6P
....................aagagUgcuauccgugaacag.......................................................................   1      1   T6P
....................Uagagagcuauccgugaacag.......................................................................   2      1   T6P
....................aagagGgcuauccgugaacag.......................................................................   2      1   T6P
....................aagagaCcuauccgugaacag.......................................................................   1      1   T6P
....................aagagaAcuauccgugaacag.......................................................................   2      1   T6P
....................aagagagcuauccgugGacag.......................................................................   1      1   T6P
....................aagagagcuauUcgugaacag.......................................................................   2      1   T6P
....................aagagagcuaucUgugaacag.......................................................................   1      1   T6P
....................aagagagcuauccgugaacGgu......................................................................   1      1   T6P
....................aagagagcuauccUugaacagu......................................................................   23     1   T6P
....................aagagagcuauccgugaacagA......................................................................   181    1   T6P
....................aagagagcuauccgugaUcagu......................................................................   5      1   T6P
....................aagagagcuaGccgugaacagu......................................................................   1      1   T6P
```

Mature **Star**

uucaauggucaguagcuauaaagagagcuauccgugaacaguacuguuagaggagacugucauggauuugcucucuuuauuacacuggucaaaaucaucagguagcaacuac			
....................aagagagcuauccgugaCcagu..	2	1	T6P
....................aagagagcuauccgugaacaCu..	22	1	T6P
....................aagagGgcuauccgugaacagu..	15	1	T6P
....................aagagagcCauccgugaacagu..	8	1	T6P
....................aagagagcuauccAugaacagu..	26	1	T6P
....................aagagagcuauccgugCacagu..	5	1	T6P
....................aagagagcGauccgugaacagu..	2	1	T6P
....................aagagUgcuauccgugaacagu..	20	1	T6P
....................aagagagcuauUcgugaacagu..	45	1	T6P
....................aagagaAcuauccgugaacagu..	20	1	T6P
....................aagagagcuauccgAgaacagu..	9	1	T6P
....................aagagaUcuauccgugaacagu..	24	1	T6P
....................aagagCgcuauccgugaacagu..	9	1	T6P
....................aaUagagcuauccgugaacagu..	26	1	T6P
....................aagagaCcuauccgugaacagu..	38	1	T6P
....................aagagagGuauccgugaacagu..	1	1	T6P
....................aagagagcuauccgugaacagG..	8	1	T6P
....................aagagagcuGuccgugaacagu..	6	1	T6P
....................aagagagUuauccgugaacagu..	63	1	T6P
....................aagagagcuauccguAaacagu..	11	1	T6P
....................aagagagcuauccgugUacagu..	24	1	T6P
....................aagagagcuauccgGgaacagu..	7	1	T6P
....................aagagagcuaucUgugaacagu..	12	1	T6P
....................Gagagagcuauccgugaacagu..	1	1	T6P
....................aagagagcuauccgugaacagC..	47	1	T6P
....................aUgagagcuauccgugaacagu..	3	1	T6P
....................aagagagcuauccguUaacagu..	42	1	T6P
....................aagUgagcuauccgugaacagu..	79	1	T6P
....................aagagagcuauAcgugaacagu..	2	1	T6P
....................aagagagcuauccgCgaacagu..	19	1	T6P
....................aagagagcuauccgugaacCgu..	1	1	T6P
....................aagCgagcuauccgugaacagu..	5	1	T6P
....................aagagagcuauccgugaGcagu..	3	1	T6P
....................aagaCagcuauccgugaacagu..	7	1	T6P
....................aagaUagcuauccgugaacagu..	12	1	T6P
....................aagagagcuauccgugaacagu..	58046	0	T6P
....................aaCagagcuauccgugaacagu..	18	1	T6P
....................aaAagagcuauccgugaacagu..	6	1	T6P
....................aagagagcuUuccgugaacagu..	5	1	T6P
....................aagagagcuauccgugaacaUu..	14	1	T6P
....................Uagagagcuauccgugaacagu..	10	1	T6P
....................Cagagagcuauccgugaacagu..	1	1	T6P
....................aagagagcuauccgugaaUagu..	7	1	T6P
....................aagGgagcuauccgugaacagu..	4	1	T6P
....................aagagagcuauccgugGacagu..	14	1	T6P
....................aagagagcuaCccgugaacagu..	7	1	T6P
....................aagagagcuauccCugaacagu..	10	1	T6P
....................aagagagcuauccguCaacagu..	28	1	T6P
....................aagagagcuauccgugaacaAu..	12	1	T6P
....................aGgagagcuauccgugaacagu..	7	1	T6P
....................aUgagagcuauccgugaacagua...	1	1	T6P
....................aagagaCcuauccgugaacagua...	6	1	T6P
....................aagaUagcuauccgugaacagua...	5	1	T6P
....................aaCagagcuauccgugaacagua...	5	1	T6P
....................aagagagcuauccguAaacagua...	3	1	T6P
....................aagagagcuauccgugaacagua...	7247	0	T6P
....................aagagagcuauccCugaacagua...	3	1	T6P
....................aagagagcuauccgugaacagAa...	15	1	T6P
....................aagagagcuUuccgugaacagua...	1	1	T6P
....................aagagagcuauccgGgaacagua...	2	1	T6P
....................aaUagagcuauccgugaacagua...	2	1	T6P
....................aagagagcuauccgCgaacagua...	7	1	T6P
....................aagagagcuauccAugaacagua...	4	1	T6P
....................aagagagcuauccgugUacagua...	4	1	T6P
....................aagagagcuCuccgugaacagua...	1	1	T6P
....................aagUgagcuauccgugaacagua...	5	1	T6P
....................aagagagcuaCccgugaacagua...	2	1	T6P
....................aagagagcuauccgugaacaUua...	1	1	T6P
....................aagagagcuauccgugaacaCua...	2	1	T6P
....................aagagagcuauccgugaaUagua...	4	1	T6P

Mature **Star**

uucaauggucaguagcuauaaagagagcuauccgugaacaguacuguuagaggagacugucauggauuugcucucuuuauuacacuggucaaaaucaucagguagcaacuac			
....................aagagagcuauccgugaCcagua...	1	1	T6P
....................aagagaUcuauccgugaacagua...	3	1	T6P
....................aagagagcuauccUugaacagua...	5	1	T6P
....................Gagagagcuauccgugaacagua...	2	1	T6P
....................aagagagcuauccgugGacagua...	2	1	T6P
....................aagagagcuauUcgugaacagua...	3	1	T6P
....................aagagagUuauccgugaacagua...	8	1	T6P
....................aagagagcuauccgugaGcagua...	1	1	T6P
....................aagagaAcuauccgugaacagua...	1	1	T6P
....................aagagagcuaucUgugaacagua...	2	1	T6P
....................aagagagcuauccgugaacaguG...	21	1	T6P
....................aagagagcuaucAgugaacagua...	1	1	T6P
....................aagagagcuauccgugaacaAua...	2	1	T6P
....................aagagagcuauAcgugaacagua...	1	1	T6P
....................aagagagcuauccgugaacaguU...	3766	1	T6P
....................aagagagcCauccgugaacagua...	2	1	T6P
....................Uagagagcuauccgugaacagua...	2	1	T6P
....................aagagGgcuauccgugaacagua...	1	1	T6P
....................aCgagagcuauccgugaacagua...	1	1	T6P
....................aagagagcuauccgugaacaguC...	8	1	T6P
....................aagagagcuauccguUaacagua...	10	1	T6P
....................aagagagcuauccguCaacagua...	11	1	T6P
....................aagagagcuauccgugaacaguaU..	7101	1	T6P
....................aagagagcuauccgugaacaguac..	33	0	T6P
....................aagagagcuauccgugaacaguaA..	346	1	T6P
....................aagagagcuauccgugaacaguaG..	14	1	T6P
....................aagagagcuauccgugaacaguaGu...	4	1	T6P
....................aagagagcuauccgugaacaguaUu...	342	1	T6P
....................aagagagcuauccgugaacaguacA...	2	1	T6P
....................aagagagcuauccgugaacaguaAu...	179	1	T6P
....................aagagagcuauccgugaacaguacu...	3	0	T6P
....................aagagagcuauccgugaacaguacugu...	3	0	T6P
....................aagagagcuauccgugaacaguaUugu...	1	1	T6P
.....................aCagagcuauccgugaac...	1	1	T6P
.....................agagagcuauccgugaacagu..	28	0	T6P
.....................agagGgcuauccgugaacagu..	1	1	T6P
.....................aCagagcuauccgugaacagu..	2	1	T6P
.....................aAagagcuauccgugaacagu..	1	1	T6P
.....................aUagagcuauccgugaacagu..	1	1	T6P
.....................agagagcuauccUugaacagua...	1	1	T6P
.....................agagagcuauccgugaacaguU...	1	1	T6P
.....................agagagcuauccgugaacagua...	10	0	T6P
.....................agagagcuauccgugaacaguaU..	9	1	T6P
......................Aagagcuauccgugaacag...	1	1	T6P
......................gagagcuauccAugaacagu..	1	1	T6P
......................gagagcuauccgugaacagu..	3	0	T6P
......................Aagagcuauccgugaacagu..	8	1	T6P
......................gagagcuauccgugaacaguU...	3	1	T6P
......................Aagagcuauccgugaacagua...	1	1	T6P
......................gagagcuauccgugaacagua...	1	0	T6P
.......................agagcuauccgugaacagA..	1	1	T6P
.......................agagcuauccgugaacagu..	9	0	T6P
.......................agagcuauccgugaacagua...	3	0	T6P
.......................agagcuauccgugaacaguaU..	1	1	T6P
........................gagcuauccgugaacagu..	6	0	T6P
........................gagcuauccgugaacaguU...	1	1	T6P
........................gagcuauccgugaacaguaA..	2	1	T6P
..........................gcuauccgugaacaguaU..	1	1	T6P
....acuguuagaggagacugucauggauu..	1	0	T6P
....gagacugucauggauuugcuc.......................................	1	0	T6P
....agaGugucauggauuugcucucuuu..................................	1	1	T6P
....agCcugucauggauuugcucucuuua.................................	1	1	T6P
....gaGugucauggauuugcucucuuua.................................	1	1	T6P
....acugucauggauuugcucucuu...................................	1	0	T6P
....acugucauggauuugcucucuuu..................................	3	0	T6P
....acugucauggauuugcucucuuua.................................	2	0	T6P
....acugucauggauuugcucucuuuU.................................	2	1	T6P
....Ucugucauggauuugcucucuuua.................................	1	1	T6P
....acugucauggauuugcucucuuuC.................................	1	1	T6P
....cugucauggauuugcucuUu....................................	1	1	T6P

Mature **Star**

uucaauggucaguagcuauaaagagagcuauccgugaacaguacuguuagaggagacugucauggauuugcucucuuuauuacacuggucaaaaucaucagguagcaacuac			
....cugucauggauuugcucucu....................................	9	0	T6P
....cugucauggauuugcucucuu...................................	56	0	T6P
....cugucauggauCugcucucuu...................................	1	1	T6P
....cugucauggauuugcucuUuuu..................................	2	1	T6P
....cugucaGggauuugcucucuuu..................................	2	1	T6P
....cugucauggauuugcucucuuA..................................	1	1	T6P
....cugucauggauuugcucucCuu..................................	1	1	T6P
....cugucauggaCuugcucucuuu..................................	1	1	T6P
....cugucauggauuugcucucuuu..................................	911	0	T6P
....Uugucauggauuugcucucuuu..................................	4	1	T6P
....cugucauggauuugcuUucuuu..................................	3	1	T6P
....cuUucauggauuugcucucuuu..................................	1	1	T6P
....cugucauggauuugcucucuuua.................................	1842	0	T6P
....cugucaCggauuugcucucuuua.................................	1	1	T6P
....cugucauggUuuugcucucuuua.................................	1	1	T6P
....cugucauggauuuAcucucuuua.................................	3	1	T6P
....Uugucauggauuugcucucuuua.................................	3	1	T6P
....cugucauggauuugUucucuuua.................................	2	1	T6P
....cugucauggauuugcucuUuuua.................................	3	1	T6P
....cugucauggauuugcucucCuua.................................	2	1	T6P
....cugCcauggauuugcucucuuua.................................	2	1	T6P
....cugucauggauuugcucucuuuU.................................	122	1	T6P
....cugAcauggauuugcucucuuua.................................	1	1	T6P
....cGgucauggauuugcucucuuua.................................	1	1	T6P
....cugucauggauuugcucucuuuG.................................	2	1	T6P
....cuUucauggauuugcucucuuua.................................	2	1	T6P
....cugucaGggauuugcucucuuua.................................	1	1	T6P
....cugucauggauuUcucucuuua.................................	1	1	T6P
....cugucauggauuugcucucuuAa.................................	1	1	T6P
....cugucauggauuugcucucuuuUu................................	12	1	T6P
....cugucauggauuugcucucuuuaA................................	23	1	T6P
....cugucauggauuugcucucuuuaC................................	16	1	T6P
....cugucauggauuugcucucuuuau................................	73	0	T6P
....cugucauggauuugcucucuuuauu...............................	12	0	T6P
....cugucauggauuugcucucuuuUuu...............................	1	1	T6P
....ugucauggauuugcucuc.....................................	1	0	T6P
....ugucauggauuugcucucu....................................	22	0	T6P
....ugucauggauuugcucuUu....................................	4	1	T6P
....ugucaugUauuugcucucuu...................................	1	1	T6P
....ugucauggauuugcucuUuu...................................	2	1	T6P
....ugucauggauuugcuGucuu...................................	1	1	T6P
....ugucauggauuugcucGcuu...................................	1	1	T6P
....ugucauggauuugcucucuu...................................	268	0	T6P
....ugucauAgauuugcucucuu...................................	1	1	T6P
....ugucauggauuugcucucuA...................................	1	1	T6P
....ugucaugUauuugcucucuuu..................................	3	1	T6P
....ugucauggauuCgcucucuuu..................................	3	1	T6P
....ugucauggauCugcucucuuu..................................	1	1	T6P
....ugucauggauuugcucucuuA..................................	12	1	T6P
....uguUauggauuugcucucuuu..................................	7	1	T6P
....ugCcauggauuugcucucuuu..................................	1	1	T6P
....uCucauggauuugcucucuuu..................................	4	1	T6P
....uguGauggauuugcucucuuu..................................	1	1	T6P
....ugGcauggauuugcucucuuu..................................	2	1	T6P
....ugucUuggauuugcucucuuu..................................	1	1	T6P
....ugucauggaAuugcucucuuu..................................	2	1	T6P
....ugucauggauuugcuUucuuu..................................	3	1	T6P
....ugucauUgauuugcucucuuu..................................	1	1	T6P
....ugucauggaCuugcucucuuu..................................	1	1	T6P
....ugucauggCuuugcucucuuu..................................	1	1	T6P
....ugucauggUuuugcucucuuu..................................	1	1	T6P
....ugucauggauuuAcucucuuu..................................	4	1	T6P
....ugucauggauuugcucucuuu..................................	6002	0	T6P
....ugucauggauuugcucucuAu..................................	1	1	T6P
....uUucauggauuugcucucuuu..................................	6	1	T6P
....ugucauggauuugUucucuuu..................................	1	1	T6P
....ugucauggauuuCcucucuuu..................................	2	1	T6P
....Ggucauggauuugcucucuuu..................................	2	1	T6P
....ugucauggauuugGucucuuu..................................	1	1	T6P
....ugucaugCauuugcucucuuu..................................	2	1	T6P

Mature **Star**

uucaauggucaguagcuauaaagagagcuauccgugaacaguacuguuagaggagacugucauggauuugcucucuuuauuacacuggucaaaaucaucagguagcaacuac			
....Agucauggauuugcucucuuu..................................	11	1	T6P
....Cgucauggauuugcucucuuu..................................	1	1	T6P
....ugucauggGuuugcucucuuu..................................	1	1	T6P
....ugucaugAauuugcucucuuu..................................	2	1	T6P
....ugucauggauuugcucuUuuu..................................	9	1	T6P
....ugucauAgauuugcucucuuu..................................	1	1	T6P
....ugucauggaGuugcucucuuu..................................	2	1	T6P
....ugAcauggauuugcucucuuu..................................	2	1	T6P
....ugucauggauuugcucucuuuG.................................	3	1	T6P
....uCucauggauuugcucucuuua.................................	1	1	T6P
....ugucauggauuugGucucuuua.................................	1	1	T6P
....ugucauggauuugcucucAuua.................................	1	1	T6P
....ugucauggauuugUucucuuua.................................	9	1	T6P
....ugucauggauuugcucucuuCa.................................	1	1	T6P
....ugucUuggauuugcucucuuua.................................	2	1	T6P
....ugGcauggauuugcucucuuua.................................	1	1	T6P
....ugucauggCuuugcucucuuua.................................	1	1	T6P
....ugucauggauuugcucGcuuua.................................	1	1	T6P
....ugucauggauuugcucCcuuua.................................	2	1	T6P
....ugucauggauuugcucucuuuU.................................	738	1	T6P
....ugucauggaAuugcucucuuua.................................	2	1	T6P
....ugucaugUauuugcucucuuua.................................	4	1	T6P
....ugucauggauuUcucucuuua.................................	9	1	T6P
....ugucauggauuugcucucuuAa.................................	3	1	T6P
....ugucauggauuugcucucuuuC.................................	11	1	T6P
....ugucauggauuugcuGucuuua.................................	1	1	T6P
....ugucaCggauuugcucucuuua.................................	1	1	T6P
....ugucauggauuugcucucCuua.................................	1	1	T6P
....uUucauggauuugcucucuuua.................................	3	1	T6P
....ugucauggauuugcucucuCua.................................	1	1	T6P
....uguGauggauuugcucucuuua.................................	1	1	T6P
....ugucauggUuuugcucucuuua.................................	5	1	T6P
....ugucauggauuugcuAucuuua.................................	2	1	T6P
....ugucauggaGuugcucucuuua.................................	1	1	T6P
....uguUauggauuugcucucuuua.................................	16	1	T6P
....ugucauggauuugcuUucuuua.................................	13	1	T6P
....ugucauggauuugcCcucuuua.................................	1	1	T6P
....Agucauggauuugcucucuuua.................................	9	1	T6P
....ugucauCgauuugcucucuuua.................................	8	1	T6P
....ugucGuggauuugcucucuuua.................................	2	1	T6P
....Ggucauggauuugcucucuuua.................................	6	1	T6P
....ugucauggauuugcucucuuGa.................................	1	1	T6P
....ugucauggauuuAcucucuuua.................................	10	1	T6P
....ugucauggauuugcucuUuuua.................................	18	1	T6P
....ugucauggauuugcucucuuua.................................	11297	0	T6P
....Cgucauggauuugcucucuuua.................................	4	1	T6P
....ugCcauggauuugcucucuuua.................................	3	1	T6P
....ugucauggauuuCcucucuuua.................................	4	1	T6P
....ugucaugAauuugcucucuuua.................................	3	1	T6P
....ugucauggaCuugcucucuuua.................................	1	1	T6P
....ugucaugCauuugcucucuuua.................................	5	1	T6P
....ugucauggauuCgcucucuuua.................................	4	1	T6P
....ugucauggGuuugcucucuuua.................................	3	1	T6P
....uguAauggauuugcucucuuua.................................	1	1	T6P
....ugucauAgauuugcucucuuua.................................	2	1	T6P
....ugucaugAauuugcucucuuuau................................	1	1	T6P
....ugucauggauuugcucucuuuaC................................	59	1	T6P
....ugucauggUuuugcucucuuuau................................	1	1	T6P
....ugucauggauuugcucuUuuuau................................	2	1	T6P
....Agucauggauuugcucucuuuau................................	1	1	T6P
....ugucauggauuugcucucuuuaA................................	52	1	T6P
....ugucauggauuugcucucuuuCu................................	1	1	T6P
....ugucauggauuugcucucuuuau................................	289	0	T6P
....ugucauggauuugcucucuuuUu................................	54	1	T6P
....ugucauggauuugcucucuuuaAu...............................	2	1	T6P
....ugucauggauuugcucucuuuauC...............................	1	1	T6P
....ugucauggauuugcucucuuuaCu...............................	2	1	T6P
....ugucauggauuugcucucuuuUuu...............................	1	1	T6P
....ugucauggauuugcucucuuuauu...............................	16	0	T6P
....ugucaugAauuugcucucuuuauu...............................	1	1	T6P

```
                    Mature                                   Star
uucaauggucaguagcuauaaagagagcuauccgugaacaguacuguuagaggagacugucauggauuugcucucuuuauuacacuggucaaaaucaucagguagcaacuac
.........................................................ugucauggauuugcucucuuuauA...............................   7    1    T6P
.........................................................ugucauggauuugcucucuuuauuU..............................   3    1    T6P
.........................................................ugucauggauuugcucucuuuauuC..............................   1    1    T6P
..........................................................gucauggauuugcucucuuu..................................   19   0    T6P
..........................................................Uucauggauuugcucucuuu..................................   3    1    T6P
..........................................................Cucauggauuugcucucuuu..................................   4    1    T6P
..........................................................Cucauggauuugcucucuuua.................................   1    1    T6P
..........................................................gucauggauuugcucucuuuU.................................   4    1    T6P
..........................................................gucaugCauuugcucucuuua.................................   2    1    T6P
..........................................................Uucauggauuugcucucuuua.................................   2    1    T6P
..........................................................gucauggauuugcucucuuua.................................   26   0    T6P
..........................................................gucauggauuugcucucuuuaC................................   1    1    T6P
..........................................................gucauggauuugcucucuuuau................................   1    0    T6P
..........................................................gucauggauuugcucucuuuauu...............................   1    0    T6P
...........................................................ucauggauuugcucucuuua.................................   2    0    T6P
...........................................................uUauggauuugcucucuuua.................................   1    1    T6P
............................................................cauggauuugcucucuuua.................................   1    0    T6P
............................................................cauggauuugcucuUuuua.................................   1    1    T6P
.............................................................auggauuugcucucuuua.................................   2    0    T6P
...............................................................................uuacacuggucaaaaucauc.............   2    0    T6P

..................uaaagagagcuauccgugaaca........................................................................   1    0    egg
...................aaagagagcuauccgugaacag.......................................................................   1    0    egg
...................aaagagagcuauccguAaacagu......................................................................   1    1    egg
...................aaagagagcuauccgugaacagu......................................................................   6    0    egg
....................Uagagagcuauccgugaac.........................................................................   1    1    egg
....................aagagagcuauccgugaaU.........................................................................   1    1    egg
....................aagagagcuaCccgugaac.........................................................................   1    1    egg
....................aagagagcuauccgugaac.........................................................................   10   0    egg
....................aagagCgcuauccgugaac.........................................................................   1    1    egg
....................aagagagcuauccgugaaca........................................................................   4    0    egg
....................aGgagagcuauccgugaacag.......................................................................   1    1    egg
....................aagagagcuauccgugaacaA.......................................................................   1    1    egg
....................aagagagcuauccgugaacag.......................................................................   17   0    egg
....................aagaAagcuauccgugaacag.......................................................................   1    1    egg
....................aagagagcuauccgugaacagu......................................................................   263  0    egg
....................aagagCgcuauccgugaacagu......................................................................   2    1    egg
....................aagaAagcuauccgugaacagu......................................................................   1    1    egg
....................aagagagcuauccgugaacagC......................................................................   18   1    egg
....................aGgagagcuauccgugaacagu......................................................................   1    1    egg
....................aagagUgcuauccgugaacagu......................................................................   1    1    egg
....................aagUgagcuauccgugaacagu......................................................................   2    1    egg
....................aagagagcuauccgugaacagG......................................................................   1    1    egg
....................aagagagcuaucUgugaacagu......................................................................   1    1    egg
....................aagagagcuauccgugaUcagu......................................................................   1    1    egg
....................aagaUagcuauccgugaacagu......................................................................   8    1    egg
....................Uagagagcuauccgugaacagu......................................................................   1    1    egg
....................aagagagUuauccgugaacagu......................................................................   1    1    egg
....................aagagagcuauccgugaacagA......................................................................   5    1    egg
....................aagagagcuauccgugaacGgu......................................................................   1    1    egg
....................aagagGgcuauccgugaacagu......................................................................   2    1    egg
....................aagagagcuauccgugUacagu......................................................................   1    1    egg
....................Nagagagcuauccgugaacagu......................................................................   2    1    egg
....................aagagagcuauccgugaGcagu......................................................................   1    1    egg
....................aagagagcCauccgugaacagu......................................................................   2    1    egg
....................aaAagagcuauccgugaacagu......................................................................   1    1    egg
....................aagagagcuauccgCgaacagu......................................................................   1    1    egg
....................aagagagcuaCccgugaacagu......................................................................   1    1    egg
....................aagagagcuauccgugaacGgua.....................................................................   1    1    egg
....................Nagagagcuauccgugaacagua.....................................................................   1    1    egg
....................aagagagcuaCccgugaacagua.....................................................................   1    1    egg
....................aagaCagcuauccgugaacagua.....................................................................   3    1    egg
....................aagagGgcuauccgugaacagua.....................................................................   1    1    egg
....................aagUgagcuauccgugaacagua.....................................................................   2    1    egg
....................aagagagcuauccgugaacagua.....................................................................   75   0    egg
....................aagagagUuauccgugaacagua.....................................................................   1    1    egg
....................aagaUagcuauccgugaacagua.....................................................................   5    1    egg
....................aagagagcuauccgugaacagCa.....................................................................   1    1    egg
....................aagagagcuauccgugaacaguU.....................................................................   7    1    egg
....................aagagagcuauccgugaacaguG.....................................................................   1    1    egg
```

```
                    Mature                               Star
uucaauggucaguagcuauaaagagagcuauccgugaacaguacuguuagaggagacugucauggauuugcucucuuuauuacacuggucaaaaucaucagguagcaacuac
....................aagagagcuauccgugaacaguaU....................................................................    14    1    egg
....................aagagagcuauccgugaacaguaA....................................................................    9     1    egg
....................aagagagcuauccgugaacaguaAu...................................................................    1     1    egg
.....................agagagcuauccgugaacagu......................................................................    1     0    egg
......................gagagcuauccgugaacagu......................................................................    1     0    egg
........................................................cugucauggauuugcucC......................................    1     1    egg
........................................................cugucauggauuugcucuc.....................................    1     0    egg
........................................................cugucauggaGuugcucuc.....................................    1     1    egg
........................................................cugucauggauuugcucucu....................................    2     0    egg
........................................................cugucauggauuugcucucuu...................................    8     0    egg
........................................................cugAcauggauuugcucucuu...................................    1     1    egg
........................................................cugucaCggauuugcucucuuu..................................    1     1    egg
........................................................cugucauggauuCgcucucuuu..................................    1     1    egg
........................................................cugucauggauuugcCcucuuu..................................    2     1    egg
........................................................cuguUauggauuugcucucuuu..................................    5     1    egg
........................................................cugCcauggauuugcucucuuu..................................    3     1    egg
........................................................cugucauggauuugcucucuuu..................................    303   0    egg
........................................................cugucauggauuugcucucuuG..................................    1     1    egg
........................................................cugAcauggauuugcucucuuu..................................    1     1    egg
........................................................cugucauggauuugcucucGuu..................................    1     1    egg
........................................................cugucauggauuugcucucCuu..................................    2     1    egg
........................................................cugucauggauuugcucucuuC..................................    13    1    egg
........................................................cugucauggauuugcucucAuu..................................    1     1    egg
........................................................cugGcauggauuugcucucuuu..................................    8     1    egg
........................................................cugucCuggauuugcucucuuu..................................    3     1    egg
........................................................cugucauggauCugcucucuuu..................................    1     1    egg
........................................................cugucauggGuuugcucucuuu..................................    3     1    egg
........................................................cugucaugUauuugcucucuuu..................................    1     1    egg
........................................................cugucauggauuugcucucuuA..................................    3     1    egg
........................................................cugucauggCuuugcucucuuu..................................    1     1    egg
........................................................cugucauggauuugcucucuCu..................................    1     1    egg
........................................................cCgucauggauuugcucucuuu..................................    1     1    egg
........................................................cuAucauggauuugcucucuuu..................................    1     1    egg
........................................................cugucauggauuugcucuUuuu..................................    2     1    egg
........................................................cAgucauggauuugcucucuuu..................................    1     1    egg
........................................................cugucauggauuugcucCcuuua.................................    2     1    egg
........................................................cugCcauggauuugcucucuuua.................................    7     1    egg
........................................................cugucauggauuCgcucucuuua.................................    1     1    egg
........................................................cugucauggauuugcucucuuuC.................................    1     1    egg
........................................................cugucauggauuugcucucuuuU.................................    18    1    egg
........................................................cugucaCggauuugcucucuuua.................................    1     1    egg
........................................................cugucauggauCugcucucuuua.................................    4     1    egg
........................................................cugucauggaGuugcucucuuua.................................    2     1    egg
........................................................cugucauggauuugcucucCuua.................................    2     1    egg
........................................................cuguUauggauuugcucucuuua.................................    6     1    egg
........................................................cugucGuggauuugcucucuuua.................................    1     1    egg
........................................................cugucauggauuugcucucuuua.................................    420   0    egg
........................................................cCgucauggauuugcucucuuua.................................    3     1    egg
........................................................cugucauggauuugcuUucuuua.................................    1     1    egg
........................................................cugucauggauuugcucuUuuua.................................    1     1    egg
........................................................cugucauggauuugUucucuuua.................................    2     1    egg
........................................................cugucauggaAuugcucucuuua.................................    2     1    egg
........................................................cugucUuggauuugcucucuuua.................................    2     1    egg
........................................................cugucCuggauuugcucucuuua.................................    5     1    egg
........................................................cAgucauggauuugcucucuuua.................................    1     1    egg
........................................................cugGcauggauuugcucucuuua.................................    11    1    egg
........................................................cugucauggauuugcucucuuuG.................................    2     1    egg
........................................................cugAcauggauuugcucucuuua.................................    3     1    egg
........................................................Nugucauggauuugcucucuuua.................................    3     1    egg
........................................................cugucauggauuugcucucuuuau................................    5     0    egg
........................................................cugucauggauuugcucucuuuaA................................    38    1    egg
........................................................cugucauggGuuugcucucuuuau................................    1     1    egg
........................................................cugGcauggauuugcucucuuuau................................    1     1    egg
........................................................cugucauggauuugcucucuuuauA...............................    1     1    egg
.........................................................ugucauggauuugcucucu....................................    3     0    egg
.........................................................ugucauggauuugcucucuC...................................    2     1    egg
.........................................................ugucauggauuugcucucuu...................................    12    0    egg
.........................................................ugucGuggauuugcucucuu...................................    1     1    egg
.........................................................ugucauggGuuugcucucuu...................................    1     1    egg
.........................................................uAucauggauuugcucucuuu..................................    1     1    egg
```

```
                    Mature                                 Star
uucaauggucaguagcuauaaagagagcuauccgugaacaguacuguuagaggagacugucauggauuugcucucuuuauuacacuggucaaaaucaucagguagcaacuac
.........................................................ugucauggauuAgcucucuuu..................................  1     1  egg
.........................................................ugucauggauuugcucucuuu..................................  329   0  egg
.........................................................Cgucauggauuugcucucuuu..................................  1     1  egg
.........................................................ugucauggauuugUucucuuu..................................  3     1  egg
.........................................................ugucUuggauuugcucucuuu..................................  7     1  egg
.........................................................ugucaugAauuugcucucuuu..................................  1     1  egg
.........................................................ugucauggauuugcucucuuG..................................  1     1  egg
.........................................................ugucauggauuGgcucucuuu..................................  1     1  egg
.........................................................ugucauggauuCgcucucuuu..................................  1     1  egg
.........................................................ugucauggauuugcucucuuC..................................  21    1  egg
.........................................................ugucauggauuugcucucuuA..................................  2     1  egg
.........................................................ugucauggauCugcucucuuu..................................  1     1  egg
.........................................................ugucauUgauuugcucucuuu..................................  1     1  egg
.........................................................ugucauggauuugcucCcuuu..................................  2     1  egg
.........................................................ugucauggauuugcAcucuuu..................................  1     1  egg
.........................................................ugucaCggauuugcucucuuu..................................  3     1  egg
.........................................................ugucauggauuugcCcucuuu..................................  3     1  egg
.........................................................ugCcauggauuugcucucuuu..................................  2     1  egg
.........................................................uUucauggauuugcucucuuu..................................  1     1  egg
.........................................................ugucGuggauuugcucucuuu..................................  6     1  egg
.........................................................ugucauggauuugcucucCuu..................................  4     1  egg
.........................................................uguUauggauuugcucucuuu..................................  2     1  egg
.........................................................ugucauggauAugcucucuuu..................................  1     1  egg
.........................................................ugucauggauuuAcucucuuu..................................  1     1  egg
.........................................................Cgucauggauuugcucucuuua.................................  2     1  egg
.........................................................ugCcauggauuugcucucuuua.................................  4     1  egg
.........................................................ugucauggauuugcucuUuuua.................................  1     1  egg
.........................................................ugucaugCauuugcucucuuua.................................  1     1  egg
.........................................................ugucauggauAugcucucuuua.................................  1     1  egg
.........................................................ugAcauggauuugcucucuuua.................................  1     1  egg
.........................................................ugucauggauCugcucucuuua.................................  3     1  egg
.........................................................ugucauggauuugcucucCuua.................................  7     1  egg
.........................................................ugucauggauuCgcucucuuua.................................  4     1  egg
.........................................................ugucauggauuugcucAcuuua.................................  1     1  egg
.........................................................ugucaAggauuugcucucuuua.................................  2     1  egg
.........................................................ugucauggauuugcucucuuuG.................................  2     1  egg
.........................................................uguUauggauuugcucucuuua.................................  12    1  egg
.........................................................ugucauggauuAgcucucuuua.................................  1     1  egg
.........................................................ugucauAgauuugcucucuuua.................................  2     1  egg
.........................................................ugucGuggauuugcucucuuua.................................  17    1  egg
.........................................................Agucauggauuugcucucuuua.................................  1     1  egg
.........................................................ugucauggauuugcucucuuAa.................................  1     1  egg
.........................................................ugucaugAauuugcucucuuua.................................  2     1  egg
.........................................................ugucauggaCuugcucucuuua.................................  5     1  egg
.........................................................ugucauggaAuugcucucuuua.................................  2     1  egg
.........................................................uAucauggauuugcucucuuua.................................  4     1  egg
.........................................................ugucauggauuugcAcucuuua.................................  1     1  egg
.........................................................ugucauggauuugcucucGuua.................................  1     1  egg
.........................................................uguGauggauuugcucucuuua.................................  1     1  egg
.........................................................ugucaGggauuugcucucuuua.................................  1     1  egg
.........................................................ugucauggauuugcuUucuuua.................................  1     1  egg
.........................................................ugucauggauuugcCcucuuua.................................  7     1  egg
.........................................................ugucauUgauuugcucucuuua.................................  2     1  egg
.........................................................ugucauggGuuugcucucuuua.................................  11    1  egg
.........................................................ugucauggauuGgcucucuuua.................................  6     1  egg
.........................................................ugucauggauuugcucucuuCa.................................  1     1  egg
.........................................................ugucauggauuugcucucuAua.................................  1     1  egg
.........................................................ugucauggauuugcGcucuuua.................................  1     1  egg
.........................................................ugucUuggauuugcucucuuua.................................  17    1  egg
.........................................................uCucauggauuugcucucuuua.................................  1     1  egg
.........................................................Ngucauggauuugcucucuuua.................................  3     1  egg
.........................................................ugucauggauuugcucucuuGa.................................  3     1  egg
.........................................................ugucauggauGugcucucuuua.................................  3     1  egg
.........................................................ugucaCggauuugcucucuuua.................................  4     1  egg
.........................................................ugucauggauuugcucucuuua.................................  1249  0  egg
.........................................................ugucauggUuuugcucucuuua.................................  1     1  egg
.........................................................ugucauggauuugcucucuuuC.................................  6     1  egg
.........................................................ugucauggauuugcucucuuuU.................................  31    1  egg
.........................................................ugucauggauuugcucCcuuua.................................  6     1  egg
.........................................................ugucauggauuugUucucuuua.................................  1     1  egg
```

```
                    Mature                                      Star
uucaauggucaguagcuauaaagagagcuauccgugaacaguacuguuagaggagacugucauggauuugcucucuuuauuacacuggucaaaaucaucagguagcaacuac
.........................................................ugucauggauuugcuUucuuuau................................   1     1    egg
.........................................................ugucauggauuugcucucuuuaG................................   1     1    egg
.........................................................ugucauggauuugcucucuuuaC................................   2     1    egg
.........................................................ugucauggauuugcucucuuuUu................................   5     1    egg
.........................................................ugucauggauuugcucucuuuau................................   19    0    egg
.........................................................ugucauggauuugcucucuuuaA................................   79    1    egg
.........................................................ugucauggauuugcucucuuuauA...............................   2     1    egg
..........................................................gucauggauuugcucucuuuaA................................   1     1    egg

....................aagagagcuauccgugaac.........................................................................   3     0    te1
....................aagagagcuauccgugaaca........................................................................   1     0    te1
....................aagagagcuauccgugaacU........................................................................   1     1    te1
....................aagagagcuauccguUaacag.......................................................................   1     1    te1
....................aagagagcuauccgugaacag.......................................................................   5     0    te1
....................aagagagcuauccgugaacagu......................................................................   35    0    te1
....................aagagagcuauccgugaacagG......................................................................   1     1    te1
....................aagagagcuauccgugaacaguU.....................................................................   1     1    te1
....................aagagagcuauccgugaacaguaU....................................................................   2     1    te1
........................................................cugucauggauuugcucucuuu..................................   1     0    te1
.........................................................ugucauggauuugcucucuuu..................................   10    0    te1
.........................................................ugucauggauuugcucucuuua.................................   7     0    te1
.........................................................ugucauggauuugcucucuuuU.................................   5     1    te1

................uCuaaagagagcuauccgugaacagu......................................................................   1     1    T53
..................uaaagagagcuauccgugaac.........................................................................   4     0    T53
..................uaaagagagcuauccgugaacUg.......................................................................   1     1    T53
..................uUaagagagcuauccgugaacag.......................................................................   2     1    T53
..................uUaagagagcuauccgugaacagu......................................................................   9     1    T53
..................uaaagagagcuauccgugaacagu......................................................................   5     0    T53
..................uUaagagagcuauccgugaacagua.....................................................................   1     1    T53
..................uaaagagagcuauccgugaacagua.....................................................................   1     0    T53
...................aaagagagcuauccguga...........................................................................   1     0    T53
...................aaagagagcuauccgugaa..........................................................................   3     0    T53
...................Uaagagagcuauccgugaac.........................................................................   2     1    T53
...................aaagagagcuauccgugaac.........................................................................   142   0    T53
...................aaagagagUuauccgugaac.........................................................................   1     1    T53
...................aaagagagcuauccgugaaA.........................................................................   1     1    T53
...................Uaagagagcuauccgugaaca........................................................................   1     1    T53
...................aaagagagcuauccgugaaca........................................................................   80    0    T53
...................aaagagagcuauccgugaacaU.......................................................................   1     1    T53
...................aaagagagcuauccgugaacaA.......................................................................   1     1    T53
...................aaagagagcuauccgugaacag.......................................................................   83    0    T53
...................aGagagagcuauccgugaacag.......................................................................   1     1    T53
...................aaagagagUuauccgugaacagu......................................................................   2     1    T53
...................aaagagagcuauccgugUacagu......................................................................   1     1    T53
...................aaagagagcuauccgugaacagu......................................................................   1038  0    T53
...................aaagagagcCauccgugaacagu......................................................................   1     1    T53
...................Caagagagcuauccgugaacagu......................................................................   1     1    T53
...................aaagagGgcuauccgugaacagu......................................................................   1     1    T53
...................aaagagUgcuauccgugaacagu......................................................................   3     1    T53
...................Uaagagagcuauccgugaacagu......................................................................   32    1    T53
...................aaagagagcuauccgugaacagA......................................................................   16    1    T53
...................Gaagagagcuauccgugaacagu......................................................................   2     1    T53
...................aaagagagcuauccgugGacagu......................................................................   1     1    T53
...................aaagagagcuauccgAgaacagu......................................................................   3     1    T53
...................aaagagagAuauccgugaacagu......................................................................   1     1    T53
...................aaagagagcuauccgugaacagC......................................................................   2     1    T53
...................aCagagagcuauccgugaacagu......................................................................   2     1    T53
...................aaagagagcuauccgugaacUgu......................................................................   1     1    T53
...................aaagagaCcuauccgugaacagua.....................................................................   1     1    T53
...................aaagagagcuauccAugaacagua.....................................................................   1     1    T53
...................aaagagagcuauccgugaacaguU.....................................................................   56    1    T53
...................Uaagagagcuauccgugaacagua.....................................................................   2     1    T53
...................aaagagagcuauccgugaacagua.....................................................................   55    0    T53
...................aaagagagUuauccgugaacagua.....................................................................   1     1    T53
...................aaagagagcuauccgugaacaguaU....................................................................   85    1    T53
...................aaagagagcuauccgugaacaguac....................................................................   1     0    T53
...................aaagagagcuauccgugaacaguaUu...................................................................   10    1    T53
...................aaagagagcuauccgugaacaguacu...................................................................   1     0    T53
...................aaagagagcuauccgugaacaguacA...................................................................   1     1    T53
```

```
                   Mature                Star
uucaauggucaguagcuauaaagagagcuauccgugaacaguacuguuagaggagacugucauggauuugcucucuuuauuacacuggucaaaaucaucagguagcaacuac
...................aaagagagcuauccgugaacaguaAu...................................................................   4     1   T53
....................aagagCgcuauccgugaa..........................................................................   1     1   T53
....................aagagagcuauccgugaa..........................................................................   170   0   T53
....................aagagagcuauccgugaU..........................................................................   2     1   T53
....................aagagGgcuauccgugaa..........................................................................   1     1   T53
....................aGgagagcuauccgugaa..........................................................................   3     1   T53
....................aagagagcuauAcgugaac.........................................................................   1     1   T53
....................aaUagagcuauccgugaac.........................................................................   1     1   T53
....................aagagUgcuauccgugaac.........................................................................   5     1   T53
....................aagagGgcuauccgugaac.........................................................................   1     1   T53
....................aagagagcuauccguCaac.........................................................................   1     1   T53
....................aCgagagcuauccgugaac.........................................................................   1     1   T53
....................aagagaCcuauccgugaac.........................................................................   1     1   T53
....................aagagagAuauccgugaac.........................................................................   2     1   T53
....................aagagagcuauccgugaUc.........................................................................   2     1   T53
....................aagagagcAauccgugaac.........................................................................   2     1   T53
....................aagUgagcuauccgugaac.........................................................................   1     1   T53
....................aagagagcuauccgugaaG.........................................................................   2     1   T53
....................aagagagcuauccgugaaA.........................................................................   47    1   T53
....................aagagagcuauccgugaac.........................................................................   4763  0   T53
....................aagagagcuauccguAaac.........................................................................   1     1   T53
....................aGgagagcuauccgugaac.........................................................................   3     1   T53
....................aUgagagcuauccgugaac.........................................................................   3     1   T53
....................aagagaAcuauccgugaac.........................................................................   1     1   T53
....................aagagagcuCuccgugaac.........................................................................   1     1   T53
....................aagagagUuauccgugaac.........................................................................   4     1   T53
....................aagagagcCauccgugaac.........................................................................   1     1   T53
....................aaAagagcuauccgugaac.........................................................................   1     1   T53
....................aagagagcuauccgugaaU.........................................................................   11    1   T53
....................aagagagcuauccgugCac.........................................................................   1     1   T53
....................aagagagcuauccgugaacC........................................................................   2     1   T53
....................aagagagcuauccgugaaAa........................................................................   2     1   T53
....................aagagagcuauccgugUaca........................................................................   2     1   T53
....................aagagaAcuauccgugaaca........................................................................   1     1   T53
....................aagagagcuauccgugaacG........................................................................   2     1   T53
....................aagagagcuauccgugaaca........................................................................   1846  0   T53
....................aUgagagcuauccgugaaca........................................................................   1     1   T53
....................aagagGgcuauccgugaaca........................................................................   2     1   T53
....................aagagagcAauccgugaaca........................................................................   2     1   T53
....................aagUgagcuauccgugaaca........................................................................   1     1   T53
....................aagagagcuauUcgugaaca........................................................................   1     1   T53
....................aagagagcuauccgugaaGa........................................................................   1     1   T53
....................aagagagcuauccgugCaca........................................................................   2     1   T53
....................aagagagcuauccgugaacU........................................................................   7     1   T53
....................Uagagagcuauccgugaaca........................................................................   1     1   T53
....................aagagagcuauGcgugaaca........................................................................   1     1   T53
....................aagagagUuauccgugaacag.......................................................................   9     1   T53
....................aagagagcuauccguUaacag.......................................................................   1     1   T53
....................aagagaCcuauccgugaacag.......................................................................   1     1   T53
....................aagagUgcuauccgugaacag.......................................................................   2     1   T53
....................aaCagagcuauccgugaacag.......................................................................   1     1   T53
....................aagagagcuauccgugaacaC.......................................................................   1     1   T53
....................aagagagcuaCccgugaacag.......................................................................   2     1   T53
....................aagUgagcuauccgugaacag.......................................................................   4     1   T53
....................aagagagcuauUcgugaacag.......................................................................   2     1   T53
....................aUgagagcuauccgugaacag.......................................................................   5     1   T53
....................aagagagcuaAccgugaacag.......................................................................   1     1   T53
....................aagagagcuauccgugaacag.......................................................................   5679  0   T53
....................aaAagagcuauccgugaacag.......................................................................   3     1   T53
....................aagagagAuauccgugaacag.......................................................................   1     1   T53
....................aagagagcuauccgugGacag.......................................................................   1     1   T53
....................aagagGgcuauccgugaacag.......................................................................   2     1   T53
....................aagagagcuauccgugUacag.......................................................................   2     1   T53
....................aGgagagcuauccgugaacag.......................................................................   1     1   T53
....................aagagagcuauccUugaacag.......................................................................   1     1   T53
....................aagagagcuauccgugaacaU.......................................................................   36    1   T53
....................aagagCgcuauccgugaacag.......................................................................   1     1   T53
....................aagagagcAauccgugaacag.......................................................................   1     1   T53
....................aagagaAcuauccgugaacag.......................................................................   4     1   T53
....................aagagagcuCuccgugaacag.......................................................................   1     1   T53
```

```
                    Mature               Star
uucaauggucaguagcuauaaagagagcuauccgugaacaguacuguuagaggagacugucauggauuugcucucuuuauuacacuggucaaaaucaucagguagcaacuac
....................aagagagcuauccAugaacag.......................................................................	4	1	T53
....................aagagagcCauccgugaacag.......................................................................	1	1	T53
....................aaUagagcuauccgugaacag.......................................................................	1	1	T53
....................aagagagcuauccgCgaacag.......................................................................	1	1	T53
....................aagaUagcuauccgugaacag.......................................................................	2	1	T53
....................aagagagcuauccgugaacaA.......................................................................	17	1	T53
....................aagagaUcuauccgugaacag.......................................................................	1	1	T53
....................aagagagcuauccgAgaacag.......................................................................	1	1	T53
....................Cagagagcuauccgugaacagu......................................................................	1	1	T53
....................aagagagcuaGccgugaacagu......................................................................	1	1	T53
....................aagagagcuaCccgugaacagu......................................................................	9	1	T53
....................aagagaAcuauccgugaacagu......................................................................	29	1	T53
....................Uagagagcuauccgugaacagu......................................................................	5	1	T53
....................aagCgagcuauccgugaacagu......................................................................	5	1	T53
....................aagagagcuauccguCaacagu......................................................................	8	1	T53
....................aagagagcuauccgugaacGgu......................................................................	9	1	T53
....................aagagagcuauccgugaacaCu......................................................................	13	1	T53
....................aaCagagcuauccgugaacagu......................................................................	7	1	T53
....................aagagagcCauccgugaacagu......................................................................	4	1	T53
....................aagagagcuUuccgugaacagu......................................................................	2	1	T53
....................aagagagGuauccgugaacagu......................................................................	4	1	T53
....................aagagagcuauccgugaacagC......................................................................	124	1	T53
....................aagagagcuauccgGgaacagu......................................................................	2	1	T53
....................aagagagcuCuccgugaacagu......................................................................	48	1	T53
....................aagagagcuauccgugaacagu......................................................................	130105	0	T53
....................aagagagcuauccgugaacagG......................................................................	40	1	T53
....................aagagagcuauccAugaacagu......................................................................	59	1	T53
....................aagagagAuauccgugaacagu......................................................................	8	1	T53
....................aagagagcAauccgugaacagu......................................................................	54	1	T53
....................aagagagcGauccgugaacagu......................................................................	3	1	T53
....................aagaUagcuauccgugaacagu......................................................................	6	1	T53
....................aagagaUcuauccgugaacagu......................................................................	8	1	T53
....................aagaAagcuauccgugaacagu......................................................................	2	1	T53
....................aagaCagcuauccgugaacagu......................................................................	6	1	T53
....................aaUagagcuauccgugaacagu......................................................................	17	1	T53
....................aUgagagcuauccgugaacagu......................................................................	74	1	T53
....................aagagagcuauccgugaacaUu......................................................................	36	1	T53
....................aagagagcuauccguAaacagu......................................................................	26	1	T53
....................aagagagcuauccgugaUcagu......................................................................	8	1	T53
....................aagagagcuaucAgugaacagu......................................................................	2	1	T53
....................aCgagagcuauccgugaacagu......................................................................	20	1	T53
....................aagagagcuauccgugaacUgu......................................................................	11	1	T53
....................aagagaCcuauccgugaacagu......................................................................	5	1	T53
....................aagagagcuauccCugaacagu......................................................................	3	1	T53
....................aagagagcuaucUgugaacagu......................................................................	22	1	T53
....................aagagGgcuauccgugaacagu......................................................................	74	1	T53
....................aGgagagcuauccgugaacagu......................................................................	60	1	T53
....................aagagagcuauccgugaaUagu......................................................................	34	1	T53
....................aagagagcuauccgugaacagA......................................................................	920	1	T53
....................aagagagcuauccgugCacagu......................................................................	11	1	T53
....................aagagagcuauccgCgaacagu......................................................................	20	1	T53
....................aagagUgcuauccgugaacagu......................................................................	84	1	T53
....................aagagagcuauccgAgaacagu......................................................................	28	1	T53
....................aagagagcuauUcgugaacagu......................................................................	49	1	T53
....................aagagagUuauccgugaacagu......................................................................	104	1	T53
....................aagagagcuauccgugaaAagu......................................................................	7	1	T53
....................aagagagcuauGcgugaacagu......................................................................	3	1	T53
....................aagagagcuauAcgugaacagu......................................................................	4	1	T53
....................aagagCgcuauccgugaacagu......................................................................	27	1	T53
....................aagagagcuauccgugGacagu......................................................................	22	1	T53
....................aagagagcuauccgugaGcagu......................................................................	13	1	T53
....................aagagagcuauccUugaacagu......................................................................	9	1	T53
....................aagagagcuauccguUaacagu......................................................................	17	1	T53
....................aagagagcuauccgugaCcagu......................................................................	1	1	T53
....................aagagagcuGuccgugaacagu......................................................................	2	1	T53
....................aagagagcuauccgugaacaAu......................................................................	19	1	T53
....................aagUgagcuauccgugaacagu......................................................................	36	1	T53
....................aagGgagcuauccgugaacagu......................................................................	7	1	T53
....................aagagagcuauccgugaaGagu......................................................................	5	1	T53
....................aagagagcuauccgugUacagu......................................................................	31	1	T53
```

Mature Star

```
uucaauggucaguagcuauaaagagagcuauccgugaacaguacuguuagaggagacugucauggauuugcucucuuuauuacacuggucaaaaucaucagguagcaacuac
....................aaAagagcuauccgugaacagu......................................................................  11     1  T53
....................Gagagagcuauccgugaacagu......................................................................  3      1  T53
....................aagagagcuaAccgugaacagu......................................................................  1      1  T53
....................aagagagcuaucGgugaacagu......................................................................  7      1  T53
....................aagagaAcuauccgugaacagua.....................................................................  1      1  T53
....................aagagagcuCuccgugaacagua.....................................................................  1      1  T53
....................aagagagcuauccgugaacagAa.....................................................................  36     1  T53
....................aagagagcuauccgugaacGgua.....................................................................  2      1  T53
....................aagagagcuauccAugaacagua.....................................................................  9      1  T53
....................aagagagUuauccgugaacagua.....................................................................  11     1  T53
....................aagagagcuaucUgugaacagua.....................................................................  4      1  T53
....................aagagagcuauccgugaacaguU.....................................................................  6429   1  T53
....................aagagagcuauccgugaacagCa.....................................................................  3      1  T53
....................aagagagcuauccgugaacagua.....................................................................  14124  0  T53
....................aagagagcuauccgugaaUagua.....................................................................  2      1  T53
....................aagagagcuauccgAgaacagua.....................................................................  6      1  T53
....................aagagGgcuauccgugaacagua.....................................................................  10     1  T53
....................aagagagcuaAccgugaacagua.....................................................................  1      1  T53
....................aCgagagcuauccgugaacagua.....................................................................  3      1  T53
....................aagagagcuauccgCgaacagua.....................................................................  2      1  T53
....................aagagagcuauccgugaaGagua.....................................................................  1      1  T53
....................aagagagcuauccgGgaacagua.....................................................................  1      1  T53
....................aaAagagcuauccgugaacagua.....................................................................  2      1  T53
....................aagagagcuauUcgugaacagua.....................................................................  4      1  T53
....................Uagagagcuauccgugaacagua.....................................................................  3      1  T53
....................aagagagcuaucGgugaacagua.....................................................................  1      1  T53
....................aUgagagcuauccgugaacagua.....................................................................  7      1  T53
....................aaUagagcuauccgugaacagua.....................................................................  2      1  T53
....................aagagCgcuauccgugaacagua.....................................................................  2      1  T53
....................aagagagcuauccgugaacagGa.....................................................................  1      1  T53
....................aagagagcuauccgugaacaguC.....................................................................  47     1  T53
....................aagagagcAauccgugaacagua.....................................................................  6      1  T53
....................aagagagcuauccgugaGcagua.....................................................................  3      1  T53
....................aagagUgcuauccgugaacagua.....................................................................  10     1  T53
....................aagagagcuauccgugaacaguG.....................................................................  30     1  T53
....................aagagagcuauccgugCacagua.....................................................................  1      1  T53
....................aagagagcuauccguAaacagua.....................................................................  1      1  T53
....................aagUgagcuauccgugaacagua.....................................................................  5      1  T53
....................aagagagcuauccgugaacaAua.....................................................................  1      1  T53
....................aagagagcuaGccgugaacagua.....................................................................  1      1  T53
....................Gagagagcuauccgugaacagua.....................................................................  2      1  T53
....................aagCgagcuauccgugaacagua.....................................................................  1      1  T53
....................aagagagcuauccgugGacagua.....................................................................  1      1  T53
....................aagagagcuauccgugUacagua.....................................................................  6      1  T53
....................aagagagcuauGcgugaacagua.....................................................................  1      1  T53
....................aagagagcuauccgugaacaCua.....................................................................  4      1  T53
....................aagaCagcuauccgugaacagua.....................................................................  1      1  T53
....................aagagagcuauccguUaacagua.....................................................................  2      1  T53
....................aGgagagcuauccgugaacagua.....................................................................  14     1  T53
....................aagagagcuauccguCaacagua.....................................................................  2      1  T53
....................aagaUagcuauccgugaacagua.....................................................................  1      1  T53
....................aagagagcuauccgugaacaUua.....................................................................  2      1  T53
....................aagagUgcuauccgugaacaguac....................................................................  1      1  T53
....................aagagagcuauccgugaacaguaA....................................................................  555    1  T53
....................aagagagcuauccgugaacaguaG....................................................................  20     1  T53
....................aagagagcuauccgugaacaguUc....................................................................  1      1  T53
....................aagagagcuauccgugaacaguac....................................................................  40     0  T53
....................aagagagcuauccgugaacaguaU....................................................................  8328   1  T53
....................Gagagagcuauccgugaacaguac....................................................................  1      1  T53
....................aagagagcuauccgugaacagAac....................................................................  2      1  T53
....................aagagagcuauccgugaacaguaUu...................................................................  450    1  T53
....................aagagagcuauAcgugaacaguacu...................................................................  1      1  T53
....................aagagagcuauccgugaacaguacu...................................................................  14     0  T53
....................aagagagcuauccgugaacaguUcu...................................................................  1      1  T53
....................aagagagcuauccgugaacaguacA...................................................................  8      1  T53
....................aagagagcuauccgugaacaguacG...................................................................  1      1  T53
....................aagagagcuauccgugaacaguaGu...................................................................  1      1  T53
....................aagagagcuauccgugaacaguaAu...................................................................  381    1  T53
....................aagagagcuauccgugaacaguacugu.................................................................  4      0  T53
.....................agagagcuauccgugaac.........................................................................  2      0  T53
```

```
                    Mature              Star
uucaauggucaguagcuauaaagagagcuauccgugaacaguacuguuagaggagacugucauggauuugcucucuuuauuacacuggucaaaaucaucagguagcaacuac
.....................agagagcuauccgugaaca........................................................................ 1 0 T53
.....................agagagcuauccgugaacag....................................................................... 3 0 T53
.....................agagagcuauccgugaacagu...................................................................... 80 0 T53
.....................aUagagcuauccgugaacagua..................................................................... 1 1 T53
.....................agagagcuauccgugaacaguU..................................................................... 7 1 T53
.....................agagagcuauccgugaacagua..................................................................... 13 0 T53
.....................agagagcuauccgugaacaguaU.................................................................... 13 1 T53
.....................agagagcuauccgugaacaguaA.................................................................... 1 1 T53
.....................agagagcuauccgugaacaguaAu................................................................... 1 1 T53
.....................agagagcuauccgugaacaguacugu................................................................. 2 0 T53
.....................agagagcuauccgugaacaguacuguu................................................................ 1 0 T53
......................Aagagcuauccgugaacagu...................................................................... 10 1 T53
......................gagagcuauccgugaacagu...................................................................... 8 0 T53
.......................agagcuauccgugaacag....................................................................... 2 0 T53
.......................agagcuauccgugaacagA...................................................................... 1 1 T53
.......................agagcuauccgugaacagu...................................................................... 18 0 T53
.......................agagcuauccgugaacaguU..................................................................... 2 1 T53
.......................agagcuauccgugaacagua..................................................................... 3 0 T53
.......................agagcuauccgugaacaguaU.................................................................... 1 1 T53
........................gagcuauccgugaacagu...................................................................... 8 0 T53
........................gagcuauccgugaacaguU..................................................................... 1 1 T53
........................gagcuauccgugaacaguaAu................................................................... 2 1 T53
.......................................aguacuguuagaggagacugucau................................................. 1 0 T53
..........................................acuguuagaggagacugucauggauu............................................ 1 0 T53
..........................................acuguuagaggagacugucauggauuu........................................... 1 0 T53
............................................uguuagaggagacugucauggau............................................. 1 0 T53
............................................uguuagaggagacugucauggauuugc......................................... 4 0 T53
...................................................ggagCcugucauggauuugcucucuuu.................................. 1 1 T53
.....................................................Ggacugucauggauuugcucucuuua................................. 1 1 T53
.....................................................agaAugucauggauuugcucucuuuau................................ 1 1 T53
......................................................gacugucauggauuugcucucuuuU................................. 1 1 T53
.......................................................acugucauggauuugcucuc..................................... 2 0 T53
.......................................................acugucauggauuugcucucu.................................... 1 0 T53
.......................................................acugucauggauuugcucucuu................................... 1 0 T53
.......................................................acugucauggauuugcucucuuC.................................. 1 1 T53
.......................................................acugucauggauuugcucucuuu.................................. 4 0 T53
.......................................................acugucauggauuugcucucuuua................................. 1 0 T53
.......................................................acugucauggauuugcucucuuuU................................. 1 1 T53
........................................................cugucauggauuugcucu...................................... 7 0 T53
........................................................cugucauggauuugcucuc..................................... 5 0 T53
........................................................cugucauggauuugcucucu.................................... 58 0 T53
........................................................cugucauggauuugcucuUu.................................... 1 1 T53
........................................................cugucauggaGuugcucucuu................................... 2 1 T53
........................................................cugucauggauuugcucucuu................................... 528 0 T53
........................................................cugucaAggauuugcucucuu................................... 1 1 T53
........................................................cugucauggauuugcucucuA................................... 3 1 T53
........................................................cugucauggauuugcucucuuC.................................. 4 1 T53
........................................................cugucauggauuugcucucuuu.................................. 5091 0 T53
........................................................cugucaugAauuugcucucuuu.................................. 6 1 T53
........................................................cugucauggauuugcAcucuuu.................................. 5 1 T53
........................................................cugucGuggauuugcucucuuu.................................. 2 1 T53
........................................................Gugucauggauuugcucucuuu.................................. 1 1 T53
........................................................cugCcauggauuugcucucuuu.................................. 1 1 T53
........................................................cugucUuggauuugcucucuuu.................................. 1 1 T53
........................................................cugucauggCuuugcucucuuu.................................. 1 1 T53
........................................................cugucauggauCugcucucuuu.................................. 1 1 T53
........................................................cugucaugUauuugcucucuuu.................................. 3 1 T53
........................................................cugucauggauuuAcucucuuu.................................. 10 1 T53
........................................................cuguUauggauuugcucucuuu.................................. 1 1 T53
........................................................cugucauggauAugcucucuuu.................................. 1 1 T53
........................................................cugAcauggauuugcucucuuu.................................. 1 1 T53
........................................................cugucauggauuugcCcucuuu.................................. 1 1 T53
........................................................cugucauggauuugcucucCuu.................................. 1 1 T53
........................................................cugucauggauuugcucAcuuu.................................. 1 1 T53
........................................................cugucauggauuugcucucAuu.................................. 2 1 T53
........................................................cugucauggGuuugcucucuuu.................................. 1 1 T53
........................................................cugucauggauuugAucucuuu.................................. 1 1 T53
........................................................cugucauggauuugcucuUuuu.................................. 4 1 T53
........................................................cugucaAggauuugcucucuuu.................................. 1 1 T53
........................................................cuUucauggauuugcucucuuu.................................. 1 1 T53
```

```
                    Mature                                      Star
uucaauggucaguagcuauaaagagagcuauccgugaacaguacuguuagaggagacugucauggauuugcucucuuuauuacacuggucaaaaucaucagguagcaacuac
........................................................cugucauggauuugcucucuAu..................................  2     1   T53
........................................................cugucauggauuCgcucucuuu..................................  1     1   T53
........................................................cugucauggauuugcucucuuA..................................  23    1   T53
........................................................cugucauAgauuugcucucuuu..................................  1     1   T53
........................................................cugucauggauuugUucucuuu..................................  1     1   T53
........................................................cugucauggauuAgcucucuuu..................................  1     1   T53
........................................................cugucauggauuugcuAucuuu..................................  1     1   T53
........................................................Uugucauggauuugcucucuuu..................................  3     1   T53
........................................................cugucauggauuugcuUucuuu..................................  1     1   T53
........................................................cugucaCggauuugcucucuuu..................................  3     1   T53
........................................................cugucauggaGuugcucucuuu..................................  2     1   T53
........................................................cugucauggauuugcucucuuG..................................  3     1   T53
........................................................cugucaugCauuugcucucuuu..................................  1     1   T53
........................................................Augucauggauuugcucucuuua.................................  1     1   T53
........................................................cugucauggaGuugcucucuuua.................................  1     1   T53
........................................................cugucauggauuuAcucucuuua.................................  7     1   T53
........................................................cugucauggauuugUucucuuua.................................  3     1   T53
........................................................cugucauggauuugcucuUuuua.................................  5     1   T53
........................................................cugucauggauuugcucCcuuua.................................  1     1   T53
........................................................cugucauggauuugcuGucuuua.................................  1     1   T53
........................................................cugucauggauuugcucucuuuC.................................  12    1   T53
........................................................cugucauggauuugcucucuuua.................................  3960  0   T53
........................................................cugucauggauuUcucucuuua.................................  1     1   T53
........................................................cugucauggaCuugcucucuuua.................................  1     1   T53
........................................................cugucauggGuuugcucucuuua.................................  1     1   T53
........................................................cugucauggauuugcucucCuua.................................  1     1   T53
........................................................cugucauggauuugcucucuuAa.................................  4     1   T53
........................................................cugucaugCauuugcucucuuua.................................  1     1   T53
........................................................cugucauggauuugcucAcuuua.................................  1     1   T53
........................................................cugCcauggauuugcucucuuua.................................  1     1   T53
........................................................cGgucauggauuugcucucuuua.................................  1     1   T53
........................................................cugucauggCuuugcucucuuua.................................  1     1   T53
........................................................cugucGuggauuugcucucuuua.................................  1     1   T53
........................................................Uugucauggauuugcucucuuua.................................  5     1   T53
........................................................cugucauggauuugcucucuAua.................................  1     1   T53
........................................................cuguUauggauuugcucucuuua.................................  1     1   T53
........................................................cugucauggauuugGucucuuua.................................  1     1   T53
........................................................cugucaugAauuugcucucuuua.................................  2     1   T53
........................................................cugucauggauAugcucucuuua.................................  1     1   T53
........................................................cugucauggauuugcucucuCua.................................  1     1   T53
........................................................cugucauggauuugcucucuuuU.................................  441   1   T53
........................................................cugucauggauuugcucucuuuG.................................  8     1   T53
........................................................cugGcauggauuugcucucuuua.................................  2     1   T53
........................................................cugucCuggauuugcucucuuua.................................  1     1   T53
........................................................cugAcauggauuugcucucuuua.................................  2     1   T53
........................................................cugucaCggauuugcucucuuua.................................  1     1   T53
........................................................cugucauggUuuugcucucuuua.................................  1     1   T53
........................................................cugucauggauuugcucucuuCa.................................  1     1   T53
........................................................cuAucauggauuugcucucuuua.................................  2     1   T53
........................................................cugucauggauuugcucucuuuau................................  174   0   T53
........................................................cugucauggauuugcucucuuuUu................................  63    1   T53
........................................................cugucauggauuugcucucuuuCu................................  1     1   T53
........................................................cugucauggauuugcucucuuuaC................................  32    1   T53
........................................................cugucauggauuugcucucuuuaA................................  34    1   T53
........................................................cugucauggaGuugcucucuuuau................................  1     1   T53
........................................................cugucauggauuugcucucuuuaG................................  1     1   T53
........................................................cugucauggauuugcucucuuuaGu...............................  1     1   T53
........................................................cugucauggauuugcucucuuuauA...............................  5     1   T53
........................................................cugucauggauuugcucucuuuaAu...............................  5     1   T53
........................................................cugucauggauuugcucucuuuUuu...............................  7     1   T53
........................................................cugucauggauuugcucucuuuauu...............................  24    0   T53
........................................................cugucauggauuugcucucuuuauG...............................  1     1   T53
........................................................cugucauggauuugcucucuuuauuC..............................  1     1   T53
........................................................cugucauggauuugcucucuuuauuU..............................  3     1   T53
.........................................................ugucauggauuugcucuc.....................................  8     0   T53
.........................................................ugucauggauCugcucucu....................................  1     1   T53
.........................................................ugucauggauuugcucucu....................................  121   0   T53
.........................................................ugucauggauuugGucucuu...................................  1     1   T53
.........................................................uUucauggauuugcucucuu...................................  1     1   T53
.........................................................ugucauggauuuAcucucuu...................................  1     1   T53
```

```
                    Mature                                 Star
uucaauggucaguagcuauaaagagagcuauccgugaacaguacuguuagaggagacugucauggauuugcucucuuuauuacacuggucaaaaucaucagguagcaacuac
.........................................................Ggucauggauuugcucucuu...................................    1       1     T53
.........................................................ugucauggauuugcucuUuu...................................    1       1     T53
.........................................................ugucauggauuugcucCcuu...................................    1       1     T53
.........................................................ugucauggauuugcucucuu...................................    855     0     T53
.........................................................ugucauggCuuugcucucuu...................................    1       1     T53
.........................................................ugucauggauuugUucucuu...................................    1       1     T53
.........................................................ugucaugAauuugcucucuu...................................    1       1     T53
.........................................................ugucauUgauuugcucucuuu..................................    1       1     T53
.........................................................ugucauggauuAgcucucuuu..................................    3       1     T53
.........................................................ugucauAgauuugcucucuuu..................................    2       1     T53
.........................................................ugucauggauuugcucuUuuu..................................    15      1     T53
.........................................................ugucauggauuugcucucuuC..................................    3       1     T53
.........................................................ugucaCggauuugcucucuuu..................................    2       1     T53
.........................................................ugucauggauuuAcucucuuu..................................    21      1     T53
.........................................................ugucauggGuuugcucucuuu..................................    4       1     T53
.........................................................ugucauggaGuugcucucuuu..................................    10      1     T53
.........................................................ugucauCgauuugcucucuuu..................................    1       1     T53
.........................................................ugucauggauuugcAcucuuu..................................    1       1     T53
.........................................................ugucauggauuCgcucucuuu..................................    1       1     T53
.........................................................Cgucauggauuugcucucuuu..................................    3       1     T53
.........................................................uUucauggauuugcucucuuu..................................    1       1     T53
.........................................................ugucauggauuugcucucAuu..................................    1       1     T53
.........................................................ugucaugCauuugcucucuuu..................................    2       1     T53
.........................................................ugucauggauuugcucuGuuu..................................    1       1     T53
.........................................................ugucauggauuugcuUucuuu..................................    4       1     T53
.........................................................uguUauggauuugcucucuuu..................................    4       1     T53
.........................................................ugucauggauuugcucucuGu..................................    1       1     T53
.........................................................ugucauggauuugcucucuuG..................................    1       1     T53
.........................................................ugucaugUauuugcucucuuu..................................    4       1     T53
.........................................................uguGauggauuugcucucuuu..................................    1       1     T53
.........................................................ugucauggauuugcucucuuu..................................    11543   0     T53
.........................................................ugucauggauuugcucucCuu..................................    3       1     T53
.........................................................ugucauggauuugcucCcuuu..................................    3       1     T53
.........................................................ugucUuggauuugcucucuuu..................................    1       1     T53
.........................................................uCucauggauuugcucucuuu..................................    3       1     T53
.........................................................ugucaugAauuugcucucuuu..................................    3       1     T53
.........................................................Agucauggauuugcucucuuu..................................    18      1     T53
.........................................................ugucauggauuugGucucuuu..................................    2       1     T53
.........................................................Ggucauggauuugcucucuuu..................................    3       1     T53
.........................................................ugucauggauuugcucucuuA..................................    20      1     T53
.........................................................uguAauggauuugcucucuuu..................................    1       1     T53
.........................................................ugucauggauuugUucucuuu..................................    5       1     T53
.........................................................ugucauggauuuUcucucuuu..................................    2       1     T53
.........................................................uAucauggauuugcucucuuu..................................    1       1     T53
.........................................................ugucauggUuuugcucucuuu..................................    9       1     T53
.........................................................ugucauggCuuugcucucuuu..................................    1       1     T53
.........................................................ugucauggaGuugcucucuuua.................................    2       1     T53
.........................................................ugucauggauAugcucucuuua.................................    1       1     T53
.........................................................ugucauggauuugcucucCuua.................................    1       1     T53
.........................................................ugucaugCauuugcucucuuua.................................    1       1     T53
.........................................................ugucauggauuugcucuAuuua.................................    1       1     T53
.........................................................Ggucauggauuugcucucuuua.................................    4       1     T53
.........................................................ugucauggauuugcAcucuuua.................................    1       1     T53
.........................................................ugucauggGuuugcucucuuua.................................    1       1     T53
.........................................................ugucauAgauuugcucucuuua.................................    1       1     T53
.........................................................ugucauggauuugcucucuuuU.................................    860     1     T53
.........................................................ugucauggCuuugcucucuuua.................................    1       1     T53
.........................................................ugAcauggauuugcucucuuua.................................    1       1     T53
.........................................................ugucauggaCuugcucucuuua.................................    5       1     T53
.........................................................ugucaugAauuugcucucuuua.................................    6       1     T53
.........................................................uAucauggauuugcucucuuua.................................    1       1     T53
.........................................................ugucauggauuugcucucuuGa.................................    3       1     T53
.........................................................ugucauggauuugcucucuuuC.................................    22      1     T53
.........................................................uguGauggauuugcucucuuua.................................    1       1     T53
.........................................................ugucauggauuugcucucuGua.................................    1       1     T53
.........................................................uCucauggauuugcucucuuua.................................    4       1     T53
.........................................................ugucauggauuugcucucuuuG.................................    7       1     T53
.........................................................ugucaCggauuugcucucuuua.................................    2       1     T53
.........................................................ugucauggauuugcucCcuuua.................................    5       1     T53
.........................................................ugCcauggauuugcucucuuua.................................    1       1     T53
```

Mature Star

```
uucaauggucaguagcuauaaagagagcuauccgugaacaguacuguuagaggagacugucauggauuugcucucuuuauuacacuggucaaaaucaucagguagcaacuac
.........................................................ugucauggauuugcuAucuuua.................................	1	1	T53
.........................................................ugucauggauuugcucuUuuua.................................	24	1	T53
.........................................................ugucauggauuugcucGcuuua.................................	1	1	T53
.........................................................ugucaGggauuugcucucuuua.................................	2	1	T53
.........................................................ugucauggauuugcucucuuCa.................................	1	1	T53
.........................................................ugucaAggauuugcucucuuua.................................	1	1	T53
.........................................................ugucauggauuugcucAcuuua.................................	5	1	T53
.........................................................Agucauggauuugcucucuuua.................................	26	1	T53
.........................................................ugucauggauuuAcucucuuua.................................	28	1	T53
.........................................................ugucUuggauuugcucucuuua.................................	1	1	T53
.........................................................ugucauggaAuugcucucuuua.................................	2	1	T53
.........................................................ugucauggauuugcucucuuAa.................................	2	1	T53
.........................................................ugucauggUuuugcucucuuua.................................	8	1	T53
.........................................................ugucauggauuugcucucuuua.................................	12120	0	T53
.........................................................uguUauggauuugcucucuuua.................................	5	1	T53
.........................................................ugucauggauuuUcucucuuua.................................	1	1	T53
.........................................................ugucauggauuugcuUucuuua.................................	3	1	T53
.........................................................ugucaugUauuugcucucuuua.................................	3	1	T53
.........................................................Cgucauggauuugcucucuuua.................................	4	1	T53
.........................................................ugucauggauuugUucucuuua.................................	3	1	T53
.........................................................ugucauggauuugcucucuAua.................................	1	1	T53
.........................................................ugucauggauuugcucucuuuaA................................	100	1	T53
.........................................................ugucauggauuugcucucuuuCu................................	1	1	T53
.........................................................ugucauggauuCgcucucuuuau................................	1	1	T53
.........................................................ugucauggauuugcucucuuAau................................	1	1	T53
.........................................................ugucauggaGuugcucucuuuau................................	2	1	T53
.........................................................Ggucauggauuugcucucuuuau................................	1	1	T53
.........................................................Agucauggauuugcucucuuuau................................	1	1	T53
.........................................................ugucauggauuugcucuGuuuau................................	1	1	T53
.........................................................ugucauggauuugcCcucuuuau................................	1	1	T53
.........................................................ugucauggauuugcucucuuuUu................................	55	1	T53
.........................................................ugucauggUuuugcucucuuuau................................	1	1	T53
.........................................................ugucauggauuugcucucuuuaC................................	94	1	T53
.........................................................ugucauggauuugcucucuuuau................................	413	0	T53
.........................................................ugucaCggauuugcucucuuuau................................	1	1	T53
.........................................................ugucauggauuugcucucuuuaG................................	7	1	T53
.........................................................ugucauggauuugcucucuuuauA...............................	20	1	T53
.........................................................ugucauggauuugcucucuuuaCu...............................	3	1	T53
.........................................................ugucauggauuugcucucuuuaAu...............................	17	1	T53
.........................................................ugucauggauuugcucucuuuUuu...............................	3	1	T53
.........................................................ugucauggauuugcucucuuuauu...............................	52	0	T53
.........................................................ugucauggauuugcucucuuuauG...............................	4	1	T53
.........................................................ugucauggauuugcucucuuuaAua..............................	2	1	T53
.........................................................ugucauggauuugcucucuuuauuU..............................	4	1	T53
.........................................................ugucauggauuugcucucuuuauua..............................	1	0	T53
.........................................................ugucauggauuugcucucuuuauAa..............................	1	1	T53
..........................................................gucauggauuugcucucuu...................................	4	0	T53
..........................................................Uucauggauuugcucucuuu..................................	2	1	T53
..........................................................gucauggauuugcAcucuuu..................................	1	1	T53
..........................................................gucauggauuugcucucuuu..................................	17	0	T53
..........................................................gucauggauuugcucucuuua.................................	15	0	T53
..........................................................gucauggauuugcucucuuuau................................	2	0	T53
..........................................................gucauggauuugcucucuuuauu...............................	2	0	T53
...........................................................ucauggauuugcucucuuu..................................	1	0	T53
...........................................................ucauggauuugcucucuuua.................................	4	0	T53
............................................................cauggauuugcucucuuu..................................	4	0	T53
............................................................cauggauuugcucucuuua.................................	1	0	T53
.............................................................auggauuugcucucuuua.................................	3	0	T53
...............................................................................uuacacuggucaaaaucauc.............	6	0	T53
................................................................................uacacuggucaaaaucauc.............	1	0	T53
................................................................................uacacuggucaUaaucauc.............	1	1	T53
```

Provisional ID	:	Scaffold_4228_7091
Score total	:	5084.8
Score for star read(s)	:	3.9
Score for read counts	:	5074.4
Score for mfe	:	1.8
Score for randfold	:	1.6
Score for cons. seed	:	3
Total read count	:	9965
Mature read count	:	9858
Loop read count	:	0
Star read count	:	107

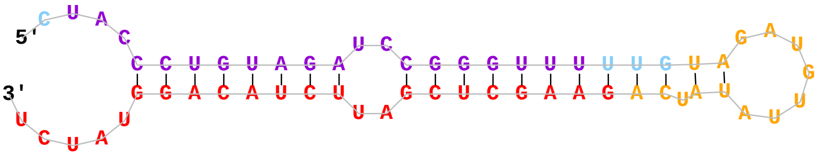

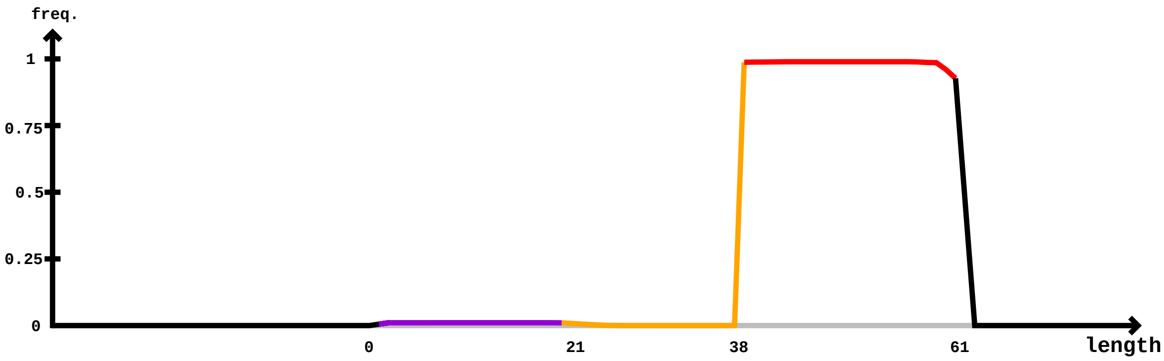


```
                                  Star                                  Mature
5'- uacgucgagcaagacgauggucauuucguaaacuacccuguagauccggguuuuuguagauguuauaucagaagcucgauucuacagguaucuuacggaggggacgccagu  -3'   obs
    uacgucgagcaagacgauggucauuucguaaacuacccuguagauccggguuuuuguagauguuauaucagaagcucgauucuacagguaucuuacggaggggacgccagu        exp
    ..((((......))))...(((.((((((((..((((.((((((..((((((((((((.......)).))))))))))..))))))))))..))))))))..))).....   reads     mm      sample
    ................................cuacccuguagauccggg.............................................................   1         0       T63
    ................................cuacccuguagauccggguu...........................................................   1         0       T63
    ................................cuaccUuguagauccggguuu..........................................................   1         1       T63
    ................................cuacccuguagauccggguuu..........................................................   5         0       T63
    ................................cuacccuguagauccggguuuu.........................................................   4         0       T63
    ................................cuacccuguagauccggguuuuu........................................................   3         0       T63
    .................................uacccuguagauccgAguuu..........................................................   1         1       T63
    .....................................................................Ugaagcucgauucuacagguaucu..................   1         1       T63
    ......................................................................gaagcucgauucuacaggu......................   2         0       T63
    ......................................................................gaagcucgauucuacagguU.....................   1         1       T63
    ......................................................................gaagcucgauucuacagguau....................   21        0       T63
    ......................................................................gaagcucgauucuacagguauc...................   55        0       T63
    ......................................................................gaUgcucgauucuacagguaucu..................   2         1       T63
    ......................................................................gaagcucgUuucuacagguaucu..................   2         1       T63
    ......................................................................gaagcucgauucuacagguaucu..................   1046      0       T63
    ......................................................................gaagcucgauuUuacagguaucu..................   1         1       T63
    ......................................................................gaagcuUgauucuacagguaucu..................   1         1       T63
    ......................................................................gaagcucgauucuacagguaucuA.................   9         1       T63
    ......................................................................gCagcucgauucuacagguaucu..................   2         1       T63
    ......................................................................gUagcucgauucuacagguaucu..................   2         1       T63
    .........................................................................gcucgauucuacagguaucu..................   2         0       T63
    ...........................................................................ucgauucuacagguaucu..................   1         0       T63

    ................................cuacccuguagauccggguuuu.........................................................   1         0       MOL
    ................................cuacccuguagauccggguuuuu........................................................   1         0       MOL
    ................................cuacccuguagauccggguuuuug.......................................................   1         0       MOL
    ................................cuacccuguagauccggguuuuugu......................................................   1         0       MOL
    .................................uacccuguagauccgAgu............................................................   1         1       MOL
    .................................uacccuguagauccgUgu............................................................   2         1       MOL
    .................................uacccuguagauccgAguu...........................................................   1         1       MOL
    .................................uacccuguagauccgAguuu..........................................................   4         1       MOL
    .................................uacccuguagauccggAuuu..........................................................   1         1       MOL
    .................................uacccuguagauccgggCuuuug.......................................................   1         1       MOL
```

```
                                 Star                                 Mature
uacgucgagcaagacgauggucauuucguaaacuacccuguagauccggguuuuuguagauguuauaucagaagcucgauucuacagguaucuuacggaggggacgccagu
.................................uacccuguagauccggguuuuug.......................................................  1     0  MOL
.................................uacccuguagauccggUuuuuug.......................................................  1     1  MOL
.....................................................................Ugaagcucgauucuacagguaucu..................  1     1  MOL
.....................................................................Ggaagcucgauucuacagguaucu..................  1     1  MOL
......................................................................gaagcucgauucuacagg.......................  1     0  MOL
......................................................................gaagcucgauucuacaggu......................  4     0  MOL
......................................................................gaagcucgauucuacaggua.....................  3     0  MOL
......................................................................gUagcucgauucuacagguau....................  1     1  MOL
......................................................................gGagcucgauucuacagguau....................  1     1  MOL
......................................................................gaagcucgauucuacagguau....................  65    0  MOL
......................................................................gaagcucgauucuacagguaA....................  1     1  MOL
......................................................................gaagcucgauucuacagguauc...................  68    0  MOL
......................................................................gaagcucgauucuacagguauA...................  1     1  MOL
......................................................................gaagcucgauucuacaggGauc...................  1     1  MOL
......................................................................gaagcucgUuucuacagguaucu..................  1     1  MOL
......................................................................gaagcucgauucuacagguGucu..................  1     1  MOL
......................................................................gaagcucgGuucuacagguaucu..................  1     1  MOL
......................................................................gUagcucgauucuacagguaucu..................  5     1  MOL
......................................................................gaagcucgauucuacagAuaucu..................  1     1  MOL
......................................................................gaaUcucgauucuacagguaucu..................  2     1  MOL
......................................................................gaagcucgaCucuacagguaucu..................  1     1  MOL
......................................................................gaagcucgauucuacagguaucu..................  1608  0  MOL
......................................................................gaagcCcgauucuacagguaucu..................  1     1  MOL
......................................................................gaagcGcgauucuacagguaucu..................  1     1  MOL
......................................................................gaagcucgauucuacagguaucC..................  1     1  MOL
......................................................................gaagcucgauucuacagCuaucu..................  2     1  MOL
......................................................................gaagcucgauuGuacagguaucu..................  1     1  MOL
......................................................................gaagcucgauucuaUagguaucu..................  1     1  MOL
......................................................................gaagcucgauucuacagguaucA..................  8     1  MOL
......................................................................gGagcucgauucuacagguaucu..................  3     1  MOL
......................................................................gaagcucgauucuacagguaucuu.................  3     0  MOL
......................................................................gaagcucgauucuacagguaucuA.................  5     1  MOL
.......................................................................aagcucgauucuacagguau....................  1     0  MOL

................................cuacccuguagauccggguuuu.........................................................  4     0  egg
................................cuacccuguagauccggguuuuu........................................................  7     0  egg
.................................uacccuguagauccggAuuu..........................................................  1     1  egg
.................................uacccuguagauccgAguuu..........................................................  2     1  egg
......................................................................gaagcucgauucuacagguau....................  3     0  egg
......................................................................gaagcucgGuucuacagguauc...................  1     1  egg
......................................................................gaagcucgauucuacagguauA...................  1     1  egg
......................................................................gaagcucgauucuacagguauc...................  21    0  egg
......................................................................gaagUucgauucuacagguauc...................  1     1  egg
......................................................................gaagUucgauucuacagguaucu..................  1     1  egg
......................................................................gaagcucgauucuacagguaucC..................  13    1  egg
......................................................................gaGgcucgauucuacagguaucu..................  1     1  egg
......................................................................gaaAcucgauucuacagguaucu..................  1     1  egg
......................................................................gGagcucgauucuacagguaucu..................  1     1  egg
......................................................................gaagcucgauucuacagguaucA..................  6     1  egg
......................................................................gaagcucgGuucuacagguaucu..................  3     1  egg
......................................................................gaagcucgauuUuacagguaucu..................  1     1  egg
......................................................................Aaagcucgauucuacagguaucu..................  1     1  egg
......................................................................gaagcucgauucuacagguaucu..................  214   0  egg
......................................................................gaagcucgauucuacaggCaucu..................  1     1  egg
......................................................................gaagcucgauucuacGgguaucu..................  2     1  egg
......................................................................gaagcucgauucuacagguCucu..................  1     1  egg
......................................................................gaagGucgauucuacagguaucu..................  2     1  egg
......................................................................gaagcucgaCucuacagguaucu..................  1     1  egg
......................................................................gUagcucgauucuacagguaucu..................  1     1  egg
......................................................................gaagcucgauucUcagguaucu...................  1     1  egg
......................................................................gaagcCcgauucuacagguaucu..................  2     1  egg
......................................................................Naagcucgauucuacagguaucu..................  1     1  egg
......................................................................gaagcucgauucuacagguaucuu.................  9     0  egg
......................................................................gaagcucgauucuacagguaucuC.................  1     1  egg
......................................................................gaagUucgauucuacagguaucuu.................  1     1  egg
......................................................................gaagcucgauucuacagguaucuA.................  6     1  egg
......................................................................gaaAcucgauucuacagguaucuu.................  1     1  egg

................................cuacccuguagauccggguuuu.........................................................  3     0  T6P
................................cuacccuguagauccggguuuuu........................................................  2     0  T6P
```

```
                                 Star                                   Mature
uacgucgagcaagacgaugguucauuucguaaacuacccuguagauccggguuuuuguagauguuauaucagaagcucgauucuacagguaucuuacggaggggacgccagu
................................cuacccuguagauccggguuuuug.......................................................  3     0   T6P
................................cuacccuguagauccggguuuuugu......................................................  3     0   T6P
.................................uacccuguagauccgAgu............................................................  1     1   T6P
.................................uacccuguagauccgAguuu..........................................................  3     1   T6P
.................................uacccuguagauccggCuuuu.........................................................  1     1   T6P
.................................uacccuguagauccggguuuuug.......................................................  1     0   T6P
.................................uacccuguagauccgggCuuuug.......................................................  2     1   T6P
......................................................................gaagcucgauucuacagg.......................  1     0   T6P
......................................................................gaagcucgauucuacaggu......................  7     0   T6P
......................................................................gaagcucgauucuacaggua.....................  1     0   T6P
......................................................................gaagcucgauucuacagguau....................  75    0   T6P
......................................................................gGagcucgauucuacagguau....................  1     1   T6P
......................................................................gaagcucgauucuacagguauc...................  67    0   T6P
......................................................................gaagcuGgauucuacagguauc...................  1     1   T6P
......................................................................gaaCcucgauucuacagguaucu..................  1     1   T6P
......................................................................Caagcucgauucuacagguaucu..................  1     1   T6P
......................................................................gaagcucgauucuacagAuaucu..................  2     1   T6P
......................................................................gaagcucgGuucuacagguaucu..................  1     1   T6P
......................................................................gaagcucgauucuacagguauUu..................  1     1   T6P
......................................................................Uaagcucgauucuacagguaucu..................  3     1   T6P
......................................................................gaagUucgauucuacagguaucu..................  1     1   T6P
......................................................................gCagcucgauucuacagguaucu..................  3     1   T6P
......................................................................gaagcucgauucuacagUuaucu..................  2     1   T6P
......................................................................gaagcucgauucuacGgguaucu..................  3     1   T6P
......................................................................gaagcucgauucuacagguaucu..................  3389  0   T6P
......................................................................gaCgcucgauucuacagguaucu..................  2     1   T6P
......................................................................gaagcucgUuucuacagguaucu..................  1     1   T6P
......................................................................gaagcucgauucuacaggCaucu..................  1     1   T6P
......................................................................gaagcuUgauucuacagguaucu..................  1     1   T6P
......................................................................gaagcucgauucuacaUguaucu..................  2     1   T6P
......................................................................gaagcucgauucuaUagguaucu..................  2     1   T6P
......................................................................gaagcucgauucuacagguauAu..................  1     1   T6P
......................................................................gaagcuGgauucuacagguaucu..................  2     1   T6P
......................................................................gaagcucgauucuacagguaucA..................  11    1   T6P
......................................................................gUagcucgauucuacagguaucu..................  4     1   T6P
......................................................................gaagcucgauuUuacagguaucu..................  1     1   T6P
......................................................................gaaUcucgauucuacagguaucu..................  3     1   T6P
......................................................................gaagcucgauucuacagCuaucu..................  1     1   T6P
......................................................................gaagcucgauucuacagguaucuu.................  1     0   T6P
......................................................................gaagcucgauucuacagguaucuA.................  2     1   T6P
.......................................................................aagcucgauucuacagguaucu..................  7     0   T6P
........................................................................agcucgauucuacagguauc...................  1     0   T6P
.........................................................................gcucgauucuacagguaucu..................  2     0   T6P
..........................................................................cucgauucuacagguaucu..................  3     0   T6P

................................cuacccuguagauccggguuuuu........................................................  1     0   te1
................................cuacccuguagauccggguuuuug.......................................................  2     0   te1
................................cuacccuguagauccggguuuuugu......................................................  1     0   te1
.................................uacccuguagauccgAguuu..........................................................  1     1   te1
......................................................................gaagcucgauucuacagg.......................  1     0   te1
......................................................................gaagcucgauucuacaggu......................  1     0   te1
......................................................................gaagcucgauucuacaggua.....................  2     0   te1
......................................................................gaagcucgauucuacagguau....................  36    0   te1
......................................................................gaagcucgauucuacagguauc...................  24    0   te1
......................................................................gGagcucgauucuacagguaucu..................  1     1   te1
......................................................................gaagcucgauucuacagguaucA..................  4     1   te1
......................................................................gaagUucgauucuacagguaucu..................  1     1   te1
......................................................................gaagcucgauucuacaggGaucu..................  1     1   te1
......................................................................gaagcucgauucuacagguaucu..................  618   0   te1
......................................................................gaagcucgauucuacagguaucG..................  2     1   te1
......................................................................gaagcucgGuucuacagguaucu..................  1     1   te1
......................................................................gaagcucgauucuacagguaucuu.................  3     0   te1
..........................................................................cucgauucuacagguaucA..................  1     1   te1

................................cuacccuguagauccggguu...........................................................  1     0   T53
................................cuacccuguagauccggguuu..........................................................  3     0   T53
................................cuacccuguagauccggguuuu.........................................................  2     0   T53
................................cuacccuguagauccggguuuuA........................................................  1     1   T53
................................cuacccuguagauccggguuuuu........................................................  3     0   T53
.................................uacccuguagauccggAuu...........................................................  1     1   T53
```

Star **Mature**

```
uacgucgagcaagacgauggucauuucguaaacuacccuguagauccggguuuuuguagauguuauaucagaagcucgauucuacagguaucuuacggaggggacgccagu
.................................uacccuguagauccgAguu...........................................................    2     1   T53
.................................uacccuguagauccgAguuu..........................................................    16    1   T53
.................................uacccuguagauccggAuuu..........................................................    5     1   T53
.................................uacccuguagauccggguuuuu........................................................    1     0   T53
.................................uacccuguagauccggguuuuuUu......................................................    1     1   T53
......................................uguagauccggguuuuuguagau..................................................    1     0   T53
.....................................................................Ugaagcucgauucuacagguaucu..................    1     1   T53
......................................................................gaagcucgauucuacagA.......................    2     1   T53
......................................................................gaagcucgauucuacagg.......................    2     0   T53
......................................................................gaagcucgauucuacaggu......................    6     0   T53
......................................................................gaagcucgauucuacaggua.....................    1     0   T53
......................................................................gaagcucgauucuacagguau....................    50    0   T53
......................................................................gaagcucgauucuacagguaA....................    1     1   T53
......................................................................gaagcucgauucuacagguauc...................    84    0   T53
......................................................................gaagcucgUuucuacagguauc...................    1     1   T53
......................................................................gaagcucgauucuacagguauUu..................    1     1   T53
......................................................................gaagcucUauucuacagguaucu..................    1     1   T53
......................................................................gaagcucgaCucuacagguaucu..................    1     1   T53
......................................................................gGagcucgauucuacagguaucu..................    1     1   T53
......................................................................gaaAcucgauucuacagguaucu..................    1     1   T53
......................................................................gaagcucgauucuacagAuaucu..................    1     1   T53
......................................................................gCagcucgauucuacagguaucu..................    1     1   T53
......................................................................gaagcucgauuUuacagguaucu..................    2     1   T53
......................................................................gaagcucgauucuacagguaucA..................    13    1   T53
......................................................................gaagcucgauucuacaAguaucu..................    1     1   T53
......................................................................gaagcucgUuucuacagguaucu..................    1     1   T53
......................................................................gaagcuUgauucuacagguaucu..................    2     1   T53
......................................................................gaagcucgauucuacaggAaucu..................    1     1   T53
......................................................................Caagcucgauucuacagguaucu..................    1     1   T53
......................................................................gaUgcucgauucuacagguaucu..................    2     1   T53
......................................................................gaagcucgauucuacagguaucu..................    2128  0   T53
......................................................................gaagcGcgauucuacagguaucu..................    1     1   T53
......................................................................gaagcucgauucuacagguaucuA.................    1     1   T53
.......................................................................aagcucgauucuacagguaucu..................    1     0   T53
........................................................................agcucgauucuacagguaucu..................    1     0   T53
..........................................................................cucgauucuacagguauc...................    1     0   T53
...........................................................................ucgauucuacagguaucu..................    1     0   T53
```

Provisional ID	:	Scaffold_4228_7678
Score total	:	36362.9
Score for star read(s)	:	3.9
Score for read counts	:	36353.2
Score for mfe	:	1.2
Score for randfold	:	1.6
Score for cons. seed	:	3
Total read count	:	71317
Mature read count	:	71097
Loop read count	:	0
Star read count	:	220

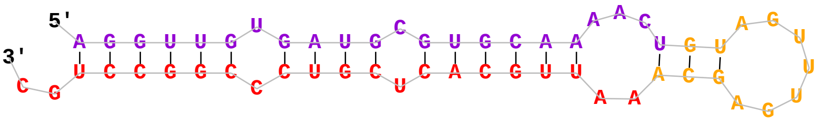

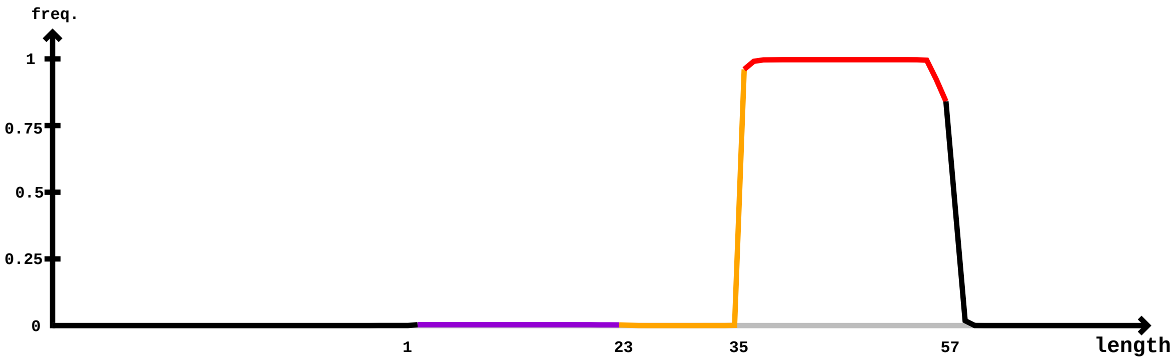

Star Mature

```
5'- ucuauaguucaacucauaauuguuugcagaagugucagguugugaugcgugcaaaacuguaguuugagcaaauugcacucgucccggccugcucacucugacuguacaaccg   -3'   obs
    ucuauaguucaacucauaauuguuugcagaagugucagguugugaugcgugcaaaacuguaguuugagcaaauugcacucgucccggccugcucacucugacuguacaaccg         exp
    ...................((((..(((((.(((.(((((((.((((.((((((...(((.......)))..)))))).)))).)))))))..))))))).)...))))...     reads   mm   sample
    ................................ugucagguugugaugcgugc............................................................     3       0    T53
    ................................ugucagguugugaugcgugca...........................................................     2       0    T53
    ....................................agguugugaugcgugcaaa.........................................................     7       0    T53
    ....................................agguugugaugcgugcaaaac.......................................................     3       0    T53
    ....................................agguugugaugcgugcaaaacA......................................................     2       1    T53
    ....................................agguugugaugcgugcaaaacu......................................................     24      0    T53
    ....................................agguugugaugcgugcaaaacug.....................................................     6       0    T53
    ....................................agguugugaugcgugcaaUacug.....................................................     1       1    T53
    .....................................................................aaauugcacucgucccggccugc....................     1       0    T53
    ......................................................................aauugcacucgucccggc........................     1       0    T53
    ......................................................................aauugcacucgucccggU........................     1       1    T53
    ......................................................................aauugcacucgucccggcc.......................     8       0    T53
    ......................................................................aauugcacucgucccggcA.......................     2       1    T53
    ......................................................................Gauugcacucgucccggccu......................     1       1    T53
    ......................................................................aauugcacucgucUcggccu......................     3       1    T53
    ......................................................................aauugcacucgucccggccC......................     1       1    T53
    ......................................................................aauugcacucgCcccggccu......................     1       1    T53
    ......................................................................aauugcacucgucccggGcu......................     2       1    T53
    ......................................................................aauugcacAcgucccggccu......................     1       1    T53
    ......................................................................aauugcacucgucccggccu......................     346     0    T53
    ......................................................................aauugcacucguccUggccu......................     1       1    T53
    ......................................................................aauugcacucguUccggccu......................     2       1    T53
    ......................................................................aauugcacuAgucccggccu......................     3       1    T53
    ......................................................................aauugUacucgucccggccug.....................     1       1    T53
    ......................................................................aauugcacucgucccggccCg.....................     1       1    T53
    ......................................................................Cauugcacucgucccggccug.....................     1       1    T53
    ......................................................................aUuugcacucgucccggccug.....................     1       1    T53
    ......................................................................aauugcacucgucccggccug.....................     142     0    T53
    ......................................................................aauugcacucgucccgAccug.....................     1       1    T53
    ......................................................................aauugcacucguUccggccug.....................     1       1    T53
    ......................................................................aauugcacucgucccggccuC.....................     1       1    T53
    ......................................................................aauugcacucgucccggccuA.....................     4       1    T53
    ......................................................................aauuAcacucgucccggccug.....................     1       1    T53
```

```
                                    Star                              Mature
ucuauaguucaacucauaauuguuugcagaagugucagguugugaugcgugcaaaacuguaguuugagcaaauugcacucgucccggccugcucacucugacuguacaaccg
......................................................................aauugcacucgucccggccuU.....................  15   1  T53
......................................................................aauugcacucgucUcggccug.....................  2    1  T53
......................................................................aauugcacucgucccggccuCc....................  1    1  T53
......................................................................aauugcacucgucccCgccugc....................  1    1  T53
......................................................................aauugcacucgucUcggccugc....................  1    1  T53
......................................................................aauugcacucguUccggccugc....................  3    1  T53
......................................................................aauugcacucgucccggccAgc....................  1    1  T53
......................................................................aauugcacuAgucccggccugc....................  5    1  T53
......................................................................aauugcacucgucccggccuUc....................  2    1  T53
......................................................................aCuugcacucgucccggccugc....................  1    1  T53
......................................................................aauugcacucgucccggccugc....................  887  0  T53
......................................................................aauugcacucgucccggAcugc....................  1    1  T53
......................................................................aauugcacucgucccggccugA....................  5    1  T53
......................................................................aauugcacucgucccggccuAc....................  3    1  T53
......................................................................aauugcacucgucccggccugU....................  145  1  T53
......................................................................aauugcacucgucccgAccugc....................  1    1  T53
......................................................................aauugcacucgucccggcAugc....................  1    1  T53
......................................................................aUuugcacucgucccggccugc....................  3    1  T53
......................................................................aauAgcacucgucccggccugc....................  1    1  T53
......................................................................aauugcacuGgucccggccugc....................  9    1  T53
......................................................................aauugcacucgGcccggccugc....................  1    1  T53
......................................................................aauugcGcucgucccggccugc....................  1    1  T53
......................................................................aauugcacucgucccggccugUu...................  66   1  T53
......................................................................aauugcacucgucccggccugcu...................  118  0  T53
......................................................................aauugcacucguccUggccugcu...................  1    1  T53
......................................................................aauugcacucgucccggccugcC...................  1    1  T53
......................................................................aauugcacucgucccggccugcA...................  73   1  T53
......................................................................aauugcacucgGcccggccugcu...................  1    1  T53
......................................................................aauugcacucgucccgAccugcu...................  1    1  T53
......................................................................Uauugcacucgucccggccugcu...................  248  1  T53
......................................................................aauugcacucgucccggccugcuU..................  29   1  T53
......................................................................Uauugcacucgucccggccugcuc..................  1    1  T53
......................................................................aauugcacucgucccggccugcuG..................  1    1  T53
......................................................................aauugcacucgucccggccugcuA..................  1    1  T53
.......................................................................auugcacucgucccggccug.....................  1    0  T53
.......................................................................auugcacuUgucccggccug.....................  2    1  T53
.......................................................................auugcacucguccGggccugc....................  1    1  T53
.......................................................................auugUacucgucccggccugc....................  1    1  T53
.......................................................................auugcacucgucccggccugA....................  1    1  T53
.......................................................................auugcacucgucccggccuUc....................  1    1  T53
.......................................................................auugcacucgucccggccugU....................  1    1  T53
.......................................................................auugcacucgucccggccugc....................  102  0  T53
.......................................................................auugcacucgucccggccugcu...................  4    0  T53
.......................................................................auugcacucgucccggccugcC...................  1    1  T53
........................................................................uugcacucgucccggccugc....................  5    0  T53
........................................................................uugcacucgucccggccugcC...................  4    1  T53
........................................................................uugcacucgucccggccugcu...................  9    0  T53
........................................................................uugcacucgucccggccugcuU..................  4    1  T53
........................................................................uugcacucgucccggccugcuc..................  1    0  T53
........................................................................uugcacucgucccggccugcuA..................  3    1  T53
.........................................................................ugcacucgucccggccugc....................  1    0  T53

....................................agguugugaugcgugcaaa.........................................................  2    0  te1
....................................agguugugaugcgugcaaaacu......................................................  7    0  te1
....................................agguugugaugcgugcaaaacug.....................................................  3    0  te1
.....................................................................Uaauugcacucgucccggccugc....................  2    1  te1
.....................................................................aaauugcacucgucccggccugc....................  1    0  te1
......................................................................aauugcacucgucccggc........................  1    0  te1
......................................................................aauugcacucgucccggcA.......................  1    1  te1
......................................................................aauugcacucgucccggcc.......................  3    0  te1
......................................................................aauugcacucgucccggccG......................  5    1  te1
......................................................................aauugcacuUgucccggccu......................  1    1  te1
......................................................................aauugUacucgucccggccu......................  1    1  te1
......................................................................aauugcacucAucccggccu......................  2    1  te1
......................................................................aauugcacucgucGcggccu......................  1    1  te1
......................................................................aauugcacucgucccggUcu......................  1    1  te1
......................................................................aauugcacucgucccggccu......................  745  0  te1
......................................................................aauugcacucguUccggccu......................  6    1  te1
......................................................................aauugcacucgucccUgccu......................  1    1  te1
......................................................................aauugcacucgucccggccA......................  4    1  te1
```

Star Mature

ucuauaguucaacucauaauuguuugcagaaguguc**agguugugaugcgugcaaaacu**guaguuugagca**aauugcacucgucccggccugc**ucacucugacuguacaaccg

....Gauugcacucgucccggccu......................	1	1	te1
....aGuugcacucgucccggccu......................	2	1	te1
....aauugcacucgCcccggccu......................	1	1	te1
....aauugcacucgAcccggccu......................	1	1	te1
....aauugcacucguccUggccu......................	3	1	te1
....aauugcacucgucUcggccu......................	7	1	te1
....aauugcacucguccUggccug.....................	1	1	te1
....aauugcacucgucccggccuC.....................	3	1	te1
....aauugcacuUgucccggccug.....................	1	1	te1
....aauugcacucgucccgAccug.....................	1	1	te1
....aauuUcacucgucccggccug.....................	1	1	te1
....aauugcacucgucccggccuA.....................	10	1	te1
....aCuugcacucgucccggccug.....................	1	1	te1
....aauugcacucgucccggccGg.....................	58	1	te1
....aauugcacucguUccggccug.....................	2	1	te1
....aauugcacucgucccggccAg.....................	1	1	te1
....aUuugcacucgucccggccug.....................	1	1	te1
....aauugcacucgucccggccuU.....................	61	1	te1
....aauugcacucgucccggccug.....................	601	0	te1
....aauugcacucgucccgAccugc....................	1	1	te1
....aauugcacuUgucccggccugc....................	1	1	te1
....aauugcacucgucccggccCgc....................	2	1	te1
....aauugcacucgucccggccGgc....................	2	1	te1
....aauuAcacucgucccggccugc....................	1	1	te1
....aauGgcacucgucccggccugc....................	1	1	te1
....aGuugcacucgucccggccugc....................	5	1	te1
....aauugcacucgucccggccuAc....................	2	1	te1
....aaGugcacucgucccggccugc....................	1	1	te1
....aauugcacucgucccggccugG....................	6	1	te1
....aauugcacucgucccggccuUc....................	4	1	te1
....aauugcacucgCcccggccugc....................	1	1	te1
....aCuugcacucgucccggccugc....................	1	1	te1
....aauugcacucgucccggcUugc....................	2	1	te1
....aauugcacucgucccggccugA....................	13	1	te1
....aauugcacucguccUggccugc....................	2	1	te1
....aauugcacucUucccggccugc....................	1	1	te1
....aauugcacucgucccgUccugc....................	1	1	te1
....aauugcacucgucccggccugc....................	2372	0	te1
....aauugcacucgucccggccugU....................	210	1	te1
....Cauugcacucgucccggccugc....................	5	1	te1
....aauugcacucgucUcggccugc....................	3	1	te1
....Gauugcacucgucccggccugc....................	1	1	te1
....aauugcacucgucccggUcugc....................	3	1	te1
....aaCugcacucgucccggccugc....................	1	1	te1
....aauugcacucguUccggccugc....................	4	1	te1
....aauugUacucgucccggccugc....................	2	1	te1
....aauugcacucgucccggccugUu...................	72	1	te1
....aauugcacucgGcccggccugcu...................	1	1	te1
....aauugcacucgucccggccugcC...................	3	1	te1
....Uauugcacucgucccggccugcu...................	267	1	te1
....aauuCcacucgucccggccugcu...................	1	1	te1
....aauugcacucgucccggccugcu...................	255	0	te1
....aauugcacucgucccggccugAu...................	4	1	te1
....Gauugcacucgucccggccugcu...................	1	1	te1
....aauugcacucguccUggccugcu...................	1	1	te1
....aauugcacucgucccggccugcA...................	153	1	te1
....aauugcacuUgucccggccugcu...................	1	1	te1
....aauugcacucgucccggccugcG...................	11	1	te1
....Uauugcacucgucccggccugcuc..................	1	1	te1
....aauugcacucgucccggccugcuA..................	4	1	te1
....aauugcacucgucccggccugcuU..................	100	1	te1
....aauugcacucgucccggccugcuG..................	1	1	te1
....aauugcacucgucccggccugcucU.................	1	1	te1
....auugcacucgucccggccu......................	5	0	te1
....auugcacucgucccggccug.....................	20	0	te1
....auugcacucgucccggccuU.....................	1	1	te1
....auugcacucgucccggccugc....................	623	0	te1
....auugcacucgucccggccugU....................	3	1	te1
....auugcacucgucccCgccugc....................	1	1	te1
....auugcacucgucccggccGgc....................	1	1	te1

Star **Mature**

ucuauaguucaacucauaauuguuugcagaagugucagguugugaugcgugcaaaacuguaguuugagcaaauugcacucgucccggccugcucacucugacuguacaaccg			
....Uuugcacucgucccggccugc....................	1	1	te1
....aAugcacucgucccggccugc....................	2	1	te1
....auugcacucgucccgAccugc....................	1	1	te1
....Guugcacucgucccggccugc....................	1	1	te1
....auugcacucgucUcggccugc....................	1	1	te1
....auugcacucCucccggccugc....................	1	1	te1
....auuAcacucgucccggccugc....................	1	1	te1
....auugcacucgucccAgccugc....................	1	1	te1
....auugcacucgucccggccugcu...................	13	0	te1
....auugcacucgucccggccugcC...................	2	1	te1
....auugcacucgucccggccugUu...................	1	1	te1
....auugcacucgucccggccugcuU..................	3	1	te1
....uugcacucgucccggccugc....................	16	0	te1
....uugcacucgucccggccugcu...................	19	0	te1
....uugcacucgucccggccugcuA..................	2	1	te1
....uugcacucgucccggccugcuU..................	9	1	te1
....ugcacucgucccggccugc....................	3	0	te1
....ugcacucgucccggccugUu...................	1	1	te1
....gcacucgucccggccugU....................	1	1	te1
....gcacucgucccggccugc....................	4	0	te1
....gcacucgucccggccugcu...................	1	0	te1
....gcacucgucccggccugUu...................	1	1	te1
....................................agguugugaugcgugcaaaacu..	3	0	egg
....................................agguCgugaugcgugcaaaacug...	1	1	egg
....................................agguugugaugcgugcaaaacug...	34	0	egg
....................................agguugugaugcgugcaaaacugu..	3	0	egg
....aaauugcacucgucccggccugc....................	1	0	egg
....Caauugcacucgucccggccugc....................	10	1	egg
....aUauugcacucgucccggccugcu...................	2	1	egg
....aaauugcacucgucccggccugcu...................	1	0	egg
....Nauugcacucgucccggc........................	1	1	egg
....aauugcacucgucccggc........................	4	0	egg
....aauugcacucgucccggcA.......................	2	1	egg
....aauuCcacucgucccggcc.......................	1	1	egg
....aUuugcacucgucccggcc.......................	1	1	egg
....aauGgcacucgucccggcc.......................	1	1	egg
....aauugcacucgucccggcc.......................	10	0	egg
....aauugcacucgucccggccG......................	1	1	egg
....aauCgcacucgucccggccu......................	2	1	egg
....aauugcacucgucccgAccu......................	1	1	egg
....aauAgcacucgucccggccu......................	1	1	egg
....aauugcacucgucccggccA......................	2	1	egg
....aauugUacucgucccggccu......................	1	1	egg
....aauuUcacucgucccggccu......................	3	1	egg
....Gauugcacucgucccggccu......................	3	1	egg
....aauugcUcucgucccggccu......................	2	1	egg
....aauugcacucgucccggccC......................	26	1	egg
....aauugcacucguccUggccu......................	1	1	egg
....aauugcacucgucccggccu......................	270	0	egg
....aauGgcacucgucccggccu......................	3	1	egg
....aGuugcacucgucccggccu......................	1	1	egg
....aauugcacucAucccggccug.....................	1	1	egg
....aauugcacucgucccggccuC.....................	2	1	egg
....aauugcacucgucccAgccug.....................	3	1	egg
....aauugcacucgucccggccCg.....................	9	1	egg
....aauugcacucgCcccggccug.....................	4	1	egg
....aauugUacucgucccggccug.....................	1	1	egg
....aauugcacucgucccggccuU.....................	10	1	egg
....aauugcacuUgucccggccug.....................	2	1	egg
....Nauugcacucgucccggccug.....................	5	1	egg
....aauugcacucgucUcggccug.....................	3	1	egg
....aauugcacucguccUggccug.....................	1	1	egg
....aauugcacucgucccggccGg.....................	1	1	egg
....aauugcGcucgucccggccug.....................	3	1	egg
....aauAgcacucgucccggccug.....................	4	1	egg
....aauGgcacucgucccggccug.....................	15	1	egg
....aauugcacAcgucccggccug.....................	1	1	egg
....aauugcacucguUccggccug.....................	1	1	egg
....aauugcacCcgucccggccug.....................	6	1	egg

Star **Mature**

```
ucuauaguucaacucauaauuguuugcagaagugucagguugugaugcgugcaaaacuguaguuugagcaaauugcacucgucccggccugcucacucugacuguacaaccg
......................................................................aauugcacuGgucccggccug.....................   1      1   egg
......................................................................aauCgcacucgucccggccug.....................   11     1   egg
......................................................................aGuugcacucgucccggccug.....................   3      1   egg
......................................................................aauugcacucgucccgAccug.....................   1      1   egg
......................................................................aaCugcacucgucccggccug.....................   5      1   egg
......................................................................aauugcaUucgucccggccug.....................   2      1   egg
......................................................................aauugcacucgucAcggccug.....................   1      1   egg
......................................................................aauugcacucgucccggUcug.....................   1      1   egg
......................................................................Cauugcacucgucccggccug.....................   5      1   egg
......................................................................aauuAcacucgucccggccug.....................   3      1   egg
......................................................................aauugcacucguccGggccug.....................   1      1   egg
......................................................................aauuCcacucgucccggccug.....................   2      1   egg
......................................................................aauugcUcucgucccggccug.....................   1      1   egg
......................................................................aauugcacucgucccggcUug.....................   1      1   egg
......................................................................Gauugcacucgucccggccug.....................   5      1   egg
......................................................................aauugcacucgucccCgccug.....................   1      1   egg
......................................................................aauugcacucgucccggccug.....................   1031   0   egg
......................................................................aauugAacucgucccggccug.....................   1      1   egg
......................................................................aCuugcacucgucccggccug.....................   3      1   egg
......................................................................aauuUcacucgucccggccug.....................   41     1   egg
......................................................................aauugcacucgucccggccuA.....................   47     1   egg
......................................................................aUuugcacucgucccggccug.....................   1      1   egg
......................................................................aauugcaUucgucccggccugc....................   16     1   egg
......................................................................aauugcacucgGcccggccugc....................   10     1   egg
......................................................................aGuugcacucgucccggccugc....................   45     1   egg
......................................................................aauugcacucgucccgCccugc....................   6      1   egg
......................................................................aauugcUcucgucccggccugc....................   12     1   egg
......................................................................aauugcacuUgucccggccugc....................   15     1   egg
......................................................................aaGugcacucgucccggccugc....................   2      1   egg
......................................................................aauugcacucgucccggcGugc....................   1      1   egg
......................................................................aauugcacucguAccggccugc....................   1      1   egg
......................................................................aauugcacucgucccggccugU....................   1002   1   egg
......................................................................aauugcacucgucccgUccugc....................   3      1   egg
......................................................................aauugcacucguccAggccugc....................   3      1   egg
......................................................................aauugcacucgucccggccuAc....................   21     1   egg
......................................................................aauugcacucAucccggccugc....................   17     1   egg
......................................................................aauuAcacucgucccggccugc....................   47     1   egg
......................................................................aauugcacucgucccggcUugc....................   12     1   egg
......................................................................aauugcacucguUccggccugc....................   10     1   egg
......................................................................aauugcacucgucccggccGgc....................   2      1   egg
......................................................................aauGgcacucgucccggccugc....................   233    1   egg
......................................................................aauugcacuAgucccggccugc....................   4      1   egg
......................................................................aauugcacucgAcccggccugc....................   3      1   egg
......................................................................aauugcacucgucccggcAugc....................   1      1   egg
......................................................................aauugcacGcgucccggccugc....................   1      1   egg
......................................................................aauuUcacucgucccggccugc....................   451    1   egg
......................................................................aauugcacucgucccggccCgc....................   54     1   egg
......................................................................aauugcacucguccGggccugc....................   3      1   egg
......................................................................aauugUacucgucccggccugc....................   21     1   egg
......................................................................aauugcacucgucccggccugc....................   12528  0   egg
......................................................................aauugcacucgucccAgccugc....................   21     1   egg
......................................................................aauugcacucgucccggccugG....................   5      1   egg
......................................................................aauugcacucguGccggccugc....................   1      1   egg
......................................................................aauugcacucgCcccggccugc....................   39     1   egg
......................................................................aauugcacucgucUcggccugc....................   13     1   egg
......................................................................Cauugcacucgucccggccugc....................   73     1   egg
......................................................................aauCgcacucgucccggccugc....................   113    1   egg
......................................................................aauugcacAcgucccggccugc....................   7      1   egg
......................................................................aauugAacucgucccggccugc....................   15     1   egg
......................................................................aauugcacucgucccUgccugc....................   4      1   egg
......................................................................aauugcacucgucccggUcugc....................   21     1   egg
......................................................................aauugcaAucgucccggccugc....................   1      1   egg
......................................................................aauugcacucgucAcggccugc....................   1      1   egg
......................................................................aauugcacCcgucccggccugc....................   57     1   egg
......................................................................aauugcGcucgucccggccugc....................   50     1   egg
......................................................................aauugcacucUucccggccugc....................   4      1   egg
......................................................................aaAugcacucgucccggccugc....................   5      1   egg
......................................................................Gauugcacucgucccggccugc....................   48     1   egg
......................................................................aauugcCcucgucccggccugc....................   5      1   egg
......................................................................aauugcacuGgucccggccugc....................   3      1   egg
```

Star **Mature**

```
ucuauaguucaacucauaauuguuugcagaagugucagguugugaugcgugcaaaacuguaguuugagcaaauugcacucgucccggccugcucacucugacuguacaaccg
......................................................................aauuCcacucgucccggccugc....................   41     1   egg
......................................................................aauugcacucgucGcggccugc....................   1      1   egg
......................................................................aaCugcacucgucccggccugc....................   38     1   egg
......................................................................aauugcacucgucccggccugA....................   294    1   egg
......................................................................aauugcacucgucccggGcugc....................   3      1   egg
......................................................................aauugcacucgucccggccuUc....................   8      1   egg
......................................................................Nauugcacucgucccggccugc....................   66     1   egg
......................................................................aauugcacucgucccggccuCc....................   2      1   egg
......................................................................aauugcacucgucccggccAgc....................   10     1   egg
......................................................................aCuugcacucgucccggccugc....................   14     1   egg
......................................................................aauugcacucCucccggccugc....................   1      1   egg
......................................................................aauugcacucgucccgAccugc....................   14     1   egg
......................................................................aauAgcacucgucccggccugc....................   34     1   egg
......................................................................aauugcacucgucccCgccugc....................   2      1   egg
......................................................................aauugcaGucgucccggccugc....................   1      1   egg
......................................................................aUuugcacucgucccggccugc....................   7      1   egg
......................................................................aauugcacucguccUggccugc....................   24     1   egg
......................................................................aauugcacucgucccCgccugcu...................   2      1   egg
......................................................................aauugcacucgucccggccugcu...................   1863   0   egg
......................................................................aauugcacucgucccggUcugcu...................   2      1   egg
......................................................................aaGugcacucgucccggccugcu...................   1      1   egg
......................................................................aauugcacucgAcccggccugcu...................   1      1   egg
......................................................................aauugcacGcgucccggccugcu...................   1      1   egg
......................................................................aauugUacucgucccggccugcu...................   7      1   egg
......................................................................aauugcacucgucccggccugcG...................   20     1   egg
......................................................................aGuugcacucgucccggccugcu...................   4      1   egg
......................................................................aauugcacucguccGggccugcu...................   1      1   egg
......................................................................aauugcacucgCcccggccugcu...................   5      1   egg
......................................................................aauugcacucguccUggccugcu...................   4      1   egg
......................................................................aauugcacucgucccggccugUu...................   133    1   egg
......................................................................aCuugcacucgucccggccugcu...................   2      1   egg
......................................................................aauugcacucCucccggccugcu...................   1      1   egg
......................................................................aauugcacucgucccAgccugcu...................   2      1   egg
......................................................................aaCugcacucgucccggccugcu...................   3      1   egg
......................................................................aauGgcacucgucccggccugcu...................   41     1   egg
......................................................................aauugcacucgucccUgccugcu...................   1      1   egg
......................................................................aauAgcacucgucccggccugcu...................   11     1   egg
......................................................................aauugcacucgucccggccCgcu...................   9      1   egg
......................................................................aauugcacucgucUcggccugcu...................   2      1   egg
......................................................................aauugcacucAucccggccugcu...................   2      1   egg
......................................................................Nauugcacucgucccggccugcu...................   3      1   egg
......................................................................aauugcGcucgucccggccugcu...................   5      1   egg
......................................................................aauugAacucgucccggccugcu...................   2      1   egg
......................................................................aauugcacucgucccggAcugcu...................   1      1   egg
......................................................................aauuCcacucgucccggccugcu...................   6      1   egg
......................................................................aUuugcacucgucccggccugcu...................   1      1   egg
......................................................................aauugcacucgucccgAccugcu...................   1      1   egg
......................................................................aauugcacucgucccggccugcC...................   121    1   egg
......................................................................Cauugcacucgucccggccugcu...................   9      1   egg
......................................................................aauugcacucgucAcggccugcu...................   1      1   egg
......................................................................aauugcacucgucccggccugcA...................   831    1   egg
......................................................................aauugcUcucgucccggccugcu...................   2      1   egg
......................................................................aauugcacuUgucccggccugcu...................   4      1   egg
......................................................................aauugcacucgucccggcUugcu...................   3      1   egg
......................................................................Uauugcacucgucccggccugcu...................   2640   1   egg
......................................................................aauuAcacucgucccggccugcu...................   7      1   egg
......................................................................Gauugcacucgucccggccugcu...................   5      1   egg
......................................................................aauugcacucgucccggccugAu...................   2      1   egg
......................................................................aauugcaUucgucccggccugcu...................   3      1   egg
......................................................................aauuUcacucgucccggccugcu...................   46     1   egg
......................................................................aauugcacCcgucccggccugcu...................   6      1   egg
......................................................................aauCgcacucgucccggccugcu...................   20     1   egg
......................................................................aauugcacucgucccggccuAcu...................   2      1   egg
......................................................................aaAugcacucgucccggccugcu...................   1      1   egg
......................................................................aauugcacucgucccgCccugcu...................   2      1   egg
......................................................................aauugcacucgucccggccugcuG..................   2      1   egg
......................................................................aauugcacucgucccggccugcAc..................   2      1   egg
......................................................................Uauugcacucgucccggccugcuc..................   16     1   egg
......................................................................aauugcacucgucccggccugcuU..................   120    1   egg
......................................................................aauugcacucgucccggccugcuA..................   24     1   egg
```

```
                                    Star                              Mature
ucuauaguucaacucauaauuguuugcagaaguguc agguugugaugcgugcaaaacu guaguuugagca aauugcacucgucccggccugc ucacucugacuguacaaccg
......................................................................aauugcacucgucccggccugcuc..................  8     0  egg
......................................................................aauugcacucgucccggccugcucU.................  7     1  egg
.......................................................................auugcacucgucccggccug.....................  1     0  egg
.......................................................................auugcacucgucccggccugU....................  4     1  egg
.......................................................................aCugcacucgucccggccugc....................  1     1  egg
.......................................................................aAugcacucgucccggccugc....................  2     1  egg
.......................................................................auugcacucgucccCgccugc....................  1     1  egg
.......................................................................auugcCcucgucccggccugc....................  2     1  egg
.......................................................................auuCcacucgucccggccugc....................  1     1  egg
.......................................................................auugcacucgucccggccuAc....................  1     1  egg
.......................................................................auugcacucgucccggccugc....................  46    0  egg
.......................................................................auugcacucgucccggccugcu...................  6     0  egg
.......................................................................auugcacucgucccggccugcC...................  4     1  egg
.......................................................................Guugcacucgucccggccugcu...................  1     1  egg
........................................................................uugUacucgucccggccugc....................  1     1  egg
........................................................................uugcacucgucccggccugc....................  6     0  egg
........................................................................uugcUcucgucccggccugc....................  1     1  egg
........................................................................uugcGcucgucccggccugcu...................  1     1  egg
........................................................................uugcacucgucccggccugcC...................  3     1  egg
........................................................................uugcacucgucccggccugcu...................  24    0  egg
........................................................................uugcacucgucccggccugcuc..................  3     0  egg
........................................................................uugcacucgucccggccugcuU..................  13    1  egg
........................................................................uugcUcucgucccggccugcuc..................  2     1  egg
........................................................................uugcacucgucccggccugcuA..................  17    1  egg

................................ugucagguugugaugcgugc............................................................  2     0  T6P
................................ugucagguugugaugcgugca...........................................................  2     0  T6P
....................................Ggguugugaugcgugcaaa.........................................................  1     1  T6P
....................................agguugugaugcgugcaaa.........................................................  5     0  T6P
....................................agguugugaugcgugcaaaa........................................................  1     0  T6P
....................................agguugugaugcgugcaaaac.......................................................  5     0  T6P
....................................agguugugaugcguAcaaaacu......................................................  1     1  T6P
....................................agguugugaugcgugcaaaacu......................................................  16    0  T6P
....................................agguugugaugcgugcaaaacA......................................................  1     1  T6P
....................................agguugugaugcgugcaaaacug.....................................................  13    0  T6P
....................................agguugugaugcgugcaaaacuU.....................................................  1     1  T6P
....................................agguugugaugcgugcaaaacugu....................................................  1     0  T6P
.....................................................................aaauugcacucgucccggccu......................  2     0  T6P
.....................................................................Uaauugcacucgucccggccugc....................  4     1  T6P
.....................................................................aaauugcacucgucccggccugc....................  3     0  T6P
.....................................................................Uaauugcacucgucccggccugcu...................  1     1  T6P
......................................................................aauugcacucgucccggcA.......................  1     1  T6P
......................................................................aauugcacucgucccggcc.......................  32    0  T6P
......................................................................aauugcacucgucccgCccu......................  2     1  T6P
......................................................................aauugcacucgucccggccA......................  3     1  T6P
......................................................................aauugcacucgucccggccu......................  1468  0  T6P
......................................................................aauugcacucgucccUgccu......................  2     1  T6P
......................................................................aauugcacucgucccggcUu......................  1     1  T6P
......................................................................aauugcacucguccUggccu......................  7     1  T6P
......................................................................aauugcacucgCcccggccu......................  1     1  T6P
......................................................................aauugcacucguUccggccu......................  9     1  T6P
......................................................................aauugcacucgucUcggccu......................  10    1  T6P
......................................................................aauugcacucCucccggccu......................  1     1  T6P
......................................................................aaCugcacucgucccggccu......................  1     1  T6P
......................................................................aauugcaUucgucccggccu......................  1     1  T6P
......................................................................aauugcacucUucccggccu......................  2     1  T6P
......................................................................aauugcacucgucccCgccu......................  1     1  T6P
......................................................................aauugcUcucgucccggccu......................  1     1  T6P
......................................................................aauugcacucgucccgAccu......................  2     1  T6P
......................................................................aauuUcacucgucccggccu......................  1     1  T6P
......................................................................aauugcacucgucccggUcu......................  1     1  T6P
......................................................................aauugcacucgucccgUccu......................  2     1  T6P
......................................................................aauugcCcucgucccggccu......................  1     1  T6P
......................................................................aauugcacucgucccggccuC.....................  3     1  T6P
......................................................................aauugcacucgucccgUccug.....................  1     1  T6P
......................................................................aauugcacucguUccggccug.....................  6     1  T6P
......................................................................aauugcacucgucccggccug.....................  1014  0  T6P
......................................................................aauugcacucgucccggUcug.....................  1     1  T6P
......................................................................aauugcacucgucccggccuU.....................  40    1  T6P
......................................................................aauuAcacucgucccggccug.....................  1     1  T6P
```

```
                                    Star                              Mature
ucuauaguucaacucauaauuguuugcagaagugucagguugugaugcgugcaaaacuguaguuugagcaaauugcacucgucccggccugcucacucugacuguacaaccg
......................................................................aauugcacucgucUcggccug.....................    5      1   T6P
......................................................................aauugcacucgucccggccuA.....................    3      1   T6P
......................................................................aauugcaUucgucccggccug.....................    1      1   T6P
......................................................................aauugcacucgucccggccGg.....................    1      1   T6P
......................................................................Cauugcacucgucccggccug.....................    1      1   T6P
......................................................................aauugcacucgucccgAccug.....................    1      1   T6P
......................................................................aauugcacucgAcccggccugc....................    2      1   T6P
......................................................................aauugcacucgucUcggccugc....................    15     1   T6P
......................................................................aauugUacucgucccggccugc....................    3      1   T6P
......................................................................aauugcacucgucccggccuCc....................    4      1   T6P
......................................................................aauugcacuAgucccggccugc....................    2      1   T6P
......................................................................aauugcacucgucccggccugU....................    282    1   T6P
......................................................................aauugcacucguccAggccugc....................    1      1   T6P
......................................................................aauugcacucgucccUgccugc....................    4      1   T6P
......................................................................aauugcGcucgucccggccugc....................    9      1   T6P
......................................................................aauugcacAcgucccggccugc....................    1      1   T6P
......................................................................aauugcacucCucccggccugc....................    1      1   T6P
......................................................................aauugcacucgucccggccugc....................    9824   0   T6P
......................................................................aauugcaUucgucccggccugc....................    5      1   T6P
......................................................................aauugcacucgucccggccCgc....................    5      1   T6P
......................................................................aauugcacucgucccggcUugc....................    3      1   T6P
......................................................................aauugcUcucgucccggccugc....................    1      1   T6P
......................................................................aaCugcacucgucccggccugc....................    2      1   T6P
......................................................................aauugcacucgucccggccGgc....................    2      1   T6P
......................................................................aauugcacucgucccggccAgc....................    1      1   T6P
......................................................................aauuUcacucgucccggccugc....................    1      1   T6P
......................................................................aauuCcacucgucccggccugc....................    1      1   T6P
......................................................................aauCgcacucgucccggccugc....................    1      1   T6P
......................................................................aauugcacucgucccgUccugc....................    4      1   T6P
......................................................................aauugcacCcgucccggccugc....................    2      1   T6P
......................................................................aauugcacucgucccggccuAc....................    4      1   T6P
......................................................................aauugAacucgucccggccugc....................    1      1   T6P
......................................................................Cauugcacucgucccggccugc....................    1      1   T6P
......................................................................aauuAcacucgucccggccugc....................    4      1   T6P
......................................................................aGuugcacucgucccggccugc....................    1      1   T6P
......................................................................aauugcacucgucccggccuUc....................    2      1   T6P
......................................................................aauugcacucgucccgAccugc....................    5      1   T6P
......................................................................aauugcacucguUccggccugc....................    25     1   T6P
......................................................................aauugcacucgucccCgccugc....................    3      1   T6P
......................................................................aauGgcacucgucccggccugc....................    3      1   T6P
......................................................................aauugcacucgCcccggccugc....................    1      1   T6P
......................................................................aauugcacucguccGggccugc....................    1      1   T6P
......................................................................aauugcCcucgucccggccugc....................    1      1   T6P
......................................................................Gauugcacucgucccggccugc....................    2      1   T6P
......................................................................aauugcacucgucccggccugA....................    13     1   T6P
......................................................................aauugcacucgucccggccugG....................    5      1   T6P
......................................................................aauugcacucgucccggUcugc....................    9      1   T6P
......................................................................aauugcacucgucccgCccugc....................    3      1   T6P
......................................................................aauugcacucguccUggccugc....................    15     1   T6P
......................................................................aauugcacucUucccggccugc....................    3      1   T6P
......................................................................aauugcacuUgucccggccugc....................    8      1   T6P
......................................................................aGuugcacucgucccggccugcu...................    1      1   T6P
......................................................................Uauugcacucgucccggccugcu...................    1308   1   T6P
......................................................................aauugcacucgucccUgccugcu...................    1      1   T6P
......................................................................aauugcacucgucccggccugcA...................    571    1   T6P
......................................................................aauugcacucgucccggUcugcu...................    2      1   T6P
......................................................................aauugcacucgucccggccuCcu...................    1      1   T6P
......................................................................aauugcacucgucccggccugUu...................    65     1   T6P
......................................................................aauugcacucgucccggccugcC...................    21     1   T6P
......................................................................aauugcacucgucccggccugAu...................    2      1   T6P
......................................................................aauugcacucgucccggccCgcu...................    1      1   T6P
......................................................................aauugcacucgucUcggccugcu...................    1      1   T6P
......................................................................aauugcacucAucccggccugcu...................    1      1   T6P
......................................................................aauugcacucgucccggccugcu...................    1273   0   T6P
......................................................................aauugcacucgucccggccugcG...................    12     1   T6P
......................................................................aauugUacucgucccggccugcu...................    1      1   T6P
......................................................................aauugcacucguUccggccugcu...................    2      1   T6P
......................................................................aauugcacucgucccggccuAcu...................    1      1   T6P
......................................................................aauuUcacucgucccggccugcu...................    2      1   T6P
......................................................................aauugcacucgucccCgccugcu...................    1      1   T6P
```

Star Mature

```
ucuauaguucaacucauaauuguuugcagaagugucagguugugaugcgugcaaaacuguaguuugagcaaauugcacucgucccggccugcucacucugacuguacaaccg
......................................................................aauugcacucgucccggccugcuU..................	292	1	T6P
......................................................................aauugcacucgucccggccugcAc..................	1	1	T6P
......................................................................aauugcacucgucccggccugcuA..................	22	1	T6P
......................................................................Uauugcacucgucccggccugcuc..................	2	1	T6P
......................................................................aauugcacucgucccggccugcuc..................	3	0	T6P
......................................................................aauugcacucgucccggccugcucU.................	2	1	T6P
.......................................................................auugcacucgucccUgccu......................	1	1	T6P
.......................................................................auugcacucgucccggccu......................	7	0	T6P
.......................................................................auugcacucgucccggccug.....................	11	0	T6P
.......................................................................Guugcacucgucccggccug.....................	2	1	T6P
.......................................................................auugcacucguccUggccugc....................	1	1	T6P
.......................................................................auugcacucgucccggccugA....................	1	1	T6P
.......................................................................auugcacucgucUcggccugc....................	2	1	T6P
.......................................................................auugcacucgucccgCccugc....................	1	1	T6P
.......................................................................auugcacucgucccggccugU....................	2	1	T6P
.......................................................................auugcacucgucccggccugc....................	354	0	T6P
.......................................................................auugcacucgucccgAccugc....................	1	1	T6P
.......................................................................auugcacucgucccggUcugc....................	1	1	T6P
.......................................................................auugcacuAgucccggccugc....................	1	1	T6P
.......................................................................auugcacucgucccggccugcu...................	14	0	T6P
.......................................................................auugcacucgucccggccugcC...................	2	1	T6P
.......................................................................auugcacucgucccggccugcuA..................	1	1	T6P
.......................................................................auugcacucgucccggccugcuU..................	1	1	T6P
........................................................................uugcacucgucccggccu......................	1	0	T6P
........................................................................uugcacucgucccggccugc....................	19	0	T6P
........................................................................uugcacucgucccggccugcC...................	2	1	T6P
........................................................................uugcacucgucccggccCgcu...................	1	1	T6P
........................................................................uugcacucgucccggccugcu...................	53	0	T6P
........................................................................uugcacucgucccggccugcuA..................	10	1	T6P
........................................................................uugcacucgucccggccugcuU..................	23	1	T6P
.........................................................................ugcacucgucccggccugc....................	4	0	T6P
.........................................................................ugcacucgucccggccugcuU..................	1	1	T6P
..........................................................................gcacucgucccggccugc....................	4	0	T6P
..........................................................................gcacucgucccggccugcu...................	3	0	T6P
..........................................................................gcacucgucccggccugcuU..................	1	1	T6P

................................ugucagguugugaugcgugc............................................................	1	0	T63
....................................agguugugaugcgugcaaa.........................................................	6	0	T63
....................................agguugugaugcgugcaaaac.......................................................	4	0	T63
....................................agguugugaugcgugcaaaacu......................................................	19	0	T63
....................................agguugugaugcgugcaaaacA......................................................	2	1	T63
....................................agguugugaugUgugcaaaacu......................................................	1	1	T63
....................................agguugugaugcgugcaaaacug.....................................................	2	0	T63
..................................................................agAaaauugcacucgucccggccugc....................	1	1	T63
.....................................................................Uaauugcacucgucccggccu......................	1	1	T63
......................................................................aauugcacucgucccggA........................	1	1	T63
......................................................................aauugcacucgucccggc........................	2	0	T63
......................................................................aauugcacucgucccggcA.......................	1	1	T63
......................................................................aauugcacucgucccggcc.......................	5	0	T63
......................................................................aauugcacuGgucccggccu......................	1	1	T63
......................................................................aauugcacucgucccgAccu......................	1	1	T63
......................................................................aauugcacucgucccgCccu......................	1	1	T63
......................................................................aauugcacucguccUggccu......................	1	1	T63
......................................................................aauugcacucgucccggUcu......................	2	1	T63
......................................................................aauugcacucgucccggccu......................	295	0	T63
......................................................................aauugcacucguUccggccu......................	3	1	T63
......................................................................aauugcGcucgucccggccu......................	1	1	T63
......................................................................aaAugcacucgucccggccu......................	1	1	T63
......................................................................aauugcacuAgucccggccu......................	1	1	T63
......................................................................aauugcacucAucccggccu......................	1	1	T63
......................................................................aauugcacucgucUcggccug.....................	1	1	T63
......................................................................aGuugcacucgucccggccug.....................	1	1	T63
......................................................................aauugcacucgucccggccuU.....................	5	1	T63
......................................................................aauugcacuAgucccggccug.....................	2	1	T63
......................................................................aauugcacucgucccggccug.....................	130	0	T63
......................................................................aauGgcacucgucccggccugc....................	1	1	T63
......................................................................aauugcacucgucccggccGgc....................	1	1	T63
......................................................................aauugcacucgucccggUcugc....................	1	1	T63
......................................................................aauugcCcucgucccggccugc....................	1	1	T63
......................................................................aauugcacucgucccCgccugc....................	1	1	T63
```

Star **Mature**

```
ucuauaguucaacucauaauuguuugcagaagugucagguugugaugcgugcaaaacuguaguuugagcaaauugcacucgucccggccugcucacucugacuguacaaccg
......................................................................aauugcacucgucccggccugU....................  144   1  T63
......................................................................aauugcacucgucccggccuAc....................  2     1  T63
......................................................................aauugcacucgucccggccugA....................  7     1  T63
......................................................................aCuugcacucgucccggccugc....................  2     1  T63
......................................................................aauugcacuGgucccggccugc....................  10    1  T63
......................................................................aauugcacucgucccggccugc....................  595   0  T63
......................................................................aauugcacuAgucccggccugc....................  8     1  T63
......................................................................aauugcacucgucccggccugAu...................  2     1  T63
......................................................................aauugcacucgucccggccugcu...................  141   0  T63
......................................................................Uauugcacucgucccggccugcu...................  273   1  T63
......................................................................aauugcacucgucccggccugUu...................  59    1  T63
......................................................................aauugcacucgucccggccugcG...................  1     1  T63
......................................................................aauugcacucgucccggccugcA...................  69    1  T63
......................................................................aauugcacucgucccggccugcC...................  4     1  T63
......................................................................aauugcacucgucccggccugcuA..................  1     1  T63
......................................................................aauugcacucgucccggccugcuU..................  46    1  T63
......................................................................aauugcacucgucccggccugcuAa.................  2     1  T63
.......................................................................auugcacucgucccggccug.....................  3     0  T63
.......................................................................auugcacuUgucccggccug.....................  2     1  T63
.......................................................................auugcacucgucccggccugc....................  79    0  T63
.......................................................................auugcacucgucccggccugU....................  4     1  T63
.......................................................................auugcacucgucccggccugcu...................  3     0  T63
........................................................................uugcacucgucccggccugc....................  1     0  T63
........................................................................uugcacucgucccggccugcC...................  2     1  T63
........................................................................uugcacucgucccggccugcu...................  9     0  T63
........................................................................uugcacucgucccggccugcuU..................  6     1  T63
..........................................................................gcacucgucccggccugcu...................  1     0  T63

................................ugucagguugugaugcgugca...........................................................  1     0  MOL
....................................agguugugaugcgugcaaa.........................................................  4     0  MOL
....................................agguugugaugcgugcaaaa........................................................  2     0  MOL
....................................agguugugaugcgugcaaaac.......................................................  2     0  MOL
....................................agguugugaugcgugcaaaacu......................................................  29    0  MOL
....................................agguugugaugcgugcaaaacuU.....................................................  2     1  MOL
....................................agguugugaugcgugcaaaacug.....................................................  6     0  MOL
.................................................................gagGaaauugcacucgucccggccu......................  1     1  MOL
..................................................................agAaaauugcacucgucccggccu......................  1     1  MOL
..................................................................agAaaauugcacucgucccggccugcu...................  1     1  MOL
.....................................................................Uaauugcacucgucccggcc.......................  1     1  MOL
.....................................................................aaauugcacucgucccggccu......................  2     0  MOL
.....................................................................aaUuugcacucgucccggccug.....................  1     1  MOL
.....................................................................aaauugcacucgucccggccug.....................  1     0  MOL
.....................................................................aaauugcacucgucccgAccug.....................  1     1  MOL
.....................................................................aaauugcacucgucccggccugc....................  4     0  MOL
.....................................................................Uaauugcacucgucccggccugc....................  11    1  MOL
......................................................................aauugcacucgucccggc........................  2     0  MOL
......................................................................aauuAcacucgucccggc........................  1     1  MOL
......................................................................aauugcacucgucccggcc.......................  39    0  MOL
......................................................................aauugcacucgucccggcA.......................  1     1  MOL
......................................................................aauugcacucUucccggcc.......................  1     1  MOL
......................................................................aauugcacucguUccggcc.......................  1     1  MOL
......................................................................aauugcacucgucccggccA......................  7     1  MOL
......................................................................aauugcacuAgucccggccu......................  3     1  MOL
......................................................................aauugUacucgucccggccu......................  1     1  MOL
......................................................................aauugcacuUgucccggccu......................  2     1  MOL
......................................................................aauugcacucgucccggUcu......................  2     1  MOL
......................................................................aauugcacucgucccggcUu......................  1     1  MOL
......................................................................aauugcacucgucccgUccu......................  2     1  MOL
......................................................................aauugcacucguccUggccu......................  3     1  MOL
......................................................................Gauugcacucgucccggccu......................  1     1  MOL
......................................................................aauugcacucgucccggGcu......................  1     1  MOL
......................................................................aauugcacucgucccgCccu......................  1     1  MOL
......................................................................aauugcaAucgucccggccu......................  1     1  MOL
......................................................................aGuugcacucgucccggccu......................  1     1  MOL
......................................................................aauugcacAcgucccggccu......................  1     1  MOL
......................................................................aauugcacucgucUcggccu......................  10    1  MOL
......................................................................aauugGacucgucccggccu......................  1     1  MOL
......................................................................aauugcacucgucccggccu......................  1792  0  MOL
......................................................................aauugcacucgucccgAccu......................  1     1  MOL
......................................................................aauugcacucguUccggccu......................  12    1  MOL
```

Star Mature

```
ucuauaguucaacucauaauuguuugcagaagugucagguugugaugcgugcaaaacuguaguuugagcaaauugcacucgucccggccugcucacucugacuguacaaccg
......................................................................aauAgcacucgucccggccu......................   1      1   MOL
......................................................................aauugcacucgucccggccC......................   1      1   MOL
......................................................................aauuCcacucgucccggccug.....................   1      1   MOL
......................................................................aauugcacucgucccggccug.....................   2230   0   MOL
......................................................................aauuUcacucgucccggccug.....................   1      1   MOL
......................................................................aauugcacucgucccggccuC.....................   2      1   MOL
......................................................................aauugcacucgucccggcAug.....................   1      1   MOL
......................................................................Cauugcacucgucccggccug.....................   2      1   MOL
......................................................................aauugcacucgGcccggccug.....................   1      1   MOL
......................................................................aauugcacucguUccggccug.....................   6      1   MOL
......................................................................aauCgcacucgucccggccug.....................   1      1   MOL
......................................................................aauugcacucgucccggccAg.....................   1      1   MOL
......................................................................aCuugcacucgucccggccug.....................   1      1   MOL
......................................................................aauugUacucgucccggccug.....................   1      1   MOL
......................................................................aauugcacucgucccggccuU.....................   86     1   MOL
......................................................................aauugcacucgucccUgccug.....................   1      1   MOL
......................................................................aauugcacAcgucccggccug.....................   1      1   MOL
......................................................................aauugcacucAucccggccug.....................   1      1   MOL
......................................................................aauugcacucgucccggccCg.....................   5      1   MOL
......................................................................aUuugcacucgucccggccug.....................   5      1   MOL
......................................................................aauugAacucgucccggccug.....................   1      1   MOL
......................................................................Gauugcacucgucccggccug.....................   2      1   MOL
......................................................................aauugcacucgucccgAccug.....................   3      1   MOL
......................................................................aauugcacucgucUcggccug.....................   10     1   MOL
......................................................................aauugcacucgucccggccuA.....................   14     1   MOL
......................................................................aauugcacucgucccggUcug.....................   3      1   MOL
......................................................................aauugcacucguccUggccug.....................   5      1   MOL
......................................................................aauuAcacucgucccggccug.....................   2      1   MOL
......................................................................aauugcacucgAcccggccug.....................   2      1   MOL
......................................................................aauugcacucguAccggccug.....................   1      1   MOL
......................................................................aauugcacucgucccggGcugc....................   1      1   MOL
......................................................................aauugcacucgucccggccuUc....................   7      1   MOL
......................................................................aauugcacGcgucccggccugc....................   1      1   MOL
......................................................................aauugcacucgucccggccuCc....................   6      1   MOL
......................................................................aauugcacucgucccggccugG....................   2      1   MOL
......................................................................aauugcacucgucccUgccugc....................   2      1   MOL
......................................................................Cauugcacucgucccggccugc....................   5      1   MOL
......................................................................aaCugcacucgucccggccugc....................   1      1   MOL
......................................................................aauAgcacucgucccggccugc....................   8      1   MOL
......................................................................aUuugcacucgucccggccugc....................   7      1   MOL
......................................................................aauugcacucgucccggccugU....................   556    1   MOL
......................................................................aauugcacAcgucccggccugc....................   2      1   MOL
......................................................................aauugcGcucgucccggccugc....................   1      1   MOL
......................................................................aauugUacucgucccggccugc....................   10     1   MOL
......................................................................aauugcacucAucccggccugc....................   2      1   MOL
......................................................................aauugcacucguccUggccugc....................   12     1   MOL
......................................................................aauugcacucgucccggccGgc....................   8      1   MOL
......................................................................aauugcacucgucccggccCgc....................   5      1   MOL
......................................................................aauugcacucgAcccggccugc....................   1      1   MOL
......................................................................aauugcacucgucccggcAugc....................   4      1   MOL
......................................................................aauugcacucgucccggUcugc....................   5      1   MOL
......................................................................aauugcacucgucUcggccugc....................   16     1   MOL
......................................................................aauugcacucgucccggcUugc....................   3      1   MOL
......................................................................Gauugcacucgucccggccugc....................   8      1   MOL
......................................................................aauugcacucgucccggccugc....................   10166  0   MOL
......................................................................aauugcacucguGccggccugc....................   1      1   MOL
......................................................................aauugcacuAgucccggccugc....................   4      1   MOL
......................................................................aauugcacucgucccAgccugc....................   2      1   MOL
......................................................................aauuAcacucgucccggccugc....................   2      1   MOL
......................................................................aauugcacucguUccggccugc....................   37     1   MOL
......................................................................aauugcacucgCcccggccugc....................   2      1   MOL
......................................................................aauugcacucgucccgAccugc....................   16     1   MOL
......................................................................aauugcacucgucccggccAgc....................   3      1   MOL
......................................................................aauugcUcucgucccggccugc....................   1      1   MOL
......................................................................aauugcacucgucccggccugA....................   24     1   MOL
......................................................................aauugcaUucgucccggccugc....................   4      1   MOL
......................................................................aauugcacucgGcccggccugc....................   3      1   MOL
......................................................................aauugcacucCucccggccugc....................   1      1   MOL
......................................................................aauugcacucgucccggccuAc....................   7      1   MOL
......................................................................aauugcacucgucccCgccugc....................   1      1   MOL
```

```
                                             Star                                    Mature
ucuauaguucaacucauaauuguuugcagaagugucagguugugaugcgugcaaaacuguaguuugagcaaauugcacucgucccggccugcucacucugacuguacaaccg
......................................................................aauugcacucgucAcggccugc....................   2     1   MOL
......................................................................aauugcacuGgucccggccugc....................   4     1   MOL
......................................................................aCuugcacucgucccggccugc....................   4     1   MOL
......................................................................aauugcacucUucccggccugc....................   1     1   MOL
......................................................................aGuugcacucgucccggccugc....................   8     1   MOL
......................................................................aauugcaAucgucccggccugc....................   2     1   MOL
......................................................................aauugcacucgucccggccugAu...................   2     1   MOL
......................................................................aauugcacucgucccggccugcC...................   21    1   MOL
......................................................................aUuugcacucgucccggccugcu...................   2     1   MOL
......................................................................aauugcacAcgucccggccugcu...................   1     1   MOL
......................................................................aauugAacucgucccggccugcu...................   1     1   MOL
......................................................................aauugcacucgucccggccugcu...................   1447  0   MOL
......................................................................aauugUacucgucccggccugcu...................   1     1   MOL
......................................................................aauugcacucguccUggccugcu...................   1     1   MOL
......................................................................aauugcacucgucccggccAgcu...................   2     1   MOL
......................................................................aauugcacucgucccggcUugcu...................   1     1   MOL
......................................................................aGuugcacucgucccggccugcu...................   1     1   MOL
......................................................................aauugcacucUucccggccugcu...................   1     1   MOL
......................................................................aauugcacucgGcccggccugcu...................   2     1   MOL
......................................................................aauugcacucgucccggAcugcu...................   1     1   MOL
......................................................................aauugcacucgAcccggccugcu...................   1     1   MOL
......................................................................aauugcacucgucccggccugUu...................   161   1   MOL
......................................................................aauugcacucgucccggccuUcu...................   1     1   MOL
......................................................................aauugGacucgucccggccugcu...................   1     1   MOL
......................................................................aauugcacuAgucccggccugcu...................   1     1   MOL
......................................................................aauugcacucgucccggUcugcu...................   2     1   MOL
......................................................................Uauugcacucgucccggccugcu...................   1854  1   MOL
......................................................................aauugcacucgucUcggccugcu...................   5     1   MOL
......................................................................aauugcacucgucccggccugcG...................   13    1   MOL
......................................................................aauugcacucgucccggccugcA...................   518   1   MOL
......................................................................aauugcacucgucccgAccugcu...................   1     1   MOL
......................................................................aauugcacucguUccggccugcu...................   2     1   MOL
......................................................................aauGgcacucgucccggccugcu...................   1     1   MOL
......................................................................Cauugcacucgucccggccugcu...................   1     1   MOL
......................................................................aauugcacucgCcccggccugcu...................   1     1   MOL
......................................................................aauugcacucgucAcggccugcu...................   2     1   MOL
......................................................................aauugcacucgucccggccugcuG..................   2     1   MOL
......................................................................Uauugcacucgucccggccugcuc..................   10    1   MOL
......................................................................aauugcacucgucccggccugcuc..................   4     0   MOL
......................................................................aauugcacucgucccggccugcuA..................   14    1   MOL
......................................................................aauugcacucgucccggccugUuc..................   1     1   MOL
......................................................................aauugcacucgucccggccugcuU..................   430   1   MOL
......................................................................aauugcacucgucccggccugcAca.................   1     1   MOL
......................................................................aauugcacucgucccggccugcucU.................   3     1   MOL
......................................................................aauugcacucgucccggccugcuUa.................   1     1   MOL
.......................................................................auugcacucgucccggcc.......................   1     0   MOL
.......................................................................auugcacucgucccggccu......................   11    0   MOL
.......................................................................auugcacucgucccggccuU.....................   2     1   MOL
.......................................................................auugcacucgucccggccug.....................   34    0   MOL
.......................................................................auugcacucgucUcggccug.....................   1     1   MOL
.......................................................................auugcacuUgucccggccug.....................   2     1   MOL
.......................................................................auugUacucgucccggccug.....................   1     1   MOL
.......................................................................auAgcacucgucccggccugc....................   1     1   MOL
.......................................................................auugcacucgucccggccuAc....................   1     1   MOL
.......................................................................Cuugcacucgucccggccugc....................   1     1   MOL
.......................................................................auugcacucgucccggccAgc....................   1     1   MOL
.......................................................................auugcGcucgucccggccugc....................   1     1   MOL
.......................................................................auugcacucgucccgAccugc....................   1     1   MOL
.......................................................................auugcacucCucccggccugc....................   1     1   MOL
.......................................................................auugcacucguUccggccugc....................   4     1   MOL
.......................................................................auugcacucgucccggUcugc....................   1     1   MOL
.......................................................................auugcacucgucccCgccugc....................   1     1   MOL
.......................................................................auugcacucgucccggccugc....................   682   0   MOL
.......................................................................auugcacuAgucccggccugc....................   3     1   MOL
.......................................................................auugcacucgucccggccugU....................   5     1   MOL
.......................................................................auugcacucgucccggccugcu...................   25    0   MOL
.......................................................................auugcGcucgucccggccugcu...................   1     1   MOL
.......................................................................auugcacucgucccggccugUu...................   2     1   MOL
.......................................................................aAugcacucgucccggccugcu...................   1     1   MOL
.......................................................................auugcacucgucccggccugcuU..................   10    1   MOL
```

```
                                    Star                              Mature
ucuauaguucaacucauaauuguuugcagaagugucagguugugaugcgugcaaaacuguaguuugagcaaauugcacucgucccggccugcucacucugacuguacaaccg
.......................................................................auugcacucgucccggccugcuA..................	1	1	MOL
........................................................................uugcacucgucccggccugU....................	1	1	MOL
........................................................................uugcacucgucccggccugc....................	22	0	MOL
........................................................................Cugcacucgucccggccugcu...................	1	1	MOL
........................................................................uugcacucgucccggccugUu...................	2	1	MOL
........................................................................uugcacucgucccggccugcG...................	1	1	MOL
........................................................................uugcacucgucccggccugcC...................	2	1	MOL
........................................................................uugcacucgucccggccugcu...................	42	0	MOL
........................................................................uugcacucgucccggccugcuA..................	11	1	MOL
........................................................................uugcacucgucccggccugcuU..................	27	1	MOL
........................................................................uugcacucgucccggccugcuc..................	2	0	MOL
.........................................................................ugcacucgucccggccug.....................	1	0	MOL
.........................................................................ugcacucgucccggccugc....................	3	0	MOL
.........................................................................ugcacucgucccggcUugc....................	1	1	MOL
.........................................................................ugcacucgucccggccugUu...................	1	1	MOL
.........................................................................ugcacucgucccggccugcuc..................	1	0	MOL
..........................................................................gcacucgucccggccugc....................	4	0	MOL
```

Provisional ID	:	Scaffold_4228_7680
Score total	:	52180.4
Score for star read(s)	:	3.9
Score for read counts	:	52170.5
Score for mfe	:	1.4
Score for randfold	:	1.6
Score for cons. seed	:	3
Total read count	:	102342
Mature read count	:	100921
Loop read count	:	68
Star read count	:	1353

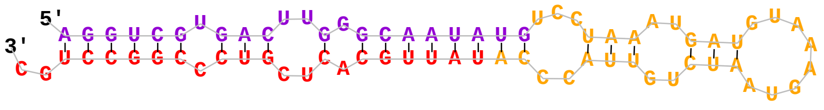

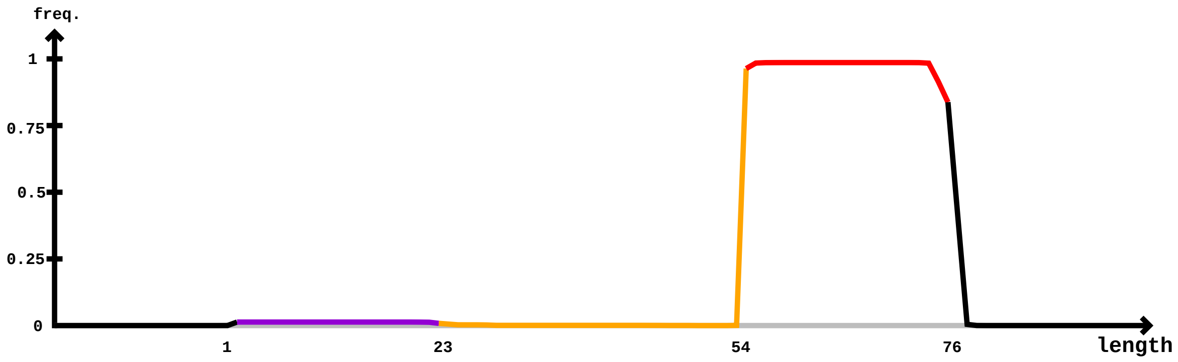

Star **Mature**

```
5'- auaagaguaacucuugaaggucgugacuugggcaauauguccuaaaugauguaaaguaaucuguuacccauauugcacucgucccggccugcaagggcuuccucaaaagcaa   -3'    obs
    auaagaguaacucuugaaggucgugacuugggcaauauguccuaaaugauguaaaguaaucuguuacccauauugcacucgucccggccugcaagggcuuccucaaaagcaa          exp
    ....(((.((((((((.((((((.(((..(.((((((((...(((..(((........)))..)))..)))))))).)..))).)))))).)))))).)).)))........   reads   mm   sample
    ................aaggucgugacuugggcaa.............................................................................   1       0    MOL
    ................aaggucgugacuugggcaauau..........................................................................   1       0    MOL
    .................aggucgugacuugggcaau............................................................................   2       0    MOL
    .................aggucgugacuugggcaaua...........................................................................   3       0    MOL
    .................agAucgugacuugggcaauau..........................................................................   1       1    MOL
    .................aggucgugacuugggcaauau..........................................................................   70      0    MOL
    .................aggucgugacuugggcaauauA.........................................................................   1       1    MOL
    .................aggucgugacuugggcaauaug.........................................................................   59      0    MOL
    .................aggucgugacuugggcaauauU.........................................................................   1       1    MOL
    .................aggucgugacuugggcaauaugA........................................................................   5       1    MOL
    .......................................uccuaaaugauguaaaguaauc...................................................   1       0    MOL
    .......................................uccuaaaugauguaaaguaaucu..................................................   1       0    MOL
    .......................................uccuaaaugauguaaaguaaucuguu...............................................   2       0    MOL
    .......................................uccuaaaugauguaaaguaaucuguua..............................................   2       0    MOL
    .....................................................................auauugcacucgucccggccu......................   2       0    MOL
    .....................................................................auauugcacucgucccggccug.....................   1       0    MOL
    .....................................................................auauugcacucgucccggccugc....................   2       0    MOL
    .....................................................................Uuauugcacucgucccggccugc....................   7       1    MOL
    ......................................................................uauugcacucgucccggc........................   1       0    MOL
    ......................................................................uauugcacuUgucccggcc.......................   4       1    MOL
    ......................................................................uauugcacucguUccggcc.......................   1       1    MOL
    ......................................................................uauugcacucgucccggcA.......................   2       1    MOL
    ......................................................................uauugcacucgucccggcc.......................   55      0    MOL
    ......................................................................uauugcacucgucccUgcc.......................   1       1    MOL
    ......................................................................uauugcacucgucccAgcc.......................   1       1    MOL
    ......................................................................uauugcUcucgucccggccu......................   1       1    MOL
    ......................................................................uauugcacucgAcccggccu......................   1       1    MOL
    ......................................................................uauugcacucgucccgAccu......................   1       1    MOL
    ......................................................................uauugcacucgucccgUccu......................   1       1    MOL
    ......................................................................uauugcacucgucccggGcu......................   1       1    MOL
    ......................................................................uauugcacucguUccggccu......................   13      1    MOL
    ......................................................................uauugcacucguccUggccu......................   6       1    MOL
    ......................................................................uauugUacucgucccggccu......................   1       1    MOL
```

Star **Mature**

```
auaagaguaacucuugaaggucgugacuugggcaauauguccuaaaugauguaaaguaaucuguuacccauauugcacucgucccggccugcaagggcuuccucaaaagcaa
......................................................................uauugcacucgucUcggccu......................  7      1  MOL
......................................................................uauugcacucgucAcggccu......................  1      1  MOL
......................................................................uauCgcacucgucccggccu......................  1      1  MOL
......................................................................uauugcGcucgucccggccu......................  1      1  MOL
......................................................................uauugcacucgucccggccA......................  8      1  MOL
......................................................................uauAgcacucgucccggccu......................  1      1  MOL
......................................................................uauugcacucgucccggUcu......................  1      1  MOL
......................................................................uauugcacucgucccggccu......................  2302   0  MOL
......................................................................uauugcacucguccAggccu......................  1      1  MOL
......................................................................uauugcacucgucccgCccu......................  1      1  MOL
......................................................................uauugcacucgucccggccC......................  3      1  MOL
......................................................................uauugcacuUgucccggccu......................  17     1  MOL
......................................................................Gauugcacucgucccggccu......................  1      1  MOL
......................................................................uauAgcacucgucccggccug.....................  1      1  MOL
......................................................................uauugcacucgucccggccug.....................  2503   0  MOL
......................................................................uauugcacucgucccgCccug.....................  1      1  MOL
......................................................................uauugcacucgucccgAccug.....................  2      1  MOL
......................................................................uauuAcacucgucccggccug.....................  1      1  MOL
......................................................................uauugcUcucgucccggccug.....................  1      1  MOL
......................................................................uauugcacucgucccggcUug.....................  2      1  MOL
......................................................................uauugcacucgucccggccGg.....................  1      1  MOL
......................................................................uauugcacucguccUggccug.....................  6      1  MOL
......................................................................uauugUacucgucccggccug.....................  1      1  MOL
......................................................................uauugcacucgucccggccCg.....................  2      1  MOL
......................................................................uauugAacucgucccggccug.....................  1      1  MOL
......................................................................uauugGacucgucccggccug.....................  1      1  MOL
......................................................................Gauugcacucgucccggccug.....................  2      1  MOL
......................................................................uauugcacucgAcccggccug.....................  1      1  MOL
......................................................................uauugcacucgucccggccuA.....................  19     1  MOL
......................................................................uauugcacuGgucccggccug.....................  1      1  MOL
......................................................................uauugcacucgCcccggccug.....................  1      1  MOL
......................................................................uauugcacuUgucccggccug.....................  58     1  MOL
......................................................................uauugcacucguUccggccug.....................  17     1  MOL
......................................................................uauugcacucgucUcggccug.....................  10     1  MOL
......................................................................Cauugcacucgucccggccug.....................  2      1  MOL
......................................................................uauugcacucgucccUgccug.....................  3      1  MOL
......................................................................uauugcacucgucccggUcug.....................  1      1  MOL
......................................................................uauugcacucgucccggccuU.....................  246    1  MOL
......................................................................uauugcacucgucccggccuC.....................  6      1  MOL
......................................................................uauugcacuAgucccggccug.....................  7      1  MOL
......................................................................uauuUcacucgucccggccug.....................  2      1  MOL
......................................................................uauugcacucguccGggccug.....................  2      1  MOL
......................................................................uauuCcacucgucccggccugc....................  2      1  MOL
......................................................................uauugcacucgucccggccugU....................  626    1  MOL
......................................................................Cauugcacucgucccggccugc....................  5      1  MOL
......................................................................uauugcacucguccUggccugc....................  15     1  MOL
......................................................................uauugcacucgucccggccAgc....................  7      1  MOL
......................................................................uUuugcacucgucccggccugc....................  3      1  MOL
......................................................................uauugcacucgucccggccugG....................  2      1  MOL
......................................................................uauugcacucgucccAgccugc....................  5      1  MOL
......................................................................uauugcaAucgucccggccugc....................  1      1  MOL
......................................................................uauugcacucgucccCgccugc....................  3      1  MOL
......................................................................uauugcacucgucccggcGugc....................  1      1  MOL
......................................................................uauugcaUucgucccggccugc....................  1      1  MOL
......................................................................uauugcacucgucccggccuAc....................  20     1  MOL
......................................................................Gauugcacucgucccggccugc....................  8      1  MOL
......................................................................uGuugcacucgucccggccugc....................  1      1  MOL
......................................................................uauugcGcucgucccggccugc....................  4      1  MOL
......................................................................uauugcacucgucccggccugc....................  16656  0  MOL
......................................................................uauugcacucguccAggccugc....................  1      1  MOL
......................................................................uauugcacucgucccggcUugc....................  13     1  MOL
......................................................................uauugcacucgucccggccCgc....................  12     1  MOL
......................................................................uaCugcacucgucccggccugc....................  1      1  MOL
......................................................................uauugcacucgAcccggccugc....................  2      1  MOL
......................................................................uauugcacucgucccggccugA....................  141    1  MOL
......................................................................uauugcacuUgucccggccugc....................  4      1  MOL
......................................................................uauugcacucgCcccggccugc....................  2      1  MOL
......................................................................uaAugcacucgucccggccugc....................  1      1  MOL
......................................................................uauugcacucAucccggccugc....................  3      1  MOL
......................................................................uauugcacucCucccggccugc....................  3      1  MOL
```

Star Mature

auaagaguaacucuugaaggucgugacuugggcaauauguccuaaaugauguaaaguaaucuguuacccauauugcacucgucccggccugcaagggcuuccucaaaagcaa			
....uauugAacucgucccggccugc....................	1	1	MOL
....uauugcacucgucccggUcugc....................	12	1	MOL
....uauugcacucguUccggccugc....................	33	1	MOL
....uauugcacAcgucccggccugc....................	8	1	MOL
....uauugcacucgucAcggccugc....................	3	1	MOL
....uauugGacucgucccggccugc....................	6	1	MOL
....uauugcacucgucccggccuUc....................	25	1	MOL
....uauugcacucgucccUgccugc....................	2	1	MOL
....uauugcacucgucccgUccugc....................	3	1	MOL
....uauugcUcucgucccggccugc....................	3	1	MOL
....uauugcacucgucccgAccugc....................	12	1	MOL
....uauugcacucgucccggccGgc....................	12	1	MOL
....uauugcacucgucccggccuCc....................	4	1	MOL
....uauugcaGucgucccggccugc....................	1	1	MOL
....uauugcacucgucUcggccugc....................	32	1	MOL
....uauugcacuAgucccggccugc....................	57	1	MOL
....uauugcacucgucccggAcugc....................	1	1	MOL
....uauugcacucgGcccggccugc....................	1	1	MOL
....uauugUacucgucccggccugc....................	8	1	MOL
....uauugcacGcgucccggccugc....................	1	1	MOL
....uauAgcacucgucccggccugc....................	12	1	MOL
....uauugUacucgucccggccugca...................	2	1	MOL
....uauugcacucgucccggGcugca...................	1	1	MOL
....uauugcacucgucccgAccugca...................	1	1	MOL
....uaGugcacucgucccggccugca...................	1	1	MOL
....uauugcacucgucccggccugUa...................	53	1	MOL
....uauugcacucgucccggccugAa...................	15	1	MOL
....uauugcacuAgucccggccugca...................	3	1	MOL
....uauugcacucgucccggUcugca...................	2	1	MOL
....Gauugcacucgucccggccugca...................	1	1	MOL
....Aauugcacucgucccggccugca...................	518	1	MOL
....uauugcacucgucAcggccugca...................	1	1	MOL
....uauugcacucgucccggccugcG...................	20	1	MOL
....uauugcacucgucccggccugcU...................	1854	1	MOL
....uauugcaAucgucccggccugca...................	1	1	MOL
....uauugcacucgucccggccugca...................	956	0	MOL
....uauugcacucgucccggccugcC...................	15	1	MOL
....uauCgcacucgucccggccugca...................	1	1	MOL
....uauugcacucgCcccggccugca...................	1	1	MOL
....uauugcacCcgucccggccugca...................	1	1	MOL
....Cauugcacucgucccggccugca...................	1	1	MOL
....uauugcacucgucccggccugAaa..................	2	1	MOL
....uauugcacucgucccggccugcaa..................	17	0	MOL
....uauugcacucgucccggccugcCa..................	1	1	MOL
....Aauugcacucgucccggccugcaa..................	5	1	MOL
....uauugcacucgucccggccugcGa..................	1	1	MOL
....uauugcacucgucccggccugcUa..................	23	1	MOL
....uauugcacucgucccggccugcaU..................	56	1	MOL
....uauugcacucgucccggccugUaa..................	8	1	MOL
....uauugcacucgucccggccugcaaU.................	4	1	MOL
....auugcacucgucccggcc.......................	1	0	MOL
....auugcacucgucccggccu......................	11	0	MOL
....auugUacucgucccggccug.....................	1	1	MOL
....auugcacuUgucccggccug.....................	2	1	MOL
....auugcacucgucccggccuU.....................	2	1	MOL
....auugcacucgucUcggccug.....................	1	1	MOL
....auugcacucgucccggccug.....................	34	0	MOL
....auugcacucgucccggccuAc....................	1	1	MOL
....auugcacucgucccggccAgc....................	1	1	MOL
....auugcacucguUccggccugc....................	4	1	MOL
....auugcacuAgucccggccugc....................	3	1	MOL
....auugcacucgucccggccugU....................	5	1	MOL
....auAgcacucgucccggccugc....................	1	1	MOL
....auugcacucgucccCgccugc....................	1	1	MOL
....auugcacucCucccggccugc....................	1	1	MOL
....auugcacucgucccggUcugc....................	1	1	MOL
....Cuugcacucgucccggccugc....................	1	1	MOL
....auugcacucgucccgAccugc....................	1	1	MOL
....auugcGcucgucccggccugc....................	1	1	MOL
....auugcacucgucccggccugc....................	682	0	MOL

```
                 Star                                                 Mature
auaagaguaacucuugaaggucgugacuugggcaauauguccuaaaugauguaaaguaaucuguuacccauauugcacucgucccggccugcaagggcuuccucaaaagcaa
.......................................................................auugcacucgucccggccugca...................    13     0    MOL
.......................................................................auugcacucgucccggccugcUa..................    1      1    MOL
.......................................................................auugcacucgucccggccugcaU..................    1      1    MOL
........................................................................uugcacucgucccggccugU....................    1      1    MOL
........................................................................uugcacucgucccggccugc....................    22     0    MOL
........................................................................uugcacucgucccggccugcG...................    1      1    MOL
........................................................................uugcacucgucccggccugcC...................    2      1    MOL
........................................................................uugcacucgucccggccugca...................    5      0    MOL
........................................................................uugcacucgucccggccugcaU..................    1      1    MOL
........................................................................uugcacucgucccggccugcUa..................    11     1    MOL
.........................................................................ugcacucgucccggccug.....................    1      0    MOL
.........................................................................ugcacucgucccggcUugc....................    1      1    MOL
.........................................................................ugcacucgucccggccugc....................    3      0    MOL
..........................................................................gcacucgucccggccugc....................    4      0    MOL

................aaggucgugacuugggcaa.............................................................................    2      0    T63
.................aggucgugacuugggcaa.............................................................................    1      0    T63
.................aggucgugacuugggcaau............................................................................    5      0    T63
.................aggucgugacuugggcaaua...........................................................................    7      0    T63
.................aggucgugacuugggcaauau..........................................................................    31     0    T63
.................aggucgugUcuugggcaauaug.........................................................................    1      1    T63
.................aggucgugacuugggcaauauA.........................................................................    2      1    T63
.................aggucgugacuugggcaauaug.........................................................................    49     0    T63
.................aggucgugacuugggcaauauU.........................................................................    2      1    T63
.................aggucgugacuugggcaauauguccu.....................................................................    2      0    T63
.......................................uccuaaaugauguaaaguaau....................................................    1      0    T63
.......................................uccuaaaugauguaaaguaauc...................................................    1      0    T63
.......................................uccuaaaugauguaaaguaaucuguu...............................................    1      0    T63
.....................................................................Uuauugcacucgucccggccu......................    1      1    T63
.....................................................................auauugcacucgucccggccug.....................    1      0    T63
.....................................................................Uuauugcacucgucccggccugc....................    2      1    T63
.....................................................................auauugcacucgucccggccugc....................    2      0    T63
......................................................................uauugcacucgucccggc........................    4      0    T63
......................................................................uauugcacuUgucccggcc.......................    10     1    T63
......................................................................uauuUcacucgucccggcc.......................    1      1    T63
......................................................................uauugcacucgucccggcA.......................    9      1    T63
......................................................................uauugcacucgucccggcc.......................    13     0    T63
......................................................................uauugcacucgucUcggccu......................    2      1    T63
......................................................................uauugcacCcgucccggccu......................    1      1    T63
......................................................................uauugcacucguUccggccu......................    3      1    T63
......................................................................uauugcacuUgucccggccu......................    13     1    T63
......................................................................uauugcacucgucccggccu......................    601    0    T63
......................................................................uauugcacucAucccggccu......................    2      1    T63
......................................................................uauugcacucgucccggccC......................    1      1    T63
......................................................................uauugcacAcgucccggccu......................    1      1    T63
......................................................................uauugcacucgucccggcUu......................    1      1    T63
......................................................................uauugcacucgucUcggccug.....................    2      1    T63
......................................................................uauuCcacucgucccggccug.....................    1      1    T63
......................................................................uauugcacucgucccggccug.....................    242    0    T63
......................................................................uauugcacucgucccggccuC.....................    2      1    T63
......................................................................uauugcacucgucccggccuU.....................    36     1    T63
......................................................................uauugcacucgucccgAccug.....................    1      1    T63
......................................................................uauugcacuUgucccggccug.....................    52     1    T63
......................................................................uauugcacucgucccggUcug.....................    1      1    T63
......................................................................uCuugcacucgucccggccug.....................    1      1    T63
......................................................................uauugcacucgucccAgccug.....................    1      1    T63
......................................................................uauugcacucguUccggccug.....................    1      1    T63
......................................................................uauugcacucgucccggccuA.....................    9      1    T63
......................................................................uauugcacucgucccggccGg.....................    2      1    T63
......................................................................uauugcacucgucccgUccugc....................    1      1    T63
......................................................................uauugcacucguccAggccugc....................    1      1    T63
......................................................................uauugcacucgucccggccugc....................    1589   0    T63
......................................................................uauugcacAcgucccggccugc....................    1      1    T63
......................................................................uauugcacucgucccCgccugc....................    1      1    T63
......................................................................uauugcacucgucccggcAugc....................    1      1    T63
......................................................................uauugcacucgucUcggccugc....................    2      1    T63
......................................................................uauugcacucgucccgAccugc....................    3      1    T63
......................................................................uauugcacucgucccggccugU....................    158    1    T63
......................................................................uauugcacuUgucccggccugc....................    1      1    T63
......................................................................uauugcacucgucccggccuAc....................    2      1    T63
```

```
                Star                                               Mature
auaagaguaacucuugaaggucgugacuugggcaauauguccuaaaugauguaaaguaaucuguuacccauauugcacucgucccggccugcaagggcuuccucaaaagcaa
......................................................................uauAgcacucgucccggccugc....................   2     1    T63
......................................................................uauugcacucgucccggccCgc....................   1     1    T63
......................................................................uauugcacucgucccggccugA....................   5     1    T63
......................................................................uauugcacucgucccggccuUc....................   16    1    T63
......................................................................uauugcacucguccUggccugc....................   2     1    T63
......................................................................uauugcacucgucccggccugG....................   2     1    T63
......................................................................uauugcacucgucccggccGgc....................   1     1    T63
......................................................................uauugcacucgucccggccAgc....................   1     1    T63
......................................................................uauugcacucgucccggccugcC...................   2     1    T63
......................................................................uauugcacucgucccggccugcU...................   273   1    T63
......................................................................uauugcacucgucccggccugUa...................   23    1    T63
......................................................................uauugcacucgucccggccugAa...................   3     1    T63
......................................................................uauugcacucgucccggccugcG...................   5     1    T63
......................................................................uauugcacucgucccggccugca...................   101   0    T63
......................................................................Aauugcacucgucccggccugca...................   69    1    T63
......................................................................uauugcacucgucccggccuUca...................   1     1    T63
......................................................................uauugcacucgucccggccugcaa..................   5     0    T63
......................................................................uauugcacucgucccggccugcaU..................   10    1    T63
......................................................................Aauugcacucgucccggccugcaa..................   2     1    T63
......................................................................uauugcacucgucccggccugUaa..................   9     1    T63
......................................................................uauugcacucgucccggccugcaaU.................   2     1    T63
.......................................................................auugcacuUgucccggccug.....................   2     1    T63
.......................................................................auugcacucgucccggccug.....................   3     0    T63
.......................................................................auugcacucgucccggccugc....................   79    0    T63
.......................................................................auugcacucgucccggccugU....................   4     1    T63
........................................................................uugcacucgucccggccugc....................   1     0    T63
........................................................................uugcacucgucccggccugca...................   1     0    T63
........................................................................uugcacucgucccggccugcC...................   2     1    T63

................aaggucgugacuugggcaauau..........................................................................   2     0    T6P
.................aggucgugacuugggcaa.............................................................................   2     0    T6P
.................aggucgugacuugggcaau............................................................................   3     0    T6P
.................aggucgugacuugggcaaua...........................................................................   11    0    T6P
.................aggucgugacuugggcaauau..........................................................................   128   0    T6P
.................aggucgugacuuUggcaauau..........................................................................   1     1    T6P
.................aggucgugacuugggAaauau..........................................................................   1     1    T6P
.................aggucgugacuugggcaauaug.........................................................................   137   0    T6P
.................aggucgugacuugggcaauauU.........................................................................   4     1    T6P
.................aggucgugacuugggcaauauA.........................................................................   1     1    T6P
.................aggucgugacuugggcaauaugA........................................................................   15    1    T6P
.................aggucgugacuugggcaauaugu........................................................................   2     0    T6P
.................aggucgugacuugggcaauauguccu.....................................................................   1     0    T6P
.......................................uccuaaaugauguaaaguaau....................................................   7     0    T6P
.......................................uccuaaaugauguaaaguaauc...................................................   8     0    T6P
.......................................uccuaaaugauguaaaguaaucu..................................................   9     0    T6P
.......................................uccuaaGugauguaaaguaaucu..................................................   1     1    T6P
.......................................uccuaaaugauguaaaguaaucugu................................................   1     0    T6P
.......................................uccuaaaugauguaaaguaaucuguu...............................................   15    0    T6P
.....................................................................Uuauugcacucgucccggccu......................   3     1    T6P
.....................................................................auauugcacucgucccggccugc....................   2     0    T6P
.....................................................................Uuauugcacucgucccggccugc....................   3     1    T6P
.....................................................................Uuauugcacucgucccggccugca...................   1     1    T6P
......................................................................uauugcacucgucccggc........................   1     0    T6P
......................................................................uauugcacucgucccggcA.......................   2     1    T6P
......................................................................uauuUcacucgucccggcc.......................   1     1    T6P
......................................................................uauugcacucgucccggcc.......................   30    0    T6P
......................................................................uauugcacucCucccggccu......................   1     1    T6P
......................................................................uauugcaUucgucccggccu......................   1     1    T6P
......................................................................uauugcacucgCcccggccu......................   1     1    T6P
......................................................................uauugcacucgucccUgccu......................   1     1    T6P
......................................................................uauugcacucgucccggccA......................   3     1    T6P
......................................................................uauuUcacucgucccggccu......................   1     1    T6P
......................................................................uauugcacucgucccgUccu......................   1     1    T6P
......................................................................uauugcacucgucccggccC......................   1     1    T6P
......................................................................uauugcacucguUccggccu......................   4     1    T6P
......................................................................uauuCcacucgucccggccu......................   2     1    T6P
......................................................................uauugcacucgucccgCccu......................   2     1    T6P
......................................................................uauugcacucgucccggUcu......................   1     1    T6P
......................................................................uauugUacucgucccggccu......................   2     1    T6P
......................................................................uauugcacucguccUggccu......................   2     1    T6P
```

```
                 Star                                                 Mature
auaagaguaacucuugaaggucgugacuugggcaauauguccuaaaugauguaaaguaaucuguuacccauauugcacucgucccggccugcaagggcuuccucaaaagcaa
......................................................................uauugcacucgucccgAccu......................    2      1    T6P
......................................................................uauugcacucgucccggccu......................    1894   0    T6P
......................................................................uauugcacucgucUcggccu......................    7      1    T6P
......................................................................uauugcCcucgucccggccu......................    1      1    T6P
......................................................................uauugcacuUgucccggccu......................    1      1    T6P
......................................................................uauugcacucgGcccggccug.....................    1      1    T6P
......................................................................uauugUacucgucccggccug.....................    1      1    T6P
......................................................................uauugcCcucgucccggccug.....................    1      1    T6P
......................................................................uauugcacucgucccggccGg.....................    3      1    T6P
......................................................................uauugcacucgucccggccuC.....................    3      1    T6P
......................................................................uauugcacucguUccggccug.....................    7      1    T6P
......................................................................uauugcacucgucccggccCg.....................    2      1    T6P
......................................................................Cauugcacucgucccggccug.....................    1      1    T6P
......................................................................uauugcacucgucccgAccug.....................    1      1    T6P
......................................................................uauugcacucAucccggccug.....................    2      1    T6P
......................................................................uauugcacuAgucccggccug.....................    3      1    T6P
......................................................................uauugcacucCucccggccug.....................    1      1    T6P
......................................................................uauugcacucguccUggccug.....................    1      1    T6P
......................................................................uauugcacucgucccggccug.....................    1312   0    T6P
......................................................................uauugcacucgucUcggccug.....................    4      1    T6P
......................................................................uauugcacucgucccggccuA.....................    5      1    T6P
......................................................................uauuCcacucgucccggccug.....................    1      1    T6P
......................................................................uauugcacucgucccggcUug.....................    1      1    T6P
......................................................................uauugcacucgucccgUccug.....................    1      1    T6P
......................................................................uauugcacucgucccggccuU.....................    143    1    T6P
......................................................................uauugcacucgucccggccAg.....................    1      1    T6P
......................................................................uauugcUcucgucccggccug.....................    1      1    T6P
......................................................................uauugcacucgucccAgccug.....................    1      1    T6P
......................................................................uauugcacucgucccggUcug.....................    3      1    T6P
......................................................................uauugcacucgucccCgccugc....................    5      1    T6P
......................................................................uauugcacucgucccggccugU....................    346    1    T6P
......................................................................uauugcCcucgucccggccugc....................    1      1    T6P
......................................................................uauugcGcucgucccggccugc....................    6      1    T6P
......................................................................uauugcacuAgucccggccugc....................    34     1    T6P
......................................................................uauugcacucgucccgAccugc....................    8      1    T6P
......................................................................uauugcacucCucccggccugc....................    2      1    T6P
......................................................................uauugcacucgucccggccugG....................    3      1    T6P
......................................................................uauugcacucgucccggcUugc....................    7      1    T6P
......................................................................uauugcacCcgucccggccugc....................    1      1    T6P
......................................................................uauugcacucgGcccggccugc....................    1      1    T6P
......................................................................uauugcacucgucccgUccugc....................    6      1    T6P
......................................................................uauugcacucguccGggccugc....................    1      1    T6P
......................................................................uauugcacucAucccggccugc....................    2      1    T6P
......................................................................uauugcacucgucccUgccugc....................    7      1    T6P
......................................................................uauugcacucgucccggccuAc....................    5      1    T6P
......................................................................uauugcacucguccUggccugc....................    12     1    T6P
......................................................................uauugcacucgucUcggccugc....................    15     1    T6P
......................................................................uauugcacucguUccggccugc....................    43     1    T6P
......................................................................uauugcacucgucccggccuUc....................    5      1    T6P
......................................................................uauuUcacucgucccggccugc....................    3      1    T6P
......................................................................uauugcacucgucccggccAgc....................    5      1    T6P
......................................................................uauugcacucgucccggUcugc....................    7      1    T6P
......................................................................uauugcacucgucccggccugA....................    28     1    T6P
......................................................................uauugcacucgucccggccCgc....................    2      1    T6P
......................................................................uauugcacucgucccggccGgc....................    10     1    T6P
......................................................................uauuAcacucgucccggccugc....................    2      1    T6P
......................................................................Gauugcacucgucccggccugc....................    2      1    T6P
......................................................................Cauugcacucgucccggccugc....................    1      1    T6P
......................................................................uauugcaUucgucccggccugc....................    4      1    T6P
......................................................................uauugcacucgucccggccugc....................    10930  0    T6P
......................................................................uauugcacuUgucccggccugc....................    5      1    T6P
......................................................................uauugcacucgucGcggccugc....................    3      1    T6P
......................................................................uauugcacucUucccggccugc....................    5      1    T6P
......................................................................uauugcacucgucccggccuCc....................    4      1    T6P
......................................................................uUuugcacucgucccggccugc....................    1      1    T6P
......................................................................uauugcacucgucccggcAugc....................    1      1    T6P
......................................................................uauugcacucgucccggAcugc....................    2      1    T6P
......................................................................uauugcacucgAcccggccugc....................    4      1    T6P
......................................................................uauugcacucgucccAgccugc....................    3      1    T6P
......................................................................uaCugcacucgucccggccugc....................    1      1    T6P
```

```
                Star                                              Mature
auaagaguaacucuugaaggucgugacuugggcaauauguccuaaaugauguaaaguaaucuguuacccauauugcacucgucccggccugcaagggcuuccucaaaagcaa
......................................................................uauugUacucgucccggccugc....................   7     1   T6P
......................................................................uauugcacucgCcccggccugc....................   1     1   T6P
......................................................................uauugcacucgucccgCccugc....................   5     1   T6P
......................................................................uauugUacucgucccggccugca...................   2     1   T6P
......................................................................uauugcacucgucccggccugcG...................   14    1   T6P
......................................................................uauugcacucgucccggccugUa...................   37    1   T6P
......................................................................uauugcacucgucccggccugca...................   805   0   T6P
......................................................................uauugcacucgucccggccugcU...................   1308  1   T6P
......................................................................uauugcacuAgucccggccugca...................   3     1   T6P
......................................................................uauugcacucgucccggccugcC...................   12    1   T6P
......................................................................uauugcacucgucccgUccugca...................   1     1   T6P
......................................................................uauugcacucgucccggccugAa...................   3     1   T6P
......................................................................uauugcacucgucUcggccugca...................   1     1   T6P
......................................................................Aauugcacucgucccggccugca...................   571   1   T6P
......................................................................uauugcacuUgucccggccugca...................   2     1   T6P
......................................................................uauugcacucguccUggccugca...................   2     1   T6P
......................................................................Aauugcacucgucccggccugcaa..................   9     1   T6P
......................................................................uauugcacucgucccggccugcaG..................   2     1   T6P
......................................................................uauugcacucgucccggccugcaa..................   20    0   T6P
......................................................................uauugcacucgucccggccugAaa..................   2     1   T6P
......................................................................uauugcacucgucccggccugcUa..................   19    1   T6P
......................................................................uauugcacucgucccggccugcaU..................   46    1   T6P
......................................................................uauugcacucgucccggccugcGa..................   1     1   T6P
......................................................................uauugcacucgucccggccugUaa..................   7     1   T6P
......................................................................uauugcacucgucccggccugcaC..................   1     1   T6P
......................................................................uauugcacucgucccggccugcCa..................   1     1   T6P
......................................................................uauugcacucgucccggccugcUag.................   1     1   T6P
.......................................................................auugcacucgucccggccu......................   7     0   T6P
.......................................................................auugcacucgucccUgccu......................   1     1   T6P
.......................................................................auugcacucgucccggccug.....................   11    0   T6P
.......................................................................Guugcacucgucccggccug.....................   2     1   T6P
.......................................................................auugcacucgucccggccugU....................   2     1   T6P
.......................................................................auugcacucgucccggccugc....................   354   0   T6P
.......................................................................auugcacucguccUggccugc....................   1     1   T6P
.......................................................................auugcacucgucccggccugA....................   1     1   T6P
.......................................................................auugcacucgucccgCccugc....................   1     1   T6P
.......................................................................auugcacuAgucccggccugc....................   1     1   T6P
.......................................................................auugcacucgucccggUcugc....................   1     1   T6P
.......................................................................auugcacucgucUcggccugc....................   2     1   T6P
.......................................................................auugcacucgucccgAccugc....................   1     1   T6P
.......................................................................auugcacucgucccggccugca...................   7     0   T6P
.......................................................................auugcacucgucccggccugcC...................   2     1   T6P
.......................................................................auugcacucgucccggccugcUa..................   1     1   T6P
........................................................................uugcacucgucccggccu......................   1     0   T6P
........................................................................uugcacucgucccggccugc....................   19    0   T6P
........................................................................uugcacucgucccggccugcC...................   2     1   T6P
........................................................................uugcacucgucccggccugca...................   6     0   T6P
........................................................................uugcacucgucccggccugcUa..................   10    1   T6P
........................................................................uugcacucgucccggccugcCa..................   2     1   T6P
.........................................................................ugcacucgucccggccugc....................   4     0   T6P
..........................................................................gcacucgucccggccugc....................   4     0   T6P
..........................................................................gcacucgucccggccugca...................   1     0   T6P

................aaggucgugacuugggcaauau..........................................................................   1     0   egg
................aaggucgugacuugggcaauauU.........................................................................   3     1   egg
.................aggucgugacuugggcaau............................................................................   6     0   egg
.................aggucgugacuugggcaaua...........................................................................   9     0   egg
.................aggGcgugacuugggcaauau..........................................................................   2     1   egg
.................aggucgugaUuugggcaauau..........................................................................   1     1   egg
.................aggucgugacuugggcaauaC..........................................................................   2     1   egg
.................aggCcgugacuugggcaauau..........................................................................   1     1   egg
.................aggucgugacuugggcaauau..........................................................................   130   0   egg
.................Nggucgugacuugggcaauau..........................................................................   1     1   egg
.................aggucgugacuugAgcaauau..........................................................................   1     1   egg
.................agguUgugacuugggcaauau..........................................................................   2     1   egg
.................aggucgugacuugggcaGuau..........................................................................   1     1   egg
.................aggucgugacuugggcaUuau..........................................................................   1     1   egg
.................aggAcgugacuugggcaauau..........................................................................   1     1   egg
.................aggucgugacuugggcaauaA..........................................................................   2     1   egg
.................aggucgugacuugggcaaGau..........................................................................   2     1   egg
```

Star Mature

```
auaagaguaacucuugaaggucgugacuugggcaauauguccuaaaugauguaaaguaaucuguuacccauauugcacucgucccggccugcaagggcuuccucaaaagcaa
.................aggucgugacuugggcaauauU.........................................................................   2     1   egg
.................aggucgugacCugggcaauaug.........................................................................   1     1   egg
.................aggucgugUcuugggcaauaug.........................................................................   2     1   egg
.................aggucgugGcuugggcaauaug.........................................................................   1     1   egg
.................aggucgugacuugggcaauauA.........................................................................   9     1   egg
.................aggucgugacuAgggcaauaug.........................................................................   1     1   egg
.................Uggucgugacuugggcaauaug.........................................................................   2     1   egg
.................aggAcgugacuugggcaauaug.........................................................................   3     1   egg
.................Nggucgugacuugggcaauaug.........................................................................   1     1   egg
.................agguGgugacuugggcaauaug.........................................................................   3     1   egg
.................aggCcgugacuugggcaauaug.........................................................................   2     1   egg
.................aggucgugacuCgggcaauaug.........................................................................   1     1   egg
.................aggucgugacuugggcaauaug.........................................................................   230   0   egg
.................aggucgugacuugggcaGuaug.........................................................................   1     1   egg
.................aggGcgugacuugggcaauaug.........................................................................   7     1   egg
.................agguUgugacuugggcaauaug.........................................................................   2     1   egg
.................aggucgugacuugggcaauaugA........................................................................   2     1   egg
.................aggucgugacuugggcaauaugu........................................................................   3     0   egg
.................aggucgugacuugggcaauaugC........................................................................   1     1   egg
.................aggucgugacuugggcaauauguU.......................................................................   1     1   egg
.................aggucgugacuugggcaauauguc.......................................................................   5     0   egg
.................aggucgugacuugggcaauauguccc......................................................................   1     0   egg
.................aggucgugacuugggcaauaugucA......................................................................   1     1   egg
.................aggucgugacuugggcaauauguccC.....................................................................   2     1   egg
.................aggucgugacuugggcaauauguccu.....................................................................   37    0   egg
.................aggucgugacuugggcaauaCguccu.....................................................................   1     1   egg
.................agguUgugacuugggcaauauguccu.....................................................................   1     1   egg
.................aggucgugacCugggcaauauguccu.....................................................................   1     1   egg
.................aggGcgugacuugggcaauauguccu.....................................................................   2     1   egg
.................aggucgugacuugggcaGuauguccua....................................................................   1     1   egg
.................aggGcgugacuugggcaauauguccua....................................................................   5     1   egg
.................Uggucgugacuugggcaauauguccua....................................................................   2     1   egg
.................aggucgugacuugggcaauauguccua....................................................................   127   0   egg
.................aggucgugacuugggcaauauguccGa....................................................................   2     1   egg
.................aggCcgugacuugggcaauauguccua....................................................................   2     1   egg
.................aggucgugacuugggcaauauguUcua....................................................................   1     1   egg
.................aggucgugacuugggcaauauguccCa....................................................................   1     1   egg
.................aggucgugacuugggcaauauguccuG....................................................................   1     1   egg
.................aggucgugacuugggcGauauguccua....................................................................   1     1   egg
.................aggucgugacuugggcaCuauguccua....................................................................   1     1   egg
.................aggucAugacuugggcaauauguccua....................................................................   1     1   egg
.................aggucgugacuugggcaauGuguccua....................................................................   1     1   egg
.................aggucgugacuugggcaauaCguccua....................................................................   2     1   egg
.................agguUgugacuugggcaauauguccua....................................................................   2     1   egg
.................aggAcgugacuugggcaauauguccua....................................................................   1     1   egg
.................aggucgugacCugggcaauauguccua....................................................................   1     1   egg
.................aggucgugacuugggcaauauguccuU....................................................................   4     1   egg
.................aggucgugacuugggcaauaugCccua....................................................................   2     1   egg
.......................................uccuaaaugauguaaaguaaucu..................................................   3     0   egg
.......................................uccuaaaugauguaaaguaaucCg.................................................   1     1   egg
.......................................uccCaaaugauguaaaguaaucuguu...............................................   1     1   egg
.......................................uccuaaaugauguaaaguaaucuguu...............................................   8     0   egg
.....................................................................auauugcacucgucccggccugc....................   7     0   egg
.....................................................................Cuauugcacucgucccggccugc....................   14    1   egg
.....................................................................auauugcacucgucccggccugU....................   1     1   egg
.....................................................................Cuauugcacucgucccggccugca...................   1     1   egg
.....................................................................auauugcacucgucccggccugcC...................   2     1   egg
.....................................................................auauugcacucgucccggccugcU...................   2     1   egg
......................................................................uauugcacucgucccggU........................   1     1   egg
......................................................................Nauugcacucgucccggc........................   1     1   egg
......................................................................uauugcacucgucccggc........................   4     0   egg
......................................................................uauugcacucgucccggcA.......................   9     1   egg
......................................................................uauugcacucgucccggcU.......................   4     1   egg
......................................................................uauugcacucgucccggcc.......................   10    0   egg
......................................................................uaCugcacucgucccggcc.......................   1     1   egg
......................................................................uauuUcacucgucccggcc.......................   1     1   egg
......................................................................Gauugcacucgucccggccu......................   3     1   egg
......................................................................uauugcacucgucccggcGu......................   1     1   egg
......................................................................uauGgcacucgucccggccu......................   10    1   egg
......................................................................uauugcacucgucccggccu......................   527   0   egg
```

Star Mature

```
auaagaguaacucuugaaggucgugacuugggcaauauguccuaaaugauguaaaguaaucuguuacccauauugcacucgucccggccugcaagggcuuccucaaaagcaa
......................................................................uauugcacucgucccggccC......................   35     1    egg
......................................................................uGuugcacucgucccggccu......................   1      1    egg
......................................................................uauugcGcucgucccggccu......................   2      1    egg
......................................................................uauugcacucgucccggccG......................   1      1    egg
......................................................................uauugcacucgGcccggccu......................   2      1    egg
......................................................................uUuugcacucgucccggccu......................   1      1    egg
......................................................................uauugcacucgucccAgccu......................   3      1    egg
......................................................................uauugcacuUgucccggccu......................   4      1    egg
......................................................................uauugcacucgucUcggccu......................   1      1    egg
......................................................................uauugcacucgucccggcUu......................   5      1    egg
......................................................................uauugAacucgucccggccu......................   1      1    egg
......................................................................uauCgcacucgucccggccu......................   4      1    egg
......................................................................uauugcacAcgucccggccu......................   1      1    egg
......................................................................uauugcacCcgucccggccu......................   1      1    egg
......................................................................uauAgcacucgucccggccu......................   1      1    egg
......................................................................uauuCcacucgucccggccu......................   3      1    egg
......................................................................uauugcacucgucccggUcu......................   1      1    egg
......................................................................uauugcacucgucccggccA......................   19     1    egg
......................................................................uauuUcacucgucccggccu......................   13     1    egg
......................................................................uauuAcacucgucccggccu......................   2      1    egg
......................................................................uauugcaUucgucccggccu......................   1      1    egg
......................................................................uauugUacucgucccggccug.....................   3      1    egg
......................................................................uauuAcacucgucccggccug.....................   7      1    egg
......................................................................uauugcGcucgucccggccug.....................   5      1    egg
......................................................................uauGgcacucgucccggccug.....................   22     1    egg
......................................................................uauugcacucgucccggUcug.....................   5      1    egg
......................................................................uauuCcacucgucccggccug.....................   1      1    egg
......................................................................uauugcacucUucccggccug.....................   1      1    egg
......................................................................uauCgcacucgucccggccug.....................   15     1    egg
......................................................................uauugcacucgucccgAccug.....................   1      1    egg
......................................................................uauugcacucgucccggAcug.....................   1      1    egg
......................................................................uauAgcacucgucccggccug.....................   7      1    egg
......................................................................uauuUcacucgucccggccug.....................   38     1    egg
......................................................................uauugcacucgucccggccug.....................   1516   0    egg
......................................................................uauugcacuUgucccggccug.....................   3      1    egg
......................................................................uauugGacucgucccggccug.....................   1      1    egg
......................................................................uGuugcacucgucccggccug.....................   6      1    egg
......................................................................uauugcacucAucccggccug.....................   3      1    egg
......................................................................Gauugcacucgucccggccug.....................   5      1    egg
......................................................................uauugcacucgucccggccCg.....................   8      1    egg
......................................................................uauugcacucgucccggccuU.....................   18     1    egg
......................................................................uauugcacAcgucccggccug.....................   1      1    egg
......................................................................uauugcaUucgucccggccug.....................   3      1    egg
......................................................................uauugcCcucgucccggccug.....................   1      1    egg
......................................................................Nauugcacucgucccggccug.....................   5      1    egg
......................................................................uauugcacucguccUggccug.....................   2      1    egg
......................................................................uauugcacucguUccggccug.....................   1      1    egg
......................................................................uauugcacucgucccggccuC.....................   7      1    egg
......................................................................uauugcacucgucccggccuA.....................   60     1    egg
......................................................................uaAugcacucgucccggccug.....................   1      1    egg
......................................................................uauugcacucgucccAgccug.....................   1      1    egg
......................................................................uauugcacucgucUcggccug.....................   7      1    egg
......................................................................uauugcacucgucccggcUug.....................   2      1    egg
......................................................................Cauugcacucgucccggccug.....................   5      1    egg
......................................................................uauugcUcucgucccggccug.....................   2      1    egg
......................................................................uauugcacucgucccggccAg.....................   1      1    egg
......................................................................uauugcacCcgucccggccug.....................   4      1    egg
......................................................................uauugAacucgucccggccug.....................   2      1    egg
......................................................................uaCugcacucgucccggccug.....................   5      1    egg
......................................................................uauugcacucgCcccggccug.....................   6      1    egg
......................................................................uCuugcacucgucccggccug.....................   3      1    egg
......................................................................uauuCcacucgucccggccugc....................   46     1    egg
......................................................................uauugcacucgucccggccuCc....................   4      1    egg
......................................................................uauugcacucgucccggccuAc....................   38     1    egg
......................................................................uauuAcacucgucccggccugc....................   126    1    egg
......................................................................uauugcacucguGccggccugc....................   3      1    egg
......................................................................uauugcacucgucccggccAgc....................   25     1    egg
......................................................................uGuugcacucgucccggccugc....................   62     1    egg
......................................................................Nauugcacucgucccggccugc....................   66     1    egg
......................................................................uaAugcacucgucccggccugc....................   10     1    egg
```

Star **Mature**

auaagaguaacucuugaaggucgugacuugggcaauauguccuaaaugauguaaaguaaucuguuacccauauugcacucgucccggccugcaagggcuuccucaaaagcaa			
....uauugGacucgucccggccugc....................	5	1	egg
....uauugcacucgucAcggccugc....................	9	1	egg
....uauugcacucgucGcggccugc....................	4	1	egg
....Gauugcacucgucccggccugc....................	48	1	egg
....uauugcacucgucccggccugG....................	7	1	egg
....uauugcacucgucccgAccugc....................	24	1	egg
....uauugcacucgucccUgccugc....................	16	1	egg
....Cauugcacucgucccggccugc....................	73	1	egg
....uauuUcacucgucccggccugc....................	863	1	egg
....uauugcacucguAccggccugc....................	3	1	egg
....uauugAacucgucccggccugc....................	29	1	egg
....uauugcacuUgucccggccugc....................	48	1	egg
....uauugcUcucgucccggccugc....................	29	1	egg
....uauugcacucgucccAgccugc....................	51	1	egg
....uauugcacucCucccggccugc....................	4	1	egg
....uauugcacucgucccggUcugc....................	37	1	egg
....uauugcacucgucccggccuUc....................	17	1	egg
....uauugcacucgCcccggccugc....................	110	1	egg
....uauugcacucgucccggcUugc....................	15	1	egg
....uauugcacucgucccggccGgc....................	5	1	egg
....uauugcacucgucccgCccugc....................	2	1	egg
....uauugcacucgGcccggccugc....................	15	1	egg
....uauugcacucgucUcggccugc....................	39	1	egg
....uauugcacucgucccgUccugc....................	6	1	egg
....uaGugcacucgucccggccugc....................	5	1	egg
....uauugcacucgucccggAcugc....................	4	1	egg
....uauCgcacucgucccggccugc....................	195	1	egg
....uauugcacucgucccggcAugc....................	1	1	egg
....uauugcacucUucccggccugc....................	6	1	egg
....uauAgcacucgucccggccugc....................	94	1	egg
....uauugcaAucgucccggccugc....................	2	1	egg
....uauugUacucgucccggccugc....................	37	1	egg
....uauugcacucgucccCgccugc....................	5	1	egg
....uauugcacucguccUggccugc....................	43	1	egg
....uauugcacAcgucccggccugc....................	16	1	egg
....uauugcCcucgucccggccugc....................	10	1	egg
....uauugcacucguUccggccugc....................	31	1	egg
....uauGgcacucgucccggccugc....................	498	1	egg
....uauugcacCcgucccggccugc....................	106	1	egg
....uauugcacucgAcccggccugc....................	7	1	egg
....uauugcGcucgucccggccugc....................	116	1	egg
....uCuugcacucgucccggccugc....................	32	1	egg
....uauugcacucAucccggccugc....................	39	1	egg
....uauugcacucgucccggccugU....................	1861	1	egg
....uauugcacGcgucccggccugc....................	3	1	egg
....uauugcacuGgucccggccugc....................	3	1	egg
....uauugcacucgucccggccugc....................	24758	0	egg
....uauugcacucguccGggccugc....................	10	1	egg
....uauugcacucguccAggccugc....................	5	1	egg
....uauugcaUucgucccggccugc....................	20	1	egg
....uUuugcacucgucccggccugc....................	24	1	egg
....uauugcacucgucccggccugA....................	617	1	egg
....uauugcacucgucccggGcugc....................	2	1	egg
....uaCugcacucgucccggccugc....................	84	1	egg
....uauugcacuAgucccggccugc....................	7	1	egg
....uauugcacucgucccggccCgc....................	96	1	egg
....uauugcacucguccAggccugca...................	2	1	egg
....uauAgcacucgucccggccugca...................	6	1	egg
....uauugcacucgucccgCccugca...................	1	1	egg
....uauugcacucAucccggccugca...................	4	1	egg
....uauugcacucgucccggccugUa...................	60	1	egg
....uauugcacucgucccggccugcG...................	22	1	egg
....uauugcacucgucccgAccugca...................	6	1	egg
....uauugcacucgucccggccCgca...................	2	1	egg
....uauugcacucgucccggccugAa...................	4	1	egg
....uaCugcacucgucccggccugca...................	6	1	egg
....uauuCcacucgucccggccugca...................	5	1	egg
....Gauugcacucgucccggccugca...................	3	1	egg
....uauugcacucguccGggccugca...................	1	1	egg
....uauugcacucgucccggccugca...................	2590	0	egg

```
                 Star                                                 Mature
auaagaguaacucuugaaggucgugacuugggcaauauguccuaaaugauguaaaguaaucuguuacccauauugcacucgucccggccugcaagggcuuccucaaaagcaa
......................................................................uauugcacucgucUcggccugca...................  3     1  egg
......................................................................uauugcacucgucccgUccugca...................  1     1  egg
......................................................................uauugcacucgucccCgccugca...................  1     1  egg
......................................................................uauugcacucgucccggAcugca...................  1     1  egg
......................................................................uauugcacucgucccggcUugca...................  5     1  egg
......................................................................uauugcacCcgucccggccugca...................  7     1  egg
......................................................................uauugcacucUucccggccugca...................  1     1  egg
......................................................................uauugcacuUgucccggccugca...................  5     1  egg
......................................................................uauugcacucgCcccggccugca...................  6     1  egg
......................................................................uauugcUcucgucccggccugca...................  1     1  egg
......................................................................Nauugcacucgucccggccugca...................  3     1  egg
......................................................................uauGgcacucgucccggccugca...................  44    1  egg
......................................................................uauugcGcucgucccggccugca...................  8     1  egg
......................................................................uauugcacucgAcccggccugca...................  1     1  egg
......................................................................uauugcacucgucccggccAgca...................  2     1  egg
......................................................................Aauugcacucgucccggccugca...................  831   1  egg
......................................................................uauugcacucgucccggccugcU...................  2640  1  egg
......................................................................uauugcacucgucccggccuCca...................  1     1  egg
......................................................................uauugcacucgucccggUcugca...................  3     1  egg
......................................................................uauugcacucgucAcggccugca...................  3     1  egg
......................................................................uCuugcacucgucccggccugca...................  2     1  egg
......................................................................uauugcacucgucccggccugcC...................  199   1  egg
......................................................................uauugUacucgucccggccugca...................  7     1  egg
......................................................................Cauugcacucgucccggccugca...................  3     1  egg
......................................................................uauugcacucguAccggccugca...................  1     1  egg
......................................................................uauugcacucgucccAgccugca...................  6     1  egg
......................................................................uauCgcacucgucccggccugca...................  29    1  egg
......................................................................uauugcacucguUccggccugca...................  7     1  egg
......................................................................uauugcacucguccUggccugca...................  3     1  egg
......................................................................uGuugcacucgucccggccugca...................  5     1  egg
......................................................................uauuUcacucgucccggccugca...................  92    1  egg
......................................................................uauugcaUucgucccggccugca...................  2     1  egg
......................................................................uauugcacuAgucccggccugca...................  1     1  egg
......................................................................uauugAacucgucccggccugca...................  1     1  egg
......................................................................uauugcacAcgucccggccugca...................  2     1  egg
......................................................................uaAugcacucgucccggccugca...................  1     1  egg
......................................................................uauuAcacucgucccggccugca...................  18    1  egg
......................................................................uauugcacucgucccggccuAca...................  5     1  egg
......................................................................uauugcacucgucccggccugcUa..................  30    1  egg
......................................................................uauuUcacucgucccggccugcaa..................  1     1  egg
......................................................................Aauugcacucgucccggccugcaa..................  5     1  egg
......................................................................uauugcacucgucccggccugcGa..................  1     1  egg
......................................................................uauugcacucgucccggccugcaa..................  11    0  egg
......................................................................uauugcacucgucccggccugcaU..................  17    1  egg
......................................................................uauugcacucgucccggccugcaG..................  3     1  egg
......................................................................uauugcacucgucccggccugcaaU.................  2     1  egg
......................................................................uauugcacucgucccggccugcaaC.................  2     1  egg
......................................................................uauugcacucgucccggccugcaaA.................  4     1  egg
.......................................................................auugcacucgucccggccug.....................  1     0  egg
.......................................................................auugcCcucgucccggccugc....................  2     1  egg
.......................................................................auugcacucgucccggccugc....................  46    0  egg
.......................................................................auuCcacucgucccggccugc....................  1     1  egg
.......................................................................auugcacucgucccCgccugc....................  1     1  egg
.......................................................................auugcacucgucccggccugU....................  4     1  egg
.......................................................................aCugcacucgucccggccugc....................  1     1  egg
.......................................................................auugcacucgucccggccuAc....................  1     1  egg
.......................................................................aAugcacucgucccggccugc....................  2     1  egg
.......................................................................auugcacucgucccggccugcC...................  4     1  egg
........................................................................uugcUcucgucccggccugc....................  1     1  egg
........................................................................uugcacucgucccggccugc....................  6     0  egg
........................................................................uugUacucgucccggccugc....................  1     1  egg
........................................................................uugcacucgucccggccugcC...................  3     1  egg
........................................................................uugcacucgCcccggccugca...................  1     1  egg
........................................................................uugcacucgucccggccugca...................  4     0  egg
........................................................................uugcacucgucccggccugcCa..................  1     1  egg
........................................................................uugcacucgucccggccugcaU..................  1     1  egg
........................................................................uugcacucgucccggccugcUa..................  17    1  egg

.................aggucgugacuugggcaau............................................................................  1     0  te1
.................aggucgugacuugggcaaua...........................................................................  1     0  te1
```

Star / Mature			
auaagaguaacucuugaaggucgugacuugggcaauauguccuaaaugauguaaaguaaucuguuacccauauugcacucgucccggccugcaagggcuuccucaaaagcaa			
.................Cggucgugacuugggcaauau..	1	1	te1
.................aggucgugacuugggcaauau..	9	0	te1
.................aggucgugacuugggcaauaug...	12	0	te1
....ccauauugcacucgucccgg.........................	1	0	te1
....Uuauugcacucgucccggccugc....................	1	1	te1
....Uuauugcacucgucccggccugca...................	1	1	te1
....uauugcacucgucccggc........................	3	0	te1
....uauugcacuUgucccggc........................	2	1	te1
....uauugcacucgucccggcc.......................	8	0	te1
....uauugcacuUgucccggcc.......................	3	1	te1
....uauugcacucgucccggccA......................	7	1	te1
....uauugcacucgAcccggccu......................	1	1	te1
....Gauugcacucgucccggccu......................	1	1	te1
....uauugcacucgucccggccG......................	7	1	te1
....uGuugcacucgucccggccu......................	2	1	te1
....uauugcacucUucccggccu......................	1	1	te1
....uauugcacucguccUggccu......................	2	1	te1
....uauugcacucCucccggccu......................	1	1	te1
....uauugcacuUgucccggccu......................	3	1	te1
....uauugcacucgucUcggccu......................	1	1	te1
....uauugUacucgucccggccu......................	1	1	te1
....uauugcacucguUccggccu......................	2	1	te1
....uauugcacucgucccgAccu......................	1	1	te1
....uauugcacucgucccggUcu......................	1	1	te1
....uauugcacucgucccggccu......................	766	0	te1
....uauCgcacucgucccggccu......................	1	1	te1
....uauugcacucgucccggUcug.....................	1	1	te1
....uauugcacucgucccggccug.....................	612	0	te1
....uauugcacucgGcccggccug.....................	1	1	te1
....uauugcacucguccUggccug.....................	1	1	te1
....uauugcacucgucccggccuC.....................	2	1	te1
....uauugcacucguUccggccug.....................	1	1	te1
....uauugcacucAucccggccug.....................	1	1	te1
....uauugcacucgucccggccAg.....................	4	1	te1
....uauugcacucgucUcggccug.....................	1	1	te1
....uauugUacucgucccggccug.....................	1	1	te1
....uauugcacucgucccggccuA.....................	1	1	te1
....uGuugcacucgucccggccug.....................	1	1	te1
....uauugcacucgucccggccGg.....................	59	1	te1
....uauugcacucgucccggccuU.....................	185	1	te1
....uauugcacucgucccgAccug.....................	1	1	te1
....uauugcacuUgucccggccug.....................	18	1	te1
....uauugcacucgucccggccAgc....................	2	1	te1
....uauugUacucgucccggccugc....................	2	1	te1
....uauugcacucUucccggccugc....................	1	1	te1
....uauugcacuUgucccggccugc....................	2	1	te1
....uauugcGcucgucccggccugc....................	1	1	te1
....uauuAcacucgucccggccugc....................	1	1	te1
....uauugcacucgucAcggccugc....................	1	1	te1
....uauugcacucgucccgUccugc....................	1	1	te1
....uauugcacucgucccggcAugc....................	2	1	te1
....uauugcacuAgucccggccugc....................	1	1	te1
....uauugcacucgucUcggccugc....................	5	1	te1
....uauugcacucgucccggccugA....................	28	1	te1
....uauugcacucAucccggccugc....................	3	1	te1
....uCuugcacucgucccggccugc....................	2	1	te1
....uauugcacucguUccggccugc....................	5	1	te1
....uauugcacucgucccggccugc....................	3677	0	te1
....uauugcacucgCcccggccugc....................	1	1	te1
....uauAgcacucgucccggccugc....................	2	1	te1
....uauugcacucgucccggccugU....................	227	1	te1
....uaAugcacucgucccggccugc....................	1	1	te1
....Gauugcacucgucccggccugc....................	1	1	te1
....uauugcacucguccUggccugc....................	1	1	te1
....uauugcacucgucccgAccugc....................	4	1	te1
....uauugcacucgucccggUcugc....................	3	1	te1
....uauugcacucgucccggccuAc....................	1	1	te1
....uauugcacucgucccggccGgc....................	1	1	te1
....uauugcacucgucccggccCgc....................	1	1	te1
....uGuugcacucgucccggccugc....................	4	1	te1

Star Mature

```
auaagaguaacucuugaaggucgugacuugggcaauauguccuaaaugauguaaaguaaucuguuacccauauugcacucgucccggccugcaagggcuuccucaaaagcaa
......................................................................Cauugcacucgucccggccugc....................    5    1    te1
......................................................................uauugcacucgucccggccuCc....................    8    1    te1
......................................................................uauugcacucgucccAgccugc....................    1    1    te1
......................................................................uauugcacucgucccggccugG....................    6    1    te1
......................................................................uauugcacucgucccggccuUc....................    3    1    te1
......................................................................Cauugcacucgucccggccugca...................    1    1    te1
......................................................................uauugcacucgucccggccugcC...................    3    1    te1
......................................................................uauugcacucgucccggccugcU...................    267  1    te1
......................................................................uauugcacucgucccggccugAa...................    10   1    te1
......................................................................uauugcacucgucccggccugcG...................    12   1    te1
......................................................................uauugcacucgucccggccugUa...................    25   1    te1
......................................................................Aauugcacucgucccggccugca...................    153  1    te1
......................................................................uauugcacucgucccggccugca...................    245  0    te1
......................................................................uauugcacucgucccggccugcUa..................    4    1    te1
......................................................................uauugcacucgucccggccugcaU..................    15   1    te1
......................................................................uauugcacucgucccggccugcaa..................    6    0    te1
......................................................................uauugcacucgucccggccugUaa..................    11   1    te1
......................................................................Aauugcacucgucccggccugcaa..................    5    1    te1
......................................................................uauugcacucgucccggccugAaa..................    3    1    te1
......................................................................uauugcacucgucccggccugcaaU.................    2    1    te1
.......................................................................auugcacucgucccggccu......................    5    0    te1
.......................................................................auugcacucgucccggccug.....................    20   0    te1
.......................................................................auugcacucgucccggccuU.....................    1    1    te1
.......................................................................auugcacucgucccAgccugc....................    1    1    te1
.......................................................................auugcacucgucccggccugc....................    623  0    te1
.......................................................................Uuugcacucgucccggccugc....................    1    1    te1
.......................................................................auugcacucgucccggccugU....................    3    1    te1
.......................................................................auugcacucgucUcggccugc....................    1    1    te1
.......................................................................auuAcacucgucccggccugc....................    1    1    te1
.......................................................................auugcacucgucccgAccugc....................    1    1    te1
.......................................................................Guugcacucgucccggccugc....................    1    1    te1
.......................................................................auugcacucgucccggccGgc....................    1    1    te1
.......................................................................auugcacucgucccCgccugc....................    1    1    te1
.......................................................................auugcacucCucccggccugc....................    1    1    te1
.......................................................................aAugcacucgucccggccugc....................    2    1    te1
.......................................................................auugcacucgucccggccugcC...................    2    1    te1
.......................................................................auugcacucgucccggccugca...................    11   0    te1
........................................................................uugcacucgucccggccugc....................    16   0    te1
........................................................................uugcacucgucccggccugca...................    3    0    te1
........................................................................uugcacucgucccggccugcUa..................    2    1    te1
.........................................................................ugcacucgucccggccugc....................    3    0    te1
..........................................................................gcacucgucccggccugc....................    4    0    te1
..........................................................................gcacucgucccggccugU....................    1    1    te1
..........................................................................gcacucgucccggccugca...................    1    0    te1

................aaggucgugacuugggcaauaug.........................................................................    3    0    T53
.................aggucgugacuugggcaau............................................................................    3    0    T53
.................aggucgugacuugggcaaua...........................................................................    12   0    T53
.................aggucgugacuugggcaauau..........................................................................    45   0    T53
.................aggAcgugacuugggcaauau..........................................................................    1    1    T53
.................aggucgugacuugggcaauauA.........................................................................    2    1    T53
.................aggucgugacuugggcaauauU.........................................................................    1    1    T53
.................agAucgugacuugggcaauaug.........................................................................    1    1    T53
.................aggAcgugacuugggcaauaug.........................................................................    1    1    T53
.................aggucgugacuugggcaauaug.........................................................................    52   0    T53
.................aggucgugCcuugggcaauaug.........................................................................    1    1    T53
.................aggucgugacuugggcaauaugA........................................................................    2    1    T53
.................aggucgugacuugggcaauauguccu.....................................................................    1    0    T53
.......................................uccuaaaugauguaaaguaauc...................................................    3    0    T53
.......................................uccuaaaugauguaaaguaaucu..................................................    2    0    T53
.............................................................uguuacccauauugcacucguc.............................    1    0    T53
...............................................................uuacccauauugcacucgAcc............................    1    1    T53
.....................................................................Uuauugcacucgucccggccugc....................    3    1    T53
......................................................................uauugcacucgucccggc........................    2    0    T53
......................................................................uauugcacucgucccggU........................    2    1    T53
......................................................................uauugcacuUgucccggcc.......................    12   1    T53
......................................................................uauugcacucgucccggcA.......................    1    1    T53
......................................................................uauugcacucgucccggcc.......................    21   0    T53
......................................................................uauugcacucgCcccggccu......................    1    1    T53
......................................................................uauugcacucgucccggccG......................    1    1    T53
```

```
                 Star                                               Mature
auaagaguaacucuugaaggucgugacuugggcaauauguccuaaaugauguaaaguaaucuguuacccauauugcacucgucccggccugcaagggcuuccucaaaagcaa
......................................................................uauugcacucguccUggccu......................    3      1    T53
......................................................................uauugcacucgucccggcUu......................    1      1    T53
......................................................................uauugcacuUgucccggccu......................    20     1    T53
......................................................................uauugAacucgucccggccu......................    1      1    T53
......................................................................uauugcacucCucccggccu......................    1      1    T53
......................................................................uauugcacucgucccggUcu......................    3      1    T53
......................................................................uauugcacucgucUcggccu......................    4      1    T53
......................................................................uauugUacucgucccggccu......................    3      1    T53
......................................................................uauugcacucguUccggccu......................    9      1    T53
......................................................................uauugcacucgucccggccu......................    642    0    T53
......................................................................uauugcacucgucccggccA......................    2      1    T53
......................................................................Gauugcacucgucccggccu......................    1      1    T53
......................................................................uauugcacucgucccggcUug.....................    1      1    T53
......................................................................uauugcacucgucccgAccug.....................    1      1    T53
......................................................................uauugcacucguUccggccug.....................    2      1    T53
......................................................................uauugcacucgucccggccug.....................    219    0    T53
......................................................................Cauugcacucgucccggccug.....................    1      1    T53
......................................................................uauugcacucgucccgCccug.....................    1      1    T53
......................................................................uauugcacucgucccggccAg.....................    1      1    T53
......................................................................uauugcacucgucccggccuU.....................    57     1    T53
......................................................................uauCgcacucgucccggccug.....................    1      1    T53
......................................................................uauugcacucgucccAgccug.....................    1      1    T53
......................................................................uauugcacuUgucccggccug.....................    69     1    T53
......................................................................uauugcacucgucccggccuA.....................    6      1    T53
......................................................................uauugcacucCucccggccugc....................    1      1    T53
......................................................................uauugUacucgucccggccugc....................    4      1    T53
......................................................................uauugcacucgucccgAccugc....................    2      1    T53
......................................................................uauugcacAcgucccggccugc....................    2      1    T53
......................................................................uauugcacucgucccggccugA....................    11     1    T53
......................................................................uauugcacucgucUcggccugc....................    5      1    T53
......................................................................uauugcacucUucccggccugc....................    1      1    T53
......................................................................uauugcacucgucccgCccugc....................    1      1    T53
......................................................................uauugcacucgucccggccuAc....................    7      1    T53
......................................................................uauugcacucgucccggccugG....................    1      1    T53
......................................................................uauugcacCcgucccggccugc....................    1      1    T53
......................................................................uauugcacucgucccggccGgc....................    1      1    T53
......................................................................uauuAcacucgucccggccugc....................    1      1    T53
......................................................................uauugcacucgucccgUccugc....................    3      1    T53
......................................................................uCuugcacucgucccggccugc....................    1      1    T53
......................................................................uauugcacucgucccggccCgc....................    2      1    T53
......................................................................uauugcacucgucccAgccugc....................    1      1    T53
......................................................................uauugcacuUgucccggccugc....................    2      1    T53
......................................................................uauugcacuAgucccggccugc....................    1      1    T53
......................................................................uauugcacucgucccggUcugc....................    1      1    T53
......................................................................uauugcacucgucccggccugU....................    197    1    T53
......................................................................uauugcacucgGcccggccugc....................    1      1    T53
......................................................................uauugcacucgucccggcUugc....................    2      1    T53
......................................................................uauugcacucgucccggccuUc....................    25     1    T53
......................................................................uauugcacucgucccggccuCc....................    3      1    T53
......................................................................uauugcacucguUccggccugc....................    7      1    T53
......................................................................uauugcacucgucccCgccugc....................    1      1    T53
......................................................................uauCgcacucgucccggccugc....................    1      1    T53
......................................................................uauugGacucgucccggccugc....................    1      1    T53
......................................................................uauugcacucguccUggccugc....................    4      1    T53
......................................................................uauugcacucgucccggccugc....................    2563   0    T53
......................................................................uauugcacucgucccggccugUa...................    17     1    T53
......................................................................uauugcacucgucccggccugcC...................    4      1    T53
......................................................................Aauugcacucgucccggccugca...................    73     1    T53
......................................................................uauugcacucgucccggccugcU...................    248    1    T53
......................................................................uauugcacucgucccggccugAa...................    5      1    T53
......................................................................uauugcacucgucccggccugcG...................    7      1    T53
......................................................................uauugcacucgucccggcUugca...................    1      1    T53
......................................................................uauugcacucgucccggccugca...................    132    0    T53
......................................................................uauugcacucgucccggccugUaa..................    7      1    T53
......................................................................uauugcacucgucccggccugcUa..................    2      1    T53
......................................................................uauugcacucgucccggccugcaa..................    1      0    T53
......................................................................uauugcacucgucccggccugcaU..................    12     1    T53
......................................................................Aauugcacucgucccggccugcaa..................    1      1    T53
......................................................................uauugcacucgucccggccugcaaU.................    4      1    T53
.......................................................................auugcacucgucccggccug.....................    1      0    T53
```

Star Mature

```
auaagaguaacucuugaaggucgugacuugggcaauauguccuaaaugauguaaaguaaucuguuacccauauugcacucgucccggccugcaagggcuuccucaaaagcaa
.......................................................................auugcacuUgucccggccug.....................	2	1	T53
.......................................................................auugcacucgucccggccugc....................	102	0	T53
.......................................................................auugcacucgucccggccugA....................	1	1	T53
.......................................................................auugcacucguccGggccugc....................	1	1	T53
.......................................................................auugcacucgucccggccugU....................	1	1	T53
.......................................................................auugcacucgucccggccuUc....................	1	1	T53
.......................................................................auugUacucgucccggccugc....................	1	1	T53
.......................................................................auugcacucgucccggccugca...................	3	0	T53
.......................................................................auugcacucgucccggccugcC...................	1	1	T53
........................................................................uugcacucgucccggccugc....................	5	0	T53
........................................................................uugcacucgucccggccugca...................	2	0	T53
........................................................................uugcacucgucccggccugcC...................	4	1	T53
........................................................................uugcacucgucccggccugcUa..................	3	1	T53
........................................................................uugcacucgucccggccugcCa..................	1	1	T53
.........................................................................ugcacucgucccggccugc....................	1	0	T53
............................................................................acucgucccggccugcaaU.................	1	1	T53
```
