# Supplementary material for: Millipede genomes reveal unique adaptations during myriapod evolution
Source: PLoS Biol. 2020 Sep 29;18(9):e3000636. doi: 10.1371/journal.pbio.3000636 (PMC7523956; doi:10.1371/journal.pbio.3000636)
Supplement: S5 Fig — (PDF) [file pbio.3000636.s005.pdf]

Trigoniulus corallinus alignment		NUUHUUUUUHNUUHUUUUUUUUUHUHHUUUUNUHUNXUNUUUUHUUUUUUUUUUUNUUNH
Strigamia maritima alignment		XUUNUUUUUNNUU?UUUUUUUUUHUHHUUUUNUXU??UUUUUUHUUUUUUUUUUUNUUNN
Trigoniulus corallinus	Hox3a	FKRARTAYTSSQLVELEKEFHFNRYLCRPRRMEMAKMLHLTERQIKIWFQNRRMKLKKGQ
Trigoniulus corallinus	Hox3b	....
Strigamia maritima	Hox3a	N..S.....Q...L.................L.L.SL.N................I..IK
Strigamia maritima	Hox3b	N..S.....Q.....................V.L.S..N................N..IK
Branchiostoma lanceolatum	Xlox	N..T.....RG..L.........K.IS....I.L.A..N....H...........W..E.
Archaster typicus	Xlox	N..T.....RG..L.........K.IS....I.L.A..N....H...........W..EE
Strongylocentrotus purpuratus	Xlox	N..T.....RG..L.........K.IS....I.L.A..N....H...........W..EE
Platynereis dumerilii	Xlox	N..T.....R...L.........K.IS....I.L.S..S....H...........W..DE
Capitella teleta	Xlox	N..T.....RA..L...........IT....V.L.AH.N...QH...........W..DV
Nucula tumidula	Xlox	N..T.....RG..L.........K.IS....I.L.A..N....H...........W..DE
Crassostrea gigas	Xlox	N..T.....RG..L.........K.IS....I.L.A..N....H...........W..DE
Branchiostoma floridae	Hox3	G.........A....................V...A..N................Y..E.
Strongylocentrotus purpuratus	Hox3	P..N...F..A....................V....S.N................Y.RDM
Capitella teleta	Hox3	S.........A....................I...AL.N................Y..D.
Crassostrea gigas	Hox3	T.........A....................I...AL.S................F..E.
Daphnia pulex	Hox3	A.........A....................I...TL.N................F..E.
Parasteatoda tepidariorum	Hox3	A.........A....................I...SL.N................Y..E.
Stegodyphus mimosarum	Hox3	S.........A....................I...SL.N................Y..E.
Cupiennius salei	Hox3	A.........A....................I...NL.N.S..............Y..E.
Priapulus caudatus	Hox3	S.........A....................I...A..N................Y..D.
Centruroides sculpturatus	Hox3	I.........A....................I...AL.N................F..E.
Orchesella cincta	Hox3	T.........A....................I...A..N................Y..E.
Euperipatoides kanangrensis	Hox3	A.........A....................I...AL.N.S..............Y..D.
Folsomia candida	Hox3	T.........A..........Y.........I...SL.S................Y..EL
Archegozetes longisetosus	Hox3	A....................TS........I...SL.K.S..............F..E.
