# Supplementary material for: Millipede genomes reveal unique adaptations during myriapod evolution
Source: PLoS Biol. 2020 Sep 29;18(9):e3000636. doi: 10.1371/journal.pbio.3000636 (PMC7523956; doi:10.1371/journal.pbio.3000636)
Supplement: S20 Fig — (PDF) [file pbio.3000636.s020.pdf]

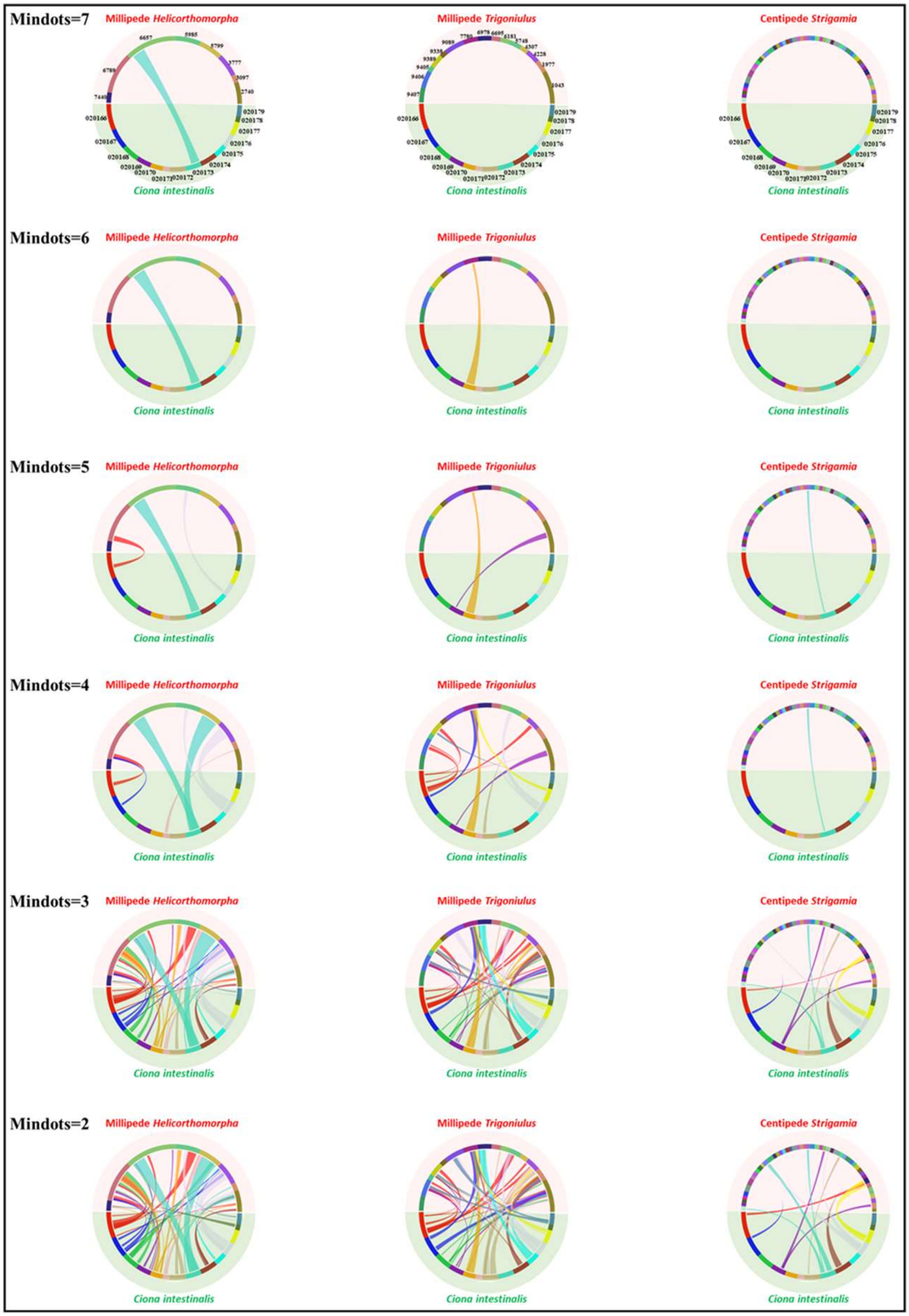

Mindots=7
Millipede Helicorthomorpha
Millipede Trigoniulus
Centipede Strigamia
6657
5985
5799
6789
2740
020166
020167
020168
020169
020170
020171
020172
020173
020174
020175
020176
020177
020178
020179
Ciona intestinalis
Mindots=6
Mindots=5
Mindots=4
Mindots=3
Mindots=2
