# Supplementary material for: Millipede genomes reveal unique adaptations during myriapod evolution
Source: PLoS Biol. 2020 Sep 29;18(9):e3000636. doi: 10.1371/journal.pbio.3000636 (PMC7523956; doi:10.1371/journal.pbio.3000636)
Supplement: S5 Data — (PDF) [file pbio.3000636.s033.pdf]

Provisional ID	:	SczTNLB_1880_1366
Score total	:	103438.1
Score for star read(s)	:	3.9
Score for read counts	:	103428.3
Score for mfe	:	1.2
Score for randfold	:	1.6
Score for cons. seed	:	3
Total read count	:	202882
Mature read count	:	202764
Loop read count	:	0
Star read count	:	118

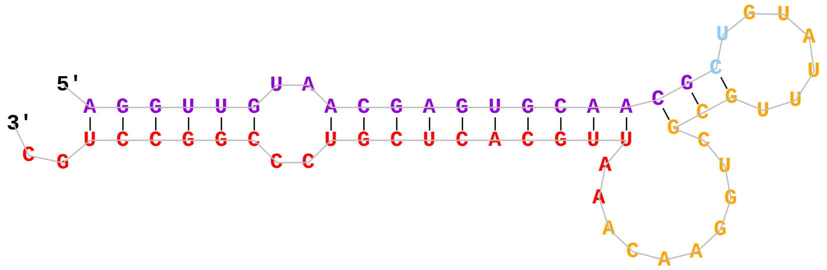

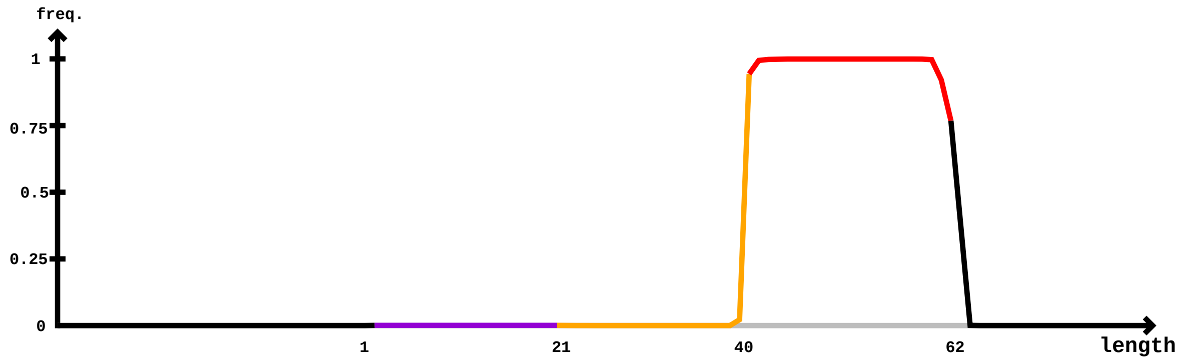

Star Mature

```
5'- uaaaccggauguuuaaaaaggcaaaagugacagguuguaacgagugcaacgcuguauuugcgcuggaacaaauugcacucgucccggccugcccacccuacauugcauaaac   -3'   obs
    uaaaccggauguuuaaaaaggcaaaagugacagguuguaacgagugcaacgcuguauuugcgcuggaacaaauugcacucgucccggccugcccacccuacauugcauaaac         exp
    (((((.....))))).....((((..(((.(((((((..(((((((((((((.......)))..........))))))))))..)))))))..)))......))))......   reads   mm   sample
    ...............................agguuguaacgagugcaacg.............................................................   3       0    HS6
    ...............................agguuguaacgagugcaacgcugu.........................................................   1       0    HS6
    .....................................................................aaauugcacucgucccggcc.......................   3       0    HS6
    .....................................................................aaauugcacucguUccggccu......................   1       1    HS6
    .....................................................................aaauugcacucgucccggccu......................   47      0    HS6
    .....................................................................aaauugcacucguccUggccu......................   2       1    HS6
    .....................................................................aaauugcacucgucccggccug.....................   79      0    HS6
    .....................................................................aaauugcacucgucccggccuA.....................   1       1    HS6
    .....................................................................aaauugcacucgucccggccuU.....................   2       1    HS6
    .....................................................................Uaauugcacucgucccggccug.....................   1       1    HS6
    .....................................................................aaauugcacucgucccggccGg.....................   9       1    HS6
    .....................................................................Uaauugcacucgucccggccugc....................   7       1    HS6
    .....................................................................aaauugcacucgucccggccugU....................   23      1    HS6
    .....................................................................aaauugcacucgucccggccugc....................   89      0    HS6
    .....................................................................aaauugcacucgucccggccuUc....................   1       1    HS6
    .....................................................................aaauugcacucgucccggccugcA...................   2       1    HS6
    ......................................................................aauugcacucgucccggc........................   2       0    HS6
    ......................................................................aauugcacucgucccggcc.......................   42      0    HS6
    ......................................................................aauugcacucgucccggcA.......................   3       1    HS6
    ......................................................................aauugcacucguccUggccu......................   3       1    HS6
    ......................................................................aauugcacucgucccUgccu......................   1       1    HS6
    ......................................................................aauugcaUucgucccggccu......................   1       1    HS6
    ......................................................................aauuUcacucgucccggccu......................   1       1    HS6
    ......................................................................aauugcacucgucccggccG......................   5       1    HS6
    ......................................................................aaCugcacucgucccggccu......................   1       1    HS6
    ......................................................................aauugcacucgucUcggccu......................   3       1    HS6
    ......................................................................aauugcacucguUccggccu......................   2       1    HS6
    ......................................................................aauugcacucgucccgCccu......................   1       1    HS6
    ......................................................................aCuugcacucgucccggccu......................   1       1    HS6
    ......................................................................aauugcacucgucccggccA......................   4       1    HS6
    ......................................................................aauugcacucgucccggccu......................   974     0    HS6
    ......................................................................aauugcacucgucccggUcu......................   1       1    HS6
    ......................................................................aauugcacucgucccggccug.....................   1950    0    HS6
```

Star Mature

```
uaaaccggauguuuaaaaaggcaaaagugacagguuguaacgagugcaacgcuguauuugcgcuggaacaaauugcacucgucccggccugcccacccuacauugcauaaac
......................................................................aauugcacucguccUggccug.....................   3     1   HS6
......................................................................aauugcacucgucccggccAg.....................   6     1   HS6
......................................................................aauugcacucgucccggcAug.....................   1     1   HS6
......................................................................aauugcacucgucccggccuU.....................   17    1   HS6
......................................................................aauugcacucgucUcggccug.....................   11    1   HS6
......................................................................aauuUcacucgucccggccug.....................   2     1   HS6
......................................................................aauugcacucgucccggccuC.....................   2     1   HS6
......................................................................aauuAcacucgucccggccug.....................   1     1   HS6
......................................................................aUuugcacucgucccggccug.....................   1     1   HS6
......................................................................aauugcacucguUccggccug.....................   9     1   HS6
......................................................................aauugcacucgucccggccGg.....................   279   1   HS6
......................................................................aauugcacucgucccggUcug.....................   1     1   HS6
......................................................................aaCugcacucgucccggccug.....................   2     1   HS6
......................................................................Gauugcacucgucccggccug.....................   2     1   HS6
......................................................................aauugcacucgucccggccuA.....................   3     1   HS6
......................................................................aauugcacucgucccggccCg.....................   2     1   HS6
......................................................................aauugcacucgucccgAccug.....................   3     1   HS6
......................................................................aauugcacucgucccggccGgc....................   3     1   HS6
......................................................................aauugcacucAucccggccugc....................   1     1   HS6
......................................................................aauugcacucgucccggAcugc....................   1     1   HS6
......................................................................aauugcacucgucUcggccugc....................   22    1   HS6
......................................................................aauugcacucgucccAgccugc....................   2     1   HS6
......................................................................Gauugcacucgucccggccugc....................   3     1   HS6
......................................................................aauugcacucgucccggUcugc....................   8     1   HS6
......................................................................aauugcacucgucccggccugc....................   6188  0   HS6
......................................................................aauugcacucgucccggccugG....................   22    1   HS6
......................................................................aauugcacucgucccUgccugc....................   2     1   HS6
......................................................................aauugcCcucgucccggccugc....................   1     1   HS6
......................................................................aauugcacucgucccgAccugc....................   6     1   HS6
......................................................................aauugcacucguUccggccugc....................   32    1   HS6
......................................................................aauugcacucgucccggccugU....................   429   1   HS6
......................................................................aauugcacucgucccggccuAc....................   6     1   HS6
......................................................................aauugcacucgCcccggccugc....................   2     1   HS6
......................................................................aauCgcacucgucccggccugc....................   5     1   HS6
......................................................................aauugcacucgucccggccuUc....................   2     1   HS6
......................................................................aGuugcacucgucccggccugc....................   2     1   HS6
......................................................................aauugcacucgucccCgccugc....................   2     1   HS6
......................................................................aUuugcacucgucccggccugc....................   1     1   HS6
......................................................................aaGugcacucgucccggccugc....................   1     1   HS6
......................................................................aauugcacucgucccggcGugc....................   2     1   HS6
......................................................................aauugUacucgucccggccugc....................   4     1   HS6
......................................................................aauuUcacucgucccggccugc....................   3     1   HS6
......................................................................aauugcGcucgucccggccugc....................   2     1   HS6
......................................................................aauugcacCcgucccggccugc....................   1     1   HS6
......................................................................aauuAcacucgucccggccugc....................   1     1   HS6
......................................................................aauugcacucgucccggccugA....................   18    1   HS6
......................................................................aauugcacucgucccggccuCc....................   1     1   HS6
......................................................................aauugcaUucgucccggccugc....................   4     1   HS6
......................................................................aauugcacucgucccggccCgc....................   3     1   HS6
......................................................................aauugcacucguccUggccugc....................   2     1   HS6
......................................................................aauugcacucgucccggccugUc...................   1     1   HS6
......................................................................aauugcacucgucccggccugcG...................   8     1   HS6
......................................................................aauugcacucgucccggccugcc...................   3     0   HS6
......................................................................Uauugcacucgucccggccugcc...................   12    1   HS6
......................................................................aauugcacucgucccggccugcA...................   321   1   HS6
.......................................................................auugcacucgucccggccu......................   11    0   HS6
.......................................................................auugcacucgucccggccGg.....................   10    1   HS6
.......................................................................auugcacucgucccggccug.....................   38    0   HS6
.......................................................................auugUacucgucccggccug.....................   1     1   HS6
.......................................................................auugcacucgucccggUcugc....................   1     1   HS6
.......................................................................auugcacucgucccggccugG....................   2     1   HS6
.......................................................................auuCcacucgucccggccugc....................   2     1   HS6
.......................................................................auugcacucgucUcggccugc....................   2     1   HS6
.......................................................................auugcacucgucAcggccugc....................   1     1   HS6
.......................................................................aAugcacucgucccggccugc....................   1     1   HS6
.......................................................................auugcacucgucccggccugc....................   343   0   HS6
.......................................................................auugcacucgucccggccugU....................   9     1   HS6
.......................................................................auugUacucgucccggccugc....................   1     1   HS6
.......................................................................auugcacucgucccggccugcA...................   24    1   HS6
.......................................................................auugcacucgucccgAccugcc...................   1     1   HS6
```

Star **Mature**

```
uaaaccggauguuuaaaaaggcaaaagugacagguuguaacgagugcaacgcuguauuugcgcuggaacaaauugcacucgucccggccugcccacccuacauugcauaaac
.......................................................................auugcacucgucccggccugcc...................    22    0    HS6
.......................................................................auugcacucgucccggccugccU..................    3     1    HS6
........................................................................uugcacucgucccggccu......................    2     0    HS6
........................................................................uugcacucgucccggccug.....................    3     0    HS6
........................................................................Augcacucgucccggccugc....................    1     1    HS6
........................................................................uugcacucgucccggccugU....................    1     1    HS6
........................................................................uugcacucgucccggccugc....................    26    0    HS6
........................................................................uugcacucgucccggccugcc...................    1     0    HS6
.........................................................................ugcacucgucccggccug.....................    5     0    HS6
.........................................................................ugcacucgucccggccugU....................    1     1    HS6
.........................................................................ugcacucgucccgAccugc....................    1     1    HS6
.........................................................................ugcacucgucccggccugc....................    11    0    HS6
.........................................................................ugcacucgucccggccugcA...................    3     1    HS6
..........................................................................gcacucgucccggccugG....................    1     1    HS6
..........................................................................gcacucgucccggccugc....................    20    0    HS6
..........................................................................gcacucgucccggccugU....................    6     1    HS6
..........................................................................gcacucgucccggccugcA...................    3     1    HS6

......................................................................aauugcacucgucccggccu......................    36    0    HS2
......................................................................aauugcacucgucccggccugc....................    390   0    HS2
......................................................................aauugcacucgucccgAccugc....................    4     1    HS2
......................................................................aauugcacucgucccggUcugc....................    2     1    HS2
......................................................................aauugcacucgucccggccugcA...................    1     1    HS2
.......................................................................auugcacucgucccggccugc....................    216   0    HS2
.......................................................................auuUcacucgucccggccugc....................    1     1    HS2
.......................................................................auugcacucgucccggccugcc...................    2     0    HS2
.......................................................................auugcacucgucccggccugcA...................    1     1    HS2

...............................agguuguaacgagugcaaA..............................................................    2     1    HA1
...............................agguuguaacgagugcaacg.............................................................    38    0    HA1
...............................agguuguaacgagugcGacg.............................................................    1     1    HA1
...............................agguuguaacgagugcaacgc............................................................    2     0    HA1
...............................agguuguaacgagugcaacgcu...........................................................    3     0    HA1
...............................agguuguaacgagugcaacgcuU..........................................................    1     1    HA1
...............................agguuguaacgagugcaacgcugA.........................................................    1     1    HA1
...............................agguuguaacgagugcaacgcugu.........................................................    2     0    HA1
...............................agguuguaacgagugcaacgcuUu.........................................................    1     1    HA1
....................................................................Uaaauugcacucgucccggccu......................    1     1    HA1
....................................................................Aaaauugcacucgucccggccugc....................    1     1    HA1
.....................................................................aaauugcacucgucccggcc.......................    3     0    HA1
.....................................................................aaauugcacucgucccgAccu......................    1     1    HA1
.....................................................................aaauugcacucgucccggccA......................    1     1    HA1
.....................................................................aaauugcacucgucccggccu......................    79    0    HA1
.....................................................................Uaauugcacucgucccggccu......................    6     1    HA1
.....................................................................aaauugcacucgucccggccuA.....................    1     1    HA1
.....................................................................aaauugcacucgucccggUcug.....................    1     1    HA1
.....................................................................Uaauugcacucgucccggccug.....................    3     1    HA1
.....................................................................aaauugcacucgucccggccuU.....................    2     1    HA1
.....................................................................aaaGugcacucgucccggccug.....................    1     1    HA1
.....................................................................aaauugcacucgucccggccGg.....................    3     1    HA1
.....................................................................aaauugcacucgucccggccug.....................    64    0    HA1
.....................................................................aaauugcacucgucccgAccugc....................    1     1    HA1
.....................................................................aaauugcacucgucccggccugU....................    42    1    HA1
.....................................................................aaauugcacucgucccggccugc....................    96    0    HA1
.....................................................................aaauugcacucgucUcggccugc....................    1     1    HA1
.....................................................................Uaauugcacucgucccggccugc....................    19    1    HA1
.....................................................................aaauCgcacucgucccggccugc....................    1     1    HA1
.....................................................................aaauuAcacucgucccggccugc....................    1     1    HA1
.....................................................................aaauugcacucgucccggccugcA...................    3     1    HA1
.....................................................................aaauugcacucgucccggccugcc...................    1     0    HA1
......................................................................aauugcacucgucccggc........................    5     0    HA1
......................................................................aauugcacucgucccggA........................    2     1    HA1
......................................................................aauugcacucgucccggcA.......................    2     1    HA1
......................................................................aauugcacucgucccgUcc.......................    1     1    HA1
......................................................................aauugcacucgucccggcc.......................    61    0    HA1
......................................................................aauugcacucgucccggcU.......................    1     1    HA1
......................................................................aauugcaGucgucccggccu......................    1     1    HA1
......................................................................aauugcacucgucccggccC......................    2     1    HA1
......................................................................aauugcUcucgucccggccu......................    1     1    HA1
......................................................................aaAugcacucgucccggccu......................    1     1    HA1
```

Star Mature

uaaaccggauguuuaaaaaggcaaaagugacagguuguaacgagugcaacgcuguauuugcgcuggaacaaauugcacucgucccggccugcccacccuacauugcauaaac			
....aauugUacucgucccggccu......................	5	1	HA1
....aauugcacucgucUcggccu......................	30	1	HA1
....aauugcacucgucccgCccu......................	1	1	HA1
....aauugGacucgucccggccu......................	1	1	HA1
....aauugcacucgucccggUcu......................	3	1	HA1
....aauugcacucguccUggccu......................	8	1	HA1
....aGuugcacucgucccggccu......................	5	1	HA1
....aUuugcacucgucccggccu......................	2	1	HA1
....aauugcacucgucccggAcu......................	1	1	HA1
....aauugcacucgucccggccA......................	21	1	HA1
....Cauugcacucgucccggccu......................	7	1	HA1
....aauugcacucgGcccggccu......................	1	1	HA1
....Gauugcacucgucccggccu......................	7	1	HA1
....aauugcaUucgucccggccu......................	2	1	HA1
....aauugcacucCucccggccu......................	1	1	HA1
....aauugcCcucgucccggccu......................	3	1	HA1
....aauugcacuAgucccggccu......................	1	1	HA1
....aauugcacucgucccCgccu......................	1	1	HA1
....aauugcacucgucccggccG......................	3	1	HA1
....aauugcacucgucccgAccu......................	3	1	HA1
....aauugcacucgucccggccu......................	2943	0	HA1
....aauugcacucguUccggccu......................	21	1	HA1
....aauugcacuUgucccggccu......................	3	1	HA1
....aauugcacucgCcccggccu......................	2	1	HA1
....aCuugcacucgucccggccu......................	2	1	HA1
....aauugcacucgucGcggccu......................	1	1	HA1
....aauugcaUucgucccggccug.....................	2	1	HA1
....aauugcacucguccUggccug.....................	3	1	HA1
....aauugcacucgucccggccGg.....................	159	1	HA1
....aauugcacucguUccggccug.....................	29	1	HA1
....aauugcUcucgucccggccug.....................	2	1	HA1
....aauugcacuGgucccggccug.....................	1	1	HA1
....aauugcacucguccGggccug.....................	1	1	HA1
....aauugcacucgucccggccAg.....................	1	1	HA1
....aauuAcacucgucccggccug.....................	1	1	HA1
....aauugcacucgAcccggccug.....................	1	1	HA1
....aauugUacucgucccggccug.....................	2	1	HA1
....aauugcacucgucccUgccug.....................	1	1	HA1
....aauugcacucgucccggccug.....................	3574	0	HA1
....aauAgcacucgucccggccug.....................	1	1	HA1
....aauugcacucgucUcggccug.....................	23	1	HA1
....aauugcacucgGcccggccug.....................	1	1	HA1
....aauugcacucguAccggccug.....................	1	1	HA1
....aUuugcacucgucccggccug.....................	2	1	HA1
....aauugcacucgucccggUcug.....................	3	1	HA1
....aauugcacucguccAggccug.....................	1	1	HA1
....aaGugcacucgucccggccug.....................	1	1	HA1
....aauugcGcucgucccggccug.....................	3	1	HA1
....aauugcacGcgucccggccug.....................	1	1	HA1
....aGuugcacucgucccggccug.....................	1	1	HA1
....aauuCcacucgucccggccug.....................	1	1	HA1
....aauugcacuAgucccggccug.....................	3	1	HA1
....aauugcacucgucccggccCg.....................	4	1	HA1
....aCuugcacucgucccggccug.....................	1	1	HA1
....aauugcacucgucccggccuC.....................	2	1	HA1
....aauCgcacucgucccggccug.....................	1	1	HA1
....Cauugcacucgucccggccug.....................	7	1	HA1
....aauugcacucgucccggccuA.....................	28	1	HA1
....aauugcacuUgucccggccug.....................	4	1	HA1
....aauugcacucgucccggccuU.....................	131	1	HA1
....Gauugcacucgucccggccug.....................	5	1	HA1
....aauugcacucgucccgAccug.....................	2	1	HA1
....aauugcacucgucccggccuAc....................	7	1	HA1
....aauugcacucgucccggGcugc....................	1	1	HA1
....aGuugcacucgucccggccugc....................	7	1	HA1
....aauugcacucgucccggccuUc....................	4	1	HA1
....aauugcacucgucccgAccugc....................	5	1	HA1
....aauugcacucguGccggccugc....................	2	1	HA1
....aauugcacucgucccggUcugc....................	4	1	HA1
....aauugcacuAgucccggccugc....................	20	1	HA1

Star **Mature**

uaaaccggauguuuaaaaaggcaaaagugacagguuguaacgagugcaacgcuguauuugcgcuggaacaaauugcacucgucccggccugcccacccuacauugcauaaac			
....Cauugcacucgucccggccugc....................	12	1	HA1
....aauugcacucgucccggccugA....................	73	1	HA1
....aauugcacucgucccggccugc....................	8781	0	HA1
....aauugcacucguccUggccugc....................	3	1	HA1
....aauugcacucgucccggcAugc....................	1	1	HA1
....aauAgcacucgucccggccugc....................	1	1	HA1
....aauugcacucgucccgUccugc....................	2	1	HA1
....aauugcUcucgucccggccugc....................	2	1	HA1
....aauugcacucgucUcggccugc....................	22	1	HA1
....aauugcacucgucccggccCgc....................	7	1	HA1
....aauugcCcucgucccggccugc....................	6	1	HA1
....aauugcGcucgucccggccugc....................	6	1	HA1
....aCuugcacucgucccggccugc....................	5	1	HA1
....aUuugcacucgucccggccugc....................	2	1	HA1
....aauuCcacucgucccggccugc....................	2	1	HA1
....aauugcacucgGcccggccugc....................	2	1	HA1
....aauugcacucgucccggccGgc....................	2	1	HA1
....aauugcacucgucccggccAgc....................	3	1	HA1
....aauugcacucgucccgCccugc....................	2	1	HA1
....aaCugcacucgucccggccugc....................	1	1	HA1
....aauuAcacucgucccggccugc....................	2	1	HA1
....aauugcacucgucccggcUugc....................	1	1	HA1
....aauugcacuUgucccggccugc....................	6	1	HA1
....aauugcacGcgucccggccugc....................	2	1	HA1
....aauugcacucgucccUgccugc....................	1	1	HA1
....aauugcacucAucccggccugc....................	1	1	HA1
....aauugcacucgAcccggccugc....................	1	1	HA1
....aauugUacucgucccggccugc....................	13	1	HA1
....aauugcacucguUccggccugc....................	40	1	HA1
....aauugcacucgucccggccugU....................	2013	1	HA1
....aauugcacucgCcccggccugc....................	5	1	HA1
....aauugcaUucgucccggccugc....................	1	1	HA1
....Gauugcacucgucccggccugc....................	8	1	HA1
....aauugcacucgucccggccugG....................	10	1	HA1
....aauugcacucgucccggccugUc...................	4	1	HA1
....aauugcacucgucccggccugcA...................	873	1	HA1
....aauugcacucgucccggccugcc...................	25	0	HA1
....Uauugcacucgucccggccugcc...................	26	1	HA1
....aauugcacucgucccggccugcG...................	13	1	HA1
....aauugcacuUgucccggccugcc...................	1	1	HA1
....aauugcacucgucccggccugcUc..................	1	1	HA1
....aauugcacucgucccggccugccA..................	1	1	HA1
....aauugcacucgucccggccugccU..................	2	1	HA1
....aauugcacucgucccggccugccUa.................	1	1	HA1
....aAugcacucgucccggccu......................	1	1	HA1
....auugcaUucgucccggccu......................	1	1	HA1
....auugcacucgucccggccu......................	31	0	HA1
....Uuugcacucgucccggccu......................	1	1	HA1
....auugcacucguUccggccu......................	1	1	HA1
....auugcacucgucccggGcug.....................	1	1	HA1
....auugcacucgucccggccuA.....................	1	1	HA1
....auugcacucguUccggccug.....................	3	1	HA1
....auugcacucgucccggccuU.....................	5	1	HA1
....auugcacucgucccggccug.....................	143	0	HA1
....auugcacucgucccggccGg.....................	6	1	HA1
....Uuugcacucgucccggccugc....................	1	1	HA1
....auugcacucgucccggccugc....................	496	0	HA1
....auugcacuAgucccggccugc....................	2	1	HA1
....auugcacucgucccggccuAc....................	1	1	HA1
....auugcaUucgucccggccugc....................	1	1	HA1
....auugcacucgucUcggccugc....................	3	1	HA1
....auugUacucgucccggccugc....................	1	1	HA1
....auugcacucguUccggccugc....................	2	1	HA1
....auugcacucgucccggccCgc....................	1	1	HA1
....auugcacucgucccggccugU....................	22	1	HA1
....auugcacucgucccggccugUc...................	1	1	HA1
....auugcacucgucccggccugcA...................	86	1	HA1
....auugcacucgucccggccugcc...................	42	0	HA1
....auugcacucgucccggccugcG...................	1	1	HA1
....auugcacucgucccggccugccU..................	5	1	HA1

Star Mature

uaaaccggauguuuaaaaaggcaaaagugacagguuguaacgagugcaacgcuguauuugcgcuggaacaaauugcacucgucccggccugcccacccuacauugcauaaac			
....auugcacucgucccggccugccA..................	1	1	HA1
....auugcacucgucccggccugccUa.................	1	1	HA1
....uugcacucgucccggccu......................	12	0	HA1
....uugcacucgucccggccug.....................	22	0	HA1
....uugcacucguUccggccugc....................	1	1	HA1
....uugcacucgucccggccugc....................	242	0	HA1
....uugcacucUucccggccugc....................	1	1	HA1
....uugcacucCucccggccugc....................	1	1	HA1
....uugcacucguccUggccugc....................	1	1	HA1
....uugcUcucgucccggccugc....................	1	1	HA1
....uugcacucgucccggccugU....................	4	1	HA1
....uugcacucgucccggccugcc...................	16	0	HA1
....uugcacucgucccggccugcA...................	25	1	HA1
....uugcacucgucccggccugcG...................	1	1	HA1
....uugcacucgucccggccugccU..................	2	1	HA1
....uugcacucgucccggccugccA..................	2	1	HA1
....ugcacucgucccggccug.....................	15	0	HA1
....ugcacucgucccggccGg.....................	1	1	HA1
....ugUacucgucccggccug.....................	1	1	HA1
....ugcacucgucccggccugc....................	24	0	HA1
....ugUacucgucccggccugc....................	1	1	HA1
....ugcacucgucccggccugU....................	2	1	HA1
....ugcacucgucccggccugcA...................	3	1	HA1
....gcacucgucccggccugc....................	5	0	HA1
....gcacucgucccggccugcA...................	5	1	HA1
....auuugcgcuggaacaaauugcacuc................................	2	0	HS1
....aaauugcacucgucccggccug.....................	3	0	HS1
....aaauugcacucgucccggccugc....................	163	0	HS1
....Caauugcacucgucccggccugc....................	2	1	HS1
....aaauugcacucgucccggccugcA...................	9	1	HS1
....aauugcacucgucccggcA.......................	5	1	HS1
....aauugcacucgucccggcc.......................	1	0	HS1
....aauugcacucgucccggccA......................	1	1	HS1
....aauugcacucgucccggccu......................	22	0	HS1
....aauugcacucgucccggccuU.....................	2	1	HS1
....aauugcacucgucccggccuA.....................	2	1	HS1
....aauugcacucgucccggccug.....................	178	0	HS1
....aauugcacucgucccgAccugc....................	10	1	HS1
....aauugcacucgucccggAcugc....................	1	1	HS1
....aauugcacucgucccggccAgc....................	2	1	HS1
....aauugcacucgucccggccugU....................	35	1	HS1
....aauugcacucgucccggcUugc....................	2	1	HS1
....aauugcacucgucccggccuAc....................	21	1	HS1
....aauuAcacucgucccggccugc....................	8	1	HS1
....aauugcacucgucAcggccugc....................	1	1	HS1
....Gauugcacucgucccggccugc....................	5	1	HS1
....Cauugcacucgucccggccugc....................	12	1	HS1
....aauGgcacucgucccggccugc....................	1	1	HS1
....aauCgcacucgucccggccugc....................	3	1	HS1
....aauAgcacucgucccggccugc....................	1	1	HS1
....aauugcacuUgucccggccugc....................	1	1	HS1
....aauugcacucguccGggccugc....................	1	1	HS1
....aauugcacucgucccggccCgc....................	3	1	HS1
....aauugcacucguccUggccugc....................	3	1	HS1
....aauugcacucgucUcggccugc....................	1	1	HS1
....aauugcGcucgucccggccugc....................	6	1	HS1
....aauugcacucgucccggccugc....................	6317	0	HS1
....aauugcacucgucccggccugA....................	3	1	HS1
....aauugcacucgucccggUcugc....................	2	1	HS1
....aauugcacucAucccggccugc....................	1	1	HS1
....aGuugcacucgucccggccugc....................	2	1	HS1
....aaCugcacucgucccggccugc....................	3	1	HS1
....aauugcacucgucccggcGugc....................	1	1	HS1
....aauugcacucgucccAgccugc....................	3	1	HS1
....aauugcacucguUccggccugc....................	2	1	HS1
....aaGugcacucgucccggccugc....................	1	1	HS1
....aauugUacucgucccggccugc....................	4	1	HS1
....aauugcacCcgucccggccugc....................	1	1	HS1
....aauugcacGcgucccggccugc....................	1	1	HS1

Star Mature

```
uaaaccggauguuuaaaaaggcaaaagugacagguuguaacgagugcaacgcuguauuugcgcuggaacaaauugcacucgucccggccugcccacccuacauugcauaaac
......................................................................aauugcacucgucccggcGgc.....................    2     1   HS1
......................................................................Uauugcacucgucccggccugcc...................    29    1   HS1
......................................................................aauugcacucgucccggccugcA...................    929   1   HS1
......................................................................aauugcacucgucccggccugcG...................    2     1   HS1
......................................................................aauugcacucgucccggccugcc...................    11    0   HS1
.......................................................................auugcacucgucccggccu......................    5     0   HS1
.......................................................................auugcacucgucccggccC......................    1     1   HS1
.......................................................................auuUcacucgucccggccug.....................    1     1   HS1
.......................................................................auugcacuUgucccggccug.....................    1     1   HS1
.......................................................................auugcacucgucccggccug.....................    24    0   HS1
.......................................................................auugcacucgucccggccugc....................    1643  0   HS1
.......................................................................auugcacucgucccggccugU....................    9     1   HS1
.......................................................................auugcacucgucUcggccugc....................    1     1   HS1
.......................................................................auugcacucguAccggccugc....................    1     1   HS1
.......................................................................auugcacucgucccggccCgc....................    3     1   HS1
.......................................................................auugcacAcgucccggccugc....................    1     1   HS1
.......................................................................auugcacucAucccggccugc....................    1     1   HS1
.......................................................................auugcacucgucccggccuAc....................    2     1   HS1
.......................................................................auuUcacucgucccggccugc....................    1     1   HS1
.......................................................................auugcacucgucccggUcugc....................    3     1   HS1
.......................................................................auugcacucgucccggccAgc....................    1     1   HS1
.......................................................................auuAcacucgucccggccugc....................    1     1   HS1
.......................................................................auugUacucgucccggccugc....................    3     1   HS1
.......................................................................auugcacucgucccAgccugc....................    1     1   HS1
.......................................................................auugcaAucgucccggccugc....................    2     1   HS1
.......................................................................auugcacucgucccCgccugc....................    2     1   HS1
.......................................................................auugcacucgucccggccugcA...................    267   1   HS1
.......................................................................auugcacucgucccggccugcG...................    1     1   HS1
.......................................................................auugcacucgucccggccugcc...................    17    0   HS1
........................................................................uugcacucgucccggccugA....................    1     1   HS1
........................................................................uugcacucgCcccggccugc....................    1     1   HS1
........................................................................uugcacucgucccggccugc....................    25    0   HS1
........................................................................uugcacucgucccggccugcA...................    2     1   HS1
........................................................................uugcacucgucccggccugcc...................    2     0   HS1
..........................................................................gcacucgucccggccugc....................    2     0   HS1

...............................agguuguaacgagugcaacg.............................................................    4     0   HS7
...............................agguuguaacgagugcaacgc............................................................    1     0   HS7
...............................agguuguaacgagugcaacgcu...........................................................    2     0   HS7
..................................uuguaacgagugcaacgcu...........................................................    1     0   HS7
.....................................................................aaauugcacucgucccgg.........................    1     0   HS7
.....................................................................aaauugcacucgucccggcc.......................    3     0   HS7
.....................................................................aaauugcacucgucccgAccu......................    2     1   HS7
.....................................................................Uaauugcacucgucccggccu......................    1     1   HS7
.....................................................................aaauugcacucgucUcggccu......................    1     1   HS7
.....................................................................aaauugcacucgucccggccu......................    84    0   HS7
.....................................................................aaauugcacucguUccggccug.....................    1     1   HS7
.....................................................................Uaauugcacucgucccggccug.....................    3     1   HS7
.....................................................................aaauugcacucgucccggccug.....................    138   0   HS7
.....................................................................aaauugcacucgucccggccuU.....................    3     1   HS7
.....................................................................aaauugcacucgucccggccCg.....................    1     1   HS7
.....................................................................aaauugcacucgucccggccGg.....................    9     1   HS7
.....................................................................aaUuugcacucgucccggccug.....................    1     1   HS7
.....................................................................aaauugcUcucgucccggccugc....................    2     1   HS7
.....................................................................aaauugcacucgucccggccugU....................    35    1   HS7
.....................................................................Uaauugcacucgucccggccugc....................    20    1   HS7
.....................................................................aaauugcacucgucccggccugA....................    1     1   HS7
.....................................................................aaauugcacucgucccggccugc....................    207   0   HS7
.....................................................................aaauugcacucgucccUgccugc....................    1     1   HS7
.....................................................................aaauugcacucgucccCgccugc....................    1     1   HS7
.....................................................................aaauugcacucgucUcggccugc....................    2     1   HS7
.....................................................................aaauugcacucguUccggccugc....................    1     1   HS7
.....................................................................aaauugcacucgucccggccugcc...................    1     0   HS7
......................................................................aauugcacucgucccggc........................    3     0   HS7
......................................................................aauugcacucgucccggcU.......................    2     1   HS7
......................................................................aauugcacucgucccggcc.......................    48    0   HS7
......................................................................aauuCcacucgucccggccu......................    1     1   HS7
......................................................................aauugcacucgGcccggccu......................    1     1   HS7
......................................................................aauugcacucgucccggccu......................    1171  0   HS7
......................................................................aauugcacucgucccUgccu......................    1     1   HS7
```

Star **Mature**

```
uaaaccggauguuuaaaaaggcaaaagugacagguuguaacgagugcaacgcuguauuugcgcuggaacaaauugcacucgucccggccugcccacccuacauugcauaaac
......................................................................aauugcacucgucccgAccu......................   1       1    HS7
......................................................................aauugcacucgucUcggccu......................   5       1    HS7
......................................................................aauugcacucgucccggccA......................   4       1    HS7
......................................................................aauugcacucgucccggUcu......................   1       1    HS7
......................................................................aauugcacucguccGggccu......................   2       1    HS7
......................................................................aauugcacucguccUggccu......................   2       1    HS7
......................................................................aauugcacucguUccggccu......................   3       1    HS7
......................................................................aauugcacucgucccggccG......................   5       1    HS7
......................................................................aauuUcacucgucccggccu......................   1       1    HS7
......................................................................aauugcaUucgucccggccu......................   1       1    HS7
......................................................................aaCugcacucgucccggccu......................   1       1    HS7
......................................................................aauugcacucgucccggcUu......................   1       1    HS7
......................................................................aauugcacucgucccgAccug.....................   2       1    HS7
......................................................................aauuAcacucgucccggccug.....................   2       1    HS7
......................................................................aauugcacucgucUcggccug.....................   26      1    HS7
......................................................................aauugcaUucgucccggccug.....................   2       1    HS7
......................................................................aauugUacucgucccggccug.....................   2       1    HS7
......................................................................aauugcacucCucccggccug.....................   1       1    HS7
......................................................................aauugcacucgucccggccuU.....................   23      1    HS7
......................................................................aauugcacucgucccggAcug.....................   1       1    HS7
......................................................................aauugcacucgucccggccug.....................   2817    0    HS7
......................................................................aauugcacucguUccggccug.....................   18      1    HS7
......................................................................aauugcacucgucccggccAg.....................   4       1    HS7
......................................................................aauugcacucguccUggccug.....................   8       1    HS7
......................................................................Cauugcacucgucccggccug.....................   1       1    HS7
......................................................................aauugcacucgucccggccCg.....................   4       1    HS7
......................................................................aauugcacucguAccggccug.....................   1       1    HS7
......................................................................aauugcacucgucccggcGug.....................   1       1    HS7
......................................................................aaCugcacucgucccggccug.....................   1       1    HS7
......................................................................aauugcacucgucccggccuA.....................   13      1    HS7
......................................................................aauugcaGucgucccggccug.....................   1       1    HS7
......................................................................aauugcacucgucccCgccug.....................   2       1    HS7
......................................................................aauugcacucgucccgUccug.....................   3       1    HS7
......................................................................aUuugcacucgucccggccug.....................   1       1    HS7
......................................................................aauugcacucgucccgCccug.....................   2       1    HS7
......................................................................aauugcacucgucccggUcug.....................   3       1    HS7
......................................................................aauugcacucgucccggccGg.....................   223     1    HS7
......................................................................aauugcacucUucccggccug.....................   1       1    HS7
......................................................................aauugcacucAucccggccugc....................   3       1    HS7
......................................................................aauugcacucgucccggccugc....................   11767   0    HS7
......................................................................aauugcaAucgucccggccugc....................   1       1    HS7
......................................................................aauugcacucgucccggccuCc....................   1       1    HS7
......................................................................aauugcacucguGccggccugc....................   3       1    HS7
......................................................................aauugcacucgucccggcAugc....................   1       1    HS7
......................................................................aauugcaGucgucccggccugc....................   4       1    HS7
......................................................................aauCgcacucgucccggccugc....................   1       1    HS7
......................................................................aauugcUcucgucccggccugc....................   1       1    HS7
......................................................................aauugcacucgucccggcUugc....................   3       1    HS7
......................................................................aauugcacucgucccggccugG....................   16      1    HS7
......................................................................aauugcacucgucccggccugU....................   531     1    HS7
......................................................................aauugcacucguUccggccugc....................   39      1    HS7
......................................................................aauugcacucgGcccggccugc....................   1       1    HS7
......................................................................aauugcacucgAcccggccugc....................   1       1    HS7
......................................................................aauugcacucgucccggccAgc....................   5       1    HS7
......................................................................aauugcGcucgucccggccugc....................   1       1    HS7
......................................................................aauugcacucgucccgAccugc....................   4       1    HS7
......................................................................aauugcacucgucccUgccugc....................   2       1    HS7
......................................................................Cauugcacucgucccggccugc....................   2       1    HS7
......................................................................aauugcacucgucccggccugA....................   13      1    HS7
......................................................................aauugcacucguccUggccugc....................   16      1    HS7
......................................................................aauugcacucgucccggccCgc....................   3       1    HS7
......................................................................aaCugcacucgucccggccugc....................   5       1    HS7
......................................................................aauugcacucgucccggUcugc....................   11      1    HS7
......................................................................aaGugcacucgucccggccugc....................   3       1    HS7
......................................................................aauugcacuGgucccggccugc....................   2       1    HS7
......................................................................Gauugcacucgucccggccugc....................   1       1    HS7
......................................................................aCuugcacucgucccggccugc....................   1       1    HS7
......................................................................aauugcacucgucUcggccugc....................   39      1    HS7
......................................................................aauuAcacucgucccggccugc....................   7       1    HS7
......................................................................aauugcacucgucccggccuAc....................   7       1    HS7
```

```
                              Star                                    Mature
uaaaccggauguuuaaaaaggcaaaagugacagguuguaacgagugcaacgcuguauuugcgcuggaacaaauugcacucgucccggccugcccacccuacauugcauaaac
......................................................................aauugcacucgucccggccuUc....................    10    1    HS7
......................................................................aauugcacucgucccgCccugc....................    2     1    HS7
......................................................................aauugUacucgucccggccugc....................    12    1    HS7
......................................................................aauugcaUucgucccggccugc....................    3     1    HS7
......................................................................aauugcacucUucccggccugc....................    7     1    HS7
......................................................................aGuugcacucgucccggccugc....................    1     1    HS7
......................................................................aauuCcacucgucccggccugc....................    3     1    HS7
......................................................................aauugcacucgucAcggccugc....................    1     1    HS7
......................................................................aauugcacucgucccAgccugc....................    4     1    HS7
......................................................................aUuugcacucgucccggccugc....................    2     1    HS7
......................................................................aauugcacucgCcccggccugc....................    2     1    HS7
......................................................................aauugcacucgucccCgccugc....................    2     1    HS7
......................................................................aauugcacuUgucccggccugc....................    3     1    HS7
......................................................................aauugcacucgucccggccGgc....................    4     1    HS7
......................................................................aauuUcacucgucccggccugc....................    7     1    HS7
......................................................................aauugcacAcgucccggccugc....................    1     1    HS7
......................................................................aauugcacucCucccggccugc....................    1     1    HS7
......................................................................aauugcacucgucccgUccugc....................    3     1    HS7
......................................................................aauugcacucgucccggccugcA...................    405   1    HS7
......................................................................aauugcacucgucccggccugUc...................    4     1    HS7
......................................................................Uauugcacucgucccggccugcc...................    20    1    HS7
......................................................................aauugcacucgucccggccugcc...................    18    0    HS7
......................................................................aauugcacucgucccggccugcG...................    14    1    HS7
......................................................................aauugcacucgucccggccugccU..................    2     1    HS7
......................................................................aauugcacucgucccggccugcUc..................    1     1    HS7
.......................................................................auugcacucgucccggccu......................    18    0    HS7
.......................................................................auugcacucgucccggccGg.....................    4     1    HS7
.......................................................................auugcacucgucUcggccug.....................    1     1    HS7
.......................................................................auugcacucgucccggccug.....................    43    0    HS7
.......................................................................auugcacucgucUcggccugc....................    2     1    HS7
.......................................................................auugcacucUucccggccugc....................    1     1    HS7
.......................................................................auugcacucgGcccggccugc....................    1     1    HS7
.......................................................................auugcacucgucccggccAgc....................    1     1    HS7
.......................................................................Uuugcacucgucccggccugc....................    1     1    HS7
.......................................................................auugcacucgucccggccuAc....................    1     1    HS7
.......................................................................auugcacucgucccgAccugc....................    1     1    HS7
.......................................................................auugcacucgucccggccugc....................    303   0    HS7
.......................................................................auugcacucgucccCgccugc....................    1     1    HS7
.......................................................................auugcacucgucccggccugU....................    10    1    HS7
.......................................................................auCgcacucgucccggccugc....................    1     1    HS7
.......................................................................auugcacucgucccggccugA....................    1     1    HS7
.......................................................................auugcacCcgucccggccugc....................    1     1    HS7
.......................................................................auugcacucgucccggccugcG...................    2     1    HS7
.......................................................................auugcacucgucccggccugcA...................    19    1    HS7
.......................................................................auugcacucgucccggccuUcc...................    1     1    HS7
.......................................................................auugcacucgucccggccugcc...................    67    0    HS7
.......................................................................auugcacucgucccggccugccU..................    7     1    HS7
.......................................................................auugcacucgucccggccugccA..................    3     1    HS7
.......................................................................auugcacucgucccggccugcUc..................    1     1    HS7
........................................................................uugcacucgucccggccu......................    1     0    HS7
........................................................................uugcacucgucccggccug.....................    3     0    HS7
........................................................................uugcacucgucccggccugc....................    22    0    HS7
........................................................................uugcacucgucccggccugcc...................    1     0    HS7
........................................................................uugcacucgucccggccugcG...................    1     1    HS7
........................................................................uugcacucgucccggccugccU..................    1     1    HS7
.........................................................................ugcacucgucccggccugc....................    14    0    HS7
..........................................................................gcacucgucccggccugc....................    28    0    HS7
..........................................................................gcacucgucccggccugU....................    8     1    HS7
..........................................................................gcacucguccUggccugc....................    1     1    HS7
..........................................................................gcacucgucccggccugcA...................    1     1    HS7

...............................agguuguaacgagugcaaA..............................................................    1     1    HS5
...............................agguuguaacgagugcaacg.............................................................    6     0    HS5
...............................agguuguaacgagugcaacgcu...........................................................    1     0    HS5
...............................agguuguaacgagugcaacgcuU..........................................................    1     1    HS5
..................................................................aacaaauugcacucgucccggccugc....................    1     0    HS5
.....................................................................aaauugcacucgucccggcc.......................    4     0    HS5
.....................................................................aaauugcacucguUccggccu......................    1     1    HS5
.....................................................................aaauugcacucgucUcggccu......................    1     1    HS5
.....................................................................aaauugUacucgucccggccu......................    1     1    HS5
```

Star Mature

```
uaaaccggauguuuaaaaaggcaaaagugacagguuguaacgagugcaacgcuguauuugcgcuggaacaaauugcacucgucccggccugcccacccuacauugcauaaac
.....................................................................aCauugcacucgucccggccu......................    1     1    HS5
.....................................................................aaauugcacucgucccggccu......................    80    0    HS5
.....................................................................aaauugcacucgCcccggccu......................    2     1    HS5
.....................................................................Uaauugcacucgucccggccu......................    1     1    HS5
.....................................................................Uaauugcacucgucccggccug.....................    1     1    HS5
.....................................................................aaauugcacucgucccggccCg.....................    1     1    HS5
.....................................................................aaauugcacucgucccggccuU.....................    3     1    HS5
.....................................................................aaauugcaUucgucccggccug.....................    1     1    HS5
.....................................................................aaauugcacucgucccggccug.....................    158   0    HS5
.....................................................................aaauuAcacucgucccggccug.....................    1     1    HS5
.....................................................................aaauugcacucgucUcggccug.....................    1     1    HS5
.....................................................................aaauugcacucguUccggccug.....................    8     1    HS5
.....................................................................aaauugcacucguccUggccug.....................    1     1    HS5
.....................................................................aaauugcacucgucccggccugA....................    1     1    HS5
.....................................................................aaauugcacucgucccggccGgc....................    2     1    HS5
.....................................................................aaauuAcacucgucccggccugc....................    1     1    HS5
.....................................................................aaauugcacucgucUcggccugc....................    2     1    HS5
.....................................................................aaauugcacucguUccggccugc....................    2     1    HS5
.....................................................................Uaauugcacucgucccggccugc....................    6     1    HS5
.....................................................................aaauugUacucgucccggccugc....................    1     1    HS5
.....................................................................aaauugcacucgucccggccugU....................    19    1    HS5
.....................................................................aaauugcacucgucccggccugc....................    326   0    HS5
.....................................................................aaauugcGcucgucccggccugc....................    1     1    HS5
.....................................................................aaauugcacucgucccggccugcA...................    6     1    HS5
......................................................................aauugcacucgucccCgc........................    1     1    HS5
......................................................................aauugcacucgucccggc........................    3     0    HS5
......................................................................aauugcacucgucccggcc.......................    17    0    HS5
......................................................................aauugcacucgucccUgccu......................    3     1    HS5
......................................................................aauugcacucgucccggccG......................    1     1    HS5
......................................................................aauugcacucguGccggccu......................    1     1    HS5
......................................................................aauugcacucgucccgUccu......................    1     1    HS5
......................................................................aauugcacucgucccggccA......................    3     1    HS5
......................................................................aauugcacucgucUcggccu......................    11    1    HS5
......................................................................aauugcacuUgucccggccu......................    1     1    HS5
......................................................................aauugcacucguccUggccu......................    2     1    HS5
......................................................................aauugUacucgucccggccu......................    3     1    HS5
......................................................................aauugcacucgucccggccu......................    1442  0    HS5
......................................................................aauugcacucguUccggccu......................    21    1    HS5
......................................................................aauugcacucUucccggccu......................    1     1    HS5
......................................................................aauugcacucgucccAgccu......................    2     1    HS5
......................................................................aauugcacucgucccggcUu......................    1     1    HS5
......................................................................aauugcUcucgucccggccu......................    2     1    HS5
......................................................................aauugcacucgucccgAccu......................    3     1    HS5
......................................................................aauugcacucgucccggUcu......................    2     1    HS5
......................................................................aauCgcacucgucccggccu......................    1     1    HS5
......................................................................aauugcacucgucccggccC......................    1     1    HS5
......................................................................Gauugcacucgucccggccu......................    3     1    HS5
......................................................................aauugcacucgCcccggccu......................    3     1    HS5
......................................................................aauugcacucgGcccggccu......................    1     1    HS5
......................................................................aauugcacucguccAggccug.....................    1     1    HS5
......................................................................aauugcacucgucccggcUug.....................    1     1    HS5
......................................................................aauugcacucgucccggccuU.....................    35    1    HS5
......................................................................aaCugcacucgucccggccug.....................    1     1    HS5
......................................................................aauugcacucgucccggccug.....................    2990  0    HS5
......................................................................aGuugcacucgucccggccug.....................    3     1    HS5
......................................................................aauugcacucgucccCgccug.....................    1     1    HS5
......................................................................aauugcacuUgucccggccug.....................    1     1    HS5
......................................................................aauugcacucgucccggccCg.....................    5     1    HS5
......................................................................aauugUacucgucccggccug.....................    7     1    HS5
......................................................................aauugcacucguccUggccug.....................    6     1    HS5
......................................................................aauugcacucgucccggUcug.....................    6     1    HS5
......................................................................aCuugcacucgucccggccug.....................    1     1    HS5
......................................................................aauugcacucgucccgAccug.....................    5     1    HS5
......................................................................aaAugcacucgucccggccug.....................    1     1    HS5
......................................................................aauugcacucAucccggccug.....................    1     1    HS5
......................................................................aauugcacucUucccggccug.....................    1     1    HS5
......................................................................aauugcacCcgucccggccug.....................    1     1    HS5
......................................................................aauugcacucgucccAgccug.....................    2     1    HS5
......................................................................aauugcacucgCcccggccug.....................    4     1    HS5
......................................................................aauugcacucguUccggccug.....................    48    1    HS5
```

Star **Mature**

uaaaccggauguuuaaaaaggcaaaagugacagguuguaacgagugcaacgcuguauuugcgcuggaacaaauugcacucgucccggccugcccacccuacauugcauaaac			
....aauugcacucgucccggccuA.....................	6	1	HS5
....aauugcacucguGccggccug.....................	1	1	HS5
....aauuAcacucgucccggccug.....................	2	1	HS5
....aauugcacucgucUcggccug.....................	32	1	HS5
....aauugcacucgucccgAccugc....................	13	1	HS5
....aauugcUcucgucccggccugc....................	13	1	HS5
....aauugcacucgAcccggccugc....................	6	1	HS5
....aauugcacucgucccgCccugc....................	3	1	HS5
....aauugcacucgucccUgccugc....................	5	1	HS5
....aauugcacuUgucccggccugc....................	7	1	HS5
....aauugcacucgucUcggccugc....................	81	1	HS5
....aauugcacucgucccggccugc....................	18504	0	HS5
....aauugcacuAgucccggccugc....................	2	1	HS5
....aauugcacucgucccggccugG....................	2	1	HS5
....aauCgcacucgucccggccugc....................	3	1	HS5
....aauugcGcucgucccggccugc....................	4	1	HS5
....aauugAacucgucccggccugc....................	1	1	HS5
....aauugcaUucgucccggccugc....................	9	1	HS5
....aauugcacucgucccggccuCc....................	3	1	HS5
....aGuugcacucgucccggccugc....................	4	1	HS5
....aUuugcacucgucccggccugc....................	2	1	HS5
....Gauugcacucgucccggccugc....................	10	1	HS5
....aauugUacucgucccggccugc....................	25	1	HS5
....aauugcacucgCcccggccugc....................	17	1	HS5
....aauugcacucguUccggccugc....................	134	1	HS5
....aauugcacucgucccggccuAc....................	12	1	HS5
....aauugcacucgucccggccGgc....................	11	1	HS5
....aauugcacucgGcccggccugc....................	6	1	HS5
....aauugcacucguGccggccugc....................	5	1	HS5
....aauugcacucgucccCgccugc....................	5	1	HS5
....aauugcacucgucccggUcugc....................	18	1	HS5
....Cauugcacucgucccggccugc....................	12	1	HS5
....aauugcacucgucccggccugA....................	18	1	HS5
....aauugcacucUucccggccugc....................	2	1	HS5
....aauugcacucgucAcggccugc....................	1	1	HS5
....aaGugcacucgucccggccugc....................	1	1	HS5
....aauugcacucgucccggccCgc....................	8	1	HS5
....aauugcaAucgucccggccugc....................	1	1	HS5
....aauugcacucAucccggccugc....................	1	1	HS5
....aauugcacucgucccggccugU....................	542	1	HS5
....aauugcacucgucccgUccugc....................	3	1	HS5
....aauugcacucguccUggccugc....................	22	1	HS5
....aauugcCcucgucccggccugc....................	3	1	HS5
....aauugcacuGgucccggccugc....................	1	1	HS5
....aauuUcacucgucccggccugc....................	6	1	HS5
....aauugcacucgucccggAcugc....................	2	1	HS5
....aauugcacucgucccAgccugc....................	21	1	HS5
....aaAugcacucgucccggccugc....................	1	1	HS5
....aCuugcacucgucccggccugc....................	5	1	HS5
....aauugcacucCucccggccugc....................	2	1	HS5
....aauugcacucgucccggcAugc....................	1	1	HS5
....aauugcacucgucccggGcugc....................	1	1	HS5
....aauugcacucgucccggcUugc....................	21	1	HS5
....aauuCcacucgucccggccugc....................	4	1	HS5
....aauuAcacucgucccggccugc....................	9	1	HS5
....aauugcacucguccGggccugc....................	1	1	HS5
....Uauugcacucgucccggccugcc...................	9	1	HS5
....aauugcacucgucccggccugcc...................	15	0	HS5
....aauugcacucgucccggccugcA...................	476	1	HS5
....aauugcacucgucccggccugUc...................	1	1	HS5
....aauugcacucgucccgUccugcc...................	1	1	HS5
....aauugcacucgucccggccugcG...................	7	1	HS5
....aauugcacucgucccggccugcUc..................	2	1	HS5
....aauugcacucgucccggccugccU..................	6	1	HS5
....auugcacucgucccggccA......................	1	1	HS5
....auugcacucgucGcggccu......................	1	1	HS5
....auugcacucgucccggccu......................	13	0	HS5
....auugcacucgucccggccGg.....................	1	1	HS5
....auugcacucgucccggccug.....................	54	0	HS5
....auugcacucgucccggccuU.....................	1	1	HS5

Star **Mature**

```
uaaaccggauguuuaaaaaggcaaaagugacagguuguaacgagugcaacgcuguauuugcgcuggaacaaauugcacucgucccggccugcccacccuacauugcauaaac
.......................................................................auugcacucgucccggccCg.....................  1     1  HS5
.......................................................................auugcacucguUccggccugc....................  4     1  HS5
.......................................................................auugcacucguccUggccugc....................  1     1  HS5
.......................................................................auugcacucgucUcggccugc....................  4     1  HS5
.......................................................................auugcaUucgucccggccugc....................  1     1  HS5
.......................................................................auugcacuUgucccggccugc....................  2     1  HS5
.......................................................................auugcacucgucccggccugc....................  554   0  HS5
.......................................................................auugcacucgucccggccugU....................  8     1  HS5
.......................................................................aAugcacucgucccggccugc....................  2     1  HS5
.......................................................................auugcacucgucccgAccugc....................  2     1  HS5
.......................................................................auugcacucgucccggccuAc....................  3     1  HS5
.......................................................................auugcacucgucccggccugcc...................  53    0  HS5
.......................................................................auugcacucgucccggccugcA...................  44    1  HS5
.......................................................................auugcacucgucccggccugccU..................  6     1  HS5
........................................................................uugcacucgucccggccug.....................  2     0  HS5
........................................................................uugcacucgucccggccugc....................  20    0  HS5
........................................................................uugcacucgucccggccugcA...................  1     1  HS5
........................................................................uugcacucgucccggccugcUc..................  1     1  HS5
.........................................................................uUcacucgucccggccug.....................  1     1  HS5
.........................................................................ugcacucgucccggccug.....................  3     0  HS5
.........................................................................ugcacucgucccggccugc....................  13    0  HS5
.........................................................................Ggcacucgucccggccugc....................  1     1  HS5
..........................................................................gcacucgucccggccugc....................  6     0  HS5
..........................................................................gcacucgucccggccugU....................  1     1  HS5

...............................agguuguaacgagugcaa...............................................................  1     0  HFA
...............................agguuguaacgagugcaacg.............................................................  20    0  HFA
...................................uguaacgagugcaacgcu...........................................................  2     0  HFA
.....................................................................aaauugcacucgucccggcc.......................  1     0  HFA
.....................................................................Uaauugcacucgucccggccu......................  2     1  HFA
.....................................................................aaauugcacucgucccggccA......................  2     1  HFA
.....................................................................aaauugcacucgucccggccu......................  34    0  HFA
.....................................................................aaauugcacucgucccggccug.....................  23    0  HFA
.....................................................................aaauugcacucgucccggccCg.....................  1     1  HFA
.....................................................................aaauugcacucgucccggccuU.....................  2     1  HFA
.....................................................................Uaauugcacucgucccggccug.....................  2     1  HFA
.....................................................................Uaauugcacucgucccggccugc....................  4     1  HFA
.....................................................................aaauugcacucgucccggccugU....................  5     1  HFA
.....................................................................aaauugcacucgucccgCccugc....................  1     1  HFA
.....................................................................aaauugcacucgucccggccugc....................  21    0  HFA
.....................................................................aaauugcacucgucccggccugcA...................  2     1  HFA
......................................................................aauugcacucgucccggU........................  5     1  HFA
......................................................................aauugcacucgucccUgc........................  1     1  HFA
......................................................................aauugcacucgucccggc........................  9     0  HFA
......................................................................aauugcacucgucccggA........................  2     1  HFA
......................................................................aauugcacucgucccgCc........................  2     1  HFA
......................................................................aauugcacucgucccggcc.......................  39    0  HFA
......................................................................aauugcacucgucccggcA.......................  4     1  HFA
......................................................................aauugcacucguUccggccu......................  8     1  HFA
......................................................................aauugcacucgucccgAccu......................  2     1  HFA
......................................................................aauugcacucgucUcggccu......................  5     1  HFA
......................................................................aauugcacucCucccggccu......................  1     1  HFA
......................................................................aauugcacucgucccgCccu......................  1     1  HFA
......................................................................aauugcacucgucccggccC......................  5     1  HFA
......................................................................aauugcacucgucccggccG......................  2     1  HFA
......................................................................aauugcacucgucccggccu......................  1227  0  HFA
......................................................................aauugcacucgucccgUccu......................  1     1  HFA
......................................................................aauugcacucgucccggccA......................  4     1  HFA
......................................................................aauugcacuUgucccggccu......................  2     1  HFA
......................................................................aauugUacucgucccggccu......................  1     1  HFA
......................................................................aauugcacucguccUggccu......................  3     1  HFA
......................................................................aauugcacucguGccggccu......................  1     1  HFA
......................................................................aauugcacucgGcccggccu......................  1     1  HFA
......................................................................aauugcacucUucccggccu......................  1     1  HFA
......................................................................aauugcaUucgucccggccu......................  2     1  HFA
......................................................................aauugcacucgucccggUcu......................  2     1  HFA
......................................................................aauugcacucgucccggccuA.....................  19    1  HFA
......................................................................aauugcacucgucAcggccug.....................  1     1  HFA
......................................................................aauugcacucgucccggccug.....................  1036  0  HFA
......................................................................aauugcacucgucccggccCg.....................  1     1  HFA
```

Star Mature

```
uaaaccggauguuuaaaaaggcaaaagugacagguuguaacgagugcaacgcuguauuugcgcuggaacaaauugcacucgucccggccugcccacccuacauugcauaaac
......................................................................aauugcacucgucccCgccug.....................  1     1  HFA
......................................................................aauugcacucguccUggccug.....................  2     1  HFA
......................................................................aauugcacucguUccggccug.....................  10    1  HFA
......................................................................aauugcacucgucccggccGg.....................  1     1  HFA
......................................................................aauugcacucgucUcggccug.....................  5     1  HFA
......................................................................aauugcacucgucccggAcug.....................  2     1  HFA
......................................................................aauugcacucgucccggccuU.....................  65    1  HFA
......................................................................aauugcacucUucccggccugc....................  2     1  HFA
......................................................................aauugcacucgucccggcAugc....................  1     1  HFA
......................................................................aauugcaUucgucccggccugc....................  3     1  HFA
......................................................................aauugcacucgucccUgccugc....................  1     1  HFA
......................................................................aauugcacucgucccgCccugc....................  2     1  HFA
......................................................................aauugcacucgucccggccGgc....................  3     1  HFA
......................................................................aauugcacucgCcccggccugc....................  1     1  HFA
......................................................................aauugcacucgucccggccuUc....................  8     1  HFA
......................................................................aauugcacucAucccggccugc....................  1     1  HFA
......................................................................aauugcacucgucUcggccugc....................  3     1  HFA
......................................................................aauugcacuUgucccggccugc....................  2     1  HFA
......................................................................aauugcacucgucccggcUugc....................  2     1  HFA
......................................................................aauugcacucguccAggccugc....................  1     1  HFA
......................................................................aauugcacucgucccCgccugc....................  1     1  HFA
......................................................................aauugcacucgucccggccCgc....................  4     1  HFA
......................................................................aauugcacucguUccggccugc....................  3     1  HFA
......................................................................aauugcacucgucccgAccugc....................  1     1  HFA
......................................................................aauugcacucguccUggccugc....................  1     1  HFA
......................................................................aauugcacucgucccggccuAc....................  3     1  HFA
......................................................................aauugcacucgucccgUccugc....................  3     1  HFA
......................................................................aauugcacucgucccggccugU....................  389   1  HFA
......................................................................aauugcacucgucccggccugc....................  2831  0  HFA
......................................................................aauugcacucgucccggUcugc....................  4     1  HFA
......................................................................aauugcacucguccGggccugc....................  2     1  HFA
......................................................................aauugcacucgucccggccuCc....................  1     1  HFA
......................................................................aauugcacucguAccggccugc....................  1     1  HFA
......................................................................Gauugcacucgucccggccugc....................  1     1  HFA
......................................................................aauugcacucgucccggccugA....................  25    1  HFA
......................................................................aauugcacucgucccggccugcG...................  1     1  HFA
......................................................................Uauugcacucgucccggccugcc...................  5     1  HFA
......................................................................aauugcacucgucccggccugcA...................  248   1  HFA
......................................................................aauugcacucgucccggccugcc...................  5     0  HFA
.......................................................................auugcacucgucccggcc.......................  2     0  HFA
.......................................................................auugcacucgucccggcAu......................  1     1  HFA
.......................................................................auugcacucgucccggccu......................  15    0  HFA
.......................................................................auugcacucgucccggccug.....................  89    0  HFA
.......................................................................auuUcacucgucccggccugc....................  1     1  HFA
.......................................................................auugcacucgucccggccugc....................  362   0  HFA
.......................................................................auugcacucgucccgAccugc....................  1     1  HFA
.......................................................................Uuugcacucgucccggccugc....................  2     1  HFA
.......................................................................auugcacucgucccggccGgc....................  2     1  HFA
.......................................................................auugcacucgucUcggccugc....................  1     1  HFA
.......................................................................auugcacucgucccggccugU....................  8     1  HFA
.......................................................................auugcacucgucccggccuAc....................  1     1  HFA
.......................................................................aAugcacucgucccggccugc....................  2     1  HFA
.......................................................................auugcacucguccUggccugc....................  1     1  HFA
.......................................................................auuAcacucgucccggccugc....................  1     1  HFA
.......................................................................auugcacucgucccggccugcc...................  12    0  HFA
.......................................................................auugcacucgucccggccugcA...................  32    1  HFA
.......................................................................auugcacucgucccggccugccU..................  1     1  HFA
.......................................................................auugcacucgucccggccugccA..................  1     1  HFA
........................................................................uugcacucgucccggccu......................  6     0  HFA
........................................................................uugcacucgucccggccGg.....................  1     1  HFA
........................................................................Augcacucgucccggccug.....................  1     1  HFA
........................................................................uugcacucgucccggccug.....................  13    0  HFA
........................................................................uugcacucgucccggcUugc....................  1     1  HFA
........................................................................uugcacucgucccggccugU....................  3     1  HFA
........................................................................uugcacucgucccggccugc....................  107   0  HFA
........................................................................uugcacucgucccggccuAc....................  1     1  HFA
........................................................................uugcacuUgucccggccugc....................  1     1  HFA
........................................................................uugcacucgucccggccugcA...................  9     1  HFA
........................................................................uugcacucgucccggccugcc...................  8     0  HFA
........................................................................uugcacucgucccggccugccU..................  1     1  HFA
```

Star / Mature			
uaaaccggauguuuaaaaaggcaaaagugacagguuguaacgagugcaacgcuguauuugcgcuggaacaaauugcacucgucccggccugcccacccuacauugcauaaac			
....uUcacucgucccggccug.....................	1	1	HFA
....ugcacucgucccggccug.....................	1	0	HFA
....ugcacucgucccggccuU.....................	2	1	HFA
....ugcacucgucccggccugc....................	10	0	HFA
....ugcacucgucccggccugU....................	1	1	HFA
....ugcacucgucccggccugcA...................	1	1	HFA
....gcacucgucccggccugU....................	7	1	HFA
....gcacucgucccggccugc....................	10	0	HFA
....aaauugcacucgucccggccAgc....................	1	1	HMA
....aaauugcacucgucccggccugc....................	111	0	HMA
....aaauugcacucgucccggUcugc....................	2	1	HMA
....aaauugcacucgucccggccuAc....................	3	1	HMA
....aauugcacucgucccggccu......................	1	0	HMA
....aauugcacucgucccgAccug.....................	3	1	HMA
....aauugcacucgucccggccug.....................	368	0	HMA
....aauugcacucUucccggccugc....................	1	1	HMA
....aauugcacucgucccggccCgc....................	1	1	HMA
....aauugcacucgucccggccugU....................	4	1	HMA
....aauugcacucgucccggccugc....................	1845	0	HMA
....Cauugcacucgucccggccugc....................	5	1	HMA
....aauCgcacucgucccggccugc....................	1	1	HMA
....Gauugcacucgucccggccugc....................	2	1	HMA
....aauugcacucgucccggccuAc....................	4	1	HMA
....aauugcacucgGcccggccugc....................	1	1	HMA
....aauuAcacucgucccggccugc....................	3	1	HMA
....aauugcacucgucccggccAgc....................	1	1	HMA
....aauugcacucguccGggccugc....................	1	1	HMA
....aauugUacucgucccggccugc....................	2	1	HMA
....aauugcacucguUccggccugc....................	3	1	HMA
....aauugcacucAucccggccugc....................	1	1	HMA
....aauugcacucgucccggUcugc....................	4	1	HMA
....aauugcacGcgucccggccugc....................	3	1	HMA
....aauugcGcucgucccggccugc....................	1	1	HMA
....aauugGacucgucccggccugc....................	2	1	HMA
....aauugcacucgucccggccugUc...................	12	1	HMA
....aauugcacucgucccggccugcA...................	668	1	HMA
....aauugcacucgucccggcAugcc...................	1	1	HMA
....aauugcacucgucccggccugcc...................	75	0	HMA
....Uauugcacucgucccggccugcc...................	25	1	HMA
....auugcacucgucccggccu......................	2	0	HMA
....auugcacucgucccggccug.....................	111	0	HMA
....auugcacucgucGcggccugc....................	1	1	HMA
....auugcacucgucUcggccugc....................	1	1	HMA
....auugcacucgucccggccugc....................	858	0	HMA
....auuAcacucgucccggccugc....................	1	1	HMA
....auugcacuAgucccggccugc....................	1	1	HMA
....auugcacucgucccggccugA....................	2	1	HMA
....auugcacucgucccggccCgc....................	1	1	HMA
....auugcacCcgucccggccugc....................	1	1	HMA
....auugUacucgucccggccugc....................	2	1	HMA
....auugcacucgucccggccugU....................	4	1	HMA
....auugcacucgucccggccugcA...................	543	1	HMA
....uugcacucgucccgAccugcc...................	1	1	HMA
....uugcacucgucccggccugcA...................	23	1	HMA
....uugcacucgucccggccugcc...................	4	0	HMA
...........................ugacagguuguaacgagugcaa...	1	0	HS4
...............................agguuguaacgagugcaac..	1	0	HS4
...............................agguuguaacgagugcaacg...	8	0	HS4
...............................agguuguaacgagugUaacg...	1	1	HS4
...............................agguuguaacgagugcaacgc..	1	0	HS4
...............................agguuguaacgagugcaacgcuU..	1	1	HS4
..................................uuguaacgagugcaacgcuUu...	1	1	HS4
....Uaaauugcacucgucccggccugc....................	1	1	HS4
....aaauugcacucgucccgg.........................	2	0	HS4
....aaauugcacucgucccggc........................	1	0	HS4
....aaauugcacucgucccggcc.......................	9	0	HS4
....Uaauugcacucgucccggccu......................	1	1	HS4
....aaauugGacucgucccggccu......................	1	1	HS4

Star Mature

uaaaccggauguuuaaaaaggcaaaagugacagguuguaacgagugcaacgcuguauuugcgcuggaacaaauugcacucgucccggccugcccacccuacauugcauaaac			
....aaauugcacucgucccggccu.......................	174	0	HS4
....aaauugcacucguUccggccu.......................	5	1	HS4
....aaauugcacucguccUggccu.......................	1	1	HS4
....aaaGugcacucgucccggccu.......................	1	1	HS4
....aaauugcacucguGccggccu.......................	1	1	HS4
....aaauugcacucgucccggccCg......................	1	1	HS4
....aaauugcacucgucccAgccug......................	1	1	HS4
....aaauugcacucguUccggccug......................	2	1	HS4
....aaGuugcacucgucccggccug......................	1	1	HS4
....aaauugcacucgucccggccug......................	344	0	HS4
....aaauugcacCcgucccggccug......................	1	1	HS4
....aaauugcacucgucccggccuU......................	5	1	HS4
....aaauugUacucgucccggccug......................	1	1	HS4
....aaauugcacucgucccggUcug......................	1	1	HS4
....Uaauugcacucgucccggccug......................	3	1	HS4
....aaauugcacucCucccggccug......................	2	1	HS4
....aaauugcacAcgucccggccug......................	1	1	HS4
....aaauugcacucguccUggccugc.....................	1	1	HS4
....aaauugGacucgucccggccugc.....................	1	1	HS4
....aaauugcacucgucUcggccugc.....................	2	1	HS4
....aaauugcacCcgucccggccugc.....................	1	1	HS4
....Uaauugcacucgucccggccugc.....................	16	1	HS4
....aaauugcacucgucccUgccugc.....................	1	1	HS4
....aaauugcUcucgucccggccugc.....................	1	1	HS4
....aaauugcacucgucccggccugU.....................	73	1	HS4
....aaauugcacucgucccggccugA.....................	2	1	HS4
....aaauugcacucgucccgAccugc.....................	2	1	HS4
....aaauugcacucgucccggcUugc.....................	1	1	HS4
....aaauugcacucgucccggccGgc.....................	2	1	HS4
....aaauugcacucgucccggccugc.....................	725	0	HS4
....aaauugcacucgucccggccugcA....................	2	1	HS4
....aauugcacucgucccUgc.........................	1	1	HS4
....aauugcacucgucccggc.........................	3	0	HS4
....aauugcacucgucccUgcc........................	1	1	HS4
....aauugcacucgucccggcc........................	107	0	HS4
....aauugcacucgucccggcA........................	1	1	HS4
....aauugcacucgucccgAcc........................	1	1	HS4
....aauuUcacucgucccggccu.......................	1	1	HS4
....Gauugcacucgucccggccu.......................	2	1	HS4
....aauugcacuAgucccggccu.......................	2	1	HS4
....aauugcacucguUccggccu.......................	15	1	HS4
....aauugcacucgucccUgccu.......................	4	1	HS4
....aaAugcacucgucccggccu.......................	1	1	HS4
....aauuCcacucgucccggccu.......................	6	1	HS4
....aauugcacucgucAcggccu.......................	1	1	HS4
....aauugcacucUucccggccu.......................	1	1	HS4
....aauugcacucgucccggccC.......................	7	1	HS4
....aauugcacucguccUggccu.......................	6	1	HS4
....aaCugcacucgucccggccu.......................	2	1	HS4
....aauugUacucgucccggccu.......................	4	1	HS4
....aauugcacucgucccggccA.......................	11	1	HS4
....aauugcacucgucccggUcu.......................	6	1	HS4
....Cauugcacucgucccggccu.......................	1	1	HS4
....aauugcacucgucccCgccu.......................	4	1	HS4
....aGuugcacucgucccggccu.......................	1	1	HS4
....aauugcacucgucccgAccu.......................	9	1	HS4
....aauugcacucgucccgUccu.......................	3	1	HS4
....aauugcacucgucccggccu.......................	4166	0	HS4
....aauugcGcucgucccggccu.......................	2	1	HS4
....aauugcacucgucUcggccu.......................	20	1	HS4
....aauugcacucgucccgCccu.......................	2	1	HS4
....aauugcacucAucccggccu.......................	2	1	HS4
....aauugcacucCucccggccu.......................	1	1	HS4
....aauugcUcucgucccggccug......................	4	1	HS4
....aauugcacucgucccggccuA......................	33	1	HS4
....aaAugcacucgucccggccug......................	1	1	HS4
....aauugcacucgucccggcUug......................	1	1	HS4
....aauugcacucgucUcggccug......................	20	1	HS4
....aauCgcacucgucccggccug......................	2	1	HS4
....aauugcacAcgucccggccug......................	1	1	HS4

```
                                                Star                                Mature
uaaaccggauguuuaaaaaggcaaaagugacagguuguaacgagugcaacgcuguauuugcgcuggaacaaauugcacucgucccggccugcccacccuacauugcauaaac
......................................................................aauugcCcucgucccggccug.....................   2      1   HS4
......................................................................aauugcacucgucccggccug.....................   7743   0   HS4
......................................................................aauugcacucgucccggccuU.....................   125    1   HS4
......................................................................aauugcacucgucccggcAug.....................   1      1   HS4
......................................................................aaGugcacucgucccggccug.....................   1      1   HS4
......................................................................aCuugcacucgucccggccug.....................   1      1   HS4
......................................................................aauugcacucguUccggccug.....................   22     1   HS4
......................................................................aauugcacucgucccgUccug.....................   4      1   HS4
......................................................................aauugcaUucgucccggccug.....................   1      1   HS4
......................................................................aauugcacucguAccggccug.....................   1      1   HS4
......................................................................aauugcacucUucccggccug.....................   3      1   HS4
......................................................................aauugcacucgucccggUcug.....................   9      1   HS4
......................................................................aauugcacucgucccgCccug.....................   6      1   HS4
......................................................................Gauugcacucgucccggccug.....................   2      1   HS4
......................................................................aauugcacucguccUggccug.....................   7      1   HS4
......................................................................aauugcacucgucccgAccug.....................   18     1   HS4
......................................................................aauugcacucgucccggccAg.....................   1      1   HS4
......................................................................aauugcacucgucccggccCg.....................   6      1   HS4
......................................................................aGuugcacucgucccggccug.....................   1      1   HS4
......................................................................aauugcacucgCcccggccug.....................   1      1   HS4
......................................................................aauugcacucgucccggccGg.....................   1      1   HS4
......................................................................aauuCcacucgucccggccug.....................   7      1   HS4
......................................................................aauugcaAucgucccggccug.....................   1      1   HS4
......................................................................aauugcacucgucccUgccug.....................   6      1   HS4
......................................................................aauuAcacucgucccggccug.....................   1      1   HS4
......................................................................aauugcacucCucccggccug.....................   7      1   HS4
......................................................................aauugcacuAgucccggccug.....................   2      1   HS4
......................................................................aauugcacucgucccggccuC.....................   4      1   HS4
......................................................................aauugUacucgucccggccug.....................   6      1   HS4
......................................................................aauugcacucAucccggccug.....................   2      1   HS4
......................................................................aauuUcacucgucccggccug.....................   4      1   HS4
......................................................................aauugcacucgucccAgccug.....................   1      1   HS4
......................................................................aauugcacucgAcccggccug.....................   4      1   HS4
......................................................................aauugcacucgucccCgccug.....................   5      1   HS4
......................................................................aauugcGcucgucccggccug.....................   12     1   HS4
......................................................................aauugcacucgucGcggccug.....................   2      1   HS4
......................................................................aauugcacuUgucccggccugc....................   13     1   HS4
......................................................................aauuAcacucgucccggccugc....................   8      1   HS4
......................................................................aauugcaUucgucccggccugc....................   14     1   HS4
......................................................................aauugcacucgucccAgccugc....................   6      1   HS4
......................................................................Gauugcacucgucccggccugc....................   11     1   HS4
......................................................................aauugcacucgucccggcGugc....................   1      1   HS4
......................................................................aauugcacucgucGcggccugc....................   7      1   HS4
......................................................................aauugcacucgucccggccugA....................   73     1   HS4
......................................................................aauugcacucgucccggcUugc....................   14     1   HS4
......................................................................aauuUcacucgucccggccugc....................   12     1   HS4
......................................................................aauGgcacucgucccggccugc....................   3      1   HS4
......................................................................aauugcaAucgucccggccugc....................   1      1   HS4
......................................................................aauugcacAcgucccggccugc....................   1      1   HS4
......................................................................aauugcacucgAcccggccugc....................   3      1   HS4
......................................................................aauugcacucgucccggUcugc....................   29     1   HS4
......................................................................aauugUacucgucccggccugc....................   27     1   HS4
......................................................................aauugcGcucgucccggccugc....................   39     1   HS4
......................................................................aauCgcacucgucccggccugc....................   8      1   HS4
......................................................................aauugcacucgucUcggccugc....................   58     1   HS4
......................................................................aauugcacucguUccggccugc....................   85     1   HS4
......................................................................aauugcacucgucccgUccugc....................   11     1   HS4
......................................................................aGuugcacucgucccggccugc....................   4      1   HS4
......................................................................aauugcacucgGcccggccugc....................   14     1   HS4
......................................................................aauugcacucguccGggccugc....................   1      1   HS4
......................................................................Cauugcacucgucccggccugc....................   9      1   HS4
......................................................................aauAgcacucgucccggccugc....................   1      1   HS4
......................................................................aauugAacucgucccggccugc....................   1      1   HS4
......................................................................aauugcacucgucccggGcugc....................   1      1   HS4
......................................................................aauugcacucgucccggccGgc....................   21     1   HS4
......................................................................aaGugcacucgucccggccugc....................   6      1   HS4
......................................................................aauugcacucgucccgAccugc....................   33     1   HS4
......................................................................aauugcacucgucccggccugU....................   2103   1   HS4
......................................................................aauugcUcucgucccggccugc....................   4      1   HS4
......................................................................aaAugcacucgucccggccugc....................   2      1   HS4
```

Star **Mature**

```
uaaaccggauguuuaaaaaggcaaaagugacagguuguaacgagugcaacgcuguauuugcgcuggaacaaauugcacucgucccggccugcccacccuacauugcauaaac
......................................................................aauugcacucgucccggccCgc....................  20     1  HS4
......................................................................aauugcacucguccAggccugc....................  4      1  HS4
......................................................................aauugcacucAucccggccugc....................  7      1  HS4
......................................................................aauugcacuGgucccggccugc....................  1      1  HS4
......................................................................aauugcacucgucccUgccugc....................  19     1  HS4
......................................................................aauugcCcucgucccggccugc....................  5      1  HS4
......................................................................aauuCcacucgucccggccugc....................  9      1  HS4
......................................................................aauugcacucgucccggccugc....................  41508  0  HS4
......................................................................aauugcacucgucccggcAugc....................  4      1  HS4
......................................................................aauugcacucUucccggccugc....................  3      1  HS4
......................................................................aauugcacucgucccCgccugc....................  16     1  HS4
......................................................................aauugcacucguAccggccugc....................  1      1  HS4
......................................................................aauugcacucgucccggccuCc....................  13     1  HS4
......................................................................aauugcacuAgucccggccugc....................  11     1  HS4
......................................................................aauugcacucgucccggccuAc....................  71     1  HS4
......................................................................aaCugcacucgucccggccugc....................  7      1  HS4
......................................................................aauugcacucguccUggccugc....................  23     1  HS4
......................................................................aauugcacucgCcccggccugc....................  25     1  HS4
......................................................................aauugcacucgucccggccugG....................  1      1  HS4
......................................................................aauugcacucgucAcggccugc....................  5      1  HS4
......................................................................aauugcacucgucccggccuUc....................  8      1  HS4
......................................................................aauugcacucgucccggAcugc....................  3      1  HS4
......................................................................aauugcacucgucccggccAgc....................  8      1  HS4
......................................................................aauugcacucgucccgCccugc....................  12     1  HS4
......................................................................aCuugcacucgucccggccugc....................  2      1  HS4
......................................................................aauugcacCcgucccggccugc....................  2      1  HS4
......................................................................aauugcacucgucccggccugcc...................  49     0  HS4
......................................................................aauugcacucgucccggccugUc...................  1      1  HS4
......................................................................aauugcacucgucccggccugcG...................  4      1  HS4
......................................................................Uauugcacucgucccggccugcc...................  64     1  HS4
......................................................................aauugcacucgucccggccugcA...................  1023   1  HS4
......................................................................aauugcacucgucccggccugcUc..................  1      1  HS4
......................................................................aauugcacucgucccggccugccU..................  9      1  HS4
.......................................................................auugcacucgucccggccu......................  53     0  HS4
.......................................................................aAugcacucgucccggccu......................  1      1  HS4
.......................................................................auugcacucguUccggccu......................  1      1  HS4
.......................................................................auugcacucguUccggccug.....................  2      1  HS4
.......................................................................auugcacucgucccggccug.....................  109    0  HS4
.......................................................................auAgcacucgucccggccug.....................  1      1  HS4
.......................................................................auugcacucgucccggccuU.....................  1      1  HS4
.......................................................................auuUcacucgucccggccug.....................  1      1  HS4
.......................................................................auugcacucguGccggccugc....................  1      1  HS4
.......................................................................Uuugcacucgucccggccugc....................  3      1  HS4
.......................................................................auugcacucgucUcggccugc....................  1      1  HS4
.......................................................................auugUacucgucccggccugc....................  2      1  HS4
.......................................................................auugcacucgucccgAccugc....................  2      1  HS4
.......................................................................aGugcacucgucccggccugc....................  1      1  HS4
.......................................................................auugcacucgucccggccAgc....................  1      1  HS4
.......................................................................auugcaAucgucccggccugc....................  1      1  HS4
.......................................................................auugcacucgucccggccugc....................  1410   0  HS4
.......................................................................auugcaGucgucccggccugc....................  1      1  HS4
.......................................................................auugcacucguUccggccugc....................  5      1  HS4
.......................................................................auugcacucgucccggccGgc....................  3      1  HS4
.......................................................................auugcacucgucccggAcugc....................  1      1  HS4
.......................................................................auugcacucgucccggccCgc....................  1      1  HS4
.......................................................................auugcacucguccAggccugc....................  1      1  HS4
.......................................................................auuCcacucgucccggccugc....................  1      1  HS4
.......................................................................auugcacucguccUggccugc....................  3      1  HS4
.......................................................................auuUcacucgucccggccugc....................  4      1  HS4
.......................................................................auugcacucAucccggccugc....................  1      1  HS4
.......................................................................aAugcacucgucccggccugc....................  1      1  HS4
.......................................................................auugcacucgucccggccugA....................  1      1  HS4
.......................................................................auugcacucgucccggccugU....................  27     1  HS4
.......................................................................auugcacucgucccggccAgcc...................  1      1  HS4
.......................................................................auugcacucgucccggccugcG...................  1      1  HS4
.......................................................................auugcacucgucccggccugcA...................  88     1  HS4
.......................................................................auugcacucgucccggccugcc...................  149    0  HS4
.......................................................................auugcacucgucccggccugccU..................  20     1  HS4
.......................................................................auugcacucgucccggccugccA..................  2      1  HS4
........................................................................uugcacucgucccggccug.....................  5      0  HS4
```

```
                                Star                                     Mature
uaaaccggauguuuaaaaaggcaaaagugacagguuguaacgagugcaacgcuguauuugcgcuggaacaaauugcacucgucccggccugcccacccuacauugcauaaac
........................................................................uugcacucgucccgAccugc....................   1     1   HS4
........................................................................uugcacucgucccggccugU....................   1     1   HS4
........................................................................uugcacucguccUggccugc....................   1     1   HS4
........................................................................uugcacucguUccggccugc....................   1     1   HS4
........................................................................uugcacucgucccggccugc....................   49    0   HS4
........................................................................uuUcacucgucccggccugc....................   1     1   HS4
........................................................................uugcacucgucccggccugcG...................   1     1   HS4
........................................................................uugcacucgucccggccugcA...................   1     1   HS4
.........................................................................ugcacucgucccggccug.....................   2     0   HS4
.........................................................................ugcacucgCcccggccugc....................   1     1   HS4
.........................................................................ugcacucgucccggccugc....................   8     0   HS4
.........................................................................uUcacucgucccggccugc....................   2     1   HS4
..........................................................................gcacucgucccggccugc....................   14    0   HS4
..........................................................................gcacucgucccggccugU....................   3     1   HS4
..........................................................................Ucacucgucccggccugc....................   4     1   HS4
..........................................................................Ccacucgucccggccugc....................   1     1   HS4
..........................................................................gcacucgucccggccugcc...................   2     0   HS4

...........................ugacagguuguaacgagugcaa...............................................................   1     0   J17
...............................agguuguaacgagugcaacg.............................................................   2     0   J17
...............................agguuguaacgagugcaacgcu...........................................................   3     0   J17
...............................agguuguaacgagugcaacgcug..........................................................   1     0   J17
...............................agguuguaacgagugcaacgcuU..........................................................   1     1   J17
...............................agguuguaacgagugcaacgcugu.........................................................   1     0   J17
.....................................................................aaauugcacucgucccggc........................   1     0   J17
.....................................................................aaauugcacucgucccggcc.......................   5     0   J17
.....................................................................aaauugcacucgucccggccu......................   107   0   J17
.....................................................................aaauugUacucgucccggccu......................   1     1   J17
.....................................................................Uaauugcacucgucccggccu......................   2     1   J17
.....................................................................aaauugcacucgucccggccG......................   1     1   J17
.....................................................................aaauugcacucgucUcggccu......................   1     1   J17
.....................................................................Uaauugcacucgucccggccug.....................   7     1   J17
.....................................................................aaauuAcacucgucccggccug.....................   1     1   J17
.....................................................................aaauugcacucgucccggccug.....................   339   0   J17
.....................................................................aaauugUacucgucccggccug.....................   1     1   J17
.....................................................................aaauugcacucgucccggccCg.....................   1     1   J17
.....................................................................aaauugcacucgucccggccuU.....................   1     1   J17
.....................................................................aaauugcacucgucccgAccugc....................   1     1   J17
.....................................................................aaauugcacucgucccggccugU....................   77    1   J17
.....................................................................Uaauugcacucgucccggccugc....................   23    1   J17
.....................................................................aaauugcacucgucccggccugc....................   414   0   J17
.....................................................................Caauugcacucgucccggccugc....................   1     1   J17
.....................................................................aaauugUacucgucccggccugc....................   1     1   J17
.....................................................................aaauugcacucgucccggccAgc....................   1     1   J17
.....................................................................aaauugcacucgucccUgccugc....................   1     1   J17
.....................................................................aaauugcacucgucccggccugcA...................   5     1   J17
......................................................................aauugcacucgucccggc........................   1     0   J17
......................................................................aauugcacucgucccggcA.......................   1     1   J17
......................................................................aauugcacucgucccggGc.......................   1     1   J17
......................................................................aauugcacucgucccggcc.......................   76    0   J17
......................................................................Cauugcacucgucccggccu......................   1     1   J17
......................................................................aauugcacucgCcccggccu......................   2     1   J17
......................................................................aauugcacucgucccggccu......................   2098  0   J17
......................................................................aauugcacucguUccggccu......................   1     1   J17
......................................................................aauugcacucguccAggccu......................   1     1   J17
......................................................................aauugcacucgucccggUcu......................   1     1   J17
......................................................................aauugcacucgucccggccA......................   8     1   J17
......................................................................aauugcaUucgucccggccu......................   1     1   J17
......................................................................aauugcacAcgucccggccu......................   2     1   J17
......................................................................aauugcacucgGcccggccu......................   1     1   J17
......................................................................aauugcacucguccUggccu......................   4     1   J17
......................................................................aauugcacucgucccgAccu......................   2     1   J17
......................................................................aauugcUcucgucccggccu......................   2     1   J17
......................................................................aauugcacucgucUcggccu......................   3     1   J17
......................................................................aauugUacucgucccggccu......................   2     1   J17
......................................................................aauugcacucgucccgCccu......................   1     1   J17
......................................................................aauugcacucgucccggccC......................   1     1   J17
......................................................................aauugcacuUgucccggccu......................   3     1   J17
......................................................................aauugcacucgucccggccug.....................   6299  0   J17
......................................................................aauugcacucAucccggccug.....................   1     1   J17
```

Star **Mature**

uaaaccggauguuuaaaaaggcaaaagugacagguuguaacgagugcaacgcuguauuugcgcuggaacaaauugcacucgucccggccugcccacccuacauugcauaaac			
....aauugcacucgCcccggccug.....................	1	1	J17
....aauugcacucguAccggccug.....................	1	1	J17
....aauugcacAcgucccggccug.....................	2	1	J17
....aauugcacucgAcccggccug.....................	4	1	J17
....aauugcacucguccUggccug.....................	4	1	J17
....aauugcaUucgucccggccug.....................	2	1	J17
....aauuUcacucgucccggccug.....................	1	1	J17
....aauAgcacucgucccggccug.....................	3	1	J17
....aauugcacucgGcccggccug.....................	1	1	J17
....aauugcacucgucccggcUug.....................	1	1	J17
....aauugcacucgucccggUcug.....................	4	1	J17
....aauugcacucguUccggccug.....................	21	1	J17
....aauugcacucgucccgCccug.....................	2	1	J17
....aauugcacucgucccggGcug.....................	1	1	J17
....aauugcacucgucccggccCg.....................	7	1	J17
....aauugcacucCucccggccug.....................	1	1	J17
....aauugcUcucgucccggccug.....................	1	1	J17
....aauugcacucgucccggccuU.....................	39	1	J17
....aauugcacucgucccggccuC.....................	2	1	J17
....aauGgcacucgucccggccug.....................	1	1	J17
....aauugcacucguGccggccug.....................	1	1	J17
....Cauugcacucgucccggccug.....................	2	1	J17
....aauugcacucgucccggccuA.....................	20	1	J17
....aauugcacucgucccgUccug.....................	2	1	J17
....aauugcacucgucUcggccug.....................	18	1	J17
....Gauugcacucgucccggccug.....................	1	1	J17
....aaAugcacucgucccggccug.....................	1	1	J17
....aauugcacucgucccggccGg.....................	2	1	J17
....aauugcacucgucccgAccug.....................	4	1	J17
....aUuugcacucgucccggccug.....................	4	1	J17
....aauugcacucgucccAgccug.....................	1	1	J17
....aauugcacCcgucccggccug.....................	2	1	J17
....aGuugcacucgucccggccug.....................	1	1	J17
....aauugcacucguccAggccug.....................	1	1	J17
....aauugUacucgucccggccug.....................	6	1	J17
....aauugcacuAgucccggccug.....................	1	1	J17
....aauugcacucgucccggccAg.....................	1	1	J17
....aauugcacucgucccggccugG....................	4	1	J17
....aauAgcacucgucccggccugc....................	7	1	J17
....aauugcacucgucccggcGugc....................	1	1	J17
....aauugcaGucgucccggccugc....................	2	1	J17
....aauugcacucgucAcggccugc....................	2	1	J17
....aaAugcacucgucccggccugc....................	1	1	J17
....aauugcacuAgucccggccugc....................	13	1	J17
....aauugcacucCucccggccugc....................	3	1	J17
....aauugcacucguAccggccugc....................	1	1	J17
....aauugcacAcgucccggccugc....................	10	1	J17
....aauugcacGcgucccggccugc....................	1	1	J17
....aauugcacucgucccggccuCc....................	12	1	J17
....aauugUacucgucccggccugc....................	23	1	J17
....aauugcacucgucccggccuAc....................	23	1	J17
....aauugcacucgCcccggccugc....................	3	1	J17
....aauugcacucgucccggUcugc....................	21	1	J17
....aauuAcacucgucccggccugc....................	11	1	J17
....aauugcacucgGcccggccugc....................	5	1	J17
....aauugcacucgucccggccGgc....................	13	1	J17
....aauuCcacucgucccggccugc....................	2	1	J17
....aauugcacucgucGcggccugc....................	2	1	J17
....aauugcacucgucccggccugU....................	1956	1	J17
....aauuUcacucgucccggccugc....................	1	1	J17
....aCuugcacucgucccggccugc....................	1	1	J17
....aauugcacucgucccggccCgc....................	12	1	J17
....aauugcacucgucccggccugA....................	107	1	J17
....aauugcacucgucccCgccugc....................	13	1	J17
....aauugcacucgucccggccugc....................	27259	0	J17
....aauugcaUucgucccggccugc....................	15	1	J17
....aauugcUcucgucccggccugc....................	6	1	J17
....aauugcacucguccGggccugc....................	2	1	J17
....aauugcacucgucccggGcugc....................	1	1	J17
....aauugcacucguUccggccugc....................	43	1	J17

Star Mature

```
uaaaccggauguuuaaaaaggcaaaagugacagguuguaacgagugcaacgcuguauuugcgcuggaacaaauugcacucgucccggccugcccacccuacauugcauaaac
......................................................................aauugAacucgucccggccugc....................  2     1  J17
......................................................................aauugcacucgucccUgccugc....................  7     1  J17
......................................................................aauugcacucgucccgAccugc....................  18    1  J17
......................................................................aauugGacucgucccggccugc....................  3     1  J17
......................................................................aauugcacucUucccggccugc....................  3     1  J17
......................................................................aauugcacucgucUcggccugc....................  34    1  J17
......................................................................aGuugcacucgucccggccugc....................  8     1  J17
......................................................................aauugcacucgucccggccAgc....................  18    1  J17
......................................................................aauugcacucgucccggcUugc....................  14    1  J17
......................................................................aaGugcacucgucccggccugc....................  2     1  J17
......................................................................aauugcacucgucccggccuUc....................  11    1  J17
......................................................................aauugcacucgAcccggccugc....................  5     1  J17
......................................................................aauugcacuUgucccggccugc....................  9     1  J17
......................................................................aauugcacuGgucccggccugc....................  9     1  J17
......................................................................Cauugcacucgucccggccugc....................  5     1  J17
......................................................................aauugcacucguccUggccugc....................  14    1  J17
......................................................................aauugcacucgucccggcAugc....................  1     1  J17
......................................................................Gauugcacucgucccggccugc....................  9     1  J17
......................................................................aauCgcacucgucccggccugc....................  2     1  J17
......................................................................aauugcacucgucccgCccugc....................  11    1  J17
......................................................................aauugcacucgucccAgccugc....................  6     1  J17
......................................................................aauugcGcucgucccggccugc....................  5     1  J17
......................................................................aauugcacucgucccgUccugc....................  8     1  J17
......................................................................aUuugcacucgucccggccugc....................  8     1  J17
......................................................................aauugcacucgucccggAcugc....................  1     1  J17
......................................................................Uauugcacucgucccggccugcc...................  52    1  J17
......................................................................aauugcacucgucccggccugcA...................  1013  1  J17
......................................................................aauugcacucgucccggccugcc...................  47    0  J17
......................................................................aauugcacucgucccggccugcG...................  13    1  J17
......................................................................aauugcacucgucccggccugUc...................  4     1  J17
......................................................................aauugcacucgucccggccugccU..................  11    1  J17
......................................................................aauugcacucgucccggccugcUc..................  2     1  J17
......................................................................aauugcacucgucccggccugccG..................  1     1  J17
......................................................................aauugcacucgucccggccugccA..................  4     1  J17
.......................................................................auugcacuUgucccggcc.......................  1     1  J17
.......................................................................auugcacuUgucccggccu......................  1     1  J17
.......................................................................auugcacucgucccggccu......................  10    0  J17
.......................................................................auugcacucgucccggccuU.....................  1     1  J17
.......................................................................auugcacucguccUggccug.....................  1     1  J17
.......................................................................auugcacucgucccggccug.....................  145   0  J17
.......................................................................auugcacuUgucccggccugc....................  1     1  J17
.......................................................................auugcacucgucccggccuAc....................  1     1  J17
.......................................................................auugcacucgucccgAccugc....................  2     1  J17
.......................................................................aAugcacucgucccggccugc....................  1     1  J17
.......................................................................auAgcacucgucccggccugc....................  1     1  J17
.......................................................................Guugcacucgucccggccugc....................  1     1  J17
.......................................................................auugcacucgucccggccugc....................  1074  0  J17
.......................................................................auugcacucgucccgUccugc....................  1     1  J17
.......................................................................auugcacucgucccggccugU....................  40    1  J17
.......................................................................auugcacucgucccggccGgc....................  1     1  J17
.......................................................................auugcacucgucccggccugA....................  1     1  J17
.......................................................................auugcacucguUccggccugc....................  2     1  J17
.......................................................................auugcacAcgucccggccugc....................  3     1  J17
.......................................................................auugcacuGgucccggccugc....................  1     1  J17
.......................................................................auugcacucgucccCgccugc....................  1     1  J17
.......................................................................auugcacuAgucccggccugc....................  1     1  J17
.......................................................................auugcacucgucccggcAugcc...................  1     1  J17
.......................................................................auugcacucgucccggccugcc...................  117   0  J17
.......................................................................auugcacucgucccggccugcG...................  2     1  J17
.......................................................................auugcacucgucccggccugUc...................  1     1  J17
.......................................................................auugcacucgucccggUcugcc...................  1     1  J17
.......................................................................auugcacucgucccggccugcA...................  65    1  J17
.......................................................................auugcacucgucccggccugccU..................  21    1  J17
.......................................................................auugcacucgucccggccugccA..................  1     1  J17
.......................................................................auugcacucgucccggccugcUc..................  1     1  J17
........................................................................uugcacucgucccggccuU.....................  2     1  J17
........................................................................uugcacucgucccggccug.....................  9     0  J17
........................................................................uugcacucgucccggccuA.....................  1     1  J17
........................................................................uugcacucgucccggccugc....................  42    0  J17
........................................................................uugcaAucgucccggccugc....................  1     1  J17
```

Star Mature

```
uaaaccggauguuuaaaaaggcaaaagugacagguuguaacgagugcaacgcuguauuugcgcuggaacaaauugcacucgucccggccugcccacccuacauugcauaaac
........................................................................uugcacucgucccggccugU....................   1   1   J17
........................................................................uugcacucgucccggccugcc...................   2   0   J17
........................................................................uugcacucgucccggccugcA...................   3   1   J17
........................................................................uugcacucgucccggccugccU..................   1   1   J17
........................................................................uugcacucgucccggccugcUc..................   1   1   J17
.........................................................................ugcacucgucccggccug.....................   7   0   J17
.........................................................................ugcacucgucccggccugc....................   9   0   J17
.........................................................................ugcacucgucccggccugcA...................   4   1   J17
..........................................................................gcacucgucccggccugc....................   13  0   J17
..........................................................................gcacucgucccggccugU....................   4   1   J17

...............................agguuguaacgagugcaacg.............................................................   2   0   EGG
.....................................................................aaauugcacucgucccggccuU.....................   1   1   EGG
.....................................................................aaauugcacucgucccggccugc....................   1   0   EGG
......................................................................aauugcacucgucccggc........................   1   0   EGG
......................................................................aauugcacucgucccggcA.......................   2   1   EGG
......................................................................aauugcacucgucccggcc.......................   2   0   EGG
......................................................................aauugcGcucgucccggccu......................   1   1   EGG
......................................................................aauugcacucgucccggccu......................   37  0   EGG
......................................................................aauugcacucguUccggccu......................   1   1   EGG
......................................................................aauugcacucguUccggccug.....................   1   1   EGG
......................................................................aauugcacucgucccUgccug.....................   1   1   EGG
......................................................................aauugcacucgucccggccug.....................   45  0   EGG
......................................................................aauugcacucgucccggccCg.....................   1   1   EGG
......................................................................aauugcacucgucccggccuU.....................   5   1   EGG
......................................................................aauugcacucgucccggccugc....................   70  0   EGG
......................................................................aauugcacucgucccggccugU....................   19  1   EGG
......................................................................aauugcacucgucccggccugcA...................   8   1   EGG
......................................................................aauugcacucgucccggccugcG...................   1   1   EGG
.......................................................................auugcacucgucccggccu......................   4   0   EGG
.......................................................................auugcacucgucccggccug.....................   4   0   EGG
.......................................................................auugcacucgucccggccugc....................   9   0   EGG
........................................................................uugcacucgucccggccug.....................   2   0   EGG
........................................................................uugcacucgucccggccugc....................   1   0   EGG
.........................................................................ugcacucgucccggccugc....................   1   0   EGG
.........................................................................ugcacucgucccggccugccA..................   1   1   EGG
..........................................................................gcacucgucccggccugc....................   1   0   EGG
..........................................................................gcacucgucccggccugU....................   2   1   EGG
```

Provisional ID	:	SczTNLB_2740_1876
Score total	:	22.8
Score for star read(s)	:	3.9
Score for read counts	:	14.4
Score for mfe	:	0
Score for randfold	:	1.6
Score for cons. seed	:	3
Total read count	:	40
Mature read count	:	23
Loop read count	:	0
Star read count	:	17

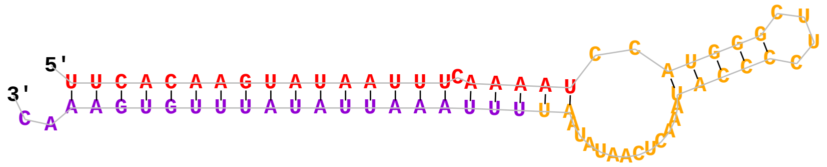

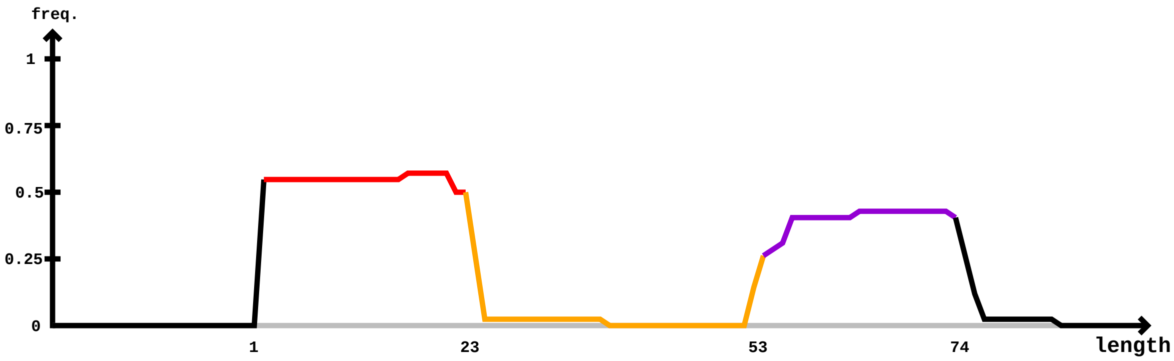

Mature Star

```
5'- acguauauucuaugcucuuuuucacaaguauaauuucaaaauccaugggcuuccccauaaacucaauauaauuuuaaauuauauuugugaaacugaauauaguaauggguua  -3'   obs
    acguauauucuaugcucuuuuucacaaguauaauuucaaaauccaugggcuuccccauaaacucaauauaauuuuaaauuauauuugugaaacugaauauaguaauggguua         exp
    ((.(((...(((((.((..((((((((((((((((.(((((..(((((....))))).........)))))))))))))))))))))..)).))))).))).))..   reads   mm   sample
    ....................uucacaaguauaauuucaaU........................................................................   1   1   HS4
    ....................uucacaaguauaauuucaaa........................................................................   1   0   HS4
    ....................uucacaaguauaauuucaaaau......................................................................   6   0   HS4
    ....................uucacaaguauaauuucaaaauA.....................................................................   1   1   HS4
    ....................uucacaaguauaauuucaaaauU.....................................................................   1   1   HS4
    ...................................ucaaaauccaugggcuucccc........................................................   1   0   HS4
    .......................................................................uuuuaaauuauauuugugaaac...................   1   0   HS4
    .......................................................................uuuuaaauuauauuugugaaacu..................   1   0   HS4
    ........................................................................uuuaaauuauauuugugaaac...................   1   0   HS4

    ....................uucacaaguauaauuucaaaau......................................................................   1   0   J17

    ........................................................................uuuaaauuauauuugugaaac...................   2   0   HFA

    ....................uucacaaguauaauuucaaa........................................................................   1   0   HS5
    ....................uucacaaguauaauuucaaaau......................................................................   3   0   HS5
    .......................................................................uuuuaaauuauauuugugaaac...................   1   0   HS5
    .......................................................................uuuuaaauuauauuugugaaacu..................   1   0   HS5
    ..........................................................................uaaauuauauuugugaaacu..................   1   0   HS5

    ....................uucacaaguauaauuucaaaau......................................................................   3   0   HS7
    ....................uucacaaguauaauuucaaaauA.....................................................................   1   1   HS7
    .......................................................................uuuuaaauuauauuugugaaa....................   1   0   HS7

    ....................uucacaaguauaauuucaaaau......................................................................   3   0   HA1
    ........................................................................uuuaaauuauauuugugaaac...................   1   0   HA1
    ........................................................................uuuaaauuauauuugugaaacA..................   1   1   HA1
    ..........................................................................uaaauuauauuugugaaacu..................   1   0   HA1

    ...........................................................................aaauuauauuugugaaacug.................   4   0   HS1
    ..................................................................................auuugugaaacugaauauagu.........   1   0   HS1

    ....................uucacaaguauaauuucaaaau......................................................................   1   0   HS6
```

Mature Star

acguauauucuaugcucuuuuucacaaguauaauuucaaaauccaugggcuuccccauaaacucaauauaauuuuaaauuauauuugugaaacugaauauaguaauggguua

....uuuuaaauuauauuugugaaac................... 1 0 HS6

Provisional ID	:	SczTNLB_2740_1990
Score total	:	87767.5
Score for star read(s)	:	3.9
Score for read counts	:	87757.3
Score for mfe	:	1.6
Score for randfold	:	1.6
Score for cons. seed	:	3
Total read count	:	172144
Mature read count	:	96023
Loop read count	:	0
Star read count	:	76121

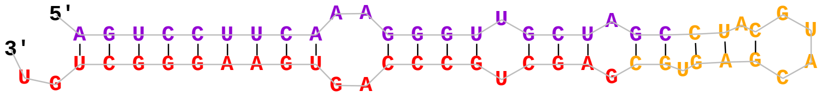

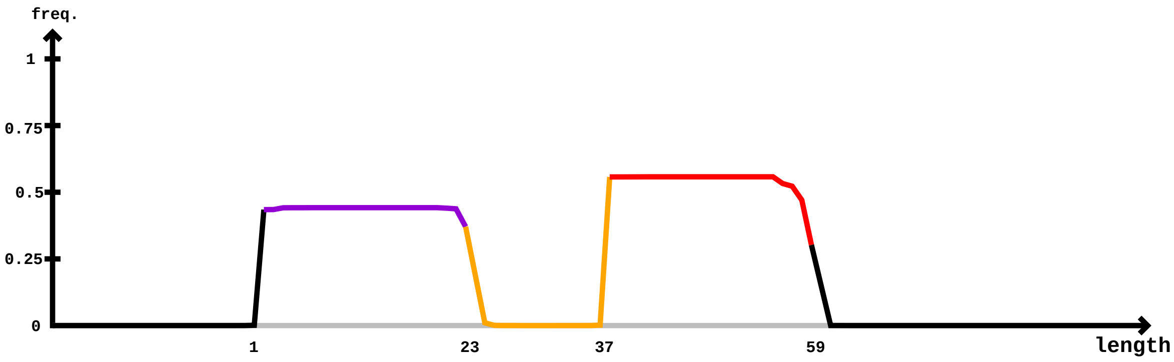

Star Mature

```
5'- guuacauugaacuguugaauaguccuucaaaggguugcuagccuacguacgagugcgagcugcccagugaagggcuguuugucguucacucuaucacacaucuugcagaaaa  -3'  obs
    guuacauugaacuguugaauaguccuucaaaggguugcuagccuacguacgagugcgagcugcccagugaagggcuguuugucguucacucuaucacacaucuugcagaaaa       exp
    .......(((((...(((((((((((((..((((.(((.((((.(....))).)).))).))))..)))))))))))))..))))).........................  reads  mm  sample
    ....................aguccuucaaaggguugcu.........................................................................  15  0  EGG
    ....................aguccuucaaaggguugcua........................................................................  4  0  EGG
    ....................aguccuucaaaggguugcuag.......................................................................  120  0  EGG
    ....................aguccuucaaaggguugUuag.......................................................................  1  1  EGG
    ....................aguccuucaaaggguuAcuag.......................................................................  1  1  EGG
    ....................aguccuucaaaggguugcuaU.......................................................................  6  1  EGG
    ....................aguccuucaaaggguugcuagU......................................................................  36  1  EGG
    ....................aguccuucaaaggguugcuagc......................................................................  19  0  EGG
    ....................aguccuucaaaggguugcuagA......................................................................  2  1  EGG
    ....................aguccuucaaaggguugcuagcU.....................................................................  29  1  EGG
    ....................aguccuucaaaggguugcuagcc.....................................................................  11  0  EGG
    ....................aguccuucaaaggguugcuagAcu....................................................................  1  1  EGG
    ....................aguccuucaaaggguugcuagccA....................................................................  2  1  EGG
    ....................aguccuucaaaggguugcuagcUu....................................................................  3  1  EGG
    ....................aguccuucaaaggguugcuagccu....................................................................  7  0  EGG
    ....................aguccuucaaaggguugcuagccua...................................................................  6  0  EGG
    ....................aguccuucaaaggguugcuagccuacg.................................................................  5  0  EGG
    ......................uccuucaaaggguugcuag.......................................................................  1  0  EGG
    ......................uccuucaaaggguugcuagc......................................................................  1  0  EGG
    .........................uucaaaggguugcuagcU.....................................................................  1  1  EGG
    ....................................................gugcgagcugcccagugaagggcu....................................  1  0  EGG
    ......................................................cgagcugcccagugaagggA......................................  1  1  EGG
    ......................................................cgagcugcccagugaagggcu.....................................  1  0  EGG
    .......................................................Ugagcugcccagugaagggcug...................................  1  1  EGG
    ........................................................gagcugcccagugaagAg......................................  1  1  EGG
    ........................................................gagcugcccagugaaggg......................................  2  0  EGG
    ........................................................gagcugcccagugaaggA......................................  32  1  EGG
    ........................................................gagcugcccagugaaggAc.....................................  1  1  EGG
    ........................................................gagcugcccagugaagggA.....................................  2  1  EGG
    ........................................................gagcugcccagugaagggc.....................................  3  0  EGG
    ........................................................gagcugcccagugaagggcu....................................  19  0  EGG
    ........................................................gagcugcccagugaagggcuU...................................  15  1  EGG
    ........................................................gagcugcccagugaaCggcug...................................  1  1  EGG
```

```
                    Star                                   Mature
guuacauugaacuguugaauaguccuucaaaggguugcuagccuacguacgagugcgagcugcccagugaagggcuguuugucguucacucuaucacacaucuugcagaaaa
........................................................gagcugcccagugaagggcuA...................................    6     1   EGG
........................................................gagcugccGagugaagggcug...................................    1     1   EGG
........................................................gagcugcccagugGagggcug...................................    1     1   EGG
........................................................gagcugcccagugaagggcug...................................    335   0   EGG
........................................................gagcugcccagCgaagggcug...................................    1     1   EGG
........................................................gGgcugcccagugaagggcug...................................    1     1   EGG
........................................................gagcugcccagGgaagggcug...................................    1     1   EGG
........................................................gagcugcccagugaagggcuUu..................................    1     1   EGG
........................................................gagcugcccagugUagggcugu..................................    1     1   EGG
........................................................gagcugcccagugaagggcugu..................................    152   0   EGG
........................................................gGgcugcccagugaagggcugu..................................    1     1   EGG
........................................................gagcuAcccagugaagggcugu..................................    1     1   EGG
........................................................gagcugcccagugaagggcugC..................................    1     1   EGG
........................................................gagcugcccagugaagggcuguu.................................    50    0   EGG
........................................................gagcugcccagugaagggcuguA.................................    2     1   EGG
.........................................................agcugcccagugaagggcugu..................................    1     0   EGG
............................................................ugcccagugaagggcugu..................................    1     0   EGG
.............................................................gcccagugaagggcuguu.................................    1     0   EGG

...................uaguccuucaaaggguugcuag.......................................................................    2     0   J17
...................uaguccuucaaaggguugcuagc......................................................................    9     0   J17
...................uaguccuucaaaggguugcuagcU.....................................................................    4     1   J17
....................aguccuucaaaggguugc..........................................................................    4     0   J17
....................aguccuucaaaggguugcu.........................................................................    34    0   J17
....................aguccuucaaaggguugcua........................................................................    61    0   J17
....................aguccuucaaaggguugcuU........................................................................    5     1   J17
....................aguccuucaaaggguugcuag.......................................................................    1389  0   J17
....................agucUuucaaaggguugcuag.......................................................................    2     1   J17
....................aguccuucaaaAgguugcuag.......................................................................    1     1   J17
....................aguccuucaaaggAuugcuag.......................................................................    2     1   J17
....................aguccuucaaaggguugUuag.......................................................................    2     1   J17
....................aguccuucUaaggguugcuag.......................................................................    1     1   J17
....................aCuccuucaaaggguugcuag.......................................................................    1     1   J17
....................aguccuucaaaggguugcuaU.......................................................................    38    1   J17
....................aguUcuucaaaggguugcuag.......................................................................    1     1   J17
....................aAuccuucaaaggguugcuag.......................................................................    1     1   J17
....................aguccuucaUaggguugcuag.......................................................................    1     1   J17
....................aguccuucaaagUguugcuagc......................................................................    2     1   J17
....................aguccuucGaaggguugcuagc......................................................................    1     1   J17
....................aguccuucaaaggguugcuagU......................................................................    412   1   J17
....................aguccuucaaaggguuUcuagc......................................................................    2     1   J17
....................Gguccuucaaaggguugcuagc......................................................................    2     1   J17
....................aguccuucUaaggguugcuagc......................................................................    2     1   J17
....................aguccuucaaaggguugGuagc......................................................................    2     1   J17
....................aguccuucaaagggAugcuagc......................................................................    3     1   J17
....................aguccuucaaaggAuugcuagc......................................................................    1     1   J17
....................agAccuucaaaggguugcuagc......................................................................    3     1   J17
....................aguccuucaaaggguugcuGgc......................................................................    1     1   J17
....................aguccuucaCaggguugcuagc......................................................................    2     1   J17
....................aguccuucaaaggguuAcuagc......................................................................    2     1   J17
....................aguccuucaaagAguugcuagc......................................................................    2     1   J17
....................aguccuucaaagggCugcuagc......................................................................    2     1   J17
....................aguccuucaaaAgguugcuagc......................................................................    2     1   J17
....................aguccuucaaaggguugcuagc......................................................................    7247  0   J17
....................aguccuucaaaggguGgcuagc......................................................................    1     1   J17
....................aguccuucaaaggguAgcuagc......................................................................    1     1   J17
....................aguccuucaaaggguugcuagA......................................................................    52    1   J17
....................aguccuucaaUggguugcuagc......................................................................    2     1   J17
....................agucUuucaaaggguugcuagc......................................................................    4     1   J17
....................aguccuucaaaggguugcuaUc......................................................................    5     1   J17
....................aguccuucaaaUgguugcuagc......................................................................    2     1   J17
....................aguccuucaaaggguugcuaCc......................................................................    3     1   J17
....................aguccuAcaaaggguugcuagc......................................................................    3     1   J17
....................aguccuucaaaggguugcuUgc......................................................................    1     1   J17
....................aguccuucaaGgguugcuagc.......................................................................    1     1   J17
....................aguccuucaaaggguugUuagc......................................................................    1     1   J17
....................aguccCucaaaggguugcuagc......................................................................    3     1   J17
....................aguccuucaaagCguugcuagc......................................................................    1     1   J17
....................aguccGucaaaggguugcuagc......................................................................    2     1   J17
....................aCuccuucaaaggguugcuagc......................................................................    2     1   J17
```

Star **Mature**

```
guuacauugaacuguugaauaguccuucaaaggguugcuagccuacguacgagugcgagcugcccagugaagggcuguuugucguucacucuaucacacaucuugcagaaaa
....................aguccuCcaaaggguugcuagc......................................................................  3     1   J17
....................aAuccuucaaaggguugcuagc......................................................................  6     1   J17
....................aguccuuUaaaggguugcuagc......................................................................  1     1   J17
....................aguccuucaaaggguugcuagG......................................................................  1     1   J17
....................aguUcuucaaaggguugcuagc......................................................................  1     1   J17
....................aguccuuAaaaggguugcuagc......................................................................  2     1   J17
....................aguccuucaaaggguugcCagc......................................................................  1     1   J17
....................aguccuucaaaggguugcuagcc.....................................................................  191   0   J17
....................aguccuucaaaggguugcuagcA.....................................................................  157   1   J17
....................aguccuucaaaggguugcuagUc.....................................................................  2     1   J17
....................aguccuucaaaggguugcuagcG.....................................................................  3     1   J17
....................aguccuucaaaggguugcuagcU.....................................................................  4218  1   J17
....................aguccuucaaaggguugcuagcGu....................................................................  1     1   J17
....................aguccuucaaGggguugcuagccu....................................................................  1     1   J17
....................aguccuucaaaggguugcuagccu....................................................................  46    0   J17
....................aguccuucaaaggguugcuagcUu....................................................................  273   1   J17
....................aguccuucaaaggguugcuagccA....................................................................  39    1   J17
....................aguccuucaaaggguugcuagcAu....................................................................  2     1   J17
....................aguccuucaaaggguugcuagcUua...................................................................  1     1   J17
....................aguccuucaaaggguugcuagccua...................................................................  6     0   J17
....................aguccuucaaaggguugcuagccuU...................................................................  2     1   J17
....................aguccuucaaaggguugcuagcAua...................................................................  3     1   J17
....................aguccuucaaaggguugcuagccuac..................................................................  1     0   J17
....................aguccuucaaaggguugcuagccuacg.................................................................  1     0   J17
....................aguccuucaaaggguugcuagccuacU.................................................................  1     1   J17
.....................guccuucaaaggguugcuag.......................................................................  3     0   J17
......................uccuucaaaggguugcuag.......................................................................  2     0   J17
......................uccuucaaaggguugcuagc......................................................................  94    0   J17
......................uUcuucaaaggguugcuagc......................................................................  1     1   J17
......................uccuucaaaggguugcuagU......................................................................  4     1   J17
......................uccuucaaaggguugcuagcU.....................................................................  63    1   J17
......................uccuucaaaggguugcuagcc.....................................................................  1     0   J17
......................uccuucaaaggguugcuagcA.....................................................................  2     1   J17
......................uccuucaaaggguugcuagcUu....................................................................  2     1   J17
......................uccuucaaaggguugcuagccu....................................................................  2     0   J17
.......................ccuucaaaggguugcuagc......................................................................  1     0   J17
.......................ccuucaaaggguugcuagccu....................................................................  1     0   J17
.......................ccuucaaaggguugcuagcUu....................................................................  1     1   J17
.........................uucaaaggguugcuagcU.....................................................................  1     1   J17
.........................uucaaaggguugcuagccuacg.................................................................  1     0   J17
.....................................................ugcgagcugcccagugaagg.......................................  1     0   J17
.....................................................ugcgagcugcccagugaagggcugu..................................  1     0   J17
......................................................gGgagcugcccagugaagggcugu..................................  2     1   J17
.......................................................cgagcugcccagugaagggcu....................................  3     0   J17
.......................................................cgagcugcccagugaagggcug...................................  6     0   J17
.......................................................cgagcugcccagugaagggcuU...................................  1     1   J17
.......................................................Ugagcugcccagugaagggcug...................................  3     1   J17
.......................................................Ugagcugcccagugaagggcugu..................................  8     1   J17
.......................................................Agagcugcccagugaagggcugu..................................  3     1   J17
.......................................................cgagcugcccagugaagggcugu..................................  4     0   J17
.......................................................Ugagcugcccagugaagggcuguu.................................  1     1   J17
........................................................gagcugcccagugaaggg......................................  10    0   J17
........................................................gagcugcccagugaaggA......................................  28    1   J17
........................................................gaUcugcccagugaaggg......................................  1     1   J17
........................................................gagcugcccagugaagggA.....................................  1     1   J17
........................................................gagcugcccagugaagggc.....................................  24    0   J17
........................................................gagcugcccagugaagggcu....................................  93    0   J17
........................................................gagcugcccagugaGgggcu....................................  1     1   J17
........................................................gagcugcccagugaagggUu....................................  2     1   J17
........................................................gagcugcccagugaagggAu....................................  2     1   J17
........................................................gagcugcccagugaaggCcu....................................  1     1   J17
........................................................gCgcugcccagugaagggcug...................................  3     1   J17
........................................................gagcugcccagugaagggcug...................................  3989  0   J17
........................................................gagcugcccUgugaagggcug...................................  1     1   J17
........................................................gagcugccGagugaagggcug...................................  1     1   J17
........................................................gagcugcccaguUaagggcug...................................  3     1   J17
........................................................gUgcugcccagugaagggcug...................................  6     1   J17
........................................................gagcugcccagugaUgggcug...................................  9     1   J17
........................................................gagcugccAagugaagggcug...................................  1     1   J17
........................................................gagcugcccagugaaggCcug...................................  1     1   J17
```

Star **Mature**

guuacauugaacuguugaauaguccuucaaaggguugcuagccuacguacgagugcgagcugcccagugaagggcuguuugucguucacucuaucacacaucuugcagaaaa

....gagcuAcccagugaagggcug...................................	1	1	J17
....gagcugcccagugGagggcug...................................	3	1	J17
....gagcugcccagugaGggcug...................................	1	1	J17
....gagcugcccagugaaggAcug...................................	1	1	J17
....gagcugcccagAgaagggcug...................................	1	1	J17
....gagcugcccagugaagggcuU...................................	107	1	J17
....gGgcugcccagugaagggcug...................................	3	1	J17
....gagcugcccagugaaAggcug...................................	1	1	J17
....gagcugcccagugaaUggcug...................................	1	1	J17
....gagcugcGcagugaagggcug...................................	1	1	J17
....gagcugcccagGgaagggcug...................................	1	1	J17
....gagcugcAcagugaagggcug...................................	1	1	J17
....gagcugcccagugaagggcCg...................................	1	1	J17
....gagcugcccagugaaggUcug...................................	1	1	J17
....gagUugcccagugaagggcug...................................	3	1	J17
....gagcugcccagugaagggcAg...................................	1	1	J17
....gagcugcccagugaagggUug...................................	1	1	J17
....gagcugcccaUugaagggcug...................................	4	1	J17
....gagcugcccagugaagggcuA...................................	37	1	J17
....gagcugUccagugaagggcug...................................	1	1	J17
....gagcugcccagugaagUgcug...................................	2	1	J17
....Aagcugcccagugaagggcug...................................	4	1	J17
....gagcugcccagugaagggGug...................................	1	1	J17
....gagcugcccagugUagggcug...................................	3	1	J17
....gagcugcccCgugaagggcug...................................	1	1	J17
....gagcuAcccagugaagggcugu..................................	10	1	J17
....gagcugcccagugUagggcugu..................................	5	1	J17
....gGgcugcccagugaagggcugu..................................	15	1	J17
....gagcugcccagugaagggcuCu..................................	1	1	J17
....gagcugcccagugaaAggcugu..................................	2	1	J17
....gagcugcccagugaagggUugu..................................	7	1	J17
....gagAugcccagugaagggcugu..................................	2	1	J17
....gagcugcccagugaaggUcugu..................................	2	1	J17
....gagcugcccagugaCggcugu..................................	3	1	J17
....gagcugcccagugaagggcuUu..................................	29	1	J17
....gagcugcccagugaagggcGgu..................................	1	1	J17
....gCgcugcccagugaagggcugu..................................	4	1	J17
....gagcugcccagugaagCgcugu..................................	4	1	J17
....gagcugcccagugaUgggcugu..................................	20	1	J17
....gagcugcccagugaagggcuAu..................................	2	1	J17
....gagcugcccaAugaagggcugu..................................	2	1	J17
....gagcugcccUgugaagggcugu..................................	2	1	J17
....gagcugcccagugaagggcAgu..................................	2	1	J17
....gagcugcccaUugaagggcugu..................................	2	1	J17
....gaAcugcccagugaagggcugu..................................	3	1	J17
....gUgcugcccagugaagggcugu..................................	16	1	J17
....gagcugcccagugaagggcugC..................................	19	1	J17
....Uagcugcccagugaagggcugu..................................	1	1	J17
....gagcugcccagugaagggcugG..................................	9	1	J17
....gagcugcccagugaaCggcugu..................................	4	1	J17
....gagUugcccagugaagggcugu..................................	12	1	J17
....gagcuCcccagugaagggcugu..................................	1	1	J17
....gagcugcccagugGagggcugu..................................	5	1	J17
....gagcugccUagugaagggcugu..................................	7	1	J17
....gagcugcccagAgaagggcugu..................................	3	1	J17
....gagGugcccagugaagggcugu..................................	1	1	J17
....gagcugcccagugaaUggcugu..................................	3	1	J17
....gagcGgcccagugaagggcugu..................................	1	1	J17
....gagcugcccagugaagggAugu..................................	2	1	J17
....gagcCgcccagugaagggcugu..................................	2	1	J17
....gagcugcccagugaagggcugA..................................	99	1	J17
....gagcugcGcagugaagggcugu..................................	1	1	J17
....gagcugcccagugaagUgcugu..................................	2	1	J17
....gaUcugcccagugaagggcugu..................................	4	1	J17
....gagcugcccGgugaagggcugu..................................	1	1	J17
....gagcugcUcagugaagggcugu..................................	4	1	J17
....gagcugcccagugaagggcugu..................................	15484	0	J17
....gagcugAccagugaagggcugu..................................	1	1	J17
....gagcugcccagugaagAgcugu..................................	3	1	J17
....gagcugcccaCugaagggcugu..................................	1	1	J17

```
                    Star                                Mature
guuacauugaacuguugaauaguccuucaaaggguugcuagccuacguacgagugcgagcugcccagugaagggcuguuugucguucacucuaucacacaucuugcagaaaa
........................................................gagcugUccagugaagggcugu..................................  8     1  J17
........................................................gagcugccAagugaagggcugu..................................  4     1  J17
........................................................Aagcugcccagugaagggcugu..................................  7     1  J17
........................................................gagcugcccagugaaggAcugu..................................  10    1  J17
........................................................gagcuUcccagugaagggcugu..................................  2     1  J17
........................................................gagcugcccagugaGgggcugu..................................  3     1  J17
........................................................gGgcugcccagugaagggcuguu.................................  1     1  J17
........................................................gagcugcccagugaagggcugCu.................................  2     1  J17
........................................................gagcugcccagGgaagggcuguu.................................  1     1  J17
........................................................gagcugcccagAgaagggcuguu.................................  1     1  J17
........................................................gagcugcccagugaaAggcuguu.................................  1     1  J17
........................................................Aagcugcccagugaagggcuguu.................................  1     1  J17
........................................................gagcugcccagugaagggcugAu.................................  3     1  J17
........................................................gagcugcccagugaagggcuguu.................................  1196  0  J17
........................................................gagcugcccagugaagggcuguA.................................  190   1  J17
........................................................gagcugcccagugaaUggcuguu.................................  1     1  J17
........................................................gagcugcccCgugaagggcuguu.................................  1     1  J17
........................................................gagcugcccagugaaggUcuguu.................................  1     1  J17
........................................................gagcuUcccagugaagggcuguu.................................  1     1  J17
........................................................gagcugccUagugaagggcuguu.................................  1     1  J17
........................................................gUgcugcccagugaagggcuguu.................................  4     1  J17
........................................................gagcugcccagugaagggcuguC.................................  4     1  J17
........................................................gagcugcccagugaagggUuguu.................................  1     1  J17
........................................................gagcuAcccagugaagggcuguu.................................  1     1  J17
........................................................gagcugcccagugaUgggcuguu.................................  2     1  J17
........................................................gagcugcccagugaagggcuguAu................................  1     1  J17
........................................................gagcugcccagugaagggcuguuA................................  4     1  J17
........................................................gagcugcccagugaagggcuguuu................................  2     0  J17
........................................................gagcugcccagugaagggcuguuC................................  1     1  J17
........................................................gagcugcccagugaagggcuguuuC...............................  1     1  J17
........................................................gagcugcccagugaagggcuguuuU...............................  4     1  J17
.........................................................agcugcccagugaagggcugu..................................  5     0  J17
.........................................................agcugcccagugaagggcuguu.................................  1     0  J17
..........................................................gcugcccagugaagggcuguu.................................  1     0  J17
...........................................................cugcccagugaagggcugu..................................  2     0  J17
............................................................ugcccagugaagggcugu..................................  3     0  J17
.............................................................gcccagugaagggcuguC.................................  1     1  J17
.............................................................gcccagugaagggcuguu.................................  1     0  J17

...............ugaauaguccuucaaaggguugcu.........................................................................  1     0  HS4
...................uaguccuucaaaggguugcuag.......................................................................  3     0  HS4
...................uaguccuucaaaggguugcuagc......................................................................  35    0  HS4
...................uaguccuucaaaggguugcuagU......................................................................  1     1  HS4
...................uaguccuucaaaggguugcuagcU.....................................................................  1     1  HS4
....................aguccuucaaaggguugcu.........................................................................  19    0  HS4
....................aguccuucaaaggguugcua........................................................................  14    0  HS4
....................aguccuucaaaggguuCcuag.......................................................................  1     1  HS4
....................aguccuucaaUggguugcuag.......................................................................  1     1  HS4
....................agAccuucaaaggguugcuag.......................................................................  1     1  HS4
....................aguccuGcaaaggguugcuag.......................................................................  1     1  HS4
....................aguUcuucaaaggguugcuag.......................................................................  2     1  HS4
....................aguccuucaaaggguugUuag.......................................................................  1     1  HS4
....................aguccuucaaagggCugcuag.......................................................................  1     1  HS4
....................aguccuucaUaggguugcuag.......................................................................  1     1  HS4
....................aguccuucaaGggguugcuag.......................................................................  1     1  HS4
....................aguccuucaaaggCuugcuag.......................................................................  1     1  HS4
....................agucGuucaaaggguugcuag.......................................................................  1     1  HS4
....................aguccuucaaaggguGgcuag.......................................................................  1     1  HS4
....................aguccuAcaaaggguugcuag.......................................................................  1     1  HS4
....................aguccuucaaaggguugcuag.......................................................................  1451  0  HS4
....................aguccuucaaagCguugcuag.......................................................................  1     1  HS4
....................aguccuucaaaggguugcuaU.......................................................................  33    1  HS4
....................aguccuucaaaggguugcCagc......................................................................  3     1  HS4
....................aguccuucaaaggguAgcuagc......................................................................  1     1  HS4
....................aguccuucaaagUguugcuagc......................................................................  3     1  HS4
....................aguccuucaaaggguugcuaCc......................................................................  2     1  HS4
....................aguccuucaaaggguugcuagA......................................................................  53    1  HS4
....................aUuccuucaaaggguugcuagc......................................................................  3     1  HS4
....................aguccuucaaaggguuAcuagc......................................................................  1     1  HS4
....................aguccuucaaaUgguugcuagc......................................................................  2     1  HS4
```

```
                    Star                                  Mature
guuacauugaacuguugaauaguccuucaaaggguugcuagccuacguacgagugcgagcugcccagugaagggcuguuugucguucacucuaucacacaucuugcagaaaa
....................aguccCucaaaggguugcuagc......................................................................  2     1  HS4
....................aguccuucaaaggAuugcuagc......................................................................  3     1  HS4
....................aguccuucaaaggUuugcuagc......................................................................  4     1  HS4
....................agGccuucaaaggguugcuagc......................................................................  1     1  HS4
....................aguccuucaaaAgguugcuagc......................................................................  2     1  HS4
....................aguccuucaaaggCuugcuagc......................................................................  3     1  HS4
....................Uguccuucaaaggguugcuagc......................................................................  4     1  HS4
....................aguccuucaUaggguugcuagc......................................................................  2     1  HS4
....................aguccuucaaaggguGgcuagc......................................................................  1     1  HS4
....................aguccuucaaUggguugcuagc......................................................................  2     1  HS4
....................aguUcuucaaaggguugcuagc......................................................................  5     1  HS4
....................aguccuucaCaggguugcuagc......................................................................  1     1  HS4
....................aguccuucaaaggguugcuaUc......................................................................  1     1  HS4
....................aguccuucaaaggguugcuagc......................................................................  7742  0  HS4
....................agucGuucaaaggguugcuagc......................................................................  2     1  HS4
....................aguccuuUaaaggguugcuagc......................................................................  5     1  HS4
....................aguccuucaaGggguugcuagc......................................................................  1     1  HS4
....................aguccuucaaaggguCgcuagc......................................................................  4     1  HS4
....................aguccuGcaaaggguugcuagc......................................................................  6     1  HS4
....................aAuccuucaaaggguugcuagc......................................................................  1     1  HS4
....................agucAuucaaaggguugcuagc......................................................................  1     1  HS4
....................aguccuucaaagggGugcuagc......................................................................  2     1  HS4
....................aguccuucaaagggAugcuagc......................................................................  1     1  HS4
....................aguccuucaaagggCugcuagc......................................................................  1     1  HS4
....................aguccuucaaaggguugcuaAc......................................................................  1     1  HS4
....................agucUuucaaaggguugcuagc......................................................................  1     1  HS4
....................aguccuucaaaggguugcuagU......................................................................  710   1  HS4
....................aguccuuAaaaggguugcuagc......................................................................  1     1  HS4
....................aguccuCcaaaggguugcuagc......................................................................  2     1  HS4
....................aguccuucaaaggguugcuagG......................................................................  5     1  HS4
....................aguccuucaaaCgguugcuagc......................................................................  3     1  HS4
....................agCccuucaaaggguugcuagc......................................................................  1     1  HS4
....................aCuccuucaaaggguugcuagc......................................................................  6     1  HS4
....................aguccuucaaaggguugcuagcc.....................................................................  130   0  HS4
....................aguccuGcaaaggguugcuagcc.....................................................................  1     1  HS4
....................aguccuucaaaggguugcuagcG.....................................................................  1     1  HS4
....................agucGuucaaaggguugcuagcc.....................................................................  1     1  HS4
....................aguccuucaaaggguugcuagcA.....................................................................  107   1  HS4
....................aguccuucaaaggguugcuagcU.....................................................................  3285  1  HS4
....................aguccuucaaaggguugcuagcUu....................................................................  190   1  HS4
....................aguccuucaaaggguugcuagcAu....................................................................  2     1  HS4
....................aguccuucaaaggguugcuagccA....................................................................  14    1  HS4
....................aguccuucaaaggguugcuagccu....................................................................  35    0  HS4
....................aguccuucaaaggguugcuagccuU...................................................................  1     1  HS4
....................aguccuucaaaggguugcuagccua...................................................................  4     0  HS4
.....................guccuucaaaggguugcuagc......................................................................  1     0  HS4
.....................guccuucaaaggguugcuagcU.....................................................................  1     1  HS4
......................uccuucaaaggguugcuag.......................................................................  10    0  HS4
......................uccuucaaaggguGgcuagc......................................................................  1     1  HS4
......................uccuucaaaggguugcuagc......................................................................  73    0  HS4
......................uccuucaaaggguugcuagU......................................................................  9     1  HS4
......................uccuucaaaggguugcuagcU.....................................................................  80    1  HS4
......................uccuucaaaggguugcuagcUu....................................................................  2     1  HS4
.......................ccuucaaaggguugcuag.......................................................................  2     0  HS4
.......................ccuucaaaggguugcuagc......................................................................  6     0  HS4
.......................ccuucaaaggguugcuagcU.....................................................................  2     1  HS4
........................cuucaaaggguugcuagc......................................................................  1     0  HS4
.........................uucaaaggguugcuagcU.....................................................................  2     1  HS4
.........................uucaaaggguugcuagccuacg.................................................................  4     0  HS4
.....................................................gGgcgagcugcccagugaagggcugu..................................  1     1  HS4
.......................................................cgagcugcccagugaagggcu....................................  5     0  HS4
.......................................................Ugagcugcccagugaagggcug...................................  1     1  HS4
.......................................................cgagcugcccagugaagggcug...................................  2     0  HS4
.......................................................Ugagcugcccagugaagggcugu..................................  3     1  HS4
.......................................................cgagcugcccagugaagggcugu..................................  1     0  HS4
.......................................................cgagcugcccagugaagggcuguu.................................  1     0  HS4
........................................................gagcugcccagugaaggC......................................  1     1  HS4
........................................................gagcugcccagugaaggA......................................  252   1  HS4
........................................................gagcugcccagugaaggg......................................  7     0  HS4
........................................................gagcugcccagugaaggU......................................  1     1  HS4
```

Star Mature

```
guuacauugaacuguugaauaguccuucaaaggguugcuagccuacguacgagugcgagcugcccagugaagggcuguuugucguucacucuaucacacaucuugcagaaaa
........................................................gagcugUccagugaagggc.....................................     1     1     HS4
........................................................gagcugcccagugaagggc.....................................     21    0     HS4
........................................................gagcugcccagugaagggUu....................................     1     1     HS4
........................................................gagcugcccagugaagggcu....................................     48    0     HS4
........................................................gagcugcccagugaaUggcu....................................     1     1     HS4
........................................................gagcugcccagugaaggUcu....................................     1     1     HS4
........................................................gagcugcccagugaagUgcu....................................     1     1     HS4
........................................................gagcugcccagugaagggcA....................................     1     1     HS4
........................................................gagcugAccagugaagggcug...................................     2     1     HS4
........................................................gagcuUcccagugaagggcug...................................     1     1     HS4
........................................................gagcugcccagugaagggcug...................................     3186  0     HS4
........................................................gagcugcccagugCagggcug...................................     1     1     HS4
........................................................gagcugcccagugaGgggcug...................................     2     1     HS4
........................................................gagcugcccGgugaagggcug...................................     1     1     HS4
........................................................gUgcugcccagugaagggcug...................................     2     1     HS4
........................................................gCgcugcccagugaagggcug...................................     1     1     HS4
........................................................gagcugcccagugaaggUcug...................................     1     1     HS4
........................................................Cagcugcccagugaagggcug...................................     1     1     HS4
........................................................gagcugcccaguCaagggcug...................................     1     1     HS4
........................................................gagcugcccagugGagggcug...................................     1     1     HS4
........................................................gagcugcccagAgaagggcug...................................     1     1     HS4
........................................................gGgcugcccagugaagggcug...................................     1     1     HS4
........................................................gagcugcccagugaagggcuC...................................     1     1     HS4
........................................................gagcugcccagugaagggcuU...................................     79    1     HS4
........................................................gagcugcccagugaCgggcug...................................     1     1     HS4
........................................................gagcuAcccagugaagggcug...................................     1     1     HS4
........................................................gagcugcccaCugaagggcug...................................     2     1     HS4
........................................................gagcugcccagugaaggAcug...................................     5     1     HS4
........................................................gagcugcccagugaaCggcug...................................     1     1     HS4
........................................................gagcugcccagugaUgggcug...................................     1     1     HS4
........................................................gagcugcccaguUaagggcug...................................     4     1     HS4
........................................................gagcugcccagugUagggcug...................................     1     1     HS4
........................................................gaCcugcccagugaagggcug...................................     2     1     HS4
........................................................gagUugcccagugaagggcug...................................     8     1     HS4
........................................................gagcugcccagugaagggcuA...................................     24    1     HS4
........................................................gagcugcccagugaagggcCg...................................     2     1     HS4
........................................................gagcugUccagugaagggcug...................................     2     1     HS4
........................................................gagcugcccaUugaagggcug...................................     6     1     HS4
........................................................gagcugUccagugaagggcugu..................................     2     1     HS4
........................................................gagcugcccagugaagggcuUu..................................     7     1     HS4
........................................................gagcugccGagugaagggcugu..................................     1     1     HS4
........................................................gagcugcUcagugaagggcugu..................................     2     1     HS4
........................................................gagcugcccagugaGgggcugu..................................     1     1     HS4
........................................................gagcugcccagAgaagggcugu..................................     1     1     HS4
........................................................gagcugcccagugaaUggcugu..................................     4     1     HS4
........................................................gagcugcccagugaaggUcugu..................................     2     1     HS4
........................................................gagcugcccaguCaagggcugu..................................     2     1     HS4
........................................................gagcugcccaCugaagggcugu..................................     3     1     HS4
........................................................gagcugcccagugaagggcGgu..................................     1     1     HS4
........................................................gagcugcccagugaagggcugA..................................     8     1     HS4
........................................................gagcugcccagugaaCggcugu..................................     2     1     HS4
........................................................gGgcugcccagugaagggcugu..................................     5     1     HS4
........................................................gagcugcccagugaagggcuCu..................................     1     1     HS4
........................................................gagcugGccagugaagggcugu..................................     4     1     HS4
........................................................gagcugcccagugaagggGugu..................................     1     1     HS4
........................................................gagcugcccagugaagggcugC..................................     1     1     HS4
........................................................gagcugcccagugaCgggcugu..................................     1     1     HS4
........................................................gagcugcccagugaagUgcugu..................................     2     1     HS4
........................................................gUgcugcccagugaagggcugu..................................     2     1     HS4
........................................................gagUugcccagugaagggcugu..................................     7     1     HS4
........................................................gagcugcccagugCagggcugu..................................     1     1     HS4
........................................................gagcugcccagugaagggUugu..................................     4     1     HS4
........................................................gagcugcccaUugaagggcugu..................................     1     1     HS4
........................................................gagcugcccagugaagggcugu..................................     2780  0     HS4
........................................................gaCcugcccagugaagggcugu..................................     13    1     HS4
........................................................gagcugcccagugaagCgcugu..................................     5     1     HS4
........................................................gagcugcccaguUaagggcugu..................................     3     1     HS4
........................................................gagcugcccagugaagggcuAu..................................     2     1     HS4
........................................................gaUcugcccagugaagggcugu..................................     10    1     HS4
........................................................gagcugcccagugaagUgcuguu.................................     1     1     HS4
```

Star **Mature**

guuacauugaacuguugaauaguccuucaaaggguugcuagccuacguacgagugcgagcugcccagugaagggcuguuugucguucacucuaucacacaucuugcagaaaa			
....gagcugcccagugaaAggcuguu.................................	1	1	HS4
....gagcugcccagugaagggcuguC.................................	2	1	HS4
....gagcugcccagugaagggcuguA.................................	28	1	HS4
....gagcuAcccagugaagggcuguu.................................	1	1	HS4
....gagcugcccagugaaUggcuguu.................................	1	1	HS4
....gCgcugcccagugaagggcuguu.................................	1	1	HS4
....gagcugcccagugaagggcuguu.................................	210	0	HS4
....gagcugcccagugaagCgcuguu.................................	1	1	HS4
....cugcccagugaagggcuguu.................................	1	0	HS4
....................aguccuucaaaggguugcuagc..	1	0	HMA
....gagcugcccagugaaggA......................................	45	1	HMA
....gagcugcccagugaagggcu....................................	2	0	HMA
..................Uuaguccuucaaaggguugcuag...	1	1	HFA
...................uaguccuucaaaggguugcu...	2	0	HFA
...................uaguccuucaaaggguugcuag...	3	0	HFA
...................uaguccuucaaaggguugcuagU..	2	1	HFA
...................uaguccuucaaaggguugcuagA..	2	1	HFA
...................uaguccuucaaaggguugcuagc..	10	0	HFA
...................uaguccuucaaaggguugcuagcU...	4	1	HFA
....................aguccuucaaaggguugc..	5	0	HFA
....................aguccuCcaaaggguugcu...	1	1	HFA
....................aguccuucaaaggguugcu...	107	0	HFA
....................Gguccuucaaaggguugcu...	2	1	HFA
....................aAuccuucaaaggguugcu...	1	1	HFA
....................aguccuucaaaggguugcua..	54	0	HFA
....................aguccuucaaaggguugcuU..	14	1	HFA
....................aguccuucaaaggguuAcua..	1	1	HFA
....................aguccuucaaagCguugcuag...	1	1	HFA
....................aguccuucaaagggCugcuag...	1	1	HFA
....................aguccuucaaaggguugcuag...	1627	0	HFA
....................aguccuucaaaggguugcuUg...	1	1	HFA
....................agucUuucaaaggguugcuag...	2	1	HFA
....................aAuccuucaaaggguugcuag...	1	1	HFA
....................aguccuucaaaggguugcuaA...	1	1	HFA
....................Cguccuucaaaggguugcuag...	1	1	HFA
....................aguccuucaaaggguugcuaU...	50	1	HFA
....................aguccuucaaaggguuAcuag...	1	1	HFA
....................aguUcuucaaaggguugcuag...	1	1	HFA
....................aguccuucaaagggGugcuagc..	1	1	HFA
....................aguUcuucaaaggguugcuagc..	2	1	HFA
....................aUuccuucaaaggguugcuagc..	1	1	HFA
....................aguccuucaaaggguugUuagc..	1	1	HFA
....................aguccuucaaaggguugcuagU..	490	1	HFA
....................agucUuucaaaggguugcuagc..	1	1	HFA
....................aguccuucaaaggguugcuagc..	1839	0	HFA
....................aguccuucaaagCguugcuagc..	2	1	HFA
....................aguccuucUaaggguugcuagc..	1	1	HFA
....................aguccuucaaaggguuAcuagc..	1	1	HFA
....................aguccuucaCaggguugcuagc..	1	1	HFA
....................aguccuucaaaggguugcuaCc..	1	1	HFA
....................aguccuucaaaggguugcuagA..	54	1	HFA
....................aguccuucaaaggguugcuagcc...	112	0	HFA
....................aguccuucaaaggguugcuagcU...	1707	1	HFA
....................aguccuucaaaggguugcuagcG...	4	1	HFA
....................aguccuucaaaggguugcuagUc...	1	1	HFA
....................aguccuucaaaggguugcuagcA...	39	1	HFA
....................aguccuucaaaggguugcuagcUu..	79	1	HFA
....................aguccuucaaaggguugcuagccA..	10	1	HFA
....................aguccuucaaaggguugcuagccu..	41	0	HFA
....................Gguccuucaaaggguugcuagccua...	1	1	HFA
....................aguccuucaaaggguugcuagccua...	15	0	HFA
....................aguccuucaaaggguugcuagccuU...	2	1	HFA
....................aguccuucaaaggguugcuagccuac..	3	0	HFA
.....................guccuucaaaggguugcu...	1	0	HFA
......................uccuucaaaggguugcuag...	21	0	HFA
......................uccuucaaaggguugcuagU..	4	1	HFA
......................uccuucaaaggguugcuagc..	44	0	HFA
......................uccuucaaaggguugcuagA..	2	1	HFA

Star **Mature**

```
guuacauugaacuguugaauaguccuucaaaggguugcuagccuacguacgagugcgagcugcccagugaagggcuguuugucguucacucuaucacacaucuugcagaaaa
......................uccuucaaaggguugcuagcU.....................................................................  78    1  HFA
......................uccuucaaaggguugcuagcA.....................................................................  9     1  HFA
......................uccuucaaaggguugcuagcc.....................................................................  2     0  HFA
......................uccuucaaaggguugcuagcUu....................................................................  3     1  HFA
......................uccuucaaaggguugcuagccu....................................................................  1     0  HFA
.......................ccuucaaaggguugcuag.......................................................................  6     0  HFA
.......................ccuucaaaggguugcuagU......................................................................  2     1  HFA
.......................ccuucaaaggguugcuagc......................................................................  2     0  HFA
.......................ccuucaaaggguugcuagcU.....................................................................  2     1  HFA
........................cuucaaaggguugcuagU......................................................................  3     1  HFA
........................cuucaaaggguugcuagcU.....................................................................  3     1  HFA
........................cuucaaaggguugcuagccu....................................................................  2     0  HFA
.........................uucaaaggguugcuagcU.....................................................................  6     1  HFA
.........................uucaaaggguugcuagcc.....................................................................  2     0  HFA
.........................uucaaaggguugcuagcA.....................................................................  2     1  HFA
.........................uucaaaggguugcuagcUu....................................................................  1     1  HFA
.........................uucaaaggguugcuagccuac..................................................................  1     0  HFA
...................................................agugcgagcugcccagugaagggcug...................................  1     0  HFA
...................................................agugcgagcugcccagugaagggcugu..................................  1     0  HFA
....................................................gGgcgagcugcccagugaagggcu....................................  3     1  HFA
....................................................gugcgagcugcccagugaagggcugu..................................  1     0  HFA
.....................................................uUcgagcugcccagugaagggcug...................................  1     1  HFA
.....................................................Ggcgagcugcccagugaagggcug...................................  1     1  HFA
......................................................gGgagcugcccagugaaggg......................................  1     1  HFA
......................................................gcgagcugcccagugaagggcu....................................  1     0  HFA
......................................................gGgagcugcccagugaagggcu....................................  1     1  HFA
......................................................gGgagcugcccagugaagggcug...................................  1     1  HFA
......................................................gcgagcugcccagugaagggcug...................................  2     0  HFA
......................................................gcgagcCgcccagugaagggcugu..................................  1     1  HFA
......................................................gcgagcugcccagugaagggcugu..................................  5     0  HFA
......................................................gcgagcugcccagugaagggcuguu.................................  1     0  HFA
.......................................................cgagcugcccagugaagA.......................................  1     1  HFA
.......................................................Ugagcugcccagugaaggg......................................  1     1  HFA
.......................................................Ugagcugcccagugaagggc.....................................  1     1  HFA
.......................................................cgagcugcccagugaagggcu....................................  1     0  HFA
.......................................................Ugagcugcccagugaagggcu....................................  7     1  HFA
.......................................................Ugagcugcccagugaagggcug...................................  9     1  HFA
.......................................................cgagcugcccagugaagggcug...................................  1     0  HFA
.......................................................cgagcugcccagugaagggcugu..................................  1     0  HFA
.......................................................Ugagcugcccagugaagggcugu..................................  11    1  HFA
.......................................................Ugagcugcccagugaagggcuguu.................................  1     1  HFA
.......................................................Ggagcugcccagugaagggcuguu.................................  1     1  HFA
........................................................gagcugcccagugaCggg......................................  1     1  HFA
........................................................gagcugccUagugaaggg......................................  1     1  HFA
........................................................Uagcugcccagugaaggg......................................  2     1  HFA
........................................................gagcugcccagugaUggg......................................  1     1  HFA
........................................................gagcugcccagugaaAgg......................................  2     1  HFA
........................................................gCgcugcccagugaaggg......................................  3     1  HFA
........................................................gagcugcccagugaaggA......................................  733   1  HFA
........................................................gagcuUcccagugaaggg......................................  1     1  HFA
........................................................gGgcugcccagugaaggg......................................  1     1  HFA
........................................................gagcugccGagugaaggg......................................  1     1  HFA
........................................................gagcugcUcagugaaggg......................................  1     1  HFA
........................................................gagcugcccagugaaggg......................................  1185  0  HFA
........................................................gagcugcccagugaaggU......................................  6     1  HFA
........................................................gUgcugcccagugaaggg......................................  1     1  HFA
........................................................gagcugcccagugaaggC......................................  1     1  HFA
........................................................gagcugcccaUugaaggg......................................  2     1  HFA
........................................................gagcugcccagugaaggAc.....................................  1     1  HFA
........................................................gUgcugcccagugaagggc.....................................  3     1  HFA
........................................................Uagcugcccagugaagggc.....................................  2     1  HFA
........................................................gagUugcccagugaagggc.....................................  1     1  HFA
........................................................gagcugcccagugaagggc.....................................  614   0  HFA
........................................................gagcugcccagugaagggA.....................................  716   1  HFA
........................................................gagcugcccagugUagggc.....................................  1     1  HFA
........................................................gGgcugcccagugaagggc.....................................  1     1  HFA
........................................................gagcugcccagugaagggU.....................................  29    1  HFA
........................................................gagcugcccagugaagggG.....................................  1     1  HFA
........................................................gagcugcccagugaUgggcu....................................  5     1  HFA
........................................................gagcugcUcagugaagggcu....................................  3     1  HFA
```

```
                    Star                               Mature
guuacauugaacuguugaauaguccuucaaaggguugcuagccuacguacgagugcgagcugcccagugaagggcuguuugucguucacucuaucacacaucuugcagaaaa
........................................................gaAcugcccagugaagggcu....................................  3     1  HFA
........................................................gagcuCcccagugaagggcu....................................  3     1  HFA
........................................................gagcugcccaguCaagggcu....................................  2     1  HFA
........................................................gagcugcccaguAaagggcu....................................  1     1  HFA
........................................................gagcugcccagugaagggcu....................................  7507  0  HFA
........................................................gagcugcccagugaaggAcu....................................  2     1  HFA
........................................................gagcugcccagCgaagggcu....................................  1     1  HFA
........................................................gagcuUcccagugaagggcu....................................  3     1  HFA
........................................................gUgcugcccagugaagggcu....................................  7     1  HFA
........................................................gagcugUccagugaagggcu....................................  6     1  HFA
........................................................gagcugcccagugaCgggcu....................................  3     1  HFA
........................................................gagcugcccagugGagggcu....................................  3     1  HFA
........................................................gagcuAcccagugaagggcu....................................  9     1  HFA
........................................................gagcugcccagugUagggcu....................................  8     1  HFA
........................................................gagUugcccagugaagggcu....................................  8     1  HFA
........................................................gagcugcccagugaagggcA....................................  61    1  HFA
........................................................gCgcugcccagugaagggcu....................................  3     1  HFA
........................................................gagcugcccaUugaagggcu....................................  5     1  HFA
........................................................gagcGgcccagugaagggcu....................................  1     1  HFA
........................................................gagcugcccagugaaggUcu....................................  1     1  HFA
........................................................gGgcugcccagugaagggcu....................................  10    1  HFA
........................................................gagcugcccagugCagggcu....................................  2     1  HFA
........................................................Uagcugcccagugaagggcu....................................  2     1  HFA
........................................................gagcugcccagugaaAggcu....................................  2     1  HFA
........................................................gagcugcccagugaagggUu....................................  11    1  HFA
........................................................gaUcugcccagugaagggcu....................................  1     1  HFA
........................................................gagcugcccagugaagggcG....................................  5     1  HFA
........................................................gagcugcccagugaagggcC....................................  11    1  HFA
........................................................gagcCgcccagugaagggcu....................................  1     1  HFA
........................................................gagcugcccagugaGgggcu....................................  8     1  HFA
........................................................gagcugcccaAugaagggcu....................................  4     1  HFA
........................................................gagcugcccagugaagUgcu....................................  1     1  HFA
........................................................Cagcugcccagugaagggcu....................................  1     1  HFA
........................................................gagcugcccagugaaUggcu....................................  1     1  HFA
........................................................gagcugccUagugaagggcu....................................  7     1  HFA
........................................................gagcugcccaguUaagggcu....................................  5     1  HFA
........................................................gagcugcccagugaagggAu....................................  9     1  HFA
........................................................gagcugcccagugaagggcug...................................  5895  0  HFA
........................................................gagcugcccagugaagggUug...................................  8     1  HFA
........................................................gagcugcccagugaGgggcug...................................  6     1  HFA
........................................................gagcugcccaguUaagggcug...................................  1     1  HFA
........................................................gagUugcccagugaagggcug...................................  5     1  HFA
........................................................gCgcugcccagugaagggcug...................................  3     1  HFA
........................................................gagcugcccagugaagggcuA...................................  134   1  HFA
........................................................gagcugcccagugaagggcuU...................................  363   1  HFA
........................................................gagcugcccaCugaagggcug...................................  3     1  HFA
........................................................gagcuCcccagugaagggcug...................................  1     1  HFA
........................................................gagcugcccagugaCgggcug...................................  2     1  HFA
........................................................gagcugcccagugaaUggcug...................................  2     1  HFA
........................................................Uagcugcccagugaagggcug...................................  2     1  HFA
........................................................gagcugcccagugaagggcuC...................................  4     1  HFA
........................................................gagcugcccagugaagggcGg...................................  2     1  HFA
........................................................gagcugcUcagugaagggcug...................................  2     1  HFA
........................................................gagcugcccagugaaggCcug...................................  1     1  HFA
........................................................gagcugUccagugaagggcug...................................  1     1  HFA
........................................................gagcugcccaguCaagggcug...................................  1     1  HFA
........................................................gagcugcccagugaagggcCg...................................  3     1  HFA
........................................................gagcugcccagugCagggcug...................................  1     1  HFA
........................................................gagcugcccagugaagAgcug...................................  1     1  HFA
........................................................gagcugcccagugaUgggcug...................................  3     1  HFA
........................................................gagcuAcccagugaagggcug...................................  3     1  HFA
........................................................gagcugccUagugaagggcug...................................  2     1  HFA
........................................................gagcugcccagugaagCgcug...................................  1     1  HFA
........................................................gagcugcccagCgaagggcug...................................  5     1  HFA
........................................................gagcugcccagugaaggAcug...................................  4     1  HFA
........................................................gaCcugcccagugaagggcug...................................  1     1  HFA
........................................................gUgcugcccagugaagggcug...................................  10    1  HFA
........................................................gagcugcccagGgaagggcug...................................  1     1  HFA
........................................................gagcugcccagugGagggcug...................................  1     1  HFA
........................................................gagcugcccaUugaagggcug...................................  3     1  HFA
```

Star Mature

```
guuacauugaacuguugaauaguccuucaaaggguugcuagccuacguacgagugcgagcugcccagugaagggcuguuugucguucacucuaucacacaucuugcagaaaa
........................................................gagcugcccagugaagggAug...................................  1     1  HFA
........................................................gGgcugcccagugaagggcug...................................  7     1  HFA
........................................................gagcugcccagugUagggcug...................................  2     1  HFA
........................................................gagcugccUagugaagggcugu..................................  1     1  HFA
........................................................gUgcugcccagugaagggcugu..................................  9     1  HFA
........................................................gagcugcccagugaagUgcugu..................................  1     1  HFA
........................................................gagcugcccaCugaagggcugu..................................  1     1  HFA
........................................................gagcuAcccagugaagggcugu..................................  7     1  HFA
........................................................gagcugcccagugaagggcuCu..................................  2     1  HFA
........................................................gagcugcccaUugaagggcugu..................................  1     1  HFA
........................................................gagcugcccaguCaagggcugu..................................  2     1  HFA
........................................................gagcugcccagugaGgggcugu..................................  5     1  HFA
........................................................gagcugcccagugaagCgcugu..................................  1     1  HFA
........................................................gaCcugcccagugaagggcugu..................................  1     1  HFA
........................................................gagUugcccagugaagggcugu..................................  4     1  HFA
........................................................gagcugcccCgugaagggcugu..................................  1     1  HFA
........................................................gagcugcccagugaagggcugC..................................  21    1  HFA
........................................................gagcugcccagugaagggcugu..................................  4929  0  HFA
........................................................gagcugcccagugaaAggcugu..................................  1     1  HFA
........................................................gagcugcccagugUagggcugu..................................  3     1  HFA
........................................................gagcugcccagugCagggcugu..................................  1     1  HFA
........................................................gCgcugcccagugaagggcugu..................................  4     1  HFA
........................................................gaUcugcccagugaagggcugu..................................  3     1  HFA
........................................................gagcuUcccagugaagggcugu..................................  1     1  HFA
........................................................gagcugcccagugaagggcuAu..................................  14    1  HFA
........................................................Uagcugcccagugaagggcugu..................................  2     1  HFA
........................................................gagcugcccagugaUgggcugu..................................  2     1  HFA
........................................................gagcugcccagugaagggcugA..................................  29    1  HFA
........................................................gagcugUccagugaagggcugu..................................  3     1  HFA
........................................................gGgcugcccagugaagggcugu..................................  16    1  HFA
........................................................gagcugcccagugaagggcuUu..................................  33    1  HFA
........................................................gagcugcccagugGagggcugu..................................  1     1  HFA
........................................................gagcugcccagugaagggUugu..................................  8     1  HFA
........................................................gagcugcccagCgaagggcugu..................................  4     1  HFA
........................................................gagcugcccagugaaUggcugu..................................  3     1  HFA
........................................................gagcugcccagugaagggUuguu.................................  1     1  HFA
........................................................gagcugcccagugaagggcuguu.................................  742   0  HFA
........................................................gagcugcccagugaagggcuguC.................................  1     1  HFA
........................................................gagUugcccagugaagggcuguu.................................  2     1  HFA
........................................................gagcugccUagugaagggcuguu.................................  1     1  HFA
........................................................gagcugcccaAugaagggcuguu.................................  1     1  HFA
........................................................gGgcugcccagugaagggcuguu.................................  1     1  HFA
........................................................gagcugcccagugaagggcuguA.................................  66    1  HFA
........................................................gagcugcccagugaagggcuAuu.................................  1     1  HFA
........................................................gagcugcccagugaagUgcuguu.................................  1     1  HFA
........................................................gagcugcccagugaagggcuguuA................................  1     1  HFA
........................................................gagcugcccagugaagggcuguuuU...............................  2     1  HFA
.........................................................agcugcccagugaagggcu....................................  2     0  HFA
.........................................................agcugcccagugaagggcug...................................  3     0  HFA
.........................................................agcugcccagugaagggcugu..................................  4     0  HFA
.........................................................agcugcccagugaagggcuguu.................................  2     0  HFA
..........................................................gcugcccagugaagggcu....................................  2     0  HFA
..........................................................Ccugcccagugaagggcug...................................  1     1  HFA
..........................................................gcugcccagugaagggcugu..................................  4     0  HFA
...........................................................cugcccagugaagggcugu..................................  6     0  HFA
...........................................................cugcccagugaagggcuguu.................................  1     0  HFA
............................................................ugcccagugaagggcugu..................................  4     0  HFA
............................................................ugcccagugaagggcuguu.................................  3     0  HFA
.............................................................gcccagugaagggcuguu.................................  6     0  HFA

..................Uuaguccuucaaaggguugcuagc......................................................................  1     1  HS5
...................uaguccuucaaaggguugcuag.......................................................................  1     0  HS5
...................uaguccuucaaaggguugcuagc......................................................................  9     0  HS5
...................uaguccuucaaaggguugcuagA......................................................................  1     1  HS5
...................uaguccuucaaaggguugcuagcU.....................................................................  1     1  HS5
....................aguccuucaaaggguugcu.........................................................................  17    0  HS5
....................aguccuucaaaggguugcua........................................................................  16    0  HS5
....................aguccuucaaaggguugcuU........................................................................  2     1  HS5
....................aguccuucaaaggguCgcuag.......................................................................  2     1  HS5
....................aguccuucaaaggguugcuag.......................................................................  1165  0  HS5
```

Star Mature

```
guuacauugaacuguugaauaguccuucaaaggguugcuagccuacguacgagugcgagcugcccagugaagggcuguuugucguucacucuaucacacaucuugcagaaaa
....................aguccuucaaaggguugcuaU...........................................................................	28	1	HS5
....................aguccuucaaaggguuAcuag...........................................................................	2	1	HS5
....................aguccuucaaagAguugcuag...........................................................................	1	1	HS5
....................aguccuucaaaCgguugcuag...........................................................................	2	1	HS5
....................aguUcuucaaaggguugcuag...........................................................................	2	1	HS5
....................aguccuucaaaggguAgcuag...........................................................................	2	1	HS5
....................aguccuucaaaggguugcuaC...........................................................................	1	1	HS5
....................agCccuucaaaggguugcuag...........................................................................	1	1	HS5
....................aguccuucaaaggguGgcuag...........................................................................	1	1	HS5
....................Gguccuucaaaggguugcuagc..........................................................................	1	1	HS5
....................aguccuucaaaggguugcuagA..........................................................................	39	1	HS5
....................aguccuucaaaggguCgcuagc..........................................................................	4	1	HS5
....................aguccuucaaaggguugcCagc..........................................................................	2	1	HS5
....................aguccuucaaaggguugcuagU..........................................................................	469	1	HS5
....................aguccuucaaagCguugcuagc..........................................................................	9	1	HS5
....................aguccuucaaUggguugcuagc..........................................................................	1	1	HS5
....................aguccuucaaaggguAgcuagc..........................................................................	2	1	HS5
....................aguccuuUaaaggguugcuagc..........................................................................	1	1	HS5
....................aguccuucaaaggguugcuaAc..........................................................................	2	1	HS5
....................aguccuucaaGggguugcuagc..........................................................................	1	1	HS5
....................aguccuucaaaggguugcuGgc..........................................................................	1	1	HS5
....................aguccuucaCaggguugcuagc..........................................................................	1	1	HS5
....................aguccuucaaaggguugcuagG..........................................................................	2	1	HS5
....................aguccuucaaaggguuAcuagc..........................................................................	2	1	HS5
....................aguccuucaGaggguugcuagc..........................................................................	1	1	HS5
....................aguccuucaaaCgguugcuagc..........................................................................	1	1	HS5
....................aguccuucaaaggguGgcuagc..........................................................................	1	1	HS5
....................aguccuucaaaggguugcuagc..........................................................................	4396	0	HS5
....................aguccuucaaagUguugcuagc..........................................................................	2	1	HS5
....................aguccuucaaaggguugcuaCc..........................................................................	1	1	HS5
....................aguccuucaaaggUuugcuagc..........................................................................	1	1	HS5
....................aCuccuucaaaggguugcuagc..........................................................................	1	1	HS5
....................aguccuCcaaaggguugcuagc..........................................................................	2	1	HS5
....................aguAcuucaaaggguugcuagc..........................................................................	1	1	HS5
....................agucUuucaaaggguugcuagc..........................................................................	2	1	HS5
....................aguccuucaaagAguugcuagc..........................................................................	2	1	HS5
....................aguccuucaCaggguugcuagcc.........................................................................	1	1	HS5
....................aguccuucaaaggguugcuagcc.........................................................................	79	0	HS5
....................aguccuucaaaggguugcuagcA.........................................................................	102	1	HS5
....................aguccuucaaaggguugcuagcG.........................................................................	2	1	HS5
....................aguccuucaaaggguugcuagcU.........................................................................	2015	1	HS5
....................aguccuucaaaggguugcuaAcc.........................................................................	1	1	HS5
....................aguccuucaaaggguugcuagccu........................................................................	50	0	HS5
....................aguccuucaaaAgguugcuagccu........................................................................	1	1	HS5
....................aguccuucaaaggguugcuagcUu........................................................................	166	1	HS5
....................aguccuucaaaggguugcuagccA........................................................................	33	1	HS5
....................aguccuucaaaggguugcuagccuU.......................................................................	3	1	HS5
....................aguccuucaaaggguugcuagccua.......................................................................	9	0	HS5
....................aguccuucaaaggguugcuagcUua.......................................................................	1	1	HS5
.....................guccuucaaaggguugcuag...........................................................................	1	0	HS5
......................uccuucaaaggguugcuagA..........................................................................	1	1	HS5
......................uccuucaaaggguugcuagc..........................................................................	40	0	HS5
......................uccuucaaaggguugcuagU..........................................................................	4	1	HS5
......................uccuucaaaggguugcuagcA.........................................................................	7	1	HS5
......................uccuucaaaggguugcuagcU.........................................................................	25	1	HS5
......................uccuucaaaggguugcuagcUu........................................................................	2	1	HS5
.......................ccuucaaaggguugcuagc..........................................................................	1	0	HS5
.......................ccuucaaaggguugcuagcU.........................................................................	1	1	HS5
.......................................................cgagcugcccagugaagggcug...................................	1	0	HS5
.......................................................Ugagcugcccagugaagggcugu..................................	1	1	HS5
.......................................................cgagcugcccagugaagggcugu..................................	1	0	HS5
.......................................................cgagcugcccagugaagggcuguu.................................	1	0	HS5
........................................................gagcugcccagAgaaggg......................................	1	1	HS5
........................................................gagcugcccagugaaggA......................................	28	1	HS5
........................................................gagcugcccagugaagggc.....................................	11	0	HS5
........................................................gagcugcccagugaagggU.....................................	1	1	HS5
........................................................gagcugcccagugaaggUcu....................................	1	1	HS5
........................................................gagcugcccagugaaggAcu....................................	1	1	HS5
........................................................gagcugcccagugaagggcu....................................	14	0	HS5
........................................................gagcugcccagugaagggGu....................................	2	1	HS5
```

```
                    Star                                Mature
guuacauugaacuguugaauaguccuucaaaggguugcuagccuacguacgagugcgagcugcccagugaagggcuguuugucguucacucuaucacacaucuugcagaaaa
........................................................gGgcugcccagugaagggcu....................................    1     1   HS5
........................................................gagcugcccagugaaggUcug...................................    1     1   HS5
........................................................gagcugcccaguCaagggcug...................................    1     1   HS5
........................................................gagcugcccagugaagggcuU...................................    7     1   HS5
........................................................gagcugcccagugaagCgcug...................................    1     1   HS5
........................................................gagcugcccagugaagggcCg...................................    1     1   HS5
........................................................gagcugcccagugaagggcug...................................    282   0   HS5
........................................................gCgcugcccagugaagggcug...................................    1     1   HS5
........................................................gUgcugcccagugaagggcug...................................    3     1   HS5
........................................................gaCcugcccagugaagggcug...................................    1     1   HS5
........................................................gagUugcccagugaagggcug...................................    1     1   HS5
........................................................gagcugcccagugaaggAcug...................................    1     1   HS5
........................................................gagcugcccagugaagggcuA...................................    3     1   HS5
........................................................gagcuUcccagugaagggcug...................................    1     1   HS5
........................................................gaAcugcccagugaagggcug...................................    1     1   HS5
........................................................gCgcugcccagugaagggcugu..................................    3     1   HS5
........................................................gagcuUcccagugaagggcugu..................................    1     1   HS5
........................................................gagcugcccagugaaUggcugu..................................    1     1   HS5
........................................................gUgcugcccagugaagggcugu..................................    3     1   HS5
........................................................gagcugcccagugaGgggcugu..................................    2     1   HS5
........................................................gagcugcccaUugaagggcugu..................................    2     1   HS5
........................................................gagcugcccagugaagggcugA..................................    5     1   HS5
........................................................gagcugcccagAgaagggcugu..................................    1     1   HS5
........................................................gagcugcccagugaaAggcugu..................................    5     1   HS5
........................................................gagcugUccagugaagggcugu..................................    2     1   HS5
........................................................gaCcugcccagugaagggcugu..................................    5     1   HS5
........................................................gagcugcccagugaaggCcugu..................................    1     1   HS5
........................................................gagcugcccagugaagggcuAu..................................    3     1   HS5
........................................................gagUugcccagugaagggcugu..................................    4     1   HS5
........................................................gagcugcccagugaagggUugu..................................    4     1   HS5
........................................................gagcugcccagugaagUgcugu..................................    2     1   HS5
........................................................gGgcugcccagugaagggcugu..................................    4     1   HS5
........................................................gagcugcccagugaagggcugu..................................    1189  0   HS5
........................................................gaUcugcccagugaagggcugu..................................    1     1   HS5
........................................................gagcugcccagugaagggcugC..................................    2     1   HS5
........................................................gagcugcccagugaagggcuUu..................................    6     1   HS5
........................................................gagcuCcccagugaagggcugu..................................    2     1   HS5
........................................................gagcuAcccagugaagggcugu..................................    1     1   HS5
........................................................gUgcugcccagugaagggcuguu.................................    1     1   HS5
........................................................gGgcugcccagugaagggcuguu.................................    1     1   HS5
........................................................gagcugcccagugaagggcuguu.................................    111   0   HS5
........................................................gagcugccUagugaagggcuguu.................................    1     1   HS5
........................................................gagcugcccagugaagggcuguA.................................    9     1   HS5
........................................................gagcugcccagugaagggcuguuG................................    1     1   HS5
........................................................gagcugcccagugaagggcuguuuU...............................    1     1   HS5
..........................................................gcugcccagugaagggcugu..................................    1     0   HS5
...........................................................cugcccagugaagggcugu..................................    1     0   HS5

..................Uuaguccuucaaaggguugcu.........................................................................    1     1   HS7
..................Uuaguccuucaaaggguugcuagc......................................................................    2     1   HS7
...................uaguccuucaaaggguugcuagc......................................................................    17    0   HS7
...................uaguccuucaaaggguugcuagU......................................................................    1     1   HS7
...................uaguccuucaaaggguugcuagA......................................................................    2     1   HS7
...................uaguccuucaaaggguugcuagcU.....................................................................    2     1   HS7
....................aguccuucaaaggguugcu.........................................................................    24    0   HS7
....................aguccuucaaaggguugcuU........................................................................    1     1   HS7
....................aguccuucaaaggguugcua........................................................................    25    0   HS7
....................agucAuucaaaggguugcua........................................................................    1     1   HS7
....................aUuccuucaaaggguugcuag.......................................................................    1     1   HS7
....................aguccuucaaaggguugcuag.......................................................................    1171  0   HS7
....................aguccuucaaaggguugcAag.......................................................................    3     1   HS7
....................aguccuucaaaggguugcuaU.......................................................................    22    1   HS7
....................aguccuuUaaaggguugcuag.......................................................................    1     1   HS7
....................aguccuucaaagggCugcuag.......................................................................    1     1   HS7
....................agucUuucaaaggguugcuag.......................................................................    2     1   HS7
....................aguccuucaaaggguugcCag.......................................................................    2     1   HS7
....................aguccuuGaaaggguugcuag.......................................................................    1     1   HS7
....................agucGuucaaaggguugcuagc......................................................................    1     1   HS7
....................aCuccuucaaaggguugcuagc......................................................................    1     1   HS7
....................aguccuucaaaggguugcuagc......................................................................    3309  0   HS7
```

Star　　Mature

```
guuacauugaacuguugaauaguccuucaaaggguugcuagccuacguacgagugcgagcugcccagugaagggcuguuugucguucacucuaucacacaucuugcagaaaa
....................aguccuucaaaggguugcuagU......................................................................  380   1  HS7
....................aguccuucaaaggguugcuagA......................................................................  31    1  HS7
....................Uguccuucaaaggguugcuagc......................................................................  1     1  HS7
....................agucUuucaaaggguugcuagc......................................................................  3     1  HS7
....................aguccCucaaaggguugcuagc......................................................................  2     1  HS7
....................aguccuCcaaaggguugcuagc......................................................................  1     1  HS7
....................aguccuucaaaggguugUuagc......................................................................  1     1  HS7
....................Gguccuucaaaggguugcuagc......................................................................  1     1  HS7
....................aguccuucaaaggguugcuaAc......................................................................  3     1  HS7
....................aguccuucaaCggguugcuagc......................................................................  2     1  HS7
....................aguccuucaaaggguugcuagG......................................................................  5     1  HS7
....................aguccuucaaaggguugcuagcc.....................................................................  104   0  HS7
....................aguccuucaaaggguugcuagcU.....................................................................  2241  1  HS7
....................aguccuucaaaggguugcuagcG.....................................................................  7     1  HS7
....................aguccuucaaaggguugcuagcA.....................................................................  56    1  HS7
....................aguccuucaaaggguugcGagccu....................................................................  1     1  HS7
....................aguccuucaaaggguugcuagccA....................................................................  19    1  HS7
....................aguccuucaaaggguugcuagcGu....................................................................  1     1  HS7
....................aguccuucaaaggguugcuagccu....................................................................  44    0  HS7
....................aguccuucaaaggguugcuagcUu....................................................................  148   1  HS7
....................aguccCucaaaggguugcuagccua...................................................................  1     1  HS7
....................aguccuucaaaggguugcuagccua...................................................................  3     0  HS7
....................aguccuucaaaggguugcuagccuaU..................................................................  1     1  HS7
....................aguccuucaaaggguugcuagccuacU.................................................................  2     1  HS7
......................uccuucaaaggguugcuag.......................................................................  12    0  HS7
......................uccuucaaaggCuugcuagc......................................................................  1     1  HS7
......................uccuucaaaAgguugcuagc......................................................................  1     1  HS7
......................uccuucaaaggguugcuagc......................................................................  80    0  HS7
......................uccuucaaaggguugcuagU......................................................................  6     1  HS7
......................uccuucaaaggguugcuagcA.....................................................................  1     1  HS7
......................uccuucaaaggguugcuagcU.....................................................................  91    1  HS7
......................uccuucaaaggguugcuagcc.....................................................................  2     0  HS7
......................uccuucaaaggguugcuagcAu....................................................................  1     1  HS7
......................uccuucaaaggguugcuagcUu....................................................................  1     1  HS7
.......................ccuucaaaggguugcuag.......................................................................  3     0  HS7
.......................ccuucaaaggguugcuagc......................................................................  3     0  HS7
.......................ccuucaaaggguugcuagU......................................................................  2     1  HS7
.......................ccuucaaaggguugcuagcc.....................................................................  1     0  HS7
.......................ccuucaaaggguugcuagcU.....................................................................  2     1  HS7
.......................ccuucaaaggguugcuagcUu....................................................................  2     1  HS7
........................cuucaaaggguugcuagc......................................................................  1     0  HS7
.........................uucaaaggguugcuagcU.....................................................................  2     1  HS7
.........................uucaaaggguugcuagcc.....................................................................  1     0  HS7
...............................................uacgagugcgagcugcccagu............................................  1     0  HS7
.......................................................Ugagcugcccagugaagg.......................................  1     1  HS7
.......................................................cgagcugcccagugaaggA......................................  1     1  HS7
.......................................................cgagcugcccagugaaAgg......................................  1     1  HS7
.......................................................cgagcugcccagugaagggU.....................................  1     1  HS7
.......................................................cgagcugcccagugaagggA.....................................  1     1  HS7
.......................................................cgagcugcccagugaagggcu....................................  6     0  HS7
.......................................................Ugagcugcccagugaagggcu....................................  2     1  HS7
.......................................................cgagcugcccagugaagggcug...................................  11    0  HS7
.......................................................Ggagcugcccagugaagggcug...................................  2     1  HS7
.......................................................Ugagcugcccagugaagggcug...................................  16    1  HS7
.......................................................cgagcugcccagugaagggcugu..................................  2     0  HS7
.......................................................Ggagcugcccagugaagggcugu..................................  1     1  HS7
.......................................................Ugagcugcccagugaagggcugu..................................  14    1  HS7
.......................................................cgagcugcccagugaagggcuguu.................................  1     0  HS7
.......................................................Ugagcugcccagugaagggcuguu.................................  5     1  HS7
........................................................gagcugcccagugaaggg......................................  56    0  HS7
........................................................gagcugcccagugaaggA......................................  933   1  HS7
........................................................Cagcugcccagugaaggg......................................  1     1  HS7
........................................................gagcugcccagugaaggU......................................  4     1  HS7
........................................................gagcugcccagugaaggC......................................  1     1  HS7
........................................................gagcugcccagugaaAgg......................................  7     1  HS7
........................................................Uagcugcccagugaagggc.....................................  1     1  HS7
........................................................gagcugcccagugaagggU.....................................  1     1  HS7
........................................................gagcugcccagugaaggCc.....................................  1     1  HS7
........................................................gagcugccUagugaagggc.....................................  1     1  HS7
........................................................gagcugcccagugaagggA.....................................  26    1  HS7
```

```
                 Star                                   Mature
guuacauugaacuguugaauaguccuucaaaggguugcuagccuacguacgagugcgagcugcccagugaagggcuguuugucguucacucuaucacacaucuugcagaaaa
........................................................gagcugcccagugaagggc.....................................  111   0  HS7
........................................................gaCcugcccagugaagggcu....................................  1     1  HS7
........................................................gagcugcccagugaagggAu....................................  1     1  HS7
........................................................gUgcugcccagugaagggcu....................................  1     1  HS7
........................................................gagcuCcccagugaagggcu....................................  1     1  HS7
........................................................gagcugcccagugaagggcG....................................  1     1  HS7
........................................................gagcugcUcagugaagggcu....................................  1     1  HS7
........................................................gagcugcccagugaagggcu....................................  597   0  HS7
........................................................gagcugcccagugaCgggcu....................................  1     1  HS7
........................................................gagcugcccagugaagggUu....................................  1     1  HS7
........................................................gGgcugcccagugaagggcu....................................  2     1  HS7
........................................................gagcugcccagugaagggcug...................................  6664  0  HS7
........................................................gagcugcAcagugaagggcug...................................  1     1  HS7
........................................................gagcugcccagugaaUggcug...................................  1     1  HS7
........................................................gagcuAcccagugaagggcug...................................  3     1  HS7
........................................................gagcugcccaUugaagggcug...................................  2     1  HS7
........................................................gagcugcccagugaGgggcug...................................  1     1  HS7
........................................................gaCcugcccagugaagggcug...................................  7     1  HS7
........................................................gagcugcccagugGagggcug...................................  2     1  HS7
........................................................gagcugcccaguUaagggcug...................................  2     1  HS7
........................................................gagcAgcccagugaagggcug...................................  1     1  HS7
........................................................gagcugcccagugaaggAcug...................................  5     1  HS7
........................................................gagcugcccagugaaggUcug...................................  3     1  HS7
........................................................gagcugcccaguCaagggcug...................................  1     1  HS7
........................................................gagcugcccaAugaagggcug...................................  1     1  HS7
........................................................gagcugcccagugaagUgcug...................................  1     1  HS7
........................................................gagcugcccagugaagggcuU...................................  105   1  HS7
........................................................gagUugcccagugaagggcug...................................  8     1  HS7
........................................................gagcugcccagugaagggcCg...................................  8     1  HS7
........................................................gagcugcccagugaagCgcug...................................  2     1  HS7
........................................................gagcugcccagugaagggUug...................................  3     1  HS7
........................................................gUgcugcccagugaagggcug...................................  6     1  HS7
........................................................gagcugcccagugaagggcAg...................................  1     1  HS7
........................................................gGgcugcccagugaagggcug...................................  1     1  HS7
........................................................Aagcugcccagugaagggcug...................................  1     1  HS7
........................................................gagcugUccagugaagggcug...................................  5     1  HS7
........................................................gagcugcGcagugaagggcug...................................  1     1  HS7
........................................................gagcugcccagugaaAggcug...................................  1     1  HS7
........................................................gagcugcccagugaagggcuA...................................  66    1  HS7
........................................................gaUcugcccagugaagggcug...................................  1     1  HS7
........................................................gagcugcccaCugaagggcug...................................  2     1  HS7
........................................................gagcugcccagugaagggcGg...................................  53    1  HS7
........................................................gagcugcccagugaagggcuUu..................................  3     1  HS7
........................................................gGgcugcccagugaagggcugu..................................  1     1  HS7
........................................................gagcugcccagugaagggcugA..................................  15    1  HS7
........................................................gagcugcGcagugaagggcugu..................................  1     1  HS7
........................................................gagcugcccagugaagCgcugu..................................  3     1  HS7
........................................................gagUugcccagugaagggcugu..................................  2     1  HS7
........................................................gagcugUccagugaagggcugu..................................  3     1  HS7
........................................................gagcuCcccagugaagggcugu..................................  1     1  HS7
........................................................gagcugcccagugaGgggcugu..................................  4     1  HS7
........................................................gagcCgcccagugaagggcugu..................................  1     1  HS7
........................................................gagcugcccaguUaagggcugu..................................  1     1  HS7
........................................................gagcugcUcagugaagggcugu..................................  3     1  HS7
........................................................gagcugcccagugUagggcugu..................................  1     1  HS7
........................................................Aagcugcccagugaagggcugu..................................  1     1  HS7
........................................................gagcugcccaguCaagggcugu..................................  3     1  HS7
........................................................gagcugcccagugaagggcugG..................................  6     1  HS7
........................................................gagcugccUagugaagggcugu..................................  2     1  HS7
........................................................gagcugcccagugaaggUcugu..................................  1     1  HS7
........................................................gagcAgcccagugaagggcugu..................................  3     1  HS7
........................................................gagcugcccagugaagggcuAu..................................  5     1  HS7
........................................................gagcugcccagugaUgggcugu..................................  1     1  HS7
........................................................Uagcugcccagugaagggcugu..................................  3     1  HS7
........................................................gaUcugcccagugaagggcugu..................................  1     1  HS7
........................................................gagcugcccagugaagggcugu..................................  4653  0  HS7
........................................................gagcugcccagugCagggcugu..................................  1     1  HS7
........................................................gUgcugcccagugaagggcugu..................................  3     1  HS7
........................................................gagcugcccagugaaUggcugu..................................  2     1  HS7
........................................................gaAcugcccagugaagggcugu..................................  2     1  HS7
```

Star Mature

```
guuacauugaacuguugaauaguccuucaaaggguugcuagccuacguacgagugcgagcugcccagugaagggcuguuugucguucacucuaucacacaucuugcagaaaa
........................................................gagcugcccaUugaagggcugu..................................   1     1   HS7
........................................................gagcuAcccagugaagggcugu..................................   4     1   HS7
........................................................gCgcugcccagugaagggcugu..................................   1     1   HS7
........................................................gagcugcccagugaagUgcugu..................................   2     1   HS7
........................................................gagcugcccagugaagggUugu..................................   6     1   HS7
........................................................gagcugcccagugaagggcugC..................................   9     1   HS7
........................................................gagcugcccagugaagggcuguu.................................   645   0   HS7
........................................................gagcugcccagugaagggcuguA.................................   43    1   HS7
........................................................gagcugcccagugaagggcuguC.................................   2     1   HS7
........................................................gagcugcccagugaagggUuguu.................................   1     1   HS7
........................................................gagcugccUagugaagggcuguu.................................   1     1   HS7
........................................................gagcugcccaUugaagggcuguu.................................   3     1   HS7
........................................................gagUugcccagugaagggcuguu.................................   1     1   HS7
........................................................gagcugUccagugaagggcuguu.................................   2     1   HS7
........................................................gagcugcccagugaagggcuUuu.................................   1     1   HS7
........................................................gagcugcccagugaagggcuguuA................................   2     1   HS7
........................................................gagcugcccagugaagggcuguuG................................   1     1   HS7
........................................................gagcugcccagugaagggcuguuuA...............................   1     1   HS7
........................................................gagcugcccagugaagggcuguuuU...............................   1     1   HS7
.........................................................agcugcccagugaagggcu....................................   2     0   HS7
.........................................................agcugcccagugaagggcug...................................   1     0   HS7
...........................................................cugcccagugaagggcug...................................   1     0   HS7
...........................................................cugcccagugaagggcugu..................................   2     0   HS7
...........................................................cugcccagugaagggcuguu.................................   4     0   HS7
............................................................uUcccagugaagggcugu..................................   1     1   HS7
............................................................ugcccagugaagggcugu..................................   8     0   HS7
............................................................ugcccagugaagggcuguu.................................   1     0   HS7
.............................................................gcccagugaagggcuguu.................................   1     0   HS7

guuacauugaacuguugaaua...........................................................................................   5     0   HS1
....................aguccuucaaaggguugcuagc......................................................................   15    0   HS1
.....................guccuucaaaggguugcuagc......................................................................   13    0   HS1
........................................................gagcugcccagugaaggA......................................   5     1   HS1
........................................................gagcugcccagugaaggg......................................   8     0   HS1
........................................................gagcugcccagugaagggcu....................................   7     0   HS1
........................................................gagcugcccagugaagggcug...................................   16    0   HS1
........................................................gagcugcccagugaagggcugu..................................   12    0   HS1

........................................................gagcugcccagugaagggcu....................................   47    0   HS2

..................Uuaguccuucaaaggguugcuagc......................................................................   2     1   HA1
...................uaguccuucaaaggguugcu.........................................................................   2     0   HA1
...................uaguccuucaaaggguugcuagc......................................................................   32    0   HA1
...................Gaguccuucaaaggguugcuagc......................................................................   1     1   HA1
...................uaguccuucaaaggguugcuagcU.....................................................................   4     1   HA1
....................aguccuucaaaggguugcC.........................................................................   1     1   HA1
....................aguccuucaaaggguugcu.........................................................................   64    0   HA1
....................Gguccuucaaaggguugcua........................................................................   1     1   HA1
....................aguccuucaaaggguugcuU........................................................................   9     1   HA1
....................aguccuucaaaggguugcua........................................................................   207   0   HA1
....................aguccuucaaagAguugcuag.......................................................................   1     1   HA1
....................aguccuucaaaCgguugcuag.......................................................................   1     1   HA1
....................aguccuucaaCggguugcuag.......................................................................   1     1   HA1
....................aguccuucaaaggguugcuaU.......................................................................   74    1   HA1
....................aguAcuucaaaggguugcuag.......................................................................   3     1   HA1
....................Cguccuucaaaggguugcuag.......................................................................   1     1   HA1
....................aguccuucaaaggguugcuCg.......................................................................   1     1   HA1
....................agucGuucaaaggguugcuag.......................................................................   1     1   HA1
....................aguUcuucaaaggguugcuag.......................................................................   2     1   HA1
....................aguccCucaaaggguugcuag.......................................................................   2     1   HA1
....................aguccuucaaagggAugcuag.......................................................................   2     1   HA1
....................aguccuucaaaggguugUuag.......................................................................   1     1   HA1
....................aCuccuucaaaggguugcuag.......................................................................   1     1   HA1
....................aguccuucaaagCguugcuag.......................................................................   1     1   HA1
....................aguccuucaaaggguugcuaA.......................................................................   1     1   HA1
....................aguccuucaaaggguuAcuag.......................................................................   1     1   HA1
....................aguccuucaaaUgguugcuag.......................................................................   2     1   HA1
....................aguccuucaaaggguugcuag.......................................................................   3212  0   HA1
....................agucUuucaaaggguugcuag.......................................................................   3     1   HA1
....................aguccuucaaaggguAgcuag.......................................................................   1     1   HA1
```

Star **Mature**

```
guuacauugaacuguugaauaguccuucaaaggguugcuagccuacguacgagugcgagcugcccagugaagggcuguuugucguucacucuaucacacaucuugcagaaaa
....................aguccuucaaagUguugcuag.......................................................................  2     1  HA1
....................aguccuuAaaaggguugcuag.......................................................................  1     1  HA1
....................Gguccuucaaaggguugcuag.......................................................................  1     1  HA1
....................aguccuucaaaggguugcAag.......................................................................  3     1  HA1
....................aguccuucGaaggguugcuag.......................................................................  1     1  HA1
....................aguccuucaaaggguugcuUg.......................................................................  1     1  HA1
....................aguccuucaaaAgguugcuag.......................................................................  1     1  HA1
....................aguccuucaaaggAuugcuag.......................................................................  1     1  HA1
....................aguccuucaaGggguugcuag.......................................................................  1     1  HA1
....................Uguccuucaaaggguugcuag.......................................................................  2     1  HA1
....................aguccuucaaaggguugcCag.......................................................................  3     1  HA1
....................aguccuucaaaggguugUuagc......................................................................  4     1  HA1
....................aguccuucaaaggguugcuagU......................................................................  534   1  HA1
....................agCccuucaaaggguugcuagc......................................................................  2     1  HA1
....................aUuccuucaaaggguugcuagc......................................................................  3     1  HA1
....................aguccuucaaaggguugcuagG......................................................................  2     1  HA1
....................agucUuucaaaggguugcuagc......................................................................  1     1  HA1
....................aguccuucaaaggguugcuagc......................................................................  9443  0  HA1
....................agGccuucaaaggguugcuagc......................................................................  2     1  HA1
....................aguccuucaaaggguugcuGgc......................................................................  2     1  HA1
....................aguccuucaUaggguugcuagc......................................................................  2     1  HA1
....................aguccuucaaaggguCgcuagc......................................................................  3     1  HA1
....................aguccuucaaagCguugcuagc......................................................................  1     1  HA1
....................aCuccuucaaaggguugcuagc......................................................................  4     1  HA1
....................aguccuAcaaaggguugcuagc......................................................................  1     1  HA1
....................aguccuucaaagAguugcuagc......................................................................  1     1  HA1
....................aguccuucaaaggguugcAagc......................................................................  2     1  HA1
....................aguccuucGaaggguugcuagc......................................................................  1     1  HA1
....................aguccuucaaaAgguugcuagc......................................................................  1     1  HA1
....................aguccuucaaagUguugcuagc......................................................................  3     1  HA1
....................Cguccuucaaaggguugcuagc......................................................................  2     1  HA1
....................aguccuucaaaggguGgcuagc......................................................................  1     1  HA1
....................aguccuucaaaggguugcuagA......................................................................  136   1  HA1
....................aguccuucaaagggCugcuagc......................................................................  2     1  HA1
....................aguccuuGaaaggguugcuagc......................................................................  1     1  HA1
....................aAuccuucaaaggguugcuagc......................................................................  2     1  HA1
....................aguccuuUaaaggguugcuagc......................................................................  1     1  HA1
....................aguccuGcaaaggguugcuagc......................................................................  5     1  HA1
....................aguccuucUaaggguugcuagc......................................................................  4     1  HA1
....................aguccuucaaagggAugcuagc......................................................................  2     1  HA1
....................agucAuucaaaggguugcuagc......................................................................  1     1  HA1
....................aguccuucaaaggguugcuaAc......................................................................  8     1  HA1
....................aguccCucaaaggguugcuagc......................................................................  4     1  HA1
....................aguccuucaaaggguugcCagc......................................................................  2     1  HA1
....................Gguccuucaaaggguugcuagc......................................................................  7     1  HA1
....................aguccuucaaaggguuAcuagc......................................................................  2     1  HA1
....................aguccuCcaaaggguugcuagc......................................................................  7     1  HA1
....................Uguccuucaaaggguugcuagc......................................................................  2     1  HA1
....................aguGcuucaaaggguugcuagc......................................................................  1     1  HA1
....................agucGuucaaaggguugcuagc......................................................................  2     1  HA1
....................agucUuucaaaggguugcuagcc.....................................................................  1     1  HA1
....................aguccuucaaaggguugcuagcU.....................................................................  4016  1  HA1
....................aguccuucaaaggguugcuagcc.....................................................................  77    0  HA1
....................aguccuucaaaggguugcuagcA.....................................................................  279   1  HA1
....................aguccuucaaaggguugcuagcG.....................................................................  9     1  HA1
....................aguccuucaaaggguugcuagAcu....................................................................  1     1  HA1
....................aguccuucaaaggguugcuagccA....................................................................  16    1  HA1
....................aguccuucaaaggguugcuagcAu....................................................................  5     1  HA1
....................aguccuucaaaggguugcuagccu....................................................................  40    0  HA1
....................aguccuucaaaggguugcuagcUu....................................................................  193   1  HA1
....................aguccuucaaaggguugcuagccua...................................................................  15    0  HA1
....................aguccuucaaaggguugcuagcGua...................................................................  1     1  HA1
....................aguccuucaaaggguugcuagcUua...................................................................  3     1  HA1
....................aguccuucaaaggguugcuagcAua...................................................................  1     1  HA1
....................aguccuucaaaggguugcuagccuac..................................................................  1     0  HA1
....................aguccuucaaaggguugcuagccuacg.................................................................  5     0  HA1
.....................guccuucaaaggguugcuag.......................................................................  1     0  HA1
.....................guccuucaaaggguugcuagcc.....................................................................  1     0  HA1
.....................guccuucaaaggguugcuagcU.....................................................................  1     1  HA1
......................uccuucaaaggguugcuag.......................................................................  4     0  HA1
```

Star Mature

guuacauugaacuguugaauaguccuucaaaggguugcuagccuacguacgagugcgagcugcccagugaagggcuguuugucguucacucuaucacacaucuugcagaaaa

......................ucUuucaaaggguugcuagc..	1	1	HA1
......................uccuucaaaggguugcuagU..	3	1	HA1
......................uccuucaaaggguugcuagA..	1	1	HA1
......................uccuucaaaggguugcuagc..	62	0	HA1
......................uccuucaaaggguugcuagcU...	101	1	HA1
......................uccuucaaaggguugcuagcA...	8	1	HA1
......................uccuucaaaggguugcuagcG...	1	1	HA1
......................uccuucaaaggguugcuagcc...	2	0	HA1
......................uccuucaaaggguugcuagccu..	1	0	HA1
......................uccuucaaaggguugcuagcUu..	2	1	HA1
......................uccuucaaaggguugcuagccuacg...	1	0	HA1
......................uccuucaaaggguugcuagccuacgu..	1	0	HA1
.......................ccuucaaaggguugcuagc..	1	0	HA1
.......................ccuucaaaggguugcuagA..	1	1	HA1
.......................ccuucaaaggguugcuagccu..	1	0	HA1
.........................uucaaaggguugcuagccua...	2	0	HA1
.........................uucaaaggguugcuagccuacg...	1	0	HA1
.........................uucaaaggguugcuagccuacA...	1	1	HA1
..........................ucaaaggguugcuagccuacg...	2	0	HA1
..................................uugcuagccuacguacgagug...	1	0	HA1
....agugcgagcugcccagugaagggcug...................................	1	0	HA1
....agugcgagcugcccagugaagggcugu..................................	4	0	HA1
....ugcgagcugcccagugaaggg......................................	1	0	HA1
....Ggcgagcugcccagugaagggcugu..................................	1	1	HA1
....gcgagcugcccagugaagggcu....................................	1	0	HA1
....gcgagcugcccagugaagggcugu..................................	2	0	HA1
....gUgagcugcccagugaagggcugu..................................	1	1	HA1
....cgagcugcccagugaaggA......................................	5	1	HA1
....cgagcugcccagugaagggcu....................................	3	0	HA1
....Ugagcugcccagugaagggcug...................................	2	1	HA1
....cgagcugcccagugaagggcug...................................	2	0	HA1
....Ggagcugcccagugaagggcugu..................................	2	1	HA1
....cgagcugcccagugaagggcugu..................................	4	0	HA1
....cgagcugcccagugaagggcugA.................................	1	1	HA1
....Ugagcugcccagugaagggcugu..................................	24	1	HA1
....Ugagcugcccagugaagggcuguu.................................	2	1	HA1
....gagcugcccagugaaggA......................................	106	1	HA1
....gagUugcccagugaaggg......................................	1	1	HA1
....gagcugcccagugaaggg......................................	78	0	HA1
....gagcugcccagugGaggg......................................	1	1	HA1
....gagcugcccagugaaggU......................................	1	1	HA1
....gagcugcccagugaagggU.....................................	2	1	HA1
....gCgcugcccagugaagggc.....................................	1	1	HA1
....gagcugcccagugaagggA.....................................	8	1	HA1
....gagcugcccagugaagggc.....................................	48	0	HA1
....gagcugcccagugaaggAc.....................................	3	1	HA1
....gagcugcccagugaagggcG....................................	1	1	HA1
....gagcugcccagugaagggcu....................................	127	0	HA1
....gagcugcccagGgaagggcu....................................	4	1	HA1
....gGgcugcccagugaagggcu....................................	1	1	HA1
....gagcugcccagugaagggUu....................................	2	1	HA1
....gagcugcccagugaagggAu....................................	5	1	HA1
....gagcugcccagugaagggUug...................................	4	1	HA1
....gagcugcccagugCagggcug...................................	1	1	HA1
....gagcugcccagGgaagggcug...................................	1	1	HA1
....gagcugcccagugGagggcug...................................	1	1	HA1
....gagcugcccagugaaggAcug...................................	4	1	HA1
....gagcugcccagugaagggcCg...................................	2	1	HA1
....gagcugccUagugaagggcug...................................	4	1	HA1
....gagcugcccagCgaagggcug...................................	2	1	HA1
....gagcugcccagugaaAggcug...................................	1	1	HA1
....gagcugcccagugaagggcGg...................................	2	1	HA1
....gagUugcccagugaagggcug...................................	3	1	HA1
....gagcugcccagugaagggcuA...................................	23	1	HA1
....gagcugUccagugaagggcug...................................	1	1	HA1
....gagcugcccagugaagggcug...................................	1041	0	HA1
....gagcugcccagugaagggcuU...................................	54	1	HA1
....gCgcugcccagugaagggcug...................................	5	1	HA1
....gagcugcccagugaagggAug...................................	1	1	HA1
....gagcugcccagugaaggUcugu..................................	1	1	HA1

Star **Mature**

guuacauugaacuguugaauaguccuucaaaggguugcuagccuacguacgagugcgagcugcccagugaagggcuguuugucguucacucuaucacacaucuugcagaaaa

....gagcGgcccagugaagggcugu..................................	2	1	HA1
....gagcugcccagugaagUgcugu..................................	7	1	HA1
....gagcugccGagugaagggcugu..................................	1	1	HA1
....gagcugcccagugaagggGugu..................................	1	1	HA1
....gagcugcccagugaagggcuAu..................................	27	1	HA1
....gagcugcccagugaaggAcugu..................................	1	1	HA1
....gagcugccUagugaagggcugu..................................	5	1	HA1
....gagcugcccagugGagggcugu..................................	3	1	HA1
....gagcugcAcagugaagggcugu..................................	2	1	HA1
....gagcugAccagugaagggcugu..................................	2	1	HA1
....Cagcugcccagugaagggcugu..................................	1	1	HA1
....gagcugcccaguAaagggcugu..................................	1	1	HA1
....gagcugcccagugaaCggcugu..................................	1	1	HA1
....gagcugcccagugaagggcugA..................................	73	1	HA1
....gagcugGccagugaagggcugu..................................	1	1	HA1
....gUgcugcccagugaagggcugu..................................	12	1	HA1
....gagcugcccagugaGgggcugu..................................	1	1	HA1
....gagcugcccCgugaagggcugu..................................	1	1	HA1
....gGgcugcccagugaagggcugu..................................	48	1	HA1
....gagcugcccagugaagggcugC..................................	3	1	HA1
....gagcugUccagugaagggcugu..................................	5	1	HA1
....gagcugcccagugaagggcCgu..................................	2	1	HA1
....gagcugcccaguUaagggcugu..................................	9	1	HA1
....gagcugcccagugaagCgcugu..................................	2	1	HA1
....gagcugcccagAgaagggcugu..................................	1	1	HA1
....gagcugcccaguCaagggcugu..................................	2	1	HA1
....gagUugcccagugaagggcugu..................................	11	1	HA1
....gaAcugcccagugaagggcugu..................................	2	1	HA1
....Aagcugcccagugaagggcugu..................................	3	1	HA1
....gagcugcccagGgaagggcugu..................................	2	1	HA1
....gagcugcccaUugaagggcugu..................................	1	1	HA1
....gagGugcccagugaagggcugu..................................	1	1	HA1
....gagcugccAagugaagggcugu..................................	2	1	HA1
....gagcugcccagugaaUggcugu..................................	2	1	HA1
....gagcuAcccagugaagggcugu..................................	9	1	HA1
....gagcugcccagugaagggAugu..................................	2	1	HA1
....gagcugcUcagugaagggcugu..................................	3	1	HA1
....gagcuCcccagugaagggcugu..................................	1	1	HA1
....gagcugcccagugaUgggcugu..................................	1	1	HA1
....gagcugcccagugaagggcugu..................................	12931	0	HA1
....gagcugcccagugaagggcuUu..................................	126	1	HA1
....gagcugcccagugaagAgcugu..................................	2	1	HA1
....gagcugcccagugaagggcugG..................................	11	1	HA1
....gagcugcccagugaagggcuCu..................................	3	1	HA1
....gagcugcccagCgaagggcugu..................................	7	1	HA1
....gagAugcccagugaagggcugu..................................	5	1	HA1
....gCgcugcccagugaagggcugu..................................	35	1	HA1
....gagcugcccagugaagggUugu..................................	12	1	HA1
....gagcugUccagugaagggcuguu.................................	1	1	HA1
....Cagcugcccagugaagggcuguu.................................	1	1	HA1
....gagcugcccagGgaagggcuguu.................................	1	1	HA1
....gagcugGccagugaagggcuguu.................................	1	1	HA1
....gagcugcccagugaagUgcuguu.................................	1	1	HA1
....gagcugcccagugaagggcuguu.................................	965	0	HA1
....gagcugcccagugaagggcuguC.................................	3	1	HA1
....gagcugcccCgugaagggcuguu.................................	1	1	HA1
....gagcugcccagugGagggcuguu.................................	1	1	HA1
....gaCcugcccagugaagggcuguu.................................	1	1	HA1
....gagcugcccagugaagggcuUuu.................................	2	1	HA1
....gagcuAcccagugaagggcuguu.................................	1	1	HA1
....gUgcugcccagugaagggcuguu.................................	1	1	HA1
....gagcugccUagugaagggcuguu.................................	2	1	HA1
....gGgcugcccagugaagggcuguu.................................	3	1	HA1
....gagcugcccagugaagggcuguA.................................	263	1	HA1
....gagcugcccaguAaagggcuguu.................................	1	1	HA1
....gagcugcccagugaagggAuguu.................................	1	1	HA1
....gCgcugcccagugaagggcuguu.................................	6	1	HA1
....gagcugcccagugaagggcuCuu.................................	1	1	HA1
....gagcugcccagugaagggcuguuu................................	2	0	HA1
....gagcugcccagugaagggcuguuA................................	5	1	HA1

Star **Mature**

```
guuacauugaacuguugaauaguccuucaaaggguugcuagccuacguacgagugcgagcugcccagugaagggcuguuugucguucacucuaucacacaucuugcagaaaa
........................................................gagcugcccagugaagggcuguAu................................   3     1   HA1
........................................................gagcugcccagugaagggcuguCu................................   1     1   HA1
........................................................gagcugcccagugaagggcuguuuU...............................   3     1   HA1
.........................................................agcugcccagugaagggcug...................................   1     0   HA1
..........................................................gcugcccagugaagggcugu..................................   1     0   HA1
...........................................................cugcccagugaagggcugu..................................   2     0   HA1
...........................................................cugcccagugaagggcuguu.................................   1     0   HA1
............................................................ugcccagugaagggcugu..................................   1     0   HA1
.............................................................gcccagugaagggcuguu.................................   1     0   HA1

...................uaguccuucaaaggguugcu.........................................................................   3     0   HS6
...................uaguccuucaaaggguugcuag.......................................................................   2     0   HS6
...................uaguccuucaaaggguugcuagc......................................................................   6     0   HS6
...................uaguccuucaaaggguugcuagA......................................................................   1     1   HS6
...................uaguccuucaaaggguugcuagcU.....................................................................   1     1   HS6
....................aguccuucaaaggguugcu.........................................................................   15    0   HS6
....................aguUcuucaaaggguugcua........................................................................   1     1   HS6
....................aguccuucaaaggguugcuU........................................................................   1     1   HS6
....................aguccuucaaaggguugcua........................................................................   12    0   HS6
....................aguccuucaaaggguugcuaU.......................................................................   18    1   HS6
....................aguccuucaaCggguugcuag.......................................................................   1     1   HS6
....................Cguccuucaaaggguugcuag.......................................................................   1     1   HS6
....................aguccuCcaaaggguugcuag.......................................................................   2     1   HS6
....................aguccuucaaaggguugcuag.......................................................................   950   0   HS6
....................aguccuucaaaggguugcGag.......................................................................   1     1   HS6
....................aguccuucaaaUgguugcuag.......................................................................   1     1   HS6
....................aguccuucaaaggguugUuag.......................................................................   1     1   HS6
....................aguccuucaaaggguugcAag.......................................................................   1     1   HS6
....................aguccuuUaaaggguugcuag.......................................................................   1     1   HS6
....................agucUuucaaaggguugcuagc......................................................................   1     1   HS6
....................agGccuucaaaggguugcuagc......................................................................   1     1   HS6
....................Gguccuucaaaggguugcuagc......................................................................   3     1   HS6
....................aguccuucaaaggguugcGagc......................................................................   2     1   HS6
....................aguccuucaaaggguugcuaAc......................................................................   1     1   HS6
....................agCccuucaaaggguugcuagc......................................................................   1     1   HS6
....................aguccuucaaaggguugcuagG......................................................................   15    1   HS6
....................aguccuucaaaggUuugcuagc......................................................................   1     1   HS6
....................aguccuucaaaggguugcuagc......................................................................   2080  0   HS6
....................aguccuucaaaggguugcuagA......................................................................   26    1   HS6
....................aguccuucaaaggguugcuagU......................................................................   331   1   HS6
....................aguccuucUaaggguugcuagc......................................................................   1     1   HS6
....................aguccuucaaCggguugcuagc......................................................................   1     1   HS6
....................Uguccuucaaaggguugcuagc......................................................................   3     1   HS6
....................aguccuucaaagggCugcuagc......................................................................   1     1   HS6
....................aguccuuUaaaggguugcuagc......................................................................   1     1   HS6
....................aguUcuucaaaggguugcuagc......................................................................   2     1   HS6
....................aguccuucaaaggguugcuagcU.....................................................................   1490  1   HS6
....................aguccuucaaaggguugcuagcG.....................................................................   1     1   HS6
....................agucUuucaaaggguugcuagcc.....................................................................   1     1   HS6
....................aguccuucaaaggguugcuagcc.....................................................................   37    0   HS6
....................aguccuucaaaggguugcuagcA.....................................................................   31    1   HS6
....................aguccuucaaaggguugcuagccA....................................................................   19    1   HS6
....................aguccuucaaaggguugcuagcUu....................................................................   112   1   HS6
....................aguccuucaaaggguugcuagccu....................................................................   24    0   HS6
....................aguccuucaaaggguugcuagcAu....................................................................   1     1   HS6
....................aguccuucaaaggguugcuagccuU...................................................................   1     1   HS6
....................aguccuucaaaggguugcuagccua...................................................................   2     0   HS6
.....................guccuucaaaggguugcuag.......................................................................   3     0   HS6
......................uccuucaaaggguugcuag.......................................................................   11    0   HS6
......................uccuucaaaggguugcuagc......................................................................   62    0   HS6
......................uccuucaaaggguugcuagU......................................................................   9     1   HS6
......................uccuucaaaggguugcuagA......................................................................   2     1   HS6
......................uccuuUaaaggguugcuagc......................................................................   1     1   HS6
......................uccuucaaaggguugcuagcU.....................................................................   70    1   HS6
......................uccuucaaaggguugcuagcc.....................................................................   1     0   HS6
......................uccuucaaaggguugcuagcA.....................................................................   1     1   HS6
......................uccuucaaaggguugcuagccu....................................................................   1     0   HS6
......................uccuucaaaggguugcuagcUua...................................................................   1     1   HS6
......................uccuucaaaggguugcuagccuacU.................................................................   1     1   HS6
.......................ccuucaaaggguugcuag.......................................................................   3     0   HS6
```

Star Mature

```
guuacauugaacuguugaauaguccuucaaaggguugcuagccuacguacgagugcgagcugcccagugaagggcuguuugucguucacucuaucacacaucuugcagaaaa
.......................ccuucaaaggguugcuagc......................................................................   3     0   HS6
.......................ccuucaaaggguugcuagU......................................................................   1     1   HS6
.......................ccuucaaaggguugcuagcU.....................................................................   1     1   HS6
........................cuucaaaggguugcuagcU.....................................................................   3     1   HS6
.........................uucaaaggguugcuagcU.....................................................................   4     1   HS6
.....................................................Ggcgagcugcccagugaagggcuguu.................................   1     1   HS6
.......................................................Ugagcugcccagugaagg.......................................   1     1   HS6
.......................................................cgagcugcccagugaaggA......................................   2     1   HS6
.......................................................cgagcugcccagugaagggcu....................................   2     0   HS6
.......................................................Ugagcugcccagugaagggcug...................................   12    1   HS6
.......................................................cgagcugcccagugaagggcug...................................   2     0   HS6
.......................................................Ugagcugcccagugaagggcugu..................................   6     1   HS6
.......................................................cgagcugcccagugaagggcuguu.................................   2     0   HS6
.......................................................cgagcugcccagugaagggcuguA.................................   1     1   HS6
.......................................................Ugagcugcccagugaagggcuguu.................................   1     1   HS6
........................................................gagcugcccagugaaggg......................................   76    0   HS6
........................................................gagcugcccagugaaggA......................................   670   1   HS6
........................................................gagcugcccagugaaAgg......................................   5     1   HS6
........................................................gagcugcccagugaaggU......................................   2     1   HS6
........................................................gagcugcccagugaaggAc.....................................   1     1   HS6
........................................................gagcugcccagugaagggc.....................................   114   0   HS6
........................................................gagcugcccagugaagggA.....................................   2     1   HS6
........................................................gUgcugcccagugaagggc.....................................   1     1   HS6
........................................................gagcugcUcagugaagggc.....................................   1     1   HS6
........................................................gagcugcccagugaagggcu....................................   186   0   HS6
........................................................gagcugcccagugaagggAu....................................   1     1   HS6
........................................................gagcugcccagugaagggcA....................................   1     1   HS6
........................................................gagcugcccagugaagggUu....................................   1     1   HS6
........................................................gagcugcccagugaagggUug...................................   8     1   HS6
........................................................gagcugcccagugaagggcGg...................................   68    1   HS6
........................................................gagcugcccagugaaggCcug...................................   2     1   HS6
........................................................gagcugcccagugaagggcuU...................................   74    1   HS6
........................................................gagcugcccagugaagggcuA...................................   58    1   HS6
........................................................gagcugcccagugaagggcug...................................   5787  0   HS6
........................................................gagcugcccagugaagggcCg...................................   3     1   HS6
........................................................gagUugcccagugaagggcug...................................   5     1   HS6
........................................................gagcugcccagugCagggcug...................................   1     1   HS6
........................................................gagcugcccagugaaggAcug...................................   10    1   HS6
........................................................gCgcugcccagugaagggcug...................................   6     1   HS6
........................................................gagcuAcccagugaagggcug...................................   8     1   HS6
........................................................gagcugcccaguUaagggcug...................................   2     1   HS6
........................................................gagcugcccaCugaagggcug...................................   4     1   HS6
........................................................gagcugcccaguCaagggcug...................................   1     1   HS6
........................................................gagcugcccaUugaagggcug...................................   3     1   HS6
........................................................gagcugccUagugaagggcug...................................   3     1   HS6
........................................................gaUcugcccagugaagggcug...................................   3     1   HS6
........................................................gaCcugcccagugaagggcug...................................   1     1   HS6
........................................................gagcugcccaAugaagggcug...................................   1     1   HS6
........................................................gagcugUccagugaagggcug...................................   7     1   HS6
........................................................gagcugcccagugaaUggcug...................................   2     1   HS6
........................................................gGgcugcccagugaagggcug...................................   7     1   HS6
........................................................gagcugcccagugGagggcug...................................   1     1   HS6
........................................................gagcugcccagugaagggGug...................................   1     1   HS6
........................................................gagcugcccagugaagggAug...................................   1     1   HS6
........................................................gagcugcccagugaagCgcug...................................   3     1   HS6
........................................................gagcugcccaUugaagggcugu..................................   1     1   HS6
........................................................gagcugcccagugaagggGugu..................................   1     1   HS6
........................................................gagcugcccagugaagggcCgu..................................   1     1   HS6
........................................................gagcugcccagugaagggUugu..................................   2     1   HS6
........................................................gagcugcccagugaagggcuAu..................................   2     1   HS6
........................................................gagcugcccagugaagggAugu..................................   1     1   HS6
........................................................gagcugcccagugaaggAcugu..................................   1     1   HS6
........................................................gagcugcccagugaagggcugA..................................   14    1   HS6
........................................................gagcuAcccagugaagggcugu..................................   3     1   HS6
........................................................gagcugcccaCugaagggcugu..................................   1     1   HS6
........................................................gaUcugcccagugaagggcugu..................................   4     1   HS6
........................................................gUgcugcccagugaagggcugu..................................   6     1   HS6
........................................................gagUugcccagugaagggcugu..................................   6     1   HS6
........................................................gCgcugcccagugaagggcugu..................................   1     1   HS6
........................................................gagcugcccagugaagggcuUu..................................   6     1   HS6
```

```
                    Star                                  Mature
guuacauugaacuguugaauaguccuucaaaggguugcuagccuacguacgagugcgagcugcccagugaagggcuguuugucguucacucuaucacacaucuugcagaaaa
........................................................gagcugcccagugaagggcugC..................................    11      1       HS6
........................................................gagcugUccagugaagggcugu..................................    1       1       HS6
........................................................gagcAgcccagugaagggcugu..................................    1       1       HS6
........................................................gagcuCcccagugaagggcugu..................................    1       1       HS6
........................................................gGgcugcccagugaagggcugu..................................    7       1       HS6
........................................................gagcugcccagugaagCgcugu..................................    1       1       HS6
........................................................gagcugcccagugaUgggcugu..................................    1       1       HS6
........................................................gagcugcccagugaagggcugG..................................    13      1       HS6
........................................................gagcugcccaguUaagggcugu..................................    1       1       HS6
........................................................gagcugcccagugaGgggcugu..................................    2       1       HS6
........................................................gagcugcccagugaagggcugu..................................    3205    0       HS6
........................................................gagcugccUagugaagggcugu..................................    2       1       HS6
........................................................Uagcugcccagugaagggcuguu.................................    1       1       HS6
........................................................gagcugcccagugaagggcuguA.................................    37      1       HS6
........................................................gagcugcccagugaaggUcuguu.................................    1       1       HS6
........................................................gagcugUccagugaagggcuguu.................................    1       1       HS6
........................................................gagcugcccagugaagggcuguu.................................    461     0       HS6
........................................................gagcugcccagugaagggcugCu.................................    1       1       HS6
........................................................gagcCgcccagugaagggcuguu.................................    1       1       HS6
........................................................gagcugcccagugaagggcuguuu................................    1       0       HS6
.........................................................agcugcccagugaagggcugu..................................    2       0       HS6
..........................................................gAugcccagugaagggcugu..................................    1       1       HS6
..........................................................gcugcccagugaagggcugu..................................    1       0       HS6
...........................................................cugcccagugaagggcugu..................................    1       0       HS6
............................................................ugcccagugaagggcugu..................................    4       0       HS6
.............................................................gcccagugaagggcuguu.................................    2       0       HS6
```

Provisional ID : SczTNLB_2740_2139
Score total : 5
Score for star read(s) : -1.3
Score for read counts : 0
Score for mfe : 1.7
Score for randfold : 1.6
Score for cons. seed : 3
Total read count : 326100
Mature read count : 279639
Loop read count : 238
Star read count : 46223

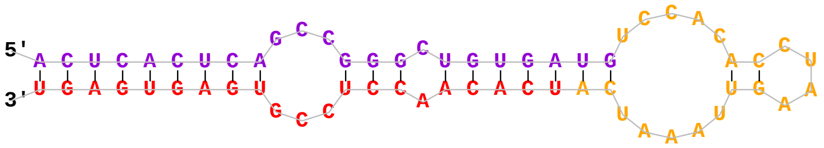

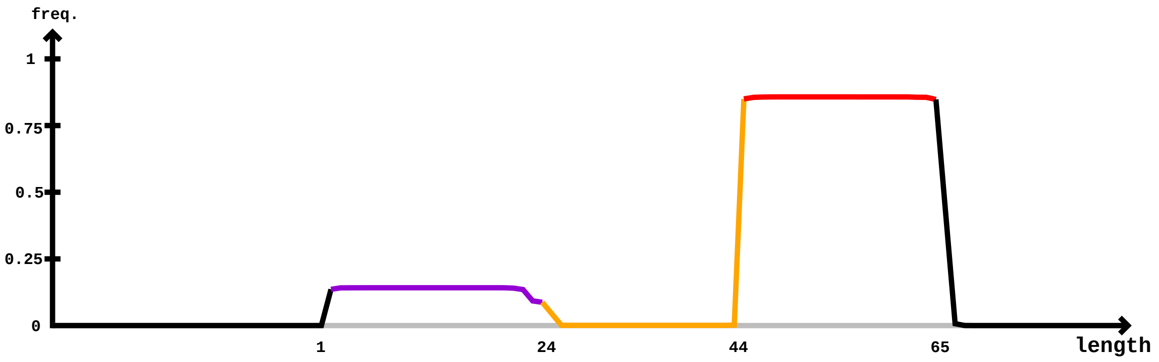

Star Mature

```
5'- cccuguggaaguccuguucuauacuaaacucacucagccgggcugugauguccacaccuaaguuaaaaucaucacaaccuccgugagugagugacguauacaggaccaauc  -3'   obs
    cccuguggaaguccuguucuauacuaaacucacucagccgggcugugauguccacaccuaaguuaaaaucaucacaaccuccgugagugagugacguauacaggaccaauc         exp
    ..........((((((...((((...(((((((((...(((.(((((((.....((....)).....))))))).)))...)))))))))...))))))))))..... reads   mm   sample
    .........................aaacucacucagccgggcugugU............................................................... 1   1   HS5
    ...........................acucacucagccgggcug.................................................................. 1   0   HS5
    ...........................acucacucagccgggcugu................................................................. 5   0   HS5
    ...........................acucUcucagccgggcugug................................................................ 1   1   HS5
    ...........................acucacucagccgggcugug................................................................ 61   0   HS5
    ...........................acucacucagccgggcuguU................................................................ 2   1   HS5
    ...........................acucacucagccggAcuguga............................................................... 2   1   HS5
    ...........................acucacucagccgggcugugG............................................................... 2   1   HS5
    ...........................acucacuUagccgggcuguga............................................................... 1   1   HS5
    ...........................acucacucagcAgggcuguga............................................................... 2   1   HS5
    ...........................acucacucagccgggcuUuga............................................................... 1   1   HS5
    ...........................acucacucagccgggcGguga............................................................... 1   1   HS5
    ...........................acucacucagccCggcuguga............................................................... 1   1   HS5
    ...........................acucacucaAccgggcuguga............................................................... 1   1   HS5
    ...........................acucacucagccgggAuguga............................................................... 5   1   HS5
    ...........................acucaUucagccgggcuguga............................................................... 3   1   HS5
    ...........................aGucacucagccgggcuguga............................................................... 3   1   HS5
    ...........................acucacucagccgggcugugU............................................................... 9   1   HS5
    ...........................acucacucagccAggcuguga............................................................... 2   1   HS5
    ...........................acucacucagccgggcugAga............................................................... 2   1   HS5
    ...........................acucaAucagccgggcuguga............................................................... 2   1   HS5
    ...........................acucacucagccgggcuguga............................................................... 2104   0   HS5
    ...........................acucacucagUcgggcuguga............................................................... 3   1   HS5
    ...........................acucacucagcGgggcuguga............................................................... 1   1   HS5
    ...........................acucacucagAcgggcuguga............................................................... 1   1   HS5
    ...........................acuUacucagccgggcuguga............................................................... 2   1   HS5
    ...........................acucacucagccgggcugCga............................................................... 2   1   HS5
    ...........................acucacucagccgggUuguga............................................................... 4   1   HS5
    ...........................acucacucagccggUcuguga............................................................... 3   1   HS5
    ...........................acucacuUagccgggcugugau.............................................................. 1   1   HS5
    ...........................acucacucagccgggcugugaA.............................................................. 5   1   HS5
    ...........................acucacucagccgggcugugGu.............................................................. 1   1   HS5
    ...........................acucacucagccgggcugugau.............................................................. 396   0   HS5
```

Star **Mature**

cccuguggaaguccuguucuauacuaaacucacucagccgggcugugauguccacaccuaaguuaaaucaucacaaccuccgugagugagugacguauacaggaccaauc			
...........................acucacucaAccgggcugugau...	2	1	HS5
...........................acucacucagccgggAugugau...	1	1	HS5
...........................acCcacucagccgggcugugau...	1	1	HS5
...........................acucaUucagccgggcugugau...	1	1	HS5
...........................acucacucagUcgggcugugau...	1	1	HS5
...........................acucacucagccgggcugugUu...	2	1	HS5
...........................acucUcucagccgggcugugau...	1	1	HS5
...........................acucacucagccCggcugugau...	1	1	HS5
...........................acucacucagccgggcugugauU..	160	1	HS5
...........................acucacucagccgggcugugaug..	122	0	HS5
...........................acucacucagccCggcugugaug..	1	1	HS5
...........................acucacucagcUgggcugugaug..	1	1	HS5
...........................acucacucagccgggAugugaug..	2	1	HS5
...........................aUucacucagccgggcugugaug..	1	1	HS5
...........................acucacucagccgggcugugauA..	26	1	HS5
...........................acucacucagccgggcugugauC..	1	1	HS5
...........................acucaUucagccgggcugugaug..	1	1	HS5
...........................acucacucagccgggcugugauUu...	3	1	HS5
...........................acucacucagccgggcugugaugu...	8	0	HS5
...........................acucacucagccgggcugCgaugu...	1	1	HS5
...........................acucacucagccgggcugugauguA..	1	1	HS5
...........................acucacucagccgggcugugauguU..	9	1	HS5
............................cucacucagccgggcugugaug..	2	0	HS5
.............................ucacucagccgggcugugau...	1	0	HS5
.............................ucacucagccgggcugugaug..	3	0	HS5
.............................ucacucagccgggcugugauU..	1	1	HS5
.............................Ccacucagccgggcugugaug..	1	1	HS5
.............................ucacucagccgggcugugaugu...	1	0	HS5
..............................cacucagccgggcuguga..	1	0	HS5
................................cucagccgggcugugauU..	1	1	HS5
................................cucagccgggcugugaug..	1	0	HS5
....uguccacaccuaaguuaaauc...	1	0	HS5
....guccacaccuaaguuaaaucau.......................................	1	0	HS5
....uccacaccuaaguuaaauc...	4	0	HS5
....uccacaccuaaguuaaauca..	43	0	HS5
....uccacaccuaaguuaaaucau.......................................	5	0	HS5
....uccacaccuaaguuaaaucaA.......................................	2	1	HS5
....uuaaaucaucacaaccuccgugag........................	1	0	HS5
....Uaucacaaccuccgugagugagu...................	1	1	HS5
....Uucacaaccuccgugagugagu...................	8	1	HS5
....aucacaaccuccgugagugagu...................	2	0	HS5
....Cucacaaccuccgugagugagug..................	1	1	HS5
....ucacaaccuccgugagug......................	25	0	HS5
....ucacaaccuccgugaguga.....................	1	0	HS5
....ucacaaccuccgugaCugag....................	2	1	HS5
....ucacaaccuccgugagugag....................	112	0	HS5
....ucacaaccuccguCagugag....................	1	1	HS5
....ucacaaccuccUugagugag....................	1	1	HS5
....ucacaaccuccCugagugag....................	1	1	HS5
....uUacaaccuccgugagugag....................	2	1	HS5
....ucacaaccuccgugGgugag....................	1	1	HS5
....ucacaaccuccgugaguCagu...................	5	1	HS5
....ucacaaccuccgugagugaAu...................	2	1	HS5
....ucacaaccuGcgugagugagu...................	3	1	HS5
....ucacaaccuccAugagugagu...................	21	1	HS5
....ucacaaccuccgugGgugagu...................	3	1	HS5
....ucacaaccuccgugagugagG...................	5	1	HS5
....ucacaaccCccgugagugagu...................	6	1	HS5
....ucacaCccuccgugagugagu...................	3	1	HS5
....ucaAaaccuccgugagugagu...................	1	1	HS5
....Gcacaaccuccgugagugagu...................	21	1	HS5
....ucacaGccuccgugagugagu...................	9	1	HS5
....ucacaaccuccguUagugagu...................	5	1	HS5
....ucacaaccuccUugagugagu...................	12	1	HS5
....ucacaaccucAgugagugagu...................	2	1	HS5
....Acacaaccuccgugagugagu...................	17	1	HS5
....ucacaaccuccgugagGgagu...................	2	1	HS5
....uUacaaccuccgugagugagu...................	10	1	HS5
....ucGcaaccuccgugagugagu...................	5	1	HS5
....ucacaaccuccgugagAgagu...................	8	1	HS5

Star Mature

```
cccuguggaaguccuguucuauacuaaacucacucagccgggcugugaauguccacaccuaaguuaaaucaucacaaccuccgugagugagugacguauacaggaccaauc
......................................................................ucacaaccuccgugagugagC...................  25     1   HS5
......................................................................ucacaaccuccgugUgugagu...................  7      1   HS5
......................................................................ucacUaccuccgugagugagu...................  16     1   HS5
......................................................................ucacaaccucUgugagugagu...................  5      1   HS5
......................................................................ucacaaccuccgugaUugagu...................  13     1   HS5
......................................................................ucacaaccuccgugagugGgu...................  4      1   HS5
......................................................................ucacaacAuccgugagugagu...................  1      1   HS5
......................................................................ucacaaccuccgugagugagu...................  29797  0   HS5
......................................................................ucaGaaccuccgugagugagu...................  1      1   HS5
......................................................................ucacaacUuccgugagugagu...................  6      1   HS5
......................................................................ucacaaccuccgAgagugagu...................  1      1   HS5
......................................................................ucacaaccuccgugaAugagu...................  16     1   HS5
......................................................................ucacaaUcuccgugagugagu...................  9      1   HS5
......................................................................Ccacaaccuccgugagugagu...................  14     1   HS5
......................................................................ucacaaccuccgugagCgagu...................  8      1   HS5
......................................................................ucacaaccuccgugagugUgu...................  8      1   HS5
......................................................................ucUcaaccuccgugagugagu...................  1      1   HS5
......................................................................ucacaaccuccgugCgugagu...................  2      1   HS5
......................................................................ucacCaccuccgugagugagu...................  6      1   HS5
......................................................................ucacaaccuccguCagugagu...................  6      1   HS5
......................................................................ucacaaccuccgGgagugagu...................  3      1   HS5
......................................................................ucacaaccuccgCgagugagu...................  29     1   HS5
......................................................................ucacaUccuccgugagugagu...................  14     1   HS5
......................................................................ucaUaaccuccgugagugagu...................  15     1   HS5
......................................................................ucacaaccuccgugaguAagu...................  3      1   HS5
......................................................................ucacaaccuccgugagugCgu...................  1      1   HS5
......................................................................ucacaaccuAcgugagugagu...................  3      1   HS5
......................................................................ucacaaccuccCugagugagu...................  21     1   HS5
......................................................................ucacaaccAccgugagugagu...................  2      1   HS5
......................................................................ucacaaccuccgugaguUagu...................  3      1   HS5
......................................................................ucacaaccuUcgugagugagu...................  2      1   HS5
......................................................................ucacGaccuccgugagugagu...................  8      1   HS5
......................................................................ucacaaccuccgugagugagA...................  62     1   HS5
......................................................................ucacaaccuccgugaCugagu...................  26     1   HS5
......................................................................ucacaaccuccgugagugaUu...................  1      1   HS5
......................................................................ucacaaccuccUugagugagug..................  3      1   HS5
......................................................................ucacaaccuccAugagugagug..................  2      1   HS5
......................................................................ucacaaUcuccgugagugagug..................  2      1   HS5
......................................................................ucacCaccuccgugagugagug..................  1      1   HS5
......................................................................ucacaacAuccgugagugagug..................  1      1   HS5
......................................................................ucacaaccuccCugagugagug..................  3      1   HS5
......................................................................ucacaaccuccgugagugGgug..................  2      1   HS5
......................................................................ucacaGccuccgugagugagug..................  1      1   HS5
......................................................................ucacGaccuccgugagugagug..................  4      1   HS5
......................................................................ucacaaccuUcgugagugagug..................  1      1   HS5
......................................................................ucacaaccuccgCgagugagug..................  3      1   HS5
......................................................................ucacaaccuccgugaAugagug..................  3      1   HS5
......................................................................ucacaaccuccgugagugagug..................  4578   0   HS5
......................................................................Acacaaccuccgugagugagug..................  5      1   HS5
......................................................................ucacaCccuccgugagugagug..................  1      1   HS5
......................................................................ucacaaccuccgugagGgagug..................  2      1   HS5
......................................................................ucacaaccuccgugagugaUug..................  3      1   HS5
......................................................................uUacaaccuccgugagugagug..................  1      1   HS5
......................................................................ucacaaccuccgugaguAagug..................  1      1   HS5
......................................................................ucacaaccuccgugaUugagug..................  5      1   HS5
......................................................................ucacaaccuccgugaguCagug..................  1      1   HS5
......................................................................ucacaaccuccguCagugagug..................  3      1   HS5
......................................................................ucGcaaccuccgugagugagug..................  1      1   HS5
......................................................................ucacaaccCccgugagugagug..................  1      1   HS5
......................................................................ucacaaccuccgugagugagCg..................  1      1   HS5
......................................................................ucacaUccuccgugagugagug..................  2      1   HS5
......................................................................ucacaaccuccgugagugaguC..................  4      1   HS5
......................................................................ucacaaccuccgugUgugagug..................  2      1   HS5
......................................................................ucacaaccuccguUagugagug..................  1      1   HS5
......................................................................ucacaaccuccgugagCgagug..................  1      1   HS5
......................................................................ucacaaccuccgugGgugagug..................  1      1   HS5
......................................................................ucacaaccuccgugagugaguA..................  21     1   HS5
......................................................................ucacaaccuccgugaCugagug..................  5      1   HS5
......................................................................ucaUaaccuccgugagugagug..................  3      1   HS5
......................................................................Ccacaaccuccgugagugagug..................  3      1   HS5
```

Star Mature

```
cccuguggaaguccuguucuauacuaaacucacucagccgggcugugauguccacaccuaaguuaaaucaucacaaccuccgugagugagugacguauacaggaccaauc
......................................................................ucacaaccuccgugagugaguU..................   65     1   HS5
......................................................................Gcacaaccuccgugagugagug..................   2      1   HS5
......................................................................ucacaaccuccgugagugagugG.................   2      1   HS5
......................................................................ucacaaccuccgCgagugaguga.................   1      1   HS5
......................................................................ucacaaccuccgugaAugaguga.................   1      1   HS5
......................................................................ucacaaccuccgugagugagugU.................   106    1   HS5
......................................................................ucacaaccuccgugagugaguga.................   40     0   HS5
......................................................................ucacaaccuccgugaCugaguga.................   1      1   HS5
......................................................................ucacaaccuccgugagugagugaA................   1      1   HS5
......................................................................ucacaaccuccgugagugagugaU................   1      1   HS5
......................................................................ucacaaccuccgugagugagugacA...............   1      1   HS5
.......................................................................cacaaccuccgugagugag....................   2      0   HS5
.......................................................................cacaaccCccgugagugagu...................   1      1   HS5
.......................................................................cacaaccuccgugagugUgu...................   2      1   HS5
.......................................................................cacaaccuAcgugagugagu...................   1      1   HS5
.......................................................................cacaaccuccgugagugagu...................   149    0   HS5
.......................................................................cacaaccuccgugagugaguU..................   1      1   HS5
.......................................................................cacaaccuccgugagugagug..................   59     0   HS5
.......................................................................cacaaccuccgugagugaguga.................   59     0   HS5
.......................................................................cacaaccuccgugagugagugU.................   13     1   HS5
.......................................................................cacaaccuccgugagugagugaU................   2      1   HS5
.......................................................................cacaaccuccgugagugagugaA................   3      1   HS5
........................................................................acaaccuccCugagugagu...................   2      1   HS5
........................................................................Ucaaccuccgugagugagu...................   3      1   HS5
........................................................................acaaccucUgugagugagu...................   1      1   HS5
........................................................................acaaccuccgugagugagu...................   5      0   HS5
........................................................................acaaccuccgugagugaguU..................   1      1   HS5
........................................................................acaaccuccgugagugaguga.................   7      0   HS5
.........................................................................caaccuccgugaCugagu...................   1      1   HS5
.........................................................................caaccuccgugagugagu...................   5      0   HS5
.........................................................................caaccuccgugagugagug..................   5      0   HS5

.........................aaacucacucagccgggcugug...............................................................   1      0   HS7
..........................Uacucacucagccgggcuguga..............................................................   2      1   HS7
..........................Uacucacucagccgggcugugaug............................................................   1      1   HS7
...........................acucacucagccgggcug.................................................................   2      0   HS7
...........................acucacucagccgggcugG................................................................   1      1   HS7
...........................acucacucagccgggcugu................................................................   40     0   HS7
...........................acucacuGagccgggcugug...............................................................   1      1   HS7
...........................Ucucacucagccgggcugug...............................................................   1      1   HS7
...........................acucacucagccgggcuCug...............................................................   1      1   HS7
...........................acucacucagccgggcugug...............................................................   284    0   HS7
...........................acucacucagccgggcugCg...............................................................   1      1   HS7
...........................acucacucagccgggcuguU...............................................................   3      1   HS7
...........................acucacucagccggUcugug...............................................................   1      1   HS7
...........................acucacucagccgggcuUuga..............................................................   1      1   HS7
...........................acucacucagccgggcuguga..............................................................   1218   0   HS7
...........................acucacucagccgggcuAuga..............................................................   1      1   HS7
...........................acucacuUagccgggcuguga..............................................................   1      1   HS7
...........................acucacucagccgggcugugU..............................................................   31     1   HS7
...........................acucacucagUcgggcuguga..............................................................   1      1   HS7
...........................aGucacucagccgggcuguga..............................................................   2      1   HS7
...........................acucacucagccgggcugugG..............................................................   2      1   HS7
...........................acucacucagccgggcugugaA.............................................................   2      1   HS7
...........................acucacucagccgggcugugUu.............................................................   1      1   HS7
...........................Ucucacucagccgggcugugau.............................................................   1      1   HS7
...........................acuUacucagccgggcugugau.............................................................   1      1   HS7
...........................acucacucagccgggcugugau.............................................................   129    0   HS7
...........................acucacucagccgggcugugauU............................................................   134    1   HS7
...........................acucacucagccgggcugugauA............................................................   9      1   HS7
...........................acucacucagccgggcugugaug............................................................   481    0   HS7
...........................acucacucagccgggcugugauC............................................................   2      1   HS7
...........................acucacucagccgggcugugaGg............................................................   1      1   HS7
...........................acucaUucagccgggcugugaug............................................................   2      1   HS7
...........................acucacucagccggCcugugaug............................................................   1      1   HS7
...........................acucacucUgccgggcugugaug............................................................   1      1   HS7
...........................acucacucagccgggcuCugaug............................................................   1      1   HS7
...........................acucacuUagccgggcugugaug............................................................   1      1   HS7
...........................aGucacucagccgggcugugaug............................................................   1      1   HS7
...........................acucacucagUcgggcugugaug............................................................   1      1   HS7
```

Star Mature

cccuguggaagucuguucuauacuaaacucacucagccgggcugugauguccacaccuaaguuaaaucaucacaaccuccgugagugagugacguauacaggaccaauc			
..........................acucacucagccggAcugugaug..	2	1	HS7
..........................Gcucacucagccgggcugugaug..	1	1	HS7
..........................acucacucagccgggcugugaugA...	13	1	HS7
..........................acucacucagccgggcugugauUu...	3	1	HS7
..........................acucacucagccgggcugugaugu...	15	0	HS7
..........................acucacucagccgggcugugauguU..	21	1	HS7
...........................cucacucagccgggcugugau...	1	0	HS7
............................ucacucagccgggcugug...	3	0	HS7
............................ucacucagccgggcuguga..	3	0	HS7
............................ucacucagccgggcugugaug..	2	0	HS7
............................ucacucagccgggcugugaugu...	1	0	HS7
.............................cacucagccgggcuguga..	1	0	HS7
.............................cacucagccgggcugugaug..	3	0	HS7
..............................acucagccgggcugugaug..	2	0	HS7
..............................acucagccgggcugugauU..	2	1	HS7
...............................cucagccgggcugugaug..	4	0	HS7
....uguccacaccuaaguuaaaucauc......................................	3	0	HS7
....uccacaccuaaguuaaauc...	5	0	HS7
....uccacaccuaaguuaaauca..	4	0	HS7
....uccacaccuaaguuaaaucau.......................................	2	0	HS7
....uccacaccuaaguuaaaucaA.......................................	1	1	HS7
....Aaucacaaccuccgugagugagu...................	1	1	HS7
....Uucacaaccuccgugagugagu...................	23	1	HS7
....Gucacaaccuccgugagugagu...................	2	1	HS7
....aucacaaccuccgugagugagu...................	8	0	HS7
....Uucacaaccuccgugagugagug..................	3	1	HS7
....ucacGaccuccgugagug......................	1	1	HS7
....ucacaaccuccgugagug......................	29	0	HS7
....ucacaaccuccgugagGg......................	4	1	HS7
....ucacaaccuccgugagCg......................	6	1	HS7
....ucacaaccuccgugaguga.....................	2	0	HS7
....ucacaaccuccgugagugag....................	172	0	HS7
....ucacaaccuccgugaCugag....................	1	1	HS7
....ucacaaccucAgugagugag....................	1	1	HS7
....ucaUaaccuccgugagugagu...................	14	1	HS7
....ucacaaccuccgCgagugagu...................	1	1	HS7
....ucacaaccuccgugagugaUu...................	1	1	HS7
....ucacaaccuccgugagugagG...................	386	1	HS7
....uUacaaccuccgugagugagu...................	4	1	HS7
....ucacaaccuccgugaUugagu...................	3	1	HS7
....ucUcaaccuccgugagugagu...................	40	1	HS7
....ucacaaccuccgugaguAagu...................	1	1	HS7
....ucacaaccuccCugagugagu...................	3	1	HS7
....ucacaGccuccgugagugagu...................	1	1	HS7
....Gcacaaccuccgugagugagu...................	1	1	HS7
....ucacaaccuccgugaCugagu...................	1	1	HS7
....ucacGaccuccgugagugagu...................	3	1	HS7
....ucacaaccuccUugagugagu...................	20	1	HS7
....ucacaaccuccguCagugagu...................	3	1	HS7
....ucacaaccuccgugagugaAu...................	4	1	HS7
....ucacaaUcuccgugagugagu...................	7	1	HS7
....ucacaaccuccgugGgugagu...................	2	1	HS7
....Acacaaccuccgugagugagu...................	2	1	HS7
....ucGcaaccuccgugagugagu...................	6	1	HS7
....ucacaaccucGgugagugagu...................	4	1	HS7
....ucacaaccGccgugagugagu...................	1	1	HS7
....ucacaaccucUgugagugagu...................	7	1	HS7
....ucacaaccuccgugCgugagu...................	1	1	HS7
....ucacaaccuccgugagugagA...................	62	1	HS7
....ucacCaccuccgugagugagu...................	1	1	HS7
....uGacaaccuccgugagugagu...................	1	1	HS7
....ucacaaccuccgugUgugagu...................	1	1	HS7
....ucacaaccuccguUagugagu...................	3	1	HS7
....ucaAaaccuccgugagugagu...................	1	1	HS7
....ucacaaccuccgugagugagC...................	15	1	HS7
....ucacaaccuUcgugagugagu...................	7	1	HS7
....ucacaaccuccgugaguUagu...................	3	1	HS7
....ucacaaccuccgugaguCagu...................	4	1	HS7
....ucacaaccCccgugagugagu...................	8	1	HS7
....ucacaaccuccAugagugagu...................	1	1	HS7

Star Mature

cccuguggaaguccuguucuauacuaaacucacucagccgggcugugauguccacaccuaaguuaaaucaucacaaccuccgugagugagugacguauacaggaccaauc			
....uAacaaccuccgugagugagu...................	1	1	HS7
....ucacaaccAccgugagugagu...................	1	1	HS7
....ucacaaccuccgugaAugagu...................	1	1	HS7
....ucacaaccuccgugagugGgu...................	8	1	HS7
....ucacaaccuccgugagugUgu...................	12	1	HS7
....ucacaaccuccgugagugagu...................	23136	0	HS7
....ucacUaccuccgugagugagu...................	6	1	HS7
....ucacaacGuccgugagugagu...................	6	1	HS7
....ucacaaccuccgGgagugagu...................	2	1	HS7
....ucacaaccuccgugagGgagu...................	2	1	HS7
....ucacaacUuccgugagugagu...................	6	1	HS7
....ucaUaaccuccgugagugagug..................	1	1	HS7
....ucacaaccuccgugagugaguA..................	24	1	HS7
....ucacaaccuccUugagugagug..................	1	1	HS7
....ucacaaccuccgugagugaguC..................	1	1	HS7
....ucacaaccuccgugagugagCg..................	3	1	HS7
....ucUcaaccuccgugagugagug..................	5	1	HS7
....ucacaaccuccgugagugGgug..................	1	1	HS7
....ucacaacAuccgugagugagug..................	1	1	HS7
....ucacaaccuccCugagugagug..................	1	1	HS7
....ucacaaccuccgugaguCagug..................	1	1	HS7
....uUacaaccuccgugagugagug..................	2	1	HS7
....ucacaaccuccgugagugaguU..................	18	1	HS7
....ucacaaccuccgugaUugagug..................	1	1	HS7
....ucacaaccuccgugagugagGg..................	2	1	HS7
....ucacaaccuccgugagugagug..................	2181	0	HS7
....ucacaaUcuccgugagugagug..................	1	1	HS7
....ucacaaccuccgugagugagugU.................	22	1	HS7
....ucacaaccuccgugagugaguga.................	65	0	HS7
....ucacaaccuccgugagugagugC.................	1	1	HS7
....ucacaaccuccgugagugagugGc................	1	1	HS7
....ucacaaccuccgugagugagugCc................	1	1	HS7
....ucacaaccuccgugagugagugaU................	1	1	HS7
....ucacaaccuccgugagugagugaA................	2	1	HS7
....ucacaaccuccgugagugagugacU...............	1	1	HS7
....cacaaccuccgugagugag....................	2	0	HS7
....cacaaccuccUugagugagu...................	1	1	HS7
....cacaacUuccgugagugagu...................	1	1	HS7
....cacaaccuccgugagugagG...................	1	1	HS7
....cacaaccuccgugagugagu...................	132	0	HS7
....cacaaccuccgugagugagug..................	26	0	HS7
....cacaaccuccgugagugaguga.................	17	0	HS7
....cacaaccuccgugagugagugU.................	4	1	HS7
....cacaaccuccgugagugagugaA................	4	1	HS7
....cacaaccuccgugagugagugaU................	1	1	HS7
....Ucaaccuccgugagugagu...................	3	1	HS7
....acaaccuccgugagugagG...................	1	1	HS7
....acaaccuccgugagugagu...................	12	0	HS7
....acaaccuccgugagugagugU.................	2	1	HS7
....caaccuccgugagugagu...................	18	0	HS7
....caaccuccgugagugagug..................	4	0	HS7
....aaccuccgugagugagug..................	1	0	HS7
...........................acucacucagcUgggcugugaug..	1	1	HS2
...........................acucacucagccgggcugugaug..	284	0	HS2
...........................acucacucaAccgggcugugaug..	1	1	HS2
...........................acCcacucagccgggcugugaug..	1	1	HS2
...........................acucacucagUcgggcugugaug..	4	1	HS2
............................cucacucagccgggcugugaug..	44	0	HS2
....ucacaaccuccgugagugagu...................	469	0	HS2
....ucaUaaccuccgugagugagu...................	1	1	HS2
....ucacaaccuccgugagugagC...................	3	1	HS2
....ucacaaccuccgugagGgagu...................	1	1	HS2
....ucacaaccucAgugagugagu...................	1	1	HS2
....ucacaaccCccgugagugagug..................	1	1	HS2
....ucacaaccuccgugagugagug..................	136	0	HS2
.........................aaacucacucagccgggcuguA...	1	1	HA1
..........................Uacucacucagccgggcuguga..	2	1	HA1
...........................acucacucagccgggcug...	7	0	HA1

Star **Mature**

cccuguggaaguccuguucuauacuaaacucacucagccgggcugugauguccacaccuaaguuaaaucaucacaaccuccgugagugagugacguauacaggaccaauc			
...........................acucacucagccgggcugu..	12	0	HA1
...........................acucacucagccgggcugA..	1	1	HA1
...........................acucacucagUcgggcugug...	1	1	HA1
...........................acucacucagccAggcugug...	1	1	HA1
...........................acucacuUagccgggcugug...	1	1	HA1
...........................acucacucagccgggcugug...	262	0	HA1
...........................acucacucagccgggcuguU...	6	1	HA1
...........................acucacucUgccgggcugug...	1	1	HA1
...........................Ucucacucagccgggcugug...	1	1	HA1
...........................acucacucagcAgggcuguga..	1	1	HA1
...........................acucacucagccgggcugugC..	4	1	HA1
...........................acucacucagccgCgcuguga..	1	1	HA1
...........................Ucucacucagccgggcuguga..	1	1	HA1
...........................aGucacucagccgggcuguga..	5	1	HA1
...........................acucacucagUcgggcuguga..	6	1	HA1
...........................acucacucagccgggcugCga..	3	1	HA1
...........................acucacucagccggAcuguga..	5	1	HA1
...........................acucacucagccUggcuguga..	3	1	HA1
...........................acucacucagccgUgcuguga..	3	1	HA1
...........................acucaUucagccgggcuguga..	2	1	HA1
...........................acucacGcagccgggcuguga..	1	1	HA1
...........................acucacucagccgggcugugU..	80	1	HA1
...........................acucacucagccgggcugAga..	1	1	HA1
...........................acucacuUagccgggcuguga..	1	1	HA1
...........................acucacucCgccgggcuguga..	1	1	HA1
...........................acucacucaAccgggcuguga..	2	1	HA1
...........................acucacucagccggCcuguga..	1	1	HA1
...........................acucacucUgccgggcuguga..	2	1	HA1
...........................acucacucagccgggcugugG..	7	1	HA1
...........................acucacucagccgggcCguga..	2	1	HA1
...........................acucacucagccgggcAguga..	1	1	HA1
...........................acucacucagcGgggcuguga..	2	1	HA1
...........................acucacucagccgggcuguAa..	6	1	HA1
...........................acucacucGgccgggcuguga..	2	1	HA1
...........................acucacucagccAggcuguga..	2	1	HA1
...........................acucacCcagccgggcuguga..	3	1	HA1
...........................aUucacucagccgggcuguga..	3	1	HA1
...........................acucacucagccgggcuguga..	4345	0	HA1
...........................acuAacucagccgggcuguga..	3	1	HA1
...........................acuUacucagccgggcuguga..	1	1	HA1
...........................acucacucagcUgggcuguga..	1	1	HA1
...........................acCcacucagccgggcuguga..	1	1	HA1
...........................acucacucagccgggcugugau...	355	0	HA1
...........................acucacucagccgggcugugUu...	6	1	HA1
...........................acucaUucagccgggcugugau...	1	1	HA1
...........................acucacucagccgggcugugaA...	76	1	HA1
...........................acucacucagccAggcugugau...	1	1	HA1
...........................acucacucagGcgggcugugau...	1	1	HA1
...........................acucacucagccgggcugugaAg..	7	1	HA1
...........................acucacucagUcgggcugugaug..	2	1	HA1
...........................acucacucagccgggcugugauA..	118	1	HA1
...........................acucacucagccgggcugugaug..	728	0	HA1
...........................acucacucagccgggcugugUug..	2	1	HA1
...........................acucacucagccgggcugugGug..	5	1	HA1
...........................acucacucagccAggcugugaug..	1	1	HA1
...........................acucacucagccgggcugugauU..	158	1	HA1
...........................acucGcucagccgggcugugaug..	1	1	HA1
...........................acucacucagccgggcugugaCg..	1	1	HA1
...........................acucacucagcUgggcugugaug..	1	1	HA1
...........................aGucacucagccgggcugugaug..	1	1	HA1
...........................acucacucagccgggcugugCug..	4	1	HA1
...........................acucacucagccgggcugugaGg..	1	1	HA1
...........................acucacucagccgggcCgugaug..	1	1	HA1
...........................acucacucagccgggcugugauC..	6	1	HA1
...........................acucacucagccggAcugugaug..	2	1	HA1
...........................acucacucagccgggcugugauUu...	6	1	HA1
...........................acucacucagccgggcugugaugA...	15	1	HA1
...........................acucacucagccgggcugugaugu...	8	0	HA1
...........................acucacucagccgggcugugauguc..	6	0	HA1
...........................acucacucagccgggcugugauguU..	14	1	HA1

```
                          Star                                        Mature
cccuguggaaguccuguucuauacuaaacucacucagccgggcugugauguccacaccuaaguuaaaucaucacaaccuccgugagugagugacguauacaggaccaauc
...........................acucacucagccgggcugugauguA..........................................................   2      1     HA1
...........................acucacucagccgggcugugaAguc..........................................................   2      1     HA1
...........................acucacucagccgggcugugaugucc.........................................................   1      0     HA1
...........................acucacucagccgggcugugaugucca........................................................   2      0     HA1
............................cuUacucagccgggcuguga..............................................................   1      1     HA1
............................Aucacucagccgggcuguga..............................................................   1      1     HA1
............................cucacucagccgggcuguga..............................................................   6      0     HA1
............................cucacucagccgggcugugau.............................................................   5      0     HA1
............................cucacucagccgggcugugauU............................................................   3      1     HA1
............................cucacucagccgggcugugauA............................................................   2      1     HA1
.............................ucacucagccgggcuguga..............................................................   3      0     HA1
.............................ucacucagccgggcugugau.............................................................   2      0     HA1
.............................ucacucagccgggcugugauU............................................................   3      1     HA1
.............................ucacucagccgggcugugaug............................................................   6      0     HA1
..............................cacucagccgggcuguga..............................................................   9      0     HA1
..............................cacucagccgggcugugaug............................................................   7      0     HA1
..............................cacucagccgggcugugauU............................................................   2      1     HA1
..............................cacucagccgggcugugauguU..........................................................   1      1     HA1
...............................acucagccgggcugugaug............................................................   3      0     HA1
................................cucagccgggcugugaug............................................................   1      0     HA1
.................................ucagccgggcugugauguccac.......................................................   1      0     HA1
................................................Aguccacaccuaaguuaaaucau.......................................   1      1     HA1
................................................uguccacaccuaaguuaaaucau.......................................   2      0     HA1
................................................uguccacaccuaaguuaaaucauc......................................   20     0     HA1
................................................uguAcacaccuaaguuaaaucauc......................................   1      1     HA1
..................................................uccacaccuaaguuaaauc.........................................   2      0     HA1
..................................................uccacaccuaaguuaaauca........................................   20     0     HA1
..................................................uccacaccuaaguuaaaucau.......................................   2      0     HA1
....................................................................Gaucacaaccuccgugagugagu...................   1      1     HA1
....................................................................Uaucacaaccuccgugagugagu...................   1      1     HA1
....................................................................Aaucacaaccuccgugagugagu...................   2      1     HA1
....................................................................Gaucacaaccuccgugagugagug..................   1      1     HA1
.....................................................................Uucacaaccuccgugagugag....................   1      1     HA1
.....................................................................Uucacaaccuccgugagugagu...................   59     1     HA1
.....................................................................Cucacaaccuccgugagugagu...................   1      1     HA1
.....................................................................aucacaaccuccgugagugagu...................   4      0     HA1
.....................................................................Uucacaaccuccgugagugagug..................   3      1     HA1
......................................................................uGacaaccuccgugagug......................   1      1     HA1
......................................................................ucacGaccuccgugagug......................   1      1     HA1
......................................................................ucacaaccuccgugagAg......................   2      1     HA1
......................................................................ucacaaccuccgugaguU......................   1      1     HA1
......................................................................ucacaaccuccgugagGg......................   1      1     HA1
......................................................................ucacaaccucAgugagug......................   1      1     HA1
......................................................................ucacaaccuccgugagug......................   203    0     HA1
......................................................................ucaAaaccuccgugagug......................   1      1     HA1
......................................................................ucacaaGcuccgugaguga.....................   1      1     HA1
......................................................................ucacaaccuccgugaguga.....................   17     0     HA1
......................................................................ucacaaccuccgugagugCg....................   1      1     HA1
......................................................................ucacaaccuccgCgagugag....................   1      1     HA1
......................................................................uAacaaccuccgugagugag....................   1      1     HA1
......................................................................ucacaaccuccgugagugag....................   1010   0     HA1
......................................................................ucacaaccuccgugagugGg....................   2      1     HA1
......................................................................ucacaaccuccgugagugaU....................   1      1     HA1
......................................................................ucacaaccuccgugagAgag....................   2      1     HA1
......................................................................ucacaaccucAgugagugag....................   1      1     HA1
......................................................................Ccacaaccuccgugagugag....................   1      1     HA1
......................................................................ucacaaccuccUugagugag....................   5      1     HA1
......................................................................ucacaaccuccgugCgugag....................   1      1     HA1
......................................................................ucacaaccucUgugagugag....................   1      1     HA1
......................................................................ucGcaaccuccgugagugag....................   1      1     HA1
......................................................................ucacaaccuccgugagugaA....................   3      1     HA1
......................................................................uUacaaccuccgugagugagu...................   15     1     HA1
......................................................................ucCcaaccuccgugagugagu...................   2      1     HA1
......................................................................ucacaaccuccgugCgugagu...................   7      1     HA1
......................................................................ucacaaccuccgugaAugagu...................   2      1     HA1
......................................................................ucacaaccuccgugagugaAu...................   9      1     HA1
......................................................................ucacaaccuccgugagugUgu...................   15     1     HA1
......................................................................ucacaCccuccgugagugagu...................   5      1     HA1
......................................................................ucacaaccuccgGgagugagu...................   12     1     HA1
......................................................................ucacaaGcuccgugagugagu...................   13     1     HA1
```

```
                                    Star                                     Mature
cccuguggaaguccuguucuauacuaaacucacucagccgggcugugauguccacaccuaaguuaaaucaucacaaccuccgugagugagugacguauacaggaccaauc
......................................................................uAacaaccuccgugagugagu...................    10      1    HA1
......................................................................ucacaaccuccgugagugagC...................    57      1    HA1
......................................................................ucacaaccuccgugagugaCu...................    1       1    HA1
......................................................................ucacaaccAccgugagugagu...................    10      1    HA1
......................................................................ucacaaccuccgugagugagG...................    297     1    HA1
......................................................................Ccacaaccuccgugagugagu...................    34      1    HA1
......................................................................ucacaacUuccgugagugagu...................    6       1    HA1
......................................................................ucacaaccuccgugaguUagu...................    1       1    HA1
......................................................................ucacaaccuUcgugagugagu...................    8       1    HA1
......................................................................ucacaaccuccgugUgugagu...................    8       1    HA1
......................................................................ucacaaccucUgugagugagu...................    16      1    HA1
......................................................................ucaUaaccuccgugagugagu...................    13      1    HA1
......................................................................ucacaaccCccgugagugagu...................    14      1    HA1
......................................................................ucacaaccuccgugagugGgu...................    44      1    HA1
......................................................................ucacaaccuccgugagugagu...................    60251   0    HA1
......................................................................ucacaaccuGcgugagugagu...................    2       1    HA1
......................................................................ucacaaccuccgAgagugagu...................    3       1    HA1
......................................................................ucacaaccGccgugagugagu...................    8       1    HA1
......................................................................ucacaaccuccgugagugCgu...................    23      1    HA1
......................................................................ucacCaccuccgugagugagu...................    6       1    HA1
......................................................................ucacaacAuccgugagugagu...................    5       1    HA1
......................................................................ucacaUccuccgugagugagu...................    2       1    HA1
......................................................................Gcacaaccuccgugagugagu...................    55      1    HA1
......................................................................ucGcaaccuccgugagugagu...................    13      1    HA1
......................................................................ucacUaccuccgugagugagu...................    5       1    HA1
......................................................................ucacaaccuccgugGgugagu...................    20      1    HA1
......................................................................ucacaaccuccgugaUugagu...................    22      1    HA1
......................................................................ucacaaccuccgugaguAagu...................    1       1    HA1
......................................................................ucaAaaccuccgugagugagu...................    9       1    HA1
......................................................................ucUcaaccuccgugagugagu...................    1       1    HA1
......................................................................ucacaaccucGgugagugagu...................    3       1    HA1
......................................................................ucacaacGuccgugagugagu...................    2       1    HA1
......................................................................ucacaaUcuccgugagugagu...................    15      1    HA1
......................................................................ucacaaccuccguUagugagu...................    7       1    HA1
......................................................................ucaGaaccuccgugagugagu...................    6       1    HA1
......................................................................ucacGaccuccgugagugagu...................    6       1    HA1
......................................................................ucacaaccuccAugagugagu...................    8       1    HA1
......................................................................ucacaaccuccgugagugagA...................    1617    1    HA1
......................................................................ucacaaccuccgugagCgagu...................    7       1    HA1
......................................................................ucacaaAcuccgugagugagu...................    11      1    HA1
......................................................................ucacaaccuccUugagugagu...................    17      1    HA1
......................................................................ucacaaccuccgugagAgagu...................    11      1    HA1
......................................................................ucacaaccuccguCagugagu...................    5       1    HA1
......................................................................ucacaaccuccgCgagugagu...................    25      1    HA1
......................................................................ucacaaccuccgugagugaUu...................    3       1    HA1
......................................................................ucacaaccucAgugagugagu...................    1       1    HA1
......................................................................ucacaaccuAcgugagugagu...................    10      1    HA1
......................................................................ucacaaccuccgugagGgagu...................    1       1    HA1
......................................................................ucacaaccuccCugagugagu...................    3       1    HA1
......................................................................ucacaaccuccgugaCugagu...................    5       1    HA1
......................................................................Acacaaccuccgugagugagu...................    13      1    HA1
......................................................................ucacaGccuccgugagugagu...................    7       1    HA1
......................................................................uGacaaccuccgugagugagu...................    11      1    HA1
......................................................................ucacaaccuccgugCgugagug..................    2       1    HA1
......................................................................ucacaaccuccgugUgugagug..................    1       1    HA1
......................................................................ucaUaaccuccgugagugagug..................    1       1    HA1
......................................................................ucacaaccuccCugagugagug..................    1       1    HA1
......................................................................ucacaaccuccgugagugaguC..................    4       1    HA1
......................................................................ucacaaGcuccgugagugagug..................    1       1    HA1
......................................................................ucacaaccuccgugagugCgug..................    1       1    HA1
......................................................................ucacaaccAccgugagugagug..................    2       1    HA1
......................................................................uAacaaccuccgugagugagug..................    1       1    HA1
......................................................................ucacaacUuccgugagugagug..................    2       1    HA1
......................................................................ucacaaccuccguUagugagug..................    1       1    HA1
......................................................................ucacaaccuccgugagugagug..................    2974    0    HA1
......................................................................ucacaaUcuccgugagugagug..................    2       1    HA1
......................................................................ucacaaccuccgugagugaguU..................    186     1    HA1
......................................................................ucacaaccuccgCgagugagug..................    3       1    HA1
......................................................................ucacaCccuccgugagugagug..................    2       1    HA1
......................................................................ucacaaccuAcgugagugagug..................    1       1    HA1
```

```
                           Star                                        Mature
cccuguggaaguccuguucuauacuaaacucacucagccgggcugugauguccacaccuaaguuaaaucaucacaaccuccgugagugagugacguauacaggaccaauc
......................................................................ucacaaccuccgugagugagCg..................   1     1     HA1
......................................................................ucacaaccuccgugaCugagug..................   1     1     HA1
......................................................................ucacaaccCccgugagugagug..................   1     1     HA1
......................................................................ucacaaccuccgugagugaguA..................   70    1     HA1
......................................................................ucacaaccuccgugagCgagug..................   1     1     HA1
......................................................................Ccacaaccuccgugagugagug..................   4     1     HA1
......................................................................Gcacaaccuccgugagugagug..................   3     1     HA1
......................................................................ucacaaccuccgAgagugagug..................   1     1     HA1
......................................................................ucacaaccuccgugagugGgug..................   1     1     HA1
......................................................................ucacaaccuccgugagugagGg..................   1     1     HA1
......................................................................ucGcaaccuccgugagugagug..................   2     1     HA1
......................................................................ucacaaccuccgugGgugagug..................   4     1     HA1
......................................................................ucacaaccuccgugagugaUug..................   1     1     HA1
......................................................................ucacaaccuccgugGgugaguga.................   1     1     HA1
......................................................................Acacaaccuccgugagugaguga.................   1     1     HA1
......................................................................ucacaaccuccgugagugagugG.................   1     1     HA1
......................................................................ucacaaccuccgugagugagugU.................   72    1     HA1
......................................................................ucaUaaccuccgugagugaguga.................   1     1     HA1
......................................................................ucacaaccuccgugagugaguga.................   306   0     HA1
......................................................................ucacGaccuccgugagugaguga.................   1     1     HA1
......................................................................ucacaaccuccgugagugaguAa.................   7     1     HA1
......................................................................ucacaaccuccgugagugagugaA................   8     1     HA1
......................................................................ucacaaccuccgugagugagugaU................   1     1     HA1
......................................................................ucacaaccuccgugagugagugCc................   1     1     HA1
......................................................................ucacaaccuccgugagugagugaAg...............   1     1     HA1
......................................................................ucacaaccuccgugagugagugacU...............   1     1     HA1
.......................................................................cacaaccuccgugagugaA....................   1     1     HA1
.......................................................................cacaaccuccgugagugag....................   4     0     HA1
.......................................................................cacaaccuccUugagugag....................   1     1     HA1
.......................................................................cacaaccuccgugagugagu...................   150   0     HA1
.......................................................................cacaaccuccgCgagugagu...................   1     1     HA1
.......................................................................cacaaccuccgugagugagA...................   4     1     HA1
.......................................................................Uacaaccuccgugagugagu...................   1     1     HA1
.......................................................................cacaaccuccgugagGgagu...................   1     1     HA1
.......................................................................Aacaaccuccgugagugagu...................   1     1     HA1
.......................................................................cacaaccuccgugagugGgu...................   3     1     HA1
.......................................................................cacaaccuccgugagugaguA..................   1     1     HA1
.......................................................................cacaaccuccgugagugagug..................   26    0     HA1
.......................................................................cacaaccuccgugagugaguU..................   1     1     HA1
.......................................................................cacaaccuccgugagugagugC.................   1     1     HA1
.......................................................................cacaaccuccgugagugaguga.................   39    0     HA1
.......................................................................cacaaccuccgugagugagugU.................   14    1     HA1
.......................................................................cacaaccuccgugagugaguAa.................   1     1     HA1
.......................................................................cacaaccuccgugagugagugaA................   10    1     HA1
........................................................................acaaccuccgugagugag....................   2     0     HA1
........................................................................Ucaaccuccgugagugagu...................   1     1     HA1
........................................................................acaaccuccgugagugGgu...................   3     1     HA1
........................................................................acaaccuccgugagugagu...................   10    0     HA1
........................................................................acaaccuccgugagugaAu...................   1     1     HA1
........................................................................acaaccuccgugagugagG...................   1     1     HA1
........................................................................acaaccuccgugagugaguA..................   1     1     HA1
........................................................................acaaccuccgugagugagug..................   1     0     HA1
........................................................................acaaccucAgugagugaguga.................   1     1     HA1
........................................................................acaaccuccgugagugaguga.................   97    0     HA1
........................................................................acaaccuccgugagugagugU.................   4     1     HA1
........................................................................acaaccuccgugagugagugaA................   8     1     HA1
.........................................................................caaccuccgugagugagu...................   9     0     HA1
.........................................................................caaccuccgugagugagug..................   1     0     HA1
..........................................................................aaccucGgugagugaguga.................   1     1     HA1
......................................................................................ugagugacguauacaggaccaa..   1     0     HA1
......................................................................................ugagugacguauacaggaccaUu.   1     1     HA1

..........................aacucacucagccgggcugugaug............................................................   1     0     HS1
...........................acucacucagccgggcugug...............................................................   2     0     HS1
...........................acucacucagccgggcuguga..............................................................   37    0     HS1
...........................acucacucagccgggcugugau.............................................................   18    0     HS1
...........................acucacucagcUgggcugugau.............................................................   1     1     HS1
...........................aGucacucagccgggcugugaug............................................................   1     1     HS1
...........................acucacCcagccgggcugugaug............................................................   4     1     HS1
...........................Gcucacucagccgggcugugaug............................................................   3     1     HS1
```

Star Mature

cccuguggaaguccuguucuauacuaaacucacucagccgggcugugauguccacaccuaaguuaaaucaucacaaccuccgugagugagugacguauacaggaccaauc			
...........................acucacucagccgggcugugUug..	1	1	HS1
...........................acucacucagccggAcugugaug..	19	1	HS1
...........................acucacucagccgggcugugauU..	59	1	HS1
...........................Ccucacucagccgggcugugaug..	1	1	HS1
...........................acucacucagccgggcugugaug..	8358	0	HS1
...........................aUucacucagccgggcugugaug..	4	1	HS1
...........................acucGcucagccgggcugugaug..	4	1	HS1
...........................acucacucagccUggcugugaug..	1	1	HS1
...........................acucacucagUcgggcugugaug..	26	1	HS1
...........................acucacucagccgggUugugaug..	2	1	HS1
...........................Ucucacucagccgggcugugaug..	2	1	HS1
...........................acucaUucagccgggcugugaug..	3	1	HS1
...........................acucacucagccgggcugugaAg..	6	1	HS1
...........................acucacucagccgggcugugaCg..	1	1	HS1
...........................acucacucagccgggcugGgaug..	1	1	HS1
...........................acuUacucagccgggcugugaug..	5	1	HS1
...........................acucacucagccAggcugugaug..	1	1	HS1
...........................acucacucagccgggcuAugaug..	1	1	HS1
...........................acucacucagccgggcugugaGg..	2	1	HS1
...........................acucacucagccgggcugugGug..	2	1	HS1
...........................acucacucagcUgggcugugaug..	2	1	HS1
...........................acucacucagccgggcuguUaug..	1	1	HS1
...........................acGcacucagccgggcugugaug..	2	1	HS1
...........................acucacucagccgggcCgugaug..	4	1	HS1
...........................acCcacucagccgggcugugaug..	2	1	HS1
...........................acucacucaAccgggcugugaug..	4	1	HS1
...........................acucacucagccgggcuguAaug..	1	1	HS1
...........................acucacucagGcgggcugugaug..	3	1	HS1
...........................acucacucagccgggcugugauA..	8	1	HS1
...........................acucacucagccgggcugugaugA...	14	1	HS1
............................cucacucagccgggcuguga..	2	0	HS1
............................cucacCcagccgggcuguga..	5	1	HS1
............................cucacucagccgggcugugau...	2	0	HS1
............................cucacucagccgggcugugauU..	10	1	HS1
............................cucGcucagccgggcugugaug..	1	1	HS1
............................cucacucagccgggcugugaCg..	1	1	HS1
............................cucacucagUcgggcugugaug..	1	1	HS1
............................cucacucagccgggcugugaGg..	1	1	HS1
............................cucacucagccgggcugugaug..	648	0	HS1
.............................ucacucagccgggcugugaug..	19	0	HS1
..............................cacucagccgggcugugaug..	4	0	HS1
...............................acucagccgggcugugaug..	9	0	HS1
....uccacaccuaaguuaaauca..	9	0	HS1
....Cucacaaccuccgugagugagug..................	6	1	HS1
....ucacaaccuccgugagugagC...................	56	1	HS1
....ucacaaccuccgugGgugagu...................	2	1	HS1
....ucacaaccuccgugagCgagu...................	2	1	HS1
....ucacaaccCccgugagugagu...................	1	1	HS1
....ucacaaccuccgugagugagu...................	1282	0	HS1
....ucacaaccuccgugagugaUu...................	1	1	HS1
....Ccacaaccuccgugagugagu...................	2	1	HS1
....ucacGaccuccgugagugagu...................	1	1	HS1
....Acacaaccuccgugagugagu...................	1	1	HS1
....ucacaaccuccgCgagugagu...................	1	1	HS1
....ucacaacUuccgugagugagug..................	3	1	HS1
....ucacaaccuccgugagugaguA..................	1	1	HS1
....ucacaaccuccgugagugagug..................	352	0	HS1
....ucacaaccuccgugagugagugU.................	5	1	HS1
....ucacaaccuccgugagugaguga.................	33	0	HS1
....ucacaaccuccgugagugagugacA...............	5	1	HS1
....cacaaccuccgugagugagC...................	3	1	HS1
....cacaaccuccgugagugagu...................	100	0	HS1
....cacaaccuccgugagugagug..................	31	0	HS1
....cacaaccuccgugagugagugU.................	2	1	HS1
....cacaaccuccgugagugaguga.................	4	0	HS1
....acaaccuccgugagugagu...................	8	0	HS1
..........................aacucacucagccgggcugu..	1	0	HS6
..........................Uacucacucagccgggcuguga..	1	1	HS6
...........................acucacucagccgggcug...	1	0	HS6

```
                            Star                                        Mature
cccuguggaaguccuguucuauacuaaacucacucagccgggcugugauguccacaccuaaguuaaaucaucacaaccuccgugagugagugacguauacaggaccaauc
...........................acucacucagccgggcugu................................................................ 18   0  HS6
...........................acucacucagccgggcugug............................................................... 298  0  HS6
...........................acucacucagccgggcugGg............................................................... 1    1  HS6
...........................acucacucaCccgggcugug............................................................... 1    1  HS6
...........................acucacucagccgggcuguU............................................................... 3    1  HS6
...........................acucUcucagccgggcugug............................................................... 1    1  HS6
...........................acucacucagUcgggcugug............................................................... 1    1  HS6
...........................acucacucagccgggcuguA............................................................... 1    1  HS6
...........................acucacucagccgggcugugU.............................................................. 11   1  HS6
...........................acucacucagccgggcugugG.............................................................. 4    1  HS6
...........................acucacucagUcgggcuguga.............................................................. 1    1  HS6
...........................acucacucagccgggcuAuga.............................................................. 1    1  HS6
...........................Ucucacucagccgggcuguga.............................................................. 1    1  HS6
...........................acucacucagccgggcAguga.............................................................. 1    1  HS6
...........................acuUacucagccgggcuguga.............................................................. 1    1  HS6
...........................acucacucagccgggcuguga.............................................................. 756  0  HS6
...........................acucacucagccgggcuguUau............................................................. 1    1  HS6
...........................acucacuUagccgggcugugau............................................................. 1    1  HS6
...........................acucacucagccgggcugugau............................................................. 57   0  HS6
...........................acucacucagccgggcugugUu............................................................. 2    1  HS6
...........................acucacucagccgggcugugaA............................................................. 2    1  HS6
...........................acucacucagccgggcGgugaug............................................................ 1    1  HS6
...........................acucacucagccgggcugugauA............................................................ 3    1  HS6
...........................acucacucagccgggcuUugaug............................................................ 1    1  HS6
...........................acucacucagccgggcugugaGg............................................................ 3    1  HS6
...........................acucacucagccgggcugugauU............................................................ 77   1  HS6
...........................acucacucagccgggcuguAaug............................................................ 1    1  HS6
...........................acuUacucagccgggcugugaug............................................................ 2    1  HS6
...........................acucacucagccggCcugugaug............................................................ 1    1  HS6
...........................acucacucagccgggcugugaug............................................................ 323  0  HS6
...........................acucacuGagccgggcugugaug............................................................ 1    1  HS6
...........................acucacucagccgggcugugauUu........................................................... 1    1  HS6
...........................acucacucagccgggcugugaugA........................................................... 5    1  HS6
...........................acucacucagccgggcugugaugu........................................................... 3    0  HS6
...........................acucacucagccgggcugugauguA.......................................................... 1    1  HS6
...........................acucacucagccgggcugugauguU.......................................................... 9    1  HS6
............................cucacucagccgggcugugauU............................................................ 1    1  HS6
............................cucacucagccgggcugugaug............................................................ 1    0  HS6
.............................ucacucagccgggcugugau............................................................. 1    0  HS6
.............................ucacucagccgggcugugaug............................................................ 3    0  HS6
.............................ucacucagccgggcugugaugu........................................................... 1    0  HS6
..............................cacucagccgggcuguga.............................................................. 1    0  HS6
..............................cacucagccgggcugugaug............................................................ 6    0  HS6
.................................................guccacaccuaaguuaaaucau....................................... 1    0  HS6
..................................................uccacaccuaaguuaaauc......................................... 2    0  HS6
..................................................uccacaccuaaguuaaaucau....................................... 1    0  HS6
....................................................................Gaucacaaccuccgugagugagu................... 1    1  HS6
.....................................................................Uucacaaccuccgugagu....................... 2    1  HS6
.....................................................................aucacaaccuccgugagugag.................... 1    0  HS6
.....................................................................aucacaaccuccgugagugagu................... 1    0  HS6
.....................................................................Uucacaaccuccgugagugagu................... 34   1  HS6
.....................................................................Uucacaaccuccgugagugagug.................. 4    1  HS6
......................................................................ucacaaccuccgugagug...................... 34   0  HS6
......................................................................ucacaaccuccgugaguga..................... 1    0  HS6
......................................................................ucacUaccuccgugagugag.................... 1    1  HS6
......................................................................ucacaaccuccgugagugaU.................... 1    1  HS6
......................................................................uUacaaccuccgugagugag.................... 1    1  HS6
......................................................................ucacaaccuccguAagugag.................... 1    1  HS6
......................................................................ucacaaccuccgugaUugag.................... 1    1  HS6
......................................................................ucacaaccuccgugagugag.................... 206  0  HS6
......................................................................ucacaaccucAgugagugag.................... 1    1  HS6
......................................................................Gcacaaccuccgugagugagu................... 3    1  HS6
......................................................................ucacaaccuccgugagugaAu................... 2    1  HS6
......................................................................ucacaaccuccAugagugagu................... 4    1  HS6
......................................................................ucCcaaccuccgugagugagu................... 3    1  HS6
......................................................................ucacaaccucUgugagugagu................... 7    1  HS6
......................................................................ucacaaccuccgugagugCgu................... 6    1  HS6
......................................................................ucacaaccuccCugagugagu................... 4    1  HS6
......................................................................ucacaaccuccgugGgugagu................... 2    1  HS6
......................................................................ucGcaaccuccgugagugagu................... 3    1  HS6
```

Star Mature

```
cccuguggaaguccuguucuauacuaaacucacucagccgggcugugauguccacaccuaaguuaaaucaucacaaccuccgugagugagugacguauacaggaccaauc
......................................................................ucacaaccuccguCagugagu...................   1      1   HS6
......................................................................Ccacaaccuccgugagugagu...................   6      1   HS6
......................................................................ucacaacUuccgugagugagu...................   6      1   HS6
......................................................................ucacaaccuUcgugagugagu...................   7      1   HS6
......................................................................ucacaaccuccgugaguUagu...................   2      1   HS6
......................................................................ucacaaccuccguAagugagu...................   1      1   HS6
......................................................................ucacaaccCccgugagugagu...................   10     1   HS6
......................................................................ucacaaccuccgugaCugagu...................   1      1   HS6
......................................................................ucacaaccuccgugaUugagu...................   1      1   HS6
......................................................................ucacaaccuccgAgagugagu...................   1      1   HS6
......................................................................ucacaaccuccguUagugagu...................   6      1   HS6
......................................................................ucacaaccuccgugagCgagu...................   1      1   HS6
......................................................................ucacaaccuccgugagugGgu...................   3      1   HS6
......................................................................ucaGaaccuccgugagugagu...................   2      1   HS6
......................................................................ucacaaccuccgugagugagA...................   77     1   HS6
......................................................................ucacaaccuccgugaAugagu...................   1      1   HS6
......................................................................ucacaaccuccgugCgugagu...................   1      1   HS6
......................................................................ucacaaccucGgugagugagu...................   2      1   HS6
......................................................................ucacaaccuccgGgagugagu...................   2      1   HS6
......................................................................ucacaGccuccgugagugagu...................   5      1   HS6
......................................................................ucacaaccuccgugagugagC...................   22     1   HS6
......................................................................ucacaaccuccUugagugagu...................   14     1   HS6
......................................................................ucacaCccuccgugagugagu...................   3      1   HS6
......................................................................uUacaaccuccgugagugagu...................   4      1   HS6
......................................................................ucacaaccuccgugagugagG...................   772    1   HS6
......................................................................ucacaaUcuccgugagugagu...................   7      1   HS6
......................................................................ucacaaccuccgugaguCagu...................   2      1   HS6
......................................................................ucacaaccuccgugagGgagu...................   1      1   HS6
......................................................................ucacaaccuccgugagugUgu...................   10     1   HS6
......................................................................ucacaaccAccgugagugagu...................   1      1   HS6
......................................................................ucacaaccuccgCgagugagu...................   7      1   HS6
......................................................................ucacaaccuccgugUgugagu...................   3      1   HS6
......................................................................uAacaaccuccgugagugagu...................   2      1   HS6
......................................................................uGacaaccuccgugagugagu...................   1      1   HS6
......................................................................ucaUaaccuccgugagugagu...................   16     1   HS6
......................................................................ucacUaccuccgugagugagu...................   13     1   HS6
......................................................................ucacGaccuccgugagugagu...................   5      1   HS6
......................................................................ucacaaccuccgugaguAagu...................   2      1   HS6
......................................................................ucUcaaccuccgugagugagu...................   28     1   HS6
......................................................................ucacaaccuccgugagugagu...................   28840  0   HS6
......................................................................ucacaaccuccUugagugagug..................   1      1   HS6
......................................................................ucacaaccuccgugaguUagug..................   1      1   HS6
......................................................................ucacaaccuccgugaCugagug..................   1      1   HS6
......................................................................ucacaaccuccgCgagugagug..................   1      1   HS6
......................................................................ucacaaccuccgugagugagug..................   1988   0   HS6
......................................................................ucacaaccuccgugagugaguA..................   20     1   HS6
......................................................................ucaUaaccuccgugagugagug..................   1      1   HS6
......................................................................ucacaaccuccgugagugUgug..................   1      1   HS6
......................................................................ucacaaccuccgugagugagCg..................   1      1   HS6
......................................................................ucacaaccuccgugagugaguU..................   22     1   HS6
......................................................................ucacaaccuccgugagugaUug..................   1      1   HS6
......................................................................ucUcaaccuccgugagugagug..................   3      1   HS6
......................................................................ucacaaccCccgugagugagug..................   1      1   HS6
......................................................................ucacaacUuccgugagugagug..................   1      1   HS6
......................................................................ucacaaccuccAugagugagug..................   1      1   HS6
......................................................................ucacaaccuccgugagugaguC..................   1      1   HS6
......................................................................ucacaaccuccgugagugagGg..................   5      1   HS6
......................................................................ucacaaccuccgugagugaAug..................   1      1   HS6
......................................................................ucacaaccuccgugagugagugC.................   2      1   HS6
......................................................................ucacaaccuccgugagugaguga.................   29     0   HS6
......................................................................ucacaaccuccgugaAugaguga.................   2      1   HS6
......................................................................ucacaaccuccgugagugagugU.................   16     1   HS6
......................................................................ucacaaccuccgugagugCguga.................   1      1   HS6
......................................................................ucacaaccuccgugagugagugG.................   1      1   HS6
......................................................................ucacaaccuccgugagugagugaA................   1      1   HS6
......................................................................ucacaaccuccgugagugagugaU................   1      1   HS6
......................................................................ucacaaccuccgugagugagugacU...............   1      1   HS6
.......................................................................cacaaccuccgugagugagG...................   5      1   HS6
.......................................................................cacaaccuccgugagugagu...................   120    0   HS6
.......................................................................cacaaccuccgugagugagug..................   13     0   HS6
```

Star Mature

```
cccuguggaaguccuguucuauacuaaacucacucagccgggcugugauguccacaccuaaguuaaaucaucacaaccuccgugagugagugacguauacaggaccaauc
.......................................................................cacaaccuccgugagugaguga.................	9	0	HS6
.......................................................................cacaaccuccgugagugagugC.................	1	1	HS6
.......................................................................cacaaccuccgugagugagugaU................	2	1	HS6
........................................................................acaaccuccgugagugagu...................	6	0	HS6
........................................................................acaaccuccgugagugGgu...................	1	1	HS6
........................................................................acaaccuccCugagugagu...................	1	1	HS6
........................................................................Ucaaccuccgugagugagu...................	4	1	HS6
........................................................................acaaccuccgugagugagug..................	2	0	HS6
........................................................................acaaccuccgugagugaguga.................	3	0	HS6
.........................................................................caaccuccgugagugagu...................	21	0	HS6
.........................................................................caaccuccgugagugagug..................	8	0	HS6

...........................acucacucagccgggcugu................................................................	1	0	EGG
...........................acucacucagccgggcugug...............................................................	11	0	EGG
...........................acucacucagccgggcuguga..............................................................	36	0	EGG
...........................acucacucagccgggcugugU..............................................................	2	1	EGG
...........................acucacucagccgggcugugau.............................................................	8	0	EGG
...........................acucacucagccgggcugugaug............................................................	4	0	EGG
...........................acucacucagccgggcugugauU............................................................	1	1	EGG
.................................ucagccgggcugugaugucc.........................................................	1	0	EGG
....................................................................cGucacaaccuccgugagugagu...................	1	1	EGG
.....................................................................Uucacaaccuccgugagugagu...................	20	1	EGG
.....................................................................Gucacaaccuccgugagugagu...................	1	1	EGG
.....................................................................Uucacaaccuccgugagugagug..................	1	1	EGG
......................................................................ucacaaccuccgugagug......................	2	0	EGG
......................................................................ucacaaccuccgugaguga.....................	3	0	EGG
......................................................................ucacaaccuccgCgagugag....................	1	1	EGG
......................................................................ucacaaccuccgugagugag....................	47	0	EGG
......................................................................Gcacaaccuccgugagugagu...................	1	1	EGG
......................................................................ucacaaccuccgugagugagu...................	6760	0	EGG
......................................................................ucacaaccuccgugagugagG...................	19	1	EGG
......................................................................Acacaaccuccgugagugagu...................	1	1	EGG
......................................................................ucacaaccuccgugagugGgu...................	1	1	EGG
......................................................................ucacaaccuccAugagugagu...................	1	1	EGG
......................................................................ucacaaccuccgugagugagA...................	52	1	EGG
......................................................................ucacUaccuccgugagugagu...................	7	1	EGG
......................................................................ucacaaccuccgAgagugagu...................	1	1	EGG
......................................................................ucacaCccuccgugagugagu...................	1	1	EGG
......................................................................ucacaacGuccgugagugagu...................	2	1	EGG
......................................................................ucacaaccuccgugaUugagu...................	1	1	EGG
......................................................................ucacaaccuccgugaCugagu...................	2	1	EGG
......................................................................ucacCaccuccgugagugagu...................	2	1	EGG
......................................................................ucacaaccuccgugaguAagu...................	1	1	EGG
......................................................................ucacaaccuUcgugagugagu...................	2	1	EGG
......................................................................ucacaaccuAcgugagugagu...................	1	1	EGG
......................................................................ucacaaccuccUugagugagu...................	5	1	EGG
......................................................................ucacaaccuccgugagugaAu...................	1	1	EGG
......................................................................ucacaaccuccgugUgugagu...................	9	1	EGG
......................................................................ucacGaccuccgugagugagu...................	4	1	EGG
......................................................................ucacaaccuccgugGgugagu...................	2	1	EGG
......................................................................ucacaacUuccgugagugagu...................	2	1	EGG
......................................................................ucacaaccCccgugagugagu...................	2	1	EGG
......................................................................ucaUaaccuccgugagugagu...................	1	1	EGG
......................................................................uUacaaccuccgugagugagu...................	1	1	EGG
......................................................................uGacaaccuccgugagugagu...................	1	1	EGG
......................................................................ucaAaaccuccgugagugagu...................	1	1	EGG
......................................................................ucacaaccuccgugagugaCu...................	1	1	EGG
......................................................................ucacaaccuccgCgagugagu...................	3	1	EGG
......................................................................ucacaaccucUgugagugagu...................	1	1	EGG
......................................................................ucacaaccuccgugagugagC...................	25	1	EGG
......................................................................ucacaaccuccgugagugUgu...................	3	1	EGG
......................................................................ucacaaccuccgugagugaguC..................	1	1	EGG
......................................................................ucacaaccuccgugGgugagug..................	1	1	EGG
......................................................................ucacaaccuccgugagugaguA..................	7	1	EGG
......................................................................ucacaaccuccgugagugagug..................	120	0	EGG
......................................................................ucacaaccuccgugaCugagug..................	1	1	EGG
......................................................................ucacaaccuccgugagugaguU..................	6	1	EGG
......................................................................ucacaaccuccgugagugagugU.................	1	1	EGG
......................................................................ucacaaccuccgugagugaguga.................	2	0	EGG
......................................................................ucacaaccuccgugagugagugCc................	1	1	EGG
```

```
                          Star                                        Mature
cccuguggaaguccuguucuauacuaaacucacucagccgggcugugauguccacaccuaaguuaaaucaucacaaccuccgugagugagugacguauacaggaccaauc
.......................................................................cacaaccuccgugagugag....................    2     0    EGG
.......................................................................cacaaccuccgugaguUagu...................    1     1    EGG
.......................................................................Uacaaccuccgugagugagu...................    2     1    EGG
.......................................................................cacaaccuccgugagugagu...................    58    0    EGG
........................................................................Ucaaccuccgugagugagu...................    2     1    EGG
........................................................................acaaccuccgugagugagu...................    4     0    EGG
........................................................................acaaccuccgugagugagug..................    2     0    EGG
........................................................................acaaccuccgugagugaguga.................    3     0    EGG
.........................................................................caaccuccgugagugagC...................    1     1    EGG
.........................................................................caaccuccgugagugagu...................    8     0    EGG

...uguggaaguccuguucuaua.......................................................................................    1     0    HS4
.........................aaacucacucagccgggcugu................................................................    2     0    HS4
..........................aacucacucagccgggcuguga..............................................................    1     0    HS4
..........................Uacucacucagccgggcuguga..............................................................    1     1    HS4
...........................acucacucagccgggcugu................................................................    20    0    HS4
...........................acucacucagccgggcugug...............................................................    126   0    HS4
...........................acucacucagccgggcuguU...............................................................    3     1    HS4
...........................acucacucagccgAgcuguga..............................................................    1     1    HS4
...........................acucacGcagccgggcuguga..............................................................    4     1    HS4
...........................acucacucagccgggcugCga..............................................................    1     1    HS4
...........................acucacucagccggAcuguga..............................................................    3     1    HS4
...........................acCcacucagccgggcuguga..............................................................    2     1    HS4
...........................acucaUucagccgggcuguga..............................................................    2     1    HS4
...........................acucacucagccgggcugugU..............................................................    16    1    HS4
...........................acucacucagccgggcuCuga..............................................................    1     1    HS4
...........................acucacucagccgggUuguga..............................................................    2     1    HS4
...........................aGucacucagccgggcuguga..............................................................    1     1    HS4
...........................acucacCcagccgggcuguga..............................................................    2     1    HS4
...........................acucacucaUccgggcuguga..............................................................    2     1    HS4
...........................acucacucagccgggcugAga..............................................................    1     1    HS4
...........................acucacucagccgggcuAuga..............................................................    1     1    HS4
...........................acuUacucagccgggcuguga..............................................................    2     1    HS4
...........................Ucucacucagccgggcuguga..............................................................    1     1    HS4
...........................acGcacucagccgggcuguga..............................................................    1     1    HS4
...........................acucacucagUcgggcuguga..............................................................    2     1    HS4
...........................acucacucagccgggcuguga..............................................................    2053  0    HS4
...........................acucacucagccggCcuguga..............................................................    1     1    HS4
...........................acucacucagccgggcugugaA.............................................................    13    1    HS4
...........................acucacGcagccgggcugugau.............................................................    1     1    HS4
...........................acucacucagccgggcuCugau.............................................................    2     1    HS4
...........................acucacucagccgggcugugUu.............................................................    4     1    HS4
...........................acucacucagccggAcugugau.............................................................    1     1    HS4
...........................acucacucagccgCgcugugau.............................................................    1     1    HS4
...........................aGucacucagccgggcugugau.............................................................    1     1    HS4
...........................acucacucagccgUgcugugau.............................................................    1     1    HS4
...........................acucacucagccgggcuAugau.............................................................    1     1    HS4
...........................acucacucagccgggUugugau.............................................................    1     1    HS4
...........................acucacucagccgggcugugau.............................................................    307   0    HS4
...........................acucacucagccgggcugugauU............................................................    72    1    HS4
...........................acucacucagccgggcuCugaug............................................................    1     1    HS4
...........................acucacucagccgggcugugaug............................................................    82    0    HS4
...........................acucaUucagccgggcugugaug............................................................    1     1    HS4
...........................acucacucagccgggcugugauA............................................................    14    1    HS4
...........................acucacucagccgggcugugauC............................................................    1     1    HS4
...........................acucacucagccgggcugugauUu...........................................................    3     1    HS4
...........................acucacucagccgggcugugaugA...........................................................    4     1    HS4
...........................acucacucagccgggcugugaugu...........................................................    10    0    HS4
...........................acucacucagccgggcugugauguU..........................................................    8     1    HS4
............................cucacucagccgggcugugau.............................................................    1     0    HS4
............................cucacucagccgggcugugaug............................................................    1     0    HS4
............................cucacucagccgggcugugauU............................................................    1     1    HS4
............................cucacucagccgggcugugauguU..........................................................    1     1    HS4
.............................ucacucagccgggcugugaug............................................................    3     0    HS4
.............................ucacucagccgggcugugaugu...........................................................    3     0    HS4
.................................ucagccgggcugugauguccac.......................................................    1     0    HS4
.................................................guccacaccuaaguuaaaucau.......................................    3     0    HS4
..................................................uccacaccuaaguuaaauc.........................................    5     0    HS4
..................................................uccacaccuaaguuaaauca........................................    25    0    HS4
..................................................uccacaccuaaguuaaaucau.......................................    15    0    HS4
```

Star **Mature**

cccuguggaaguccuguucuauacuaaacucacucagccgggcugugauguccacaccuaaguuaaaucaucacaaccuccgugagugagugacguauacaggaccaauc

....uaaaucaucacaaccuccgugag........................	1	0	HS4
....aucacaaccuccgugagugagu...................	2	0	HS4
....Uucacaaccuccgugagugagug..................	1	1	HS4
....aucacaaccuccgugagugaguU..................	1	1	HS4
....ucacaaccuccgugagug......................	15	0	HS4
....ucacaaccucCugaguga......................	1	1	HS4
....ucacaaccuccgugaguga.....................	4	0	HS4
....ucacaaccuccgugaUugag....................	1	1	HS4
....ucacaaccuccgugagugag....................	119	0	HS4
....Gcacaaccuccgugagugagu...................	2	1	HS4
....ucacaaccAccgugagugagu...................	1	1	HS4
....ucacaaccuUcgugagugagu...................	12	1	HS4
....uUacaaccuccgugagugagu...................	1	1	HS4
....ucacaaccGccgugagugagu...................	1	1	HS4
....ucacaaccuccguCagugagu...................	6	1	HS4
....ucacaaccuccgugagugGgu...................	7	1	HS4
....ucacaacUuccgugagugagu...................	5	1	HS4
....ucacaaccuccgugaguCagu...................	5	1	HS4
....ucaGaaccuccgugagugagu...................	2	1	HS4
....ucacaaccuccgugaguAagu...................	1	1	HS4
....ucacaaccuccgugUgugagu...................	2	1	HS4
....ucacGaccuccgugagugagu...................	5	1	HS4
....ucacaaccuccgugagugUgu...................	3	1	HS4
....ucacaaccuccgugagugagA...................	25	1	HS4
....ucacaaccuccgugaguUagu...................	1	1	HS4
....ucacaaccuccgugagugagu...................	16148	0	HS4
....ucacaaccuccgGgagugagu...................	2	1	HS4
....ucacUaccuccgugagugagu...................	4	1	HS4
....ucacaaccuccgugaAugagu...................	1	1	HS4
....ucacaaccuccgAgagugagu...................	1	1	HS4
....ucacaaccuccgCgagugagu...................	2	1	HS4
....ucacaaccuccCugagugagu...................	9	1	HS4
....Acacaaccuccgugagugagu...................	3	1	HS4
....ucacaaccCccgugagugagu...................	5	1	HS4
....ucacaaccuccgugaCugagu...................	16	1	HS4
....ucaUaaccuccgugagugagu...................	21	1	HS4
....ucacaaccuccgugagugagG...................	2	1	HS4
....ucacaaUcuccgugagugagu...................	8	1	HS4
....ucacaaccuccgugagugaUu...................	1	1	HS4
....ucacCaccuccgugagugagu...................	2	1	HS4
....ucacaaAcuccgugagugagu...................	1	1	HS4
....ucacaaccuAcgugagugagu...................	1	1	HS4
....ucacaaccuccguAagugagu...................	2	1	HS4
....ucacaaccuccgugagugagC...................	10	1	HS4
....ucacaaccucUgugagugagu...................	7	1	HS4
....ucacaaccuccgugaUugagu...................	6	1	HS4
....ucacaaccuccAugagugagu...................	3	1	HS4
....ucacaaccuccguUagugagu...................	10	1	HS4
....ucacaaccuccgugagCgagu...................	1	1	HS4
....ucacaaccucGgugagugagu...................	1	1	HS4
....ucacaaccuccUugagugagu...................	21	1	HS4
....Ccacaaccuccgugagugagu...................	2	1	HS4
....ucacaaGcuccgugagugagu...................	16	1	HS4
....uGacaaccuccgugagugagu...................	1	1	HS4
....ucacaaccuccgugGgugagu...................	4	1	HS4
....ucacaaccuccgugagugaAu...................	2	1	HS4
....ucacaGccuccgugagugagu...................	1	1	HS4
....ucGcaaccuccgugagugagu...................	6	1	HS4
....ucCcaaccuccgugagugagu...................	2	1	HS4
....ucacaaccuccgugagugaguC..................	1	1	HS4
....ucacaaccucUgugagugagug..................	1	1	HS4
....Ccacaaccuccgugagugagug..................	1	1	HS4
....ucCcaaccuccgugagugagug..................	1	1	HS4
....ucacaaccuccgugCgugagug..................	1	1	HS4
....ucacaaccuccgugagugagug..................	1808	0	HS4
....ucacaaAcuccgugagugagug..................	1	1	HS4
....ucacaaccuccgugagugaUug..................	3	1	HS4
....ucacaaccuccgAgagugagug..................	1	1	HS4
....ucacaaccuccUugagugagug..................	3	1	HS4
....ucaUaaccuccgugagugagug..................	1	1	HS4

Star **Mature**

```
cccuguggaaguccuguucuauacuaaacucacucagccgggcugugauguccacaccuaaguuaaaucaucacaaccuccgugagugagugacguauacaggaccaauc
......................................................................ucacaaccuccgugaCugagug..................   2     1   HS4
......................................................................ucacUaccuccgugagugagug..................   1     1   HS4
......................................................................ucacaaccuccgugagugaguU..................   35    1   HS4
......................................................................ucacaacUuccgugagugagug..................   1     1   HS4
......................................................................ucacaaccuccgugaguCagug..................   1     1   HS4
......................................................................ucacaaccuccgugaAugagug..................   1     1   HS4
......................................................................ucacaaccuccguCagugagug..................   4     1   HS4
......................................................................ucacaaccuccgugagugaguA..................   7     1   HS4
......................................................................ucacaaccCccgugagugagug..................   1     1   HS4
......................................................................ucacaaccuccguUagugagug..................   4     1   HS4
......................................................................ucacaaccuAcgugagugagug..................   1     1   HS4
......................................................................ucacaaccuccgugagugaguga.................   17    0   HS4
......................................................................ucacaaccuccguUagugaguga.................   1     1   HS4
......................................................................ucacaaccuccgugagugagugU.................   67    1   HS4
......................................................................ucacaaccuccgugagugagugaU................   1     1   HS4
.......................................................................cacaaccuccgugagugag....................   1     0   HS4
.......................................................................cacaaccuccgugagugagu...................   167   0   HS4
.......................................................................cacaacUuccgugagugagu...................   1     1   HS4
.......................................................................cacaaccuccgugUgugagu...................   1     1   HS4
.......................................................................cacaaccuccgugagugaguU..................   1     1   HS4
.......................................................................cacaaccuccgugagugagug..................   16    0   HS4
.......................................................................cacaaccuccgugagugagugU.................   6     1   HS4
.......................................................................cacaaccuccgugagugaguga.................   22    0   HS4
.......................................................................cacaaccuccgugagugagugaA................   3     1   HS4
........................................................................acaaccuccgugagugagu...................   2     0   HS4
........................................................................Ucaaccuccgugagugagu...................   1     1   HS4
........................................................................acaaccuccCugagugagu...................   2     1   HS4
........................................................................acaaccuccgugagugaguga.................   4     0   HS4
.........................................................................caaccuccgugagugagu...................   2     0   HS4
.........................................................................caaccuccgugagugagug..................   1     0   HS4

...uguggaaguccuguucuauacua....................................................................................   1     0   J17
........................uaaacucacucagccgggcugu................................................................   2     0   J17
..........................aacucacucagccgggcugu................................................................   1     0   J17
..........................aacucacucagccgggcuguga..............................................................   2     0   J17
...........................acucacucagccgggcug.................................................................   1     0   J17
...........................acucacucagccgggcugu................................................................   3     0   J17
...........................acucacucagccgggcuguU...............................................................   1     1   J17
...........................acucacucagccgggcuguA...............................................................   3     1   J17
...........................acucacucagccgggcugug...............................................................   67    0   J17
...........................acucaUucagccgggcuguga..............................................................   1     1   J17
...........................aAucacucagccgggcuguga..............................................................   1     1   J17
...........................acucacucagccggAcuguga..............................................................   3     1   J17
...........................acucacucagUcgggcuguga..............................................................   1     1   J17
...........................acucacucagccgggcugugG..............................................................   1     1   J17
...........................acucacucagcUgggcuguga..............................................................   1     1   J17
...........................acucaGucagccgggcuguga..............................................................   1     1   J17
...........................acucacucagccgggcuguga..............................................................   1143  0   J17
...........................acucacucagccgggcuguAa..............................................................   2     1   J17
...........................acCcacucagccgggcuguga..............................................................   1     1   J17
...........................acucacucagccgggUuguga..............................................................   2     1   J17
...........................acucacucagccgggcugugU..............................................................   7     1   J17
...........................aGucacucagccgggcuguga..............................................................   3     1   J17
...........................acucacucagccgggcugugau.............................................................   155   0   J17
...........................acucacucagccgggcugugUu.............................................................   1     1   J17
...........................acuUacucagccgggcugugau.............................................................   1     1   J17
...........................acucacucagccgggcugugaA.............................................................   7     1   J17
...........................acucacucagccgUgcugugau.............................................................   1     1   J17
...........................aGucacucagccgggcugugau.............................................................   1     1   J17
...........................acucacucagccgggcugugauA............................................................   6     1   J17
...........................acucacucagccgggcAgugaug............................................................   1     1   J17
...........................acucacucagccgggcugugauU............................................................   38    1   J17
...........................acucacucagccgggcugugGug............................................................   1     1   J17
...........................acucacucagccgggcuCugaug............................................................   1     1   J17
...........................acucacucagccgggcugugaug............................................................   153   0   J17
...........................acucacucGgccgggcugugaug............................................................   1     1   J17
...........................acucacucUgccgggcugugaug............................................................   1     1   J17
...........................acucacucagccgggcugugaugA...........................................................   6     1   J17
...........................acucacucagccgggcugugaugu...........................................................   12    0   J17
...........................acucacucagccgggcugugauguU..........................................................   9     1   J17
```

Star Mature

cccuguggaaguccuguucuauacuaaacucacucagccgggcugugauguccacaccuaaguuaaaucaucacaaccuccgugagugagugacguauacaggaccaauc			
...........................acucacucagccgggcugugauguсс...	1	0	J17
............................Aucacucagccgggcuguga..	2	1	J17
............................cucacucagccgggcugugau...	3	0	J17
............................cucacucagccgggcugugauguU..	1	1	J17
.............................ucacucagccgggcugugauU..	1	1	J17
..............................cacucagccgggcugugaug..	1	0	J17
....uguccacaccuaaguuaaaucauc......................................	1	0	J17
....guccacaccuaaguuaaaucau.......................................	1	0	J17
....uccacaccuaaguuaaauc...	3	0	J17
....uccacaccuaaguuaaauca..	33	0	J17
....uccacaccuaaguuaaaucau.......................................	2	0	J17
....uccacaccuaaguuaaaucaA.......................................	1	1	J17
....uccacaccuaaguuaaaucauU......................................	1	1	J17
....ucaucacaaccuccgugag........................	1	0	J17
....Gaucacaaccuccgugagugagu...................	1	1	J17
....Aaucacaaccuccgugagugagu...................	1	1	J17
....Uaucacaaccuccgugagugagu...................	1	1	J17
....Uaucacaaccuccgugagugagug..................	1	1	J17
....Uucacaaccuccgugagugag....................	2	1	J17
....aucacaaccuccgugagugag....................	1	0	J17
....aucacaaccuccgugagugagu...................	1	0	J17
....Gucacaaccuccgugagugagu...................	1	1	J17
....Uucacaaccuccgugagugagu...................	23	1	J17
....aucacaaccuccgugagugagug..................	1	0	J17
....Uucacaaccuccgugagugagug..................	8	1	J17
....ucacaaccuccgugagug......................	12	0	J17
....ucacaaccuccgugaguga.....................	39	0	J17
....Ccacaaccuccgugagugag....................	1	1	J17
....ucacaaccuccgugagugag....................	376	0	J17
....ucacaCccuccgugagugag....................	1	1	J17
....ucacaaccuccCugagugag....................	1	1	J17
....ucacaaccuccUugagugag....................	2	1	J17
....ucacaaccuccgugagugUg....................	2	1	J17
....ucacaaccuccguAagugagu...................	1	1	J17
....ucacGaccuccgugagugagu...................	3	1	J17
....ucGcaaccuccgugagugagu...................	6	1	J17
....ucacaaccuccgugCgugagu...................	1	1	J17
....ucacaaccuccAugagugagu...................	1	1	J17
....ucacaaccuccgCgagugagu...................	2	1	J17
....ucacaGccuccgugagugagu...................	5	1	J17
....uGacaaccuccgugagugagu...................	3	1	J17
....ucaAaaccuccgugagugagu...................	1	1	J17
....ucacaaccuccgugaguCagu...................	1	1	J17
....ucacaaccuccguCagugagu...................	2	1	J17
....ucacCaccuccgugagugagu...................	2	1	J17
....ucacaaccuccgugagugagG...................	9	1	J17
....ucacaaccuGcgugagugagu...................	5	1	J17
....ucacaaccCccgugagugagu...................	5	1	J17
....ucacaaccuccgugaguUagu...................	1	1	J17
....ucacaaccuccgugaCugagu...................	2	1	J17
....ucacaaccuccgugagCgagu...................	2	1	J17
....ucacaaccucUgugagugagu...................	8	1	J17
....Ccacaaccuccgugagugagu...................	1	1	J17
....ucacaaccuUcgugagugagu...................	3	1	J17
....ucacaaccuccgugUgugagu...................	17	1	J17
....ucacaaccuccgugagugaAu...................	2	1	J17
....ucacaaUcuccgugagugagu...................	2	1	J17
....ucacaaccuccgugaguAagu...................	1	1	J17
....ucacaaccuccgugagugagu...................	15925	0	J17
....ucacaaccuccgugagAgagu...................	13	1	J17
....ucacaaccuccUugagugagu...................	44	1	J17
....Gcacaaccuccgugagugagu...................	2	1	J17
....ucacUaccuccgugagugagu...................	3	1	J17
....ucacaaccuccguUagugagu...................	1	1	J17
....ucaUaaccuccgugagugagu...................	4	1	J17
....ucacaaccuccgugagGgagu...................	1	1	J17
....ucacaacAuccgugagugagu...................	1	1	J17
....ucacaaccuccgugagugUgu...................	12	1	J17
....ucacaaccuccgGgagugagu...................	3	1	J17
....ucacaaccuccgugagugaCu...................	2	1	J17

Star Mature

```
cccuguggaaguccuguucuauacuaaacucacucagccgggcugugauguccacaccuaaguuaaaucaucacaaccuccgugagugagugacguauacaggaccaauc
......................................................................ucacaaccucGgugagugagu...................   1     1   J17
......................................................................ucacaaccuccgugagugagA...................   101   1   J17
......................................................................ucacaCccuccgugagugagu...................   1     1   J17
......................................................................Acacaaccuccgugagugagu...................   16    1   J17
......................................................................ucacaaccuccgugagugGgu...................   3     1   J17
......................................................................ucacaaccuccgAgagugagu...................   10    1   J17
......................................................................ucacaaccuAcgugagugagu...................   1     1   J17
......................................................................ucacaaccAccgugagugagu...................   12    1   J17
......................................................................ucacaaccuccgugagugagC...................   16    1   J17
......................................................................ucacaaccuccCugagugagu...................   6     1   J17
......................................................................ucacaaccuccgugGgugagu...................   7     1   J17
......................................................................ucacaaccGccgugagugagu...................   2     1   J17
......................................................................ucacaacUuccgugagugagu...................   3     1   J17
......................................................................uUacaaccuccgugagugagu...................   8     1   J17
......................................................................ucUcaaccuccgugagugagu...................   3     1   J17
......................................................................ucacaaccuccgugaAugagu...................   2     1   J17
......................................................................ucacaaccuccgugaUugagu...................   2     1   J17
......................................................................ucacaaccuccgugagugaUu...................   1     1   J17
......................................................................ucacaaccuccgCgagugagug..................   2     1   J17
......................................................................ucacaaccuccgugagugaguU..................   44    1   J17
......................................................................ucacaaccuccUugagugagug..................   1     1   J17
......................................................................ucacaaccuccgugagAgagug..................   2     1   J17
......................................................................ucacaaccuccgugagugagAg..................   2     1   J17
......................................................................ucacaaccuGcgugagugagug..................   2     1   J17
......................................................................ucacaaccuccgugaAugagug..................   1     1   J17
......................................................................ucacaaccuccgugGgugagug..................   1     1   J17
......................................................................uUacaaccuccgugagugagug..................   1     1   J17
......................................................................ucaAaaccuccgugagugagug..................   2     1   J17
......................................................................ucUcaaccuccgugagugagug..................   1     1   J17
......................................................................ucacaaccuccgugagugaguC..................   4     1   J17
......................................................................ucacaaccuccgugagugGgug..................   1     1   J17
......................................................................ucacaaccuccgugagugaguA..................   103   1   J17
......................................................................ucaUaaccuccgugagugagug..................   3     1   J17
......................................................................ucacGaccuccgugagugagug..................   1     1   J17
......................................................................ucacaaccuccgugagugagCg..................   4     1   J17
......................................................................Acacaaccuccgugagugagug..................   6     1   J17
......................................................................ucacaaccuccgAgagugagug..................   9     1   J17
......................................................................ucacaaccuccCugagugagug..................   2     1   J17
......................................................................ucacaaccuccgugUgugagug..................   5     1   J17
......................................................................ucacaaccuccgugagugagug..................   4749  0   J17
......................................................................ucacaaAcuccgugagugagug..................   1     1   J17
......................................................................ucacaaccuccgugCgugagug..................   1     1   J17
......................................................................ucacaaccucUgugagugagug..................   1     1   J17
......................................................................ucacaaccuccgugagugagugU.................   57    1   J17
......................................................................ucacaUccuccgugagugaguga.................   1     1   J17
......................................................................ucacaaccuccgugagugagugC.................   2     1   J17
......................................................................ucacaaccuccgugagugaguga.................   778   0   J17
......................................................................ucacaaccuccgugagugagugG.................   1     1   J17
......................................................................ucacaaccuccgugUgugaguga.................   2     1   J17
......................................................................ucacaaccuccgugagugagugaA................   3     1   J17
.......................................................................cacaaccuccgugagugag....................   3     0   J17
.......................................................................cacaaccuccgugagugagu...................   61    0   J17
.......................................................................cacaaccuccgugagugagug..................   33    0   J17
.......................................................................cacaaccuccgugagugaguga.................   9     0   J17
.......................................................................cacaaccuccgugagugagugU.................   10    1   J17
.......................................................................cacaaccuccgugagugagugaU................   1     1   J17
.......................................................................cacaaccuccgugagugagugaA................   2     1   J17
........................................................................Ucaaccuccgugagugag....................   1     1   J17
........................................................................acaaccuccgugagugagu...................   2     0   J17
........................................................................acaaccuccgugagugagug..................   1     0   J17
........................................................................acaaccuccgugagugaguA..................   1     1   J17
........................................................................Ucaaccuccgugagugagug..................   1     1   J17
........................................................................acaaccuccgugagugagugU.................   2     1   J17
........................................................................acaaccuccgugagugaguga.................   13    0   J17
.........................................................................caaccuccgugagugagu...................   1     0   J17
.........................................................................caaccuccgugagugagug..................   10    0   J17

...........................acucacucagccgggcugug...............................................................   391   0   HMA
...........................acucacucagGcgggcugug...............................................................   1     1   HMA
...........................acucacucagUcgggcugug...............................................................   2     1   HMA
```

```
                          Star                                    Mature
cccuguggaaguccuguucuauacuaaacucacucagccgggcugugauguccacaccuaaguuaaaaucaucacaaccuccgugagugagugacguauacaggaccaauc
...........................acucacucagccAggcugug................................................................ 1      1   HMA
...........................Gcucacucagccgggcugug................................................................ 1      1   HMA
...........................acucacucagUcgggcuguga............................................................... 1      1   HMA
...........................acucacucagccgggcugugC............................................................... 1      1   HMA
...........................acucacucagccgggcugugU............................................................... 61     1   HMA
...........................acucacucagccgggcuguga............................................................... 568    0   HMA
...........................acCcacucagccgggcuguga............................................................... 1      1   HMA
...........................acucacucagccggAcuguga............................................................... 2      1   HMA
...........................acucacucagccgggcCguga............................................................... 1      1   HMA
...........................acucacucagccgggcGguga............................................................... 1      1   HMA
...........................acucacucagccgggcugGga............................................................... 1      1   HMA
...........................acucUcucagccgggcugugaug............................................................. 2      1   HMA
...........................acucacCcagccgggcugugaug............................................................. 5      1   HMA
...........................acucacucagccgggcuCugaug............................................................. 1      1   HMA
...........................acucacucagccgUgcugugaug............................................................. 1      1   HMA
...........................acucacucaCccgggcugugaug............................................................. 1      1   HMA
...........................acucacucagccgggUugugaug............................................................. 5      1   HMA
...........................acCcacucagccgggcugugaug............................................................. 5      1   HMA
...........................acucacucagccgggcugugaAg............................................................. 1      1   HMA
...........................acucacucagccgggcCgugaug............................................................. 2      1   HMA
...........................Ucucacucagccgggcugugaug............................................................. 1      1   HMA
...........................acucacucagccgggcugugaCg............................................................. 5      1   HMA
...........................acucacucagccgggcuAugaug............................................................. 1      1   HMA
...........................acucacucagccgggcGgugaug............................................................. 1      1   HMA
...........................acucacucagccgggcugugaGg............................................................. 5      1   HMA
...........................acucacucagccgggcugugaug............................................................. 13976  0   HMA
...........................acucacucagccgggcugugGug............................................................. 7      1   HMA
...........................acucacucagccgggcAgugaug............................................................. 1      1   HMA
...........................acucacucaUccgggcugugaug............................................................. 1      1   HMA
...........................acucacucagccgggcugGgaug............................................................. 4      1   HMA
...........................acucacucaAccgggcugugaug............................................................. 1      1   HMA
...........................acuGacucagccgggcugugaug............................................................. 1      1   HMA
...........................acucacGcagccgggcugugaug............................................................. 2      1   HMA
...........................acucacucagccgAgcugugaug............................................................. 2      1   HMA
...........................acucacucagccggAcugugaug............................................................. 25     1   HMA
...........................acucacucagcUgggcugugaug............................................................. 4      1   HMA
...........................acuAacucagccgggcugugaug............................................................. 1      1   HMA
...........................acucGcucagccgggcugugaug............................................................. 1      1   HMA
...........................acucacucagccgggAugugaug............................................................. 1      1   HMA
...........................acucacucagcGgggcugugaug............................................................. 1      1   HMA
...........................acucaGucagccgggcugugaug............................................................. 1      1   HMA
...........................Gcucacucagccgggcugugaug............................................................. 1      1   HMA
...........................acucacucagccgggcuguAaug............................................................. 1      1   HMA
...........................acucacucagccAggcugugaug............................................................. 3      1   HMA
...........................acucacucagccgggcugCgaug............................................................. 3      1   HMA
...........................acucacucagccgggcuguCaug............................................................. 1      1   HMA
...........................acucacucagccgggcugugauU............................................................. 413    1   HMA
...........................aGucacucagccgggcugugaug............................................................. 1      1   HMA
...........................acucacucagccgggcugugauA............................................................. 6      1   HMA
...........................acAcacucagccgggcugugaug............................................................. 3      1   HMA
...........................acucacucagUcgggcugugaug............................................................. 39     1   HMA
...........................acucacucagccgggcuUugaug............................................................. 5      1   HMA
...........................acucacucGgccgggcugugaug............................................................. 4      1   HMA
...........................acucacucagccggUcugugaug............................................................. 1      1   HMA
............................cucacucagUcgggcugugaug............................................................. 4      1   HMA
............................cucacucagccggAcugugaug............................................................. 2      1   HMA
............................cucacucagccgggcugugaGg............................................................. 1      1   HMA
............................cucacucagccgggcCgugaug............................................................. 1      1   HMA
............................cucacucagccgggcugugauC............................................................. 1      1   HMA
............................cucacucagcGgggcugugaug............................................................. 1      1   HMA
............................cucacucagccgggcugugCug............................................................. 1      1   HMA
............................cucacucagccgggcuguUaug............................................................. 1      1   HMA
............................cucacucagccgggcugugaAg............................................................. 2      1   HMA
............................cucacucagccgggcugugaug............................................................. 1048   0   HMA
.......................................................................ucacaaccuccgugagugUgu................... 1      1   HMA
.......................................................................ucacaaccuAcgugagugagu................... 3      1   HMA
.......................................................................ucGcaaccuccgugagugagu................... 2      1   HMA
.......................................................................Ccacaaccuccgugagugagu................... 1      1   HMA
.......................................................................ucacaaccuccgugagugagG................... 1      1   HMA
.......................................................................ucacaaccucAgugagugagu................... 1      1   HMA
```

```
                            Star                                        Mature
cccuguggaaguccuguucuauacuaaacucacucagccgggcugugauguccacaccuaaguuaaaucaucacaaccuccgugagugagugacguauacaggaccaauc
......................................................................ucacaaccuccgugagugagA...................   4      1   HMA
......................................................................ucaAaaccuccgugagugagu...................   1      1   HMA
......................................................................ucacaaccuUcgugagugagu...................   1      1   HMA
......................................................................ucacaaccuccgugagugagu...................   6605   0   HMA
......................................................................ucacaaccuccgugaguAagu...................   1      1   HMA
......................................................................uUacaaccuccgugagugagu...................   1      1   HMA
......................................................................ucacaaccGccgugagugagu...................   1      1   HMA
......................................................................ucacaaccuccgGgagugagu...................   6      1   HMA
......................................................................ucacaaccuccgCgagugagu...................   4      1   HMA
......................................................................ucacaaccAccgugagugagu...................   1      1   HMA
......................................................................Gcacaaccuccgugagugagu...................   3      1   HMA
......................................................................ucacaaccuccgugagAgagu...................   2      1   HMA
......................................................................ucacGaccuccgugagugagu...................   1      1   HMA
......................................................................ucacaaccuccgugagCgagu...................   5      1   HMA
......................................................................ucacaaccuccgugGgugagu...................   1      1   HMA
......................................................................ucacaaccuccgugaAugagu...................   1      1   HMA
......................................................................ucacaaccuccgugagugagC...................   194    1   HMA
......................................................................ucacaGccuccgugagugagu...................   1      1   HMA
......................................................................ucacaaccuccgugagGgagu...................   5      1   HMA
......................................................................Acacaaccuccgugagugagu...................   2      1   HMA
......................................................................ucacaaccuccgugagugagug..................   3      0   HMA
......................................................................ucacaaccuccgugagugaguga.................   125    0   HMA
......................................................................ucacaaccuccgugGgugaguga.................   1      1   HMA
......................................................................ucacaaccuccgugagugagugU.................   1      1   HMA
.......................................................................cacaaccuccgugagugagu...................   263    0   HMA
.......................................................................cacaaccuccgGgagugagu...................   1      1   HMA
.......................................................................cacaaccuccgugagugagC...................   10     1   HMA
.......................................................................cacaaccCccgugagugagu...................   2      1   HMA
........................................................................acaaccuccgugagAgagu...................   1      1   HMA
........................................................................acaaccuccgugagugagC...................   1      1   HMA
........................................................................acaaccuccgugagugagu...................   99     0   HMA

........................uaaacucacucagccgggcugugU..............................................................   1      1   HFA
..........................aacucacucagccgggcugug...............................................................   1      0   HFA
..........................aacucacucagccgggcugugG..............................................................   1      1   HFA
..........................aacucacucagccgggcuguga..............................................................   1      0   HFA
...........................acucacucagccgggcug.................................................................   15     0   HFA
...........................acucacucagccgggcuA.................................................................   2      1   HFA
...........................acucacucagccgggcuU.................................................................   4      1   HFA
...........................acucacucGgccgggcugu................................................................   1      1   HFA
...........................acucacuUagccgggcugu................................................................   1      1   HFA
...........................acucacucagccgggcugA................................................................   1      1   HFA
...........................acucacucagccgggcugu................................................................   331    0   HFA
...........................acucacucagcUgggcugu................................................................   1      1   HFA
...........................aGucacucagccgggcugug...............................................................   1      1   HFA
...........................acucacucagccgggcugCg...............................................................   1      1   HFA
...........................acucacucagccgggcugug...............................................................   188    0   HFA
...........................acucacucagccgggcuguA...............................................................   3      1   HFA
...........................acucacucagccgggcuguU...............................................................   16     1   HFA
...........................acucacucagccgggUuguga..............................................................   1      1   HFA
...........................acucacucagUcgggcuguga..............................................................   2      1   HFA
...........................acucacucagccgggcugGga..............................................................   2      1   HFA
...........................acucacucagccggUcuguga..............................................................   1      1   HFA
...........................acucacucagccggAcuguga..............................................................   1      1   HFA
...........................aGucacucagccgggcuguga..............................................................   2      1   HFA
...........................acucacCcagccgggcuguga..............................................................   1      1   HFA
...........................acucacucagccgggcuguga..............................................................   1120   0   HFA
...........................acucacucagccgggcugugU..............................................................   38     1   HFA
...........................acucacucagccgggcuAuga..............................................................   1      1   HFA
...........................acucaUucagccgggcuguga..............................................................   1      1   HFA
...........................acucacucagccgggcugugaC.............................................................   1      1   HFA
...........................acucacucagccgggcugugaA.............................................................   10     1   HFA
...........................acucacucagccgggcugugau.............................................................   39     0   HFA
...........................acucacucagccgggcugugUu.............................................................   1      1   HFA
...........................acucacucagccgggcugugauA............................................................   6      1   HFA
...........................acucacucagccgggcugugaug............................................................   178    0   HFA
...........................acucacucagccgggcugugauU............................................................   29     1   HFA
...........................acucacucagccggAcugugaug............................................................   2      1   HFA
...........................acucacucagccggCcugugaug............................................................   1      1   HFA
...........................acucacucagccgggcugugaugA...........................................................   1      1   HFA
```

```
                           Star                                          Mature
cccuguggaaguccuguucuauacuaaacucacucagccgggcugugauguccacaccuaaguuaaaucaucacaaccuccgugagugagugacguauacaggaccaauc
...........................acucacucagccgggcugugauguU..........................................................  2    1  HFA
...........................acucacucagccgggcugugaugucc.........................................................  1    0  HFA
............................cucacucagccgggcugugau.............................................................  1    0  HFA
.............................ucacucagccgggcugug...............................................................  1    0  HFA
.............................ucacucagccgggcugugaug............................................................  1    0  HFA
..............................cacucagccgggcuguga..............................................................  3    0  HFA
..............................Uacucagccgggcuguga..............................................................  1    1  HFA
..............................cacucagccgggcugugaug............................................................  1    0  HFA
................................cucagccgggcugugaug............................................................  3    0  HFA
......................................cgggcugugauguccacacc....................................................  1    0  HFA
................................................uguccacaccuaaguuaaauc.........................................  2    0  HFA
................................................uguccacaccuaaguuaaaucau.......................................  2    0  HFA
................................................uguccacaccuaaguuaaaucauc......................................  2    0  HFA
..................................................uccacaccuaaguuaaauc.........................................  4    0  HFA
..................................................uccacaccuaaguuaaauca........................................  5    0  HFA
.....................................................acaccuaaguuaaaucau.......................................  1    0  HFA
.............................................................guuaaaucaucacaaccuccg............................  1    0  HFA
...............................................................uaaaucaucacaaccuccgu...........................  1    0  HFA
....................................................................Uaucacaaccuccgugagugagu...................  1    1  HFA
....................................................................Gaucacaaccuccgugagugagug..................  1    1  HFA
.....................................................................Uucacaaccuccgugagu.......................  1    1  HFA
.....................................................................Uucacaaccuccgugagugag....................  1    1  HFA
.....................................................................aucacaaccuccgugagugag....................  1    0  HFA
.....................................................................aucacaaccuccgugagugagu...................  4    0  HFA
.....................................................................Uucacaaccuccgugagugagu...................  51   1  HFA
.....................................................................Gucacaaccuccgugagugagu...................  3    1  HFA
.....................................................................Uucacaaccuccgugagugagug..................  5    1  HFA
......................................................................ucacaaccuccUugagug......................  1    1  HFA
......................................................................ucacaaccuccgugagug......................  69   0  HFA
......................................................................ucacaaccuccgugaguA......................  2    1  HFA
......................................................................ucacaaccuccgCgagug......................  1    1  HFA
......................................................................ucacaaccuccgugaguga.....................  35   0  HFA
......................................................................ucacaaccuccgugagugaA....................  1    1  HFA
......................................................................ucacaaccuccgugagugaU....................  1    1  HFA
......................................................................ucacaaccuccCugagugag....................  1    1  HFA
......................................................................ucacaaccuccgugCgugag....................  1    1  HFA
......................................................................ucacaaccuccgugagugag....................  466  0  HFA
......................................................................ucacaaccuccgugGgugag....................  1    1  HFA
......................................................................ucacaaccuGcgugagugagu...................  5    1  HFA
......................................................................ucacaacGuccgugagugagu...................  2    1  HFA
......................................................................uUacaaccuccgugagugagu...................  19   1  HFA
......................................................................ucacaaccuccgugagAgagu...................  1    1  HFA
......................................................................ucacaaccuccgugagugaUu...................  1    1  HFA
......................................................................ucacaaccucAgugagugagu...................  2    1  HFA
......................................................................ucacaaccuccgugaguCagu...................  2    1  HFA
......................................................................ucacaaccuccgugagugaCu...................  8    1  HFA
......................................................................ucacaaccuccgAgagugagu...................  5    1  HFA
......................................................................Gcacaaccuccgugagugagu...................  4    1  HFA
......................................................................ucacaaccuccgugCgugagu...................  15   1  HFA
......................................................................ucUcaaccuccgugagugagu...................  1    1  HFA
......................................................................ucacaaccuccgugaCugagu...................  3    1  HFA
......................................................................ucacaUccuccgugagugagu...................  1    1  HFA
......................................................................ucacaaccucGgugagugagu...................  5    1  HFA
......................................................................ucacGaccuccgugagugagu...................  5    1  HFA
......................................................................Acacaaccuccgugagugagu...................  9    1  HFA
......................................................................ucaUaaccuccgugagugagu...................  21   1  HFA
......................................................................uGacaaccuccgugagugagu...................  12   1  HFA
......................................................................ucacaaccCccgugagugagu...................  10   1  HFA
......................................................................ucacaacUuccgugagugagu...................  18   1  HFA
......................................................................ucaAaaccuccgugagugagu...................  1    1  HFA
......................................................................ucacaaccuccgugagugUgu...................  6    1  HFA
......................................................................ucacaaccuccgugagugCgu...................  4    1  HFA
......................................................................ucacaaccuccguAagugagu...................  3    1  HFA
......................................................................ucacaaccuccgugagugagA...................  470  1  HFA
......................................................................ucacaaccuccgugUgugagu...................  59   1  HFA
......................................................................ucacaaccuccgugaUugagu...................  9    1  HFA
......................................................................ucCcaaccuccgugagugagu...................  1    1  HFA
......................................................................Ccacaaccuccgugagugagu...................  6    1  HFA
......................................................................ucaGaaccuccgugagugagu...................  1    1  HFA
......................................................................ucacaaccuccgugagugagC...................  77   1  HFA
```

Star **Mature**

```
cccuguggaaguccuguucuauacuaaacucacucagccgggcugugauguccacaccuaaguuaaaucaucacaaccuccgugagugagugacguauacaggaccaauc
......................................................................ucacaaccuccgugagGgagu...................   2      1   HFA
......................................................................ucacaaccucUgugagugagu...................   12     1   HFA
......................................................................ucacUaccuccgugagugagu...................   8      1   HFA
......................................................................uAacaaccuccgugagugagu...................   18     1   HFA
......................................................................ucacaaccAccgugagugagu...................   4      1   HFA
......................................................................ucacaaccuccgCgagugagu...................   11     1   HFA
......................................................................ucacaaccuccguCagugagu...................   8      1   HFA
......................................................................ucacaaccuccgugaAugagu...................   1      1   HFA
......................................................................ucacaaccuccAugagugagu...................   9      1   HFA
......................................................................ucacaaccuccguUagugagu...................   5      1   HFA
......................................................................ucacaCccuccgugagugagu...................   3      1   HFA
......................................................................ucacaaccuccgugagugaAu...................   1      1   HFA
......................................................................ucacaaccuccgugagCgagu...................   5      1   HFA
......................................................................ucacaaccuUcgugagugagu...................   11     1   HFA
......................................................................ucacaaccuccgugGgugagu...................   29     1   HFA
......................................................................ucacaaccuccgugagugagu...................   53934  0   HFA
......................................................................ucacaaccuccCugagugagu...................   14     1   HFA
......................................................................ucacaaccuccgugagugGgu...................   12     1   HFA
......................................................................ucGcaaccuccgugagugagu...................   12     1   HFA
......................................................................ucacaaccuccgugagugagG...................   29     1   HFA
......................................................................ucacaGccuccgugagugagu...................   3      1   HFA
......................................................................ucacaaUcuccgugagugagu...................   21     1   HFA
......................................................................ucacaaccuccUugagugagu...................   16     1   HFA
......................................................................uUacaaccuccgugagugagug..................   2      1   HFA
......................................................................ucacaaccucUgugagugagug..................   2      1   HFA
......................................................................ucacaaccuccgugagugagug..................   1826   0   HFA
......................................................................ucacaaccuccgugagugaguC..................   3      1   HFA
......................................................................ucacaaUcuccgugagugagug..................   1      1   HFA
......................................................................ucacaaccuccgugagugagCg..................   3      1   HFA
......................................................................ucacaaccuccgugaguUagug..................   1      1   HFA
......................................................................ucacaaccucGgugagugagug..................   1      1   HFA
......................................................................ucacaaccuccUugagugagug..................   2      1   HFA
......................................................................ucacaaccuccgCgagugagug..................   1      1   HFA
......................................................................ucacaaccuccgugagugagGg..................   1      1   HFA
......................................................................ucacaaccuccgugagugaguU..................   52     1   HFA
......................................................................ucacGaccuccgugagugagug..................   1      1   HFA
......................................................................ucacaaccuccguCagugagug..................   1      1   HFA
......................................................................ucacaaccuccgAgagugagug..................   1      1   HFA
......................................................................ucacaUccuccgugagugagug..................   1      1   HFA
......................................................................ucacaaccuccgugaUugagug..................   1      1   HFA
......................................................................ucacaaccuccgugagugaguA..................   102    1   HFA
......................................................................ucacaacUuccgugagugagug..................   1      1   HFA
......................................................................ucacaaccuccgugagugUgug..................   2      1   HFA
......................................................................ucGcaaccuccgugagugagug..................   1      1   HFA
......................................................................ucacaaccuccCugagugagug..................   1      1   HFA
......................................................................ucacaaccuccgugagugagugC.................   1      1   HFA
......................................................................ucacaacGuccgugagugaguga.................   1      1   HFA
......................................................................ucacaaccuccgugagugagugG.................   1      1   HFA
......................................................................ucacaaUcuccgugagugaguga.................   1      1   HFA
......................................................................ucacaaccuccgugagugagugU.................   18     1   HFA
......................................................................ucacaaccuccgugagugaguga.................   19     0   HFA
......................................................................ucacaaccuccgugagugagugaA................   1      1   HFA
.......................................................................cacaaccuccgugagugag....................   6      0   HFA
.......................................................................cacaaccuccgugagugagG...................   1      1   HFA
.......................................................................Uacaaccuccgugagugagu...................   1      1   HFA
.......................................................................Aacaaccuccgugagugagu...................   1      1   HFA
.......................................................................cacaaccuccgugagugagA...................   2      1   HFA
.......................................................................cacaaUcuccgugagugagu...................   1      1   HFA
.......................................................................cacaaccuccgugagugagu...................   239    0   HFA
.......................................................................cacaaccuccguCagugagug..................   1      1   HFA
.......................................................................cacaaccuccgugagugagug..................   11     0   HFA
.......................................................................cacaaccuccgugagugaguA..................   1      1   HFA
.......................................................................Uacaaccuccgugagugagug..................   1      1   HFA
.......................................................................cacaaccuccgugagugaguga.................   3      0   HFA
.......................................................................cacaaccuccgugagugagugU.................   3      1   HFA
.......................................................................cacaaccuccgugagugagugaA................   1      1   HFA
........................................................................acaaccuccgugagugaA....................   2      1   HFA
........................................................................acaaccuccgugagugag....................   12     0   HFA
........................................................................acaaccuccgugagugagu...................   30     0   HFA
........................................................................acaaccuccgugagugagA...................   1      1   HFA
```

```
                           Star                                       Mature
cccuguggaaguccuguucuauacuaaacucacucagccgggcugugauguccacaccuaaguuaaaaucaucacaaccuccgugagugagugacguauacaggaccaauc
.........................................................................Ucaaccuccgugagugagu...................      8      1      HFA
.........................................................................acaaccuccgugagugaguA..................      2      1      HFA
.........................................................................acaaccuccgugagugagug..................      7      0      HFA
.........................................................................acaaccuccgugagugagugU.................      9      1      HFA
.........................................................................acaaccuccgugagugaguga.................      32     0      HFA
.........................................................................acaaccuccgugagugagugac................      1      0      HFA
.........................................................................acaaccuccgugagugagugaA................      1      1      HFA
..........................................................................caaccuccgugagugagu...................      43     0      HFA
..........................................................................caaccuccgugaguUagu...................      1      1      HFA
..........................................................................caaccuUcgugagugagu...................      1      1      HFA
..........................................................................caaccuccgugagugagug..................      4      0      HFA
...........................................................................aaccuccgugagugagug..................      2      0      HFA
...........................................................................aaccuccgugagugaguga.................      1      0      HFA
```

Provisional ID	:	SczTNLB_2740_2807
Score total	:	143.7
Score for star read(s)	:	3.9
Score for read counts	:	133.7
Score for mfe	:	1.5
Score for randfold	:	1.6
Score for cons. seed	:	3
Total read count	:	274
Mature read count	:	223
Loop read count	:	3
Star read count	:	48

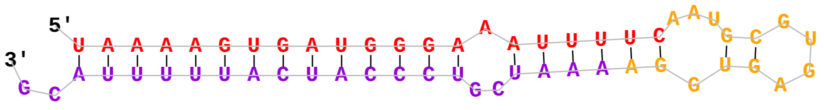

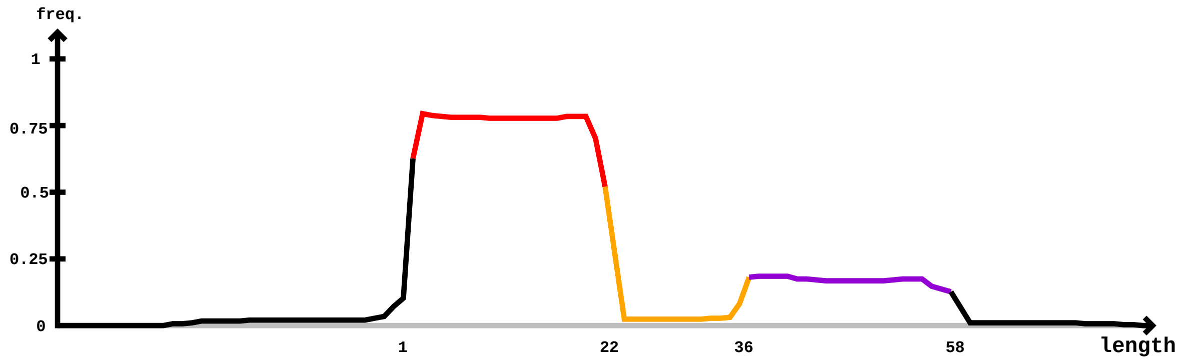

Mature Star

5'- gggguguugaguaauuggauauggacuuuccuaugu**uaaaagugaugggaaauuuuc**aaugcgugagugga**aaaucgucccaucauuuuuacg**guacaagucuaagcacggga -3'	obs			
ggguguugaguaauuggauauggacuuuccuauguuaaaagugaugggaaauuuucaaugcgugaguggaaaaucgucccaucauuuuuacgguacaagucuaagcacggga	exp			
..(((((.((...(((.((((((......))))))((((((((((((((.((((((...((....)).))))))..)))))))))))))).....)))..)).)))))....		reads	mm	sample
...................................Aaaaagugaugggaaauuuuc..		1	1	EGG
....................................aaaagugaugggaaauuuuca...		1	0	EGG
....aaaaucgucccaucauuuuu.......................		1	0	EGG
...............................auguuaaaagugaugggaaauuu..		1	0	HS4
................................ugCuaaaagugaugggaaauuu..		1	1	HS4
.................................guuaaaagugaugggaaauu...		2	0	HS4
.................................guuaaaagugaugggaaauuuu...		3	0	HS4
..................................uuaaaagugaugggaaauuuu...		3	0	HS4
...................................Aaaaagugaugggaaauuuu...		16	1	HS4
...................................Aaaaagugaugggaaauuuuc..		23	1	HS4
...................................Aaaaagugaugggaaauuuuca...		6	1	HS4
....................................aaaagugaugggaaauuu..		2	0	HS4
....................................aaaagugaugggaaauuuu...		2	0	HS4
....................................aUaagugaugggaaauuuuc..		1	1	HS4
....................................aaaagugaugggaaauuuuc..		1	0	HS4
....................................aaaagugaugggaaauuuuca...		4	0	HS4
....................................aUaagugaugggaaauuuuca...		1	1	HS4
......................................aagugaugggaaauuuuca...		1	0	HS4
....gaaaaucgucccaucaCuuuua......................		1	1	HS4
....aaaaucgucccaucauuuuu.......................		1	0	HS4
....aaaaucgucccaucauuuuuacg....................		2	0	HS4
....aaaucgucccaucauuuuuacg....................		8	0	HS4
....aaaucgucccaucaAuuuuacg....................		1	1	HS4
....aaaucgucccaucauCuuuacg....................		1	1	HS4
....aaaucguUccaucauuuuuacg....................		1	1	HS4
....aaucgucccaucauuuuuacg....................		1	0	HS4
................................uguuaaaagugaugggaaauuuuA..		1	1	J17
.................................guuaaaagugaugggaaauuu..		1	0	J17
..................................uuaaaagugaugggaaauuuuc..		2	0	J17
...................................Aaaaagugaugggaaauuu..		4	1	J17
...................................Aaaaagugaugggaaauuuu...		4	1	J17

Mature Star

ggguguugaguaauuggauauggacuuuccuauguuaaaagugaugggaaauuuucaaugcgugaguggaaaaucgucccaucauuuuuacgguacaagucuaagcacggga			
...................................Aaaaagugaugggaaauuuuc..	7	1	J17
...................................uUaaagugaugggaaauuuuc..	1	1	J17
...................................Aaaaagugaugggaaauuuuca...	9	1	J17
....................................aaaagugaugggaaauuuuc..	3	0	J17
....................................aaaagugaugggaaauuuuca...	11	0	J17
....Guuuucaaugcgugaguggaa...	1	1	J17
....uuuGcaaugcgugaguggaaaaucguc..................................	1	1	J17
....aaaaucgucccaucauuuuu.......................	1	0	J17
....aaaaucgucccaucauuuuua......................	1	0	J17
....aaaaucgucccaucauCuuuacg....................	1	1	J17
....aaaucgucccaucauuuuuac.....................	1	0	J17
....aaaucgucccaucauuuuuacg....................	5	0	J17
....aaaucguUccaucauuuuuacg....................	1	1	J17
....cgguacaagucuaagcacg...	1	0	J17
.................................guuaaaagugaugggaaauuu..	1	0	HFA
...................................Aaaaagugaugggaaauuu..	2	1	HFA
...................................Aaaaagugaugggaaauuuu...	3	1	HFA
...................................Aaaaagugaugggaaauuuuc..	3	1	HFA
...................................Aaaaagugaugggaaauuuuca...	1	1	HFA
....................................aaaagugaugggaaauuuu...	1	0	HFA
....................................aaaagugaugggaaauuuuca...	2	0	HFA
....aaaaucgucccaucauuuuu.......................	1	0	HFA
....aaaaucgucccaucauuuuua......................	1	0	HFA
....uuuuacgguacaagucGaagcacggg.	1	1	HFA
.................................guuaaaagugaugggaaauuuu...	1	0	HS5
..................................uuaaaagugaugggaaauuuu...	2	0	HS5
...................................Aaaaagugaugggaaauuu..	3	1	HS5
...................................Aaaaagugaugggaaauuuu...	3	1	HS5
...................................uaaaagugaugggaaauuuuc..	1	0	HS5
...................................Aaaaagugaugggaaauuuuc..	18	1	HS5
...................................Aaaaagugaugggaaauuuuca...	9	1	HS5
....................................aaaagugaugggaaauuuu...	1	0	HS5
....................................aaaagugaugggaaauuuuc..	2	0	HS5
....................................aaaagugaugggaaauuuuca...	7	0	HS5
....aaaaucgucccaucauuuuuac.....................	1	0	HS5
....aaaucgucccaucauuuuuacg....................	2	0	HS5
....aaucguUccaucauuuuuacgg...................	1	1	HS5
...................................Aaaaagugaugggaaauuu..	3	1	HS7
...................................Aaaaagugaugggaaauuuuc..	5	1	HS7
...................................Aaaaagugaugggaaauuuuca...	1	1	HS7
....................................aaaagugaugggaaauuuuc..	2	0	HS7
....................................aaaagugaugggaaauuuuca...	1	0	HS7
....aaaucgucccaucauuuuuacg....................	2	0	HS7
....aaaucgucccaucauCuuuacgg...................	1	1	HS7
..........uaauuggauauggacuuucUuauguua...	2	1	HA1
............auuggauauggacuuuccuauguuaa..	1	0	HA1
.............uuggauauggacuuucUuauguuaa..	1	1	HA1
.............uuggauauggacuuucUuauguuaaa...	1	1	HA1
..................uauggacuuuccuauguuaaaagug...	1	0	HA1
...............................auguuaaaagugaugggaa..	1	0	HA1
.................................guuaaaagugaugggaaauuu..	1	0	HA1
.................................guuaaaagugaugggaaauuuu...	1	0	HA1
.................................gCuaaaagugaugggaaauuuuc..	1	1	HA1
..................................uuaaaagugaugggaaauuu..	1	0	HA1
..................................uuaaaagugaugggaaauuuuU..	1	1	HA1
...................................Aaaaagugaugggaaauuu..	5	1	HA1
...................................uGaaagugaugggaaauuuu...	1	1	HA1
...................................Aaaaagugaugggaaauuuu...	8	1	HA1
...................................Aaaaagugaugggaaauuuuc..	10	1	HA1
...................................Aaaaagugaugggaaauuuuca...	4	1	HA1
....................................aaaagugaugggaaauuuuc..	3	0	HA1
....................................aaaagugaugggaaauuuuca...	2	0	HA1
....uuuucaaugcgugagugga..	1	0	HA1
....aaugcgugaguggaaaaucgu...................................	1	0	HA1
....uggaaaaucgucccaucaCuuuuac.....................	1	1	HA1
....aaaaucgucccaucauuuuu.......................	1	0	HA1

```
                                   Mature                             Star
ggguguugaguaauuggauauggacuuuccuauguuaaaagugaugggaaauuuucaaugcgugaguggaaaaucgucccaucauuuuuacgguacaagucuaagcacggga
.....................................................................aaaaucgucccaucauCuuu.......................    2    1    HA1
.....................................................................aaaaucgucccaucauuuuua......................    1    0    HA1
......................................................................aaaucgucccaucauuuuuacg....................    6    0    HA1

....................................aaaagugaugggaaauuuucU.......................................................    1    1    HS1
.........................................................augcgugaguggaaaauc.....................................    3    0    HS1

...................................Aaaaagugaugggaaauuuu.........................................................    1    1    HS6
...................................Aaaaagugaugggaaauuuuc........................................................    1    1    HS6
....................................aaaagugaugggaaauuuuca.......................................................    1    0    HS6
.....................................................................aaaaucgucccaucauuuuu.......................    1    0    HS6
......................................................................aaaucgucccaucauuuuuacg....................    1    0    HS6
......................................................................................uuuacgguGcaagucuaag.......    1    1    HS6
```

Provisional ID	:	SczTNLB_2740_3667
Score total	:	589.5
Score for star read(s)	:	3.9
Score for read counts	:	580.8
Score for mfe	:	0.2
Score for randfold	:	1.6
Score for cons. seed	:	3
Total read count	:	1151
Mature read count	:	1135
Loop read count	:	0
Star read count	:	16

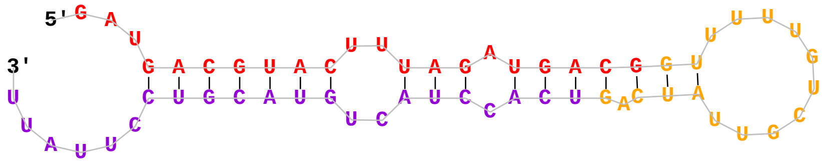

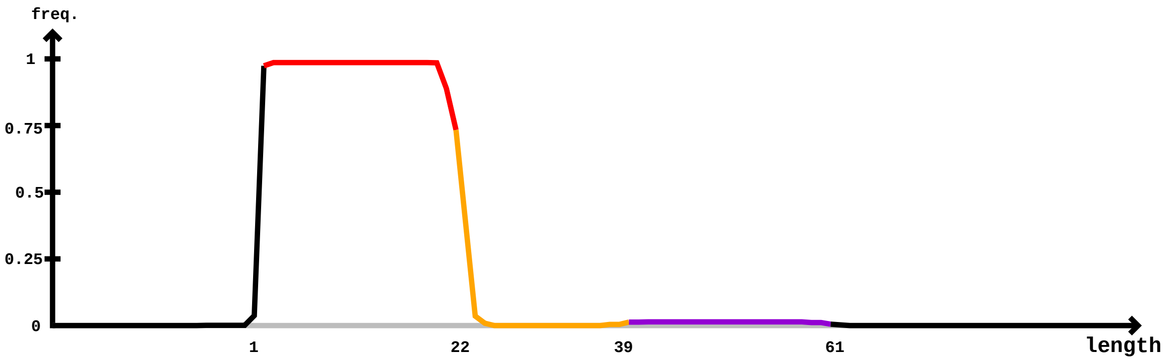

Mature **Star**

```
5'- ucaaauuuuguauggaugaugaugacguacuuuagaugacgguuuuugucguuaucagucaccuacuguacguccuuauuuucauuaacaaaaauuuugaaguaaauuucu  -3'  obs
    ucaaauuuuguauggaugaugaugacguacuuuagaugacgguuuuugucguuaucagucaccuacuguacguccuuauuuucauuaacaaaaauuuugaaguaaauuucu       exp
    ((((((((((((((.(((((.(((((((..(((.(((((((.........))).)))).)))..))))))).))))))))))..))))))..))))).......... reads   mm   sample
    ....................gaugacguacuuuagaugacg......................................................................  1   0   HFA

    ....................gaugacguacuuuagauga........................................................................  1   0   HMA
    ....................gaugacguacuuuagaugac.......................................................................  3   0   HMA
    ....................gaugacguacuuuagauAacg......................................................................  1   1   HMA
    ....................gaugacguacuuuagaugacg......................................................................  133 0   HMA
    ....................gaugacguacuuuagaugacgg.....................................................................  2   0   HMA
    ..........................................................ucaccuacuguacguccuuauu...............................  1   0   HMA

    ...................ugaugacguacuuuagaugacg......................................................................  2   0   J17
    ....................gaugacguacuuuagaugac.......................................................................  3   0   J17
    ....................gaugacguacuuuagaugacg......................................................................  20  0   J17
    ....................gaugacguacuuuagaugUcg......................................................................  1   1   J17
    ....................gaugacguacuuuagaugacgg.....................................................................  9   0   J17
    ....................gaugacguacuuuagaugacggu....................................................................  1   0   J17
    ........................................................agucaccuacuguacguccuu..................................  2   0   J17
    ........................................................agucaccuacuguacguccuuau................................  1   0   J17
    ..........................................................ucaccuacuguacguccuuau................................  2   0   J17
    ............................................................accuacuguacguccuuauuu..............................  1   0   J17

    ....................gaugacguacuuuagauga........................................................................  1   0   HS4
    ....................gaugacguacuuuagaugac.......................................................................  2   0   HS4
    ....................gaugacguacuuuagaugacg......................................................................  35  0   HS4
    ....................gaugacguacuuuagaugacA......................................................................  1   1   HS4
    ....................gaugacguacuuuagaugacgg.....................................................................  12  0   HS4
    ....................gaugacguacuuuagaugacgguu...................................................................  4   0   HS4
    ........................................................agucaccuacuguacguccuu..................................  1   0   HS4
    ........................................................agucaccuacuguacguccuuau................................  1   0   HS4
    ..........................................................ucaccuacuguacguccuuau................................  2   0   HS4

    ....................gaugacguacuuuagaugacA......................................................................  1   1   HS6
    ....................gaugacguacuuuagaugacg......................................................................  10  0   HS6
    ....................gaugacguacuuuagaugacgg.....................................................................  3   0   HS6
```

Mature **Star**

```
ucaaauuuuguauggaugaugaugacguacuuuagaugacgguuuuugucguuaucagucaccuacuguacguccuuauuuucauuaacaaaaauuuugaaguaaauuucu
....................gaugacguacuuuagaugacggu....................................................................   1     0     HS6

..............gaugaugaugacguacuuuagaug.........................................................................   1     0     HS1
...................ugaugacguacuuuagaugac.......................................................................   19    0     HS1
...................ugaugacguacuuuagaugacg......................................................................   20    0     HS1
....................gaugacguacuuuagauga........................................................................   48    0     HS1
....................gaugacguacuuuagaugac.......................................................................   119   0     HS1
....................gaugacguacuuuagaugacU......................................................................   5     1     HS1
....................Uaugacguacuuuagaugacg......................................................................   1     1     HS1
....................gaugacguacuuuagaugacg......................................................................   322   0     HS1
....................gaugacguacuuuagaugacA......................................................................   13    1     HS1
....................gaugacguacuuuagaugaGg......................................................................   1     1     HS1
....................gaugaUguacuuuagaugacg......................................................................   1     1     HS1
....................gaugacguacuuuagaugacgg.....................................................................   92    0     HS1
....................gaugacguacuuuagaugacggu....................................................................   17    0     HS1
....................gaugacguacuuuagaugacggC....................................................................   1     1     HS1
.....................augacguacuuuagauga........................................................................   6     0     HS1
.....................augacguacuuuagaugacggu....................................................................   8     0     HS1
..........................................................ucaccuacuguacguccuuauu...............................   4     0     HS1

....................gaugacguacuuuagauga........................................................................   52    0     HS2
....................gaugacguacuuuagaugac.......................................................................   28    0     HS2
....................gaugacguacuuuagaugacg......................................................................   57    0     HS2

...................ugaugacguacuuuagaugacA......................................................................   1     1     HA1
....................gaugacguacuuuagauga........................................................................   2     0     HA1
....................gaugacguacuuuagaugac.......................................................................   1     0     HA1
....................gaugacguacuuuagaugacg......................................................................   14    0     HA1
....................gaugacguacuuuagaugacgguu...................................................................   1     0     HA1
..........................................................ucaccuacuguacguccuuau................................   1     0     HA1

....................gaugacguacuuuagaugac.......................................................................   1     0     HS7
....................gaugacguacuuuagaugacA......................................................................   1     1     HS7
....................gaugacguacuuuagaugacg......................................................................   6     0     HS7
....................gaugacguacuuuagaugacgg.....................................................................   8     0     HS7
....................gaugacguacuuuagaugacggu....................................................................   1     0     HS7
....................gaugacguacuuuagaugacgguu...................................................................   1     0     HS7

....................gaugacguacuuuagauga........................................................................   1     0     HS5
....................gaugacguacuuuagaugac.......................................................................   3     0     HS5
....................gaugacguacuuuagaugacg......................................................................   12    0     HS5
....................gaugacguacuuuagaugacgg.....................................................................   19    0     HS5
....................gaugacguacuuuagaugacggu....................................................................   2     0     HS5
....................gaugacguacuuuagaugacgguu...................................................................   3     0     HS5
....................gUugacguacuuuagaugacgguu...................................................................   1     1     HS5
```

Provisional ID	:	SczTNLB_2740_3996
Score total	:	567658.8
Score for star read(s)	:	3.9
Score for read counts	:	567648.6
Score for mfe	:	1.8
Score for randfold	:	1.6
Score for cons. seed	:	3
Total read count	:	1113430
Mature read count	:	1036311
Loop read count	:	0
Star read count	:	77119

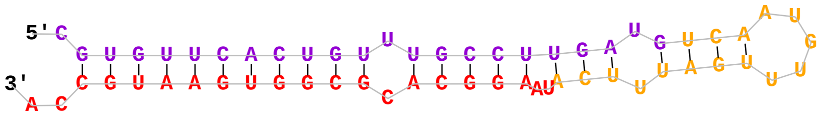

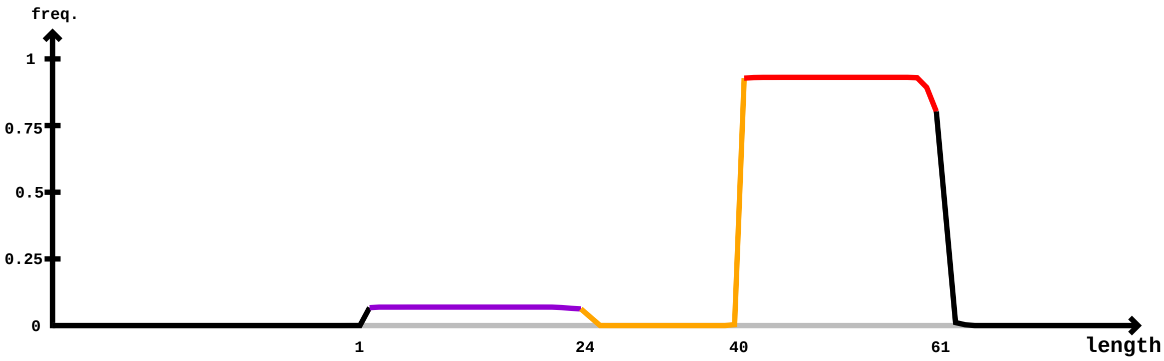


```
                                Star                                  Mature
5'- gugaugcuuuguggaccuuuugugaucgcuucguguucacuguuugccuugaugucaauguuugauuucauaaggcacgcggugaaugccaagugcaaaggucaucauucg  -3'   obs
    gugaugcuuuguggaccuuuugugaucgcuucguguucacuguuugccuugaugucaauguuugauuucauaaggcacgcggugaaugccaagugcaaaggucaucauucg         exp
    .............(((((((.((....((((.(((((((((((.((((((((.((((.....)))).)))..))))).))))))))))).)))))))))))))........   reads   mm   sample
    ........................aucgcuucguguucacuguuugc................................................................   1       0    HS6
    .............................uucguguucacuguuugccuug............................................................   1       0    HS6
    ...............................cguguucacuguuugccuug............................................................   23      0    HS6
    ...............................cguguucacuguuugccuuga...........................................................   10      0    HS6
    ...............................cguguucacuguuugccuugau..........................................................   22      0    HS6
    ...............................cguguucacuguuugccuugaug.........................................................   351     0    HS6
    ...............................Gguguucacuguuugccuugaug.........................................................   1       1    HS6
    ...............................cguguucacuguuugccuugaGg.........................................................   1       1    HS6
    ...............................cguguucacuguuugUcuugaug.........................................................   1       1    HS6
    ...............................cguguucacuguuugccuugaugA........................................................   2       1    HS6
    ................................guguucacuguuugccuugaug.........................................................   2       0    HS6
    ..................................guucacuguuugccuugaug.........................................................   1       0    HS6
    .....................................................................Uuaaggcacgcggugaaugc......................   1       1    HS6
    .....................................................................auaaggcacgcggugaaugc......................   10      0    HS6
    .....................................................................auaaggcacgcggugaaugcc.....................   23      0    HS6
    .....................................................................auaaggcacgcggugaaugcU.....................   1       1    HS6
    .....................................................................Uuaaggcacgcggugaaugcc.....................   14      1    HS6
    .....................................................................auaaggcacgcggugaaugcca....................   64      0    HS6
    .....................................................................auaaggcacgcggugGaugcca....................   1       1    HS6
    .....................................................................Uuaaggcacgcggugaaugcca....................   54      1    HS6
    .....................................................................Guaaggcacgcggugaaugcca....................   1       1    HS6
    .....................................................................auaaggcacgcggugaUugcca....................   1       1    HS6
    .....................................................................auaaggcacgcggugaaugcAa....................   1       1    HS6
    .....................................................................auaaggcacgcggugaaugccaa...................   1       0    HS6
    .....................................................................auaaggcacgcggugaaugccaU...................   1       1    HS6
    ......................................................................uaaggcacgcggugaauA.......................   1       1    HS6
    ......................................................................uaaggcacgcggugaaug.......................   67      0    HS6
    ......................................................................uaaggcacgcggugaaGg.......................   7       1    HS6
    ......................................................................uaaCgcacgcggugaaugc......................   2       1    HS6
    ......................................................................uaaggcacgcAgugaaugc......................   1       1    HS6
    ......................................................................uaaggcacgUggugaaugc......................   3       1    HS6
    ......................................................................uaaggcacgcggugaauAc......................   1       1    HS6
    ......................................................................uaaggcacgcggugaaugc......................   2025    0    HS6
```

```
                              Star                                  Mature
gugaugcuuuguggaccuuuugugaucgcuucguguucacuguuugccuugaugucaauguuugauuucauaaggcacgcggugaaugccaagugcaaaggucaucauucg
......................................................................uaaggcaUgcggugaaugc......................  2     1   HS6
......................................................................uaaggUacgcggugaaugc......................  1     1   HS6
......................................................................uaUggcacgcggugaaugc......................  4     1   HS6
......................................................................uGaggcacgcggugaaugc......................  1     1   HS6
......................................................................uaaggcacgcggugaaugA......................  10    1   HS6
......................................................................uaaggcacgcggugaaugG......................  1     1   HS6
......................................................................uaaggcacgcggugaaugU......................  9     1   HS6
......................................................................uaaggcacgcggugaauUc......................  1     1   HS6
......................................................................uaaggcacgcggugCaugc......................  1     1   HS6
......................................................................uUaggcacgcggugaaugc......................  1     1   HS6
......................................................................uaaggcacgcggugaaugcc.....................  8918  0   HS6
......................................................................uaaggcacgcCgugaaugcc.....................  2     1   HS6
......................................................................uaaggcacgcgguAaaugcc.....................  1     1   HS6
......................................................................uaaggcacgcggugGaugcc.....................  12    1   HS6
......................................................................uaaggcaUgcggugaaugcc.....................  3     1   HS6
......................................................................uaaggcacgcggugaCugcc.....................  4     1   HS6
......................................................................uaaggcacgcggugaaGgcc.....................  1     1   HS6
......................................................................uaUggcacgcggugaaugcc.....................  17    1   HS6
......................................................................uaaggcacgcAgugaaugcc.....................  1     1   HS6
......................................................................uaaggcacgcggugaauCcc.....................  5     1   HS6
......................................................................uaaggcaGgcggugaaugcc.....................  1     1   HS6
......................................................................uaaCgcacgcggugaaugcc.....................  4     1   HS6
......................................................................uaCggcacgcggugaaugcc.....................  2     1   HS6
......................................................................uaaggcacgcggugaaCgcc.....................  1     1   HS6
......................................................................Caaggcacgcggugaaugcc.....................  7     1   HS6
......................................................................uaaggcacgcggugaGugcc.....................  4     1   HS6
......................................................................uUaggcacgcggugaaugcc.....................  2     1   HS6
......................................................................uaaggcacgcgAugaaugcc.....................  2     1   HS6
......................................................................uaaggUacgcggugaaugcc.....................  5     1   HS6
......................................................................uaaggcacgcggAgaaugcc.....................  1     1   HS6
......................................................................uaaggAacgcggugaaugcc.....................  1     1   HS6
......................................................................uaaggcUcgcggugaaugcc.....................  1     1   HS6
......................................................................uaaggcacgcggugCaugcc.....................  5     1   HS6
......................................................................uaaggcacgcggGgaaugcc.....................  1     1   HS6
......................................................................uaaggcacgcggugaaAgcc.....................  1     1   HS6
......................................................................uaaggcacgcggugaauAcc.....................  6     1   HS6
......................................................................uaaggcacgUggugaaugcc.....................  9     1   HS6
......................................................................uaaggcacgcggugaaugcU.....................  12    1   HS6
......................................................................uaaggcacgcggugUaugcc.....................  3     1   HS6
......................................................................uaagAcacgcggugaaugcc.....................  4     1   HS6
......................................................................Aaaggcacgcggugaaugcc.....................  1     1   HS6
......................................................................uaaggcacgcggugaaugGc.....................  1     1   HS6
......................................................................uaaUgcacgcggugaaugcc.....................  3     1   HS6
......................................................................uaaggcacgcggugaaugUc.....................  24    1   HS6
......................................................................uaaggcacgcggugaaugcA.....................  75    1   HS6
......................................................................uaCggcacgcggugaaugcca....................  24    1   HS6
......................................................................uUaggcacgcggugaaugcca....................  8     1   HS6
......................................................................uaaggcacgcggugaGugcca....................  28    1   HS6
......................................................................uaaggcaUgcggugaaugcca....................  21    1   HS6
......................................................................uaaggcacgcggugaaugcUa....................  9     1   HS6
......................................................................uaaggcacgcgguUaaugcca....................  1     1   HS6
......................................................................uaaggcacgcggugaaugccC....................  2     1   HS6
......................................................................uaagAcacgcggugaaugcca....................  17    1   HS6
......................................................................uaaggcacgcggugaUugcca....................  8     1   HS6
......................................................................uaaggcacgcggugaaugAca....................  4     1   HS6
......................................................................uaagUcacgcggugaaugcca....................  16    1   HS6
......................................................................uaaggcacgcgAugaaugcca....................  29    1   HS6
......................................................................uaaggcacgcgguCaaugcca....................  6     1   HS6
......................................................................uaaAgcacgcggugaaugcca....................  2     1   HS6
......................................................................uaaggcacgcggugGaugcca....................  73    1   HS6
......................................................................uaaggcacgcggugaaugccU....................  24    1   HS6
......................................................................uaaggcGcgcggugaaugcca....................  6     1   HS6
......................................................................uaaggcacgcggugaauAcca....................  104   1   HS6
......................................................................Aaaggcacgcggugaaugcca....................  6     1   HS6
......................................................................uaaCgcacgcggugaaugcca....................  28    1   HS6
......................................................................uaaggcacgcUgugaaugcca....................  3     1   HS6
......................................................................uaaggcacgcggugaaCgcca....................  10    1   HS6
......................................................................uaaggcacgcggugCaugcca....................  23    1   HS6
......................................................................uaaggcacgcggugaaugGca....................  4     1   HS6
......................................................................Caaggcacgcggugaaugcca....................  69    1   HS6
```

Star **Mature**

```
gugaugcuuuguggaccuuuugugaucgcuucguguucacuguuugccuugaugucaauguuugauuucauaaggcacgcggugaaugccaagugcaaaggucaucauucg
......................................................................uaagCcacgcggugaaugcca....................   6      1   HS6
......................................................................uaaggcacgGggugaaugcca....................   2      1   HS6
......................................................................uaaggcacUcggugaaugcca....................   4      1   HS6
......................................................................uaGggcacgcggugaaugcca....................   20     1   HS6
......................................................................uaaggcacgcgCugaaugcca....................   3      1   HS6
......................................................................uaaggcCcgcggugaaugcca....................   4      1   HS6
......................................................................uaUggcacgcggugaaugcca....................   105    1   HS6
......................................................................Gaaggcacgcggugaaugcca....................   1      1   HS6
......................................................................uGaggcacgcggugaaugcca....................   19     1   HS6
......................................................................uaaggcacgcggugUaugcca....................   31     1   HS6
......................................................................uaaggcacgcggugaaGgcca....................   5      1   HS6
......................................................................uaaggcacgcAgugaaugcca....................   15     1   HS6
......................................................................uaaggcacgcggugaCugcca....................   14     1   HS6
......................................................................uaaggcacgcggugaauUcca....................   18     1   HS6
......................................................................uaaggcacCcggugaaugcca....................   1      1   HS6
......................................................................uaaggcacgcgguAaaugcca....................   6      1   HS6
......................................................................uaaggcacgcCgugaaugcca....................   22     1   HS6
......................................................................uaaggUacgcggugaaugcca....................   40     1   HS6
......................................................................uaaggcacgcggugaaugcca....................   72126  0   HS6
......................................................................uaaggcacgcggCgaaugcca....................   4      1   HS6
......................................................................uaaUgcacgcggugaaugcca....................   11     1   HS6
......................................................................uaaggcacgcggugaaugUca....................   128    1   HS6
......................................................................uaaggcacgcggugaauCcca....................   6      1   HS6
......................................................................uaaggcacgcggugaaugcAa....................   16     1   HS6
......................................................................uaaggcacgUggugaaugcca....................   72     1   HS6
......................................................................uaaggcacgcggugaaugccG....................   47     1   HS6
......................................................................uaaggcacgcgUugaaugcca....................   2      1   HS6
......................................................................uaaggcacgcggugaaAgcca....................   2      1   HS6
......................................................................uaaggcaAgcggugaaugcca....................   1      1   HS6
......................................................................uaaggcacAcggugaaugcca....................   6      1   HS6
......................................................................uaaggcacgcggugaaugcGa....................   1      1   HS6
......................................................................uaaggcaGgcggugaaugcca....................   1      1   HS6
......................................................................uaaggcacgcggugGaugccaa...................   2      1   HS6
......................................................................uaaggcacgcggugaaugccaG...................   2      1   HS6
......................................................................uaaggcacgcUgugaaugccaa...................   1      1   HS6
......................................................................uaaggcacgcggugaaugccaU...................   496    1   HS6
......................................................................uaaggcacgcggugaauAccaa...................   1      1   HS6
......................................................................uaaggcacgUggugaaugccaa...................   3      1   HS6
......................................................................Caaggcacgcggugaaugccaa...................   1      1   HS6
......................................................................uaaggcacgcggugaaugAcaa...................   2      1   HS6
......................................................................uaaggcacgcggugaaugccaC...................   281    1   HS6
......................................................................uaaggcacgcggugaaugccaa...................   552    0   HS6
......................................................................uaaggUacgcggugaaugccaa...................   1      1   HS6
......................................................................uaUggcacgcggugaaugccaa...................   2      1   HS6
......................................................................uaaggcacgcggugaaugccaGg..................   1      1   HS6
......................................................................uaaggcacgcggugaaugccaaU..................   181    1   HS6
......................................................................uaaggcacgcggugaaugccaaC..................   1      1   HS6
......................................................................uaaggcacgcggugaaugccaaA..................   41     1   HS6
......................................................................uaaggcacgcggugaaugccaCg..................   3      1   HS6
......................................................................uaaggcacgcggugaaugccaag..................   8      0   HS6
......................................................................uaaggcacgcggugaaugccaUg..................   18     1   HS6
......................................................................uaaggcacgcggugaaugccaagG.................   556    1   HS6
......................................................................uaaggcacgcggugaaugccaUgu.................   1      1   HS6
......................................................................uaaggcacgcggugaaugccaaUu.................   47     1   HS6
......................................................................uaaggcacgcggugaaugccaagu.................   1      0   HS6
......................................................................uaaggcacgcggugaaugccaGgu.................   4      1   HS6
......................................................................uaaggcacgcggugaaugccaaCu.................   1      1   HS6
.......................................................................aaggcacgcggugaaugc......................   8      0   HS6
.......................................................................Uaggcacgcggugaaugcc.....................   1      1   HS6
.......................................................................aaggcacgcggugaaugcc.....................   18     0   HS6
.......................................................................Uaggcacgcggugaaugcca....................   13     1   HS6
.......................................................................aagCcacgcggugaaugcca....................   1      1   HS6
.......................................................................aaggcacgcggugaauAcca....................   1      1   HS6
.......................................................................aagUcacgcggugaaugcca....................   1      1   HS6
.......................................................................aaggcacgcggugaaugcUa....................   1      1   HS6
.......................................................................aaCgcacgcggugaaugcca....................   1      1   HS6
.......................................................................aaggcacgcggugaaugcca....................   156    0   HS6
.......................................................................aaggcacgcggugaaugccaC...................   1      1   HS6
.......................................................................aaggcacgcggugaaugccaa...................   1      0   HS6
.......................................................................aaggcacgcggugaaugccaagG.................   4      1   HS6
```

Star Mature

```
gugaugcuuuguggaccuuuugugaucgcuucguguucacuguuugccuugaugucaauguuugauuucauaaggcacgcggugaaugccaagugcaaaggucaucauucg
........................................................................aggcacgcggugaaugcca....................    4     0    HS6
.........................................................................ggcacgcggugaaugcca....................    4     0    HS6
..........................................................................gcacgcggugaaugccaagG.................    4     1    HS6
...........................................................................cacgcggugaaugccaagG.................    2     1    HS6

...............................cguguucacuguuugUcuug............................................................    1     1    HS2
...............................cguguucacuguuugccuug............................................................    50    0    HS2
...............................cguguucacuguuugccuuga...........................................................    61    0    HS2
...............................cguguucacuguuugccuugaug.........................................................    1889  0    HS2
...............................cguguucacuguuCgccuugaug.........................................................    1     1    HS2
...............................cguguucacuguuugccuugGug.........................................................    1     1    HS2
...............................cguguucacuguuugccuuAaug.........................................................    1     1    HS2
...............................cguguGcacuguuugccuugaug.........................................................    1     1    HS2
...............................cguguucacuguuugccuugaGg.........................................................    1     1    HS2
...............................cguguucacuguuugccuugauA.........................................................    49    1    HS2
...............................cUuguucacuguuugccuugaug.........................................................    1     1    HS2
...............................cguguucacuguuugUcuugaug.........................................................    1     1    HS2
...............................cguguucacuguuugccuugaugA........................................................    23    1    HS2
................................guguucacuguuugccuugaug.........................................................    96    0    HS2
................................guguucacuguuugccuugauA.........................................................    45    1    HS2
......................................................................Caaggcacgcggugaaug.......................    1     1    HS2
......................................................................uaaggcaUgcggugaaug.......................    1     1    HS2
......................................................................uaaggcacgcggugaaug.......................    51    0    HS2
......................................................................uaaggcacgcggugaaugc......................    120   0    HS2
......................................................................uaaggcacgcggugaaugcca....................    62    0    HS2
......................................................................uaaggcacgUggugaaugccaa...................    2     1    HS2
......................................................................uaaggcacgcggugaaugccaa...................    45    0    HS2

.............................uucguguucacuguuugccuug............................................................    3     0    HA1
...............................cguguucacuguuugccuug............................................................    22    0    HA1
...............................cguguucacuUuuugccuuga...........................................................    1     1    HA1
...............................cguguucacuguuugccuuga...........................................................    23    0    HA1
...............................cguguucacuguuugccuugau..........................................................    24    0    HA1
...............................cguguucacuguuugccuugaA..........................................................    1     1    HA1
...............................cguguucacuguuugccuugCug.........................................................    1     1    HA1
...............................cguguCcacuguuugccuugaug.........................................................    1     1    HA1
...............................cguguucacuguuugccuugauA.........................................................    2     1    HA1
...............................cguguucacuguuugccuugaug.........................................................    291   0    HA1
...............................cguguucacuguuugccuugGug.........................................................    1     1    HA1
...............................cguguucacuguuugccuugauU.........................................................    1     1    HA1
...............................cguguucacuguuugccuugaugA........................................................    3     1    HA1
................................guguucacuguuugccuugaug.........................................................    1     0    HA1
............................................................uuugauuucauaaggcacgcggu............................    1     0    HA1
...................................................................ucauaaggcacgcggugaaugc......................    1     0    HA1
.....................................................................auaaggcacgcggugaaug.......................    7     0    HA1
.....................................................................auaaggcacgcggugaauU.......................    1     1    HA1
.....................................................................Uuaaggcacgcggugaaugc......................    12    1    HA1
.....................................................................auaaggcacgcggugaaugc......................    18    0    HA1
.....................................................................auaaggcacgcggugaaugcA.....................    1     1    HA1
.....................................................................auaaggcacgcggugaaugcc.....................    41    0    HA1
.....................................................................Uuaaggcacgcggugaaugcc.....................    1     1    HA1
.....................................................................auaaggcaGgcggugaaugcc.....................    1     1    HA1
.....................................................................auaaggcacgcggugaaugcU.....................    1     1    HA1
.....................................................................auaaggcacgcggugGaugcca....................    1     1    HA1
.....................................................................auaaggcacgcggugaaugcca....................    73    0    HA1
.....................................................................auaaggcacgcggugaaugUca....................    3     1    HA1
.....................................................................auaaggcacgcggugaaugcUa....................    1     1    HA1
.....................................................................auaaggcacgcggugaaugccU....................    1     1    HA1
.....................................................................Uuaaggcacgcggugaaugcca....................    6     1    HA1
.....................................................................auaaggcacgcggugaaugccaC...................    1     1    HA1
.....................................................................auaaggcacgcggugaaugccaU...................    1     1    HA1
.....................................................................Uuaaggcacgcggugaaugccaa...................    3     1    HA1
.....................................................................auaaggcacgcggugaaugccaUg..................    1     1    HA1
.....................................................................auaaggcacgcggugaaugccaaU..................    1     1    HA1
......................................................................uaaggcacgcggugaauU.......................    4     1    HA1
......................................................................uaaggcacgcggugaaug.......................    42    0    HA1
......................................................................uaaggcUcgcggugaaugc......................    5     1    HA1
......................................................................uaaggcacgcggGgaaugc......................    1     1    HA1
......................................................................uaaggcacgcgguAaaugc......................    1     1    HA1
......................................................................uaaggUacgcggugaaugc......................    6     1    HA1
```

Star   Mature

gugaugcuuuguggaccuuuugugaucgcuucguguucacuguuugccuugaugucaauguuugauuucauaaggcacgcggugaaugccaagugcaaaggucaucauucg			
....uaaggcacCcggugaaugc......................	1	1	HA1
....Gaaggcacgcggugaaugc......................	4	1	HA1
....uaaggcacgcggugaaugA......................	274	1	HA1
....uaaggcacgcAgugaaugc......................	3	1	HA1
....uaaggcacUcggugaaugc......................	4	1	HA1
....uGaggcacgcggugaaugc......................	3	1	HA1
....uaaggcacgcggugUaugc......................	2	1	HA1
....uaaggcacgcggugaaugU......................	51	1	HA1
....uaagAcacgcggugaaugc......................	5	1	HA1
....uaaggcacgUggugaaugc......................	3	1	HA1
....uaaggcacgcggugGaugc......................	4	1	HA1
....uaaggcacgcggugCaugc......................	1	1	HA1
....uaaggcacgAggugaaugc......................	1	1	HA1
....uaaggcacgcggugaUugc......................	2	1	HA1
....uaagUcacgcggugaaugc......................	1	1	HA1
....uaaggcacgcgCugaaugc......................	3	1	HA1
....uaaggcacgcggugaauUc......................	1	1	HA1
....uaaggcacgcggugaaugc......................	6751	0	HA1
....uaUggcacgcggugaaugc......................	2	1	HA1
....uaaggcacgcUgugaaugc......................	2	1	HA1
....uaaAgcacgcggugaaugc......................	4	1	HA1
....uaaggcacAcggugaaugc......................	2	1	HA1
....uaaggcaUgcggugaaugc......................	4	1	HA1
....uaaggcacgcgguCaaugc......................	2	1	HA1
....uaaggcacgcggugaGugc......................	1	1	HA1
....uaaggcGcgcggugaaugc......................	1	1	HA1
....Caaggcacgcggugaaugc......................	3	1	HA1
....uaaggcacgcggCgaaugc......................	2	1	HA1
....uaaggcacgcgguUaaugc......................	2	1	HA1
....uaCggcacgcggugaaugc......................	1	1	HA1
....uaGggcacgcggugaaugc......................	4	1	HA1
....uaaggcacgcggugaaugG......................	2	1	HA1
....uaaggcacgcggugaCugc......................	2	1	HA1
....uUaggcacgcggugaaugc......................	3	1	HA1
....uaaggcacgcggugaaCgc......................	1	1	HA1
....uaaggcaGgcggugaaugc......................	1	1	HA1
....uaaggcacgcggugaaGgc......................	1	1	HA1
....uaaggcacgcAgugaaugcc.....................	1	1	HA1
....uaaAgcacgcggugaaugcc.....................	2	1	HA1
....Gaaggcacgcggugaaugcc.....................	2	1	HA1
....uaaggcacgcggugCaugcc.....................	1	1	HA1
....uaaggcacgcggugaaCgcc.....................	1	1	HA1
....uaaggcUcgcggugaaugcc.....................	1	1	HA1
....uaaggcacgcggugaaugUc.....................	38	1	HA1
....uaaggUacgcggugaaugcc.....................	7	1	HA1
....uaaggcacgcggugaaugcA.....................	627	1	HA1
....uaaggcacgUggugaaugcc.....................	2	1	HA1
....uaaggcacgcggugaaugcU.....................	70	1	HA1
....uaaggcacgcggugaaGgcc.....................	2	1	HA1
....uaaggcacUcggugaaugcc.....................	1	1	HA1
....uaaggcacgcggugGaugcc.....................	1	1	HA1
....uaaggcacgcggugaauAcc.....................	1	1	HA1
....uaagCcacgcggugaaugcc.....................	2	1	HA1
....uaaggcacgcggugaaugcG.....................	8	1	HA1
....uaaggcacgcgguAaaugcc.....................	1	1	HA1
....uaaggcacgcggugaUugcc.....................	1	1	HA1
....uaaggcCcgcggugaaugcc.....................	1	1	HA1
....uaaggcacgcggugaaugcc.....................	1659	0	HA1
....uaaggcacgAggugaaugcc.....................	1	1	HA1
....uaCggcacgcggugaaugcc.....................	1	1	HA1
....uaaUgcacgcggugaaugcc.....................	4	1	HA1
....uaaggGacgcggugaaugcc.....................	1	1	HA1
....uaaggcacgcggugaauCcc.....................	19	1	HA1
....uaaggcacgcggugaCugcc.....................	1	1	HA1
....uaaggcacgcggugaauUcc.....................	4	1	HA1
....uaaggcacgcggugaGugcc.....................	1	1	HA1
....uaaggcaUgcggugaaugcca....................	2	1	HA1
....uaaggcacgcggCgaaugcca....................	3	1	HA1
....uaaAgcacgcggugaaugcca....................	3	1	HA1
....uaaggcacgcggugaaugGca....................	1	1	HA1

```
                               Star                                 Mature
gugaugcuuuguggaccuuuugugaucgcuucguguucacuguuugccuugaugucaauguuugauuucauaaggcacgcggugaaugccaagugcaaaggucaucauucg
......................................................................uaaggcUcgcggugaaugcca....................    4     1    HA1
......................................................................uaaggcacgcggugaUugcca....................    2     1    HA1
......................................................................uaaggcacgcggugaaugccG....................    3     1    HA1
......................................................................uaaggcacgAggugaaugcca....................    3     1    HA1
......................................................................uGaggcacgcggugaaugcca....................    9     1    HA1
......................................................................uUaggcacgcggugaaugcca....................    2     1    HA1
......................................................................uaaggcacgUggugaaugcca....................    11    1    HA1
......................................................................uaaggcacgcAgugaaugcca....................    5     1    HA1
......................................................................uaaggcacgcgguCaaugcca....................    3     1    HA1
......................................................................uaaggcacgcgguUaaugcca....................    2     1    HA1
......................................................................uaaggcacgcggugaaugcUa....................    18    1    HA1
......................................................................uaagUcacgcggugaaugcca....................    2     1    HA1
......................................................................uaaggUacgcggugaaugcca....................    17    1    HA1
......................................................................uaaggcacgcggugaaugccC....................    2     1    HA1
......................................................................uaaggcacgcggugCaugcca....................    2     1    HA1
......................................................................uaaggcacgcggugaaGgcca....................    3     1    HA1
......................................................................uaaggcacgcggugaauUcca....................    6     1    HA1
......................................................................uaaggcacgcggugaaugUca....................    219   1    HA1
......................................................................uaaggcacgcggugGaugcca....................    5     1    HA1
......................................................................uaCggcacgcggugaaugcca....................    2     1    HA1
......................................................................uaaggcacCcggugaaugcca....................    4     1    HA1
......................................................................uaGggcacgcggugaaugcca....................    5     1    HA1
......................................................................uaaggcacgcggugaaugAca....................    2     1    HA1
......................................................................uaaggcacgcggugaaugcAa....................    52    1    HA1
......................................................................uaaggcacgcggugaaAgcca....................    2     1    HA1
......................................................................uaUggcacgcggugaaugcca....................    1     1    HA1
......................................................................uaaggcacgcggugaauAcca....................    12    1    HA1
......................................................................uaaggcacgcgguAaaugcca....................    2     1    HA1
......................................................................uaaggcacAcggugaaugcca....................    3     1    HA1
......................................................................uaagAcacgcggugaaugcca....................    1     1    HA1
......................................................................uaaggcacUcggugaaugcca....................    1     1    HA1
......................................................................Gaaggcacgcggugaaugcca....................    1     1    HA1
......................................................................uaaggcacgcggugaaCgcca....................    3     1    HA1
......................................................................uaaggcacgcgCugaaugcca....................    3     1    HA1
......................................................................uaaCgcacgcggugaaugcca....................    17    1    HA1
......................................................................uaagCcacgcggugaaugcca....................    4     1    HA1
......................................................................uaaggcacgcggugaaugccU....................    4     1    HA1
......................................................................uaaggcacgcCgugaaugcca....................    3     1    HA1
......................................................................uaaggcacgcgAugaaugcca....................    7     1    HA1
......................................................................uaaUgcacgcggugaaugcca....................    22    1    HA1
......................................................................uaaggcacgcggugaaugcca....................    8906  0    HA1
......................................................................uaaggcGcgcggugaaugcca....................    8     1    HA1
......................................................................uCaggcacgcggugaaugcca....................    1     1    HA1
......................................................................uaaggcacgcUgugaaugcca....................    2     1    HA1
......................................................................Caaggcacgcggugaaugcca....................    2     1    HA1
......................................................................uaaggcacgcggugaauCcca....................    16    1    HA1
......................................................................uaaggcacgcggugaCugcca....................    1     1    HA1
......................................................................uaaggcaUgcggugaaugccaa..................    4     1    HA1
......................................................................uaaCgcacgcggugaaugccaa..................    1     1    HA1
......................................................................uaaggcacgcggugaaugcUaa..................    1     1    HA1
......................................................................uaaggcacgcggugaaugUcaa..................    5     1    HA1
......................................................................uaUggcacgcggugaaugccaa..................    2     1    HA1
......................................................................uaaggcCcgcggugaaugccaa..................    1     1    HA1
......................................................................uaaggcacgcggugaaugccaU..................    4507  1    HA1
......................................................................uaaggcacgcggGgaaugccaa..................    1     1    HA1
......................................................................uaaggcacgcgAugaaugccaa..................    3     1    HA1
......................................................................uaaggcacgcggugaauCccaa..................    1     1    HA1
......................................................................uaaggcacgcAgugaaugccaa..................    1     1    HA1
......................................................................uaaggcacgcggugGaugccaa..................    2     1    HA1
......................................................................uaaggcacgcggugCaugccaa..................    1     1    HA1
......................................................................uaaggcacgUggugaaugccaa..................    8     1    HA1
......................................................................uaaggcacgcggugaauAccaa..................    5     1    HA1
......................................................................uaaggcacgcggugaaugccaC..................    1268  1    HA1
......................................................................uaGggcacgcggugaaugccaa..................    3     1    HA1
......................................................................uaaggUacgcggugaaugccaa..................    1     1    HA1
......................................................................uaaggcacgcggugaaugccaG..................    3     1    HA1
......................................................................uaaggcacUcggugaaugccaa..................    2     1    HA1
......................................................................uaaggcacgcggugaCugccaa..................    1     1    HA1
......................................................................uaaggcacgcggugaaugccGa..................    1     1    HA1
......................................................................uGaggcacgcggugaaugccaa..................    2     1    HA1
```

Star Mature

gugaugcuuuguggaccuuuugugaucgcuucguguucacuguuugccuugaugucaauguuugauuucauaaggcacgcggugaaugccaagugcaaaggucaucauucg			
....uaaggcacgcggugaaugccCa...................	2	1	HA1
....uaaggcacgcggugaaugcAaa...................	1	1	HA1
....uaaggcUcgcggugaaugccaa...................	2	1	HA1
....uaCggcacgcggugaaugccaa...................	1	1	HA1
....Caaggcacgcggugaaugccaa...................	2	1	HA1
....uaaggcacgcggugaaugccaa...................	3447	0	HA1
....uaaAgcacgcggugaaugccaa...................	1	1	HA1
....uaaggcacgcggugaaugccaag..................	10	0	HA1
....uaaggcacgcggugaaugccaaU..................	617	1	HA1
....uaaggcacgcggugaaugccaGg..................	2	1	HA1
....uaaggcacgcggugaaugccaUg..................	57	1	HA1
....uaaggcacgcggugaaugccaaC..................	20	1	HA1
....uaaggcacgcggugaaugccaaA..................	278	1	HA1
....uaaggcacgcggugaaugccaaCu.................	2	1	HA1
....uaaggcacgcggugaaugccaUgu.................	6	1	HA1
....uaaggcacgcggugaaugccaagG.................	45	1	HA1
....uaaggcacgcggugaaugccaaUu.................	215	1	HA1
....uaaggcacgcggugaaugccaaAu.................	4	1	HA1
....uaaggcacgcggugaaugccaagu.................	1	0	HA1
....uaaggcacgcggugaaugccaaguC................	4	1	HA1
....aaggcacgcggugaaugc......................	25	0	HA1
....aaggcacgcggugaaAgc......................	1	1	HA1
....aaggcacgcggGgaaugc......................	1	1	HA1
....aaggcacgcggugGaugc......................	1	1	HA1
....aaggcacgcggugaaugcA.....................	1	1	HA1
....aaggcacgcggugaaugcc.....................	3	0	HA1
....aaggcacgcggugGaugcca....................	1	1	HA1
....aaAgcacgcggugaaugcca....................	1	1	HA1
....aaggcacgcggugaaugcca....................	15	0	HA1
....aaggcacgcggugaaugUca....................	2	1	HA1
....aaUgcacgcggugaaugcca....................	1	1	HA1
....aaggcacgcggugaaugccaa...................	10	0	HA1
....aaggcacgcggugaaugccaU...................	14	1	HA1
....aaggcacgcggugaaugccaaU..................	3	1	HA1
....aggcacgcggugaaugcA.....................	2	1	HA1
....aUgcacgcggugaaugcca....................	1	1	HA1
....aggcacgcggugaaugcca....................	2	0	HA1
....ggcacgcggugaaugcca....................	2	0	HA1
....gcacgcggugaaugccaa...................	1	0	HA1
....cacgcggugaaugccaaA..................	1	1	HA1
....cacgcggugaaugccaaUu.................	1	1	HA1
.....gcuuuguggaccuuuugugaucgcuu..	5	0	HS1
.....gcuuuguggaccuuuugugaucgcuC..	2	1	HS1
......cuuuguggaccuuuugugaucgcuu..	3	0	HS1
.......uuuguggaccuuuugugaucgcuu..	8	0	HS1
..............................Ccguguucacuguuugccuug..	4	1	HS1
..............................Acguguucacuguuugccuuga...	5	1	HS1
..............................Ccguguucacuguuugccuugaug...	62	1	HS1
...............................cguguucacuguuugccu..	2	0	HS1
...............................cguguucacuguuugccuG...	2	1	HS1
...............................cguguucUcuguuugccuug..	1	1	HS1
...............................cguguuGacuguuugccuug..	1	1	HS1
...............................cAuguucacuguuugccuug..	3	1	HS1
...............................cguguucGcuguuugccuug..	1	1	HS1
...............................cguguucacuguuugccuug..	1376	0	HS1
...............................cguguucaUuguuugccuug..	1	1	HS1
...............................cguguucacuguuugccuAg..	1	1	HS1
...............................cguguucacuguuugUcuug..	3	1	HS1
...............................Uguguucacuguuugccuug..	2	1	HS1
...............................cguguucacuguuugccuuA..	4	1	HS1
...............................cgGguucacuguuugccuug..	1	1	HS1
...............................cguguCcacuguuugccuuga...	1	1	HS1
...............................cguguucacugCuugccuuga...	1	1	HS1
...............................cgugAucacuguuugccuuga...	1	1	HS1
...............................cguguucacuguuugccuugU...	6	1	HS1
...............................cguguucacuguuugccCuga...	1	1	HS1
...............................cguguucacuguuugccuuga...	2646	0	HS1
...............................Gguguucacuguuugccuuga...	1	1	HS1
...............................cguguucacuguCugccuuga...	2	1	HS1

```
                              Star                                  Mature
gugaugcuuuguggaccuuuugugaucgcuucguguucacuguuugccuugaugucaauguuugauuucauaaggcacgcggugaaugccaagugcaaaggucaucauucg
...............................cgugCucacuguuugccuuga...........................................................	2	1	HS1
...............................cguguucaUuguuugccuuga...........................................................	1	1	HS1
...............................cAuguucacuguuugccuuga...........................................................	3	1	HS1
...............................cguguucacCguuugccuuga...........................................................	2	1	HS1
...............................cguguucacugGuugccuuga...........................................................	1	1	HS1
...............................cgugGucacuguuugccuuga...........................................................	2	1	HS1
...............................cguAuucacuguuugccuuga...........................................................	1	1	HS1
...............................cguguucacuguuugUcuuga...........................................................	8	1	HS1
...............................cguguucacCguuugccuugau..........................................................	1	1	HS1
...............................cguguucacugCuugccuugau..........................................................	1	1	HS1
...............................cguguucacuguuGgccuugau..........................................................	1	1	HS1
...............................cguguucacuguuCgccuugau..........................................................	1	1	HS1
...............................cguguucacuguuugccuugaC..........................................................	5	1	HS1
...............................cguguucacuguuugccuugGu..........................................................	1	1	HS1
...............................cguguucacuguuugccuugau..........................................................	1345	0	HS1
...............................cguguucacuguuugccAugau..........................................................	2	1	HS1
...............................cguguGcacuguuugccuugau..........................................................	1	1	HS1
...............................cguguucacuguuugUcuugau..........................................................	1	1	HS1
...............................cguguucacuguuugccuugaA..........................................................	1	1	HS1
...............................cguguucacuguuuCccuugau..........................................................	1	1	HS1
...............................cguguuAacuguuugccuugaug.........................................................	3	1	HS1
...............................cguguucacuguAugccuugaug.........................................................	1	1	HS1
...............................cgugCucacuguuugccuugaug.........................................................	30	1	HS1
...............................cguguucacuguCugccuugaug.........................................................	9	1	HS1
...............................cguguucaGuguuugccuugaug.........................................................	1	1	HS1
...............................cgCguucacuguuugccuugaug.........................................................	14	1	HS1
...............................cguguGcacuguuugccuugaug.........................................................	4	1	HS1
...............................cguguucacuguuugccuugaCg.........................................................	18	1	HS1
...............................cguguuUacuguuugccuugaug.........................................................	6	1	HS1
...............................cguguucacuguuugccuCgaug.........................................................	11	1	HS1
...............................cguguucacugGuugccuugaug.........................................................	4	1	HS1
...............................cguguucaAuguuugccuugaug.........................................................	1	1	HS1
...............................cguguCcacuguuugccuugaug.........................................................	21	1	HS1
...............................cguguucacuguuugGcuugaug.........................................................	2	1	HS1
...............................cguguucCcuguuugccuugaug.........................................................	2	1	HS1
...............................cguguucacuguuugccuugauC.........................................................	11	1	HS1
...............................Uguguucacuguuugccuugaug.........................................................	13	1	HS1
...............................cguguucacuguuuAccuugaug.........................................................	25	1	HS1
...............................cguguuGacuguuugccuugaug.........................................................	5	1	HS1
...............................cguguucacuguuugccuugauU.........................................................	239	1	HS1
...............................cguguucacuguuugCuuugaug.........................................................	9	1	HS1
...............................cguguucacuguuugccuuUaug.........................................................	2	1	HS1
...............................cguguucacuAuuugccuugaug.........................................................	6	1	HS1
...............................cguguucacuguuugccCugaug.........................................................	19	1	HS1
...............................cUguucacuguuugccuugaug..........................................................	3	1	HS1
...............................cguguucUcuguuugccuugaug.........................................................	1	1	HS1
...............................cgugAucacuguuugccuugaug.........................................................	2	1	HS1
...............................cAuguucacuguuugccuugaug.........................................................	88	1	HS1
...............................cguguucacuguuugccuugCug.........................................................	2	1	HS1
...............................cguguucacuguuugAcuugaug.........................................................	2	1	HS1
...............................cguguucacuguuCgccuugaug.........................................................	8	1	HS1
...............................cguguucacuguuugUcuugaug.........................................................	136	1	HS1
...............................cgGguucacuguuugccuugaug.........................................................	2	1	HS1
...............................cguguucaUuguuugccuugaug.........................................................	4	1	HS1
...............................cguguucacuguuugccuugaug.........................................................	51999	0	HS1
...............................cguguucacuguuGgccuugaug.........................................................	6	1	HS1
...............................cguguucacuguuugccuugaAg.........................................................	26	1	HS1
...............................cguguucacuguuugccuugaGg.........................................................	7	1	HS1
...............................cguguucacuguuugccuGgaug.........................................................	1	1	HS1
...............................cguguucacuguuugccuugUug.........................................................	1	1	HS1
...............................cguguucacuguuugccuugGug.........................................................	9	1	HS1
...............................cgugGucacuguuugccuugaug.........................................................	7	1	HS1
...............................cguguucacGguuugccuugaug.........................................................	1	1	HS1
...............................cguguucGcuguuugccuugaug.........................................................	14	1	HS1
...............................cguguucacuguuugccuugauA.........................................................	160	1	HS1
...............................cguguucacuguuugccuuAaug.........................................................	4	1	HS1
...............................cguguucacuguGugccuugaug.........................................................	2	1	HS1
...............................cguguAcacuguuugccuugaug.........................................................	1	1	HS1
...............................cguguucacuguuugccuAgaug.........................................................	2	1	HS1
...............................cguAuucacuguuugccuugaug.........................................................	8	1	HS1
```

```
                              Star                                   Mature
gugaugcuuuguggaccuuuugugaucgcuucguguucacuguuugccuugaugucaauguuugauuucauaaggcacgcggugaaugccaagugcaaaggucaucauucg
...............................cguguucacuguuugccAugaug.........................................................   4      1    HS1
...............................cguguucacugCuugccuugaug.........................................................   10     1    HS1
...............................cguUuucacuguuugccuugaug.........................................................   3      1    HS1
...............................cguguucacuguuAgccuugaug.........................................................   1      1    HS1
...............................Gguguucacuguuugccuugaug.........................................................   2      1    HS1
...............................cguguucacuguuuUccuugaug.........................................................   3      1    HS1
...............................cguguucacCguuugccuugaug.........................................................   5      1    HS1
...............................Aguguucacuguuugccuugaug.........................................................   3      1    HS1
...............................cguguucacuguuugccuugaugu........................................................   33     0    HS1
...............................cguguucacuguuugccuugaugA........................................................   794    1    HS1
...............................cguguucacuguuugccuugauguc.......................................................   6      0    HS1
...............................cguguucacuguuugccuugauguU.......................................................   2      1    HS1
...............................cguguucacuguuugccuugauguca......................................................   13     0    HS1
...............................cguguucacuguuugccuugaugucaa.....................................................   2      0    HS1
................................guguCcacuguuugccuug............................................................   1      1    HS1
................................guguucacuguuugccuug............................................................   38     0    HS1
................................guguucacuguuugccuuga...........................................................   115    0    HS1
................................Uuguucacuguuugccuuga...........................................................   1      1    HS1
................................guguucacuguuugUcuuga...........................................................   1      1    HS1
................................guguCcacuguuugccuuga...........................................................   1      1    HS1
................................guguucacuguuugccuugau..........................................................   79     0    HS1
................................guguucacuguuugccAugaug.........................................................   1      1    HS1
................................guguucacuAuuugccuugaug.........................................................   1      1    HS1
................................guguucacuguuugccuugauU.........................................................   16     1    HS1
................................guguucacuguuugccuugauA.........................................................   5      1    HS1
................................guguucacuguuuAccuugaug.........................................................   2      1    HS1
................................guguucacuguuugcUuugaug.........................................................   1      1    HS1
................................guguucacuguuugccuugaug.........................................................   1839   0    HS1
................................guguCcacuguuugccuugaug.........................................................   1      1    HS1
................................guguucGcuguuugccuugaug.........................................................   2      1    HS1
................................guguucacuguuugUcuugaug.........................................................   8      1    HS1
................................Uuguucacuguuugccuugaug.........................................................   2      1    HS1
................................Auguucacuguuugccuugaug.........................................................   1      1    HS1
................................guguucacuguuCgccuugaug.........................................................   2      1    HS1
................................guguucacuguuugccuugaugA........................................................   23     1    HS1
................................guguucacuguuugccuugaugu........................................................   4      0    HS1
.................................uguucacuguuugccuugaug.........................................................   10     0    HS1
...................................uucacuguuugccuugaug.........................................................   12     0    HS1
....................................ucacuguuugccuugaug.........................................................   6      0    HS1
......................................................................uaaggcacgcggugaaug.......................   14     0    HS1
......................................................................uaaggcacgUggugaaugc......................   3      1    HS1
......................................................................uaaggcacgcggugaaugc......................   17     0    HS1
......................................................................uaaggcacgcggugaaugcc.....................   15     0    HS1
......................................................................uaaggcacgcgguAaaugcc.....................   1      1    HS1
......................................................................uaaggcacgcggugaaugcca....................   50     0    HS1
......................................................................uaaAgcacgcggugaaugcca....................   1      1    HS1
......................................................................uaaggcacgcggugaaugcUa....................   1      1    HS1
......................................................................uaaggcacgcggugaaugccaa...................   28     0    HS1
......................................................................uaaggcacgcggugaaugccaaC..................   3      1    HS1
......................................................................uaaggcacgcggugaaugccaaA..................   3      1    HS1
.......................................................................aaggcacgcggugaaugc......................   3      0    HS1
.......................................................................aaggcacgcggugaaugcca....................   7      0    HS1
.......................................................................aaggcacgcggugaaugccaa...................   8      0    HS1

.......uuuguggaccuuuugugaucgcuu................................................................................   1      0    HS7
........................aucgcuucguguucacuguuugcc...............................................................   1      0    HS7
.............................uucguguucacuguuugccuug............................................................   1      0    HS7
...............................cguguucacuguuugccuu.............................................................   1      0    HS7
...............................cguguucacuguuugccuug............................................................   20     0    HS7
...............................cguguucacuguuugccuuga...........................................................   4      0    HS7
...............................cguguucacuguuugcUuuga...........................................................   1      1    HS7
...............................cguguucacuguuugccuugau..........................................................   17     0    HS7
...............................cgCguucacuguuugccuugaug.........................................................   1      1    HS7
...............................cguguucaUuguuugccuugaug.........................................................   1      1    HS7
...............................cguguucacuguuugccuugaug.........................................................   424    0    HS7
...............................cguguucacuguuugccuugaugA........................................................   4      1    HS7
...............................cguguucacuguuugccuugauguc.......................................................   3      0    HS7
................................guguucacuguuugccuugaug.........................................................   2      0    HS7
..................................guucacuguuugccuugaug.........................................................   1      0    HS7
...................................uucacuguuugccuugaug.........................................................   2      0    HS7
```

Star **Mature**

gugaugcuuuguggaccuuuugugaucgcuucguguucacuguuugccuugaugucaauguuugauuucauaaggcacgcggugaaugccaagugcaaaggucaucauucg			
....uuugauuucauaaggcacgcggu............................	1	0	HS7
....auaaggcacgcggugaaugc......................	8	0	HS7
....Uuaaggcacgcggugaaugc......................	6	1	HS7
....auaagAcacgcggugaaugcc.....................	1	1	HS7
....Uuaaggcacgcggugaaugcc.....................	17	1	HS7
....auaaggcacgcggugaaugcc.....................	30	0	HS7
....Uuaaggcacgcggugaaugcca....................	41	1	HS7
....auUaggcacgcggugaaugcca....................	1	1	HS7
....Guaaggcacgcggugaaugcca....................	1	1	HS7
....auaaggcacgcggugaaugcca....................	67	0	HS7
....auaaggcacgcggugaaugccaaU..................	1	1	HS7
....uaaggcacgcggugaaug.......................	90	0	HS7
....uaaggcacgcggugaaGg.......................	6	1	HS7
....uaaggcacgUggugaaug.......................	1	1	HS7
....uaaggcacgcggugaauU.......................	2	1	HS7
....uaaggcacgcggugaaugA......................	12	1	HS7
....uaagAcacgcggugaaugc......................	3	1	HS7
....uaaggcacgcggugaaugU......................	8	1	HS7
....uaaggcaUgcggugaaugc......................	3	1	HS7
....uaaggcacgcggugaaugc......................	2393	0	HS7
....Gaaggcacgcggugaaugc......................	1	1	HS7
....uaCggcacgcggugaaugc......................	1	1	HS7
....uaaggcacgcCgugaaugc......................	1	1	HS7
....uaUggcacgcggugaaugc......................	4	1	HS7
....uaaggcacCcggugaaugc......................	1	1	HS7
....uaaggUacgcggugaaugc......................	1	1	HS7
....uaaggcacAcggugaaugc......................	1	1	HS7
....uaaggcacgcggugaaugG......................	1	1	HS7
....uaaggcacgcgguUaaugc......................	1	1	HS7
....uaaggcacgcggugaauUc......................	3	1	HS7
....uaaggcacgcAgugaaugc......................	1	1	HS7
....uaaggcacgcUgugaaugc......................	1	1	HS7
....uaaggcacgUggugaaugc......................	4	1	HS7
....Caaggcacgcggugaaugcc.....................	4	1	HS7
....uaaggUacgcggugaaugcc.....................	12	1	HS7
....uaaggcacgcggugaaugcA.....................	75	1	HS7
....uaaggcacgcggugaaugcU.....................	17	1	HS7
....uaaCgcacgcggugaaugcc.....................	8	1	HS7
....uaaggcacgcggugaaCgcc.....................	3	1	HS7
....uaaggcacUcggugaaugcc.....................	1	1	HS7
....uaaggcaGgcggugaaugcc.....................	3	1	HS7
....uGaggcacgcggugaaugcc.....................	1	1	HS7
....uaaggcacgcggugaauAcc.....................	7	1	HS7
....uaaggcacgcggugGaugcc.....................	6	1	HS7
....uaaggcacgcggCgaaugcc.....................	2	1	HS7
....uaUggcacgcggugaaugcc.....................	19	1	HS7
....uUaggcacgcggugaaugcc.....................	1	1	HS7
....uaagCcacgcggugaaugcc.....................	2	1	HS7
....uaagUcacgcggugaaugcc.....................	2	1	HS7
....uaaggcacgcggugaGugcc.....................	2	1	HS7
....uaaggcacgcgguCaaugcc.....................	3	1	HS7
....uaaggcacgcgAugaaugcc.....................	2	1	HS7
....uaaggcacgcggugaauUcc.....................	1	1	HS7
....uaaggcacgcAgugaaugcc.....................	3	1	HS7
....uaaggcacgcggugaaugUc.....................	33	1	HS7
....uaaggcacgcUgugaaugcc.....................	2	1	HS7
....uaaggcacgcgguUaaugcc.....................	1	1	HS7
....uaaUgcacgcggugaaugcc.....................	10	1	HS7
....uaaggcacgcgguAaaugcc.....................	1	1	HS7
....uaaggcacgUggugaaugcc.....................	14	1	HS7
....uaaggcacgcggugaaugcc.....................	10106	0	HS7
....uaaggcacgcCgugaaugcc.....................	2	1	HS7
....uaaggcaUgcggugaaugcc.....................	4	1	HS7
....uaaggcacgcggugaUugcc.....................	1	1	HS7
....uaaggcacgcggugaauCcc.....................	1	1	HS7
....uaGggcacgcggugaaugcc.....................	1	1	HS7
....uaCggcacgcggugaaugcc.....................	1	1	HS7
....uaaggcacgcggugCaugcc.....................	2	1	HS7
....uaagAcacgcggugaaugcc.....................	4	1	HS7
....uaaggcacgcgCugaaugcc.....................	3	1	HS7

Star Mature

```
gugaugcuuuguggaccuuuugugaucgcuuccguguucacuguuugccuugaugucaauguuugauuucauaaggcacgcggugaaugccaagugcaaaggucaucauucg
.......................................................................uaaggcacgcggugaaugUca....................   116     1   HS7
.......................................................................uaaAgcacgcggugaaugcca....................   2       1   HS7
.......................................................................uaagAcacgcggugaaugcca....................   28      1   HS7
.......................................................................uaaggcacgcggugaUugcca....................   2       1   HS7
.......................................................................uaaggcacgcggugaGugcca....................   6       1   HS7
.......................................................................uaaCgcacgcggugaaugcca....................   40      1   HS7
.......................................................................uaGggcacgcggugaaugcca....................   19      1   HS7
.......................................................................uaaggcacgUggugaaugcca....................   77      1   HS7
.......................................................................uaaggcaGgcggugaaugcca....................   10      1   HS7
.......................................................................uGaggcacgcggugaaugcca....................   9       1   HS7
.......................................................................uaaggcacgcUgugaaugcca....................   2       1   HS7
.......................................................................uaaggcacgcgAugaaugcca....................   11      1   HS7
.......................................................................uaaggcacgcggugaaugcca....................   63824   0   HS7
.......................................................................uaaggcacgcgguCaaugcca....................   4       1   HS7
.......................................................................uaaggcacgcggugaaugcGa....................   1       1   HS7
.......................................................................uaaggcacCcggugaaugcca....................   1       1   HS7
.......................................................................uaaggcacgcggugaCugcca....................   1       1   HS7
.......................................................................uaUggcacgcggugaaugcca....................   88      1   HS7
.......................................................................uaCggcacgcggugaaugcca....................   19      1   HS7
.......................................................................uaaggcacgcggugaaugcAa....................   10      1   HS7
.......................................................................uUaggcacgcggugaaugcca....................   17      1   HS7
.......................................................................uaaggcacgcggugGaugcca....................   15      1   HS7
.......................................................................uaaggcacgcggugUaugcca....................   8       1   HS7
.......................................................................uaaggcacgcggugaaGgcca....................   4       1   HS7
.......................................................................uaaggcacgcgCugaaugcca....................   1       1   HS7
.......................................................................uaaggcacgcgguUaaugcca....................   2       1   HS7
.......................................................................uaaggcacgcgUugaaugcca....................   1       1   HS7
.......................................................................uaaggcacgcggugaaugccC....................   2       1   HS7
.......................................................................uaaggcacgcggugaauCcca....................   16      1   HS7
.......................................................................uaaggcacgcggugaaugAca....................   1       1   HS7
.......................................................................uaaggcacgcggCgaaugcca....................   7       1   HS7
.......................................................................uaaggcacgcggugaaugccU....................   20      1   HS7
.......................................................................uaaggcacAcggugaaugcca....................   4       1   HS7
.......................................................................uaaggcacgcggugaaugccG....................   59      1   HS7
.......................................................................uaaggcacgcCgugaaugcca....................   8       1   HS7
.......................................................................uaaggcGcgcggugaaugcca....................   4       1   HS7
.......................................................................Caaggcacgcggugaaugcca....................   5       1   HS7
.......................................................................uaaggcaUgcggugaaugcca....................   18      1   HS7
.......................................................................uaaggcacUcggugaaugcca....................   6       1   HS7
.......................................................................uaagUcacgcggugaaugcca....................   10      1   HS7
.......................................................................Aaaggcacgcggugaaugcca....................   1       1   HS7
.......................................................................uaaggcUcgcggugaaugcca....................   3       1   HS7
.......................................................................uaaggcacgcggugaaCgcca....................   10      1   HS7
.......................................................................uaaggcacgcggGgaaugcca....................   3       1   HS7
.......................................................................uaaggcacgcgguAaaugcca....................   14      1   HS7
.......................................................................uaagCcacgcggugaaugcca....................   2       1   HS7
.......................................................................uaaggUacgcggugaaugcca....................   65      1   HS7
.......................................................................uaaggcacgcggugaauAcca....................   78      1   HS7
.......................................................................uaaggcacgcggugCaugcca....................   3       1   HS7
.......................................................................uaaUgcacgcggugaaugcca....................   35      1   HS7
.......................................................................uaaggcacgcggugaaugGca....................   4       1   HS7
.......................................................................uaaggcacgcggugaauUcca....................   28      1   HS7
.......................................................................uaaggcacgcAgugaaugcca....................   15      1   HS7
.......................................................................uaaggcacgcggugaaugcUa....................   15      1   HS7
.......................................................................uaaggcacgcggugCaugccaa...................   1       1   HS7
.......................................................................uaaUgcacgcggugaaugccaa...................   2       1   HS7
.......................................................................uaaggcaUgcggugaaugccaa...................   1       1   HS7
.......................................................................uaaggcacgcggugaaugUcaa...................   3       1   HS7
.......................................................................uaaCgcacgcggugaaugccaa...................   1       1   HS7
.......................................................................uaaggcacgcggugaaugccaG...................   2       1   HS7
.......................................................................uaaggcacgcCgugaaugccaa...................   1       1   HS7
.......................................................................uaUggcacgcggugaaugccaa...................   1       1   HS7
.......................................................................uaaggcacgcggugaaugccaa...................   620     0   HS7
.......................................................................uaaggcacgcggugaaugccaC...................   280     1   HS7
.......................................................................uaaggcacgUggugaaugccaa...................   1       1   HS7
.......................................................................uaaggcacgcggugaaugAcaa...................   1       1   HS7
.......................................................................uaaggcacgcggugaaugccaU...................   449     1   HS7
.......................................................................uaaggcacgcggugaauUccaa...................   6       1   HS7
.......................................................................uaaggUacgcggugaaugccaa...................   2       1   HS7
.......................................................................uaaggcacgcggugaaugccaag..................   10      0   HS7
```

```
                               Star                                   Mature
gugaugcuuuguggaccuuuugugaucgcuucguguucacuguuugccuugaugucaauguuugauuucauaaggcacgcggugaaugccaagugcaaaggucaucauucg
......................................................................uaaggcacgcggugaaugccaUg..................    13     1    HS7
......................................................................uaaggcacgcggugaaugccaaA..................    44     1    HS7
......................................................................uaaggcacgcggugaaugccaaC..................    4      1    HS7
......................................................................uaaggcacgcggugaaugccaaU..................    132    1    HS7
......................................................................uaaggcacgcggugaaugccaGgu.................    1      1    HS7
......................................................................uaaggcacgcggugaaugccaaUu.................    44     1    HS7
......................................................................uaaggcacgcggugaaugccaagu.................    4      0    HS7
......................................................................uaaggcacgcggugaaugccaagG.................    583    1    HS7
......................................................................uaaggcacgcAgugaaugccaagug................    1      1    HS7
.......................................................................aaggcacgcAgugaaugc......................    1      1    HS7
.......................................................................aaggcacgcggugaaugc......................    6      0    HS7
.......................................................................aaggcacgcggugaaugcc.....................    22     0    HS7
.......................................................................Uaggcacgcggugaaugcc.....................    4      1    HS7
.......................................................................aagUcacgcggugaaugcc.....................    1      1    HS7
.......................................................................Uaggcacgcggugaaugcca....................    12     1    HS7
.......................................................................aaggcacgcggugaaugcca....................    145    0    HS7
.......................................................................Uaggcacgcggugaaugccaa...................    1      1    HS7
.......................................................................aaggcacgcggugaaugccaC...................    2      1    HS7
.......................................................................aaggcacgcggugaaugccaa...................    1      0    HS7
.......................................................................aaggcacgcggugaaugccaag..................    1      0    HS7
.......................................................................aaggcacgcggugaaugccaagG.................    1      1    HS7
........................................................................aggcacgcggugaaugcc.....................    1      0    HS7
........................................................................aCgcacgcggugaaugcc.....................    2      1    HS7
........................................................................aUgcacgcggugaaugcc.....................    2      1    HS7
........................................................................aUgcacgcggugaaugcca....................    1      1    HS7
........................................................................aggcacgcggugaaugcca....................    3      0    HS7
........................................................................aAgcacgcggugaaugcca....................    3      1    HS7
........................................................................aCgcacgcggugaaugcca....................    1      1    HS7
.........................................................................ggcacgcggugaaugcca....................    1      0    HS7
..........................................................................gcacgcggugaaugccaagG.................    1      1    HS7
...........................................................................cacgcggugaaugccaagG.................    1      1    HS7
............................................................................acgcggugaaugccaagG.................    1      1    HS7

......cuuuguggaccuuuugugaucgcuu................................................................................    1      0    HS5
.......uuuguggaccuuuugugaucgcuu................................................................................    2      0    HS5
.......uuuguggaccuuuugugaucgcuuA...............................................................................    1      1    HS5
.........................ucgcuucguguucacuguuugc................................................................    1      0    HS5
...............................cguguucacuguuugccuu.............................................................    1      0    HS5
...............................cguguucacuguuugccuug............................................................    65     0    HS5
...............................cUuguucacuguuugccuug............................................................    1      1    HS5
...............................cguguucacuguuugccuuga...........................................................    30     0    HS5
...............................cguguucacuguuugAcuugau..........................................................    2      1    HS5
...............................cguguucacuguuugccuugau..........................................................    119    0    HS5
...............................cguguucacuguuugccuugauA.........................................................    1      1    HS5
...............................cguguucacuguuugAcuugaug.........................................................    1      1    HS5
...............................cguguucacuguuCgccuugaug.........................................................    2      1    HS5
...............................cguguCcacuguuugccuugaug.........................................................    1      1    HS5
...............................cguguucacuguuugccuugGug.........................................................    1      1    HS5
...............................cguguucacuguuugUcuugaug.........................................................    2      1    HS5
...............................Uguguucacuguuugccuugaug.........................................................    1      1    HS5
...............................cguguucacuAuuugccuugaug.........................................................    2      1    HS5
...............................cguguucacuguuugccuugaug.........................................................    1150   0    HS5
...............................cguguucacAguuugccuugaug.........................................................    1      1    HS5
...............................cguguucacuguuugccuugauU.........................................................    1      1    HS5
...............................cgugGucacuguuugccuugaug.........................................................    1      1    HS5
...............................cguguucacuguuugcUuugaug.........................................................    1      1    HS5
...............................cguguucacugCuugccuugaug.........................................................    1      1    HS5
...............................cguguucacuguuugccuugaugu........................................................    3      0    HS5
...............................cguguucacuguuugccuugaugA........................................................    30     1    HS5
...............................cguguucacuguuugccuugauguc.......................................................    1      0    HS5
...............................cguguucacuguuugccuugaugucaa.....................................................    1      0    HS5
................................guguucacuguuugccuug............................................................    2      0    HS5
................................guguucacuguuugccuugau..........................................................    2      0    HS5
................................guguucacuguuugccuugaug.........................................................    2      0    HS5
.................................uguucacuguuugccuugau..........................................................    1      0    HS5
.................................Cguucacuguuugccuugaug.........................................................    2      1    HS5
...................................uucacuguuugccuugaug.........................................................    2      0    HS5
...................................uucacuguuugccuugauguc.......................................................    1      0    HS5
....................................ucacuguuugccuugaug.........................................................    1      0    HS5
...........................................................guuugauuucauaaggcacgcgg.............................    1      0    HS5
```

```
                              Star                                Mature
gugaugcuuuguggaccuuuugugaucgcuucguguucacuguuugccuugaugucaauguuugauuucauaaggcacgcggugaaugccaagugcaaaggucaucauucg
............................................................uuugauuucauaaggcacgcggug...........................   1      0   HS5
.................................................................uuucauaaggcacgcggugaau........................   1      0   HS5
.....................................................................auaaggcacgcggugaaug.......................   7      0   HS5
.....................................................................Uuaaggcacgcggugaaugc......................   5      1   HS5
.....................................................................auaaggcacgcggugaaugc......................   35     0   HS5
.....................................................................auaaggcacgcggugaaugA......................   1      1   HS5
.....................................................................auaaggcacgcggugaaugcA.....................   1      1   HS5
.....................................................................auaagUcacgcggugaaugcc.....................   1      1   HS5
.....................................................................Uuaaggcacgcggugaaugcc.....................   1      1   HS5
.....................................................................auaaggcacgcggugaaugUc.....................   1      1   HS5
.....................................................................auaaggcacgcggugaCugcc.....................   1      1   HS5
.....................................................................auaaggcacgUggugaaugcc.....................   1      1   HS5
.....................................................................auaaggcacgcggugaaugcc.....................   121    0   HS5
.....................................................................auaaggcacgcgAugaaugcc.....................   1      1   HS5
.....................................................................auaaggcacgcggugaaugUca....................   1      1   HS5
.....................................................................auaagAcacgcggugaaugcca....................   1      1   HS5
.....................................................................auaaggcacgcggGgaaugcca....................   1      1   HS5
.....................................................................auaaggcacgcggugaaugcca....................   290    0   HS5
.....................................................................auaUggcacgcggugaaugcca....................   1      1   HS5
.....................................................................auaaggcacgcgCugaaugcca....................   2      1   HS5
.....................................................................Uuaaggcacgcggugaaugcca....................   8      1   HS5
.....................................................................auaaggcacgcggugaGugcca....................   1      1   HS5
.....................................................................auaaggcacgcggugaauUcca....................   1      1   HS5
.....................................................................auaaggcacgcggugaCugcca....................   1      1   HS5
.....................................................................auaaggcacgcggugaaugccaU...................   4      1   HS5
.....................................................................Uuaaggcacgcggugaaugccaa...................   2      1   HS5
......................................................................uaaggcacgcggugaauC.......................   1      1   HS5
......................................................................uaaggcacgcggugaauA.......................   2      1   HS5
......................................................................uaaggcacgcggugaaug.......................   57     0   HS5
......................................................................uaaggcacgcggugaauU.......................   1      1   HS5
......................................................................uaaggcacgUggugaaugc......................   4      1   HS5
......................................................................uaaggcacgcgguCaaugc......................   1      1   HS5
......................................................................uaaggcacgcggugaauAc......................   1      1   HS5
......................................................................uaaggcacgcggugaCugc......................   2      1   HS5
......................................................................uaaggcacgcggugaaugc......................   5436   0   HS5
......................................................................uaaggGacgcggugaaugc......................   1      1   HS5
......................................................................uaaggcacgcggugaaugU......................   26     1   HS5
......................................................................uaaggcacAcggugaaugc......................   1      1   HS5
......................................................................uaaggcacgcggugaauUc......................   1      1   HS5
......................................................................uaaggcacgcggugaaugA......................   86     1   HS5
......................................................................uaaggcacgcggugaaAgc......................   1      1   HS5
......................................................................uaaggcacgcggugUaugc......................   1      1   HS5
......................................................................uaagAcacgcggugaaugc......................   1      1   HS5
......................................................................uaaggcaUgcggugaaugc......................   1      1   HS5
......................................................................uaGggcacgcggugaaugc......................   2      1   HS5
......................................................................uaaggcacgcggAgaaugc......................   1      1   HS5
......................................................................uaaggUacgcggugaaugc......................   9      1   HS5
......................................................................uaaggcacgcggugaaCgc......................   1      1   HS5
......................................................................uaaggcacgcggCgaaugc......................   5      1   HS5
......................................................................uaaggcacgcgCugaaugc......................   5      1   HS5
......................................................................uaaggcacgcggugaaGgc......................   1      1   HS5
......................................................................uaaggcaGgcggugaaugc......................   1      1   HS5
......................................................................uaaggcacgcggugCaugc......................   3      1   HS5
......................................................................uaaggcacgcgAugaaugc......................   5      1   HS5
......................................................................uaaggcacgcgUugaaugc......................   1      1   HS5
......................................................................uaaAgcacgcggugaaugc......................   1      1   HS5
......................................................................uaaggcacgcAgugaaugc......................   2      1   HS5
......................................................................uaaggcUcgcggugaaugc......................   1      1   HS5
......................................................................Gaaggcacgcggugaaugc......................   2      1   HS5
......................................................................uaaggcacgcggugaGugc......................   2      1   HS5
......................................................................uaaggcacgcggugGaugc......................   4      1   HS5
......................................................................uaagUcacgcggugaaugc......................   1      1   HS5
......................................................................Aaaggcacgcggugaaugc......................   2      1   HS5
......................................................................uaaggcacgcggugaaugUc.....................   27     1   HS5
......................................................................uaaggAacgcggugaaugcc.....................   1      1   HS5
......................................................................uaaggcacgcggugaUugcc.....................   1      1   HS5
......................................................................uaaggcacgcAgugaaugcc.....................   1      1   HS5
......................................................................uaaggcacgcggugaaugcA.....................   279    1   HS5
......................................................................uaaggcacgcgUugaaugcc.....................   1      1   HS5
......................................................................uaaggcacgAggugaaugcc.....................   1      1   HS5
```

Star Mature

gugaugcuuuguggaccuuuugugaucgcuucguguucacuguuugccuugaugucaauguuugauuucauaaggcacgcggugaaugccaagugcaaaggucaucauucg			
....uaagUcacgcggugaaugcc.....................	2	1	HS5
....uaaggcacgcgguAaaugcc.....................	2	1	HS5
....uaaggcacgcggugCaugcc.....................	1	1	HS5
....uaaggcacgcggCgaaugcc.....................	6	1	HS5
....uaaggcacgcggugaaCgcc.....................	2	1	HS5
....uaaggcacgcgCugaaugcc.....................	3	1	HS5
....uaaggcacgcggugaauAcc.....................	2	1	HS5
....uaaggcacgcggugGaugcc.....................	5	1	HS5
....uaaggcacCcggugaaugcc.....................	2	1	HS5
....uaaggcacgcggugaaugcU.....................	36	1	HS5
....uaagAcacgcggugaaugcc.....................	3	1	HS5
....uaaggcacgcgAugaaugcc.....................	1	1	HS5
....uaaggcacgcggugaaugcG.....................	1	1	HS5
....uaaCgcacgcggugaaugcc.....................	6	1	HS5
....Gaaggcacgcggugaaugcc.....................	1	1	HS5
....uaaggcUcgcggugaaugcc.....................	1	1	HS5
....uaaggUacgcggugaaugcc.....................	7	1	HS5
....uaGggcacgcggugaaugcc.....................	1	1	HS5
....uaaUgcacgcggugaaugcc.....................	10	1	HS5
....uaaggcacgcggugaaugcc.....................	2921	0	HS5
....uaaggcacgcggugaauCcc.....................	6	1	HS5
....uaaggcacgUggugaaugcc.....................	6	1	HS5
....uaaggcacUcggugaaugcc.....................	1	1	HS5
....uaaggcacgcggugUaugcc.....................	1	1	HS5
....uaaggcacgcgguCaaugcc.....................	2	1	HS5
....uaaggcacgcggugaCugcc.....................	8	1	HS5
....uaaggcacgcggugaaAgcc.....................	2	1	HS5
....uaaggcacUcggugaaugcca....................	4	1	HS5
....uaaggcacgcAgugaaugcca....................	5	1	HS5
....uaaggcacgcggugaaugGca....................	2	1	HS5
....uaaggcacgcggugaaugccU....................	10	1	HS5
....uaaggGacgcggugaaugcca....................	1	1	HS5
....uaaggcacgAggugaaugcca....................	4	1	HS5
....Gaaggcacgcggugaaugcca....................	1	1	HS5
....uaaggcGcgcggugaaugcca....................	4	1	HS5
....uaaggcacgcggugaaugccC....................	2	1	HS5
....uaaggcacgcggugUaugcca....................	22	1	HS5
....uaaggcacgcggugaauUcca....................	54	1	HS5
....uaaggcacgcggugaaGgcca....................	3	1	HS5
....uaaggcacgcCgugaaugcca....................	3	1	HS5
....uaaCgcacgcggugaaugcca....................	135	1	HS5
....uaaggAacgcggugaaugcca....................	1	1	HS5
....uaaggcacgcgguUaaugcca....................	5	1	HS5
....uaaggcacgcggugaGugcca....................	29	1	HS5
....uaUggcacgcggugaaugcca....................	7	1	HS5
....Caaggcacgcggugaaugcca....................	3	1	HS5
....uUaggcacgcggugaaugcca....................	1	1	HS5
....uaaUgcacgcggugaaugcca....................	86	1	HS5
....uaaggcacgcggugaaAgcca....................	4	1	HS5
....uaaggcaUgcggugaaugcca....................	8	1	HS5
....uaGggcacgcggugaaugcca....................	2	1	HS5
....uaaggcacgcggugaaugccG....................	2	1	HS5
....uaaggcacgcggugCaugcca....................	9	1	HS5
....uaaggcacgcgAugaaugcca....................	15	1	HS5
....uaaAgcacgcggugaaugcca....................	2	1	HS5
....uaaggcacgcggugaaugcAa....................	10	1	HS5
....uaaggcacgcUgugaaugcca....................	2	1	HS5
....uaaggcacgcggugaauAcca....................	43	1	HS5
....uaaggcacgcggugaCugcca....................	36	1	HS5
....uaaggcacgcggugaUugcca....................	15	1	HS5
....uaagUcacgcggugaaugcca....................	19	1	HS5
....uaaggcacgcggugaauCcca....................	35	1	HS5
....uGaggcacgcggugaaugcca....................	3	1	HS5
....uaaggcacgcggugaaugUca....................	157	1	HS5
....uaaggcacgcggGgaaugcca....................	1	1	HS5
....uaaggcacgcggugaaCgcca....................	10	1	HS5
....uaaggcacgcgCugaaugcca....................	15	1	HS5
....uaaggcacgcgguAaaugcca....................	7	1	HS5
....Aaaggcacgcggugaaugcca....................	10	1	HS5
....uaaggUacgcggugaaugcca....................	33	1	HS5

```
                                Star                                   Mature
gugaugcuuuguggaccuuuugugaucgcuucguguucacuguuugccuugaugucaauguuugauuucauaaggcacgcggugaaugccaagugcaaaggucaucauucg
......................................................................uaaggcacgcgUugaaugcca....................   5      1   HS5
......................................................................uaaggcacgcggugaaugcca....................   21490  0   HS5
......................................................................uaaggcacgcggAgaaugcca....................   1      1   HS5
......................................................................uaaggcacAcggugaaugcca....................   5      1   HS5
......................................................................uaaggcacgcggCgaaugcca....................   19     1   HS5
......................................................................uaaggcacgcgguCaaugcca....................   3      1   HS5
......................................................................uaCggcacgcggugaaugcca....................   1      1   HS5
......................................................................uaaggcacCcggugaaugcca....................   3      1   HS5
......................................................................uaaggcacgUggugaaugcca....................   19     1   HS5
......................................................................uaaggcacgcggugGaugcca....................   17     1   HS5
......................................................................uaagCcacgcggugaaugcca....................   7      1   HS5
......................................................................uaagAcacgcggugaaugcca....................   14     1   HS5
......................................................................uaaggcacgcggugaaugcUa....................   9      1   HS5
......................................................................uaaggcUcgcggugaaugcca....................   8      1   HS5
......................................................................uaaCgcacgcggugaaugccaa...................   1      1   HS5
......................................................................uaaggcacgcggugaaGgccaa...................   1      1   HS5
......................................................................Aaaggcacgcggugaaugccaa...................   1      1   HS5
......................................................................uaaggcacgcggugaGugccaa...................   6      1   HS5
......................................................................uaaggcacgcggugaauUccaa...................   7      1   HS5
......................................................................uaaggUacgcggugaaugccaa...................   5      1   HS5
......................................................................uaaggcacgcgCugaaugccaa...................   3      1   HS5
......................................................................uaaggcacUcggugaaugccaa...................   2      1   HS5
......................................................................Gaaggcacgcggugaaugccaa...................   2      1   HS5
......................................................................uaaggcacgcggugaaugccaU...................   2680   1   HS5
......................................................................uaaggcacgcggugaaugccaC...................   948    1   HS5
......................................................................uaGggcacgcggugaaugccaa...................   2      1   HS5
......................................................................uaaggcacgcggugaaugcUaa...................   2      1   HS5
......................................................................uaaggcaUgcggugaaugccaa...................   1      1   HS5
......................................................................uGaggcacgcggugaaugccaa...................   1      1   HS5
......................................................................uaagUcacgcggugaaugccaa...................   1      1   HS5
......................................................................uaaggcacgcgUugaaugccaa...................   1      1   HS5
......................................................................uaaggcacgcggugaaugccaG...................   1      1   HS5
......................................................................uaaggcacgcggCgaaugccaa...................   1      1   HS5
......................................................................uaaggcacgcggugUaugccaa...................   3      1   HS5
......................................................................uaaggcacgcggugaaugUcaa...................   16     1   HS5
......................................................................uaaggcacgcggugaaugGcaa...................   1      1   HS5
......................................................................uaaggcacgcgAugaaugccaa...................   2      1   HS5
......................................................................uaaggcacgcggugaauCccaa...................   5      1   HS5
......................................................................uaaggAacgcggugaaugccaa...................   2      1   HS5
......................................................................uaagAcacgcggugaaugccaa...................   2      1   HS5
......................................................................uaaggcacgcggugaaugccaa...................   4037   0   HS5
......................................................................uaaggcacgcggugaUugccaa...................   5      1   HS5
......................................................................uaaggcacgUggugaaugccaa...................   2      1   HS5
......................................................................uaaggcacgcggAgaaugccaa...................   1      1   HS5
......................................................................uaaggcacgcggugaCugccaa...................   9      1   HS5
......................................................................uaaggcacgcggugaauAccaa...................   7      1   HS5
......................................................................uaCggcacgcggugaaugccaa...................   2      1   HS5
......................................................................Caaggcacgcggugaaugccaa...................   2      1   HS5
......................................................................uaUggcacgcggugaaugccaa...................   2      1   HS5
......................................................................uaaggcacgcggugGaugccaa...................   4      1   HS5
......................................................................uaaggcacgcggugaaugccaaA..................   289    1   HS5
......................................................................uaaggcacgcggugaaugccaUg..................   21     1   HS5
......................................................................uaaggcacgcggugaaugccaag..................   4      0   HS5
......................................................................uaaggcacgcggugaaugccaaC..................   47     1   HS5
......................................................................uaaggcacgcggugaaugccaaU..................   1094   1   HS5
......................................................................uaaggcacgcggugaaugccaaUu.................   370    1   HS5
......................................................................uaaggcacgcggugaaugccaaAu.................   3      1   HS5
......................................................................uaaggcacgcggugaaugccaagA.................   2      1   HS5
......................................................................uaaggcacgcggugaaugccaaCu.................   8      1   HS5
......................................................................uaaggcacgcggugaaugccaagG.................   1      1   HS5
......................................................................uaaggcacgcggugaaugccaaUug................   1      1   HS5
.......................................................................Uaggcacgcggugaaugc......................   2      1   HS5
.......................................................................aaggcacgcggugaaugc......................   8      0   HS5
.......................................................................aaggcacgcggugaaugUc.....................   1      1   HS5
.......................................................................aaggcacgcggugaaugcc.....................   14     0   HS5
.......................................................................aaUgcacgcggugaaugcca....................   5      1   HS5
.......................................................................aaggcacgcggugaaCgcca....................   1      1   HS5
.......................................................................aaCgcacgcggugaaugcca....................   2      1   HS5
.......................................................................aaggcacgcggugaaugcca....................   68     0   HS5
.......................................................................aaggcacgcggugGaugcca....................   1      1   HS5
```

Star Mature

gugaugcuuuguggaccuuuugugaucgcuucguguucacuguuugccuugaugucaauguuugauuucauaaggcacgcggugaaugccaagugcaaaggucaucauucg			
....Uaggcacgcggugaaugcca....................	7	1	HS5
....aaggcacgcggugaaugccaU...................	13	1	HS5
....aaggcacgcggugaaugccaC...................	3	1	HS5
....aaggcacgcggugaaugccaa...................	9	0	HS5
....aaggcacgcggugaaugccaaU..................	2	1	HS5
....aaggcacgcggugaaugccaaUu.................	3	1	HS5
....aAgcacgcggugaaugcca....................	1	1	HS5
....aCgcacgcggugaaugcca....................	14	1	HS5
....aggcacgcggugaaugcca....................	9	0	HS5
....aUgcacgcggugaaugcca....................	25	1	HS5
....aggcacgcggugaaugccaagu.................	1	0	HS5
....ggcacgcggugaaugcca....................	1	0	HS5
....gcacgcggugaaugccaU...................	2	1	HS5
....gcacgcggugaaugccaaA..................	1	1	HS5
....cacgcggugaaugccaaUu.................	1	1	HS5
...............................cguguucacuguuugccuug..	10	0	HFA
...............................cguguucacuguuugccuugau..	2	0	HFA
...............................cguguucacuguuugccuugaug...	58	0	HFA
....................................ucacuguuugccuugaug...	1	0	HFA
....auaaggcacgcggugaaug.......................	1	0	HFA
....Guaaggcacgcggugaaugc......................	1	1	HFA
....Uuaaggcacgcggugaaugc......................	10	1	HFA
....auaaggcacgcggugaaugc......................	22	0	HFA
....Guaaggcacgcggugaaugcc.....................	1	1	HFA
....auaaggcacgcggugaaugcc.....................	11	0	HFA
....Cuaaggcacgcggugaaugcc.....................	1	1	HFA
....Uuaaggcacgcggugaaugcc.....................	42	1	HFA
....Uuaaggcacgcggugaaugcca....................	41	1	HFA
....auaaggcacgcggugaaugcca....................	12	0	HFA
....uaaggcacgUggugaaug.......................	1	1	HFA
....uaaggcacgcgguAaaug.......................	1	1	HFA
....uaaggcacgcggugaauC.......................	1	1	HFA
....uaaggcacgcggugaauU.......................	11	1	HFA
....uaagUcacgcggugaaug.......................	2	1	HFA
....uaaggcacgcgguCaaug.......................	1	1	HFA
....uaaggcacgcggugaauA.......................	7	1	HFA
....uaaggcacgcggugaaug.......................	217	0	HFA
....uaaggUacgcggugaaug.......................	1	1	HFA
....uaaggcacgAggugaaugc......................	1	1	HFA
....uaaggcacgcggugaauAc......................	6	1	HFA
....uaaggcacgcggugaaugU......................	43	1	HFA
....uaaggcacgcgCugaaugc......................	1	1	HFA
....uaaggcacgcggGgaaugc......................	2	1	HFA
....uaGggcacgcggugaaugc......................	1	1	HFA
....uaaggcacgcUgugaaugc......................	1	1	HFA
....uaaggcacgcggugaaugc......................	5437	0	HFA
....Gaaggcacgcggugaaugc......................	1	1	HFA
....uaCggcacgcggugaaugc......................	1	1	HFA
....uaaggcacgcggugCaugc......................	4	1	HFA
....uUaggcacgcggugaaugc......................	3	1	HFA
....uaaggcacgcggugaaugA......................	104	1	HFA
....uaaggcacgcggugaauUc......................	1	1	HFA
....uaaggcacgcggugUaugc......................	9	1	HFA
....uaagAcacgcggugaaugc......................	1	1	HFA
....uaaggcacgcggugGaugc......................	9	1	HFA
....uaaggcacgcggCgaaugc......................	1	1	HFA
....uaaggcacgcAgugaaugc......................	1	1	HFA
....uaaggcacUcggugaaugc......................	1	1	HFA
....uaagCcacgcggugaaugc......................	2	1	HFA
....uaaggcacgUggugaaugc......................	16	1	HFA
....uaaggUacgcggugaaugc......................	2	1	HFA
....uaUggcacgcggugaaugc......................	1	1	HFA
....uaaggcacgcgguUaaugc......................	1	1	HFA
....uaaggcaUgcggugaaugc......................	2	1	HFA
....uaaggcacgcggugaauCc......................	3	1	HFA
....uaaggcacgcCgugaaugc......................	1	1	HFA
....uaaggcacgcgAugaaugc......................	2	1	HFA
....uaaggcacgcggugaaCgcc.....................	2	1	HFA
....uaaggcacgcggCgaaugcc.....................	1	1	HFA

Star **Mature**

```
gugaugcuuuguggaccuuuugugaucgcuucguguucacuguuugccuugaugucaauguuugauuucauaaggcacgcggugaaugccaagugcaaaggucaucauucg
......................................................................uaaggcacgcggugGaugcc.....................  37     1  HFA
......................................................................uaaggcaUgcggugaaugcc.....................  10     1  HFA
......................................................................uUaggcacgcggugaaugcc.....................  2      1  HFA
......................................................................uaaggcacgcggugaauAcc.....................  9      1  HFA
......................................................................uaaggcacCcggugaaugcc.....................  2      1  HFA
......................................................................uaaUgcacgcggugaaugcc.....................  12     1  HFA
......................................................................uaaggcacgcggugaaugcc.....................  12124  0  HFA
......................................................................uaaggcacgcggugaaugcU.....................  63     1  HFA
......................................................................uaaggcacgcgguAaaugcc.....................  5      1  HFA
......................................................................uaaggcacgcggugaauUcc.....................  5      1  HFA
......................................................................uaaggcacgcggugaaugcA.....................  521    1  HFA
......................................................................uaaggcacgcggugaauCcc.....................  22     1  HFA
......................................................................uaaggcacgcAgugaaugcc.....................  6      1  HFA
......................................................................uaaggcacgcggugaaugUc.....................  51     1  HFA
......................................................................uaaggcacgcggugUaugcc.....................  32     1  HFA
......................................................................uaaggcacgAggugaaugcc.....................  1      1  HFA
......................................................................uaaggcaGgcggugaaugcc.....................  1      1  HFA
......................................................................uaUggcacgcggugaaugcc.....................  1      1  HFA
......................................................................uaaggcacgcggugCaugcc.....................  11     1  HFA
......................................................................uaaAgcacgcggugaaugcc.....................  1      1  HFA
......................................................................Gaaggcacgcggugaaugcc.....................  1      1  HFA
......................................................................Aaaggcacgcggugaaugcc.....................  1      1  HFA
......................................................................uaaggcacgcggugaGugcc.....................  3      1  HFA
......................................................................uaaggcacgUggugaaugcc.....................  22     1  HFA
......................................................................uaaggcacgcggugaaugGc.....................  1      1  HFA
......................................................................uaaggcacgcggugaUugcc.....................  1      1  HFA
......................................................................uaaggcacgcggugaaGgcc.....................  1      1  HFA
......................................................................uaaggcacUcggugaaugcc.....................  2      1  HFA
......................................................................uaaggcacgcUgugaaugcc.....................  1      1  HFA
......................................................................uaCggcacgcggugaaugcc.....................  1      1  HFA
......................................................................uaaggUacgcggugaaugcc.....................  11     1  HFA
......................................................................uGaggcacgcggugaaugcc.....................  7      1  HFA
......................................................................uaagUcacgcggugaaugcc.....................  3      1  HFA
......................................................................uaaCgcacgcggugaaugcc.....................  2      1  HFA
......................................................................uaaggcacgcgAugaaugcc.....................  4      1  HFA
......................................................................uaaggcacgcggugaCugcc.....................  2      1  HFA
......................................................................uaaggcacgcggugaaugcG.....................  4      1  HFA
......................................................................uaagAcacgcggugaaugcc.....................  3      1  HFA
......................................................................uaaggcacgcggGgaaugcc.....................  2      1  HFA
......................................................................uaaggcacAcggugaaugcca....................  8      1  HFA
......................................................................uaaggcacgcgguUaaugcca....................  5      1  HFA
......................................................................uaaggcacgcggugaaugccC....................  21     1  HFA
......................................................................uaaggcacgcggugaaugcAa....................  12     1  HFA
......................................................................uaaggcacgcggugCaugcca....................  33     1  HFA
......................................................................uaaggcacgcggugaaCgcca....................  4      1  HFA
......................................................................uaaggcacgcggugaGugcca....................  7      1  HFA
......................................................................uaaggcUcgcggugaaugcca....................  2      1  HFA
......................................................................uaaggcacgcgUugaaugcca....................  3      1  HFA
......................................................................uaaggcacgcggugaaugccG....................  26     1  HFA
......................................................................uaaggcGcgcggugaaugcca....................  2      1  HFA
......................................................................uaaggcacgcggugaUugcca....................  4      1  HFA
......................................................................uaaCgcacgcggugaaugcca....................  19     1  HFA
......................................................................uaaggcacgcggugaaGgcca....................  2      1  HFA
......................................................................uaagAcacgcggugaaugcca....................  12     1  HFA
......................................................................uaGggcacgcggugaaugcca....................  5      1  HFA
......................................................................Gaaggcacgcggugaaugcca....................  3      1  HFA
......................................................................uaaggcacgcCgugaaugcca....................  1      1  HFA
......................................................................uaagUcacgcggugaaugcca....................  6      1  HFA
......................................................................uaaggcacgcggugaauCcca....................  13     1  HFA
......................................................................uaaggcacgcggAgaaugcca....................  4      1  HFA
......................................................................uaaggcacgcUgugaaugcca....................  2      1  HFA
......................................................................uaaggcacgcggugGaugcca....................  97     1  HFA
......................................................................uaaggUacgcggugaaugcca....................  33     1  HFA
......................................................................uaaAgcacgcggugaaugcca....................  4      1  HFA
......................................................................uaaggcacCcggugaaugcca....................  2      1  HFA
......................................................................uaaggcacgcggugaaugcca....................  40701  0  HFA
......................................................................uaaUgcacgcggugaaugcca....................  17     1  HFA
......................................................................uaaggcacgAggugaaugcca....................  1      1  HFA
......................................................................uaaggcacgcggugaaAgcca....................  1      1  HFA
......................................................................uaaggcacgcggugUaugcca....................  79     1  HFA
```

```
                               Star                                   Mature
gugaugcuuuguggaccuuuugugaucgcuucguguucacuguuugccuugaugucaauguuugauuucauaaggcacgcggugaaugccaagugcaaaggucaucauucg
......................................................................uaCggcacgcggugaaugcca....................  3    1  HFA
......................................................................uaaggcacgcAgugaaugcca....................  14   1  HFA
......................................................................uaaggcacgcggCgaaugcca....................  5    1  HFA
......................................................................uaaggcacgcggugaaugUca....................  131  1  HFA
......................................................................uaaggcacgcggugaaugccU....................  45   1  HFA
......................................................................uaaggcaUgcggugaaugcca....................  20   1  HFA
......................................................................uaaggcCcgcggugaaugcca....................  1    1  HFA
......................................................................Aaaggcacgcggugaaugcca....................  2    1  HFA
......................................................................uUaggcacgcggugaaugcca....................  14   1  HFA
......................................................................uaaggcacgcgAugaaugcca....................  10   1  HFA
......................................................................uaaggcacgcgguCaaugcca....................  5    1  HFA
......................................................................Caaggcacgcggugaaugcca....................  5    1  HFA
......................................................................uaaggcacgcgguAaaugcca....................  10   1  HFA
......................................................................uaaggcacgcggugaaugcUa....................  14   1  HFA
......................................................................uaaggcacgcggugaCugcca....................  6    1  HFA
......................................................................uaaggcaGgcggugaaugcca....................  3    1  HFA
......................................................................uCaggcacgcggugaaugcca....................  1    1  HFA
......................................................................uaaggcacgcggugaauAcca....................  73   1  HFA
......................................................................uaaggcaAgcggugaaugcca....................  1    1  HFA
......................................................................uaUggcacgcggugaaugcca....................  6    1  HFA
......................................................................uaaggcacgcggGgaaugcca....................  2    1  HFA
......................................................................uaaggcacgcggugaauUcca....................  22   1  HFA
......................................................................uaaggcacUcggugaaugcca....................  3    1  HFA
......................................................................uaaggcacgcgCugaaugcca....................  1    1  HFA
......................................................................uaaggcacgUggugaaugcca....................  34   1  HFA
......................................................................uGaggcacgcggugaaugcca....................  12   1  HFA
......................................................................uaaggcacgcggugaaugccaU...................  464  1  HFA
......................................................................uaaggcacgcggugaauUccaa...................  1    1  HFA
......................................................................uaaggcacgcggugaaugccaa...................  307  0  HFA
......................................................................uaaggcacgcggugaaugccaC...................  249  1  HFA
......................................................................uaaggcacgcggugaaugccaaU..................  62   1  HFA
......................................................................uaaggcacgcggugaaugccaaA..................  15   1  HFA
......................................................................uaaggcacgcggugaaugccaUg..................  4    1  HFA
......................................................................uaaggcacgcggugaaugccaaC..................  1    1  HFA
......................................................................uaaggcacgcggugaaugccaag..................  1    0  HFA
......................................................................uaaggcacgcggugaaugccaGgu.................  1    1  HFA
......................................................................uaaggcacgcggugaaugccaaAu.................  1    1  HFA
......................................................................uaaggcacgcggugaaugccaaUu.................  15   1  HFA
......................................................................uaaggcacgcggugaaugccaaCu.................  1    1  HFA
......................................................................uaaggcacgcggugaaugccaagG.................  3    1  HFA
.......................................................................aaggcacgcggugaaugc......................  24   0  HFA
.......................................................................aaggcacgcggugaaugUc.....................  2    1  HFA
.......................................................................Uaggcacgcggugaaugcc.....................  5    1  HFA
.......................................................................aaggcacgcggugaauCcc.....................  1    1  HFA
.......................................................................aaggcacgcggugUaugcc.....................  1    1  HFA
.......................................................................aaggcacgcggugaaugcc.....................  25   0  HFA
.......................................................................aaAgcacgcggugaaugcca....................  1    1  HFA
.......................................................................aaUgcacgcggugaaugcca....................  3    1  HFA
.......................................................................aagUcacgcggugaaugcca....................  1    1  HFA
.......................................................................Uaggcacgcggugaaugcca....................  8    1  HFA
.......................................................................aaggUacgcggugaaugcca....................  1    1  HFA
.......................................................................aaggcacgcggugaCugcca....................  1    1  HFA
.......................................................................aaggcacgcggugaaugcca....................  91   0  HFA
.......................................................................aaggcacgcggugaaugccaa...................  1    0  HFA
.......................................................................aaggcacgcggugaaugccaU...................  1    1  HFA
........................................................................aggcacgcggugaaugcc.....................  1    0  HFA
........................................................................aUgcacgcggugaaugcca....................  3    1  HFA
........................................................................aCgcacgcggugaaugcca....................  2    1  HFA
........................................................................aggcacgcggugaaugcca....................  14   0  HFA
........................................................................aAgcacgcggugaaugccaa...................  1    1  HFA
.........................................................................ggcacgcggugaaugcca....................  4    0  HFA
.........................................................................gUcacgcggugaaugcca....................  1    1  HFA
.........................................................................Agcacgcggugaaugcca....................  2    1  HFA
..........................................................................gcacgcggugaaugccaU...................  2    1  HFA
...........................................................................cacgcggugaaugccaaA..................  1    1  HFA
...........................................................................cacgcggugaaugccaaU..................  1    1  HFA
...........................................................................cacgcggugaaugccaaUu.................  1    1  HFA

...............................cguguucacuguuugccuug............................................................  8    0  HMA
...............................cguguucacuguuugccuugU...........................................................  1    1  HMA
```

```
                               Star                                   Mature
gugaugcuuuguggaccuuuugugaucgcuucguguucacuguuugccuugaugucaauguuugauuucauaaggcacgcggugaaugccaagugcaaaggucaucauucg
...............................cguguucacuguuugccuuga............................................................  15     0  HMA
...............................cguguucacuguuugccuugau...........................................................  8      0  HMA
...............................cgugAucacuguuugccuugaug.........................................................  1      1  HMA
...............................cguguucacuguuuUccuugaug.........................................................  1      1  HMA
...............................cguguucacugGuugccuugaug.........................................................  2      1  HMA
...............................cguguucacuguuugccGugaug.........................................................  1      1  HMA
...............................cguUuucacuguuugccuugaug.........................................................  1      1  HMA
...............................cguguucacuguuugccuugauA.........................................................  2      1  HMA
...............................cguguGcacuguuugccuugaug.........................................................  2      1  HMA
...............................cguguCcacuguuugccuugaug.........................................................  1      1  HMA
...............................cguguucacuguuugccuugauU.........................................................  1      1  HMA
...............................cguguucacuguuugccuugaug.........................................................  339    0  HMA
...............................cgugGucacuguuugccuugaug.........................................................  1      1  HMA
...............................cguguucacuguuugUcuugaug.........................................................  1      1  HMA
...............................cguguucacuguuugccuugaugA........................................................  5      1  HMA
................................guguucacugGuugccuugaug.........................................................  1      1  HMA
................................guguucacuguuugccuugauA.........................................................  2      1  HMA
................................guguucacuguuugccuugaug.........................................................  8      0  HMA
................................guguucacuguuugccuugaugA........................................................  1      1  HMA
......................................................................uaaggcacgcggugaaug.......................  86     0  HMA
......................................................................uaagAcacgcggugaaug.......................  1      1  HMA
......................................................................uaaggcacgcggugaaugc......................  218    0  HMA
......................................................................uaagUcacgcggugaaugc......................  1      1  HMA
......................................................................uaUggcacgcggugaaugc......................  3      1  HMA
......................................................................uaaggcacgcggugaaugcca....................  1      0  HMA
......................................................................uaaggcacgcggugaaugccaa...................  1      0  HMA

.....gcuuuguggaccuuuugugaucgcuu................................................................................  1      0  HS4
......cuuuguggaccuuuugugaucgcuu................................................................................  2      0  HS4
.......uuuguggaccuuuugugaucgcuu................................................................................  3      0  HS4
...........uggaccuuuugugaucgcuu................................................................................  1      0  HS4
.........................ucgcuucguguucacuguuug.................................................................  1      0  HS4
.........................ucgcuucguguucacuguuugc................................................................  1      0  HS4
.............................uucguguucacuguuugccuug............................................................  1      0  HS4
..............................ucguguucacuguuugccuugaug.........................................................  3      0  HS4
...............................cguguucacuguuugccu..............................................................  4      0  HS4
...............................cguguucacuguuugccuu.............................................................  4      0  HS4
...............................cguguucacuguuugccuuA............................................................  1      1  HS4
...............................cguguucacuguuCgccuug............................................................  1      1  HS4
...............................cguguucacuguuugccuug............................................................  143    0  HS4
...............................cCuguucacuguuugccuuga...........................................................  1      1  HS4
...............................cgCguucacuguuugccuuga...........................................................  1      1  HS4
...............................cguguucacuguuugccuuga...........................................................  76     0  HS4
...............................cguguucacuguuugccuugau..........................................................  260    0  HS4
...............................cguguuUacuguuugccuugau..........................................................  1      1  HS4
...............................cguguucacuguuugUcuugau..........................................................  1      1  HS4
...............................cguguucacuguuugccuugaA..........................................................  1      1  HS4
...............................cguguucacuguuAgccuugaug.........................................................  1      1  HS4
...............................cguguucacuUuuugccuugaug.........................................................  1      1  HS4
...............................cCuguucacuguuugccuugaug.........................................................  2      1  HS4
...............................cguguucacuguuugccCugaug.........................................................  1      1  HS4
...............................cgCguucacuguuugccuugaug.........................................................  2      1  HS4
...............................cAuguucacuguuugccuugaug.........................................................  1      1  HS4
...............................cguguucacuguuCgccuugaug.........................................................  1      1  HS4
...............................cgugGucacuguuugccuugaug.........................................................  1      1  HS4
...............................cguguucacuguuugccuugaug.........................................................  2425   0  HS4
...............................cguguuGacuguuugccuugaug.........................................................  2      1  HS4
...............................cguguuUacuguuugccuugaug.........................................................  1      1  HS4
...............................cguguucacuguuugAcuugaug.........................................................  1      1  HS4
...............................cguguuAacuguuugccuugaug.........................................................  1      1  HS4
...............................cguguucacuguuugccuugaGg.........................................................  1      1  HS4
...............................cUuguucacuguuugccuugaug.........................................................  3      1  HS4
...............................cguguucacugCuugccuugaug.........................................................  1      1  HS4
...............................cguguucacuguuuCccuugaug.........................................................  1      1  HS4
...............................cguguucacuguuugccuCgaug.........................................................  1      1  HS4
...............................cguguucacuguuGgccuugaug.........................................................  1      1  HS4
...............................cguguucacuguuugccuugGug.........................................................  1      1  HS4
...............................cguguucacuguuugccuugCug.........................................................  1      1  HS4
...............................cguguucaUuguuugccuugaug.........................................................  1      1  HS4
...............................cguguucacuguuugcUuugaug.........................................................  1      1  HS4
```

Star   Mature

```
gugaugcuuuguggaccuuuugugaucgcuucguguucacuguuugccuugaugucaauguuugauuucauaaggcacgcggugaaugccaagugcaaaggucaucauucg
...............................cguguucacuguuugUcuugaug.........................................................  3     1     HS4
...............................cguUuucacuguuugccuugaug.........................................................  1     1     HS4
...............................cguguucacuguuugccuugaugA........................................................  39    1     HS4
...............................cguguucacuguuugccuugaugu........................................................  5     0     HS4
...............................cguguucacuguuugccuugauguc.......................................................  4     0     HS4
...............................cguguucacuguuugccuugauguU.......................................................  2     1     HS4
...............................cguguucacuguuugccuugauguca......................................................  1     0     HS4
...............................cguguucacuguuugccuugaugucaa.....................................................  1     0     HS4
................................guguucacuguuugccuug.............................................................  1     0     HS4
................................guguucacuguuugccuugau...........................................................  1     0     HS4
................................guguucacuguuugccuugaug..........................................................  2     0     HS4
.................................uguucacuguuugccuugau...........................................................  3     0     HS4
.................................Cguucacuguuugccuugaug..........................................................  1     1     HS4
.................................uguucacuguuugccuugaug..........................................................  3     0     HS4
..................................guucacuguuugccuugaug..........................................................  4     0     HS4
..................................guucacuguuugccuugauguc........................................................  1     0     HS4
...................................uucacuguuugccuugaug..........................................................  3     0     HS4
...................................uucacuguuugccuugauguc........................................................  1     0     HS4
...................................uucacuguuugccuugaugucU.......................................................  1     1     HS4
....................................ucacuguuugccuugaug..........................................................  3     0     HS4
...........................................................guuugauuucauaaggcacgcggu............................  1     0     HS4
............................................................uuugauuucauaaggcacgcggu............................  1     0     HS4
............................................................uuugauAucauaaggcacgcggug...........................  1     1     HS4
............................................................uuugauuucauaaggcacgcggugU..........................  1     1     HS4
.................................................................uuucauaaggcacgcggug...........................  1     0     HS4
...................................................................ucauaaggcacgcggugaaugc......................  2     0     HS4
....................................................................cauaaggcacgcggugaaugcca...................  1     0     HS4
.....................................................................auaaggcacgcggugaaug.......................  1     0     HS4
.....................................................................Uuaaggcacgcggugaaugc......................  1     1     HS4
.....................................................................auaaggcacgcggugaaugc......................  68    0     HS4
.....................................................................auaaggcacgcggugaaugA......................  1     1     HS4
.....................................................................auaaggcacgcggugaaugUc.....................  1     1     HS4
.....................................................................Uuaaggcacgcggugaaugcc.....................  14    1     HS4
.....................................................................auaaggcacgcggugaaugcA.....................  3     1     HS4
.....................................................................auaaggcacgcggugaauCcc.....................  1     1     HS4
.....................................................................auaaggcacgcggugaaugcc.....................  322   0     HS4
.....................................................................auaaggcacgcggugGaugcc.....................  1     1     HS4
.....................................................................auaaggcacgcggugaGugcc.....................  1     1     HS4
.....................................................................auaaggcacUcggugaaugcc.....................  1     1     HS4
.....................................................................auaaggcacgcCgugaaugcc.....................  1     1     HS4
.....................................................................auaaggcacAcggugaaugcc.....................  1     1     HS4
.....................................................................auaUggcacgcggugaaugcca....................  3     1     HS4
.....................................................................auaaggcacgcggugaaugcca....................  868   0     HS4
.....................................................................Uuaaggcacgcggugaaugcca....................  75    1     HS4
.....................................................................auaaggcacgcgAugaaugcca....................  1     1     HS4
.....................................................................auaaggcacgcggugGaugcca....................  2     1     HS4
.....................................................................auaaggcacgcggugaaugccU....................  1     1     HS4
.....................................................................auaaggcacgcggugaaugUca....................  3     1     HS4
.....................................................................auaaggcacgcggugaauCcca....................  1     1     HS4
.....................................................................Cuaaggcacgcggugaaugcca....................  4     1     HS4
.....................................................................auaagAcacgcggugaaugcca....................  2     1     HS4
.....................................................................auaaggcacgcggugaauAcca....................  2     1     HS4
.....................................................................auaaggAacgcggugaaugcca....................  1     1     HS4
.....................................................................auaaUgcacgcggugaaugcca....................  1     1     HS4
.....................................................................auaaggcacgcggugaaugccaU...................  2     1     HS4
.....................................................................auaaggcacgcggugaaugccaC...................  2     1     HS4
.....................................................................auaaggcacgcggugaaugccaa...................  1     0     HS4
......................................................................uaaggcacgcggugaaug.......................  351   0     HS4
......................................................................uaaggcacgcggugaauU.......................  5     1     HS4
......................................................................uaaggcacgcggugUaug.......................  1     1     HS4
......................................................................uaaggcacgcggugaauA.......................  2     1     HS4
......................................................................uGaggcacgcggugaaug.......................  1     1     HS4
......................................................................uaaggcacgUggugaaug.......................  1     1     HS4
......................................................................uaaggcacgcggugaUugc......................  3     1     HS4
......................................................................uaaggcacgcggugaaGgc......................  1     1     HS4
......................................................................uaUggcacgcggugaaugc......................  4     1     HS4
......................................................................uGaggcacgcggugaaugc......................  8     1     HS4
......................................................................uaaggcacAcggugaaugc......................  3     1     HS4
......................................................................uaaggcUcgcggugaaugc......................  1     1     HS4
......................................................................uaaggcacgcggugaaugU......................  53    1     HS4
```

Star Mature

gugaugcuuuguggaccuuuugugaucgcuucguguucacuguuugccuugaugucaauguuugauuucauaaggcacgcggugaaugccaagugcaaaggucaucauucg			
....uaagUcacgcggugaaugc......................	3	1	HS4
....uaaggcacgcggugUaugc......................	6	1	HS4
....uaaggcacgcUgugaaugc......................	1	1	HS4
....uaaggcGcgcggugaaugc......................	8	1	HS4
....uaaggcacgUggugaaugc......................	13	1	HS4
....uaaggcacgcggugaGugc......................	1	1	HS4
....uaaggcacgcCgugaaugc......................	2	1	HS4
....uaagAcacgcggugaaugc......................	4	1	HS4
....uaaggcaUgcggugaaugc......................	5	1	HS4
....uaaggcacgcggugaauUc......................	2	1	HS4
....uaaggcacgcggugaauAc......................	5	1	HS4
....uaaggcacgcggugaaugA......................	50	1	HS4
....uaaggcacgcgguAaaugc......................	3	1	HS4
....uaaggcacgcggugaaAgc......................	2	1	HS4
....uaCggcacgcggugaaugc......................	6	1	HS4
....uaaUgcacgcggugaaugc......................	9	1	HS4
....uaaggcacgcggugaaugc......................	9263	0	HS4
....uaaggcacgcAgugaaugc......................	4	1	HS4
....uaaggcacgcgguCaaugc......................	1	1	HS4
....uaaggcacgcgUugaaugc......................	4	1	HS4
....uaaggcacgcggugCaugc......................	8	1	HS4
....uaaggAacgcggugaaugc......................	1	1	HS4
....uaGggcacgcggugaaugc......................	2	1	HS4
....uaaggcacgcggugaauCc......................	6	1	HS4
....uaaggcacgcgAugaaugc......................	6	1	HS4
....uaaggcacgcggugGaugc......................	9	1	HS4
....uaaggcacgcggugaaugG......................	1	1	HS4
....uaaggUacgcggugaaugc......................	11	1	HS4
....uaagCcacgcggugaaugc......................	1	1	HS4
....uaaggcacgcggugaauAcc.....................	42	1	HS4
....uaaggcacgGggugaaugcc.....................	4	1	HS4
....Aaaggcacgcggugaaugcc.....................	3	1	HS4
....uaaggcacgcgUugaaugcc.....................	3	1	HS4
....uaaggcacAcggugaaugcc.....................	9	1	HS4
....uaaggcacgcggugaaCgcc.....................	4	1	HS4
....uGaggcacgcggugaaugcc.....................	34	1	HS4
....uaaggcacgcggGgaaugcc.....................	1	1	HS4
....uaaggcacCcggugaaugcc.....................	5	1	HS4
....uaaggcacgcAgugaaugcc.....................	16	1	HS4
....uaaggcacgcggugaaugGc.....................	2	1	HS4
....uaaggcaGgcggugaaugcc.....................	2	1	HS4
....uaaggcacgcggAgaaugcc.....................	1	1	HS4
....uaaggcCcgcggugaaugcc.....................	8	1	HS4
....uaaggcacgcggugaauCcc.....................	40	1	HS4
....uaaggcacgcCgugaaugcc.....................	3	1	HS4
....uaaggcacgcggugCaugcc.....................	53	1	HS4
....uaagCcacgcggugaaugcc.....................	6	1	HS4
....Caaggcacgcggugaaugcc.....................	5	1	HS4
....uaaggcacgcggugaaugUc.....................	134	1	HS4
....uaaggcacgcggugaaugcU.....................	97	1	HS4
....uaCggcacgcggugaaugcc.....................	15	1	HS4
....uaUggcacgcggugaaugcc.....................	14	1	HS4
....uaaggcacgAggugaaugcc.....................	13	1	HS4
....uaaggcacgcggugaaugcA.....................	392	1	HS4
....uaaggcacgcggugaCugcc.....................	10	1	HS4
....uaaggcacgcgguAaaugcc.....................	8	1	HS4
....uaaggUacgcggugaaugcc.....................	55	1	HS4
....Gaaggcacgcggugaaugcc.....................	1	1	HS4
....uaaggcacgcgguUaaugcc.....................	12	1	HS4
....uaagUcacgcggugaaugcc.....................	25	1	HS4
....uaaggcacgcggugaaugcc.....................	47300	0	HS4
....uUaggcacgcggugaaugcc.....................	2	1	HS4
....uaaggcacgcggugGaugcc.....................	81	1	HS4
....uaaCgcacgcggugaaugcc.....................	65	1	HS4
....uaaggGacgcggugaaugcc.....................	1	1	HS4
....uaaggcacgcggugUaugcc.....................	101	1	HS4
....uaaggcacgcggugaGugcc.....................	9	1	HS4
....uaaggcaUgcggugaaugcc.....................	22	1	HS4
....uaaggcacgcggugaUugcc.....................	16	1	HS4
....uaaggcacgcggugaauUcc.....................	11	1	HS4

Star **Mature**

gugaugcuuuguggaccuuuugugaucgcuucguguucacuguuugccuugaugucaauguuugauuucauaaggcacgcggugaaugccaagugcaaaggucaucauucg			
....uaaggcacgcgguCaaugcc.....................	25	1	HS4
....uaaggcUcgcggugaaugcc.....................	10	1	HS4
....uaaggcacgcggugaaGgcc.....................	2	1	HS4
....uaaggcGcgcggugaaugcc.....................	39	1	HS4
....uaaggcacUcggugaaugcc.....................	6	1	HS4
....uaaggcacgcggugaaugAc.....................	3	1	HS4
....uaaggcacgcgAugaaugcc.....................	22	1	HS4
....uaagAcacgcggugaaugcc.....................	15	1	HS4
....uaaAgcacgcggugaaugcc.....................	11	1	HS4
....uaaggcacgcgCugaaugcc.....................	9	1	HS4
....uaaggcacgcggugaaAgcc.....................	3	1	HS4
....uaaggcacgcggCgaaugcc.....................	2	1	HS4
....uaaggcacgcUgugaaugcc.....................	6	1	HS4
....uaGggcacgcggugaaugcc.....................	13	1	HS4
....uaaUgcacgcggugaaugcc.....................	74	1	HS4
....uaaggcacgUggugaaugcc.....................	51	1	HS4
....uaaggcacgcggugaauAcca....................	1140	1	HS4
....uCaggcacgcggugaaugcca....................	5	1	HS4
....Caaggcacgcggugaaugcca....................	45	1	HS4
....uaaggGacgcggugaaugcca....................	3	1	HS4
....uaaggcacgcggugaaugccC....................	36	1	HS4
....uaaggcacAcggugaaugcca....................	64	1	HS4
....uaaggcacgUggugaaugcca....................	581	1	HS4
....Gaaggcacgcggugaaugcca....................	14	1	HS4
....uaaggcacgcgguAaaugcca....................	112	1	HS4
....uaaggcacgcggugUaugcca....................	934	1	HS4
....uaaggcacgcggugaaCgcca....................	35	1	HS4
....uaUggcacgcggugaaugcca....................	125	1	HS4
....uaaggcacUcggugaaugcca....................	37	1	HS4
....uaaggcacgcggugaaugccG....................	124	1	HS4
....uaaAgcacgcggugaaugcca....................	49	1	HS4
....uaaggcacgcggCgaaugcca....................	41	1	HS4
....uaaggcacgcCgugaaugcca....................	11	1	HS4
....uaaUgcacgcggugaaugcca....................	1021	1	HS4
....uaaggcacgcggugaaAgcca....................	18	1	HS4
....uaaggcCcgcggugaaugcca....................	124	1	HS4
....uaaggcaAgcggugaaugcca....................	6	1	HS4
....uaaggcacgcggugaaGgcca....................	34	1	HS4
....uaaggcacgcAgugaaugcca....................	125	1	HS4
....uaaggcacgcggugaauUcca....................	351	1	HS4
....uaaggcacgcgUugaaugcca....................	38	1	HS4
....uaaggcacCcggugaaugcca....................	55	1	HS4
....uaaggcacgcggugaGugcca....................	157	1	HS4
....uaaggcaGgcggugaaugcca....................	5	1	HS4
....uaaggcacgcggGgaaugcca....................	7	1	HS4
....uaaggcacgcgAugaaugcca....................	184	1	HS4
....uaaggAacgcggugaaugcca....................	9	1	HS4
....uaaCgcacgcggugaaugcca....................	1495	1	HS4
....uaaggcUcgcggugaaugcca....................	66	1	HS4
....uaaggcacgcggugaUugcca....................	136	1	HS4
....uaagAcacgcggugaaugcca....................	146	1	HS4
....uaaggcacgcUgugaaugcca....................	14	1	HS4
....uaagCcacgcggugaaugcca....................	74	1	HS4
....uaaggUacgcggugaaugcca....................	468	1	HS4
....uaaggcacgcggugaauCcca....................	302	1	HS4
....uaaggcacgcggugCaugcca....................	615	1	HS4
....uaaggcacgcggugaaugcca....................	481271	0	HS4
....uaaggcacgGggugaaugcca....................	12	1	HS4
....uaagUcacgcggugaaugcca....................	147	1	HS4
....uaaggcacgcggugGaugcca....................	854	1	HS4
....uaGggcacgcggugaaugcca....................	107	1	HS4
....uaaggcacgcggugaaugcUa....................	79	1	HS4
....uaCggcacgcggugaaugcca....................	172	1	HS4
....uaaggcacgcgCugaaugcca....................	84	1	HS4
....uaaggcacgcggugaaugccU....................	111	1	HS4
....uaaggcacgcgguCaaugcca....................	175	1	HS4
....uaaggcacgcggugaaugcAa....................	71	1	HS4
....uGaggcacgcggugaaugcca....................	286	1	HS4
....uaaggcacgcggugaaugAca....................	28	1	HS4
....uaaggcacgcgguUaaugcca....................	136	1	HS4

Star **Mature**

gugaugcuuuguggaccuuuugugaucgcuucguguucacuguuugccuugaugucaauguuugauuucauaaggcacgcggugaaugccaagugcaaaggucaucauucg			
....uaaggcacgcggugaaugGca....................	14	1	HS4
....uaaggcacgAggugaaugcca....................	116	1	HS4
....uaaggcacgcggugaaugUca....................	1099	1	HS4
....uaaggcacgcggAgaaugcca....................	10	1	HS4
....uaaggcacgcggugaCugcca....................	88	1	HS4
....uaaggcacgcggugaaugcGa....................	7	1	HS4
....uUaggcacgcggugaaugcca....................	10	1	HS4
....Aaaggcacgcggugaaugcca....................	38	1	HS4
....uaaggcaUgcggugaaugcca....................	134	1	HS4
....uaaggcGcgcggugaaugcca....................	470	1	HS4
....uaaggcacgcggugaaugccaa...................	7849	0	HS4
....uaaggcacgGggugaaugccaa...................	1	1	HS4
....uaaggcacgcggAgaaugccaa...................	1	1	HS4
....uaaggcacgcggugaaGgccaa...................	2	1	HS4
....uaaggcacgcggugaauUccaa...................	12	1	HS4
....uaaggcacgcggugaCugccaa...................	1	1	HS4
....uaagAcacgcggugaaugccaa...................	1	1	HS4
....uaGggcacgcggugaaugccaa...................	1	1	HS4
....uaaggcacgcggugCaugccaa...................	4	1	HS4
....uaaggcacgcggugaaugccGa...................	1	1	HS4
....uaaggcacgcggugGaugccaa...................	7	1	HS4
....uaUggcacgcggugaaugccaa...................	1	1	HS4
....uaaggcacgcgguCaaugccaa...................	2	1	HS4
....uaaggcacgcgguUaaugccaa...................	5	1	HS4
....Caaggcacgcggugaaugccaa...................	1	1	HS4
....uaaggcacgcAgugaaugccaa...................	4	1	HS4
....uaaggcacCcggugaaugccaa...................	2	1	HS4
....uaaggcCcgcggugaaugccaa...................	3	1	HS4
....uaaCgcacgcggugaaugccaa...................	8	1	HS4
....uaaggcacgcggugaaugccaC...................	4219	1	HS4
....uaagUcacgcggugaaugccaa...................	3	1	HS4
....uaaggUacgcggugaaugccaa...................	6	1	HS4
....uaaggcacgcggugaaugccaG...................	5	1	HS4
....uaaggcacgAggugaaugccaa...................	3	1	HS4
....uaagCcacgcggugaaugccaa...................	2	1	HS4
....uGaggcacgcggugaaugccaa...................	5	1	HS4
....uaaggcacgcggugaaugGcaa...................	2	1	HS4
....uaaggcUcgcggugaaugccaa...................	1	1	HS4
....uaaggcacgcggugaauAccaa...................	13	1	HS4
....uaaggcacgcggugaauCccaa...................	11	1	HS4
....uaaUgcacgcggugaaugccaa...................	16	1	HS4
....uaaggcacgcggugaaugcUaa...................	3	1	HS4
....uaaggcacgcggCgaaugccaa...................	1	1	HS4
....uaaggcacgcggugaaugccaU...................	10168	1	HS4
....uaaggcacAcggugaaugccaa...................	5	1	HS4
....Aaaggcacgcggugaaugccaa...................	1	1	HS4
....uaaggcacgcggugUaugccaa...................	18	1	HS4
....uaaggcacgcggugaUugccaa...................	2	1	HS4
....uaaggcGcgcggugaaugccaa...................	8	1	HS4
....uaaggcacgUggugaaugccaa...................	7	1	HS4
....uaaggcacgcgAugaaugccaa...................	2	1	HS4
....uaaggcacgcggugaaugccUa...................	1	1	HS4
....uaaggcaUgcggugaaugccaa...................	3	1	HS4
....uaaggcacgcggugaGugccaa...................	8	1	HS4
....uaaggcacgcggugaaugAcaa...................	1	1	HS4
....uaaggcacgcggugaaugUcaa...................	23	1	HS4
....uaCggcacgcggugaaugccaa...................	2	1	HS4
....uaaggcacgcggugaaugccaag..................	8	0	HS4
....uaaggcacgcgAugaaugccaag..................	1	1	HS4
....uaaggcacgcggugaaugccaCg..................	1	1	HS4
....uaaggcacgcggugaaugccaaU..................	2331	1	HS4
....uaaggcacgcggugaaugccaaC..................	50	1	HS4
....uaaggcacgcggugaaugccaUg..................	77	1	HS4
....uaaggcacgcggugaaugccaaA..................	408	1	HS4
....uaaggcacgcggugaaugccaaAu.................	10	1	HS4
....uaaggcacgcggugaaugccaagG.................	1	1	HS4
....uaaggcacgcggugaaugccaagA.................	2	1	HS4
....uaaggcacgcggugaaugccaUgu.................	2	1	HS4
....uaaggcacgcggugaaugccaaUu.................	656	1	HS4
....uaaggcacgcggugaaugccaaCu.................	14	1	HS4

```
                              Star                                  Mature
gugaugcuuuguggaccuuuugugaucgcuucguguucacuguuugccuugaugucaauguuugauuucauaaggcacgcggugaaugccaagugcaaaggucaucauucg
......................................................................uaaggcacgcggugaaugccaaUug................   2     1   HS4
.......................................................................aaggcacgcggugaauAc......................   1     1   HS4
.......................................................................aaggcacgcgguAaaugc......................   1     1   HS4
.......................................................................aaggcacgcggugaaugA......................   1     1   HS4
.......................................................................Uaggcacgcggugaaugc......................   5     1   HS4
.......................................................................aaggcacgcggugaaugc......................   21    0   HS4
.......................................................................Uaggcacgcggugaaugcc.....................   10    1   HS4
.......................................................................aaggcacgcggugGaugcc.....................   1     1   HS4
.......................................................................aagUcacgcggugaaugcc.....................   1     1   HS4
.......................................................................aaCgcacgcggugaaugcc.....................   1     1   HS4
.......................................................................aaggcacgcggugaGugcc.....................   1     1   HS4
.......................................................................aaUgcacgcggugaaugcc.....................   1     1   HS4
.......................................................................aGggcacgcggugaaugcc.....................   2     1   HS4
.......................................................................aaggcacgcggugaaugcc.....................   70    0   HS4
.......................................................................aaggcacgcggugaaugcca....................   996   0   HS4
.......................................................................aaUgcacgcggugaaugcca....................   37    1   HS4
.......................................................................Caggcacgcggugaaugcca....................   1     1   HS4
.......................................................................aaggcacgcAgugaaugcca....................   2     1   HS4
.......................................................................Uaggcacgcggugaaugcca....................   117   1   HS4
.......................................................................aaggUacgcggugaaugcca....................   4     1   HS4
.......................................................................aaggcacgUggugaaugcca....................   2     1   HS4
.......................................................................aaggcacgcggugGaugcca....................   11    1   HS4
.......................................................................aaggcacgcggugaCugcca....................   3     1   HS4
.......................................................................aaggcacgcggugaauAcca....................   1     1   HS4
.......................................................................aaggcacgcggugaaugUca....................   5     1   HS4
.......................................................................aaggcacgcggugaauUcca....................   1     1   HS4
.......................................................................aaggcaUgcggugaaugcca....................   1     1   HS4
.......................................................................aagUcacgcggugaaugcca....................   1     1   HS4
.......................................................................aaggcacgcggGgaaugcca....................   2     1   HS4
.......................................................................aaggcacgcggugaGugcca....................   3     1   HS4
.......................................................................aGggcacgcggugaaugcca....................   1     1   HS4
.......................................................................aaggcacgcggugaaAgcca....................   1     1   HS4
.......................................................................aUggcacgcggugaaugcca....................   1     1   HS4
.......................................................................aaCgcacgcggugaaugcca....................   37    1   HS4
.......................................................................aaggcacgcgguCaaugcca....................   1     1   HS4
.......................................................................Uaggcacgcggugaaugccaa...................   1     1   HS4
.......................................................................aaggcacgcggugaaugccaC...................   1     1   HS4
.......................................................................aaggcacgcggugGaugccaa...................   1     1   HS4
.......................................................................aaggcacgcggugaaugccaa...................   14    0   HS4
.......................................................................aaggcacgcggugaaugccaU...................   39    1   HS4
.......................................................................aaCgcacgcggugaaugccaa...................   1     1   HS4
.......................................................................aaggcacgcggugaaugccaaU..................   5     1   HS4
.......................................................................aaggcacgcggugaaugccaaUu.................   2     1   HS4
........................................................................aCgcacgcggugaaugcc.....................   4     1   HS4
........................................................................aUgcacgcggugaaugcc.....................   18    1   HS4
........................................................................aAgcacgcggugaaugcc.....................   3     1   HS4
........................................................................aggcacgcggugaaugcc.....................   2     0   HS4
........................................................................aggcacgcggugaaugcca....................   38    0   HS4
........................................................................aCgcacgcggugaaugcca....................   149   1   HS4
........................................................................aUgcacgcggugaaugcca....................   302   1   HS4
........................................................................Gggcacgcggugaaugcca....................   1     1   HS4
........................................................................aAgcacgcggugaaugcca....................   25    1   HS4
........................................................................aggcacgcggugaaugccaU...................   1     1   HS4
........................................................................aAgcacgcggugaaugccaa...................   2     1   HS4
........................................................................aUgcacgcggugaaugccaa...................   2     1   HS4
........................................................................aCgcacgcggugaaugccaa...................   1     1   HS4
........................................................................Uggcacgcggugaaugccaa...................   1     1   HS4
.........................................................................Agcacgcggugaaugcca....................   2     1   HS4
.........................................................................ggcacgcggugaaugcca....................   5     0   HS4
.........................................................................Ugcacgcggugaaugcca....................   1     1   HS4
.........................................................................ggcacgcggugaaugccaU...................   1     1   HS4
.........................................................................ggcacgcggugaaugccaa...................   1     0   HS4
..........................................................................gcacgcggugaaugccaa...................   2     0   HS4
..........................................................................gcacgcggugaaugccaU...................   4     1   HS4
............................................................................acgcggugaaugccaaUu.................   1     1   HS4

.....gcuuuguggaccuuuugugaucgcuu................................................................................   1     0   J17
.......uuguggaccuuuugugaucgcuu................................................................................   9     0   J17
........uuguggaccuuuugugaucgcu.................................................................................   1     0   J17
........uuguggaccuuuugugaucgcuu................................................................................   3     0   J17
```

```
                              Star                                    Mature
gugaugcuuuguggaccuuuugugaucgcuucguguucacuguuugccuugaugucaauguuugauuucauaaggcacgcggugaaugccaagugcaaaggucaucauucg
........................aucgcuucguguucacuguuug.................................................................   1     0   J17
.........................ucgcuucguguucacuguuug.................................................................   2     0   J17
.........................ucgcuucguguucacuguuugc................................................................   2     0   J17
.............................uucguguucacuguuugccuug............................................................   2     0   J17
..............................ucguguucacuguuugccuugau..........................................................   1     0   J17
..............................Acguguucacuguuugccuugaug.........................................................   1     1   J17
..............................ucguguucacuguuugccuugaug.........................................................   1     0   J17
...............................cguguucacuguuugccu..............................................................   4     0   J17
...............................cguguucacuguuugccuA.............................................................   1     1   J17
...............................cguguucacuguuugccuu.............................................................   9     0   J17
...............................cguguucacuguuugccuuA............................................................   2     1   J17
...............................cguguucacuguCugccuug............................................................   1     1   J17
...............................cguguucacuguuugccuug............................................................   181   0   J17
...............................cguguucacuguuugccuugU...........................................................   1     1   J17
...............................cguguucacuguuugccuuga...........................................................   163   0   J17
...............................cguguucacCguuugccuuga...........................................................   1     1   J17
...............................cguguucacuguuugccuugau..........................................................   418   0   J17
...............................cguUuucacuguuugccuugau..........................................................   1     1   J17
...............................cguguAcacuguuugccuugau..........................................................   1     1   J17
...............................Aguguucacuguuugccuugau..........................................................   1     1   J17
...............................cguguucacuguAugccuugau..........................................................   1     1   J17
...............................cguguucacuguuCgccuugau..........................................................   1     1   J17
...............................cgGguucacuguuugccuugaug.........................................................   1     1   J17
...............................cguguucacuUuuugccuugaug.........................................................   1     1   J17
...............................cguguucacuguuugGcuugaug.........................................................   1     1   J17
...............................cguguucacuguuuAccuugaug.........................................................   3     1   J17
...............................Uguguucacuguuugccuugaug.........................................................   2     1   J17
...............................cguguucacCguuugccuugaug.........................................................   1     1   J17
...............................cguguucacuguuugcUuugaug.........................................................   1     1   J17
...............................cguguucacuguuGgccuugaug.........................................................   1     1   J17
...............................cguguucacuguuAgccuugaug.........................................................   4     1   J17
...............................cguguucacuguuugccuCgaug.........................................................   1     1   J17
...............................cguguucacuguuugccuugauC.........................................................   1     1   J17
...............................cguguuUacuguuugccuugaug.........................................................   1     1   J17
...............................cguguucacuguuugccuuCaug.........................................................   3     1   J17
...............................cgAguucacuguuugccuugaug.........................................................   2     1   J17
...............................cguguucacuguuugccuugauA.........................................................   14    1   J17
...............................cguguucacuguGugccuugaug.........................................................   2     1   J17
...............................cgugAucacuguuugccuugaug.........................................................   1     1   J17
...............................cguguucacuguuugccAugaug.........................................................   1     1   J17
...............................cguguucacuguuugccuugGug.........................................................   1     1   J17
...............................cguguucacuguuugUcuugaug.........................................................   7     1   J17
...............................cguguucacuguuugccuugaAg.........................................................   7     1   J17
...............................Aguguucacuguuugccuugaug.........................................................   9     1   J17
...............................cUuguucacuguuugccuugaug.........................................................   2     1   J17
...............................cguguucacuguuuCccuugaug.........................................................   1     1   J17
...............................cguguucacuguuugccuugauU.........................................................   5     1   J17
...............................cguguucacugGuugccuugaug.........................................................   3     1   J17
...............................cguguGcacuguuugccuugaug.........................................................   1     1   J17
...............................cguguucacugAuugccuugaug.........................................................   1     1   J17
...............................cguguucacuguuugccuGgaug.........................................................   1     1   J17
...............................cguguucacAguuugccuugaug.........................................................   1     1   J17
...............................cguCuucacuguuugccuugaug.........................................................   1     1   J17
...............................cguguCcacuguuugccuugaug.........................................................   1     1   J17
...............................cguguucaUuguuugccuugaug.........................................................   2     1   J17
...............................cguUuucacuguuugccuugaug.........................................................   4     1   J17
...............................cguguucacuguuugccuugaGg.........................................................   1     1   J17
...............................cguguucacuguCugccuugaug.........................................................   2     1   J17
...............................cguguucacuguuugAcuugaug.........................................................   2     1   J17
...............................cguguucacuguuugccuuUaug.........................................................   3     1   J17
...............................cguguucacuguuugccuugaug.........................................................   6163  0   J17
...............................cguguucaAuguuugccuugaug.........................................................   3     1   J17
...............................cguguucacuguuCgccuugaug.........................................................   2     1   J17
...............................cCuguucacuguuugccuugaug.........................................................   2     1   J17
...............................cguguucacuguuugccuugUug.........................................................   2     1   J17
...............................cguguucacuguAugccuugaug.........................................................   2     1   J17
...............................cguguucacuguuugccuugaugu........................................................   13    0   J17
...............................cguguucacuguuugccuugaugA........................................................   60    1   J17
...............................cguguucacuguuugccuugauguc.......................................................   14    0   J17
...............................cguguucacuguuugccuugauguca......................................................   2     0   J17
```

Star Mature

```
gugaugcuuugugaccuuuugugaucgcuucguguucacuguuugccuugaugucaauguuugauuucauaaggcacgcggugaaugccaagugcaaaggucaucauucg
..............................cguguucacuguuugccuugaugucaa..................................................... 3 0 J17
...............................guguucacuguuugccuuga........................................................... 1 0 J17
...............................guguucacuguuugccuugau.......................................................... 4 0 J17
...............................guguucacuguuugccuugaug......................................................... 10 0 J17
...............................guguucacuguuugccuugauC......................................................... 1 1 J17
...............................guguucacuguuugccuugaugA........................................................ 1 1 J17
................................uguucacuguuugccuugau.......................................................... 2 0 J17
................................uguucacuguuugccuugaug......................................................... 6 0 J17
..................................uucacuguuugccuugaug......................................................... 3 0 J17
.....................................................ucaauguuugauuucauaaggc................................... 1 0 J17
.....................................................ucaauguuugauuAcauaaggc................................... 1 1 J17
................................................................uuucauaaggcacgcggugaaugc...................... 1 0 J17
.................................................................uucauaaggcacgcggugaaugc...................... 1 0 J17
.................................................................uucauaaggcacgcggugaaugcc..................... 1 0 J17
..................................................................ucauaaggcacgcggugaaugcc..................... 1 0 J17
...................................................................Gauaaggcacgcggugaaugcca.................... 1 1 J17
...................................................................Aauaaggcacgcggugaaugcca.................... 1 1 J17
....................................................................auaaggcacgcggugaau........................ 1 0 J17
....................................................................Uuaaggcacgcggugaaug....................... 1 1 J17
....................................................................auaaggcacgcggugaaug....................... 1 0 J17
....................................................................Uuaaggcacgcggugaaugc...................... 3 1 J17
....................................................................auaaggcacgcggugaaugc...................... 70 0 J17
....................................................................auaaggcacgcggugaaugcU..................... 1 1 J17
....................................................................auaaggcacgcggugUaugcc..................... 2 1 J17
....................................................................auaaggcacgcggugGaugcc..................... 1 1 J17
....................................................................Uuaaggcacgcggugaaugcc..................... 12 1 J17
....................................................................auaaggcacgcggugaaAgcc..................... 1 1 J17
....................................................................auaaggcacgcggugaaugcA..................... 1 1 J17
....................................................................auaaggcacgcggugaaugcc..................... 306 0 J17
....................................................................auaaggcacgcggAgaaugcc..................... 1 1 J17
....................................................................auaaggcacgcggugaaGgcca.................... 1 1 J17
....................................................................Guaaggcacgcggugaaugcca.................... 2 1 J17
....................................................................auaaggcacgcggAgaaugcca.................... 2 1 J17
....................................................................auaaggcacgcggugaCugcca.................... 1 1 J17
....................................................................auaaggcacgcggugaaugccU.................... 3 1 J17
....................................................................auaaggcacgcggugaGugcca.................... 1 1 J17
....................................................................auaaggcacgcggugaaugcca.................... 961 0 J17
....................................................................auaaggcacAcggugaaugcca.................... 1 1 J17
....................................................................auaaggcacgcggugaaugcAa.................... 1 1 J17
....................................................................auaaggcaAgcggugaaugcca.................... 1 1 J17
....................................................................auaagAcacgcggugaaugcca.................... 1 1 J17
....................................................................Uuaaggcacgcggugaaugcca.................... 39 1 J17
....................................................................auaaggcacgcggugaaugcUa.................... 2 1 J17
....................................................................auaaggcacgcggugaaugccaC................... 4 1 J17
....................................................................Uuaaggcacgcggugaaugccaa................... 3 1 J17
....................................................................auaaggcacgcggugaaugccaU................... 4 1 J17
....................................................................auaaggcacgcggugaaugUcaa................... 1 1 J17
....................................................................auaaggcacgcggugaaugccaa................... 11 0 J17
....................................................................auaaggcacgcggugaaugccaaU.................. 1 1 J17
.....................................................................uaaggcacgcggugGaug....................... 1 1 J17
.....................................................................uaaggcacgcggugaauA....................... 1 1 J17
.....................................................................uaaggcacgcggugaaug....................... 285 0 J17
.....................................................................uaaggcacgcUgugaaug....................... 1 1 J17
.....................................................................uaaggcacgcggugaauAc...................... 3 1 J17
.....................................................................uaaggcacgcggCgaaugc...................... 2 1 J17
.....................................................................uaaggcaGgcggugaaugc...................... 1 1 J17
.....................................................................uaaggcacgcggugaaugA...................... 77 1 J17
.....................................................................uaaggcacgcggugaaGgc...................... 1 1 J17
.....................................................................uaaggcacgcggugGaugc...................... 15 1 J17
.....................................................................uaaggAacgcggugaaugc...................... 2 1 J17
.....................................................................Aaaggcacgcggugaaugc...................... 1 1 J17
.....................................................................uaaggcacgcggugUaugc...................... 15 1 J17
.....................................................................uaaggcacgcggugaaugU...................... 31 1 J17
.....................................................................uaUggcacgcggugaaugc...................... 2 1 J17
.....................................................................uaagCcacgcggugaaugc...................... 3 1 J17
.....................................................................uaagAcacgcggugaaugc...................... 4 1 J17
.....................................................................uaaggcacUcggugaaugc...................... 1 1 J17
.....................................................................uaaggcacgcgAugaaugc...................... 3 1 J17
.....................................................................uaaggUacgcggugaaugc...................... 4 1 J17
.....................................................................uGaggcacgcggugaaugc...................... 1 1 J17
```

Star Mature

```
gugaugcuuuguggaccuuuugugaucgcuucguguucacuguuugccuugaugucaauguuugauuucauaaggcacgcggugaaugccaagugcaaaggucaucauucg
......................................................................uaaggcacgcggugCaugc......................  1      1  J17
......................................................................uaaggcaUgcggugaaugc......................  1      1  J17
......................................................................uaaggcacgcggugaCugc......................  2      1  J17
......................................................................uaaAgcacgcggugaaugc......................  2      1  J17
......................................................................uaaggcacgcggugaauCc......................  2      1  J17
......................................................................uaaggcacgUggugaaugc......................  8      1  J17
......................................................................uaGggcacgcggugaaugc......................  1      1  J17
......................................................................uaaggcacgcggugaaugc......................  6694   0  J17
......................................................................uaaggcacgAggugaaugc......................  1      1  J17
......................................................................uaaggcacgcggAgaaugc......................  3      1  J17
......................................................................uaaggcacgcggugaaCgc......................  2      1  J17
......................................................................uaaggcacgGggugaaugc......................  1      1  J17
......................................................................uaaggcacgcggGgaaugc......................  3      1  J17
......................................................................uaaggcacgcggugaaAgc......................  2      1  J17
......................................................................uaaggcacAcggugaaugc......................  5      1  J17
......................................................................uaaggcacgcAgugaaugc......................  4      1  J17
......................................................................uaaggGacgcggugaaugc......................  1      1  J17
......................................................................uaaggcUcgcggugaaugc......................  5      1  J17
......................................................................uaaggcacgcAgugaaugcc.....................  5      1  J17
......................................................................uaUggcacgcggugaaugcc.....................  3      1  J17
......................................................................uaaggcacUcggugaaugcc.....................  1      1  J17
......................................................................uaGggcacgcggugaaugcc.....................  2      1  J17
......................................................................uaaggcacgcggugaaugcA.....................  250    1  J17
......................................................................uaaggcacgcggugaaugcc.....................  10339  0  J17
......................................................................uaagUcacgcggugaaugcc.....................  5      1  J17
......................................................................uaaggcacAcggugaaugcc.....................  7      1  J17
......................................................................uUaggcacgcggugaaugcc.....................  1      1  J17
......................................................................uaaggcacgcggugaaugcU.....................  30     1  J17
......................................................................uaaggcacgcgAugaaugcc.....................  3      1  J17
......................................................................uaaggcacgcgguUaaugcc.....................  2      1  J17
......................................................................uaaggcacgcggugGaugcc.....................  34     1  J17
......................................................................uaaggcacgcggugaaugcG.....................  1      1  J17
......................................................................uaaUgcacgcggugaaugcc.....................  11     1  J17
......................................................................uaaggcacgcggGgaaugcc.....................  3      1  J17
......................................................................uaaggcacgcggugaauUcc.....................  3      1  J17
......................................................................uGaggcacgcggugaaugcc.....................  1      1  J17
......................................................................uaaAgcacgcggugaaugcc.....................  2      1  J17
......................................................................uaaggcaUgcggugaaugcc.....................  7      1  J17
......................................................................uaagCcacgcggugaaugcc.....................  6      1  J17
......................................................................uaaggcacgcggugaauAcc.....................  5      1  J17
......................................................................uaaggcacgUggugaaugcc.....................  13     1  J17
......................................................................uaaggcacgcggugUaugcc.....................  38     1  J17
......................................................................uaaggcacgcggugCaugcc.....................  5      1  J17
......................................................................uaaggcacgcggugaUugcc.....................  1      1  J17
......................................................................uaaggUacgcggugaaugcc.....................  10     1  J17
......................................................................uaaggcacgcggugaaugAc.....................  2      1  J17
......................................................................uaaCgcacgcggugaaugcc.....................  8      1  J17
......................................................................uaaggcacgcggugaaAgcc.....................  5      1  J17
......................................................................Aaaggcacgcggugaaugcc.....................  5      1  J17
......................................................................uaaggcacgcggugaauCcc.....................  20     1  J17
......................................................................uaaggcacgcggugaaugUc.....................  36     1  J17
......................................................................uaCggcacgcggugaaugcc.....................  2      1  J17
......................................................................uaaggcacgcggugaaCgcc.....................  3      1  J17
......................................................................uaaggcacgcggugaCugcc.....................  1      1  J17
......................................................................uaaggcacgcgCugaaugcc.....................  1      1  J17
......................................................................uaaggcacgcgguAaaugcc.....................  2      1  J17
......................................................................uaaggcacgcggugaaugGc.....................  1      1  J17
......................................................................uaaggcUcgcggugaaugcc.....................  2      1  J17
......................................................................uaaggcacgcggCgaaugcc.....................  1      1  J17
......................................................................uaaggcacgcggAgaaugcc.....................  6      1  J17
......................................................................uaUggcacgcggugaaugcca....................  46     1  J17
......................................................................uaaggUacgcggugaaugcca....................  82     1  J17
......................................................................uaaggcacgcggugaCugcca....................  42     1  J17
......................................................................uaaggcacgcggugaaugcAa....................  13     1  J17
......................................................................uaagCcacgcggugaaugcca....................  32     1  J17
......................................................................uaagAcacgcggugaaugcca....................  16     1  J17
......................................................................Caaggcacgcggugaaugcca....................  6      1  J17
......................................................................uaaggcacUcggugaaugcca....................  8      1  J17
......................................................................uaaggGacgcggugaaugcca....................  10     1  J17
......................................................................uaaggcacgcggugaaugccC...................  6      1  J17
```

```
                              Star                          Mature
gugaugcuuuguggaccuuuugugaucgcuucguguucacuguuugccuugaugucaauguuugauuucauaaggcacgcggugaaugccaagugcaaaggucaucauucg
......................................................................uaaggcacgcAgugaaugcca....................   22      1     J17
......................................................................uaaggcacgcggugaaugcGa....................   10      1     J17
......................................................................uaaggcacgcggugaaugcca....................   92208   0     J17
......................................................................uaaggcacgcgguCaaugcca....................   6       1     J17
......................................................................uaaggcacgcggugaaugccG....................   16      1     J17
......................................................................uaaggcacgcggugaauAcca....................   138     1     J17
......................................................................uaaCgcacgcggugaaugcca....................   99      1     J17
......................................................................uaaggcacgcggGgaaugcca....................   23      1     J17
......................................................................uaaggcacgUggugaaugcca....................   69      1     J17
......................................................................uaCggcacgcggugaaugcca....................   6       1     J17
......................................................................uaaggcacgcggugaaugGca....................   7       1     J17
......................................................................uaaggcacgcgguAaaugcca....................   10      1     J17
......................................................................Gaaggcacgcggugaaugcca....................   8       1     J17
......................................................................uaaggcacgcgguUaaugcca....................   6       1     J17
......................................................................uaaggcacgGggugaaugcca....................   7       1     J17
......................................................................uaaggcacgcggugaaugccU....................   37      1     J17
......................................................................uaaggcUcgcggugaaugcca....................   8       1     J17
......................................................................uaaggcacgcggugUaugcca....................   338     1     J17
......................................................................uaaggcacgcggugCaugcca....................   47      1     J17
......................................................................uGaggcacgcggugaaugcca....................   12      1     J17
......................................................................uaaggcacgcggugaaCgcca....................   17      1     J17
......................................................................uaGggcacgcggugaaugcca....................   25      1     J17
......................................................................uaaggcacgAggugaaugcca....................   4       1     J17
......................................................................uUaggcacgcggugaaugcca....................   18      1     J17
......................................................................uaaggcaUgcggugaaugcca....................   36      1     J17
......................................................................uaaggcacgcggugaauCcca....................   68      1     J17
......................................................................uaaggcacgcUgugaaugcca....................   3       1     J17
......................................................................uaaggcacgcgUugaaugcca....................   10      1     J17
......................................................................uaaggcaAgcggugaaugcca....................   2       1     J17
......................................................................uaaggcaGgcggugaaugcca....................   8       1     J17
......................................................................uaaggcacgcggugaauUcca....................   46      1     J17
......................................................................uaaggcacgcgCugaaugcca....................   4       1     J17
......................................................................uaaUgcacgcggugaaugcca....................   73      1     J17
......................................................................uaaggcacgcggugaaugUca....................   385     1     J17
......................................................................uaaggcGcgcggugaaugcca....................   5       1     J17
......................................................................uaaggcacgcggCgaaugcca....................   9       1     J17
......................................................................uaaggcacgcgAugaaugcca....................   23      1     J17
......................................................................uaaggcacgcggugaaugcUa....................   25      1     J17
......................................................................uaaAgcacgcggugaaugcca....................   12      1     J17
......................................................................uCaggcacgcggugaaugcca....................   11      1     J17
......................................................................uaaggcacgcCgugaaugcca....................   2       1     J17
......................................................................uaaggcacgcggAgaaugcca....................   43      1     J17
......................................................................uaagUcacgcggugaaugcca....................   38      1     J17
......................................................................uaaggcacgcggugaGugcca....................   22      1     J17
......................................................................uaaggcacAcggugaaugcca....................   48      1     J17
......................................................................uaaggcacgcggugaUugcca....................   35      1     J17
......................................................................Aaaggcacgcggugaaugcca....................   55      1     J17
......................................................................uaaggcacgcggugaaGgcca....................   15      1     J17
......................................................................uaaggAacgcggugaaugcca....................   9       1     J17
......................................................................uaaggcacCcggugaaugcca....................   4       1     J17
......................................................................uaaggcacgcggugaaugAca....................   8       1     J17
......................................................................uaaggcacgcggugGaugcca....................   177     1     J17
......................................................................uaaggcacgcggugaaAgcca....................   50      1     J17
......................................................................uaaggcacgcggugaaugcUaa...................   2       1     J17
......................................................................uaaggcacgcggugaaGgccaa...................   1       1     J17
......................................................................uCaggcacgcggugaaugccaa...................   1       1     J17
......................................................................uaaggcacgcggugaaugccaC...................   5323    1     J17
......................................................................uaaggcacgcggugaaugUcaa...................   25      1     J17
......................................................................uaaCgcacgcggugaaugccaa...................   2       1     J17
......................................................................uaaggcacgAggugaaugccaa...................   1       1     J17
......................................................................uaaggcacgcggugaauCccaa...................   2       1     J17
......................................................................uaaggcacgcggugaaugGcaa...................   3       1     J17
......................................................................uaaggcacgcgCugaaugccaa...................   1       1     J17
......................................................................uaaggcacgcggugaGugccaa...................   4       1     J17
......................................................................uaaggcGcgcggugaaugccaa...................   1       1     J17
......................................................................uaaggcacgcAgugaaugccaa...................   4       1     J17
......................................................................uaagUcacgcggugaaugccaa...................   2       1     J17
......................................................................uaaggcacgcgAugaaugccaa...................   7       1     J17
......................................................................uaaggcUcgcggugaaugccaa...................   4       1     J17
......................................................................uaaggcacgcgguAaaugccaa...................   2       1     J17
```

Star Mature

gugaugcuuuguggaccuuuugugaucgcuucguguucacuguuugccuugaugucaauguuugauuucauaaggcacgcggugaaugccaagugcaaaggucaucauucg

....uaaggcaUgcggugaaugccaa...................	5	1	J17
....uaaggcacgcgguUaaugccaa...................	2	1	J17
....uaUggcacgcggugaaugccaa...................	15	1	J17
....uaaggcacgcCgugaaugccaa...................	1	1	J17
....uaaggcacgUggugaaugccaa...................	15	1	J17
....uaaggcacgcggugCaugccaa...................	12	1	J17
....uaaggGacgcggugaaugccaa...................	5	1	J17
....uaaggcacgcggugaaugccaG...................	6	1	J17
....uGaggcacgcggugaaugccaa...................	3	1	J17
....uaaggcacgcggugaaugcGaa...................	1	1	J17
....uaaggcacgcggAgaaugccaa...................	9	1	J17
....uaaggcacgGggugaaugccaa...................	1	1	J17
....uaaggcacgcggugaaugccaa...................	13847	0	J17
....Caaggcacgcggugaaugccaa...................	1	1	J17
....uaaggcacUcggugaaugccaa...................	2	1	J17
....uaaggcacgcggugaaugccUa...................	4	1	J17
....uaaggcacgcggugaaCgccaa...................	2	1	J17
....uaaggcacgcggugaaugccGa...................	5	1	J17
....uaGggcacgcggugaaugccaa...................	1	1	J17
....uaaggcacgcggugaaugccaU...................	9906	1	J17
....uaagAcacgcggugaaugccaa...................	1	1	J17
....uaaggcacgcggugaaugcAaa...................	2	1	J17
....uaaggcacgcggGgaaugccaa...................	3	1	J17
....uaaggcacgcggugaCugccaa...................	4	1	J17
....uaaggcacgcggugaauAccaa...................	18	1	J17
....uUaggcacgcggugaaugccaa...................	1	1	J17
....uaaggcacgcggugaauUccaa...................	10	1	J17
....uaaggcacCcggugaaugccaa...................	1	1	J17
....uaaggcacAcggugaaugccaa...................	7	1	J17
....uaaggcacgcggugaUugccaa...................	8	1	J17
....uaaggcacgcggugGaugccaa...................	23	1	J17
....uaaUgcacgcggugaaugccaa...................	5	1	J17
....uaaggUacgcggugaaugccaa...................	6	1	J17
....Aaaggcacgcggugaaugccaa...................	9	1	J17
....uaaggcacgcggCgaaugccaa...................	1	1	J17
....uaaggcacgcggugUaugccaa...................	42	1	J17
....uaaggcacgcUgugaaugccaa...................	1	1	J17
....uaaggcacgcggugaaAgccaa...................	13	1	J17
....uaaggcacgcggugaaugccaag..................	28	0	J17
....uaaggcacgcggugaaugccaaC..................	85	1	J17
....uaaggcacgcggugaaugccaaA..................	850	1	J17
....uaaggcacgcggugaaugccaUg..................	148	1	J17
....uaaggcacgcggugaaugccaaU..................	2123	1	J17
....uaaggcacgcggugaaugccaUgu.................	6	1	J17
....uaaggcacgcggugaaugccaagA.................	2	1	J17
....uaaggcacgcggugaaugccaaAu.................	17	1	J17
....uaaggcacgcggAgaaugccaagu.................	1	1	J17
....uaaggcacgcggugaaugccaaCu.................	22	1	J17
....uaaggcacgcggugaaugccaaUu.................	754	1	J17
....uaaggcacgcggugaaugccaagu.................	5	0	J17
....aaggcacgcggugaaugc......................	15	0	J17
....Gaggcacgcggugaaugcc.....................	1	1	J17
....Uaggcacgcggugaaugcc.....................	4	1	J17
....aaggcacgcggugGaugcc.....................	1	1	J17
....aaggcacgcggugaaugcc.....................	18	0	J17
....aaggcacgcggugaaugcA.....................	1	1	J17
....aaggGacgcggugaaugcca....................	1	1	J17
....aaUgcacgcggugaaugcca....................	3	1	J17
....aaggcacgcggugaaugUca....................	3	1	J17
....aaCgcacgcggugaaugcca....................	5	1	J17
....Uaggcacgcggugaaugcca....................	15	1	J17
....aaggcacgcggugaaugcca....................	142	0	J17
....aGggcacgcggugaaugcca....................	2	1	J17
....aaggcacgcggugaaugccaa...................	17	0	J17
....aaggcacgcggugaaugccaU...................	27	1	J17
....Uaggcacgcggugaaugccaa...................	1	1	J17
....aaggcacgcggugaauUccaa...................	2	1	J17
....aaggcacgcggugaaugccaC...................	5	1	J17
....aaggcacgcggugaaugccaaA..................	2	1	J17
....aaggcacgcggugaaugccaaU..................	3	1	J17

```
                              Star                                   Mature
gugaugcuuuguggaccuuuugugaucgcuucguguucacuguuugccuugaugucaauguuugauuucauaaggcacgcggugaaugccaagugcaaaggucaucauucg
.......................................................................aaggcacgcggugaaugccaaUu.................   4    1    J17
........................................................................aAgcacgcggugaaugcc.....................   1    1    J17
........................................................................aUgcacgcggugaaugcc.....................   2    1    J17
........................................................................aCgcacgcggugaaugcca....................   4    1    J17
........................................................................aUgcacgcggugaaugcca....................   19   1    J17
........................................................................aAgcacgcggugaaugcca....................   8    1    J17
........................................................................aggcacgcggugaaugcca....................   11   0    J17
........................................................................aUgcacgcggugaaugccaa...................   1    1    J17
.........................................................................Ugcacgcggugaaugcca....................   1    1    J17
.........................................................................ggcacgcggugaaugccaa...................   2    0    J17
..........................................................................gcacgcggugaaugccaa...................   3    0    J17
..........................................................................gcacgcggugaaugccaU...................   1    1    J17
..........................................................................gcacgcggugaaugccaaU..................   1    1    J17
..........................................................................gcacgcggugaaugccaaA..................   1    1    J17
..........................................................................gcacgcggugaaugccaaUu.................   1    1    J17
...........................................................................cacgcggugaaugccaaA..................   1    1    J17
...........................................................................cacgcggugaaugccaaU..................   6    1    J17

...............................cguguucacuguuugccu..............................................................   1    0    EGG
...............................cguguucacuguuugccuug............................................................   2    0    EGG
...............................cguguucacuguuugccuugaug.........................................................   1    0    EGG
.....................................................................auaaggcacgcggugaau........................   1    0    EGG
.....................................................................Guaaggcacgcggugaaugc......................   1    1    EGG
.....................................................................Uuaaggcacgcggugaaugc......................   1    1    EGG
.....................................................................auaaggcacgcggugaaugc......................   2    0    EGG
.....................................................................auaaggcacgcggugaaugcc.....................   1    0    EGG
.....................................................................Uuaaggcacgcggugaaugcc.....................   4    1    EGG
.....................................................................Uuaaggcacgcggugaaugcca....................   4    1    EGG
.....................................................................auaaggcacgcggugaaugcca....................   2    0    EGG
......................................................................uaaggcacgcggugaauU.......................   4    1    EGG
......................................................................uaaggcacgcggugaaug.......................   25   0    EGG
......................................................................uaaggUacgcggugaaug.......................   2    1    EGG
......................................................................uaagAcacgcggugaaugc......................   1    1    EGG
......................................................................uaaggcacgcggugaaugc......................   510  0    EGG
......................................................................uaaggcacgcggugaCugc......................   1    1    EGG
......................................................................uaaggcacgcggugaaugA......................   6    1    EGG
......................................................................uaaggcacgcggugaaugU......................   3    1    EGG
......................................................................uaaggcacgcggugCaugc......................   1    1    EGG
......................................................................uaaggcacCcggugaaugc......................   1    1    EGG
......................................................................uaaggcacgUggugaaugc......................   3    1    EGG
......................................................................uaaCgcacgcggugaaugcc.....................   1    1    EGG
......................................................................uaaggcacgcggugaaCgcc.....................   2    1    EGG
......................................................................uaaggUacgcggugaaugcc.....................   1    1    EGG
......................................................................uaaggcacgcggugaUugcc.....................   1    1    EGG
......................................................................uaaggcacgcggugaaugUc.....................   5    1    EGG
......................................................................uaaggcacgcggugCaugcc.....................   1    1    EGG
......................................................................uaaggcaUgcggugaaugcc.....................   1    1    EGG
......................................................................uaagCcacgcggugaaugcc.....................   1    1    EGG
......................................................................uaaggcacgcggugaaugcG.....................   2    1    EGG
......................................................................uGaggcacgcggugaaugcc.....................   1    1    EGG
......................................................................uaaggcacgcggugGaugcc.....................   1    1    EGG
......................................................................uaaggcacgcggugaauAcc.....................   2    1    EGG
......................................................................uaaggcacgcggugaaugcc.....................   900  0    EGG
......................................................................Caaggcacgcggugaaugcc.....................   1    1    EGG
......................................................................uaaggcacgcggugaauCcc.....................   4    1    EGG
......................................................................uaaggcacgcggugaaugcA.....................   47   1    EGG
......................................................................uaaggcacgcggugaaugcU.....................   8    1    EGG
......................................................................uaaggcacgcggugUaugcc.....................   1    1    EGG
......................................................................uaaggcacgcggugaaAgcca....................   1    1    EGG
......................................................................uaaggcaGgcggugaaugcca....................   2    1    EGG
......................................................................uaagUcacgcggugaaugcca....................   1    1    EGG
......................................................................uaaggcacgcggCgaaugcca....................   1    1    EGG
......................................................................uaaggcacgcCgugaaugcca....................   1    1    EGG
......................................................................uaaggcCcgcggugaaugcca....................   1    1    EGG
......................................................................uaaggcaUgcggugaaugcca....................   1    1    EGG
......................................................................uaaggcacgcggugaCugcca....................   2    1    EGG
......................................................................uaaggcacgcggugUaugcca....................   3    1    EGG
......................................................................uaaggcacgcggugaaugcca....................   2519 0    EGG
......................................................................uaaggcacgcggugaauAcca....................   2    1    EGG
......................................................................uaagCcacgcggugaaugcca....................   1    1    EGG
```

Star Mature

```
gugaugcuuuguggaccuuuugugaucgcuucguguucacuguuugccuugaugucaauguuugauuucauaaggcacgcggugaaugccaagugcaaaggucaucauucg
......................................................................uaaggcacgcggugaaugccC....................	2	1	EGG
......................................................................uaaggcacgcggugaaugccU....................	8	1	EGG
......................................................................uaaggcacgUggugaaugcca....................	4	1	EGG
......................................................................uaaggcacgcggugaaugccG....................	2	1	EGG
......................................................................uaaggcacgcggugaaCgcca....................	1	1	EGG
......................................................................uaaggcacgcggugaGugcca....................	1	1	EGG
......................................................................uaaggcacgcggugGaugcca....................	1	1	EGG
......................................................................uaaggcacgcggugaaugcAa....................	1	1	EGG
......................................................................uaaggcacgcggugaaugUca....................	2	1	EGG
......................................................................uaaggcacgcggugaUugcca....................	1	1	EGG
......................................................................uaaggcacgcggugaauAccaa...................	1	1	EGG
......................................................................uaaggcacgcggugaaugccaa...................	25	0	EGG
......................................................................uaaggcacgcggugaaugccaU...................	55	1	EGG
......................................................................uaaggcacgcggugaaugccaC...................	9	1	EGG
......................................................................uaaggcacgcggugaaugccaaU..................	13	1	EGG
......................................................................uaaggcacgcggugaaugccaaA..................	2	1	EGG
......................................................................uaaggcacgcggugaaugccaaUu.................	3	1	EGG
......................................................................uaaggcacgcggugaaugccaagG.................	1	1	EGG
.......................................................................aaggcacgcggugaaugc.......................	1	0	EGG
.......................................................................aaggcacgcggugaaugcc......................	3	0	EGG
.......................................................................aaggcacgcggugaaugcca.....................	6	0	EGG
........................................................................aAgcacgcggugaaugcca.....................	1	1	EGG
```

Provisional ID	: SczTNLB_2740_4202
Score total	: 6863581.9
Score for star read(s)	: 3.9
Score for read counts	: 6863571.8
Score for mfe	: 1.5
Score for randfold	: 1.6
Score for cons. seed	: 3
Total read count	: 13462612
Mature read count	: 13208385
Loop read count	: 26
Star read count	: 254201

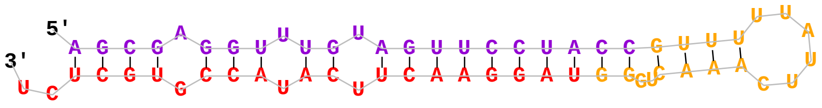

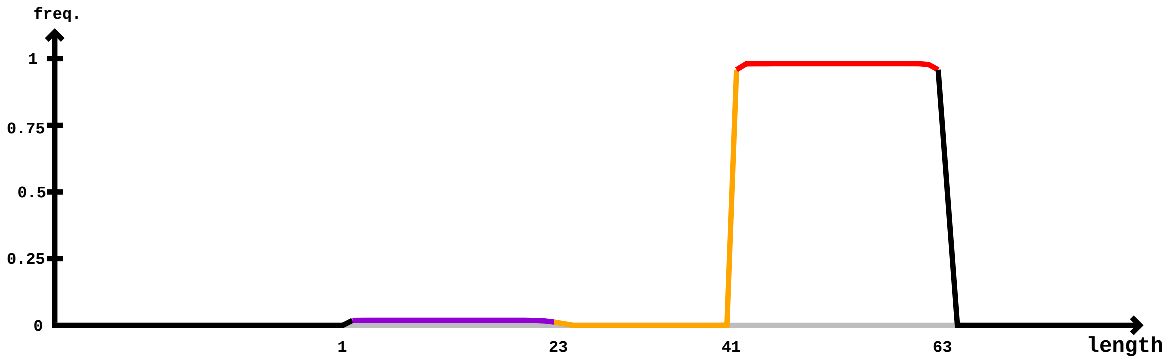

Star   Mature

```
5'- caaaaaaaaucgaauuacacuucgucacgagcgagguuuguaguuccuaccguuuuuauucaaacuggguaggaacuucauaccgugcucuugaacgaugcugacgaucaa  -3'   obs
    caaaaaaaaucgaauuacacuucgucacgagcgagguuuguaguuccuaccguuuuuauucaaacuggguaggaacuucauaccgugcucuugaacgaugcugacgaucaa         exp
    ........((((..(((((..((((((.(((((.(((.((.((((((((((((((......))))..)))))))))).)).))).))))).)).)))))).)))))))...   reads   mm   sample
    .............................agcgagguuuguaguucc................................................................   17      0    EGG
    .............................agcgagguuuguaguuccu...............................................................   104     0    EGG
    .............................agcgagguuuguaCuuccu...............................................................   1       1    EGG
    .............................agcgUgguuuguaguuccua..............................................................   1       1    EGG
    .............................agcgagguuuguaguuccua..............................................................   154     0    EGG
    .............................agUgagguuuguaguuccua..............................................................   1       1    EGG
    .............................agcgagguuuguaguuccuac.............................................................   127     0    EGG
    .............................agcgagguuuguaguucUuac.............................................................   1       1    EGG
    .............................agcgagguuuguaguuccuaU.............................................................   2       1    EGG
    .............................agcgagguuuguaguuccuacU............................................................   95      1    EGG
    .............................agcgagguuuguaguuccuacc............................................................   86      0    EGG
    .............................agcgagguuuguaguuccuacA............................................................   48      1    EGG
    .............................agcgagguuuguaguuccuaccU...........................................................   26      1    EGG
    .............................agcgagguuuguaguuccuaccA...........................................................   8       1    EGG
    .............................agcgagguuuguaguuccuaccguu.........................................................   1       0    EGG
    ................................gagguuuguaguuccuac.............................................................   2       0    EGG
    ................................gagguuuguaguuccuacU............................................................   2       1    EGG
    ................................gagguuuguaguuccuaccU...........................................................   1       1    EGG
    .................................agguuuguaguuccuaccguu.........................................................   1       0    EGG
    ...................................................................Aguaggaacuucauaccgugcucu....................   1       1    EGG
    ....................................................................Uuaggaacuucauaccgugcucu....................   18      1    EGG
    .....................................................................uaggaacuucauaccgug........................   55      0    EGG
    .....................................................................uaggaacuucauacUgug........................   1       1    EGG
    .....................................................................uaggaacuucauaccgugA.......................   8       1    EGG
    .....................................................................uaggaacuucauaccgugG.......................   1       1    EGG
    .....................................................................uaggaacuucauaccgugc.......................   38      0    EGG
    .....................................................................uaggaacuucauaccgugU.......................   2       1    EGG
    .....................................................................uaggaacuucauaUcgugc.......................   1       1    EGG
    .....................................................................uaggaacuucauaccgugcG......................   1       1    EGG
    .....................................................................uagCaacuucauaccgugcu......................   1       1    EGG
    .....................................................................uaggaacuucauaccgugcC......................   2       1    EGG
    .....................................................................uaggaacuuUauaccgugcu......................   1       1    EGG
    .....................................................................uaggaacuucauaccgugcA......................   2       1    EGG
```

Star Mature

```
caaaaaaaaucgaauuacacuucgucacgagcgagguuuguaguuccuaccguuuuuauucaaacuggguaggaacuucauaccgugcucuugaacgaugcugacgaucaa
.....................................................................uaggaacuucauaccgugcu......................  256    0  EGG
.....................................................................uaggaacuucauaccgugcAc.....................  2      1  EGG
.....................................................................uaggaacuucauacUgugcuc.....................  1      1  EGG
.....................................................................uaggaacuucauaccgugcuc.....................  936    0  EGG
.....................................................................uaggaacuucauaccAugcuc.....................  1      1  EGG
.....................................................................uaggaacuucauaccgugcuU.....................  39     1  EGG
.....................................................................uaggaacuAcauaccgugcuc.....................  1      1  EGG
.....................................................................uaggaacuucauaccUugcuc.....................  2      1  EGG
.....................................................................uaggGacuucauaccgugcuc.....................  1      1  EGG
.....................................................................uaggaacuucauaccguAcuc.....................  2      1  EGG
.....................................................................uaggaacuucauaccgugcuA.....................  9      1  EGG
.....................................................................uaggaacuucauaccgugcCc.....................  1      1  EGG
.....................................................................uaggaacuucauaccgGgcucu....................  3      1  EGG
.....................................................................uaggaacuuUauaccgugcucu....................  23     1  EGG
.....................................................................uaggaacuucauUccgugcucu....................  3      1  EGG
.....................................................................uaggCacuucauaccgugcucu....................  1      1  EGG
.....................................................................uaggaacuucauaccgugcucC....................  207    1  EGG
.....................................................................uaggaacuucauaUcgugcucu....................  7      1  EGG
.....................................................................uaggaacuucauaccguUcucu....................  1      1  EGG
.....................................................................uaggaacuucauaccUugcucu....................  6      1  EGG
.....................................................................uaggaacCucauaccgugcucu....................  1      1  EGG
.....................................................................uaggaacuCcauaccgugcucu....................  3      1  EGG
.....................................................................uagCaacuucauaccgugcucu....................  2      1  EGG
.....................................................................uaCgaacuucauaccgugcucu....................  2      1  EGG
.....................................................................uGggaacuucauaccgugcucu....................  2      1  EGG
.....................................................................uaggGacuucauaccgugcucu....................  10     1  EGG
.....................................................................uaggaacuucauaccguCcucu....................  1      1  EGG
.....................................................................uaggaacuucauaccgugcucG....................  5      1  EGG
.....................................................................uaggaacuucauaccgugUucu....................  9      1  EGG
.....................................................................uaggaacuucUuaccgugcucu....................  2      1  EGG
.....................................................................uaggaacuucaCaccgugcucu....................  2      1  EGG
.....................................................................uaggaGcuucauaccgugcucu....................  2      1  EGG
.....................................................................uaggaacuucaGaccgugcucu....................  2      1  EGG
.....................................................................uaggaacuucauaccgAgcucu....................  1      1  EGG
.....................................................................uaggaacuucauaccgugcuUu....................  4      1  EGG
.....................................................................uaAgaacuucauaccgugcucu....................  1      1  EGG
.....................................................................uaggaaAuucauaccgugcucu....................  1      1  EGG
.....................................................................uaggaacuucauaccguAcucu....................  4      1  EGG
.....................................................................uaggaacuucauaccgugcCcu....................  6      1  EGG
.....................................................................uaggUacuucauaccgugcucu....................  7      1  EGG
.....................................................................uUggaacuucauaccgugcucu....................  2      1  EGG
.....................................................................uaggaacuucauaccgugcucu....................  17118  0  EGG
.....................................................................uaggaacAucauaccgugcucu....................  1      1  EGG
.....................................................................uaggaacuucauacUgugcucu....................  11     1  EGG
.....................................................................uaggaacuucauacGgugcucu....................  1      1  EGG
.....................................................................uaggaacuucauaccgCgcucu....................  1      1  EGG
.....................................................................uaggaacuucauaccgugcucA....................  10     1  EGG
.....................................................................uaggaacuucauaccAugcucu....................  5      1  EGG
.....................................................................Gaggaacuucauaccgugcucu....................  4      1  EGG
.....................................................................uagUaacuucauaccgugcucu....................  2      1  EGG
.....................................................................uaggaacuucauaccCugcucu....................  3      1  EGG
.....................................................................uaggaacuucauaccgugcAcu....................  3      1  EGG
.....................................................................uaggaaUuucauaccgugcucu....................  3      1  EGG
.....................................................................uagAaacuucauaccgugcucu....................  2      1  EGG
.....................................................................uaggaacGucauaccgugcucu....................  10     1  EGG
.....................................................................uaggaacuucauaccgugcucuu...................  68     0  EGG
.....................................................................uaggaacuucauaccgugcucuC...................  1      1  EGG
.....................................................................uaggaacuuUauaccgugcucuu...................  2      1  EGG
.....................................................................uaggaacuucauaccgugcucCu...................  3      1  EGG
.....................................................................uaggaacuucauaccgugcucuG...................  1      1  EGG
.....................................................................uaggaacuucauaccgugcucuA...................  42     1  EGG
.....................................................................uaggaacuucauaccgugcucuuA..................  1      1  EGG
.....................................................................uaggaacuucauaccgugcucuuU..................  4      1  EGG
......................................................................aggaacuucauaccgugcu......................  1      0  EGG
......................................................................aggaacuucauaccgugcuc.....................  3      0  EGG
......................................................................aggaacuucauaccgugcucC....................  1      1  EGG
......................................................................aggaacuucauaccgugcucu....................  40     0  EGG
......................................................................aggaacuGcauaccgugcucu....................  1      1  EGG
.......................................................................Ugaacuucauaccgugcuc.....................  1      1  EGG
.......................................................................ggaacuucauaccgugcucC....................  1      1  EGG
```

```
                             Star                                    Mature
caaaaaaaaucgaauuacacuucgucacgagcgagguuuguaguuccuaccguuuuauucaaacuggguaggaacuucauaccgugcucuugaacgaugcugacgaucaa
......................................................................Agaacuucauaccgugcucu....................   2      1     EGG
......................................................................ggaacuucauaccgugcucu....................   8      0     EGG
.......................................................................gaacuucauaccgugcucu....................   4      0     EGG
.......................................................................Uaacuucauaccgugcucu....................   1      1     EGG
........................................................................aacuucauaccgugcucu....................   20     0     EGG

..........cgaauuacacuucgucacg.................................................................................   1      0     HS4
..........................acgagcgagguuuguaguucc...............................................................   2      0     HS4
..........................acgagcgagguuuguaguuccua.............................................................   1      0     HS4
............................gagcgagguuuguaguuccu..............................................................   2      0     HS4
............................Uagcgagguuuguaguuccuac............................................................   2      1     HS4
............................Uagcgagguuuguaguuccuacc...........................................................   6      1     HS4
............................Uagcgagguuuguaguuccuaccg..........................................................   1      1     HS4
.............................agcgagguuuguaguucc...............................................................   15     0     HS4
.............................agcgagguuuguaguucA...............................................................   1      1     HS4
.............................agcgagguuuguaguuccA..............................................................   1      1     HS4
.............................aCcgagguuuguaguuccu..............................................................   1      1     HS4
.............................agcgagguuuguaguuccu..............................................................   764    0     HS4
.............................agcgagguuuguaguCccu..............................................................   2      1     HS4
.............................agcgagguuuguaCuuccu..............................................................   3      1     HS4
.............................agUgagguuuguaguuccua.............................................................   2      1     HS4
.............................agcgagUuuuguaguuccua.............................................................   1      1     HS4
.............................agcgagAuuuguaguuccua.............................................................   2      1     HS4
.............................agcgagguuAguaguuccua.............................................................   1      1     HS4
.............................agcgagguuuguaguuccuU.............................................................   6      1     HS4
.............................agcgagCuuuguaguuccua.............................................................   4      1     HS4
.............................agAgagguuuguaguuccua.............................................................   1      1     HS4
.............................agcgaggAuuguaguuccua.............................................................   1      1     HS4
.............................agcgagguuuguaAuuccua.............................................................   1      1     HS4
.............................agcgagguuuguaguGccua.............................................................   4      1     HS4
.............................agcCagguuuguaguuccua.............................................................   2      1     HS4
.............................agcUagguuuguaguuccua.............................................................   5      1     HS4
.............................agcgagguuuguaguuUcua.............................................................   1      1     HS4
.............................agcgagguuuguaguAccua.............................................................   1      1     HS4
.............................agcgagguuuguaguuccCa.............................................................   4      1     HS4
.............................agcgaUguuuguaguuccua.............................................................   1      1     HS4
.............................agcgagguuuguaCuuccua.............................................................   3      1     HS4
.............................agcgagguuuguagAuccua.............................................................   1      1     HS4
.............................agcgagguuuguaUuuccua.............................................................   1      1     HS4
.............................aCcgagguuuguaguuccua.............................................................   3      1     HS4
.............................Ugcgagguuuguaguuccua.............................................................   2      1     HS4
.............................agcgaCguuuguaguuccua.............................................................   2      1     HS4
.............................agcgaggGuuguaguuccua.............................................................   4      1     HS4
.............................agcgagguuuguaguuccua.............................................................   2423   0     HS4
.............................agcgagguuGguaguuccua.............................................................   1      1     HS4
.............................agcgagguuuguaguCccuac............................................................   2      1     HS4
.............................agcgagguuugCaguuccuac............................................................   1      1     HS4
.............................agcgagguuuguaguuccuac............................................................   3885   0     HS4
.............................agcgagguuuguaguuccuUc............................................................   1      1     HS4
.............................agcgagguuuguaguuccuaU............................................................   25     1     HS4
.............................agcgagCuuuguaguuccuac............................................................   5      1     HS4
.............................agcgagguuAguaguuccuac............................................................   3      1     HS4
.............................agcgagAuuuguaguuccuac............................................................   1      1     HS4
.............................agcgagguuuguaguuccuaG............................................................   1      1     HS4
.............................agcgagguuuguaguAccuac............................................................   2      1     HS4
.............................aUcgagguuuguaguuccuac............................................................   6      1     HS4
.............................agcCagguuuguaguuccuac............................................................   1      1     HS4
.............................agcUagguuuguaguuccuac............................................................   3      1     HS4
.............................agcgagguuuguUguuccuac............................................................   2      1     HS4
.............................agUgagguuuguaguuccuac............................................................   5      1     HS4
.............................agcgagguuuguGguuccuac............................................................   2      1     HS4
.............................aCcgagguuuguaguuccuac............................................................   5      1     HS4
.............................agAgagguuuguaguuccuac............................................................   1      1     HS4
.............................agcgaggGuuguaguuccuac............................................................   5      1     HS4
.............................agcgagUuuuguaguuccuac............................................................   3      1     HS4
.............................agGgagguuuguaguuccuac............................................................   1      1     HS4
.............................agcgagguuuguaguucUuac............................................................   5      1     HS4
.............................agcgagguuuguaguuUcuac............................................................   1      1     HS4
.............................agcgagguuuguaguuccuGc............................................................   1      1     HS4
.............................agcgUgguuuguaguuccuac............................................................   2      1     HS4
```

Star **Mature**

```
caaaaaaaaucgaauuacacuucgucacgagcgagguuuguaguuccuaccguuuuuauucaaacugggcuaggaacuucauaccgugcucuugaacgaugcugacgaucaa
.............................agcgagguuuAuaguuccuac.............................................................	1	1	HS4
.............................agcgagguuuguagAuccuac.............................................................	1	1	HS4
.............................agcgagguuuguaguGccuac.............................................................	1	1	HS4
.............................agcgagguuuguaguuccGac.............................................................	1	1	HS4
.............................agcgagguuCguaguuccuac.............................................................	1	1	HS4
.............................agcgGgguuuguaguuccuac.............................................................	1	1	HS4
.............................agcgagguuuguaguuccuaA.............................................................	5	1	HS4
.............................agcgagAuuuguaguuccuacc............................................................	7	1	HS4
.............................agcgagguuuguaguuccCacc............................................................	2	1	HS4
.............................Ugcgagguuuguaguuccuacc............................................................	8	1	HS4
.............................agcgagguuuAuaguuccuacc............................................................	1	1	HS4
.............................agcgagguuuguaUuuccuacc............................................................	1	1	HS4
.............................agcgagguuugAaguuccuacc............................................................	2	1	HS4
.............................agcgagguuuguaguucUuacc............................................................	6	1	HS4
.............................agcgagguuuguaguucGuacc............................................................	1	1	HS4
.............................agcCagguuuguaguuccuacc............................................................	1	1	HS4
.............................aUcgagguuuguaguuccuacc............................................................	5	1	HS4
.............................agcgagguuuguaguAccuacc............................................................	6	1	HS4
.............................agcgagUuuuguaguuccuacc............................................................	6	1	HS4
.............................agcgagCuuuguaguuccuacc............................................................	9	1	HS4
.............................agcgagguuuguaguuccGacc............................................................	2	1	HS4
.............................agUgagguuuguaguuccuacc............................................................	17	1	HS4
.............................agcgagguuuguaguuccuaUc............................................................	5	1	HS4
.............................agcgagguuuguaguGccuacc............................................................	2	1	HS4
.............................agcgagguuuguaguuUcuacc............................................................	11	1	HS4
.............................agcgagguuuguaCuuccuacc............................................................	3	1	HS4
.............................agcgagguuuguaguuccuacA............................................................	276	1	HS4
.............................agcgUgguuuguaguuccuacc............................................................	4	1	HS4
.............................agcgagguuuguaguCccuacc............................................................	7	1	HS4
.............................agcgagguuuguagCuccuacc............................................................	2	1	HS4
.............................agcgaggGuuguaguuccuacc............................................................	25	1	HS4
.............................agcgagguuuguUguuccuacc............................................................	1	1	HS4
.............................agcgagguuAguaguuccuacc............................................................	5	1	HS4
.............................agcgaCguuuguaguuccuacc............................................................	1	1	HS4
.............................agcgaUguuuguaguuccuacc............................................................	2	1	HS4
.............................agcgagguuuguaAuuccuacc............................................................	2	1	HS4
.............................agcgagguuuguagGuccuacc............................................................	1	1	HS4
.............................agcgagguuuguaguuccuacU............................................................	1288	1	HS4
.............................agcgagguuuguaguuccuaAc............................................................	2	1	HS4
.............................agcgagguuuguaguuccuacc............................................................	12720	0	HS4
.............................agcUagguuuguaguuccuacc............................................................	6	1	HS4
.............................agcgagguuugCaguuccuacc............................................................	1	1	HS4
.............................Ggcgagguuuguaguuccuacc............................................................	2	1	HS4
.............................aCcgagguuuguaguuccuacc............................................................	2	1	HS4
.............................agcgagguuuguaguuccuacG............................................................	3	1	HS4
.............................agcgagguuuguaguuccAacc............................................................	1	1	HS4
.............................agcgagguuCguaguuccuacc............................................................	1	1	HS4
.............................agGgagguuuguaguuccuacc............................................................	2	1	HS4
.............................agcgagguuuguaguuccuaccU...........................................................	1316	1	HS4
.............................agcgagguuuguaguuccuaccA...........................................................	108	1	HS4
.............................agcgagguuuguaguuccuaccC...........................................................	3	1	HS4
.............................agcgagguuuguaguucUuaccg...........................................................	2	1	HS4
.............................agcgaCguuuguaguuccuaccg...........................................................	1	1	HS4
.............................agcgagguuuguaguuccuaccg...........................................................	324	0	HS4
.............................agcgagguuuguaguuccuaccCu..........................................................	1	1	HS4
.............................agcgagguuuguaguuccuaccUu..........................................................	7	1	HS4
.............................agcgagguuuguaguuccuaccgu..........................................................	4	0	HS4
.............................agcgagguuuguaguuccuaccguu.........................................................	1	0	HS4
.............................agcgagguuuguaguuccuaccUuu.........................................................	2	1	HS4
.............................agcgagguuuguaguuccuaccguuuu.......................................................	1	0	HS4
..............................gcgagguuuguaguuccuac.............................................................	14	0	HS4
..............................gcgagguuuguaguGccuacc............................................................	1	1	HS4
..............................gcgagguuuguaguuccuacU............................................................	1	1	HS4
..............................Acgagguuuguaguuccuacc............................................................	1	1	HS4
..............................gcgagguuuguaguuccuacc............................................................	58	0	HS4
..............................gcgagguuuguaguuccuaccA...........................................................	2	1	HS4
..............................gcgagguuuguaguuccuaccg...........................................................	79	0	HS4
...............................cgagguuuguaguuccuacc............................................................	19	0	HS4
...............................cgagguuuguaguuccuaccU...........................................................	2	1	HS4
...............................cgagguuuguaguuccuaccC...........................................................	8	1	HS4
```

```
                                 Star                                    Mature
caaaaaaaaucgaauuacacuucgucacgagcgagguuuguaguuccuaccguuuuuauucaaacuggguaggaacuucauaccgugcucuugaacgaugcugacgaucaa
...............................cgagguuuguaguuccuaccg...........................................................  19   0   HS4
...............................cgagguuuguaguuccuaccgA..........................................................  2    1   HS4
................................gagguuuguaguuccuac.............................................................  4    0   HS4
................................gagguuuguaguuccuacU............................................................  1    1   HS4
................................gagguuuguaguuccuacc............................................................  6    0   HS4
................................gagguuuguaguuccuaccguu.........................................................  1    0   HS4
................................gagguuuguaguuccuaccguuu........................................................  1    0   HS4
.................................agguuuguaguuccuacU............................................................  2    1   HS4
.................................agguuuguaguuccuacc............................................................  1    0   HS4
.................................agguuuguaguuccuaccguu.........................................................  1    0   HS4
.................................agguuuguaguuccuaccguuu........................................................  2    0   HS4
...................................guuuguaguuccuaccguuuu.......................................................  1    0   HS4
...................................guuuguaguuccuaccguuuuuau....................................................  2    0   HS4
....................................uuuguaguuccuaccguuuuu......................................................  1    0   HS4
...................................................guuuuuauucaaacuggg..........................................  10   0   HS4
...................................................guuuuuauuAaaacuggg..........................................  1    1   HS4
...................................................guuuuuauucaaacugggA.........................................  1    1   HS4
...........................................................ucaaacuggguaggaacuucau..............................  1    0   HS4
.................................................................Gggguaggaacuucauaccgugcu......................  1    1   HS4
.................................................................uggAuaggaacuucauaccgugcucu....................  1    1   HS4
.................................................................Cggguaggaacuucauaccgugcucu....................  1    1   HS4
..................................................................ggCuaggaacuucauaccgugcucu....................  8    1   HS4
..................................................................Agguaggaacuucauaccgugcucu....................  1    1   HS4
..................................................................ggUuaggaacuucauaccgugcucu....................  1    1   HS4
..................................................................ggguaggaacuucauaccgugcucu....................  13   0   HS4
..................................................................ggAuaggaacuucauaccgugcucu....................  2    1   HS4
...................................................................gCuaggaacuucauaccgugcucu....................  3    1   HS4
....................................................................guaggaacuucauaccgugc.......................  3    0   HS4
....................................................................Cuaggaacuucauaccgugcu......................  1    1   HS4
....................................................................guaggaacuuUauaccgugcu......................  1    1   HS4
....................................................................guaggaacuucauaccgugcu......................  89   0   HS4
....................................................................guaggaacuucauaccgCgcuc.....................  1    1   HS4
....................................................................Uuaggaacuucauaccgugcuc.....................  5    1   HS4
....................................................................guaggaacuucauaccgugcuU.....................  4    1   HS4
....................................................................guaggaacuucauaccgugcuc.....................  75   0   HS4
....................................................................Cuaggaacuucauaccgugcucu....................  11   1   HS4
....................................................................guaggaacuuUauaccgugcucu....................  1    1   HS4
....................................................................Auaggaacuucauaccgugcucu....................  2    1   HS4
....................................................................guaggaacuucauaccgugcucC....................  3    1   HS4
....................................................................Uuaggaacuucauaccgugcucu....................  321  1   HS4
....................................................................guaggaacuucauaccgugcucu....................  136  0   HS4
....................................................................guaggaacuucauaccgugcucuu...................  1    0   HS4
....................................................................Uuaggaacuucauaccgugcucuu...................  3    1   HS4
.....................................................................uaggaacuucauaccgug........................  127  0   HS4
.....................................................................uagCaacuucauaccgug........................  1    1   HS4
.....................................................................Caggaacuucauaccgug........................  1    1   HS4
.....................................................................uaggaacuucauaccgugG.......................  1    1   HS4
.....................................................................uaggaacuucauaccgugc.......................  170  0   HS4
.....................................................................uaggaacuucauaccgugU.......................  5    1   HS4
.....................................................................uaggaacuucauaccgugA.......................  26   1   HS4
.....................................................................uaggaacuuAauaccgugc.......................  1    1   HS4
.....................................................................uagCaacuucauaccgugc.......................  2    1   HS4
.....................................................................uaggaacuucauaccgugcA......................  1    1   HS4
.....................................................................uaggaacuucauacAgugcu......................  2    1   HS4
.....................................................................uaggaacuCcauaccgugcu......................  3    1   HS4
.....................................................................uaggaacuucauaGcgugcu......................  2    1   HS4
.....................................................................uaggaacuucauaUcgugcu......................  1    1   HS4
.....................................................................uaggUacuucauaccgugcu......................  4    1   HS4
.....................................................................uaggaacuucGuaccgugcu......................  1    1   HS4
.....................................................................uagCaacuucauaccgugcu......................  7    1   HS4
.....................................................................uaggaacuucauaccgugcC......................  2    1   HS4
.....................................................................uaggaaGuucauaccgugcu......................  6    1   HS4
.....................................................................uaggaacuuUauaccgugcu......................  3    1   HS4
.....................................................................uaggaaUuucauaccgugcu......................  1    1   HS4
.....................................................................uaggaacuucauaccUugcu......................  2    1   HS4
.....................................................................uaggaacuucauaccgugUu......................  9    1   HS4
.....................................................................uaggaacuucCuaccgugcu......................  1    1   HS4
.....................................................................uaggaacuucauaccCugcu......................  2    1   HS4
.....................................................................uagUaacuucauaccgugcu......................  9    1   HS4
.....................................................................uaggaaAuucauaccgugcu......................  2    1   HS4
```

Star **Mature**

caaaaaaaaucgaauuacacuucgucacgagcgagguuuguaguuccuaccguuuuuauucaaacugggauaggaacuucauaccgugcucuugaacgaugcugacgaucaa			
....uaggaacuucauacUgugcu......................	1	1	HS4
....uaggaacuucauaccgGgcu......................	1	1	HS4
....uaggaacuuGauaccgugcu......................	1	1	HS4
....uGggaacuucauaccgugcu......................	7	1	HS4
....uaggaacuucauaAcgugcu......................	1	1	HS4
....Aaggaacuucauaccgugcu......................	1	1	HS4
....uaggaacuucauaccgugcu......................	5422	0	HS4
....uagAaacuucauaccgugcu......................	2	1	HS4
....uaggaacuucauaccAugcu......................	2	1	HS4
....uaggaacuucauaccguAcu......................	2	1	HS4
....uaggaacuucauaccguCcu......................	54	1	HS4
....uaUgaacuucauaccgugcu......................	1	1	HS4
....uaggaacuucauaccgAgcu......................	2	1	HS4
....uaCgaacuucauaccgugcu......................	4	1	HS4
....uaggaacuucaCaccgugcu......................	1	1	HS4
....uaggaacuucauaccguUcu......................	33	1	HS4
....Gaggaacuucauaccgugcuc.....................	4	1	HS4
....Aaggaacuucauaccgugcuc.....................	2	1	HS4
....uaggaacuuGauaccgugcuc.....................	1	1	HS4
....uaggaacuucauaccUugcuc.....................	6	1	HS4
....uaggaacuucaGaccgugcuc.....................	1	1	HS4
....uaggaacuucGuaccgugcuc.....................	4	1	HS4
....uaggaacuucaAaccgugcuc.....................	1	1	HS4
....uaggaCcuucauaccgugcuc.....................	1	1	HS4
....uagAaacuucauaccgugcuc.....................	9	1	HS4
....uagCaacuucauaccgugcuc.....................	46	1	HS4
....uaUgaacuucauaccgugcuc.....................	20	1	HS4
....uaggaacuucauaccgugcuA.....................	88	1	HS4
....uaggaacuucauaccCugcuc.....................	13	1	HS4
....uaggaacuucauaccgugcuc.....................	27942	0	HS4
....uaggaGcuucauaccgugcuc.....................	2	1	HS4
....uaggaacuucauaAcgugcuc.....................	4	1	HS4
....uaggaacuucauacAgugcuc.....................	2	1	HS4
....uGggaacuucauaccgugcuc.....................	13	1	HS4
....uaggaacCucauaccgugcuc.....................	4	1	HS4
....uaggaUcuucauaccgugcuc.....................	1	1	HS4
....uaggaacuucaCaccgugcuc.....................	5	1	HS4
....uaggGacuucauaccgugcuc.....................	11	1	HS4
....uaggUacuucauaccgugcuc.....................	14	1	HS4
....uaggaacuCcauaccgugcuc.....................	5	1	HS4
....uUggaacuucauaccgugcuc.....................	3	1	HS4
....uaggaacuucauaccgugAuc.....................	4	1	HS4
....uagUaacuucauaccgugcuc.....................	34	1	HS4
....uaggaaGuucauaccgugcuc.....................	22	1	HS4
....Caggaacuucauaccgugcuc.....................	4	1	HS4
....uaCgaacuucauaccgugcuc.....................	39	1	HS4
....uaggaacuucauaccgugcuG.....................	1	1	HS4
....uaggaacuucauaccgCgcuc.....................	5	1	HS4
....uaggaacuuAauaccgugcuc.....................	10	1	HS4
....uaggaaAuucauaccgugcuc.....................	8	1	HS4
....uaggaacuucauaGcgugcuc.....................	11	1	HS4
....uaggaacuucauacUgugcuc.....................	8	1	HS4
....uaggaacuucauaccgugcAc.....................	1	1	HS4
....uaggaacuucauaccgAgcuc.....................	1	1	HS4
....uaggaacuucauaccguAcuc.....................	64	1	HS4
....uaggaacuucauaccguCcuc.....................	5	1	HS4
....uaggaacuucauaccgugcCc.....................	8	1	HS4
....uaggaacuuUauaccgugcuc.....................	22	1	HS4
....uaggaacuucauaccgGgcuc.....................	2	1	HS4
....uaggaacuucauaccgugcuU.....................	376	1	HS4
....uaggaacuucauaUcgugcuc.....................	26	1	HS4
....uaggCacuucauaccgugcuc.....................	5	1	HS4
....uaggaacuucauaccgugcGc.....................	1	1	HS4
....uaggaacuucauaccguUcuc.....................	4	1	HS4
....uaggaacuucUuaccgugcuc.....................	3	1	HS4
....uaggaacuucCuaccgugcuc.....................	2	1	HS4
....uaggaacuucauaccAugcuc.....................	9	1	HS4
....uaggaacAucauaccgugcuc.....................	3	1	HS4
....uaggaacuucauaccgugUuc.....................	17	1	HS4
....uaggaaUuucauaccgugcuc.....................	8	1	HS4

Star **Mature**

caaaaaaaaucgaauuacacuucgucacgagcgagguuuguaguuccuaccguuuuuauucaaacuggguaggaacuucauaccgugcucuugaacgaugcugacgaucaa			
....uCggaacuucauaccgugcucu....................	9	1	HS4
....uaggaaGuucauaccgugcucu....................	1738	1	HS4
....uaUgaacuucauaccgugcucu....................	796	1	HS4
....uaggaacuucauaccgugcuAu....................	94	1	HS4
....uaggaacuGcauaccgugcucu....................	12	1	HS4
....uaggaacuucauaccgAgcucu....................	210	1	HS4
....Gaggaacuucauaccgugcucu....................	142	1	HS4
....uaggaacuucauaccgugcuUu....................	322	1	HS4
....uaggaacuuAauaccgugcucu....................	338	1	HS4
....uaggaacuuUauaccgugcucu....................	627	1	HS4
....uaggUacuucauaccgugcucu....................	724	1	HS4
....uaggaacuAcauaccgugcucu....................	78	1	HS4
....uaggaCcuucauaccgugcucu....................	15	1	HS4
....uaggaacuuGauaccgugcucu....................	121	1	HS4
....uaggaacuucauaccguAcucu....................	586	1	HS4
....uaCgaacuucauaccgugcucu....................	1731	1	HS4
....uaggaacuucauacGgugcucu....................	26	1	HS4
....uaggaaAuucauaccgugcucu....................	399	1	HS4
....uaggaacuucGuaccgugcucu....................	139	1	HS4
....uaggaacuucaCaccgugcucu....................	178	1	HS4
....uaggaacuucaGaccgugcucu....................	33	1	HS4
....uaggGacuucauaccgugcucu....................	533	1	HS4
....uaggaacAucauaccgugcucu....................	95	1	HS4
....uaggaacuucauaccgugcucA....................	573	1	HS4
....Aaggaacuucauaccgugcucu....................	164	1	HS4
....uaAgaacuucauaccgugcucu....................	126	1	HS4
....Caggaacuucauaccgugcucu....................	132	1	HS4
....uaggaacGucauaccgugcucu....................	15	1	HS4
....uaggaacuucauaccAugcucu....................	642	1	HS4
....uaggaacuucauaccUugcucu....................	323	1	HS4
....uaggaacuucauaccgugcuGu....................	62	1	HS4
....uaggaacuucauaccguUcucu....................	334	1	HS4
....uagAaacuucauaccgugcucu....................	600	1	HS4
....uGggaacuucauaccgugcucu....................	836	1	HS4
....uaggaacuucauGccgugcucu....................	60	1	HS4
....uaggCacuucauaccgugcucu....................	261	1	HS4
....uaggaacuucauaccgGgcucu....................	211	1	HS4
....uaggaacuucauaccgugcucG....................	51	1	HS4
....uaggaacuucauaccgugcGcu....................	51	1	HS4
....uaggaacuucauUccgugcucu....................	47	1	HS4
....uaggaacuucCuaccgugcucu....................	134	1	HS4
....uaggaacuucauaGcgugcucu....................	375	1	HS4
....uaggaacuucauaccgugGucu....................	42	1	HS4
....uaggaacuucauaccguCcucu....................	410	1	HS4
....uaggaacuucauaAcgugcucu....................	159	1	HS4
....uaggaacuucauaccgugcucu....................	1795315	0	HS4
....uaggaacuucauaccgugcucC....................	2288	1	HS4
....uUggaacuucauaccgugcucu....................	57	1	HS4
....uaggaGcuucauaccgugcucu....................	81	1	HS4
....uaggaacuucaAaccgugcucu....................	45	1	HS4
....uaggaacuucauacAgugcucu....................	117	1	HS4
....uagCaacuucauaccgugcucu....................	1766	1	HS4
....uagUaacuucauaccgugcucu....................	2318	1	HS4
....uaggaacuucauaUcgugcucu....................	631	1	HS4
....uaggaacuucauacUgugcucu....................	447	1	HS4
....uaggaacuCcauaccgugcucu....................	415	1	HS4
....uaggaacuucauaccgugUucu....................	958	1	HS4
....uaggaacuucauCccgugcucu....................	7	1	HS4
....uaggaaUuucauaccgugcucu....................	463	1	HS4
....uaggaacuucUuaccgugcucu....................	407	1	HS4
....uaggaUcuucauaccgugcucu....................	26	1	HS4
....uaggaacuucauaccCugcucu....................	257	1	HS4
....uaggaacCucauaccgugcucu....................	102	1	HS4
....uaggaacuucauaccgugAucu....................	54	1	HS4
....uaggaacuucauaccgugcCcu....................	126	1	HS4
....uaggaacuucauaccgugcAcu....................	59	1	HS4
....uaggaacuucauaccgCgcucu....................	202	1	HS4
....uaggaacuucauaccgugcuUuu...................	3	1	HS4
....uaggaacuucauaccgAgcucuu...................	2	1	HS4
....uaggaacuucauaccguCcucuu...................	1	1	HS4

Star **Mature**

```
caaaaaaaaucgaauuacacuucgucacgagcgagguuuguaguuccuaccguuuuuauucaaacuggguaggaacuucauaccgugcucuugaacgaugcugacgaucaa
.....................................................................uaggaacuucauaccgugcCcuu...................   2       1    HS4
.....................................................................uGggaacuucauaccgugcucuu...................   1       1    HS4
.....................................................................uaggaacuAcauaccgugcucuu...................   1       1    HS4
.....................................................................uaggaacuucauaccguAcucuu...................   5       1    HS4
.....................................................................uaggaacuucauaccgugcucAu...................   6       1    HS4
.....................................................................uaggUacuucauaccgugcucuu...................   5       1    HS4
.....................................................................uaggaacuuGauaccgugcucuu...................   2       1    HS4
.....................................................................uagCaacuucauaccgugcucuu...................   7       1    HS4
.....................................................................uaggaacuucauaccgugAucuu...................   1       1    HS4
.....................................................................uaggaacuucauaccgugcucuu...................   10150   0    HS4
.....................................................................uaggaacuucGuaccgugcucuu...................   2       1    HS4
.....................................................................uaggaacuucauaccAugcucuu...................   8       1    HS4
.....................................................................uaggaacuucaCaccgugcucuu...................   1       1    HS4
.....................................................................uUggaacuucauaccgugcucuu...................   2       1    HS4
.....................................................................uaggaacuuAauaccgugcucuu...................   2       1    HS4
.....................................................................uaggaacuucauaccgGgcucuu...................   1       1    HS4
.....................................................................uagAaacuucauaccgugcucuu...................   5       1    HS4
.....................................................................uaggaaUuucauaccgugcucuu...................   4       1    HS4
.....................................................................uaggaacuucauaccgugUucuu...................   1       1    HS4
.....................................................................uaggaacuucauacUgugcucuu...................   3       1    HS4
.....................................................................Caggaacuucauaccgugcucuu...................   2       1    HS4
.....................................................................uaggaacuucauaccgugcucCu...................   33      1    HS4
.....................................................................uaggaaGuucauaccgugcucuu...................   11      1    HS4
.....................................................................uaggaacuucauaccguUcucuu...................   1       1    HS4
.....................................................................uaUgaacuucauaccgugcucuu...................   4       1    HS4
.....................................................................uaggaacuCcauaccgugcucuu...................   2       1    HS4
.....................................................................uaggaacuuUauaccgugcucuu...................   5       1    HS4
.....................................................................uaggaacuucauaccgugcucuC...................   24      1    HS4
.....................................................................uaggaacuucauaccgugcucuA...................   1312    1    HS4
.....................................................................uaggGacuucauaccgugcucuu...................   3       1    HS4
.....................................................................Aaggaacuucauaccgugcucuu...................   2       1    HS4
.....................................................................uagUaacuucauaccgugcucuu...................   17      1    HS4
.....................................................................uaggaacuucauaccgugcucuG...................   20      1    HS4
.....................................................................uaggaacuucauaAcgugcucuu...................   1       1    HS4
.....................................................................uaAgaacuucauaccgugcucuu...................   1       1    HS4
.....................................................................uaggaacuucauacAgugcucuu...................   1       1    HS4
.....................................................................uaggaacuucauaUcgugcucuu...................   1       1    HS4
.....................................................................uaggaacAucauaccgugcucuu...................   1       1    HS4
.....................................................................uaggCacuucauaccgugcucuu...................   2       1    HS4
.....................................................................uaggaacuucauaccgCgcucuu...................   2       1    HS4
.....................................................................uaggaacuucauaccUugcucuu...................   1       1    HS4
.....................................................................uaggaCcuucauaccgugcucuu...................   1       1    HS4
.....................................................................uaggaacCucauaccgugcucuu...................   1       1    HS4
.....................................................................uaggaacuucauaGcgugcucuu...................   2       1    HS4
.....................................................................uaggaaAuucauaccgugcucuu...................   4       1    HS4
.....................................................................uaggaacuucauaccgugGucuu...................   1       1    HS4
.....................................................................uaCgaacuucauaccgugcucuu...................   3       1    HS4
.....................................................................uaggaacuucUuaccgugcucuu...................   1       1    HS4
.....................................................................uaggaacuucauaccgugcucuGg..................   4       1    HS4
.....................................................................uaggaacuucauaccgugcucuuA..................   23      1    HS4
.....................................................................uaggaacuucauaccgugcucuuU..................   128     1    HS4
.....................................................................uaggaacuucauaccgugcucuug..................   7       0    HS4
.....................................................................uaggaacuucauaccgugcucuAg..................   2       1    HS4
.....................................................................uaggaacuucauaccgugcucuCg..................   3       1    HS4
.....................................................................uaggaacuucauaccgugcucuuC..................   6       1    HS4
.....................................................................uaggaacuucauaccgugcucuuUa.................   2       1    HS4
......................................................................aggaacuucauaccgugc.......................   1       0    HS4
......................................................................aggaacuucauaccguUcu......................   2       1    HS4
......................................................................aggaacuucauaccgugcu......................   8       0    HS4
......................................................................aUgaacuucauaccgugcu......................   1       1    HS4
......................................................................aCgaacuucauaccgugcuc.....................   8       1    HS4
......................................................................aUgaacuucauaccgugcuc.....................   3       1    HS4
......................................................................aggaacuucauaccgugcuc.....................   69      0    HS4
......................................................................aggaacuucauaccgugcuU.....................   1       1    HS4
......................................................................aUgaacuucauaccgugcucu....................   131     1    HS4
......................................................................aAgaacuucauaccgugcucu....................   5       1    HS4
......................................................................aggCacuucauaccgugcucu....................   1       1    HS4
......................................................................aggaacuucauaUcgugcucu....................   2       1    HS4
......................................................................aggaacuAcauaccgugcucu....................   1       1    HS4
......................................................................aggaacuucauaccCugcucu....................   1       1    HS4
```

Star Mature

```
caaaaaaaaucgaauuacacuucgucacgagcgagguuuguaguuccuaccguuuuuauucaaacugggu aggaacuucauaccgugcucuugaacgaugcugacgaucaa
......................................................................aggaacAucauaccgugcucu....................   2     1   HS4
......................................................................aggaaGuucauaccgugcucu....................   2     1   HS4
......................................................................aggaacuucauaccgAgcucu....................   3     1   HS4
......................................................................Cggaacuucauaccgugcucu....................   3     1   HS4
......................................................................aggaacuuGauaccgugcucu....................   1     1   HS4
......................................................................aggaacuucauaccgCgcucu....................   2     1   HS4
......................................................................aggaacuucauaccAugcucu....................   2     1   HS4
......................................................................Gggaacuucauaccgugcucu....................   10    1   HS4
......................................................................Uggaacuucauaccgugcucu....................   10    1   HS4
......................................................................aggaacuucauaccgugcuUu....................   1     1   HS4
......................................................................aggaacuucauacGgugcucu....................   1     1   HS4
......................................................................aggaacuucauaccgugUucu....................   2     1   HS4
......................................................................agUaacuucauaccgugcucu....................   4     1   HS4
......................................................................aggaacCucauaccgugcucu....................   2     1   HS4
......................................................................aCgaacuucauaccgugcucu....................   143   1   HS4
......................................................................aggaacuucauaccgugcucu....................   3718  0   HS4
......................................................................aggUacuucauaccgugcucu....................   8     1   HS4
......................................................................aggaacuucauaccgugcCcu....................   1     1   HS4
......................................................................aggaaUuucauaccgugcucu....................   1     1   HS4
......................................................................aggGacuucauaccgugcucu....................   1     1   HS4
......................................................................aggaaAuucauaccgugcucu....................   1     1   HS4
......................................................................aggaUcuucauaccgugcucu....................   2     1   HS4
......................................................................aggaacGucauaccgugcucu....................   2     1   HS4
......................................................................aggaacuucauaccgugcucC....................   16    1   HS4
......................................................................agCaacuucauaccgugcucu....................   3     1   HS4
......................................................................aggaacuucUuaccgugcucu....................   2     1   HS4
......................................................................aggaacuucaCaccgugcucu....................   1     1   HS4
......................................................................aggaGcuucauaccgugcucu....................   1     1   HS4
......................................................................aggaacuucauaccgugcucuA...................   3     1   HS4
......................................................................aggaacuucauaccgugcucuu...................   12    0   HS4
.......................................................................ggaacuucauaccgugcu......................   1     0   HS4
.......................................................................Ugaacuucauaccgugcu......................   1     1   HS4
.......................................................................Ugaacuucauaccgugcuc.....................   12    1   HS4
.......................................................................ggaacuucauaccgugcuc.....................   1     0   HS4
.......................................................................ggaaUuucauaccgugcucu....................   1     1   HS4
.......................................................................ggaacuucauaccguUcucu....................   1     1   HS4
.......................................................................Agaacuucauaccgugcucu....................   30    1   HS4
.......................................................................ggaacuucauUccgugcucu....................   1     1   HS4
.......................................................................Cgaacuucauaccgugcucu....................   3     1   HS4
.......................................................................gCaacuucauaccgugcucu....................   1     1   HS4
.......................................................................gUaacuucauaccgugcucu....................   7     1   HS4
.......................................................................ggCacuucauaccgugcucu....................   1     1   HS4
.......................................................................ggaacuucauaccgugcucu....................   355   0   HS4
.......................................................................ggaacuucauaccgugcucC....................   1     1   HS4
.......................................................................ggaacuucauaccgAgcucu....................   1     1   HS4
.......................................................................Ugaacuucauaccgugcucu....................   491   1   HS4
.......................................................................ggaUcuucauaccgugcucu....................   1     1   HS4
.......................................................................Ugaacuucauaccgugcucuu...................   1     1   HS4
.......................................................................ggaacuucauaccgugcucuA...................   1     1   HS4
.......................................................................ggaacuucauaccgugcucuu...................   4     0   HS4
.......................................................................ggaacuucauaccgugcucuug..................   1     0   HS4
........................................................................gaacuucauaccgugcuc.....................   4     0   HS4
........................................................................Uaacuucauaccgugcuc.....................   1     1   HS4
........................................................................gaacuucauaccgugcucu....................   186   0   HS4
........................................................................gaacuuGauaccgugcucu....................   1     1   HS4
........................................................................Aaacuucauaccgugcucu....................   1     1   HS4
........................................................................Caacuucauaccgugcucu....................   3     1   HS4
........................................................................gaacuucauGccgugcucu....................   1     1   HS4
........................................................................Uaacuucauaccgugcucu....................   136   1   HS4
........................................................................gaacuucauaccgugcucuu...................   1     0   HS4
.........................................................................aacuucauaccgugcucu....................   830   0   HS4
.........................................................................aGcuucauaccgugcucu....................   1     1   HS4
.........................................................................aacuucauaccguCcucu....................   1     1   HS4
.........................................................................Gacuucauaccgugcucu....................   1     1   HS4
.........................................................................aacuucauaccgugcucC....................   2     1   HS4
.........................................................................aacuuUauaccgugcucu....................   1     1   HS4
.........................................................................aacuucauaccgugUucu....................   2     1   HS4
.........................................................................aacuucGuaccgugcucu....................   2     1   HS4
.........................................................................aacuucauaccguUcucu....................   2     1   HS4
.........................................................................aaGuucauaccgugcucu....................   1     1   HS4
```

```
                              Star                                      Mature
caaaaaaaaucgaauuacacuucgucacgagcgagguuuguaguuccuaccguuuuuauucaaacuggguaggaacuucauaccgugcucuugaacgaugcugacgaucaa
.........................................................................aacuucauaccguAcucu....................   1      1      HS4
.........................................................................aaUuucauaccgugcucu....................   2      1      HS4
.........................................................................aacuuAauaccgugcucu....................   1      1      HS4
.........................................................................aacuucauaccgugcucuG...................   1      1      HS4
.........................................................................aacuucauaccgugcucuu...................   3      0      HS4
.........................................................................aacuucauaccgugcucuuU..................   1      1      HS4
..........................................................................acuucauaccgugcucuu...................   3      0      HS4

.........ucgaauuacacuucgucacg..................................................................................   2      0      J17
.........ucAaauuacacuucgucacg..................................................................................   1      1      J17
..........................acgagcgagguuuguaguuccu...............................................................   2      0      J17
............................gagcgagguuuguaguuccu...............................................................   1      0      J17
............................Uagcgagguuuguaguuccua..............................................................   2      1      J17
............................Uagcgagguuuguaguuccuac.............................................................   3      1      J17
............................Uagcgagguuuguaguuccuacc............................................................   5      1      J17
.............................agcgagguuuguaguucc................................................................   12     0      J17
.............................agcgagguuuguaguuccu...............................................................   452    0      J17
.............................agcgagguAuguaguuccu...............................................................   1      1      J17
.............................agcgagguuuguaguuccA...............................................................   1      1      J17
.............................agcgagguuuguaguuAcu...............................................................   1      1      J17
.............................agcgUgguuuguaguuccu...............................................................   1      1      J17
.............................agcgaggGuuguaguuccua..............................................................   4      1      J17
.............................agcgaggAuuguaguuccua..............................................................   1      1      J17
.............................agcgagguuuguUguuccua..............................................................   1      1      J17
.............................agcgagguAuguaguuccua..............................................................   1      1      J17
.............................agcgagguuuguaguuccuU..............................................................   11     1      J17
.............................agcgagguuCguaguuccua..............................................................   1      1      J17
.............................agcgagguuuguaguAccua..............................................................   1      1      J17
.............................agcgagguuuguaguuccua..............................................................   1618   0      J17
.............................agcgagguuuguaguucGua..............................................................   2      1      J17
.............................agcgagguuugAaguuccua..............................................................   1      1      J17
.............................agcgagguuuguaguuccCa..............................................................   1      1      J17
.............................agUgagguuuguaguuccua..............................................................   2      1      J17
.............................agcgagguuuguaguucGuac.............................................................   1      1      J17
.............................agcAagguuuguaguuccuac.............................................................   1      1      J17
.............................agcgagguuuguaguucUuac.............................................................   4      1      J17
.............................aAcgagguuuguaguuccuac.............................................................   1      1      J17
.............................agcgagguAuguaguuccuac.............................................................   3      1      J17
.............................aCcgagguuuguaguuccuac.............................................................   2      1      J17
.............................agcgaggAuuguaguuccuac.............................................................   2      1      J17
.............................agcgagguuuguagAuccuac.............................................................   2      1      J17
.............................agcgagguuuCuaguuccuac.............................................................   1      1      J17
.............................agcgagguuugCaguuccuac.............................................................   1      1      J17
.............................agcgagguuuguaguuUcuac.............................................................   1      1      J17
.............................Cgcgagguuuguaguuccuac.............................................................   1      1      J17
.............................agcUagguuuguaguuccuac.............................................................   1      1      J17
.............................agcgUgguuuguaguuccuac.............................................................   2      1      J17
.............................agcgagguuuguaguucAuac.............................................................   2      1      J17
.............................aUcgagguuuguaguuccuac.............................................................   1      1      J17
.............................agcgagguuuguaguuccuaU.............................................................   44     1      J17
.............................agcgagguuuAuaguuccuac.............................................................   1      1      J17
.............................agcgaggCuuguaguuccuac.............................................................   1      1      J17
.............................agcgagguuCguaguuccuac.............................................................   1      1      J17
.............................agcgagguuuguaguuccuac.............................................................   4438   0      J17
.............................agcgagCuuuguaguuccuac.............................................................   1      1      J17
.............................agcgagguuuguaguAccuac.............................................................   1      1      J17
.............................agcgagguuuguaguCccuac.............................................................   1      1      J17
.............................agcgagguuuguaguuccuaA.............................................................   4      1      J17
.............................agUgagguuuguaguuccuac.............................................................   3      1      J17
.............................agcgaggGuuguaguuccuac.............................................................   9      1      J17
.............................agcgagguuuguGguuccuac.............................................................   1      1      J17
.............................agcgagguuuguaguuccAac.............................................................   1      1      J17
.............................agcgagguuuguaguuccuGc.............................................................   4      1      J17
.............................agcgagguuuguaguuccuaGc............................................................   1      1      J17
.............................agcgagUuuuguaguuccuacc............................................................   2      1      J17
.............................agcgagguuuguaguucUuacc............................................................   1      1      J17
.............................agcgagguuuguaguuccCacc............................................................   3      1      J17
.............................agcgagguuuUuaguuccuacc............................................................   1      1      J17
.............................agcgagguuuguaguuUcuacc............................................................   11     1      J17
.............................agcgagguuuguaAuuccuacc............................................................   1      1      J17
```

Star **Mature**

caaaaaaaaucgaauuacacuucgucacgagcgagguuuguaguuccuaccguuuuuauucaaacuggguaggaacuucauaccgugcucuugaacgaugcugacgaucaa			
.............................agcgaggCuuguaguuccuacc..	3	1	J17
.............................aAcgagguuuguaguuccuacc..	2	1	J17
.............................agcgagguuuguaguAccuacc..	2	1	J17
.............................agcgagCuuuguaguuccuacc..	2	1	J17
.............................agcgagguuuguaguuccuUcc..	2	1	J17
.............................agcgaggGuuguaguuccuacc..	26	1	J17
.............................agcgagguuuguaguuccuaUc..	4	1	J17
.............................agUgagguuuguaguuccuacc..	23	1	J17
.............................agcgaggAuuguaguuccuacc..	7	1	J17
.............................agcgagguuuguagGuccuacc..	1	1	J17
.............................agcgagguuuguaguuccuacA..	290	1	J17
.............................agcgagguAuguaguuccuacc..	2	1	J17
.............................agcgagguuuguaguuccuacG..	4	1	J17
.............................agcgaCguuuguaguuccuacc..	4	1	J17
.............................agcgCgguuuguaguuccuacc..	3	1	J17
.............................agcgagguuuguaguuccuacU..	329	1	J17
.............................agcgagguuuguaUuuccuacc..	1	1	J17
.............................agcgagguuuguaguucAuacc..	1	1	J17
.............................agcUagguuuguaguuccuacc..	2	1	J17
.............................agcgagguuuguaguucGuacc..	1	1	J17
.............................agcgagguuuguaguuccAacc..	1	1	J17
.............................agcgaAguuuguaguuccuacc..	1	1	J17
.............................agcCagguuuguaguuccuacc..	3	1	J17
.............................agcgagguuugGaguuccuacc..	1	1	J17
.............................agcgaUguuuguaguuccuacc..	1	1	J17
.............................agcgagguuuguaguuccuaAc..	1	1	J17
.............................agcgagguuuguagAuccuacc..	3	1	J17
.............................agcgagguuCguaguuccuacc..	2	1	J17
.............................agcgUgguuuguaguuccuacc..	2	1	J17
.............................agcgagguuuguUguuccuacc..	2	1	J17
.............................agcgagguuuguGguuccuacc..	1	1	J17
.............................aCcgagguuuguaguuccuacc..	2	1	J17
.............................aUcgagguuuguaguuccuacc..	4	1	J17
.............................agcgagguuuguaguuccuCcc..	1	1	J17
.............................agcgagguuuguaguuccuacc..	10224	0	J17
.............................agcgagguuAguaguuccuacc..	1	1	J17
.............................agcgagguuuguaguuccuGcc..	2	1	J17
.............................agcgagguuuguaguuccuaccU...	378	1	J17
.............................agcgagguuugAaguuccuaccg...	1	1	J17
.............................agcgagguuuguaguuccuaccA...	210	1	J17
.............................agcgaggGuuguaguuccuaccg...	1	1	J17
.............................agcgagguuuguaguuUcuaccg...	2	1	J17
.............................agcgagguuuguaguuccuaccg...	386	0	J17
.............................agcgagguuuguaguAccuaccg...	1	1	J17
.............................Ugcgagguuuguaguuccuaccg...	1	1	J17
.............................agcgagguuuguaguuccuaccC...	8	1	J17
.............................agcgagguuuguGguuccuaccg...	1	1	J17
.............................agcgagguuuguaguuccuaccgA..	3	1	J17
.............................agcgagguuuguaguuccuaccUu..	3	1	J17
.............................agcgagguuuguaguuccuaccgu..	7	0	J17
.............................agcgagguuuguaguuccuaccguu...	5	0	J17
.............................agcgagguuuguaguuccuaccguuu..	1	0	J17
..............................Acgagguuuguaguuccu...	1	1	J17
..............................gcgagguuuguaguuccuac...	9	0	J17
..............................gcgagguuuguaguuccuacc..	60	0	J17
..............................gGgagguuuguaguuccuacc..	1	1	J17
..............................gcgagguuuguaguuccuaccg...	94	0	J17
..............................gcgagguuuguGguuccuaccg...	1	1	J17
..............................gcgagguuuguaguuccuaccA...	2	1	J17
..............................gcgagguuuguaguuccuaccgA..	1	1	J17
...............................cgagguuuguaguuccuac...	4	0	J17
...............................cgagguuuguaguuccuacc..	5	0	J17
...............................cgagguuuguaguuccuaccU...	1	1	J17
...............................cgagguuuguaguuccuaccg...	48	0	J17
...............................cgGgguuuguaguuccuaccg...	1	1	J17
...............................cgagguuuguaguuccuaccC...	12	1	J17
...............................cgagguuuguaguuccuaccA...	3	1	J17
...............................cgagguuuguaguuccuaccCu..	1	1	J17
................................gagguuuguaguuccuacc..	2	0	J17
................................gagguuuguaguuccuaccgu..	2	0	J17

Star **Mature**

```
caaaaaaaaucgaauuacacuucgucacgagcgagguuuguaguuccuaccguuuuuauucaaacuggguaggaacuucauaccgugcucuugaacgaugcugacgaucaa
.................................agguuuguaguuccuacc............................................................ 1 0 J17
.................................agguuuguaguuccuaccA........................................................... 1 1 J17
................................................................cGggguaggaacuucauaccgugcucu.................... 1 1 J17
.................................................................uAgguaggaacuucauaccgugcuc..................... 1 1 J17
.................................................................uggAuaggaacuucauaccgugcucu.................... 3 1 J17
.................................................................uggguaggaacuucauaccgugcucu.................... 1 0 J17
..................................................................Ugguaggaacuucauaccgugcuc..................... 1 1 J17
..................................................................Agguaggaacuucauaccgugcuc..................... 1 1 J17
..................................................................gAguaggaacuucauaccgugcucu.................... 2 1 J17
..................................................................ggAuaggaacuucauaccgugcucu.................... 4 1 J17
..................................................................ggCuaggaacuucauaccgugcucu.................... 13 1 J17
..................................................................ggUuaggaacuucauaccgugcucu.................... 2 1 J17
..................................................................Ugguaggaacuucauaccgugcucu.................... 1 1 J17
..................................................................Agguaggaacuucauaccgugcucu.................... 1 1 J17
..................................................................ggguaggaacuucauaccgugcucu.................... 10 0 J17
...................................................................gAuaggaacuucauaccgugcucu.................... 5 1 J17
...................................................................gguaggaacuucauaccgugcucu.................... 4 0 J17
...................................................................gCuaggaacuucauaccgugcucu.................... 9 1 J17
...................................................................Aguaggaacuucauaccgugcucu.................... 1 1 J17
....................................................................guaggaacuucauaccgugc....................... 3 0 J17
....................................................................Uuaggaacuucauaccgugcu...................... 2 1 J17
....................................................................guaggaacuucauaccgugcu...................... 44 0 J17
....................................................................guaggGacuucauaccgugcuc..................... 1 1 J17
....................................................................Auaggaacuucauaccgugcuc..................... 2 1 J17
....................................................................guaggaacuucauaccgugcuc..................... 93 0 J17
....................................................................guaggaacuucauaccgGgcuc..................... 1 1 J17
....................................................................Uuaggaacuucauaccgugcuc..................... 22 1 J17
....................................................................guaggaacuucauaccgugcuU..................... 2 1 J17
....................................................................Cuaggaacuucauaccgugcuc..................... 1 1 J17
....................................................................guaggaacCucauaccgugcuc..................... 1 1 J17
....................................................................Uuaggaacuucauaccgugcucu.................... 835 1 J17
....................................................................Cuaggaacuucauaccgugcucu.................... 16 1 J17
....................................................................guaggaacuucauaccgugcucC................... 1 1 J17
....................................................................Auaggaacuucauaccgugcucu.................... 34 1 J17
....................................................................guaggaacuucauaccgugcucu.................... 132 0 J17
.....................................................................uaggaacuucauaccgug........................ 77 0 J17
.....................................................................uaggaacuucauaccgugU....................... 9 1 J17
.....................................................................uaggaacuAcauaccgugc....................... 1 1 J17
.....................................................................uaggaacuucauaUcgugc....................... 1 1 J17
.....................................................................uaggaacuucauaccgugA....................... 24 1 J17
.....................................................................uaggaacuucauaccgugc....................... 333 0 J17
.....................................................................uaggaacuucauaccguAcu...................... 8 1 J17
.....................................................................uaggaacuucauaUcgugcu...................... 2 1 J17
.....................................................................Caggaacuucauaccgugcu...................... 3 1 J17
.....................................................................uaggUacuucauaccgugcu...................... 1 1 J17
.....................................................................uaggaacuucauaccUugcu...................... 2 1 J17
.....................................................................uaggaaUuucauaccgugcu...................... 2 1 J17
.....................................................................uaAgaacuucauaccgugcu...................... 2 1 J17
.....................................................................uagCaacuucauaccgugcu...................... 1 1 J17
.....................................................................uaggaacuAcauaccgugcu...................... 4 1 J17
.....................................................................uaggaacuucauaccgAgcu...................... 7 1 J17
.....................................................................uaggaacuCcauaccgugcu...................... 7 1 J17
.....................................................................uaggaCcuucauaccgugcu...................... 1 1 J17
.....................................................................uaggaacuuAauaccgugcu...................... 1 1 J17
.....................................................................uaggaacCucauaccgugcu...................... 2 1 J17
.....................................................................uaggaacuucauaccgugcA...................... 13 1 J17
.....................................................................uagAaacuucauaccgugcu...................... 3 1 J17
.....................................................................uaggaacuucauUccgugcu...................... 1 1 J17
.....................................................................uGggaacuucauaccgugcu...................... 2 1 J17
.....................................................................uagUaacuucauaccgugcu...................... 2 1 J17
.....................................................................uaggaacuucauaccgugcu...................... 6376 0 J17
.....................................................................uaggaacuucauacUgugcu...................... 3 1 J17
.....................................................................uaggaacuucauaccgCgcu...................... 2 1 J17
.....................................................................uaggaacAucauaccgugcu...................... 2 1 J17
.....................................................................uaggaGcuucauaccgugcu...................... 1 1 J17
.....................................................................uaggaacuucauaccgugUu...................... 4 1 J17
.....................................................................uaggaacuucauaccguCcu...................... 62 1 J17
.....................................................................uaggaacuucauaAcgugcu...................... 2 1 J17
.....................................................................uUggaacuucauaccgugcu...................... 1 1 J17
.....................................................................uaggaacuuUauaccgugcu...................... 3 1 J17
```

Star **Mature**

caaaaaaaaucgaauuacacuucgucacgagcgagguuuguaguuccuaccguuuuuauucaaacuggguaggaacuucauaccgugcucuugaacgaugcugacgaucaa

....uaggaacuucauaccgugcC......................	1	1	J17
....uaggaacuucauaccguUcu......................	5	1	J17
....uaggaaAuucauaccgugcu......................	1	1	J17
....Aaggaacuucauaccgugcu......................	6	1	J17
....uaggaacuucauaccgugcuc.....................	79420	0	J17
....uaCgaacuucauaccgugcuc.....................	11	1	J17
....uaggaacuucauaccgugcuG.....................	2	1	J17
....uaggUacuucauaccgugcuc.....................	48	1	J17
....uaAgaacuucauaccgugcuc.....................	7	1	J17
....uaggaacuucauaccUugcuc.....................	12	1	J17
....uaggaacuucauaccgugcuU.....................	347	1	J17
....uaggaGcuucauaccgugcuc.....................	13	1	J17
....uUggaacuucauaccgugcuc.....................	10	1	J17
....uaggaacuucGuaccgugcuc.....................	2	1	J17
....uaggaacuucauaccgCgcuc.....................	15	1	J17
....Gaggaacuucauaccgugcuc.....................	12	1	J17
....uaggaacuucaAaccgugcuc.....................	9	1	J17
....uaUgaacuucauaccgugcuc.....................	7	1	J17
....uaggaacuGcauaccgugcuc.....................	1	1	J17
....uaggaacuucauaccAugcuc.....................	19	1	J17
....uaggaacuucauaUcgugcuc.....................	24	1	J17
....Aaggaacuucauaccgugcuc.....................	38	1	J17
....uaggaacuucauaccguUcuc.....................	8	1	J17
....uagAaacuucauaccgugcuc.....................	14	1	J17
....uaggaacGucauaccgugcuc.....................	3	1	J17
....uaggaacuucauaccgugcuA.....................	101	1	J17
....uaggaacuucaCaccgugcuc.....................	9	1	J17
....uaggaacuucauaccgugAuc.....................	7	1	J17
....uaggaacuucauaccgugcCc.....................	18	1	J17
....uaggaaGuucauaccgugcuc.....................	1	1	J17
....uaggaacuAcauaccgugcuc.....................	30	1	J17
....uaggaacCucauaccgugcuc.....................	11	1	J17
....uaggaacAucauaccgugcuc.....................	29	1	J17
....uaggaacuucauaccguAcuc.....................	93	1	J17
....uaggaCcuucauaccgugcuc.....................	4	1	J17
....uaggaacuCcauaccgugcuc.....................	17	1	J17
....uaggaacuucUuaccgugcuc.....................	8	1	J17
....uagCaacuucauaccgugcuc.....................	10	1	J17
....uaggaacuucauaccgugcAc.....................	15	1	J17
....uaggaacuucauaccgugGuc.....................	8	1	J17
....uaggaacuucauaGcgugcuc.....................	1	1	J17
....uaggaUcuucauaccgugcuc.....................	13	1	J17
....uaggaacuucauaccgugUuc.....................	48	1	J17
....uCggaacuucauaccgugcuc.....................	5	1	J17
....uaggaaUuucauaccgugcuc.....................	15	1	J17
....uaggaacuucauacAgugcuc.....................	3	1	J17
....uaggaaAuucauaccgugcuc.....................	7	1	J17
....uaggaacuucauGccgugcuc.....................	1	1	J17
....uaggaacuuGauaccgugcuc.....................	1	1	J17
....uaggaacuucauaccgugcGc.....................	7	1	J17
....uaggaacuuAauaccgugcuc.....................	8	1	J17
....uagUaacuucauaccgugcuc.....................	33	1	J17
....uaggaacuuUauaccgugcuc.....................	20	1	J17
....uaggaacuucauaAcgugcuc.....................	8	1	J17
....uaggaacuucauacUgugcuc.....................	12	1	J17
....uaggGacuucauaccgugcuc.....................	13	1	J17
....Caggaacuucauaccgugcuc.....................	7	1	J17
....uaggaacuucCuaccgugcuc.....................	1	1	J17
....uaggaacuucauaccgAgcuc.....................	92	1	J17
....uaggaacuucauaccCugcuc.....................	18	1	J17
....uGggaacuucauaccgugcuc.....................	10	1	J17
....uaggCacuucauaccgugcuc.....................	7	1	J17
....uaggaacuucauUccgugcuc.....................	2	1	J17
....uaggaacuucauaccguCcuc.....................	7	1	J17
....uaggaacuucauaccgGgcuc.....................	43	1	J17
....uaUgaacuucauaccgugcucu....................	220	1	J17
....uaggaacuuUauaccgugcucu....................	485	1	J17
....uaggaacuucauaccguAcucu....................	561	1	J17
....uaggaacuucauaccgugcucu....................	2212598	0	J17
....uaggaacuucauaccgAgcucu....................	2623	1	J17

Star Mature

caaaaaaaaucgaauuacacuucgucacgagcgagguuuguaguuccuaccguuuuuauucaaacuggguaggaacuucauaccgugcucuugaacgaugcugacgaucaa			
....uaggaacuucaAaccgugcucu....................	216	1	J17
....uaggaacuucauaccgugAucu....................	144	1	J17
....uaggaacuucauaccgCgcucu....................	387	1	J17
....uaggaaUuucauaccgugcucu....................	294	1	J17
....uaggaacuucauaccgugcuUu....................	764	1	J17
....uGggaacuucauaccgugcucu....................	362	1	J17
....uaggaacuucauCccgugcucu....................	5	1	J17
....uaggaacuucauaccgugGucu....................	69	1	J17
....uaggaacuucauaccgugcAcu....................	409	1	J17
....uaggaacuucauUccgugcucu....................	107	1	J17
....uaggaacAucauaccgugcucu....................	957	1	J17
....uaggaacuGcauaccgugcucu....................	36	1	J17
....uCggaacuucauaccgugcucu....................	164	1	J17
....uaggaCcuucauaccgugcucu....................	188	1	J17
....uaggaGcuucauaccgugcucu....................	332	1	J17
....uaggaacuucauaGcgugcucu....................	29	1	J17
....uaggaacuucUuaccgugcucu....................	168	1	J17
....uaAgaacuucauaccgugcucu....................	169	1	J17
....uaggGacuucauaccgugcucu....................	505	1	J17
....uaggaacuucauaccUugcucu....................	179	1	J17
....uaggaacuucauaccgugcGcu....................	110	1	J17
....uaggaacCucauaccgugcucu....................	372	1	J17
....uaggaacuucauaccgugcCcu....................	192	1	J17
....uaggaaGuucauaccgugcucu....................	23	1	J17
....uaggaacuucaCaccgugcucu....................	176	1	J17
....Caggaacuucauaccgugcucu....................	196	1	J17
....uaggCacuucauaccgugcucu....................	271	1	J17
....uaggaacGucauaccgugcucu....................	60	1	J17
....uaggaacuucauacGgugcucu....................	41	1	J17
....uaggaacuucauaccguCcucu....................	279	1	J17
....uaggaacuCcauaccgugcucu....................	410	1	J17
....uaCgaacuucauaccgugcucu....................	363	1	J17
....uagCaacuucauaccgugcucu....................	367	1	J17
....uaggaacuucauGccgugcucu....................	32	1	J17
....uaggaacuucauaccgugcuGu....................	68	1	J17
....Gaggaacuucauaccgugcucu....................	198	1	J17
....uaggaacuuAauaccgugcucu....................	139	1	J17
....uaggUacuucauaccgugcucu....................	1035	1	J17
....uaggaacuucauaccgugcucC....................	3521	1	J17
....uaggaacuucauaccAugcucu....................	610	1	J17
....uaggaaAuucauaccgugcucu....................	145	1	J17
....uaggaacuucaGaccgugcucu....................	23	1	J17
....uUggaacuucauaccgugcucu....................	304	1	J17
....Aaggaacuucauaccgugcucu....................	925	1	J17
....uaggaacuAcauaccgugcucu....................	986	1	J17
....uaggaacuucauaccgugcuAu....................	39	1	J17
....uagUaacuucauaccgugcucu....................	701	1	J17
....uaggaacuuGauaccgugcucu....................	48	1	J17
....uaggaacuucauaccguUcucu....................	200	1	J17
....uaggaacuucauaccgGgcucu....................	1085	1	J17
....uaggaacuucauaccgugcucA....................	3231	1	J17
....uaggaacuucauacUgugcucu....................	508	1	J17
....uaggaUcuucauaccgugcucu....................	209	1	J17
....uagAaacuucauaccgugcucu....................	425	1	J17
....uaggaacuucauaUcgugcucu....................	506	1	J17
....uaggaacuucauaccgugcucG....................	219	1	J17
....uaggaacuucauacAgugcucu....................	67	1	J17
....uaggaacuucauaAcgugcucu....................	175	1	J17
....uaggaacuucauaccCugcucu....................	693	1	J17
....uaggaacuucauaccgugUucu....................	1020	1	J17
....uaggaacuucCuaccgugcucu....................	39	1	J17
....uaggaacuucGuaccgugcucu....................	51	1	J17
....uaggaacuucauaUcgugcucuu...................	4	1	J17
....uaggaacuuAauaccgugcucuu...................	1	1	J17
....Gaggaacuucauaccgugcucuu...................	2	1	J17
....uaggaacuucauaccgCgcucuu...................	4	1	J17
....uaggaacuucauaccCugcucuu...................	5	1	J17
....uaggGacuucauaccgugcucuu...................	1	1	J17
....uCggaacuucauaccgugcucuu...................	1	1	J17
....uagUaacuucauaccgugcucuu...................	2	1	J17

Star Mature

caaaaaaaaucgaauuacacuucgucacgagcgagguuuguaguuccuaccguuuuuauucaaacuggguaggaacuucauaccgugcucuugaacgaugcugacgaucaa			
....uaggaacuucauaccgGgcucuu...................	5	1	J17
....uaggaacuucauaccgugcucuG...................	12	1	J17
....uagAaacuucauaccgugcucuu...................	2	1	J17
....uaggaacuucauaccgugcAcuu...................	2	1	J17
....uaggaacuucauaccgugcucCu...................	14	1	J17
....uaggaacuucauaccgAgcucuu...................	12	1	J17
....uaggaacuucauaccgugcucGu...................	1	1	J17
....uaggaacuucauaccgugAucuu...................	3	1	J17
....uaggaacuucauaccguAcucuu...................	4	1	J17
....uaggaaUuucauaccgugcucuu...................	4	1	J17
....uaggaacuucauaccgugcucuC...................	44	1	J17
....uagCaacuucauaccgugcucuu...................	3	1	J17
....uaggaacuucauacUgugcucuu...................	2	1	J17
....uaggaacuucaAaccgugcucuu...................	2	1	J17
....uaggaacuuUauaccgugcucuu...................	2	1	J17
....uaggaacuucauaccgugcCcuu...................	1	1	J17
....uaggaacuucauaccAugcucuu...................	6	1	J17
....uaggaUcuucauaccgugcucuu...................	2	1	J17
....uaggaacuucaCaccgugcucuu...................	1	1	J17
....uaggaacuucauaccgugcuGuu...................	1	1	J17
....uaggaacuucauaccgugcucuA...................	10364	1	J17
....Caggaacuucauaccgugcucuu...................	1	1	J17
....uaggaacuucauaccguCcucuu...................	1	1	J17
....uaggaacCucauaccgugcucuu...................	1	1	J17
....uaggaacuucauaccgugcuUuu...................	6	1	J17
....uaggaacuucauaccgugcucAu...................	13	1	J17
....uaggCacuucauaccgugcucuu...................	2	1	J17
....uaggaacuucauaccgugcucuu...................	9355	0	J17
....Aaggaacuucauaccgugcucuu...................	5	1	J17
....uaggUacuucauaccgugcucuu...................	4	1	J17
....uaggaacuucauaccgugUucuu...................	4	1	J17
....uaggaacuCcauaccgugcucuu...................	2	1	J17
....uaggaacuucaGaccgugcucuu...................	1	1	J17
....uaggaacuAcauaccgugcucuu...................	2	1	J17
....uaggaacuucauaccgugcucuuA..................	54	1	J17
....uaggaacuucauaccgugcucuuU..................	182	1	J17
....uaggaacuucauaccgugcucuuC..................	5	1	J17
....uaggaacuucauaccgugcucuGg..................	5	1	J17
....uaggaacuucauaccgugcucuAg..................	42	1	J17
....uaggaacuucauaccgugcucCug..................	1	1	J17
....uaggaacuucauaccgugcucuug..................	6	0	J17
....uaggaacuucauaccgugcucuugU.................	1	1	J17
....uaggaacuucauaccgugcucuuCa.................	1	1	J17
....uaggaacuucauaccgugcucuuAa.................	2	1	J17
....uaggaacuucauaccgugcucuuUa.................	4	1	J17
....uaggaacuucauaccgugcucuAgaac...............	1	1	J17
....aggaacuucauaccgugc.......................	2	0	J17
....aggaacuucauaccgugcu......................	9	0	J17
....aUgaacuucauaccgugcuc.....................	2	1	J17
....aggaacuucauaccgugcCc.....................	1	1	J17
....aggaacuucauaccgugcuc.....................	139	0	J17
....aUgaacuucauaccgugcucu....................	22	1	J17
....aggaacuucCuaccgugcucu....................	1	1	J17
....aggaacuAcauaccgugcucu....................	3	1	J17
....aggCacuucauaccgugcucu....................	1	1	J17
....aggaacuucauaccgugcucC....................	6	1	J17
....aggaacuucaCaccgugcucu....................	1	1	J17
....aggaacuuAauaccgugcucu....................	3	1	J17
....Gggaacuucauaccgugcucu....................	8	1	J17
....aggaaGuucauaccgugcucu....................	2	1	J17
....aggaacuCcauaccgugcucu....................	1	1	J17
....Uggaacuucauaccgugcucu....................	8	1	J17
....aggaacuucauUccgugcucu....................	2	1	J17
....aggaacuucauaccgugcucG....................	1	1	J17
....aggaacuucauaccgAgcucu....................	3	1	J17
....aggaacAucauaccgugcucu....................	2	1	J17
....aggaacuucauacUgugcucu....................	2	1	J17
....aggaacuucauaccgugcuGu....................	1	1	J17
....aCgaacuucauaccgugcucu....................	20	1	J17
....aggGacuucauaccgugcucu....................	1	1	J17

Star **Mature**

caaaaaaaaucgaauuacacuucgucacgagcgagguuuguaguuccuaccguuuuuauucaaacugggguaggaacuucauaccgugcucuugaacgaugcugacgaucaa

....aggaUcuucauaccgugcucu....................	3	1	J17
....aAgaacuucauaccgugcucu....................	3	1	J17
....agAaacuucauaccgugcucu....................	2	1	J17
....aggaacuuUauaccgugcucu....................	2	1	J17
....aggaacuucauaccgugcucu....................	3417	0	J17
....agUaacuucauaccgugcucu....................	1	1	J17
....aggaacuGcauaccgugcucu....................	1	1	J17
....aggaacuucauaccgugUucu....................	1	1	J17
....aggaacuucauaccgugcucuu...................	15	0	J17
....aggaacuucauaccgugcucuA...................	15	1	J17
....aggaacuucauaccgugcucuuU..................	1	1	J17
....ggaacuucauaccguAcuc.....................	1	1	J17
....ggaacuucauaccgugcuc.....................	11	0	J17
....Ugaacuucauaccgugcucu....................	55	1	J17
....ggaacuucauaccgugcucu....................	296	0	J17
....Agaacuucauaccgugcucu....................	14	1	J17
....ggaacuucCuaccgugcucu....................	1	1	J17
....Ugaacuucauaccgugcucuu...................	1	1	J17
....ggaacuucauaccgugcucuA...................	1	1	J17
....gaacuucauaccgugcuc.....................	4	0	J17
....Uaacuucauaccgugcucu....................	14	1	J17
....gaacuucauaccgugcuGu....................	1	1	J17
....gaacuucauaccgugcucu....................	116	0	J17
....Aaacuucauaccgugcucu....................	2	1	J17
....gaGcuucauaccgugcucu....................	1	1	J17
....gaacuucauaccgugcucuu...................	1	0	J17
....aacuucauaccgugcucu....................	268	0	J17
....aacuucaCaccgugcucu....................	1	1	J17
....aacuucauaccAugcucu....................	1	1	J17
....aacuCcauaccgugcucu....................	1	1	J17
....aacuucauaccgugcucuA...................	4	1	J17
....aacuucauaccgugcucuu...................	4	0	J17
....acuucauaccgugcucuu...................	1	0	J17

.............................agcgagguuCguaguucc..	1	1	HMA
.............................agcgagguuuguaguucc..	92	0	HMA
.............................agcgaggAuuguaguucc..	1	1	HMA
.............................agcgagguuuguaguucA..	1	1	HMA
.............................agcgagguuuguaguuccu...	1939	0	HMA
.............................agcgagguuUuaguuccu...	1	1	HMA
.............................agcgagguGuguaguuccu...	1	1	HMA
.............................agcgagguuuguaguuccC..	73	1	HMA
.............................aUcgagguuuguaguuccu...	1	1	HMA
.............................agcgagguuGguaguuccu...	1	1	HMA
.............................agcgaggGuuguaguuccu...	1	1	HMA
.............................Ugcgagguuuguaguuccu...	1	1	HMA
.............................agcgagguuugCaguuccu...	1	1	HMA
.............................agcgagguuuguagCuccu...	1	1	HMA
.............................agcgagguCuguaguuccu...	1	1	HMA
.............................agcgagguuCguaguuccu...	1	1	HMA
.............................Cgcgagguuuguaguuccu...	1	1	HMA
.............................agcgaggAuuguaguuccu...	2	1	HMA
.............................agcgagguuuguaguuccA...	17	1	HMA
.............................agcgagguuuguGguuccu...	1	1	HMA
.............................agcgGgguuuguaguuccua..	1	1	HMA
.............................agcgaggGuuguaguuccua..	2	1	HMA
.............................agcgagguuuguaguuccua..	702	0	HMA
.............................agcgagguuUuaguuccua..	1	1	HMA
.............................agcgagguuuguaguuccuG..	1	1	HMA
.............................agcgaggCuuguaguuccua..	1	1	HMA
.............................agcgaggAuuguaguuccua..	1	1	HMA
.............................agcgaggCuuguaguuccuac...	4	1	HMA
.............................agcgGgguuuguaguuccuac...	3	1	HMA
.............................aUcgagguuuguaguuccuac...	1	1	HMA
.............................agUgagguuuguaguuccuac...	6	1	HMA
.............................agcgagguuUuaguuccuac...	1	1	HMA
.............................agcgaggGuuguaguuccuac...	2	1	HMA
.............................agcAagguuuguaguuccuac...	4	1	HMA
.............................agcgaUguuuguaguuccuac...	1	1	HMA
.............................agcgagguuuguaguuccuGc...	1	1	HMA

```
                             Star                         Mature
caaaaaaaaucgaauuacacuucgucacgagcgagguuuguaguuccuaccguuuuauucaaacuggguaggaacuucauaccgugcucuugaacgaugcugacgaucaa
.............................agcUagguuuguaguuccuac............................................................  2      1  HMA
.............................agcgagguuuguaguAccuac............................................................  1      1  HMA
.............................agcgagguuAguaguuccuac............................................................  2      1  HMA
.............................agcgagguuuguaguuccuaU............................................................  2      1  HMA
.............................agcgagguuugCaguuccuac............................................................  4      1  HMA
.............................agcgagguuuguagGuccuac............................................................  4      1  HMA
.............................agcgagguuuguagCuccuac............................................................  3      1  HMA
.............................agcgagguuuguaAuuccuac............................................................  1      1  HMA
.............................agcgagguuuAuaguuccuac............................................................  1      1  HMA
.............................agcgagguuuguaguuccuCc............................................................  1      1  HMA
.............................agcgagguuCguaguuccuac............................................................  4      1  HMA
.............................agcgagguuuguaguuUcuac............................................................  197    1  HMA
.............................agcgagguuuguaguCccuac............................................................  7      1  HMA
.............................Ggcgagguuuguaguuccuac............................................................  3      1  HMA
.............................agcgagguuuCuaguuccuac............................................................  1      1  HMA
.............................agcgaggAuuguaguuccuac............................................................  2      1  HMA
.............................agcgagguCuguaguuccuac............................................................  3      1  HMA
.............................agcgagguuuguaguuccuac............................................................  12762  0  HMA
.............................agcgagguGuguaguuccuac............................................................  1      1  HMA
.............................aAcgagguuuguaguuccuac............................................................  9      1  HMA
.............................Ugcgagguuuguaguuccuac............................................................  1      1  HMA
.............................agcgagguuuguGguuccuac............................................................  3      1  HMA
.............................agcgagguuuguaguuccCac............................................................  3      1  HMA
.............................agcgagguuGguaguuccuacc...........................................................  1      1  HMA
.............................agcgagguuugCaguuccuacc...........................................................  2      1  HMA
.............................agcgagguuuguaAuuccuacc...........................................................  1      1  HMA
.............................agcgagguuuguaguuccuacA...........................................................  2079   1  HMA
.............................agUgagguuuguaguuccuacc...........................................................  3      1  HMA
.............................agcgagguuuguaguuccuacU...........................................................  3654   1  HMA
.............................agcgagguuuguaguuccuacc...........................................................  4328   0  HMA
.............................agcgaUguuuguaguuccuacc...........................................................  1      1  HMA
.............................agcgagguuuAuaguuccuacc...........................................................  3      1  HMA
.............................agcgCgguuuguaguuccuacc...........................................................  1      1  HMA
.............................Ggcgagguuuguaguuccuacc...........................................................  2      1  HMA
.............................agcgagguuugGaguuccuacc...........................................................  1      1  HMA
.............................agcgagguuCguaguuccuacc...........................................................  1      1  HMA
.............................agcgagguuuguGguuccuacc...........................................................  3      1  HMA
.............................agcAagguuuguaguuccuacc...........................................................  3      1  HMA
.............................agcgGgguuuguaguuccuacc...........................................................  1      1  HMA
.............................agcgagguuuguaguuccuGcc...........................................................  1      1  HMA
.............................agcgagguuuguagCuccuacc...........................................................  1      1  HMA
.............................agcgaAguuuguaguuccuacc...........................................................  1      1  HMA
.............................aAcgagguuuguaguuccuacc...........................................................  1      1  HMA
.............................agcgagguuuguaguCccuacc...........................................................  3      1  HMA
.............................agcgagguuuguaguuccGacc...........................................................  1      1  HMA
.............................agcgagguuuguaguuccuaGc...........................................................  1      1  HMA
.............................agcgagguuuguagGuccuacc...........................................................  2      1  HMA
.............................agcgagAuuuguaguuccuacc...........................................................  1      1  HMA
.............................agcgagguuuguaguuccCacc...........................................................  2      1  HMA
.............................agcgaggGuuguaguuccuacc...........................................................  4      1  HMA
.............................agcgagguuuguaguuccuaccU..........................................................  103    1  HMA
.............................agcgagguuuguaguuccuaccC..........................................................  1      1  HMA
.............................agcgagguuuguaguuccuaccg..........................................................  11     0  HMA
.............................agcgagguuuguaguuccuaccA..........................................................  4      1  HMA
.............................agcgagguuuguaguuccuaccgA.........................................................  125    1  HMA
..............................gcgagguuuguaguuccu..............................................................  73     0  HMA
..............................gcgagguuuguaguuccC..............................................................  1      1  HMA
..............................gcgagguuuguaguuccuac............................................................  645    0  HMA
..............................gcgagguuuguGguuccuac............................................................  1      1  HMA
..............................Acgagguuuguaguuccuac............................................................  1      1  HMA
..............................gcgagguuuguaguGccuac............................................................  1      1  HMA
..............................gcAagguuuguaguuccuac............................................................  3      1  HMA
..............................gcgagguuugGaguuccuac............................................................  1      1  HMA
..............................gcgagguuuguaguuccuacc...........................................................  131    0  HMA
..............................gcgagguuuguaguuccuacU...........................................................  93     1  HMA
..............................gcgagguuuguaguuccuaccC..........................................................  1      1  HMA
..............................gcgagguuuguaguuccuaccU..........................................................  94     1  HMA
...............................cgagguuuguaguuccuaccg..........................................................  156    0  HMA
...................................................................Cuaggaacuucauaccgugcucu....................  11     1  HMA
...................................................................guaggaacuucauaccgugcucu....................  11     0  HMA
```

Star Mature

caaaaaaaaucgaauuacacuucgucacgagcgagguuuguaguuccuaccguuuuuauucaaacuggguaggaacuucauaccgugcucuugaacgaugcugacgaucaa			
....uaggaacuucauaccgug........................	96	0	HMA
....uGggaacuucauaccgug........................	1	1	HMA
....uaggaacuucauaccgugc.......................	2	0	HMA
....uaggaacuucauaccgugcu......................	1	0	HMA
....Aaggaacuucauaccgugcuc.....................	1	1	HMA
....uaggaacuucauaccguCcuc.....................	1	1	HMA
....uaggaacuucauaccgugcuc.....................	3890	0	HMA
....uaggaacuucauaccgugcGc.....................	1	1	HMA
....uaggaGcuucauaccgugcuc.....................	1	1	HMA
....uaggaacuucauaccguAcuc.....................	1	1	HMA
....uaggaacuucauaccgugcuU.....................	2	1	HMA
....uaggaacuucaAaccgugcuc.....................	2	1	HMA
....uaggaacGucauaccgugcuc.....................	1	1	HMA
....uGggaacuucauaccgugcuc.....................	2	1	HMA
....Gaggaacuucauaccgugcuc.....................	2	1	HMA
....uaggaacuucauaccgugUuc.....................	3	1	HMA
....uaggaacuucauaAcgugcuc.....................	1	1	HMA
....uaggaacuucauaccgugcuA.....................	3	1	HMA
....uaCgaacuucauaccgugcuc.....................	1	1	HMA
....uaggaacuucauaUcgugcuc.....................	2	1	HMA
....uaggaacuucauaccgugcuG.....................	1	1	HMA
....uaggaacuucauaccgugcAc.....................	1	1	HMA
....uaggaacuucauacUgugcuc.....................	114	1	HMA
....uaggaacuucauaccgugcCc.....................	5	1	HMA
....uaggaacuucauaccAugcuc.....................	3	1	HMA
....uaggaacuucauacAgugcuc.....................	1	1	HMA
....uaggaacuucaCaccgugcuc.....................	2	1	HMA
....uaggaacuucauaccgugcuUu....................	26	1	HMA
....uaggaacuucGuaccgugcucu....................	59	1	HMA
....uagUaacuucauaccgugcucu....................	36	1	HMA
....uUggaacuucauaccgugcucu....................	11	1	HMA
....uaggaacuucauaccgAgcucu....................	21	1	HMA
....uaggaacuucaCaccgugcucu....................	58	1	HMA
....uaggaacuucaGaccgugcucu....................	44	1	HMA
....uaggaacuucauaAcgugcucu....................	20	1	HMA
....uaggaacuucauaccgugcucu....................	330499	0	HMA
....uaggaacuucauaccgugcucG....................	74	1	HMA
....uaggaacuucauaccguAcucu....................	737	1	HMA
....uaggaacuucauacGgugcucu....................	3	1	HMA
....uaggaCcuucauaccgugcucu....................	7	1	HMA
....uaggaGcuucauaccgugcucu....................	97	1	HMA
....Caggaacuucauaccgugcucu....................	130	1	HMA
....uaggaacuucUuaccgugcucu....................	4	1	HMA
....uaggaacuucauaccgugcuAu....................	37	1	HMA
....uaggaacuucauaccguCcucu....................	13	1	HMA
....uaggaacuucauaccgugcucC....................	3147	1	HMA
....uaggCacuucauaccgugcucu....................	5	1	HMA
....uaggaUcuucauaccgugcucu....................	4	1	HMA
....uaggaacuGcauaccgugcucu....................	35	1	HMA
....uaggaacuucauaccgugcAcu....................	38	1	HMA
....uaggaacGucauaccgugcucu....................	43	1	HMA
....uaggaacCucauaccgugcucu....................	95	1	HMA
....uaggaacAucauaccgugcucu....................	23	1	HMA
....uaggaacuucauaccgugcuGu....................	37	1	HMA
....uaggaacuucauUccgugcucu....................	4	1	HMA
....uaggaaUuucauaccgugcucu....................	142	1	HMA
....uaggaacuucauaGcgugcucu....................	7	1	HMA
....uaggaacuucauaccgGgcucu....................	33	1	HMA
....uaggaacuucaAaccgugcucu....................	8	1	HMA
....uaggaacuucauaccgugcGcu....................	170	1	HMA
....uaggaacuucCuaccgugcucu....................	3	1	HMA
....uaggaacuucauaUcgugcucu....................	40	1	HMA
....uaggaacuucauaccgCgcucu....................	48	1	HMA
....uaggaacuucauacUgugcucu....................	225	1	HMA
....uaggUacuucauaccgugcucu....................	10	1	HMA
....uaggaaGuucauaccgugcucu....................	4	1	HMA
....uaggaacuucauaccAugcucu....................	157	1	HMA
....uaggaacuucauaccguUcucu....................	114	1	HMA
....uaggaacuCcauaccgugcucu....................	143	1	HMA
....uaggaacuucauaccgugcucA....................	591	1	HMA

Star Mature

```
caaaaaaaaucgaauuacacuucgucacgagcgagguuuguaguuccuaccguuuuuauucaaacuggguaggaacuucauaccgugcucuugaacgaugcugacgaucaa
.....................................................................uaggaacuuAauaccgugcucu....................  9      1  HMA
.....................................................................uaUgaacuucauaccgugcucu....................  31     1  HMA
.....................................................................uaAgaacuucauaccgugcucu....................  46     1  HMA
.....................................................................uaCgaacuucauaccgugcucu....................  9      1  HMA
.....................................................................uaggaacuucauaccgugcCcu....................  241    1  HMA
.....................................................................uaggaacuuUauaccgugcucu....................  457    1  HMA
.....................................................................uaggaacuucauaccgugGucu....................  9      1  HMA
.....................................................................uaggaacuucauaccUugcucu....................  30     1  HMA
.....................................................................Gaggaacuucauaccgugcucu....................  112    1  HMA
.....................................................................uagAaacuucauaccgugcucu....................  52     1  HMA
.....................................................................uaggGacuucauaccgugcucu....................  92     1  HMA
.....................................................................uaggaacuucauaccCugcucu....................  4      1  HMA
.....................................................................uaggaacuAcauaccgugcucu....................  21     1  HMA
.....................................................................uaggaacuucauaccgugUucu....................  132    1  HMA
.....................................................................Aaggaacuucauaccgugcucu....................  67     1  HMA
.....................................................................uaggaaAuucauaccgugcucu....................  21     1  HMA
.....................................................................uaggaacuucauCccgugcucu....................  2      1  HMA
.....................................................................uCggaacuucauaccgugcucu....................  14     1  HMA
.....................................................................uaggaacuucauGccgugcucu....................  50     1  HMA
.....................................................................uGggaacuucauaccgugcucu....................  121    1  HMA
.....................................................................uaggaacuucauaccgugAucu....................  11     1  HMA
.....................................................................uaggaacuucauacAgugcucu....................  10     1  HMA
.....................................................................uagCaacuucauaccgugcucu....................  7      1  HMA
.....................................................................uaggaacuucauaccgCgcucuu...................  1      1  HMA
.....................................................................uaggaacuucauaccgugcGcuu...................  1      1  HMA
.....................................................................uaggaacuucauaccgugcucuA...................  2      1  HMA
.....................................................................uaggaacuucauaccgugcucuu...................  372    0  HMA
.....................................................................uaggaacuucauaccgugcucAu...................  3      1  HMA
.....................................................................uaggaacuucauaccgugcucuAg..................  1      1  HMA
.....................................................................uaggaacuucauaccgugcucuuU..................  2      1  HMA
.....................................................................uaggaacuucauaccgugcucuAga.................  2      1  HMA
......................................................................aggaacuucauaAcgugcuc.....................  1      1  HMA
......................................................................aggaacuucauaccgugcuc.....................  253    0  HMA
......................................................................aggaacuucauacAgugcuc.....................  1      1  HMA
......................................................................aggaacuucGuaccgugcucu....................  4      1  HMA
......................................................................aggaacuucauaccguAcucu....................  81     1  HMA
......................................................................aggCacuucauaccgugcucu....................  2      1  HMA
......................................................................aCgaacuucauaccgugcucu....................  2      1  HMA
......................................................................aggaacuucauaccAugcucu....................  23     1  HMA
......................................................................aUgaacuucauaccgugcucu....................  1      1  HMA
......................................................................aggaacuucauaccgGgcucu....................  3      1  HMA
......................................................................aggaacuucauaccgugUucu....................  7      1  HMA
......................................................................aggaacuucauacAgugcucu....................  7      1  HMA
......................................................................aggaacuucauaccgugcCcu....................  15     1  HMA
......................................................................aggaGcuucauaccgugcucu....................  4      1  HMA
......................................................................aggaacuucaAaccgugcucu....................  1      1  HMA
......................................................................aggaacuucauaccgugAucu....................  1      1  HMA
......................................................................aggaacuCcauaccgugcucu....................  7      1  HMA
......................................................................aggaacuucauaccgugcucu....................  20794  0  HMA
......................................................................aggaacuucauGccgugcucu....................  3      1  HMA
......................................................................aggaacuuAauaccgugcucu....................  2      1  HMA
......................................................................aggaacuucauaUcgugcucu....................  1      1  HMA
......................................................................aggaacuucauaAcgugcucu....................  3      1  HMA
......................................................................aAgaacuucauaccgugcucu....................  4      1  HMA
......................................................................Uggaacuucauaccgugcucu....................  4      1  HMA
......................................................................aggaacuucauaccgugGucu....................  1      1  HMA
......................................................................aggaaAuucauaccgugcucu....................  3      1  HMA
......................................................................aggGacuucauaccgugcucu....................  8      1  HMA
......................................................................aggaacuucauaGcgugcucu....................  1      1  HMA
......................................................................aggaacuuUauaccgugcucu....................  107    1  HMA
......................................................................Gggaacuucauaccgugcucu....................  11     1  HMA
......................................................................aggaacuucauaccgugcucC....................  221    1  HMA
......................................................................aggaacuucauaccgugcucA....................  33     1  HMA
......................................................................aggaacuucauaccgAgcucu....................  3      1  HMA
......................................................................aggaacuucauaccgugcGcu....................  26     1  HMA
......................................................................aggaacuucauaccgugcAcu....................  2      1  HMA
......................................................................aggaacuucaGaccgugcucu....................  2      1  HMA
......................................................................aggaacuucauaccgugcuUu....................  4      1  HMA
......................................................................aggaaGuucauaccgugcucu....................  1      1  HMA
......................................................................aggaacuucCuaccgugcucu....................  2      1  HMA
```

Star Mature

caaaaaaaaucgaauuacacuucgucacgagcgagguuuguaguuccuaccguuuuuauucaaacuggguaggaacuucauaccgugcucuugaacgaugcugacgaucaa			
....aggaacuucauacUgugcucu....................	4	1	HMA
....aggaacuucauaccgugcucG....................	9	1	HMA
....aggaacuucaCaccgugcucu....................	1	1	HMA
....agAaacuucauaccgugcucu....................	1	1	HMA
....aggaacuucauCccgugcucu....................	2	1	HMA
....aggaacuucUuaccgugcucu....................	1	1	HMA
....aggaacGucauaccgugcucu....................	3	1	HMA
....aggaacuGcauaccgugcucu....................	4	1	HMA
....aggaacuucauaccUugcucu....................	1	1	HMA
....aggaacuucauaccgugcuGu....................	1	1	HMA
....aggaacuucauaccgCgcucu....................	6	1	HMA
....agUaacuucauaccgugcucu....................	4	1	HMA
....aggaacCucauaccgugcucu....................	5	1	HMA
....aggaacuucauaccguUcucu....................	4	1	HMA
....aggaacuucauaccCugcucu....................	13	1	HMA
....aggaacuucauaccgugcucuu...................	1	0	HMA
....aggaacuucauaccgugcucAu...................	1	1	HMA
....ggaacuucauaccgugcGcu....................	1	1	HMA
....ggaGcuucauaccgugcucu....................	1	1	HMA
....ggaacuucauaccgugcucu....................	109	0	HMA
....Caacuucauaccgugcucu....................	1	1	HMA
.........................cacgagcgagguuuguaguucU..	1	1	HFA
...........................cgagcgagguuuguaguuccua..	2	0	HFA
............................Uagcgagguuuguaguuccuac...	1	1	HFA
............................Uagcgagguuuguaguuccuacc..	2	1	HFA
.............................agcgagguuuguaguucU..	1	1	HFA
.............................agcgagguuuguaguucc..	75	0	HFA
.............................agcgagguuuguaguuccC...	8	1	HFA
.............................agcgagguuuguaguCccu...	1	1	HFA
.............................agcgagguuuguaguuUcu...	1	1	HFA
.............................agcgagguuuguaguucUu...	1	1	HFA
.............................agcgagguuuguaguuccA...	2	1	HFA
.............................agcgaggGuuguaguuccu...	2	1	HFA
.............................agUgagguuuguaguuccu...	3	1	HFA
.............................aAcgagguuuguaguuccu...	1	1	HFA
.............................agcgagguuuguagGuccu...	4	1	HFA
.............................agcgUgguuuguaguuccu...	1	1	HFA
.............................aUcgagguuuguaguuccu...	3	1	HFA
.............................agcgagguuuguaguuccu...	2023	0	HFA
.............................agcgagguuuguagCuccu...	1	1	HFA
.............................agcgagguuuguaguuccG...	3	1	HFA
.............................agcgagguuuguaUuuccu...	1	1	HFA
.............................agcUagguuuguaguuccu...	2	1	HFA
.............................agcgagguuuguaguAccu...	1	1	HFA
.............................agcgagguuuguUguuccu...	1	1	HFA
.............................agcgagguuuguaguuccCa..	1	1	HFA
.............................agcgagguuuguGguuccua..	1	1	HFA
.............................agcgaggGuuguaguuccua..	7	1	HFA
.............................agcgagguuuguaguuccuC..	1	1	HFA
.............................agcgagguuuguaguuccuU..	7	1	HFA
.............................Ugcgagguuuguaguuccua..	1	1	HFA
.............................agcgaggCuuguaguuccua..	3	1	HFA
.............................aCcgagguuuguaguuccua..	1	1	HFA
.............................agcgagguuuguagGuccua..	1	1	HFA
.............................Ggcgagguuuguaguuccua..	1	1	HFA
.............................agcgagguuuguagAuccua..	1	1	HFA
.............................agUgagguuuguaguuccua..	3	1	HFA
.............................agcgagguuuguaguuccua..	1792	0	HFA
.............................agcgagguuuguaguuccGa..	2	1	HFA
.............................agcgagguuuguaguuccAa..	2	1	HFA
.............................agcgagguuuguCguuccua..	1	1	HFA
.............................agcgagAuuuguaguuccua..	1	1	HFA
.............................agcgagguuuguagCuccua..	1	1	HFA
.............................agcgagUuuuguaguuccua..	4	1	HFA
.............................agcgGgguuuguaguuccua..	1	1	HFA
.............................agcgagguGuguaguuccuac...	1	1	HFA
.............................agcgagguuCguaguuccuac...	1	1	HFA
.............................agcgagguuuguUguuccuac...	1	1	HFA
.............................agcgagguuuguagGuccuac...	1	1	HFA

```
                             Star                                    Mature
caaaaaaaaucgaauuacacuucgucacgagcgagguuuguaguuccuaccguuuuuauucaaacuggguaggaacuucauaccgugcucuugaacgaugcugacgaucaa
.............................aUcgagguuuguaguuccuac.............................................................    1      1    HFA
.............................agcgagguuuguaguuccuac.............................................................    2776   0    HFA
.............................agcgagguuuguCguuccuac.............................................................    1      1    HFA
.............................agcgagguuuguaguuccuGc.............................................................    3      1    HFA
.............................agcgagguuuguaguuccuaG.............................................................    1      1    HFA
.............................agcgagguuuguaguuccCac.............................................................    1      1    HFA
.............................aCcgagguuuguaguuccuac.............................................................    1      1    HFA
.............................agcgagguuuguaguuUcuac.............................................................    4      1    HFA
.............................agcgaUguuuguaguuccuac.............................................................    1      1    HFA
.............................agcgagguuuguagAuccuac.............................................................    1      1    HFA
.............................agcgagguuuguaguuccuaA.............................................................    7      1    HFA
.............................aAcgagguuuguaguuccuac.............................................................    1      1    HFA
.............................agUgagguuuguaguuccuac.............................................................    8      1    HFA
.............................agcgagguuuguaguuccuaU.............................................................    35     1    HFA
.............................agcgaggGuuguaguuccuac.............................................................    5      1    HFA
.............................agcgagguuuguaguucUuac.............................................................    1      1    HFA
.............................agcgagguuuguaguuccAac.............................................................    2      1    HFA
.............................agcUagguuuguaguuccuac.............................................................    2      1    HFA
.............................agcgagguuuguaguuccuacc............................................................    1993   0    HFA
.............................agcgaUguuuguaguuccuacc............................................................    1      1    HFA
.............................agcgagguuuguaguuccuacG............................................................    6      1    HFA
.............................agcUagguuuguaguuccuacc............................................................    2      1    HFA
.............................agcgagguuuguaguuccuUcc............................................................    1      1    HFA
.............................agcgagguuuguaguuccCacc............................................................    1      1    HFA
.............................agcgaggCuuguaguuccuacc............................................................    1      1    HFA
.............................agcgagguuuguaguuccuaAc............................................................    1      1    HFA
.............................agcgagguuuAuaguuccuacc............................................................    1      1    HFA
.............................agcgagguuuguaguuUcuacc............................................................    1      1    HFA
.............................agcgagguuuguaguuccuacU............................................................    979    1    HFA
.............................agcgagguuuguaguucUuacc............................................................    3      1    HFA
.............................agcgaggGuuguaguuccuacc............................................................    7      1    HFA
.............................agcgagUuuuguaguuccuacc............................................................    1      1    HFA
.............................agUgagguuuguaguuccuacc............................................................    2      1    HFA
.............................agcgagguuuguaguuccuaUc............................................................    2      1    HFA
.............................agcgagguuuguaguuccuacA............................................................    791    1    HFA
.............................agcgagguuuguaguuccuaccA...........................................................    64     1    HFA
.............................agcgagguuuguaguuccuaccg...........................................................    31     0    HFA
.............................agcgagguuuguaguuccuaccC...........................................................    2      1    HFA
.............................agcgagguuuguaguuccuaccU...........................................................    313    1    HFA
.............................agcgagguuuguaguuccuaccgu..........................................................    2      0    HFA
.............................agcgagguuuguaguuccuaccUu..........................................................    3      1    HFA
.............................agcgagguuuguaguuccuaccguu.........................................................    14     0    HFA
.............................agcgagguuuguaguuccuaccguA.........................................................    1      1    HFA
..............................gcgagguuuguaguuccu...............................................................    2      0    HFA
..............................gcgagguuuguaguuccua..............................................................    1      0    HFA
..............................gcgagguuuguaguuccuac.............................................................    8      0    HFA
..............................Acgagguuuguaguuccuacc............................................................    1      1    HFA
..............................gcgagguuuguaguuccuacA............................................................    1      1    HFA
..............................gcgagguuuguaguuccuacc............................................................    5      0    HFA
..............................gcgagguuuguaguuccuacU............................................................    1      1    HFA
..............................gcgagguuuguaguuccuaccA...........................................................    1      1    HFA
..............................gcgagguuuguaguuccuaccg...........................................................    16     0    HFA
...............................cgagguuuguaguuccuac.............................................................    2      0    HFA
...............................cgagguuuguaguuccuacA............................................................    1      1    HFA
...............................cgagguuuguaguuccuacc............................................................    1      0    HFA
...............................cgagguuuguaguuccuaccg...........................................................    1      0    HFA
................................gagguuuguaguuccuac.............................................................    1      0    HFA
................................gagguuuguaguuccuacA............................................................    1      1    HFA
................................gagguuuguaguuccuacc............................................................    3      0    HFA
................................gagguuuguaguuccuacU............................................................    3      1    HFA
................................gagguuuguaguuccuaccU...........................................................    3      1    HFA
................................gagguuuguaguuccuaccguu.........................................................    1      0    HFA
.................................agguuuguaguuccuacU............................................................    2      1    HFA
.................................agguuuguaguuccuacc............................................................    1      0    HFA
.................................agguuuguaguuccuaccguuu........................................................    1      0    HFA
...................................guuuguaguuccuaccguuuu.......................................................    2      0    HFA
....................................uuuguaguuccuaccguuuu.......................................................    1      0    HFA
................................................................cuggguaggaacuucauaccgugcu......................    1      0    HFA
................................................................cuggguaggaacuucauaccgugcuc.....................    1      0    HFA
................................................................cuggguaggaacuucauaccgugcucu....................    1      0    HFA
```

```
                              Star                                       Mature
caaaaaaaaucgaauuacacuucgucacgagcgagguuuguaguuccuaccguuuuuauucaaacuggguaggaacuucauaccgugcucuugaacgaugcugacgaucaa
.................................................................uggguaggaacuucauaccgugcu......................   1     0   HFA
.................................................................uggguaggaacuucauaccgugcuc.....................   1     0   HFA
.................................................................uggguaggaacuucauaccgugcucC....................   1     1   HFA
.................................................................uAgguaggaacuucauaccgugcucu....................   3     1   HFA
..................................................................ggguaggaacuucauaccgugcuc.....................   1     0   HFA
..................................................................ggguaggaacuucauaccgugcuU....................   1     1   HFA
..................................................................ggguaggaacuucauaccgugcucu....................   2     0   HFA
..................................................................gCguaggaacuucauaccgugcucu....................   1     1   HFA
..................................................................Ugguaggaacuucauaccgugcucu....................   1     1   HFA
..................................................................ggUuaggaacuucauaccgugcucu....................   2     1   HFA
..................................................................ggCuaggaacuucauaccgugcucu....................   3     1   HFA
...................................................................gCuaggaacuucauaccgugcuc.....................   1     1   HFA
...................................................................gUuaggaacuucauaccgugcucu....................   1     1   HFA
...................................................................gguaggaacuucauaccgugcucu....................   2     0   HFA
...................................................................gCuaggaacuucauaccgugcucu....................   3     1   HFA
....................................................................Uuaggaacuucauaccgugcu......................   4     1   HFA
....................................................................guaggaacuucauaccgugcu......................   8     0   HFA
....................................................................Uuaggaacuucauaccgugcuc.....................   3     1   HFA
....................................................................guaggaacuucauaccgugcuU....................   1     1   HFA
....................................................................guaggaacuucauaccgugcuc.....................   2     0   HFA
....................................................................Uuaggaacuucauaccgugcucu....................   156   1   HFA
....................................................................Auaggaacuucauaccgugcucu....................   4     1   HFA
....................................................................guaggaacuucauaccgugcucu....................   9     0   HFA
....................................................................Cuaggaacuucauaccgugcucu....................   8     1   HFA
.....................................................................uaggaacuuUauaccgug........................   1     1   HFA
.....................................................................uaggaacuucauaccgug........................   175   0   HFA
.....................................................................uaggaacuucauaccguU........................   2     1   HFA
.....................................................................uaggaacuucauaccguA........................   1     1   HFA
.....................................................................uaggUacuucauaccgug........................   1     1   HFA
.....................................................................uaggaacuucauaccgugG.......................   1     1   HFA
.....................................................................uaggaacuCcauaccgugc.......................   1     1   HFA
.....................................................................uaggaacuucauaccgugA.......................   58    1   HFA
.....................................................................uaggaacuucauaccgugc.......................   131   0   HFA
.....................................................................uaggaacuucauaccgugU.......................   5     1   HFA
.....................................................................uaggaaUuucauaccgugcu......................   1     1   HFA
.....................................................................uaggaacuucauaccgugcG......................   2     1   HFA
.....................................................................uaUgaacuucauaccgugcu......................   2     1   HFA
.....................................................................uaggaacuucauaccgugAu......................   2     1   HFA
.....................................................................uaggaacuCcauaccgugcu......................   1     1   HFA
.....................................................................uaggaacuucauaccgAgcu......................   2     1   HFA
.....................................................................uaggaacuucauaccgugUu......................   2     1   HFA
.....................................................................uaggaacuuUauaccgugcu......................   3     1   HFA
.....................................................................uaggaacuucauaccguUcu......................   7     1   HFA
.....................................................................uaggaacuucauacUgugcu......................   1     1   HFA
.....................................................................uaggaacuucauaccgCgcu......................   2     1   HFA
.....................................................................uaggaacuucauaccgugcA......................   9     1   HFA
.....................................................................uaggaacuAcauaccgugcu......................   2     1   HFA
.....................................................................uaggaacuucauaccgGgcu......................   1     1   HFA
.....................................................................uaggaacuucauacGgugcu......................   6     1   HFA
.....................................................................uaggaacuucauaccgugcC......................   11    1   HFA
.....................................................................uCggaacuucauaccgugcu......................   1     1   HFA
.....................................................................uaggaacuucauaccgugcu......................   3818  0   HFA
.....................................................................uaCgaacuucauaccgugcu......................   1     1   HFA
.....................................................................uaggaacuucauaccguAcu......................   1     1   HFA
.....................................................................uaggGacuucauaccgugcu......................   3     1   HFA
.....................................................................uaggaacuucauaUcgugcu......................   3     1   HFA
.....................................................................uaggaacuucauUccgugcu......................   2     1   HFA
.....................................................................uaggUacuucauaccgugcu......................   4     1   HFA
.....................................................................uaggaacuucauaccguCcu......................   10    1   HFA
.....................................................................uaggaacuucauaccAugcu......................   2     1   HFA
.....................................................................uaggaacuucaCaccgugcu......................   4     1   HFA
.....................................................................uaggCacuucauaccgugcu......................   2     1   HFA
.....................................................................uaggaacuucauaccCugcu......................   1     1   HFA
.....................................................................uaAgaacuucauaccgugcu......................   1     1   HFA
.....................................................................uagAaacuucauaccgugcu......................   1     1   HFA
.....................................................................uaggaacuucaGaccgugcuc.....................   2     1   HFA
.....................................................................uaggaacuucauaccgugcuG.....................   6     1   HFA
.....................................................................uaggaacuuUauaccgugcuc.....................   7     1   HFA
.....................................................................uagUaacuucauaccgugcuc.....................   4     1   HFA
.....................................................................uagAaacuucauaccgugcuc.....................   1     1   HFA
```

Star Mature

caaaaaaaaucgaauuacacuucgucacgagcgagguuuguaguuccuaccguuuuuauucaaacuggguaggaacuucauaccgugcucuugaacgaugcugacgaucaa			
....uaCgaacuucauaccgugcuc.....................	5	1	HFA
....uaggaacuucauaGcgugcuc.....................	3	1	HFA
....uaggaacuucauaccgugcAc.....................	1	1	HFA
....uaggaacuucauaccgugUuc.....................	17	1	HFA
....uaggaacuucauaccgAgcuc.....................	1	1	HFA
....uaggaacuucaCaccgugcuc.....................	3	1	HFA
....uaggaGcuucauaccgugcuc.....................	1	1	HFA
....uaggaacuucGuaccgugcuc.....................	2	1	HFA
....uaggGacuucauaccgugcuc.....................	6	1	HFA
....uaggaUcuucauaccgugcuc.....................	1	1	HFA
....uUggaacuucauaccgugcuc.....................	2	1	HFA
....uaggaacuucauGccgugcuc.....................	1	1	HFA
....uaggaacuuGauaccgugcuc.....................	1	1	HFA
....Caggaacuucauaccgugcuc.....................	2	1	HFA
....uaggaacuucauaccguCcuc.....................	1	1	HFA
....uaggaacuucauUccgugcuc.....................	6	1	HFA
....uaggaacuCcauaccgugcuc.....................	2	1	HFA
....uaggaacuucUuaccgugcuc.....................	2	1	HFA
....uaggaacuucauacUgugcuc.....................	8	1	HFA
....uagCaacuucauaccgugcuc.....................	6	1	HFA
....Gaggaacuucauaccgugcuc.....................	2	1	HFA
....uaggaacCucauaccgugcuc.....................	1	1	HFA
....uaggaacuucauaccgugcuU.....................	742	1	HFA
....uaggaacuucauaUcgugcuc.....................	1	1	HFA
....uaggaacuucauaccgugcGc.....................	2	1	HFA
....uaggaaUuucauaccgugcuc.....................	4	1	HFA
....uaggaacuAcauaccgugcuc.....................	2	1	HFA
....uaggaacuucauaccCugcuc.....................	1	1	HFA
....uaggaacuucauCccgugcuc.....................	2	1	HFA
....uGggaacuucauaccgugcuc.....................	2	1	HFA
....uaggaacuucauaccgugcCc.....................	18	1	HFA
....uaggaacuucauaccgCgcuc.....................	2	1	HFA
....uaggUacuucauaccgugcuc.....................	1	1	HFA
....uaggaacuucauaccguAcuc.....................	19	1	HFA
....uaggaacuucauaccgugcuc.....................	12383	0	HFA
....uaggaacuucauaccAugcuc.....................	13	1	HFA
....uaggCacuucauaccgugcuc.....................	1	1	HFA
....uaggaacuucauaccgugGuc.....................	1	1	HFA
....uaggaacuucauacGgugcuc.....................	7	1	HFA
....uaggaacuucauaccgugcuA.....................	145	1	HFA
....uaggaacuucauaccgGgcuc.....................	8	1	HFA
....uaggaGcuucauaccgugcucu....................	14	1	HFA
....uaAgaacuucauaccgugcucu....................	15	1	HFA
....uaggaacuucauaccgugGucu....................	5	1	HFA
....uaggaacuucaAaccgugcucu....................	4	1	HFA
....uaggaacuucauaccguUcucu....................	24	1	HFA
....uaggaacuucauaccguCcucu....................	20	1	HFA
....uaggaacuucauaccCugcucu....................	49	1	HFA
....uaCgaacuucauaccgugcucu....................	44	1	HFA
....uaggaUcuucauaccgugcucu....................	17	1	HFA
....uaggUacuucauaccgugcucu....................	82	1	HFA
....uCggaacuucauaccgugcucu....................	17	1	HFA
....uaggaacuucauacUgugcucu....................	215	1	HFA
....uaggaacuuAauaccgugcucu....................	2	1	HFA
....uaggaacuucauaccgGgcucu....................	95	1	HFA
....uaggaacCucauaccgugcucu....................	36	1	HFA
....uaggaacuucauGccgugcucu....................	17	1	HFA
....uaggGacuucauaccgugcucu....................	211	1	HFA
....uaggaacuucauaccgugcCcu....................	163	1	HFA
....uaggaacAucauaccgugcucu....................	26	1	HFA
....uaggaacuucauaccgugcucA....................	134	1	HFA
....uaggaaAuucauaccgugcucu....................	1	1	HFA
....uaggaacuucauaccgCgcucu....................	34	1	HFA
....uaggaacuuUauaccgugcucu....................	280	1	HFA
....uaggaacuucauaUcgugcucu....................	99	1	HFA
....uGggaacuucauaccgugcucu....................	78	1	HFA
....uaggaacuucauaccgugcuGu....................	55	1	HFA
....uaggaacGucauaccgugcucu....................	10	1	HFA
....uaggaacuucUuaccgugcucu....................	32	1	HFA
....uagUaacuucauaccgugcucu....................	54	1	HFA

```
                               Star                                    Mature
caaaaaaaaucgaauuacacuucgucacgagcgagguuuguaguuccuaccguuuuuauucaaacuggguaggaacuucauaccgugcucuugaacgaugcugacgaucaa
.....................................................................Caggaacuucauaccgugcucu....................   17      1   HFA
.....................................................................uaggaaGuucauaccgugcucu....................   4       1   HFA
.....................................................................uaggaacuucauaccgugcucC....................   3790    1   HFA
.....................................................................uUggaacuucauaccgugcucu....................   70      1   HFA
.....................................................................uaggaacuucauaccgAgcucu....................   32      1   HFA
.....................................................................uaggaacuucauaccUugcucu....................   28      1   HFA
.....................................................................uaggaacuucauaAcgugcucu....................   4       1   HFA
.....................................................................Gaggaacuucauaccgugcucu....................   18      1   HFA
.....................................................................uaggaacuCcauaccgugcucu....................   91      1   HFA
.....................................................................uaggaacuucauaccgugcucu....................   310956  0   HFA
.....................................................................uaggaacuucauaccgugcuAu....................   34      1   HFA
.....................................................................uaggaacuucCuaccgugcucu....................   8       1   HFA
.....................................................................uaggaacuucauaGcgugcucu....................   29      1   HFA
.....................................................................uaggaacuucauUccgugcucu....................   113     1   HFA
.....................................................................uaggaacuucauaccAugcucu....................   47      1   HFA
.....................................................................Aaggaacuucauaccgugcucu....................   23      1   HFA
.....................................................................uaggaacuucauaccgugcuUu....................   85      1   HFA
.....................................................................uaggaacuucauaccgugUucu....................   227     1   HFA
.....................................................................uaggaacuucauCccgugcucu....................   30      1   HFA
.....................................................................uaggaacuucauaccgugcAcu....................   12      1   HFA
.....................................................................uaggaacuGcauaccgugcucu....................   4       1   HFA
.....................................................................uaggaacuucauacGgugcucu....................   154     1   HFA
.....................................................................uaggaacuuGauaccgugcucu....................   17      1   HFA
.....................................................................uaggCacuucauaccgugcucu....................   50      1   HFA
.....................................................................uagCaacuucauaccgugcucu....................   56      1   HFA
.....................................................................uaggaacuucauaccgugcGcu....................   19      1   HFA
.....................................................................uaggaaUuucauaccgugcucu....................   51      1   HFA
.....................................................................uaggaacuAcauaccgugcucu....................   17      1   HFA
.....................................................................uagAaacuucauaccgugcucu....................   14      1   HFA
.....................................................................uaggaacuucauaccguAcucu....................   58      1   HFA
.....................................................................uaggaacuucauaccgugcucG....................   60      1   HFA
.....................................................................uaUgaacuucauaccgugcucu....................   54      1   HFA
.....................................................................uaggaacuucauaccgugAucu....................   4       1   HFA
.....................................................................uaggaacuucGuaccgugcucu....................   31      1   HFA
.....................................................................uaggaacuucauacAgugcucu....................   47      1   HFA
.....................................................................uaggaacuucaCaccgugcucu....................   47      1   HFA
.....................................................................uaggaacuucaGaccgugcucu....................   7       1   HFA
.....................................................................uaggaacuGcauaccgugcucuu...................   1       1   HFA
.....................................................................uaggaacuucauaccguAcucuu...................   2       1   HFA
.....................................................................uaggaacuucauaccgGgcucuu...................   1       1   HFA
.....................................................................uaggaacuucauaccgugcucuG...................   8       1   HFA
.....................................................................Gaggaacuucauaccgugcucuu...................   1       1   HFA
.....................................................................uaggaacuucauacUgugcucuu...................   1       1   HFA
.....................................................................uaggaacuuUauaccgugcucuu...................   1       1   HFA
.....................................................................uaggaacuucauaccgugUucuu...................   2       1   HFA
.....................................................................uaggaacuucauacGgugcucuu...................   1       1   HFA
.....................................................................uaggaacuucauaccgugcucuA...................   845     1   HFA
.....................................................................uaggaacuucauaccgugcucuC...................   11      1   HFA
.....................................................................uaggaacuucauaccgugcucuu...................   1509    0   HFA
.....................................................................uaggaacuucaCaccgugcucuu...................   1       1   HFA
.....................................................................uaggaacuCcauaccgugcucuu...................   1       1   HFA
.....................................................................uaggaacuucauaccgugcucAu...................   2       1   HFA
.....................................................................uaggaaUuucauaccgugcucuu...................   1       1   HFA
.....................................................................uaggaacuucauaccgugcucCu...................   46      1   HFA
.....................................................................uaggaacuucauaccgugcucuuU..................   5       1   HFA
.....................................................................uaggaacuucauaccgugcucuGg..................   3       1   HFA
.....................................................................uaggaacuucauaccgugcucuAg..................   1       1   HFA
.....................................................................uaggaacuucauaccgugcucuuC..................   2       1   HFA
.....................................................................uaggaacuucauaccgugcucuuA..................   1       1   HFA
......................................................................aggaacuucauaccgugc.......................   2       0   HFA
......................................................................aggaacuucauaccgugcu......................   13      0   HFA
......................................................................aggaacuucauaccgugcuU.....................   1       1   HFA
......................................................................aUgaacuucauaccgugcuc.....................   1       1   HFA
......................................................................aggaacuucauaccguAcuc.....................   1       1   HFA
......................................................................aggaacuucauaccgugcuc.....................   44      0   HFA
......................................................................aggaacuGcauaccgugcucu....................   1       1   HFA
......................................................................Cggaacuucauaccgugcucu....................   1       1   HFA
......................................................................Gggaacuucauaccgugcucu....................   3       1   HFA
......................................................................aggaaUuucauaccgugcucu....................   1       1   HFA
......................................................................aUgaacuucauaccgugcucu....................   10      1   HFA
```

Star Mature

caaaaaaaaucgaauuacacuucgucacgagcgagguuuguaguuccuaccguuuuuauucaaacuggguaggaacuucauaccgugcucuugaacgaugcugacgaucaa			
....aggaacuucauaccgugUucu....................	2	1	HFA
....aggaacuucauaccgugcAcu....................	1	1	HFA
....Uggaacuucauaccgugcucu....................	3	1	HFA
....aAgaacuucauaccgugcucu....................	3	1	HFA
....aggaacuucauaccgugcucu....................	1230	0	HFA
....aggaacuucauaccgGgcucu....................	1	1	HFA
....aggaacuucaGaccgugcucu....................	2	1	HFA
....agUaacuucauaccgugcucu....................	2	1	HFA
....aCgaacuucauaccgugcucu....................	7	1	HFA
....agCaacuucauaccgugcucu....................	1	1	HFA
....aggaacuucauaccgugcucC....................	23	1	HFA
....aggaacuucauaccgugcucuu...................	7	0	HFA
....aggaacuucauaccgugcucCu...................	1	1	HFA
....aggaacuucauaccgugcucuA...................	5	1	HFA
....aggaacuucauaccgugcucuuU..................	1	1	HFA
....Ugaacuucauaccgugcu......................	1	1	HFA
....ggaacuucauaccgugcu......................	2	0	HFA
....gUaacuucauaccgugcuc.....................	1	1	HFA
....ggaacuucauaccgugcuc.....................	6	0	HFA
....gUaacuucauaccgugcucu....................	3	1	HFA
....ggaacuucauaccgugcucu....................	81	0	HFA
....Ugaacuucauaccgugcucu....................	19	1	HFA
....Cgaacuucauaccgugcucu....................	1	1	HFA
....ggaacuucauaccgugcucC....................	1	1	HFA
....Agaacuucauaccgugcucu....................	5	1	HFA
....ggaacuucauaccgugcucuA...................	1	1	HFA
....gaacuucauaccgugcuc.....................	1	0	HFA
....Uaacuucauaccgugcuc.....................	2	1	HFA
....Aaacuucauaccgugcuc.....................	1	1	HFA
....Uaacuucauaccgugcucu....................	11	1	HFA
....gaacuucauaccgugcucC....................	1	1	HFA
....gaacuucauaccgugcucu....................	60	0	HFA
....Aaacuucauaccgugcucu....................	1	1	HFA
....gaacuucauaccguAcucu....................	2	1	HFA
....gaacuucauaccgugcucuA...................	1	1	HFA
....gaacuucauaccgugcucCu...................	1	1	HFA
....aacuuUauaccgugcucu....................	1	1	HFA
....aacuucauaccgugcucC....................	4	1	HFA
....aacuucauaccgugcucu....................	381	0	HFA
....aacuucauaccUugcucu....................	2	1	HFA
....aCcuucauaccgugcucu....................	2	1	HFA
....Gacuucauaccgugcucu....................	1	1	HFA
....aacuucaCaccgugcucu....................	2	1	HFA
....aacuucauaccgugUucu....................	2	1	HFA
....aacuucauaccgugcucuA...................	1	1	HFA
....acuucauaccgugcucuA...................	1	1	HFA
....acuucauaccgugcucuu...................	3	0	HFA
.......aaucgaauuacacuucgucacg..	1	0	HS5
.........................cacgagcgagguuuguaguucc..	1	0	HS5
.........................cacgagcgagguuuguaguuccu...	1	0	HS5
..........................acgagcgagguuuguaguuccu...	1	0	HS5
..........................aGgagcgagguuuguaguuccuacc..	1	1	HS5
............................Uagcgagguuuguaguuccuac...	1	1	HS5
............................Uagcgagguuuguaguuccuacc..	4	1	HS5
.............................agcgagguuuguaguucc..	3	0	HS5
.............................agcgagguuuguaguCccu...	1	1	HS5
.............................agcgagguuuguaguGccu...	1	1	HS5
.............................agcgagguuuguaguuccC...	1	1	HS5
.............................agcgagguuuguaguuccG...	2	1	HS5
.............................agcgagguuuguaguuccu...	250	0	HS5
.............................Ugcgagguuuguaguuccua..	1	1	HS5
.............................agcgaUguuuguaguuccua..	1	1	HS5
.............................agcgagguuuguaguucUua..	1	1	HS5
.............................agcgagguuuguaguuccCa..	2	1	HS5
.............................agcgagguuugCaguuccua..	3	1	HS5
.............................agcgaggGuuguaguuccua..	2	1	HS5
.............................agUgagguuuguaguuccua..	1	1	HS5
.............................agAgagguuuguaguuccua..	1	1	HS5
.............................agcgagguuuguaguuccua..	1216	0	HS5

```
                             Star                                   Mature
caaaaaaaaucgaauuacacuucgucacgagcgagguuuguaguuccuaccguuuuauucaaacugggSuaggaacuucauaccgugcucuugaacgaugcugacgaucaa
.............................agcgagguuuguaguAccua.............................................................	3	1	HS5
.............................agcgagguuuguCguuccua.............................................................	1	1	HS5
.............................agcgagguuuguaguuGcua.............................................................	1	1	HS5
.............................agcgagguuuAuaguuccua.............................................................	1	1	HS5
.............................agcgaCguuuguaguuccua.............................................................	1	1	HS5
.............................agcgaggCuuguaguuccua.............................................................	1	1	HS5
.............................agcgaggGuuguaguuccuac............................................................	2	1	HS5
.............................agcgagguuuguUguuccuac............................................................	1	1	HS5
.............................agAgagguuuguaguuccuac............................................................	1	1	HS5
.............................agcUagguuuguaguuccuac............................................................	1	1	HS5
.............................agcgagguuuguaguCccuac............................................................	5	1	HS5
.............................agUgagguuuguaguuccuac............................................................	5	1	HS5
.............................agcgagguuAguaguuccuac............................................................	1	1	HS5
.............................agcgaUguuuguaguuccuac............................................................	1	1	HS5
.............................agcgagguuuguaguAccuac............................................................	2	1	HS5
.............................aCcgagguuuguaguuccuac............................................................	2	1	HS5
.............................agcgagguuuguaguuccuaU............................................................	15	1	HS5
.............................agcgagguuugAaguuccuac............................................................	1	1	HS5
.............................agcgagguuuguaguucUuac............................................................	9	1	HS5
.............................agcgagguuuguCguuccuac............................................................	1	1	HS5
.............................agcgagguuugGaguuccuac............................................................	1	1	HS5
.............................agcgagguuuguaguuccuac............................................................	1733	0	HS5
.............................agcgagguuugCaguuccuac............................................................	3	1	HS5
.............................Ggcgagguuuguaguuccuac............................................................	1	1	HS5
.............................agcgagguuuguaguuUcuac............................................................	3	1	HS5
.............................agcgagguuuguaguuccuaA............................................................	2	1	HS5
.............................agcgUgguuuguaguuccuac............................................................	1	1	HS5
.............................agcgGgguuuguaguuccuacc...........................................................	2	1	HS5
.............................agcgaCguuuguaguuccuacc...........................................................	2	1	HS5
.............................aAcgagguuuguaguuccuacc...........................................................	1	1	HS5
.............................agGgagguuuguaguuccuacc...........................................................	1	1	HS5
.............................agcgagguuuguaguuUcuacc...........................................................	25	1	HS5
.............................agcgagguuuguagGuccuacc...........................................................	1	1	HS5
.............................agcgagguuuguagCuccuacc...........................................................	2	1	HS5
.............................Cgcgagguuuguaguuccuacc...........................................................	1	1	HS5
.............................agcUagguuuguaguuccuacc...........................................................	3	1	HS5
.............................agcgagguuuguaAuuccuacc...........................................................	1	1	HS5
.............................agcgaUguuuguaguuccuacc...........................................................	4	1	HS5
.............................agcgagguuugGaguuccuacc...........................................................	5	1	HS5
.............................agcgagguuugCaguuccuacc...........................................................	8	1	HS5
.............................agcgaggAuuguaguuccuacc...........................................................	1	1	HS5
.............................agcgagguuuguaguuccuaUc...........................................................	3	1	HS5
.............................agcgagguuuguaguCccuacc...........................................................	6	1	HS5
.............................agcgagguuuguaguuccCacc...........................................................	2	1	HS5
.............................agcgagguuuguaguAccuacc...........................................................	8	1	HS5
.............................agcgagguuuguaguuccuacG...........................................................	5	1	HS5
.............................agcgagguuuguaguuccuacc...........................................................	9913	0	HS5
.............................aUcgagguuuguaguuccuacc...........................................................	1	1	HS5
.............................agcgagAuuuguaguuccuacc...........................................................	2	1	HS5
.............................aCcgagguuuguaguuccuacc...........................................................	3	1	HS5
.............................agAgagguuuguaguuccuacc...........................................................	1	1	HS5
.............................agcgagguuuguagAuccuacc...........................................................	1	1	HS5
.............................agUgagguuuguaguuccuacc...........................................................	12	1	HS5
.............................agcgagguuuguaguuccAacc...........................................................	1	1	HS5
.............................agcgagUuuuguaguuccuacc...........................................................	3	1	HS5
.............................agcgagguuuguaguuccuacU...........................................................	450	1	HS5
.............................agcgagguuuguCguuccuacc...........................................................	10	1	HS5
.............................agcgagguuuguaguGccuacc...........................................................	5	1	HS5
.............................agcgagguuAguaguuccuacc...........................................................	2	1	HS5
.............................agcgagguuuguUguuccuacc...........................................................	1	1	HS5
.............................agcgagguuugAaguuccuacc...........................................................	2	1	HS5
.............................agcgagguuCguaguuccuacc...........................................................	5	1	HS5
.............................agcgaggCuuguaguuccuacc...........................................................	1	1	HS5
.............................agcgagguuuguaguuccuGcc...........................................................	1	1	HS5
.............................agcgagguCuguaguuccuacc...........................................................	1	1	HS5
.............................agcgUgguuuguaguuccuacc...........................................................	2	1	HS5
.............................agcgagguuuguGguuccuacc...........................................................	4	1	HS5
.............................Ugcgagguuuguaguuccuacc...........................................................	1	1	HS5
.............................Ggcgagguuuguaguuccuacc...........................................................	1	1	HS5
.............................agcgagguuuguaguucUuacc...........................................................	12	1	HS5
```

```
                             Star                                      Mature
caaaaaaaaucgaauuacacuucgucacgagcgagguuuguaguuccuaccguuuuuauucaaacuggguaggaacuucauaccgugcucuugaacgaugcugacgaucaa
.............................agcgagguuuguaguuccuacA............................................................   115   1   HS5
.............................agcgaAguuuguaguuccuacc............................................................   1     1   HS5
.............................agcgaggGuuguaguuccuacc............................................................   28    1   HS5
.............................agcgagguuuguaguuccuaccU...........................................................   666   1   HS5
.............................agcgagguuuguaguuccuaccC...........................................................   9     1   HS5
.............................agcgagguuuguaguCccuaccg...........................................................   2     1   HS5
.............................agUgagguuuguaguuccuaccg...........................................................   1     1   HS5
.............................agcgagguuuguCguuccuaccg...........................................................   1     1   HS5
.............................agcgagguuugCaguuccuaccg...........................................................   1     1   HS5
.............................agcgagguuuguaguuccuaccg...........................................................   334   0   HS5
.............................agcgagguuuguaguucUuaccg...........................................................   2     1   HS5
.............................agcgagguuuguaguuccuaccA...........................................................   105   1   HS5
.............................agcgagguuuguaguuccuaccUu..........................................................   7     1   HS5
.............................agcgagguuuguaguuccuaccgu..........................................................   7     0   HS5
.............................agcgagguuuguaguuccuaccCu..........................................................   2     1   HS5
.............................agcgagguuuguaguuccuaccUuu.........................................................   2     1   HS5
..............................gcgagguuuguaguuccuac.............................................................   3     0   HS5
..............................gcgagguuuguaguuccuacc............................................................   31    0   HS5
..............................gcgagguuuguCguuccuaccg...........................................................   1     1   HS5
..............................gcgagguuuguaguuccuaccg...........................................................   40    0   HS5
..............................gcgagguuuguaguuccuaccU...........................................................   2     1   HS5
..............................gcgagguuuguaguuccuaccguu.........................................................   1     0   HS5
...............................cgagguuuguaguuccuacc............................................................   5     0   HS5
...............................cgagguuuguaguuccuaccC...........................................................   3     1   HS5
...............................cgagguuuguaguuccuaccg...........................................................   15    0   HS5
................................gagguuuguaguuccuac.............................................................   1     0   HS5
................................Uagguuuguaguuccuacc............................................................   1     1   HS5
................................gagguuuguaguuccuacc............................................................   7     0   HS5
.................................agguuuguaguuccuacc............................................................   1     0   HS5
.................................agguuuguaguuccuaccguuu........................................................   1     0   HS5
...................................................guuuuuauucaaacuggg..........................................   3     0   HS5
.................................................................Gggguaggaacuucauaccgugcucu....................   1     1   HS5
..................................................................ggCuaggaacuucauaccgugcucu....................   5     1   HS5
..................................................................gAguaggaacuucauaccgugcucu....................   4     1   HS5
..................................................................Agguaggaacuucauaccgugcucu....................   1     1   HS5
..................................................................ggguaggaacuucauaccgugcucu....................   4     0   HS5
..................................................................ggUuaggaacuucauaccgugcucu....................   1     1   HS5
...................................................................gguaggaacuucauaccgugcucu....................   1     0   HS5
...................................................................gCuaggaacuucauaccgugcucu....................   4     1   HS5
....................................................................guaggaacuucauaccgugc.......................   1     0   HS5
....................................................................guaggaacuucauaccgugcu......................   38    0   HS5
....................................................................Uuaggaacuucauaccgugcu......................   1     1   HS5
....................................................................guaggUacuucauaccgugcu......................   1     1   HS5
....................................................................guaggaacuucauaccgugcuU.....................   1     1   HS5
....................................................................guaggaacuucauaccgugcuc.....................   65    0   HS5
....................................................................Uuaggaacuucauaccgugcuc.....................   6     1   HS5
....................................................................Cuaggaacuucauaccgugcucu....................   10    1   HS5
....................................................................guaggaacuucauaccgugcucu....................   194   0   HS5
....................................................................guaggaacuuUauaccgugcucu....................   1     1   HS5
....................................................................guaggaacuucauaccgAgcucu....................   1     1   HS5
....................................................................gCaggaacuucauaccgugcucu....................   1     1   HS5
....................................................................Auaggaacuucauaccgugcucu....................   3     1   HS5
....................................................................Uuaggaacuucauaccgugcucu....................   294   1   HS5
....................................................................Uuaggaacuucauaccgugcucuu...................   1     1   HS5
....................................................................guaggaacuucauaccgugcucuu...................   3     0   HS5
.....................................................................uaggaacuucauCccgug........................   1     1   HS5
.....................................................................uaggaGcuucauaccgug........................   1     1   HS5
.....................................................................uaggUacuucauaccgug........................   1     1   HS5
.....................................................................uaggaacuucauaccgug........................   73    0   HS5
.....................................................................uaggaacuucauaccgugA.......................   22    1   HS5
.....................................................................uaggGacuucauaccgugc.......................   1     1   HS5
.....................................................................uaggaacuucauaccgugc.......................   123   0   HS5
.....................................................................uaggaacuucaGaccgugcu......................   3     1   HS5
.....................................................................uaggaaUuucauaccgugcu......................   1     1   HS5
.....................................................................uaggaacuucauaccgAgcu......................   1     1   HS5
.....................................................................uaggaacCucauaccgugcu......................   1     1   HS5
.....................................................................uaggaacuucauaccguAcu......................   8     1   HS5
.....................................................................uaggaCcuucauaccgugcu......................   1     1   HS5
.....................................................................uagAaacuucauaccgugcu......................   2     1   HS5
.....................................................................uaggaacuucauaccgGgcu......................   1     1   HS5
```

Star **Mature**

caaaaaaaaucgaauuacacuucgucacgagcgagguuuguaguuccuaccguuuuuauucaaacuggguaggaacuucauaccgugcucuugaacgaugcugacgaucaa

....uaggaacuucauacUgugcu......................	2	1	HS5
....uaggaacuucauaccgugcu......................	2285	0	HS5
....uaggaacuucauaccgugcG......................	1	1	HS5
....uaggaacuucauaccguUcu......................	17	1	HS5
....uGggaacuucauaccgugcu......................	1	1	HS5
....uaggaacuucauaccUugcu......................	3	1	HS5
....uaggaacuucauaccgCgcu......................	1	1	HS5
....Caggaacuucauaccgugcu......................	1	1	HS5
....uaggaacuucauaccgugUu......................	2	1	HS5
....uaggaacuucauaccguCcu......................	237	1	HS5
....Aaggaacuucauaccgugcu......................	1	1	HS5
....uaggaacuucauaUcgugcu......................	6	1	HS5
....uaggaacuucauaccAugcu......................	3	1	HS5
....uaggaacuucauaccgugcA......................	2	1	HS5
....uaggaacuucauaccgugcC......................	4	1	HS5
....uaggUacuucauaccgugcu......................	3	1	HS5
....Gaggaacuucauaccgugcu......................	1	1	HS5
....uaggaUcuucauaccgugcu......................	1	1	HS5
....uaggaaGuucauaccgugcu......................	1	1	HS5
....Caggaacuucauaccgugcuc.....................	3	1	HS5
....uaggaacAucauaccgugcuc.....................	2	1	HS5
....uaggaacuucauUccgugcuc.....................	6	1	HS5
....uaggaacuucCuaccgugcuc.....................	1	1	HS5
....uaggaacuucUuaccgugcuc.....................	3	1	HS5
....uaggaacuucauaccgugcuU.....................	246	1	HS5
....uaggaacuucauaccgugcAc.....................	1	1	HS5
....Gaggaacuucauaccgugcuc.....................	4	1	HS5
....uaggaacuucauaccgCgcuc.....................	8	1	HS5
....uaggaaUuucauaccgugcuc.....................	4	1	HS5
....uaggaacuucauaccgugcCc.....................	8	1	HS5
....uaggUacuucauaccgugcuc.....................	7	1	HS5
....uaggaacuucauaccgugUuc.....................	9	1	HS5
....uaggaacuucaCaccgugcuc.....................	11	1	HS5
....uaggaacuucauCccgugcuc.....................	8	1	HS5
....uaggaacuucauaccgugAuc.....................	2	1	HS5
....uaggaacuucauaUcgugcuc.....................	11	1	HS5
....uagCaacuucauaccgugcuc.....................	4	1	HS5
....uaCgaacuucauaccgugcuc.....................	7	1	HS5
....uaggaacuucauaAcgugcuc.....................	1	1	HS5
....uaggaacuucauaccgugcuc.....................	18548	0	HS5
....uaggaacuucauaccgugcGc.....................	1	1	HS5
....uaggaacuGcauaccgugcuc.....................	1	1	HS5
....uaggaacuucGuaccgugcuc.....................	1	1	HS5
....uaggaacuAcauaccgugcuc.....................	2	1	HS5
....uaggaacCucauaccgugcuc.....................	1	1	HS5
....uagAaacuucauaccgugcuc.....................	3	1	HS5
....uagUaacuucauaccgugcuc.....................	12	1	HS5
....uaggaacuucauacUgugcuc.....................	12	1	HS5
....uaggaacuuUauaccgugcuc.....................	5	1	HS5
....uaUgaacuucauaccgugcuc.....................	4	1	HS5
....uaggaacuuGauaccgugcuc.....................	2	1	HS5
....uaggCacuucauaccgugcuc.....................	4	1	HS5
....Aaggaacuucauaccgugcuc.....................	6	1	HS5
....uaggaacuucauaccCugcuc.....................	3	1	HS5
....uaggaacuucauaccgugcuA.....................	34	1	HS5
....uaggaacuucauaccAugcuc.....................	10	1	HS5
....uaggaGcuucauaccgugcuc.....................	2	1	HS5
....uaggaacuucauaccgGgcuc.....................	2	1	HS5
....uaggGacuucauaccgugcuc.....................	6	1	HS5
....uaggaacuucaGaccgugcuc.....................	9	1	HS5
....uaggaacuucauaccgAgcuc.....................	9	1	HS5
....uaggaacuucauaccUugcuc.....................	4	1	HS5
....uaggaacuucauaccguAcuc.....................	19	1	HS5
....uaggaacuucauaccguCcuc.....................	2	1	HS5
....uaggaacuucauGccgugcuc.....................	3	1	HS5
....uaggaacuucauacAgugcuc.....................	2	1	HS5
....uGggaacuucauaccgugcuc.....................	3	1	HS5
....uaggaUcuucauaccgugcuc.....................	3	1	HS5
....uUggaacuucauaccgugcucu....................	55	1	HS5
....uaggaacuucauaccgugGucu....................	17	1	HS5

Star **Mature**

```
caaaaaaaaucgaauuacacuucgucacgagcgagguuuguaguuccuaccguuuuuauucaaacuggguaggaacuucauaccgugcucuugaacgaugcugacgaucaa
.....................................................................uaggaacuucauaccgugcucA....................  258      1  HS5
.....................................................................uaggaacuucauaccgugcuGu....................  6        1  HS5
.....................................................................uagUaacuucauaccgugcucu....................  394      1  HS5
.....................................................................uaggaacuucauaGcgugcucu....................  18       1  HS5
.....................................................................uaggaUcuucauaccgugcucu....................  431      1  HS5
.....................................................................uaggUacuucauaccgugcucu....................  711      1  HS5
.....................................................................uaggaacuAcauaccgugcucu....................  61       1  HS5
.....................................................................uaggaacuucauaccgugcAcu....................  27       1  HS5
.....................................................................uaggaacuucauaccgugcGcu....................  22       1  HS5
.....................................................................uaggaacuucauaAcgugcucu....................  15       1  HS5
.....................................................................uaggaacuucUuaccgugcucu....................  33       1  HS5
.....................................................................uaggaacuucauaccgCgcucu....................  421      1  HS5
.....................................................................uaggaacuucGuaccgugcucu....................  38       1  HS5
.....................................................................uaggaacuucauaccgugcuAu....................  11       1  HS5
.....................................................................uGggaacuucauaccgugcucu....................  177      1  HS5
.....................................................................uaggaacuucCuaccgugcucu....................  13       1  HS5
.....................................................................uaggaacuucauaUcgugcucu....................  548      1  HS5
.....................................................................uaggaacGucauaccgugcucu....................  26       1  HS5
.....................................................................uaggCacuucauaccgugcucu....................  304      1  HS5
.....................................................................uCggaacuucauaccgugcucu....................  11       1  HS5
.....................................................................uaggaacuucauaccguAcucu....................  252      1  HS5
.....................................................................uaggaacuucauaccgugUucu....................  1078     1  HS5
.....................................................................uaggaacuucauaccgugcuUu....................  266      1  HS5
.....................................................................uaggaacuuGauaccgugcucu....................  111      1  HS5
.....................................................................uaggaacuucaCaccgugcucu....................  723      1  HS5
.....................................................................uaggaacuuUauaccgugcucu....................  478      1  HS5
.....................................................................uaggaacAucauaccgugcucu....................  118      1  HS5
.....................................................................uagAaacuucauaccgugcucu....................  233      1  HS5
.....................................................................uaggaacuucaGaccgugcucu....................  480      1  HS5
.....................................................................uaggaacuucauGccgugcucu....................  145      1  HS5
.....................................................................uaggaacuucauaccgugcCcu....................  132      1  HS5
.....................................................................uaggaacuucauaccgAgcucu....................  661      1  HS5
.....................................................................uaUgaacuucauaccgugcucu....................  282      1  HS5
.....................................................................uaggaacuucauaccUugcucu....................  277      1  HS5
.....................................................................uaggaAuucauaccgugcucu....................  30       1  HS5
.....................................................................uaggGacuucauaccgugcucu....................  448      1  HS5
.....................................................................uaggaacuucauaccgGgcucu....................  275      1  HS5
.....................................................................uaggaacuucauacGgugcucu....................  11       1  HS5
.....................................................................uaCgaacuucauaccgugcucu....................  313      1  HS5
.....................................................................uaggaGcuucauaccgugcucu....................  92       1  HS5
.....................................................................uaggaacuucaAaccgugcucu....................  49       1  HS5
.....................................................................uagCaacuucauaccgugcucu....................  390      1  HS5
.....................................................................uaAgaacuucauaccgugcucu....................  93       1  HS5
.....................................................................uaggaacCucauaccgugcucu....................  102      1  HS5
.....................................................................Aaggaacuucauaccgugcucu....................  539      1  HS5
.....................................................................uaggaacuucauaccguCcucu....................  116      1  HS5
.....................................................................uaggaacuucauaccgugcucu....................  1272965  0  HS5
.....................................................................uaggaacuucauaccAugcucu....................  1435     1  HS5
.....................................................................uaggaacuuAauaccgugcucu....................  33       1  HS5
.....................................................................uaggaacuucauaccguUcucu....................  157      1  HS5
.....................................................................uaggaacuucauaccgugcucC....................  1543     1  HS5
.....................................................................uaggaacuucauacUgugcucu....................  577      1  HS5
.....................................................................uaggaacuucauUccgugcucu....................  203      1  HS5
.....................................................................uaggaaGuucauaccgugcucu....................  17       1  HS5
.....................................................................uaggaacuucauaccgugAucu....................  51       1  HS5
.....................................................................uaggaacuGcauaccgugcucu....................  14       1  HS5
.....................................................................uaggaacuCcauaccgugcucu....................  280      1  HS5
.....................................................................Gaggaacuucauaccgugcucu....................  489      1  HS5
.....................................................................Caggaacuucauaccgugcucu....................  258      1  HS5
.....................................................................uaggaacuucauaccgugcucG....................  91       1  HS5
.....................................................................uaggaCcuucauaccgugcucu....................  107      1  HS5
.....................................................................uaggaaUuucauaccgugcucu....................  268      1  HS5
.....................................................................uaggaacuucauacAgugcucu....................  40       1  HS5
.....................................................................uaggaacuucauaccCugcucu....................  320      1  HS5
.....................................................................uaggaacuucauCccgugcucu....................  979      1  HS5
.....................................................................uaggaacuucauaccgugcuUuu...................  5        1  HS5
.....................................................................uaggaaAuucauaccgugcucuu...................  1        1  HS5
.....................................................................Gaggaacuucauaccgugcucuu...................  2        1  HS5
.....................................................................uaggaacuucaGaccgugcucuu...................  7        1  HS5
.....................................................................uaggCacuucauaccgugcucuu...................  1        1  HS5
```

Star Mature

caaaaaaaaucgaauuacacuucgucacgagcgagguuuguaguuccuaccguuuuuauucaaacuggguaggaacuucauaccgugcucuugaacgaugcugacgaucaa			
....uaggaacuucauaccgAgcucuu...................	5	1	HS5
....uaggaacuucaCaccgugcucuu...................	4	1	HS5
....uaggaacuucauaccCugcucuu...................	4	1	HS5
....Aaggaacuucauaccgugcucuu...................	1	1	HS5
....uaggaacuucauaAcgugcucuu...................	1	1	HS5
....uaggaacuucauaUcgugcucuu...................	1	1	HS5
....uaUgaacuucauaccgugcucuu...................	1	1	HS5
....uaggaacuucauaccguCcucuu...................	1	1	HS5
....uaggaacuucauaccguUcucuu...................	1	1	HS5
....uaggaacuucauaccgugcucuA...................	1102	1	HS5
....uaggaacuucauaccgugcucAu...................	1	1	HS5
....uaggaCcuucauaccgugcucuu...................	2	1	HS5
....uaggaacuucauaccgugcucCu...................	15	1	HS5
....uaggaacuucauaccAugcucuu...................	8	1	HS5
....uaggaacuucauaccgugcucuG...................	9	1	HS5
....uaggaUcuucauaccgugcucuu...................	1	1	HS5
....uaggaacuucauaccgugcucuu...................	6253	0	HS5
....uaggGacuucauaccgugcucuu...................	2	1	HS5
....uaggaacuucauaccgGgcucuu...................	1	1	HS5
....Caggaacuucauaccgugcucuu...................	2	1	HS5
....uaggaacuucauaccgugcucuC...................	25	1	HS5
....uagUaacuucauaccgugcucuu...................	2	1	HS5
....uaggaacuucauaccguAcucuu...................	4	1	HS5
....uaggaacuucauaccgCgcucuu...................	2	1	HS5
....uaggaacuucauacUgugcucuu...................	2	1	HS5
....uaggaacuucauUccgugcucuu...................	1	1	HS5
....uaggaacuuUauaccgugcucuu...................	1	1	HS5
....uGggaacuucauaccgugcucuu...................	1	1	HS5
....uaggaacuCcauaccgugcucuu...................	1	1	HS5
....uaggUacuucauaccgugcucuu...................	4	1	HS5
....uaggaacuucauaccgugUucuu...................	6	1	HS5
....uaggaacuucauaccUugcucuu...................	2	1	HS5
....uaggaacuucauCccgugcucuu...................	3	1	HS5
....uaggaacuucauaccgugcucuuU..................	108	1	HS5
....uaggaacuucauaccgugcucuug..................	6	0	HS5
....uaggaacuucauaccgugcucuuC..................	10	1	HS5
....uaggaacuucauaccgugcucuAg..................	1	1	HS5
....uaggaacuucaCaccgugcucuug..................	1	1	HS5
....uaggaacuucauaccgugcucuGg..................	3	1	HS5
....uaggaacuucauaccgugcucuCg..................	4	1	HS5
....uaggaacuucauaccgugcucuuA..................	18	1	HS5
....uaggaacuucauaccgugcucuugU.................	1	1	HS5
....uaggaacuucauaccgugcucuGgaa................	1	1	HS5
....uaggaacuucauaccgugcucuuUaa................	1	1	HS5
....aggaacuucauaccgugcu......................	7	0	HS5
....aggaacuCcauaccgugcuc.....................	1	1	HS5
....aggaacuucauaccgugcuU.....................	1	1	HS5
....aggaacuucauaccgugcuc.....................	69	0	HS5
....aggaacuucauaccgugcCcu....................	1	1	HS5
....aggaacuucauaUcgugcucu....................	1	1	HS5
....aggaacuucauCccgugcucu....................	2	1	HS5
....aggaacuucauaccAugcucu....................	3	1	HS5
....aggaacuuUauaccgugcucu....................	1	1	HS5
....Uggaacuucauaccgugcucu....................	5	1	HS5
....aggaacuucauacUgugcucu....................	2	1	HS5
....aggaacuucauacAgugcucu....................	1	1	HS5
....aggaUcuucauaccgugcucu....................	1	1	HS5
....aggGacuucauaccgugcucu....................	2	1	HS5
....aggaaUuucauaccgugcucu....................	1	1	HS5
....aggaacuucauaccgugcucC....................	7	1	HS5
....aggaacuucauaccgugcGcu....................	1	1	HS5
....aggaacuucauGccgugcucu....................	1	1	HS5
....aAgaacuucauaccgugcucu....................	3	1	HS5
....agCaacuucauaccgugcucu....................	5	1	HS5
....aggaacuucauaccgugUucu....................	4	1	HS5
....aUgaacuucauaccgugcucu....................	40	1	HS5
....aggaacuucauaccgGgcucu....................	1	1	HS5
....aggaacuucauaccgCgcucu....................	1	1	HS5
....aggaacuCcauaccgugcucu....................	2	1	HS5
....Gggaacuucauaccgugcucu....................	7	1	HS5

```
                             Star                                      Mature
caaaaaaaaucgaauuacacuucgucacgagcgagguuuguaguuccuaccguuuuuauucaaacuggguaggaacuucauaccgugcucuugaacgaugcugacgaucaa
......................................................................aCgaacuucauaccgugcucu....................   34     1   HS5
......................................................................aggaacuucauaccgugcucA....................   1      1   HS5
......................................................................aggaacuucCuaccgugcucu....................   2      1   HS5
......................................................................aggaacuucauaccgAgcucu....................   6      1   HS5
......................................................................aggaacuucauaccgugcucu....................   3767   0   HS5
......................................................................aggaacuucauaccgugcucuu...................   19     0   HS5
......................................................................aUgaacuucauaccgugcucuu...................   1      1   HS5
......................................................................aggaacuucauaccgugcucuA...................   5      1   HS5
.......................................................................Agaacuucauaccgugcu......................   1      1   HS5
.......................................................................Agaacuucauaccgugcuc.....................   2      1   HS5
.......................................................................ggaacuucauaccgugcuc.....................   4      0   HS5
.......................................................................ggaacuucauaccguUcucu....................   1      1   HS5
.......................................................................ggaacuucauaccgGgcucu....................   1      1   HS5
.......................................................................Agaacuucauaccgugcucu....................   22     1   HS5
.......................................................................ggaacuucauaAcgugcucu....................   1      1   HS5
.......................................................................ggaacuucauaccgugcucu....................   385    0   HS5
.......................................................................ggGacuucauaccgugcucu....................   1      1   HS5
.......................................................................Ugaacuucauaccgugcucu....................   91     1   HS5
.......................................................................gUaacuucauaccgugcucu....................   1      1   HS5
.......................................................................ggaacuucauaccguAcucu....................   1      1   HS5
.......................................................................ggaacCucauaccgugcucu....................   1      1   HS5
.......................................................................ggaacuCcauaccgugcucu....................   1      1   HS5
.......................................................................ggaacuucauaccgugcucuA...................   1      1   HS5
.......................................................................ggaacuucauaccgugcucuu...................   2      0   HS5
.......................................................................ggaacuucauaccgugcucuuA..................   2      1   HS5
........................................................................Uaacuucauaccgugcuc.....................   2      1   HS5
........................................................................gaacuucauUccgugcucu....................   1      1   HS5
........................................................................gaacuucauaccgugcucu....................   44     0   HS5
........................................................................Uaacuucauaccgugcucu....................   30     1   HS5
........................................................................Aaacuucauaccgugcucu....................   2      1   HS5
........................................................................gaacuucauaccgugcucuu...................   1      0   HS5
.........................................................................aacuucauaccgugcucu....................   368    0   HS5
.........................................................................aacuucCuaccgugcucu....................   1      1   HS5
.........................................................................aacuucauaccgugcucC....................   1      1   HS5
.........................................................................aacuucauaccgugcuUu....................   1      1   HS5
.........................................................................aacuucauaccguUcucu....................   1      1   HS5
.........................................................................aacuucauaccgugUucu....................   1      1   HS5
.........................................................................aacuucauaccgugcucuu...................   2      0   HS5
..........................................................................acuucauaccgugcucuu...................   5      0   HS5

............................Uagcgagguuuguaguuccuacc............................................................   1      1   HS7
............................Cagcgagguuuguaguuccuacc............................................................   1      1   HS7
.............................agcgagguuuguaguucc................................................................   11     0   HS7
.............................agcgagCuuuguaguuccu...............................................................   1      1   HS7
.............................agcgagguuuguaguuccu...............................................................   294    0   HS7
.............................agUgagguuuguaguuccu...............................................................   1      1   HS7
.............................agcgagguuuguaguuccC...............................................................   1      1   HS7
.............................agcgagguuuguaguuccG...............................................................   1      1   HS7
.............................agcUagguuuguaguuccu...............................................................   1      1   HS7
.............................aUcgagguuuguaguuccua..............................................................   1      1   HS7
.............................Ugcgagguuuguaguuccua..............................................................   1      1   HS7
.............................agcgaggGuuguaguuccua..............................................................   2      1   HS7
.............................agcgagguuuguaguucUua..............................................................   1      1   HS7
.............................agcgagguuuguaguuccua..............................................................   616    0   HS7
.............................agcCagguuuguaguuccuac.............................................................   1      1   HS7
.............................agcgagguuuguGguuccuac.............................................................   1      1   HS7
.............................Ugcgagguuuguaguuccuac.............................................................   1      1   HS7
.............................agcgagUuuuguaguuccuac.............................................................   1      1   HS7
.............................agUgagguuuguaguuccuac.............................................................   3      1   HS7
.............................agcgagguuuguaguuccuaA.............................................................   1      1   HS7
.............................agcgaggGuuguaguuccuac.............................................................   6      1   HS7
.............................agcgagguuuguaUuuccuac.............................................................   1      1   HS7
.............................aUcgagguuuguaguuccuac.............................................................   2      1   HS7
.............................agcgagguuuguaguuccuGc.............................................................   1      1   HS7
.............................agcgagguuuguaguuccuaU.............................................................   8      1   HS7
.............................agcgagguuuguaguucUuac.............................................................   6      1   HS7
.............................agcgagguuuguaguuUcuac.............................................................   1      1   HS7
.............................agcgagguuuguaguuccuac.............................................................   1497   0   HS7
.............................agcgGgguuuguaguuccuac.............................................................   1      1   HS7
.............................agcgagguuuguaguuccCacc............................................................   1      1   HS7
```

```
                             Star                                   Mature
caaaaaaaaucgaauuacacuucgucacgagcgagguuuguaguuccuaccguuuuuauucaaacuggguaggaacuucauaccgugcucuugaacgaugcugacgaucaa
.............................agcgUgguuuguaguuccuacc............................................................   1      1   HS7
.............................agcgagguuuguaguuccuGcc............................................................   2      1   HS7
.............................agUgagguuuguaguuccuacc............................................................   12     1   HS7
.............................agcgagguuuguaguucUuacc............................................................   2      1   HS7
.............................agcgagguuuguaguuccGacc............................................................   2      1   HS7
.............................agcgagguuuAuaguuccuacc............................................................   1      1   HS7
.............................agcgagguuugCaguuccuacc............................................................   1      1   HS7
.............................Ugcgagguuuguaguuccuacc............................................................   1      1   HS7
.............................agcgaggCuuguaguuccuacc............................................................   2      1   HS7
.............................aUcgagguuuguaguuccuacc............................................................   3      1   HS7
.............................agcgGgguuuguaguuccuacc............................................................   2      1   HS7
.............................agcgagguuuguaguuUcuacc............................................................   9      1   HS7
.............................agcgaUguuuguaguuccuacc............................................................   2      1   HS7
.............................agcgagguuuguaguuccuacU............................................................   425    1   HS7
.............................agcgagguuuguagCuccuacc............................................................   1      1   HS7
.............................agcgaggGuuguaguuccuacc............................................................   15     1   HS7
.............................agcgagguuuguaguuccuacc............................................................   4552   0   HS7
.............................agcgagguuuguaguCccuacc............................................................   1      1   HS7
.............................agcgagguuuguUguuccuacc............................................................   1      1   HS7
.............................agcgagguuuguaguuccuacA............................................................   140    1   HS7
.............................agcgagguuuUuaguuccuacc............................................................   1      1   HS7
.............................agcgagguuuguaguuccuaccC...........................................................   5      1   HS7
.............................agcgagguuuguaguuccuaccg...........................................................   98     0   HS7
.............................agcgagguuuguaguuccuaccU...........................................................   470    1   HS7
.............................agcgaggGuuguaguuccuaccg...........................................................   1      1   HS7
.............................agcgagguuuguaguuccuaccA...........................................................   31     1   HS7
.............................agcgagguuuguaguuccuaccgA..........................................................   1      1   HS7
.............................agcgagguuuguaguuccuaccUu..........................................................   4      1   HS7
.............................agcgagguuuguaguuccuaccgC..........................................................   1      1   HS7
.............................agcgagguuuguaguuccuaccUuu.........................................................   2      1   HS7
..............................gcgagguuuguaguuccuac.............................................................   5      0   HS7
..............................gcgagguuuguaguuccuacU............................................................   1      1   HS7
..............................gcgagguuuguaguuccuacc............................................................   27     0   HS7
..............................gcgagguuuguaguuccuacA............................................................   3      1   HS7
..............................gcgagguuuguaguuccuaccg...........................................................   16     0   HS7
...............................cgagguuuguaguuccua..............................................................   1      0   HS7
...............................cgagguuuguaguuccuac.............................................................   1      0   HS7
...............................cgagguuuguaguuccuacc............................................................   1      0   HS7
...............................cgagguuuguaguuccuaccg...........................................................   6      0   HS7
...............................cgagguuuguaguuccuaccC...........................................................   2      1   HS7
................................gagguuuguaguuccuac.............................................................   1      0   HS7
................................gagguuuguaguuccuacc............................................................   11     0   HS7
................................gagguuuguaguuccuaccU...........................................................   1      1   HS7
.................................agguuuguaguuccuacc............................................................   1      0   HS7
...................................................guuuuuauucaaacuggg..........................................   5      0   HS7
.................................................................Gggguaggaacuucauaccgugcucu....................   2      1   HS7
..................................................................ggUuaggaacuucauaccgugcuc.....................   1      1   HS7
..................................................................Ugguaggaacuucauaccgugcuc.....................   1      1   HS7
..................................................................Cgguaggaacuucauaccgugcuc.....................   1      1   HS7
..................................................................ggCuaggaacuucauaccgugcucu....................   5      1   HS7
..................................................................ggAuaggaacuucauaccgugcucu....................   3      1   HS7
..................................................................gAguaggaacuucauaccgugcucu....................   2      1   HS7
..................................................................ggUuaggaacuucauaccgugcucu....................   1      1   HS7
..................................................................Agguaggaacuucauaccgugcucu....................   2      1   HS7
..................................................................ggguaggaacuucauaccgugcucu....................   7      0   HS7
..................................................................ggCuaggaacuucauaccgugcucuu...................   1      1   HS7
...................................................................gCuaggaacuucauaccgugcucu....................   3      1   HS7
....................................................................guaggaacuucauaccgug........................   1      0   HS7
....................................................................guaggaacuucauaccgugc.......................   2      0   HS7
....................................................................guaggaacuucauaccgugcu......................   18     0   HS7
....................................................................Uuaggaacuucauaccgugcu......................   1      1   HS7
....................................................................Uuaggaacuucauaccgugcuc.....................   11     1   HS7
....................................................................guaggaacuucauaccgugcuc.....................   30     0   HS7
....................................................................Auaggaacuucauaccgugcucu....................   5      1   HS7
....................................................................guaggaacuucauaccgugcucu....................   69     0   HS7
....................................................................Uuaggaacuucauaccgugcucu....................   358    1   HS7
....................................................................Cuaggaacuucauaccgugcucu....................   16     1   HS7
....................................................................Uuaggaacuucauaccgugcucuu...................   3      1   HS7
....................................................................Cuaggaacuucauaccgugcucuu...................   1      1   HS7
.....................................................................uaggaacuucauaccgug........................   237    0   HS7
```

Star **Mature**

caaaaaaaaucgaauuacacuucgucacgagcgagguuuguaguuccuaccguuuuuauucaaacugggtuaggaacuucauaccgugcucuugaacgaugcugacgaucaa			
....uagUaacuucauaccgug........................	1	1	HS7
....uaggaacuucauaccgGg........................	1	1	HS7
....uaggaacuucauaccguU........................	2	1	HS7
....uaggaacuucauaccgugc.......................	100	0	HS7
....uaggaacuucauaccgugU.......................	1	1	HS7
....uaggaacuucauaccAugc.......................	1	1	HS7
....uaggaacuucauaccgugA.......................	25	1	HS7
....uaggaacuucauaccAugcu......................	1	1	HS7
....uaggaacuucauacUgugcu......................	1	1	HS7
....uaggaacuucauaccguUcu......................	10	1	HS7
....uaggaacuucauaccguAcu......................	1	1	HS7
....uaggaGcuucauaccgugcu......................	1	1	HS7
....uaggaacuucauaccgugcC......................	2	1	HS7
....uaggaacuucauaccguCcu......................	19	1	HS7
....Caggaacuucauaccgugcu......................	1	1	HS7
....uaggCacuucauaccgugcu......................	1	1	HS7
....uaggaacuucauaccgugcu......................	1674	0	HS7
....uaggaacuucauaccgugcG......................	1	1	HS7
....uaggaacuuUauaccgugcu......................	1	1	HS7
....uagUaacuucauaccgugcu......................	4	1	HS7
....uaggaacGucauaccgugcu......................	1	1	HS7
....uaggaacuucauaUcgugcu......................	1	1	HS7
....Gaggaacuucauaccgugcu......................	1	1	HS7
....uaggaacCucauaccgugcuc.....................	3	1	HS7
....uaggaacuucauaccgugcAc.....................	1	1	HS7
....uaUgaacuucauaccgugcuc.....................	9	1	HS7
....uaggaaAuucauaccgugcuc.....................	1	1	HS7
....uUggaacuucauaccgugcuc.....................	1	1	HS7
....uaCgaacuucauaccgugcuc.....................	7	1	HS7
....uaggaacuucauaccguAcuc.....................	7	1	HS7
....uaggaacuucauaccgugUuc.....................	9	1	HS7
....uaggaacuucauacUgugcuc.....................	2	1	HS7
....uaggaacuucauaccUugcuc.....................	1	1	HS7
....uagUaacuucauaccgugcuc.....................	5	1	HS7
....uaggaacuucauaccgugcCc.....................	9	1	HS7
....uaggaacuucauaccguUcuc.....................	3	1	HS7
....uaAgaacuucauaccgugcuc.....................	1	1	HS7
....uaggaacuucauaccgugcGc.....................	2	1	HS7
....uaggaacuucauaccgugcuG.....................	4	1	HS7
....uaggaacuucGuaccgugcuc.....................	1	1	HS7
....uaggaacuucauaccgCgcuc.....................	3	1	HS7
....uaggaacuucauaccgugcuc.....................	14264	0	HS7
....uagCaacuucauaccgugcuc.....................	5	1	HS7
....uaggaacuucauaccgugcuU.....................	200	1	HS7
....uaggaacuuGauaccgugcuc.....................	1	1	HS7
....uaggaacuucaCaccgugcuc.....................	2	1	HS7
....uaggaacAucauaccgugcuc.....................	1	1	HS7
....uaggaacuucauaUcgugcuc.....................	6	1	HS7
....uagAaacuucauaccgugcuc.....................	2	1	HS7
....uaggaacuucCuaccgugcuc.....................	1	1	HS7
....uaggaacGucauaccgugcuc.....................	2	1	HS7
....uGggaacuucauaccgugcuc.....................	2	1	HS7
....uaggGacuucauaccgugcuc.....................	4	1	HS7
....uaggaacuucauaccgGgcuc.....................	1	1	HS7
....uaggaacuuUauaccgugcuc.....................	2	1	HS7
....uaggaacuCcauaccgugcuc.....................	1	1	HS7
....uaggaacuucauaccAugcuc.....................	4	1	HS7
....uaggaacuucauaccgugcuA.....................	32	1	HS7
....uaggUacuucauaccgugcuc.....................	5	1	HS7
....uaggaacuuAauaccgugcucu....................	3	1	HS7
....uaggaaGuucauaccgugcucu....................	85	1	HS7
....uaggaacuucauaccgugUucu....................	408	1	HS7
....uaggaacuucauaccgugcucC....................	1361	1	HS7
....uaggaacuucauaccguAcucu....................	127	1	HS7
....uaggaGcuucauaccgugcucu....................	35	1	HS7
....uaCgaacuucauaccgugcucu....................	238	1	HS7
....uaggaacuucauaccUugcucu....................	48	1	HS7
....uaggaacuucauaccgugcuGu....................	12	1	HS7
....uaggaacuucauaccgugcAcu....................	7	1	HS7
....uaggaacuucauaccgugGucu....................	61	1	HS7

Star **Mature**

```
caaaaaaaaucgaauuacacuucgucacgagcgagguuuguaguuccuaccguuuuuauucaaacuggguaggaacuucauaccgugcucuugaacgaugcugacgaucaa
.....................................................................uCggaacuucauaccgugcucu....................    7       1    HS7
.....................................................................uaggCacuucauaccgugcucu....................    74      1    HS7
.....................................................................uaggaacGucauaccgugcucu....................    110     1    HS7
.....................................................................uaggaacuucauaGcgugcucu....................    9       1    HS7
.....................................................................uaggaacuucauaccAugcucu....................    148     1    HS7
.....................................................................uaggaacuucauaccgCgcucu....................    61      1    HS7
.....................................................................uaggGacuucauaccgugcucu....................    262     1    HS7
.....................................................................uaggaacuucauaccgugcucu....................    761374  0    HS7
.....................................................................uaggaaUuucauaccgugcucu....................    125     1    HS7
.....................................................................uaggaacuucaAaccgugcucu....................    3       1    HS7
.....................................................................uaggUacuucauaccgugcucu....................    305     1    HS7
.....................................................................uagCaacuucauaccgugcucu....................    293     1    HS7
.....................................................................uaggaacuucauaccgugcuUu....................    118     1    HS7
.....................................................................uaggaacuucauacGgugcucu....................    16      1    HS7
.....................................................................uaggaUcuucauaccgugcucu....................    4       1    HS7
.....................................................................uaggaacuucUuaccgugcucu....................    15      1    HS7
.....................................................................uaggaacuucauCccgugcucu....................    4       1    HS7
.....................................................................Aaggaacuucauaccgugcucu....................    16      1    HS7
.....................................................................uaggaacCucauaccgugcucu....................    72      1    HS7
.....................................................................uaggaacAucauaccgugcucu....................    73      1    HS7
.....................................................................uagUaacuucauaccgugcucu....................    202     1    HS7
.....................................................................uGggaacuucauaccgugcucu....................    106     1    HS7
.....................................................................uaggaacuucaGaccgugcucu....................    7       1    HS7
.....................................................................uaggaacuucCuaccgugcucu....................    79      1    HS7
.....................................................................uaggaacuucauGccgugcucu....................    4       1    HS7
.....................................................................uagAaacuucauaccgugcucu....................    25      1    HS7
.....................................................................uaggaacuucauaccgugcGcu....................    12      1    HS7
.....................................................................uaUgaacuucauaccgugcucu....................    572     1    HS7
.....................................................................uUggaacuucauaccgugcucu....................    102     1    HS7
.....................................................................uaggaacuucauaccgAgcucu....................    12      1    HS7
.....................................................................uaggaacuCcauaccgugcucu....................    142     1    HS7
.....................................................................uaggaacuucauacUgugcucu....................    286     1    HS7
.....................................................................uaggaacuGcauaccgugcucu....................    12      1    HS7
.....................................................................Gaggaacuucauaccgugcucu....................    36      1    HS7
.....................................................................uaggaacuucauaccCugcucu....................    61      1    HS7
.....................................................................uaggaacuucauaccgGgcucu....................    40      1    HS7
.....................................................................uaggaacuucauaAcgugcucu....................    4       1    HS7
.....................................................................uaggaacuucauaUcgugcucu....................    223     1    HS7
.....................................................................uaggaacuucauaccgugAucu....................    8       1    HS7
.....................................................................uaggaacuucGuaccgugcucu....................    51      1    HS7
.....................................................................uaggaacuuGauaccgugcucu....................    16      1    HS7
.....................................................................uaggaaAuucauaccgugcucu....................    56      1    HS7
.....................................................................uaggaacuucauaccgugcuAu....................    17      1    HS7
.....................................................................uaggaacuucauaccgugcCcu....................    57      1    HS7
.....................................................................uaggaacuucauaccgugcucA....................    74      1    HS7
.....................................................................uaggaacuucauaccgugcucG....................    281     1    HS7
.....................................................................Caggaacuucauaccgugcucu....................    49      1    HS7
.....................................................................uaggaacuuUauaccgugcucu....................    248     1    HS7
.....................................................................uaggaacuucaCaccgugcucu....................    53      1    HS7
.....................................................................uaggaacuAcauaccgugcucu....................    28      1    HS7
.....................................................................uaggaacuucauaccguUcucu....................    133     1    HS7
.....................................................................uaggaacuucauaccguCcucu....................    56      1    HS7
.....................................................................uaggaacuucauacAgugcucu....................    7       1    HS7
.....................................................................uaAgaacuucauaccgugcucu....................    23      1    HS7
.....................................................................uaggaacuucauUccgugcucu....................    19      1    HS7
.....................................................................uaggaCcuucauaccgugcucu....................    8       1    HS7
.....................................................................uaggaacCucauaccgugcucuu...................    1       1    HS7
.....................................................................uaggaacuucauaccgugcucAu...................    3       1    HS7
.....................................................................uaggaacuucauaccgugcucuG...................    6       1    HS7
.....................................................................uaggaacuCcauaccgugcucuu...................    1       1    HS7
.....................................................................uaggaacuucCuaccgugcucuu...................    1       1    HS7
.....................................................................uagUaacuucauaccgugcucuu...................    1       1    HS7
.....................................................................uaggaacuucauaccgugcucuu...................    4430    0    HS7
.....................................................................uaggaacuucauaccgugcuUuu...................    2       1    HS7
.....................................................................uaggaacuucGuaccgugcucuu...................    1       1    HS7
.....................................................................uaggaacuucauaccgugcCcuu...................    2       1    HS7
.....................................................................uaggaacuucauaccguUcucuu...................    1       1    HS7
.....................................................................uaggaacuucauaccgugUucuu...................    4       1    HS7
.....................................................................uaggaacuucauaccAugcucuu...................    1       1    HS7
.....................................................................uaUgaacuucauaccgugcucuu...................    1       1    HS7
```

Star Mature

caaaaaaaaucgaauuacacuucgucacgagcgagguuuguaguuccuaccguuuuuauucaaacuggguaggaacuucauaccgugcucuugaacgaugcugacgaucaa			
....uaggaacAucauaccgugcucuu...................	1	1	HS7
....uagCaacuucauaccgugcucuu...................	3	1	HS7
....uaggaacuucauaccgugcucCu...................	16	1	HS7
....uaggaacuucauaccCugcucuu...................	1	1	HS7
....uaggaaGuucauaccgugcucuu...................	2	1	HS7
....uaggaacuucauaUcgugcucuu...................	1	1	HS7
....uaggUacuucauaccgugcucuu...................	1	1	HS7
....uaggaacuucauaccgugcucuC...................	13	1	HS7
....uaggCacuucauaccgugcucuu...................	1	1	HS7
....uaggaacuucauacGgugcucuu...................	1	1	HS7
....uaCgaacuucauaccgugcucuu...................	2	1	HS7
....uaggaaAuucauaccgugcucuu...................	1	1	HS7
....uaggaacuuUauaccgugcucuu...................	2	1	HS7
....uaggaacuucauaccgugcucuA...................	1050	1	HS7
....uaggaacuucauaccgugcucuug..................	5	0	HS7
....uaggaacuucauaccgugcucuuA..................	8	1	HS7
....uaggaacuucauaccgugcucuuU..................	43	1	HS7
....uaggaacuucauaccgugcucuAg..................	1	1	HS7
....uaggaacuucauaccgugcucuGg..................	1	1	HS7
....uaggaacuucauaccgugcucuuC..................	7	1	HS7
....uaggaacuucauaccgugcucuCg..................	1	1	HS7
....uaggaacuucauaccgugcucuugU.................	3	1	HS7
....uaggaacuucauaccgugcucuugG.................	1	1	HS7
....uaggaacuucauaccgugcucuGgaa................	2	1	HS7
....aggaacuucauaccgugc.......................	1	0	HS7
....aggaacuucauaccgugcu......................	6	0	HS7
....aggaacuucauaccgugcuU.....................	2	1	HS7
....aggaacuucauaccgugcuc.....................	23	0	HS7
....aUgaacuucauaccgugcuc.....................	2	1	HS7
....aggaacuucauaccgugGucu....................	2	1	HS7
....aggaacuucauaccgugcucu....................	1353	0	HS7
....aggaacuucUuaccgugcucu....................	1	1	HS7
....aggaUcuucauaccgugcucu....................	3	1	HS7
....aggaacuucauaccgugcucC....................	5	1	HS7
....Uggaacuucauaccgugcucu....................	3	1	HS7
....aAgaacuucauaccgugcucu....................	1	1	HS7
....agUaacuucauaccgugcucu....................	6	1	HS7
....aUgaacuucauaccgugcucu....................	17	1	HS7
....agCaacuucauaccgugcucu....................	2	1	HS7
....aggaaGuucauaccgugcucu....................	1	1	HS7
....aggGacuucauaccgugcucu....................	1	1	HS7
....aCgaacuucauaccgugcucu....................	12	1	HS7
....aggaacuucauaccguAcucu....................	1	1	HS7
....aggaGcuucauaccgugcucu....................	2	1	HS7
....Gggaacuucauaccgugcucu....................	5	1	HS7
....aggaacuucauaccgugcucuu...................	7	0	HS7
....aggaacuucauaccgugcucuA...................	2	1	HS7
....ggaacuucauaccgugcu......................	1	0	HS7
....Ugaacuucauaccgugcuc.....................	3	1	HS7
....ggaacuucauaccgugcuc.....................	3	0	HS7
....Agaacuucauaccgugcucu....................	9	1	HS7
....Cgaacuucauaccgugcucu....................	3	1	HS7
....gAaacuucauaccgugcucu....................	1	1	HS7
....gUaacuucauaccgugcucu....................	1	1	HS7
....ggaacuucauaccgugcucu....................	185	0	HS7
....Ugaacuucauaccgugcucu....................	43	1	HS7
....Ugaacuucauaccgugcucuu...................	1	1	HS7
....gaacuucauaccgugcucu....................	126	0	HS7
....gaUcuucauaccgugcucu....................	1	1	HS7
....Aaacuucauaccgugcucu....................	1	1	HS7
....Uaacuucauaccgugcucu....................	17	1	HS7
....gaacuucauaccgugcucuu...................	2	0	HS7
....aacuucauaccgugcucC....................	2	1	HS7
....Uacuucauaccgugcucu....................	1	1	HS7
....aacuucauaccgugcucu....................	957	0	HS7
....aGcuucauaccgugcucu....................	1	1	HS7
....aCcuucauaccgugcucu....................	1	1	HS7
....aacuucauCccgugcucu....................	1	1	HS7
....aaUuucauaccgugcucu....................	1	1	HS7
....aacuucauaccgugcucuu...................	3	0	HS7

```
                              Star                                       Mature
caaaaaaaaucgaauuacacuucgucacgagcgagguuuguaguuccuaccguuuuuauucaaacugggguaggaacuucauaccgugcucuugaacgaugcugacgaucaa
..........................................................................acuucauaccgugcucuu...................   4     0   HS7

.............................agcgagguuuguGguuccu...............................................................   1     1   HS2
.............................agcgagguuuguaguuccu...............................................................   128   0   HS2
.............................agcgagguuuguaguuccC...............................................................   1     1   HS2
.............................agcgagguuuguaguuccua..............................................................   44    0   HS2
.............................agUgagguuuguaguuccuac.............................................................   1     1   HS2
.............................agcgagguuuguaguuccCac.............................................................   1     1   HS2
.............................agcgagguuCguaguuccuac.............................................................   1     1   HS2
.............................agcgagguuuguaguuccuac.............................................................   3822  0   HS2
.............................Ugcgagguuuguaguuccuac.............................................................   1     1   HS2
.............................agcgagguuuguagCuccuac.............................................................   1     1   HS2
.............................agcgagguuuguaguCccuac.............................................................   1     1   HS2
.............................agcgaggAuuguaguuccuac.............................................................   1     1   HS2
.............................aUcgagguuuguaguuccuac.............................................................   1     1   HS2
.............................agAgagguuuguaguuccuac.............................................................   1     1   HS2
.............................agcgagguuuguaguuccuGc.............................................................   1     1   HS2
.............................agcgagguuugCaguuccuac.............................................................   1     1   HS2
.............................agcgagguuugGaguuccuac.............................................................   1     1   HS2
.............................agcgagguuuguaguuccuaU.............................................................   1     1   HS2
.............................aAcgagguuuguaguuccuacc............................................................   1     1   HS2
.............................Ggcgagguuuguaguuccuacc............................................................   1     1   HS2
.............................agcgagguuuguaguuccuacA............................................................   63    1   HS2
.............................agcgagguCuguaguuccuacc............................................................   1     1   HS2
.............................agcgagguuuguaguuccuacU............................................................   185   1   HS2
.............................agUgagguuuguaguuccuacc............................................................   1     1   HS2
.............................agcgagguuuguagCuccuacc............................................................   1     1   HS2
.............................agcgagguuuguaguucAuacc............................................................   1     1   HS2
.............................agcgagguuCguaguuccuacc............................................................   1     1   HS2
.............................agcgagguuuguaguuccGacc............................................................   1     1   HS2
.............................agcgagguuuguaguuccuacc............................................................   1703  0   HS2
.............................agcgagguuugCaguuccuacc............................................................   1     1   HS2
.............................agcgGgguuuguaguuccuacc............................................................   1     1   HS2
.............................agcgagguuuguaguuccuaccA...........................................................   166   1   HS2
..............................gcgagguuuguaguuccuac.............................................................   121   0   HS2
..............................Ucgagguuuguaguuccuacc............................................................   1     1   HS2
..............................gcgagguuuguaguuccuacU............................................................   33    1   HS2
..............................gcgagguuuguaguuccuacc............................................................   125   0   HS2
....................................................................guaggaacuucauaccgugcucu....................   27    0   HS2
....................................................................Cuaggaacuucauaccgugcucu....................   200   1   HS2
.....................................................................uaggaacuucauaccgug........................   84    0   HS2
.....................................................................uaggaacuucauaccgugcu......................   1     0   HS2
.....................................................................uaggaacuucauaccgugcuc.....................   2068  0   HS2
.....................................................................Gaggaacuucauaccgugcuc.....................   1     1   HS2
.....................................................................uaggaacuCcauaccgugcuc.....................   2     1   HS2
.....................................................................uaggGacuucauaccgugcuc.....................   1     1   HS2
.....................................................................uGggaacuucauaccgugcuc.....................   1     1   HS2
.....................................................................uagAaacuucauaccgugcuc.....................   1     1   HS2
.....................................................................uaggaacCucauaccgugcuc.....................   1     1   HS2
.....................................................................uaggaacuucaGaccgugcuc.....................   1     1   HS2
.....................................................................uaggaacCucauaccgugcucu....................   95    1   HS2
.....................................................................uaggaGcuucauaccgugcucu....................   74    1   HS2
.....................................................................Gaggaacuucauaccgugcucu....................   54    1   HS2
.....................................................................Caggaacuucauaccgugcucu....................   117   1   HS2
.....................................................................uagUaacuucauaccgugcucu....................   11    1   HS2
.....................................................................uaggaacuuUauaccgugcucu....................   45    1   HS2
.....................................................................uaAgaacuucauaccgugcucu....................   47    1   HS2
.....................................................................uaggaacuCcauaccgugcucu....................   180   1   HS2
.....................................................................Aaggaacuucauaccgugcucu....................   32    1   HS2
.....................................................................uaggaacuucauaccgugAucu....................   7     1   HS2
.....................................................................uagAaacuucauaccgugcucu....................   28    1   HS2
.....................................................................uaUgaacuucauaccgugcucu....................   10    1   HS2
.....................................................................uaggaacuucauaccgugGucu....................   4     1   HS2
.....................................................................uaggaacuucauaGcgugcucu....................   1     1   HS2
.....................................................................uaggaacuucauaccgugcuUu....................   17    1   HS2
.....................................................................uaggaacuucauaccgugUucu....................   109   1   HS2
.....................................................................uaCgaacuucauaccgugcucu....................   11    1   HS2
.....................................................................uaggaacuucauacGgugcucu....................   3     1   HS2
.....................................................................uaggaacuucaAaccgugcucu....................   7     1   HS2
.....................................................................uaggaacuucaGaccgugcucu....................   6     1   HS2
```

```
                              Star                                    Mature
caaaaaaaaucgaauuacacuucgucacgagcgagguuuguaguuccuaccguuuuuauucaaacuggguaggaacuucauaccgugcucuugaacgaugcugacgaucaa
.....................................................................uCggaacuucauaccgugcucu....................    4       1    HS2
.....................................................................uaggaaAuucauaccgugcucu....................    20      1    HS2
.....................................................................uaggaacuAcauaccgugcucu....................    10      1    HS2
.....................................................................uaggGacuucauaccgugcucu....................    73      1    HS2
.....................................................................uaggaacuucauUccgugcucu....................    1       1    HS2
.....................................................................uaggaacuGcauaccgugcucu....................    2       1    HS2
.....................................................................uGggaacuucauaccgugcucu....................    99      1    HS2
.....................................................................uaggaacuuAauaccgugcucu....................    50      1    HS2
.....................................................................uaggaacuucauaccgugcuGu....................    4       1    HS2
.....................................................................uaggaacuuGauaccgugcucu....................    7       1    HS2
.....................................................................uaggaacuucauaccguUcucu....................    23      1    HS2
.....................................................................uaggaacuucauaccguCcucu....................    4       1    HS2
.....................................................................uaggaacuucGuaccgugcucu....................    65      1    HS2
.....................................................................uaggaacuucauaccgugcAcu....................    19      1    HS2
.....................................................................uaggaacuucaCaccgugcucu....................    48      1    HS2
.....................................................................uaggaacAucauaccgugcucu....................    32      1    HS2
.....................................................................uaggaacuucauaccgGgcucu....................    15      1    HS2
.....................................................................uaggCacuucauaccgugcucu....................    2       1    HS2
.....................................................................uaggaacuucUuaccgugcucu....................    1       1    HS2
.....................................................................uaggaacuucauaccgugcCcu....................    130     1    HS2
.....................................................................uaggaacuucauaccgAgcucu....................    15      1    HS2
.....................................................................uaggaacuucauaccgugcucA....................    193     1    HS2
.....................................................................uaggaacuucauCccgugcucu....................    1       1    HS2
.....................................................................uaggaacuucauaccgugcGcu....................    48      1    HS2
.....................................................................uaggaCcuucauaccgugcucu....................    1       1    HS2
.....................................................................uaggaacuucauaccgugcuAu....................    12      1    HS2
.....................................................................uaggaacuucauaccCugcucu....................    4       1    HS2
.....................................................................uUggaacuucauaccgugcucu....................    7       1    HS2
.....................................................................uagCaacuucauaccgugcucu....................    2       1    HS2
.....................................................................uaggaacuucauacAgugcucu....................    11      1    HS2
.....................................................................uaggaaUuucauaccgugcucu....................    8       1    HS2
.....................................................................uaggaacuucauaccAugcucu....................    149     1    HS2
.....................................................................uaggaacuucauaccUugcucu....................    5       1    HS2
.....................................................................uaggaacuucauaUcgugcucu....................    52      1    HS2
.....................................................................uaggaacuucauaccgugcucu....................    356813  0    HS2
.....................................................................uaggaacuucauGccgugcucu....................    55      1    HS2
.....................................................................uaggaacuucauaAcgugcucu....................    11      1    HS2
.....................................................................uaggaacGucauaccgugcucu....................    10      1    HS2
.....................................................................uaggaacuucauaccguAcucu....................    515     1    HS2
.....................................................................uaggaacuucauacUgugcucu....................    16      1    HS2
.....................................................................uaggaaGuucauaccgugcucu....................    8       1    HS2
.....................................................................uaggaacuucauaccgCgcucu....................    53      1    HS2
.....................................................................uaggaacuucauaccgugcucG....................    84      1    HS2
.....................................................................uaggaacuucauaccgugcucC....................    440     1    HS2
.....................................................................uaggUacuucauaccgugcucu....................    6       1    HS2
.....................................................................uaggaacuucauaccgugcucuu...................    77      0    HS2
.....................................................................uaggaacuucauaccgugcucuA...................    2       1    HS2
.....................................................................uGggaacuucauaccgugcucuu...................    1       1    HS2
.....................................................................uaggaacuucauaccgugcucuAga.................    1       1    HS2
......................................................................aggaacuucauaccgugcCc.....................    1       1    HS2
......................................................................aggaacuCcauaccgugcuc.....................    2       1    HS2
......................................................................aggaacuucauaccgugcuc.....................    238     0    HS2
......................................................................Uggaacuucauaccgugcuc.....................    1       1    HS2
......................................................................Gggaacuucauaccgugcucu....................    3       1    HS2
......................................................................aggGacuucauaccgugcucu....................    9       1    HS2
......................................................................aUgaacuucauaccgugcucu....................    2       1    HS2
......................................................................aggaacuucauaccgugcucu....................    26472   0    HS2
......................................................................aggaacuucauaccgugGucu....................    2       1    HS2
......................................................................aggaacuuUauaccgugcucu....................    5       1    HS2
......................................................................aggaacuucauaccgugcucG....................    2       1    HS2
......................................................................aggaacAucauaccgugcucu....................    4       1    HS2
......................................................................aggaacuucGuaccgugcucu....................    2       1    HS2
......................................................................aggaacuucauaccguAcucu....................    52      1    HS2
......................................................................aggaaCucauaccgugcucu.....................    8       1    HS2
......................................................................aggaacuucauaccAugcucu....................    6       1    HS2
......................................................................agUaacuucauaccgugcucu....................    1       1    HS2
......................................................................aggaacuucauaccgugcGcu....................    11      1    HS2
......................................................................aggaacuucauaccgugcCcu....................    18      1    HS2
......................................................................agAaacuucauaccgugcucu....................    2       1    HS2
......................................................................aggaacuucauaccguUcucu....................    4       1    HS2
```

```
                             Star                                     Mature
caaaaaaaaucgaauuacacuucgucacgagcgagguuuguaguuccuaccguuuuuauucaaacuggguaggaacuucauaccgugcucuugaacgaugcugacgaucaa
......................................................................aggaacuucauaccgugUucu....................   3      1   HS2
......................................................................aggaGcuucauaccgugcucu....................   3      1   HS2
......................................................................aggaacuCcauaccgugcucu....................   6      1   HS2
......................................................................aggaacuucauGccgugcucu....................   4      1   HS2
......................................................................aggaacuucauaccgugcuAu....................   1      1   HS2
......................................................................Uggaacuucauaccgugcucu....................   11     1   HS2
......................................................................aggaacuucauaccgugcAcu....................   2      1   HS2
......................................................................aggaacuucauaUcgugcucu....................   3      1   HS2
......................................................................aAgaacuucauaccgugcucu....................   3      1   HS2
......................................................................aggaacGucauaccgugcucu....................   1      1   HS2
......................................................................aggaacuucauaccgugcuUu....................   1      1   HS2
......................................................................aggaacuucauaccgugcucA....................   12     1   HS2
......................................................................aggaacuucaCaccgugcucu....................   3      1   HS2
......................................................................aggUacuucauaccgugcucu....................   1      1   HS2
......................................................................aggaacuucauaccUugcucu....................   1      1   HS2
......................................................................aggaacuucauacAgugcucu....................   3      1   HS2
......................................................................aggaacuucauaAcgugcucu....................   3      1   HS2
......................................................................aggaacuucCuaccgugcucu....................   3      1   HS2
......................................................................aggaaGuucauaccgugcucu....................   1      1   HS2
......................................................................aggaacuGcauaccgugcucu....................   1      1   HS2
......................................................................aggaacuucauaccgCgcucu....................   6      1   HS2
......................................................................aggaacuucauaccgAgcucu....................   1      1   HS2
......................................................................aggaaAuucauaccgugcucu....................   1      1   HS2
......................................................................aggaacuucauaccgugcucC....................   37     1   HS2
......................................................................aggaacuucauacUgugcucu....................   3      1   HS2
......................................................................aggaacuucauaccCugcucu....................   18     1   HS2
......................................................................aggaacuucauaccgugcucuA...................   1      1   HS2
.......................................................................ggaacuucauaccgugcucu....................   170    0   HS2
.......................................................................ggaacuucauaccguAcucu....................   1      1   HS2

.........ucAaauuacacuucgucacg..................................................................................   2      1   HA1
..........cAaauuacacuucgucacg..................................................................................   1      1   HA1
..........................acgagcgagguuuguaguucc................................................................   1      0   HA1
..........................acgagcgagguuuguaguuccuacc............................................................   1      0   HA1
............................Uagcgagguuuguaguuccu...............................................................   1      1   HA1
............................Uagcgagguuuguaguuccua..............................................................   2      1   HA1
............................Cagcgagguuuguaguuccua..............................................................   2      1   HA1
............................Uagcgagguuuguaguuccuac.............................................................   12     1   HA1
............................Uagcgagguuuguaguuccuacc............................................................   4      1   HA1
.............................Cgcgagguuuguaguucc................................................................   1      1   HA1
.............................agcgagguuuguaguuAc................................................................   2      1   HA1
.............................agcgaggGuuguaguucc................................................................   1      1   HA1
.............................agcgagguuuguaguucc................................................................   85     0   HA1
.............................agcgagguuCguaguuccu...............................................................   2      1   HA1
.............................agUgagguuuguaguuccu...............................................................   10     1   HA1
.............................agcgagguuuguaAuuccu...............................................................   1      1   HA1
.............................agcUagguuuguaguuccu...............................................................   1      1   HA1
.............................agcgGgguuuguaguuccu...............................................................   1      1   HA1
.............................agcgagguuuguagCuccu...............................................................   1      1   HA1
.............................agcgagguuuguaguuccA...............................................................   20     1   HA1
.............................agcAagguuuguaguuccu...............................................................   3      1   HA1
.............................agcgagguuuguaguucUu...............................................................   1      1   HA1
.............................agcgagguuuguaguuAcu...............................................................   2      1   HA1
.............................aUcgagguuuguaguuccu...............................................................   2      1   HA1
.............................agcgagguGuguaguuccu...............................................................   1      1   HA1
.............................Ugcgagguuuguaguuccu...............................................................   1      1   HA1
.............................agcgagguCuguaguuccu...............................................................   1      1   HA1
.............................agcgaAguuuguaguuccu...............................................................   1      1   HA1
.............................agcgagCuuuguaguuccu...............................................................   2      1   HA1
.............................agcgagguuuguaguuccG...............................................................   5      1   HA1
.............................agcgagguuuguGguuccu...............................................................   1      1   HA1
.............................agcgaggGuuguaguuccu...............................................................   8      1   HA1
.............................agcgagguuuguaguGccu...............................................................   2      1   HA1
.............................agcgagguuuguagAuccu...............................................................   1      1   HA1
.............................agcgagguuuguaguuccu...............................................................   4119   0   HA1
.............................agcgagguuuUuaguuccu...............................................................   1      1   HA1
.............................agcgagguuuCuaguuccu...............................................................   1      1   HA1
.............................agcgaCguuuguaguuccu...............................................................   1      1   HA1
.............................agcgagguuuguaguuccC...............................................................   5      1   HA1
.............................agcgaggCuuguaguuccu...............................................................   1      1   HA1
```

Star Mature

caaaaaaaaucgaauuacacuucgucacgagcgagguuuguaguuccuaccguuuuuauucaaacuggguaggaacuucauaccgugcucuugaacgaugcugacgaucaa			
.............................agcgagguuuguUguuccu...	7	1	HA1
.............................Ggcgagguuuguaguuccu...	3	1	HA1
.............................agGgagguuuguaguuccua..	1	1	HA1
.............................agcgagguuugAaguuccua..	1	1	HA1
.............................agcgGgguuuguaguuccua..	5	1	HA1
.............................agcgagguGuguaguuccua..	1	1	HA1
.............................agcgagguuuguaguuccuG..	2	1	HA1
.............................Ggcgagguuuguaguuccua..	10	1	HA1
.............................agcgagguuugGaguuccua..	1	1	HA1
.............................agcgagguuuguUguuccua..	8	1	HA1
.............................agUgagguuuguaguuccua..	21	1	HA1
.............................agcgagguuuguaguuAcua..	5	1	HA1
.............................agcgagguuuguaguuccuU..	37	1	HA1
.............................agcgaggAuuguaguuccua..	1	1	HA1
.............................agcgagCuuuguaguuccua..	4	1	HA1
.............................agcgagguuuAuaguuccua..	2	1	HA1
.............................agcgagguuugCaguuccua..	2	1	HA1
.............................Ugcgagguuuguaguuccua..	2	1	HA1
.............................agcgagguuuguaguuccua..	10827	0	HA1
.............................aCcgagguuuguaguuccua..	1	1	HA1
.............................agcgagguuGguaguuccua..	1	1	HA1
.............................agcgagguuuguaguuccAa..	4	1	HA1
.............................agcgagguCuguaguuccua..	3	1	HA1
.............................agcgagguuuguaguuUcua..	2	1	HA1
.............................agcgagguuuguaguuccCa..	14	1	HA1
.............................agcgaggGuuguaguuccua..	26	1	HA1
.............................agcgagguuuguaCuuccua..	1	1	HA1
.............................agcgagguuuguGguuccua..	3	1	HA1
.............................agcgagguuuguaUuuccua..	1	1	HA1
.............................agcCagguuuguaguuccua..	2	1	HA1
.............................agcgUgguuuguaguuccua..	2	1	HA1
.............................agcgagguuuguagGuccua..	1	1	HA1
.............................Cgcgagguuuguaguuccua..	3	1	HA1
.............................aAcgagguuuguaguuccua..	3	1	HA1
.............................agcgagguuuguaguCccua..	5	1	HA1
.............................agcgaUguuuguaguuccua..	2	1	HA1
.............................agcgaggCuuguaguuccua..	1	1	HA1
.............................agcgaCguuuguaguuccua..	1	1	HA1
.............................agcgagAuuuguaguuccua..	5	1	HA1
.............................agcgagguuuCuaguuccua..	1	1	HA1
.............................agcgCgguuuguaguuccua..	4	1	HA1
.............................agcgagguuuguaguuccGa..	1	1	HA1
.............................agcgagguuuguaAuuccua..	2	1	HA1
.............................agcgagguAuguaguuccua..	2	1	HA1
.............................aUcgagguuuguaguuccua..	1	1	HA1
.............................agcgagUuuuguaguuccua..	4	1	HA1
.............................agcgagguuuguagCuccua..	1	1	HA1
.............................agcgagguuuguagAuccua..	1	1	HA1
.............................agcgagguuuguCguuccua..	1	1	HA1
.............................Ggcgagguuuguaguuccuac...	3	1	HA1
.............................agcgaggGuuguaguuccuac...	20	1	HA1
.............................agcgagguuuguGguuccuac...	2	1	HA1
.............................agcgagguuuguaguuccuCc...	4	1	HA1
.............................agcgagguAuguaguuccuac...	1	1	HA1
.............................agcgagguuuguaguuUcuac...	16	1	HA1
.............................Cgcgagguuuguaguuccuac...	5	1	HA1
.............................aAcgagguuuguaguuccuac...	1	1	HA1
.............................agcgagguuuguaguuccCac...	1	1	HA1
.............................agcgagguuuguaguuccAac...	1	1	HA1
.............................agcUagguuuguaguuccuac...	1	1	HA1
.............................agcgagguCuguaguuccuac...	2	1	HA1
.............................aCcgagguuuguaguuccuac...	1	1	HA1
.............................agcgagguuuguaguuccuaG...	2	1	HA1
.............................agcAagguuuguaguuccuac...	1	1	HA1
.............................agcgagguuuguaAuuccuac...	1	1	HA1
.............................agcgagCuuuguaguuccuac...	1	1	HA1
.............................agcgagguuuguaguuccuaA...	23	1	HA1
.............................agcgagguuuguUguuccuac...	5	1	HA1
.............................agcgCgguuuguaguuccuac...	1	1	HA1
.............................agcgGgguuuguaguuccuac...	3	1	HA1

```
                            Star                                   Mature
caaaaaaaaucgaauuacacuucgucacgagcgagguuuguaguuccuaccguuuuuauucaaacuggguaggaacuucauaccgugcucuugaacgaugcugacgaucaa
.............................agcgagguuuguaguuccuac.............................................................   5190   0   HA1
.............................agcgagguuuguagCuccuac.............................................................   2      1   HA1
.............................agUgagguuuguaguuccuac.............................................................   12     1   HA1
.............................agcgagguuuguaguuccuGc.............................................................   11     1   HA1
.............................agcgaggAuuguaguuccuac.............................................................   1      1   HA1
.............................agcgagguuuguagGuccuac.............................................................   1      1   HA1
.............................agcgagguGuguaguuccuac.............................................................   2      1   HA1
.............................Ugcgagguuuguaguuccuac.............................................................   2      1   HA1
.............................agcgagguuCguaguuccuac.............................................................   1      1   HA1
.............................agcgaCguuuguaguuccuac.............................................................   1      1   HA1
.............................agcgagguuuguaguuccuaU.............................................................   117    1   HA1
.............................agcgagguuuguCguuccuac.............................................................   1      1   HA1
.............................agcgaggCuuguaguuccuac.............................................................   1      1   HA1
.............................agcgagguuuguaguCccuac.............................................................   2      1   HA1
.............................agcgagguuuguaguucUuac.............................................................   10     1   HA1
.............................agcgagguuuguaguuccuUc.............................................................   1      1   HA1
.............................agcgagguuugCaguuccuac.............................................................   2      1   HA1
.............................agcgagguuuguagAuccuacc............................................................   1      1   HA1
.............................agcgagCuuuguaguuccuacc............................................................   3      1   HA1
.............................agcgagguuuguGguuccuacc............................................................   1      1   HA1
.............................agcgCgguuuguaguuccuacc............................................................   1      1   HA1
.............................agcgagguuuguUguuccuacc............................................................   9      1   HA1
.............................agcgagguuuguaCuuccuacc............................................................   1      1   HA1
.............................agcgagguuuguagGuccuacc............................................................   1      1   HA1
.............................agcgagguCuguaguuccuacc............................................................   1      1   HA1
.............................agcgagguuuguCguuccuacc............................................................   1      1   HA1
.............................Ggcgagguuuguaguuccuacc............................................................   6      1   HA1
.............................agcgGgguuuguaguuccuacc............................................................   1      1   HA1
.............................agcgagguuuguaguuUcuacc............................................................   15     1   HA1
.............................agcgagguuuguaguuccuaAc............................................................   1      1   HA1
.............................agcgaAguuuguaguuccuacc............................................................   1      1   HA1
.............................agcAagguuuguaguuccuacc............................................................   1      1   HA1
.............................agcgagguuuguaguCccuacc............................................................   1      1   HA1
.............................agcgagguuCguaguuccuacc............................................................   1      1   HA1
.............................agcgagguuuguaguuccuCcc............................................................   9      1   HA1
.............................agcgagguuuguaguucGuacc............................................................   1      1   HA1
.............................agcgaggGuuguaguuccuacc............................................................   23     1   HA1
.............................agcgagguuuguaguucUuacc............................................................   3      1   HA1
.............................agcUagguuuguaguuccuacc............................................................   1      1   HA1
.............................agcgagguuuguaguuccuacc............................................................   7013   0   HA1
.............................agcgagguuuguaguuccCacc............................................................   4      1   HA1
.............................agcgagguuuguaguuccuUcc............................................................   4      1   HA1
.............................agcgagguuuguaguuccuacG............................................................   46     1   HA1
.............................agUgagguuuguaguuccuacc............................................................   14     1   HA1
.............................aCcgagguuuguaguuccuacc............................................................   1      1   HA1
.............................Ugcgagguuuguaguuccuacc............................................................   4      1   HA1
.............................agcgagAuuuguaguuccuacc............................................................   1      1   HA1
.............................agcgagguuugCaguuccuacc............................................................   2      1   HA1
.............................agcgagguuuguaguuccGacc............................................................   1      1   HA1
.............................agcgagguuuguaguuccuaUc............................................................   1      1   HA1
.............................agcgagUuuuguaguuccuacc............................................................   3      1   HA1
.............................agcgagguuGguaguuccuacc............................................................   1      1   HA1
.............................aUcgagguuuguaguuccuacc............................................................   2      1   HA1
.............................agcgagguuuguaguuccuacA............................................................   1638   1   HA1
.............................agcCagguuuguaguuccuacc............................................................   1      1   HA1
.............................Cgcgagguuuguaguuccuacc............................................................   7      1   HA1
.............................agcgagguuuguaguuccuGcc............................................................   13     1   HA1
.............................agcgagguuuguaguuccuacU............................................................   1411   1   HA1
.............................agcgagguuuguaguuccuaccC...........................................................   9      1   HA1
.............................agcgagguuuguaguuccuaccA...........................................................   344    1   HA1
.............................agcgagguuuguaguucUuaccg...........................................................   1      1   HA1
.............................agcgagguuuguaguuccuaccU...........................................................   549    1   HA1
.............................agcgagguuuguaguuccuacAg...........................................................   18     1   HA1
.............................agcgagguuuguaguuccuaccg...........................................................   148    0   HA1
.............................agcgagguuuguaguuccuacAgu..........................................................   3      1   HA1
.............................agcgagguuuguaguuccuaccgA..........................................................   2      1   HA1
.............................agcgaggGuuguaguuccuaccgu..........................................................   1      1   HA1
.............................agcgagguuuguaguuccuaccUu..........................................................   22     1   HA1
.............................agcgagguuuguaguuccuaccgu..........................................................   20     0   HA1
.............................agcgagguuuguaguuccuaccAu..........................................................   1      1   HA1
```

```
                            Star                                        Mature
caaaaaaaaucgaauuacacuucgucacgagcgagguuuguaguuccuaccguuuuuauucaaacuggguaggaacuucauaccgugcucuugaacgaugcugacgaucaa
.............................agcgagguuuguaguuccuaccguA.........................................................   1    1    HA1
.............................agcgagguuuguaguuccuaccUuu.........................................................   8    1    HA1
.............................agcgagguuuguaguuccuaccAuu.........................................................   2    1    HA1
.............................agcgagguuuguaguuccuaccguu.........................................................   17   0    HA1
.............................agcgagguuuguaguuccuaccguuu........................................................   3    0    HA1
..............................gcgagguuuguaguuccu...............................................................   1    0    HA1
..............................gcgagguuuguaguuccuU..............................................................   1    1    HA1
..............................gcgagguuuguaguuccua..............................................................   1    0    HA1
..............................gcgagguuuguaguuccuac.............................................................   14   0    HA1
..............................gcgagguuuguaguuccuacA............................................................   3    1    HA1
..............................gcgagguuuguaguuccuGcc............................................................   1    1    HA1
..............................gcgagguuuguaguuccuacc............................................................   89   0    HA1
..............................gcgagguuuguaguuccuacUg...........................................................   1    1    HA1
..............................gcgagguuuguaguuccuaccg...........................................................   64   0    HA1
..............................gcgagguuuguaguuccuaccC...........................................................   1    1    HA1
..............................gcgagguuuguaguuccuaccA...........................................................   10   1    HA1
...............................cgagguuuguaguuccuacc............................................................   9    0    HA1
...............................cgagguuuguaguuccuaccg...........................................................   7    0    HA1
...............................cgagguuuguaguuccuaccU...........................................................   3    1    HA1
...............................cgagguuuguaguuccuaccCuuu........................................................   1    1    HA1
.................................agguuuguaguuccuacU............................................................   1    1    HA1
.................................agguuuguaguuccuaccguu.........................................................   2    0    HA1
...................................guuuguaguuccuaccguuuu.......................................................   4    0    HA1
....................................uuuguaguuccuaccguuuu.......................................................   3    0    HA1
..............................................................aacuggguaggaacuucauaccgugc.......................   1    0    HA1
..............................................................aacuggguaggaacuucauaccgugcu......................   9    0    HA1
...............................................................acuggguaggaacuucauaccgugcu......................   4    0    HA1
................................................................cCggguaggaacuucauaccgugcu......................   1    1    HA1
................................................................cuggguaggaacuucauaccgugcu......................   14   0    HA1
................................................................cAggguaggaacuucauaccgugcucu....................   1    1    HA1
................................................................cuggguaggaacuucauaccgugcucu....................   1    0    HA1
................................................................Guggguaggaacuucauaccgugcucu....................   1    1    HA1
.................................................................uggguaggaacuucauaccgugcu......................   3    0    HA1
.................................................................uggguaggaacuucauaccgugcuc.....................   5    0    HA1
.................................................................uggguaggaacuucauaccgugcucu....................   3    0    HA1
.................................................................uggAuaggaacuucauaccgugcucu....................   1    1    HA1
.................................................................Gggguaggaacuucauaccgugcucu....................   1    1    HA1
.................................................................Cggguaggaacuucauaccgugcucu....................   1    1    HA1
..................................................................ggCuaggaacuucauaccgugcuc.....................   4    1    HA1
..................................................................ggguaggaacuucauaccgugcuc.....................   1    0    HA1
..................................................................ggguaggaacuucauaccgugcucu....................   6    0    HA1
..................................................................ggCuaggaacuucauaccgugcucu....................   4    1    HA1
..................................................................gAguaggaacuucauaccgugcucu....................   1    1    HA1
...................................................................gCuaggaacuucauaccgugcucu....................   2    1    HA1
...................................................................gguaggaacuucauaccgugcucu....................   3    0    HA1
....................................................................guaggaacuucauaccgug........................   1    0    HA1
....................................................................guaggaacuucauaccgugc.......................   7    0    HA1
....................................................................Uuaggaacuucauaccgugcu......................   11   1    HA1
....................................................................guaggaacuucauaccgugcu......................   63   0    HA1
....................................................................guaggaUcuucauaccgugcu......................   1    1    HA1
....................................................................guaggaacuucauaccguUcu......................   1    1    HA1
....................................................................Cuaggaacuucauaccgugcuc.....................   1    1    HA1
....................................................................guaggaacuucauaccgugcuc.....................   31   0    HA1
....................................................................guaggaacuucauaccgugcuU.....................   20   1    HA1
....................................................................Uuaggaacuucauaccgugcuc.....................   34   1    HA1
....................................................................Auaggaacuucauaccgugcuc.....................   1    1    HA1
....................................................................guaggaacuucauaccgugcucu....................   17   0    HA1
....................................................................guGggaacuucauaccgugcucu....................   1    1    HA1
....................................................................Uuaggaacuucauaccgugcucu....................   434  1    HA1
....................................................................Cuaggaacuucauaccgugcucu....................   5    1    HA1
....................................................................Auaggaacuucauaccgugcucu....................   14   1    HA1
....................................................................Uuaggaacuucauaccgugcucuu...................   2    1    HA1
.....................................................................uaggaacuucauaccgug........................   137  0    HA1
.....................................................................uaggaacuucauaccguU........................   1    1    HA1
.....................................................................uaggaaGuucauaccgug........................   1    1    HA1
.....................................................................uaggaacuucauacUgugc.......................   2    1    HA1
.....................................................................uaggaacuucauaccgugc.......................   599  0    HA1
.....................................................................uaggaaUuucauaccgugc.......................   1    1    HA1
.....................................................................uaggaacuucauaccgugA.......................   231  1    HA1
.....................................................................uUggaacuucauaccgugc.......................   1    1    HA1
```

Star **Mature**

```
caaaaaaaaucgaauuacacuucgucacgagcgagguuuguaguuccuaccguuuuuauucaaacuggguaggaacuucauaccgugcucuugaacgaugcugacgaucaa
.....................................................................uaggaacuucauaccgugU.......................  8      1  HA1
.....................................................................uaggaacuucauaccgugG.......................  1      1  HA1
.....................................................................uaggaaGuucauaccgugc.......................  2      1  HA1
.....................................................................uaggaacuAcauaccgugc.......................  1      1  HA1
.....................................................................uaggaacuuUauaccgugcu......................  11     1  HA1
.....................................................................uaCgaacuucauaccgugcu......................  1      1  HA1
.....................................................................uaggaacuGcauaccgugcu......................  1      1  HA1
.....................................................................uaggGacuucauaccgugcu......................  20     1  HA1
.....................................................................uaggaacuucauUccgugcu......................  26     1  HA1
.....................................................................uaggaUcuucauaccgugcu......................  6      1  HA1
.....................................................................uaggaacuucauaccgCgcu......................  2      1  HA1
.....................................................................uaggaacuucauaccguUcu......................  5      1  HA1
.....................................................................uagUaacuucauaccgugcu......................  1      1  HA1
.....................................................................uaggaacuucaGaccgugcu......................  1      1  HA1
.....................................................................uaggaCcuucauaccgugcu......................  14     1  HA1
.....................................................................uGggaacuucauaccgugcu......................  8      1  HA1
.....................................................................uaggaacuucauaccgugUu......................  19     1  HA1
.....................................................................uaggCacuucauaccgugcu......................  15     1  HA1
.....................................................................uaggaaAuucauaccgugcu......................  30     1  HA1
.....................................................................uaggaacuucauaccguAcu......................  3      1  HA1
.....................................................................Gaggaacuucauaccgugcu......................  6      1  HA1
.....................................................................uaggaacuucauGccgugcu......................  1      1  HA1
.....................................................................uaggaacCucauaccgugcu......................  4      1  HA1
.....................................................................uaggaacuucauacUgugcu......................  5      1  HA1
.....................................................................uaggaacuucaCaccgugcu......................  6      1  HA1
.....................................................................uaggaacuucauaccAugcu......................  6      1  HA1
.....................................................................uaggaacuucCuaccgugcu......................  3      1  HA1
.....................................................................uaggaacuucauaccgugAu......................  1      1  HA1
.....................................................................uaggaaGuucauaccgugcu......................  51     1  HA1
.....................................................................Caggaacuucauaccgugcu......................  10     1  HA1
.....................................................................uaUgaacuucauaccgugcu......................  1      1  HA1
.....................................................................uaggaacuucauaccgugcC......................  30     1  HA1
.....................................................................uagAaacuucauaccgugcu......................  11     1  HA1
.....................................................................Aaggaacuucauaccgugcu......................  1      1  HA1
.....................................................................uaggUacuucauaccgugcu......................  12     1  HA1
.....................................................................uCggaacuucauaccgugcu......................  5      1  HA1
.....................................................................uaggaacuucauaccgAgcu......................  7      1  HA1
.....................................................................uaggaacuucauaccguCcu......................  45     1  HA1
.....................................................................uaggaaUuucauaccgugcu......................  22     1  HA1
.....................................................................uaggaacuucauaUcgugcu......................  8      1  HA1
.....................................................................uaggaacuucauaccgGgcu......................  2      1  HA1
.....................................................................uaggaacuucauaccUugcu......................  8      1  HA1
.....................................................................uaggaacuucauaccgugcA......................  84     1  HA1
.....................................................................uagCaacuucauaccgugcu......................  4      1  HA1
.....................................................................uaggaacuuAauaccgugcu......................  2      1  HA1
.....................................................................uaggaGcuucauaccgugcu......................  9      1  HA1
.....................................................................uaggaacAucauaccgugcu......................  3      1  HA1
.....................................................................uaggaacuucauaccgugcG......................  6      1  HA1
.....................................................................uaggaacuucUuaccgugcu......................  1      1  HA1
.....................................................................uaggaacGucauaccgugcu......................  3      1  HA1
.....................................................................uaggaacuCcauaccgugcu......................  8      1  HA1
.....................................................................uaggaacuucaAaccgugcu......................  1      1  HA1
.....................................................................uaggaacuucauaccgugcu......................  16543  0  HA1
.....................................................................uaggaacuucauaccgugUuc.....................  82     1  HA1
.....................................................................Aaggaacuucauaccgugcuc.....................  11     1  HA1
.....................................................................uaggaacuCcauaccgugcuc.....................  36     1  HA1
.....................................................................Gaggaacuucauaccgugcuc.....................  26     1  HA1
.....................................................................uaCgaacuucauaccgugcuc.....................  11     1  HA1
.....................................................................uaggaacuucauaccguUcuc.....................  4      1  HA1
.....................................................................uaggaacuucauaccgAgcuc.....................  13     1  HA1
.....................................................................uaggaacuucauaUcgugcuc.....................  33     1  HA1
.....................................................................uaggUacuucauaccgugcuc.....................  65     1  HA1
.....................................................................uaggaacCucauaccgugcuc.....................  14     1  HA1
.....................................................................uaggaacuucauaccgugcAc.....................  4      1  HA1
.....................................................................uCggaacuucauaccgugcuc.....................  11     1  HA1
.....................................................................uaggaaUuucauaccgugcuc.....................  100    1  HA1
.....................................................................uaggaacGucauaccgugcuc.....................  5      1  HA1
.....................................................................uaggaacuucaAaccgugcuc.....................  1      1  HA1
.....................................................................uagUaacuucauaccgugcuc.....................  16     1  HA1
.....................................................................uaggaacuucauaccCugcuc.....................  4      1  HA1
```

Star Mature

caaaaaaaaucgaauuacacuucgucacgagcgagguuuguaguuccuaccguuuuuauucaaacuggguaggaacuucauaccgugcucuugaacgaugcugacgaucaa

....uaggaacuucGuaccgugcuc.....................	6	1	HA1
....uaggaacuGcauaccgugcuc.....................	12	1	HA1
....uaggaacuucauaccgCgcuc.....................	11	1	HA1
....uaggaUcuucauaccgugcuc.....................	25	1	HA1
....uaggCacuucauaccgugcuc.....................	93	1	HA1
....uaggaacuuAauaccgugcuc.....................	10	1	HA1
....uaggaacuucauaccgugAuc.....................	6	1	HA1
....uaggaacuucCuaccgugcuc.....................	2	1	HA1
....uaggaacuucauacGgugcuc.....................	1	1	HA1
....uaggaGcuucauaccgugcuc.....................	71	1	HA1
....uaggaacuucauaccUugcuc.....................	24	1	HA1
....uaggGacuucauaccgugcuc.....................	110	1	HA1
....uagCaacuucauaccgugcuc.....................	37	1	HA1
....uaggaacuucauaccgugcuc.....................	72836	0	HA1
....uaggaacuucauaccgugcuU.....................	2605	1	HA1
....uaggaacuAcauaccgugcuc.....................	15	1	HA1
....uaggaacuucauaccgGgcuc.....................	8	1	HA1
....uaggaCcuucauaccgugcuc.....................	64	1	HA1
....uaggaacuucauaccAugcuc.....................	17	1	HA1
....Caggaacuucauaccgugcuc.....................	30	1	HA1
....uaggaacuucauaccgugcuG.....................	12	1	HA1
....uaUgaacuucauaccgugcuc.....................	11	1	HA1
....uaggaacuucaCaccgugcuc.....................	30	1	HA1
....uaggaacuucauaccgugcuA.....................	1360	1	HA1
....uaggaacuucauaccgugcGc.....................	4	1	HA1
....uaggaacuucaGaccgugcuc.....................	7	1	HA1
....uUggaacuucauaccgugcuc.....................	6	1	HA1
....uaggaaAuucauaccgugcuc.....................	101	1	HA1
....uaggaacAucauaccgugcuc.....................	6	1	HA1
....uaggaacuucauaGcgugcuc.....................	1	1	HA1
....uaggaacuucauaccgugGuc.....................	3	1	HA1
....uaggaaGuucauaccgugcuc.....................	270	1	HA1
....uGggaacuucauaccgugcuc.....................	24	1	HA1
....uaggaacuucauacUgugcuc.....................	36	1	HA1
....uaggaacuucauUccgugcuc.....................	105	1	HA1
....uaggaacuucUuaccgugcuc.....................	3	1	HA1
....uagAaacuucauaccgugcuc.....................	24	1	HA1
....uaggaacuuUauaccgugcuc.....................	43	1	HA1
....uaggaacuucauaccguCcuc.....................	6	1	HA1
....uaggaacuucauaccgugcCc.....................	71	1	HA1
....uaggaacuucauaccguAcuc.....................	60	1	HA1
....uaggaacuucauaccgGgcucu....................	100	1	HA1
....Aaggaacuucauaccgugcucu....................	133	1	HA1
....uGggaacuucauaccgugcucu....................	471	1	HA1
....uaggaacuucauaccgugcucu....................	1217636	0	HA1
....uaggaUcuucauaccgugcucu....................	595	1	HA1
....uaggaacuucauaccgugGucu....................	26	1	HA1
....uaggaacuucauaccUugcucu....................	356	1	HA1
....uaggaacuuGauaccgugcucu....................	42	1	HA1
....uaggaacuucauaccgugcuGu....................	19	1	HA1
....uaggaacuucauaccgAgcucu....................	239	1	HA1
....uaggCacuucauaccgugcucu....................	2064	1	HA1
....uaggaacuucauaccgugUucu....................	872	1	HA1
....uaggaaUuucauaccgugcucu....................	1969	1	HA1
....uaggaacuucauUccgugcucu....................	2135	1	HA1
....uaggaacuucauaccgCgcucu....................	137	1	HA1
....Gaggaacuucauaccgugcucu....................	396	1	HA1
....uaggaacuucauaccguUcucu....................	23	1	HA1
....uaggaacuucGuaccgugcucu....................	80	1	HA1
....uaggaacuucauaccgugcucG....................	299	1	HA1
....uaggaacuucCuaccgugcucu....................	57	1	HA1
....uaggaacuucUuaccgugcucu....................	133	1	HA1
....uaggaGcuucauaccgugcucu....................	1317	1	HA1
....uaggaacuucauaccguAcucu....................	247	1	HA1
....uaggaacuucauaccgugcucA....................	1203	1	HA1
....uaggaacuucauaccgugcuUu....................	250	1	HA1
....uaggaacuucauacGgugcucu....................	16	1	HA1
....uCggaacuucauaccgugcucu....................	155	1	HA1
....uaggaacuucauaUcgugcucu....................	266	1	HA1
....uaggaacuGcauaccgugcucu....................	205	1	HA1

```
                             Star                                    Mature
caaaaaaaaucgaauuacacuucgucacgagcgagguuuguaguuccuaccguuuuuauucaaacuggguaggaacuucauaccgugcucuugaacgaugcugacgaucaa
.....................................................................uaggaacGucauaccgugcucu....................   75    1   HA1
.....................................................................uaggaacuucauaccgugcGcu....................   49    1   HA1
.....................................................................uaggaacuucauaAcgugcucu....................   3     1   HA1
.....................................................................uaggaacuucauaccgugcuAu....................   23    1   HA1
.....................................................................uaggaacuuUauaccgugcucu....................   912   1   HA1
.....................................................................uaggGacuucauaccgugcucu....................   1994  1   HA1
.....................................................................uaggaacuucauaccCugcucu....................   99    1   HA1
.....................................................................uaggaacuucauCccgugcucu....................   21    1   HA1
.....................................................................uaggaacuuAauaccgugcucu....................   133   1   HA1
.....................................................................uaggaacAucauaccgugcucu....................   123   1   HA1
.....................................................................uaggaacuucauaccgugcAcu....................   42    1   HA1
.....................................................................uaggaacuucauaccgugcCcu....................   933   1   HA1
.....................................................................uagCaacuucauaccgugcucu....................   752   1   HA1
.....................................................................uaCgaacuucauaccgugcucu....................   201   1   HA1
.....................................................................uaggaCcuucauaccgugcucu....................   1347  1   HA1
.....................................................................uaggaacuucauacAgugcucu....................   54    1   HA1
.....................................................................uaggaacuCcauaccgugcucu....................   519   1   HA1
.....................................................................uagUaacuucauaccgugcucu....................   233   1   HA1
.....................................................................uaggaaGuucauaccgugcucu....................   4470  1   HA1
.....................................................................uaggaacuucauaccguCcucu....................   54    1   HA1
.....................................................................uaggaacuucauacUgugcucu....................   235   1   HA1
.....................................................................uaggaacuucauGccgugcucu....................   67    1   HA1
.....................................................................Caggaacuucauaccgugcucu....................   443   1   HA1
.....................................................................uaggaacuucaGaccgugcucu....................   70    1   HA1
.....................................................................uaAgaacuucauaccgugcucu....................   41    1   HA1
.....................................................................uaggaacuucaCaccgugcucu....................   436   1   HA1
.....................................................................uaggaacuucauaccgugAucu....................   101   1   HA1
.....................................................................uaggaacuucaAaccgugcucu....................   11    1   HA1
.....................................................................uUggaacuucauaccgugcucu....................   141   1   HA1
.....................................................................uaggaacuAcauaccgugcucu....................   184   1   HA1
.....................................................................uaggaacuucauaGcgugcucu....................   8     1   HA1
.....................................................................uagAaacuucauaccgugcucu....................   392   1   HA1
.....................................................................uaggUacuucauaccgugcucu....................   1072  1   HA1
.....................................................................uaUgaacuucauaccgugcucu....................   95    1   HA1
.....................................................................uaggaacCucauaccgugcucu....................   168   1   HA1
.....................................................................uaggaaAuucauaccgugcucu....................   1857  1   HA1
.....................................................................uaggaacuucauaccAugcucu....................   362   1   HA1
.....................................................................uaggaacuucauaccgugcucC....................   9879  1   HA1
.....................................................................uaggaacuCcauaccgugcucuu...................   2     1   HA1
.....................................................................uaggaaGuucauaccgugcucuu...................   22    1   HA1
.....................................................................uaggCacuucauaccgugcucuu...................   8     1   HA1
.....................................................................uGggaacuucauaccgugcucuu...................   2     1   HA1
.....................................................................uaggUacuucauaccgugcucuu...................   6     1   HA1
.....................................................................uaggGacuucauaccgugcucuu...................   9     1   HA1
.....................................................................Gaggaacuucauaccgugcucuu...................   3     1   HA1
.....................................................................uaggaacuucauaccUugcucuu...................   1     1   HA1
.....................................................................uaggaacuucauaccgugcucuA...................   1162  1   HA1
.....................................................................Caggaacuucauaccgugcucuu...................   4     1   HA1
.....................................................................uaggaacuucauaUcgugcucuu...................   2     1   HA1
.....................................................................uaggaacuucauaccgugcucCu...................   56    1   HA1
.....................................................................uaggaacuucauaccgugcucuG...................   10    1   HA1
.....................................................................uaggaacuuUauaccgugcucuu...................   4     1   HA1
.....................................................................uaggaacCucauaccgugcucuu...................   1     1   HA1
.....................................................................uaggaaUuucauaccgugcucuu...................   9     1   HA1
.....................................................................uaggaacuucauaccgGgcucuu...................   1     1   HA1
.....................................................................uaggaacuucauaccgCgcucuu...................   2     1   HA1
.....................................................................uaggaacuuAauaccgugcucuu...................   2     1   HA1
.....................................................................uaggaacuucauaccgugcCcuu...................   1     1   HA1
.....................................................................uaggaacuucaCaccgugcucuu...................   4     1   HA1
.....................................................................uaggaUcuucauaccgugcucuu...................   2     1   HA1
.....................................................................uaggaacuucauaccgugcucuu...................   6658  0   HA1
.....................................................................uaggaGcuucauaccgugcucuu...................   3     1   HA1
.....................................................................uaggaacuAcauaccgugcucuu...................   1     1   HA1
.....................................................................uaggaacuucCuaccgugcucuu...................   1     1   HA1
.....................................................................uaggaacuucauaccgugcucuC...................   22    1   HA1
.....................................................................uCggaacuucauaccgugcucuu...................   2     1   HA1
.....................................................................uaCgaacuucauaccgugcucuu...................   3     1   HA1
.....................................................................uagAaacuucauaccgugcucuu...................   2     1   HA1
.....................................................................uagCaacuucauaccgugcucuu...................   5     1   HA1
.....................................................................uagUaacuucauaccgugcucuu...................   1     1   HA1
```

Star Mature

caaaaaaaaucgaauuacacuucgucacgagcgagguuuguaguuccuaccguuuuuauucaaacuggguaggaacuucauaccgugcucuugaacgaugcugacgaucaa			
....uaggaacuucauUccgugcucuu...................	14	1	HA1
....uaggaacuucauaccguCcucuu...................	1	1	HA1
....uaggaacuucauaccgugAucuu...................	1	1	HA1
....uaggaacuucauaccgAgcucuu...................	1	1	HA1
....uaggaaAuucauaccgugcucuu...................	11	1	HA1
....uaggaacuucauaccAugcucuu...................	4	1	HA1
....uaggaCcuucauaccgugcucuu...................	7	1	HA1
....uaggaacuucauaccgugcucuAg..................	337	1	HA1
....uaggaacuucauaccgugcucuuU..................	62	1	HA1
....uaggaacuucauaccgugcucuuC..................	3	1	HA1
....uaggaacuucauaccgugcucuGg..................	4	1	HA1
....uaggaacuucauaccgugcucuug..................	11	0	HA1
....uaggaacuucauaccgugcucuuA..................	10	1	HA1
....uaggCacuucauaccgugcucuug..................	1	1	HA1
....uaggaacuucauaccgugcucuuUa.................	1	1	HA1
....uaggaacuucauaccgugcucuuga.................	1	0	HA1
....aggaacuucauaccgugc.......................	1	0	HA1
....aggaacuucauaccgugU.......................	1	1	HA1
....aggaacuucauaccgugcu......................	28	0	HA1
....aggaacuucauaccgugcA......................	1	1	HA1
....aggaacuCcauaccgugcuc.....................	1	1	HA1
....aggaacuucauaccguAcuc.....................	1	1	HA1
....aggaacuucauaUcgugcuc.....................	1	1	HA1
....aggaacuucauaccgugcuU.....................	5	1	HA1
....aAgaacuucauaccgugcuc.....................	1	1	HA1
....aggaacuuAauaccgugcuc.....................	1	1	HA1
....aggaacuucauaccgugcuc.....................	223	0	HA1
....aggaacAucauaccgugcuc.....................	1	1	HA1
....aggaacuucauCccgugcuc.....................	1	1	HA1
....Cggaacuucauaccgugcuc.....................	1	1	HA1
....aggaacuucauaccgugcuA.....................	4	1	HA1
....aggaacuucaCaccgugcucu....................	1	1	HA1
....aggaacuCcauaccgugcucu....................	1	1	HA1
....aggaacuucauaccgugcucC....................	50	1	HA1
....aggaacuucauaccgugUucu....................	1	1	HA1
....aggUacuucauaccgugcucu....................	2	1	HA1
....aUgaacuucauaccgugcucu....................	11	1	HA1
....Gggaacuucauaccgugcucu....................	4	1	HA1
....aggCacuucauaccgugcucu....................	4	1	HA1
....aggaacuucauaccguAcucu....................	3	1	HA1
....aggaacuucauaccguUcucu....................	1	1	HA1
....aggaCcuucauaccgugcucu....................	4	1	HA1
....aCgaacuucauaccgugcucu....................	1	1	HA1
....aggaaAuucauaccgugcucu....................	2	1	HA1
....aggaacuucauaccgugcuUu....................	1	1	HA1
....aggaacuucauaUcgugcucu....................	3	1	HA1
....aggaUcuucauaccgugcucu....................	2	1	HA1
....aggaaUuucauaccgugcucu....................	3	1	HA1
....aggaacuucauaccUugcucu....................	1	1	HA1
....aggaacuucauaccgugcCcu....................	2	1	HA1
....aggaacuucauaccgugcAcu....................	1	1	HA1
....Uggaacuucauaccgugcucu....................	2	1	HA1
....aggaacuucauaccgCgcucu....................	1	1	HA1
....agCaacuucauaccgugcucu....................	1	1	HA1
....aggaacGucauaccgugcucu....................	1	1	HA1
....aggaacuucauaccCugcucu....................	1	1	HA1
....aggaacuucauaccgugcucu....................	2391	0	HA1
....aggaGcuucauaccgugcucu....................	3	1	HA1
....aggGacuucauaccgugcucu....................	2	1	HA1
....aggaacuucGuaccgugcucu....................	1	1	HA1
....aggaacuuUauaccgugcucu....................	2	1	HA1
....aggaacuucauaccgugcucA....................	1	1	HA1
....aggaaGuucauaccgugcucu....................	6	1	HA1
....aggaacuucauaccgugcucuu...................	10	0	HA1
....aggaacuucauaccgugcucuA...................	3	1	HA1
....aggaacuucaAaccgugcucuu...................	1	1	HA1
....aggaacuucauaccgugcucuuU..................	1	1	HA1
....aggaacuucauaccgugcucuAg..................	2	1	HA1
....ggaacuucauaccgugcu......................	2	0	HA1
....Ugaacuucauaccgugcu......................	1	1	HA1

```
                             Star                                      Mature
caaaaaaaaucgaauuacacuucgucacgagcgagguuuguaguuccuaccguuuuauucaaacuggguaggaacuucauaccgugcucuugaacgaugcugacgaucaa
......................................................................ggaacuucauaccgugcuA.....................   1     1   HA1
......................................................................ggGacuucauaccgugcuc.....................   1     1   HA1
......................................................................ggaacuCcauaccgugcuc.....................   1     1   HA1
......................................................................ggaacuucauaccgugcuc.....................   19    0   HA1
......................................................................Cgaacuucauaccgugcucu....................   1     1   HA1
......................................................................ggaaUuucauaccgugcucu....................   1     1   HA1
......................................................................ggaacuucauaccgugcucu....................   248   0   HA1
......................................................................ggaacuucauaccgugcucC....................   3     1   HA1
......................................................................ggaacuucauaccgugUucu....................   1     1   HA1
......................................................................ggGacuucauaccgugcucu....................   2     1   HA1
......................................................................Ugaacuucauaccgugcucu....................   18    1   HA1
......................................................................Agaacuucauaccgugcucu....................   3     1   HA1
.......................................................................Uaacuucauaccgugcuc.....................   1     1   HA1
.......................................................................gaacuucauaccgugcuc.....................   2     0   HA1
.......................................................................gaacuucauaccgugcucu....................   38    0   HA1
.......................................................................Uaacuucauaccgugcucu....................   12    1   HA1
.......................................................................gaacuucauaccgugcucuu...................   1     0   HA1
.......................................................................gaacuucauaccgugcucuuU..................   1     1   HA1
........................................................................aacuucGuaccgugcucu....................   1     1   HA1
........................................................................aacuucauaccgugcucu....................   87    0   HA1
........................................................................aaUuucauaccgugcucu....................   1     1   HA1
........................................................................aacuucauaccgugcucuA...................   1     1   HA1

.........ucgaauuacacuucgucacg.................................................................................   1     0   HS1
.........................cacgagcgagguuuguaguuccu..............................................................   18    0   HS1
.........................cacgagcgagguuuguaguuccuU.............................................................   3     1   HS1
..........................acgagcgagguuuguaguuccu..............................................................   58    0   HS1
..........................acgagcgagguuuguaguuccC..............................................................   4     1   HS1
..........................acgagcgagguuuguaguuccuU.............................................................   2     1   HS1
...........................cgagcgagguuuguaguuccC..............................................................   2     1   HS1
...........................cgagcgagguuuguaguuccu..............................................................   12    0   HS1
............................Cagcgagguuuguaguuccuac............................................................   13    1   HS1
............................Cagcgagguuuguaguuccuacc...........................................................   74    1   HS1
............................Cagcgagguuuguaguuccuaccg..........................................................   3     1   HS1
.............................agcgagguuuguaguucc...............................................................   9     0   HS1
.............................agcgaggGuuguaguuccu..............................................................   1     1   HS1
.............................agcgagguuuguaguuccu..............................................................   171   0   HS1
.............................agcgaggAuuguaguuccu..............................................................   1     1   HS1
.............................agcgagguuuguaguuccC..............................................................   10    1   HS1
.............................agcgagguuuguaguuccA..............................................................   3     1   HS1
.............................agcgagguuuguaguuccuU.............................................................   1     1   HS1
.............................agcgagguuuguaguuccua.............................................................   114   0   HS1
.............................agcgGgguuuguaguuccuac............................................................   2     1   HS1
.............................Ggcgagguuuguaguuccuac............................................................   4     1   HS1
.............................agAgagguuuguaguuccuac............................................................   1     1   HS1
.............................agUgagguuuguaguuccuac............................................................   14    1   HS1
.............................agcAagguuuguaguuccuac............................................................   1     1   HS1
.............................agcgagguuGguaguuccuac............................................................   1     1   HS1
.............................agcgagguuuguaguuccCac............................................................   1     1   HS1
.............................agcgagguuuguagCuccuac............................................................   1     1   HS1
.............................aAcgagguuuguaguuccuac............................................................   13    1   HS1
.............................agcgaggGuuguaguuccuac............................................................   3     1   HS1
.............................agcgagguuuguaguuccuaU............................................................   19    1   HS1
.............................agcgagguuuguaguuccAac............................................................   3     1   HS1
.............................agcgaggAuuguaguuccuac............................................................   1     1   HS1
.............................agcgagguuCguaguuccuac............................................................   2     1   HS1
.............................agcgagguuugAaguuccuac............................................................   1     1   HS1
.............................agcgagguuuguaguuccuaA............................................................   2     1   HS1
.............................Cgcgagguuuguaguuccuac............................................................   1     1   HS1
.............................agcgagguuuguaguuccuCc............................................................   1     1   HS1
.............................agcgagguuuguagGuccuac............................................................   2     1   HS1
.............................agcgagguuuguGguuccuac............................................................   2     1   HS1
.............................agcgagguuAguaguuccuac............................................................   1     1   HS1
.............................agcgaAguuuguaguuccuac............................................................   1     1   HS1
.............................agcgagguuuguaguucUuac............................................................   4     1   HS1
.............................agcgagguuugCaguuccuac............................................................   1     1   HS1
.............................aUcgagguuuguaguuccuac............................................................   1     1   HS1
.............................agcgagguuuguUguuccuac............................................................   2     1   HS1
.............................agcgagguuuAuaguuccuac............................................................   1     1   HS1
.............................agcgaggCuuguaguuccuac............................................................   4     1   HS1
```

```
                             Star                                        Mature
caaaaaaaaucgaauuacacuucgucacgagcgagguuuguaguuccuaccguuuuauucaaacuggguaggaacuucauaccgugcucuugaacgaugcugacgaucaa
.............................agcCagguuuguaguuccuac............................................................ 2      1  HS1
.............................agcgagguCuguaguuccuac............................................................ 2      1  HS1
.............................agcgagguuuguaguuccuac............................................................ 15117  0  HS1
.............................agcgagguuuguaguCccuac............................................................ 1      1  HS1
.............................agcgagguuugCaguuccuacc........................................................... 9      1  HS1
.............................agcgagguuuguaCuuccuacc........................................................... 2      1  HS1
.............................agUgagguuuguaguuccuacc........................................................... 35     1  HS1
.............................agcgUgguuuguaguuccuacc........................................................... 3      1  HS1
.............................agcgagguuuguaguuccuacA........................................................... 302    1  HS1
.............................agcgagguuuguaAuuccuacc........................................................... 3      1  HS1
.............................agcgagguuuguaguuccuaUc........................................................... 15     1  HS1
.............................agcgagguuuguagAuccuacc........................................................... 2      1  HS1
.............................agcgagguuuguaguuAcuacc........................................................... 1      1  HS1
.............................agcgagguuuguaguucUuacc........................................................... 6      1  HS1
.............................agcgagguuCguaguuccuacc........................................................... 9      1  HS1
.............................agcgagguuuguagGuccuacc........................................................... 3      1  HS1
.............................agcgaggCuuguaguuccuacc........................................................... 19     1  HS1
.............................agcgaAguuuguaguuccuacc........................................................... 2      1  HS1
.............................agcgagguuugAaguuccuacc........................................................... 11     1  HS1
.............................agcgagguAuguaguuccuacc........................................................... 2      1  HS1
.............................Cgcgagguuuguaguuccuacc........................................................... 1      1  HS1
.............................agcgagguCuguaguuccuacc........................................................... 8      1  HS1
.............................agcgagguuuguaguuccuaAc........................................................... 1      1  HS1
.............................agcgaggAuuguaguuccuacc........................................................... 1      1  HS1
.............................Ugcgagguuuguaguuccuacc........................................................... 7      1  HS1
.............................aUcgagguuuguaguuccuacc........................................................... 3      1  HS1
.............................agcgagUuuuguaguuccuacc........................................................... 2      1  HS1
.............................agcgagguuuCuaguuccuacc........................................................... 2      1  HS1
.............................agcgagguuAguaguuccuacc........................................................... 2      1  HS1
.............................agcgagguuuguGguuccuacc........................................................... 19     1  HS1
.............................agcgagguuuguaguuccuacU........................................................... 407    1  HS1
.............................agcgGgguuuguaguuccuacc........................................................... 18     1  HS1
.............................agcgagguuuguaguucAuacc........................................................... 3      1  HS1
.............................agGgagguuuguaguuccuacc........................................................... 3      1  HS1
.............................agcgagguuuguaguAccuacc........................................................... 1      1  HS1
.............................agcgagguuuguUguuccuacc........................................................... 1      1  HS1
.............................agcgagguuuguaUuuccuacc........................................................... 1      1  HS1
.............................agcgagguuuUuaguuccuacc........................................................... 3      1  HS1
.............................agcgagguuuguaguuccuaGc........................................................... 1      1  HS1
.............................aAcgagguuuguaguuccuacc........................................................... 38     1  HS1
.............................agcgagguuuAuaguuccuacc........................................................... 7      1  HS1
.............................agcgagguuugGaguuccuacc........................................................... 1      1  HS1
.............................agcgagguuuguagCuccuacc........................................................... 25     1  HS1
.............................agcUagguuuguaguuccuacc........................................................... 1      1  HS1
.............................agcgagguuuguaguuccuGcc........................................................... 8      1  HS1
.............................agcgagguuuguaguucGuacc........................................................... 2      1  HS1
.............................agcgCgguuuguaguuccuacc........................................................... 1      1  HS1
.............................agcgagguuuguaguuUcuacc........................................................... 30     1  HS1
.............................Ggcgagguuuguaguuccuacc........................................................... 23     1  HS1
.............................agcAagguuuguaguuccuacc........................................................... 19     1  HS1
.............................agcgagguuuguaguCccuacc........................................................... 25     1  HS1
.............................agcgaggGuuguaguuccuacc........................................................... 10     1  HS1
.............................agcgagguuuguaguuccuacG........................................................... 1      1  HS1
.............................agcgagguuuguaguGccuacc........................................................... 4      1  HS1
.............................agcgagguuuguaguuccuacc........................................................... 77925  0  HS1
.............................agcgagAuuuguaguuccuacc........................................................... 3      1  HS1
.............................agcgagguuuguaguuccAacc........................................................... 2      1  HS1
.............................agcgagguuuguaguuccuUcc........................................................... 5      1  HS1
.............................agcgagguuGguaguuccuacc........................................................... 2      1  HS1
.............................agcgagguuuguaguuccGacc........................................................... 2      1  HS1
.............................agcgagguuuguaguuccCacc........................................................... 32     1  HS1
.............................agcgagguuuguaguuccuaccU.......................................................... 308    1  HS1
.............................agcgagguuuguaguuccuaccg.......................................................... 1324   0  HS1
.............................aAcgagguuuguaguuccuaccg.......................................................... 1      1  HS1
.............................agcAagguuuguaguuccuaccg.......................................................... 1      1  HS1
.............................agUgagguuuguaguuccuaccg.......................................................... 2      1  HS1
.............................agcgagguuuguGguuccuaccg.......................................................... 1      1  HS1
.............................Ggcgagguuuguaguuccuaccg.......................................................... 2      1  HS1
.............................agcgagguuuguaguuccuaccA.......................................................... 97     1  HS1
.............................agcgagguuuguaguuccuaccC.......................................................... 82     1  HS1
```

Star **Mature**

```
caaaaaaaaucgaauuacacuucgucacgagcgagguuuguaguuccuaccguuuuuauucaaacuggguaggaacuucauaccgugcucuugaacgaugcugacgaucaa
.............................agcgGgguuuguaguuccuaccg...........................................................	2	1	HS1
.............................agcgagguuuguaCuuccuaccg...........................................................	1	1	HS1
.............................agcgagguuuAuaguuccuaccg...........................................................	1	1	HS1
.............................agcgagguuuguaguuccuaccgu..........................................................	8	0	HS1
.............................agcgagguuuguaguuccuaccgA..........................................................	1	1	HS1
.............................agcgagguuuguaguuccuaccguu.........................................................	1	0	HS1
..............................gcgagguuuguaguuccC...............................................................	3	1	HS1
..............................gcgagguuuguaguuccu...............................................................	7	0	HS1
..............................gcgagguuuguaguuccua..............................................................	1	0	HS1
..............................gcgagguuugCaguuccuac.............................................................	1	1	HS1
..............................gcgagguuuguagGuccuac.............................................................	1	1	HS1
..............................Ucgagguuuguaguuccuac.............................................................	1	1	HS1
..............................gcgagguuuguaguuccuac.............................................................	713	0	HS1
..............................gcAagguuuguaguuccuac.............................................................	1	1	HS1
..............................gcgagguuuguaguuccuacA............................................................	5	1	HS1
..............................gcgCgguuuguaguuccuacc............................................................	1	1	HS1
..............................gcgagguGuguaguuccuacc............................................................	1	1	HS1
..............................gcgagguuuguaguuccuaUc............................................................	1	1	HS1
..............................gcgGgguuuguaguuccuacc............................................................	1	1	HS1
..............................gcgagguuuguaguuccuacc............................................................	3417	0	HS1
..............................Acgagguuuguaguuccuacc............................................................	2	1	HS1
..............................Ucgagguuuguaguuccuacc............................................................	4	1	HS1
..............................gcgagguuuguaguuccuacU............................................................	9	1	HS1
..............................gcgagguuCguaguuccuacc............................................................	1	1	HS1
..............................gcgagguuugCaguuccuacc............................................................	1	1	HS1
..............................gcgagguuuguagCuccuacc............................................................	1	1	HS1
..............................gcgagguuuguaguuccuaccU...........................................................	7	1	HS1
..............................gcgagguuuguaguuccuaccC...........................................................	7	1	HS1
..............................gcgagguuuguaguuccuaccg...........................................................	125	0	HS1
..............................gcgagguuuguaguucGuaccg...........................................................	1	1	HS1
..............................gcgagguuuguaguuccuaccgA..........................................................	1	1	HS1
..............................gcgagguuuguaguuccuaccgu..........................................................	2	0	HS1
...............................cgagguuuguaguuccuac.............................................................	7	0	HS1
...............................cgagguuuguaguuccuacc............................................................	12	0	HS1
...............................cgagguuuguaguuccuacU............................................................	4	1	HS1
...............................cgagguuuguaguuccuaccC...........................................................	20	1	HS1
...............................cgagguuuguaguuccuaccg...........................................................	46	0	HS1
................................gagguuuguaguuccuac.............................................................	4	0	HS1
................................gagguuuguaguuccuacc............................................................	2	0	HS1
................................gagguuuguaguuccuaccg...........................................................	5	0	HS1
.................................agguuuguaguuccuacc............................................................	6	0	HS1
...................................................guuuuuauucaaacuggg..........................................	4	0	HS1
....................................................................guaggaacuucauaccgug........................	2	0	HS1
....................................................................guaggaacuucauaccgugcuc.....................	8	0	HS1
....................................................................guaggaacuucauaccgugcucC....................	12	1	HS1
....................................................................guaggaacuucauaccgCgcucu....................	1	1	HS1
....................................................................guaggaacuucauaccgugcucA....................	3	1	HS1
....................................................................Cuaggaacuucauaccgugcucu....................	2364	1	HS1
....................................................................guaggaacuucauaccgugcucu....................	1101	0	HS1
....................................................................guaggaacuCcauaccgugcucu....................	1	1	HS1
....................................................................Auaggaacuucauaccgugcucu....................	23	1	HS1
....................................................................Uuaggaacuucauaccgugcucu....................	12	1	HS1
....................................................................Cuaggaacuucauaccgugcucuu...................	1	1	HS1
....................................................................guaggaacuucauaccgugcucuCg..................	1	1	HS1
.....................................................................uaggaacuucauaccgug........................	10	0	HS1
.....................................................................Gaggaacuucauaccgugc.......................	1	1	HS1
.....................................................................uaggaacuucauaccgugA.......................	4	1	HS1
.....................................................................uaggaacuucauaccgugc.......................	8	0	HS1
.....................................................................uaggaacuucauaccgCgc.......................	1	1	HS1
.....................................................................uaggaacuucauaccgugcu......................	28	0	HS1
.....................................................................uaggaacuucauaccgugUuc.....................	3	1	HS1
.....................................................................uaggaacuucGuaccgugcuc.....................	2	1	HS1
.....................................................................uaggaacuucauaccguAcuc.....................	6	1	HS1
.....................................................................uaggaacuucauaccgCgcuc.....................	1	1	HS1
.....................................................................uaggaacuucauacUgugcuc.....................	4	1	HS1
.....................................................................uaggaacuucauaccgugcuG.....................	1	1	HS1
.....................................................................uaggaacuucauaccgugcuA.....................	1	1	HS1
.....................................................................uaggaacuucaCaccgugcuc.....................	1	1	HS1
.....................................................................uaggaacuucauaccgGgcuc.....................	3	1	HS1
.....................................................................uaggaaUuucauaccgugcuc.....................	1	1	HS1
```

Star **Mature**

caaaaaaaaucgaauuacacuucgucacgagcgagguuuguaguuccuaccguuuuuauucaaacuggguaggaacuucauaccgugcucuugaacgaugcugacgaucaa			
....uaggaacuucauaccgugcCc.....................	6	1	HS1
....uaggaacuucauaccgugcuc.....................	4836	0	HS1
....uUggaacuucauaccgugcuc.....................	1	1	HS1
....Gaggaacuucauaccgugcuc.....................	1	1	HS1
....uagCaacuucauaccgugcuc.....................	1	1	HS1
....uaggaacuucauaUcgugcuc.....................	1	1	HS1
....uaggaacuucauaccgugcuU.....................	5	1	HS1
....uaggaacuucaGaccgugcuc.....................	1	1	HS1
....uaggaacuCcauaccgugcuc.....................	1	1	HS1
....uaggaacuucauaccgugAuc.....................	1	1	HS1
....uaggaGcuucauaccgugcuc.....................	1	1	HS1
....uaggaacuucauaccgugcucG....................	462	1	HS1
....uaggGacuucauaccgugcucu....................	910	1	HS1
....uaggaacuuAauaccgugcucu....................	84	1	HS1
....uaggaacuucauaccgugcAcu....................	148	1	HS1
....uaggaacuGcauaccgugcucu....................	99	1	HS1
....uaggaacAucauaccgugcucu....................	243	1	HS1
....uaggaacuucauaccgugcuGu....................	48	1	HS1
....uaggaacuucauaccgGgcucu....................	115	1	HS1
....uaUgaacuucauaccgugcucu....................	165	1	HS1
....uaggaacuucauacAgugcucu....................	80	1	HS1
....uaggaacuucauaccguCcucu....................	58	1	HS1
....uCggaacuucauaccgugcucu....................	43	1	HS1
....uaggaacuucauaccgugcucC....................	40278	1	HS1
....uaggaacuucauaccUugcucu....................	82	1	HS1
....uaggaacuucCuaccgugcucu....................	19	1	HS1
....uagUaacuucauaccgugcucu....................	67	1	HS1
....uaggaacuucauaAcgugcucu....................	57	1	HS1
....uaggaacuucaCaccgugcucu....................	583	1	HS1
....uaggaacuucauaccAugcucu....................	1338	1	HS1
....uagCaacuucauaccgugcucu....................	40	1	HS1
....uUggaacuucauaccgugcucu....................	91	1	HS1
....uGggaacuucauaccgugcucu....................	1081	1	HS1
....uaggaacuCcauaccgugcucu....................	1430	1	HS1
....uaggaacuucauaccgAgcucu....................	103	1	HS1
....uaggaacuucauGccgugcucu....................	541	1	HS1
....uaggaUcuucauaccgugcucu....................	23	1	HS1
....uaggaacuucauacGgugcucu....................	27	1	HS1
....uaggaCcuucauaccgugcucu....................	15	1	HS1
....uaggaacuucauaccgugcGcu....................	593	1	HS1
....uaggaacuucauaccgugGucu....................	59	1	HS1
....uaggaacuuUauaccgugcucu....................	399	1	HS1
....Caggaacuucauaccgugcucu....................	1086	1	HS1
....uagAaacuucauaccgugcucu....................	281	1	HS1
....uaggaaGuucauaccgugcucu....................	34	1	HS1
....uaggaacuucauUccgugcucu....................	24	1	HS1
....uaggaacuucauaccgugcucA....................	7174	1	HS1
....uaggaacuucauacUgugcucu....................	503	1	HS1
....uaggaacuucauaccgugcCcu....................	1486	1	HS1
....uaAgaacuucauaccgugcucu....................	268	1	HS1
....uaggaacGucauaccgugcucu....................	75	1	HS1
....uaggaacuuGauaccgugcucu....................	34	1	HS1
....uaggaacuucauaccgugAucu....................	99	1	HS1
....uaggaacuucauaGcgugcucu....................	45	1	HS1
....uaggCacuucauaccgugcucu....................	36	1	HS1
....uaggaaAuucauaccgugcucu....................	69	1	HS1
....uaggaacCucauaccgugcucu....................	1056	1	HS1
....Gaggaacuucauaccgugcucu....................	495	1	HS1
....uaggaaUuucauaccgugcucu....................	166	1	HS1
....uaggaacuucauaUcgugcucu....................	477	1	HS1
....uaggaacuucauaccgugcuAu....................	114	1	HS1
....uaggaGcuucauaccgugcucu....................	811	1	HS1
....uaggUacuucauaccgugcucu....................	57	1	HS1
....uaggaacuucauaccguUcucu....................	266	1	HS1
....uaggaacuucauaccCugcucu....................	39	1	HS1
....uaggaacuAcauaccgugcucu....................	156	1	HS1
....uaggaacuucauaccgugcuUu....................	166	1	HS1
....uaggaacuuUuaccgugcucu....................	35	1	HS1
....uaggaacuucGuaccgugcucu....................	566	1	HS1
....Aaggaacuucauaccgugcucu....................	328	1	HS1

Star **Mature**

caaaaaaaaucgaauuacacuucgucacgagcgagguuuguaguuccuaccguuuuuauucaaacuggguaggaacuucauaccgugcucuugaacgaugcugacgaucaa			
....uaggaacuucauCccgugcucu....................	15	1	HS1
....uaggaacuucaGaccgugcucu....................	54	1	HS1
....uaCgaacuucauaccgugcucu....................	85	1	HS1
....uaggaacuucaAaccgugcucu....................	66	1	HS1
....uaggaacuucauaccgugUucu....................	980	1	HS1
....uaggaacuucauaccgCgcucu....................	592	1	HS1
....uaggaacuucauaccgugcucu....................	3513421	0	HS1
....uaggaacuucauaccguAcucu....................	6177	1	HS1
....uaggaacuucauaccAugcucuu...................	1	1	HS1
....uaggaacuucauaccgugcucuA...................	69	1	HS1
....uagCaacuucauaccgugcucuu...................	1	1	HS1
....uaggaacuucauaccgugcucuu...................	1097	0	HS1
....uaggaacuucauaccgugcucAu...................	6	1	HS1
....uaggaacuucauCccgugcucuu...................	1	1	HS1
....uaggaacuucauaccgugcucuC...................	68	1	HS1
....uaggaacuucauaccgugcGcuu...................	1	1	HS1
....uaggaacuucauaccgugcucuuC..................	18	1	HS1
....uaggaacuucauaccgugcucuuU..................	103	1	HS1
....uaggaacuucauaccgugcucuAg..................	10	1	HS1
....uaggaacuucauaccgugcucuAga.................	11	1	HS1
....aggaacuucauaccgugcu......................	1	0	HS1
....aggaacuucauaccgugcuc.....................	369	0	HS1
....aggaacuucauaccgCgcuc.....................	1	1	HS1
....aggaacuucauaccgugcuAu....................	5	1	HS1
....aggaacuucCuaccgugcucu....................	14	1	HS1
....aggaacuucauaccgugUucu....................	56	1	HS1
....aggaacuucaCaccgugcucu....................	35	1	HS1
....aggGacuucauaccgugcucu....................	80	1	HS1
....aggaacuucauUccgugcucu....................	4	1	HS1
....aggaaAuucauaccgugcucu....................	9	1	HS1
....aggaacuucauGccgugcucu....................	49	1	HS1
....aggaacuucauacGgugcucu....................	5	1	HS1
....aggaacuucaGaccgugcucu....................	5	1	HS1
....aggaacuucauaccgugcucA....................	474	1	HS1
....aggaacuCcauaccgugcucu....................	95	1	HS1
....aggaacuAcauaccgugcucu....................	8	1	HS1
....aggaacuucauaccgugcGcu....................	83	1	HS1
....aggaacuucauaccUugcucu....................	7	1	HS1
....agUaacuucauaccgugcucu....................	2	1	HS1
....aggaacuucauacUgugcucu....................	35	1	HS1
....aggaacuucauaccgugcuUu....................	10	1	HS1
....aggaUcuucauaccgugcucu....................	3	1	HS1
....aggaaGuucauaccgugcucu....................	2	1	HS1
....aggaacuucauaUcgugcucu....................	23	1	HS1
....aggaCcuucauaccgugcucu....................	1	1	HS1
....aggaacuucauaccgugcucC....................	2551	1	HS1
....aggaacuuGauaccgugcucu....................	3	1	HS1
....aggaGcuucauaccgugcucu....................	49	1	HS1
....aggaacAucauaccgugcucu....................	9	1	HS1
....aggaacGucauaccgugcucu....................	22	1	HS1
....aggaacuucGuaccgugcucu....................	36	1	HS1
....aggaaUuucauaccgugcucu....................	8	1	HS1
....aggaacuucauaAcgugcucu....................	13	1	HS1
....aggaacuuAauaccgugcucu....................	7	1	HS1
....aggaacuucaAaccgugcucu....................	7	1	HS1
....aggaacuucauaccCugcucu....................	150	1	HS1
....aggaacuucauaccgAgcucu....................	9	1	HS1
....aggaacuucauaccgugGucu....................	7	1	HS1
....aggaacuucauaccgGgcucu....................	31	1	HS1
....aggaacuucauaccgugcuGu....................	3	1	HS1
....aggaacuucUuaccgugcucu....................	5	1	HS1
....aggaacuucauaccguUcucu....................	41	1	HS1
....aggCacuucauaccgugcucu....................	4	1	HS1
....aggaacCucauaccgugcucu....................	68	1	HS1
....aggaacuGcauaccgugcucu....................	11	1	HS1
....aUgaacuucauaccgugcucu....................	17	1	HS1
....aggaacuucauaccAugcucu....................	102	1	HS1
....aggaacuucauaccguCcucu....................	2	1	HS1
....Cggaacuucauaccgugcucu....................	1	1	HS1
....agAaacuucauaccgugcucu....................	15	1	HS1

Star Mature

caaaaaaaaucgaauuacacuucgucacgagcgagguuuguaguuccuaccguuuuuauucaaacuggguaggaacuucauaccgugcucuugaacgaugcugacgaucaa			
....agCaacuucauaccgugcucu....................	1	1	HS1
....aggaacuucauaccgugcAcu....................	18	1	HS1
....aggaacuucauaccgugcucG....................	44	1	HS1
....aCgaacuucauaccgugcucu....................	3	1	HS1
....aggaacuucauaGcgugcucu....................	4	1	HS1
....aggaacuucauaccgugcucu....................	224079	0	HS1
....Uggaacuucauaccgugcucu....................	58	1	HS1
....aggUacuucauaccgugcucu....................	5	1	HS1
....aggaacuucauaccgugcCcu....................	210	1	HS1
....aggaacuucauaccguAcucu....................	405	1	HS1
....aggaacuucauaccgCgcucu....................	27	1	HS1
....Gggaacuucauaccgugcucu....................	97	1	HS1
....aggaacuucauaccgugAucu....................	18	1	HS1
....aAgaacuucauaccgugcucu....................	20	1	HS1
....aggaacuucauacAgugcucu....................	11	1	HS1
....aggaacuuUauaccgugcucu....................	50	1	HS1
....aggaacuucauaccgugcucuu...................	84	0	HS1
....aggaacuucauaccgugcucuC...................	2	1	HS1
....aggaacuucauaccgugcucuA...................	3	1	HS1
....aggaacuucauaccgugcucuuU..................	8	1	HS1
....ggaacuucauaccgugcuc.....................	3	0	HS1
....ggaacuucauaccgugcucu....................	1371	0	HS1
....ggaacuucauGccgugcucu....................	1	1	HS1
....ggaacuucauaccgugcucG....................	2	1	HS1
....ggaacuucauaccgugcucA....................	3	1	HS1
....Agaacuucauaccgugcucu....................	1	1	HS1
....ggaacuucauaccgugcucC....................	16	1	HS1
....ggaacuucauaccgugcCcu....................	1	1	HS1
....ggaacuucauacUgugcucu....................	2	1	HS1
....ggaacuucauaccguAcucu....................	2	1	HS1
....Ugaacuucauaccgugcucu....................	2	1	HS1
....Caacuucauaccgugcucu....................	1	1	HS1
....aacuucauaccgugcucu....................	3	0	HS1
.........ucAaauuacacuucgucacg..	2	1	HS6
..........cAaauuacacuucgucacg..	1	1	HS6
............................Uagcgagguuuguaguuccuac...	1	1	HS6
............................Uagcgagguuuguaguuccuacc..	1	1	HS6
.............................agcgagguuuguaguucc..	21	0	HS6
.............................agcgagguuuguaguuccA...	1	1	HS6
.............................agcgUgguuuguaguuccu...	1	1	HS6
.............................agcgagguuuguaguuccG...	1	1	HS6
.............................agcgagguuuguaguuccu...	292	0	HS6
.............................agcgaggGuuguaguuccua..	1	1	HS6
.............................agcgUgguuuguaguuccua..	1	1	HS6
.............................agcgagguuuguaguuccGa..	1	1	HS6
.............................agcgagguuuguaUuuccua..	1	1	HS6
.............................Ugcgagguuuguaguuccua..	1	1	HS6
.............................agcgagUuuuguaguuccua..	1	1	HS6
.............................agcgagguuuguaguuccuU..	1	1	HS6
.............................agcgagguuugCaguuccua..	1	1	HS6
.............................agcgagguuuguaguuccua..	633	0	HS6
.............................agcgagguuuguaguuccuac...	2832	0	HS6
.............................agcgagguuugCaguuccuac...	1	1	HS6
.............................agcgagguuuguaguuccuaU...	7	1	HS6
.............................agcgagguuuguaguucGuac...	1	1	HS6
.............................agcgagguuuguaguuccGac...	1	1	HS6
.............................agUgagguuuguaguuccuac...	1	1	HS6
.............................agcgagguuuguaguuccuaG...	1	1	HS6
.............................agcgagguuuguaguucUuac...	2	1	HS6
.............................agcgagguuuguaguuccuaA...	2	1	HS6
.............................agcgagguuuguaguuUcuac...	3	1	HS6
.............................agcgaggGuuguaguuccuac...	7	1	HS6
.............................Ggcgagguuuguaguuccuac...	1	1	HS6
.............................agcgagguuuCuaguuccuac...	2	1	HS6
.............................agcgagguuuguagCuccuac...	1	1	HS6
.............................agcgagAuuuguaguuccuac...	1	1	HS6
.............................agcgaUguuuguaguuccuac...	1	1	HS6
.............................aAcgagguuuguaguuccuac...	1	1	HS6
.............................agcgUgguuuguaguuccuac...	1	1	HS6

Star **Mature**

```
caaaaaaaaucgaauuacacuucgucacgagcgagguuuguaguuccuaccguuuuuauucaaacuggguaggaacuucauaccgugcucuugaacgaugcugacgaucaa
.............................Ugcgagguuuguaguuccuac.............................................................  3     1  HS6
.............................agcgagguuuguaguuccAac.............................................................  1     1  HS6
.............................agcgagguuuguaguuccuGc.............................................................  2     1  HS6
.............................agcgagguuuguaguucUuacc............................................................  4     1  HS6
.............................agcgaCguuuguaguuccuacc............................................................  1     1  HS6
.............................agcgagguuuguaguuccuacU............................................................  473   1  HS6
.............................agcgaggCuuguaguuccuacc............................................................  1     1  HS6
.............................agUgagguuuguaguuccuacc............................................................  4     1  HS6
.............................agcgagguuuguaguuccuaUc............................................................  1     1  HS6
.............................agcgagguuugCaguuccuacc............................................................  1     1  HS6
.............................agcgagguuuguGguuccuacc............................................................  1     1  HS6
.............................agcUagguuuguaguuccuacc............................................................  1     1  HS6
.............................agcCagguuuguaguuccuacc............................................................  3     1  HS6
.............................agcgagguuuguaguucGuacc............................................................  1     1  HS6
.............................agcgagguAuguaguuccuacc............................................................  1     1  HS6
.............................Cgcgagguuuguaguuccuacc............................................................  1     1  HS6
.............................agcgagUuuuguaguuccuacc............................................................  2     1  HS6
.............................Ugcgagguuuguaguuccuacc............................................................  1     1  HS6
.............................agcgagguuuguaUuuccuacc............................................................  2     1  HS6
.............................agcgagguuuguaguuccuacc............................................................  3835  0  HS6
.............................agcgagguuuguaguuGcuacc............................................................  1     1  HS6
.............................agcgagguuuguaguuUcuacc............................................................  4     1  HS6
.............................agcgaggGuuguaguuccuacc............................................................  7     1  HS6
.............................agcgGgguuuguaguuccuacc............................................................  1     1  HS6
.............................agcgagguuuguaguCccuacc............................................................  1     1  HS6
.............................agcgagguuuguaguuccuacG............................................................  6     1  HS6
.............................agcgagguuuguaguuccuacA............................................................  252   1  HS6
.............................agcgagguuuguaguuccuaccC...........................................................  8     1  HS6
.............................agcgagguuuguaguuccuaccg...........................................................  53    0  HS6
.............................agcgagguuuguaguuccuaccA...........................................................  85    1  HS6
.............................agcgagUuuuguaguuccuaccg...........................................................  1     1  HS6
.............................agcgagguuuguaguuccuaccU...........................................................  430   1  HS6
.............................agcgagguuuguaguuccuaccgA..........................................................  1     1  HS6
.............................agcgagguuuguaguuccuaccAu..........................................................  1     1  HS6
..............................Acgagguuuguaguuccua..............................................................  1     1  HS6
..............................gcgagguuuguaguuccuac.............................................................  8     0  HS6
..............................gcgagguuuguaguuccuacc............................................................  9     0  HS6
..............................gcgagguuuguaguuccuacA............................................................  1     1  HS6
..............................gcgagguuuguaguuccuaccg...........................................................  9     0  HS6
...............................cgagguuuguaguuccuac.............................................................  2     0  HS6
...............................cgagguuuguaguuccuacc............................................................  3     0  HS6
...............................cgagguuuguaguuccuaccg...........................................................  2     0  HS6
...............................cgagguuuguaguuccuaccC...........................................................  2     1  HS6
................................gagguuuguaguuccuac.............................................................  2     0  HS6
................................gagguuuguaguuccuacc............................................................  6     0  HS6
................................gagguuuguaguuccuacU............................................................  4     1  HS6
................................gagguuuguaguuccuaccU...........................................................  1     1  HS6
................................gagguuuguaguuccuaccA...........................................................  2     1  HS6
.................................agguuuguaguuccuacc............................................................  1     0  HS6
.................................agguuuguaguuccuaccguu.........................................................  1     0  HS6
...................................................guuuuuauucaaacuggg..........................................  2     0  HS6
.................................................................uggCuaggaacuucauaccgugcucu....................  1     1  HS6
..................................................................Agguaggaacuucauaccgugcucu....................  3     1  HS6
..................................................................ggCuaggaacuucauaccgugcucu....................  2     1  HS6
..................................................................ggguaggaacuucauaccgugcucu....................  6     0  HS6
...................................................................Uguaggaacuucauaccgugcu......................  1     1  HS6
...................................................................gCuaggaacuucauaccgugcuc.....................  1     1  HS6
...................................................................gCuaggaacuucauaccgugcucu....................  4     1  HS6
...................................................................gUuaggaacuucauaccgugcucu....................  1     1  HS6
...................................................................gguaggaacuucauaccgugcucu....................  1     0  HS6
....................................................................Uuaggaacuucauaccgug........................  1     1  HS6
....................................................................guaggaacuucauaccgugc.......................  2     0  HS6
....................................................................Uuaggaacuucauaccgugcu......................  2     1  HS6
....................................................................guaggaacuucauaccgugcu......................  21    0  HS6
....................................................................guaggaacuucauaccgugcuc.....................  12    0  HS6
....................................................................Cuaggaacuucauaccgugcuc.....................  1     1  HS6
....................................................................Uuaggaacuucauaccgugcuc.....................  5     1  HS6
....................................................................guaggaacuucauaccgugcuU....................  1     1  HS6
....................................................................Cuaggaacuucauaccgugcucu....................  7     1  HS6
....................................................................Auaggaacuucauaccgugcucu....................  1     1  HS6
```

Star Mature			
caaaaaaaaucgaauuacacuucgucacgagcgagguuuguaguuccuaccguuuuuauucaaacuggguaggaacuucauaccgugcucuugaacgaugcugacgaucaa			
....guaggaacuucauaccgugcucu....................	18	0	HS6
....Uuaggaacuucauaccgugcucu....................	145	1	HS6
....Uuaggaacuucauaccgugcucuu...................	1	1	HS6
....uaggGacuucauaccgug........................	1	1	HS6
....uaggaacuucauaccguU........................	1	1	HS6
....uaggaacuucauaccgug........................	122	0	HS6
....uaggaacuucauaccguA........................	1	1	HS6
....uaggaacuucauaUcgugc.......................	1	1	HS6
....uaggaacuucauaccgugU.......................	4	1	HS6
....uaggaacuucauaccUugc.......................	1	1	HS6
....uaggaacuucauaccgugA.......................	23	1	HS6
....uaggaacuucauaccgugc.......................	93	0	HS6
....uaggaacuuGauaccgugcu......................	1	1	HS6
....uaCgaacuucauaccgugcu......................	1	1	HS6
....uaggaacuucauaccgugcA......................	1	1	HS6
....uaggaacuucauaccguUcu......................	13	1	HS6
....uagCaacuucauaccgugcu......................	1	1	HS6
....uaggaacuucauacUgugcu......................	1	1	HS6
....uaggaacuucauaccguCcu......................	11	1	HS6
....uaggaacuucauaccgugUu......................	1	1	HS6
....uaggUacuucauaccgugcu......................	1	1	HS6
....uaggaacuuUauaccgugcu......................	2	1	HS6
....uaggaacuucauaccgugcu......................	1685	0	HS6
....uaggaacuucauaccgugcG......................	6	1	HS6
....uaggaaUuucauaccgugcu......................	1	1	HS6
....Aaggaacuucauaccgugcu......................	3	1	HS6
....uaggaacGucauaccgugcu......................	1	1	HS6
....Gaggaacuucauaccgugcu......................	1	1	HS6
....uaggaacuucauaUcgugcu......................	1	1	HS6
....uaggaacAucauaccgugcuc.....................	2	1	HS6
....uaggaacuucauaUcgugcuc.....................	9	1	HS6
....uaggaacuucauaccgGgcuc.....................	2	1	HS6
....uaggaacuucauaccgugUuc.....................	4	1	HS6
....uaggaacuucauaccgCgcuc.....................	2	1	HS6
....uaCgaacuucauaccgugcuc.....................	3	1	HS6
....uagCaacuucauaccgugcuc.....................	3	1	HS6
....uaggaacuucauaccguAcuc.....................	7	1	HS6
....uUggaacuucauaccgugcuc.....................	1	1	HS6
....uagUaacuucauaccgugcuc.....................	3	1	HS6
....uaggaacuucaCaccgugcuc.....................	1	1	HS6
....uGggaacuucauaccgugcuc.....................	5	1	HS6
....uaggGacuucauaccgugcuc.....................	4	1	HS6
....uaggaaAuucauaccgugcuc.....................	1	1	HS6
....uaggaacuucauaccguCcuc.....................	3	1	HS6
....uaggaacuucauacUgugcuc.....................	7	1	HS6
....uaggaacuucauaccgugcuU.....................	159	1	HS6
....uaggaacuucauaccgugcuA.....................	33	1	HS6
....uaggaacuucauaccguUcuc.....................	3	1	HS6
....uaggUacuucauaccgugcuc.....................	5	1	HS6
....uaggaacuucauaGcgugcuc.....................	1	1	HS6
....uaggaCcuucauaccgugcuc.....................	1	1	HS6
....uaggaUcuucauaccgugcuc.....................	1	1	HS6
....uaAgaacuucauaccgugcuc.....................	1	1	HS6
....uaggaacuucauaccgugcGc.....................	2	1	HS6
....uaggaacuucauaccgugcuc.....................	10945	0	HS6
....uaggaacuucauaccAugcuc.....................	7	1	HS6
....Gaggaacuucauaccgugcuc.....................	1	1	HS6
....uaggaacCucauaccgugcuc.....................	1	1	HS6
....uaggaacuuGauaccgugcuc.....................	2	1	HS6
....uaggaacuucauaccgugcCc.....................	5	1	HS6
....uaggaacuucCuaccgugcuc.....................	1	1	HS6
....uaggaacuGcauaccgugcuc.....................	2	1	HS6
....uaggaacuucGuaccgugcuc.....................	2	1	HS6
....Aaggaacuucauaccgugcuc.....................	6	1	HS6
....uaggaacuucauGccgugcuc.....................	1	1	HS6
....uaggaacuucaAaccgugcuc.....................	1	1	HS6
....uaggCacuucauaccgugcuc.....................	1	1	HS6
....uaUgaacuucauaccgugcuc.....................	2	1	HS6
....uaggaacuCcauaccgugcuc.....................	5	1	HS6
....uaggaacuucGuaccgugcucu....................	57	1	HS6

```
                              Star                                    Mature
caaaaaaaaucgaauuacacuucgucacgagcgagguuuguaguuccuaccguuuuuauucaaacuggguaggaacuucauaccgugcucuugaacgaugcugacgaucaa
.....................................................................uaggaacuucauCccgugcucu....................  8       1   HS6
.....................................................................uaggaacuucauaGcgugcucu....................  8       1   HS6
.....................................................................uaggaacAucauaccgugcucu....................  44      1   HS6
.....................................................................uaggaacuucauaccAugcucu....................  133     1   HS6
.....................................................................uaggaacuucauaccgugcuUu....................  99      1   HS6
.....................................................................uaggaacuuAauaccgugcucu....................  5       1   HS6
.....................................................................uaAgaacuucauaccgugcucu....................  18      1   HS6
.....................................................................uaggaacuucauaccgugUucu....................  314     1   HS6
.....................................................................uaUgaacuucauaccgugcucu....................  268     1   HS6
.....................................................................uaggaacuucCuaccgugcucu....................  92      1   HS6
.....................................................................uaggaacuucauaccCugcucu....................  32      1   HS6
.....................................................................uaggaUcuucauaccgugcucu....................  4       1   HS6
.....................................................................uaggaacuucauaccguAcucu....................  115     1   HS6
.....................................................................uaggCacuucauaccgugcucu....................  122     1   HS6
.....................................................................uaggUacuucauaccgugcucu....................  334     1   HS6
.....................................................................uaggaacuucauaccgugcucG....................  341     1   HS6
.....................................................................uaggaacuCcauaccgugcucu....................  117     1   HS6
.....................................................................uaggGacuucauaccgugcucu....................  217     1   HS6
.....................................................................uaggaacuucauaUcgugcucu....................  182     1   HS6
.....................................................................uaggaacuucauacAgugcucu....................  10      1   HS6
.....................................................................uaggaacuucauaccguCcucu....................  41      1   HS6
.....................................................................uaggaaUuucauaccgugcucu....................  89      1   HS6
.....................................................................Gaggaacuucauaccgugcucu....................  23      1   HS6
.....................................................................uaggaacuucauaccgGgcucu....................  40      1   HS6
.....................................................................uaggaGcuucauaccgugcucu....................  25      1   HS6
.....................................................................Aaggaacuucauaccgugcucu....................  96      1   HS6
.....................................................................uaggaacuucUuaccgugcucu....................  14      1   HS6
.....................................................................uaggaCcuucauaccgugcucu....................  13      1   HS6
.....................................................................uGggaacuucauaccgugcucu....................  103     1   HS6
.....................................................................uagUaacuucauaccgugcucu....................  77      1   HS6
.....................................................................uaggaacuucauUccgugcucu....................  8       1   HS6
.....................................................................uaggaacuucauaccgugcucC....................  1570    1   HS6
.....................................................................uaggaacGucauaccgugcucu....................  19      1   HS6
.....................................................................uaggaaGuucauaccgugcucu....................  32      1   HS6
.....................................................................uaggaacuucauaccgAgcucu....................  9       1   HS6
.....................................................................uaggaacuucauaccgugcucA....................  84      1   HS6
.....................................................................uagAaacuucauaccgugcucu....................  26      1   HS6
.....................................................................uaggaacuGcauaccgugcucu....................  14      1   HS6
.....................................................................uaggaacuucaGaccgugcucu....................  3       1   HS6
.....................................................................uaggaacuucauaccUugcucu....................  28      1   HS6
.....................................................................uagCaacuucauaccgugcucu....................  271     1   HS6
.....................................................................uaggaacuucauaccgugcucu....................  522978  0   HS6
.....................................................................uaggaacuucauGccgugcucu....................  13      1   HS6
.....................................................................uaggaacuucauacUgugcucu....................  159     1   HS6
.....................................................................uaggaacuucauaccgugcCcu....................  46      1   HS6
.....................................................................uaggaacuucauacGgugcucu....................  5       1   HS6
.....................................................................uaggaacuucaCaccgugcucu....................  29      1   HS6
.....................................................................uaCgaacuucauaccgugcucu....................  115     1   HS6
.....................................................................uaggaacuucauaccgugcuGu....................  24      1   HS6
.....................................................................Caggaacuucauaccgugcucu....................  54      1   HS6
.....................................................................uaggaacuucauaAcgugcucu....................  6       1   HS6
.....................................................................uCggaacuucauaccgugcucu....................  8       1   HS6
.....................................................................uaggaacuuGauaccgugcucu....................  16      1   HS6
.....................................................................uaggaacuucauaccgugAucu....................  9       1   HS6
.....................................................................uaggaaAuucauaccgugcucu....................  21      1   HS6
.....................................................................uaggaacuuUauaccgugcucu....................  187     1   HS6
.....................................................................uaggaacuucauaccgugcuAu....................  24      1   HS6
.....................................................................uaggaacuucauaccguUcucu....................  96      1   HS6
.....................................................................uaggaacuucauaccgugcGcu....................  17      1   HS6
.....................................................................uaggaacuucauaccgugGucu....................  47      1   HS6
.....................................................................uaggaacCucauaccgugcucu....................  49      1   HS6
.....................................................................uaggaacuucaAaccgugcucu....................  1       1   HS6
.....................................................................uaggaacuAcauaccgugcucu....................  20      1   HS6
.....................................................................uUggaacuucauaccgugcucu....................  72      1   HS6
.....................................................................uaggaacuucauaccgCgcucu....................  57      1   HS6
.....................................................................uaggaacuucauaccgugcAcu....................  11      1   HS6
.....................................................................uGggaacuucauaccgugcucuu...................  2       1   HS6
.....................................................................uagCaacuucauaccgugcucuu...................  3       1   HS6
.....................................................................Aaggaacuucauaccgugcucuu...................  3       1   HS6
.....................................................................Caggaacuucauaccgugcucuu...................  1       1   HS6
```

Star Mature

```
caaaaaaaaucgaauuacacuucgucacgagcgagguuuguaguuccuaccguuuuuauucaaacuggguaggaacuucauaccgugcucuugaacgaugcugacgaucaa
.....................................................................uaggUacuucauaccgugcucuu...................   1      1      HS6
.....................................................................uaUgaacuucauaccgugcucuu...................   1      1      HS6
.....................................................................uaggaacuucauaccgugUucuu...................   2      1      HS6
.....................................................................uaggaacuucauaccgugcucuC...................   18     1      HS6
.....................................................................uaCgaacuucauaccgugcucuu...................   1      1      HS6
.....................................................................uaggaacuCcauaccgugcucuu...................   1      1      HS6
.....................................................................uaggaacAucauaccgugcucuu...................   1      1      HS6
.....................................................................uaggaacuucauaccgugcuUuu...................   3      1      HS6
.....................................................................uaggaacuucauaccgugcucuG...................   10     1      HS6
.....................................................................uaggaacuuUauaccgugcucuu...................   2      1      HS6
.....................................................................uaggaacuucauaccCugcucuu...................   1      1      HS6
.....................................................................uaggaacuucauaccgugcucuu...................   2805   0      HS6
.....................................................................uaggaacuucauaccAugcucuu...................   1      1      HS6
.....................................................................uaggaacuucauaccgugcucCu...................   24     1      HS6
.....................................................................uaggaacuucauaccgugcucuA...................   578    1      HS6
.....................................................................uaggaacuucauaccgugcucuCg..................   1      1      HS6
.....................................................................uaggaacuucauaccgugcucuug..................   7      0      HS6
.....................................................................uaggaacuucauaccgugcucuuC..................   1      1      HS6
.....................................................................uaggaacuucauaccgugcucuGg..................   8      1      HS6
.....................................................................uaggaacuucauaccgugcucuuA..................   10     1      HS6
.....................................................................uaggaacuucauaccgugcucuAg..................   1      1      HS6
.....................................................................uaggaacuucauaccgugcucuuU..................   38     1      HS6
.....................................................................uaggaacuucauaccgugcucuugU.................   1      1      HS6
.....................................................................uaggaacuucauaccgugcucuGga.................   1      1      HS6
......................................................................aggaacuucauaccgugcu......................   5      0      HS6
......................................................................aggaacuucauaccgugcuc.....................   24     0      HS6
......................................................................aggaacuucauaccgugcucA....................   1      1      HS6
......................................................................agAaacuucauaccgugcucu....................   1      1      HS6
......................................................................aggaacuucauaccgugUucu....................   1      1      HS6
......................................................................aggGacuucauaccgugcucu....................   2      1      HS6
......................................................................aggaacuucauaUcgugcucu....................   1      1      HS6
......................................................................aggaUcuucauaccgugcucu....................   1      1      HS6
......................................................................aggaacuucGuaccgugcucu....................   1      1      HS6
......................................................................Uggaacuucauaccgugcucu....................   3      1      HS6
......................................................................aCgaacuucauaccgugcucu....................   4      1      HS6
......................................................................aggaacuucauaccgugcucu....................   1100   0      HS6
......................................................................Gggaacuucauaccgugcucu....................   3      1      HS6
......................................................................aUgaacuucauaccgugcucu....................   5      1      HS6
......................................................................aggaacuucauaccgugcGcu....................   1      1      HS6
......................................................................aggaacuucauaccgugcucC....................   5      1      HS6
......................................................................agUaacuucauaccgugcucu....................   3      1      HS6
......................................................................aggaacuucauaccgugcucuA...................   1      1      HS6
......................................................................aggaacuucauaccgugcucuu...................   7      0      HS6
.......................................................................ggaacuucauaccgugcuc.....................   3      0      HS6
.......................................................................Agaacuucauaccgugcucu....................   3      1      HS6
.......................................................................Ugaacuucauaccgugcucu....................   13     1      HS6
.......................................................................ggaacuucauaccgugcucu....................   101    0      HS6
.......................................................................gUaacuucauaccgugcucu....................   1      1      HS6
.......................................................................gAaacuucauaccgugcucu....................   1      1      HS6
.......................................................................ggGacuucauaccgugcucu....................   1      1      HS6
.......................................................................ggaacuucauaccgugcucuu...................   1      0      HS6
........................................................................gaacuucauaccgugcuc.....................   1      0      HS6
........................................................................gaacuucauaccAugcuc.....................   1      1      HS6
........................................................................gaacuucauaccgugcucC....................   1      1      HS6
........................................................................Caacuucauaccgugcucu....................   1      1      HS6
........................................................................Uaacuucauaccgugcucu....................   12     1      HS6
........................................................................gaacuucauaccgugcucu....................   80     0      HS6
.........................................................................aacuucauaccgugcucC....................   1      1      HS6
.........................................................................aacGucauaccgugcucu....................   1      1      HS6
.........................................................................aGcuucauaccgugcucu....................   1      1      HS6
.........................................................................aacuucauaccgugcucu....................   605    0      HS6
.........................................................................aacuucauaccgugcucuA...................   1      1      HS6
.........................................................................aacuucauaccgugcucuu...................   4      0      HS6
..........................................................................acuucauaccgugcucuA...................   1      1      HS6
```

Provisional ID	:	SczTNLB_3097_7421
Score total	:	238.4
Score for star read(s)	:	3.9
Score for read counts	:	228.5
Score for mfe	:	1.4
Score for randfold	:	1.6
Score for cons. seed	:	3
Total read count	:	460
Mature read count	:	364
Loop read count	:	0
Star read count	:	96

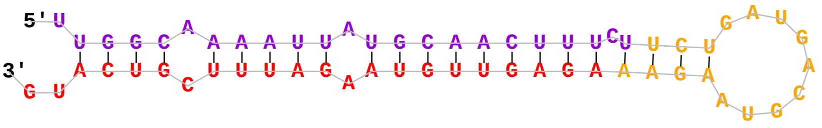

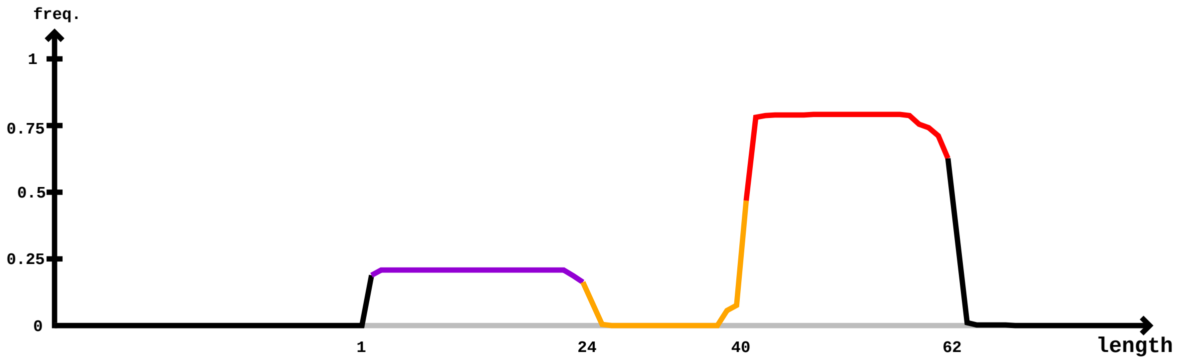

Star Mature

```
5'- uugucacucgcacaaauaaacuuugaccauuuuggcaaaauuaugcaacuuucuucugaugacguaagaaagaguuguaagauuucgucaugaugguuaaaguuauuucaga   -3'   obs
    uugucacucgcacaaauaaacuuugaccauuuuggcaaaauuaugcaacuuucuucugaugacguaagaaagaguuguaagauuucgucaugaugguuaaaguuauuucaga         exp
    ..................(((((((((((((.((((.(((((.(((((((((.((((.........))))))))))))).))))).)))).)))))))))))))........   reads   mm   sample
    ...............................uuggcaaaauuaugcaacuuuc...........................................................   2   0   HS6
    ...............................uuggcaaaauuaugcaacuuucu..........................................................   2   0   HS6
    ....................................................................aaagaguuguaagauuucguc.......................   2   0   HS6
    .....................................................................aagaguuguaagauuucgu........................   1   0   HS6
    .....................................................................aagaguuguaagauuucguca......................   1   0   HS6
    ......................................................................agaguuguaagauuucguca......................   1   0   HS6
    ......................................................................agaguuguaagauuucgucauU....................   1   1   HS6
    ......................................................................agaguuguaagauuucgucaug....................   8   0   HS6
    .......................................................................gaguuguaagauuucgucaug....................   3   0   HS6
    .......................................................................gaguuguaagauuucgucauga...................   8   0   HS6

    ...............................uuggcaaaauuGugcaacuuu............................................................   7   1   HA1
    ...............................uuggcaaaauuGugcaacuuuc...........................................................   2   1   HA1
    ...............................uuggcaaaauuGugcaacuuucu..........................................................   2   1   HA1
    ...............................uuggcaaaauuGugcaacuuucuu.........................................................   1   1   HA1
    ................................uggcaaaauuGugcaacuuucu..........................................................   1   1   HA1
    ................................uggcaaaauuGugcaacuuucuu.........................................................   1   1   HA1
    .......................................................................gaguuguaagauuuGguc.......................   1   1   HA1
    .......................................................................gaguuguaagauuuGgucau.....................   2   1   HA1
    .......................................................................gaguuguaagauuuGgucauga...................   4   1   HA1

    ...............................uuggcaaaauuGugcaacuuuc...........................................................   3   1   HS1
    ...............................uuggcaaaauuaugcaacuuucu..........................................................   1   0   HS1
    ...............................uuggcaaaauuGugcaacuuucu..........................................................   32   1   HS1
    ...............................uuggcaaaauuGugcaacuuucuu.........................................................   14   1   HS1
    ...............................uuggcaaaauuGugcaacuuucuuc........................................................   2   1   HS1
    ................................uggcaaaauuUugcaacuuu............................................................   1   1   HS1
    ................................uggcaaaauuGugcaacuuucu..........................................................   4   1   HS1
    ......................................................................agaguuguaagauuucgucaug....................   11   0   HS1
    .......................................................................gaguuguaagauuucgucauga...................   2   0   HS1

    ...............................uuggcaaaauuaugcaacuuucu..........................................................   1   0   HS7
    ....................................................................aaagaguuguaagauuucgu........................   1   0   HS7
```

Star Mature

```
uugucacucgcacaaauaaacuuugaccauuuuggcaaaauuaugcaacuuucuucugaugacguaagaaagaguuguaagauuucgucaugaugguuaaaguuauuucaga
......................................................................agaguuguaagauuucgucau......................    6    0    HS7
......................................................................agaguuguaagauuucgucauU.....................    3    1    HS7
......................................................................agaguuguaagauuucgucaug....................    3    0    HS7
......................................................................agaguuguaagauuucgucaugU...................    1    1    HS7
......................................................................agaguuguaagauuucgucauga...................    1    0    HS7
.......................................................................gaguuguaagauuucguca......................    1    0    HS7
.......................................................................gaguuguaagauuucgucauga...................    18   0    HS7
.......................................................................gaguuguaagauuucgucaugaA..................    1    1    HS7

...............................uuggcaaaauuGugcaacuuuc...........................................................    1    1    HS5
...............................uuggcaaaauuaugcaacuuucu..........................................................    3    0    HS5
...............................uuggcaaaauuGugcaacuuucuu........................................................    1    1    HS5
...............................uuggcaaaauuaugcaacuuucuu........................................................    1    0    HS5
......................................................................agaguuguaagauuucgucau......................    3    0    HS5
......................................................................agaguuguaagauCcgucaug....................    1    1    HS5
......................................................................agaguuguaagauuucgucauU.....................    2    1    HS5
......................................................................agaguuguaagauuucgucaug....................    11   0    HS5
......................................................................agaguuguaagauuucgucauga...................    1    0    HS5
.......................................................................gaguuguaagauuuGgucaug....................    2    1    HS5
.......................................................................gaguuguaagauuuUgucauga...................    1    1    HS5
.......................................................................gaguuguaagauuucgucauga...................    2    0    HS5

....................................................................aaagaguuguaagauuucg.........................    1    0    HFA
......................................................................agaguuguaagauuucgucau......................    1    0    HFA
......................................................................agaguuguaagauuucgucaug....................    4    0    HFA
......................................................................agaguuguaagauuucgucauU.....................    2    1    HFA
......................................................................agaguuguaagauuucgucaugU...................    1    1    HFA
.......................................................................gaguuguaagauuucgucaug....................    1    0    HFA
.......................................................................gaguuguaagauuucgucauga...................    8    0    HFA
.......................................................................gaguuguaagauuucgucaugaA..................    1    1    HFA
.........................................................................aguuguaagauuucgucaugaA.................    1    1    HFA
..........................................................................guuguaagauuucgucaug...................    1    0    HFA

...............................uuggcaaaauuGugcaacuuu............................................................    1    1    HS4
...............................uuggcaaaauuaugcaacuuuc...........................................................    1    0    HS4
...............................uuggcaaaauuGugcaacuuuc...........................................................    1    1    HS4
...............................uuggcaaaauuGugcaacuuucu..........................................................    1    1    HS4
...............................uuggcaaaauuaugcaacuuucu..........................................................    2    0    HS4
...............................uuggcaaaauuaugcaacuuucuu........................................................    1    0    HS4
....................................................................aaagaguuguaagauuucg.........................    1    0    HS4
....................................................................aaagaguuguaagauuucgu........................    10   0    HS4
....................................................................aaagaguuguaagauuucguc.......................    1    0    HS4
....................................................................aaagaguuguaagauuucguca......................    2    0    HS4
....................................................................aaagaguuguaagauuucgucau.....................    1    0    HS4
.....................................................................aagaguuguaagauuucgu........................    1    0    HS4
.....................................................................aagaguuguaagauuucgucau.....................    2    0    HS4
......................................................................agaguuguaagauuucguca.......................    1    0    HS4
......................................................................agaguuguaagauuucgucau......................    10   0    HS4
......................................................................agaguuguaagauuucgucauU.....................    4    1    HS4
......................................................................agaguuguaagauuucgucauA.....................    1    1    HS4
......................................................................agaguuguaaUauuucgucaug....................    1    1    HS4
......................................................................agaguuguaagauuucgucaug....................    29   0    HS4
......................................................................agaguuguaagauuucgucauUa....................    1    1    HS4
......................................................................agaguuguaagauuucgucauga...................    2    0    HS4
.......................................................................gaguuguaagauuucguca......................    1    0    HS4
.......................................................................gaguuguaagauuuGgucaug....................    1    1    HS4
.......................................................................gaguuguaagauuucgucaug....................    3    0    HS4
.......................................................................gaguuguaagauuucgucaugU...................    1    1    HS4
.......................................................................gaguuguaagauuuGgucauga...................    2    1    HS4
.......................................................................gaguuguaagauuucgucauga...................    21   0    HS4
.......................................................................gaguuguaagauuucgucaugaA..................    1    1    HS4
.............................................................................uaagauuucgucaugaugguu..............    1    0    HS4

...............................uuggcaaaauuGugcaacuuu............................................................    1    1    J17
...............................uuggcaaaauuGugcaacuuuc...........................................................    1    1    J17
...............................uuggcaaaauuaugcaacuuucu..........................................................    1    0    J17
...............................uuggcaaaauuGugcaacuuucu..........................................................    1    1    J17
...............................uuggcaaaauuaugcaacuuucuu........................................................    1    0    J17
................................uggcaaaauuaugcaacuuucu..........................................................    1    0    J17
....................................................................aaagaguuguaagauuucgu........................    2    0    J17
```

Star Mature

```
uugucacucgcacaaauaaacuuugaccauuuuggcaaaauuaugcaacuuucuucugaugacguaagaaagaguuguaagauuucgucaugaugguuaaaguuauuucaga
....................................................................aaagaguuguaagauuucguc.......................	1	0	J17
....................................................................aaagaguuguaagauuucguca......................	3	0	J17
....................................................................aaagaguuguaagauuucgucaug....................	1	0	J17
.....................................................................aagaguuguaagauuucguc.......................	1	0	J17
.....................................................................aagaguuguaagauuucgucau.....................	1	0	J17
.....................................................................aagaguuguaagauuucgucaug....................	1	0	J17
.....................................................................aagaguuguaagauuucgucauU....................	1	1	J17
......................................................................agaguuguaagauuucguca......................	4	0	J17
......................................................................agaguuguaagauuucgucau.....................	11	0	J17
......................................................................agaguuguaagauuucgucauU....................	8	1	J17
......................................................................agaguuguaagauuucgucaug....................	43	0	J17
......................................................................agaguuguaagauuucgucauga...................	5	0	J17
.......................................................................gaguuguaagauuucgucau.....................	2	0	J17
.......................................................................gaguuguaagauuucgucaug....................	10	0	J17
.......................................................................gaguuguaagauuuGgucaug....................	4	1	J17
.......................................................................gaguuguaagauuuGgucauga...................	2	1	J17
.......................................................................gaguuguaagauuAcgucauga...................	1	1	J17
.......................................................................gaguuguaagauuucgucauga...................	40	0	J17
........................................................................aguuguaagauuucgucaug....................	1	0	J17
........................................................................aAuuguaagauuucgucauga...................	1	1	J17

................................uggcaaaauuaugcaacuuucu..........................................................	1	0	EGG
```

Provisional ID : SczTNLB_3097_9654
Score total : 22237819.9
Score for star read(s) : 3.9
Score for read counts : 22237809.8
Score for mfe : 1.5
Score for randfold : 1.6
Score for cons. seed : 3
Total read count : 43618517
Mature read count : 43595867
Loop read count : 5
Star read count : 22645

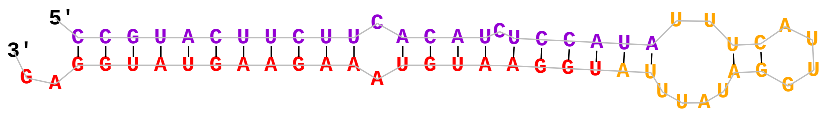

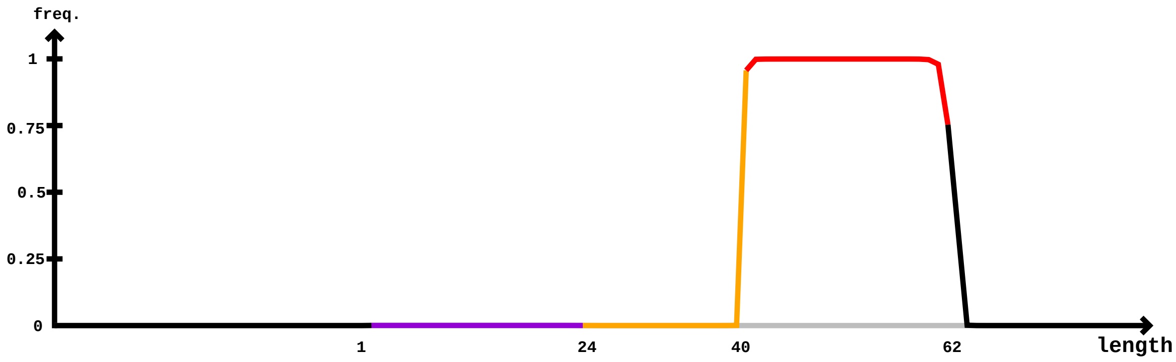

Star Mature

```
5'- acauuucucaaucggugcccucuaguggaucccguacuucuucacaucuccauauuucauuggauauuuauggaauguaaagaaguauggaguuuacuaauguaaaacgagg -3' obs
    acauuucucaaucggugcccucuaguggaucccguacuucuucacaucuccauauuucauuggauauuuauggaauguaaagaaguauggaguuuacuaauguaaaacgagg     exp
    ...........(((.(((....(((((((..(((((((((((.((((.((((((..((....))....))))))))))).)))))))))))..)))))))..)))...)))..   reads mm sample
    ...........ucggugcccucuaguggauc.................................................................................   3  0  HS5
    .............................uUccguacuucuucacaucuccaua..........................................................   1  1  HS5
    ..............................cccguacuucuucacaucucc.............................................................   4  0  HS5
    ..............................cccguacuucuucacaucucca............................................................   37 0  HS5
    ..............................cccguacuucuGcacaucuccau...........................................................   1  1  HS5
    ..............................cccguacuucuucacaucuccau...........................................................   23 0  HS5
    ..............................cccguacuucuucacaucuccaua..........................................................   25 0  HS5
    ..............................cccguacuucuucacaucuccauU..........................................................   2  1  HS5
    ..............................cccguacuucuucacaucuccauau.........................................................   2  0  HS5
    ...............................ccguacuucuucacaucu...............................................................   1  0  HS5
    ...............................ccguacuucuucacaucucc.............................................................   3  0  HS5
    ...............................ccguacuucuucacaucCcca............................................................   1  1  HS5
    ...............................ccguacuucuuUacaucucca............................................................   1  1  HS5
    ...............................ccguacuucuucGcaucucca............................................................   1  1  HS5
    ...............................ccUuacuucuucacaucucca............................................................   1  1  HS5
    ...............................ccguacuucuucacaucuUca............................................................   2  1  HS5
    ...............................ccguacuucuucUcaucucca............................................................   1  1  HS5
    ...............................ccguacuucuucacauUucca............................................................   1  1  HS5
    ...............................ccguacuucuucacaucucca............................................................   425 0 HS5
    ...............................ccguacuucuucacaAcucca............................................................   1  1  HS5
    ...............................ccguacuucuucacaCcuccau...........................................................   1  1  HS5
    ...............................ccguacuucuucacauUuccau...........................................................   1  1  HS5
    ...............................ccguacuucuucaUaucuccau...........................................................   1  1  HS5
    ...............................ccguacuucuucacaucuccau...........................................................   220 0 HS5
    ...............................ccguacuucuucacauUuccaua..........................................................   30 1  HS5
    ...............................ccguacuucuucacaucuccauU..........................................................   253 1 HS5
    ...............................ccguacuucuucacaucuccauG..........................................................   4  1  HS5
    ...............................ccguacuucuuGacaucuccaua..........................................................   1  1  HS5
    ...............................ccguGcuucuucacaucuccaua..........................................................   1  1  HS5
    ...............................ccAuacuucuucacaucuccaua..........................................................   3  1  HS5
    ...............................ccguacuucuucacaucuccaua..........................................................   3302 0 HS5
    ...............................ccguaGuucuucacaucuccaua..........................................................   2  1  HS5
    ...............................cGguacuucuucacaucuccaua..........................................................   1  1  HS5
```

Star **Mature**

```
acauuucucaaucggugcccucuaguggaucccguacuucuucacaucuccauauuucauuggauauuuauggaauguaaagaaguauggaguuuacuaauguaaaacgagg
...............................ccUuacuucuucacaucuccaua..........................................................	2	1	HS5
...............................ccguacuAcuucacaucuccaua..........................................................	1	1	HS5
...............................Acguacuucuucacaucuccaua..........................................................	2	1	HS5
...............................ccguacuucuucacaucuccauC..........................................................	2	1	HS5
...............................ccguaUuucuucacaucuccaua..........................................................	2	1	HS5
...............................ccguacuucuucacaCcuccaua..........................................................	1	1	HS5
...............................ccguacuucuucacaucuAcaua..........................................................	1	1	HS5
...............................ccguacuucuucacaucucUaua..........................................................	3	1	HS5
...............................cUguacuucuucacaucuccaua..........................................................	1	1	HS5
...............................ccguacuuGuucacaucuccaua..........................................................	1	1	HS5
...............................ccguacuucuucacaucuccCua..........................................................	1	1	HS5
...............................ccguacuuUuucacaucuccaua..........................................................	3	1	HS5
...............................ccguacuucuucacaucCccaua..........................................................	2	1	HS5
...............................ccguacuucuucacaAcuccaua..........................................................	1	1	HS5
...............................ccguacuucuucacaGcuccaua..........................................................	2	1	HS5
...............................ccguacuucuucacaucuccaCa..........................................................	1	1	HS5
...............................ccguacuucuucUcaucuccaua..........................................................	1	1	HS5
...............................ccguacuucuucacauGuccaua..........................................................	2	1	HS5
...............................ccguacuCcuucacaucuccaua..........................................................	1	1	HS5
...............................ccguacuucuucacaucuUcaua..........................................................	18	1	HS5
...............................ccCuacuucuucacaucuccaua..........................................................	1	1	HS5
...............................ccguacuucuucacaucuUcauau.........................................................	1	1	HS5
...............................ccguacuucuucacaucuccauau.........................................................	9	0	HS5
...............................ccguacuucuucacaucuccauUu.........................................................	3	1	HS5
...............................ccguacuucuucacaucuccauaA.........................................................	39	1	HS5
...............................ccguacuucuucacaucuccauaG.........................................................	1	1	HS5
...............................ccguacuucuucacaucuccauauu........................................................	3	0	HS5
...............................ccguacuucuucacaucuccauauA........................................................	2	1	HS5
...............................ccguacuucuucacaucuccauaAuu.......................................................	1	1	HS5
................................cgGacuucuucacaucucca............................................................	1	1	HS5
................................cguacuucuucacaucuccaua..........................................................	11	0	HS5
............................................................uggauauuuauggaauguaa................................	1	0	HS5
................................................................uauuuauggaauguaaagaagu..........................	1	0	HS5
.................................................................auuuauggaauguaaagaagu..........................	2	0	HS5
.................................................................auuuauggaauguaaagaagua.........................	2	0	HS5
..................................................................Cuuauggaauguaaagaaguaugga.....................	1	1	HS5
...................................................................uuauggaauguaaagaagua.........................	5	0	HS5
...................................................................uuauggaauguaaagaaguau........................	1	0	HS5
...................................................................uuauggaauguaaagaaguaug.......................	4	0	HS5
...................................................................uCauggaauguaaagaaguauggag....................	1	1	HS5
...................................................................Cuauggaauguaaagaaguauggag....................	1	1	HS5
....................................................................uauggaauguaaagaaguaug.......................	4	0	HS5
....................................................................uaugAaauguaaagaaguaug.......................	1	1	HS5
....................................................................uauggaauguaaagaaguaugg......................	1	0	HS5
....................................................................uUuggaauguaaagaaguaugg......................	1	1	HS5
....................................................................uUuggaauguaaagaaguaugga.....................	6	1	HS5
....................................................................uauggaauguaaagaaguaugga.....................	1	0	HS5
....................................................................uauggaauguaaagaaguauggag....................	1	0	HS5
....................................................................uUuggaauguaaagaaguauggag....................	91	1	HS5
....................................................................uCuggaauguaaagaaguauggag....................	1	1	HS5
....................................................................Aauggaauguaaagaaguauggag....................	1	1	HS5
....................................................................uauggaauguaaagaaguauggagA...................	1	1	HS5
....................................................................uUuggaauguaaagaaguauggagu...................	5	1	HS5
.....................................................................Guggaauguaaagaaguaug.......................	2	1	HS5
.....................................................................auggaauguaaagaaguaugg......................	12	0	HS5
.....................................................................Uuggaauguaaagaaguaugg......................	14	1	HS5
.....................................................................Cuggaauguaaagaaguaugga.....................	2	1	HS5
.....................................................................Uuggaauguaaagaaguaugga.....................	50	1	HS5
.....................................................................auggaauguaaagaaguauggU.....................	2	1	HS5
.....................................................................auggaauguaaagaaguaugga.....................	17	0	HS5
.....................................................................Guggaauguaaagaaguaugga.....................	1	1	HS5
.....................................................................auCgaauguaaagaaguauggag....................	1	1	HS5
.....................................................................auggaauguaaagaaguauggag....................	54	0	HS5
.....................................................................Uuggaauguaaagaaguauggag....................	645	1	HS5
.....................................................................auggaauguaaagaaCuauggag....................	1	1	HS5
.....................................................................aAggaauguaaagaaguauggag....................	1	1	HS5
.....................................................................Guggaauguaaagaaguauggag....................	8	1	HS5
.....................................................................Cuggaauguaaagaaguauggag....................	16	1	HS5
.....................................................................auggaauguaaagaaguauggaA....................	1	1	HS5
.....................................................................Uuggaauguaaagaaguauggagu...................	28	1	HS5
```

Star Mature

```
acauuucucaaucggugcccucuaguggaucccguacuucuucacaucuccauauuucauuggauauuuauggaauguaaagaaguauggaguuuacuaauguaaaacgagg
.....................................................................auggaauguaaagaaguauggagA...................  9     1  HS5
.....................................................................auggaauguaaagaaguauggagu...................  7     0  HS5
......................................................................uggaauguaaaUaaguau........................  1     1  HS5
......................................................................Aggaauguaaagaaguau........................  3     1  HS5
......................................................................uggaauguGaagaaguau........................  1     1  HS5
......................................................................uggaauguaaagaaguaG........................  10    1  HS5
......................................................................Cggaauguaaagaaguau........................  1     1  HS5
......................................................................uUgaauguaaagaaguau........................  1     1  HS5
......................................................................ugCaauguaaagaaguau........................  1     1  HS5
......................................................................uggaauguaaagaagCau........................  1     1  HS5
......................................................................uggaauguaaagaaAuau........................  1     1  HS5
......................................................................uggaauguaaagaaguau........................  548   0  HS5
......................................................................uggaauguaaagaagAau........................  3     1  HS5
......................................................................Gggaauguaaagaaguau........................  1     1  HS5
......................................................................uggaauguUaagaaguau........................  1     1  HS5
......................................................................uggaauguaaagaaguaGg.......................  2     1  HS5
......................................................................uggaauguaaagaaguauA.......................  4     1  HS5
......................................................................uggaauguaaagaaguGug.......................  1     1  HS5
......................................................................uggaauguaaagaagCaug.......................  1     1  HS5
......................................................................ugUaauguaaagaaguaug.......................  3     1  HS5
......................................................................uggaauguaaagaaguauU.......................  2     1  HS5
......................................................................Gggaauguaaagaaguaug.......................  2     1  HS5
......................................................................uggaaugCaaagaaguaug.......................  1     1  HS5
......................................................................uggaauguaaaCaaguaug.......................  1     1  HS5
......................................................................uggaauguaGagaaguaug.......................  1     1  HS5
......................................................................ugAaauguaaagaaguaug.......................  1     1  HS5
......................................................................uggaauguaaagaaguaug.......................  2003  0  HS5
......................................................................uggaauguaaagaaguCug.......................  1     1  HS5
......................................................................uggaauguaaagaagAaug.......................  1     1  HS5
......................................................................uggaaCguaaagaaguaug.......................  2     1  HS5
......................................................................Aggaauguaaagaaguaug.......................  1     1  HS5
......................................................................uggaauguaaaAaaguaug.......................  1     1  HS5
......................................................................uggaauguaaagCaguaug.......................  2     1  HS5
......................................................................uggaaugAaaagaaguaugg......................  2     1  HS5
......................................................................uggaauguaUagaaguaugg......................  3     1  HS5
......................................................................uggaauguaaagaagCaugg......................  15    1  HS5
......................................................................uggaauguaaagCaguaugg......................  33    1  HS5
......................................................................uggaaGguaaagaaguaugg......................  3     1  HS5
......................................................................ugCaauguaaagaaguaugg......................  53    1  HS5
......................................................................uggaauguUaagaaguaugg......................  3     1  HS5
......................................................................uggaauguaaagaaCuaugg......................  17    1  HS5
......................................................................uggaauguaaagaaguauCg......................  1     1  HS5
......................................................................uggGauguaaagaaguaugg......................  3     1  HS5
......................................................................uggaauguaGagaaguaugg......................  7     1  HS5
......................................................................uggaauguaaagGaguaugg......................  4     1  HS5
......................................................................Cggaauguaaagaaguaugg......................  7     1  HS5
......................................................................uggaaAguaaagaaguaugg......................  1     1  HS5
......................................................................uggaauguaaagaaguGugg......................  3     1  HS5
......................................................................uggaauguaaagaagGaugg......................  11    1  HS5
......................................................................ugUaauguaaagaaguaugg......................  35    1  HS5
......................................................................uggaauguaaagaaguauAg......................  45    1  HS5
......................................................................uggaauUuaaagaaguaugg......................  14    1  HS5
......................................................................uggaauguaaagaaguaAgg......................  2     1  HS5
......................................................................uggUauguaaagaaguaugg......................  1     1  HS5
......................................................................uggaauguaaagaGguaugg......................  2     1  HS5
......................................................................uUgaauguaaagaaguaugg......................  7     1  HS5
......................................................................uggaaCguaaagaaguaugg......................  14    1  HS5
......................................................................uggaauguaaaCaaguaugg......................  33    1  HS5
......................................................................uggaauguaaaAaaguaugg......................  6     1  HS5
......................................................................uggaauguaaagaCguaugg......................  1     1  HS5
......................................................................uggaauguaaagUaguaugg......................  13    1  HS5
......................................................................uggaauguaaagaagAaugg......................  40    1  HS5
......................................................................uggaauguaaagaaguaugU......................  31    1  HS5
......................................................................uggaauguaaaUaaguaugg......................  16    1  HS5
......................................................................uggaaugGaaagaaguaugg......................  1     1  HS5
......................................................................uggaUuguaaagaaguaugg......................  6     1  HS5
......................................................................uggaauguaaagaaguaugC......................  16    1  HS5
......................................................................uggaauguaaagaaAuaugg......................  2     1  HS5
......................................................................uggaauguaaagaaUuaugg......................  18    1  HS5
......................................................................uCgaauguaaagaaguaugg......................  6     1  HS5
```

```
                              Star                                       Mature
acauuucucaaucggugcccucuaguggaucccguacuucuucacaucuccauauuucauuggauauuuauggaauguaaagaaguauggaguuuacuaauguaaaacgagg
......................................................................uggaauguaaagaaguUugg......................   3       1   HS5
......................................................................uggCauguaaagaaguaugg......................   2       1   HS5
......................................................................uggaauguaaGgaaguaugg......................   1       1   HS5
......................................................................uggaauAuaaagaaguaugg......................   2       1   HS5
......................................................................uggaauguaaagaaguaugg......................   38314   0   HS5
......................................................................uggaauguaaagaaguaugA......................   149     1   HS5
......................................................................Aggaauguaaagaaguaugg......................   49      1   HS5
......................................................................uggaauguaaagaaguaCgg......................   2       1   HS5
......................................................................uggaauguGaagaaguaugg......................   2       1   HS5
......................................................................uAgaauguaaagaaguaugg......................   3       1   HS5
......................................................................uggaauguaCagaaguaugg......................   4       1   HS5
......................................................................uggaauCuaaagaaguaugg......................   5       1   HS5
......................................................................uggaaugCaaagaaguaugg......................   2       1   HS5
......................................................................Gggaauguaaagaaguaugg......................   32      1   HS5
......................................................................uggaauguaaagaaguaGgg......................   25      1   HS5
......................................................................ugAaauguaaagaaguaugg......................   46      1   HS5
......................................................................uggaauguaaagaaguaugga.....................   185235  0   HS5
......................................................................uggaauguaGagaaguaugga.....................   35      1   HS5
......................................................................uggaaugAaaagaaguaugga.....................   17      1   HS5
......................................................................uggCauguaaagaaguaugga.....................   6       1   HS5
......................................................................Cggaauguaaagaaguaugga.....................   36      1   HS5
......................................................................uggGauguaaagaaguaugga.....................   24      1   HS5
......................................................................uggaauguaaaCaaguaugga.....................   186     1   HS5
......................................................................uggaauguaaagaaguauCga.....................   7       1   HS5
......................................................................uggaauguaaGgaaguaugga.....................   4       1   HS5
......................................................................Aggaauguaaagaaguaugga.....................   218     1   HS5
......................................................................uggaauguaaCgaaguaugga.....................   2       1   HS5
......................................................................uggaaugGaaagaaguaugga.....................   15      1   HS5
......................................................................uggaauguaCagaaguaugga.....................   11      1   HS5
......................................................................ugCaauguaaagaaguaugga.....................   277     1   HS5
......................................................................uggaauguaaagaaguaugCa.....................   80      1   HS5
......................................................................uggaauguaaagaaguGugga.....................   9       1   HS5
......................................................................uggaauguaaagaaguUugga.....................   4       1   HS5
......................................................................uUgaauguaaagaaguaugga.....................   31      1   HS5
......................................................................uggaauguaaagaaguaugAa.....................   148     1   HS5
......................................................................uggaaCguaaagaaguaugga.....................   54      1   HS5
......................................................................uggaaAguaaagaaguaugga.....................   18      1   HS5
......................................................................uggaauguaaagaaUuaugga.....................   60      1   HS5
......................................................................uggaaugCaaagaaguaugga.....................   11      1   HS5
......................................................................ugUaauguaaagaaguaugga.....................   188     1   HS5
......................................................................uggaauguaaagaGguaugga.....................   6       1   HS5
......................................................................uAgaauguaaagaaguaugga.....................   18      1   HS5
......................................................................uggaauguaaagaaguaCgga.....................   8       1   HS5
......................................................................uggaauguaaagaaguauggU.....................   167     1   HS5
......................................................................uggaauguaaaAaaguaugga.....................   27      1   HS5
......................................................................uCgaauguaaagaaguaugga.....................   30      1   HS5
......................................................................uggaauguaaagaaguauggC.....................   69      1   HS5
......................................................................uggaauUuaaagaaguaugga.....................   47      1   HS5
......................................................................uggaauguaaagaagGaugga.....................   50      1   HS5
......................................................................Gggaauguaaagaaguaugga.....................   171     1   HS5
......................................................................uggaauguaaagaUguaugga.....................   2       1   HS5
......................................................................uggaauguaaagaaguaugUa.....................   155     1   HS5
......................................................................uggaauguaaagaaAuaugga.....................   20      1   HS5
......................................................................uggaauguaaaGgaguaugga.....................   22      1   HS5
......................................................................uggaauguUaagaaguaugga.....................   6       1   HS5
......................................................................uggaCuguaaagaaguaugga.....................   1       1   HS5
......................................................................uggaauguaaagaagAaugga.....................   219     1   HS5
......................................................................uggaauguGaagaaguaugga.....................   5       1   HS5
......................................................................uggaaGguaaagaaguaugga.....................   13      1   HS5
......................................................................uggaauAuaaagaaguaugga.....................   11      1   HS5
......................................................................uggaauguaaagaCguaugga.....................   3       1   HS5
......................................................................ugAaauguaaagaaguaugga.....................   222     1   HS5
......................................................................uggaauCuaaagaaguaugga.....................   16      1   HS5
......................................................................uggaauguaaagaaguauAga.....................   11      1   HS5
......................................................................uggaauguaaUgaaguaugga.....................   2       1   HS5
......................................................................uggaauguaaagaaguauUga.....................   11      1   HS5
......................................................................uggaauguaaagUaguaugga.....................   61      1   HS5
......................................................................uggaauguaaagaaCuaugga.....................   27      1   HS5
......................................................................uggaGuguaaagaaguaugga.....................   9       1   HS5
......................................................................uggaauguaaagaagCaugga.....................   94      1   HS5
```

Star **Mature**

acauuucucaaucggugcccucuaguggaucccguacuucuucacaucuccauauuucauuggauauuuauggaauguaaagaaguauggaguuuacuaauguaaaacgagg			
....uggUauguaaagaaguaugga.....................	10	1	HS5
....uggaauguaaagaaguauggG.....................	294	1	HS5
....uggaauguaaagCaguaugga.....................	211	1	HS5
....uggaauguaaaUaaguaugga.....................	66	1	HS5
....uggaauguaUagaaguaugga.....................	11	1	HS5
....uggaauguaaagaaguaAgga.....................	3	1	HS5
....uggaUuguaaagaaguaugga.....................	15	1	HS5
....uggaauguaaagaaguCuggag....................	14	1	HS5
....Cggaauguaaagaaguauggag....................	511	1	HS5
....uggaauUuaaagaaguauggag....................	381	1	HS5
....uggaauguaaagaagAauggag....................	2392	1	HS5
....uggaaugGaaagaaguauggag....................	109	1	HS5
....ugUaauguaaagaaguauggag....................	1709	1	HS5
....uggaauguaaagaaguaugUag....................	1349	1	HS5
....Gggaauguaaagaaguauggag....................	1374	1	HS5
....uAgaauguaaagaaguauggag....................	119	1	HS5
....uggaauguaaagaaguauCgag....................	242	1	HS5
....uggaauguaaCgaaguauggag....................	7	1	HS5
....uggaauguaCagaaguauggag....................	146	1	HS5
....uggaauguaUagaaguauggag....................	66	1	HS5
....uggaauguaaagaagGauggag....................	517	1	HS5
....uggaauguaaagaCguauggag....................	10	1	HS5
....uggaauguaaagaGguauggag....................	65	1	HS5
....uggaauCuaaagaaguauggag....................	276	1	HS5
....uggaauguaGagaaguauggag....................	343	1	HS5
....uggaaGguaaagaaguauggag....................	155	1	HS5
....uggaaCguaaagaaguauggag....................	583	1	HS5
....uggaauguaaagaaguauggUg....................	706	1	HS5
....uggaaAguaaagaaguauggag....................	110	1	HS5
....uggCauguaaagaaguauggag....................	56	1	HS5
....uggaauguaaagGaguauggag....................	172	1	HS5
....uggaauguaaagaaguauAgag....................	120	1	HS5
....uggaauguaaagaaguaCggag....................	40	1	HS5
....uggaauguaaagaaguauUgag....................	349	1	HS5
....uggaauguCaagaaguauggag....................	2	1	HS5
....uggaGuguaaagaaguauggag....................	32	1	HS5
....uggaauguaaagaUguauggag....................	45	1	HS5
....uggaauguaaaCaaguauggag....................	2251	1	HS5
....uggaUuguaaagaaguauggag....................	163	1	HS5
....uggaauguaaaUaaguauggag....................	685	1	HS5
....uggaauguaaUgaaguauggag....................	40	1	HS5
....uUgaauguaaagaaguauggag....................	234	1	HS5
....uggaauguaaagaaguauggaA....................	11447	1	HS5
....uggaauguaaGgaaguauggag....................	46	1	HS5
....uggaauguaaagaaUuauggag....................	712	1	HS5
....uCgaauguaaagaaguauggag....................	400	1	HS5
....uggaauguaaagUaguauggag....................	612	1	HS5
....uggaauguaaagaaguaGggag....................	16	1	HS5
....uggGauguaaagaaguauggag....................	311	1	HS5
....ugAaauguaaagaaguauggag....................	1972	1	HS5
....uggaauguaaagaagCauggag....................	1048	1	HS5
....uggaauguaaagaaguGuggag....................	100	1	HS5
....uggaauguaaagaaguaugCag....................	951	1	HS5
....uggaauguaaagaaguauggCg....................	233	1	HS5
....uggaauguGaagaaguauggag....................	66	1	HS5
....uggaauguaaagaaguaugAag....................	1206	1	HS5
....uggaauguaaagaaAuauggag....................	142	1	HS5
....uggaauguaaagaaguauggGg....................	930	1	HS5
....uggaauguaaagaaguauggaU....................	5709	1	HS5
....uggaaugAaaagaaguauggag....................	223	1	HS5
....uggaaugCaaagaaguauggag....................	124	1	HS5
....uggaauguaaagaaCuauggag....................	565	1	HS5
....uggaauguaaagaaguaAggag....................	25	1	HS5
....uggaauAuaaagaaguauggag....................	85	1	HS5
....uggaauguaaagaaguauggag....................	2070452	0	HS5
....uggaCuguaaagaaguauggag....................	14	1	HS5
....uggaauguaaaAaaguauggag....................	310	1	HS5
....uggaauguUaagaaguauggag....................	41	1	HS5
....uggaauguaaagaaguauggaC....................	9651	1	HS5
....uggaauguaaagaaguUuggag....................	45	1	HS5

Star Mature

acauuucucaaucggugcccucuaguggauccoguacuucuucacaucuccauauuucauuggauauuuauggaauguaaagaaguauggaguuuacuaauguaaaacgagg			
....Aggaauguaaagaaguauggag....................	2452	1	HS5
....uggaauguaaagCaguauggag....................	2112	1	HS5
....ugCaauguaaagaaguauggag....................	2543	1	HS5
....uggUauguaaagaaguauggag....................	87	1	HS5
....uggaauguaaagaagGauggagu...................	32	1	HS5
....Gggaauguaaagaaguauggagu...................	86	1	HS5
....uggaaAguaaagaaguauggagu...................	6	1	HS5
....uggaauguaaaUaaguauggagu...................	23	1	HS5
....uggUauguaaagaaguauggagu...................	4	1	HS5
....uggaauguaaagaaguauggCgu...................	10	1	HS5
....uggaauguaaagaaguauAgagu...................	4	1	HS5
....uggaauguaaagaaguaugAagu...................	50	1	HS5
....uggaauCuaaagaaguauggagu...................	20	1	HS5
....uggaauguaaagaaguauCgagu...................	23	1	HS5
....uggaauguaaagaaguUuggagu...................	4	1	HS5
....uggGauguaaagaaguauggagu...................	20	1	HS5
....uggaauguaaagaaCuauggagu...................	41	1	HS5
....uggaUuguaaagaaguauggagu...................	5	1	HS5
....uggaauguaaagaaguauggaCu...................	324	1	HS5
....uggaauguaaagaaguauggagG...................	836	1	HS5
....uggaauguaGagaaguauggagu...................	15	1	HS5
....uggaauguaaagaaUuauggagu...................	33	1	HS5
....uggaauguaaaCaaguauggagu...................	95	1	HS5
....uggaaugCaaagaaguauggagu...................	4	1	HS5
....uUgaauguaaagaaguauggagu...................	13	1	HS5
....uggaauguaUagaaguauggagu...................	1	1	HS5
....uggaauguUaagaaguauggagu...................	1	1	HS5
....uggaauguaaagaaguauggaUu...................	280	1	HS5
....uggaauguaaagaaguauggagu...................	99405	0	HS5
....uggaauguaaagaGguauggagu...................	4	1	HS5
....Cggaauguaaagaaguauggagu...................	37	1	HS5
....uCgaauguaaagaaguauggagu...................	17	1	HS5
....ugAaauguaaagaaguauggagu...................	103	1	HS5
....uggaauguaaaAaaguauggagu...................	20	1	HS5
....uggaauguaaagaagCauggagu...................	42	1	HS5
....uggaauguaaagaaguauggagA...................	135869	1	HS5
....uggaauguaaagaaguauUgagu...................	21	1	HS5
....Aggaauguaaagaaguauggagu...................	142	1	HS5
....uggaaugGaaagaaguauggagu...................	6	1	HS5
....uggaauguaaagaaguauggaAu...................	94	1	HS5
....uggaauguaaagaaAuauggagu...................	6	1	HS5
....uggaauguaaagaaguaGggagu...................	1	1	HS5
....uggaauguaaagUaguauggagu...................	18	1	HS5
....uggCauguaaagaaguauggagu...................	6	1	HS5
....uggaauguaaagaaguaugUagu...................	93	1	HS5
....uggaauguaaagaaguaCggagu...................	1	1	HS5
....uggaauUuaaagaaguauggagu...................	18	1	HS5
....ugCaauguaaagaaguauggagu...................	139	1	HS5
....uggaauguaaagaaguauggUgu...................	51	1	HS5
....uggaauguaaGgaaguauggagu...................	6	1	HS5
....uggaauguaaagaagAauggagu...................	114	1	HS5
....uAgaauguaaagaaguauggagu...................	5	1	HS5
....uggaauguaaagaaguauggGgu...................	41	1	HS5
....uggaauAuaaagaaguauggagu...................	12	1	HS5
....uggaauguaCagaaguauggagu...................	6	1	HS5
....uggaauguaaUgaaguauggagu...................	1	1	HS5
....uggaauguaaagGaguauggagu...................	8	1	HS5
....uggaaCguaaagaaguauggagu...................	26	1	HS5
....uggaauguaaagaaguaugCagu...................	56	1	HS5
....uggaaugAaaagaaguauggagu...................	16	1	HS5
....uggaaGguaaagaaguauggagu...................	13	1	HS5
....uggaauguaaagaaguGuggagu...................	3	1	HS5
....uggaauguaaagCaguauggagu...................	96	1	HS5
....uggaGuguaaagaaguauggagu...................	5	1	HS5
....uggaauguaaagaaguauggagC...................	756	1	HS5
....ugUaauguaaagaaguauggagu...................	91	1	HS5
....uggaauguGaagaaguauggagu...................	3	1	HS5
....uggaauguaaagaaguauggagAu..................	327	1	HS5
....ugAaauguaaagaaguauggaguu..................	2	1	HS5
....uggaauguaaagaaguauggaAuu..................	2	1	HS5

```
                              Star                                      Mature
acauuucucaaucggugcccucuaguggaucccguacuucuucacaucuccauauuucauuggauauuuauggaauguaaagaaguauggaguuuacuaauguaaaacgagg
......................................................................uggaauguaaagaaguauggaCuu..................    1      1     HS5
......................................................................Cggaauguaaagaaguauggaguu..................    2      1     HS5
......................................................................uggaauguaaagaaguauggaguA..................    203    1     HS5
......................................................................Gggaauguaaagaaguauggaguu..................    1      1     HS5
......................................................................uggaauguaaagaaguauggaguC..................    2      1     HS5
......................................................................uggaauguaaagaaguauggagGu..................    8      1     HS5
......................................................................uggaauguaaagaagCauggaguu..................    2      1     HS5
......................................................................uggaaugAaaagaaguauggaguu..................    2      1     HS5
......................................................................uggaauguaaagaaguaugCaguu..................    2      1     HS5
......................................................................uggaauguaaagaaguauggGguu..................    1      1     HS5
......................................................................uggaauguaaagaaguaugAaguu..................    1      1     HS5
......................................................................uggaauguaaagaaguauggaguG..................    2      1     HS5
......................................................................uggaauguaaagaaguauggaguu..................    970    0     HS5
......................................................................uggaauguaaagaaguGuggaguu..................    1      1     HS5
......................................................................uggaauguaaagaaguauggaUuu..................    5      1     HS5
......................................................................uggaauguaaaCaaguauggaguu..................    1      1     HS5
......................................................................uggaauguaaagaaUuauggaguu..................    1      1     HS5
......................................................................uggaauguaaagaaguauggagCu..................    2      1     HS5
......................................................................uggaauUuaaagaaguauggaguu..................    1      1     HS5
......................................................................uggaauguaaaUaaguauggaguu..................    1      1     HS5
......................................................................uggaauguaaagaaguauggaguuu.................    47     0     HS5
......................................................................uggaauguaaagCaguauggaguuu.................    1      1     HS5
......................................................................uggaauguaaagaaguauggaguAu.................    12     1     HS5
......................................................................uggaauguaaagaaguauggaguGu.................    1      1     HS5
......................................................................uggaauguaaagaaguauggaguCu.................    1      1     HS5
......................................................................ugAaauguaaagaaguauggaguuu.................    1      1     HS5
......................................................................uggaauguaaagaaguauggaguuG.................    1      1     HS5
......................................................................uggaauguaaagaaguauggaguuC.................    2      1     HS5
......................................................................uggaauguaaagaaguauggaUuuu.................    1      1     HS5
......................................................................uggaauguaaagaaguauggagGuu.................    3      1     HS5
......................................................................uggaauguaaagaaguauggagAuu.................    20     1     HS5
......................................................................uggaauguaaagaaguauggaguuA.................    8      1     HS5
......................................................................uggaauguaaagaaguauggaguuuU................    4      1     HS5
......................................................................uggaauguaaagaaguauggaguuua................    1      0     HS5
......................................................................uggaauguaaagaaguauggaguuuCc...............    1      1     HS5
.......................................................................ggaauguaaagaaguaug.......................    5      0     HS5
.......................................................................Ugaauguaaagaaguaug.......................    1      1     HS5
.......................................................................ggaauguaaagaaguaugg......................    75     0     HS5
.......................................................................ggaauguaaagaaguaugA......................    1      1     HS5
.......................................................................ggaauguaaaAaaguaugg......................    1      1     HS5
.......................................................................ggaGuguaaagaaguaugg......................    1      1     HS5
.......................................................................Cgaauguaaagaaguaugg......................    2      1     HS5
.......................................................................gAaauguaaagaaguaugg......................    1      1     HS5
.......................................................................Ugaauguaaagaaguaugg......................    5      1     HS5
.......................................................................ggaauguaaagaaguauggG.....................    2      1     HS5
.......................................................................ggaauguaaagUaguaugga.....................    1      1     HS5
.......................................................................ggaauCuaaagaaguaugga.....................    2      1     HS5
.......................................................................Ugaauguaaagaaguaugga.....................    37     1     HS5
.......................................................................gUaauguaaagaaguaugga.....................    1      1     HS5
.......................................................................ggaauguaaagaaguaugga.....................    268    0     HS5
.......................................................................Cgaauguaaagaaguaugga.....................    2      1     HS5
.......................................................................ggaauguaaagaaguaugUa.....................    1      1     HS5
.......................................................................gCaauguaaagaaguaugga.....................    1      1     HS5
.......................................................................Agaauguaaagaaguaugga.....................    1      1     HS5
.......................................................................ggaauguaaagaCguauggag....................    5      1     HS5
.......................................................................Agaauguaaagaaguauggag....................    3      1     HS5
.......................................................................ggaauguaaagaaguauggUg....................    1      1     HS5
.......................................................................ggaauguaaagaaguauggaU....................    9      1     HS5
.......................................................................ggaGuguaaagaaguauggag....................    1      1     HS5
.......................................................................Cgaauguaaagaaguauggag....................    82     1     HS5
.......................................................................gCaauguaaagaaguauggag....................    10     1     HS5
.......................................................................ggaauguaaagaaguaugUag....................    3      1     HS5
.......................................................................ggaauguaaaCaaguauggag....................    2      1     HS5
.......................................................................ggaauUuaaagaaguauggag....................    1      1     HS5
.......................................................................ggaauguaUagaaguauggag....................    1      1     HS5
.......................................................................ggaauguaaagaaguaugAag....................    2      1     HS5
.......................................................................ggaauguaaagCaguauggag....................    5      1     HS5
.......................................................................ggaauguaaagaaguCuggag....................    1      1     HS5
.......................................................................ggaauguaaagaaguaugCag....................    1      1     HS5
.......................................................................ggaauguaaagGaguauggag....................    1      1     HS5
```

```
                              Star                                    Mature
acauuucucaaucggugcccucuaguggaucccguacuucuucacaucuccauauuucauuggauauuuauggaauguaaagaaguauggaguuuacuaauguaaaacgagg
.......................................................................ggaauguaaagaaguUuggag....................  2     1  HS5
.......................................................................ggaauguaaagaaguauggaA....................  13    1  HS5
.......................................................................gUaauguaaagaaguauggag....................  5     1  HS5
.......................................................................ggaauguaaagaaguauggaC....................  17    1  HS5
.......................................................................ggaauguaaagaaguauggGg....................  4     1  HS5
.......................................................................ggaauguaaagaUguauggag....................  1     1  HS5
.......................................................................ggaauguaaagaaguauggag....................  3462  0  HS5
.......................................................................ggaauguaaaUaaguauggag....................  1     1  HS5
.......................................................................ggGauguaaagaaguauggag....................  1     1  HS5
.......................................................................ggaauguaaagaaguauCgag....................  2     1  HS5
.......................................................................ggaauguaaagUaguauggag....................  1     1  HS5
.......................................................................ggaaAguaaagaaguauggag....................  1     1  HS5
.......................................................................ggaauAuaaagaaguauggag....................  2     1  HS5
.......................................................................ggaauguaaagaagCauggag....................  1     1  HS5
.......................................................................ggaauguaaGgaaguauggag....................  1     1  HS5
.......................................................................ggaauguaaaAaaguauggag....................  15    1  HS5
.......................................................................Ugaauguaaagaaguauggag....................  436   1  HS5
.......................................................................ggaauCuaaagaaguauggag....................  1     1  HS5
.......................................................................gAaauguaaagaaguauggag....................  12    1  HS5
.......................................................................gCaauguaaagaaguauggagu...................  1     1  HS5
.......................................................................ggaauguaaagaaguauggaAu...................  1     1  HS5
.......................................................................gUaauguaaagaaguauggagu...................  2     1  HS5
.......................................................................ggaauguaaagaaguauggagu...................  255   0  HS5
.......................................................................ggaauguaaagaaguauggagA...................  266   1  HS5
.......................................................................ggaauguaaaAaaguauggagu...................  1     1  HS5
.......................................................................gAaauguaaagaaguauggagu...................  1     1  HS5
.......................................................................ggaauguaaagaaguauCgagu...................  1     1  HS5
.......................................................................ggaauguaaagaaguauggagG...................  3     1  HS5
.......................................................................ggaauguaaagCaguauggagu...................  1     1  HS5
.......................................................................ggaauguaaagaaguauggagC...................  11    1  HS5
.......................................................................ggaaAguaaagaaguauggagu...................  1     1  HS5
.......................................................................ggaauguaaagaUguauggagu...................  1     1  HS5
.......................................................................Cgaauguaaagaaguauggagu...................  4     1  HS5
.......................................................................Ugaauguaaagaaguauggagu...................  30    1  HS5
.......................................................................ggaauguaaagaaguauggaguu..................  5     0  HS5
.......................................................................ggaauguaaagaaguauggagCu..................  1     1  HS5
.......................................................................ggaauguaaagaaguauggaguA..................  2     1  HS5
.......................................................................ggaauguaaagaaguauggagAu..................  1     1  HS5
.......................................................................ggaauguaaagaaguauggaguuuU................  1     1  HS5
........................................................................gaauguaaagaaguaugA......................  1     1  HS5
........................................................................Aaauguaaagaaguaugg......................  2     1  HS5
........................................................................gaauguaaagaaguaugU......................  1     1  HS5
........................................................................Uaauguaaagaaguaugg......................  3     1  HS5
........................................................................gaauguaaagaaguaugg......................  16    0  HS5
........................................................................Aaauguaaagaaguaugga.....................  6     1  HS5
........................................................................gaauguaaagaaguaugga.....................  75    0  HS5
........................................................................Uaauguaaagaaguaugga.....................  4     1  HS5
........................................................................gaauguaaaAaaguauggag....................  1     1  HS5
........................................................................gGauguaaagaaguauggag....................  1     1  HS5
........................................................................gaauguaaagaaguaCggag....................  1     1  HS5
........................................................................gaauguaaagaaguaugUag....................  3     1  HS5
........................................................................gaauguaaagaaguauggaC....................  10    1  HS5
........................................................................gaauguaaagaaguaAggag....................  1     1  HS5
........................................................................gaaugGaaagaaguauggag....................  1     1  HS5
........................................................................Caauguaaagaaguauggag....................  13    1  HS5
........................................................................Uaauguaaagaaguauggag....................  42    1  HS5
........................................................................gaauguaaagaaguaugCag....................  3     1  HS5
........................................................................gaauguaaagaagAauggag....................  1     1  HS5
........................................................................Aaauguaaagaaguauggag....................  50    1  HS5
........................................................................gaauguaaagaaguauggaU....................  5     1  HS5
........................................................................gaauguaaagaaguauggag....................  1200  0  HS5
........................................................................gaauguaaagaaguauggaA....................  6     1  HS5
........................................................................gaauguaaagaaCuauggag....................  3     1  HS5
........................................................................gaauguaaagaagAauggagu...................  1     1  HS5
........................................................................gaauguaaagaaguauggagC...................  15    1  HS5
........................................................................Aaauguaaagaaguauggagu...................  4     1  HS5
........................................................................gaauguaaagaaCuauggagu...................  2     1  HS5
........................................................................gaauguaaagaaguaugAagu...................  1     1  HS5
........................................................................gaauguaaagaaguauggaAu...................  1     1  HS5
........................................................................Uaauguaaagaaguauggagu...................  3     1  HS5
```

```
                              Star                                 Mature
acauuucucaaucggugcccucuaguggaucccguacuucuucacaucuccauauuucauuggauauuuauggaauguaaagaaguauggaguuuacuaauguaaaacgagg
........................................................................gaauguaaagaaguauggaUu...................   6     1   HS5
........................................................................gaauguaaagaaguauggagu...................   740   0   HS5
........................................................................gaauguaaaUaaguauggagu...................   1     1   HS5
........................................................................gaauguaaagaCguauggagu...................   1     1   HS5
........................................................................gaauguaaagaaguauggUgu...................   1     1   HS5
........................................................................gaauguaaagaaguauggaCu...................   2     1   HS5
........................................................................gaauguaaagaaUuauggagu...................   2     1   HS5
........................................................................gaauguaaagaaguauggagA...................   462   1   HS5
........................................................................gUauguaaagaaguauggagu...................   1     1   HS5
........................................................................gaauguaaagaaCuauggaguu..................   2     1   HS5
........................................................................gaauguaaagaaguaugUaguu..................   1     1   HS5
........................................................................gaauUuaaagaaguauggaguu..................   1     1   HS5
........................................................................gaauguaaagaaguauggGguu..................   1     1   HS5
........................................................................gaauguaaagaaguauggagCu..................   2     1   HS5
........................................................................gaauguaaagaaguaugAaguu..................   1     1   HS5
........................................................................gUauguaaagaaguauggaguu..................   1     1   HS5
........................................................................gaauguaaagaaguauggaguu..................   378   0   HS5
........................................................................gaauguaaagaaguauggaguA..................   274   1   HS5
........................................................................gaauguaaagaaguauggagAu..................   31    1   HS5
........................................................................gaauguaaagaaguauggaguG..................   4     1   HS5
........................................................................gaaugAaaagaaguauggaguu..................   1     1   HS5
........................................................................gaauguaaagaaUuauggaguu..................   1     1   HS5
........................................................................gaauguaaagaaguauggaguC..................   6     1   HS5
........................................................................gaauguaaagaaguauggagAuu.................   1     1   HS5
........................................................................gaauguaaagaaguauggaguuA.................   10    1   HS5
........................................................................gaauguaaagaaguauggaguuu.................   9     0   HS5
........................................................................gaauguaaagaaguauggaguAu.................   1     1   HS5
........................................................................gaauguaaagaaguauggaguuua................   1     0   HS5
.........................................................................aauguaaagaaguaugga.....................   22    0   HS5
.........................................................................Uauguaaagaaguauggag....................   1     1   HS5
.........................................................................aauguaaagaaguaugCag....................   1     1   HS5
.........................................................................aauUuaaagaaguauggag....................   1     1   HS5
.........................................................................aauguaaagaaUuauggag....................   1     1   HS5
.........................................................................aauguaaagUaguauggag....................   1     1   HS5
.........................................................................aauguaaagGaguauggag....................   1     1   HS5
.........................................................................aauguaaagaagCauggag....................   1     1   HS5
.........................................................................aauguaaagCaguauggag....................   1     1   HS5
.........................................................................aauguaaagaaguauggag....................   245   0   HS5
.........................................................................aauguaaagaaguauggaU....................   1     1   HS5
.........................................................................aauguaaagaaguauggaA....................   6     1   HS5
.........................................................................aauguaaagaaguauggagA...................   24    1   HS5
.........................................................................aauguUaagaaguauggagu...................   1     1   HS5
.........................................................................aauguaaagaaguauggagu...................   25    0   HS5
.........................................................................aauguaaagaaguauggaguu..................   1     0   HS5
..........................................................................auguaaagaaguauggaU....................   2     1   HS5
..........................................................................auguaaagaagCauggag....................   1     1   HS5
..........................................................................Uuguaaagaaguauggag....................   1     1   HS5
..........................................................................auguaaagaaguauggaC....................   2     1   HS5
..........................................................................auguaaagaaguauggag....................   218   0   HS5
..........................................................................auguaaagaaguauggagu...................   22    0   HS5
..........................................................................auguaaagaaguauggagA...................   25    1   HS5
..........................................................................auguaaagaaUuauggagu...................   1     1   HS5
..........................................................................auguaaagaaguauggaguu..................   2     0   HS5
...........................................................................uguaaagaaguauggagu...................   16    0   HS5
...........................................................................Aguaaagaaguauggagu...................   1     1   HS5
...........................................................................uguaaagaaguauggagA...................   18    1   HS5
...........................................................................uguaaagaaguauggaguu..................   1     0   HS5
............................................................................guaaagaaguauggaguA..................   1     1   HS5
............................................................................guaaagaaguauggagAu..................   1     1   HS5

..........aucggugcccucuaguggauc.................................................................................   2     0   HS7
............................aucccguacuucuucacaucu...............................................................   2     0   HS7
..............................cccguacuucuucacaucucca............................................................   24    0   HS7
..............................cccguacuucuucacaucuccaC...........................................................   2     1   HS7
..............................cccguacuucuucacaucuccau...........................................................   16    0   HS7
..............................cccguacuucuucacaucuccauU..........................................................   4     1   HS7
..............................cccguacuucuucacaucuccaua..........................................................   20    0   HS7
...............................ccguacuucuucacaucu...............................................................   2     0   HS7
...............................ccguacuucuucacaucuc..............................................................   1     0   HS7
...............................ccguacuucuucacaucuA..............................................................   1     1   HS7
```

```
                              Star                                    Mature
acauuucucaaucggugcccucuagguggaucccguacuucuucacaucuccauauuucauuggauauuuauggaauguaaagaaguauggaguuuacuaauguaaaacgagg
................................ccguacuucuucacaucuU..............................................................   1      1   HS7
................................ccguacuucuucacaucucc.............................................................   7      0   HS7
................................ccguacuucuucacauUucca............................................................   1      1   HS7
................................ccguacuucuucacaucucca............................................................   180    0   HS7
................................ccguacuucuucacaucuUcau...........................................................   1      1   HS7
................................ccguacuucuucacaucuccaA...........................................................   1      1   HS7
................................ccguacuucuucacaucuccau...........................................................   92     0   HS7
................................ccguaUuucuucacaucuccaua..........................................................   1      1   HS7
................................ccguGcuucuucacaucuccaua..........................................................   1      1   HS7
................................ccguacuucuucacaucuccaua..........................................................   1217   0   HS7
................................Ucguacuucuucacaucuccaua..........................................................   1      1   HS7
................................ccguacuucuuUacaucuccaua..........................................................   1      1   HS7
................................ccguacuucuucacaucCccaua..........................................................   1      1   HS7
................................ccguacuucuucacaucuUcaua..........................................................   1      1   HS7
................................ccguacuucuucacauUuccaua..........................................................   1      1   HS7
................................ccguacuucuucacaucuccaAa..........................................................   2      1   HS7
................................ccguacuucuucacaucucUaua..........................................................   1      1   HS7
................................ccguacuucuucacaucuccauU..........................................................   72     1   HS7
................................ccguacuucuucacaucuccauC..........................................................   1      1   HS7
................................ccguacuucuucacaucuccGua..........................................................   1      1   HS7
................................ccguacuucuucacaucuccauUu.........................................................   4      1   HS7
................................ccguacuucuucacaucuccauau.........................................................   1      0   HS7
................................ccguacuucuucacaucuccauaA.........................................................   8      1   HS7
................................ccguacuucuucacaucuccauaAu........................................................   1      1   HS7
................................ccguacuucuucacaucuccauauu........................................................   1      0   HS7
.................................cguacuucuucacaucuccaua..........................................................   5      0   HS7
..................................guacuucuucacaucucca............................................................   1      0   HS7
....................................acuucuucacaucuccaua..........................................................   1      0   HS7
.....................................cuucuucacaucuccaua..........................................................   1      0   HS7
.............................................................uggauauuuauggaauguaa................................   1      0   HS7
..................................................................auuuauggaauguaaagaagu..........................   1      0   HS7
...................................................................uuuUuggaauguaaagaaguauggag....................   1      1   HS7
...................................................................Cuuauggaauguaaagaaguauggag....................   1      1   HS7
...................................................................uCuauggaauguaaagaaguauggag....................   1      1   HS7
....................................................................uuauggaauguaaagaagua.........................   3      0   HS7
....................................................................uuauggaauguaaagaaguaug.......................   8      0   HS7
....................................................................uuauggaauguaaagaaguauU.......................   2      1   HS7
....................................................................uuauggaauguaaagaaguaugg......................   1      0   HS7
....................................................................uuauggaauguaaagaaguaugga.....................   1      0   HS7
....................................................................uCauggaauguaaagaaguauggag....................   1      1   HS7
....................................................................uuUuggaauguaaagaaguauggag....................   19     1   HS7
....................................................................uuUuggaauguaaagaaguauggagu...................   2      1   HS7
.....................................................................uauggaauguaaagaaguaug.......................   4      0   HS7
.....................................................................uUuggaauguaaagaaguaug.......................   1      1   HS7
.....................................................................uUuggaauguaaagaaguaugg......................   1      1   HS7
.....................................................................uauggaauguaaagaaguaugg......................   3      0   HS7
.....................................................................uUuggaauguaaagaaguaugga.....................   6      1   HS7
.....................................................................uCuggaauguaaagaaguaugga.....................   1      1   HS7
.....................................................................Cauggaauguaaagaaguauggag....................   2      1   HS7
.....................................................................uCuggaauguaaagaaguauggag....................   2      1   HS7
.....................................................................uauggaauguaaagaaguauggag....................   5      0   HS7
.....................................................................Aauggaauguaaagaaguauggag....................   3      1   HS7
.....................................................................Gauggaauguaaagaaguauggag....................   3      1   HS7
.....................................................................uUuggaauguaaagaaguauggag....................   208    1   HS7
.....................................................................uUuggaauguaaagaaguauggagu...................   8      1   HS7
.....................................................................uCuggaauguaaagaaguauggagu...................   1      1   HS7
.....................................................................Aauggaauguaaagaaguauggagu...................   1      1   HS7
......................................................................Uuggaauguaaagaaguaug.......................   2      1   HS7
......................................................................Cuggaauguaaagaaguaugg......................   1      1   HS7
......................................................................auggaauguaaagaaguaugg......................   3      0   HS7
......................................................................Guggaauguaaagaaguaugg......................   1      1   HS7
......................................................................Uuggaauguaaagaaguaugg......................   13     1   HS7
......................................................................Guggaauguaaagaaguaugga.....................   2      1   HS7
......................................................................auggaauguaaagaaguauggC.....................   3      1   HS7
......................................................................Uuggaauguaaagaaguaugga.....................   39     1   HS7
......................................................................auggaauguaaagaaguauCga.....................   1      1   HS7
......................................................................Cuggaauguaaagaaguaugga.....................   1      1   HS7
......................................................................auggaauguaaagaaguaugga.....................   12     0   HS7
......................................................................Uuggaauguaaagaaguauggag....................   706    1   HS7
......................................................................Cuggaauguaaagaaguauggag....................   27     1   HS7
```

Star **Mature**

```
acauuucucaaucggugcccucuaguggaucccguacuucuucacaucuccauauuucauuggauauuuauggaauguaaagaaguauggaguuuacuaauguaaaacgagg
.....................................................................auggaauguaaagaaguauggag....................  29     0  HS7
.....................................................................Guggaauguaaagaaguauggag....................  11     1  HS7
.....................................................................Uuggaauguaaagaaguauggagu...................  22     1  HS7
.....................................................................auggaauguaaagaaguauggagu...................  8      0  HS7
.....................................................................Cuggaauguaaagaaguauggagu...................  2      1  HS7
.....................................................................auggaauguaaagaaguauggagA...................  2      1  HS7
......................................................................Gggaauguaaagaaguau........................  1      1  HS7
......................................................................uggaauguaaagaaguaG........................  5      1  HS7
......................................................................uggaauguaaagaaguau........................  244    0  HS7
......................................................................uggaauguaaagaaguaA........................  2      1  HS7
......................................................................ugAaauguaaagaaguau........................  1      1  HS7
......................................................................uggaauguaaagaaguaGg.......................  2      1  HS7
......................................................................ugUaauguaaagaaguaug.......................  2      1  HS7
......................................................................Aggaauguaaagaaguaug.......................  1      1  HS7
......................................................................uggaauguaaagaaguauA.......................  3      1  HS7
......................................................................uggaauguaaagaaguaug.......................  1479   0  HS7
......................................................................uggaauguaaagaaUuaug.......................  2      1  HS7
......................................................................uggaauguaaaCaaguaugg......................  4      1  HS7
......................................................................uggaauguaaagaaUuaugg......................  3      1  HS7
......................................................................uggaauguaaagaaguauUg......................  2      1  HS7
......................................................................ugCaauguaaagaaguaugg......................  16     1  HS7
......................................................................uggaauguaGagaaguaugg......................  3      1  HS7
......................................................................uggaauguaaaUaaguaugg......................  8      1  HS7
......................................................................uggaGuguaaagaaguaugg......................  12     1  HS7
......................................................................ugAaauguaaagaaguaugg......................  13     1  HS7
......................................................................uggCauguaaagaaguaugg......................  7      1  HS7
......................................................................uggaauguaaagaaguaCgg......................  2      1  HS7
......................................................................uggaauguaaagaaguauAg......................  36     1  HS7
......................................................................Gggaauguaaagaaguaugg......................  2      1  HS7
......................................................................uggaauguaUagaaguaugg......................  1      1  HS7
......................................................................uggaauAuaaagaaguaugg......................  2      1  HS7
......................................................................uggaauguaaagaagCaugg......................  3      1  HS7
......................................................................Aggaauguaaagaaguaugg......................  11     1  HS7
......................................................................uggaauguaaagaaguaugA......................  86     1  HS7
......................................................................uggaauUuaaagaaguaugg......................  7      1  HS7
......................................................................uggaauguaaagGaguaugg......................  3      1  HS7
......................................................................uggGauguaaagaaguaugg......................  4      1  HS7
......................................................................uggaauguaaagaaguaugg......................  25676  0  HS7
......................................................................uggaaCguaaagaaguaugg......................  1      1  HS7
......................................................................uggaauguaaagaaguauCg......................  5      1  HS7
......................................................................uggaauCuaaagaaguaugg......................  7      1  HS7
......................................................................uggaUuguaaagaaguaugg......................  3      1  HS7
......................................................................uggaauguUaagaaguaugg......................  3      1  HS7
......................................................................uCgaauguaaagaaguaugg......................  3      1  HS7
......................................................................uggaauguaaaAaaguaugg......................  2      1  HS7
......................................................................uggaauguaaagaaguaugC......................  5      1  HS7
......................................................................uggaauguaaGgaaguaugg......................  3      1  HS7
......................................................................uUgaauguaaagaaguaugg......................  3      1  HS7
......................................................................uggaauguaaCgaaguaugg......................  4      1  HS7
......................................................................uggaauguaaagUaguaugg......................  1      1  HS7
......................................................................uggaaugCaaagaaguaugg......................  5      1  HS7
......................................................................uggaauguaaagaaguaugU......................  7      1  HS7
......................................................................uggaauguGaagaaguaugg......................  2      1  HS7
......................................................................uggaauguaaagaaguaAgg......................  153    1  HS7
......................................................................ugUaauguaaagaaguaugg......................  9      1  HS7
......................................................................uggaauguaaagaaguUugg......................  1      1  HS7
......................................................................uggaauguaaagaaguaGgg......................  5      1  HS7
......................................................................uggaauguaaagaaCuaugg......................  6      1  HS7
......................................................................Cggaauguaaagaaguaugg......................  2      1  HS7
......................................................................ugCaauguaaagaaguaugga.....................  48     1  HS7
......................................................................uggaGuguaaagaaguaugga.....................  27     1  HS7
......................................................................uggaauguaaagaaguauggC.....................  22     1  HS7
......................................................................uggaauguaaagaagCaugga.....................  7      1  HS7
......................................................................uggaauguaaagaaguauCga.....................  27     1  HS7
......................................................................uggaaugGaaagaaguaugga.....................  6      1  HS7
......................................................................uggaauguaaagGaguaugga.....................  8      1  HS7
......................................................................uggaauguaUagaaguaugga.....................  3      1  HS7
......................................................................uggCauguaaagaaguaugga.....................  18     1  HS7
......................................................................uggaauguaaagaaguauUga.....................  1      1  HS7
......................................................................uggaauguaaGgaaguaugga.....................  7      1  HS7
```

```
                              Star                               Mature
acauuucucaaucggugcccucuaguggaucccguacuucuucacaucuccauauuucauuggauauuuauggaauguaaagaaguauggaguuuacuaauguaaaacgagg
......................................................................ugAaauguaaagaaguaugga.....................    23     1   HS7
......................................................................uggaauAuaaagaaguaugga.....................    4      1   HS7
......................................................................uggaauguaaagaaguaugUa.....................    20     1   HS7
......................................................................uggaaGguaaagaaguaugga.....................    3      1   HS7
......................................................................uggaauguaaCgaaguaugga.....................    10     1   HS7
......................................................................uggaauguaaaUaaguaugga.....................    24     1   HS7
......................................................................uggaauguaaagaaguauggU.....................    66     1   HS7
......................................................................uggaauguaaaAaaguaugga.....................    3      1   HS7
......................................................................uggaauguaaagaaguaugga.....................    93842  0   HS7
......................................................................uggaauguaaagCaguaugga.....................    3      1   HS7
......................................................................uggaauguaaagaaAuaugga.....................    1      1   HS7
......................................................................Cggaauguaaagaaguaugga.....................    12     1   HS7
......................................................................uCgaauguaaagaaguaugga.....................    12     1   HS7
......................................................................uggaauguaGagaaguaugga.....................    6      1   HS7
......................................................................uggUauguaaagaaguaugga.....................    6      1   HS7
......................................................................uggaauguaaagaaguaCgga.....................    5      1   HS7
......................................................................uggaauguaaagaagGaugga.....................    1      1   HS7
......................................................................uggaauguGaagaaguaugga.....................    6      1   HS7
......................................................................uggaaugCaaagaaguaugga.....................    8      1   HS7
......................................................................uggaauguCaagaaguaugga.....................    3      1   HS7
......................................................................uggaUuguaaagaaguaugga.....................    33     1   HS7
......................................................................uggaauguaCagaaguaugga.....................    1      1   HS7
......................................................................uAgaauguaaagaaguaugga.....................    3      1   HS7
......................................................................uggaauguaaagaaguaGgga.....................    2      1   HS7
......................................................................uggaauguaaagaaguauggG.....................    156    1   HS7
......................................................................uggaauguaaagaaCuaugga.....................    6      1   HS7
......................................................................ugUaauguaaagaaguaugga.....................    47     1   HS7
......................................................................uggaauguaaagaGguaugga.....................    3      1   HS7
......................................................................uggaaugAaaagaaguaugga.....................    2      1   HS7
......................................................................uggaauguaaagaaUuaugga.....................    18     1   HS7
......................................................................uggaauCuaaagaaguaugga.....................    15     1   HS7
......................................................................uggaauguUaagaaguaugga.....................    8      1   HS7
......................................................................uggaauguaaagaaguUugga.....................    1      1   HS7
......................................................................uggaauguaaaCaaguaugga.....................    7      1   HS7
......................................................................uggaauguaaagaaguaugCa.....................    17     1   HS7
......................................................................uggaauguaaagaaguauAga.....................    2      1   HS7
......................................................................uggGauguaaagaaguaugga.....................    18     1   HS7
......................................................................uggaaCguaaagaaguaugga.....................    3      1   HS7
......................................................................Aggaauguaaagaaguaugga.....................    57     1   HS7
......................................................................Gggaauguaaagaaguaugga.....................    13     1   HS7
......................................................................uggaCuguaaagaaguaugga.....................    6      1   HS7
......................................................................uggaauguaaagaagAaugga.....................    1      1   HS7
......................................................................uggaauguaaagaaguGugga.....................    6      1   HS7
......................................................................uUgaauguaaagaaguaugga.....................    13     1   HS7
......................................................................uggaauguaaagUaguaugga.....................    1      1   HS7
......................................................................uggaauUuaaagaaguaugga.....................    16     1   HS7
......................................................................uggaauguaaagaaguaugAa.....................    14     1   HS7
......................................................................uggaauguaCagaaguauggag....................    8      1   HS7
......................................................................ugUaauguaaagaaguauggag....................    889    1   HS7
......................................................................uggaauguaaagUaguauggag....................    25     1   HS7
......................................................................uggaauguaaagaaguaugAag....................    109    1   HS7
......................................................................Aggaauguaaagaaguauggag....................    516    1   HS7
......................................................................uggaauCuaaagaaguauggag....................    117    1   HS7
......................................................................uggaauguaaagaagGauggag....................    14     1   HS7
......................................................................uggaauguaaagaGguauggag....................    56     1   HS7
......................................................................uggaauUuaaagaaguauggag....................    222    1   HS7
......................................................................uggaaugCaaagaaguauggag....................    54     1   HS7
......................................................................uggaGuguaaagaaguauggag....................    357    1   HS7
......................................................................uggaaCguaaagaaguauggag....................    58     1   HS7
......................................................................uggaauguaaagCaguauggag....................    40     1   HS7
......................................................................uggaauguaaagaaguauggGg....................    403    1   HS7
......................................................................uggaauguaaagaaguaAggag....................    2      1   HS7
......................................................................uggaauguaaagaCguauggag....................    13     1   HS7
......................................................................uggaauguaaaCaaguauggag....................    135    1   HS7
......................................................................uggaauguaaagaaguGuggag....................    55     1   HS7
......................................................................uggaauguaaagaagAauggag....................    4      1   HS7
......................................................................uggaauguaaagaaguaugCag....................    140    1   HS7
......................................................................uggaaAguaaagaaguauggag....................    6      1   HS7
......................................................................uggaauguGaagaaguauggag....................    49     1   HS7
......................................................................uggaauguaaagaaguaCggag....................    35     1   HS7
```

```
                               Star                                   Mature
acauuucucaaucggugcccucuaguggauc ccguacuucuucacaucuccaua uuucauuggauauuua uggaauguaaagaaguauggag uuuacuaauguaaaacgagg
```

```
acauuucucaaucggugcccucuaguggaucccguacuucuucacaucuccauauuucauuggauauuuauggaauguaaagaaguauggaguuuacuaauguaaaacgagg
......................................................................Cggaauguaaagaaguauggag....................	100	1	HS7
......................................................................uggaauguaaagaUguauggag....................	35	1	HS7
......................................................................uggaauguaaagaaCuauggag....................	229	1	HS7
......................................................................uggaauguaaagaaguauggaU....................	1622	1	HS7
......................................................................uggaaGguaaagaaguauggag....................	21	1	HS7
......................................................................uggaauguaaagaaguauggUg....................	241	1	HS7
......................................................................ugCaauguaaagaaguauggag....................	567	1	HS7
......................................................................uggaCuguaaagaaguauggag....................	47	1	HS7
......................................................................uggaUuguaaagaaguauggag....................	322	1	HS7
......................................................................uggaauguaaGgaaguauggag....................	27	1	HS7
......................................................................uggaauguCaagaaguauggag....................	23	1	HS7
......................................................................uCgaauguaaagaaguauggag....................	190	1	HS7
......................................................................uggaauAuaaagaaguauggag....................	36	1	HS7
......................................................................uggaauguaaagaaguaGggag....................	21	1	HS7
......................................................................uggaauguaaaUaaguauggag....................	209	1	HS7
......................................................................uggaauguaaCgaaguauggag....................	120	1	HS7
......................................................................uggaauguaaagaaguauggag....................	1127353	0	HS7
......................................................................uggaauguaaagaaguauAgag....................	31	1	HS7
......................................................................ugAaauguaaagaaguauggag....................	239	1	HS7
......................................................................uggaaugGaaagaaguauggag....................	47	1	HS7
......................................................................uggaauguaaagGaguauggag....................	69	1	HS7
......................................................................uggCauguaaagaaguauggag....................	219	1	HS7
......................................................................uggaauguaaagaagCauggag....................	69	1	HS7
......................................................................uggaauguaaagaaguauggCg....................	140	1	HS7
......................................................................uggaauguaaagaaguauUgag....................	124	1	HS7
......................................................................uggGauguaaagaaguauggag....................	137	1	HS7
......................................................................uggaauguUaagaaguauggag....................	124	1	HS7
......................................................................uggaauguaaagaaAuauggag....................	25	1	HS7
......................................................................uggaauguaaaAaaguauggag....................	39	1	HS7
......................................................................uggaauguaaagaaguaugUag....................	156	1	HS7
......................................................................Gggaauguaaagaaguauggag....................	146	1	HS7
......................................................................uggaauguaaagaaguauggaC....................	7169	1	HS7
......................................................................uggaaugAaaagaaguauggag....................	38	1	HS7
......................................................................uggaauguaaagaaguUuggag....................	27	1	HS7
......................................................................uggaauguaaagaaguauggaA....................	6373	1	HS7
......................................................................uAgaauguaaagaaguauggag....................	30	1	HS7
......................................................................uggaauguaGagaaguauggag....................	40	1	HS7
......................................................................uggaauguaUagaaguauggag....................	18	1	HS7
......................................................................uggaauguaaagaaUuauggag....................	447	1	HS7
......................................................................uggaauguaaagaaguauCgag....................	297	1	HS7
......................................................................uUgaauguaaagaaguauggag....................	144	1	HS7
......................................................................uggUauguaaagaaguauggag....................	66	1	HS7
......................................................................uggaauguaaagaaguCuggag....................	16	1	HS7
......................................................................uggaauguaaUgaaguauggag....................	12	1	HS7
......................................................................uggaauguaaagCaguauggagu...................	7	1	HS7
......................................................................uggaauguaaagaaguauggagC...................	954	1	HS7
......................................................................uggaauguaaCgaaguauggagu...................	11	1	HS7
......................................................................uggaauguaaagaaguGuggagu...................	3	1	HS7
......................................................................uggaauguaaaAaaguauggagu...................	3	1	HS7
......................................................................uggaauguaCagaaguauggagu...................	2	1	HS7
......................................................................uggaauguaaagaaguauggaAu...................	40	1	HS7
......................................................................uggGauguaaagaaguauggagu...................	10	1	HS7
......................................................................uggaauguaaagaGguauggagu...................	1	1	HS7
......................................................................Cggaauguaaagaaguauggagu...................	10	1	HS7
......................................................................uggaauguaaagaaguaugAagu...................	8	1	HS7
......................................................................ugCaauguaaagaaguauggagu...................	28	1	HS7
......................................................................uggaauguaaGgaaguauggagu...................	4	1	HS7
......................................................................uggaauCuaaagaaguauggagu...................	13	1	HS7
......................................................................uggaGuguaaagaaguauggagu...................	20	1	HS7
......................................................................uggaaugAaaagaaguauggagu...................	11	1	HS7
......................................................................ugAaauguaaagaaguauggagu...................	15	1	HS7
......................................................................uggaauguaaagaaguauUgagu...................	9	1	HS7
......................................................................uggaauguaaagaaguauggaUu...................	104	1	HS7
......................................................................uAgaauguaaagaaguauggagu...................	1	1	HS7
......................................................................uggaCuguaaagaaguauggagu...................	2	1	HS7
......................................................................uggaauguaaagaaguauggUgu...................	5	1	HS7
......................................................................uggaaCguaaagaaguauggagu...................	3	1	HS7
......................................................................uggaauguaaagaaguCuggagu...................	1	1	HS7
......................................................................uggaauguaaagUaguauggagu...................	3	1	HS7
......................................................................uggaauguaaaUaaguauggagu...................	20	1	HS7
```

```
                              Star                                     Mature
acauuucucaaucggugcccucuaguggaucccguacuucuucacaucuccauauuucauuggauauuuauggaauguaaagaaguauggaguuuacuaaauguaaaacgagg
......................................................................uggaauguaaagaaguauggaCu....................  172    1  HS7
......................................................................uggaauguaaaCaaguauggagu....................  10     1  HS7
......................................................................Aggaauguaaagaaguauggagu....................  30     1  HS7
......................................................................uggCauguaaagaaguauggagu....................  13     1  HS7
......................................................................ugUaauguaaagaaguauggagu....................  52     1  HS7
......................................................................uggaauguaaagaaguauggagA....................  78108  1  HS7
......................................................................uggaauguaaagaaAuauggagu....................  1      1  HS7
......................................................................Gggaauguaaagaaguauggagu....................  6      1  HS7
......................................................................uggaauguaaagaagCauggagu....................  3      1  HS7
......................................................................uggaauguaaagaaUuauggagu....................  23     1  HS7
......................................................................uggaauguaaagaaguauggagG....................  1220   1  HS7
......................................................................uggaauguaaagaaguaGggagu....................  1      1  HS7
......................................................................uUgaauguaaagaaguauggagu....................  9      1  HS7
......................................................................uggaauguaaagaaguauggCgu....................  27     1  HS7
......................................................................uggaauguaaagaaguaCggagu....................  7      1  HS7
......................................................................uggaauguaaagaaguauAgagu....................  1      1  HS7
......................................................................uggaauguaaagaaguauCgagu....................  17     1  HS7
......................................................................uggaUuguaaagaaguauggagu....................  15     1  HS7
......................................................................uggUauguaaagaaguauggagu....................  2      1  HS7
......................................................................uggaauguaaagaaguauggGgu....................  15     1  HS7
......................................................................uggaauUuaaagaaguauggagu....................  30     1  HS7
......................................................................uCgaauguaaagaaguauggagu....................  13     1  HS7
......................................................................uggaauguaaagaaguaugUagu....................  23     1  HS7
......................................................................uggaauguUaagaaguauggagu....................  6      1  HS7
......................................................................uggaauAuaaagaaguauggagu....................  3      1  HS7
......................................................................uggaauguGaagaaguauggagu....................  2      1  HS7
......................................................................uggaauguaaagaaguaugCagu....................  17     1  HS7
......................................................................uggaauguaaagaaguauggagu....................  65199  0  HS7
......................................................................uggaauguaaagaaCuauggagu....................  22     1  HS7
......................................................................uggaauguaUagaaguauggagu....................  2      1  HS7
......................................................................uggaaugGaaagaaguauggagu....................  2      1  HS7
......................................................................uggaauguaaUgaaguauggagu....................  2      1  HS7
......................................................................uggaauguaaagGaguauggagu....................  3      1  HS7
......................................................................uggaauguaaagaaguauggagCu...................  15     1  HS7
......................................................................uggaauguaaagaaguauggaguC...................  11     1  HS7
......................................................................uggaauguaaagaaguauggagAu...................  140    1  HS7
......................................................................uggaauguaaagaaguauggaguG...................  1      1  HS7
......................................................................uggaauAuaaagaaguauggaguu...................  1      1  HS7
......................................................................uggaauguaaagaaguauggaguu...................  576    0  HS7
......................................................................uggaauguaaagaaguaugCaguu...................  1      1  HS7
......................................................................uggaauguaaagaaguauggCguu...................  1      1  HS7
......................................................................uggaauguaaaUaaguauggaguu...................  1      1  HS7
......................................................................uggaauguaaagaaguauggagGu...................  18     1  HS7
......................................................................uggaauguaaagaaguauggaguA...................  110    1  HS7
......................................................................Aggaauguaaagaaguauggaguu...................  1      1  HS7
......................................................................uggaauguaaagaaguauggaCuu...................  1      1  HS7
......................................................................uggaauguaaagaaguauggagGuu..................  5      1  HS7
......................................................................uggaauguaaagaaguauggaguGu..................  4      1  HS7
......................................................................uggaauguaaagaaguauggaguuA..................  1      1  HS7
......................................................................uggaauguaaagaaguauggagAuu..................  14     1  HS7
......................................................................uggaauguaaagaaguauggaguAu..................  10     1  HS7
......................................................................uggaauguaaagaaguauggaguuC..................  1      1  HS7
......................................................................uggaauguaaagaaguauggaUuuu..................  1      1  HS7
......................................................................uggaauguaaagaaguauggaguuu..................  22     0  HS7
......................................................................uggaauguaaagaaguauggaguuuU.................  3      1  HS7
.......................................................................ggaauguaaagaaCuaugg.......................  1      1  HS7
.......................................................................Ugaauguaaagaaguaugg.......................  7      1  HS7
.......................................................................ggaauguaaagaaguaAgg.......................  1      1  HS7
.......................................................................ggaauguaaagaaguaugg.......................  27     0  HS7
.......................................................................gAaauguaaagaaguaugga......................  2      1  HS7
.......................................................................Cgaauguaaagaaguaugga......................  2      1  HS7
.......................................................................gUaauguaaagaaguaugga......................  1      1  HS7
.......................................................................Ugaauguaaagaaguaugga......................  28     1  HS7
.......................................................................ggaauguaaagaaguaugga......................  78     0  HS7
.......................................................................ggaaAguaaagaaguaugga......................  1      1  HS7
.......................................................................ggaauguaaagaaguauggaU.....................  1      1  HS7
.......................................................................ggaauguaaagaaguauggaC.....................  11     1  HS7
.......................................................................ggaauguaaagaaguauggaA.....................  4      1  HS7
.......................................................................gUaauguaaagaaguauggag.....................  5      1  HS7
.......................................................................ggaauguaaagGaguauggag.....................  1      1  HS7
```

```
                              Star                                    Mature
acauuucucaaucggugcccucuaguggaucccguacuucuucacaucuccauauuucauuggauauuuauggaauguaaagaaguauggaguuuacuaauguaaaacgagg
.......................................................................ggaauguaaagaaguauggag....................   1377   0   HS7
.......................................................................ggaauguaaagaaUuauggag....................   1      1   HS7
.......................................................................gAaauguaaagaaguauggag....................   4      1   HS7
.......................................................................ggaauguaaaUaaguauggag....................   1      1   HS7
.......................................................................ggaauguaaCgaaguauggag....................   1      1   HS7
.......................................................................gCaauguaaagaaguauggag....................   6      1   HS7
.......................................................................ggaaugGaaagaaguauggag....................   1      1   HS7
.......................................................................Ugaauguaaagaaguauggag....................   231    1   HS7
.......................................................................ggaauguaaaAaaguauggag....................   1      1   HS7
.......................................................................ggaauguaaagaaCuauggag....................   1      1   HS7
.......................................................................ggGauguaaagaaguauggag....................   3      1   HS7
.......................................................................ggaauguaaGgaaguauggag....................   1      1   HS7
.......................................................................Agaauguaaagaaguauggag....................   2      1   HS7
.......................................................................ggaauUuaaagaaguauggag....................   2      1   HS7
.......................................................................ggUauguaaagaaguauggag....................   3      1   HS7
.......................................................................ggaauguaaagaaguaCggag....................   1      1   HS7
.......................................................................ggaauguaaagCaguauggag....................   1      1   HS7
.......................................................................Cgaauguaaagaaguauggag....................   23     1   HS7
.......................................................................ggaauguaaagaaguauggGg....................   1      1   HS7
.......................................................................ggaaGguaaagaaguauggag....................   1      1   HS7
.......................................................................ggaauguaaagaaguauggGgu...................   1      1   HS7
.......................................................................Ugaauguaaagaaguauggagu...................   17     1   HS7
.......................................................................ggaauguaaagaaguauggagC...................   5      1   HS7
.......................................................................ggaauguaaagaaguauggaCu...................   1      1   HS7
.......................................................................ggaauguaaagaaguauggagA...................   127    1   HS7
.......................................................................ggaauguaaagaaguauggagu...................   136    0   HS7
.......................................................................Cgaauguaaagaaguauggagu...................   2      1   HS7
.......................................................................gAaauguaaagaaguauggagu...................   1      1   HS7
.......................................................................ggaauguaaagaaguauggaguu..................   2      0   HS7
........................................................................gaauguaaagaaguaugg......................   11     0   HS7
........................................................................Uaauguaaagaaguaugg......................   1      1   HS7
........................................................................Caauguaaagaaguaugga.....................   1      1   HS7
........................................................................gaauguaGagaaguaugga.....................   1      1   HS7
........................................................................gaauguaaagaaguaugga.....................   44     0   HS7
........................................................................gaGuguaaagaaguauggag....................   1      1   HS7
........................................................................Aaauguaaagaaguauggag....................   17     1   HS7
........................................................................gaauguaaagaaguauggaC....................   4      1   HS7
........................................................................gaauguaaaUaaguauggag....................   1      1   HS7
........................................................................gaauCuaaagaaguauggag....................   1      1   HS7
........................................................................gaauguaaagaaguauggag....................   524    0   HS7
........................................................................Caauguaaagaaguauggag....................   3      1   HS7
........................................................................gaauguaaagaaguauggaA....................   5      1   HS7
........................................................................gaauUuaaagaaguauggag....................   1      1   HS7
........................................................................gaauguaGagaaguauggag....................   1      1   HS7
........................................................................gaauguGaagaaguauggag....................   1      1   HS7
........................................................................Uaauguaaagaaguauggag....................   27     1   HS7
........................................................................gaauguaaagaaguauggagC...................   17     1   HS7
........................................................................Caauguaaagaaguauggagu...................   1      1   HS7
........................................................................gaauguaaagaaguauggagu...................   335    0   HS7
........................................................................Uaauguaaagaaguauggagu...................   1      1   HS7
........................................................................gaUuguaaagaaguauggagu...................   1      1   HS7
........................................................................gaaCguaaagaaguauggagu...................   1      1   HS7
........................................................................gaauguaaagaaguauggagA...................   145    1   HS7
........................................................................gGauguaaagaaguauggagu...................   1      1   HS7
........................................................................gaCuguaaagaaguauggagu...................   1      1   HS7
........................................................................Aaauguaaagaaguauggagu...................   1      1   HS7
........................................................................gaauguaaagaaguauggaguA..................   95     1   HS7
........................................................................gaauguaaagaaguauggaUuu..................   1      1   HS7
........................................................................gaauguaaagaaguauggaguu..................   201    0   HS7
........................................................................gaauguaaagaaguauggagGu..................   1      1   HS7
........................................................................gaauguaaagaaguauggagCu..................   1      1   HS7
........................................................................gaauguaaagaaguauggagAu..................   20     1   HS7
........................................................................gaauguaaagaaguauggaguC..................   1      1   HS7
........................................................................gaauguaaagaaguauggaguG..................   2      1   HS7
........................................................................gaauguaaagaaguaugAaguu..................   1      1   HS7
........................................................................gaauguaaagaaguauggaguuu.................   2      0   HS7
........................................................................gaauguaaagaaguauggaguuA.................   8      1   HS7
.........................................................................aauguaaagaaguaugga.....................   29     0   HS7
.........................................................................aauguaaagaaguauggaU....................   2      1   HS7
.........................................................................aauguaaagaaCuauggag....................   1      1   HS7
```

Star **Mature**

acauuucucaaucggugcccucuaguggaucccguacuucuucacaucuccauauuucauuggauauuuauggaauguaaagaaguauggaguuuacuaauguaaaacgagg			
....aauguaaagaaguauggaA....................	2	1	HS7
....aauguaaagaaguauggaC....................	1	1	HS7
....aauguaaagaUguauggag....................	1	1	HS7
....aGuguaaagaaguauggag....................	1	1	HS7
....aauguaaagaaguauggag....................	390	0	HS7
....Cauguaaagaaguauggag....................	1	1	HS7
....Uauguaaagaaguauggag....................	2	1	HS7
....aauCuaaagaaguauggag....................	1	1	HS7
....aauguaaagaaguauggagA...................	26	1	HS7
....aauguaaagaaguauggagC...................	1	1	HS7
....aauguaaagaaguauggagu...................	39	0	HS7
....Uuguaaagaaguauggag....................	1	1	HS7
....auguaaaUaaguauggag....................	1	1	HS7
....auguaaagaaguauggGg....................	1	1	HS7
....auUuaaagaaguauggag....................	3	1	HS7
....auguaaagaaguauggag....................	442	0	HS7
....auguUaagaaguauggag....................	1	1	HS7
....auguaaagaaguauggaC....................	2	1	HS7
....auguaaagaaguauggCg....................	1	1	HS7
....Guguaaagaaguauggag....................	1	1	HS7
....auguaaagaaguauggaUu...................	2	1	HS7
....auguaaagaaguauggagA...................	41	1	HS7
....auguaaagaaguauggagu...................	23	0	HS7
....auguaaagaaguauggagG...................	1	1	HS7
....uguaaagaaguauggagu...................	24	0	HS7
....uguaaagaaguauggagA...................	29	1	HS7
....uguaaagaaguauggagC...................	2	1	HS7
....guaaagaaguauggaguu..................	1	0	HS7
....guaaagaaguauggaguA..................	1	1	HS7
...............................ccguacuucuucacaucuccaua..	1	0	HS2
....uuauggaauguaaagaaguaug.......................	25	0	HS2
....uuCuggaauguaaagaaguauggag....................	1	1	HS2
....uauggaauguaaagaaguaug.......................	31	0	HS2
....uCuggaauguaaagaaguauggag....................	35	1	HS2
....auggaauguaaagaagAaugg......................	1	1	HS2
....auggaauguaaagaaguaugg......................	117	0	HS2
....auggaGuguaaagaaguaugg......................	2	1	HS2
....auggGauguaaagaaguaugga.....................	1	1	HS2
....Cuggaauguaaagaaguaugga.....................	632	1	HS2
....auggaauguaaagaaguaugga.....................	148	0	HS2
....Cuggaauguaaagaaguauggag....................	1932	1	HS2
....Uuggaauguaaagaaguauggag....................	19	1	HS2
....auggaauguaaagaaguauggag....................	275	0	HS2
....auggaauguaaagaaguauggagA...................	66	1	HS2
....Cuggaauguaaagaaguauggagu...................	146	1	HS2
....uggaauguaGagaaguau........................	1	1	HS2
....Gggaauguaaagaaguau........................	2	1	HS2
....uggaauguaaagaaguaA........................	21	1	HS2
....uggaauguaaagaaguau........................	512	0	HS2
....uggaauguaaagaaguaC........................	1	1	HS2
....uggaauguaUagaaguau........................	1	1	HS2
....uggaauguaaagaGguaug.......................	4	1	HS2
....Aggaauguaaagaaguaug.......................	5	1	HS2
....uggaaCguaaagaaguaug.......................	3	1	HS2
....ugUaauguaaagaaguaug.......................	1	1	HS2
....uggaauguaaagaaAuaug.......................	1	1	HS2
....uggaauguaaagaaguaCg.......................	2	1	HS2
....uggaaGguaaagaaguaug.......................	2	1	HS2
....uggaauguaGagaaguaug.......................	1	1	HS2
....uggGauguaaagaaguaug.......................	6	1	HS2
....Cggaauguaaagaaguaug.......................	2	1	HS2
....uggaauguaaagaaguaug.......................	7718	0	HS2
....Gggaauguaaagaaguaug.......................	7	1	HS2
....uggaUuguaaagaaguaug.......................	1	1	HS2
....uggaGuguaaagaaguaug.......................	2	1	HS2
....uggaauguaaagGaguaug.......................	2	1	HS2
....uggaauguaaagaagGaug.......................	1	1	HS2
....uggaauguaaagaagCaug.......................	5	1	HS2
....uggaauguGaagaaguaug.......................	1	1	HS2

Star **Mature**

acauuucucaaucggugcccucuaguggaucccguacuucuucacaucuccauauuucauuggauauuuauggaauguaaagaaguauggaguuuacuaauguaaaacgagg

....uggaaugGaaagaaguaug.......................	1	1	HS2
....uggaauguaaGgaaguaug.......................	1	1	HS2
....uggUauguaaagaaguaugg......................	1	1	HS2
....uggaCuguaaagaaguaugg......................	1	1	HS2
....uggaauguaaagaaguaugC......................	90	1	HS2
....uggaauguaaagCaguaugg......................	3	1	HS2
....uggaauguaaaAaaguaugg......................	1	1	HS2
....uggGauguaaagaaguaugg......................	15	1	HS2
....uggaauguaaagaaguaCgg......................	10	1	HS2
....uggaaugCaaagaaguaugg......................	10	1	HS2
....uggaUuguaaagaaguaugg......................	1	1	HS2
....uggaauguaaagaaguUugg......................	1	1	HS2
....Cggaauguaaagaaguaugg......................	34	1	HS2
....uggaauguaaagaaguaugA......................	158	1	HS2
....uggaauguaaagaaguauAg......................	5	1	HS2
....uAgaauguaaagaaguaugg......................	4	1	HS2
....uggaauguaaagaagAaugg......................	3	1	HS2
....uggaauguaaagaaguGugg......................	9	1	HS2
....uggaauguCaagaaguaugg......................	1	1	HS2
....uggCauguaaagaaguaugg......................	1	1	HS2
....uggaaugAaaagaaguaugg......................	2	1	HS2
....uggaauguaaagaaAuaugg......................	2	1	HS2
....uggaauguaaagaaUuaugg......................	1	1	HS2
....uggaauguaaGgaaguaugg......................	14	1	HS2
....uggaauguaaagaagGaugg......................	4	1	HS2
....uggaauguaaagaaguaugg......................	46586	0	HS2
....uggaauguaaagaaguaGgg......................	5	1	HS2
....uggaauguaGagaaguaugg......................	16	1	HS2
....uggaauguaaagaGguaugg......................	15	1	HS2
....uggaauguaaagaaguauUg......................	2	1	HS2
....uggaauUuaaagaaguaugg......................	2	1	HS2
....uggaauguaaagaUguaugg......................	1	1	HS2
....uggaauguaaagaagCaugg......................	12	1	HS2
....uggaaCguaaagaaguaugg......................	2	1	HS2
....Gggaauguaaagaaguaugg......................	44	1	HS2
....uggaauguaaagaaguaAgg......................	1	1	HS2
....uggaauguaaagGaguaugg......................	12	1	HS2
....uggaauguGaagaaguaugg......................	9	1	HS2
....uggaauguaUagaaguaugg......................	1	1	HS2
....uggaauguaaagUaguaugg......................	1	1	HS2
....ugUaauguaaagaaguaugg......................	1	1	HS2
....uggaaGguaaagaaguaugg......................	6	1	HS2
....Aggaauguaaagaaguaugg......................	19	1	HS2
....ugAaauguaaagaaguaugg......................	6	1	HS2
....uUgaauguaaagaaguaugg......................	2	1	HS2
....uggaGuguaaagaaguaugg......................	13	1	HS2
....uggaauguaaagaaguaugU......................	613	1	HS2
....uggaaugGaaagaaguaugg......................	4	1	HS2
....ugCaauguaaagaaguaugg......................	2	1	HS2
....uggaauAuaaagaaguaugg......................	1	1	HS2
....uggaaugAaaagaaguaugga.....................	109	1	HS2
....uggaauguaaagGaguaugga.....................	282	1	HS2
....uggaauguaaagaUguaugga.....................	12	1	HS2
....uggaCuguaaagaaguaugga.....................	5	1	HS2
....uggaauguaaagaGguaugga.....................	340	1	HS2
....uggaauguaaagaaguaugCa.....................	11	1	HS2
....uggaauguaaagaaguauggC.....................	19	1	HS2
....ugCaauguaaagaaguaugga.....................	16	1	HS2
....uggaauguaaagaaguUugga.....................	25	1	HS2
....uUgaauguaaagaaguaugga.....................	51	1	HS2
....uggaauguaaagCaguaugga.....................	24	1	HS2
....uggaauguaaagaaguaugAa.....................	130	1	HS2
....uggaauguaaaCaaguaugga.....................	6	1	HS2
....uggaauguaCagaaguaugga.....................	24	1	HS2
....uAgaauguaaagaaguaugga.....................	147	1	HS2
....uggaauguaaUgaaguaugga.....................	11	1	HS2
....Cggaauguaaagaaguaugga.....................	1109	1	HS2
....uggaauguaaagaaguaGgga.....................	34	1	HS2
....uggCauguaaagaaguaugga.....................	32	1	HS2
....uggaauguaaagaaUuaugga.....................	20	1	HS2

```
                              Star                                     Mature
acauuucucaaucggugcccucuaguggaucccguacuucuucacaucuccauauuucauuggauauuuauggaauguaaagaaguauggaguuuacuaauguaaaacgagg
......................................................................uggaauguaaagaaguaAgga.....................  45       1  HS2
......................................................................uggaGuguaaagaaguaugga.....................  361      1  HS2
......................................................................Gggaauguaaagaaguaugga.....................  1171     1  HS2
......................................................................Aggaauguaaagaaguaugga.....................  645      1  HS2
......................................................................uggaauguaaagaaguaugUa.....................  743      1  HS2
......................................................................uggaauguaaGgaaguaugga.....................  290      1  HS2
......................................................................uggaauguaGagaaguaugga.....................  335      1  HS2
......................................................................uggaauguaUagaaguaugga.....................  23       1  HS2
......................................................................uggaauguaaagaagGaugga.....................  127      1  HS2
......................................................................uggaauguaaagaagAaugga.....................  69       1  HS2
......................................................................uggaauguaaaAaaguaugga.....................  24       1  HS2
......................................................................uggaauguaaagaaCuaugga.....................  8        1  HS2
......................................................................uggaauguaaagaaguauAga.....................  157      1  HS2
......................................................................uggGauguaaagaaguaugga.....................  508      1  HS2
......................................................................uggaauguaaagaaguaugga.....................  1375330  0  HS2
......................................................................uggaauguaaaUaaguaugga.....................  24       1  HS2
......................................................................uggaaugCaaagaaguaugga.....................  221      1  HS2
......................................................................uggaauguaaagaaguauggG.....................  541      1  HS2
......................................................................uggaauguaaagaagCaugga.....................  297      1  HS2
......................................................................uggaauguaaagaaguauUga.....................  36       1  HS2
......................................................................uggaauUuaaagaaguaugga.....................  45       1  HS2
......................................................................uggaauguaaagaCguaugga.....................  7        1  HS2
......................................................................uggaauguaaagaaguaCgga.....................  220      1  HS2
......................................................................uggaaAguaaagaaguaugga.....................  87       1  HS2
......................................................................uggaauCuaaagaaguaugga.....................  16       1  HS2
......................................................................uggaauguaaagaaguauggU.....................  85       1  HS2
......................................................................uggaauguaaagUaguaugga.....................  29       1  HS2
......................................................................uggaauguaaagaaguGugga.....................  250      1  HS2
......................................................................uggaauguaaagaaguauCga.....................  39       1  HS2
......................................................................uggaauguUaagaaguaugga.....................  8        1  HS2
......................................................................uggaaGguaaagaaguaugga.....................  328      1  HS2
......................................................................uggaauguaaCgaaguaugga.....................  7        1  HS2
......................................................................uggaauAuaaagaaguaugga.....................  138      1  HS2
......................................................................uCgaauguaaagaaguaugga.....................  19       1  HS2
......................................................................uggUauguaaagaaguaugga.....................  33       1  HS2
......................................................................uggaauguaaagaaguCugga.....................  10       1  HS2
......................................................................uggaauguCaagaaguaugga.....................  6        1  HS2
......................................................................ugUaauguaaagaaguaugga.....................  40       1  HS2
......................................................................ugAaauguaaagaaguaugga.....................  145      1  HS2
......................................................................uggaauguGaagaaguaugga.....................  259      1  HS2
......................................................................uggaaCguaaagaaguaugga.....................  249      1  HS2
......................................................................uggaaugGaaagaaguaugga.....................  232      1  HS2
......................................................................uggaauguaaagaaAuaugga.....................  69       1  HS2
......................................................................uggaUuguaaagaaguaugga.....................  25       1  HS2
......................................................................uggaaugAaaagaaguauggag....................  394      1  HS2
......................................................................uggUauguaaagaaguauggag....................  171      1  HS2
......................................................................uggaauguaaagaaguCuggag....................  43       1  HS2
......................................................................uggaauguaaagaaguauAgag....................  599      1  HS2
......................................................................uggaauguUaagaaguauggag....................  38       1  HS2
......................................................................uggaaugGaaagaaguauggag....................  736      1  HS2
......................................................................uggaauguaaagGaguauggag....................  1328     1  HS2
......................................................................uggaGuguaaagaaguauggag....................  1626     1  HS2
......................................................................uggaaGguaaagaaguauggag....................  1243     1  HS2
......................................................................uggaauguaaagaaguaugAag....................  296      1  HS2
......................................................................uggaauguaaagaaAuauggag....................  185      1  HS2
......................................................................uggaauguaaaUaaguauggag....................  91       1  HS2
......................................................................uggaauguaUagaaguauggag....................  143      1  HS2
......................................................................uggaaugCaaagaaguauggag....................  990      1  HS2
......................................................................uggaaAguaaagaaguauggag....................  255      1  HS2
......................................................................uggaauguaaagUaguauggag....................  190      1  HS2
......................................................................ugCaauguaaagaaguauggag....................  84       1  HS2
......................................................................uggaauguaaagaagGauggag....................  1094     1  HS2
......................................................................uggaauguaaagaaguaCggag....................  990      1  HS2
......................................................................uCgaauguaaagaaguauggag....................  102      1  HS2
......................................................................uggaauguaaagaagAauggag....................  645      1  HS2
......................................................................uggGauguaaagaaguauggag....................  2198     1  HS2
......................................................................uggaUuguaaagaaguauggag....................  106      1  HS2
......................................................................uggaauAuaaagaaguauggag....................  518      1  HS2
......................................................................uggaauguaaagaaguauggaA....................  41907    1  HS2
......................................................................Gggaauguaaagaaguauggag....................  4471     1  HS2
```

Star Mature

```
acauuucucaaucggugcccucuaguggaucccguacuucuucacaucuccauauuucauuggauauuuauggaauguaaagaaguauggaguuuacuaauguaaaacgagg
......................................................................uggaCuguaaagaaguauggag....................   42        1    HS2
......................................................................uggaauguaaagaaguUuggag....................   65        1    HS2
......................................................................uggCauguaaagaaguauggag....................   114       1    HS2
......................................................................uggaauguaaagaaguauggUg....................   139       1    HS2
......................................................................uggaauguaaagaaguauggCg....................   85        1    HS2
......................................................................uggaauguaaagaaguauggGg....................   2342      1    HS2
......................................................................uggaauguaaagaaguauggaC....................   977       1    HS2
......................................................................uggaauguaaagaaguauggaU....................   4466      1    HS2
......................................................................uggaauguaaagaaguauUgag....................   118       1    HS2
......................................................................uggaauguaaagaaUuauggag....................   78        1    HS2
......................................................................uggaauguCaagaaguauggag....................   23        1    HS2
......................................................................uggaauguaaaAaaguauggag....................   215       1    HS2
......................................................................uggaauguaaagaaguaGggag....................   948       1    HS2
......................................................................uggaaCguaaagaaguauggag....................   1047      1    HS2
......................................................................uggaauguaaagaaCuauggag....................   46        1    HS2
......................................................................uAgaauguaaagaaguauggag....................   737       1    HS2
......................................................................uggaauguGaagaaguauggag....................   1108      1    HS2
......................................................................uggaauguaaagaCguauggag....................   43        1    HS2
......................................................................uggaauUuaaagaaguauggag....................   170       1    HS2
......................................................................ugAaauguaaagaaguauggag....................   313       1    HS2
......................................................................Aggaauguaaagaaguauggag....................   2636      1    HS2
......................................................................uggaauguaaagaGguauggag....................   1602      1    HS2
......................................................................uggaauguaGagaaguauggag....................   1554      1    HS2
......................................................................ugUaauguaaagaaguauggag....................   133       1    HS2
......................................................................uUgaauguaaagaaguauggag....................   213       1    HS2
......................................................................uggaauguaaagaagCauggag....................   1436      1    HS2
......................................................................uggaauguaaaCaaguauggag....................   26        1    HS2
......................................................................uggaauguaaagaaguauggag....................   6054998   0    HS2
......................................................................uggaauguaaGgaaguauggag....................   1262      1    HS2
......................................................................uggaauguaaagaaguaugUag....................   295       1    HS2
......................................................................uggaauguaaagaaguaugCag....................   50        1    HS2
......................................................................uggaauguaaUgaaguauggag....................   52        1    HS2
......................................................................Cggaauguaaagaaguauggag....................   4152      1    HS2
......................................................................uggaauCuaaagaaguauggag....................   63        1    HS2
......................................................................uggaauguaaagaaguauCgag....................   104       1    HS2
......................................................................uggaauguaCagaaguauggag....................   105       1    HS2
......................................................................uggaauguaaagaUguauggag....................   93        1    HS2
......................................................................uggaauguaaagaaguGuggag....................   1167      1    HS2
......................................................................uggaauguaaagCaguauggag....................   137       1    HS2
......................................................................uggaauguaaCgaaguauggag....................   41        1    HS2
......................................................................uggaauguaaagaaguaAggag....................   407       1    HS2
......................................................................uggaauguaaagCaguauggagu...................   6         1    HS2
......................................................................uggaauguaaGgaaguauggagu...................   39        1    HS2
......................................................................uggaauguaaaUaaguauggagu...................   4         1    HS2
......................................................................uggaauguaaagaaguauUgagu...................   2         1    HS2
......................................................................uggaauguaaagaUguauggagu...................   5         1    HS2
......................................................................uggaauguaaagaaguGuggagu...................   35        1    HS2
......................................................................uggaauguaaagaagAauggagu...................   19        1    HS2
......................................................................ugAaauguaaagaaguauggagu...................   63        1    HS2
......................................................................uggaauguaaagaaguauggaUu...................   256       1    HS2
......................................................................uggaauguaaaAaaguauggagu...................   5         1    HS2
......................................................................uggaauguaaagaaguaugCagu...................   5         1    HS2
......................................................................uggaauguaaagaaUuauggagu...................   1         1    HS2
......................................................................Aggaauguaaagaaguauggagu...................   76        1    HS2
......................................................................uggaauguGaagaaguauggagu...................   28        1    HS2
......................................................................uUgaauguaaagaaguauggagu...................   7         1    HS2
......................................................................uggaauUuaaagaaguauggagu...................   2         1    HS2
......................................................................uggaauguaaCgaaguauggagu...................   1         1    HS2
......................................................................uggaauguaaagaaguauggaAu...................   44        1    HS2
......................................................................uggaauguaaagaaguaugUagu...................   9         1    HS2
......................................................................uggGauguaaagaaguauggagu...................   66        1    HS2
......................................................................Cggaauguaaagaaguauggagu...................   120       1    HS2
......................................................................uggaauguaaagaaguauggaCu...................   41        1    HS2
......................................................................uggUauguaaagaaguauggagu...................   4         1    HS2
......................................................................uCgaauguaaagaaguauggagu...................   4         1    HS2
......................................................................uggaauguaaagaaguauggGgu...................   68        1    HS2
......................................................................uggaauAuaaagaaguauggagu...................   11        1    HS2
......................................................................uggaauguaaagaGguauggagu...................   46        1    HS2
......................................................................uggaauguaUagaaguauggagu...................   3         1    HS2
......................................................................ugCaauguaaagaaguauggagu...................   2         1    HS2
```

```
                              Star                                  Mature
acauuucucaaucggugcccucuaguggaucccguacuucuucacaucuccauauuucauuggauauuuauggaauguaaagaaguauggaguuuacuaauguaaaacgagg
......................................................................uggaauguaaagaagGauggagu...................    35      1   HS2
......................................................................uggaaugCaaagaaguauggagu...................    32      1   HS2
......................................................................uggaauguaGagaaguauggagu...................    39      1   HS2
......................................................................uggCauguaaagaaguauggagu...................    2       1   HS2
......................................................................uggaauguaaagaaguauAgagu...................    16      1   HS2
......................................................................uggaauguaaagaaguauggagA...................    617383  1   HS2
......................................................................uggaaugGaaagaaguauggagu...................    32      1   HS2
......................................................................uggaCuguaaagaaguauggagu...................    3       1   HS2
......................................................................uggaauguaaagaagCauggagu...................    28      1   HS2
......................................................................uggaauguaaagaaguaAggagu...................    8       1   HS2
......................................................................uggaauguaaagaaguaugAagu...................    12      1   HS2
......................................................................uggaauguaaagaaguauggagG...................    1045    1   HS2
......................................................................uggaauguaCagaaguauggagu...................    2       1   HS2
......................................................................uggaauguaaagaaguaCggagu...................    29      1   HS2
......................................................................Gggaauguaaagaaguauggagu...................    131     1   HS2
......................................................................uggaaCguaaagaaguauggagu...................    30      1   HS2
......................................................................uggaaugAaaagaaguauggagu...................    9       1   HS2
......................................................................uAgaauguaaagaaguauggagu...................    21      1   HS2
......................................................................uggaaGguaaagaaguauggagu...................    37      1   HS2
......................................................................uggaauguaaagUaguauggagu...................    8       1   HS2
......................................................................uggaauguaaagaaguauggUgu...................    5       1   HS2
......................................................................uggaauCuaaagaaguauggagu...................    2       1   HS2
......................................................................uggaauguaaaCaaguauggagu...................    1       1   HS2
......................................................................uggaauguaaagaaAuauggagu...................    6       1   HS2
......................................................................uggaauguaaagaaguaGggagu...................    21      1   HS2
......................................................................uggaauguaaagaaguauggagC...................    2344    1   HS2
......................................................................uggaauguaaagGaguauggagu...................    37      1   HS2
......................................................................uggaUuguaaagaaguauggagu...................    6       1   HS2
......................................................................uggaauguaaagaaguauggagu...................    175648  0   HS2
......................................................................ugUaauguaaagaaguauggagu...................    3       1   HS2
......................................................................uggaaAguaaagaaguauggagu...................    6       1   HS2
......................................................................uggaGuguaaagaaguauggagu...................    46      1   HS2
......................................................................uggaauguaaagaCguauggagu...................    2       1   HS2
......................................................................uggaauguaaagaaguauggaUuu..................    32      1   HS2
......................................................................uggGauguaaagaaguauggaguu..................    1       1   HS2
......................................................................uggaauguaaagaaguauggagGu..................    1       1   HS2
......................................................................uggaauguaaagaaguauggaguu..................    1384    0   HS2
......................................................................Cggaauguaaagaaguauggaguu..................    1       1   HS2
......................................................................uggaauguaaagaaguauggagAu..................    312     1   HS2
......................................................................Gggaauguaaagaaguauggaguu..................    1       1   HS2
......................................................................uggaauguGaagaaguauggaguu..................    1       1   HS2
......................................................................uggaauguaaagaaguauggaguG..................    1       1   HS2
......................................................................uggaauguaaGgaaguauggaguu..................    1       1   HS2
......................................................................Aggaauguaaagaaguauggaguu..................    1       1   HS2
......................................................................uggaauguaaagaaguauggaguA..................    237     1   HS2
......................................................................uggaauguaaagaaguauggagCu..................    43      1   HS2
......................................................................uggaaugCaaagaaguauggaguu..................    1       1   HS2
......................................................................uggaGuguaaagaaguauggaguu..................    1       1   HS2
......................................................................uggaauguaaagaaguauggagAuu.................    119     1   HS2
......................................................................uggaauguaaagaaguauggaguCu.................    39      1   HS2
......................................................................uggaauguaaagaaguauggaguuu.................    15      0   HS2
......................................................................uggaauguaaagaaguauggaguAu.................    34      1   HS2
.......................................................................ggaauguaaagGaguaug.......................    1       1   HS2
.......................................................................ggGauguaaagaaguaug.......................    1       1   HS2
.......................................................................ggaauguaaagaagGaug.......................    1       1   HS2
.......................................................................ggaaCguaaagaaguaug.......................    1       1   HS2
.......................................................................ggaauguaaagaaguauA.......................    1       1   HS2
.......................................................................ggaauguaaagaaguaug.......................    595     0   HS2
.......................................................................ggaauguaGagaaguaugg......................    2       1   HS2
.......................................................................gAaauguaaagaaguaugg......................    1       1   HS2
.......................................................................ggaaugGaaagaaguaugg......................    1       1   HS2
.......................................................................ggaauguaaagaGguaugg......................    1       1   HS2
.......................................................................ggaauguaaagaaguaugU......................    88      1   HS2
.......................................................................ggaauguaaagaaguaGgg......................    1       1   HS2
.......................................................................ggaauguaaagaaguaCgg......................    2       1   HS2
.......................................................................ggaauguaaagaagGaugg......................    3       1   HS2
.......................................................................ggUauguaaagaaguaugg......................    1       1   HS2
.......................................................................ggaauguaaagGaguaugg......................    3       1   HS2
.......................................................................Ugaauguaaagaaguaugg......................    6       1   HS2
.......................................................................ggaauguaaagaaguaugg......................    3572    0   HS2
```

Star Mature

acauuucucaaucggugcccucuaguggaucccguacuucuucacaucuccauauuucauuggauauuuauggaauguaaagaaguauggaguuuacuaauguaaaacgagg			
....ggaauguaaagaaCuaugg......................	1	1	HS2
....Agaauguaaagaaguaugg......................	1	1	HS2
....ggaauguaaagaaguauAg......................	1	1	HS2
....ggaauguaaagaaguaugA......................	4	1	HS2
....ggaauguaaagaagCaugg......................	1	1	HS2
....ggaauguaaaAaaguaugga.....................	3	1	HS2
....ggaauguaaagaaguaugCa.....................	3	1	HS2
....ggaauguaaagaaguaAgga.....................	4	1	HS2
....ggaaugCaaagaaguaugga.....................	26	1	HS2
....ggaCuguaaagaaguaugga.....................	1	1	HS2
....ggaauguaaagaaguaugUa.....................	72	1	HS2
....ggaauguaaagaaguaugAa.....................	4	1	HS2
....ggaauguaaGgaaguaugga.....................	20	1	HS2
....ggaaCguaaagaaguaugga.....................	14	1	HS2
....ggGauguaaagaaguaugga.....................	45	1	HS2
....ggaauguaaagaagGaugga.....................	24	1	HS2
....ggaGuguaaagaaguaugga.....................	32	1	HS2
....ggaauguaaagaaguauggG.....................	40	1	HS2
....ggaauCuaaagaaguaugga.....................	1	1	HS2
....ggaaugGaaagaaguaugga.....................	35	1	HS2
....gUaauguaaagaaguaugga.....................	4	1	HS2
....ggaauguaaagaaUuaugga.....................	3	1	HS2
....ggaaugAaaagaaguaugga.....................	5	1	HS2
....ggaauguaaagaaguauUga.....................	4	1	HS2
....ggaauguaaaUaaguaugga.....................	3	1	HS2
....ggaauUuaaagaaguaugga.....................	3	1	HS2
....ggaauguaaagaaguauggC.....................	5	1	HS2
....ggaauguaaagaaguauggU.....................	9	1	HS2
....Cgaauguaaagaaguaugga.....................	6	1	HS2
....ggaauguaaagaagAaugga.....................	10	1	HS2
....ggaauguaaagCaguaugga.....................	5	1	HS2
....ggaauguaaagaaguGugga.....................	22	1	HS2
....ggaaAguaaagaaguaugga.....................	20	1	HS2
....ggUauguaaagaaguaugga.....................	2	1	HS2
....Ugaauguaaagaaguaugga.....................	136	1	HS2
....ggaauAuaaagaaguaugga.....................	5	1	HS2
....gCaauguaaagaaguaugga.....................	4	1	HS2
....ggaauguaaagaaguaGgga.....................	1	1	HS2
....ggaauguaaUgaaguaugga.....................	2	1	HS2
....Agaauguaaagaaguaugga.....................	23	1	HS2
....ggaauguGaagaaguaugga.....................	26	1	HS2
....ggaauguaaagaagCaugga.....................	20	1	HS2
....ggCauguaaagaaguaugga.....................	2	1	HS2
....ggaaGguaaagaaguaugga.....................	47	1	HS2
....ggaauguaaagaaguaugga.....................	114307	0	HS2
....ggaUuguaaagaaguaugga.....................	5	1	HS2
....ggaauguaUagaaguaugga.....................	2	1	HS2
....ggaauguaaagaaguauCga.....................	3	1	HS2
....ggaauguaaagaaguauAga.....................	16	1	HS2
....ggaauguaaagaaguUugga.....................	1	1	HS2
....ggaauguaaagUaguaugga.....................	1	1	HS2
....gAaauguaaagaaguaugga.....................	11	1	HS2
....ggaauguaaCgaaguaugga.....................	3	1	HS2
....ggaauguaaagaGguaugga.....................	22	1	HS2
....ggaauguaaagGaguaugga.....................	26	1	HS2
....ggaauguaaagaaguaCgga.....................	56	1	HS2
....ggaauguaGagaaguaugga.....................	18	1	HS2
....ggaauguaaagaaAuaugga.....................	8	1	HS2
....ggaauguaaagaaguauCgag....................	7	1	HS2
....Ugaauguaaagaaguauggag....................	636	1	HS2
....ggaauguaaGgaaguauggag....................	120	1	HS2
....ggaauguaGagaaguauggag....................	123	1	HS2
....ggaauguaaagaaguaugAag....................	27	1	HS2
....ggaCuguaaagaaguauggag....................	4	1	HS2
....ggaauguaaagaaguauggGg....................	197	1	HS2
....ggaauguaaagUaguauggag....................	16	1	HS2
....ggUauguaaagaaguauggag....................	13	1	HS2
....ggaauguaaUgaaguauggag....................	13	1	HS2
....ggaauguaaagaaguaugCag....................	10	1	HS2
....ggaauguaCagaaguauggag....................	7	1	HS2

```
                                                      Star                                 Mature
acauuucucaaucggugcccucuaguggaucccguacuucuucacaucuccauauuucauuggauauuuauggaauguaaagaaguauggaguuuacuaauguaaaacgagg
.......................................................................ggaauguaaaCaaguauggag....................  3       1  HS2
.......................................................................ggaauguaaaAaaguauggag....................  15      1  HS2
.......................................................................ggaauguaaagaagCauggag....................  166     1  HS2
.......................................................................ggaGuguaaagaaguauggag....................  111     1  HS2
.......................................................................ggaauguaaagaaUuauggag....................  3       1  HS2
.......................................................................ggaauguaaagaUguauggag....................  6       1  HS2
.......................................................................ggCauguaaagaaguauggag....................  8       1  HS2
.......................................................................ggaaGguaaagaaguauggag....................  131     1  HS2
.......................................................................ggaauguaaagaaguaCggag....................  97      1  HS2
.......................................................................ggaauguaUagaaguauggag....................  5       1  HS2
.......................................................................ggaauguaaagCaguauggag....................  16      1  HS2
.......................................................................ggaauguaaagaaguUuggag....................  12      1  HS2
.......................................................................Cgaauguaaagaaguauggag....................  33      1  HS2
.......................................................................ggaauguaaagaaguaugUag....................  10      1  HS2
.......................................................................gCaauguaaagaaguauggag....................  8       1  HS2
.......................................................................ggaauUuaaagaaguauggag....................  14      1  HS2
.......................................................................ggaUuguaaagaaguauggag....................  12      1  HS2
.......................................................................Agaauguaaagaaguauggag....................  136     1  HS2
.......................................................................ggaauguaaagaagAauggag....................  77      1  HS2
.......................................................................ggaaCguaaagaaguauggag....................  70      1  HS2
.......................................................................ggaauguaaagaaCuauggag....................  3       1  HS2
.......................................................................ggaauguaaagaaAuauggag....................  16      1  HS2
.......................................................................ggaauguaaagaaguauggCg....................  15      1  HS2
.......................................................................ggaaAguaaagaaguauggag....................  74      1  HS2
.......................................................................gUaauguaaagaaguauggag....................  18      1  HS2
.......................................................................gAaauguaaagaaguauggag....................  39      1  HS2
.......................................................................ggaauguaaagaCguauggag....................  3       1  HS2
.......................................................................ggaauguCaagaaguauggag....................  1       1  HS2
.......................................................................ggaauguaaaUaaguauggag....................  10      1  HS2
.......................................................................ggaauguaaagaaguaGggag....................  54      1  HS2
.......................................................................ggaauguaaagaaguaAggag....................  62      1  HS2
.......................................................................ggaauguaaagaaguauggaU....................  611     1  HS2
.......................................................................ggGauguaaagaaguauggag....................  174     1  HS2
.......................................................................ggaauguaaagaaguauggag....................  495081  0  HS2
.......................................................................ggaauguaaagaagGauggag....................  194     1  HS2
.......................................................................ggaauguUaagaaguauggag....................  3       1  HS2
.......................................................................ggaaugGaaagaaguauggag....................  94      1  HS2
.......................................................................ggaauguaaagaaguauggaA....................  2823    1  HS2
.......................................................................ggaauguaaagGaguauggag....................  119     1  HS2
.......................................................................ggaauguaaagaaguauggUg....................  16      1  HS2
.......................................................................ggaauguaaagaGguauggag....................  115     1  HS2
.......................................................................ggaaugAaaagaaguauggag....................  18      1  HS2
.......................................................................ggaaugCaaagaaguauggag....................  112     1  HS2
.......................................................................ggaauguGaagaaguauggag....................  136     1  HS2
.......................................................................ggaauguaaagaaguauggaC....................  102     1  HS2
.......................................................................ggaauguaaagaaguauAgag....................  56      1  HS2
.......................................................................ggaauguaaCgaaguauggag....................  16      1  HS2
.......................................................................ggaauAuaaagaaguauggag....................  58      1  HS2
.......................................................................ggaauguaaagaaguGuggag....................  94      1  HS2
.......................................................................ggaauguaaagaaguCuggag....................  8       1  HS2
.......................................................................ggaauguaaagaaguauUgag....................  23      1  HS2
.......................................................................ggaauCuaaagaaguauggag....................  4       1  HS2
.......................................................................ggaauguaaagaagAauggagu...................  1       1  HS2
.......................................................................ggaauguaaagaaguauggaAu...................  1       1  HS2
.......................................................................ggaauguaaagaUguauggagu...................  1       1  HS2
.......................................................................ggaauguaaagaaguauggagG...................  163     1  HS2
.......................................................................ggaauguaaagaaguauggaUu...................  23      1  HS2
.......................................................................ggaaCguaaagaaguauggagu...................  7       1  HS2
.......................................................................ggaaAguaaagaaguauggagu...................  1       1  HS2
.......................................................................ggaauguaaagaaguauggaCu...................  1       1  HS2
.......................................................................ggaaugAaaagaaguauggagu...................  1       1  HS2
.......................................................................ggaauguaaagaagCauggagu...................  1       1  HS2
.......................................................................ggGauguaaagaaguauggagu...................  8       1  HS2
.......................................................................ggaaugCaaagaaguauggagu...................  2       1  HS2
.......................................................................ggaauguaaagaaguauAgagu...................  1       1  HS2
.......................................................................ggaauguaaagaaguauggagC...................  141     1  HS2
.......................................................................ggaauguaaagaaguaugUagu...................  1       1  HS2
.......................................................................ggaauguaaagaaguauggUgu...................  3       1  HS2
.......................................................................Agaauguaaagaaguauggagu...................  7       1  HS2
.......................................................................ggaauguGaagaaguauggagu...................  16      1  HS2
```

Star Mature

```
acauuucucaaucggugcccucuaguggaucccguacuucuucacaucuccauauuucauuggauauuuauggaauguaaagaaguauggaguuuacuaauguaaaacgagg
.......................................................................ggaauguaaagaaguauggGgu...................	3	1	HS2
.......................................................................ggaauguaaagaGguauggagu...................	5	1	HS2
.......................................................................ggaauguaaagaaguGuggagu...................	2	1	HS2
.......................................................................ggaauguaaagaaguaugAagu...................	2	1	HS2
.......................................................................ggaauguaaagaaguaGggagu...................	3	1	HS2
.......................................................................ggaauguaaagaaguauggCgu...................	1	1	HS2
.......................................................................gAaauguaaagaaguauggagu...................	1	1	HS2
.......................................................................Ugaauguaaagaaguauggagu...................	21	1	HS2
.......................................................................ggaauguaaGgaaguauggagu...................	5	1	HS2
.......................................................................ggaauguaaagaaguaAggagu...................	3	1	HS2
.......................................................................ggaaugGaaagaaguauggagu...................	2	1	HS2
.......................................................................ggaauguaaagaaguaCggagu...................	4	1	HS2
.......................................................................ggaauguaGagaaguauggagu...................	3	1	HS2
.......................................................................ggaauguaaagaaguauggagu...................	13910	0	HS2
.......................................................................ggaauguaaagaagGauggagu...................	6	1	HS2
.......................................................................ggaauguaaaUaaguauggagu...................	1	1	HS2
.......................................................................ggaGuguaaagaaguauggagu...................	2	1	HS2
.......................................................................ggaauguaaagGaguauggagu...................	2	1	HS2
.......................................................................ggaauguaaagaaguauggagA...................	46809	1	HS2
.......................................................................ggaauguaaagaaguaugCagu...................	1	1	HS2
.......................................................................ggaaGguaaagaaguauggagu...................	1	1	HS2
.......................................................................ggaauguaaagaaguauggaguA..................	39	1	HS2
.......................................................................ggaauguaaagaaguauggagAu..................	26	1	HS2
.......................................................................ggaauguaaagaaguauggaguu..................	92	0	HS2
.......................................................................ggaauguaaagaaguaCggaguu..................	1	1	HS2
........................................................................gaauguaaagaaguaugga.....................	2	0	HS2
........................................................................Uaauguaaagaaguauggag....................	1	1	HS2
........................................................................gaauguaaagaaguauggag....................	9	0	HS2
........................................................................gGauguaaagaaguauggag....................	4	1	HS2
........................................................................gaauguaaagaaguauggagA...................	1	1	HS2
........................................................................gaauguaaagaaguauggaguA..................	46	1	HS2
........................................................................gaauguaaagaaguauggaguu..................	36	0	HS2
.........................................................................aauguaaagaaguaugga.....................	34	0	HS2
.........................................................................aauguaaagaaguauggag....................	142	0	HS2
.........................................................................aauguaaagaagCauggag....................	1	1	HS2
.........................................................................aauguaaagaaguauggagA...................	30	1	HS2
..........................................................................Uuguaaagaaguauggag....................	1	1	HS2

......cucaaucggugcccucuaguggauc.................................................................................	1	0	HA1
.......ucaaucggugcccucuagugg....................................................................................	1	0	HA1
.......ucaaucggugcccucuaguggauc.................................................................................	1	0	HA1
...........ucggugcccucuaguggauc.................................................................................	3	0	HA1
.............................ucccguacuucuucacaucucca............................................................	1	0	HA1
.............................uUccguacuucuucacaucucca............................................................	2	1	HA1
..............................cccguacuucuucacaucuc..............................................................	2	0	HA1
..............................cccguacuucuucacaucucc.............................................................	43	0	HA1
..............................cccguacuucuucacaucucca............................................................	165	0	HA1
..............................Uccguacuucuucacaucucca............................................................	1	1	HA1
..............................cccguacuucuucacaCcucca............................................................	1	1	HA1
..............................cccguUcuucuucacaucucca............................................................	1	1	HA1
..............................cccguUcuucuucacaucuccau...........................................................	1	1	HA1
..............................cccguacuucuucacaucuccau...........................................................	62	0	HA1
..............................cccguacuucuucacaucuccauC..........................................................	1	1	HA1
..............................cccguacuucuucacaucuccauU..........................................................	22	1	HA1
..............................Uccguacuucuucacaucuccaua..........................................................	1	1	HA1
..............................cccguacuucuucacaucuccaua..........................................................	42	0	HA1
..............................cccguacuucuucacaAcuccaua..........................................................	1	1	HA1
..............................cccguacuucuucacaucuccauaA.........................................................	2	1	HA1
..............................cccguacuucuucacaucuccauau.........................................................	5	0	HA1
..............................cccguacuucuucacaucuccauauu........................................................	1	0	HA1
...............................ccguacuucuucacaucu...............................................................	5	0	HA1
...............................ccguacuucuucacaucuc..............................................................	1	0	HA1
...............................ccguacuucuucacaucuUc.............................................................	1	1	HA1
...............................ccguacuucuucUcaucucc.............................................................	1	1	HA1
...............................ccguacuucuucacaucucc.............................................................	58	0	HA1
...............................ccCuacuucuucacaucucca............................................................	1	1	HA1
...............................ccguacAucuucacaucucca............................................................	1	1	HA1
...............................ccguacuucuucacaucuccU............................................................	1	1	HA1
...............................ccguacuuUuucacaucucca............................................................	1	1	HA1
...............................ccguacuucuucacaucCcca............................................................	5	1	HA1
```

Star Mature

```
acauuucucaaucggugcccucuaguggaucccguacuucuucacaucuccauauuucauuggauauuuauggaauguaaagaaguauggaguuuacuaauguaaaacgagg
...............................ccgGacuucuucacaucucca............................................................    1     1    HA1
...............................ccguacuucuucacauUucca............................................................    2     1    HA1
...............................ccguacuucuucacaucuccG............................................................    1     1    HA1
...............................ccguUcuucuucacaucucca............................................................    1     1    HA1
...............................ccgAacuucuucacaucucca............................................................    1     1    HA1
...............................ccguacuucuucacaucucca............................................................    2004  0    HA1
...............................ccguacuucuCcacaucucca............................................................    2     1    HA1
...............................ccguacuucuucacaGcucca............................................................    1     1    HA1
...............................ccguacuucuucacaucuccC............................................................    1     1    HA1
...............................cUguacuucuucacaucucca............................................................    2     1    HA1
...............................ccguacuucuucacaucAcca............................................................    1     1    HA1
...............................ccAuacuucuucacaucucca............................................................    1     1    HA1
...............................ccguacuucuuUacaucucca............................................................    1     1    HA1
...............................ccguacuucuucUcaucucca............................................................    4     1    HA1
...............................ccguacuCcuucacaucucca............................................................    1     1    HA1
...............................ccguacuucuucacaucGcca............................................................    1     1    HA1
...............................ccguacuuGuucacaucucca............................................................    1     1    HA1
...............................ccguacuucuucacaucuUca............................................................    2     1    HA1
...............................ccguacuuGuucacaucuccau...........................................................    1     1    HA1
...............................ccguacuucuucaUaucuccau...........................................................    1     1    HA1
...............................ccguaGuucuucacaucuccau...........................................................    1     1    HA1
...............................ccguacuucuucUcaucuccau...........................................................    1     1    HA1
...............................ccguacuucuucacaucuGcau...........................................................    1     1    HA1
...............................ccguacuucuucacaucuccaA...........................................................    2     1    HA1
...............................ccguacuucuucacaucuccau...........................................................    412   0    HA1
...............................ccguacuucuucacaCcuccaua..........................................................    3     1    HA1
...............................ccguacuucuucacaucCccaua..........................................................    1     1    HA1
...............................cUguacuucuucacaucuccaua..........................................................    1     1    HA1
...............................ccgGacuucuucacaucuccaua..........................................................    1     1    HA1
...............................ccguacuucAucacaucuccaua..........................................................    1     1    HA1
...............................ccguacAucuucacaucuccaua..........................................................    3     1    HA1
...............................ccguacuuAuucacaucuccaua..........................................................    1     1    HA1
...............................ccguacuucuuGacaucuccaua..........................................................    1     1    HA1
...............................ccguacuucuucacaucucUaua..........................................................    1     1    HA1
...............................Acguacuucuucacaucuccaua..........................................................    2     1    HA1
...............................ccguacuucuuUacaucuccaua..........................................................    1     1    HA1
...............................ccgCacuucuucacaucuccaua..........................................................    1     1    HA1
...............................ccguUcuucuucacaucuccaua..........................................................    1     1    HA1
...............................ccguacuucuucacaucuccauU..........................................................    61    1    HA1
...............................ccguacuCcuucacaucuccaua..........................................................    1     1    HA1
...............................ccguacuucuucacauUuccaua..........................................................    7     1    HA1
...............................ccguacuucCucacaucuccaua..........................................................    1     1    HA1
...............................cGguacuucuucacaucuccaua..........................................................    2     1    HA1
...............................ccguacuuGuucacaucuccaua..........................................................    1     1    HA1
...............................ccguacuucuucacaucuccaAa..........................................................    7     1    HA1
...............................cAguacuucuucacaucuccaua..........................................................    3     1    HA1
...............................ccguacuucuucacaucuUcaua..........................................................    2     1    HA1
...............................ccguacuucuucUcaucuccaua..........................................................    2     1    HA1
...............................ccguacuucuucacaucuccaua..........................................................    2562  0    HA1
...............................ccguacuucuucacaucuccauau.........................................................    4     0    HA1
...............................ccguacuucuucacaucuccauUu.........................................................    6     1    HA1
...............................ccguacuucuucacaucuccauaA.........................................................    21    1    HA1
...............................ccguacuucuucacaucuccauaAu........................................................    1     1    HA1
...............................ccguacuucuucacaucuccauUuu........................................................    1     1    HA1
...............................ccguacuucuucacaucuccauauuu.......................................................    2     0    HA1
...............................ccguacuucuucacaucuccauUuuu.......................................................    1     1    HA1
................................cguacuucuucacaucucca............................................................    3     0    HA1
................................cguacuucuucacaucuccaua..........................................................    25    0    HA1
.................................guacuucuucacaucuccaua..........................................................    1     0    HA1
..................................uacuucuucacaucuccauau.........................................................    1     0    HA1
....................................cuucuucacaucuccauauuuc......................................................    1     0    HA1
....................................................uauuucauuggauauuuaug........................................    2     0    HA1
......................................................uuucauuggauauuuauggaau....................................    1     0    HA1
............................................................uggauauuuauggaauguaaag..............................    1     0    HA1
.................................................................auuuauggaauguaaagaag...........................    1     0    HA1
.................................................................auuuauggaauguaaagaagu..........................    1     0    HA1
.................................................................Uuuuauggaauguaaagaaguauggag....................    1     1    HA1
..................................................................uuuauggaauguaaagaagua.........................    3     0    HA1
..................................................................uuuauggaauguaaagaaguaA........................    2     1    HA1
..................................................................Cuuauggaauguaaagaaguauggag....................    1     1    HA1
```

Star	Mature		
acauuucucaaucggugcccucuaguggaucccguacuucuucacaucuccauauuucauuggauauuuauggaauguaaagaaguauggaguuuacuaauguaaaacgagg			
....uuuauggaauguaaagaaguauggag....................	2	0	HA1
....uuuUuggaauguaaagaaguauggag....................	7	1	HA1
....Auuauggaauguaaagaaguauggag....................	1	1	HA1
....uuauggaauguaaagaagu..........................	1	0	HA1
....uuauggaauguaaagaagua.........................	15	0	HA1
....uuauggaauguaaagaaguau........................	2	0	HA1
....uuauggaauguaaagaaguaA........................	2	1	HA1
....uuauggaauguaaagaaguaug.......................	20	0	HA1
....uuauggaauguaaagaaguaugg......................	1	0	HA1
....uuUuggaauguaaagaaguaugg......................	1	1	HA1
....uuauggaauguaaagaaguaugA......................	1	1	HA1
....uGauggaauguaaagaaguaugga.....................	1	1	HA1
....uuauggaauguaaagaaguaugAa.....................	1	1	HA1
....uuauggaauguaaagaaguaugga.....................	2	0	HA1
....uuUuggaauguaaagaaguaugga.....................	4	1	HA1
....Auauggaauguaaagaaguauggag....................	1	1	HA1
....uuUuggaauguaaagaaguauggag....................	13	1	HA1
....uuUuggaauguaaagaaguauggagu...................	1	1	HA1
....uuauggaauguaaagaaguauggagA...................	1	1	HA1
....uuauggaauguaaagaaguauggaguu..................	1	0	HA1
....uauggaauguaaagaaguau........................	3	0	HA1
....uauggaauguaaagaaguaug.......................	9	0	HA1
....uUuggaauguaaagaaguaug.......................	1	1	HA1
....uUuggaauguaaagaaguaugg......................	19	1	HA1
....uauggaauguaaagaaguaugg......................	6	0	HA1
....uauggaauguaaagaaguaugga.....................	8	0	HA1
....Cauggaauguaaagaaguaugga.....................	2	1	HA1
....Gauggaauguaaagaaguaugga.....................	1	1	HA1
....uUuggaauguaaagaaguaugga.....................	136	1	HA1
....uCuggaauguaaagaaguauggag....................	4	1	HA1
....Gauggaauguaaagaaguauggag....................	1	1	HA1
....uUuggaauguaaagaaguauggag....................	457	1	HA1
....uauggaauguaaagaaguauggaA....................	1	1	HA1
....uauggaauguaaagaaguauggag....................	1	0	HA1
....Aauggaauguaaagaaguauggag....................	4	1	HA1
....uauggaauguaaagaaguauggagA...................	1	1	HA1
....uUuggaauguaaagaaguauggagu...................	26	1	HA1
....Uuggaauguaaagaagua.........................	1	1	HA1
....auggaauguaaagaaguau........................	1	0	HA1
....Cuggaauguaaagaaguau........................	1	1	HA1
....auggaauguaaagaaguauU.......................	1	1	HA1
....Cuggaauguaaagaaguaug.......................	1	1	HA1
....Uuggaauguaaagaaguaug.......................	7	1	HA1
....auggaauguaaagaaguaug.......................	4	0	HA1
....Cuggaauguaaagaaguaugg......................	2	1	HA1
....Uuggaauguaaagaaguaugg......................	63	1	HA1
....auggaauguaaagaaguaAgg......................	1	1	HA1
....auggaauguaaagaaguaugg......................	36	0	HA1
....Guggaauguaaagaaguaugga.....................	4	1	HA1
....aAggaauguaaagaaguaugga.....................	1	1	HA1
....auggaauguaaagaaguaugga.....................	100	0	HA1
....Cuggaauguaaagaaguaugga.....................	18	1	HA1
....auggaaCguaaagaaguaugga.....................	1	1	HA1
....auggaauguaaagaaguauggU.....................	3	1	HA1
....auggaauguaaagaaguauggC.....................	2	1	HA1
....augCaauguaaagaaguaugga.....................	1	1	HA1
....Uuggaauguaaagaaguaugga.....................	501	1	HA1
....auggaauguaaagaaguauggaC....................	1	1	HA1
....Guggaauguaaagaaguauggag....................	10	1	HA1
....Cuggaauguaaagaaguauggag....................	49	1	HA1
....auggaauguaaagaaguauggaA....................	20	1	HA1
....auggaauguaaagaaguauggaU....................	2	1	HA1
....Uuggaauguaaagaaguauggag....................	1507	1	HA1
....auggGauguaaagaaguauggag....................	1	1	HA1
....auggaauguaaagaaguauggag....................	46	0	HA1
....Guggaauguaaagaaguauggagu...................	1	1	HA1
....Uuggaauguaaagaaguauggagu...................	88	1	HA1
....Cuggaauguaaagaaguauggagu...................	2	1	HA1
....auggaauguaaagaaguauggagA...................	19	1	HA1
....auggaauguaaagaaguauggaAu...................	1	1	HA1

Star **Mature**

acauuucucaaucggugcccucuaguggaucccguacuucuucacaucuccauauuucauuggauauuuauggaauguaaagaaguauggaguuuacuaauguaaaacgagg			
....auggaauguaaagaaguauggagu...................	5	0	HA1
....Uuggaauguaaagaaguauggaguu..................	2	1	HA1
....auggaauguaaagaaguauggagAuu.................	1	1	HA1
....Cggaauguaaagaaguau........................	3	1	HA1
....uggaauguaaagaCguau........................	1	1	HA1
....Gggaauguaaagaaguau........................	1	1	HA1
....uggaauguaaagaaguaC........................	2	1	HA1
....uggaaugGaaagaaguau........................	1	1	HA1
....uggaauguaaagaagAau........................	1	1	HA1
....uggaauguaaagaaguaG........................	3	1	HA1
....uggGauguaaagaaguau........................	2	1	HA1
....uggaauguaaagaGguau........................	1	1	HA1
....uggaauguaCagaaguau........................	1	1	HA1
....uggaauguaaagaaguGu........................	3	1	HA1
....uAgaauguaaagaaguau........................	2	1	HA1
....ugAaauguaaagaaguau........................	2	1	HA1
....uggaauCuaaagaaguau........................	2	1	HA1
....ugUaauguaaagaaguau........................	1	1	HA1
....ugCaauguaaagaaguau........................	7	1	HA1
....Aggaauguaaagaaguau........................	3	1	HA1
....uggaauguaaagaaAuau........................	1	1	HA1
....uggaauguaaagaaguau........................	2529	0	HA1
....uggaaGguaaagaaguau........................	1	1	HA1
....uggaauguaaUgaaguau........................	1	1	HA1
....uggaauguaaagaagCau........................	1	1	HA1
....uggaauguaaagaaguaA........................	9	1	HA1
....uggaauguaaagUaguau........................	6	1	HA1
....uggaauguaUagaaguau........................	1	1	HA1
....uggaauAuaaagaaguaug.......................	5	1	HA1
....uggaGuguaaagaaguaug.......................	4	1	HA1
....Gggaauguaaagaaguaug.......................	6	1	HA1
....uggaauguaaagaaguauA.......................	80	1	HA1
....uggGauguaaagaaguaug.......................	2	1	HA1
....uUgaauguaaagaaguaug.......................	2	1	HA1
....ugUaauguaaagaaguaug.......................	8	1	HA1
....uggaauguaaaUaaguaug.......................	1	1	HA1
....uggaauUuaaagaaguaug.......................	1	1	HA1
....uggaauguaaagaagAaug.......................	1	1	HA1
....uggaauguaaagaagCaug.......................	3	1	HA1
....uggaauguaaagaaguaGg.......................	8	1	HA1
....uggaauguaaagUaguaug.......................	19	1	HA1
....uggaauguaaagaagGaug.......................	3	1	HA1
....ugCaauguaaagaaguaug.......................	13	1	HA1
....uggaauguaaagaaguGug.......................	11	1	HA1
....uCgaauguaaagaaguaug.......................	7	1	HA1
....Aggaauguaaagaaguaug.......................	14	1	HA1
....uggaauguaaagaaguauU.......................	27	1	HA1
....uggaauguaaagCaguaug.......................	1	1	HA1
....uggaauguaaCgaaguaug.......................	3	1	HA1
....uggaauCuaaagaaguaug.......................	4	1	HA1
....uggaauguaGagaaguaug.......................	3	1	HA1
....uggaauguaaGgaaguaug.......................	2	1	HA1
....uAgaauguaaagaaguaug.......................	6	1	HA1
....uggUauguaaagaaguaug.......................	2	1	HA1
....uggaauguaaaCaaguaug.......................	5	1	HA1
....uggaaCguaaagaaguaug.......................	2	1	HA1
....uggaauguaaagaGguaug.......................	1	1	HA1
....uggaauguGaagaaguaug.......................	3	1	HA1
....uggaauguaaagaaguaCg.......................	2	1	HA1
....uggaauguaaagGaguaug.......................	1	1	HA1
....uggaauguaUagaaguaug.......................	1	1	HA1
....uggaauguaaagaaguauC.......................	1	1	HA1
....uggaauguaaagaaguaAg.......................	2	1	HA1
....uggaCuguaaagaaguaug.......................	1	1	HA1
....Cggaauguaaagaaguaug.......................	6	1	HA1
....uggaauguUaagaaguaug.......................	1	1	HA1
....uggaauguaaagaaguaug.......................	12511	0	HA1
....uggaauguaaagaaguUug.......................	2	1	HA1
....ugAaauguaaagaaguaug.......................	10	1	HA1
....uggaauguaaagaaCuaug.......................	1	1	HA1

```
                                Star                                    Mature
acauuucucaaucggugcccucuaguggaucccguacuucuucacaucuccauauuucauuggauauuuauggaauguaaagaaguauggaguuuacuaaauguaaaacgagg
......................................................................uggaauguaaagaaguCug........................    8       1    HA1
......................................................................uggaauguCaagaaguaug........................    1       1    HA1
......................................................................uggaauguaCagaaguaug........................    1       1    HA1
......................................................................uggCauguaaagaaguaug........................    6       1    HA1
......................................................................uggaauguUaagaaguaugg.......................    15      1    HA1
......................................................................uggaauguaaagaaguaAgg.......................    522     1    HA1
......................................................................uggaauguGaagaaguaugg.......................    35      1    HA1
......................................................................uggaauguaaagaaCuaugg.......................    12      1    HA1
......................................................................uggaauguaaagaaUuaugg.......................    5       1    HA1
......................................................................uggaauguaaagaaguauCg.......................    5       1    HA1
......................................................................uggaaGguaaagaaguaugg.......................    11      1    HA1
......................................................................uggaauguaaagaaguaCgg.......................    11      1    HA1
......................................................................uggaauCuaaagaaguaugg.......................    42      1    HA1
......................................................................uggaauguaaagaaguaugg.......................    147533  0    HA1
......................................................................uggaauUuaaagaaguaugg.......................    10      1    HA1
......................................................................uggaauguaaagaaguauUg.......................    4       1    HA1
......................................................................uggaaugAaaagaaguaugg.......................    5       1    HA1
......................................................................uggaauguaGagaaguaugg.......................    14      1    HA1
......................................................................Aggaauguaaagaaguaugg.......................    148     1    HA1
......................................................................uggaauguaaGgaaguaugg.......................    13      1    HA1
......................................................................uggaauguaaagaaguaugC.......................    14      1    HA1
......................................................................uggaauguaaagaaguaugA.......................    1444    1    HA1
......................................................................uggaCuguaaagaaguaugg.......................    11      1    HA1
......................................................................uggaaugGaaagaaguaugg.......................    3       1    HA1
......................................................................uggaauguaaagCaguaugg.......................    30      1    HA1
......................................................................uggaaCguaaagaaguaugg.......................    16      1    HA1
......................................................................uAgaauguaaagaaguaugg.......................    58      1    HA1
......................................................................uggaauguaaagUaguaugg.......................    259     1    HA1
......................................................................uggaauguaCagaaguaugg.......................    12      1    HA1
......................................................................ugUaauguaaagaaguaugg.......................    53      1    HA1
......................................................................uggaauguaaaUaaguaugg.......................    17      1    HA1
......................................................................uggaauguaaagaaguauAg.......................    76      1    HA1
......................................................................uggaauguaaagGaguaugg.......................    27      1    HA1
......................................................................uggaGuguaaagaaguaugg.......................    12      1    HA1
......................................................................uggaauguaaagaagGaugg.......................    13      1    HA1
......................................................................uggaauguaaaCaaguaugg.......................    35      1    HA1
......................................................................uggaauguaaagaagAaugg.......................    2       1    HA1
......................................................................uggaauguaUagaaguaugg.......................    9       1    HA1
......................................................................uCgaauguaaagaaguaugg.......................    60      1    HA1
......................................................................uggaauguaaagaaguCugg.......................    6       1    HA1
......................................................................uggaauguaaagaaguUugg.......................    7       1    HA1
......................................................................uggaauguaaagaaguaGgg.......................    29      1    HA1
......................................................................uggUauguaaagaaguaugg.......................    12      1    HA1
......................................................................uggaauguaaagaaAuaugg.......................    7       1    HA1
......................................................................uggaauguCaagaaguaugg.......................    24      1    HA1
......................................................................uggaaAguaaagaaguaugg.......................    1       1    HA1
......................................................................uggaauguaaagaaguGugg.......................    29      1    HA1
......................................................................uggaauguaaagaCguaugg.......................    6       1    HA1
......................................................................Cggaauguaaagaaguaugg.......................    97      1    HA1
......................................................................uggGauguaaagaaguaugg.......................    43      1    HA1
......................................................................ugCaauguaaagaaguaugg.......................    163     1    HA1
......................................................................uggaauguaaCgaaguaugg.......................    10      1    HA1
......................................................................uggaauguaaUgaaguaugg.......................    12      1    HA1
......................................................................uggaauguaaagaUguaugg.......................    2       1    HA1
......................................................................uggCauguaaagaaguaugg.......................    41      1    HA1
......................................................................Gggaauguaaagaaguaugg.......................    116     1    HA1
......................................................................uggaaugCaaagaaguaugg.......................    16      1    HA1
......................................................................uUgaauguaaagaaguaugg.......................    14      1    HA1
......................................................................uggaauguaaaAaaguaugg.......................    3       1    HA1
......................................................................uggaauAuaaagaaguaugg.......................    41      1    HA1
......................................................................uggaUuguaaagaaguaugg.......................    2       1    HA1
......................................................................uggaauguaaagaagCaugg.......................    20      1    HA1
......................................................................uggaauguaaagaGguaugg.......................    16      1    HA1
......................................................................ugAaauguaaagaaguaugg.......................    59      1    HA1
......................................................................uggaauguaaagaaguaugU.......................    126     1    HA1
......................................................................Cggaauguaaagaaguaugga......................    705     1    HA1
......................................................................uggaauguaaagCaguaugga......................    167     1    HA1
......................................................................uggaauguaaagaagAaugga......................    30      1    HA1
......................................................................uggaauguaaagaaguaugUa......................    33      1    HA1
......................................................................uggaUuguaaagaaguaugga......................    9       1    HA1
```

Star Mature

```
acauuucucaaucggugcccucuaguggaucccguacuucuucacaucuccauauuucauuggauauuuauggaauguaaagaaguauggaguuuacuaauguaaaacgagg
......................................................................uggaauUuaaagaaguaugga......................   52        1   HA1
......................................................................uggaauguaaaAaaguaugga......................   31        1   HA1
......................................................................uggaauguaaagaaguaGgga......................   27        1   HA1
......................................................................uggaauguaaagaaguauggG......................   1072      1   HA1
......................................................................uggaaugAaaagaaguaugga......................   67        1   HA1
......................................................................uUgaauguaaagaaguaugga......................   191       1   HA1
......................................................................uggaauguaaagaaguUugga......................   25        1   HA1
......................................................................uggaauguaaagUaguaugga......................   1937      1   HA1
......................................................................uggaauguaaagaaAuaugga......................   91        1   HA1
......................................................................uggaCuguaaagaaguaugga......................   58        1   HA1
......................................................................uggaauguaaagaaguauCga......................   45        1   HA1
......................................................................uAgaauguaaagaaguaugga......................   533       1   HA1
......................................................................uggaauguaaUgaaguaugga......................   83        1   HA1
......................................................................uggCauguaaagaaguaugga......................   267       1   HA1
......................................................................uggaauguaaagaaguauggC......................   368       1   HA1
......................................................................uggaauguUaagaaguaugga......................   46        1   HA1
......................................................................uggaauAuaaagaaguaugga......................   324       1   HA1
......................................................................uggaauguaaagaagCaugga......................   149       1   HA1
......................................................................ugAaauguaaagaaguaugga......................   472       1   HA1
......................................................................uggaauguaaagaaguaugCa......................   32        1   HA1
......................................................................uggaauguaaagaaguaugga......................   1240594   0   HA1
......................................................................Aggaauguaaagaaguaugga......................   1201      1   HA1
......................................................................uggaauguaaagaUguaugga......................   22        1   HA1
......................................................................uggaaugGaaagaaguaugga......................   36        1   HA1
......................................................................uggaGuguaaagaaguaugga......................   74        1   HA1
......................................................................uggaauguaaagaaguGugga......................   129       1   HA1
......................................................................uggaaAguaaagaaguaugga......................   14        1   HA1
......................................................................uggaauguaaagGaguaugga......................   205       1   HA1
......................................................................uggaauguCaagaaguaugga......................   221       1   HA1
......................................................................uggaauguaaagaCguaugga......................   27        1   HA1
......................................................................uggaauguGaagaaguaugga......................   217       1   HA1
......................................................................Gggaauguaaagaaguaugga......................   739       1   HA1
......................................................................uggaauguaaCgaaguaugga......................   61        1   HA1
......................................................................uggUauguaaagaaguaugga......................   101       1   HA1
......................................................................uggaauguaaagaaguCugga......................   91        1   HA1
......................................................................ugUaauguaaagaaguaugga......................   440       1   HA1
......................................................................uggaauguaaagaaguauUga......................   22        1   HA1
......................................................................uggaaGguaaagaaguaugga......................   107       1   HA1
......................................................................uggaauguaaagaaguaugAa......................   199       1   HA1
......................................................................ugCaauguaaagaaguaugga......................   1097      1   HA1
......................................................................uggGauguaaagaaguaugga......................   447       1   HA1
......................................................................uggaaugCaaagaaguaugga......................   112       1   HA1
......................................................................uggaauCuaaagaaguaugga......................   397       1   HA1
......................................................................uggaauguaaagaaUuaugga......................   68        1   HA1
......................................................................uggaauguaaaCaaguaugga......................   284       1   HA1
......................................................................uggaauguaaagaaguauggU......................   768       1   HA1
......................................................................uggaauguaaagaagGaugga......................   70        1   HA1
......................................................................uggaauguaaGgaaguaugga......................   77        1   HA1
......................................................................uggaauguaUagaaguaugga......................   66        1   HA1
......................................................................uggaauguaaagaGguaugga......................   48        1   HA1
......................................................................uggaauguaaagaaguaCgga......................   37        1   HA1
......................................................................uggaauguaaagaaguaAgga......................   10        1   HA1
......................................................................uggaauguaGagaaguaugga......................   86        1   HA1
......................................................................uggaaCguaaagaaguaugga......................   98        1   HA1
......................................................................uggaauguaaagaaguauAga......................   115       1   HA1
......................................................................uggaauguaaagaaCuaugga......................   73        1   HA1
......................................................................uggaauguaaaUaaguaugga......................   132       1   HA1
......................................................................uggaauguaCagaaguaugga......................   118       1   HA1
......................................................................uCgaauguaaagaaguaugga......................   508       1   HA1
......................................................................uggaauguaaagaaguaugUag.....................   137       1   HA1
......................................................................uggaauguaGagaaguauggag.....................   206       1   HA1
......................................................................uggaaugCaaagaaguauggag.....................   232       1   HA1
......................................................................uggaauguGaagaaguauggag.....................   511       1   HA1
......................................................................uggaauUuaaagaaguauggag.....................   132       1   HA1
......................................................................uggaauguaaagCaguauggag.....................   336       1   HA1
......................................................................uggaGuguaaagaaguauggag.....................   191       1   HA1
......................................................................uggaCuguaaagaaguauggag.....................   186       1   HA1
......................................................................uggaauguaaagGaguauggag.....................   334       1   HA1
......................................................................uggaauguaaagaaAuauggag.....................   192       1   HA1
......................................................................uggaauguaaagaaguUuggag.....................   50        1   HA1
```

```
                              Star                                   Mature
acauuucucaaucggugcccucuaguggaucccguacuucuucacaucuccauauuucauuggauauuuauggaauguaaagaaguauggaguuuacuaauguaaaacgagg
......................................................................uggaauguaaagaaguauggCg....................  1561     1  HA1
......................................................................uggaauguaaagaaguaAggag....................  17       1  HA1
......................................................................uggaauguaaagaagCauggag....................  283      1  HA1
......................................................................uggGauguaaagaaguauggag....................  996      1  HA1
......................................................................uggaaugAaaagaaguauggag....................  146      1  HA1
......................................................................uggaaAguaaagaaguauggag....................  12       1  HA1
......................................................................uggaauguaaaCaaguauggag....................  672      1  HA1
......................................................................uggaauguaaagaaguGuggag....................  273      1  HA1
......................................................................uggaauguaaagaaguaugAag....................  131      1  HA1
......................................................................uggCauguaaagaaguauggag....................  641      1  HA1
......................................................................uggaauguaaagaagGauggag....................  111      1  HA1
......................................................................uggaauguaaaUaaguauggag....................  218      1  HA1
......................................................................uggaauguaaagaaguauggaC....................  14205    1  HA1
......................................................................Gggaauguaaagaaguauggag....................  1687     1  HA1
......................................................................uUgaauguaaagaaguauggag....................  356      1  HA1
......................................................................uggaauguaaCgaaguauggag....................  138      1  HA1
......................................................................ugAaauguaaagaaguauggag....................  888      1  HA1
......................................................................uggaauCuaaagaaguauggag....................  762      1  HA1
......................................................................uggaauguaaagaagAauggag....................  21       1  HA1
......................................................................uCgaauguaaagaaguauggag....................  1215     1  HA1
......................................................................uggaauguaaagaaguauCgag....................  101      1  HA1
......................................................................uggaauguaaagaaguauUgag....................  101      1  HA1
......................................................................uggaauguaaagaaguaugCag....................  217      1  HA1
......................................................................uggaauguaaagUaguauggag....................  4248     1  HA1
......................................................................uggaauguaaaAaaguauggag....................  87       1  HA1
......................................................................uggaauguaCagaaguauggag....................  268      1  HA1
......................................................................uggUauguaaagaaguauggag....................  189      1  HA1
......................................................................Cggaauguaaagaaguauggag....................  1657     1  HA1
......................................................................uggaauguaaagaaguauggUg....................  618      1  HA1
......................................................................uggaauguaUagaaguauggag....................  71       1  HA1
......................................................................uggaauguaaagaaguauAgag....................  220      1  HA1
......................................................................Aggaauguaaagaaguauggag....................  2504     1  HA1
......................................................................uggaauguaaagaGguauggag....................  154      1  HA1
......................................................................ugUaauguaaagaaguauggag....................  955      1  HA1
......................................................................uggaauAuaaagaaguauggag....................  667      1  HA1
......................................................................uggaauguaaagaaguauggaA....................  222041   1  HA1
......................................................................uggaauguaaGgaaguauggag....................  205      1  HA1
......................................................................uggaUuguaaagaaguauggag....................  21       1  HA1
......................................................................uggaauguCaagaaguauggag....................  539      1  HA1
......................................................................ugCaauguaaagaaguauggag....................  2423     1  HA1
......................................................................uggaauguaaagaaguaGggag....................  82       1  HA1
......................................................................uggaauguaaagaaCuauggag....................  200      1  HA1
......................................................................uggaauguaaagaCguauggag....................  42       1  HA1
......................................................................uAgaauguaaagaaguauggag....................  1110     1  HA1
......................................................................uggaauguaaUgaaguauggag....................  162      1  HA1
......................................................................uggaauguaaagaaguaCggag....................  88       1  HA1
......................................................................uggaauguaaagaUguauggag....................  64       1  HA1
......................................................................uggaauguaaagaaguCuggag....................  193      1  HA1
......................................................................uggaaGguaaagaaguauggag....................  232      1  HA1
......................................................................uggaauguaaagaaguauggGg....................  2667     1  HA1
......................................................................uggaauguUaagaaguauggag....................  59       1  HA1
......................................................................uggaauguaaagaaUuauggag....................  185      1  HA1
......................................................................uggaauguaaagaaguauggaU....................  21730    1  HA1
......................................................................uggaaugGaaagaaguauggag....................  53       1  HA1
......................................................................uggaauguaaagaaguauggag....................  2600042  0  HA1
......................................................................uggaaCguaaagaaguauggag....................  247      1  HA1
......................................................................uggaauguaaagaaguaCggagu...................  8        1  HA1
......................................................................uggaauguaaagaaguauUgagu...................  8        1  HA1
......................................................................uggaauguaaagaaguauggagG...................  1255     1  HA1
......................................................................uggaauguaaagaaguGuggagu...................  15       1  HA1
......................................................................uggaauguaaagaaguaugCagu...................  9        1  HA1
......................................................................uggaauguaaagUaguauggagu...................  226      1  HA1
......................................................................uggaUuguaaagaaguauggagu...................  1        1  HA1
......................................................................uggaauguaaagaaguauggaUu...................  1046     1  HA1
......................................................................uggaauguaaagCaguauggagu...................  19       1  HA1
......................................................................uggaauguaaagaagCauggagu...................  17       1  HA1
......................................................................uggaauguaaagaaguauggaCu...................  1043     1  HA1
......................................................................uggaauguaaagGaguauggagu...................  22       1  HA1
......................................................................uggaauguGaagaaguauggagu...................  26       1  HA1
......................................................................uggUauguaaagaaguauggagu...................  13       1  HA1
```

Star Mature

```
acauuucucaaucggugcccucuaguggaucccguacuucuucacaucuccauauuucauuggauauuuauggaauguaaagaaguauggaguuuacuaauguaaaacgagg
......................................................................uggaauguaGagaaguauggagu...................   10       1   HA1
......................................................................uggaauguaaaAaaguauggagu...................   3        1   HA1
......................................................................uggaCuguaaagaaguauggagu...................   7        1   HA1
......................................................................uggGauguaaagaaguauggagu...................   55       1   HA1
......................................................................uggaauguaaagaaguauggagu...................   139716   0   HA1
......................................................................uggaauguaaCgaaguauggagu...................   13       1   HA1
......................................................................uAgaauguaaagaaguauggagu...................   63       1   HA1
......................................................................uggaauguaaagaaAuauggagu...................   10       1   HA1
......................................................................uggaaGguaaagaaguauggagu...................   9        1   HA1
......................................................................uggaaCguaaagaaguauggagu...................   6        1   HA1
......................................................................uggaauguUaagaaguauggagu...................   1        1   HA1
......................................................................uggaauguaaagaaguauggGgu...................   115      1   HA1
......................................................................uggaauguaaagaUguauggagu...................   2        1   HA1
......................................................................uggaauguaaagaaguauggagA...................   521865   1   HA1
......................................................................uggaauguaaagaaguauggCgu...................   66       1   HA1
......................................................................uggaauguaaagaagGauggagu...................   5        1   HA1
......................................................................uggaauguaaagaaCuauggagu...................   8        1   HA1
......................................................................uggaauguaaagaaguUuggagu...................   5        1   HA1
......................................................................uggaauguaaagaaguauggagC...................   1225     1   HA1
......................................................................Gggaauguaaagaaguauggagu...................   99       1   HA1
......................................................................uggaauguaCagaaguauggagu...................   8        1   HA1
......................................................................uggaauguCaagaaguauggagu...................   28       1   HA1
......................................................................uggaauguaaagaaguaAggagu...................   1        1   HA1
......................................................................uggaauguaUagaaguauggagu...................   5        1   HA1
......................................................................uggaauguaaagaaguauggUgu...................   28       1   HA1
......................................................................uggCauguaaagaaguauggagu...................   34       1   HA1
......................................................................ugAaauguaaagaaguauggagu...................   36       1   HA1
......................................................................uggaauguaaagaaguauAgagu...................   11       1   HA1
......................................................................uggaauguaaagaagAauggagu...................   4        1   HA1
......................................................................uggaauAuaaagaaguauggagu...................   43       1   HA1
......................................................................uggaauguaaaUaaguauggagu...................   6        1   HA1
......................................................................uggaauguaaagaaUuauggagu...................   20       1   HA1
......................................................................uggaauguaaagaCguauggagu...................   3        1   HA1
......................................................................uggaaugCaaagaaguauggagu...................   17       1   HA1
......................................................................uggaauguaaagaaguCuggagu...................   4        1   HA1
......................................................................uggaauguaaUgaaguauggagu...................   6        1   HA1
......................................................................uUgaauguaaagaaguauggagu...................   28       1   HA1
......................................................................uggaauguaaagaGguauggagu...................   13       1   HA1
......................................................................uggaauUuaaagaaguauggagu...................   8        1   HA1
......................................................................uggaauguaaaCaaguauggagu...................   33       1   HA1
......................................................................Aggaauguaaagaaguauggagu...................   164      1   HA1
......................................................................ugCaauguaaagaaguauggagu...................   126      1   HA1
......................................................................uggaauguaaagaaguaGggagu...................   6        1   HA1
......................................................................Cggaauguaaagaaguauggagu...................   76       1   HA1
......................................................................uggaauguaaagaaguauggaAu...................   2890     1   HA1
......................................................................uggaGuguaaagaaguauggagu...................   13       1   HA1
......................................................................uggaaugGaaagaaguauggagu...................   5        1   HA1
......................................................................uggaauguaaagaaguaugUagu...................   11       1   HA1
......................................................................uCgaauguaaagaaguauggagu...................   65       1   HA1
......................................................................uggaaugAaaagaaguauggagu...................   10       1   HA1
......................................................................uggaauguaaagaaguauCgagu...................   13       1   HA1
......................................................................uggaauguaaGgaaguauggagu...................   12       1   HA1
......................................................................uggaauCuaaagaaguauggagu...................   38       1   HA1
......................................................................ugUaauguaaagaaguauggagu...................   60       1   HA1
......................................................................uggaauguaaagaaguaugAagu...................   11       1   HA1
......................................................................ugCaauguaaagaaguauggaguu..................   3        1   HA1
......................................................................uggaauguaaagaaguauggaAuu..................   79       1   HA1
......................................................................uggCauguaaagaaguauggaguu..................   1        1   HA1
......................................................................uggaauguaaagaaguauggaguA..................   777      1   HA1
......................................................................Aggaauguaaagaaguauggaguu..................   2        1   HA1
......................................................................uggaauguaaagaaguauggGguu..................   3        1   HA1
......................................................................Gggaauguaaagaaguauggaguu..................   3        1   HA1
......................................................................uggaauguaaagUaguauggaguu..................   2        1   HA1
......................................................................uggaauguaaagaaguauUgaguu..................   1        1   HA1
......................................................................uggaaGguaaagaaguauggaguu..................   1        1   HA1
......................................................................uggGauguaaagaaguauggaguu..................   1        1   HA1
......................................................................uggaauguaaagaaguauggagGu..................   16       1   HA1
......................................................................uggaauguGaagaaguauggaguu..................   1        1   HA1
......................................................................uggaauguaaagaaguauggaCuu..................   1        1   HA1
......................................................................uCgaauguaaagaaguauggaguu..................   1        1   HA1
```

Star Mature

```
acauuucucaaucggugcccucuaguggaucccguacuucuucacaucuccauauuucauuggauauuuauggaauguaaagaaguauggaguuuacuaauguaaaacgagg
......................................................................uggaauAuaaagaaguauggaguu..................  2     1  HA1
......................................................................uggaauguaaagaaguauggaguC..................  20    1  HA1
......................................................................uggaauCuaaagaaguauggaguu..................  1     1  HA1
......................................................................uggaauguaaagaaguaugCaguu..................  1     1  HA1
......................................................................uggaauguaaagaaguauggaUuu..................  43    1  HA1
......................................................................uggaauguaaagaGguauggaguu..................  1     1  HA1
......................................................................uggaauguaaagGaguauggaguu..................  1     1  HA1
......................................................................uggaauguaaagaaguauggaguG..................  1     1  HA1
......................................................................uggaauguaaagaaguauggaguu..................  2922  0  HA1
......................................................................uggaauguaaagaaguauggagCu..................  30    1  HA1
......................................................................uggaaugCaaagaaguauggaguu..................  1     1  HA1
......................................................................uggaauguaaagaaguauggagAu..................  2044  1  HA1
......................................................................ugAaauguaaagaaguauggaguu..................  1     1  HA1
......................................................................Cggaauguaaagaaguauggaguu..................  2     1  HA1
......................................................................uggaauguaaagaaguauggaguuG.................  5     1  HA1
......................................................................uggaauguaaagaaguauggaguuA.................  50    1  HA1
......................................................................uggaauguaaagaaguauggaAuuu.................  11    1  HA1
......................................................................uggaauguaaagaaguauggagCuu.................  4     1  HA1
......................................................................uggaauguaaagaaguauggagAuu.................  267   1  HA1
......................................................................uggaauguaaagaaguauggaguAu.................  197   1  HA1
......................................................................uggaauguaaagaaguauggaCuuu.................  4     1  HA1
......................................................................uggaauguaaagaaguauggaguuC.................  1     1  HA1
......................................................................uggaauguaaagaaguauggaguuu.................  436   0  HA1
......................................................................uggaauguaaagaaguaGggaguuu.................  1     1  HA1
......................................................................uggaauguaaagaaguauggaguGu.................  2     1  HA1
......................................................................uggaauguaaagaaguauggaUuuu.................  1     1  HA1
......................................................................uggaauguaaagaaguauggaguuua................  6     0  HA1
......................................................................uggaauguaaagaaguauggaguuAa................  1     1  HA1
......................................................................uggaauguaaagaaguauggaguuuC................  1     1  HA1
......................................................................uggaauguaaagaaguauggaguAua................  1     1  HA1
......................................................................uggaauguaaagaaguauggagAuua................  1     1  HA1
......................................................................uggaauguaaagaaguauggaguuuU................  79    1  HA1
......................................................................uggaauguaaagaaguauggaguuuaU...............  1     1  HA1
.......................................................................ggaauguaaagaaguaug.......................  18    0  HA1
.......................................................................Ugaauguaaagaaguaug.......................  2     1  HA1
.......................................................................Ugaauguaaagaaguaugg......................  12    1  HA1
.......................................................................ggaauguaaagaaguaugg......................  165   0  HA1
.......................................................................ggaauguaaagaaguaugA......................  2     1  HA1
.......................................................................ggaauguaaagaaguaAgg......................  2     1  HA1
.......................................................................ggaauguaaaAaaguaugg......................  1     1  HA1
.......................................................................ggaauguaaagaaguaugU......................  1     1  HA1
.......................................................................Cgaauguaaagaaguaugg......................  3     1  HA1
.......................................................................ggaauguaaagaaguauggG.....................  16    1  HA1
.......................................................................ggaauguaaagaaguauggU.....................  1     1  HA1
.......................................................................ggaauguaaagaaguaGgga.....................  1     1  HA1
.......................................................................ggaaugGaaagaaguaugga.....................  1     1  HA1
.......................................................................gCaauguaaagaaguaugga.....................  3     1  HA1
.......................................................................ggaauguaGagaaguaugga.....................  2     1  HA1
.......................................................................gUaauguaaagaaguaugga.....................  5     1  HA1
.......................................................................ggaauguaaaAaaguaugga.....................  1     1  HA1
.......................................................................ggaauguaaagaaguaugga.....................  1493  0  HA1
.......................................................................ggaauguaaagaagGaugga.....................  1     1  HA1
.......................................................................Agaauguaaagaaguaugga.....................  2     1  HA1
.......................................................................ggGauguaaagaaguaugga.....................  3     1  HA1
.......................................................................ggaauguaaagaaAuaugga.....................  1     1  HA1
.......................................................................ggaaAguaaagaaguaugga.....................  1     1  HA1
.......................................................................Cgaauguaaagaaguaugga.....................  3     1  HA1
.......................................................................Ugaauguaaagaaguaugga.....................  85    1  HA1
.......................................................................gAaauguaaagaaguaugga.....................  2     1  HA1
.......................................................................ggaauguaaagaUguaugga.....................  6     1  HA1
.......................................................................ggaauguaaagaaguauggCg....................  1     1  HA1
.......................................................................ggaauguaaagaaguauggUg....................  1     1  HA1
.......................................................................ggaauguaaagaaguCuggag....................  1     1  HA1
.......................................................................ggaauguaaagCaguauggag....................  1     1  HA1
.......................................................................ggaauCuaaagaaguauggag....................  1     1  HA1
.......................................................................ggaaCguaaagaaguauggag....................  1     1  HA1
.......................................................................ggaGuguaaagaaguauggag....................  3     1  HA1
.......................................................................ggaauguaaagaaguauggaC....................  16    1  HA1
.......................................................................ggaauguaaagaaguaugCag....................  1     1  HA1
.......................................................................Cgaauguaaagaaguauggag....................  18    1  HA1
```

Star **Mature**

```
acauuucucaaucggugcccucuaguggaucccguacuucuucacaucuccauauuucauuggauauuuauggaauguaaagaaguauggaguuuacuaauguaaaacgagg
.......................................................................ggaauguaaagaaUuauggag....................    1      1      HA1
.......................................................................ggaauguaaaUaaguauggag....................    1      1      HA1
.......................................................................ggaauguaaagaaguauUgag....................    1      1      HA1
.......................................................................ggaaugAaaagaaguauggag....................    2      1      HA1
.......................................................................gUaauguaaagaaguauggag....................    4      1      HA1
.......................................................................ggaauguGaagaaguauggag....................    1      1      HA1
.......................................................................ggaauguaaCgaaguauggag....................    1      1      HA1
.......................................................................ggaauguaaagaaguauggag....................    3040   0      HA1
.......................................................................gAaauguaaagaaguauggag....................    7      1      HA1
.......................................................................ggGauguaaagaaguauggag....................    4      1      HA1
.......................................................................Agaauguaaagaaguauggag....................    1      1      HA1
.......................................................................ggaauguaCagaaguauggag....................    3      1      HA1
.......................................................................ggaauguaaagaaAuauggag....................    2      1      HA1
.......................................................................gCaauguaaagaaguauggag....................    9      1      HA1
.......................................................................ggUauguaaagaaguauggag....................    1      1      HA1
.......................................................................ggaauguaaagaGguauggag....................    1      1      HA1
.......................................................................ggaauguaaagaagGauggag....................    1      1      HA1
.......................................................................Ugaauguaaagaaguauggag....................    209    1      HA1
.......................................................................ggaauguaaagaaguauggaA....................    227    1      HA1
.......................................................................ggaauguaaagaaguauggaU....................    30     1      HA1
.......................................................................ggaauguaaaAaaguauggag....................    1      1      HA1
.......................................................................ggaauguaaagaaguauggGg....................    16     1      HA1
.......................................................................ggaauguaaagaUguauggag....................    2      1      HA1
.......................................................................ggaauguaaagaaguUuggag....................    1      1      HA1
.......................................................................ggaauguaaagaaguauggagu...................    222    0      HA1
.......................................................................ggaauguaaagaaguauggagC...................    11     1      HA1
.......................................................................ggaauguaaagaaguauggaCu...................    1      1      HA1
.......................................................................ggaauguaaagaaguauggagG...................    5      1      HA1
.......................................................................gUaauguaaagaaguauggagu...................    1      1      HA1
.......................................................................ggaauguaaagaaguauggagA...................    603    1      HA1
.......................................................................ggaauguaaagaaguauggaAu...................    2      1      HA1
.......................................................................Ugaauguaaagaaguauggagu...................    24     1      HA1
.......................................................................ggaauguaaagaaguauggGgu...................    1      1      HA1
.......................................................................ggaauguaaagaaguauggaUu...................    5      1      HA1
.......................................................................gCaauguaaagaaguauggagu...................    3      1      HA1
.......................................................................ggaauguaaagaaguauggaguu..................    6      0      HA1
.......................................................................ggaauguaaagaaguauggaguA..................    1      1      HA1
.......................................................................ggaauguaaagaaguauggaAuu..................    2      1      HA1
.......................................................................ggaauguaaagaaguauggaguuu.................    1      0      HA1
........................................................................gaauguaaagaaguaAgg......................    1      1      HA1
........................................................................gaauguaaagaaCuaugg......................    1      1      HA1
........................................................................gaauguaaagaaguaugA......................    1      1      HA1
........................................................................gaauguaaagaaguaugg......................    86     0      HA1
........................................................................gaaugGaaagaaguaugga.....................    1      1      HA1
........................................................................gaauguaaagaaguauggU.....................    3      1      HA1
........................................................................gaauguaaagCaguaugga.....................    2      1      HA1
........................................................................Uaauguaaagaaguaugga.....................    10     1      HA1
........................................................................gaauguaaagaaguauggG.....................    4      1      HA1
........................................................................gaauguaaaCaaguaugga.....................    1      1      HA1
........................................................................gaGuguaaagaaguaugga.....................    1      1      HA1
........................................................................gaaAguaaagaaguaugga.....................    1      1      HA1
........................................................................gaauguaaagaGguaugga.....................    1      1      HA1
........................................................................Caauguaaagaaguaugga.....................    3      1      HA1
........................................................................gaaugCaaagaaguaugga.....................    1      1      HA1
........................................................................Aaauguaaagaaguaugga.....................    24     1      HA1
........................................................................gCauguaaagaaguaugga.....................    1      1      HA1
........................................................................gaauguaaagaaguaugga.....................    402    0      HA1
........................................................................gaauguaaagaaguauggaU....................    35     1      HA1
........................................................................gaauguaaagaaguauggag....................    2009   0      HA1
........................................................................gaauguaaagGaguauggag....................    2      1      HA1
........................................................................gaauguaaagaCguauggag....................    1      1      HA1
........................................................................gaauguaaCgaaguauggag....................    2      1      HA1
........................................................................gCauguaaagaaguauggag....................    3      1      HA1
........................................................................gaauguaaagaaguauggCg....................    3      1      HA1
........................................................................Uaauguaaagaaguauggag....................    31     1      HA1
........................................................................gaauguaaagaaguauggGg....................    1      1      HA1
........................................................................gaauguaaagaGguauggag....................    1      1      HA1
........................................................................gaauguaaaAaaguauggag....................    2      1      HA1
........................................................................gGauguaaagaaguauggag....................    2      1      HA1
........................................................................Caauguaaagaaguauggag....................    4      1      HA1
```

Star **Mature**

```
acauuucucaaucggugcccucuaguggaucccguacuucuucacaucuccauauuucauuggauauuuauggaauguaaagaaguauggaguuuacuaauguaaaacgagg
........................................................................gaauguaaagaaguauggaA.................... 76 1 HA1
........................................................................gaauguaaagaaguauAgag.................... 1 1 HA1
........................................................................gaauguUaagaaguauggag.................... 1 1 HA1
........................................................................gaauguaaagaaguauggaC.................... 97 1 HA1
........................................................................gaaugAaaagaaguauggag.................... 1 1 HA1
........................................................................Aaauguaaagaaguauggag.................... 32 1 HA1
........................................................................gaUuguaaagaaguauggag.................... 1 1 HA1
........................................................................gaauguaaagaaguauggUg.................... 2 1 HA1
........................................................................gaauguaaGgaaguauggag.................... 1 1 HA1
........................................................................gaauguaaagaaUuauggagu................... 4 1 HA1
........................................................................gaauguaaagaGguauggagu................... 2 1 HA1
........................................................................Uaauguaaagaaguauggagu................... 1 1 HA1
........................................................................gaauguaaagaaguauggagu................... 1894 0 HA1
........................................................................gaauguaaagaaguauggagA................... 3256 1 HA1
........................................................................gaauguaaagaaguauggCgu................... 1 1 HA1
........................................................................gaaugCaaagaaguauggagu................... 1 1 HA1
........................................................................gaauguaaagaaguauggaAu................... 3 1 HA1
........................................................................gaauguaaagaaguauggagG................... 7 1 HA1
........................................................................gaauguaaagaaguauggagC................... 49 1 HA1
........................................................................gaauguaaagaaguauggUgu................... 1 1 HA1
........................................................................Caauguaaagaaguauggagu................... 1 1 HA1
........................................................................gaauguaaagaaguauggaCu................... 10 1 HA1
........................................................................gCauguaaagaaguauggagu................... 3 1 HA1
........................................................................gaauguaCagaaguauggagu................... 1 1 HA1
........................................................................Aaauguaaagaaguauggagu................... 3 1 HA1
........................................................................gaauguCaagaaguauggagu................... 2 1 HA1
........................................................................gaauguaaagCaguauggagu................... 2 1 HA1
........................................................................gGauguaaagaaguauggagu................... 1 1 HA1
........................................................................gaauguaaagaaguauggGgu................... 2 1 HA1
........................................................................gaauguaaagaaguauggaUu................... 59 1 HA1
........................................................................gaauguaaagaaguauggaguA.................. 2449 1 HA1
........................................................................gaauguaaagaaguauggaguu.................. 1213 0 HA1
........................................................................gaauguaaagaaguauggaUuu.................. 13 1 HA1
........................................................................gUauguaaagaaguauggaguu.................. 1 1 HA1
........................................................................gaauguaaaCaaguauggaguu.................. 1 1 HA1
........................................................................gaauguaaagaaUuauggaguu.................. 1 1 HA1
........................................................................gaauguCaagaaguauggaguu.................. 1 1 HA1
........................................................................gaauguaaagCaguauggaguu.................. 1 1 HA1
........................................................................gaauguaaagaaguauggaCuu.................. 3 1 HA1
........................................................................gaauguaaCgaaguauggaguu.................. 1 1 HA1
........................................................................gaauguaaagaaguauggaguG.................. 3 1 HA1
........................................................................gaauguaaUgaaguauggaguu.................. 1 1 HA1
........................................................................gaauguaaagaaguaugUaguu.................. 1 1 HA1
........................................................................gaauguaaagaaguauggGguu.................. 1 1 HA1
........................................................................gaauguaaagaaguauggagAu.................. 103 1 HA1
........................................................................gaaAguaaagaaguauggaguu.................. 1 1 HA1
........................................................................gaauguaaagaaCuauggaguu.................. 1 1 HA1
........................................................................gaauguaaagaaguauggaguC.................. 3 1 HA1
........................................................................gaauguaaagaaguauggagCu.................. 13 1 HA1
........................................................................gaauguaaagaaguauggaguAu................. 6 1 HA1
........................................................................gaauguaaagaaguauggaguuG................. 1 1 HA1
........................................................................gaauguaaagaaguauggaguuA................. 51 1 HA1
........................................................................gGauguaaagaaguauggaguuu................. 1 1 HA1
........................................................................gUauguaaagaaguauggaguuu................. 1 1 HA1
........................................................................gaauguaaagaaguauggaguCu................. 1 1 HA1
........................................................................gaauguaaagaaguauggaguuu................. 55 0 HA1
........................................................................gaauguaaagaaguauggagAuu................. 16 1 HA1
........................................................................gaauguaaagaaguauggaguuAa................ 1 1 HA1
........................................................................gaauguaaagaaguauggaguuuU................ 1 1 HA1
........................................................................gaauguaaagaaguauggaguuuaG............... 1 1 HA1
.........................................................................aauguaaagaaguaugga..................... 87 0 HA1
.........................................................................Uauguaaagaaguauggag.................... 4 1 HA1
.........................................................................aauguaaagaaguauggGg.................... 1 1 HA1
.........................................................................aauguaaagaaguauggaC.................... 2 1 HA1
.........................................................................Gauguaaagaaguauggag.................... 1 1 HA1
.........................................................................aauguaaagaaguauggaA.................... 15 1 HA1
.........................................................................aauguaaagaaguauggag.................... 266 0 HA1
.........................................................................aauguaaagaaguauggagu................... 22 0 HA1
.........................................................................aauguaaagaaguauggagA................... 54 1 HA1
.........................................................................aauguaaagaaguauggagCu.................. 1 1 HA1
```

```
                           Star                                Mature
acauuucucaaucggugcccucuaguggaucccguacuucuucacaucuccauauuucauuggauauuuauggaauguaaagaaguauggaguuuacuaauguaaaacgagg
.........................................................................aauguaaagaaguauggaguA..................  2     1  HA1
.........................................................................aauguaaagaaguauggaguu..................  4     0  HA1
..........................................................................auguaaagaaguauggaU....................  3     1  HA1
..........................................................................auguaaagaaguauggGg....................  3     1  HA1
..........................................................................auguaaagaaguauggaC....................  1     1  HA1
..........................................................................Uuguaaagaaguauggag....................  2     1  HA1
..........................................................................auguaaagaaguauggag....................  373   0  HA1
..........................................................................auguaaagaaguUuggag....................  1     1  HA1
..........................................................................auguaaagaagCauggag....................  1     1  HA1
..........................................................................Cuguaaagaaguauggag....................  2     1  HA1
..........................................................................auguaaagaaguauggagu...................  22    0  HA1
..........................................................................auguaaagaaguauggagA...................  98    1  HA1
..........................................................................auguaaagaaguauggaAu...................  1     1  HA1
..........................................................................auguaaagaaguauggagAu..................  2     1  HA1
..........................................................................auguaaagaaguauggaguu..................  1     0  HA1
...........................................................................uguaaagaaguauggagA...................  50    1  HA1
...........................................................................uguaaagaaguauggagu...................  16    0  HA1
...........................................................................Aguaaagaaguauggagu...................  1     1  HA1
...........................................................................ugGaaagaaguauggagu...................  1     1  HA1
...........................................................................uguaaaCaaguauggagu...................  1     1  HA1
...........................................................................uguaaagaaguauggaguu..................  1     0  HA1
............................................................................guaaagaaguauggaguu..................  1     0  HA1
............................................................................guaaagaaguauggaguA..................  2     1  HA1

..............................cccguacuucuucacaucuccaua..........................................................  2     0  HS1
...............................ccguacuucuucacaucucca............................................................  20    0  HS1
...............................ccguacuucuucacaucuccau...........................................................  11    0  HS1
...............................ccguacuucuCcacaucuccaua..........................................................  1     1  HS1
...............................ccguacuucuucacaucCccaua..........................................................  1     1  HS1
...............................ccguacuucuucacaucuccaua..........................................................  247   0  HS1
...............................ccguacuucuucacaucuccauaA.........................................................  2     1  HS1
................................cguacuucuucacaucuccaua..........................................................  11    0  HS1
...........................................................uuggauauuuauggaaugua.................................  1     0  HS1
...........................................................uuggauauuuauggaauguaaa...............................  1     0  HS1
.................................................................auuuauggaauguaaagaagu..........................  2     0  HS1
.................................................................auuuauggaauguaaagaagua.........................  5     0  HS1
..................................................................uuuauggaauguaaagaaguaug.......................  6     0  HS1
..................................................................uuuauggaauguaaagaaguaugA......................  1     1  HS1
...................................................................uuauggaauguaaagaaguaug.......................  125   0  HS1
...................................................................uuauggaauguaaagaaguauC.......................  2     1  HS1
...................................................................uuauggaauguaaagaaguaugg......................  6     0  HS1
...................................................................uuauggaauguaaagaaguaugga.....................  2     0  HS1
...................................................................uuauggaauguaaagaaguauggag....................  5     0  HS1
...................................................................uuauggaauguaaagaaguauggagA...................  1     1  HS1
...................................................................uuauggaauguaaagaaguauggagu...................  2     0  HS1
....................................................................uauggaauguaaagaaguaug.......................  26    0  HS1
....................................................................uauggaauguaaagaaguaugg......................  7     0  HS1
....................................................................uauggaauguaaagaaguaugga.....................  4     0  HS1
....................................................................uauggaauguaaagaaguauggag....................  10    0  HS1
....................................................................Aauggaauguaaagaaguauggag....................  25    1  HS1
....................................................................Cauggaauguaaagaaguauggag....................  3     1  HS1
....................................................................uCuggaauguaaagaaguauggag....................  17    1  HS1
....................................................................Aauggaauguaaagaaguauggagu...................  7     1  HS1
....................................................................uCuggaauguaaagaaguauggagu...................  3     1  HS1
.....................................................................auggaauguaaagaaguaug.......................  3     0  HS1
.....................................................................Cuggaauguaaagaaguaug.......................  2     1  HS1
.....................................................................auggaauguaaagaaguaugg......................  7     0  HS1
.....................................................................Cuggaauguaaagaaguaugga.....................  106   1  HS1
.....................................................................auggaauguaaagaaguaugga.....................  87    0  HS1
.....................................................................auggaauguaaagaaguauggG.....................  2     1  HS1
.....................................................................Cuggaauguaaagaaguauggag....................  1268  1  HS1
.....................................................................auggaauguaaagaaguauggag....................  111   0  HS1
.....................................................................Uuggaauguaaagaaguauggag....................  15    1  HS1
.....................................................................auggaauguaaagaaguauggaA....................  4     1  HS1
.....................................................................auggaauguaaagaaguauggagu...................  57    0  HS1
.....................................................................Cuggaauguaaagaaguauggagu...................  148   1  HS1
.....................................................................Uuggaauguaaagaaguauggagu...................  7     1  HS1
.....................................................................auggaauguaaagaaguauggagC...................  1     1  HS1
.....................................................................auggaauguaaagaaguauggagA...................  16    1  HS1
.....................................................................Cuggaauguaaagaaguauggaguu..................  3     1  HS1
```

Star **Mature**

```
acauuucucaaucggugcccucuaguggaucccguacuucuucacaucuccauauuucauuggauauuuauggaauguaaagaaguauggaguuuacuaauguaaaacgagg
......................................................................uggaauguaaagaagGau........................   1      1    HS1
......................................................................uggaaCguaaagaaguau........................   1      1    HS1
......................................................................Cggaauguaaagaaguau........................   1      1    HS1
......................................................................uggaauguaaagaaguau........................   329    0    HS1
......................................................................uggaauguaaagaaguGu........................   1      1    HS1
......................................................................uggaauguaaagaaguaA........................   5      1    HS1
......................................................................uggGauguaaagaaguau........................   1      1    HS1
......................................................................Gggaauguaaagaaguau........................   1      1    HS1
......................................................................uggaauguaaagaagCau........................   2      1    HS1
......................................................................uggaauguaaagaaguaG........................   2      1    HS1
......................................................................uAgaauguaaagaaguaug.......................   1      1    HS1
......................................................................uggaauguaaagaaguauU.......................   4      1    HS1
......................................................................Gggaauguaaagaaguaug.......................   7      1    HS1
......................................................................uggaauguaaagaaguaCg.......................   2      1    HS1
......................................................................uggaauguaaGgaaguaug.......................   2      1    HS1
......................................................................uggaauguaaagaaguGug.......................   1      1    HS1
......................................................................uggaaGguaaagaaguaug.......................   2      1    HS1
......................................................................uggaauguaaagaaguauA.......................   1      1    HS1
......................................................................Aggaauguaaagaaguaug.......................   5      1    HS1
......................................................................uggGauguaaagaaguaug.......................   6      1    HS1
......................................................................uggaauguaaaUaaguaug.......................   1      1    HS1
......................................................................ugUaauguaaagaaguaug.......................   2      1    HS1
......................................................................uggaGuguaaagaaguaug.......................   2      1    HS1
......................................................................uUgaauguaaagaaguaug.......................   6      1    HS1
......................................................................uggaauAuaaagaaguaug.......................   1      1    HS1
......................................................................uggaauguaaagaaguaug.......................   6964   0    HS1
......................................................................Cggaauguaaagaaguaug.......................   4      1    HS1
......................................................................uggaaugCaaagaaguaug.......................   1      1    HS1
......................................................................uggaauguaaagaGguaug.......................   1      1    HS1
......................................................................ugAaauguaaagaaguaug.......................   1      1    HS1
......................................................................uggaauguaaagUaguaug.......................   1      1    HS1
......................................................................uggaaCguaaagaaguaug.......................   5      1    HS1
......................................................................uggaauguaaagaaguaGg.......................   1      1    HS1
......................................................................uggaauguGaagaaguaug.......................   3      1    HS1
......................................................................uggaGuguaaagaaguaugg......................   1      1    HS1
......................................................................Cggaauguaaagaaguaugg......................   11     1    HS1
......................................................................uggUauguaaagaaguaugg......................   1      1    HS1
......................................................................uggaauguaGagaaguaugg......................   2      1    HS1
......................................................................uggaaugAaaagaaguaugg......................   2      1    HS1
......................................................................Aggaauguaaagaaguaugg......................   4      1    HS1
......................................................................uggaaCguaaagaaguaugg......................   2      1    HS1
......................................................................uggaauguaaUgaaguaugg......................   1      1    HS1
......................................................................uggaauguaaagaaguaugC......................   7      1    HS1
......................................................................uggGauguaaagaaguaugg......................   2      1    HS1
......................................................................uggaauguaaagaaguaugU......................   46     1    HS1
......................................................................uggaauguaaagaaguauAg......................   3      1    HS1
......................................................................uggaauguaaagaGguaugg......................   3      1    HS1
......................................................................uggaauguaaGgaaguaugg......................   3      1    HS1
......................................................................uggaUuguaaagaaguaugg......................   2      1    HS1
......................................................................uggaauguaaagaaguaCgg......................   1      1    HS1
......................................................................ugUaauguaaagaaguaugg......................   1      1    HS1
......................................................................uggaauguaaagaaguGugg......................   2      1    HS1
......................................................................uggaauUuaaagaaguaugg......................   3      1    HS1
......................................................................uggaauguaaagaUguaugg......................   1      1    HS1
......................................................................uggaaugGaaagaaguaugg......................   2      1    HS1
......................................................................uggaauguaaagaagCaugg......................   1      1    HS1
......................................................................Gggaauguaaagaaguaugg......................   18     1    HS1
......................................................................uggaauguaaagGaguaugg......................   5      1    HS1
......................................................................uggaauguaaagaaguaugA......................   50     1    HS1
......................................................................uggaaGguaaagaaguaugg......................   1      1    HS1
......................................................................uAgaauguaaagaaguaugg......................   1      1    HS1
......................................................................uggaauguaaagaaguaugg......................   9587   0    HS1
......................................................................uUgaauguaaagaaguaugg......................   1      1    HS1
......................................................................uggaauguaaaUaaguaugg......................   1      1    HS1
......................................................................uggaauguaaagaaguaAgg......................   1      1    HS1
......................................................................uggaauguaaagaaguGugga.....................   61     1    HS1
......................................................................uggaaugCaaagaaguaugga.....................   56     1    HS1
......................................................................uggaauguCaagaaguaugga.....................   1      1    HS1
......................................................................Gggaauguaaagaaguaugga.....................   232    1    HS1
......................................................................uggaauguGaagaaguaugga.....................   51     1    HS1
```

```
                                Star                                Mature
acauuucucaaucggugcccucuaguggaucccguacuucuucacaucuccauauuucauuggauauuuauggaauguaaagaaguauggaguuuacuaauguaaaacgagg
.....................................................................uggaauguaaagaaguUugga.....................  1  1  HS1
.....................................................................uggaaGguaaagaaguaugga.....................  60  1  HS1
.....................................................................uggaaugGaaagaaguaugga.....................  44  1  HS1
.....................................................................uggaauguaaagaaguaGgga.....................  6  1  HS1
.....................................................................uggCauguaaagaaguaugga.....................  10  1  HS1
.....................................................................uggaauUuaaagaaguaugga.....................  7  1  HS1
.....................................................................uggaauguaaagaagAaugga.....................  18  1  HS1
.....................................................................uggaauguaaagaUguaugga.....................  2  1  HS1
.....................................................................uggaauguaaagaGguaugga.....................  71  1  HS1
.....................................................................uCgaauguaaagaaguaugga.....................  6  1  HS1
.....................................................................uggaauguaGagaaguaugga.....................  67  1  HS1
.....................................................................uggaauguaaagaaguCugga.....................  2  1  HS1
.....................................................................uggaauguaaagaCguaugga.....................  2  1  HS1
.....................................................................uAgaauguaaagaaguaugga.....................  29  1  HS1
.....................................................................ugUaauguaaagaaguaugga.....................  9  1  HS1
.....................................................................Aggaauguaaagaaguaugga.....................  130  1  HS1
.....................................................................uggaauguaaagaaguaAgga.....................  10  1  HS1
.....................................................................uggaauguaaagaagGaugga.....................  23  1  HS1
.....................................................................uggaauguaaagCaguaugga.....................  6  1  HS1
.....................................................................uggaauguaaagaaguauggC.....................  21  1  HS1
.....................................................................uggaauguaaagaaguauAga.....................  31  1  HS1
.....................................................................uggaauguaaagUaguaugga.....................  7  1  HS1
.....................................................................Cggaauguaaagaaguaugga.....................  199  1  HS1
.....................................................................uggaCuguaaagaaguaugga.....................  2  1  HS1
.....................................................................uggaauguaaagaaUuaugga.....................  3  1  HS1
.....................................................................ugAaauguaaagaaguaugga.....................  11  1  HS1
.....................................................................uggaauguaCagaaguaugga.....................  3  1  HS1
.....................................................................uggaauguaaagaaAuaugga.....................  3  1  HS1
.....................................................................uggaauguaaagGaguaugga.....................  52  1  HS1
.....................................................................uggGauguaaagaaguaugga.....................  80  1  HS1
.....................................................................uggaauguaaagaaguauggG.....................  121  1  HS1
.....................................................................uggaaugAaaagaaguaugga.....................  20  1  HS1
.....................................................................uggaauguaaagaaguaugUa.....................  29  1  HS1
.....................................................................uggaauguaaagaaguauggU.....................  327  1  HS1
.....................................................................uggaauguaaGgaaguaugga.....................  49  1  HS1
.....................................................................uUgaauguaaagaaguaugga.....................  12  1  HS1
.....................................................................uggaaCguaaagaaguaugga.....................  45  1  HS1
.....................................................................uggaauguaaCgaaguaugga.....................  1  1  HS1
.....................................................................uggaGuguaaagaaguaugga.....................  57  1  HS1
.....................................................................uggaauguaaUgaaguaugga.....................  4  1  HS1
.....................................................................uggaauguaaagaaguaugga.....................  254946  0  HS1
.....................................................................uggaauguaaaAaaguaugga.....................  5  1  HS1
.....................................................................uggaauguaaagaaguaCgga.....................  37  1  HS1
.....................................................................uggaauguaaaUaaguaugga.....................  3  1  HS1
.....................................................................uggaauguaaagaaguaugCa.....................  2  1  HS1
.....................................................................uggUauguaaagaaguaugga.....................  3  1  HS1
.....................................................................uggaauguaaagaaguauCga.....................  8  1  HS1
.....................................................................uggaUuguaaagaaguaugga.....................  10  1  HS1
.....................................................................uggaauguaaagaagCaugga.....................  44  1  HS1
.....................................................................uggaauCuaaagaaguaugga.....................  4  1  HS1
.....................................................................uggaauguaUagaaguaugga.....................  7  1  HS1
.....................................................................ugCaauguaaagaaguaugga.....................  3  1  HS1
.....................................................................uggaauAuaaagaaguaugga.....................  20  1  HS1
.....................................................................uggaaAguaaagaaguaugga.....................  17  1  HS1
.....................................................................uggaauguaaagaaguauUga.....................  5  1  HS1
.....................................................................uggaauguaaagaaguaugAa.....................  16  1  HS1
.....................................................................uggaCuguaaagaaguauggag....................  17  1  HS1
.....................................................................uggaauguaaagaaCuauggag....................  42  1  HS1
.....................................................................uggaauguaaagaCguauggag....................  22  1  HS1
.....................................................................uAgaauguaaagaaguauggag....................  396  1  HS1
.....................................................................uggaauguaaagaaguaGggag....................  402  1  HS1
.....................................................................uggaauguaaagaaguauggaA....................  6133  1  HS1
.....................................................................uggaUuguaaagaaguauggag....................  72  1  HS1
.....................................................................uggaauguaaagaaguauggaU....................  3074  1  HS1
.....................................................................uggaauguaaaAaaguauggag....................  102  1  HS1
.....................................................................uggaauAuaaagaaguauggag....................  311  1  HS1
.....................................................................uggaauguaaaCaaguauggag....................  19  1  HS1
.....................................................................uggaauguaaagCaguauggag....................  70  1  HS1
.....................................................................uggaauguaaagaaguauAgag....................  338  1  HS1
.....................................................................uggaauguaaagaagCauggag....................  716  1  HS1
```

```
                               Star                                   Mature
acauuucucaaucggugcccucuaguggaucccguacuucuucacaucuccauauuucauuggauauuuauggaauguaaagaaguauggaguuuacuaauguaaaacgagg
......................................................................uggaauguaaagaaguaCggag....................  536      1  HS1
......................................................................uggaauguGaagaaguauggag....................  622      1  HS1
......................................................................uggaaugCaaagaaguauggag....................  593      1  HS1
......................................................................uggaauguCaagaaguauggag....................  9        1  HS1
......................................................................uggaauguaaagGaguauggag....................  707      1  HS1
......................................................................uggaaGguaaagaaguauggag....................  676      1  HS1
......................................................................uggaauguaaagaaguUuggag....................  47       1  HS1
......................................................................uggaaCguaaagaaguauggag....................  548      1  HS1
......................................................................uggaaAguaaagaaguauggag....................  182      1  HS1
......................................................................uggaauguaaagaaguGuggag....................  604      1  HS1
......................................................................uCgaauguaaagaaguauggag....................  49       1  HS1
......................................................................uggaauguaaagaagGauggag....................  568      1  HS1
......................................................................ugUaauguaaagaaguauggag....................  101      1  HS1
......................................................................uggaauguaaagaaguaAggag....................  192      1  HS1
......................................................................uggaauguaaagaaguaugUag....................  120      1  HS1
......................................................................ugAaauguaaagaaguauggag....................  267      1  HS1
......................................................................uggaauguaaagaaguauggUg....................  132      1  HS1
......................................................................uggaauguaaagaGguauggag....................  856      1  HS1
......................................................................uggaauguaaagaaguCuggag....................  19       1  HS1
......................................................................uggaGuguaaagaaguauggag....................  749      1  HS1
......................................................................uggaauCuaaagaaguauggag....................  32       1  HS1
......................................................................uggaauguaaagaaAuauggag....................  140      1  HS1
......................................................................uggaauguaaagUaguauggag....................  113      1  HS1
......................................................................uggaauguaaaUaaguauggag....................  45       1  HS1
......................................................................uggaauguaaagaaguauggCg....................  151      1  HS1
......................................................................uggaauguaaagaaguauggaC....................  1143     1  HS1
......................................................................uggaauguaaagaaguauCgag....................  66       1  HS1
......................................................................uggaauguaaagaaguauUgag....................  62       1  HS1
......................................................................uggaauguaCagaaguauggag....................  42       1  HS1
......................................................................uggaauguaGagaaguauggag....................  764      1  HS1
......................................................................uggaauguaaagaUguauggag....................  37       1  HS1
......................................................................uUgaauguaaagaaguauggag....................  152      1  HS1
......................................................................uggaauguUaagaaguauggag....................  7        1  HS1
......................................................................ugCaauguaaagaaguauggag....................  47       1  HS1
......................................................................uggGauguaaagaaguauggag....................  1112     1  HS1
......................................................................uggaauguaaagaaguaugCag....................  35       1  HS1
......................................................................uggaauguaaCgaaguauggag....................  23       1  HS1
......................................................................uggaauguaaagaaguauggag....................  3099828  0  HS1
......................................................................Cggaauguaaagaaguauggag....................  2371     1  HS1
......................................................................uggaauguaaagaaguauggGg....................  1316     1  HS1
......................................................................Gggaauguaaagaaguauggag....................  2274     1  HS1
......................................................................uggaaugAaaagaaguauggag....................  204      1  HS1
......................................................................uggaauguaaagaagAauggag....................  324      1  HS1
......................................................................Aggaauguaaagaaguauggag....................  1364     1  HS1
......................................................................uggaauUuaaagaaguauggag....................  77       1  HS1
......................................................................uggUauguaaagaaguauggag....................  83       1  HS1
......................................................................uggaauguaaagaaguaugAag....................  349      1  HS1
......................................................................uggaaugGaaagaaguauggag....................  351      1  HS1
......................................................................uggaauguaUagaaguauggag....................  60       1  HS1
......................................................................uggaauguaaUgaaguauggag....................  15       1  HS1
......................................................................uggaauguaaagaaUuauggag....................  37       1  HS1
......................................................................uggCauguaaagaaguauggag....................  74       1  HS1
......................................................................uggaauguaaGgaaguauggag....................  683      1  HS1
......................................................................uggaauguaUagaaguauggagu...................  10       1  HS1
......................................................................uggaauguaaagaaguaCggagu...................  56       1  HS1
......................................................................uggaauguaaagaaguauggUgu...................  19       1  HS1
......................................................................uggaauguaaagaUguauggagu...................  8        1  HS1
......................................................................uggaauguaaagaaguaugCagu...................  3        1  HS1
......................................................................uggaaGguaaagaaguauggagu...................  71       1  HS1
......................................................................uggaauguaaagaaAuauggagu...................  20       1  HS1
......................................................................uggaauguaaagaaguauggaAu...................  46       1  HS1
......................................................................uggaauguaaagCaguauggagu...................  8        1  HS1
......................................................................uggCauguaaagaaguauggagu...................  4        1  HS1
......................................................................uggaauguUaagaaguauggagu...................  2        1  HS1
......................................................................uggaauguaaagaaguauggagC...................  4092     1  HS1
......................................................................uggaauguaGagaaguauggagu...................  102      1  HS1
......................................................................uggaauUuaaagaaguauggagu...................  6        1  HS1
......................................................................uggaauguaaagaaguauCgagu...................  3        1  HS1
......................................................................uggaauguaaagaaguauggagG...................  913      1  HS1
......................................................................uggaauguaaagaGguauggagu...................  110      1  HS1
```

Star Mature

acauuucucaaucggugcccucuaguggaucccguacuucuucacaucuccauauuucauuggauauuuauggaauguaaagaaguauggaguuuacuaauguaaaacgagg

....uggaauguaaagaaguauggCgu...................	9	1	HS1
....uggaauguaaagUaguauggagu...................	8	1	HS1
....uggaauguaaagaaguauggaCu...................	123	1	HS1
....uggaauguaaGgaaguauggagu...................	70	1	HS1
....uggaauguaCagaaguauggagu...................	6	1	HS1
....uggaauguaaagaCguauggagu...................	2	1	HS1
....uUgaauguaaagaaguauggagu...................	16	1	HS1
....uggUauguaaagaaguauggagu...................	13	1	HS1
....uggaauguaaUgaaguauggagu...................	1	1	HS1
....uAgaauguaaagaaguauggagu...................	56	1	HS1
....uggaauguaaagaagAauggagu...................	28	1	HS1
....ugUaauguaaagaaguauggagu...................	9	1	HS1
....Aggaauguaaagaaguauggagu...................	185	1	HS1
....uggGauguaaagaaguauggagu...................	161	1	HS1
....uggaauguGaagaaguauggagu...................	74	1	HS1
....Gggaauguaaagaaguauggagu...................	284	1	HS1
....uggaauguaaagaaguaAggagu...................	20	1	HS1
....uggaauguaaagaaguUuggagu...................	7	1	HS1
....uggaauguaaagaaguauggaUu...................	979	1	HS1
....uggaauguaaagaaCuauggagu...................	9	1	HS1
....uggaaugCaaagaaguauggagu...................	61	1	HS1
....uggaauguCaagaaguauggagu...................	2	1	HS1
....uggaauguaaagaaUuauggagu...................	2	1	HS1
....uggaaugAaaagaaguauggagu...................	29	1	HS1
....uggaauguaaagaaguauAgagu...................	18	1	HS1
....ugAaauguaaagaaguauggagu...................	20	1	HS1
....uggaauguaaaUaaguauggagu...................	3	1	HS1
....uggaauguaaaCaaguauggagu...................	2	1	HS1
....uggaauguaaCgaaguauggagu...................	6	1	HS1
....uggaauguaaagaagCauggagu...................	85	1	HS1
....uggaauguaaagaaguauggagu...................	370764	0	HS1
....uggaGuguaaagaaguauggagu...................	92	1	HS1
....uggaauguaaagaaguaugAagu...................	30	1	HS1
....uggaauguaaagaaguaugUagu...................	11	1	HS1
....uggaaCguaaagaaguauggagu...................	66	1	HS1
....uggaauguaaagGaguauggagu...................	73	1	HS1
....uggaauguaaagaaguauUgagu...................	5	1	HS1
....uggaauAuaaagaaguauggagu...................	36	1	HS1
....uggaUuguaaagaaguauggagu...................	4	1	HS1
....uggaaugGaaagaaguauggagu...................	51	1	HS1
....Cggaauguaaagaaguauggagu...................	298	1	HS1
....uggaauguaaagaaguGuggagu...................	56	1	HS1
....uggaauguaaagaaguaGggagu...................	29	1	HS1
....uggaauguaaagaaguCuggagu...................	1	1	HS1
....uggaaAguaaagaaguauggagu...................	22	1	HS1
....uggaauCuaaagaaguauggagu...................	4	1	HS1
....ugCaauguaaagaaguauggagu...................	10	1	HS1
....uggaauguaaagaaguauggGgu...................	138	1	HS1
....uggaauguaaaAaaguauggagu...................	15	1	HS1
....uggaauguaaagaaguauggagA...................	206832	1	HS1
....uggaauguaaagaagGauggagu...................	25	1	HS1
....uAgaauguaaagaaguauggaguu..................	1	1	HS1
....uggaauguaaagaaguauggaguA..................	235	1	HS1
....uggaauguaaagaaguauggagCu..................	55	1	HS1
....uggaauguaaagaaguauggaguG..................	25	1	HS1
....uggaauguaaagaaguGuggaguu..................	1	1	HS1
....uggaauguaaagaGguauggaguu..................	1	1	HS1
....uggaauguaaagaaguauggagGu..................	1	1	HS1
....uggaauguaaagaaguauggaAuu..................	4	1	HS1
....uggaauguaGagaaguauggaguu..................	1	1	HS1
....uggaauguaaagGaguauggaguu..................	1	1	HS1
....uggaauguaaagaaguauggaguC..................	20	1	HS1
....uggaauguaaagaaguauggGguu..................	1	1	HS1
....uggaauguaaagaaguauggaUuu..................	13	1	HS1
....uggaauguaaagaaguauggagAu..................	243	1	HS1
....uggaauAuaaagaaguauggaguu..................	1	1	HS1
....uggaauguaaagaagGauggaguu..................	1	1	HS1
....uggaauguaaGgaaguauggaguu..................	2	1	HS1
....uggaauguaaagaagCauggaguu..................	1	1	HS1
....Gggaauguaaagaaguauggaguu..................	2	1	HS1

Star **Mature**

acauuucucaaucggugcccucuaguggaucccguacuucuucacaucuccauauuucauuggauauuuauggaauguaaagaaguauggaguuuacuaauguaaaacgagg

....uggaauUuaaagaaguauggaguu..................	1	1	HS1
....uggaauguaaagaaguauggaguu..................	1455	0	HS1
....uggaauguaaagaaguauggagAuu.................	94	1	HS1
....uggaauguaaagaaguauggaguuu.................	87	0	HS1
....uggaauguaaagaaguauggaguuC.................	4	1	HS1
....uggaauguaaagaaguauggaguuA.................	7	1	HS1
....uggaauguaaagaaguauggaAuuu.................	2	1	HS1
....uggaauguaaagaaguauggaguCu.................	1	1	HS1
....uggaauguaaagaaguauggaguAu.................	3	1	HS1
....uggaauguaaagaaguauggaguuuU................	5	1	HS1
....ggaauguGaagaaguaug.......................	1	1	HS1
....ggaauguaaagaaguaug.......................	504	0	HS1
....ggaauguaaagaaguaGgg......................	1	1	HS1
....ggaauguaaagaaguaugU......................	2	1	HS1
....ggaauguaaagaaguaugA......................	2	1	HS1
....ggaauguaaagaaguaugg......................	816	0	HS1
....ggGauguaaagaaguaugg......................	3	1	HS1
....ggaaCguaaagaaguaugga.....................	4	1	HS1
....Agaauguaaagaaguaugga.....................	8	1	HS1
....ggGauguaaagaaguaugga.....................	10	1	HS1
....ggaauguaaGgaaguaugga.....................	1	1	HS1
....ggaaAguaaagaaguaugga.....................	4	1	HS1
....ggaGuguaaagaaguaugga.....................	10	1	HS1
....ggaauguaaagaaguauggG.....................	13	1	HS1
....ggaauguaGagaaguaugga.....................	2	1	HS1
....ggaauguGaagaaguaugga.....................	1	1	HS1
....ggaauguaaagUaguaugga.....................	1	1	HS1
....ggaauguaaagaaguaugga.....................	21285	0	HS1
....ggaauguaaagaaguauggC.....................	1	1	HS1
....ggaauguaaagaaguGugga.....................	4	1	HS1
....ggaauguaaagaaguaCgga.....................	9	1	HS1
....ggaauAuaaagaaguaugga.....................	7	1	HS1
....ggaauguaUagaaguaugga.....................	1	1	HS1
....ggaauguaaUgaaguaugga.....................	1	1	HS1
....ggaauguaaaAaaguaugga.....................	1	1	HS1
....ggaaGguaaagaaguaugga.....................	6	1	HS1
....ggaauguaaCgaaguaugga.....................	1	1	HS1
....ggaaugGaaagaaguaugga.....................	6	1	HS1
....ggaauguaaagaaguaAgga.....................	5	1	HS1
....ggaaugAaaagaaguaugga.....................	2	1	HS1
....ggaauguaaagaaguaugAa.....................	7	1	HS1
....ggaauguaaagaagCaugga.....................	7	1	HS1
....gUaauguaaagaaguaugga.....................	2	1	HS1
....ggaauguaaagaaAuaugga.....................	1	1	HS1
....Ugaauguaaagaaguaugga.....................	26	1	HS1
....ggaauguaaagaaguauUga.....................	4	1	HS1
....ggaauguaaagaGguaugga.....................	1	1	HS1
....ggaauguaaagaaguauggU.....................	38	1	HS1
....ggaauguaaagaagGaugga.....................	3	1	HS1
....ggaauguaaagaaCuaugga.....................	1	1	HS1
....ggaauguaaagaaguCugga.....................	2	1	HS1
....ggUauguaaagaaguaugga.....................	1	1	HS1
....ggaauguaaagaaguaugCa.....................	1	1	HS1
....ggaaugCaaagaaguaugga.....................	9	1	HS1
....ggaauguaaagaaguauAga.....................	2	1	HS1
....ggaauguaaagGaguaugga.....................	4	1	HS1
....ggaauCuaaagaaguaugga.....................	1	1	HS1
....ggaauguaaagaGguauggag....................	56	1	HS1
....ggaauguaaagaagCauggag....................	60	1	HS1
....ggaauUuaaagaaguauggag....................	4	1	HS1
....ggaauguaaGgaaguauggag....................	55	1	HS1
....ggaauguaaagaaguauggaA....................	484	1	HS1
....gCaauguaaagaaguauggag....................	8	1	HS1
....ggaauguUaagaaguauggag....................	2	1	HS1
....ggaauguaaagCaguauggag....................	10	1	HS1
....ggaauguaCagaaguauggag....................	3	1	HS1
....ggaauguaaagaaguauggCg....................	27	1	HS1
....ggGauguaaagaaguauggag....................	99	1	HS1
....ggaauguaaagaaguaugCag....................	3	1	HS1
....ggaauguaaaUaaguauggag....................	6	1	HS1

Star **Mature**

acauuucucaaucggugcccucuaguggaucccguacuucuucacaucuccauauuucauuggauauuuauggaauguaaagaaguauggaguuuacuaauguaaaacgagg			
....ggaauguaaagaaguauggaU....................	208	1	HS1
....ggaauguaUagaaguauggag....................	1	1	HS1
....ggaauguaaagaagAauggag....................	39	1	HS1
....ggaauguaaagaaguauggag....................	243633	0	HS1
....ggaauguaaUgaaguauggag....................	6	1	HS1
....Ugaauguaaagaaguauggag....................	335	1	HS1
....ggCauguaaagaaguauggag....................	2	1	HS1
....ggaauAuaaagaaguauggag....................	21	1	HS1
....ggaauguaaagUaguauggag....................	6	1	HS1
....Agaauguaaagaaguauggag....................	66	1	HS1
....ggaaugGaaagaaguauggag....................	52	1	HS1
....ggaauguaaagaaguCuggag....................	3	1	HS1
....ggaaAguaaagaaguauggag....................	29	1	HS1
....ggaauguaaagaaAuauggag....................	10	1	HS1
....ggaauguaaagaaguaGggag....................	33	1	HS1
....ggaaGguaaagaaguauggag....................	86	1	HS1
....ggaauguaaagaaUuauggag....................	4	1	HS1
....ggaauguaaagaaguaugAag....................	18	1	HS1
....ggaaugCaaagaaguauggag....................	34	1	HS1
....ggaauguaaagaaguGuggag....................	65	1	HS1
....ggaauguaaagaaCuauggag....................	1	1	HS1
....ggaauguaaagaaguauCgag....................	5	1	HS1
....ggaaCguaaagaaguauggag....................	42	1	HS1
....ggaauguaaagaaguauggUg....................	12	1	HS1
....ggaauguaaagaaguauggaC....................	80	1	HS1
....ggUauguaaagaaguauggag....................	3	1	HS1
....ggaauguaaagaaguauggGg....................	101	1	HS1
....ggaauguaaagaagGauggag....................	82	1	HS1
....ggaauguaaCgaaguauggag....................	4	1	HS1
....ggaauguaGagaaguauggag....................	61	1	HS1
....ggaauguaaagaaguauAgag....................	30	1	HS1
....ggaauguaaagGaguauggag....................	52	1	HS1
....Cgaauguaaagaaguauggag....................	11	1	HS1
....ggaauguaaagaUguauggag....................	7	1	HS1
....ggaauguaaagaCguauggag....................	4	1	HS1
....ggaauguaaagaaguaugUag....................	4	1	HS1
....ggaauguCaagaaguauggag....................	1	1	HS1
....ggaGuguaaagaaguauggag....................	54	1	HS1
....ggaaugAaaagaaguauggag....................	15	1	HS1
....ggaCuguaaagaaguauggag....................	2	1	HS1
....ggaauguaaagaaguaAggag....................	19	1	HS1
....ggaauguaaagaaguauUgag....................	12	1	HS1
....ggaauguaaagaaguaCggag....................	40	1	HS1
....ggaauguaaaAaaguauggag....................	9	1	HS1
....ggaauguaaaCaaguauggag....................	1	1	HS1
....ggaauguaaagaaguUuggag....................	9	1	HS1
....ggaauguGaagaaguauggag....................	42	1	HS1
....gUaauguaaagaaguauggag....................	9	1	HS1
....gAaauguaaagaaguauggag....................	18	1	HS1
....ggaaugGaaagaaguauggagu...................	7	1	HS1
....ggaauguaGagaaguauggagu...................	5	1	HS1
....ggGauguaaagaaguauggagu...................	8	1	HS1
....ggaauguaaagaaguauAgagu...................	7	1	HS1
....ggaaGguaaagaaguauggagu...................	4	1	HS1
....ggaauguaaagaaguaAggagu...................	1	1	HS1
....ggaaugCaaagaaguauggagu...................	8	1	HS1
....ggaauguaaagaaguauggGgu...................	11	1	HS1
....Cgaauguaaagaaguauggagu...................	1	1	HS1
....ggaauguaUagaaguauggagu...................	1	1	HS1
....ggaGuguaaagaaguauggagu...................	8	1	HS1
....ggaauguaaagaagGauggagu...................	7	1	HS1
....ggaauguaaagaaguauggagG...................	69	1	HS1
....ggaaugAaaagaaguauggagu...................	2	1	HS1
....ggaaCguaaagaaguauggagu...................	3	1	HS1
....ggCauguaaagaaguauggagu...................	1	1	HS1
....Agaauguaaagaaguauggagu...................	7	1	HS1
....ggaauguaaagaaguGuggagu...................	7	1	HS1
....ggaauguaCagaaguauggagu...................	1	1	HS1
....ggaauguaaagaaguauggaUu...................	56	1	HS1
....ggaauguaaagaaguauggCgu...................	3	1	HS1

Star   Mature

```
acauuucucaaucggugcccucuaguggaucccguacuucuucacaucuccauauuucauuggauauuuauggaauguaaagaaguauggaguuuacuaauguaaaacgagg
.......................................................................ggaauguCaagaaguauggagu...................    1       1       HS1
.......................................................................ggaauguaaGgaaguauggagu...................    9       1       HS1
.......................................................................ggaauCuaaagaaguauggagu...................    1       1       HS1
.......................................................................ggaauguaaagaagCauggagu...................    5       1       HS1
.......................................................................ggaauguaaagaaguauggaCu...................    6       1       HS1
.......................................................................ggaauguaaaAaaguauggagu...................    2       1       HS1
.......................................................................ggaauAuaaagaaguauggagu...................    4       1       HS1
.......................................................................ggaauguaaagaaguaugAagu...................    1       1       HS1
.......................................................................ggaauguaaagaGguauggagu...................    8       1       HS1
.......................................................................ggaauguaaagaaguaGggagu...................    1       1       HS1
.......................................................................ggaUuguaaagaaguauggagu...................    1       1       HS1
.......................................................................ggaauguGaagaaguauggagu...................    3       1       HS1
.......................................................................ggaauUuaaagaaguauggagu...................    3       1       HS1
.......................................................................ggaauguaaagaaguauggagC...................    300     1       HS1
.......................................................................ggaauguaaagaaguUuggagu...................    2       1       HS1
.......................................................................ggaauguaaagaaguaCggagu...................    5       1       HS1
.......................................................................ggaauguaaagaaguauggUgu...................    1       1       HS1
.......................................................................ggaauguaaagaaguauggagu...................    30477   0       HS1
.......................................................................ggaauguaaagaagAauggagu...................    2       1       HS1
.......................................................................gAaauguaaagaaguauggagu...................    3       1       HS1
.......................................................................ggaauguaaagGaguauggagu...................    3       1       HS1
.......................................................................gUaauguaaagaaguauggagu...................    1       1       HS1
.......................................................................ggaaAguaaagaaguauggagu...................    8       1       HS1
.......................................................................ggaauguaaagaaguauggagA...................    16473   1       HS1
.......................................................................Ugaauguaaagaaguauggagu...................    41      1       HS1
.......................................................................ggaauguaaagaaguauggaguG..................    2       1       HS1
.......................................................................ggaauguaaagaaguauggaguA..................    18      1       HS1
.......................................................................ggaauguaaagaaguaAggaguu..................    1       1       HS1
.......................................................................ggaauguaaagaaguauggaUuu..................    1       1       HS1
.......................................................................ggaauguaaagaaguauggaguu..................    104     0       HS1
.......................................................................ggaauguaaagaaguauggagAu..................    22      1       HS1
.......................................................................ggaauguaaagaaguauggagAuu.................    2       1       HS1
.......................................................................ggaauguaaagaaguauggaguuuU................    1       1       HS1
........................................................................Uaauguaaagaaguauggag....................    1       1       HS1
........................................................................gaauguaaagaaguauggag....................    10      0       HS1
........................................................................gGauguaaagaaguauggag....................    2       1       HS1
........................................................................gaauguaaagaaguauggagu...................    75      0       HS1
........................................................................gaauguaaagaaguauggagA...................    14      1       HS1
........................................................................gaauguaaagaaguauggaUu...................    1       1       HS1
........................................................................gaauguaaagaaguauggaguu..................    5       0       HS1
........................................................................gaauguaaagaaguauggagAu..................    7       1       HS1
........................................................................gaauguaaagaaguauggaguA..................    17      1       HS1
.........................................................................aauguaaagaaguauggag....................    8       0       HS1
..........................................................................auguaaagaaguauggag....................    13      0       HS1
..........................................................................auguaaagaaguauggagA...................    2       1       HS1
...........................................................................uguaaagaaguauggagu...................    2       0       HS1

.......ucaaucggugcccucuaguggauc.................................................................................    1       0       HS6
............................aucccguacuucuucacaucu...............................................................    1       0       HS6
.............................uUccguacuucuucacaucuccaua..........................................................    1       1       HS6
..............................cccguacuucuucacaucucc.............................................................    2       0       HS6
..............................cccguacuucuucacaucucca............................................................    16      0       HS6
..............................cccguacuucuucacaucuccau...........................................................    6       0       HS6
..............................cccguacuucuucacaucuccauU..........................................................    3       1       HS6
..............................cccguacuucuucacaucuccaua..........................................................    6       0       HS6
..............................cccguacuucuucacaucuccauUu.........................................................    1       1       HS6
...............................ccguacuucuucacaucuc..............................................................    1       0       HS6
...............................ccguacuucuucacaucucc.............................................................    3       0       HS6
...............................ccgCacuucuucacaucucca............................................................    1       1       HS6
...............................ccUuacuucuucacaucucca............................................................    1       1       HS6
...............................ccguacuucuucacaucucca............................................................    281     0       HS6
...............................ccguacuucuuUacaucucca............................................................    1       1       HS6
...............................ccguacuucuCcacaucucca............................................................    1       1       HS6
...............................ccguacuucuucacaucuccau...........................................................    79      0       HS6
...............................ccguacuucuucacaucuccaA...........................................................    2       1       HS6
...............................ccguacuucuucacaucuccaAa..........................................................    1       1       HS6
...............................ccguacuucuucacaucuUcaua..........................................................    1       1       HS6
...............................ccgGacuucuucacaucuccaua..........................................................    1       1       HS6
...............................ccguacuucuucacaucucAaua..........................................................    1       1       HS6
...............................ccguacuucuucacaucuccauU..........................................................    118     1       HS6
```

Star Mature

```
acauuucucaaucggugcccucuaguggaucccguacuucuucacaucuccauauuucauuggauauuuauggaauguaaagaaguauggaguuuacuaauguaaaacgagg
...............................ccguacuucuucacaucuccaua..........................................................  516   0  HS6
...............................ccguacuucuucaUaucuccaua..........................................................  1     1  HS6
...............................ccguacuucuucacaucuccauC..........................................................  1     1  HS6
...............................ccguacuucuucacaucuccauG..........................................................  3     1  HS6
...............................ccguacuucuucacaucuccauau.........................................................  2     0  HS6
...............................ccguacuucuucacaucuccauaA.........................................................  1     1  HS6
...............................ccguacuucuucacaucuccauauu........................................................  2     0  HS6
...............................ccguacuucuucacaucuccauauA........................................................  2     1  HS6
...............................ccguacuucuucacaucuccauauG........................................................  1     1  HS6
................................cguacuucuucacaucuccauU..........................................................  1     1  HS6
................................cguacuucuucacaucuccaua..........................................................  2     0  HS6
.................................guacuucuucacaucuccaua..........................................................  1     0  HS6
...................................................................uuauggaauguaaagaagua.........................  5     0  HS6
...................................................................uuauggaauguaaagaaguaug.......................  5     0  HS6
...................................................................uuauggaauguaaagaaguaugg......................  1     0  HS6
...................................................................uuUuggaauguaaagaaguaugga.....................  1     1  HS6
...................................................................uuUuggaauguaaagaaguauggag....................  2     1  HS6
...................................................................uuauggaauguaaagaaguauggag....................  1     0  HS6
....................................................................uUuggaauguaaagaagu..........................  1     1  HS6
....................................................................uauggaauguaaagaaguaug.......................  3     0  HS6
....................................................................Aauggaauguaaagaaguaugg......................  1     1  HS6
....................................................................uUuggaauguaaagaaguaugg......................  1     1  HS6
....................................................................uauggaauguaaagaaguaugg......................  2     0  HS6
....................................................................uUuggaauguaaagaaguaugga.....................  6     1  HS6
....................................................................Cauggaauguaaagaaguaugga.....................  1     1  HS6
....................................................................uUuggaauguaaagaaguauggag....................  112   1  HS6
....................................................................Aauggaauguaaagaaguauggag....................  2     1  HS6
....................................................................uauggaauguaaagaaguauggag....................  2     0  HS6
....................................................................uCuggaauguaaagaaguauggag....................  1     1  HS6
....................................................................uauggaauguaaagaaguauggagu...................  2     0  HS6
....................................................................uUuggaauguaaagaaguauggagu...................  8     1  HS6
.....................................................................auggaauguaaagaaguaug.......................  1     0  HS6
.....................................................................Uuggaauguaaagaaguaug.......................  2     1  HS6
.....................................................................auggaauguaaagaaguaugg......................  7     0  HS6
.....................................................................Uuggaauguaaagaaguaugg......................  17    1  HS6
.....................................................................Guggaauguaaagaaguaugga.....................  1     1  HS6
.....................................................................auggaauguaaagaaguaugga.....................  8     0  HS6
.....................................................................auggaauguaaagaaguauggU.....................  2     1  HS6
.....................................................................Uuggaauguaaagaaguaugga.....................  48    1  HS6
.....................................................................auggaauguaaagaaguauggaA....................  3     1  HS6
.....................................................................aGggaauguaaagaaguauggag....................  2     1  HS6
.....................................................................Cuggaauguaaagaaguauggag....................  16    1  HS6
.....................................................................auggaauguaaagaaguauggag....................  33    0  HS6
.....................................................................auggaauguaaagaaguUuggag....................  1     1  HS6
.....................................................................Uuggaauguaaagaaguauggag....................  461   1  HS6
.....................................................................Guggaauguaaagaaguauggag....................  4     1  HS6
.....................................................................Uuggaauguaaagaaguauggagu...................  24    1  HS6
.....................................................................auggaauguaaagaaguauggaUu...................  1     1  HS6
.....................................................................auggaauguaaagaaguauggagu...................  4     0  HS6
.....................................................................Guggaauguaaagaaguauggagu...................  1     1  HS6
.....................................................................auggaauguaaagaaguauggagA...................  3     1  HS6
.....................................................................Uuggaauguaaagaaguauggaguu..................  2     1  HS6
......................................................................uggaauguaaagaaguau........................  159   0  HS6
......................................................................uggaauguaaagaaguaA........................  1     1  HS6
......................................................................uggaauguaaagaagCau........................  1     1  HS6
......................................................................uggaauguaaagaaguaAg.......................  1     1  HS6
......................................................................uggaauguaaagaaguaGg.......................  1     1  HS6
......................................................................ugCaauguaaagaaguaug.......................  1     1  HS6
......................................................................uggaauguaaagaaguauA.......................  4     1  HS6
......................................................................ugUaauguaaagaaguaug.......................  1     1  HS6
......................................................................uggaauguaaagaaguaug.......................  1083  0  HS6
......................................................................uggaauguaaUgaaguaugg......................  1     1  HS6
......................................................................uggUauguaaagaaguaugg......................  1     1  HS6
......................................................................Gggaauguaaagaaguaugg......................  7     1  HS6
......................................................................uggaauguaaagCaguaugg......................  2     1  HS6
......................................................................uUgaauguaaagaaguaugg......................  4     1  HS6
......................................................................uggaauguGaagaaguaugg......................  2     1  HS6
......................................................................uggaauguaUagaaguaugg......................  3     1  HS6
......................................................................uggaauUuaaagaaguaugg......................  1     1  HS6
......................................................................uggaauguaaagaaAuaugg......................  2     1  HS6
```

Star Mature

acauuucucaaucggugcccucuaguggaucccguacuucuucacaucuccauauuucauuggauauuuauggaauguaaagaaguauggaguuuacuaauguaaaacgagg			
....uggaauguaaagaaguaugC......................	2	1	HS6
....uggaauguaaagaGguaugg......................	3	1	HS6
....uggaauguaaagaaguaugg......................	25164	0	HS6
....uggaauguaaaUaaguaugg......................	3	1	HS6
....uggaauguaaagaaguGugg......................	2	1	HS6
....uggaauguaaagUaguaugg......................	1	1	HS6
....uggCauguaaagaaguaugg......................	6	1	HS6
....uggaauguaaagaaguaCgg......................	2	1	HS6
....uggaauguaGagaaguaugg......................	1	1	HS6
....uggaaCguaaagaaguaugg......................	3	1	HS6
....uggaauguaaagaaguaugA......................	76	1	HS6
....uggaauguaaagaaguaugU......................	7	1	HS6
....ugCaauguaaagaaguaugg......................	16	1	HS6
....Aggaauguaaagaaguaugg......................	17	1	HS6
....uggaauguaaagaagCaugg......................	1	1	HS6
....Cggaauguaaagaaguaugg......................	6	1	HS6
....uggaauAuaaagaaguaugg......................	1	1	HS6
....uggaauguaaagGaguaugg......................	1	1	HS6
....uggaUuguaaagaaguaugg......................	17	1	HS6
....uAgaauguaaagaaguaugg......................	2	1	HS6
....uggaauguUaagaaguaugg......................	4	1	HS6
....uggaauguaaGgaaguaugg......................	1	1	HS6
....ugAaauguaaagaaguaugg......................	4	1	HS6
....uggaauguaaagaaUuaugg......................	2	1	HS6
....uggGauguaaagaaguaugg......................	1	1	HS6
....uggaauguaaCgaaguaugg......................	8	1	HS6
....uggaauguaaagaaguaGgg......................	6	1	HS6
....uggaauCuaaagaaguaugg......................	2	1	HS6
....uggaauguaaagaCguaugg......................	1	1	HS6
....uggaaugCaaagaaguaugg......................	4	1	HS6
....uggaGuguaaagaaguaugg......................	2	1	HS6
....uggaauguaaaCaaguaugg......................	4	1	HS6
....uggaauguaaagaaguauCg......................	9	1	HS6
....uggaauguaaagaaguaAgg......................	276	1	HS6
....ugUaauguaaagaaguaugg......................	16	1	HS6
....uCgaauguaaagaaguaugg......................	6	1	HS6
....uggaauguaaagaaguauAg......................	26	1	HS6
....uggaaGguaaagaaguaugga.....................	3	1	HS6
....uggaCuguaaagaaguaugga.....................	1	1	HS6
....uggCauguaaagaaguaugga.....................	45	1	HS6
....ugAaauguaaagaaguaugga.....................	39	1	HS6
....uggaauCuaaagaaguaugga.....................	5	1	HS6
....uggaGuguaaagaaguaugga.....................	8	1	HS6
....uggaauguaaagaaguGugga.....................	5	1	HS6
....uggaauguaaagaaguauggU.....................	70	1	HS6
....uggaaAguaaagaaguaugga.....................	1	1	HS6
....uggaauguaaagaaguaugga.....................	113886	0	HS6
....uggaauguaaagUaguaugga.....................	5	1	HS6
....uggaauguCaagaaguaugga.....................	4	1	HS6
....uggaauguaaagaaguauggG.....................	171	1	HS6
....uggaauguaaagaaguauggC.....................	17	1	HS6
....uggaauguaaagaaguaugCa.....................	17	1	HS6
....uggaauguaaGgaaguaugga.....................	5	1	HS6
....uCgaauguaaagaaguaugga.....................	9	1	HS6
....uggaauUuaaagaaguaugga.....................	20	1	HS6
....uggaauguaaaUaaguaugga.....................	11	1	HS6
....uggaaugAaaagaaguaugga.....................	2	1	HS6
....uggaauguaaagaaguUugga.....................	1	1	HS6
....uggaauguaaagaaCuaugga.....................	5	1	HS6
....uggaauguaaagaagGaugga.....................	2	1	HS6
....uggaauguaaagaaAuaugga.....................	5	1	HS6
....uggaauguaaagaagAaugga.....................	1	1	HS6
....uggaUuguaaagaaguaugga.....................	51	1	HS6
....uggaauguaaagCaguaugga.....................	4	1	HS6
....uggaauguaaagaagCaugga.....................	7	1	HS6
....uggUauguaaagaaguaugga.....................	9	1	HS6
....uggaauguaaagaaguauCga.....................	30	1	HS6
....uggaauguaaCgaaguaugga.....................	30	1	HS6
....uggaauguaaagGaguaugga.....................	7	1	HS6
....uggaaugGaaagaaguaugga.....................	2	1	HS6

Star **Mature**

```
acauuucucaaucggugcccucuaguggaucccguacuucuucacaucuccauauuucauuggauauuuauggaauguaaagaaguauggaguuuacuaauguaaaacgagg
......................................................................uggaauguaUagaaguaugga.....................	5	1	HS6
......................................................................uggaauguaaagaaguaGgga.....................	2	1	HS6
......................................................................uggaauguaaagaaUuaugga.....................	11	1	HS6
......................................................................uggaaCguaaagaaguaugga.....................	7	1	HS6
......................................................................uggaauguaaagaaguaugUa.....................	9	1	HS6
......................................................................uUgaauguaaagaaguaugga.....................	9	1	HS6
......................................................................uggaaugCaaagaaguaugga.....................	5	1	HS6
......................................................................uggaauguaaagaCguaugga.....................	3	1	HS6
......................................................................ugCaauguaaagaaguaugga.....................	73	1	HS6
......................................................................uggaauguaaagaaguCugga.....................	2	1	HS6
......................................................................uggaauguaaaCaaguaugga.....................	2	1	HS6
......................................................................uggaauguaGagaaguaugga.....................	6	1	HS6
......................................................................Gggaauguaaagaaguaugga.....................	16	1	HS6
......................................................................uggaauAuaaagaaguaugga.....................	4	1	HS6
......................................................................uggaauguaCagaaguaugga.....................	2	1	HS6
......................................................................uggaauguUaagaaguaugga.....................	18	1	HS6
......................................................................Cggaauguaaagaaguaugga.....................	15	1	HS6
......................................................................uggaauguGaagaaguaugga.....................	3	1	HS6
......................................................................uggaauguaaagaaguauUga.....................	6	1	HS6
......................................................................uggaauguaaagaaguaugAa.....................	26	1	HS6
......................................................................uAgaauguaaagaaguaugga.....................	9	1	HS6
......................................................................uggaauguaaaAaaguaugga.....................	3	1	HS6
......................................................................uggaauguaaagaUguaugga.....................	4	1	HS6
......................................................................uggaauguaaagaGguaugga.....................	4	1	HS6
......................................................................uggaauguaaagaaguauAga.....................	6	1	HS6
......................................................................uggaauguaaagaaguaCgga.....................	6	1	HS6
......................................................................uggaauguaaUgaaguaugga.....................	4	1	HS6
......................................................................ugUaauguaaagaaguaugga.....................	63	1	HS6
......................................................................uggGauguaaagaaguaugga.....................	11	1	HS6
......................................................................Aggaauguaaagaaguaugga.....................	48	1	HS6
......................................................................uggaauguaaagaaguaAgga.....................	7	1	HS6
......................................................................uggaauCuaaagaaguauggag....................	57	1	HS6
......................................................................uggaauguaaagGaguauggag....................	68	1	HS6
......................................................................ugAaauguaaagaaguauggag....................	175	1	HS6
......................................................................uggaauguaaagaagCauggag....................	36	1	HS6
......................................................................uggaauguaaUgaaguauggag....................	24	1	HS6
......................................................................uggaauguaaagaaUuauggag....................	146	1	HS6
......................................................................uggaaCguaaagaaguauggag....................	29	1	HS6
......................................................................uggaauguaaagaagAauggag....................	7	1	HS6
......................................................................uggaauguaaagaaguauAgag....................	31	1	HS6
......................................................................uggaauguaaagaaguauggaC....................	3233	1	HS6
......................................................................uggaauguaaCgaaguauggag....................	184	1	HS6
......................................................................uggaauguaCagaaguauggag....................	7	1	HS6
......................................................................uggaauguaaaUaaguauggag....................	96	1	HS6
......................................................................uggaauguaaagaGguauggag....................	41	1	HS6
......................................................................uggaauguaaagCaguauggag....................	28	1	HS6
......................................................................uggCauguaaagaaguauggag....................	310	1	HS6
......................................................................uggaGuguaaagaaguauggag....................	74	1	HS6
......................................................................uggaauguGaagaaguauggag....................	36	1	HS6
......................................................................uggaauguaaagaaguaGggag....................	20	1	HS6
......................................................................uggGauguaaagaaguauggag....................	93	1	HS6
......................................................................uggaauguaaagaaguaugUag....................	116	1	HS6
......................................................................uAgaauguaaagaaguauggag....................	38	1	HS6
......................................................................uggaauguaaagaaguauUgag....................	64	1	HS6
......................................................................uggaauguaaagaaCuauggag....................	66	1	HS6
......................................................................uggaauguaaGgaaguauggag....................	36	1	HS6
......................................................................Aggaauguaaagaaguauggag....................	332	1	HS6
......................................................................uggaauguaaagUaguauggag....................	21	1	HS6
......................................................................uggaauguaaagaUguauggag....................	26	1	HS6
......................................................................uggaaugCaaagaaguauggag....................	45	1	HS6
......................................................................uggaauguaUagaaguauggag....................	14	1	HS6
......................................................................uggaauguaaagaaguauggaU....................	1878	1	HS6
......................................................................ugUaauguaaagaaguauggag....................	474	1	HS6
......................................................................uggaauguaaagaaguaCggag....................	25	1	HS6
......................................................................uggaauguaaagaaguGuggag....................	47	1	HS6
......................................................................uggaauguaaagaaguaugAag....................	101	1	HS6
......................................................................uggaauguaaagaaAuauggag....................	37	1	HS6
......................................................................uggaauguaaagaaguCuggag....................	12	1	HS6
......................................................................uggaUuguaaagaaguauggag....................	375	1	HS6
......................................................................uggaauguaaaCaaguauggag....................	53	1	HS6
```

```
                               Star                                   Mature
acauuucucaaucggugcccucuaguggaucccguacuucuucacaucuccauauuucauuggauauuuauggaauguaaagaaguauggaguuuacuaauguaaaacgagg
..........................................................................uggaCuguaaagaaguauggag....................  7       1   HS6
......................................................................uggaauguCaagaaguauggag....................  17      1   HS6
......................................................................Cggaauguaaagaaguauggag....................  117     1   HS6
......................................................................uggaaugAaaagaaguauggag....................  18      1   HS6
......................................................................uggaaGguaaagaaguauggag....................  22      1   HS6
......................................................................uggaauAuaaagaaguauggag....................  52      1   HS6
......................................................................uggaaAguaaagaaguauggag....................  4       1   HS6
......................................................................uggaauguaaagaaguauCgag....................  241     1   HS6
......................................................................uggaauguaaagaagGauggag....................  17      1   HS6
......................................................................uggaaugGaaagaaguauggag....................  11      1   HS6
......................................................................uggaauguaaagaaguUuggag....................  22      1   HS6
......................................................................uggaauguaaagaCguauggag....................  17      1   HS6
......................................................................uggaauguaGagaaguauggag....................  33      1   HS6
......................................................................uggaauguaaagaaguaAggag....................  6       1   HS6
......................................................................uggaauguaaagaaguauggGg....................  368     1   HS6
......................................................................uggaauguaaagaaguauggCg....................  136     1   HS6
......................................................................uCgaauguaaagaaguauggag....................  70      1   HS6
......................................................................ugCaauguaaagaaguauggag....................  432     1   HS6
......................................................................uggaauguUaagaaguauggag....................  103     1   HS6
......................................................................uggaauguaaagaaguauggag....................  825861  0   HS6
......................................................................uggaauUuaaagaaguauggag....................  97      1   HS6
......................................................................uggUauguaaagaaguauggag....................  73      1   HS6
......................................................................uggaauguaaagaaguauggUg....................  146     1   HS6
......................................................................uggaauguaaagaaguaugCag....................  84      1   HS6
......................................................................Gggaauguaaagaaguauggag....................  142     1   HS6
......................................................................uUgaauguaaagaaguauggag....................  92      1   HS6
......................................................................uggaauguaaagaaguauggaA....................  6977    1   HS6
......................................................................uggaauguaaaAaaguauggag....................  34      1   HS6
......................................................................uggaGuguaaagaaguauggagu...................  3       1   HS6
......................................................................uggaauguaaagaaguauAgagu...................  2       1   HS6
......................................................................uggaauguaaagaaguauggCgu...................  12      1   HS6
......................................................................uggaauguCaagaaguauggagu...................  1       1   HS6
......................................................................uggUauguaaagaaguauggagu...................  6       1   HS6
......................................................................uggaauguaaagaaguauggagA...................  83173   1   HS6
......................................................................uggaauguaaagaaUuauggagu...................  16      1   HS6
......................................................................ugAaauguaaagaaguauggagu...................  9       1   HS6
......................................................................uggaauguUaagaaguauggagu...................  12      1   HS6
......................................................................uggaauguaaaAaaguauggagu...................  1       1   HS6
......................................................................ugCaauguaaagaaguauggagu...................  37      1   HS6
......................................................................uggaauAuaaagaaguauggagu...................  7       1   HS6
......................................................................uggaauguaaagaaguauUgagu...................  4       1   HS6
......................................................................uggaauguGaagaaguauggagu...................  2       1   HS6
......................................................................uggaaCguaaagaaguauggagu...................  1       1   HS6
......................................................................uCgaauguaaagaaguauggagu...................  7       1   HS6
......................................................................uggaauguaaagaCguauggagu...................  1       1   HS6
......................................................................uggaauguaaagGaguauggagu...................  4       1   HS6
......................................................................uggaauguaaagUaguauggagu...................  3       1   HS6
......................................................................uggaauguaaagaaguauggaAu...................  56      1   HS6
......................................................................Gggaauguaaagaaguauggagu...................  13      1   HS6
......................................................................uggaauguaaagaagCauggagu...................  5       1   HS6
......................................................................uggaaAguaaagaaguauggagu...................  1       1   HS6
......................................................................uggCauguaaagaaguauggagu...................  29      1   HS6
......................................................................uUgaauguaaagaaguauggagu...................  5       1   HS6
......................................................................uggaauguaaagaGguauggagu...................  7       1   HS6
......................................................................uggaauguaaagaaguaugUagu...................  13      1   HS6
......................................................................uggaauguaaagaagAauggagu...................  1       1   HS6
......................................................................uggaauguaaagaaguauggGgu...................  12      1   HS6
......................................................................uggaauguaGagaaguauggagu...................  1       1   HS6
......................................................................uggaauguaaCgaaguauggagu...................  19      1   HS6
......................................................................uggaauguaaagaaguaugCagu...................  8       1   HS6
......................................................................Aggaauguaaagaaguauggagu...................  23      1   HS6
......................................................................Cggaauguaaagaaguauggagu...................  9       1   HS6
......................................................................uggaauguaaagaaguaGggagu...................  1       1   HS6
......................................................................uggaauguaaagaaguauggagG...................  1107    1   HS6
......................................................................uggaauguaaagaaguaCggagu...................  4       1   HS6
......................................................................uggaaugAaaagaaguauggagu...................  8       1   HS6
......................................................................uAgaauguaaagaaguauggagu...................  3       1   HS6
......................................................................uggaauguaaagaaguaugAagu...................  4       1   HS6
......................................................................uggaauguaaagaaguauggagC...................  703     1   HS6
......................................................................ugUaauguaaagaaguauggagu...................  33      1   HS6
```

```
                               Star                                      Mature
acauuucucaaucggugcccucuaguggaucccguacuucuucacaucuccauauuucauuggauauuuauggaauguaaagaaguauggaguuuacuaauguaaaacgagg
......................................................................uggaauguaaagaaguUuggagu...................   1      1   HS6
......................................................................uggaauguaaagaaguauCgagu...................   12     1   HS6
......................................................................uggaauUuaaagaaguauggagu...................   7      1   HS6
......................................................................uggaauguaaagaaguauggagu...................   65182  0   HS6
......................................................................uggaauguaaaCaaguauggagu...................   5      1   HS6
......................................................................uggGauguaaagaaguauggagu...................   8      1   HS6
......................................................................uggaauguaaagaaguauggaUu...................   166    1   HS6
......................................................................uggaauguaaagaaguCuggagu...................   2      1   HS6
......................................................................uggaauguaUagaaguauggagu...................   1      1   HS6
......................................................................uggaauguaaagaaCuauggagu...................   8      1   HS6
......................................................................uggaaugGaaagaaguauggagu...................   1      1   HS6
......................................................................uggaauguaaagaagGauggagu...................   2      1   HS6
......................................................................uggaauCuaaagaaguauggagu...................   5      1   HS6
......................................................................uggaUuguaaagaaguauggagu...................   30     1   HS6
......................................................................uggaauguaaagaaguGuggagu...................   1      1   HS6
......................................................................uggaauguaaaUaaguauggagu...................   10     1   HS6
......................................................................uggaaugCaaagaaguauggagu...................   4      1   HS6
......................................................................uggaauguaaagaaguauggaCu...................   160    1   HS6
......................................................................uggaauguaaagaaguauggUgu...................   7      1   HS6
......................................................................uggaauguaaagaaguaugUaguu..................   1      1   HS6
......................................................................uggaauguaaagaaguauggaguA..................   203    1   HS6
......................................................................Aggaauguaaagaaguauggaguu..................   1      1   HS6
......................................................................uAgaauguaaagaaguauggaguu..................   1      1   HS6
......................................................................uggaauguaaagaaguauggaUuu..................   2      1   HS6
......................................................................uggaauguaaagaaguauggaguC..................   7      1   HS6
......................................................................uggaauguaaagaaguauggaguG..................   1      1   HS6
......................................................................uggaauguaaagaaguauggaguu..................   1491   0   HS6
......................................................................uggaauguaaagaaguauggaAuu..................   1      1   HS6
......................................................................uggaauguaaagaaguauCgaguu..................   1      1   HS6
......................................................................uggaauguaaagaaguauggagCu..................   19     1   HS6
......................................................................uggaauguaaagaaguauggagAu..................   212    1   HS6
......................................................................uggaauguaaagaaguauggagGu..................   8      1   HS6
......................................................................uggaauguaaagaaguauggagAuu.................   14     1   HS6
......................................................................uggaauUuaaagaaguauggaguuu.................   2      1   HS6
......................................................................uggaauguaaagaaguauggaguAu.................   6      1   HS6
......................................................................uggaauguaaagaaguauggagGuu.................   1      1   HS6
......................................................................uggaauguaaagaaguauggaguuu.................   53     0   HS6
......................................................................uggaauguaaagaaguauggaguuG.................   1      1   HS6
......................................................................uggaauguaaagaaguauggagCuu.................   4      1   HS6
......................................................................uggaauguaaagaaguauggaguuA.................   7      1   HS6
......................................................................uggaauguaaagaaguauggaguuuU................   10     1   HS6
......................................................................uggaauguaaagaaguauggaguuua................   1      0   HS6
......................................................................uggaauguaaagaaguauggaguuuC................   2      1   HS6
.......................................................................Ugaauguaaagaaguaugg......................   1      1   HS6
.......................................................................ggaauguaaagaaguaugg......................   44     0   HS6
.......................................................................ggaauguaaagaaguaugA......................   1      1   HS6
.......................................................................ggaauguaaagaaguaAgg......................   2      1   HS6
.......................................................................Ugaauguaaagaaguaugga.....................   15     1   HS6
.......................................................................ggaauguaaagaaguaugga.....................   132    0   HS6
.......................................................................ggaauguaaaAaaguaugga.....................   1      1   HS6
.......................................................................ggaauguaaagaaguauUgag....................   1      1   HS6
.......................................................................ggaauCuaaagaaguauggag....................   1      1   HS6
.......................................................................gUaauguaaagaaguauggag....................   2      1   HS6
.......................................................................ggaauguaaagaaguauggaU....................   4      1   HS6
.......................................................................gAaauguaaagaaguauggag....................   7      1   HS6
.......................................................................ggGauguaaagaaguauggag....................   1      1   HS6
.......................................................................gCaauguaaagaaguauggag....................   1      1   HS6
.......................................................................ggaauguaaagaaguauggUg....................   1      1   HS6
.......................................................................Agaauguaaagaaguauggag....................   3      1   HS6
.......................................................................ggUauguaaagaaguauggag....................   1      1   HS6
.......................................................................ggaauguaaagaaguaugUag....................   2      1   HS6
.......................................................................ggaaGguaaagaaguauggag....................   1      1   HS6
.......................................................................Ugaauguaaagaaguauggag....................   111    1   HS6
.......................................................................ggaauguaaagaaguauggaC....................   3      1   HS6
.......................................................................ggaauguaaaAaaguauggag....................   4      1   HS6
.......................................................................ggaauguaaagaaguauggaA....................   6      1   HS6
.......................................................................Cgaauguaaagaaguauggag....................   9      1   HS6
.......................................................................ggaauguaaagaaguauggag....................   1044   0   HS6
.......................................................................ggaauguaaagaaAuauggag....................   1      1   HS6
.......................................................................ggaauguaaagaaguauggagA...................   122    1   HS6
```

Star Mature

acauuucucaaucggugcccucuaguggauccgguacuucuucacaucuccauauuucauuggauauuuauggaauguaaagaaguauggaguuuacuaauguaaaacgagg			
....ggaauguaaagaaguauggagC...................	4	1	HS6
....ggaauguaaagaaguauggagG...................	1	1	HS6
....ggaauguaaagaaguauggagu...................	109	0	HS6
....ggaauguaaagaaguaugUagu...................	1	1	HS6
....Ugaauguaaagaaguauggagu...................	7	1	HS6
....Ugaauguaaagaaguauggaguu..................	1	1	HS6
....ggaauguaaagaaguauggaguu..................	4	0	HS6
....gaauguaaagaaguaAgg......................	1	1	HS6
....gaauguaaagaaguaugg......................	10	0	HS6
....gaauguaaagaaUuaugga.....................	1	1	HS6
....gaauguaaagaaguaugga.....................	34	0	HS6
....Aaauguaaagaaguaugga.....................	3	1	HS6
....Uaauguaaagaaguaugga.....................	1	1	HS6
....Uaauguaaagaaguauggag....................	12	1	HS6
....gaauguaaagaaguauggaC....................	2	1	HS6
....gaauguaaagaaguauggaU....................	1	1	HS6
....Aaauguaaagaaguauggag....................	13	1	HS6
....gaauguaaagaaguauggag....................	427	0	HS6
....gaauguaaagaaguauggaA....................	3	1	HS6
....gUauguaaagaaguauggagu...................	1	1	HS6
....gaauguaaagaaguauggagG...................	1	1	HS6
....gaauguaaagaagCauggagu...................	1	1	HS6
....gaauguaaagaaguauggagu...................	401	0	HS6
....Aaauguaaagaaguauggagu...................	2	1	HS6
....gaauguaaagaaguauggagA...................	196	1	HS6
....gaauguaaagaaguauggagC...................	13	1	HS6
....gaauguGaagaaguauggagu...................	1	1	HS6
....gaauguaaagaaguauggaUu...................	4	1	HS6
....gaauguaaagaaguauggUguu..................	1	1	HS6
....gaauguaaagaaguauggagAu..................	20	1	HS6
....gaauguaaagaaguauggaguC..................	5	1	HS6
....gaauguaaagaaguauggaguu..................	317	0	HS6
....gaauguaaagaaguaugCaguu..................	1	1	HS6
....gaauguaaagaaguauggaguA..................	139	1	HS6
....gaUuguaaagaaguauggaguu..................	4	1	HS6
....gaauguaaagaaguauggaguG..................	5	1	HS6
....gaauguaaagaaguauggagCu..................	6	1	HS6
....gaauguaaagaaguauggaguuu.................	4	0	HS6
....gaauguaaagaaguauggaguuA.................	6	1	HS6
....gaauguaaagaaguauggaguuC.................	1	1	HS6
....aauguaaagaaguaugga.....................	29	0	HS6
....aauguaaagaaguauggag....................	294	0	HS6
....aauguaaagaaguauggaC....................	1	1	HS6
....aauguaaagaaguauggaU....................	1	1	HS6
....Uauguaaagaaguauggag....................	1	1	HS6
....aauguaaagaaguauggaA....................	4	1	HS6
....aauguaaagaaguauggagu...................	25	0	HS6
....aauguaaagaaguauggagA...................	37	1	HS6
....aauguaaagaaguauggaguu..................	3	0	HS6
....auUuaaagaaguauggag....................	1	1	HS6
....Uuguaaagaaguauggag....................	1	1	HS6
....auguaaagaaguauggag....................	351	0	HS6
....auguaaagaaguauggaC....................	2	1	HS6
....auCuaaagaaguauggag....................	1	1	HS6
....auguaaagaaguauggaU....................	1	1	HS6
....auguaaaAaaguauggag....................	1	1	HS6
....Cuguaaagaaguauggag....................	1	1	HS6
....auguaaagaaguauggagA...................	45	1	HS6
....auguaaagaaguauggagG...................	1	1	HS6
....auguaaagaaguauggagu...................	26	0	HS6
....auguaaagaaguauggagAu..................	1	1	HS6
....auguaaagaaguauggaguu..................	3	0	HS6
....uguaaagaUguauggagu...................	1	1	HS6
....uguaaagaaguauggagu...................	54	0	HS6
....uguaaagaaguauggagA...................	42	1	HS6
....uguaaagaaguauggaguu..................	1	0	HS6
....guaaagaaguauggagAu..................	1	1	HS6
....guaaagaaguauggaguu..................	6	0	HS6
.............................ucccguacuucuucacaucuc..	1	0	EGG

Star **Mature**

```
acauuucucaaucggugcccucuaguggaucccguacuucuucacaucuccauauuucauuggauauuuauggaauguaaagaaguauggaguuuacuaauguaaaacgagg
..............................cccguacuucuucacaucuccau...........................................................  1     0  EGG
...............................ccguacuucuucacaucuc..............................................................  3     0  EGG
...............................ccguacuucuucacaucucc.............................................................  1     0  EGG
...............................ccguacuucuucacaucucca............................................................  1     0  EGG
...............................ccguacuucuucacaucuccau...........................................................  2     0  EGG
...............................ccguacuucuucacaucuccaua..........................................................  2     0  EGG
..................................................................uuuUggaauguaaagaaguaugga......................  2     1  EGG
...................................................................uuauggaauguaaagaagua.........................  1     0  EGG
...................................................................uuCuggaauguaaagaaguauggag....................  1     1  EGG
...................................................................uuUuggaauguaaagaaguauggag....................  1     1  EGG
....................................................................uauggaauguaaagaagu..........................  1     0  EGG
....................................................................uUuggaauguaaagaaguaugg......................  4     1  EGG
....................................................................uGuggaauguaaagaaguaugga.....................  1     1  EGG
....................................................................uUuggaauguaaagaaguaugga.....................  21    1  EGG
....................................................................uUuggaauguaaagaaguauggag....................  11    1  EGG
.....................................................................auggaauguaaagaaguaug.......................  1     0  EGG
.....................................................................Uuggaauguaaagaaguaug.......................  1     1  EGG
.....................................................................auggaauguaaagaaguaugg......................  3     0  EGG
.....................................................................Uuggaauguaaagaaguaugg......................  3     1  EGG
.....................................................................Uuggaauguaaagaaguaugga.....................  32    1  EGG
.....................................................................Guggaauguaaagaaguaugga.....................  2     1  EGG
.....................................................................auggaauguaaagaaguaugga.....................  2     0  EGG
.....................................................................Uuggaauguaaagaaguauggag....................  23    1  EGG
.....................................................................Cuggaauguaaagaaguauggag....................  6     1  EGG
.....................................................................Uuggaauguaaagaaguauggagu...................  1     1  EGG
......................................................................uggaauguaaagaaguau........................  116   0  EGG
......................................................................uggaauguaaagaaguUu........................  1     1  EGG
......................................................................Aggaauguaaagaaguau........................  1     1  EGG
......................................................................uggaauguaaagaaguaA........................  6     1  EGG
......................................................................ugAaauguaaagaaguaug.......................  1     1  EGG
......................................................................Gggaauguaaagaaguaug.......................  1     1  EGG
......................................................................uggaauguUaagaaguaug.......................  1     1  EGG
......................................................................uggaauguaaagaaguauA.......................  3     1  EGG
......................................................................uggaauguaaagaagAaug.......................  1     1  EGG
......................................................................uggaauguaaagaaguauU.......................  2     1  EGG
......................................................................uggaauguaaagaaguaug.......................  807   0  EGG
......................................................................uUgaauguaaagaaguaug.......................  1     1  EGG
......................................................................uggaauguaaagaaguaCg.......................  1     1  EGG
......................................................................ugCaauguaaagaaguaugg......................  2     1  EGG
......................................................................uggaauguaaagaagCaugg......................  5     1  EGG
......................................................................uggaauUuaaagaaguaugg......................  1     1  EGG
......................................................................uggaauguaaagaaCuaugg......................  1     1  EGG
......................................................................uggaauguUaagaaguaugg......................  1     1  EGG
......................................................................uggaauguaaagUaguaugg......................  2     1  EGG
......................................................................Aggaauguaaagaaguaugg......................  5     1  EGG
......................................................................uggaauguaaagaagGaugg......................  2     1  EGG
......................................................................uggaauguaaagaUguaugg......................  1     1  EGG
......................................................................uggaaugAaaagaaguaugg......................  1     1  EGG
......................................................................uggaauguaaagaagAaugg......................  1     1  EGG
......................................................................uggaauguaaagaaguaugC......................  2     1  EGG
......................................................................uggaauguaaagaaguaCgg......................  3     1  EGG
......................................................................uggaaCguaaagaaguaugg......................  1     1  EGG
......................................................................uggaaugCaaagaaguaugg......................  1     1  EGG
......................................................................uggaauguaaUgaaguaugg......................  1     1  EGG
......................................................................uggaauguaaagaaguaugU......................  5     1  EGG
......................................................................ugAaauguaaagaaguaugg......................  1     1  EGG
......................................................................uggaauguaaagGaguaugg......................  1     1  EGG
......................................................................uggaauguaaagaaguaugg......................  5499  0  EGG
......................................................................uggaauguaaagaaguGugg......................  1     1  EGG
......................................................................Cggaauguaaagaaguaugg......................  1     1  EGG
......................................................................uggaauguaaagaaguaugA......................  119   1  EGG
......................................................................uggaauguaaagaagGaugga.....................  3     1  EGG
......................................................................uggaauguaaagaCguaugga.....................  1     1  EGG
......................................................................Aggaauguaaagaaguaugga.....................  15    1  EGG
......................................................................uggaauguaaagUaguaugga.....................  3     1  EGG
......................................................................uggCauguaaagaaguaugga.....................  1     1  EGG
......................................................................uggaauguaaagaaguGugga.....................  4     1  EGG
......................................................................uggaauguaaagaaguaugUa.....................  6     1  EGG
......................................................................uggaauCuaaagaaguaugga.....................  1     1  EGG
......................................................................ugCaauguaaagaaguaugga.....................  16    1  EGG
```

Star Mature

```
acauuucucaaucggugcccucuaguggaucccguacuucuucacaucuccauauuucauuggauauuuauggaauguaaagaaguauggaguuuacuaauguaaaacgagg
......................................................................uggaauguaaagaagAaugga.....................	9	1	EGG
......................................................................uggaauguaaCgaaguaugga.....................	1	1	EGG
......................................................................uggaauguaGagaaguaugga.....................	3	1	EGG
......................................................................Cggaauguaaagaaguaugga.....................	4	1	EGG
......................................................................uggaauguaaagaaguauCga.....................	2	1	EGG
......................................................................uggaauguaaagaaguauggC.....................	26	1	EGG
......................................................................uggaaCguaaagaaguaugga.....................	2	1	EGG
......................................................................uggaauguaaagaaUuaugga.....................	4	1	EGG
......................................................................uggaauguaaagaaguaugCa.....................	14	1	EGG
......................................................................uggaaugCaaagaaguaugga.....................	4	1	EGG
......................................................................uggaauguaaagaagCaugga.....................	13	1	EGG
......................................................................ugAaauguaaagaaguaugga.....................	12	1	EGG
......................................................................uUgaauguaaagaaguaugga.....................	1	1	EGG
......................................................................uggaauguaaagCaguaugga.....................	1	1	EGG
......................................................................uggaUuguaaagaaguaugga.....................	1	1	EGG
......................................................................uggaauguaaagaaguauggG.....................	47	1	EGG
......................................................................uggaauguaaagaGguaugga.....................	3	1	EGG
......................................................................uggaauguaaagaaguaugAa.....................	30	1	EGG
......................................................................uggaCuguaaagaaguaugga.....................	1	1	EGG
......................................................................uggaauguaaagaUguaugga.....................	1	1	EGG
......................................................................uggaauguGaagaaguaugga.....................	3	1	EGG
......................................................................uCgaauguaaagaaguaugga.....................	3	1	EGG
......................................................................uggaauguaaGgaaguaugga.....................	2	1	EGG
......................................................................uggaauguUaagaaguaugga.....................	1	1	EGG
......................................................................Gggaauguaaagaaguaugga.....................	5	1	EGG
......................................................................uAgaauguaaagaaguaugga.....................	3	1	EGG
......................................................................uggaauAuaaagaaguaugga.....................	2	1	EGG
......................................................................uggaGuguaaagaaguaugga.....................	2	1	EGG
......................................................................uggaauguaUagaaguaugga.....................	2	1	EGG
......................................................................uggaauguaaagGaguaugga.....................	6	1	EGG
......................................................................uggUauguaaagaaguaugga.....................	1	1	EGG
......................................................................ugUaauguaaagaaguaugga.....................	12	1	EGG
......................................................................uggaauguaaaCaaguaugga.....................	2	1	EGG
......................................................................uggaauguaaaUaaguaugga.....................	1	1	EGG
......................................................................uggaaugGaaagaaguaugga.....................	2	1	EGG
......................................................................uggaaugAaaagaaguaugga.....................	2	1	EGG
......................................................................uggaauguaaagaaguaAgga.....................	1	1	EGG
......................................................................uggaauguaaagaaguUugga.....................	4	1	EGG
......................................................................uggaauguaaagaaguauggU.....................	112	1	EGG
......................................................................uggaauguaaUgaaguaugga.....................	1	1	EGG
......................................................................uggGauguaaagaaguaugga.....................	5	1	EGG
......................................................................uggaauguaaagaaguauAga.....................	1	1	EGG
......................................................................uggaauguaaagaaguaugga.....................	40018	0	EGG
......................................................................uggaauguaaagaaCuaugga.....................	2	1	EGG
......................................................................uggaauguaaagaaguaGggag....................	2	1	EGG
......................................................................uggaauguaaagaaguauggag....................	23949	0	EGG
......................................................................uggaaugCaaagaaguauggag....................	6	1	EGG
......................................................................uAgaauguaaagaaguauggag....................	2	1	EGG
......................................................................uggaauguaaGgaaguauggag....................	1	1	EGG
......................................................................uggaauguaaaUaaguauggag....................	4	1	EGG
......................................................................uggaauguaaagaaUuauggag....................	3	1	EGG
......................................................................ugCaauguaaagaaguauggag....................	8	1	EGG
......................................................................uggaauguaaagaaguaugUag....................	2	1	EGG
......................................................................uggaauUuaaagaaguauggag....................	3	1	EGG
......................................................................uggaauguaaagaaguaugCag....................	3	1	EGG
......................................................................uggaauguaaagaaguauggGg....................	9	1	EGG
......................................................................uggaauguaaagaGguauggag....................	5	1	EGG
......................................................................uggaauguaaagGaguauggag....................	3	1	EGG
......................................................................uggaauguaaagaaguauggaC....................	98	1	EGG
......................................................................uggaauguaaagaagCauggag....................	11	1	EGG
......................................................................uggaauguaaagaUguauggag....................	3	1	EGG
......................................................................Cggaauguaaagaaguauggag....................	3	1	EGG
......................................................................uggaauguaaagaaguauggUg....................	14	1	EGG
......................................................................uggaauguaaagaaguUuggag....................	3	1	EGG
......................................................................ugAaauguaaagaaguauggag....................	10	1	EGG
......................................................................Aggaauguaaagaaguauggag....................	9	1	EGG
......................................................................ugUaauguaaagaaguauggag....................	1	1	EGG
......................................................................Gggaauguaaagaaguauggag....................	4	1	EGG
......................................................................uggaaCguaaagaaguauggag....................	1	1	EGG
......................................................................uggaauguaaagaaguauCgag....................	2	1	EGG
```

```
                              Star                                    Mature
acauuucucaaucggugcccucuaguggaucccguacuucuucacaucuccauauuucauuggauauuuauggaauguaaagaaguauggaguuuacuaauguaaaacgagg
......................................................................uggaauguaaagUaguauggag....................    5     1    EGG
......................................................................uggaauguaaagaaguauggCg....................    12    1    EGG
......................................................................uggaauguaaaAaaguauggag....................    1     1    EGG
......................................................................uggaauguaaagaaCuauggag....................    6     1    EGG
......................................................................uUgaauguaaagaaguauggag....................    2     1    EGG
......................................................................uggaauguaaagaaguaAggag....................    1     1    EGG
......................................................................uggaauguaaagaagGauggag....................    3     1    EGG
......................................................................uggaauguUaagaaguauggag....................    3     1    EGG
......................................................................uggaauguaaagaaguauggaA....................    2934  1    EGG
......................................................................uggaauguaaagaaguGuggag....................    2     1    EGG
......................................................................uggaauguaaagaaguauUgag....................    2     1    EGG
......................................................................uggaauguGaagaaguauggag....................    1     1    EGG
......................................................................uggaauguaaagaaguaCggag....................    1     1    EGG
......................................................................uggaauguaaagaaguaugAag....................    1     1    EGG
......................................................................uggaauguaaUgaaguauggag....................    1     1    EGG
......................................................................uggaauCuaaagaaguauggag....................    3     1    EGG
......................................................................uggGauguaaagaaguauggag....................    3     1    EGG
......................................................................uggaauguaaagaagAauggag....................    6     1    EGG
......................................................................uggaGuguaaagaaguauggag....................    1     1    EGG
......................................................................uggaaugGaaagaaguauggag....................    1     1    EGG
......................................................................uggaauguaaagaaguauggaU....................    371   1    EGG
......................................................................uCgaauguaaagaaguauggag....................    2     1    EGG
......................................................................uggaauguaaagCaguauggag....................    1     1    EGG
......................................................................ugAaauguaaagaaguauggagu...................    3     1    EGG
......................................................................uggaauguaaagaaguauggagG...................    6     1    EGG
......................................................................uggaauguaaagaaguauggaUu...................    14    1    EGG
......................................................................uggaauguaaagaaguauggGgu...................    1     1    EGG
......................................................................uggaauguaaagaaguauggagu...................    758   0    EGG
......................................................................uggaauguaaagaagCauggagu...................    1     1    EGG
......................................................................uggaauguaaagaaguauggagC...................    6     1    EGG
......................................................................ugUaauguaaagaaguauggagu...................    1     1    EGG
......................................................................uggaauguaaagaaguauggaCu...................    16    1    EGG
......................................................................uggaauguaaagaaguauggaAu...................    26    1    EGG
......................................................................uggaauguaaagUaguauggagu...................    1     1    EGG
......................................................................uggaauguaaagaagGauggagu...................    1     1    EGG
......................................................................uggaauguaaagaaguauggagA...................    1036  1    EGG
......................................................................uggGauguaaagaaguauggagu...................    1     1    EGG
......................................................................uggaauguaaagaaguauggaguC..................    1     1    EGG
......................................................................uggaauguaaagaaguauggagAu..................    14    1    EGG
......................................................................uggaauguaaagaaguauggagCu..................    2     1    EGG
......................................................................uggaauguaaagaaguauggaguu..................    22    0    EGG
......................................................................uggaauguaaagaaguauggaAuu..................    2     1    EGG
......................................................................uggaauguaaagaaguauggaguA..................    7     1    EGG
......................................................................uggaauguaaagaaguauggagGu..................    2     1    EGG
......................................................................uggaauguaaagaaguauggagAuu.................    7     1    EGG
......................................................................uggaauguaaagaaguauggagGuu.................    1     1    EGG
......................................................................uggaauguaaagaaguauggaguAu.................    1     1    EGG
......................................................................uggaauguaaagaaguauggaguuu.................    1     0    EGG
.......................................................................ggaauguaaagaaguaug.......................    3     0    EGG
.......................................................................ggaauguaaagaaguaugg......................    9     0    EGG
.......................................................................ggaauguCaagaaguaugg......................    1     1    EGG
.......................................................................ggGauguaaagaaguaugga.....................    1     1    EGG
.......................................................................Ugaauguaaagaaguaugga.....................    8     1    EGG
.......................................................................gAaauguaaagaaguaugga.....................    1     1    EGG
.......................................................................ggaauguaaagaaguaugga.....................    68    0    EGG
.......................................................................ggaauguaaagaaguauggaA....................    3     1    EGG
.......................................................................ggaauguaaagaaguauggaU....................    1     1    EGG
.......................................................................ggaauguaaagaaguauggag....................    46    0    EGG
.......................................................................Ugaauguaaagaaguauggag....................    6     1    EGG
.......................................................................gUaauguaaagaaguauggag....................    1     1    EGG
.......................................................................gAaauguaaagaaguauggag....................    1     1    EGG
.......................................................................ggaauguaaagaaguauggagA...................    4     1    EGG
.......................................................................ggaauguaaagaaguauggagu...................    2     0    EGG
........................................................................Aaauguaaagaaguaugg......................    1     1    EGG
........................................................................gaauguaaagaaguaugg......................    9     0    EGG
........................................................................gaauguaaagaaguaugga.....................    90    0    EGG
........................................................................Uaauguaaagaaguaugga.....................    1     1    EGG
........................................................................Aaauguaaagaaguaugga.....................    3     1    EGG
........................................................................Caauguaaagaaguaugga.....................    1     1    EGG
........................................................................gaauguaGagaaguauggag....................    1     1    EGG
```

Star **Mature**

acauuucucaaucggugcccucuaguggaucccguacuucuucacaucuccauauuucauuggauauuuauggaauguaaagaaguauggaguuuacuaauguaaaacgagg			
....gaauguaaagaaguauggA.....................	5	1	EGG
....gaauguaaagaaguauggU.....................	2	1	EGG
....Uaauguaaagaaguauggag....................	2	1	EGG
....gaauguaaagaaguauggag....................	74	0	EGG
....gaauguaaagaaguauggaUu...................	1	1	EGG
....gaauguaaagaaguauggaCu...................	2	1	EGG
....gaauguaaagaaguauggagu...................	88	0	EGG
....gaauguaaagaaguauggagA...................	29	1	EGG
....gaauguaaagaaguauggagC...................	3	1	EGG
....gaauguaaagaaguauggaguu..................	25	0	EGG
....gaauguaaagaaguauggaguA..................	21	1	EGG
....gaauguaaagaaguauggagAu..................	4	1	EGG
....gaauguaaagaaguauggaguuu.................	1	0	EGG
....aauguaaagaaguaugga.....................	113	0	EGG
....aauguaaGgaaguaugga.....................	1	1	EGG
....aauguaaagaaguauggag....................	94	0	EGG
....aauguaaagaaguauggaA....................	9	1	EGG
....aauguaaagaaguauggaU....................	5	1	EGG
....aauguaaagaaguauggagA...................	7	1	EGG
....aauguaaagaaguauggagu...................	6	0	EGG
....auguaaagaaguauggaU....................	2	1	EGG
....auguaaagaaguauggag....................	119	0	EGG
....auguaaagaaguauggagA...................	5	1	EGG
....auguaaagaaguauggagu...................	3	0	EGG
....auguaaagaaguauggaguu..................	1	0	EGG
....auguaaagaaguauggagAu..................	1	1	EGG
....uguaaagaaguauggagA...................	4	1	EGG
....uguaaagaaguauggagu...................	5	0	EGG
....guaaagaaguauggaguu..................	1	0	EGG
....guaaagaaguauggagCu..................	1	1	EGG
....guaaagaaguauggagAuu.................	1	1	EGG
....uucucaaucggugcccucuagug...	1	0	HS4
........caaucggugcccucuaguggauc...	1	0	HS4
.........aaucggugcccucuaguggauc...	1	0	HS4
..........aucggugcccucuaguggauc...	1	0	HS4
...........ucggugcccucuaguggauc...	2	0	HS4
...........................gaucccCuacuucuucacaucu...	1	1	HS4
............................aucccguacuucuucacaucucc...	1	0	HS4
.............................ucccguacuucuucacaucuc..	1	0	HS4
.............................uUccguacuucuucacaucuccaua..	1	1	HS4
..............................cccguacuucuucacaucuc..	1	0	HS4
..............................cccguacuucuucacaucucc...	9	0	HS4
..............................Uccguacuucuucacaucucca..	1	1	HS4
..............................cccguacuucuucacaucucca..	117	0	HS4
..............................cccguacuucuucacaucuccau...	55	0	HS4
..............................cccguacuucuucacaucCccau...	1	1	HS4
..............................cccguaUuucuucacaucuccau...	1	1	HS4
..............................Uccguacuucuucacaucuccaua..	2	1	HS4
..............................cccguacuucuucacaucuccauU..	18	1	HS4
..............................cccguacuucuucacaucuccaua..	37	0	HS4
..............................cccguacuucuucacaucuccauau...	3	0	HS4
..............................cccguacuucuucacaucuccauUu...	4	1	HS4
...............................ccguacuucuucacaucA...	2	1	HS4
...............................ccguacuucuucacaucu...	2	0	HS4
...............................ccguacuucuucacaucuc..	3	0	HS4
...............................ccguacuucuucacaucucc...	19	0	HS4
...............................ccguacGucuucacaucucca..	4	1	HS4
...............................ccguacuucuucacaucucca..	1517	0	HS4
...............................ccguacuucuucacaucCcca..	1	1	HS4
...............................ccguacCucuucacaucucca..	1	1	HS4
...............................Gcguacuucuucacaucucca..	1	1	HS4
...............................ccguacuucuucacaucAcca..	1	1	HS4
...............................ccguacuuUuucacaucucca..	1	1	HS4
...............................ccguacuucuucaUaucucca..	1	1	HS4
...............................ccguacuucuucacaucucUau...	1	1	HS4
...............................ccguUcuucuucacaucuccau...	1	1	HS4
...............................ccguacCucuucacaucuccau...	1	1	HS4
...............................ccguacuucuucacaucuccaA...	4	1	HS4
...............................ccguacuucuucacaucuccau...	473	0	HS4

```
                                 Star                                   Mature
acauuucucaaucggugcccucuaguggaucccguacuucuucacaucuccauauuucauuggauauuuauggaauguaaagaaguauggaguuuacuaauguaaaacgagg
...............................cAguacuucuucacaucuccau...........................................................    1     1    HS4
...............................ccguacuucuucacaucuUcau...........................................................    1     1    HS4
...............................ccguacAucuucacaucuccau...........................................................    1     1    HS4
...............................ccguacGucuucacaucuccau...........................................................    2     1    HS4
...............................ccguacuucGucacaucuccau...........................................................    1     1    HS4
...............................ccguacuucuucacaucuccauG..........................................................    4     1    HS4
...............................ccguacuuAuucacaucuccaua..........................................................    1     1    HS4
...............................ccguacuuUuucacaucuccaua..........................................................    2     1    HS4
...............................ccguacCucuucacaucuccaua..........................................................    1     1    HS4
...............................ccguacuucuucacaucucGaua..........................................................    1     1    HS4
...............................ccguacuucuucacaucuUcaua..........................................................    5     1    HS4
...............................ccguacAucuucacaucuccaua..........................................................    1     1    HS4
...............................ccguacuucAucacaucuccaua..........................................................    1     1    HS4
...............................ccguacuucuucacaucuccauU..........................................................    249   1    HS4
...............................ccguacuucuucacUucuccaua..........................................................    1     1    HS4
...............................ccguUcuucuucacaucuccaua..........................................................    1     1    HS4
...............................ccguacuucuuUacaucuccaua..........................................................    1     1    HS4
...............................ccguacGucuucacaucuccaua..........................................................    1     1    HS4
...............................ccguacuucuucacaucCccaua..........................................................    1     1    HS4
...............................ccguacuucuucacaAcuccaua..........................................................    3     1    HS4
...............................ccguacuucuucacaucuccauC..........................................................    3     1    HS4
...............................cGguacuucuucacaucuccaua..........................................................    1     1    HS4
...............................ccguacuucuucacaucuccaua..........................................................    2764  0    HS4
...............................ccguacuucuucacaucucUaua..........................................................    1     1    HS4
...............................ccguacuucuucacauUuccaua..........................................................    5     1    HS4
...............................ccguacuucuucacaucuGcaua..........................................................    1     1    HS4
...............................ccguacuucuucaGaucuccaua..........................................................    1     1    HS4
...............................ccguacuAcuucacaucuccaua..........................................................    1     1    HS4
...............................ccguGcuucuucacaucuccaua..........................................................    2     1    HS4
...............................ccguacuucuucacaucuccauUu.........................................................    10    1    HS4
...............................ccguacuucuucacaucuccauaA.........................................................    43    1    HS4
...............................ccguacuucuucacaucuccauaC.........................................................    1     1    HS4
...............................ccguacuucuucacaucuccauau.........................................................    19    0    HS4
...............................ccguacuucuucacaucuccauauA........................................................    4     1    HS4
...............................ccguacuucuucacaucuccauaAu........................................................    1     1    HS4
...............................ccguacuucuucacaucuccauauuuU......................................................    1     1    HS4
................................cguacuucuucacaucucca............................................................    1     0    HS4
................................cCuacuucuucacaucucca............................................................    1     1    HS4
................................cguacuucuucacaucuccau...........................................................    1     0    HS4
................................cguacuucuucacaucuccaua..........................................................    10    0    HS4
..................................uacuucuucacaucuccaua...........................................................    1     0    HS4
.................................................................auuuauggaauguaaagaagu..........................    2     0    HS4
.................................................................auuuauggaauguaaagaagua.........................    3     0    HS4
..................................................................uuuauggaauguaaagaagua.........................    5     0    HS4
...................................................................uuauggaauguaaagaagua.........................    9     0    HS4
...................................................................uuauggaauguaaagaaguau........................    2     0    HS4
...................................................................uuauggaauguaaagaaguaug.......................    13    0    HS4
...................................................................uuauggaauguaaagaaguaugg......................    1     0    HS4
...................................................................uuauggaauguaaagaaguaugga.....................    1     0    HS4
...................................................................uuUuggaauguaaagaaguauggag....................    1     1    HS4
...................................................................uuauggaauguaaagaaguauggag....................    1     0    HS4
...................................................................uuauggaauguaaagaaguauggagA...................    2     1    HS4
....................................................................uauggaauguaaagaaguau........................    1     0    HS4
....................................................................uauggaauguaaagaaguaug.......................    7     0    HS4
....................................................................uUuggaauguaaagaaguaug.......................    1     1    HS4
....................................................................uauggaauguaaagaaguaugg......................    4     0    HS4
....................................................................uUuggaauguaaagaaguaugga.....................    5     1    HS4
....................................................................uUuggaauguaaagaaguauggag....................    85    1    HS4
....................................................................uauggaauguaaagaaguauggag....................    4     0    HS4
....................................................................Cauggaauguaaagaaguauggag....................    2     1    HS4
....................................................................Aauggaauguaaagaaguauggag....................    1     1    HS4
....................................................................uUuggaauguaaagaaguauggagu...................    9     1    HS4
....................................................................uauggaauguaaagaaguauggagA...................    1     1    HS4
.....................................................................auggaauguaaagaaguau........................    1     0    HS4
.....................................................................Uuggaauguaaagaaguaug.......................    3     1    HS4
.....................................................................auggaauguaaagaaguaugg......................    34    0    HS4
.....................................................................Uuggaauguaaagaaguaugg......................    18    1    HS4
.....................................................................auUgaauguaaagaaguaugg......................    1     1    HS4
.....................................................................Uuggaauguaaagaaguaugga.....................    65    1    HS4
.....................................................................auggaauguaaagaaUuaugga.....................    1     1    HS4
```

Star **Mature**

```
acauuucucaaucggugcccucuaguggaucccguacuucuucacaucuccauauuucauuggauauuuauggaauguaaagaaguauggaguuuacuaauguaaaacgagg
.....................................................................auggaaGguaaagaaguaugga.....................   1     1   HS4
.....................................................................Cuggaauguaaagaaguaugga.....................   3     1   HS4
.....................................................................auggaauguaaagaaguauggU.....................   5     1   HS4
.....................................................................auggaauguaaagaaguaugga.....................   51    0   HS4
.....................................................................Guggaauguaaagaaguaugga.....................   1     1   HS4
.....................................................................auggaauguaaagaaguauggC.....................   2     1   HS4
.....................................................................Uuggaauguaaagaaguauggag....................   1010  1   HS4
.....................................................................Cuggaauguaaagaaguauggag....................   23    1   HS4
.....................................................................auggaauguaaagaaguaugAag....................   1     1   HS4
.....................................................................auggaauguaaagaaguauggaU....................   1     1   HS4
.....................................................................auggaauguaaagaaguauggaA....................   2     1   HS4
.....................................................................Guggaauguaaagaaguauggag....................   3     1   HS4
.....................................................................auggaauguaaagaaguauggag....................   53    0   HS4
.....................................................................auggaauguaaagaaguauggagA...................   8     1   HS4
.....................................................................auggaauguaaagaaguauggagu...................   3     0   HS4
.....................................................................Uuggaauguaaagaaguauggagu...................   70    1   HS4
.....................................................................Uuggaauguaaagaaguauggaguu..................   1     1   HS4
......................................................................uggaauguaaaCaaguau........................   1     1   HS4
......................................................................uggaauguaaagaaguaA........................   5     1   HS4
......................................................................Aggaauguaaagaaguau........................   1     1   HS4
......................................................................uggaauguaaagaCguau........................   1     1   HS4
......................................................................uggaauCuaaagaaguau........................   1     1   HS4
......................................................................uggUauguaaagaaguau........................   1     1   HS4
......................................................................ugCaauguaaagaaguau........................   1     1   HS4
......................................................................uggaauguaaagGaguau........................   1     1   HS4
......................................................................uggaauguaaagaaCuau........................   1     1   HS4
......................................................................uggaauguaUagaaguau........................   1     1   HS4
......................................................................uggaauguaaGgaaguau........................   2     1   HS4
......................................................................uggaauguaaagaaguaG........................   16    1   HS4
......................................................................uggaauUuaaagaaguau........................   1     1   HS4
......................................................................uggaaCguaaagaaguau........................   1     1   HS4
......................................................................uCgaauguaaagaaguau........................   1     1   HS4
......................................................................uggaauguaaagaaguau........................   1470  0   HS4
......................................................................uggGauguaaagaaguau........................   1     1   HS4
......................................................................uggaauguaaagUaguau........................   1     1   HS4
......................................................................ugAaauguaaagaaguau........................   1     1   HS4
......................................................................uAgaauguaaagaaguaug.......................   3     1   HS4
......................................................................uggaauguaaagaaguaug.......................   5262  0   HS4
......................................................................uggaauguaaagaagAaug.......................   2     1   HS4
......................................................................uggaauguaaUgaaguaug.......................   2     1   HS4
......................................................................uggaauguaaagGaguaug.......................   1     1   HS4
......................................................................uggaauguaaagaaguauA.......................   8     1   HS4
......................................................................uggaauguaaaAaaguaug.......................   2     1   HS4
......................................................................uggaauguaaagaGguaug.......................   4     1   HS4
......................................................................uggaauguaaagUaguaug.......................   2     1   HS4
......................................................................uggaauguaCagaaguaug.......................   1     1   HS4
......................................................................uggaauguaaagaaguCug.......................   1     1   HS4
......................................................................uggaauguaaagaaAuaug.......................   2     1   HS4
......................................................................uggaGuguaaagaaguaug.......................   3     1   HS4
......................................................................uggaauguaaagaaUuaug.......................   1     1   HS4
......................................................................uggaauguaaagaagCaug.......................   2     1   HS4
......................................................................Cggaauguaaagaaguaug.......................   1     1   HS4
......................................................................uggaauguaaagaaguaAg.......................   1     1   HS4
......................................................................Gggaauguaaagaaguaug.......................   2     1   HS4
......................................................................uggaauguaaagaaguaGg.......................   2     1   HS4
......................................................................uggaauguaaaCaaguaug.......................   3     1   HS4
......................................................................uggaauguaaagaCguaug.......................   1     1   HS4
......................................................................uggaauguaaagaaCuaug.......................   3     1   HS4
......................................................................uCgaauguaaagaaguaug.......................   1     1   HS4
......................................................................uggaauUuaaagaaguaug.......................   3     1   HS4
......................................................................uggaauCuaaagaaguaug.......................   3     1   HS4
......................................................................uggUauguaaagaaguaug.......................   6     1   HS4
......................................................................uggaauguaaagaaguauU.......................   5     1   HS4
......................................................................uggaauguaGagaaguaug.......................   1     1   HS4
......................................................................Aggaauguaaagaaguaug.......................   1     1   HS4
......................................................................uggaauguaaagaUguaug.......................   1     1   HS4
......................................................................ugCaauguaaagaaguaug.......................   4     1   HS4
......................................................................uggaauguaaagaaguUug.......................   1     1   HS4
......................................................................ugAaauguaaagaaguaug.......................   3     1   HS4
......................................................................uggaauAuaaagaaguaug.......................   2     1   HS4
```

```
                              Star                                    Mature
acauuucucaaucggugcccucuaguggaucccguacuucuucacaucuccauauuucauuggauauuuauggaauguaaagaaguauggaguuuacuaauguaaaacgagg
......................................................................uggGauguaaagaaguaug.......................    1        1    HS4
......................................................................Gggaauguaaagaaguaugg......................    9        1    HS4
......................................................................uggaauguaaagaaguaAgg......................    2        1    HS4
......................................................................uggaauguaaagUaguaugg......................    33       1    HS4
......................................................................uAgaauguaaagaaguaugg......................    9        1    HS4
......................................................................uggaaugAaaagaaguaugg......................    3        1    HS4
......................................................................uggaaugGaaagaaguaugg......................    1        1    HS4
......................................................................uggGauguaaagaaguaugg......................    15       1    HS4
......................................................................ugCaauguaaagaaguaugg......................    79       1    HS4
......................................................................uggaauguaaUgaaguaugg......................    20       1    HS4
......................................................................uggaauguaaCgaaguaugg......................    6        1    HS4
......................................................................uggaauguaaaUaaguaugg......................    39       1    HS4
......................................................................uggaauAuaaagaaguaugg......................    34       1    HS4
......................................................................uggaauguaaagaagAaugg......................    21       1    HS4
......................................................................uggaauguaaGgaaguaugg......................    10       1    HS4
......................................................................ugAaauguaaagaaguaugg......................    33       1    HS4
......................................................................uggaauguaaaCaaguaugg......................    42       1    HS4
......................................................................uggaauguaaaAaaguaugg......................    9        1    HS4
......................................................................uggaauguaaagaagGaugg......................    17       1    HS4
......................................................................uggaUuguaaagaaguaugg......................    7        1    HS4
......................................................................uggCauguaaagaaguaugg......................    1        1    HS4
......................................................................uggaauguaaagaaguGugg......................    10       1    HS4
......................................................................uggaaugCaaagaaguaugg......................    1        1    HS4
......................................................................uggaauguaGagaaguaugg......................    14       1    HS4
......................................................................uggaauguaaagaaguauAg......................    65       1    HS4
......................................................................uggaauguaaagCaguaugg......................    7        1    HS4
......................................................................uggaauguCaagaaguaugg......................    1        1    HS4
......................................................................uggaauguaaagaUguaugg......................    20       1    HS4
......................................................................uggaauguaaagGaguaugg......................    7        1    HS4
......................................................................uggaauguaaagaCguaugg......................    22       1    HS4
......................................................................uggaauguaaagaaguauCg......................    9        1    HS4
......................................................................uggaauguaaagaagCaugg......................    14       1    HS4
......................................................................uggaauguaaagaaguaugU......................    61       1    HS4
......................................................................Cggaauguaaagaaguaugg......................    12       1    HS4
......................................................................uggaauguaaagaaguauUg......................    3        1    HS4
......................................................................Aggaauguaaagaaguaugg......................    31       1    HS4
......................................................................ugUaauguaaagaaguaugg......................    44       1    HS4
......................................................................uggaauguaCagaaguaugg......................    4        1    HS4
......................................................................uggUauguaaagaaguaugg......................    138      1    HS4
......................................................................uCgaauguaaagaaguaugg......................    35       1    HS4
......................................................................uggaGuguaaagaaguaugg......................    26       1    HS4
......................................................................uggaauguaaagaaguaugC......................    19       1    HS4
......................................................................uggaauguUaagaaguaugg......................    2        1    HS4
......................................................................uggaauguaaagaaguUugg......................    13       1    HS4
......................................................................uggaauguaaagaaUuaugg......................    34       1    HS4
......................................................................uggaauguaaagaaguaugg......................    79901    0    HS4
......................................................................uggaauguaaagaaguaugA......................    231      1    HS4
......................................................................uggaauCuaaagaaguaugg......................    47       1    HS4
......................................................................uggaauguaaagaaAuaugg......................    17       1    HS4
......................................................................uUgaauguaaagaaguaugg......................    15       1    HS4
......................................................................uggaauguaUagaaguaugg......................    14       1    HS4
......................................................................uggaauguaaagaaguaGgg......................    23       1    HS4
......................................................................uggaauguaaagaaguaCgg......................    5        1    HS4
......................................................................uggaaCguaaagaaguaugg......................    1        1    HS4
......................................................................uggaauguaaagaaCuaugg......................    54       1    HS4
......................................................................uggaauguaaagaGguaugg......................    35       1    HS4
......................................................................uggaauUuaaagaaguaugg......................    39       1    HS4
......................................................................uggaauguaGagaaguaugga.....................    45       1    HS4
......................................................................uggaaAguaaagaaguaugga.....................    1        1    HS4
......................................................................uggaauguGaagaaguaugga.....................    15       1    HS4
......................................................................uggaauguaaagaaguaugga.....................    313868   0    HS4
......................................................................uggUauguaaagaaguaugga.....................    521      1    HS4
......................................................................uggaauUuaaagaaguaugga.....................    119      1    HS4
......................................................................uggaauCuaaagaaguaugga.....................    235      1    HS4
......................................................................uggaUuguaaagaaguaugga.....................    22       1    HS4
......................................................................uggaauguaaaAaaguaugga.....................    32       1    HS4
......................................................................uggaauguaaagaaguGugga.....................    19       1    HS4
......................................................................ugAaauguaaagaaguaugga.....................    123      1    HS4
......................................................................uggCauguaaagaaguaugga.....................    13       1    HS4
......................................................................uggaauguaaagaaguauCga.....................    31       1    HS4
```

Star Mature

```
acauuucucaaucggugcccucuaguggaucccguacuucuucacaucuccauauuucauuggauauuuauggaauguaaagaaguauggaguuuacuaauguaaaacgagg
......................................................................uggaauguaaagaaCuaugga.....................   105     1   HS4
......................................................................uCgaauguaaagaaguaugga.....................   102     1   HS4
......................................................................uggaauAuaaagaaguaugga.....................   134     1   HS4
......................................................................uggaauguaaagaaguaugUa.....................   138     1   HS4
......................................................................uggaauguaaagaagGaugga.....................   62      1   HS4
......................................................................Cggaauguaaagaaguaugga.....................   30      1   HS4
......................................................................uggaauguaaagaaguaCgga.....................   11      1   HS4
......................................................................uggaauguCaagaaguaugga.....................   2       1   HS4
......................................................................uggaauguaaagaCguaugga.....................   89      1   HS4
......................................................................uggaauguaaagaaguauggC.....................   89      1   HS4
......................................................................uggaauguaaagaaguauUga.....................   18      1   HS4
......................................................................uggaauguaaagaaguauggU.....................   237     1   HS4
......................................................................uggaauguaaagaUguaugga.....................   53      1   HS4
......................................................................uggaauguaaagaaguUugga.....................   34      1   HS4
......................................................................uUgaauguaaagaaguaugga.....................   63      1   HS4
......................................................................uggaaCguaaagaaguaugga.....................   11      1   HS4
......................................................................uggaauguaaagaaUuaugga.....................   69      1   HS4
......................................................................uggaauguUaagaaguaugga.....................   7       1   HS4
......................................................................uggaaugGaaagaaguaugga.....................   4       1   HS4
......................................................................Aggaauguaaagaaguaugga.....................   152     1   HS4
......................................................................uggGauguaaagaaguaugga.....................   59      1   HS4
......................................................................uggaaugCaaagaaguaugga.....................   12      1   HS4
......................................................................uggaauguaaagaagAaugga.....................   73      1   HS4
......................................................................uggaauguaaagaagCaugga.....................   68      1   HS4
......................................................................uggaauguaaagaGguaugga.....................   100     1   HS4
......................................................................uggaCuguaaagaaguaugga.....................   2       1   HS4
......................................................................uggaauguaaGgaaguaugga.....................   26      1   HS4
......................................................................uggaauguaaaCaaguaugga.....................   106     1   HS4
......................................................................uggaauguaaagaaguauAga.....................   26      1   HS4
......................................................................uggaauguaCagaaguaugga.....................   31      1   HS4
......................................................................uAgaauguaaagaaguaugga.....................   31      1   HS4
......................................................................ugUaauguaaagaaguaugga.....................   146     1   HS4
......................................................................uggaauguaaagaaguaugAa.....................   145     1   HS4
......................................................................uggaauguaaaUaaguaugga.....................   113     1   HS4
......................................................................uggaauguaaagaaguauggG.....................   361     1   HS4
......................................................................uggaauguaUagaaguaugga.....................   77      1   HS4
......................................................................uggaaugAaaagaaguaugga.....................   6       1   HS4
......................................................................uggaauguaaagCaguaugga.....................   18      1   HS4
......................................................................uggaGuguaaagaaguaugga.....................   84      1   HS4
......................................................................Gggaauguaaagaaguaugga.....................   65      1   HS4
......................................................................uggaauguaaagaaguCugga.....................   1       1   HS4
......................................................................ugCaauguaaagaaguaugga.....................   318     1   HS4
......................................................................uggaauguaaCgaaguaugga.....................   22      1   HS4
......................................................................uggaaGguaaagaaguaugga.....................   3       1   HS4
......................................................................uggaauguaaagaaguaugCa.....................   77      1   HS4
......................................................................uggaauguaaagaaAuaugga.....................   32      1   HS4
......................................................................uggaauguaaUgaaguaugga.....................   57      1   HS4
......................................................................uggaauguaaagUaguaugga.....................   61      1   HS4
......................................................................uggaauguaaagGaguaugga.....................   33      1   HS4
......................................................................uggaauguaaagaaguaGgga.....................   3       1   HS4
......................................................................uggaauguaaagaaguauggaA....................   17178   1   HS4
......................................................................uggaauguaaagaaguauggGg....................   1315    1   HS4
......................................................................uggaauguaaagaGguauggag....................   1150    1   HS4
......................................................................uggaauguaaagaaguauCgag....................   834     1   HS4
......................................................................uggaauguaCagaaguauggag....................   201     1   HS4
......................................................................uggaauguaaCgaaguauggag....................   230     1   HS4
......................................................................uggaauguaaagaUguauggag....................   546     1   HS4
......................................................................uggaauguaaagaaguCuggag....................   29      1   HS4
......................................................................uggaauUuaaagaaguauggag....................   1414    1   HS4
......................................................................uggaauguUaagaaguauggag....................   47      1   HS4
......................................................................uggaauAuaaagaaguauggag....................   1333    1   HS4
......................................................................uggaauguaaagaaAuauggag....................   559     1   HS4
......................................................................uggaauguaaagaaguaCggag....................   145     1   HS4
......................................................................uggGauguaaagaaguauggag....................   565     1   HS4
......................................................................uggaauguaaagaaguaAggag....................   22      1   HS4
......................................................................uAgaauguaaagaaguauggag....................   330     1   HS4
......................................................................uggaauguaaagaaguGuggag....................   350     1   HS4
......................................................................uggaaGguaaagaaguauggag....................   50      1   HS4
......................................................................uggaauguaUagaaguauggag....................   648     1   HS4
......................................................................Cggaauguaaagaaguauggag....................   384     1   HS4
```

Star Mature

acauuucucaaucggugcccucuaguggaucccguacuucuucacaucuccauauuucauuggauauuuauggaauguaaagaaguauggaguuuacuaauguaaaacgagg

....uggaauguaaagaagCauggag....................	932	1	HS4
....uggaauguaaagaaguauggUg....................	842	1	HS4
....uggaauguaaaCaaguauggag....................	1388	1	HS4
....uggaauguCaagaaguauggag....................	7	1	HS4
....uggaauCuaaagaaguauggag....................	2403	1	HS4
....uggaauguaaaUaaguauggag....................	1123	1	HS4
....uggaauguaaagaaguauAgag....................	216	1	HS4
....uggaUuguaaagaaguauggag....................	205	1	HS4
....ugUaauguaaagaaguauggag....................	1541	1	HS4
....uggaauguaaagaaguauggaC....................	15830	1	HS4
....uggaauguaaagaCguauggag....................	973	1	HS4
....uggaauguaaaAaaguauggag....................	276	1	HS4
....ugAaauguaaagaaguauggag....................	1191	1	HS4
....uggaauguaaagaaguaugCag....................	1100	1	HS4
....uggaaAguaaagaaguauggag....................	38	1	HS4
....uggaauguaaagaaguauUgag....................	736	1	HS4
....uggaaugGaaagaaguauggag....................	45	1	HS4
....uggaauguaGagaaguauggag....................	462	1	HS4
....uggaauguaaagaaguaGggag....................	63	1	HS4
....uggaauguaaagUaguauggag....................	757	1	HS4
....uggaauguaaagGaguauggag....................	405	1	HS4
....uggaauguaaagaaguauggCg....................	652	1	HS4
....uggaauguaaagaagAauggag....................	840	1	HS4
....uggCauguaaagaaguauggag....................	167	1	HS4
....uggaaugCaaagaaguauggag....................	119	1	HS4
....uggaauguaaagaagGauggag....................	904	1	HS4
....uggUauguaaagaaguauggag....................	5347	1	HS4
....uggaauguGaagaaguauggag....................	143	1	HS4
....uggaauguaaagaaguUuggag....................	403	1	HS4
....uUgaauguaaagaaguauggag....................	558	1	HS4
....uggaCuguaaagaaguauggag....................	21	1	HS4
....uggaauguaaagaaguauggag....................	3281873	0	HS4
....uggaauguaaagCaguauggag....................	171	1	HS4
....uggaauguaaUgaaguauggag....................	758	1	HS4
....uCgaauguaaagaaguauggag....................	1189	1	HS4
....uggaauguaaagaaguauggaU....................	13209	1	HS4
....uggaauguaaagaaCuauggag....................	1954	1	HS4
....Gggaauguaaagaaguauggag....................	547	1	HS4
....uggaauguaaagaaUuauggag....................	1414	1	HS4
....uggaauguaaagaaguaugAag....................	955	1	HS4
....uggaGuguaaagaaguauggag....................	978	1	HS4
....ugCaauguaaagaaguauggag....................	3234	1	HS4
....uggaaCguaaagaaguauggag....................	128	1	HS4
....uggaauguaaagaaguaugUag....................	1153	1	HS4
....uggaaugAaaagaaguauggag....................	86	1	HS4
....uggaauguaaGgaaguauggag....................	272	1	HS4
....Aggaauguaaagaaguauggag....................	1654	1	HS4
....uggaauguaaagaaguaCggagu...................	10	1	HS4
....uggaauguaaagCaguauggagu...................	19	1	HS4
....ugUaauguaaagaaguauggagu...................	124	1	HS4
....uggaauguaaagaaguauggGgu...................	85	1	HS4
....uggaauguaaagaaguaugCagu...................	137	1	HS4
....uggaaugAaaagaaguauggagu...................	15	1	HS4
....uggaCuguaaagaaguauggagu...................	4	1	HS4
....Aggaauguaaagaaguauggagu...................	107	1	HS4
....uggaauguaaGgaaguauggagu...................	22	1	HS4
....uggaauguGaagaaguauggagu...................	5	1	HS4
....uggaauguaaagaaguauggCgu...................	57	1	HS4
....uUgaauguaaagaaguauggagu...................	44	1	HS4
....Gggaauguaaagaaguauggagu...................	56	1	HS4
....uggaauguaaagaaguGuggagu...................	24	1	HS4
....uggGauguaaagaaguauggagu...................	34	1	HS4
....uggaauguaaagaaguaGggagu...................	4	1	HS4
....uggaauguaCagaaguauggagu...................	11	1	HS4
....uggaauguaaagaaguauAgagu...................	14	1	HS4
....uggaauguaaagaaAuauggagu...................	34	1	HS4
....uggaauguaaagaaguauggaCu...................	767	1	HS4
....uggaauguaaagGaguauggagu...................	27	1	HS4
....uggaauguaaagaaguaugUagu...................	104	1	HS4
....uggaauguaaagaaUuauggagu...................	141	1	HS4

```
                              Star                                      Mature
acauuucucaaucggugcccucuaguggauccguacuucuucacaucuccauauuucauuggauauuuauggaauguaaagaaguauggaguuuacuaauguaaaacgagg
......................................................................uggaauguaaagaaguauCgagu...................   78       1   HS4
......................................................................uggaauguaaagaagCauggagu...................   80       1   HS4
......................................................................uggaauguaaagUaguauggagu...................   52       1   HS4
......................................................................uggaaugGaaagaaguauggagu...................   3        1   HS4
......................................................................ugAaauguaaagaaguauggagu...................   95       1   HS4
......................................................................uggaaCguaaagaaguauggagu...................   7        1   HS4
......................................................................uggaauguaaagaaguauggagG...................   1234     1   HS4
......................................................................uAgaauguaaagaaguauggagu...................   23       1   HS4
......................................................................uggaauAuaaagaaguauggagu...................   116      1   HS4
......................................................................uggaauguaaagaaguauggaAu...................   206      1   HS4
......................................................................uggaauguaaagaaguauggagu...................   263910   0   HS4
......................................................................uggaauguaaagaaguauggUgu...................   101      1   HS4
......................................................................uggaauguaaagaGguauggagu...................   97       1   HS4
......................................................................uggaauguaaagaagAauggagu...................   64       1   HS4
......................................................................uggaauguaaagaaguaAggagu...................   1        1   HS4
......................................................................uggaauguaaagaaguCuggagu...................   3        1   HS4
......................................................................uggaauguaUagaaguauggagu...................   55       1   HS4
......................................................................uggaaAguaaagaaguauggagu...................   1        1   HS4
......................................................................uggaauguaaagaaguauUgagu...................   65       1   HS4
......................................................................uggaGuguaaagaaguauggagu...................   79       1   HS4
......................................................................uCgaauguaaagaaguauggagu...................   110      1   HS4
......................................................................uggaUuguaaagaaguauggagu...................   18       1   HS4
......................................................................uggaauguaaagaaguauggagC...................   1402     1   HS4
......................................................................uggaauguaaagaagGauggagu...................   88       1   HS4
......................................................................uggaauguaaUgaaguauggagu...................   66       1   HS4
......................................................................uggaaGguaaagaaguauggagu...................   5        1   HS4
......................................................................uggaauguaaaAaaguauggagu...................   18       1   HS4
......................................................................uggaauguaaagaaCuauggagu...................   203      1   HS4
......................................................................uggaauguaaagaaguauggaUu...................   826      1   HS4
......................................................................uggaaugCaaagaaguauggagu...................   16       1   HS4
......................................................................uggaauguUaagaaguauggagu...................   3        1   HS4
......................................................................uggaauCuaaagaaguauggagu...................   214      1   HS4
......................................................................uggaauguaaagaaguUuggagu...................   34       1   HS4
......................................................................uggaauguaaagaaguaugAagu...................   60       1   HS4
......................................................................uggaauguaaagaUguauggagu...................   41       1   HS4
......................................................................uggaauguaaaUaaguauggagu...................   90       1   HS4
......................................................................uggUauguaaagaaguauggagu...................   483      1   HS4
......................................................................uggaauguaaCgaaguauggagu...................   34       1   HS4
......................................................................ugCaauguaaagaaguauggagu...................   281      1   HS4
......................................................................uggaauguaaagaaguauggagA...................   242235   1   HS4
......................................................................Cggaauguaaagaaguauggagu...................   46       1   HS4
......................................................................uggaauguaaaCaaguauggagu...................   87       1   HS4
......................................................................uggaauguaGagaaguauggagu...................   33       1   HS4
......................................................................uggCauguaaagaaguauggagu...................   23       1   HS4
......................................................................uggaauUuaaagaaguauggagu...................   142      1   HS4
......................................................................uggaauguaaagaCguauggagu...................   90       1   HS4
......................................................................uggCauguaaagaaguauggaguu..................   1        1   HS4
......................................................................uggaauguaaagaaguaugCaguu..................   4        1   HS4
......................................................................uggaauguaaagaaAuauggaguu..................   2        1   HS4
......................................................................uggaauguaaagaaguauggagCu..................   16       1   HS4
......................................................................uggaauguaaagaaguauggaguu..................   4714     0   HS4
......................................................................uggaauguaaagaaguauggaUuu..................   15       1   HS4
......................................................................ugUaauguaaagaaguauggaguu..................   2        1   HS4
......................................................................uggaauguaaagaaguauggaguG..................   5        1   HS4
......................................................................uggaauguaaagaagCauggaguu..................   4        1   HS4
......................................................................uggaauguaaagaagAauggaguu..................   1        1   HS4
......................................................................ugCaauguaaagaaguauggaguu..................   5        1   HS4
......................................................................ugAaauguaaagaaguauggaguu..................   3        1   HS4
......................................................................uggaauguaaagaaguauCgaguu..................   1        1   HS4
......................................................................uggaauguaaagaGguauggaguu..................   2        1   HS4
......................................................................uggaauguaaagGaguauggaguu..................   1        1   HS4
......................................................................uggaauguaaagaaUuauggaguu..................   1        1   HS4
......................................................................uCgaauguaaagaaguauggaguu..................   3        1   HS4
......................................................................uggaauguaaagaaguauggaAuu..................   4        1   HS4
......................................................................uggaauguaaagaaguauggaCuu..................   1        1   HS4
......................................................................uggaauguGaagaaguauggaguu..................   4        1   HS4
......................................................................uggaauguaaagaaguauggagGu..................   14       1   HS4
......................................................................Cggaauguaaagaaguauggaguu..................   1        1   HS4
......................................................................uggaauguaaagaaCuauggaguu..................   2        1   HS4
......................................................................uggaauguaaagaaguauggCguu..................   2        1   HS4
```

Star Mature

acauuucucaaucggugcccucuaguggaucccguacuucuucacaucuccauauuucauuggauauuuauggaauguaaagaaguauggaguuuacuaaauguaaaacgagg			
....uggaauguaaaCaaguauggaguu...................	1	1	HS4
....uggaGuguaaagaaguauggaguu...................	2	1	HS4
....uggaauCuaaagaaguauggaguu...................	4	1	HS4
....uggGauguaaagaaguauggaguu...................	1	1	HS4
....uggaauguaaagUaguauggaguu...................	1	1	HS4
....uggaauguaaagaagGauggaguu...................	1	1	HS4
....uggaauguaaagaaguauUgaguu...................	1	1	HS4
....uggaauguaaagaaguaugAaguu...................	3	1	HS4
....uUgaauguaaagaaguauggaguu...................	1	1	HS4
....uggaauguaaagaaguaugUaguu...................	1	1	HS4
....uggaUuguaaagaaguauggaguu...................	1	1	HS4
....Gggaauguaaagaaguauggaguu...................	1	1	HS4
....uggaauguaaagaCguauggaguu...................	3	1	HS4
....uggaauguaaagCaguauggaguu...................	1	1	HS4
....uggaauAuaaagaaguauggaguu...................	7	1	HS4
....uggaauguaCagaaguauggaguu...................	1	1	HS4
....uggUauguaaagaaguauggaguu...................	10	1	HS4
....uggaauguaaagaaguauggUguu...................	1	1	HS4
....uggaauguaaagaaguauggaguC...................	8	1	HS4
....Aggaauguaaagaaguauggaguu...................	3	1	HS4
....uggaauguaaagaaguUuggaguu...................	1	1	HS4
....uggaauguaaaUaaguauggaguu...................	1	1	HS4
....uggaauguaaagaaguauggagAu...................	878	1	HS4
....uggaauguaaagaaguauggaguA...................	416	1	HS4
....uggaauguaUagaaguauggaguuu..................	1	1	HS4
....uggaauguaaagaaguauggagCuu..................	2	1	HS4
....uggaauguaaagaaguauggaguCu..................	2	1	HS4
....uggaauguaaagaaguauggagAuu..................	69	1	HS4
....uggaauguaaagaaguauggagGuu..................	1	1	HS4
....uggaauguaaagaaguauggaguuG..................	1	1	HS4
....uggaauguaaagaaguauggaguuA..................	13	1	HS4
....uggaauguaaagaaguauggaguuu..................	203	0	HS4
....uggaauguaaagaaguauggaguAu..................	33	1	HS4
....uggUauguaaagaaguauggaguuu..................	2	1	HS4
....uggaauguaaagaaguauggaguuC..................	2	1	HS4
....uggaauguaaagaaguaugCaguuu..................	1	1	HS4
....uggaauguaaagaaguauggaUuuu..................	3	1	HS4
....uggaauguaaCgaaguauggaguuu..................	1	1	HS4
....uggaauguaaagaaguauggaguAua.................	1	1	HS4
....uggaauguaaagaaguauggaguuuU.................	28	1	HS4
....uggaauguaaagaaguauggaguuAa.................	1	1	HS4
....uggaauguaaagaaguauggaguuua.................	1	0	HS4
....ggaauguaaagaaguaug........................	3	0	HS4
....Ugaauguaaagaaguaug........................	2	1	HS4
....Ugaauguaaagaaguaugg.......................	30	1	HS4
....ggaaugAaaagaaguaugg.......................	1	1	HS4
....ggaaugCaaagaaguaugg.......................	1	1	HS4
....ggaauguaaagaaguaugg.......................	100	0	HS4
....Cgaauguaaagaaguaugg.......................	3	1	HS4
....ggaauguaaagaaguauggU......................	1	1	HS4
....Cgaauguaaagaaguaugga......................	8	1	HS4
....gUaauguaaagaaguaugga......................	4	1	HS4
....ggaUuguaaagaaguaugga......................	3	1	HS4
....Ugaauguaaagaaguaugga......................	112	1	HS4
....ggaauguaaagaaCuaugga......................	1	1	HS4
....gAaauguaaagaaguaugga......................	5	1	HS4
....ggaCuguaaagaaguaugga......................	1	1	HS4
....Agaauguaaagaaguaugga......................	1	1	HS4
....ggaauguaaagaaguauUga......................	1	1	HS4
....ggaauguaaagaaguaugga......................	396	0	HS4
....ggaauguaaagaaguauggUg.....................	1	1	HS4
....ggaauguaaagaaguauggGg.....................	8	1	HS4
....ggaauguaaagaaguauggaA.....................	25	1	HS4
....Cgaauguaaagaaguauggag.....................	272	1	HS4
....Ugaauguaaagaaguauggag.....................	1340	1	HS4
....ggaGuguaaagaaguauggag.....................	4	1	HS4
....ggaauguaaaUaaguauggag.....................	4	1	HS4
....ggaauguaaagaUguauggag.....................	1	1	HS4
....ggGauguaaagaaguauggag.....................	1	1	HS4
....ggaauguaaaAaaguauggag.....................	2	1	HS4

```
                               Star                                        Mature
acauuucucaaucggugcccucuaguggaucccguacuucuucacaucuccauauuucauuggauauuuauggaauguaaagaaguauggaguuuacuaauguaaaacgagg
.......................................................................ggaauguaaagaagAauggag....................   1      1    HS4
.......................................................................ggaauguaaagaaguauggag....................   4345   0    HS4
.......................................................................gCaauguaaagaaguauggag....................   35     1    HS4
.......................................................................ggCauguaaagaaguauggag....................   1      1    HS4
.......................................................................ggUauguaaagaaguauggag....................   1      1    HS4
.......................................................................ggaauguaaUgaaguauggag....................   3      1    HS4
.......................................................................gAaauguaaagaaguauggag....................   64     1    HS4
.......................................................................ggaaugAaaagaaguauggag....................   1      1    HS4
.......................................................................ggaauguaaagaaguaugCag....................   6      1    HS4
.......................................................................ggaauCuaaagaaguauggag....................   1      1    HS4
.......................................................................ggaauguaaagaagCauggag....................   1      1    HS4
.......................................................................ggaaugCaaagaaguauggag....................   2      1    HS4
.......................................................................ggaauguaaagaaguauUgag....................   2      1    HS4
.......................................................................ggaauguaaagaaguauCgag....................   1      1    HS4
.......................................................................ggaauguaaagaaAuauggag....................   3      1    HS4
.......................................................................ggaauguaaagaaguaugUag....................   5      1    HS4
.......................................................................ggaauguaaagaaguauggaC....................   24     1    HS4
.......................................................................ggaauguaaagaagGauggag....................   1      1    HS4
.......................................................................gUaauguaaagaaguauggag....................   31     1    HS4
.......................................................................Agaauguaaagaaguauggag....................   24     1    HS4
.......................................................................ggaauUuaaagaaguauggag....................   1      1    HS4
.......................................................................ggaauguUaagaaguauggag....................   1      1    HS4
.......................................................................ggaauguaaagaaguaugAag....................   4      1    HS4
.......................................................................ggaauguaaagaaguauggaU....................   18     1    HS4
.......................................................................ggaauguaaagaaCuauggag....................   2      1    HS4
.......................................................................ggaUuguaaagaaguauggag....................   8      1    HS4
.......................................................................ggaaugGaaagaaguauggag....................   4      1    HS4
.......................................................................ggaauguaaagaaUuauggag....................   2      1    HS4
.......................................................................ggaauguaaaCaaguauggagu...................   1      1    HS4
.......................................................................ggaauguaaagaaguauggagG...................   2      1    HS4
.......................................................................Agaauguaaagaaguauggagu...................   2      1    HS4
.......................................................................ggaauguaaagaaguauggagC...................   13     1    HS4
.......................................................................gCaauguaaagaaguauggagu...................   3      1    HS4
.......................................................................ggaauguaaagaaguauggaUu...................   4      1    HS4
.......................................................................gAaauguaaagaaguauggagu...................   3      1    HS4
.......................................................................ggaauguaaaUaaguauggagu...................   1      1    HS4
.......................................................................ggaauguaaagaaguauggGgu...................   2      1    HS4
.......................................................................ggaauguaaagaaguauggagA...................   396    1    HS4
.......................................................................Cgaauguaaagaaguauggagu...................   35     1    HS4
.......................................................................Ugaauguaaagaaguauggagu...................   126    1    HS4
.......................................................................ggaauguaaUgaaguauggagu...................   1      1    HS4
.......................................................................ggaauguaaagaagAauggagu...................   1      1    HS4
.......................................................................ggaauguaaagaaAuauggagu...................   1      1    HS4
.......................................................................gUaauguaaagaaguauggagu...................   1      1    HS4
.......................................................................ggaauguaaagaaguauggagu...................   444    0    HS4
.......................................................................ggaauguaaagaaguauggaCu...................   4      1    HS4
.......................................................................Ugaauguaaagaaguauggaguu..................   1      1    HS4
.......................................................................ggaauguaaagaaguauggaguu..................   14     0    HS4
.......................................................................Agaauguaaagaaguauggaguu..................   1      1    HS4
.......................................................................ggaauguaaagaaguauggagAu..................   6      1    HS4
.......................................................................ggaauguaaagaaguauggaguA..................   3      1    HS4
.......................................................................Ugaauguaaagaaguauggaguuu.................   1      1    HS4
........................................................................Aaauguaaagaaguaugg......................   3      1    HS4
........................................................................gaauguaaagaaguaugg......................   62     0    HS4
........................................................................Uaauguaaagaaguaugg......................   1      1    HS4
........................................................................gaauguaaagaaguauAg......................   1      1    HS4
........................................................................Aaauguaaagaaguaugga.....................   19     1    HS4
........................................................................gaauguaaaUaaguaugga.....................   1      1    HS4
........................................................................gaauguaaagaaguaugUa.....................   1      1    HS4
........................................................................Uaauguaaagaaguaugga.....................   14     1    HS4
........................................................................gaauguaaagaaCuaugga.....................   1      1    HS4
........................................................................gaauguaaagaaguaugga.....................   274    0    HS4
........................................................................gaauguaaagCaguaugga.....................   1      1    HS4
........................................................................gaauguaaagaaguauggaU....................   13     1    HS4
........................................................................gaauguaaagCaguauggag....................   1      1    HS4
........................................................................gaaGguaaagaaguauggag....................   1      1    HS4
........................................................................gUauguaaagaaguauggag....................   2      1    HS4
........................................................................gaauguaaagaaguaCggag....................   2      1    HS4
........................................................................gaauguaaagaaguauggag....................   3887   0    HS4
........................................................................gaauguaaagaagGauggag....................   3      1    HS4
```

```
                              Star                     Mature
acauuucucaaucggugcccucuaguggaucccguacuucuucacaucuccauauuucauuggauauuuauggaauguaaagaaguauggaguuuacuaauguaaaacgagg
.......................................................................gaauguaaagaaguaugCag....................  7     1  HS4
.......................................................................gaauguaaaCaaguauggag....................  5     1  HS4
.......................................................................gaauguaaagaaguauggUg....................  1     1  HS4
.......................................................................gaauguaaagUaguauggag....................  4     1  HS4
.......................................................................gaauguCaagaaguauggag....................  1     1  HS4
.......................................................................gaauguaaagaagCauggag....................  1     1  HS4
.......................................................................gaauguaaagaaguaugAag....................  4     1  HS4
.......................................................................gGauguaaagaaguauggag....................  1     1  HS4
.......................................................................gaauguaaagaagAauggag....................  3     1  HS4
.......................................................................gaauguaaagGaguauggag....................  3     1  HS4
.......................................................................gaauguaaagaaguauggaA....................  15    1  HS4
.......................................................................gaauguaaagaaCuauggag....................  4     1  HS4
.......................................................................gaauguGaagaaguauggag....................  5     1  HS4
.......................................................................gaauguaaagaaguauggaC....................  31    1  HS4
.......................................................................gaauguaaagaaguaugUag....................  1     1  HS4
.......................................................................gaauCuaaagaaguauggag....................  4     1  HS4
.......................................................................gaauguaaaAaaguauggag....................  1     1  HS4
.......................................................................Caauguaaagaaguauggag....................  19    1  HS4
.......................................................................gaaugCaaagaaguauggag....................  1     1  HS4
.......................................................................gaauguaaaUaaguauggag....................  6     1  HS4
.......................................................................gaauguaaagaaguaAggag....................  2     1  HS4
.......................................................................gaauguaaagaCguauggag....................  1     1  HS4
.......................................................................Aaauguaaagaaguauggag....................  177   1  HS4
.......................................................................gaauguaaagaaguauggGg....................  1     1  HS4
.......................................................................Uaauguaaagaaguauggag....................  231   1  HS4
.......................................................................gaauguaaagaaguauUgag....................  1     1  HS4
.......................................................................gaauguaaagaaguauCgag....................  5     1  HS4
.......................................................................Aaauguaaagaaguauggagu...................  6     1  HS4
.......................................................................gaauguaaagaagCauggagu...................  1     1  HS4
.......................................................................gaauguaaagaagGauggagu...................  2     1  HS4
.......................................................................gaauguCaagaaguauggagu...................  1     1  HS4
.......................................................................gaauguaaagaagAauggagu...................  4     1  HS4
.......................................................................gaauCuaaagaaguauggagu...................  3     1  HS4
.......................................................................gaauguaaagaaguGuggagu...................  2     1  HS4
.......................................................................gaauguaaagaaguauggGgu...................  1     1  HS4
.......................................................................gaauguaaagaaguauCgagu...................  1     1  HS4
.......................................................................gaauguaaagGaguauggagu...................  1     1  HS4
.......................................................................gaaugGaaagaaguauggagu...................  1     1  HS4
.......................................................................gaauguaaagaaguauggaCu...................  7     1  HS4
.......................................................................Caauguaaagaaguauggagu...................  1     1  HS4
.......................................................................gaauguaaagaaguaAggagu...................  1     1  HS4
.......................................................................gaauguaaagaaguauggagA...................  1096  1  HS4
.......................................................................gaauguaaagaaguauggagC...................  27    1  HS4
.......................................................................gaauguaaagaaguauggaUu...................  16    1  HS4
.......................................................................gaaugAaaagaaguauggagu...................  1     1  HS4
.......................................................................gGauguaaagaaguauggagu...................  2     1  HS4
.......................................................................gaCuguaaagaaguauggagu...................  2     1  HS4
.......................................................................gaauguaaagaaguaugCagu...................  3     1  HS4
.......................................................................gaauguaaagaaguauggagu...................  1986  0  HS4
.......................................................................gaauguaaagaaUuauggagu...................  2     1  HS4
.......................................................................Uaauguaaagaaguauggagu...................  22    1  HS4
.......................................................................gaauUuaaagaaguauggagu...................  2     1  HS4
.......................................................................gaauguaaagUaguauggagu...................  2     1  HS4
.......................................................................gaauguGaagaaguauggagu...................  1     1  HS4
.......................................................................gaauguaaagaaguaugUagu...................  3     1  HS4
.......................................................................gaauguaaagaaguauggaguG..................  1     1  HS4
.......................................................................Uaauguaaagaaguauggaguu..................  2     1  HS4
.......................................................................gaauguaaagUaguauggaguu..................  1     1  HS4
.......................................................................gaauguaaagaaguauUgaguu..................  2     1  HS4
.......................................................................gaauguaaaAaaguauggaguu..................  1     1  HS4
.......................................................................gaauguaaagaaguauggaUuu..................  5     1  HS4
.......................................................................gaauguaaaUaaguauggaguu..................  1     1  HS4
.......................................................................gaauguaaagaaguauggaguC..................  7     1  HS4
.......................................................................gaauguaaagaaguauggaguu..................  1389  0  HS4
.......................................................................gaauguaaagaaCuauggaguu..................  1     1  HS4
.......................................................................gaauguaaagaaguaugUaguu..................  3     1  HS4
.......................................................................gaauguaaagaaguauggaguA..................  633   1  HS4
.......................................................................Aaauguaaagaaguauggaguu..................  1     1  HS4
.......................................................................gaauguaaagaaguaAggaguu..................  1     1  HS4
.......................................................................gaauguaaagaaguauggCguu..................  1     1  HS4
```

```
                             Star                                   Mature
acauuucucaaucggugcccucuaguggaucccguacuucuucacaucuccauauuucauuggauauuuauggaauguaaagaaguauggaguuuacuaauguaaaacgagg
........................................................................gaauguGaagaaguauggaguu..................   1     1   HS4
........................................................................gaauguaaagaaUuauggaguu..................   2     1   HS4
........................................................................gaauguaaagaaguaCggaguu..................   1     1   HS4
........................................................................gaauguaaagaaguauggagAu..................   112   1   HS4
........................................................................gaauguaaagaaguauggagCu..................   5     1   HS4
........................................................................gaauguaaagaaguauggaguuA.................   21    1   HS4
........................................................................gaauguaaagaaguauggaguAu.................   2     1   HS4
........................................................................gaauguaaagaaguauggagAuu.................   6     1   HS4
........................................................................gaauguaaagaaguauggaguuu.................   15    0   HS4
........................................................................gaauguaaagaaguauggaguuAa................   1     1   HS4
........................................................................gaauguaaagaaguauggaguuua................   1     0   HS4
.........................................................................aauguaaagaaguCugga.....................   1     1   HS4
.........................................................................aauguaaagaaguaugga.....................   37    0   HS4
.........................................................................Uauguaaagaaguauggag....................   1     1   HS4
.........................................................................aauguaaagaaguauggag....................   463   0   HS4
.........................................................................aauguaaagaaguauggaU....................   3     1   HS4
.........................................................................aauguaaagaaguauggaC....................   4     1   HS4
.........................................................................aauguaaagaaguauggaA....................   5     1   HS4
.........................................................................aauguaaagaaguauggagu...................   51    0   HS4
.........................................................................aauguaaagaaguauggagA...................   53    1   HS4
..........................................................................auguaaagaaguauCgag....................   1     1   HS4
..........................................................................auguaaagaaguauggag....................   370   0   HS4
..........................................................................auguaaagaaguauUgag....................   1     1   HS4
..........................................................................auguaaagaaguauggUg....................   1     1   HS4
..........................................................................Uuguaaagaaguauggag....................   1     1   HS4
..........................................................................Cuguaaagaaguauggag....................   1     1   HS4
..........................................................................auguaaagaaguaugAag....................   2     1   HS4
..........................................................................auguaaagaaguauggaC....................   8     1   HS4
..........................................................................auguaaagaaUuauggag....................   1     1   HS4
..........................................................................auCuaaagaaguauggag....................   1     1   HS4
..........................................................................auguaaagaaguauggaU....................   3     1   HS4
..........................................................................auguaaaUaaguauggag....................   1     1   HS4
..........................................................................auguaaagaaguauggagA...................   30    1   HS4
..........................................................................auguaaagaaguauggagu...................   40    0   HS4
..........................................................................auguaaagaaguauggaguA..................   1     1   HS4
...........................................................................uguaaagaaguauggagu...................   23    0   HS4
...........................................................................ugGaaagaaguauggagu...................   1     1   HS4
...........................................................................uguaaagaaguauggagA...................   28    1   HS4

.........aaucggugcccucuaguggauc.................................................................................   1     0   J17
...................cucuaguggaucccguacuucu.......................................................................   2     0   J17
............................aucccguacuucuucacaucuc..............................................................   1     0   J17
............................aucccguacuucuucacaucucca............................................................   1     0   J17
.............................ucccguacuucuucacaucucca............................................................   1     0   J17
.............................uUccguacuucuucacaucuccaua..........................................................   2     1   J17
..............................cccguacuucuucacaucuc..............................................................   1     0   J17
..............................cccguacuucuucacaucucc.............................................................   8     0   J17
..............................cccguaUuucuucacaucucca............................................................   1     1   J17
..............................cccguacuucuucacaucucUa............................................................   1     1   J17
..............................cccguacuucuucacaucucca............................................................   63    0   J17
..............................Uccguacuucuucacaucucca............................................................   2     1   J17
..............................cGcguacuucuucacaucucca............................................................   1     1   J17
..............................Uccguacuucuucacaucuccau...........................................................   1     1   J17
..............................cccCuacuucuucacaucuccau...........................................................   1     1   J17
..............................cccguacuucuucacaucuccau...........................................................   52    0   J17
..............................cccguacuucuucacauUuccau...........................................................   1     1   J17
..............................Uccguacuucuucacaucuccaua..........................................................   3     1   J17
..............................cccguacuucuucacaucuccauU..........................................................   8     1   J17
..............................cccguacuucuucacaucuccaua..........................................................   31    0   J17
..............................cccguacuucuucacaucuccauaA.........................................................   1     1   J17
..............................cccguacuucuucacaucuccauau.........................................................   1     0   J17
..............................cccguacuucuucacaucuccauUu.........................................................   1     1   J17
..............................cccguacuucuucacaucuccauauuA.......................................................   1     1   J17
...............................ccguacuucuucacaucu...............................................................   3     0   J17
...............................ccguacuucuucacaucuc..............................................................   1     0   J17
...............................ccguacuucuucacaucucc.............................................................   19    0   J17
...............................ccguacuucuucacauUucc.............................................................   1     1   J17
...............................ccguacuucuucacaucucca............................................................   738   0   J17
...............................ccUuacuucuucacaucucca............................................................   1     1   J17
...............................ccguacuucuucacaucuccC............................................................   1     1   J17
```

```
                                Star                                    Mature
acauuucucaaucggugcccucuaguggaucccguacuucuucacaucuccauauuucauuggauauuuauggaauguaaagaaguauggaguuuacuaauguaaaacgagg
...............................ccguacuucuucaUaucucca............................................................  1     1  J17
...............................ccguacuucuucacaucGcca............................................................  1     1  J17
...............................ccguacuucuucacaucuccU............................................................  1     1  J17
...............................ccguacuAcuucacaucucca............................................................  1     1  J17
...............................ccguacuucuucacaucucUa............................................................  1     1  J17
...............................ccguacuucuucacUucucca............................................................  1     1  J17
...............................ccguacuucuucacaucuccaA...........................................................  6     1  J17
...............................ccguacuuAuucacaucuccau...........................................................  1     1  J17
...............................ccguacuucuucacaucuUcau...........................................................  1     1  J17
...............................ccguacuucuucacaucuccau...........................................................  268   0  J17
...............................ccgCacuucuucacaucuccaua..........................................................  1     1  J17
...............................ccguacuucuucacaucuccaGa..........................................................  1     1  J17
...............................ccguacuucuucacaAcuccaua..........................................................  2     1  J17
...............................ccguacuuUuucacaucuccaua..........................................................  1     1  J17
...............................cUguacuucuucacaucuccaua..........................................................  1     1  J17
...............................ccguacuucuucacaucuccauC..........................................................  7     1  J17
...............................ccguacuucuucacaucGccaua..........................................................  1     1  J17
...............................ccguacuucuucacaucuccaCa..........................................................  1     1  J17
...............................ccguacuucuucacaucuUcaua..........................................................  5     1  J17
...............................ccguacuucuucacauUuccaua..........................................................  5     1  J17
...............................ccguacuucuucaAaucuccaua..........................................................  1     1  J17
...............................ccguacuucuucacCucuccaua..........................................................  2     1  J17
...............................Gcguacuucuucacaucuccaua..........................................................  1     1  J17
...............................ccguacuucuucaUaucuccaua..........................................................  3     1  J17
...............................ccguacuucuucacaucuccaAa..........................................................  6     1  J17
...............................ccguacuucuucacaucCccaua..........................................................  1     1  J17
...............................ccguUcuucuucacaucuccaua..........................................................  1     1  J17
...............................ccguaAuucuucacaucuccaua..........................................................  1     1  J17
...............................ccCuacuucuucacaucuccaua..........................................................  5     1  J17
...............................ccguacuucuucacaucuccaua..........................................................  1688  0  J17
...............................ccguacuucuucacUucuccaua..........................................................  2     1  J17
...............................ccguacuucuucacaucuccauG..........................................................  2     1  J17
...............................ccguacuucuucacaucAccaua..........................................................  1     1  J17
...............................ccguacuucuucacaucuccauU..........................................................  155   1  J17
...............................ccguacuucuucacaucuccauaC.........................................................  1     1  J17
...............................ccguacuucuucacaucuccauau.........................................................  11    0  J17
...............................ccguacuucuucacaucuccauaG.........................................................  1     1  J17
...............................ccguacuucuucacaucuccauaA.........................................................  45    1  J17
...............................ccguacuucuucacaucuccauUu.........................................................  4     1  J17
...............................ccguacuucuucacaucuccauauA........................................................  2     1  J17
................................cguacuucuucacaucuccaua..........................................................  6     0  J17
....................................cuucuucacaucuccauauuu.......................................................  1     0  J17
....................................................uauuucauuggauauuuaug........................................  3     0  J17
.................................................................auuuauggaauguaaagaaguU.........................  1     1  J17
.................................................................auuuauggaauguaaagaaguauggag....................  1     0  J17
..................................................................uuuauggaauguaaagaaguau........................  1     0  J17
..................................................................uuuUuggaauguaaagaaguauggag....................  3     1  J17
...................................................................uuauggaauguaaagaagu..........................  2     0  J17
...................................................................uuauggaauguaaagaagua.........................  4     0  J17
...................................................................uuauggaauguaaagaaguau........................  1     0  J17
...................................................................uuauggaauguaaagaaguaug.......................  6     0  J17
...................................................................uuauggaauguaaagaaguauA.......................  1     1  J17
...................................................................uuauggaauguaaagaaguaugg......................  4     0  J17
...................................................................uuauggaauguaaagaaguaugga.....................  1     0  J17
...................................................................uuUuggaauguaaagaaguaugga.....................  1     1  J17
...................................................................uGauggaauguaaagaaguauggag....................  2     1  J17
...................................................................Guauggaauguaaagaaguauggag....................  1     1  J17
...................................................................Cuauggaauguaaagaaguauggag....................  3     1  J17
...................................................................uuUuggaauguaaagaaguauggagu...................  2     1  J17
....................................................................uauggaauguaaagaaguaug.......................  6     0  J17
....................................................................uauggaauguaaagaaguaugg......................  5     0  J17
....................................................................uauggaauguaaagaaguaugga.....................  3     0  J17
....................................................................uUuggaauguaaagaaguaugga.....................  29    1  J17
....................................................................Cauggaauguaaagaaguauggag....................  2     1  J17
....................................................................Gauggaauguaaagaaguauggag....................  4     1  J17
....................................................................uauggaauguaaagaaguauggag....................  6     0  J17
....................................................................uUuggaauguaaagaaguauggag....................  249   1  J17
....................................................................Aauggaauguaaagaaguauggag....................  2     1  J17
....................................................................uUuggaauguaaagaaguauggagu...................  17    1  J17
....................................................................uauggaauguaaagaaguauggagA...................  2     1  J17
```

Star Mature

acauuucucaaucggugcccucuaguggaucccguacuucuucacaucuccauauuucauuggauauuuauggaauguaaagaaguauggaguuuacuaauguaaaacgagg			
....Uuggaauguaaagaaguau........................	1	1	J17
....Uuggaauguaaagaaguaug.......................	2	1	J17
....auggaauguaaagaaguaug.......................	3	0	J17
....auggaauguaaagaaguaugU......................	1	1	J17
....auggaauguaaagaaguaugg......................	19	0	J17
....Uuggaauguaaagaaguaugg......................	17	1	J17
....Cuggaauguaaagaaguaugg......................	4	1	J17
....auggaauguaaagaaguauggU.....................	2	1	J17
....Cuggaauguaaagaaguaugga.....................	7	1	J17
....auggaauguaaagaaguaugga.....................	72	0	J17
....Uuggaauguaaagaaguaugga.....................	142	1	J17
....auggaauguaaagaaguauggC.....................	1	1	J17
....Guggaauguaaagaaguaugga.....................	2	1	J17
....aGggaauguaaagaaguauggag....................	1	1	J17
....Uuggaauguaaagaaguauggag....................	1327	1	J17
....Cuggaauguaaagaaguauggag....................	72	1	J17
....auggaauguaaagaaguauggaA....................	1	1	J17
....Guggaauguaaagaaguauggag....................	14	1	J17
....auggaauguaaagaaguauggag....................	93	0	J17
....auggaauguaaagaaguauggaU....................	6	1	J17
....auggaauguaaagaaguauggagu...................	12	0	J17
....auggaauguaaagaaguauggagA...................	16	1	J17
....Uuggaauguaaagaaguauggagu...................	98	1	J17
....Cuggaauguaaagaaguauggagu...................	4	1	J17
....Uuggaauguaaagaaguauggaguu..................	1	1	J17
....uggaauguaaagaaguaC........................	1	1	J17
....uggaauguaaagaaguaA........................	12	1	J17
....uCgaauguaaagaaguau........................	1	1	J17
....uggaauguaaagaaguaG........................	3	1	J17
....Aggaauguaaagaaguau........................	2	1	J17
....uggaauguaCagaaguau........................	1	1	J17
....uggaauguaaagaaguau........................	664	0	J17
....uggaGuguaaagaaguaug.......................	1	1	J17
....Aggaauguaaagaaguaug.......................	6	1	J17
....uggaauCuaaagaaguaug.......................	1	1	J17
....uggaaugAaaagaaguaug.......................	1	1	J17
....ugUaauguaaagaaguaug.......................	1	1	J17
....uggaauguaaagaaguaug.......................	3675	0	J17
....uggGauguaaagaaguaug.......................	1	1	J17
....Gggaauguaaagaaguaug.......................	1	1	J17
....uggaauguaaagaaCuaug.......................	1	1	J17
....ugCaauguaaagaaguaug.......................	1	1	J17
....uAgaauguaaagaaguaug.......................	1	1	J17
....uggUauguaaagaaguaug.......................	1	1	J17
....uggaauguaaagUaguaug.......................	1	1	J17
....uggaauUuaaagaaguaug.......................	1	1	J17
....Cggaauguaaagaaguaug.......................	2	1	J17
....uggaauguaCagaaguaug.......................	1	1	J17
....uggaauguaaagaaguaGg.......................	3	1	J17
....uggaauguaaagaaguauA.......................	17	1	J17
....uggaauguaaUgaaguaug.......................	1	1	J17
....uggaaCguaaagaaguaug.......................	2	1	J17
....uggaaugCaaagaaguaug.......................	1	1	J17
....uggaauguaaagaaguaCg.......................	2	1	J17
....uggaauguaaagaaguaAg.......................	1	1	J17
....uCgaauguaaagaaguaug.......................	2	1	J17
....uggaaugGaaagaaguaugg......................	6	1	J17
....uggaauCuaaagaaguaugg......................	6	1	J17
....uggaauguaaagaaUuaugg......................	3	1	J17
....Aggaauguaaagaaguaugg......................	136	1	J17
....uggaauguaaagaagGaugg......................	3	1	J17
....uggaauguaCagaaguaugg......................	16	1	J17
....uggaaugCaaagaaguaugg......................	5	1	J17
....uggaGuguaaagaaguaugg......................	3	1	J17
....uggaauguUaagaaguaugg......................	6	1	J17
....uggaauguaaagCaguaugg......................	1	1	J17
....uggaauguaUagaaguaugg......................	2	1	J17
....ugUaauguaaagaaguaugg......................	20	1	J17
....uggaauguaaagaagCaugg......................	6	1	J17
....uggaauguaaagUaguaugg......................	5	1	J17

```
                              Star                                     Mature
acauuucucaaucggugcccucuaguggaucccguacuucuucacaucuccauauuucauuggauauuuauggaauguaaagaaguauggaguuuacuaauguaaaacgagg
......................................................................uggaauguaaagaaguaCgg......................    4      1    J17
......................................................................uUgaauguaaagaaguaugg......................    7      1    J17
......................................................................uggaauguaaagaaguUugg......................    6      1    J17
......................................................................uggaauguaaagaaguaGgg......................    18     1    J17
......................................................................uggaauguaaagaaAuaugg......................    6      1    J17
......................................................................uggaauguaaaAaaguaugg......................    8      1    J17
......................................................................uggUauguaaagaaguaugg......................    4      1    J17
......................................................................uggaauguaaGgaaguaugg......................    1      1    J17
......................................................................uggaauguaaagaaguaugC......................    13     1    J17
......................................................................uCgaauguaaagaaguaugg......................    10     1    J17
......................................................................uggaauguaaagaaCuaugg......................    5      1    J17
......................................................................uggaauguaaagaaguauAg......................    71     1    J17
......................................................................uggaUuguaaagaaguaugg......................    3      1    J17
......................................................................uggaauguaaUgaaguaugg......................    3      1    J17
......................................................................uggGauguaaagaaguaugg......................    6      1    J17
......................................................................uAgaauguaaagaaguaugg......................    7      1    J17
......................................................................uggaauguaaaCaaguaugg......................    3      1    J17
......................................................................uggaauguCaagaaguaugg......................    2      1    J17
......................................................................uggaCuguaaagaaguaugg......................    2      1    J17
......................................................................uggaauguaaagaagAaugg......................    16     1    J17
......................................................................uggaaugAaaagaaguaugg......................    30     1    J17
......................................................................uggaauguaaagaaguGugg......................    3      1    J17
......................................................................uggaauguaaaUaaguaugg......................    6      1    J17
......................................................................uggaaGguaaagaaguaugg......................    7      1    J17
......................................................................uggaauguGaagaaguaugg......................    3      1    J17
......................................................................uggaauguaaagaaguaAgg......................    5      1    J17
......................................................................Gggaauguaaagaaguaugg......................    31     1    J17
......................................................................uggaauguaaagaaguauCg......................    6      1    J17
......................................................................uggaauguaaagaaguauUg......................    2      1    J17
......................................................................uggaauguaaagaUguaugg......................    5      1    J17
......................................................................ugAaauguaaagaaguaugg......................    12     1    J17
......................................................................uggaaAguaaagaaguaugg......................    12     1    J17
......................................................................uggaauAuaaagaaguaugg......................    4      1    J17
......................................................................uggaauUuaaagaaguaugg......................    5      1    J17
......................................................................uggaauguaaagaaguaugg......................    56648  0    J17
......................................................................ugCaauguaaagaaguaugg......................    5      1    J17
......................................................................uggaauguaaagaaguaugU......................    60     1    J17
......................................................................Cggaauguaaagaaguaugg......................    11     1    J17
......................................................................uggaauguaaagaGguaugg......................    1      1    J17
......................................................................uggaaCguaaagaaguaugg......................    4      1    J17
......................................................................uggaauguaGagaaguaugg......................    2      1    J17
......................................................................uggaauguaaagaaguaugA......................    229    1    J17
......................................................................uggaauguaaagGaguaugg......................    11     1    J17
......................................................................uggaauguaaagaGguaugga.....................    7      1    J17
......................................................................uggaaugGaaagaaguaugga.....................    13     1    J17
......................................................................uggaaAguaaagaaguaugga.....................    58     1    J17
......................................................................uggaauguaaagaaAuaugga.....................    29     1    J17
......................................................................uggaaGguaaagaaguaugga.....................    55     1    J17
......................................................................uggaUuguaaagaaguaugga.....................    68     1    J17
......................................................................uggaauAuaaagaaguaugga.....................    32     1    J17
......................................................................uggaauguaaagaaguauggU....................    278    1    J17
......................................................................uggaauguaaagaagGaugga.....................    11     1    J17
......................................................................uggaauguaUagaaguaugga.....................    23     1    J17
......................................................................uggGauguaaagaaguaugga.....................    40     1    J17
......................................................................ugCaauguaaagaaguaugga.....................    66     1    J17
......................................................................uggaauguaaagaaCuaugga.....................    31     1    J17
......................................................................uggaauguaaGgaaguaugga.....................    4      1    J17
......................................................................uggaauguaaagaaguauUga.....................    37     1    J17
......................................................................ugAaauguaaagaaguaugga.....................    189    1    J17
......................................................................uUgaauguaaagaaguaugga.....................    38     1    J17
......................................................................uggaauguaCagaaguaugga.....................    137    1    J17
......................................................................uggaCuguaaagaaguaugga.....................    3      1    J17
......................................................................uggaauguGaagaaguaugga.....................    22     1    J17
......................................................................uggaauguaaagaagCaugga.....................    20     1    J17
......................................................................uggaauguaaaAaaguaugga.....................    50     1    J17
......................................................................uAgaauguaaagaaguaugga.....................    43     1    J17
......................................................................uggaauUuaaagaaguaugga.....................    34     1    J17
......................................................................uggaauguaaagaaUuaugga.....................    51     1    J17
......................................................................uggaauguCaagaaguaugga.....................    5      1    J17
......................................................................uggaauguUaagaaguaugga.....................    77     1    J17
```

```
                              Star                                   Mature
acauuucucaaucggugcccucuaguggaucccguacuucuucacaucuccauauuucauuggauauuuauggaauguaaagaaguauggaguuuacuaauguaaaacgagg
......................................................................uggaauguaGagaaguaugga.....................    17      1    J17
......................................................................uggaauguaaaCaaguaugga.....................    31      1    J17
......................................................................Gggaauguaaagaaguaugga.....................    218     1    J17
......................................................................uCgaauguaaagaaguaugga.....................    111     1    J17
......................................................................uggaauguaaagaaguCugga.....................    1       1    J17
......................................................................uggaauguaaagaaguauCga.....................    20      1    J17
......................................................................uggaauguaaagaaguauAga.....................    40      1    J17
......................................................................Aggaauguaaagaaguaugga.....................    1148    1    J17
......................................................................uggaauguaaagaaguaugCa.....................    96      1    J17
......................................................................uggaGuguaaagaaguaugga.....................    9       1    J17
......................................................................uggUauguaaagaaguaugga.....................    69      1    J17
......................................................................uggaaugCaaagaaguaugga.....................    21      1    J17
......................................................................uggaauguaaagCaguaugga.....................    2       1    J17
......................................................................uggaauguaaUgaaguaugga.....................    16      1    J17
......................................................................uggaauguaaagaaguaugga.....................    424536  0    J17
......................................................................uggaauguaaagaaguUugga.....................    22      1    J17
......................................................................uggaauguaaagaaguGugga.....................    7       1    J17
......................................................................uggaauguaaagaaguauggC.....................    71      1    J17
......................................................................uggaauCuaaagaaguaugga.....................    17      1    J17
......................................................................uggaauguaaagaaguauggG.....................    394     1    J17
......................................................................Cggaauguaaagaaguaugga.....................    124     1    J17
......................................................................uggaauguaaaUaaguaugga.....................    46      1    J17
......................................................................uggaauguaaCgaaguaugga.....................    2       1    J17
......................................................................uggaaCguaaagaaguaugga.....................    45      1    J17
......................................................................uggaauguaaagaaguaAgga.....................    64      1    J17
......................................................................uggaauguaaagaUguaugga.....................    46      1    J17
......................................................................uggaauguaaagaaguaugUa.....................    208     1    J17
......................................................................uggCauguaaagaaguaugga.....................    3       1    J17
......................................................................ugUaauguaaagaaguaugga.....................    140     1    J17
......................................................................uggaauguaaagGaguaugga.....................    17      1    J17
......................................................................uggaauguaaagaaguaugAa.....................    213     1    J17
......................................................................uggaauguaaagaaguaCgga.....................    19      1    J17
......................................................................uggaauguaaagUaguaugga.....................    58      1    J17
......................................................................uggaauguaaagaagAaugga.....................    122     1    J17
......................................................................uggaaugAaaagaaguaugga.....................    191     1    J17
......................................................................uggaauguaaagaaguaGgga.....................    53      1    J17
......................................................................uggaauguCaagaaguauggag....................    27      1    J17
......................................................................uggaauguaUagaaguauggag....................    72      1    J17
......................................................................uggaauguaaagaaguCuggag....................    17      1    J17
......................................................................uggaaCguaaagaaguauggag....................    432     1    J17
......................................................................uggaaugGaaagaaguauggag....................    95      1    J17
......................................................................uAgaauguaaagaaguauggag....................    239     1    J17
......................................................................uggaauguaaagaaguaGggag....................    337     1    J17
......................................................................uggaauguaaagaaguauggaC....................    13660   1    J17
......................................................................uggaauguaaaAaaguauggag....................    174     1    J17
......................................................................uggaauguaaagaCguauggag....................    7       1    J17
......................................................................uUgaauguaaagaaguauggag....................    233     1    J17
......................................................................uggaauguaaagaaguauggaU....................    9098    1    J17
......................................................................Aggaauguaaagaaguauggag....................    6653    1    J17
......................................................................uggaGuguaaagaaguauggag....................    58      1    J17
......................................................................uggaauguaaagaaguauUgag....................    268     1    J17
......................................................................ugCaauguaaagaaguauggag....................    313     1    J17
......................................................................uggaauguGaagaaguauggag....................    147     1    J17
......................................................................uggaauguaaagaagGauggag....................    59      1    J17
......................................................................uggaauAuaaagaaguauggag....................    155     1    J17
......................................................................uggaauCuaaagaaguauggag....................    125     1    J17
......................................................................uggaauguaaagaGguauggag....................    94      1    J17
......................................................................uggaUuguaaagaaguauggag....................    364     1    J17
......................................................................uggaauguaaagaaguauggUg....................    1055    1    J17
......................................................................uggaauguaaGgaaguauggag....................    68      1    J17
......................................................................uggaauUuaaagaaguauggag....................    183     1    J17
......................................................................uggUauguaaagaaguauggag....................    294     1    J17
......................................................................uggaauguaaagaUguauggag....................    318     1    J17
......................................................................uggaauguaaagaaAuauggag....................    162     1    J17
......................................................................uggCauguaaagaaguauggag....................    19      1    J17
......................................................................ugAaauguaaagaaguauggag....................    881     1    J17
......................................................................uggaauguaaCgaaguauggag....................    23      1    J17
......................................................................uggaauguaaagaaguaCggag....................    74      1    J17
......................................................................Gggaauguaaagaaguauggag....................    1238    1    J17
......................................................................uggaauguaaagaaguaugCag....................    561     1    J17
```

Star Mature

acauuucucaaucggugcccucuaguggaucccguacuucuucacaucuccauauuucauuggauauuuauggaauguaaagaaguauggaguuuacuaauguaaaacgagg

....uggGauguaaagaaguauggag....................	296	1	J17
....uggaauguaaagaaCuauggag....................	166	1	J17
....uggaCuguaaagaaguauggag....................	30	1	J17
....uCgaauguaaagaaguauggag....................	725	1	J17
....uggaauguaaagaaguauCgag....................	120	1	J17
....Cggaauguaaagaaguauggag....................	706	1	J17
....uggaauguaaagaaguUuggag....................	166	1	J17
....uggaauguaaagaaguaugUag....................	989	1	J17
....uggaaugCaaagaaguauggag....................	148	1	J17
....uggaauguaaagGaguauggag....................	152	1	J17
....uggaauguaaaUaaguauggag....................	254	1	J17
....uggaauguaaagaaguauggag....................	2756061	0	J17
....uggaauguaaaCaaguauggag....................	131	1	J17
....uggaauguaaUgaaguauggag....................	103	1	J17
....uggaauguUaagaaguauggag....................	444	1	J17
....uggaauguaGagaaguauggag....................	86	1	J17
....uggaauguaaagaaguaAggag....................	337	1	J17
....uggaauguaaagaagAauggag....................	555	1	J17
....uggaauguaaagUaguauggag....................	313	1	J17
....uggaaGguaaagaaguauggag....................	381	1	J17
....uggaaugAaaagaaguauggag....................	1054	1	J17
[truncated: 810,382 more chars]
