# Supplementary material for: Millipede genomes reveal unique adaptations during myriapod evolution
Source: PLoS Biol. 2020 Sep 29;18(9):e3000636. doi: 10.1371/journal.pbio.3000636 (PMC7523956; doi:10.1371/journal.pbio.3000636)
Supplement: Attachment — Submitted filename: Response.docx [file pbio.3000636.s037.docx]

**Editor’s comments:**

**IMPORTANT: You'll see that both reviewers are broadly positive about your study, but each makes a number of requests, largely for significant improvement of the presentation of the paper.**

**Reviewer #1 has seen your paper elsewhere, and while noting that some issues have been addressed, s/he feels that it still falls short of its potential. We *strongly* encourage you to use this further opportunity to thoroughly address these issues (the Academic Editor wonders whether it might help to move some of the secondary stories to the supplementary material and thereby enhance the main narrative).**

**You will also see that reviewer #2 makes a large number of very constructive and specific recommendations regarding the structure and balance of the manuscript, many of which may help to remedy some of the problems identified by reviewer #1. Reviewer #2 also points out that most of your methods are in the Supplementary Information; this is not appropriate, and they should be moved to the
main manuscript (there are no page constraints in PLOS Biology).**

**Response**:

We thank editor and reviewers very much for their positive comments and constructive suggestions. We have treated all of them seriously, and have made significant changes on the restructuring and rebalancing the weight of the main text of the manuscript, including:

- Rebalancing the focus of the manuscript;
- Modifying data presentation in the main figures;
- Moving several secondary stories to supplementary materials;
- Moving methodology to the main text;

In addition, we have also addressed all of the comments of both Reviewers 1 and 2 by carrying out additional experiments, as well as addressing other concerns raised.

Detailed point-by-point responses are provided below. We believe the manuscript is now significantly improved as a consequence of these helpful suggestions, and we thank you for your consideration of our manuscript for publication in PLoS Biology.

**Reviewer #1 (R1):**

***R1: Qu et al. report on the sequencing of two species of millipedes and on a genomic analysis of these species from a number of aspects. I have previously reviewed this manuscript for a different journal. I am pleased to see that the authors have taken many of my comments from the previous review into account in this revised submission. However, several of my criticisms remain. In what follows, I recap my previous review, with relevant changes and omissions, taking into account what the authors have changed in the interim.***

***Myriapods in general, and specifically millipedes are significantly under-represented in the world of arthropod genomics, and two new sequenced species are a very welcome addition, with significant potential for novel evolutionary insights. The quality of the sequencing, in terms of completeness and contig size, is very high, suggesting great potential. I therefore approached the manuscript with much enthusiasm, with the expectation that this would be a manuscript worth publishing. In that first reading, I was disappointed, but my recommendation to the authors was to make the effort to improve the manuscript and to deal with its problems so as to bring it up to a level that is suitable for publication. The quality of the data the authors have and the level of analyses they have carried out shows that there is definitely the potential for an excellent paper.***

***The previous version of the article had many technical problems, most of which have been dealt with. However, my main impression with the manuscript was that it is unfocused. There is no clear structure and the main message is not presented clearly (to the extent that there is a main message). The authors have since added a Conclusions paragraph that puts the work into a better context, but some work is needed within the rest of the manuscript to provide a stronger focus.***

***What tends to happen in most genome projects is that there are lot of "little stories" that come out of the genome, and putting them together to tell a coherent "big story" (whether evolutionary or applied) is tricky. In this case, I think the authors do not provide a correct balance of stories. Some get bogged down in details (the miRNA story) whereas others are drawn in such broad brush strokes that there is no message (the ozadene story). The "big story" - what we can learn about arthropod evolution form these two new genomes - is almost completely missing.***

**Response 1**:

We thank the reviewer for re-reading the manuscript and providing these constructive suggestions. We have followed the advice of both the editor and the reviewer in moving the details of several stories (e.g. consideration of TEs and miRNA) into the supplementary information, and we have also expanded other stories (e.g. ozadene gland analyses, and Hox gene consideration, see also Response 16-17). We hope that the restructuring and rebalancing of the main text of the manuscript can now allow the readers to obtain the message what we can learn about arthropod evolution from these two new genomes. Detailed point-to-point response are shown below.

***R1: Specific comments: Scientific issues 1) Millipedes are said to be the third largest arthropod class after insects and arachnids (line 81-82). However, these taxa are each at a different taxonomic level, so the comparison is meaningless. The reference for this claim is a taxonomic survey of Amazonian arthropods; hardly the best reference for such a claim.***

**Response 2**:

We thank the reviewer for the careful reading. We have now changed the sentence as well as reference, which now cites a major review paper on Myriapoda, as follows:

“Millipedes compose the class Diplopoda, a highly diverse group containing more than 12,000 described species (Sierwald and Bond 2007).”

Reference:

Sierwald P, Bond JE. (2007). Current status of the myriapod class Diplopoda (Millipedes): taxonomic diversity and phylogeny. Annual Review of Entomology, 52, 401-420.

***R1: 2) The section on synteny added a comparison to the human genome, following a suggestion I made to the previous version. This now tells a much more interesting story, but the story still feels under-developed. The relevant figure (Fig. 2) needs a more detailed figure legend to explain exactly what is presented in it.***

**Response 3**:

We agree that it is interesting that millipede genomes show more syntenic regions to the human genome than to the centipede genome. Incorporating the suggestions from Response 15 as well, Figure 5 (original Figure 2) has now been remade, and a more detailed figure legend is now included, as provided below:

“Figure 5. Synteny comparisons of myriapod (millipedes *Helicorthomorpha* and *Trigoniulus* and centipede *Strigamia*) and human genomes. Note that more syntenic regions could be detected between millipede genomes to the human genome than between the centipede genome and human genome. Abbreviations: Mindots = Minimum number of genes required to define a syntenic block.”

Further, we have elaborated on the significance of these findings in the main text, as follows:

“To further elucidate this situation, we also compared the syntenic relationships between the three myriapod genomes to that of human, to test specifically whether we can detect conserved syntenic blocks to deuterostomes, as for the centipede genome *Strigamia*. This finding would consolidate the idea that the last common ancestor of myripods retained significantly greater synteny with the last common ancestor of bilaterians than insect ancestors did (Chipman et al 2014), or conversely, that this is only the case for the centipede genome. As shown in Figure 5, syntenic blocks could be detected between both millipede genomes and the human genome, and indeed, millipede genomes share more syntenic blocks (regardless of the number of genes defining a syntenic block) with the human genome than with the centipede genome (Figure 5). Thus, we confirm here that last common ancestor of myripods retains greater synteny with the last common ancestor of bilaterians than insects. Consequently, these data highlight the importance of millipede genomes as a reference for the reconstruction of animal evolutionary history.”

***R1: 3) In the beginning of the Synteny section (l. 150-151) the authors say that myriapods are interesting as the outgroup to Insecta. This is incorrect. The outgroup to Insecta are crustaceans. Myriapods are the outgroup to Pancrustacea. In the following section, myriapods are not compared to insects at all, raising the question why this is mentioned. In the next section (homeobox clusters - l. 179) the comparison to insects comes up again, but it is not clear what the context is. Why does the duplication of three homeobox genes suggest suitability for a comparison with insects?***

**Response 4**:

We thank the reviewer for raising this issue. To avoid confusion, this paragraph has now been deleted. We have rewritten the section that follows on the homeobox gene and cluster to clarify the situation as follows:

“The most celebrated feature of millipedes are their many body segments and accompanying high number of legs. Homeobox genes are an ideal candidate to study body plan evolution, as they are conserved gene expression regulators in animals. To understand whether the three myriapod lineages considered here share a similar number of homeobox genes, we compared their homeobox gene content to that of all available insect genomes. We found that the three myriapod genomes have undergone three lineage-specific duplications of common homeobox genes (Otx, Barhl, Irx) (Supplementary information S1, Figure S1.3.4). These data suggest that millipede genomes have not undergone massive homeobox gene duplications comparable to that which has occurred in the centipede genome.”

However, we are willing to remove this section also, if the reviewer prefers.

***R1: 4) I am not enough of an expert on transposable elements or on miRNA to comment on the science of these two sections. However, both sections are overly detailed and overly technical for a paper with a general focus. I got lost in many of the paragraphs and felt they were lacking context or justification. Specifically, I found the discussion of host genome evolution and TEs confusing to the non-specialist.***

**Response 5**:

In the resubmitted manuscript we have taken the reviewer’s advice and greatly simplified the section on transposable elements/repeat and miRNA contents, moving the more specialist sections to the supplementary information. We have also clarified the discussion of genome evolution and TEs, to make it more accessible to a wider audience.

***R1: 5) Figure 1B,C - What is the source for the life history illustrations (or the data in them, if they are original)?***

**Response 6**:

The illustrations are original, and we built the life history illustrations based on two previously published papers that document the millipede’s life cycles. For *Helicorthomorpha holstii*, this is based on documentation for a related polydesmid species and our own observations; and the life cycle of *Trigoniulus corallinus*, its has been previously studied by Shinohara et al (2007). This information has been added back to the figure legend, as follows:

“Figure 1B) Life cycle of the orange rosary millipede, *Helicorthomorpha holstii* (based on Miley 1927 and our observations); C) Life cycle of the rusty millipede, *Trigoniulus corallinus* (based on Shinohara et al 2007)”

Reference:

Miley HH (1927). Development of the male gonopods and life history studies of a polydesmid millipede. The Ohio Journal of Science, 27(n1); 25-43.

Shinohara K, Hika Y, Niijima K. (2007). Postembryonic development of Trigoniulus corallinus (Gervais)(Diplopoda: Spirobolida, Pachybolidae). Edaphologia (Japan). Edaphologia, 82; 9-16.

***R1: 6) Figure 1F - What are we seeing here? This Venn-diagram is not explained at all.***

**Response 7**:

The information has been added back to the figure legend, as shown below:

“The Venn-diagram shows the number of *in silico* predicted non-toxic peptides present in the *Trigoniulus corallinus* ozadene, that possess antibiotic, antibacterial, antifungal, and antiviral properties.”

***R1: Technical issues 7) Several places throughout the text: Strigamia maritima is abbreviated as S. strigamia. This was fixed in a few places, but some instances remain.***

**Response 8**:

We thank the reviewer for pointing out this mistake. We have corrected all instances of this in the resubmitted manuscript.

***R1: There is an inconsistency in the level of plain-text vs. jargon. Some terms are explained, whereas others, which are not widely understood, are left with no explanation. An obvious example is in the second paragraph of the Introduction: Collum, prozonite, metazonite should be explained.***

**Response 9**:

We are grateful to the reviewer for pointing out this issue. We have now provided an explanation of the terms mentioned, and have sought to remove jargon and simplify our use of language throughout the manuscript. In cases which are deemed to be scientifically important, references have also been included. All changes are highlighted in the manuscript. An example of the second paragraph of the Introduction is provided below:

“Millipedes compose the class Diplopoda, a highly diverse group containing more than 12,000 described species (Adis 2002). Millipedes are highly important components of terrestrial ecosystems, especially regarding their roles in the breakdown of organic plant materials and nutrient recycling. In contrast to centipedes, which have one pair of legs per body segment, individual body segments are fused in pairs in millipedes, resulting in a series of double-legged segments (diplo-segments). The typical millipede body plan consists of the head, the collum (the first posterior segment next to the head which is legless, from the Latin for ‘neck’), and trunk (the remaining length of the body, with varying numbers of diplo-segments)(Hopkin and Read 1992). The primary defense mechanisms of millipedes are their tough exoskeletons and their ability to curl up into a tight coil, while a unique secondary defense system in some species involves emitting toxic liquids or gases from the ozadene gland, via ozopores located on each side of the posterior portion of diplo-segment termed ‘metazonites’ (Enghoff 1993; Figure 6A).”

***R1: The common names of one the two species is problematic. The first species, H. holstii is referred to as "The polydesmid miilipede" as if that were its common name. This is actually a very general term, since Polydesmidae is the family, so all they are saying is that it's a member of the family. From what I could see, there is no common English name for this species, and it is usually mentioned as an Asian polydesmid. Does this species have a common name in Cantonese? Rather than leaving the species orphaned and nameless, I suggest the authors give it a name - ideally a translation of an existing common name, and that will become the name it is known by (at least in the genome literature). Rusty millipede for T. corallinus is fine.***

**Response 10**:

We agree that this is a useful point. We suggest the ‘Orange rosary millipede’, which has been used in Chinese literature, since when looking the *H. holstii* from the top, the centipede looks like the chain of wooden rosary beads in the Buddha’s hand. We have modified the text accordingly.

***R1: Materials and Methods are understandingly brief, with most of the details being in the Supplementary sections. Nonetheless, there are some methods that should be introduced in brief since their results are discussed at length in the main text (e.g. double-luciferase assays, proteomics)***

**Response 11**:

We have followed both the editor and reviewer’s suggestions, and have moved the whole supplementary methodology section to the main text. This will provide immediate access to all the necessary information to readers.

***R1:* The references are poorly formatted using inconsistent style and are missing all italicization in gene names and species names.**

**Response 12**:

We apologise for this issue, and have now changed all references to the PLoS Biology format.

**Reviewer #2 (R2):**

***R2: The manuscript by Qu et al. describes the sequencing, assembly, and analysis of two millipede genomes. Myriapods are underrepresented among published genomes, so this is an important contribution to the community. I would like to congratulte the authors for such a high quality assembly, with scaffold N50 around or over 20Mbp. The contig N50 remains moderate (184~335kbp), but scaffolding with Hi-C and restriction-map seemed to have played very well.***

***Analyses accompanying each of the specific analyses are presented with substantial additional experiments and data. On the other hand, throughout the analyses and throughout the manuscript, the key research question (or questions for specific analyses) for studying the millipedes was unclear. I detail my comments below to clarify.***

**Response 13**:

We thank the reviewer for their positive comments and constructive suggestions. Detailed point-by-point responses are provided below.

***R2: 1. Ozadene gland analysis - Search for protein toxins and antimicrobial peptides seem odd, because ozopores of dilopods are basically well known for their chemical defence, mainly using hydrogen cyanide and benzaldehyde as the primary chemical. No specific mention of these chemical defense is given, and the authors seem to complete ignore the biosynthetic pathways for these chemical. Please provide the reasons that the analyzed species do not employ such chemical defense in their ozopores.***

***The protome analyses lack the information on the abundance of composite proteins. Since there were over 2600 peptides identified, it is not useful to treat each peptides equally. If protein-based defence is the key in these species, key proteins must be highly abundant. Please discuss the significance of each candidates based on their abundances. It is also possible that hypothetical proteins with high abundance may be the key protein.***

***The search for toxins and antimicrobial defence probably contains errors. For example, Tco_003099-T1 is noted as "AB AF AV Antibiotics", but blasting this sequence against RefSeq shows that this is actually mitochondrial succinate dehydrogenase (8e-148).***

**Response 14**:

We thank the reviewer for these constructive comments.

For the first point relating to proteins involved in the biosynthetic pathways of hydrogen cyanide and benzaldehyde, this is a very good point! Searching through the proteomic data generated from the ozadene gland in *T. corallinus*, we could not identify any potential peptides that are involved in the biosynthesis of hydrogen cyanide or benzaldehyde, which have been found in most of the millipede ozadene secretions.

To answer the reviewer’s question on providing reasons that the analysed species do not employ such chemical defence, we then searched the related biosynthetic genes in both *T. corallinus* and *H. holstii* genomes. To our surprise, only the *H. holstii* but not the *T. corallinus* possess most of the biosynthetic genes in its genomes. This suggests the secondarily lineage-specific rearrangements which explain the analysed species do not employ such chemical defence. The whole section of ozadene gland has been rewritten and a new figure 7 has also been added.

We have also included the abundance of composite proteins in Supplementary information S4. Given the abundance range from ~4 x 10^4^ to 1.1x 10^10^, we have decided to investigate the ones that are in the range of 10^9^ and 10^10^ which include 12 proteins. The reviewer is correct that 7 out of these 12 proteins are hypothetical proteins, and the remaining proteins are Adenosinetriphosphatase involved in purine and thiamine metabolism, Glutathione transferase which could involve in various pathways (from Metabolism of xenobiotics by cytochrome P450, Phenylpropanoid biosynthesis, Dioxin degradation, Drug metabolism - cytochrome P450, Arachidonic acid metabolism, Drug metabolism - other enzymes, Glutathione metabolism), and three proteins are Tyrosinase involved in Isoquinoline alkaloid biosynthesis, Tyrosine metabolism, Betalain biosynthesis pathways. So we further the top most abundant pathways, and the new analysed results are included in Supplementary information S1 Figure S1.3.2.

We also thank the reviewer for picking up that the search for toxins and antimicrobial defence could contain errors. Based on the suggestion, we have reanalysed all proteins with antiviral, antibacterial, antifungal, and antibiotic properties. In the original submission, we have included 3 proteomes (two with fractionation and one without) to maximise the number of proteins identified, which is ~2,600 proteins in total. Given the above suggestion to include abundance, for accuracy and consistency, we have only used the two proteomes with fractionation, which confidently assigned ~2,100 proteins. Using this confident data set, nevertheless, Tco_003099-T1 is still being picked up in both the detection of antibacterial and antibiotic categories, with a probability threshold equal or larger than 0.95. Therefore, we have performed manual eye-checking throughout the list with potential wrong annotations. Using this final list of proteins with the highest confidence assignations we can achieve from the ozdene proteomes, we have carried out the further analyses on assigning their functions (Figure 7 and Supplementary information S1, Section 1.3.3).

***R2: 2. Conserved synteny - Synteny blocks shown in Fig.2 is probably too strict. Dot plot analysis of Chipman et al. between B. floridae and S. maritima was able to show the distant relationships, but this figure is not being able to show such synteny even between Helicorthomorpha and Strigamia. Moreover, it is almost no use to compare synteny with the human genome. The genome size is too different to visualize in this form, and is also way too distant. What was the aim of comparing with humans and not B. floridae? Even if it is compared against B. floridae, it does not provide useful information that will greatly enhance what is already reported by Chipman et al. (because the distance between previously reported S. maritima and the two millipeds of this work is negligibly small compared to that between myriapods and chordates). Fig. 2 is very uninformative and could be in supplementary figures.***

**Response 15**:

We apologise for not making this clearer in the previous version of the manuscript. Similar to Response 3, we have now elaborated the significance of these findings in the main text, as outlined above in Response 3 to Reviewer 1.

In addition to using the setting of using 7 genes in assigning a syntenic block, we have now also carried out additional analyses using less numbers of genes to assign a syntenic block. The syntenic comparisons between millipedes, as well as millipedes to centipede, have been moved to the supplementary information (S1, Figure S1.3.18) as suggested. Nevertheless, we would like to retain the syntenic comparisons of millipede genomes to human, as well as the centipede genome to human in the main figure, due to the significance of these findings for animal evolution.

***R2: 3. Homeobox - I expected this section to include the key biological findings of this manuscript, because a substantial part (about 50%) of the Introduction is dedicated to the different morphology of centipedes and millipedes, as well as different development stages between the two millipedes analyzed. However, no such discussion is given here. Please at least discuss something about the difference in developmental stages that are found from the genome, somewhere in the manuscript, or consider removing such mention in dedicated Fig. 1, because it confuses the reader about the key research question of this paper.***

**Response 16**:

This is a valid point, and we have now carried out additional analyses to investigate the expression profiles for all homeobox genes at the different developmental stages considered in this study (Supplementary information S1, S1.3.7b. and S1.3.7c). Further, we have also elaborated the findings (together with Response 17) to rebalance the structure of the whole manuscript. The changes are shown as follows:

“In both millipedes, homeobox genes are generally expressed early in development rather than during later developmental stages, nevertheless, the homeobox genes in *H. holstii* generally have higher expression levels in stadium 2 than in the egg stage, while the situation is reversed in *T. corallinus* (Supplementary information S1, Figure S1.3.7b,c). Whether these observed expression patterns represent differences in the developmental modes between polydemidans and spirobolidans remains to be tested in other millipedes.”

***R2: In terms of the contribution of ANTP cluster Hox genes in centipede development, excellent work by Hughes and Kaufman (Development 2002) should not be ignored. How does the Hox cluster pattern or their expression link to the expansion of body segmentation in millipedes? Hox3 is functioning in the head segment in centipede, and appendages around the mouth is a morphologically variable feature in myriapods. Does the Hox3 conservation pattern at least partly link to such morphology in the studied species?

Again, I think some discussion on segmentation pattern is critical here.***

**Response 17**:

First of all, we apologise for not including the aforementioned point as well as other related information previously. The suggestions are excellent.

Originally we would like to carry out whole-mount *in situ* hybridisation to compare the spatiotemporal expression pattern of Hox3 in millipedes to the centipedes. Nevertheless, we have used all previously collected embryos for generating these transcriptomes. So we have instead investigated the expression profile of Hox3 via the transcriptomes, compared the Hox3 gene sequences across arthropods, as well as written a new Hox3 paragraph with inclusion of a discussion on segmentation, which is shown as follows:

“Segmentation and tagmosis (the formation of tagmata through fusion and modification of several individual segments) are considered to be key drivers for the evolutionary success of arthropod adaptive radiations (Akam 2000). Changes in Hox gene evolution are linked to these processes (Hughs and Kaufman 2002a). In particular, *Hox3* has been an important player in arthropod evolution. For example, Hox3 has undergone tandem duplication to form the three copies *bicoid*, *zen* and *z2* in dipterans, and it has duplicated extensively in lepidopterans to take up novel roles (Ferguson et al 2014). Unlike the fast evolving *Hox3* genes in these insects, the homeodomain sequences of *Hox3* duplicates in both the centipede *S. maritima* and the millipede *T. corallinus* are conserved (Panfilio and Akam 2007; Supplementary information S1, Figure S1.3.3). In both the centipede *Lithobius atkinsoni* and the millipede *Glomeris marginata*, *Hox3* appears to have typical Hox-like role (Hughs and Kaufman 2002b; Janssen and Damen 2006). In our transcriptomic analyses, both the *Hox3A* and *Hox3B* genes were found to be expressed at higher levels early in development compared to latter developmental stages, while *Hox3A* is expressed in the egg stage, while *Hox3B* is expressed in both egg and stadium 2 (Supplementary information S1, Figure S1.3.7c). Future investigations on the spatiotemporal expression of *Hox3* genes in embryos will be helpful to see if they have typical Hox-like roles. Since *H. holstii* contains no *Hox3* orthologues, while both *T. corallinus* and *S. maritima* have two *Hox3* genes outside their Hox clusters, the bottom line appears to be that the genomic “relaxation” of *Hox3* from the intact tight Hox clusters appears to have occurred in the ancestor of all myriapods (Figure 2).”

***R2: 4. Transposable elements - Genome size difference of the two millipedes is striking (449MB and 182MB), but the authors try to explain this based on the expansion of TE. However, even after considering the extent of repeats (55% or 245Mb in T. corallinus and 19% or 35Mb in H. holstii), remaining 204Mb and 147Mb is still 25% of difference. Moreover, gene count is greater in H. holstii (Table1, 23013 in H. holstii, 21361 in T. corallinus. This by the way does not match Table 1.3.1, which adds to 15285 in T. corallinus and 15767 in H. holstii). Was there not a gene duplication or loss pattern (as seen in Hox3) among the two species? Number and size of intron also typically also contribute to the differences in the genome size.***

**Response 18**:

We thank the reviewer for raising this important point. It is correct that transposons can only offer a partial explanation for the observed increase in genome size. We have modified the manuscript by including percentages in line with this point in the transposable element section.

To address additional potential explanations for the remaining 28% expansion in genome size, we have compared the average gene length, intron size, and intergenic distance, and a new table is now included in Supplementary information S1, Table 1.3.1b. The information is now also included in the main text as a new section and shown as follows:

“**Millipede genome size evolution**

The genome size difference between the two millipedes is striking (182Mb and 449Mb). A large proportion of eukaryotic genomes is typically composed of repetitive DNA, and repeats are considered one of the key determinants of genome size (Chénais et al 2019). This is true also for millipedes, where consideration of repeats demonstrates that they contribute 35Mb of the *H. holstii* genome (~19% total genomic DNA), and 245Mb of the *T. corallinus* genome (~55% total genomic DNA) (for further details, see the *Transposable Elements* section below, and Supplementary Information S1 section 1.3.2). However, repeats appear to account for only part of the difference observed in genome size between the millipedes, since if repeats are discounted, genome size remains ~28% greater in *T. corallinus* (~204Mb after exlusion of repeats in *T. corallinus*, compared to ~147Mb after exclusion of repeats in *H. holstii*). We find that variation in genome size is further accounted for by a considerably greater total protein-coding gene size in *T. corallinus* (212Mb), due particularly to an expansion in intron size, as compared to *H. holstii* (78Mb)(Supplementary information S1, Table 1.3.1b). Thus, we find that both repeat content and intron size are major contributors to the observed differences in millipede genome size.”

***R2: 5. Small RNA analysis - Many reporter assays are reported here, but please make it clear that Drosophila culture cells are used for this assay. It is good that the authors confim the mode of action of several miRNAs, but what the authors aimed to find through these experiments were difficult to follow. If genomes are analyzed in neglected groups, they would naturally have conserved aspects and also unconserved parts. Yes, millipedes do have different usage of miRNAs compared to Insecta, but what biological aspects of millipedes do these different modes actually contribute to?***

**Response 19**:

To make clear that the *Drosophila* cell line was used for the dual luciferase assays, we have adding this point to the methodology, as follows:

“Cell transfection and Dual-Luciferase Reporter Assays were carried out as described previously in *Drosophila* S2 cells using the psicheck-2-3’UTR and pAC5.1-miRNA”

Given that both reviewers share similar views regarding the lack of focus for this part, we have moved the majority of the microRNA arm switching story into the supplementary information Section 1.3.6.

***R2: I think Fig. S. 1.3.14 should be included in Fig.6.***

**Response 20**:

Given that the majority of the microRNA arm switching story has now been moved to the supplementary information, this figure has remained in the supplementary information.

***R2: So overall, the manuscript currently seems too descriptive. I would like to see some dicussions on the key research questions in studying the millipedes, and how a high quality genome actually contributes to elucidating those questions.***

**Response 21**:

We thank the reviewer for their useful suggestions. Similar to Response 1, we have expanded several key stories (e.g. Hox genes, ozadene peptides, genome rearrangement), in order to eluciadate further on key issues relating to millipede genomics. Further, the major messages we now raise have been summarise in the new conclusion. Specifically, is clear that millipede genomes, despite being in the same myriapod subphylum as the centipede *Strigamia*, have retained ancestral synteny with human, and have undergone unique adaptations to fundamental genomic components (e.g. Hox3 and microRNAs). Consequently, future research on understanding arthropod evolution, and in particular the reconstruction of the myriapod ancestral condition for comparative purposes, will require consideration of both additional centipede and millipede lineages. We have also added in discussions on the significance of each story now, rather than leaving them as simply descriptive.

***R2: Minor comments:***

***- Method section is too brief, and is almost entirely placed in Supplemental Information 1. PLoS Biology is an online journal, so space should not be a limitation. I would advice the authors to move the entire method text (S1.2) to the main manuscript, otherwise it is very difficult to follow.***

**Response 22**:

We have taken the editor’s and reviewer’s suggestions and moved the supplementary methodology section to the main text.

***R2: - Entire Results and Discussion section is constantly referred to Supplementary information S1. Supplementary Information S1.3 mostly simply contain figures and no supplementary text, so the mention to Supplementary information S1 is not necessary, and the current mention of specific supplementary text and figures already suffice. The Supplementary Information S1 contains 35 pages, and it is very difficult to flip back and forth finding necessary information from this very broad reference.***

**Response 23**:

We have now added in supplementary text to the Supplementary information S1, and also readjusted the main text in light to facilitate the readers to read.

***R2: - chromosome assembly More detailed data regarding the assembly would be informative. 1. Genome size estimate is written to be done with GenomeScope, but without the mention of exact estimate value. What was the estimate, and does the assembly size match the estimate? If not, why?***

**Response 24**:

The assembly sizes of both millipede genome assemblies (181Mb and 448Mb) matches quite well with the GenomeScope v2.0 estimation (159Mb and 437Mb). We have also carried out qPCR to estimate the genome size of both species based on the method of Wilhelm et al (2003), which revealed the genome sizes to be 162.78 ± 23 Mb and 408.74 ± 35 Mb. The GenomeScope results are now included in Supplementary information S1, Table 1.3.3c and Table 1.3.3d.

Reference

Wilhelm J, Pingoud A, Hahn M. Real-time PCR-based method for the estimation of genome sizes. Nucleic acids research. 2003;31(10):e56-e.

***R2: 2. What is known about the chromosome number of the analyzed species or other species that are phylogenetically close? Looking through PubMed there seem to be a number of studies on millipede karyotyping. Does the number of pseudomolecules corresopnding to the karryotypes? The numbers 8 and 17 seem to be very close to the reported number of chromosomes. However, then, do the assembled pseudomolecules span centromeres? Do they contain telomeric repeats at the ends? These detailed information support the quality of the assembly.***

**Response 25**:

This is a nice suggestion, and we have plotted the chromosome numbers documented in a previous study (Fontanetti et al 2002) onto millipede phylogeny, and the information is now included in Supplementary information S1, Figure 1.2.1. As one can see, the millipede chromosome number ranges from 8-30, and we could not identify chromosome fusion/fission events confined to any specific group. The reviewer is correct that the numbers 8 and 17 fall within the range of reported numbers of chromosomes.

For the other quality check on the telomeres, we have used bowtie and samtools to align the telomeric repeats (5’-TTAGG-3’) to the first and last 100,000bp of the assembled pseudomolecules of both species. The information is now included in Supplementary information S1, Table 1.3.1e and Table 1.3.1f. Repeats can also be revealed.

Reference

Fontanetti, C. S., Campos, K. A., Prado, R. A., & da Silva Souza, T. (2002). Cytogenetic studies in Diplopoda. *Cytologia*, *67*(3), 253-260.

***R2: What about the sex chromosome? Is it identifiable?***

**Response 26**:

This is a good question. We have chosen male individuals for both species for sequencing. The information regarding the sex of the millipedes being sequenced are now included in the methodology, as shown below:

“Genomic DNA (gDNA) was extracted from male *T. corallinus* and male *H.* holstii excluding the digestive tract, using a PureLink Genomic DNA Mini Kit (Invitrogen) following the manufacturer’s protocol.”

We cannot confidently demonstrate the sex chromosome based on the current genome assembly. As demonstrated in the centipede *Strigamia*, a XX/XY system of sex chromosomes can be confidently revealed by having information of both sexes (Green et al 2016). This could be a good follow-up study.

Reference

Green, JE, Dalikova, M, Sahara, K, Marec, F, Akam, M. XX/XY system of sex determination in the geophilomorph centipede *Strigamia maritima*. PLoS One, 11(2), e0150292.

***R2: 3. Screening for contamination using Kraken is noted, but the exact results are not given.***

**Response 27**:

1.45% and 0.52% sequencing reads of *H. holstii* and *T. corallinus* were marked as bacteria and removed using Kraken. The information is now mentioned in the methods section, as follows:

“Kraken was used to estimate the percentage of reads originating from bacterial contamination (Wood and Salzberg 2014), and 1.45% and 0.52% sequencing reads were marked as bacteria and removed from *H. holstii* and *T. corallinus* respectively.”.

***R2: 4. Some comparison with previous assembly of T. corallinus would be useful.***

**Response 28**:

This is a good suggestion. The information is now included in Table 1.

***R2: Data availability BioProject IDs (under embargo) are given in the manuscript, but no accession for the assembly is given. Availability of assembled genome is critical for this paper.***

**Response 29**:

The accession numbers are now included in Table 1.

***R2: Figure 1A: This tree does not even include chelicerates, which is mentioned in the text, so contains much less information than what is written in the text. This is totally unnecessary.***

**Response 30**:

We have now included chelicerates in Figure 1A, since a wide readership may not be familiar with arthropod relationships. However, if the reviewer would prefer to remove Figure 1A completely, we are fine with this.

***R2: Line 164 S. maritima species name is wrong***

**Response 31**:

We thank the reviewer for pointing out this mistake. We have corrected all instances of this in the resubmitted manuscript.

***R2: Line 321 Fig. probably S1.3.12, not 1.3.13 Supplementary Information S1 Italicizing species and gene names are wrong in many parts.***

**Response 32**:

We thank the reviewer for the careful reading. It has now been corrected.
