# Supplementary material for: Millipede genomes reveal unique adaptations during myriapod evolution
Source: PLoS Biol. 2020 Sep 29;18(9):e3000636. doi: 10.1371/journal.pbio.3000636 (PMC7523956; doi:10.1371/journal.pbio.3000636)
Supplement: Attachment — Submitted filename: Response final.docx [file pbio.3000636.s038.docx]

**Editor Comments**

***Thank you very much for submitting a revised version of your manuscript "Millipede genomes reveal unique adaptations during myriapod evolution" for consideration as a Research Article at PLOS Biology. This revised version of your manuscript has been evaluated by the PLOS Biology editors, the Academic Editor and both of the original reviewers.***

***In light of the reviews (below), we offer you the opportunity to address the remaining points from the reviewers in a revised version that we anticipate should not take you very long. We will then assess your revised manuscript and your response to the reviewers' comments and we may consult the reviewers again. I should warn you that we will consult the reviewers only one more time; if they remain unsatisfied then we will not consider the manuscript further.***

***IMPORTANT: You'll see that while both reviewers see improvements in your previous revisions, they both find the overall structure of the paper to be unsatisfactory, and suggest ways by which you should remedy this problem. However, you'll see that reviewer #2 has some more serious criticisms, which include a) scepticism about your claimed telomeres, wanting more clarity as to how you identified them, b) further queries about your analysis of conserved synteny, which is still problematic, partly because of your choice of humans as the “other” bilaterian, and c) concerns about your functional analysis of the ozadene gland transcriptome, given previous studies. The Academic Editor has asked me to emphasise these points, and especially the comparison with other species.***

***We expect to receive your revised manuscript within 1 month.***

***Please email us (***[***plosbiology@plos.org***](mailto:plosbiology@plos.org)***) if you have any questions or concerns, or would like to request an extension. At this stage, your manuscript remains formally under active consideration at our journal; please notify us by email if you do not intend to submit a revision so that we may end consideration of the manuscript at PLOS Biology.***

**Response**

We thank the editor and the reviewers for their highly valuable and constructive suggestions, which we believe have greatly improved our manuscript. We are very grateful for your kind generosity in time and effort. We have treated all reviewer concerns very seriously and have incorporated all suggested changes in the updated draft. Detailed point-by-point responses are provided below.

**REVIEWERS' COMMENTS:**

**Reviewer #1: [identifies himself as Ariel Chipman]**

**1) *This is a vastly improved version of the manuscript. The authors have addressed all of my main concerns, and the manuscript is now better focused and reads much more smoothly.***

***The manuscript needs some very minor tweaks, after which it can be accepted without re-review.***

**Response 1:**

We thank Prof. Chipman for all his constructive comments, which have significantly improved our manuscript.

**2) *The transposable elements section and the genome size section are somewhat redundant. I suggest merging them into one section (starting with TEs).***

**Response 2:**

We have combined these sections together as suggested. Please see Lines 126-174.

**3) *In the discussion of Ozadene glands, after giving details about the function and structure of these glands in millipedes, the authors ask what the function of the glands is. This seems like a strange question coming right after a paragraph explaining their function.***

**Response 3:**

We agree with this point, and have re-organised this section in line with the reviewer’s point. Please see Lines 345-391.

**4) *There are still minor typos scattered throughout - myripod instead of myriapod in a few places. Furtile instead of fertile (line 381). Missing articles in a few places. I didn't go through the manuscript and identify all of these, but I suggest the authors do one more round of thorough proofreading before resubmission.***

**Response 4:**

We have thoroughly checked the manuscript for typos as suggested.

**5) *The figures in the submitted version are all very small, and in many cases the fonts are too small even when the image is enlarged. Please follow the publishers guidelines on resolution and font size.***

**Response 5:**

We have modified the figures according to the publisher guidelines.

**6) *In line 547-548 there is a strikethrough. Presumably the authors meant to delete this sentence.***

**Response 6:**

We have amended this error.

**7) *Supplementary figure S.1.3.3 has abbreviation for some species names but not for others in seemingly arbitrary manner. Please use full names on first mention in the table, and consistently use abbreviations after that.***

**Response 7:**

These have now been amended as suggested.

**8) *Supplementary figure S.1.3.4 - the figure legend is unclear and seems to repeat itself.*
Response 8:**

We have rewritten the figure legend as suggested: “Figure S1.3.3. Specific gains of homeobox genes between myriapods and insects (upper) and in centipede and millipedes (lower).”

**Reviewer #2:**

**9) *The revised manuscript shows some improvement on restructuring and in correcting specific points addressed by myself and the other reviewer. On the other hand, I see that many points still remain in the current version.***

**Response 9:**

We thank the reviewer for all their constructive suggestions, and for their patience in helping us to improve our manuscript.

**10) *The story is still not coherent and focused. MicroRNA regulation of homeobox genes should be merged with "Millipede homeobox gene and cluster" section or at least located adjascently.***

***Subsection 1: "High quality genome of ..." is a descriptive section on the sequenced genomes.
Subsection 2: "Millipede homeobox gene cluster" mainly discusses the differences between centipede and millipede.***

***Subsection 3: "Specific duplication of argonaute protein" lacks any mention of centipedes, mainly discusses conservation within Arthropoda (but no data is given for Crustaceans and Chelicerates)***

***Subsection 4: "MicroRNA regulate homeobox…" mainly discusses the differences between centipede and millipede. (or at least about miR-96 and miR-283)***

***Subsection 5: "Conserved synteny…" discusses larger phylogenetic conservation (bilaterian)
Subsection 6: "Millipede genome size evolution" compares two millipedes***

***Subsection 7: "Millipede transposable elements"  is mostly descriptive, with a little comparison between millipedes and centipedes (Fig.6)***

***Subsection 8: "Ozadene defensive gland" is mostly descriptive, with some comparision between the millipedes.***

***So if I were to restructure, I would first group the descriptive sections 1,7, then go on to comparison within the millipedes 6, then compare centipedes and millipedes (2,4,7), and then finally discuss larger phylogenetic-scales (3,5),
or even better, group them according to the research question. Majority of the section still lacks biological questions. For example, there is no discussion on genome size difference. What is the possible ecological selection pressure to make the genome larger (or smaller) in one clade? How about the selection pressure to avoid TE in gene neighborhood? Why do they (if they. I will explain below) have different ozadene contents?***

**Response 10:**

This is an excellent suggestion and has really helped us to improve the flow of our manuscript, for which we are very grateful. In accordance with this advice, we have restructured the main text of the manuscript in line with the reviewer’s suggestion (1,7,6,2,4,3,8,5). The only slight deviation is the swapping of subsections 3 and 8, as we found the text flowed more appealingly by discussing small RNA machinery first following discussion of microRNA regulation, rather than placing this after discussion of the ozadene gland.

As suggested, we have grouped and renamed sections according to the research question to highlight the biological significance in each case, which we agree is an excellent suggestion, as follows: a) Evolution of millipede genomic composition and size; b) Homeobox gene organisation rearrangement; c) Conserved and divergent microRNA regulation and machinery; d) The ozadene: millipede chemical defence; and e) Conserved synteny among myriapod and deuterostome genomes.

All figures have also been renumbered according to order of appearance.

Regarding the genome size difference section, we have integrated this section together with the transposable element (TE) section.. As written in the main text, “Thus, we find that both repeat content and intron size are major contributors to the observed differences in millipede genome size.”.

As to the further discussion on whether there may be ecological selection pressure on genome size among clades, or selection pressure to avoid TEs in gene neighbourhoods, these are excellent questions, but currently we lack enough comparisons to be able to tackle these. In the previous round of responses to comments, we attempted to gather information on the genome sizes of millipedes from different clades, but there is unfortunately very sparse information available. Thus, we currently lack the data and statistical power necessary for broader comparative analyses. Considering the fact that there are only two millipede genomes investigated in this study, we found it undesirable to speculate on these areas further. However, we are in process of sequencing millipedes from diverse clades for a follow-up study, and these are excellent suggestions that can hopefully be answered when these additional genomes become available. We have summarised the chromosome numbers documented in millipedes (Supplementary information S1, Figure S1.2.1), but we could not observe any evidence of chromosome fusion/fission in a particular clade.

To sum up, we thank the reviewer for their excellent suggestions, and we very much hope that by carrying out the above changes we have improved the coherence and consolidated the different sections of the manuscript satisfactorily.

**11) ** Quality of the assembly***

***I am still not convinced by the telomere analysis now added as Table 1.3.1e and 1.3.1f (by the way, "8 pseudomolecules" in Table 1.3.1f title should be "17 pseudomolecules"). This is very important, because this information provides the accuracy of the scaffolding process. As I have stated in my previous comments, the overall scaffold length of the presented genome is extremely good, but the contig N50 remains modest. Therefore, I am still worrying the possibility of over-scaffolding (or mis-scaffolding). Presence of centromeres and telomeres would provide some credibility on this scaffolding process.***

***Now, looking at tables 1.3.1e-f, the estimated number of telomeric repeats are around 100~200. The authors simply seemed to have searched for the 5-bp motif TTAGG here, but the probability of finding a 5-bp motif is 4^-5 = 1/1024. Therefore, expected number of finding a random 5bp motif within a 100kbp segment is 100,000 / 1024 = 98, or including its complement, 195, which is intriguingly in-line with the numbers presented here. So I would like to ask again: are they, and are they the only, really "repeated" motifs at the ends of chromosomes, and was the very large 100kbp segment really necessary to find these motifs? Are there centromeres and rRNA clusters in regions of "shifts" in Hi-C contact maps or around the center of the scaffolds? Can you say with confidence that these are actually telomere-to-telomere (encompassing centromeric regions which is often very very difficult to assemble and scaffold) scaffolds? Note that insect telomere is very often TTAGG, but there are
also diversity in telomeric repeat motifs.***

***Again, I cannot test this myself since the genome sequence itself is not availle to the reviewers.***

**Response 11:**

We first apologise for the typos in Table 1.3.1e and 1.3.1f. They have now been amended.

In line with the reviewer’s suggestions, we have carried out additional experiments, including identifying the most probable telomere motif from the genome sequences following the standard Telomere Identification Protocol of SERF (https://bioserf.org/r/p/swebserf#telomere, Somanathan and Baysdorfer 2018), followed by using the probable telomere motif as the custom library input for RepeatMasker in default searches. The information is now included in the new Supplementary information S10, as well as the main text as shown below:

“A total of 4 out of 7 pseudomolecules for *H. holstii* and 11 out of 17 pseudomolecules for *T. corallinus* contain telomeric repeats (Supplementary information S10), illustrating that these genome assemblies represent the first close to chromosomal-level genomes available for myriapods.”

The genome assemblies of both millipedes have been submitted to NCBI, and we also apologise for being inconsiderate (as they are on embargo until after publication). However, for reviewing purposes, both millipede genome assemblies can also be downloaded at the following link: https://www.dropbox.com/sh/8yd7stv31p22o1q/AACizTeFYqVehomhTO_sW-ZSa?dl=0

Reference:

Somanathan, I., Baysdorfer, C. (2018). A bioinformatics approach to identify telomere sequences. BioTechniques, 65(1), 20-25.

**12) ** Synteny***

***Previously I stated "Synteny blocks shown in Fig.2 ... is not being able to show such synteny even between Helicorthomorpha and Strigamia". This is not yet addressed in the revision. I detail my point below.***

***In FIg.5 Mindots=7, each colored region within each of the three circles, starting from the top right human segment counter-clockwise, decreases in length, probably corresponding to the human chromosomes. Now, left circle (Helicorthomorpha) has syntenic block coming out from chromosome 1 (brown), 12 (light green), 16 (purple), 17(greenish brown). Middle circle (Trigoniulus) has in chromosome 2 (blue-green), 12 (green), 16 (brown). Here, the only "conserved" synteny blocks are the ones in chromosome 12 and 16, and all other "synteny blocks" shared with humans are just calculation artefacts, since if the gene synteny is conserved that far into the very distant end of Chordata (i.e. humans), surely such segment MUST be conserved within the millipedes. However, when we look at the right circle (centipede Strigamia), the "synteny block" is only in chromosome 9. So NONE of the synteny blocks shared among humans and millipedes or centipedes are conserved within the myriapods.***

***So again, the use of (and the only use of) humans to discuss the bilaterial conservation is not feasible. In order to discuss the "conservation", much larger number of genomes must be compared, and more phylogenetically feasible clades (such as Cephalochrdata at the root of Chordata) must be employed. At the current state, there is no statistically convincing data to suggest conservation of bilaterian synteny.***

**Response 12:**

We apologise for this oversight. A major reason that we did not carry out the synteny analyses to the scaffolds of the cephalochordate (amphioxus *Branchiostoma floridae*) was that scaffold sizes of the assembly for this taxon were relatively short at the time of the last submission of responses. Fortunately, there is a new publication availavle reporting a chromosomal-level genome assembly of amphioxus *Branchiostoma floridae* (Simakov et al 2020, Nature Ecology and Evolution), which was published in late April while we are preparing this round of responses. Consequently, we have now carried out the synteny comparison to amphioxus as suggested. In addition, we have also carried out synteny comparisons to other deuterostomes including the tunicate *Ciona intestinalis* and the echinoderm *Strongylocentrotus purpuratus*. Please see Lines 343-415.

**13) *A couple of other minor points: 1. The non-consistent color coding of human chromosomes (as exemplified above) is extremely confusing***

**Response 13:**

We have now re-coloured the figures to make this consistent.

**14) *2. What happened to the Hox cluster? (it is a synteny of more than seven genes)***

**Response 14:**

We have carried out additional syntenic analyses using Hox gene scaffolds from different arthropods as shown in Figure 3, and included these in both Figure 3 and Supplementary information Figure S1.3.17.

**15) **Ozadene defensive gland***

***I find it extremely challenging for the author's notion to say T. corallinus does not have the function to produce cyanides and other chemicals in their ozdene glands. In the first genome report of T. corallinus by Kenny et al. (2015), they clearly identified many VTGl, ARSB, MRP genes within this genome. If the authors make such strong claim, these result must be carefully outlined and compared to that of Kenny et al. (2015). Moreover, it should not be that difficult of an experiment to detect (the lack of) cyanides, benzaldehydes, and quinone derivatives from T. corralinus. There are, however, reports that some millipedes do not produce these chemicals (see for example Shear et al. (2007) BIochem. Systematics & Ecology), but such claim should be backed up with more detailed analysis.***

Firstly, we thank the reviewer for these very helpful comments. Consequently, we have thoroughly reanalysed all obtained data to clarify the situation. The clarified situation is now shown in the new Figure 6, Supplementary data S1 and S4. In line with this, the ozadene section has been greatly rewritten with the inclusion of VTGI, ARSB, and MRP genes.

***It is not really logical to search for cyanide/benzaldehyde pathways after looking for antibacterial proteins.***

We have now rearranged the writing in a more logical flow with searches for cyanide/benzaldehyde dealt with first (Lines 357-383).

***"These data and analyses strongly suggest that one of the main functions of 420 millipede T. corallinus ozadene is to provide defence against pathogenic microorganisms, 421 rather than being primarily an antipredator adaptation." Really? "strongly"? even without looking for cyanide/benzaldehyde pathways??***

We thank the reviewer for the suggestion. We have now added an examination of cyanide/benzaldehyde. We also agreed that it is unnecessary to speculate on what the prime function of the ozadene is, and have toned down our claims in this respect, in Lines 389-391, shown as “Consequently, our data add weight to the assertion that one of the main functions of the millipede ozadene, at least in *T. corallinus*, is to provide defence against pathogenic microorganisms, in addition to providing a defence mechanism against predators.”.

***The supplemental figure number seems to be wrong in many cases including this one. Figure S1.3.4 (noted as tyrosinase pathways) is actually hox phylogeny.***

All supplementary figure numbers have been renumbered.

***The list of abundances of peptides is not given in Supplemental Information S4, but the data still makes me worry.***

These were included in the previous submission in Supplementary information S4, but this may not have been clear given the multiple sheets in the Excel file. We have now rearranged these results to be on the first sheet, and the abundances can be found in column D.

***1. ATPase is just too broad. Kinases are ATPases, helicases, gyrase, some HSPs, and so on. Moreover, the top ATPase in the list (Tco_001319-T1) is a mitochondrial ATPase. It could be abundant, but abundant everywhere, not ozadene specific.***

We have carried out further annotations using the non-redundant (nr) protein database to provide more detailed descriptions for all the peptides in Supplementary information S3. This includes Tco_001319-T1, which can now be assigned as ‘ATP-binding cassette sub-family B member 7, mitochondrial”.

The previous version of the excel file did not sort the order by abundance, and might have created the wrong impression that the top (most abundant) protein is ATPase. Among the ATPases, the highest abundance is Tco_008431-T1 but not Tco_001319-T1 as stated. We apologise for not sorting the abundance in the previous version of the Excel file and resulting in this misunderstanding. The issue has been addressed in the latest version of the Excel file S3. The most abundant peptide after sorting in according to abundance is vitellogenin-like, and this information has been added in the main text, please see Lines 383-385.

***2. Top of the list tyrosinase (Tco_008220-T1) is a hemocyanin. It is also abundant, but abundant everywhere.***

Similar to the above, we have now carried out further annotations using the non-redundant (nr) protein database to provide more detailed descriptions for all the peptides in Supplementary information S3. This includes Tco_008220-T1, which can now be assigned as “putative polyxenus lagurus prophenoloxidase/hemocyanin AA6 chain-like” peptide.

***3. betalanin biosynthesis - should be beta-Alanine biosynthesis***

Originally we have used KEGG to annotate gene pathways, however this section has now been removed, given that we do not include statistical analyses of gene pathways any longer (please see next response).

***4. "relative abundance of a pathway" is not a proper statistical enrichment.***

We have completely removed all statistical analyses regarding the “relative abundance of a pathway” in both the main text as well as the supplementary information.

Lastly, if the reviewer would prefer, we are also willing to leave out the whole ozadene section as a separate follow-up paper instead.

**16) *Methods: That of synteny analysis is too simple. At least some explanation of the terminology of "Mindots" is necessary.***

**Response 16:**

We have now included and elaborated on the explanations the details of the synteny analyses in the methodology section, as well as in figure legends. This includes clarification of the term ‘mindots’, which refers to the minimum number of anchors required to define a synteny block. Please see lines 580-581, and 892-893.

**17) *line 595: were is italicized***

**Response 17:**

We thank the reviewer for their careful reading, and we have now amended this typo, and other typos in the manuscript.
