# Supplementary material for: Millipede genomes reveal unique adaptations during myriapod evolution
Source: PLoS Biol. 2020 Sep 29;18(9):e3000636. doi: 10.1371/journal.pbio.3000636 (PMC7523956; doi:10.1371/journal.pbio.3000636)
Supplement: Attachment — Submitted filename: Response.docx [file pbio.3000636.s039.docx]

Dear Dr Hui,

Thank you for resubmitting your manuscript "Millipede genomes reveal unique adaptations during myriapod evolution" to PLOS Biology. We noticed a few remaining issues that will need to be addressed before your manuscript can be formally accepted. Your submission has been sent back to you to enable you to make these changes.

We would appreciate your prompt cooperation with our requests at your earliest convenience. If you have any questions or require clarification, please do not hesitate to get in touch.

1. Please amend the names of your Supplementary files to remove the extra .pdf extension from the name as this prevents the files opening on some devices.
Currently: S1_Fig.pdf.pdf
Correct Version: S1_ Fig.pdf

2. Please ensure that all main figures are referenced in the manuscript as Fig 1, Fig 2, etc. (including capitalization), rather than Figure 1, Figure 2, etc. Please note, however, that the file names themselves must not include the space, i.e. Fig1.tif, Fig2.tif. Please ensure that all main figure files are clearly labeled as such in the File Inventory.

3. Please ensure that all Supporting Information files are clearly labled in the file inventory. i.e. S1_Fig.tif, S1_Table.xlsx, S1_Text.doc, S1_Data.xlsx, S1_PRISMA_Checklist.docx, etc.

Each file type should be numbered starting at 1. Please also make sure that the file description matches the file name (e.g. S1_Fig.tif is listed as S1 Fig).

4. Please upload your Supporting Information items individually, rather than in a single file, as you refer to them individually in the text. If you would like to retain the single file please rename internal items (e.g. tables) as A, B, C etc. Please also provide a legend for each under the heading Supporting Information. Supporting Information files should be less than 10 MB each. Please edit any citation of internal items within your Supporting Information files to using letters rather than numbers, e.g. “Table A in S1 Text”. Please ensure these changes are made throughout the manuscript and within the Supporting Information files themselves.

5. Before resubmitting your paper, please ensure that all files referenced in the manuscript are present in the File Inventory, as missing files will result in further delays. Please also ensure that all file names adhere to our naming conventions and that all in-text citations and supporting information file legends/captions do as well.


You will find your submission in the 'Submissions Sent Back to Author' folder. Once you have made these changes, please re-approve the submission following the same steps as before.

Please do not hesitate to contact me should you have any questions.

Best wishes,

Jessica Hyde
Publications Assistant
PLOS Biology

**Response**:

Thank you for the kind guidance. We have now amended all the files according to the suggestions.
